# Comprehensive analysis of MHC class II genes in teleost fish genomes reveals dispensability of the peptide-loading DM system in a large part of vertebrates

**DOI:** 10.1186/1471-2148-13-260

**Published:** 2013-11-26

**Authors:** Johannes M Dijkstra, Unni Grimholt, Jong Leong, Ben F Koop, Keiichiro Hashimoto

**Affiliations:** 1 Institute for Comprehensive Medical Science, Fujita Health University, Toyoake, Aichi 470-1192, Japan; 2 Centre for Ecology and Evolutionary Synthesis, Department of Biosciences, University of Oslo, Postboks 1066 Blindern, Oslo 0316, Norway; 3 Centre for Biomedical Research, Department of Biology, University of Victoria, PO Box 3020 STN CSC, Victoria, Canada

**Keywords:** MHC, Class II, Classical, Non-classical, DM, Peptide-loading, Teleost fish, Genomics, Evolution

## Abstract

**Background:**

Classical major histocompatibility complex (MHC) class II molecules play an essential role in presenting peptide antigens to CD4^+^ T lymphocytes in the acquired immune system. The non-classical class II DM molecule, HLA-DM in the case of humans, possesses critical function in assisting the classical MHC class II molecules for proper peptide loading and is highly conserved in tetrapod species. Although the absence of DM-like genes in teleost fish has been speculated based on the results of homology searches, it has not been definitively clear whether the DM system is truly specific for tetrapods or not. To obtain a clear answer, we comprehensively searched class II genes in representative teleost fish genomes and analyzed those genes regarding the critical functional features required for the DM system.

**Results:**

We discovered a novel ancient class II group (DE) in teleost fish and classified teleost fish class II genes into three major groups (DA, DB and DE). Based on several criteria, we investigated the classical/non-classical nature of various class II genes and showed that only one of three groups (DA) exhibits classical-type characteristics. Analyses of predicted class II molecules revealed that the critical tryptophan residue required for a classical class II molecule in the DM system could be found only in some non-classical but not in classical-type class II molecules of teleost fish.

**Conclusions:**

Teleost fish, a major group of vertebrates, do not possess the DM system for the classical class II peptide-loading and this sophisticated system has specially evolved in the tetrapod lineage.

## Background

The highly polymorphic classical MHC class II molecules can present exogenous antigenic peptides including those derived from proteins of many pathogens to CD4^+^ T lymphocytes in the acquired immune system [1]. CD4^+^ T lymphocytes then can exert various functions such as helper activities toward other immune cells, e.g. B lymphocytes, macrophages and dendritic cells for their activation [1].

In the mammalian acquired immune system, a non-classical MHC class II molecule, HLA-DM in humans (H2-DM in mice), plays an important role in proper peptide presentation by the classical MHC class II molecules [1,2]. A newly synthesized classical MHC class II molecule, which is a heterodimer composed of the α and β chains, is transported from the endoplasmic reticulum to endosomal compartments including the late endosomal MIIC (MHC class II compartment) by binding to a protein called the invariant chain. The invariant chain blocks the peptide-binding groove of a classical MHC class II molecule by its “CLIP (class II-associated invariant chain peptide)” region so that other endogenous peptides cannot bind to the groove in the endoplasmic reticulum [1,2]. After digestion of the invariant chain by endosomal proteases, CLIP is dissociated from the groove by the non-classical class II DM molecule in the MIIC, and then other peptides, including those derived from exogenous antigens, can bind to the groove of the classical MHC class II molecule [1-6]. The DM molecule also can induce the dissociation of relatively weakly bound peptides thus showing peptide-editing function. The DM molecule itself neither exhibits classical-type polymorphism nor shows binding capacity for peptide ligands [2].

DM molecules are critical for the classical MHC class II function, as exemplified by the observation that human mutant cell lines deficient in DM molecules, and also antigen-presenting cells from the DM-knockout mice, exhibited failure in proper MHC class II peptide presentation [7-10]. Similar to a classical MHC class II molecule, a DM molecule is a heterodimer composed of α and β chains, which are encoded by *DMA* and *DMB* genes, respectively, and possesses an overall structure similar to that of a classical MHC class II molecule, but with a unique narrow groove [11,12]. Orthologous *DM* genes, *DMA* and *DMB*, have been identified not only in many mammals but also in chickens [13] and frogs [14], indicating phylogenetic conservation throughout tetrapods. In all investigated tetrapods, the *DM* genes reside in the *Mhc* region along with the classical *MHC* class I and class II genes [14].

*DM*-lineage genes, however, have not been reported from the largest group of vertebrates, teleost fish, which include more than 26000 species, about 40% of all the species of vertebrates [15]. Teleost fish appear to possess effective acquired immune functions including presumable T lymphocyte-dependent responses to exogenous antigens [16]. Like in tetrapods, various important genes of the MHC class II system have been found in their genomes, which include genes for MHC class II [17,18], CD4 [19,20] and invariant chain [21-23]. The *MHC* class II loci in teleost fish display some unique features, namely their non-linkage with *MHC* class I loci and the lack of synteny of the class II loci between several teleost fish species [24-27]. Regarding non-classical class II genes in teleost fish, studies have been rather limited so far e.g. [28-30].

Previously, the absence of *DM*-like genes was speculated based on extensive homology searches using teleost fish genomes e.g. [14]. However, based on the negative results, one could not obtain a definitive answer, e.g., primitive DM-like molecules might be highly divergent from the known DM sequences. Very recently, the structure of a complex between a classical class II molecule and a DM molecule has been determined and the study revealed the interacting amino acid residues between the two molecules [31]. This allows us, for the first time, to analyze teleost fish class II molecules regarding the possession of the functional residues critically important for “the DM system” (in this paper defined as the peptide-loading system with the DM molecule-involved special mechanism). Taking advantage of recent progress of genome research projects, we searched and analyzed *MHC* class II genes in teleost fish comprehensively. We typified teleost fish class II genes and their protein products based on various classical/non-classical characteristics and then examined the possession of functional residues critical for the DM system. Based on our results, we could draw a clear conclusion about the lack of the DM system in teleost fish. We also discuss possible functions of the intriguing non-classical class II molecules revealed in teleost fish including ones newly identified in the present study.

## Results and discussion

### Comprehensive search for teleost fish *MHC* class II genes

**Figure 1:**
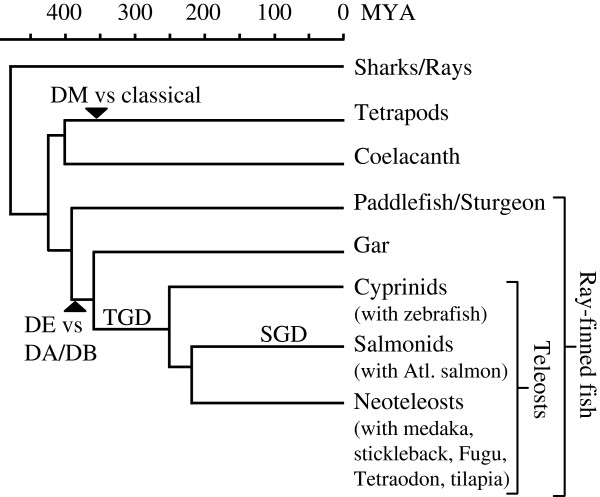
**Divergence times for selected groups of species.** Timeline in million years ago (MYA) is indicated on top. The coexistence of the different class II lineages can be traced back to the period shown by an arrowhead. TGD and SGD indicate the occurrence of teleost-specific and salmonid-specific whole genome duplication events, respectively. See Additional file 1: Figure S1 for references and additional species.

**Figure 2:**
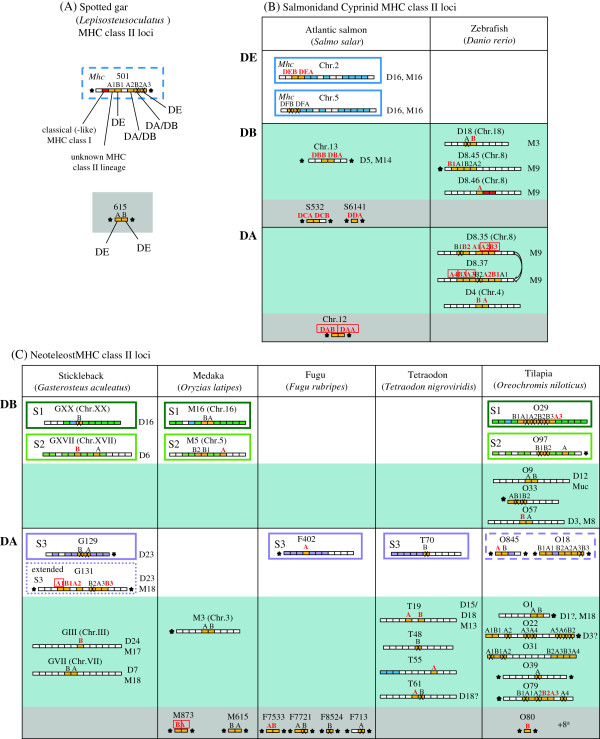
**MHC class II loci in gar and various teleost fish.** Schematic view of MHC class II *A* and *B* genomic regions as found for gar **(A)**, salmon and zebrafish **(B)**, and neoteleosts **(C)**, and, for the teleost fishes, organized per DE, DB and DA group. Small blocks indicate genes, and stars indicate ends of scaffolds. Nomenclature and gene identities are explained in Additional file 2: Figure S2, and for salmon also in Additional file 8: Text S2. Synteny with class II genomic regions in other species is indicated by similar coloring of homologues: blue for *Mhc*-scaffold genes as found in human, dark green for neoteleost S1 region genes, lime green for neoteleost S2 region genes, and purple for neoteleost S3 region genes. Dotted and dashed purple lines indicate extended and probable S3 regions, respectively. Orange and red boxes represent MHC class II and MHC class I genes, respectively. “A” stands for a class II α chain gene and “B” for a β chain gene. Class II *A* and *B* gene names are in red font if matching transcripts were found (Additional file 5: Table S2 and Additional file 7: Text S1), and the name is boxed if matching transcripts were abundant. Crosses indicate pseudogenes and/or genes with incomplete information. White backgrounds indicate that in syntenic regions in other species also MHC class II genes were found, blue backgrounds indicate scaffolds without such class II synteny, and gray backgrounds indicate lack of sufficient sequence information for estimation of synteny. Observed synteny between teleost regions regardless of MHC class II presence (Additional file 4: Table S1) is summarized behind scaffolds by “M + number” and “D + number” for the respective chromosome numbers in medaka (M) and zebrafish (D for *Danio*), respectively. Linkage of classical-type class I and class II in spotted gar scaffold 501 suggests that this is an *Mhc* region, but more information on neighboring genes is needed (therefore dashed blue line). MHC class I genes on zebrafish Chr.8 are nonclassical [56]. The MHC class II genes and the *Mhc*-scaffold gene *MSH5* (Additional file 2: Figure S2) in the neoteleost S1 group may or may not have derived from a direct translocation from the *Mhc* region on the same chromosome [33]. Tetraodon scaffold T55 has no synteny with regions in other species (Additional file 7: Table S1), so the linkage of *T55 A* gene with *Mhc*-scaffold genes (Additional file 2: Figure S2) is probably not ancestral. ^a^ Eight tilapia regions with little informative value were omitted from the figure, but are shown in Additional file 2: Figure S2.

**Figure 3:**
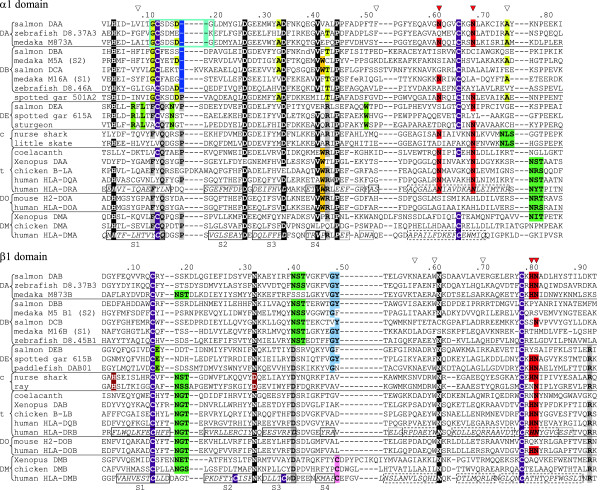
**Alignments of representative α1 and β1 domain sequences.** Representative teleost fish DA, DB and DE group sequences are compared with MHC class II of primitive fish and tetrapods. Shown sequences correspond with single exons, while residue numbers above alignments relate to mature HLA-DR molecules [38]. c: cartilaginous fish classical-type class II. t: tetrapod lineage classical-type class II. Dashes: gaps made for the alignment. Blue frame: the DAA-lineage specific GCSDXDG (or similar) motif. Downward triangles: peptide-backbone interacting residues based on mammalian studies [38], with red triangles for the positions α62, α69, β81 and β82 (see also Additional file 6: Table S3). Various color shadings represent as follows. Purple: cysteines confirmed or predicted to form a disulfide bridge. Rose: DM-specific cysteine. Green: conserved N-glycosylation motifs. Red: α62 N, α69 N, β81H and β82 N. Yellow: typical residues shared between spotted gar 501 A2 and teleost DAA and DBA sequences. Gold: tryptophan residues at the position 43 of α1 domain. Black: highly conserved residues among jawed vertebrates. Blue in β1: ray-finned fish specific residues. Lime green: DE group specific residues. Gray: residues shared by DE group and class II in cartilaginous fish and tetrapods. Brown: cartilaginous fish residues that appear to be ancestral. Dark and light blue in α1: single and two residues deletion compared to class II consensus, respectively. Italic font: human DR and DM secondary structures with solid frames for the β-strands S1-S4, dotted frames for 3_10_ helices, and dashed frames for the α -helices according to PDB structures 3PDO and 2BC4. At the site of non-capital font “nd” in salmon DBA α1 the stretch SNTCLIA was deleted for lay-out reasons, and at the site “fe” of medaka M16A α1 this was FKANLS (Additional file 10: Text S4A). Sequences are referenced in Additional file 7: Text S1; Additional file 8: Text S2; Additional file 10: Text S4.

**Figure 4:**
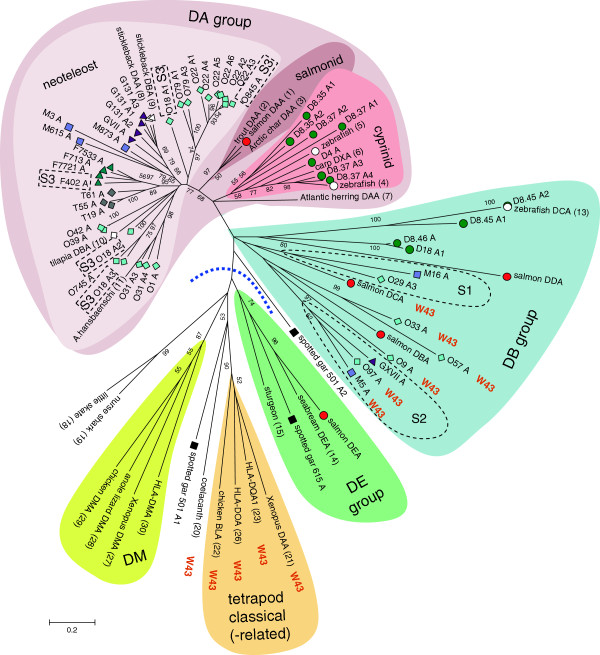
**Phylogenetic tree of MHC class II α1 domain sequences.** The sequences which possess αW43 are labeled with the orange letter “W”. The bootstrap consensus tree was inferred from 500 replicates using the Neighbor-Joining method [61]. The tree is drawn to scale, with branch lengths representing the number of amino acid substitutions per site (see scale bar). Numbers behind names relate to references listed in Additional file 10: Text S4. Sequences that are also depicted in Figure 2 have their names with colored shapes indicative for the species: black square for spotted gar, red circle for Atlantic salmon, green circle for zebrafish, purple square for medaka, violet triangle for stickleback, gray diamond for Tetraodon, teal triangle for Fugu, blue diamond for Nile tilapia. Identical shapes, but white, are used for other reported sequences of those species. DM sequences are shown with the yellow background color. Tetrapod classical (-related) sequences are shown with the orange background color. The other background colors distinguish DA, DB and DE sequences, and within the DA group also cyprinid, salmonid and neoteleost sequences are distinguished. The dashed blue line divides the DA/DB lineage from the other class II sequences. S1, S2 and S3 indications plus dashed lines refer to products of genes situated in the respective synteny regions (Figure 2). Spotted gar 501A1 shows some interesting similarity with MHC class II of other classes of vertebrates (see also Additional file 10: Text S4 and Additional file 3: Figure S3B), but this single sequence does not provide sufficient information for valid discussion.

Using various databases, we extensively searched for teleost *MHC* class II genes. The ancestors of teleost fish and tetrapods have separated from each other more than 400 million years ago (Figure 1). Evolutionary relationships among relevant species are depicted in Figure 1 and also in Additional file 1: Figure S1, with more details. We identified a total of 120 *MHC* class II genes or partial genes in the following Ensembl genomic databases: *Danio rerio* (zebrafish; ZV9), *Gasterosteus aculeatus* (stickleback; BROAD S1), *Oryzias latipes* (Medaka1), *Takifugu rubripes* (Fugu4.0), *Tetraodon nigroviridis* (Tetraodon8.0) and *Oreochromis niloticus* (Nile tilapia; Orenil 1.0). Seventy-eight of these sequences are devoid of apparent deletions, premature stop codons and/or frame-shifts. Further, we investigated our improved assembly of the Atlantic salmon genome and found five new class II genes. The *MHC* class II sequences obtained in this study are summarized in Figure 2 (their genomic locations with surrounding genes). The amino acid sequence comparison of representative class II sequences is presented in Figure 3. The phylogenetic tree analyzed based on the aligned sequences is shown in Figure 4 (α1 domain of class II α chain) as a representative. Additional file 2: Figure S2, Additional file 3: Figure S3, Additional file 4: Table S1, Additional file 5: Table S2, Additional file 6: Table S3, Additional file 7: Text S1, Additional file 8: Text S2, Additional file 9: Text S3 and Additional file 10: Text S4 provide the detailed information.

### Novel expressed *MHC* class II genes in an *Mhc* region of the Atlantic salmon genome and their ancient characteristics

In a duplicated *Mhc* region on chromosome 2 (linkage group 1 (LG1); Additional file 8: Text S2) of the genome of Atlantic salmon (*Salmo salar*), our model fish, we could find two novel class II genes, named *DEA* and *DEB*, the former encoding an α chain and the latter a β chain of an MHC class II molecule (Figure 2B and Additional file 2: Figure S2). These genes are located closely to each other like a typical pair of MHC class II genes of mammals. A teleost fish specific whole genome duplication (Figure 1 TGD) resulted in duplication of the *Mhc* region to two different chromosomes, each experiencing subsequent rearrangements and gene losses [32,33]. Teleost classical MHC class I genes were found in only one of those two *Mhc* regions e.g. [34]. Our mapping of the Atlantic salmon *DEA*/*DEB* to chromosome 2 is the first identification of intact *MHC* class II loci within a teleost *Mhc* region (Figure 2B and Additional file 2: Figure S2), representing the TGD duplicate *Mhc* region without class I [32,33]. Early in the salmonid line an additional whole genome duplication occurred (Figure 1 SGD), which presumably resulted in duplicate *Mhc* regions on chromosomes 2 and 5 (Figure 2B and Additional file 2: Figure S2). In the Atlantic salmon *Mhc* region of chromosome 5, *DE* group genes also can be found, namely the pseudogenes *DFAψ* and *DFBψ* (Figure 2B and Additional file 2: Figure S2). Both *DEA*/*DEB* on chromosome 2 and *DFAψ*/*DFBψ* on chromosome 5 are closely linked with typical *Mhc* scaffold genes such as *BRD2*, *TAP1*, *PBX2* and *RGL2* genes (Additional file 8: Text S2 and Additional file 2: Figure S2). A possible orthologue of *DFAψ*, equally partial and inactivated, has previously been described in the salmonid fish rainbow trout [32].

DE-related MHC class II sequences could be identified in the databases for a few other teleost fish including red seabream (a neoteleost fish), fathead minnow and dojo loach (both Cypriniformes), and importantly several primitive non-teleost ray-finned fish like spotted gar, sturgeon and paddlefish (Figures 3 and 4, Additional file 3: Figure S3, Additional file 10: Text S4). In the genome of the spotted gar (*Lepisosteus oculatus*; PreEnsembl Lepocu1) we could identify four DE-related sequences (Figure 2A).

In Figure 3, the predicted amino acid sequences of the membrane-distal domains of the Atlantic salmon DE molecule are compared with those of other MHC class II molecules. The DE and related sequences significantly differ from all the teleost fish class II sequences previously published. Particularly, they share several residues with class II molecules of the other jawed vertebrates (not only the classical class II molecules of tetrapods and cartilaginous fish but also the DM molecules of tetrapods) which are not observed in previously reported teleost fish class II. Those include F12, Q14, P18, R44 of class II α1 domain and D41 and R93 of class II β1 domain (shaded with grey in Figure 3). Regarding specific residues of DE and related sequences, those consist of the following (shaded with lime green in Figure 3): R8, L10, N16, W51 of α1 domain and E16 of β1 domain.

The *Mhc* regions in tetrapods and cartilaginous fish comprise a similar set of *MHC* class I and II genes, as well as other *Mhc* scaffold genes [35,36]. In tetrapods, the *DM* genes also reside in this region. The teleost fish genomes, in contrast, represent a derived situation in that the classical *MHC* class I and II genes are not linked [24,26]. In the primitive ray-finned fish spotted gar genome, we found that a classical-type class I gene (for its molecular features, see Additional file 2: Figure S2) is linked with class II genes (Figure 2A), which suggests that the non-linkage between classical class I and II was established only within teleost fish.

### Clarification of the three major MHC class II groups in teleost fish

With the addition of a newly identified group, teleost fish class II genes can be organized into three major groups, namely, DA, DB (named after a zebrafish DB sequence) and DE group, based on several sequence features including specific insertions and/or deletions (Figure 3 and Additional file 10: Text S4). Previously, reported DA and DB group sequences were classified as classical and non-classical class II, respectively, assignments which were based only on polymorphism and expression analyses and not on comparison of characteristic amino acid residues (Additional file 9: Text S3). The DE and DA groups are well supported as distinct lineages by phylogenetic tree analyses. And the tree shows that early in ray-finned fish evolution there was a separation between DE lineage and DA/DB lineage, and that from the latter the DA lineage sprouted in teleost fish (Figure 4 and Additional file 3: Figure S3). How the DA lineage relates to extant lineages within the teleost DB group is not clear at present. Three discernible lineages within the DB group are represented by (i) genes found in zebrafish, (ii) the Atlantic salmon *DCA*/*DCB* plus genes found in the neoteleost genomic synteny region “S1”, and (iii) the Atlantic salmon *DBA*/*DBB* plus genes found in the neoteleost genomic synteny region “S2” (Figures 2, 3, 4 and Additional file 4: Table S1).

Previous studies on teleost fish DA and DB group genes reported a lack of synteny between teleost fish class II genomic regions, suggesting the occurrence of multiple translocations and locus turnovers e.g. [25,27]. Our study largely agrees with and extends those previous observations in these respects (Figure 2 and Additional file 4: Table S1), although within neoteleosts, some syntenic MHC class II regions could be found (Figure 2: Additional file 1: Figure S1, Additional file 2: Figure S2 and Additional file 3: Figure S3). The observation that the classical DA lineage shows little locus conservation is unexpected since the longevity of a locus should be helpful for the maintenance of allelic variations. For example, a high rate of loci turnover was claimed to be responsible for the limited diversity of the MHC class I genes in the cotton-top tamarin [37] and, further, polymorphic MHC class I genes tend to map to more ancient genomic regions than non-polymorphic ones throughout the jawed vertebrates e.g. [34].

### Classical and non-classical features of teleost fish class II genes

To compare with tetrapod classical and non-classical class II genes, various features of teleost fish class II genes are investigated in the following and summarized in Table 1. In short, the DA group contains classical molecules whereas the DB and DE groups comprise non-classical molecules. It is intriguing that some teleost fish non-classical class II share the listed features with non-classical class II in tetrapods, since the ancestors of teleost fish and tetrapods separated from each other more than 400 million years ago (Figure 1). Characteristic classical and non-classical class II features thus have coexisted for a very long evolutionary time.

**Table 1:** Comparison of various features of class II genes and their protein products in jawed vertebrates

	**Cartilaginous fish classical**	**Gar DA/DB**	**Gar DE**	**Teleost fish DA**	**Teleost fish DB**	**Teleost fish DE**	**Tetrapods classical**	**Mammals DO**	**Tetrapods DM**
Located at *Mhc*	**+**^a^	(**+**) ^b^	(**+**) ^b^	―	―	**+**	**+**	**+**	**+**
Linked with classical-type class I	**+**^a^	**+**	**+**	―	―	―	**+**	**+**	**+**
Classical-type polymorphism	**+**^a^			**+**	―	―	**+**	―	―
Expression	**+**^a^			high	low-med	low	high	med	med
Peptide-binding residues ^c^	**+**	(**+**) ^d^	(**+**)	**+**	(―)	(―)	**+**	(―)	―
CD4-binding βS144, βE162	―		**+**	**+**	**+ /**― ^e^	**+**	**+**	**+**	―
Endosomal sorting motif ^f^	―			―	**+ /**― ^e^	―	―	―	**+**
αW43	―	―	―	―	**+ /**― ^e^	―	**+**	**+**	―
Amino acid at α125	G	N	D	G	HNDKG	D	NK	ND	N

Features of class II genes and those of their protein products are compared in the upper four and in the lower five rows, respectively. Details for expression and polymorphism are described in Additional file 8: Text S2 and Additional file 9: Text S3. Sequence comparisons are based on Figure 3, Additional file 10: Text S4 and ref. 31. Blanks indicate no information. ^a^ Based on ref. 35 and references cited therein; ^b^ Probable *Mhc*; ^c^ αN62, αN69, βH81, and βN82. Pluses show relatively high conservation, and parentheses indicate partial situations; ^d^ No information for the β chain; ^e^ Some possess these features and some do not; ^f^ Tyrosine-based.

#### Expression and polymorphism

The DA group includes all the highly expressed teleost class II genes (Figure 2, Additional file 5: Table S2) and previously reported polymorphic ones (Additional file 9: Text S3). There have been relatively few reports on teleost non-classical class II genes e.g. [28-30] (Additional file 9: Text S3), and our present study added identification, gene-specific expression and polymorphism analyses of the Atlantic salmon *DCB* (DB group), *DEA* and *DEB* (DE group) (Additional file 8: Text S2). Together with our previous analyses of Atlantic salmon DB group genes, *DBA*, *DBB*, *DCA* and *DDA*[29], and non-specific transcriptome analysis in the present study (Additional file 8: Text S2), the results show that all these genes are essentially non-polymorphic and expressed at much lower levels than the classical *DAA* and *DAB* genes of the DA group in the same species. Especially the expression of Atlantic salmon *DEA* and *DEB* is very low. The Atlantic salmon genes *DCA*, *DCB* and *DEA* have rather tissue-specific expression patterns unknown for mammalian class II genes (Additional file 8: Text S2).

#### Peptide binding capacity

Only DA molecules, but not DB and DE, display a high degree of conservation of the α1 domain residues αN62 and αN69 and the β1 domain residues βH81 and βN82, which in mammalian classical molecules make important hydrogen bonds with the backbone of peptide ligands [38,39] (Figure 3, Table 1, Additional file 10: Text S4 and Additional file 6: Table S3). Best conserved is βN82, known to be of particular importance [40] (Additional file 6: Table S3). At the other three positions, teleost fish DA molecules exhibit some variations comparable to some species in mammals (Additional file 6: Table S3). Although the presence of such peptide-binding residues in DA molecules has been reported before [41], the detailed analyses of teleost fish DB molecules have not been reported. Among the DA group, a few DA molecules lack these peptide-binding residues and might exert nonclassical functions akin to DO in mammals, which diverged from classical molecules in relatively recent times.

#### CD4 binding capacity

Recently, several amino acid residues important for the interaction between CD4 and class II molecules were revealed [42]. Two of those residues at the interface of the two molecules appear highly conserved throughout tetrapod species, namely βS144 and βE162 (Additional file 10: Text S4). We found that these two residues are also highly conserved in teleost fish DA, whereas cartilaginous fish classical molecules, tetrapod DM molecules and some teleost fish DB molecules lack these residues (Table 1 and Additional file 10: Text S4).

#### Endosomal sorting motif

A characteristic feature of the non-classical DM molecules is the possession of an endosomal sorting motif in the β chain cytoplasmic tail [43,44] (Table 1). We found potential tyrosine-based endosomal sorting motifs in the β chain of a few teleost DB group molecules although their location differs from those in the DM molecules (Table 1 and Additional file 10: Text S4E).

#### Preservation of classical genes throughout teleost fish

Based on the current database information, all the teleost fish species that we investigated have DA, most of them have DB, and a few of them have DE group genes. Conservation of gene copies preserving classical features accompanied by seemingly random loss of older non-classical gene duplicates is somewhat reminiscent of MHC class I evolution described for higher vertebrates [45]. However, the mode of the teleost fish MHC class II evolution highly contrasts with that of the tetrapod species in which not only the classical class II but also the non-classical *DM* genes are highly conserved. Recently, it was reported that Atlantic cod, a teleost fish, does not possess various genes in the MHC class II system such as those for MHC class II molecules, CD4 and invariant chain [27]. Although this apparent loss of the MHC class II system should have various disadvantages and actually the cods are known to have a poor adaptive antibody response, they do survive and thrive. The cod situation reflects the plasticity of the teleost fish immune system in which other factors may adapt to a large variation in the MHC class II system [27].

### Phylogeny of the DM system through the window of the critical functional residues

#### A critical residue of the DM system is not found in the teleost fish classical class II molecules

Recently, the structure of HLA-DM/HLA-DR1 complex was clarified [31]. The overall structure of the complex is largely consistent with the previous independent estimation of the interface of the two molecules based on experiments using mutagenesis [46-49] and tethered complexes [50]. In the side-by-side structure, the interface is mainly formed by the α chains of the two molecules, and a lateral surface of the DRα1 domain, close to the N-terminus of the peptide-binding groove, interacts with DM α chain and additionally DMβ1 [31]. The structural study revealed two key amino acid residues (αN125 of DM and αW43 of DR1) in the interaction between the DM molecule and the classical class II DR1 molecule at pH 5.5, which is within the range of the physiological late endosomal pH suitable for the DM activity [31]. In the structure of the DM/DR1 complex, a tryptophan residue of the DR1 molecule (αW43) flips from the original location and its indole ring nitrogen atom interacts with an asparagine of the DM molecule (αN125) through a hydrogen bond [31]. This was elucidated by comparison between the structures of the DM-bound [31] and -unbound DR molecule [51]. The change of αW43 position is accompanied by conformational alterations in the P1 pocket peptide-binding region of the DR1 molecule, which include the novel formation of a long α-helical segment with a short break and the repositioning of the hydrophobic αF51 into the P1 pocket [31]. These changes explain the dissociation of CLIP, the stabilization of empty class II molecules, and further the selection of high affinity peptide ligands [31]. The previous study indicated that αW43F mutation of the DR molecule greatly reduced both the DM function and the binding to DM molecule [48]. The effects of αN125A and αN125R mutations in the DM molecule were also examined and these mutations caused a loss of both the DM activity and the binding to the DR molecule [31]. HLA-DO, a human non-classical class II molecule, which can bind tightly to the DM molecule and is known to be an inhibitory modulator of the DM molecule, also possesses αW43. The structure of HLA-DM/HLA-DO complex was independently reported very recently and it also revealed the important participation of αW43 of HLA-DO and αN125 of HLA-DM in the complex formation in which HLA-DO behaves as a mimic of the classical class II molecule [52]. Very importantly, the classical class II α chains of the DA group of teleost fish do not possess the αW43 critical for the interaction with the DM molecule (Table 1, Figure 4, Additional file 10: Text S4A).

As αW43 constitutes a part of the β-strand 4 and there are a few highly conserved amino acid residues near this position, we did not have any difficulties with the alignment of the position 43 between teleost fish and tetrapod class II α chain sequences. At the amino acid position corresponding to αW43, the teleost fish classical-type class II molecules, namely, those belonging to the DA group, exhibit variability (Additional file 10: Text S4A), but no tryptophan residue was observed. Retrospectively, the absence of a tryptophan and also some variability at the position 43 of the class II α chain of teleost fish can be recognized in a previous study using a few teleost fish sequences [41] and also in the other studies when the sequence alignments are adjusted e.g. [29,53]. However, the meaning of these observations could not be understood in relation to the DM system before. In the present study, we comprehensively investigated various teleost fish genomes and examined many DA group members. We did not find any sub-lineages in the DA group in which class II α chains specifically possess αW43 (Additional file 10: Text S4A). The variable nature of the position 43 of the class II α chain without specific conserved residues suggests that the teleost fish classical class II molecules do not use this amino acid position for the interaction with some regulatory molecules. Rather, teleost fish appear to use this position to further increase the variation of the pocket.

#### Teleost fish αW43-containing class II molecules are classified as non-classical

In the DB group of teleost fish, we could find that six sequences (M5A of medaka, O97A, O9A, O57A and O33A of tilapia and DCA of Atlantic salmon) possess αW43 (Additional file 10: Text S4A). Although our previous study reported that the Atlantic salmon *DCA* gene for the α chain was not polymorphic based on the EST information [29], the identification and analysis of *DCB* gene for the β chain in the present study was important, as we know examples in which the extent of polymorphism is highly different between the genes coding for the α and β chains. In the case of human classical class II *DR* genes, *DRA* shows only limited polymorphism while *DRB* shows very high polymorphism [54]. In the present study, the low polymorphism of *DCB* gene was clarified, and therefore we could classify the plausible pair of the Atlantic salmon DCA/DCB molecule as non-classical with the other supporting observations described in the following. Consistent with the non-classical feature of lacking polymorphism, DCA lacks both peptide-binding asparagine residues (αN62 and αN69) although DCB retains βN82. For the position 69, DCA possesses a hydrophobic residue like the DM sequences, a feature shared with most of the other teleost fish DB sequences (Additional file 6: Table S3). Further, both *DCA* and *DCB* genes showed unique expression patterns. They are expressed predominantly in the digestive tract, as observed both in the RT-PCR study and in the transcriptome analysis (Additional file 8: Text S2). These expression patterns are quite different from those of the classical class II genes of both teleost fish DA group and tetrapods. The classical MHC class II genes of human and Atlantic salmon show their highest expression in various immunologically important tissues when investigated with transcriptome analyses, and also human *DM* genes, *HLA-DMA* and *HLA-DMB*, show similar expression patterns (Additional file 8: Text S2). Another characteristic of DCB is that, as already mentioned briefly, DCB interestingly possesses a putative tyrosine-based endosomal sorting motif in its cytoplasmic tail like the DM molecules, although the position of this motif is different from those observed in the DM molecules (Additional file 10: Text S4E). This endosomal sorting motif can also be observed in molecules relatively closely related to DCB (Additional file 10: Text S4E). Non-classical identity of DCB could not be deduced from the analysis of the CD4-binding residues as DCB possesses both βS144 and βE162 (Additional file 10: Text S4D), but the retention of these residues in non-classical molecules can also be observed in other cases such as HLA-DO (Additional file 10: Text S4D).

Similar to DCA, the other five teleost fish class II α chain sequences that possess αW43 could be classified as non-classical based on the analysis of the residues important for peptide-binding although we do not have clear information about their polymorphism and expression patterns (Additional file 10: Text S4). Their apparently intact, presumable β chain partners (three out of five) could also be classified as non-classical as they lack the important peptide-binding residues (both βH81 and βN82 in two cases and βH81 in one case) and also lack at least one of the CD4-binding residues (Additional file 10: Text S4). All the identified teleost fish class II α chains that contain αW43 belong to the DB group of the synteny region “S1” or “S2” of neoteleost fish or their closely related molecules including the Atlantic salmon DCA and together they seem to have descended from a common ancestral molecule in the early phase of the evolution of the DB group (Figure 4).

In addition to αW43, the residues αK38 and αE40 of the DR molecule also participate in the formation of the hydrogen bonding network between DR and DM molecules [31] and previous mutagenesis experiments supported the importance of αE40 [46]. Among the six teleost fish molecules which possess αW43, half of them also possess both αK38 and αE40. Although the chicken and frog classical class II molecules possess different amino acids, the teleost fish DB group molecules of the synteny region “S1” or “S2” group and their closely related ones and also the classical-type DA group molecules possess these residues at the relevant positions in relatively high frequency (Additional file 10: Text S4). The coelacanth class II molecule also possesses αE40 [31]. In the hydrogen bonding network, αE40 of the DR molecule interacts with αR98 of the DM molecule. The conservation of αK98 can be observed in chicken [31] and frog DM molecules (Additional file 10: Text S4), and also often in the teleost fish DB group molecules and in some DA molecules (Additional file 10: Text S4). Therefore, various preconditions for the critical hydrogen bonding network between DM and DR molecules appear to have been already established in the common ancestor between teleost fish and tetrapods.

#### A teleost fish αN125-containing class II molecule is not a DM-equivalent

αN125 is conserved in mammalian and chicken DM molecules as previously noted [31] and it is also conserved in frog DM molecules (Additional file 10: Text S4). αN125 is not specific to the DM molecule, but is also observed in many classical class II α chains of the tetrapod lineage including frog, coelacanth and human DQA-related mammalian molecules, as well as in some closely related non-classical ones like human DOA (Additional file 10: TextS4). We could find, thus far, a single αN125-containing class II sequence in teleost fish, namely, DDA of Atlantic salmon (Additional file 10: Text S4). In our previous paper, we reported that *DDA* has little polymorphism based on available EST information. DDA does not possess two conserved peptide-binding asparagines (αN62 and αN69) and it possesses a hydrophobic residue at the position of 69 as found in most of the other teleost fish DB and also tetrapod DM molecules. *DDA* is predominantly expressed in spleen although in much lower amounts compared to the classical *DAA*[29] (Additional file 8: Text S2). Based on various observations described above, DDA could be classified as a non-classical class II molecule, although we do not know about DDB at present. When we conducted homology searches with Atlantic salmon DDA, we could not retrieve DDA-like sequences from genome sequence databases of other teleost fish species. Therefore, although DDA is a non-classical class II α chain possessing αN125, it is not like DM that is highly conserved throughout tetrapod species.

#### Novel teleost fish class II group DE

In the present study, we identified a new teleost fish class II group called DE. As described above, *DEA* and *DEB* genes of Atlantic salmon do not show classical-type polymorphism and their expression levels are very low (Additional file 8: Text S2). Although the predicted Atlantic salmon DE molecule possesses both βS144 and βE162 for CD4-binding, it lacks αN62, βH81 and βN82 for peptide-binding. Based on these observations, we could exclude the Atlantic salmon DE molecule from the classical class II group. As also described in the previous section, the Atlantic salmon DE molecule shares several amino acid residues with the cartilaginous fish and tetrapod classical, and also tetrapod DM molecules (Figure 3, Additional file 10: Text S4). From the standpoint of the conservation of these residues, the DE molecule is closest to the DM molecules among all the known teleost fish class II molecules. All the available DEA sequences found in teleost fish and also in the primitive ray-finned fish possesses an aspartic acid residue at the position of 125 instead of an asparagine (Additional file 10: Text S4C). Intriguingly, an aspartic acid also can participate in a hydrogen-bond interaction with an indole ring nitrogen atom of a tryptophan like an asparagine e.g., [55].

The observation that the gar class II sequence of the conserved DE lineage possesses αD125 and additionally another gar class II sequence (Additional file 10: Text S4C) possesses αN125 suggests that αN125/D125 already appeared in the class II molecules of an ancestor of Osteichthyes, whereas available cartilaginous fish sequences possess a glycine at this position that appears ancestral to the MHC family [56] (Table 1, Additional file 10: Text S4C). It should be noted that a glycine cannot provide hydrogen-bond capacity necessary for the DM function. Together with the other residues already discussed above, αN125/D125 in ray-finned fish molecules further suggests the establishment of the preconditions for the critical hydrogen bonding network between DM and DR molecules in the common ancestor of teleost fish and tetrapods.

With the identification of DE genes in primitive ray-finned fish like the spotted gar, the coexistence of classical and non-classical class II lineages for a long evolutionary time was demonstrated in the present study (Figure 1). DE genes are found in some teleost fish, while they could not be identified in the genome databases and the EST databases of the other teleost fish. Thus, although DE molecules share some non-classical features with DM molecules, they seem to have quite different characteristics regarding stable inheritance.

#### Teleost fish do not possess the DM system

Without a complete coverage of teleost fish genomes, it is logically not possible to deny the existence of DM-equivalent genes. With this limitation, we clarified that some non-classical class II genes exist in the teleost fish genomes whose protein products partially share some characteristics with the DM molecule. Actually there exist some teleost fish non-classical class II molecules that possess αN125 or αD125. Mainly based on their conservation profiles in the teleost fish genomes, they appear not to be DM-equivalents of teleost fish. In the middle of the β2 domain, the tetrapod DM molecules have a unique insertion of several amino acid residues and near or in this region there are a few residues (e.g. βE47 and βL51) influential for the DM/DR interaction supported by the mutation studies [47] and also by the structural study [31]. So far, teleost fish genes whose protein products possess these features have not been identified.

To obtain a clear conclusion about a possible DM system in teleost fish, it was necessary to investigate the other side of the DM mechanism, namely, the possession of the critical tryptophan residue in the classical class II molecules. Based on the analyses regarding αW43 in the teleost fish classical class II molecules, we could conclude that teleost fish classical class II molecules do not possess αW43 and therefore do not possess the DM system in which the interaction between αW43 of the classical class II molecule and αN125 of the DM molecule is critically important. Although we could observe some teleost fish class II molecules which possess αW43, we could clearly classify them as non-classical. In contrast, coelacanth classical-type class II molecule has this tryptophan [31], and this might be consistent with the presence of the DM system in the primitive stage of the tetrapod lineage. Although available data are very limited, known classical polymorphic class II α chains of cartilaginous fish do not possess αW43 (Figure 3, Additional file 10: Text S4A), e.g. the nurse shark molecule has an alanine at this position which does not have hydrogen-bonding capacity. Therefore, αW43 appears fixed in the classical class II α chain only from the level of coelacanth in the lobe-finned fish/tetrapod line [31] (Figure 3, Additional file 10: Text S4A). As the coelacanth class II molecule also possesses αN125, both the important αW43 and αN125 prerequisites for the DM function may have been fixed at the early phase of the tetrapod lineage (Figure 1). All these observations support that the DM system has specifically evolved in the tetrapod lineage.

### Possible MHC class II peptide-presentation system of teleost fish without DM

Studies on the teleost fish MHC class II peptide-presentation system have thus far been very limited. If we assume that the teleost fish invariant chain [21-23] possesses functions similar to those of mammals [1,2], some basic issues need to be considered for the peptide-loading pathway without the DM system. Those include the dissociation of CLIP-equivalent fragments from classical class II molecules and the stabilization of empty classical class II molecules during peptide-exchange reactions. First, as some mammalian classical MHC class II molecules bind CLIP with low affinity, the rapid dissociation of CLIP has been observed at an endosomal low pH [2,57]. Therefore, if the binding of teleost fish CLIP-equivalent fragments to classical class II molecules is not so strong, the fragments may dissociate without help of a DM-equivalent. Second, classical class II gene duplications can produce an evolutionary reservoir of nonclassical class II genes. Some classical MHC class II molecules possess intrinsic affinity for each other [58], and similar interactive forces may have been the evolutionary basis of the tetrapod DM molecules for establishing the specific interaction wtih classical class II molecules. Some teleost fish non-classical class II molecules may also have acquired stabilizing activity toward classical class II molecules in evolution. Thus, although mechanisms cannot be identical, there might be some overlap between the tetrapod DM and the teleost non-classical class II functions in aiding classical class II molecules.

For distinct subgroups of teleost fish, we may find different strategies for the MHC class II peptide-loading. With four kinds of non-classical class II molecules, Atlantic salmon might have a peptide-loading system uniquely evolved with these molecules. Other than peptide presentation, some reports indicated that tetrapod MHC class II molecules can be involved in signaling pathways [1], and some teleost fish class II molecules may participate in similar or yet unknown functions.

## Conclusions

The accelerated progress on whole genome sequence and also expressed sequence information of various species certainly is valuable to gain an evolutionary bird’s-eye view of important biological systems. The observation that all the authentic polymorphic classical class II-type molecules of teleost fish do not possess the critical residue αW43 led us to conclude that teleost fish do not possess the DM system. The DM molecule appears to have acquired highly sophisticated and efficient mechanisms for peptide editing and stabilization of the classical class II molecules, ensuring its preservation throughout tetrapod evolution. As teleost fish comprise a significant part of the jawed vertebrates (more than 40% of all the species), the present study revealed that both DM-dependent and -independent systems are present as major fractions in the jawed vertebrates. Our study also suggests that preconditions necessary for the important hydrogen-bonding network in the DM system appeared in the common ancestor of teleost fish and tetrapods. Exploring the teleost fish class II peptide-loading system would constitute an important part for the comprehensive understanding of the MHC class II antigen-presentation systems in the jawed vertebrates. Future studies on the non-classical class II molecules of teleost fish should reveal whether they have functions to support the classical class II molecules like the DM molecules or other and yet unknown functions.

## Methods

### Data mining and bioinformatics

A mixture of annotated and un-annotated MHC class II α and β sequences were identified using Ensembl’s Biomart and the GO term for class II (GO: 0042613) supplemented with various blastN and TblastN searches of Ensembl and NCBI databases using evolutionary diverged as well as species-specific sequences. For Atlantic salmon, we supplemented the six known Atlantic salmon MHC class II genes [29] with blastN and TblastN searches using available salmon genome sequences including and mostly from our ongoing genome sequencing project (in part available at either cGRASP (http://web.uvic.ca/grasp/) or NCBI (http://www.ncbi.nlm.nih.gov/)). Open reading frames were predicted using GenScan [59] and Fgenesh [60] and by comparison with known MHC sequences. Some small pseudogene remnants which did not contribute to evolutionary understanding were neglected.

### Expression pattern of Atlantic salmon MHC class II gene transcripts

Expression of Atlantic salmon class II genes was estimated by transcriptome analysis and by gene-specific RT-PCR analysis. The transcriptome data, using tissues of a one-year old salmon, comprised >70.000 non-redundant contigs and >50 million reads per tissue (Additional file 8: Text S2 (Table TS2-2A)), and data on *MHC* class II expression agree fully with our previous RT-PCR analysis results on *DAA*, *DAB*, *DBA*, *DBB*, *DCA* and *DDA*[29] and our present RT-PCR analysis results on *DCB*, *DEA* and *DEB* for three adult salmon individuals (Additional file 8: Text S2-2B). For details of methods and results see Additional file 8: Text S2-2A and Additional file 8: Text S2-2B. The protocol for the ethics and use of the animals was in accordance with the Animal Care at the University of Victoria, and all animal experiments comply with the current laws of Norway.

### Phylogenetic analysis

The alignments of the MHC class II sequences shown in Additional file 10: Text S4 were done manually, based on structural and evolutionary considerations. The evolutionary history of these manually aligned sequences was inferred using the Neighbor-Joining method [61] using MEGA5 software [62]. See Additional file 3: Figure S3 for details.

## Abbreviations

CLIP: Class II-associated invariant chain peptide; MHC: Major histocompatibility complex; MIIC: MHC class II compartment; MYA: Million years ago; TGD: Teleost-specific whole genome duplication; SGD: Salmonid-specific whole genome duplication.

## Competing interests

The authors declare that they have no competing interests.

## Authors’ contributions

JMD, UG, JL and BFK performed experiments and analysis. JMD, UG and KH wrote the paper. All authors read and approved the final manuscript.

## Supplementary Material

Additional file 1: Figure S1 Phylogeny of relevant species. Click here for file

Additional file 2: Figure S2 Teleost fish and gar MHC class II genomic regions. Click here for file

Additional file 3: Figure S3 Phylogenetic trees of β1, α2 and β2 domains. Click here for file

Additional file 4: Table S1 MHC class II loci and their syntenic regions in selected teleosts. Click here for file

Additional file 5: Table S2 MHC class II gene Ensembl IDs, Ensembl genomic locations, and number of matching cDNA reports in GenBank non-specific datasets, for zebrafish, stickleback, medaka, Fugu, Tetraodon, tilapia, and spotted gar. Click here for file

Additional file 6: Table S3 Conservation pattern of residues which contribute to the hydrogen-bond network between classical MHC class II molecules and the backbone of peptide ligands. Click here for file

Additional file 7: Text S1 Deduced MHC class II amino acid sequences of teleost fish and gar from Ensembl database and matching GenBank cDNA reports. Click here for file

Additional file 8: Text S2 Atlantic salmon (*Salmo salar*) MHC class II sequences, transcripts and genomic regions. Click here for file

Additional file 9: Text S3 Discussion of potential polymorphism of MHC class II genes in selected teleosts and comparison with previous studies. Click here for file

Additional file 10: Text S4 MHC class II domain sequence alignments. Click here for file
