# Supplementary material for: Comprehensive analysis of MHC class II genes in teleost fish genomes reveals dispensability of the peptide-loading DM system in a large part of vertebrates
Source: BMC Evol Biol. 2013 Nov 26;13:260. doi: 10.1186/1471-2148-13-260 (PMC4219347; doi:10.1186/1471-2148-13-260)
Supplement: Additional file 1: Figure S1 — Phylogeny of relevant species. [file 1471-2148-13-260-S1.pdf]

Supplementary figure 1 (Fig. S1): Phylogeny of relevant species

(detailed version of main text Fig. 1 with provision of references)

Figure legend: Above the figure a timescale is depicted in millions of years ago (MYA). The tree is a gross summary of references 1-9 listed below, with reference [5] given particular importance. In our study the relative phylogeny of the species is more important than the exact times of separations, and we did not precisely calibrate time-scales between the referenced studies. Relevant literature for the respective branch knots is indicated by numbers which refer to the literature list below. The dashed line relates to a phylogenetic branch knot on which the referenced literature was not informative regarding the absolute timing of the event. A whole genome duplication event in a teleost ancestor (TGD), and a whole genome duplication event early in the salmonid lineage (SGD), are indicated in red font.

References are:

(1) Benton M, Donoghue PCJ (2007) Paleontological evidence to date the tree of life. *Mol Biol Evol* 24(3):26–53.

(2) Zhu M, Yu X (2009) Stem sarcopterygians have primitive polybasal fin articulation. *Biol Lett* 5(3):372-375.

(3) Inoue JG, Miya M, Venkatesh B, Nishida M (2005) The mitochondrial genome of Indonesian coelacanth *Latimeria menadoensis* (Sarcopterygii: Coelacanthiformes) and divergence time estimation between the two coelacanths. *Gene* 349:227–235.

(4) Azuma Y, Kumazawa Y, Miya M, Mabuchi K, Nishida M (2008) Mitogenomic evaluation of the historical biogeography of cichlids toward reliable dating of teleostean divergences. *BMC Evol Biol* 8:215.

(5) Near TJ, Eytan RI, Dornburg A, Kuhn KL, Moore JA, Davis MP, Wainwright PC, Friedman M, Smith WL (2012) Resolution of ray-finned fish phylogeny and timing of diversification. *Proc Natl Acad Sci U S A*109 (34) 13698-13703

(6) Yamanoue Y, Miya M, Venkatesh B, Nishida M (2006) The mitochondrial genome of spotted green pufferfish *Tetraodon nigrovidis* (Teleostei: Tetraodontiformes) and divergence time estimation among model organisms in fishes. *Genes Genet Syst* 81(1):29-39.

(7) Mikawa N, Utoh T, Horie N, Okamura A, Yamada Y, Akazawa A, Tanaka S, Tsukamoto K, Hirono I, Aoki T (2006) Cloning and characterization of vitellogenin cDNA from the common Japanese conger (*Conger myriaster*) and vitellogenin gene expression during ovarian development. *Comp Biochem Physiol Part B* 143(4):404–414.

(8) Crête-Lafrenière A, Weir LK, Bernatchez L (2012) Framing the Salmonidae family phylogenetic portrait: a more complete picture from increased taxon sampling. *PLoS One* 7(10):e46662.

(9) Nelson JS (2006) Fishes of the world. Fourth Edition (ISBN 0-471-25031-7). John Wiley & Sons, Inc. New York.

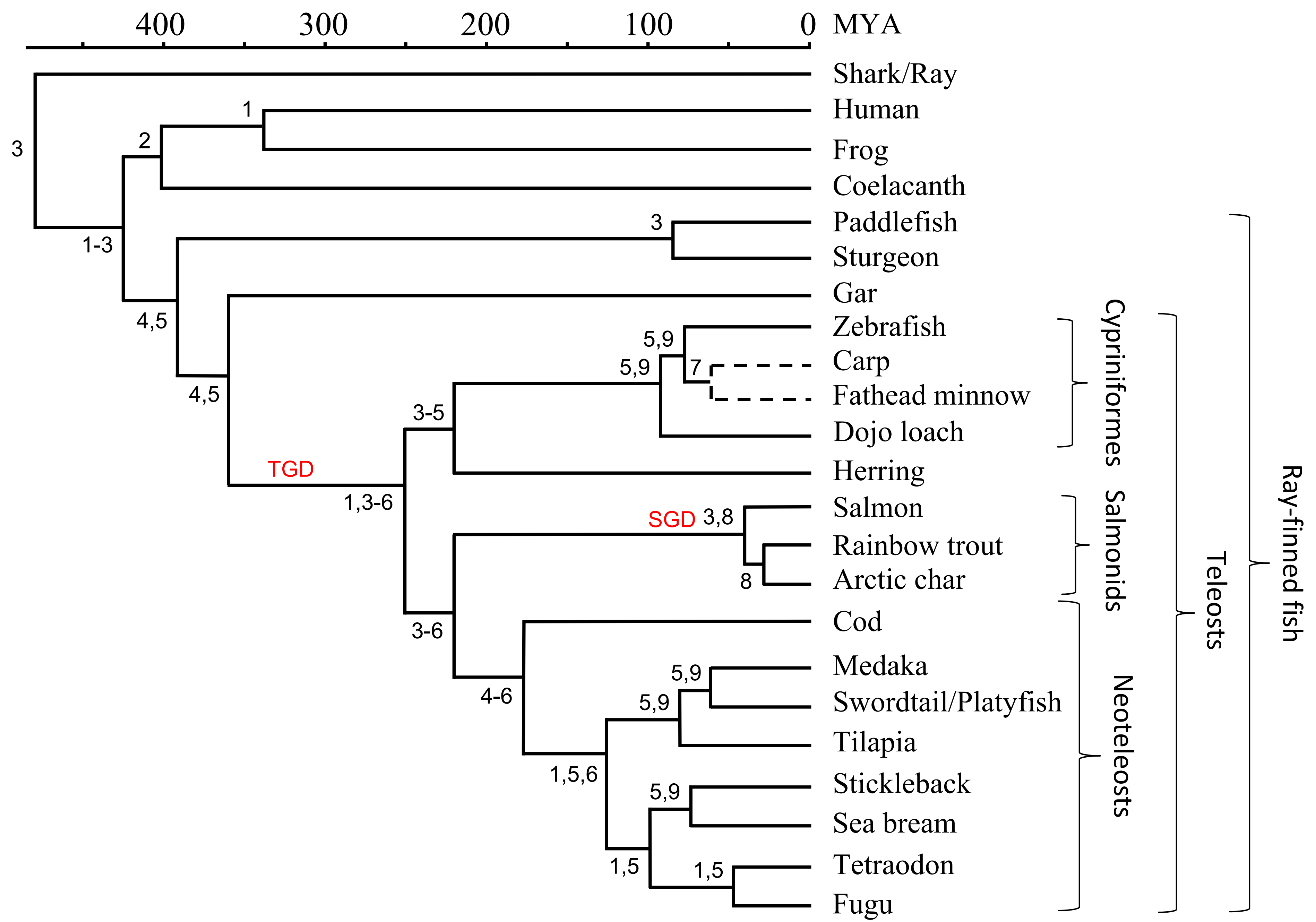

Fig.S1 Species phylogeny
