# Supplementary material for: Comprehensive analysis of MHC class II genes in teleost fish genomes reveals dispensability of the peptide-loading DM system in a large part of vertebrates
Source: BMC Evol Biol. 2013 Nov 26;13:260. doi: 10.1186/1471-2148-13-260 (PMC4219347; doi:10.1186/1471-2148-13-260)
Supplement: Additional file 2: Figure S2 — Teleost fish and gar MHC class II genomic regions. [file 1471-2148-13-260-S2.pdf]

Supplementary figure 2 (Fig. S2): Teleost fish and gar MHC class II genomic regions

(Schematic view with details of the scaffolds shown in main text Fig. 2)

**Table of Contents**	**Page**
Atlantic salmon (*Salmo salar*) MHC class II regions	2
Zebrafish (*Danio rerio*) MHC class II regions	3
Stickleback (*Gasterosteus aculeatus)* MHC class II regions	4
Medaka (*Oryzias latipes*) MHC class II regions	5
Fugu (*Takifugu rubripes*) MHC class II regions	6
Tetraodon (*Tetraodon nigroviridis*) MHC class II regions	7
Tilapia (*Oreochromis niloticus*) MHC class II regions	8
Spotted gar (*Lepisosteus oculatus*) MHC class II regions	11

Figure legend: The genomic surroundings of MHC class II genes in eight fishes are shown with genes represented by blocks. Data were derived from the Ensembl database, except those of Atlantic salmon (Additional file 8, Text S2). Several genes flanking the MHC class II genes were identified using the Ensembl genome browser and, in some cases, gene prediction software. Identities of all genes without Ensembl annotation were investigated by blasting their deduced products against GenBank. When identity was uncertain, phylogenetic analysis (Neighbor Joining method) was performed. Sequences with low but still convincing sequence identity and members of complex gene families were given the name extension "–like" (_L), while those without convincing match were defined as not determined (n.d.) and sometimes omitted for space purposes. For identifying genes in the depicted Atlantic salmon *Mhc* regions we also compared with previous analyses of this region in trout [main text reference 32] and medaka [main text reference 33]. Names of genomic regions and MHC genes used in this study often are simplified references to species and Ensembl scaffolds/contigs or linkage groups/chromosomes (e.g. O845 A for the MHC class II *A* gene on *Oreochromis niloticus* scaffold GL831845), with details given vertically. For precise location of individual MHC genes see Additional file 5, Table S2. Yellow arrows indicate availability of information on extensions of the depicted region fragments, while green stars indicate absence of such information. Syntenic regions are boxed dark green (synteny group S1), lime green (synteny group S2) or pink (synteny group S3) and individual genes supporting region synteny are depicted by the same color (see also Additional file 4, Table S1). Please be aware that some gene families such as the zinc finger protein family (ZNF) can be extensively dispersed throughout the genome and that their shared presence is not a good indication for region synteny. The tilapia O845 and O18 regions are boxed with a dashed pink line to indicate that more information on surrounding genes is needed to be certain that they belong to the S3 synteny group. The stickleback G131 region is boxed with a dotted pink line because it is syntenic with a region on zebrafish Chr.23 which maps close to the S3 synteny group region (Additional file 4, Table S1). Further colorings of individual genes represent as follows: orange= MHCI class II, red= MHC class I, blue= *Mhc* scaffold genes as found in human, gray= other genes. Obvious pseudogenes are marked with ψ, and names of MHC class II gene with evidence of transcription (Additional file 5, Table S2, and Additional file 8, Text S2) are in red font. On page 11 the sequence of the classical(-like) MHC class I sequence encoded by spotted gar scaffold 501 is shown with residues suggesting classical nature in red font. During our investigations the Ensembl platyfish genome database (XipMac4) was released. Because the platyfish MHC class II data are consistent with the MHC class II situation in the other five investigated neoteleosts, they are not shown in the present article.

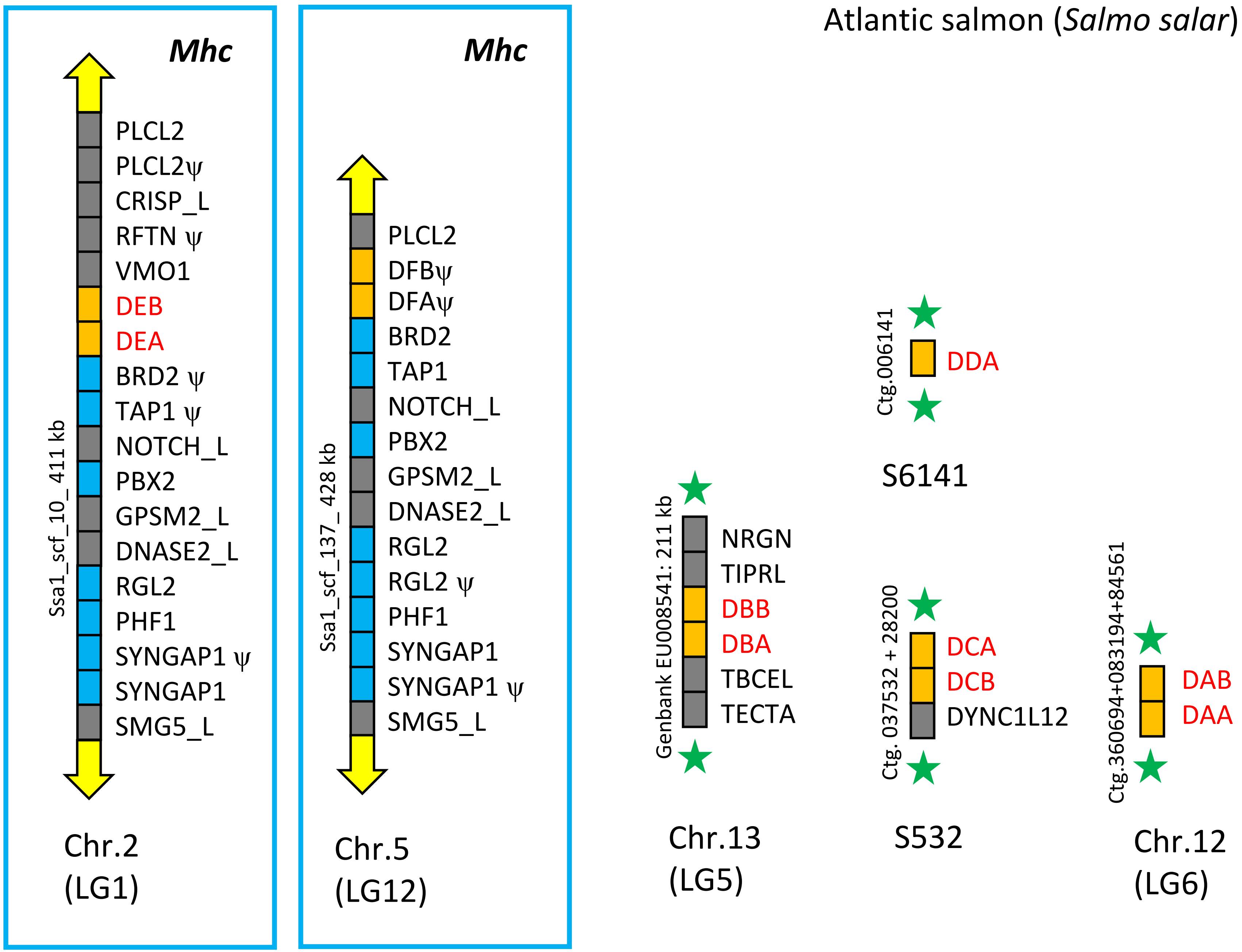

Fig.S2 MHC class II genomic regions

Zebrafish (*Danio rerio*)

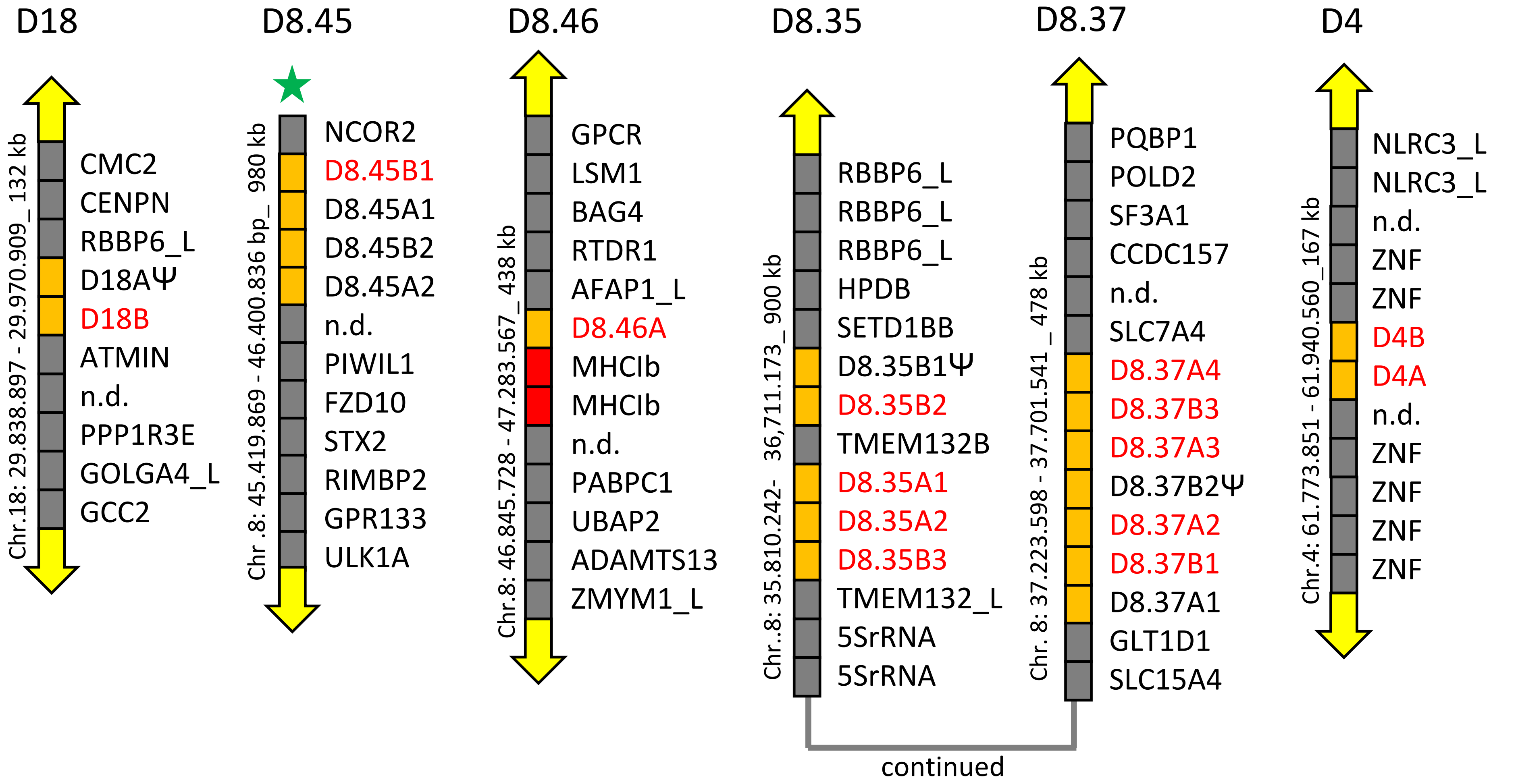

Fig.S2 MHC class II genomic regions

Stickleback (*Gasterosteus aculeatus*)

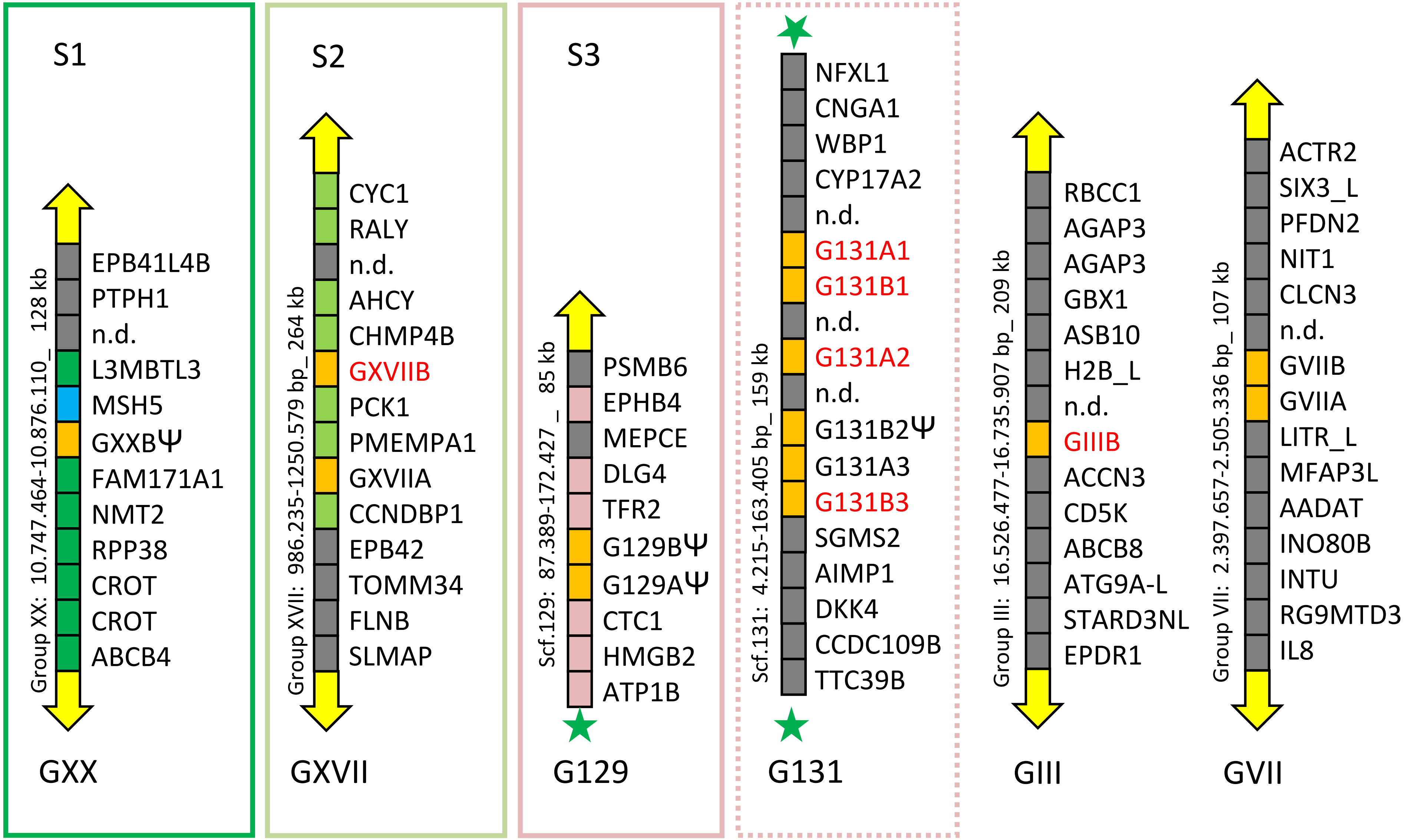

Fig.S2 MHC class II genomic regions

Medaka (*Oryzias latipes*)

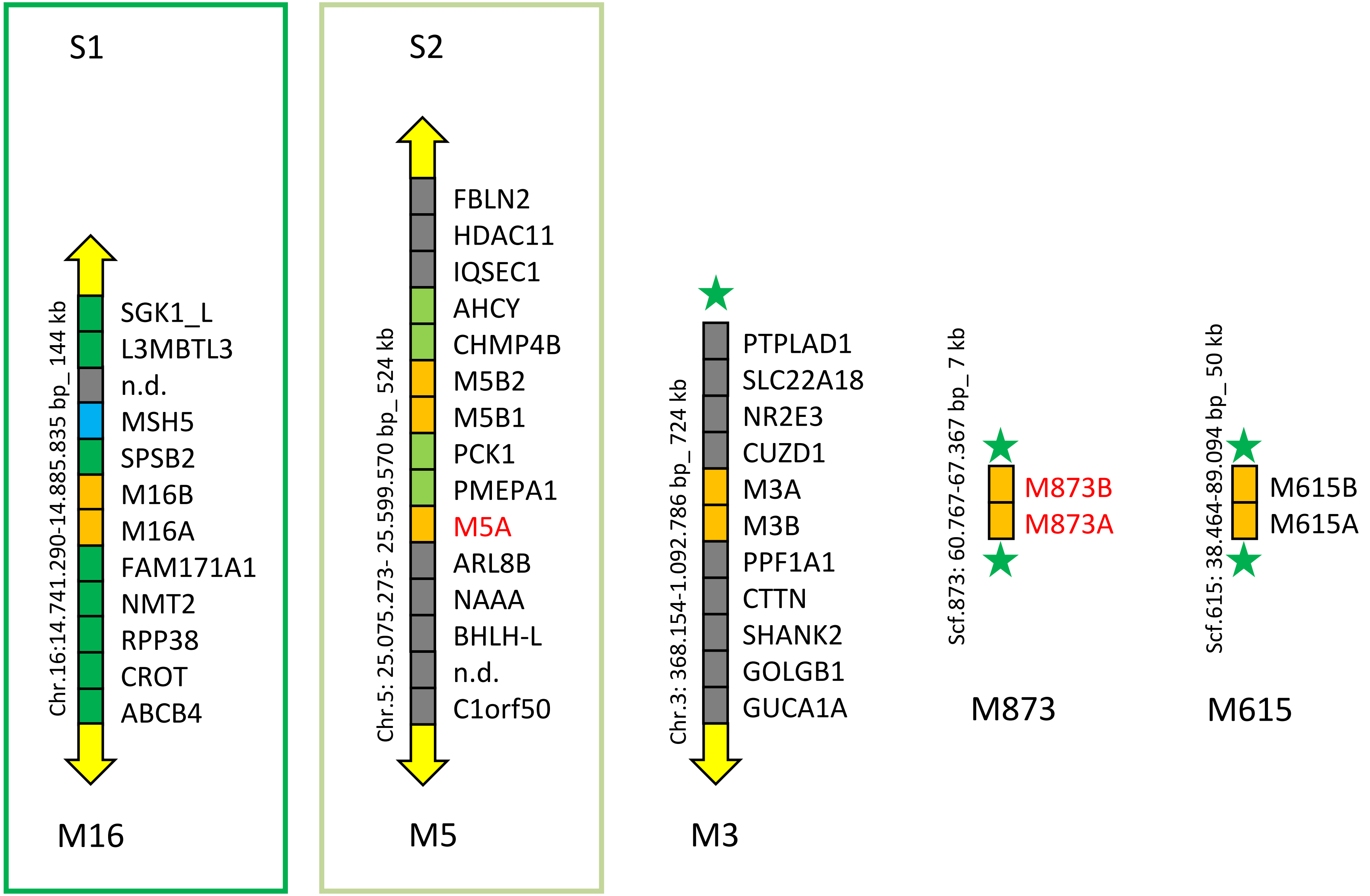

Fig.S2 MHC class II genomic regions

Fugu (*Fugu rubripes*)

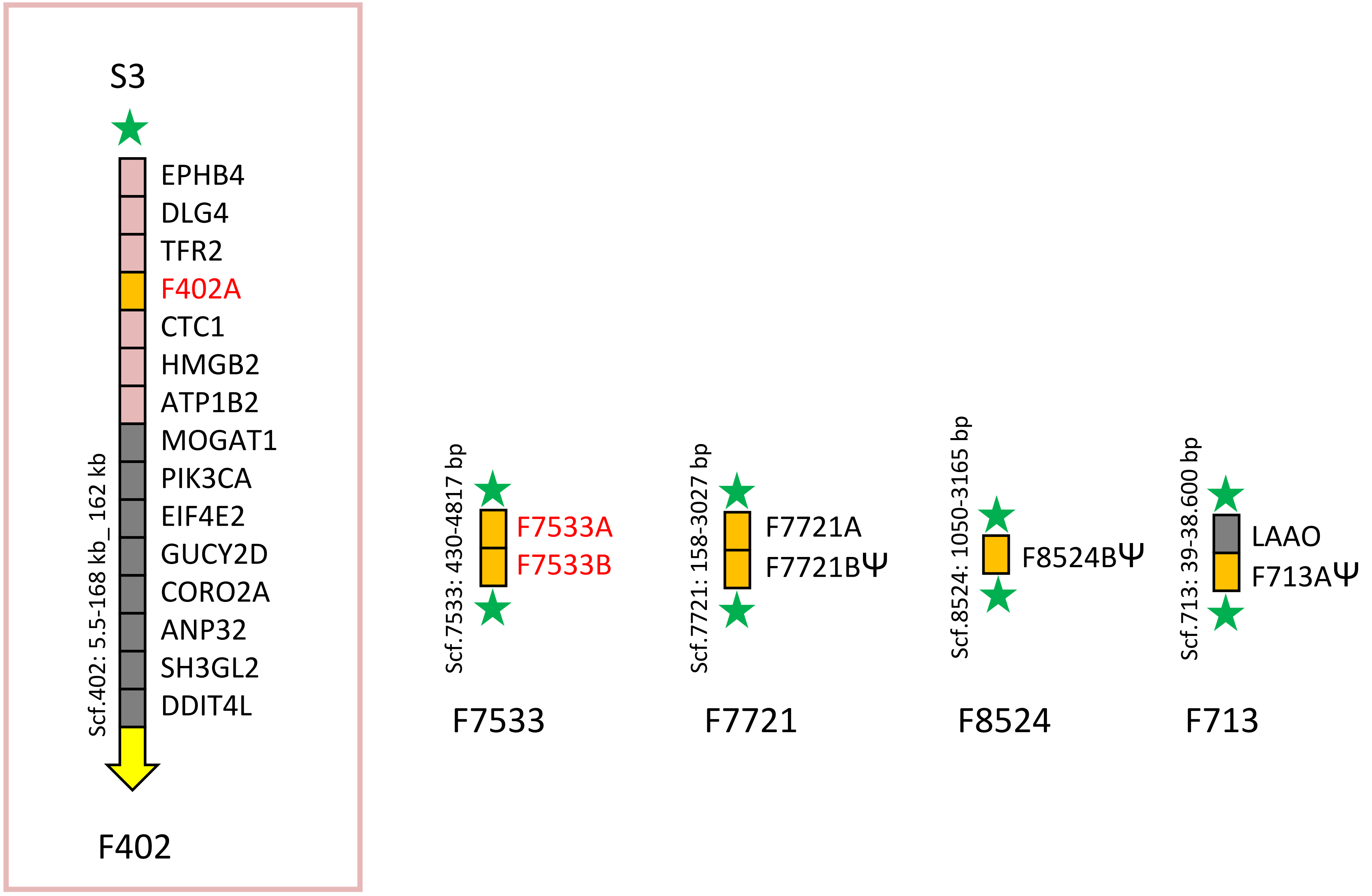

Fig.S2 MHC class II genomic regions

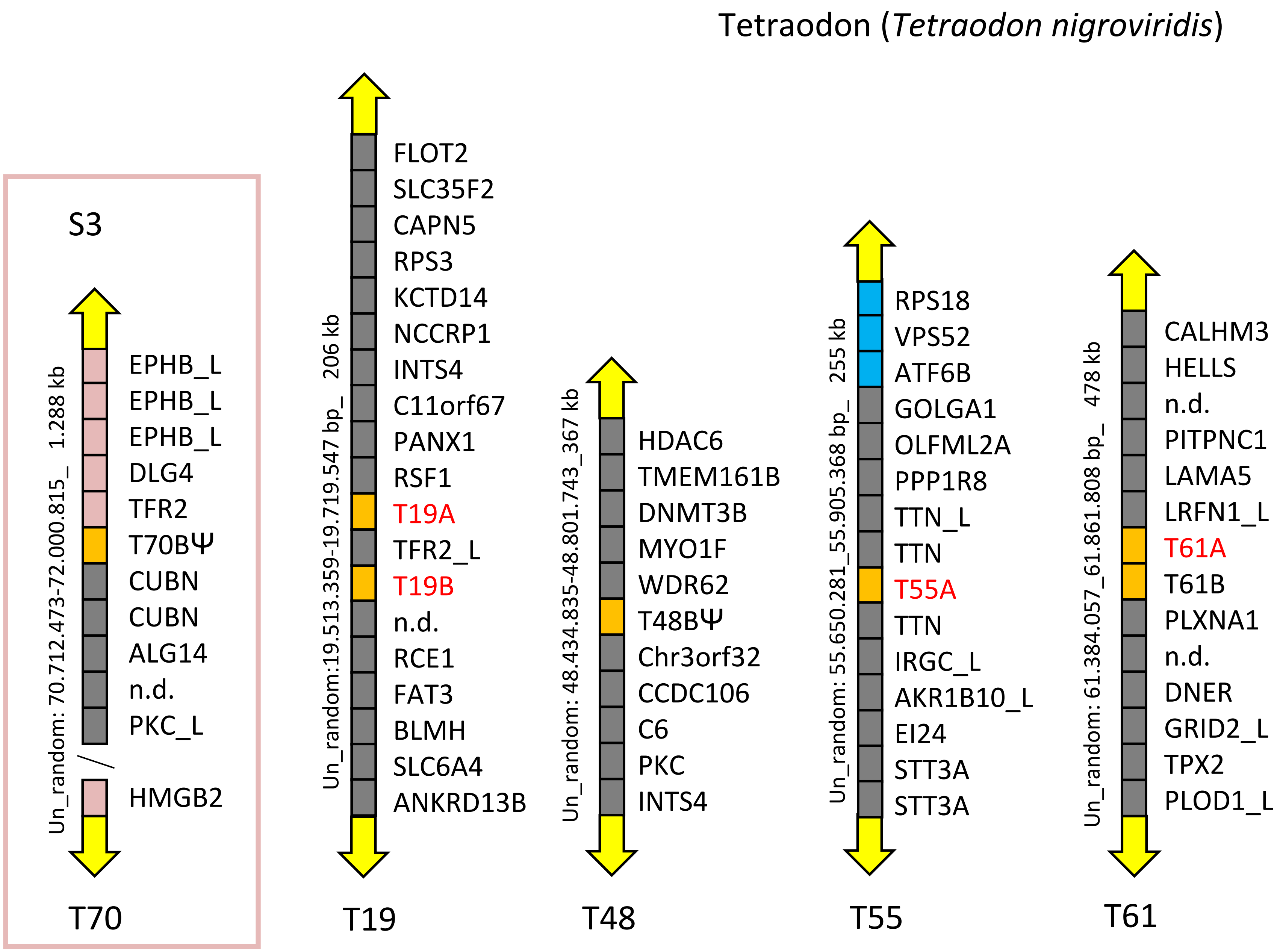
Tetraodon (*Tetraodon nigroviridis*)
S3
Un_random: 70.712.473-72.000.815_ 1.288 kb
EPHB_L
EPHB_L
EPHB_L
DLG4
TFR2
T70BΨ
CUBN
CUBN
ALG14
n.d.
PKC_L
HMGB2
T70
Un_random:19.513.359-19.719.547 bp_ 206 kb
FLOT2
SLC35F2
CAPN5
RPS3
KCTD14
NCCRP1
INTS4
C11orf67
PANX1
RSF1
T19A
TFR2_L
T19B
n.d.
RCE1
FAT3
BLMH
SLC6A4
ANKRD13B
T19
Un_random: 48.434.835-48.801.743_367 kb
HDAC6
TMEM161B
DNMT3B
MYO1F
WDR62
T48BΨ
Chr3orf32
CCDC106
C6
PKC
INTS4
T48
Un_random: 55.650.281_55.905.368 bp_ 255 kb
RPS18
VPS52
ATF6B
GOLGA1
OLFML2A
PPP1R8
TTN_L
TTN
T55A
TTN
IRGC_L
AKR1B10_L
EI24
STT3A
STT3A
T55
Un_random: 61.384.057_61.861.808 bp_ 478 kb
CALHM3
HELLS
n.d.
PITPNC1
LAMA5
LRFN1_L
T61A
T61B
PLXNA1
n.d.
DNER
GRID2_L
TPX2
PLOD1_L
T61

Tilapia (*Oreochromis niloticus*)

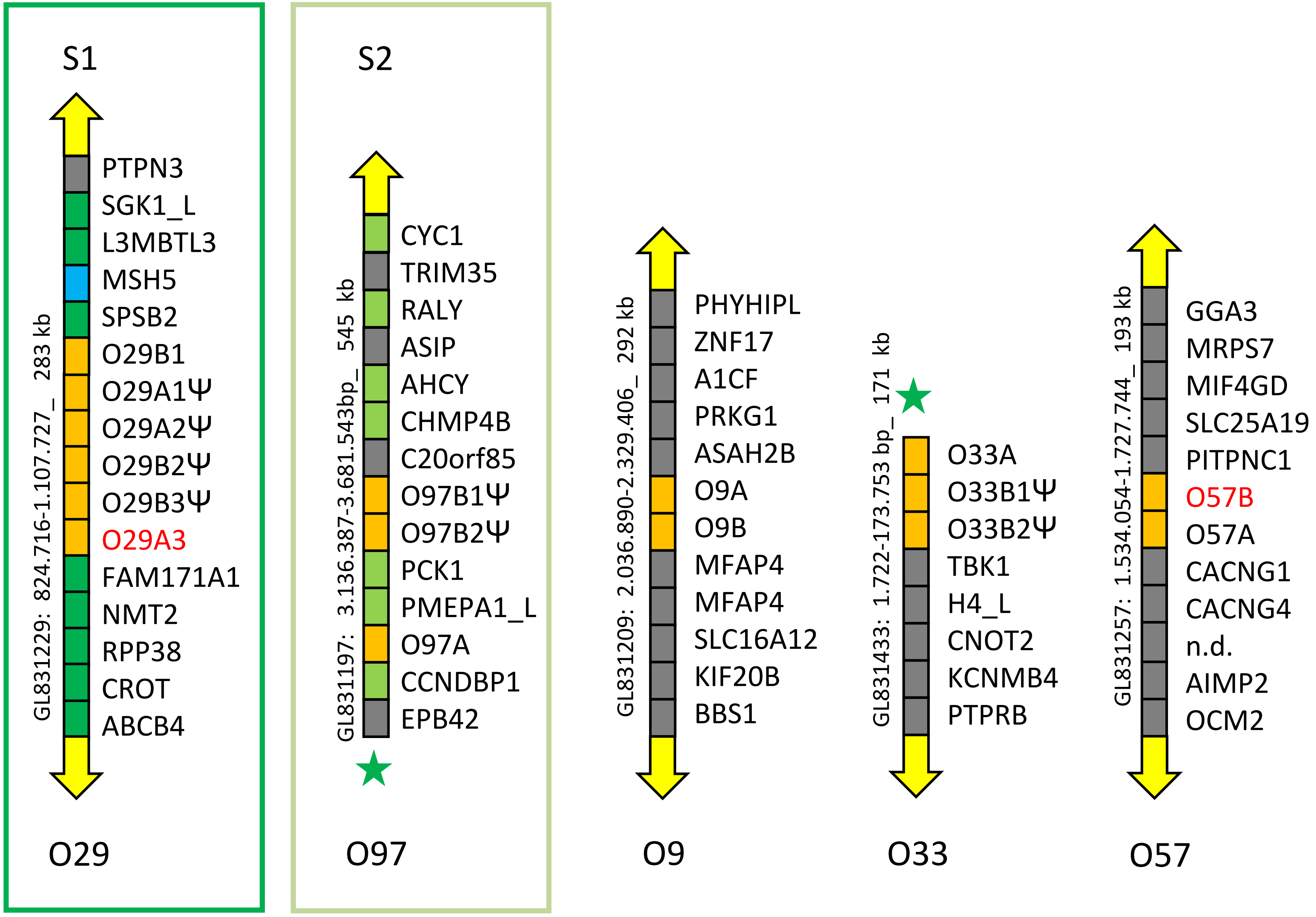

Fig.S2 MHC class II genomic regions

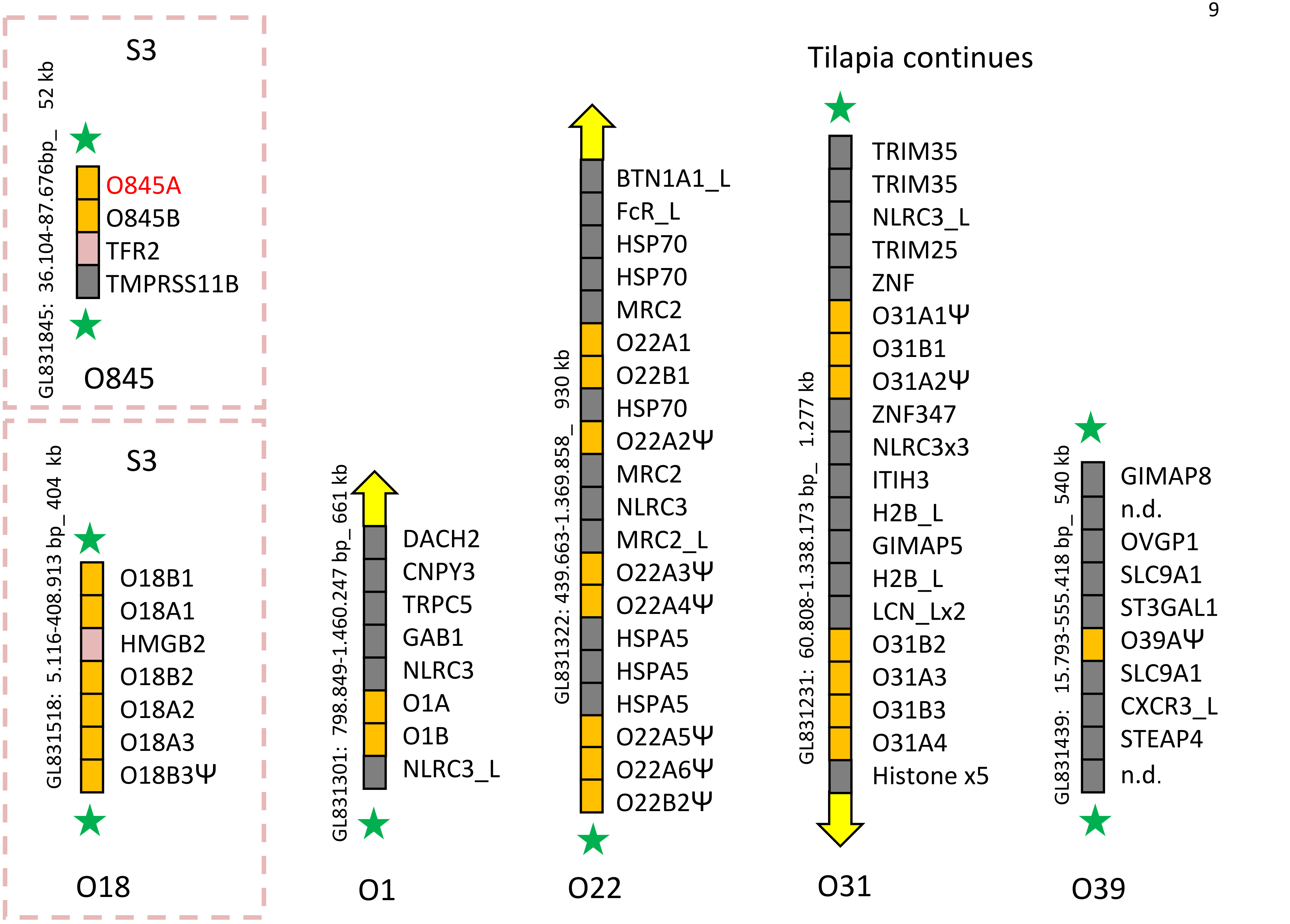

Fig.S2 MHC class II genomic regions

Tilapia continues

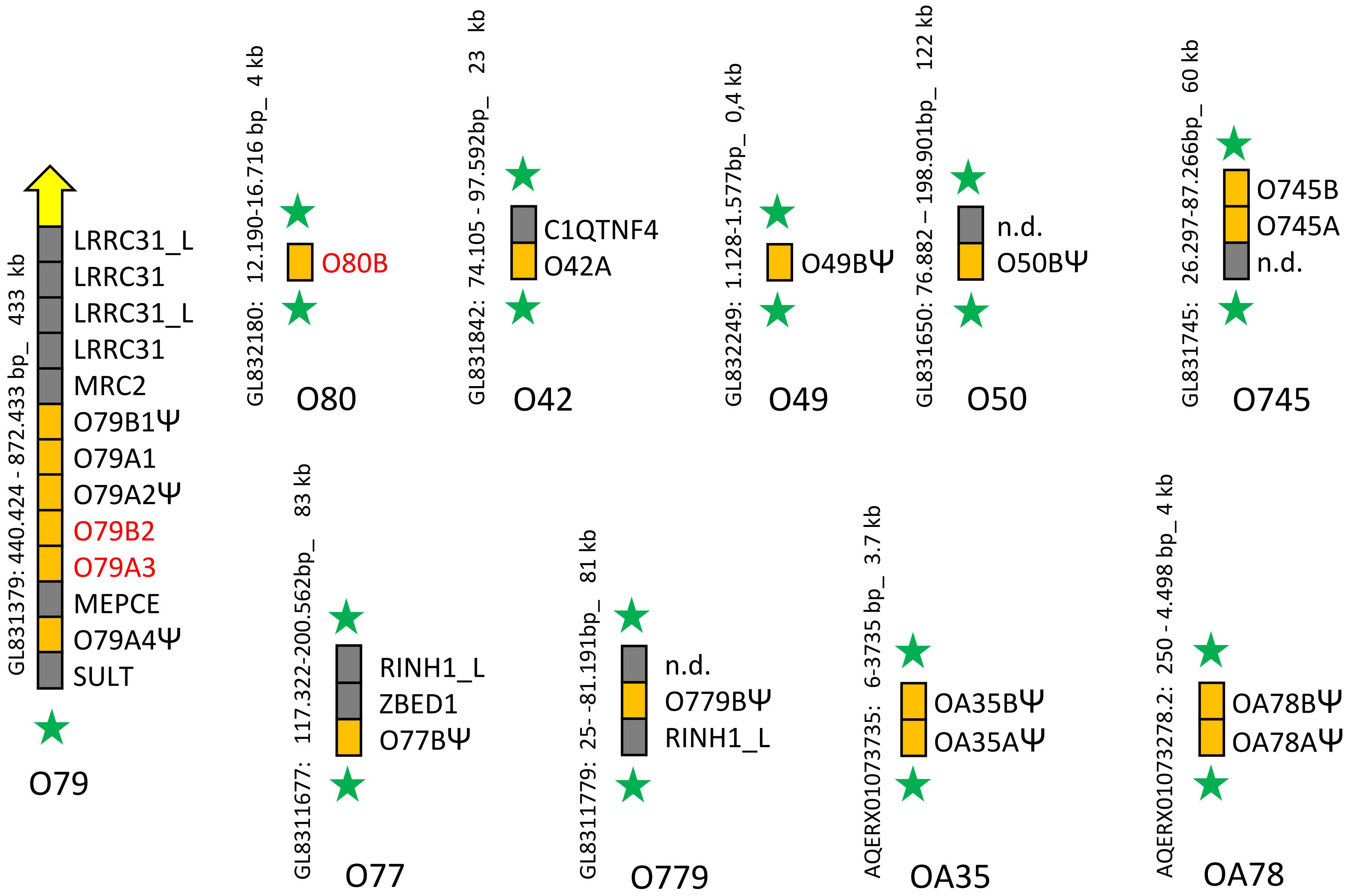

Fig.S2 MHC class II genomic regions

Spotted gar *(Lepisosteus oculatus)*

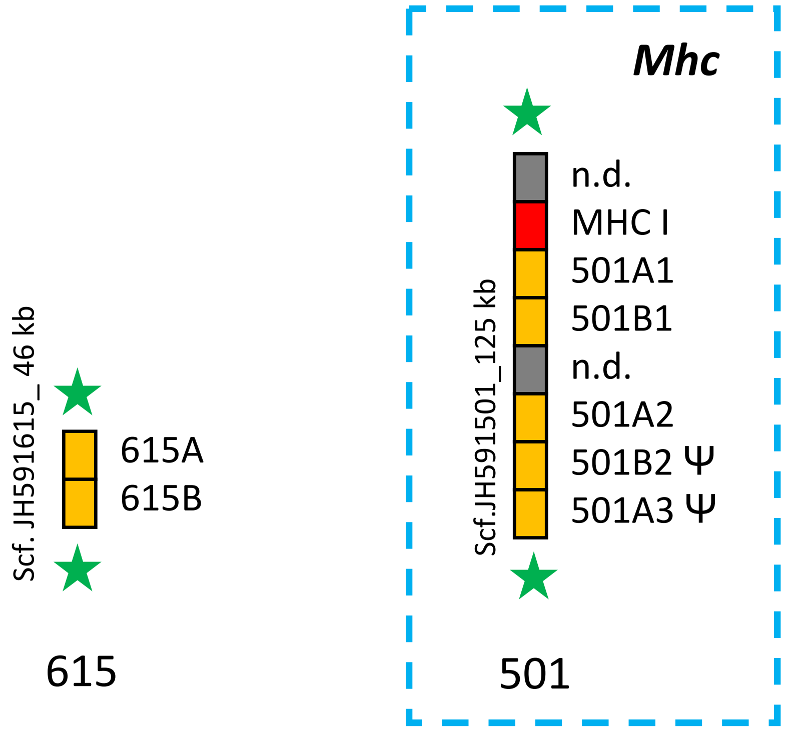

The MHC class I sequence encoded by scaffold 501 (prediction by GENSCAN software) with the “key amino acids” for binding of peptide termini, which are typical for classical molecules, indicated in red:

MRQGRLPEPPLRYNTFVTHSLR**Y**FYTGVTGVRGFPEFTIVGLVDGQEFVHYDSDIKRMIPKTEWIERNEGKD**Y**WDRQTQILIGAS
QVFKTDLVNLPQ**R**FNQSAGVHTSQSMYGCEWDDEDGTTRGFQQEGYDGEDYLVFDLKTLTWVAPTQRAFL**T**KQN**W**DADRAFNEGK
KN**Y**LTQICIEWLKK**Y**VNYGRETLNRRERPQVSVFHKDSGSGSTELTCLATGFFPRDILVSWWRDGQELHEDVDSGEVVPNGDGSF
QVRKRLRVRAGEEHKYSCRVDHTSLEKTIVQHWEPPSLVPIIAAVCAVVALAVIAVVAVVLVRRRKSSVAVSAFSRRAASDDVIG
ALRTVMAAPCRLVSGRAAIGDQSGYSSLHRSPRFTLLF

Fig.S2 MHC class II genomic regions
