# Supplementary material for: Comprehensive analysis of MHC class II genes in teleost fish genomes reveals dispensability of the peptide-loading DM system in a large part of vertebrates
Source: BMC Evol Biol. 2013 Nov 26;13:260. doi: 10.1186/1471-2148-13-260 (PMC4219347; doi:10.1186/1471-2148-13-260)

Supplementary figure 3 (Fig. S3): Phylogenetic trees of β1, α2 and β2 domains

Phylogenetic tree analyses by NJ method for MHC class II domains β1 (Fig. S3*A*), α2 (Fig. S3*B*), and β2 (Fig. S3*C*), as also performed for the MHC class II α1 domains (main text Fig. 4). Numbers behind names relate to references listed in Additional file 10, Text S4. Sequences that are also depicted in main text Fig. 2 are marked by colored shapes indicative for the species: black square for spotted gar, red circle for Atlantic salmon, green circle for zebrafish, violet triangle for stickleback, purple square for medaka, teal triangle for Fugu, gray diamond for Tetraodon, blue diamond for tilapia. Identical shapes, but white, are used for other reported sequences of those species. Background colors distinguish DA, DB and DE group sequences. The dashed blue line divides the DA/DB lineage from the other class II sequences. S1, S2 and S3 indications plus dashed lines refer to products of genes situated in the respective synteny regions (main text Fig. 2). Spotted gar 501A1 shows some interesting similarity with MHC class II of other classes of vertebrates (Fig. S3*B*; see also main text Fig. 4 and Additional file 10, Text S4), but this single sequence does not provide sufficient information for valid discussion.

A consistent finding between the α1, β1, α2 and β2 domain trees is that the DE lineage maps closer to the cartilaginous and tetrapod sequences than do the DA and DB lineages, in agreement with our analysis of conserved residues (main text Fig. 3). As for distinction between DA and DB group sequences, the α1 (main text Fig. 4) and β1 (Fig. S3*A*) phylogenetic trees fully agree with lineage-specific residue motifs found in these domains (main text Fig. 3) and collectively map the DA sequences apart from the DB sequences. In comparison with the α2 and β2 domains, the α1 and β1 domains seem to have a larger number of variable residues with a useful "molecular clock" (which concerns speed and range of amino acid exchanges) for our phylogenetic tree analysis and thus provide better statistics. We therefore estimate that the α1 and β1 trees correctly represent the evolutionary separation of DA and DB. However, for the α2 and β2 domain trees the collective clustering of all DA sequences apart from DB is not found and especially zebrafish DA and DB sequences seem closely related. We have observed before that during evolution MHC immunoglobulin-like domain sequences tend to homogenize in species-lineage specific ways [Dijkstra JM, et al., (2007) A third broad lineage of major histocompatibility complex (MHC) class I in teleost fish. *Immunogenetics* 59(4): 305-321], and we speculate that the genes of zebrafish DA and DB groups which are linked on a single chromosome (main text Fig. 2) may have experienced interlocus recombination, explaining the discrepancies in α1 vs α2 trees and β1 vs β2 trees. The relatively high similarity of zebrafish DB to teleost DA might also be explained, in part, by a possible evolutionary model in which teleost DA lineage originated from tandem duplications of genes ancestral to both DA lineage and extant zebrafish DB lineage within an early teleost equivalent region of zebrafish Chr.8. More extensive analysis of the zebrafish DA vs DB situation is beyond the scope of the present article.

Method for phylogenetic tree analysis:
The evolutionary history was inferred using the Neighbor-Joining method [Saitou N, Nei M (1987) The neighbor-joining method: A new method for reconstructing phylogenetic trees. *Molecular Biology and Evolution* 4(4):406-425]. The bootstrap consensus tree inferred from 500 replicates is taken to represent the evolutionary history of the taxa analyzed [Felsenstein J (1985) Confidence limits on phylogenies: An approach using the bootstrap. *Evolution* 39: 783-791]. Branches corresponding to partitions reproduced in less than 50% bootstrap replicates are collapsed. The tree is drawn to scale, with branch lengths in the same units as those of the evolutionary distances used to infer the phylogenetic tree. The evolutionary distances were computed using the Poisson correction method [Zuckerkandl E, Pauling L (1965) Evolutionary divergence and convergence in proteins. Edited in *Evolving Genes and Proteins* by V. Bryson and H.J. Vogel, pp. 97-166. Academic Press, New York] and are in the units of the number of amino acid substitutions per site. All ambiguous positions were removed for each sequence pair. Evolutionary analyses were conducted in MEGA5 [Tamura K, et al. (2011) MEGA5: Molecular Evolutionary Genetics Analysis using Maximum Likelihood, Evolutionary Distance, and Maximum Parsimony Methods. *Molecular Biology and Evolution* 28(10):2731-2739] using hand-made alignments as shown in Additional file 10, Text S4.

Fig. S3*A*: Phylogenetic tree of β1 domains

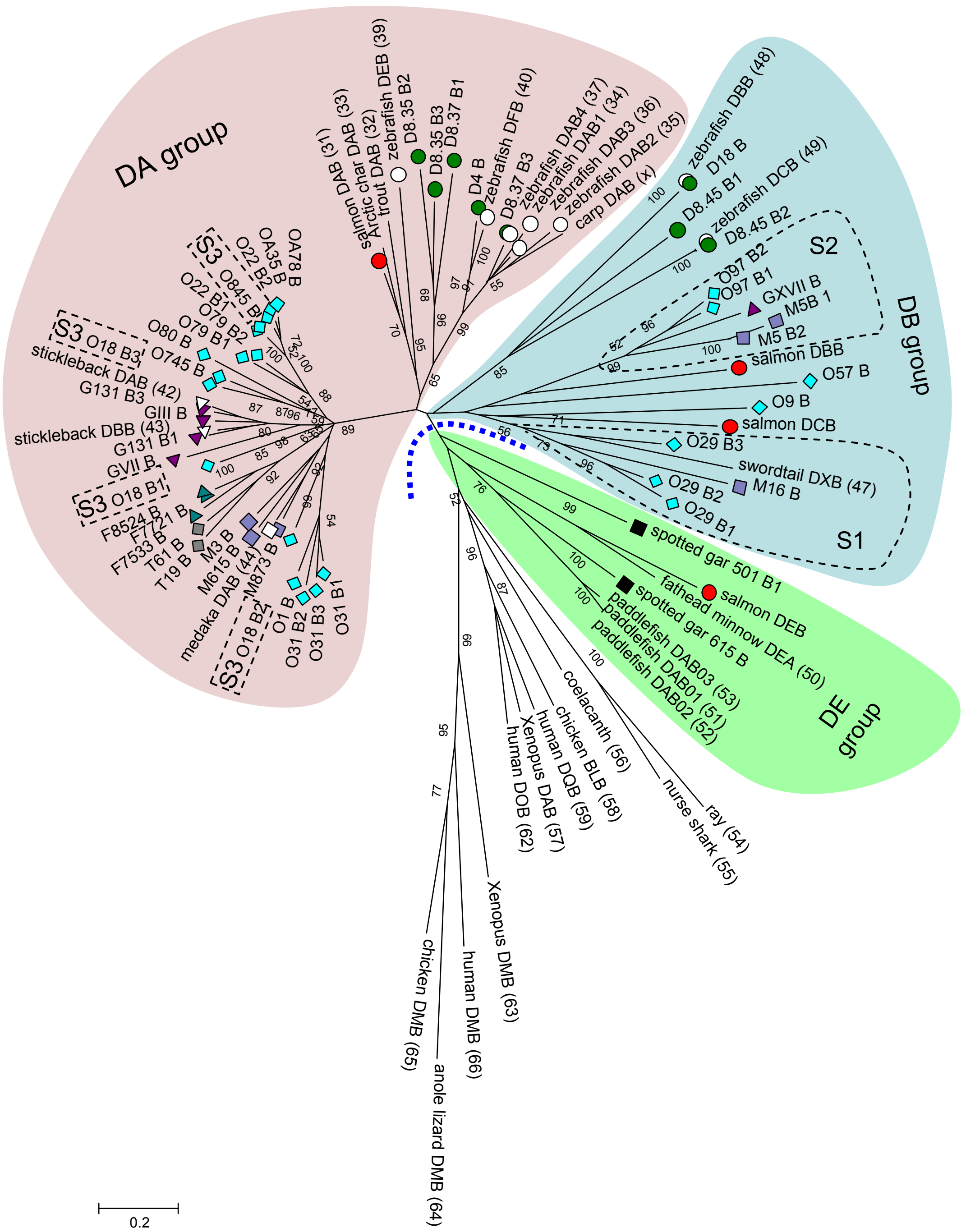

Fig. S3*B*: Phylogenetic tree of α2 domains

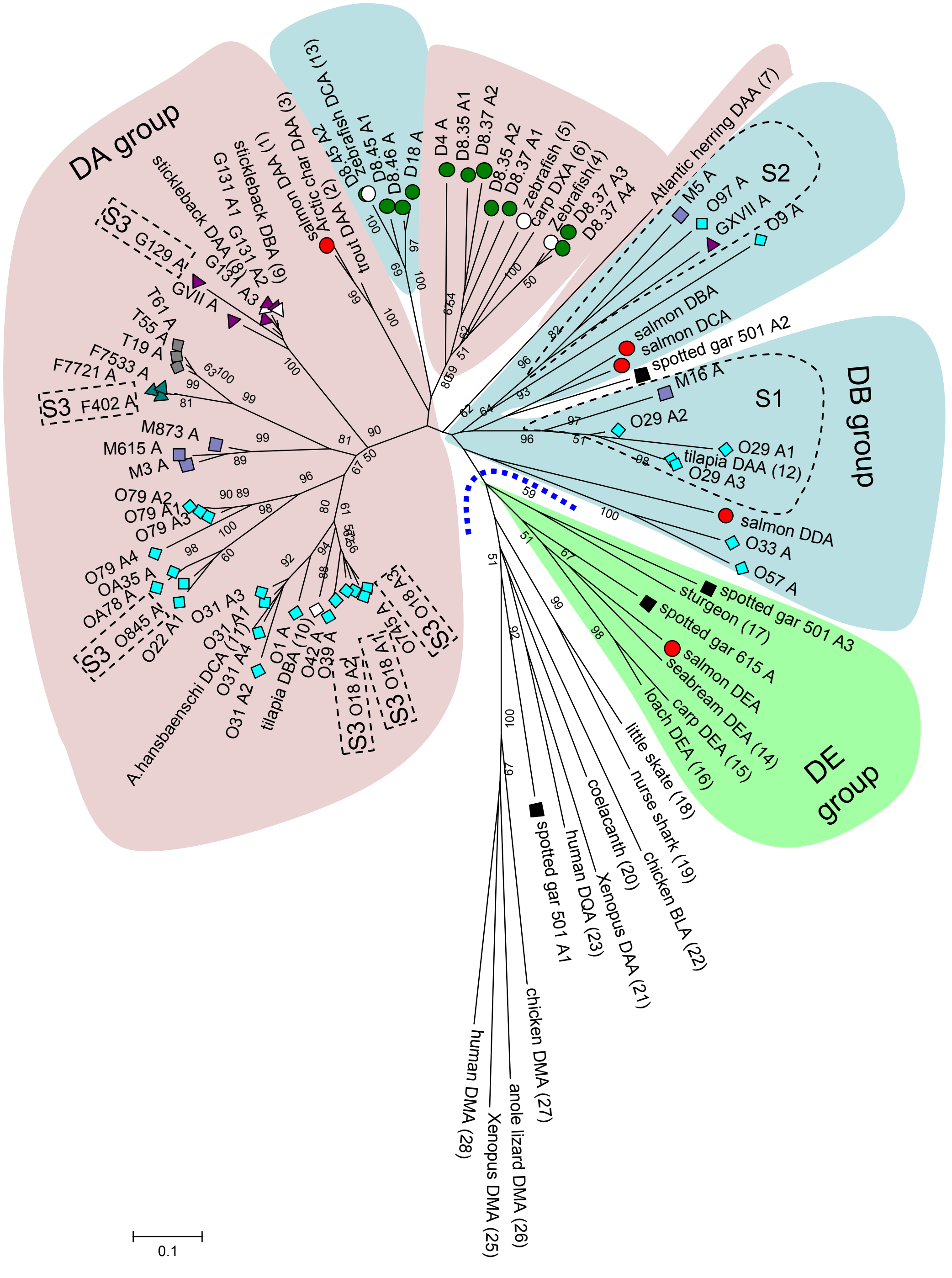

Fig. S3*C*: Phylogenetic tree of β2 domains

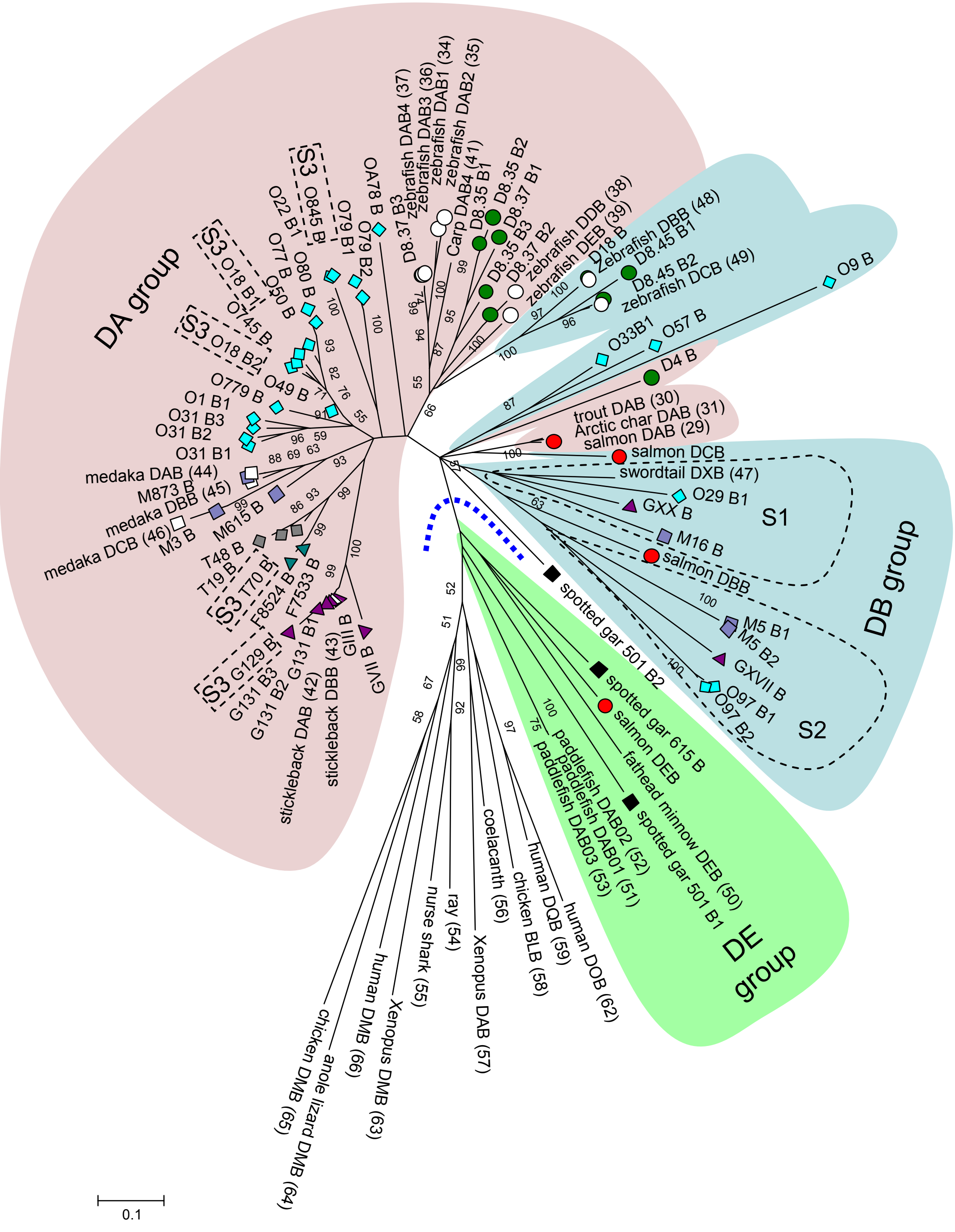

Supplement: Additional file 3: Figure S3 — Phylogenetic trees of β1, α2 and β2 domains. [file 1471-2148-13-260-S3.pdf]
