# Supplementary material for: Comprehensive analysis of MHC class II genes in teleost fish genomes reveals dispensability of the peptide-loading DM system in a large part of vertebrates
Source: BMC Evol Biol. 2013 Nov 26;13:260. doi: 10.1186/1471-2148-13-260 (PMC4219347; doi:10.1186/1471-2148-13-260)
Supplement: Additional file 4: Table S1 — MHC class II loci and their syntenic regions in selected teleosts. [file 1471-2148-13-260-S4.pdf]

Supplementary Table 1 (Table S1). MHC class II loci and their syntenic regions in selected teleosts
(in most cases, the corresponding genomic regions in other teleosts do not harbor MHC class II genes)

**Table of Contents**	Page
Zebrafish MHCclass II regions vs. Stickleback, Medaka, Fugu, Tetraodon and Tilapia	2
Stickleback MHC class II regions vs. Zebrafish, Medaka, Fugu, Tetraodon and Tilapia	3
Medaka MHC class II regions vs. Zebrafish, Stickleback, Fugu, Tetraodon and Tilapia	5
Fugu MHC class II region vs. Zebrafish, Stickleback, Medaka,Tetraodon and Tilapia	6
Tetraodon MHC class II regions vs. Zebrafish, Stickleback, Medaka, Fugu and Tilapia	6
Tilapia MHC class II regions vs. Zebrafish, Stickleback, Medaka, Fugu and Tetraodon	8
Atlantic salmon MHC class II regions vs. Zebrafish, Stickleback, Medaka, Fugu and Tetraodon	10

Zebrafish		Stickleback	Medaka	Fugu	Tetraodon	Tilapia
D4	NLRC3L	n.d.	Chr.18:2.629.600	Scf.2447:4.113	n.d.	n.d.
	Unknown	n.d.	n.d.	n.d.	n.d.	GL831209
	**D4A/B**					
	ZNFx4	n.d.	n.d.	n.d.	n.d.	n.d.
D8.35	RBBP6_L	n.d.	n.d.	Scf.53:898.327	Chr.17:1.653.446	GL831326
	HPDB	GXIII:13.290.215	Chr.9:18.622.367	n.d.	n.d.	GL831172: 1.294.114
	SETD1BB	GXIII:13.282.445	Chr.9:18.612.614	Scf. 18: 453.778	n.d.	GL831172: 1.285.788
	**D8.35B1/B2**					
	TMEM132B	GXIII:13.944.818	Chr.9:19.618.387	Scf.18:1.174.946	UnR:31.307.781	GL831172: 2.128.374
	**D8.35A1/A2/B3**					
D8.37	SLC15A4	GXIII:14.029.539	Chr.9:19.755.023	Scf.18:1.291.525	UnR:31.279.716	GL831172: 2.255.367
	GLT1D1	GXIII:14.049.028	Chr.9:19.792.739	Scf.18: 1.311.022	n.d.	GL831172: 2.282.512
	**D8.37A1-A4**					
	SLC7A4	GXIII:13.350.890	Chr.9:18.704.026	Scf.18:520.320	UnR:32.008.291	GL831172: 1.372.903
	CCDC157	GXIII:13.346.373	n.d.	Scf.18:513.776	n.d.	GL831172:1.363.660
	SF3A1	GXIII:13.339.590	Chr.9:18.692.948	Scf.18:508.693	UnR:32.021.413	GL831172:1.359.416
	POLD2	GXIII:13.220.417	Chr.9:18.518.461	Scf.264:190.621	UnR:31.222.302	GL831172:1.189.044
	PQBP1	GrXII:15.904.368	Chr.7:21.756.808	Scf.226:128.037	Chr.9:9.051.292	GL831137
D8.45	NCOR2	GXIII:14.315.394	Chr.9:20.215.207	Scf.18:1.601.763	UnR:38.312.941	GL831172: 2.634.367
	**D8.45B1-A2**					
	PIWIL1	GXIII:14.130.304	Chr.9:19.921.196	Scf.18:1.419.252	UnR:31.156.268	GL831172: 2.394.350
	FZD10	GXIII:14.111.971	Chr.9:19.890.541	n.d.	UnR:31.090.716	GL831172: 2.371.011
	STX2	GXIII:14.211.776	Chr.9:20.078.621	Scf.18:1.500.768	UnR:65.389.529	GL831172: 2.507.123
	RIMBP2	GXIII:14.143.110	Chr.9:19.943.132	Scf.18:1.436.544	UnR:70.299.887	GL831172: 2.408.450
	GPR133	n.d.	n.d.	n.d.	UnR:65.378.282	GL831172: 2.513.990
D8.46	GPCR	GXIII:13.189.929	Chr.9:18.468.058	Scf.442:56.039	n.d.	GL831172: 1.120.343
	LSM1	GXIII:13.170.146	Chr.9:18.440.734	Scf.442:41.505	UnR:65.942.413	GL831172: 1.115.727
	BAG4	GXIII:13.167.467	Chr.9:18.438.330	Scf.442:38.830	UnR:65.944.659	GL831172: 1.112.594
	RTDR1	Scf.150:112.809	n.d.	Scf.114: 604.297	n.d.	GL831312:66.763
	AFAP1_L	n.d.	n.d.	n.d.	n.d.	GL831312:112,497
	**D8.46A**					
	PABPC1	GXII:650.653	Chr.7	Scf. 75:1.035.824	Chr.9: 6.330.537	GL831137
	UBAP2	GXIII:8.149.971	Chr.9:11.395.215	Scf. 50:58.566	Chr.12:7.056.141	GL831152
	ADAMTS13	GXIII:13.880.401	Chr.9:19.453.416	n.d.	UnR:31.470.879	GL831172: 2.044.605
	ZMYM1_L	Scf.167:64.533	n.d.	n.d.	n.d.	GL832525

Zebrafish continued		Stickleback	Medaka	Fugu	Tetraodon	Tilapia
D18	CMC2	GII: 12.292.363	n.d.	Scf.14: 2.096.814	Chr.5: 4.711.852	GL831150: 1.596.536
	CENPN	GII: 12.288.560	Chr.3: 21.254.929	Scf.14: 2.095.145	Chr.5: 4.713.882	GL831150: 1.599.648
	RBBP6_L	n.d.	n.d.	Scf.53:898.327	Chr.17:1.653.446	GL831326
	D18A/B					
	ATMIN	GII: 12.283.042	Chr.3: 21.247.448	Scf.14: 2.089.379	Chr.5: 4.717.287	GL831150: 1.604.447
	PPP1R3E	n.d.	Chr.3: 21.241.354	n.d.	Chr.5: 4.724.982	GL831150: 1.620.406
	GOLGA4_L	GrVI:6.358.824	n.d.	n.d.	n.d.	GL831252
**Stickleback**		**Zebrafish**	**Medaka**	**Fugu**	**Tetraodon**	**Tilapia**
G129	PSMB6	Chr.23:44.652.957	Chr.8:4.823.628	Scf. 424:143.401	UnR:40.621.744	GL831342:278.199
	EPHB4	Zv9_NA779	n.d.	Scf. 402:5.474	UnR:70.874.307	GL831342:309.748
	MEPCE	Chr.23:44.402.392	n.d.	Scf. 424:147.998	UnR:106.275.040	GL831379:643.206
	DLG4	Chr.23:44.268.701	Scf.1442:25.444	Scf. 402:32.715	UnR:70.843.659	GL831774
	TFR2	Zv9_NA897	n.d.	Scf. 402:37.797	UnR:70.838.346	GL831845
S3	G129B/A			F402A	T70B	
	CTC1	Chr.23:45.325.889	n.d.	Scf. 402:64.273	n.d.	GL832020
	HMGB2	Chr.23:45.378.522	Scf.1967:1.837	Scf. 402:76.862	UnR:71.999.816	GL831518
	ATP1B	n.d.	n.d.	Scf. 402:82.016	UnR:72.002.429	GL831351
G131	CNGA1	Chr.23:44.017.505	UC203:176.762	Scf. 740:169	UnR:56.732.984	GL831493
	WBP1	Chr.23:45.997.451	n.d.	Scf.621:51.885	n.d.	GL831291:714.765
	CYP17A2	Chr.23:45.948.521	Chr.18:279.057	Scf.621:51.885	UnR:98.346.022	GL831291:701.015
	G131A1-B3					
	SGMS2	Chr.23:44.875.565	Chr.18:555.231	Scf. 6036:2.897	UnR:54.726.952	GL831291:570.396
	AIMP1	Chr.23:44.855.060	Chr.18:564.126	Scf. 2313:179	UnR:48.006.042	GL831291:536.611
	DKK4	n.d.	n.d.	n.d.	n.d.	GL831291:526.045
GIII	AGAP3 x 2	Chr.24:35.366.423	Chr.17:28.440.606	Scf.107:119.468	15_R:823.730	GL831134:3.466.401
	GBX1	Chr.24:35.435.371	Chr.17:28.522.709	Scf.107:111.117	15_R:907.856	GL831134:3.411.562
	ASB10	Chr.24:35.444.281	Chr.17:28.526.083	Scf. 107:105.296	15_R:911.579	GL831134:3.404.685
	H2B_L	Chr.24:35.333.778	n.d.	Scf. 107:100.745	15_R:919.098	GL831134:3.399.190
	GIIIB					
	ACCN3	Chr.24:35.130.485	Chr.17:28.644.339	Scf. 107:39.746	15_R:955.175	GL831134:3.284.342
	CD5K	Chr.24:34.834.786	Chr.17:28.671.879	Scf.107:33.167	15_R:978.390	GL831134:3.271.875
	ABCB8	Chr.24:35.043.861	Chr.17:28.677.484	Scf. 107:28.882	15_R:981.254	GL831134:3.262.257
	ATG9A-L	Chr.24:35.064.904	Chr.17:28.686.442	Scf.107:22.479	15_R:986.079	GL831134:3.254.863
	STARD3NL	Chr.2:31.556.322	Chr.17:8.746.037	Scf.468:74.384	15:695.986	GL831411:585.257

Stickleback continued		Zebrafish	Medaka	Fugu	Tetraodon	Tilapia
GVII	SIX3-L	Chr.7:8.229.740	Chr.18:19.163.388	Scf.330:59.482	UnR:1.377.273	GL831189:3.365.738
	PFDN2	Chr.7:8.305.446	Chr.18:19.133.450	Scf.330:47.191	UnR:1.365.975	GL831189:3.337.092
	NIT1	Chr.7:8.323.082	Chr.18:19.074.805	Scf. 330:45.113	UnR:1.361.293	GL831189:3.041.473
	CLCN3	Chr.7:8.339.962	Chr.18:19.074.805	Scf. 330:28.432	UnR:1.349.314	GL831189:3.001.419
	**GVIIB/A**					
	LITR-L	n.d.	n.d.	n.d.	n.d.	n.d.
	MFAP3L	Chr.7:8.490.244	Chr.18:19.068.970	Scf. 330:22.705	UnR:1.337.061	GL831189:2.989.262
	AADAT	Chr.7:8.511.219	Chr.18:19.044.703	Scf.330:17.047	UnR:1.331.803	GL831189:2.973.033
	INO80B	Chr.7:8.529.938	Chr.18:19.039.986	Scf. 330:14.388	UnR:1.329.331	GL831189:2.967.875
	INTU	Chr.7:8.542.822	Chr.18:19.019.460	Scf.330:4.400	UnR:1.321.556	GL831189:2.942.331
	RG9MTD3	Chr.7:8.604.057	Chr.18:19.011.614	Scf.421:112.617	UnR:1.314.590	GL831189:2.830.600
GXVII	CYC1	Chr.6:50.332.650	Scf.1000:10.331	Scf.617:6.363	UnR:16.886.105	GL831197:3.136.390
	RALY	Chr.6:49.973.818	n.d.	n.d.	n.d.	GL831197:3.359.158
	AHCY	Chr.6:49.877.781	Chr.5:25.394.140	Scf. 446:52.707	UnR:16.949.188	GL831197: 3.490.487
	CHMP4B	Chr.8:47.446.286	Chr.5:25.384.960	Scf. 446:47.360	UnR:16.959.941	GL831197: 3.515.710
**S2**	**GXVIIB**		**M5A/B**			**O97A/B**
	PCK1	Chr.6:59.719.127	Chr.5:25.350.583	Scf. 161:355.098	UnR:14.099.792	GL831197: 3.552.052
	PMEMPA1	Chr.6:59.740.415	Chr.5:25.267.810	n.d.	n.d.	GL831197:3.577.543
	**GXVIIA**					
	CCNDBP1	Chr.6:59.708.235	n.d.	n.d.	Chr.11:11.854.054	GL831197:3.657.642
	EPB42	n.d.	n.d.	n.d.	Chr.11:11.848.823	GL831197:3.671.186
	TOMM34	Chr.6:52.177.501	Chr.5:27.351.471	Scf. 6922:1.587	Chr.11:11.844.548	n.d.
GXX	EPB41L4B	Chr.16:28.852.157	Chr.16:14.708.369	Scf.48:199.792	Chr.8:7.617.425	GL831229:808.045
	PTPH1	Chr.16:28.797.936	Chr.16:14.720.036	Scf.48:189.554	Chr.8:7.638.302	GL831229:824.818
	L3MBTL3	Chr.16:28.761.051	Chr.16:14.750.967	Scf. 48:172.685	Chr.8:7.655.586	GL831229: 863.071
	MSH5	Chr.15:38.575.684	Chr.16:14.772.665	Scf. 48:159.703	Chr.8:7.668.232	GL831229: 885.277
**S1**	**GXXB**		**M16A/B**			**O29A/B**
	Fam171A1	Chr.16:30.750.192	Chr.16:14.794.577	Scf.48:143.031	Chr.8:7.679.120	GL831229: 943.374
	NMT2	Chr.16:30.806.591	Chr.16:14.816.398	Scf.48:136.977	Chr.8:7.692.718	GL831229: 970.929
	RPP38	Chr.16:30.820.734	Chr.16:14.825.191	Scf.48:133.649	Chr.8:7.698.737	GL831229: 980.495
	CROT x 2	Chr.16:30.830.213	Chr.16:14.828.029	Scf.48:124.127	Chr.8:7.700.349	GL831229:983.823
	ABCB4	Chr.16:30.856.980	Chr.16:14.850.646	Scf.48:103.925	Chr.8:7.716.939	GL831229:1.060.640

Medaka		Zebrafish	Stickleback	Fugu	Tetraodon	Tilapia
M3	PTPLAD1	Chr.18:18.762.788	GII: 19.620.980	Scf.1: 1.145.009	Chr.5: 12.249.251	GL831285:331.108
	SLC22A18	Chr.7:40.930.516	GII: 19.628.202	Scf. 1: 1.150.247	n.d.	GL831285:346.180
	NR2E3	n.d.	GII: 19.644.637	Scf.1: 1.163.408	Chr.5: 12.235.809	GL831285:385.728
	CUZD1	Chr.18:4.356.366	GII: 21.510.201	n.d.	Chr.5: 12.893.148	GL831133
	**M3A/B**					
	PPF1A1	n.d.	GII: 22.859.068	Scf.1: 288.852	Chr.5: 12.899.435	GL831252:2.078.984
	CTTN	Chr.18:49.869.166	n.d.	Scf. 1: 279.900	Chr.5: 12.916.554	GL831252: 2.028.419
	SHANK2	Chr.24:39.973.233	GII: 22.895.911	Scf. 1: 212.019	Chr.5: 12.924.700	GL831252: 1.976.473
	GOLGB1	n.d.	GII: 22.996.403	Scf.1: 161.425	Chr.5: 12.966.563	GL831252:1.616.213
	GUCA1A	n.d.	GII: 23.020.314	Scf.1: 158.175	Chr.5: 12.986.897	GL831252:1.580.982
M5	HDAC11	Chr.11:28.072.424	GXVII: 11.863.800	n.d.	n.d.	GL831180:2.847.726
	IQSEC1	Chr.11:27.637.741	GXVII: 11.717.942	Scf.445:97.361	Chr.11:11.619.175	GL831180:3.236.385
	AHCY	Chr.6:49.877.781	GXVII:1.107.153	Scf.446:52.707	UnR:16.949.188	GL831197:3.490.487
	CHMP4B	Chr.8:47.459.659	GXVII:1.114.739	Scf.446: 47.360	UnR:16.959.941	GL831197:3.515.710
S2	**M5B1/2**		**GXVIIA/B**			**O97A/B**
	PCK1	Chr.6:59.719.127	GXVII:1.121.929	Scf. 161:355.098	UnR:14.099.792	GL831197: 3.552.052
	PMEPA1	Chr.6:59.740.415	GXVII:1.135.673	n.d.	n.d.	GL831197:3.577.543
	**M5A**					
	ARL8B	Chr.11:36.586.179	GXVII:14.246.498	Scf.79: 957.723	Chr.11:10.309.119	GL831152:2.681.750
	NAAA	n.d.	GXVII:14.241.699	Scf.79: 961.076	Chr.11:10.311.937	GL831152:2.674.234
	BHLH_L	Chr.11:36.600.441	GXVII:14.235.937	Scf.79: 966.481	Chr.11:10.317.107	GL831152:2.653.753
	C1orf50	Chr.11:40.340.778	GXVII:11.660.012	Scf.467: 45.344	Chr.11:11.582.596	GL831180:4.339.411
M16	SGK1_L	Chr.16:28.781.210	n.d.	Scf.48:182.956	Chr.8:7.648.850	GL831229:853.361
......	L3MBTL3	Chr.16:28.761.051	GXX: 10.781.656	Scf.48:172.685	Chr.8:7.655.586	GL831229:863.080
	MSH5	Chr.15:38.580.110	GXX:10.800.083	Scf. 48:159.703	Chr.8:7.668.232	GL831229: 885.277
	SPSB2	Chr.23:44.581.024	n.d.	Scf. 48:157.643	n.d.	GL831229: 899.420
S1	**M16A/B**		**GXXB**			**O29A/B**
	FAM171A1	Chr.16:30.750.192	GXX:10.816.365	Scf. 48:143.031	Chr.8: 7.679.120	GL831229: 943.374
	NMT2	Chr.16:30.806.591	GXX:10.834.107	Scf. 48:136.977	Chr.8: 7.692.718	GL831229: 970.929
	RPP38	Chr.16:30.820.734	GXX:10.842.692	Scf.48:133.649	Chr.8:7.698.737	GL831229:980.498
	CROT	Chr.16:30.830.213	GXX:10.855.978	Scf.48:124.127	Chr.8:7.700.349	GL831229:983.823
	ABCB4	Chr.16:30.856.980	GXX:10.864.560	Scf.48:103.925	Chr.8:7.716.939	GL831229:1.060.640

Table S1 Syntenic MHC class II regions

Fugu		Zebrafish	Stickleback	Medaka	Tetraodon	Tilapia
F402	EPHB4	Zv9_NA779	Scf.129:90.158	n.d.	UnR:70.880.142	GL831342/ GL831183
	DLG4	Chr.23:44.268.701	Scf.129:118.581	Scf.1442:26.444	UnR:70.843.659	GL831774
	TFR2	Zv9_NA897	Scf.129:135.238	n.d.	UnR:70.838.346	GL831845
S3	F402A		G129A/B		T70B	
	CTC1	Chr.23:45.325.889	Scf.129:147.088	n.d.	n.d.	GL832020
	HMGB2	Chr.23:45.378.522	Scf.129:158.380	Scf.1967:1.837	UnR:71.999.816	GL831518
	ATP1B2	n.d.	Scf.129:164.036	n.d.	UnR:72.002.429	GL831351:515.715
	MOGAT1	Chr23:45.618.635	Scf.129:186.554	n.d.	UnR:35.821.686	GL831520:131.917
	PIK3CA	Chr23:44.356.963	Scf.129:231.307	n.d.	UnR:35.807.769	GL831520:153.849
	EIF4E2	Chr23:44.555.187	Scf.129:225.241	n.d.	UnR:35.802.864	GL831520:166.250
	GUCY2D	n.d.	Scf.129:206.279	Chr.22:22.666.268	UnR:35.796.245	GL831520:179.334
**Tetraodon**		**Zebrafish**	**Stickleback**	**Medaka**	**Fugu**	**Tilapia**
T19	NCCRP1	Chr.5: 38.450.813	GVII:12.647.467	Chr.14:831.206	Scf.188: 260.384	GL831214:2.185.976
	INTS4	Chr.18:47.303.990	GI: 17.421.594	n.d.	Scf.188: 264.634	GL831203:2.473.914
	C11orf67	Chr.18:47.297.135	n.d.	Chr.13:1.586.522	Scf.188: 273.726	GL831203:2.484.650
	PANX1	Chr.15 :2.466.960	GI: 17.134.277	Chr.13: 4.107.311	Scf. 188:277.686	GL831340: 1.018.422
	RSF1	Chr.18:47.273.922	n.d.	Chr.13: 4.094.390	Scf. 188:285.419	GL831340: 1.041.560
	T19A/B					
	RCE1	Chr.7:19.450.362	Scf.47:540.375	n.d.	Scf. 164:71.882	GL831627
	FAT3	Chr.15:39.318.576	Scf.119: 101.294	Chr.13:19.729.310	Scf. 188: 375.455	GL831537
	BLMH	Chr.15:27.983.464	GI: 2.798.666	Chr.13:27.211.283	Scf.188: 412.050	GL831505:143.439
	SLC6A4	Chr.15:27.957.909	GI: 2.782.049	Chr.13:27.183.908	Scf.188: 430.670	GL831505:116.870
	ANKRD13B	Chr.15:27.832.122	GI: 2.735.505	Chr.13:26.072.772	Scf.188: 450.147	GL831505:35.528
T48	HDAC6	Chr.8:7.930.067	Scf.237:44.323	n.d.	Scf.215:32.643	GL831387
	TMEM161B	Chr.5:49.810.772	GXIII:10.349.815	Chr.9:14.558.622	Scf. 4:210.065	GL831329
	DNMT3B	Chr.23:17.131.889	GXII:12.806.093	n.d.	Scf.215:437.938	GL831175
	MYO1F	n.d.	n.d.	n.d.	n.d.	GL831168
	WDR62	Chr.15:5.473.526	G I:217.115	UC149:95.098	Scf. 564:34.643	GL831718
	T48B					
	Chr3orf32	Chr.22:33.629.681	n.d.	n.d.	n.d.	GL831335
	CCDC106	Chr.16:19.704.473	G XX:12.120.567	Chr.16:16.748.136	Scf. 202:226.875	GL831225
	C6	Chr.21:19.623.347	G XIV:10.371.247	Chr.12:27.464.701	Scf. 128:628.595	GL831141
	PKC	n.d.	n.d.	n.d.	Scf.2649:11.948	GL831260
	INTS4	Chr.18:47.303.990	GI:17.421.594	Chr.13:1.577.031	Scf.188:264.634	GL831203

Tetraodon continued		Zebrafish	Stickleback	Medaka	Fugu	Tilapia
T55	RPS18	Chr.19:18.309.668	GX:15.061.664	Chr.11:2.209.205	Scf.69: 1.073.156	GL831741:86.058
	VPS52	Chr.19:18.319.012	GX:15.053.871	Chr.11:2.196.487	Scf.69: 1.076.609	GL831741:71.383
	ATF6B	Chr.19:49.751.546	GX:15.025.766	n.d.	Scf.69: 1.084.874	GL831484:165.401
	GOLGA1	Chr.8:43.506.717	GXIII:887.062	Chr.9:28.086.400	Scf.264:345.428	GL831302:572.628
	OLFML2A	Chr.8:43.320.596	GXIII:834.795	Chr.9: 27.996.857	Scf. 264:314.148	GL831302:655.412
	PPP1R8	Chr.16:5.457.755	GXX:17.783.601	n.d.	Scf. 196:28.838	GL831224
	T55A					
	TTN	Chr.9: 43.806.035	n.d.	n.d.	Scf. 348:15.779	GL831334
	IRGC_L	Chr.19:11.737.390	n.d.	n.d.	Scf. 517:93.715	GL831704
	AKR1B10_L	Chr.18:12.660.381	Scf. 163 :112.139	Chr.6:23.143.763	Scf.241:247.650	GL831139:1.860.773
	EI24	Chr.10:40.609.588	n.d.	Scf.817:62.204	Scf.536:36.913	GL831477:162.054
	STT3A	Chr.10:40.563.412	GVII: 13.275.786	Scf. 817:41.023	Scf.536:42.568	GL831477:175.674
T61	CALHM3	Chr.13:25.634.484	GVI:4.004.487	Chr.15:3.041.522	Scf.52: 47.902	GL831300:17.778
	HELLS	Chr.13:7.618.563	GVI:3.992.608	Chr.15:3.015.081	Scf.52: 51.213	GL831350:663.615
	PITPNC1	Chr.16:15.175.955	GXX:12.254.945	Chr.16:16.541.556	Scf.202:98.250	GL831225:326.753
	LAMA5	Chr.23:9.674.223	GXII:13.049.591	UC90:1.377.780	Scf. 278:117.509	GL831175
	LRFN1_L	Chr.18:48.265.500	GI:17.961.864	Chr.13:823.408	Scf. 795:29.840	GL831203
	T61A/B					
	PLXNA1	Zv9_NA907	GXII:4.743.053	Chr.7:1.999.714	Scf. 118:774.981	GL831205
	DNER	Chr.18:45.108.870	GI:5.694.625	Chr.13:22.196.384	Scf. 144:225.973	GL831147
	GRID2_L	n.d.	n.d.	n.d.	n.d.	GL831176:2.076.408
	TPX2	Chr.23:32.267.153	GXII: 18.203.851	UC90:938.627	Scf.220:353.137	GL831283:1.622.658
	PLOD1_L	Chr.8:50.009.104	GXII: 18.206.113	UC90:942.034	Scf.220:339.048	GL831283:1.627.683
T70	EPHB_L x 3	Zv9_NA779	Scf.129: 90.158	Scf.3830:2.187	Scf.402: 5.474	GL831342:309748
	DLG4	Chr.23:44.268.701	Scf.129:118.581	Scf. 1442:26.444	Scf. 402:32.715	GL831774
	TFR2	ZV9_NA897	Scf.129:135.238	n.d.	Scf. 402:37.797	GL831845
S3	T70B		G129A/B		F402A	
	CUBN x 2	Chr.24:33.276.866	Scf.95: 388.665	UC236:1.438.620	Scf.547:85.662	GL831320:423.828
	ALG14	Chr.24:31.321.583	n.d.	Chr.20:3.080.376	Scf.547:47.819	GL831383:725.870
	PKC_L	Chr.15:16.117.502	Scf.95: 249.204	n.d.	Scf.5214/ 4004	GL831383:704.291
	HMGB2	Chr.23:45.378.522	Scf.129:158.380	Scf.1967:1.837	Scf.402:76.862	GL831518

Tilapia		Zebrafish	Stickleback	Medaka	Fugu	Tetraodon
O1	O1A/B					
	GAB1	Chr.1:35.202.952	GVII:966.526	Chr.18:3.063.666	Scf.376:60.687	UnR:17.929.806
	TRPC5	Chr.1:9.662.985	GVII:20.270.942	Chr.18:3.552.469	n.d.	UnR:17.849.569
O9	PRKG1	Chr.12: 6.897.537	GV:12.160.086	n.d.	scf.581	Chr.2:2.611.712
	ASAH2B	Chr.12: 6.801.178	n.d.	UC.222:506.418	Scf.59: 102.191	Chr.18:4.051.504
	O09A/B					
	MFAP4x2	Chr.1: 59.845.805	n.d.	UC.222:537.097	Scf.59: 93.065	Chr.2:2.740.410
	SLC16A12	Chr.12: 18.034.721	GV:12: 216.057	UC.222:495.147	Scf.59: 124.588	Chr.2:2.056.409
O18	O18B1-A1					
S3?	HMGB2	Chr.23:45.378.522	Scf.129:158.380	Scf.1967:1.837	Scf.402:76.862	UnR:71.999.816
	O18B2-B3					
O22	BTN1A1_L	Chr.3: 10.134.571	GVII:439.559	n.d.	n.d.	n.d.
	MRC2	Chr.3: 19.619.398	GXI:6.248.585	Chr.8:5.646.506	Scf.15: 1.761.632	Chr.3:13.433.969
	O22A1_A5					
	HSPA5	Chr.5:2.513.293	GXIV:5.312.943	Chr.12:11.605.128	Scf.44: 42.301	Chr.4:8.303.158
O29	L3MBTL3	Chr.16:28.761.051	GXX:10.781.656	Chr.16:14.750.967	Scf.48:172.685	Chr.8:7.655.586
	MSH5	Chr.15: 38.575.684	GXX:10.800.083	Chr.16:14.772.665	Scf.48:159.703	Chr.8:7.668.232
	SPSB2	Chr.23: 44.581.024	n.d.	Chr.16:14.782.474	Scf.48:157.643	n.d.
S1	O29B1-A3		GXXB	M16A/B		
	FAM171A1	Chr.16:30.750.192	GXX:10.816.365	Chr.16:14.794.577	Scf.48:143.031	Chr.8:7.679.120
	NMT2	Chr.16:30.806.591	GXX:10.834.107	Chr.16:14.816.398	Scf.48:136.977	Chr.8:7.692.718
	RPP38	Chr.16:30.820.734	GXX:10.842.692	Chr.16:14.825.191	Scf.48:133.649	Chr.8:7.698.737
	CROT	Chr.16:30.830.213	GXX:10.845.416	Chr.16:14.828.029	Scf.48:124.127	Chr.8:7.700.349
	ABCB4	Chr.16:30.856.980	GXX:10.864.560	Chr.16:14.850.646	Scf.48:103.925	Chr.8:7.707.197
O33	O33A-B2					
	TBK1	Chr.19:46.929.677	Scf.90:143.888	Chr.5:28.682.754	Scf.79:279.045	UnR:58.576.752
	CNOT2	Chr.25:29.545.555	GXIX/ scf.231	Chr.6:26.455.950	Scf.119/ Scf.708	Chr.13:6.807.528

**Tilapia** continued		**Zebrafish**	**Stickleback**	**Medaka**	**Fugu**	**Tetraodon**
O39	OVGP1	Chr.11/ Chr.23	GXVII:12.535.457	Chr.5:19.295.343	Scf.463,Scf.634	UnR:85.205.727
	ST3GAL1	Chr.19: 5.397.769	GX/ Scf.299	n.d.	n.d.	n.d.
	**O39A**					
	STEAP4	Chr.16: 29.963.111	GXX:10.242.469	Chr.16:14.020.716	Scf.48:600.592	Chr.8:.7.228.457
O57	SLC25A19	Chr.3: 13.530	GXI:11.348.540	Chr.8:13.361.222	Scf.141:501.882	Chr.3:9.152.643
	PITPNC1	Chr.3: 61457.723	GXI:11.317.078	Chr.1:34.110.814	Scf.345:13.813	Chr.3:9.155.523
	**O57B/A**					
	AIMP2	Chr.3: 61.536.570	GXI:11.276.414	Chr.8:13.234.021	Scf.141:439.708	Chr.3:9.206.460
	OCM2	Chr.12:18.772.767	n.d.	Chr.19:2.117.402	Scf.3:2.170.256	Chr.2:6.257.483
O79	MRC2	Chr.3: 19.619.398	GXI:6.248.585	Chr.8:5.646.506	Scf.15:1.761.632	Chr.3:13.433.969
	**O79B1-A3**					
	MEPCE	Chr.5:71.671.171	GVII/ Scf.129	Chr.14:14.838.942	Scf.6:2.463.912	Chr.7:5.229.705
	**O79A4**					
	SULT3	Chr.8:48.713.195	GXII:1.048.504	Chr.7:17.232.422	n.d.	Chr.9:6.550.211
O97	CYC1	Chr.6:50.332.650	GXVII:986.235	Scf.1000:10.331	Scf.617:6,363	UnR:16.886.105
	AHCY	Chr.6:49.877.781	GXVII:1.100.095	Chr.5:25.394.140	Scf.446:52.707	UnR:16.949.188
	CHMP4B	Chr.8 :47.446.286	GXVII:1.110.308	Chr.5:25.384.960	Scf.446:47.360	UnR:16.959.941
**S2**	**O97B1-B2**		**GXVIIA/B**	**M5A/B**		
	PCK1	Chr.6:59.719.127	GXVII:1.121.929	Chr.5:25.350.583	Scf. 161:355.098	UnR:14.099.792
	PMEPA1_L	Chr.6:59.740.415	GXVII:1135673	Chr.5:25.267.810	n.d.	n.d.
	O97A					
	CCNDBP1	Chr.6:59.708.235	GXVII:1.165.796	n.d.	n.d.	Chr.11:11.854.054
O845	**O845A-B**					
**S3?**	TFR2	NA897:1.069	Scf.129:135.238	n.d.	Scf.402:37.797	UnR:70.838.346

Salmon		Zebrafish	Stickleback	Medaka	Fugu	Tetraodon
Chr.2/5	DEB/DFB					
	DEA/ DFA					
	BRD2	Chr.16:52.047.628	GXX:15.423.966	Chr.16:26.286.443	Scf.523:14.385	UnR:16.332.513
	TAP1	Chr.16:52.016.406	GXX:15.435.937	Chr.16:26.297.009	Scf.170:283.968	UnR:51.424.050
	Notch_L	Chr.16:51.985.714	GXX:15.444.113	n.d.	n.d.	UnR:51.421.235
	PBX2	Chr.16:51.950.122	GXX:15.465.731	Chr.16:26.332.781	Scf.35:1.468.422	Chr.8:1.647.368
	GPSM2_L	Chr.16:51.935.066	GXX:15.471.440	n.d.	n.d.	Chr.8:1.652.742
	DNASE2_L	Chr.16:48.401.686	GXX:15.475.753	Chr.16:26.344.217	Scf.35:1.474.634	Chr.8:1.655.888
	RGL2	Chr.16:48.428.502	GXX:15.483.091	Chr.16:26.358.136	Scf.35:1.478.555	Chr.8:1.659.325
Chr.13	NRGN	Chr.5:59.838.779	n.d.	Chr.13:15.288.355	Scf.9:1.826.292	Chr.16:811.072
	TIPRL	Chr.10:95.633	GVII:12.553.815	Chr.14:13.130.762	Scf.99:625.880	UnR:94.342.257
	DBB/DBA					
	TBCEL	Chr.5:66.448.092	GVII:24.894.073	Chr.14:30.375.487	Scf.82:990.257	UnR:57.638.616
	TECTA	Chr.5:66.384.027	GVII:24.910.348	Chr.14:30.399.001	Scf.82:973.246	UnR:52.884.328
S532	DCA/ DCB					
	DYNC1LI2	Chr.19:18.591.795	GX:8.704.088	Chr.3:15.839.156<br>Chr.11:8.463.826	Scf.45:483.586	Chr.21:827.386<br>Chr.5:7.862.069

Pink and gray boxes indicate syntenic regions with matching genes on both (red font) or only one side of the MHC class II genes, respectively. MHC class II genes are depicted in green font and S1-S3 synteny groups in blue. n.d.= no data, Scf.=scaffold, Chr.=chromosome, UnR= Unrandom, UC= ultracontig, R= random. Regions with MHC genes only or highly abundant genes were omitted from the analysis.
