# Supplementary material for: Comprehensive analysis of MHC class II genes in teleost fish genomes reveals dispensability of the peptide-loading DM system in a large part of vertebrates
Source: BMC Evol Biol. 2013 Nov 26;13:260. doi: 10.1186/1471-2148-13-260 (PMC4219347; doi:10.1186/1471-2148-13-260)
Supplement: Additional file 5: Table S2 — MHC class II gene Ensembl IDs, Ensembl genomic locations, and number of matching cDNA reports in GenBank non-specific datasets, for zebrafish, stickleback, medaka, Fugu, Tetraodon, tilapia, and spotted gar. [file 1471-2148-13-260-S5.pdf]

Supplementary Table 2 (Table S2). MHC class II gene Ensembl IDs, Ensembl genomic locations, and number of matching cDNA reports in GenBank non-specific datasets, for zebrafish, stickleback, medaka, Fugu, Tetraodon, tilapia, and spotted gar

**Species/ Name**	**MHC gene_Ensembl ID**	**Genomic location Ensembl**	**EST match alpha 2 and beta 2 domain (Genbank)**
**Zebrafish (*Danio rerio*)**			**ESTs** (#1.488.275)
D4B	ENSDARG00000054921	Chr. 4: 61.874.514-61.880.599	1 EST 100% id_100% cov. (EB769231)
D4A	ENSDARG00000020286	Chr. 4: 61.887.203-61.893.205	14 ESTs 100% id_100% cov. (e.g. CF996478)
D8.35B1ψ	ENSDARG00000093706	Chr. 8: 35.956.336-36.146.143	5' end missing_Pseudogene
D8.35B2	ENSDARG00000053738	Chr. 8: 35.956.359-35.959.768	1 EST > 97% id_100% cov. (CK030151)
D8.35A1	ENSDARG00000053739	Chr. 8: 36.131.728-36.140.054	1 EST > 97% id_100% cov. (DT880928)
D8.35A2	ENSDARG00000055441	Chr. 8: 36.283.500-36.286.998	31 ESTs 100% id_100% cov. (e.g. EH498789)
D8.35B3	ENSDARG00000055447	Chr. 8: 36.288.188-36.289.984	32 ESTs 100% id_100% cov. (e.g. DN896667)
D8.37A1	ENSDARG00000074816	Chr. 8: 37.465.644-37.474.349	<86% id_100% cov.
D8.37B1	ENSDARG00000074510	Chr. 8: 37.476.789-37.482.114	2 ESTs 100% id_100% cov. (e.g. CN023898)
D8.37A2	ENSDARG00000070206	Chr. 8: 37.488.525-37.498.906	2 ESTs 100% id_100% cov. (e.g. EG576857)
D8.37B2 ψ	ENSDARG00000076307	Chr. 8: 37.510.422-37.511.403	5'end missing_Pseudogene
D8.37A3	ENSDARG00000031745	Chr. 8: 37.539.907-37.543.263	50 ESTs 100% id_100% cov. (e.g. EB979084)
D8.37B3	ENSDARG00000079105	Chr. 8: 37.546.100-37.548.453	50 ESTs 100% id_100% cov. (e.g. DT061156)
D8.37A4	ENSDARG00000001832	Chr. 8: 37.574.076-37.576.393	39 ESTs 100% id_100% cov. (e.g. CO353326)
D8.45B1	ENSDARG00000041705	Chr. 8: 45.758,566-45.761.489	1 EST 100% id_100% cov. (CO798204)
D8.45A1	ENSDARG00000079593	Chr. 8: 45.768.098-45.769.605	<88% id_100% cov.
D8.45B2	ENSDARG00000088872	Chr. 8: 45.773.380-45.774.524	<85% id_100% cov.
D8.45A2	ENSDARG00000086294	Chr. 8: 45.778.095-45.779.781	<85% id_100% cov.
D8.46A	ENSDARG00000075932	Chr. 8: 47.078.720-47.080.882	2 ESTs 100% id_100% cov. (e.g. EH450259)
D18B	ENSDARG00000056330	Chr. 18: 29.872.428-29.875.206	6 ESTs > 98% id_100% cov. (e.g. EH441901)
D18A ψ	ENSDARG00000093885	Chr. 18: 29.867.551-29.868.594	5'+ 3' missing_ Pseudogene
**Stickleback (*Gasterosteus aculeatus*)**			**ESTs** (# 276.992)
G129B ψ	No Ensembl gene report	Scf.129_contig12675:25.778-25.611	5'+3' missing; Pseudogene
G129A ψ	No Ensembl gene report	Scf.129_contig12675:26.484-26.221	5'+TM CYT missing_ Pseudogene
G131A1	ENSGACG00000000330	Scf. 131: 52.153-54.217	22 ESTs > 97% id_100% cov. (e.g. DW672198)
G131B1	ENSGACG00000000336	Scf. 131: 56.853-59.742	3 ESTs 100% id_100% cov. (e.g. DV011001)

G131A2	ENSGACG00000000344	Scf. 131: 78.913-81.169	5 ESTs > 97% id_100% cov. (e.g. DW672198)
G131B2 ψ	ENSGACG00000000346	Scf. 131: 96.885-99.355	ORF error_ Pseudogene
G131A3	ENSGACG00000000348	Scf.131: 118.613-120.538	<99% id_97% cov.
G131B3	ENSGACG00000000350	Scf. 131: 121.399-126.270	3 ESTs 100% id_100% cov. (e.g. DV011001)
GIIIB	ENSGACG00000017967	Gr.III: 16.679.542-16.682.223	3 ESTs 100% id_100% cov. (e.g.DV011001)
GVIIB	ENSGACG00000019051	Gr.VII: 2.444.693-2,447,881	<88% id_100% cov.
GVIIA	ENSGACG00000019052	Gr.VII: 2.449.972-2,451,242	<92% id_100% cov.
GXVIIB	ENSGACG00000003680	Gr.XVII: 1.117.113-1.119.770	7 ESTs > 97% id_100% cov. (e.g. DW594610)
GXVIIA	ENSGACG00000003731	Gr.XVII: 1.163.942-1.165.035	<46% id_93% cov.
GXXB ψ	No Ensembl gene report	Gr.XX: 10.813.73-10.814.066	5'+TM CYT missing_ Pseudogene
**Medaka (*Oryzias latipes*)**			**ESTs** (# 666.891)
M3A	ENSORLG00000000021	Chr. 3: 554.841-556.227	<90% id_100% cov.
M3B	ENSORLG00000000025	Chr. 3: 558.225-564.744	<85% id_100% cov.
M5A	ENSORLG00000012794	Chr. 5: 25.231.039-25.236.341	2 ESTS 100% id_100% cov. (e.g. BJ877464)
M5B1	ENSORLG00000012822	Chr. 5: 25.371.618-25.375.673	<38% id_100% cov.
M5B2	ENSORLG00000012826	Chr. 5: 25.378.549-25.379.616	<38% id_100% cov.
M16B	ENSORLG00000009164+	Chr. 16: 14.786.594-14.790.902	<57% id_100% cov.
M16A	ENSORLP00000011499+	Chr. 16: 14.790.903-14.792.142	<50% id_100% cov.
M615B	ENSORLG00000019376	Scf.615: 38.464-43.568	<88% id_100% cov.
M615A	ENSORLG00000019409	Scf.615: 87.448-89.094	<90% id_100% cov.
M873B	ENSORLG00000018856	Scf.873: 60.767-63.324	34 ESTs 100% id_100% cov. (e.g. FS518487)
M873A	ENSORLG00000018887	Scf.873: 65.205-67.367	34 ESTs 100% id_100% cov. (e.g. BJ883759)
**Fugu (*Takifugu rubripes*)**			**ESTS** (# 26.069)
F402A	ENSTRUG00000004342	Scf. 402: 55.188-57.563	1 ESTs >97% id 100% cov. (CA846902)
F713A ψ	No Ensembl gene report	Scf. 713: 89-334	L+3' end missing_Pseudogene
F7533A	ENSTRUG00000017444	Scf. 7533: 430-1.814	1 EST > 97% id_100% cov. (CA846902)
F7533B	ENSTRUG00000017449	Scf. 7533: 2.656-4.187	2 ESTs > 97% id_100% cov. (e.g. CA846190)
F7721A	ENSTRUG00000000833	Scf. 7721: 158-1.461	TM_CYT missing_<97% id_100% cov.
F7721B ψ	No Ensembl gene report	Scf. 7721: 2.626-3.027	3' end missing_Pseudogene
F8524B ψ	ENSTRUG00000002062	Scf. 8524: 1.050-3.165	ORF error_Pseudogene

Table S2 MHC class II gene Ensembl IDs, Ensembl genomic locations, and number of matching cDNAs in GenBank

**Tetraodon (*Tetraodon nigroviridis*)**			**Nucleotide collection (nr/nt)** (# 108.463)
T19A	ENSTNIG00000005593	Chr UnR: 19.583.822-19.591.157	TM CYT missing_3 cDNAs 100% id_100% cov. (e.g. CR731060)
T19B	ENSTNIG00000005592	Chr UnR: 19.597.929-19.598.797	4 cDNAs > 97% id_100% cov. (e.g. CR727057)
T48B ψ	ENSTNIG00000002162	Chr UnR: 48.695.828-48.696.540	5’ end missing_Pseudogene
T55A	ENSTNIG00000000926	Chr UnR: 55.779.303-55.780.575	3 cDNAs > 97% id_100% cov. (e.g. CR731060)
T61A	ENSTNIG00000000531	Chr UnR: 61.617.277-61.620.814	12 cDNAs > 97% id_100% cov. (e.g. CR682088)
T61B ψ	ENSTNIG00000003745	Chr UnR: 61.621.456-61.622.716	ORF error_pseudogene
T70B ψ	ENSTNIG00000004611	Chr UnR: 70.836.268-70.837.419	5’end missing_Pseudogene
**Tilapia (*Oreochromis niloticus*)**			**ESTs** (#120.991) or Nucleotide collection (#82.618)
O1A	ENSONIG00000006325	GL831301.1: 1.431.629-1.433.771	<77% id_100% cov.
O1B	ENSONIG00000006327	GL831301.1: 1.435.653-1.439.547	<88% id_100% cov.
O9A	ENSONIG00000019112	GL831209.1: 2.227.167-2.229.515	<45% id_97% cov.
O9B	ENSONIG00000019113	GL831209.1: 2.230.917-2.233.060	<46% id_94% cov.
O18B1	ENSONIG00000019932	GL831518.1: 4.104-13.309	<90%id_100% cov.
O18B2	ENSONIG00000019937	GL831518.1: 312.391-316.936	<95% id_100% cov.
O18B3 ψ	ENSONIG00000019944	GL831518.1: 404.131-408.913	ORF error_Pseudogene
O18A1	ENSONIG00000019933	GL831518.1: 18.305-20.484	<94% id_100% cov.
O18A2	ENSONIG00000019940	GL831518.1: 317.998-320.025	<92% id_100% cov.
O18A3	ENSONIG00000019943	GL831518.1: 399.821-401.995	<96% id_100% cov.
O22A1	ENSONIG00000012437	GL831322.1: 920.733-935.169	<91% id_100% cov.
O22B1	ENSONIG00000012441	GL831322.1: 945.399-948.614	<69% id_100% cov.
O22A2 ψ	No Ensembl gene report ID	GL831322.1: 1.002.299-1.002.538	L+3’ missing_ Pseudogene
O22A3 ψ	No Ensembl gene report ID	GL831322.1: 1.234.580-1.234.813	L+3’ missing_ Pseudogene
O22A4 ψ	No Ensembl gene report ID	GL831322.1: 1.279.567-1.279.812	L+3’ missing_ Pseudogene
O22A5 ψ	No Ensembl gene report ID	GL831322.1: 1.359,006-1.361.338	L+3’ missing_ Pseudogene
O22A6 ψ	No Ensembl gene report ID	GL831322.1: 1.368.491-1.368.730	L+3’ missing_ Pseudogene
O22B2 ψ	No Ensembl gene report ID	GL831322.1: 1.369.583-1.369.858	L+3’ missing_ Pseudogene
O29B1	ENSONIG00000006116	GL831229.1: 905.654-908.009	<54% id_100% cov.
O29B2 ψ	No Ensembl gene report ID	GL831229.1: 925.993-926.199	L+3’ missing_ Pseudogene
O29B3 ψ	No Ensembl gene report ID	GL831229.1: 933.642-933.887	L+3’ missing_ Pseudogene
O29A1 ψ	No Ensembl gene report ID	GL831229.1: 909.268-909.463	5’+TM CYT missing_ Pseudogene

Table S2 MHC class II gene Ensembl IDs, Ensembl genomic locations, and number of matching cDNAs in GenBank

O29A2 ψ	No Ensembl gene report ID	GL831229.1: 920.028-920.170	5' +TM CYT missing_ Pseudogene
O29A3	ENSONIG00000006121	GL831229.1: 938.918-940.053	2 ESTs > 98% id_100% cov. (e.g. GR667686)
O31B1	ENSONIG00000007425	GL831231.1: 498.924-502.495	<84% id_100% cov.
O31B2	ENSONIG00000007445	GL831231.1: 1.171.738-1.174.377	L missing_<84% id_100% cov.
O31B3	ENSONIG00000007449	GL831231.1: 1.232.619-1.236.792	<96% id_100% cov.
O31A1ψ	No Ensembl gene report ID	GL831231.1: 463.509-463.778	5' missing_ Pseudogene
O31A2ψ	No Ensembl gene report ID	GL831231.1: 505.586-505.849	5'+TM CYT missing_ Pseudogene
O31A3	ENSONIG00000007446	GL831231.1: 1.175.562-1.178.887	<80% id_100% cov.
O31A4	ENSONIG00000007453	GL831231.1: 1.242.793-1.244.465	<78% id_100% cov.
O33A	ENSONIG00000009902	GL831433.1: 1.722-3.127	<95% id_100% cov.
O33B1 ψ	No Ensembl gene report ID	GL831433.1: 5.113-5.340	5' missing_ Pseudogene
O33B2 ψ	No Ensembl gene report ID	GL831433.1: 16.120-16.443	5' missing_ Pseudogene
O39A ψ	ENSONIG00000010744	GL831439.1: 301.122-309.267	3' missing_ Pseudogene
O42A	ENSONIG00000018452	GL831842.1: 89.157-98.104	<99% id_97% cov.
O49B ψ	No Ensembl gene report ID	GL832249.1: 577-1.128	5' missing_ Pseudogene
O50B ψ	No Ensembl gene report ID	GL831650.1: 197.860-198.901	5'+ 3' missing_ Pseudogene
O57B	ENSONIG00000002259	GL831257.1: 1.608.728-1.609.986	1 EST 100% id_100% cov. (GR627548)
O57A	ENSONIG00000002263	GL831257.1: 1.611.410-1.612.511	<73% id_100% cov.
O77B ψ	No Ensembl gene report ID	GL831677: 200.297-200.562	5'+ 3' missing_ Pseudogene
O79B1 ψ	No Ensembl gene report ID	GL831379.1: 603.325-604.139	L+3' missing_Pseudogene
O79B2	ENSONIG00000009129	GL831379.1: 618.993-627.882	1 EST > 97% id_100% cov. (FF281534)
O79A1	ENSONIG00000009128	GL831379.1: 610.569-612.303	<95 % id_97% cov.
O79A2 ψ	No Ensembl gene report ID	GL831379.1: 614.307-615.228	5' missing_Pseudogene
O79A3	ENSONIG00000009132	GL831379.1: 630.011-633.534	2 ESTs > 97% id_100% cov. (e.g. GR671091)
O79A4 ψ	No Ensembl gene report ID	GL831379.1: 677.707:677.997	5' + TM CYT missing_Pseudogene
O80B	ENSONIG00000005905	GL832180.1: 12.190-16.972	1 EST > 97% id_100% cov. (GR702301)
O97B1 ψ	ENSONIG00000004432	GL831197.1: 3.529.581-3.531.898	L+3' missing_Pseudogene
O97B2 ψ	ENSONIG00000004434	GL831197.1: 3.534.451-3.536.032	Missing 24 aa b2 domain_Pseudogene
O97A	ENSONIG00000004441	GL831197.1: 3.645.982-3.653.566	1 EST without ORF (GR679428)
O745B	ENSONIG00000003902	GL831745.1: 25.751-37.458	<95% id_100% cov.
O745A	ENSONIG00000003904	GL831745.1: 44.977-46.516	<95% id_100% cov.
O779B ψ	No Ensembl gene report ID	GL831779: 69.741-70.530	5'+ 3' missing_ Pseudogene

Table S2 MHC class II gene Ensembl IDs, Ensembl genomic locations, and number of matching cDNAs in GenBank

O845A	ENSONIG00000019001	GL831845.1: 36.104-40.196	1 EST 100% id_100% cov. (GR696948)
O845B	ENSONIG00000019004	GL831845.1: 41.863-44.745	<69% id_100% cov.
OA35A ψ	No Ensembl gene report ID	AERX01073735.2: 6-251	L+ 3’ missing_ Pseudogene
OA35B ψ	No Ensembl gene report ID	AERX01073735.2: 3.469-3.735	3’ missing_ Pseudogene
OA78B ψ	ENSONIG00000008338	AERX01073278.2: 2.515-4.762	3’ missing_ Pseudogene
OA78A ψ	ENSONIG00000008336	AERX01073278.2: 144-1.540	5’+3’ missing_ Pseudogene
**Spotted gar (*Lepisosteus oculatus)***			**No Genbank ESTs**
501A1	No ensembl gene ID	JH591501: 64.610-66.685	No data
501B1	No ensembl gene ID	JH591501: 70.640-74.060	No data
501A2	(ENSLACP00000000409)	JH591501: 82.102-84.669	No data
501B2 ψ	(ENSLACP00000000491)	JH591501: 95.883-99.847	5’missing_Pseudogene
501A3 ψ	No ensembl gene ID	JH591501: 120.683-125.781	5’+3’missing_Pseudogene
615A	(ENSLACP00000002396)	JH591615: 5.333-9.156	No data
615B	(ENSLACP00000000558)	JH591615: 11.335-16.190	No data

+ both medaka alpha and beta transcripts are defined under ENSORLG00000009164. # = Number of ESTs available in NCBI EST Databases on 01.02.2012. id= amino acid identity of translated products, cov.= coverage, EST= expressed sequence tag, LG= linkage group, ORF= open reading frame, L= leader sequence; TM= transmembrane, CYT= cytoplasmic domain. Red font= bona fide gene with EST or cDNA support above 97% identity and 100% coverage in conserved alpha 2 or beta 2 domains. 5’ and 3’ missing referring to mature extracellular protein domain (ectodomain). For expressed genes GenBank accession numbers of one matching cDNA report are given between parentheses. Some genes encode very similar constant domains, which in our system leads to assignment of some cDNAs to more than one gene; future species-specific studies should make final assignments of transcript to locus by investigation of genomic DNA and cDNA of the same fish individual.
