# Supplementary material for: Comprehensive analysis of MHC class II genes in teleost fish genomes reveals dispensability of the peptide-loading DM system in a large part of vertebrates
Source: BMC Evol Biol. 2013 Nov 26;13:260. doi: 10.1186/1471-2148-13-260 (PMC4219347; doi:10.1186/1471-2148-13-260)
Supplement: Additional file 6: Table S3 — Conservation pattern of residues which contribute to the hydrogen-bond network between classical MHC class II molecules and the backbone of peptide ligands. [file 1471-2148-13-260-S6.pdf]

Supplementary Table 3 (Table S3). Conservation pattern of residues which contribute to the hydrogen-bond network between classical MHC class II molecules and the backbone of peptide ligands

The hydrogen-bond network by which the backbone of a peptide ligand is bound is structurally well conserved among investigated mammalian classical MHC class II structures [Painter CA, Stern LJ (2012) Conformational variation in structures of classical and non-classical MHCII proteins and functional implications. *Immunol Rev 250:144-157*]. This conserved network involves ten MHC class II residues, namely, five α chain (α9, α53, α62, α69, α76) and five β chain (β57, β61, β71, β81, β82) residues. Nine of these residues, all but α53, use their side-chain to interact with the peptide, and this table summarizes the residues found at those nine positions. Four of the residues have rather well conserved identities (α62N, α69N, β81H, β82N), and among these four β82N was shown to be especially important [Zhou Z, et al. (2009) Cutting edge: HLA-DM functions through a mechanism that does not require specific conserved hydrogen bonds in class II MHC-peptide complexes. *J Immunol* 183(7):4187-4189].

In the table, residue distribution among (supposed) alleles is shown to allow a better estimation of the importance of particular residues. References are the IPD MHC database http://www.ebi.ac.uk/ipd/mhc/ and articles listed below. Nx indicates the number of alleles compared followed by percentages of detected residues, with red and green fonts indicating representation in ≥80% and 50-80% of alleles, respectively. The residues α62N, α69N, β81H and β82N are rather specific for, and well conserved among, the classical class II molecules, and yellow shading highlights cases in which these consensus residues represent the majority among compared alleles. Residue β61 tends to be hydrophobic/aromatic in both classical and non-classical molecules, which is indicated by gray shading.

Tetrapod DM and teleost fish DB molecules tend to have hydrophobic residues at position α69 which is very uncommon in the other class II lineages. Furthermore, at position β81 both lineages lack histidine which is the predominant residue throughout classical molecules. These shared features between tetrapod DM and teleost fish DB are highlighted by blue boxing. For teleost fish DE not many sequences are known yet and therefore we chose not to highlight their lack of β81H.

Sequence references are as follows:

(1) Ayane M,et al. (1986) *J Immunol* 137(3):948-952.
(2) Chu ZT, et al. (1994) *Immunogenetics* 40(1):9-20.
(3) Salomonson J, et al. (2003) *Immunogenetics* 55(9):605-614.
(4) Liu Y, et al. (2002) *Dev Comp Immunol* 26(8):735-750.
(5) Kasahara M, et al. (1993) *Eur J Immunol* 23(9):2160-2165.
(6) Sültmann H, et al. (1993) *Immunogenetis* 38(6):408-420.
(7) Hardee JJ, et al. (1995) *Immunogenetics* 41(4):229-238.
(8) Karlsson L, et al. (1992) *J Exp Med* 176(2):477-483.
(9) Wright H, et al. (1995) Immunogenetics 41(2-3):131-133.
(10) Hermel E, et al (1995) *Immunogenetics* 42(2):136-142.
(11) Chazara O, et al. (2011) *Mol Immunol* 48(9-10):1263-1271.
(12) Ohta Y, et al. (2006) *J Immunol* 176(6):3674-3685.
(13) Human DRB1 sequences analyzed are representatives listed in Table I in the article by Southwood S, et al. (1998) *J Immunol* 160(7):3363-3373.
(14) Begovich AB, et al. (1990) *J Immunol* 144(5):1957-1964.
(15) Estess P, et al. (1986) *Proc Natl Acad Sci USA* 83(11):3594-3598.
(16) Alcaide M, et al. (2008) *Mol Ecol* 17(11):2652-2665.
(17) Kobari F, et al. (1995) *Immunogenetics* 42(5): 376-385.
(18) Bartl S, Weissman IL (1994) *Proc Natl Acad Sci USA* 91(1):262-266.
(19) Wang DQ, He SP (2009) GenBank accessions ACZ26346-8.
(20) Graser R, et al. (1996) *Immunogenetics* 44(1):36-48.
(21) Ono H, et al. (1993) *Mol Biol Evolution* 10(5):1060-1072.
(22) Karlsson L et al. (1991) *Nature* 351(6326):485-488.
(23) Wright H, et al. (1996) *Immunogenetics* 43(1-2):76-79.

.

MHC class II α

Ref.	Classical	alpha9 (various)	alpha62 (N)	alpha69 (N)	alpha76 (various)
	**Mammalian classical**				
IPD	Human DRA1	7x: Q 100%	7x: N 100%	7x: N 100%	7x: R 100%
1	Mouse I-Ea	3x: Q 100%	3x: N 100%	3x: N 100%	3x: R 100%
IPD	Rat Rt1-Da	3x: Q 100%	3x: N 100%	3x: N 100%	3x: R 100%
IPD	Sheep DRA	3x: Q 100%	3x: N 100%	3x: N 100%	3x: R 67%, Y 33%
IPD	Pig DRA	13x: Q 100%	13x: N 100%	13x: N 100%	13x: R 100%
IPD	Human DPA1	30x: Y 100%	36x: N 100%	36x: N 97%, D 3%	36x: R 97%, H 3%
IPD	Human DQA1	49x: G 100%	49x: N 100%	49x: N 100%	49x: R 98%, C 2%
2	Mouse I-Aa	8x: G 100%	8x: N 87%, E 13%	8x: N 87%, T 13%	8x: R 100%
IPD	Rat Rt1-Ba	3x: G 100%	3x: N 33%, E 33%, S 33%	3x: N 100%	3x: R 67%, K 33%
IPD	Cattle DQA	58x: G 100%	60x: N 63%, E 35%, D 2%	60x: N 83%, T 13%, H 2%, D 2%	60x: R 82%, L 7%, E 5%, G 2%, F 2%, Y 2%, C 1%
IPD	Pig DQA	20x: G 100%	20x: N 95%, T 5%	20x: N 85%, T 15%	20x: R 95%, C 5%
IPD	Dog DQA1	16x: G 100%	16x: N 100%	16x: N 100%	16x: R 87%, S 13%
	**Bird classical**				
3	Chicken BLA	2x: Q 100%	2x: N 100%	2x: N 100%	2x: N 100%
	**Amphibian classical**				
4	Xenopus DAA	4x: G 100%	4x: N 75%, Q 25%	4x: N 100%	4x: S 100%
4	Xenopus DBA	4x: G 100%	4x: D 50%, E 25%, N 25%	4x: N 100%	4x: R 75%, V 25%
	**Elasmobranch classical**				
5	Nurse shark DAA	6x: Q 100%	6x: N 100%	6x: N 100%	6x: L 100%
	**Teleost fish classical**				
IPD	Salmon DAA	18x: V 39%, A 39%, Y 11%, H 11%	21x: N 100%	21x: N 100%	21x: A 100%
IPD	Trout DAA	3x: Y 67%, A 33%	3x: N 67%, D 33%	3x: N 67%, D 33%	3x: A 100%
6	Zebrafish DAA	3x: D 67%, V 33%	3x: N 33%, D 33%, Q 33%	3x: N 67%, D 33%	3x: A 100%
7	Striped bass IIA	7x: H 42%, R 29%, A 29%	7x: N 86%, Q 14%	7x: N 100%	7x: A 100%
	Highly expressed DA group genes as indicated in Fig. 2	6x: A 50%, V 17% G 17%, Q 17%	6x: N 50%, Q 34%, E 17%	6x: N 100%	6x: A 83%, G 17%
	**Non-classical**				
	**Mammalian DO**				
IPD	Human DOA	11x: G 100%	11x: G 100%	11x: H 100%	11x: R 100%
8	Mouse DOA	6x: G 100%	6x: S 100%	6x: H 100%	6x: R 100%
IPD	Rat RT1-DOa	2x: G 100%	2x: S 100%	2x: H 100%	2x: R 100%
9	Sheep DOA	1x: G 100%	1x: S 100%	1x: H 100%	1x: R 100%
	**Tetrapod DM**				
IPD	Human DMA	5x: H 100%	5x: D 100%	5x: M 100%	5x: K 100%
IPD	Rat DMA	2x: H 100%	2x: D 100%	2x: L 100%	2x: R 100%
10	Mouse DMA	1x: H 100%	1x: D 100%	1x: L 100%	1x: K 100%
IPD	Pig DMA	4x: Y 100%	4x: D 100%	4x: M 100%	4x: L 100%
11	Chicken DMA	1x: E 100%	1x: D 100%	1x: L 100%	1x: I 100%
12	Xenopus DMA	1x: Q 100%	1x: D 100%	1x: A 100%	1x: A 100%
	Teleost fish nonclassical **Teleost DB group** all intact in Text S4*A*	15x: Y 40%, L 27%, F 20%, H 7%, V 7%	15x: S 47%, N 14%, L 14%, A 14%, Y 7%, I 7%, G 7% (N=13%)	15x: V 33%, Y 13%, I 13%, F 13%, D 7%, C 7%, L 7%, A7% (N=0%)	15x: A 27%, L 27%, E 13%, V 7%, S 7%, P 7%, Q 7%, F 7%
	**Teleost DE group** all in Text S4*A*	1x: F 100%	2x: D 50%, S 50% (N=0%)	2x: N 100% (N=100%)	2x: G 50%, R 50%

Table S3 Conservation of peptide backbone binding residues

MHC class II β

Classical	beta57 (various)	beta61 (hydrophobic)	beta71 (various)	beta81 (H)	beta82 (N)
Ref.:					
**Mammalian classical**					
13 Human DRB1	10x: D 70%, V 30%	10x: W 100%	10x: R 60%, K 20%, E 10%, A 10%	10x: H 100%	10x: N 100%
14 Mouse I-Eb	8x: D 87%, E 13%	8x: W 100%	8x: K 50%, A 25%, R 13%, T 13%	8x: H 87%, Y 13%	8x: N 100%
IPD Rat RT1-Db1	4x: S 50%, D 25%, I 25%	4x: Y 50%, L 25%, R 25%	4x: E 25%, A 25%, L 25%, R 25%	4x: H 100%	4x: N 75%, D25%
IPD Cattle DRB3	130x: D 65%, V 15%, S 10%, A 10%	130x: W 91%, L 8%, C 2%	130x: K 41%, R 29%, E 22%, A 7%, G 1%	130x: H 93%, Y 7%	130x: N 100%
IPD Sheep DRB1	77x: D 53%, S 36%, E 6%, A 3%, G1%	77x: W 100%	77x: R 44%, K 29%, A 17%, T 10%	77x: H 100%	77x: N 100%
IPD Pig DRB1	80x: D 74%, V 9%, S 8%, E 6%, F 4%	80x: W 86%, Y 11%, R 3%	80x: R 55%, K 21%, S 15%, M 8%	60x: H 98%, Y 2%	57x: N 98%, D 2%
IPD Dog DRB1	52x: D 81%, V 12%, I 4%, S 4%	52x: W 100%	52x: R 37%, K 27%, E 25%, A 10%, G 2%	52x: H 100%	52x: N 100%
IPD Cat DRB	20x: D 65%, I 20%, T 15%	20x: W 70%, L 20%, M 10%	20x: K 30%, T 20%, R 15%, E 15%, S 15%, A 5%	20x: H 100%	20x: N 100%
IPD Human DPB1	155x: D 52%, A 36%, E12%	155x: W 100%	155x: K 64%, E 34%, R 4%	155x: H 100%	155x: N 100%
IPD Human DQB1	177x: D 62%, V 17%, A 16%, S 5%	177x: W 100%	176x: T 69%, A 16%, D 8%, K 7%	173x: H 99%, P 1%	173x: N 99%, S 1%
15 Mouse I-Ab	6x: D 100%	6x: W 50%, Y 50%	6x: T 100%	6x: H 100%	6x: N 100%
IPD Rat RT1-Bb	3x: S 100%	3x: F 67%, Y 33%	3x: T 100%	3x: H 100%	3x: N 100%
IPD Cattle DQB	82x: D 60%, A 23%, Q 6%, S 5%, T 2%, H 1%, E 1%, P 1%	82x: W 73%, F 27%	82x: T 74%, V 13%, R 10%, A 1%	82x: H 70%, N 29%, Y 1%	82x: N 100%
IPD Pig DQB1	44x: D 43%, E 25%, T 16%, A 14%, G 2%	44x: W 86%, L 14%	44x: T 59%, K 36%, M 9%, A 5%	44x: H 100%	44x: N 100%
IPD Dog DQB1	36x: D 53%, S 19%, V 17%, W 11%	36x: W 100%	36x: V 50%, R 19%, K 19%, E 11%	36x: H 100%	36x: N 100%
**Bird classical**					
16 Lesser kestrel DAB*1	98x: T 79%, D 11%, A 10%	98x: W 56%, F 36%, L 7%	98x : R 39%, A 34%, K 27%, T 1%	98x: H 100%	98x: N 100%
**Amphibian classical**					
17 Xenopus IIB	6x: Q 50%, D 33%, S 17%	6x: W 100%	6x: K50%, T 17%, H 17%, Y 17%	6x: H 67%, Y 33%	6x: N 100%
**Elasmobranch classical**					
18 Nurse shark DAB	2x: N 100%	2x: W 100%	2x: Q 100%	2x: H 100%	2x: N 100%
**Primitive ray-finned fish (classical?)**					
19 Paddlefish (DE-lineage)	3x: N 100%	3x: W 100%	3x: L 100%	3x: H 67%, R 33%	3x: N 100%
**Teleost fish classical**					
IPD Salmon DAB	42x: N 100%	42x: W 98%, L 2%	42x: E 93%, A 7%	42x: H 71%, L 19%, F 7%, Q 2%	42x: N 100%
IPD Trout DAB	19x: N 100%	19x: W 79%, R 5%, L 16%	19x: E 89%, M 11%	19x: H 68%, P 32%	19x: N 95%, S 5%
20 Zebrafish DAB	11x: F 82%, C 9%, S 9%	11x: F 91%, L 9%	11x: L 73%, R 9%, E 9%, W 9%	11x: H 82%, N 18%	11x: N 100%
21 Tilapia zebra DAB	14x: N 57%, Q 21%, Y 14%, T 7%	14x: W 79%, F 21%	14x: W 36%, M 14%, D 14%, V 14%, E 7%, R 7%, L 7%	14x: H 79%, P 14%, N 7%	14x: N 100%
Highly expressed DA group genes as indicated in Fig. 2	4x: N 50%, F 25%, T 25%	4x: W 50%, F 25%, A 25%	4x: E 50%, R25%, L 25%,	4x: H 100%	4x: N 100%
Non-classical					
**Mammalian DO**					
IPD Human DOB	13x: D 100%	13x: W 100%	13x: S 100%	13x: H 100%	13x: N 100%
22 Mouse DOB	3x: D 100%	3x: W 100%	3x: S 100%	3x: Q 100%	3x: K 100%
IPD Rat DOB	1x: D 100%	1x: W 100%	1x: S 100%	1x: Q 100%	1x: E 100%
23 Sheep DOB	1x: D 100%	1x: W 100%	1x: S 100%	1x: R 100%	1x: N 100%
**Tetrapod DM**					
IPD Human DMB	11x: L 100%	11x: L 100%	11x: R 100%	11x: T 100%	11x: H 100%
10 Mouse DMB (1+2)	7x: I 86%, N 14%	7x: I 100%	7x: R 100%	7x: T 86%, S 14%	7x: H 100%
IPD Rat DMB	2x: I 100%	2x: L 100%	2x: R 100%	2x: T 100%	2x: H 100%
11 Chicken DMB (1+2)	2x: L 100%	2x: L 100%	2x: R 100%	2x: E 50%, K 50%	2x: L 100%
12 Xenopus DMB	1x: I 100%	1x: L 100%	1x: R 100%	1x: A 100%	1x: Q 100%
Teleost fish nonclassical					
**Teleost DB group** all intact in Text S4*B*	15x: F 33%, W 13%, S 13%, I 13%, T 13%, L 7%, V 7%	15x: L 27%, F 27%, Y 20%, W 13%, A 7%, V 7%	15: R 40%, Q 20%, H 13%, A 13%, L 13%	15x: T 20%, R 13%, K 13%, L 13%, P 7%, D 7%, S 7%, V 7%, E 7% (H=0%)	15x: N 33%, I 13%, L 13%, F 13%, S 13%, Y 7%, H 7% (N=33%)
**Teleost DE group** all in Text S4*B*	2x: N 100%	2x: Y 100%	2x: R 100%	2x: Y 50%, A 50% (H=0%)	2x: N 50%, S 50% (N=50%)

Table S3 Conservation of peptide backbone binding residues
