# Supplementary material for: Comprehensive analysis of MHC class II genes in teleost fish genomes reveals dispensability of the peptide-loading DM system in a large part of vertebrates
Source: BMC Evol Biol. 2013 Nov 26;13:260. doi: 10.1186/1471-2148-13-260 (PMC4219347; doi:10.1186/1471-2148-13-260)
Supplement: Additional file 7: Text S1 — Deduced MHC class II amino acid sequences of teleost fish and gar from Ensembl database and matching GenBank cDNA reports. [file 1471-2148-13-260-S7.pdf]

Supplementary Text 1 (Text S1). Deduced MHC class II amino acid sequences of teleost fish and gar from Ensembl database[a] and matching GenBank cDNA reports[b]

Table of Contents	Page
Zebrafish (*Danio rerio*)	
Ensembl sequences	2
Translated Genbank cDNA report sequences	4
Stickleback (*Gasterosteus aculeatus*)	
Ensembl sequences	5
Translated Genbank cDNA report sequences	7
Medaka (*Oryzias latipes*)	
Ensembl sequences	7
Translated Genbank cDNA report sequences	8
Fugu (*Takifugu rubripes*)	
Ensembl sequences	9
Translated Genbank cDNA report sequences	10
Tetraodon (*Tetraodon nigroviridis*)	
Ensembl sequences	10
Translated Genbank cDNA report sequences	11
Tilapia (*Oreochromis niloticus*)	
Ensembl sequences	11
Translated Genbank cDNA report sequences	16
Spotted gar (*Lepisosteus oculatus*) [c]	
PreEnsembl sequences	17

[a]Deduced amino acid sequences of the MHC class II genes in the Ensembl database which were analyzed in the present study (as listed in Table S2, Fig. S2 and main text Fig. 2)
[b]Deduced amino acid sequences of GenBank cDNA reports, used for comparison and estimation whether the class II loci reported in the Ensembl database are expressed (see Table S2); not all matching cDNA reports are shown, but at least one match with above 97% constant domain sequence identity for each expressed gene and in a few cases additional matches suggestive of polymorphism
[c]For spotted gar no MHC class II cDNA reports were found in GenBank.

Zebrafish (Danio rerio) MHC class II sequences

Zebrafish Ensembl sequences:

>D4B_DFB ENSDARG00000054921_ENSDARP00000071675_4:61874514-61880599
MSPANLLLMLSVFTGAADGYYFSTWSQCIYSYPDLSDMVFILSFNFNKWMFLQFNSTVGI
FVGYTEQGVKFAENFNKNEAYLQQLRAEVDIFCRHNAEIYESAVFDKAVMPKVNLSLVQK
GDSRHPYLLRCSAYDFYPQQIKMSWLRDGRVVSDVTSSEQMPNGDWYYQSHSELVFSPKS
GETISCMVEHSSLTGPVVIDWKPPVHEFEWDFQLLSAAGDASAVLGIIIATAGYIYYKTK
TEKNLVAIY
>D4A ENSDARG00000020286_ENSDARP00000018440_4:61887203-61893205
FVPASPAKMELLYLLLLAFTVVLSTHARVKHNLFMFNGCSDTEKEALYGSEGEEMWHSDF
KLKQGVATLPEFADPLSFPGAYEESVADQEVCKQNLATSIKAYNSPPEEMEPPETSIYPR
NEVLLGVDNVLICHVTGFFPPPVDVSWSRNNVKVTEGVDVSQYRPRSDQTFHMFSSLKIR
PAAGDIYGCTVNHAALQEQPQTKLWVVPQGGVLSPCLTPVLLSGLGFTVGLLGLATGIYF
FIKGSTADVSDTAVSKKKLMQFYQ
>D8.35B1 ENSDARG00000093706_ENSDARP00000104158_8:35956336-36146143
MCSAYDFYPPHINVFWLRNGEVMTSEVTSTMEMADGDWYYQIHSELEYSPKPGERISCVI
EHASSNKPMIYDWDPSLPVFERNKISIGVSALVLGIITAAAGIIYYKKKTTGRTSVVPSR
LITLPLMFGR
>D8.35B2 ENSDARG00000053738_ENSDARP00000070311_8:35956359-35959768
MSQKKLYSSLILILALFFVGESANVYYMYRVSRCIFSSSNISAMVYFDRTYFNKNLFIQF
NSNLGRFEGFNEYGLKLAEFWNNGTFVDQEKDVVEFFCKYNSQIYENSILDKAVKPKVKL
SSVTRAGGRQSTVLMCSAYDFYPPHINVFWLRNGEVMTSEVTSTMEMADGDWYYQIHSEL
EYSPKPGERISCVIEHASSNKPMIYDWDPSLPVFERNKISIGVSALVLGIITAAAGIIYY
KKKTTGRTSVVPSRLITLPLMFGR
>D8.35A1 ENSDARG00000053739_ENSDARP00000070315_8:36131728-36140054
TLTTLLSTHAEIVHDYFSIHGCSYTDEEGMQAVDGEDMHGIDNEEMWHADYNLKTGVLSL
PDFGDPITFPRFYETSVIEMTGCKANIVSLTKIFNSPPPEMDPPQTSIYPKDDVELGVQN
TLVCHATGFYPPSIRILWTMNNVNVTEGISLSQYRPRVDGTFNIFSTFRFTPAEGDIYSC
RVIHEALLGQPQAKIYDVDVAIPSVGPSVFCGVALSLGLLGIVTGTFFFIKAFNYNVDME
VN
>D8.35A2 ENSDARG00000055441_ENSDARP00000072260_8:36283500-36286998
MELYITTLVLTIILSAGAKVVHEDFAIRGCSDTEKEDTYGMDEEELWHADFNQKKGVETL
PDFGDPMTFPGFYEGSEGEMAVCKSNLALRIKGFKSPPPEMDAPQTSIYPKDDVELGVQN
TLVCHVTGFYPPSVSVSWTKNNINVSEDITLSQYRPRIDGTFNIFSTLKFTPAEGDIYSC
MVKHRAIKDQPQTKTWDVDAVLPSVGPAVFCGIGLTLGLLGVAVGTFFLVKGNNCN
>D8.35B3 DEB_ENSDARG00000055447_ENSDARP00000072268_8:36288188-36289984
MLPLKYFTCLMLTVSLFTGAESANVYYTYRWCRCIYSSPDLSDMVYIDSKYFNKYLFAQF
NSTLGRYVGFSEYGIRNAEYWNNGTFVEQERNEVERFCKHNAQIRDSAVISKAVKPKVKL
SSVTRAGGRHPAVLMCSAYDFYPPHIKISWLRDGQVVTSDVTSTEEMADGDWYYQIHSEL
EYSPKSGEKISCAVEHASSTKPIIYDWDPSFSESEKNKLVIGASGLVLGIVLAAAGLIYY
KKKSTGRILVPH
>D8.37A1 ENSDARG00000074816_ENSDARP00000100115_8:37465644-37474349
MELHIIILSLAIVLSTGTEFEHEVNQIQVCSNTEQEFFIGYDGEEIWHADIAHRRGVKTT
PSFAQYAVIPDIYERALAFLDFCKYDLHAYIKYFNNPPPEMDAPQTSIYSKGNVQLGIQN
TLVCHVTGFYPPFVNISWTKNNVIVTEGMSLSQYHPRVDGTFNIFSTLKYTPAEGDIYSC
TVNHRTLQGQPQTRIWDVDVVLPNFGPSVFCGVGLTIGLLSVAIETFFLFKRKKSN
>D8.37B1 ENSDARG00000074510_ENSDARP00000116857_8:37476789-37482114
XDFWARFSRYSERVKMSLLRLFYGHLILTLSVLIGAETANVYYRYTWIQCISSSKNLSDM
VFIESLYFNKYLFTQFNSNIGKYVGFSEYGIRNAEFRNNDTNLLLKKKAAVNKVCKNNAI
NHDKFIRNKAVKPKVKLTLVKPAGGRHPAVLMCSAYEFYPPHIRVYWLRNGEVITSDVTS
TMEMADGDWYYQIHSELEYSPKSGERISCGIEHASSNKPIITEWDSSRPASERNKIAIGA
SG
>D8.37A2 ENSDARG00000070206_ENSDARP00000093621_8:37488525-37498906

MELYVIVLTFIAFLSSNAEFVREELEIYVCSDSESEAMVGADGEELWHADFKQKTGIITL
PDFADRIGFPGFYEISVDSIAGCRRNLANNIRGFKSPPPAMDAPHSSVYPKDDVELGVQN
SLICHATGFYPPSITISWKKNNNNVTESINLSQYRPRADGSFNIFSTLKFTPAEGDIYSC
TVNHEALQGQAQTKTWDVDVALPSAFPAVVCGVGVVFGLLGIAAGTFLLVKGNCN
>D8.37B2_DDB ENSDARG00000076307_ENSDARP00000097822_8:37510422-37511403
MLICSAYEFYPRHIKVSWLKGGKSVTSEVTSTMEMADGDWYYQIHSELEYTPGPGEKISC
LVEHASSSEPMIYDWDPSLPESERNKIAVGASGLVMGFIIAAVGILYYRKKSTGMIPIPH
>D8.37A3_ENSDARG00000031745_ENSDARP00000048447_Chr8:37539907-37543263
MDLFGFLLIFTVIVSNGAQAEHKDFGFVGCSDMEKEFLFGFDGEELFHLDFIRKEGVATA
PDFADPLSFPGFYEAGVAQMEVCKQNLATDIRAYNSPQEQLDPPVTSIYSKDEVVLDKKN
TLICHVTGFFPPPVNVSWTKNNDIVLEEISFSQYRENSDGTFNMFSALKFTPAEGDIYSC
TVKHRSIQGQTNTKTWEVDVELPSVGPAVFCGVGLVLGLLGVAAGTFFLIKGNNCN
>D8.37B3_DAB ENSDARG00000079105_ENSDARP00000100252_8:37546100-37548453
MYLLKPFLVILMLSTFTGTADGYYDYIKQQCFYSTSDYSDMVYLASYSFNKVVDTQFNSS
VGKFVGYTEQGLIFAENFNKDQAYLHQLKAQVDTFCRHNAQIWDSAVRDKAVLPEVTIKS
VRQAEGRHPAVLLCSAYEFYPKKIKMSWLRDGKEVTSDVTSTMEMADGDWYYQIHSHLEY
TPKSGEKIQCLVEHASLTQPLTKDWNPHISESDRNKFAIGASGLVLGIIIAIAGLIYYKK
KSTGRILVPN
>D8.37A4 ENSDARG00000001832_ENSDARP00000043524_8:37574076-37576393
MDLFGFLLIFTVIVSNGAQAEHRDFQFTGCSDTEKDDLNGFDGEVLYYTDFIRKEGVVIA
PDFADPLSFPGGYETGVAQMEVCKQNLATDIKAYNSPQEQLDPPVTSIYSEDEVVLDEKN
TLICYVTGFFPPPVNVSWTKNNDIVTEDISFSQYRDNSDGTFNMFSALKFTPAEGDIYSC
TVKHRSIQGQPNTKTWEVDVELPSVGPAVFCGVGLVLGLLGVAAGTFFLIKGNNCN
>D8.45B1 ENSDARG00000041705_ENSDARP00000088910_8:45758566-45761489
MNAKINYSLAAVFLSALFETVHAYYTYAQIQCHVSDSLQKIEFIFSVTYNMIELVRYNST
EDTFFGYTAIGQKFAEEYNKDKVLLAQHDFVLNQCRELGDVILPNAVWLAVKPEVIIRSV
TEAKGNRKAVLVCSAYDFYPKGIKLTWMRDDKKVTAELTSSEVMADGHWHYQIHSYLEYF
PQTGEKISCVVDHASSLKPMIYYWDPSLPESERSKIILGAVGLLMGIFTAAAGVIYYKRK
QTGFYRLPVCLLPMETMNDTELQ
>D8.45A1 ENSDARG00000079593_ENSDARP00000061131_8:45768098-45769605
MFKTFSGITFFIFCLIHTEGQSYPEFGFIETCRGSSYDKIVFILDTEEAAYKDIEKEKLV
FTLPDMANPQYSLNDLRLNDVGIPAFCTEIYHKILSLNVSEDTEPLEPPWTTVYSRNDVK
LNVKNTLICHVTGFFPPPVRVLWTKNNVNVTDGSTISRYYPNKDGTMNVFSRLSFIPEEG
DVYGCSVEHKALQQPQTRTWDVEVQQPSIGPSVFCGVSLALGLFGFATGVLFIAKGKLCK
>D8.45B2 DCB_ENSDARG00000088872_ENSDARP00000110668_8:45773380-45774524
MQFSRVACLAMILSALLEKVCGNYGYLQSQCRVLSSTKKVELIFSFIFNKIEYIRYNSTD
QKIVGYTEFGEKFVENYKNNTFVLVLAEFGIDNCKKIAKALISDGMLNHVTVKPEVIIRS
VTEAKGNQKAVLVCSAYDFYPKAIKLTWMRNDKKVTADVMSIEEMADGDWYYQIHSHLEY
FPQPGEKISCVVDHASFHKPMIYYWDPSLPETERSKIILGAVGLLMGIFTAAAGVIYYKR
KQTG
>D8.45A2 ENSDARG00000086294_ENSDARP00000112102_8:45778095-45779781
MLVYSSDHSIVELTQANMLELLCRIMLFIAYWTCSEGQMDLEYGLMESCTGSPDDEIVFT
FNTELAAYLVVVDGTLVLTVPNFIKDYPGFYELPLLDASPATVGLTALCKVVYEKTLNLN
VSEPLEPPWVLLYPRNDVKLNLKNTLICHVTGFFPPPVRVLWTKNNVNVTDGSTISRYYP
DNDRNFNVFSQLSFIPEEGDVYSCSVEHKALQQPQTRTWDVEVKQPSIGPSVFCGVGLAF
GLLSFAAGIFFNAKGKKWM
>D8.46A ENSDARG00000075932_ENSDARP00000010619_8:47078720-47080882
MEALLRITLICVCVQAKDYHKYGLIGACGDADQEDFIVQFDDEQLAHVDFKEQKDVITLP
EFAGQAVLPPIYVDAKRAEFNCKAYLAVLREVYASSPEVLEPPWSSIYPKSDPQLNLKNT
LICHVTGFFPPPVRVLWTKNNVNVTDGSTISRYYPNKDGTMNVFSRLSFIPEEGDIYSCS
VEHKALQQPQTRTWDVEVKQPSIGPSVFCGVGLALGLLGFATGVFFTAKGNNCN
>D18B ENSDARG00000056330_ENSDARP00000090055_Chr18:29872428-29875206
MFNVLVIRLALLLPFLLETAHGHYGFVQFTCHMLGSLQNVEVTYSIYFDTTELLRFNSTE
NKAVAYTEYAMKWANDLNKQPKWLHEQVEKNIADCKLFGETYFPLVAKTVKPEVFVRSLR
EASGKRPALLSCSAYNFYPKHIKLTWMRDDKVVTADVMSTKVMADGDWYYQIHSHLEYFP
QPGEKISCVVEHASSHKPMIYYWDSFLTESNKNKIITGAAGIVLGAIMAAGGLIYYKRKH
TVKSHVHVLIY

>D18A ENSDARG00000093885_ No ENSDARP_18:29867551-29868594
DYHKYGLIGASGDADQEDFIVQFDDEQLAHVDFKEQKDVITLPEFADLAVFQPLYVDAKR
AFNGKAFLAVLREVYASSPEVLEPPLSSIYPKSDPQLNVKNTLICQVTGFFPPPVRVLWT
KNNVNVTDGSTISRYYPNKDGTMNVFS*LSFIPEEGDIHSCLVEHKALQQPQTRTW

Zebrafish translated Genbank cDNA report sequences:

>EH441901 FDR103-P00029-DEPE-F_E20 FDR103 Danio rerio cDNA clone FDR103-P00029-BR_E20 5, mRNA sequence D18B match
MLGSLQNVEVTYSIYFDTTELLRFNSTENKAVAYTEYAMKWANDLNKQPKWLHEQVEKNI
ADCKLFGETYFPLVAKTVKPEVFVRSLREASGKRPALLSCSAYNFYPKHIKLTWMRDDKV
VTADVMSTKVMADGDWYYQIHSHLEYFPQPGEKISCVVEHASSHKPMIYYWDSFLTESNK
NKIITGAAGIVLGAIMAAGGLIYYKRKHTVKSHVHVLIY
>EB769231.1_1 2026670 AG03 Danio rerio cDNA clone 770142, mRNA sequence D4B match
FDKAVMPKVNLSLVQKGDSRHPYLLRCSAYDFYPQQIKMSWLRDGRVVSDVTSSEQMPNG
DWYYQSHSELVFSPKSGETISCMVEHSSLTGPVVIDWKPPVHEFEWDFQLLSAAGDAXAX
LXXXIV
>DT061156.1_3 AGENCOURT_56110132 NIH_ZGC_10 Danio rerio cDNA clone IMAGE:8000694 5', mRNA sequence D8.37B3 match
FLVILMLSTFTGTADGYYDYIKQQCFYSTSDYSDMVYLASYSFNKVVDTQFNSSVGKFVG
YTEQGLIFAENFNKDQAYLHQLKAQVDTFCRHNAQIWDSAVRDKAVLPEVTIKSVRQAEG
RHPAVLLCSAYEFYPKKIKMSWLRDGKEVTSDVTSTMEMADGDWYYQIHSHLEYTPKSGE
KIQCLVEHASLTQPLTKDWNPHISESDRNKFAIGASGLVLGIIIAIAGLIYYKKKSTGRI
LVQTDSSESNVT
>DN896667.1_2 nap12d02.y1 Zebrafish anterior segment (minus lens). Unnormalized (nap) Danio rerio cDNA clone nap12d02 5', mRNA sequence D8.35B3 match
MLTVSSFTGAESANVYYYTYRWCRCIYSSPDLSDMVYIDSYYFNKYLFTQFNSTLGKCVGF
SEYGIRNAEYWNNGTFVEQERNEVERFCKHNSQIRDSAVISKAVKPKVKLSSVTRAGGRH
PAVLMCSAYDFYPPHIKISWLRDGQVVTSDVTSTEEMADGDWYYQIHSELEYSPKSGEKI
SCAVEHASSTKPIIYDWDPSFSESEKNKLVIGASGLVLGIVLAAAGLIYX
>CK030151 AGENCOURT_16620967 NIH_ZGC_7 Danio rerio cDNA clone IMAGE:7052626 5, mRNA sequence D8.35B2 match
MILALFFVGESANVYYMYRVSRCIFSSSNISAMVYFDRTYFNKNLFIQFNSNLGRFEGFN
EYGLKLAEFWNNGTFVDQEKDVVEFFCKYNSQIYENSILDKAVKPKVKLSSVTRAGGRQS
AVLMCSAYDFYPPHINVYWLRNGEVMTSEVTSTMEMADGDWYYQIHSELEYSPKPGERIS
CVIEHASSNKPMIYDWDPSLPVFERNKISIGVSALVLGIITAAAGIIYYKKKLQGGLSTS
SR
>CO798204 AGENCOURT_30342545 NIH_ZGC_14 Danio rerio cDNA clone IMAGE:7400349 5, mRNA sequence D8.45B1 match
MSALFETVHAYYTYAQIQCHVSDSLQKIEFIFSVTYNMIELVRYNSTEDTFFGYTAIGQK
FAEEYNKDEVLLAQHDFVLNQCRELGDVILPNAVWLAVKPEVIIRSVTEAKGNRKAVLVC
SAYDFYPKGIKLTWMRDDKKVTAELTSSEVMADGHWHYQIHSYLEYFPQTGEKISCVVDH
ASSLKPMIYYWDPSLPESERSKIILGAVGLLMGDFTAAAGVIYYKRKQTGFYRLPVCLLP
METMNDTEM
>DT880928 AGENCOURT_58686230 NIH_ZGC_22 Danio rerio cDNA clone IMAGE:7924885 5, mRNA sequence D8.35A1 match
MQAVDGEDMHGIDNEEMWHADYNLKTGVLSLPDFGDPITFPRFYETSVIEMTGCKANIVS
LTKIFNSPPPEMDPPQTSIYPKDDVELGVQNTLVCHATGFYPPSIRILWTKNNVNVTEGI
SLSQYRPKVDGTFNIFSTFRFTPAEGDIYSCRVIHEALLGQPQAKIYDVVVAIPSVGPSV
FCGVALSLGLLGIATGTFFFIKHSTTMEIWK
>EH498789.1_1 FDR107-P00042-DEPE-F_J13 FDR107 Danio rerio cDNA clone FDR107-P00042-BR_J13 5', mRNA sequence D8.35A2 match
DSISRPRIRRFNKMELYITTLVLTIILSAGAKVVHEDFAIRGCSDTEKEDTYGMDEEELW
HADFNQKKGVETLPDFGDPMTFPGFYEGSEGEMAVCKSNLALRIKGFKSPPPEMDAPQTS

IYPKDDVELGVQNTLVCHVTGFYPPSVSVSWTKNNINVSEDITLSQYRPRIDGTFNIFST
LKFTPAEGDIYSCMVKHRAIKDQPQTKTWDVDAVLPSVGPAVFCGIGLTL
>CO353326.1_2 DR_ATE_FL13_G10 adult testis full-length (TLL) Danio rerio cDNA, mRNA sequence D8.37A4 match
MGLKLSTGXFQFTGCSDTEKDDLNGFDGEVLYYTDFIRKEGVVIAPDFADPLSFPGGYET
GVAQMEVCKQNLATDIKAYNSPQEQLDPPVTSIYSEDEVVLDEKNTLICYVTGFFPPPVN
VSWTKNNDIVTEDISFSQYRDNSDGTFNMFSALKFTPAEGDIYSCTVKHRSIQGQPNTKT
WEVDVELPSVGPXVFCGSGV
>EG576857 AGENCOURT_88173786 NIH_ZGC_35 Danio rerio cDNA clone IMAGE:8756121 5, mRNA sequence D8.37A2 match
MELYVIVLTFIAFLSSNAEFVREEFEIYVCSDTESEAMVGADGEELWHADFKQKTGIITL
PDFADRIGFPGFYEISVDSIAGCKRNLANNIRGFKSPPPAMDAPHSSVYPNDDVELGVQN
SLICHATGFYPPSITISWKKNNNNVTESINLSQYRPRADGSFNIFSTLKFTPAEGDIYSC
TVNHEALHGQAQTKTWDVDVALPSAFPAVVCGVGLVFGLLGIAAGTFLLVKGNCN
>EH450259 FDR103-P00052-DEPE-F_F04 FDR103 Danio rerio cDNA clone FDR103-P00052-BR_F04 5, mRNA sequence D8.46A match
MEALLRITLICVCVQAKDYHKYGLIGACGDADQEDFIVQFDDEQLAHVDFKEQKDVITLP
EFAGQAVLPPIYVDAKRAEFNCKAYLAVLREVYASSPEVLEPPWSSIYPKSDPQLNLKNT
LICHVTGFFPPPVRVLWTKNNVNVTDGSTISRYYPNKDGTMNVFSRLSFIPEEGDIYSCS
VEHKALQQPQTRTWDVEVKQPSIGPSVFCGVGLALGLLGFATGVFFTAKGNNCN
>EB979084.1_2 15505312 ZF40 Danio rerio cDNA clone 4224518, mRNA sequence D8.37A3 match
SFPGFYEAGVAQMEVCKQNLATDIRAYNSPQEQLDPPVTSIYSKDEVVLDKKNTLICHVT
GFFPPPVNVSWTKNNDIVLEEISFSQYRENSDGTFNMFSALKFTPAEGDIYSCTVKHRSI
QGQTNTKTWEVDV
>CF996478 AGENCOURT_16384897 NIH_ZGC_7 Danio rerio cDNA clone IMAGE:7037730 5, mRNA sequence D4A match
MDLTVRSEFPGSAKMELLYLLLLAFTVVLSTHARVKHNLFMFNGCSDTEKEALYGSEGEE
MWHSDFKLKHGVATLPEFADPLSFPGAYEESVAELEVCKQNLATSIKAYNSPPEEMEPPE
TSIYPRNEVLLGVDNVLICHVTGFFPPPVDVSWSRNNVKVTEGVDVSQYRPRSDQTFHMF
SSLKIRPAAGDIYGCTVNHAALQEQPQTKLWVVPQGDVLSPCLTPVLLSGLGFTVXLLGL
ATGIYFFVXGSTADVSDTAVNKRKLMQFYHXET
>CN023898 AGENCOURT_20187689 NIH_ZGC_14 Danio rerio cDNA clone IMAGE:7229870 5', mRNA sequence D8.37B1 match
MSLLRLFYGNLILTLSVLIGAETANVYYRYTWIQCISSSKNLSDMVFIESLYFNKYLFTQ
FNSNIGKYVGFSEYGIRNAEFRNNDTNLLLKIKAAVNKVCKINAINHDKFIRNKAVKPKV
KLTLVKPAGGRHPAVLMCSAYEFYPPHIRVYWLRNGEVITSDVTSTMEMADGDWYYQIHS
ELEYSPKSGERISCGIEHASSNKPIITEWGKRNMLTVILALFCGTFDILLYPTDSSRPAS
ERNKIALGHLGVVLGSSSQLICFITTRKLAKPVL

Stickleback (*Gasterosteus aculeatus*) MHC class II sequences

Stickleback Ensembl sequences:

>G129B scf129_contig12675:25778-25611
EPYVRLHSETPPGGGPLSMLVCSVYDFYPKKIIVRWTRDGRPETTGVTSTDELAGGRSGE
KMSCVVEHISLSKPLVTDW
>G129A Scf.129_contig12675:26484-26221
SHMIYRKHGVELGEKNCHVTGFFPAPVTFSWTKSQENVTEGTCKNVPFLNKDRTFNQFST
LEFTPKLGDIYSSRVEHLALDHPLVKFY
>G131A1 ENSGACG00000000330_ENSGACP00000000421_scf_131:52153-54217
MKTKTMMKMMVVLVLSGVFCVSADGPHKDIAIVGCSDSDGEEMYGLDGEEVWYADFKHGK
GVRPQPSFVDPIDYREGTYETAVGNQQICRNNLKIDLKAFKDFPLEKDPPSSHMIYPKDG
VELGEKNSLICHVTGFYPAPVTFSWTKNQDNVTEGSSRNVPYLNNDGTFNQFSTLEFTPK

LGDIYSCMVEHLALDHPLVKFYDVQVSQPSVGPAVFCGVGLTVGLLGVAAGTFFLIKGNE
CS
>G131B1 ENSGACG00000000336_ENSGACP00000000425_scf_131:56853-59742
MAPSFISVSLLFIGLHAADGFMEFVATECVFNSTELKDIEYIQSYYYNKLEYTRFSSSVG
KFVGFTERGVKNAAAWNNNPSYLSRAKAQKEVYCLNHVPVYYNNMLTKSAEPYVRLHSET
PPGGGPLSMLVCSVYDFYPKKIIVRWTRDGRPETTGVTSTDELADGDWYYQTHSHLEYTP
SRSGEKISCVVEHISLSKPLVTDWTDPSMPESERNKVAIGASGLILGLTLSLAGFIYYKR
KARGRILVPSH
>G131A2_DAA ENSGACG00000000344_ENSGACP00000000434_scf_131:78913-81169
MKTKTMMKMMVVLVLSGVFCVSADGPHKDIRVLGCSDSDGENMFGLDGEEVWYADFKHGK
GVRPLPSFVDPIEFPGGAYEQAVTDLQICRSNLKTHVKGLKDVPLEKDPPSSHMIYPKDG
VELGEKNSLICHVTGFYPAPVTFSWTKNQENVTEESSRNVPYLNNDGTFNQFSTLKFTPK
LGDIYSCMVEHLALDHPLVKFYDVQVSQPSVGPAVFCGVGLTVGLLGVAAGTFFLIKGNE
CS
>G131B2_DAB ENSGACG00000000346_ENSGACP00000000437_scf_131:96885-99355
MAPSFISVSLLFIGLHAADGFLEFMTDECVFNSTELKDIEYITSYYYNKLELTRFXINVL
VPLLYYQQNLLQFVRSFYTQWEPCRTLLLVLILVPVSSQNKPSPCHILSPAEPYVRLHSE
TPPGGGPLSMLVCSVYDFYPKKIIVRWTRDGRPETTGVTSTDELADGDWYYQTHSHLEYT
PSRSGEKISCVVEHISLSKPLVTDWNPSMPESERNKVAIGASGLILGLTLSLAGFIYYKR
KARGRILVPSH
>G131A3_DBA ENSGACG00000000348_ENSGACP00000000439_scf_131:118613-120538
MKTKTMMKMMVVLVLSGVFCVSADGPHKDIAIDGCSDSDGEKMYGLDGEEVWYADFKHGK
GVKPQPSFVDPIDYPAGAYELAVANQQTCRNDLKTDLEAYKDVPLEKDPPSSHMIYPKDG
VELGEKNSLICHVTGFYPAPVTFSWTKNQENVTEGSSRNVPYPNNDGTFNQFSTLEFTPK
LGDIYSCMVEHLALDHPLVKCVFLSPDVQVSQPSVGPAVFCGVGLTVGLLGVAAGTFFLI
KGNECS
>G131B3_DBB ENSGACG00000000350_ENSGACP00000000450_scf_131:121399-126270
MAPSFISVSLLFIGLHAADGFMEFRTDECVFNSTELKDIEFIRSSYYNKKEDTRFSSSVG
KFVGFTEQGVKYAANWNKDASYLSAMRAQKEVYCLNNVQIKYDNALTKSAEPYVRLHSET
PPGGGPLSMLVCSVYDFYPKKIIVRWTRDGRPETTGVTSTDELADGDWYYQTHSHLEYTP
SRSGEKISCVVEHISLSKPLVTDWNPSMPESERNKIAIGASGLILGLTLSLAGFIYYKRK
ARGRILVPSH
>GIIIB ENSGACG00000017967_ENSGACP00000023736_groupIII:16679542-16682223
MAPSFISVSLLFIGLHAADGFLEFMTDECVFNSTELKDIEYIRSYYYNKLELTRFSSSVG
KYVGFTEYGVRNAANWNKDASELIAMRAQKEVYCLNSIQIKYNNVLTKSAEPYVRLHSET
PPGGGPLSMLVCSVYDFYPKKIIVRWTRDGRPETTGVTSTDELADGDWYYQTHSHLEYTP
SRSGEKISCVVEHISLSKPLVTDWNPSMPESERNKVAIGASGLILGLTLSLAGFIYYKRK
ARGRILVPSH
>GVIIB ENSGACG00000019051_ENSGACP00000025189_groupVII:2444693-2447881
MAPSFHSVSLLFIFLHAAGGFMEFVKDQCVFNSTDLKGIEFIRSTYFNKLQYTKFSSSVG
KFVGFTEQGMKNAAASNNNNMYMASIRAAKETICQPNIQRMYDNILTKSAQPYVRLHSET
PLGGRTSSMLVCSVYDFYPQNIIVRWTRDGRPEATGVSSTDELADGDWYYQTHSYLEYTP
SRSGEKIACVVEHISLSKPLVTDWDPSMPESERNKIAIGGSGLILGLTLSLAGFIYYKRK
GRGRILVPSY
>GVIIA ENSGACG00000019052_ENSGACP00000025193_groupVII:2449972-2451242
MKTMKMMMMIVVLVLSGVSAYVPHEAIRISGCSDSDGEEMFGLDGEELWYADFKLGKGVS
LLPSFLDPITYPGGYEVAVAEQQFCRNNLRIDLEAYKFPLERDPPSSHMIFPKDAVELGE
KNSLICHVTGFYPAPVAFSWTKNQENATEGTSRNIPFPNNDGTFNQFSTLEFTPELGDIY
SCMVEHLALDHPLVRFYDVQMSQPSIGPAVFCGVGLTVGLLGVAAGTFFLVKRNECS
>GXVIIB ENSGACG00000003680_ENSGACP00000004837_groupXVII:1117113-1119770
MSSSTFLLLLCLSSCSAADGHGYFMYADFWCNMQTARPQQVEYLVDWYFNKEFTMQYNST
VGKWTGFTAAGLVSAAVFNGNHFDVLQRKEERRLICVDNVGHALNATEDNMAAPSVRLAE
ASGSGHNTTLVCSAYDFYPRRIRLAWLRDGQEVTSGATFSEVTTNGNWTYAVHSSLSFTP
GGRDRVSCKVEHAGLQEPALRTWAAHGARDWETGFLVGGVCALLLGAACLSSGLIVHRRK
YSNIS
>GXVIIA ENSGACG00000003731_ ENSGACP00000004896_groupXVII:1163942-1165035
MAVTLMLALAAALCTSAAGSSHDFHYTYGCYESGEVRVDVLLDGDVVAYADFGREEVVFL

IPRLPPFLRDLKKLGYEFAKSSFTHCRSVLAKAKRASPNVTIPQDAPVLSVYSRHEGRGG
AANTLFCLADGFYPPSVNFTWTKNGARVTGGVWDLPYGHNRDGTFHRISTLSTTPREGDV
YSCSVEHRAARRPLASSWAEPKEGPSRVSPAAGFFGASLVVCLIGVASGAYFFTKQPNFG
>GXXB Gr.XX: 1081373-10814066
EPSVRLRSVHAADSRHPRMLLCTGYGFYPKQIRVMWLRNGNNVTSDVTSTEELSNGNWLY
QIHSYLEFTPRSGGEISCVVEHASLTQPKVYDW

Stickleback translated Genbank cDNA report sequences:

>DW594610 CGX114-A05.y1d-s SHGC-CGX2 Gasterosteus aculeatus cDNA clone CGX114-A05 5, mRNA sequence GXVIIB match
MSSSTFLLLLCLSSCSAADGHGYFMYADFWCNMQTARPQQVEYLVDWYFNKEFTMQYNST
VGKWTGFTAAGLVSAAVFNGNHFDVLQRKEERRLICVDNVGHALNATEDNMAAPSVRLAE
ASGSGHNTTLVCSAYDFYPGRIRLAWLRDGQEVTSGATFSEVTTNGNWTYAVHSSLSFTP
GGRDRVSCKVEHAGLQKPALRTWAAHGARDWETGFLVGGVCALLLGAACLSSGLIVHRRK
YSNIS
>DV011001 CNB266-F02.y1d-s SHGC-CNB Gasterosteus aculeatus cDNA clone CNB266-F02 5, mRNA sequence G131B1, G1313B3 and GIIIB match
MAPSFISVSLLFIGLHAADGFMEFWTDECVFNSTELKDIEFIRSYYFNKLEFTRFSSSVG
KYVGFTEYGVRNAEYWNKDASQLSAMRAQKEVYCLNNVQIDYNNILTKSAEPYVRLHSET
PPGGGPLSMLVCSVYDFYPKKIIVRWTRDGRPETTGVTSTDELADGDWYYQTHSHLEYTP
SRSGEKISCVVEHISLSKPLVTDWNPSMPESERNKVAIGASGLILGLTLSLAGFIYYKRK
ARGRILVPSH
>DW672198.1_3 CNB369-F02.y1d-s SHGC-CNB2 Gasterosteus aculeatus cDNA clone CNB369-F02 5', mRNA sequence G131A1 and G131A2 match
MKTKTMMKMMVVLVLSGVFCVSADGPHRDIAINGCSDSDGELMYGLDGEEIWYADFKHGK
GVKAQPSFVDPIEFQGAYEQAVANQQTCRSNLKNDLKAFKDVPLEKDPPSSHMIYPKDGV
ELGEKNSLICHVTGFYPAPVTFSWTKNQENVTEGSSRNVPYLNNDGTFNQFSTLEFTPKL
GDIYSCMVEHLALDHPLVKFYDVQVSQPSVGPAVFCGVGLTVGLLGVAAGTFFLIXGNEC
S

Medaka (*Oryzias latipes*) MHC class II sequences

Medaka Ensembl sequences:

>M3A ENSORLG00000000025_ENSORLP00000000027_Chr.3:558225-564744
LLSDLKVMKVKLLLFLCEILCGILSGTAEVLHEDFGILGCSDYDQEYMYALDGEEKWYAD
YKKETGVGPQLPLGGHFINPDGYQFAKVYEQVCRRNLKALKKTTKGLQLEQDPPSNVMVY
SRDEVELGQQNTLICHVSGFYPAPVNVSWTRNGEKVPGSINVPLPSSDGTLTQISRLQFV
PQLGDIYSCSVEHPALPEVQTKIWDFEKSQPSVGPALFCGLGLVVGLLGVATGTFFLIKG
NECS
>M3B ENSORLG00000000021_ENSORLP00000000030_Chr.3:554841-556227
MDSSSLRVFLLILTLYPADGFIHYILNRCLFNSSDLKDIENIYSYYYNKEEFLRFSSSSG
KFVGYTEIGVKTAELANNDPEKMSRRRAEKETFCKPNIDNDYSTILTKSVQPRVRVQSLE
PSGGNHPAMLICSVYDFYPKKIKVSWLQDQEEVSSDVTSTAEMEDGDWFYQIHSYLEYTP
RSGGKISCRVEHISLKDPLITDWDPSMPESEKNKIAIGASGLILGLVLSLAGFVYYKRKT
RGRILVPSS
>M5A ENSORLG00000012794_ENSORLP00000016018_Chr.5:25231039-25236341
MSLTSLLLITGAVCASSTTPRHMFHFIYGCYETDEVRVDLVVDDDTIGYADFTKQEMVWC
LPYVPPSGKDLEKEAFKFAKNSIANCHSVLAKAKKADHGTPLRQEPPDLSIYTRYKAEEG
VLDTLFCSANHFYPPTINFTWTKNGAEVTEGLLNLRFSHNKDGTFRGISTLSFTPQRGDV
YSCWVSHEALERPRIITWESRRRRSPARMFFCASLILCLAGIGTGLYFFIKKPNYCCGCQ
>M5B1 ENSORLG00000012822_ENSORLP00000016051_Chr.5:25371618-25375673

ILSSLIYFYLLFLTSYYSKDQHGYFMFSDFFCYIPSRNPKEVQYLIDWYFNMELTMQYNS
SVGGWTGFTPAGLITAAKFNADKYDVVQRILERELVCQRSVEMVYNGTEEAKVEPNVSLQ
TVEDNDSTLECSALDFYPKHIRLTWFSNGQEVTEGVTFSDVLPNGDWTYQAHTYLTLTPG
KQDHISCMVQHTSLKEPKIYNWEPSMNQTDRDYIIGGVCALLLGAVFLCVGLIHYKQK
>M5B2 modified from ENSORLG00000012826_ENSORLP00000016062_Chr5:25378453-25379616
YSKDQHGYFMFSDFLCYLPSRNPKEVQYLIDWYFNMELTMQYNSSVGGWTGFTPAGLITA
AKFNADKHDVVQRILERELVCQRSVEIIYETTETAKVEPNVSLQTVEDNDSTLECSALDF
YPKHIRLTWFSNGQEVTEGVTFSDVLPNGDWTYQAHTYLTLTPGKQDHISCMVQHTSLKE
PKIYNWVCALLLGAVFLCGGLIHYK
>M16B ENSORLG00000009164_ENSORLP00000011497_16:14786594-14792142
TGKSQNCSLGFLPLFLLFSSTNAFYGHGTLKCQFTSSHDLVYLEQVYFNKRLMVQYNSTL
GKYEGYTKKAKDLADGFSKSKPFLEQAVKNREKCRTHMDLVFELQSHPVEPSVRVTPVVR
QGSSHQAMLACSAYNFYPKQIRLTWLRNGEKVINYVTSTEELPDGNWLYQIHSYLEYTPS
PREEITCMVEHASPKEPKLYNWEVLSPPMFGAVRNKIAVGTALLFGSFVFVAGLFFYKRT
T
>M16A ENSORLG00000009164_ENSORLP00000011499_16: 14790903-14792142
SRKQEGFKCSLIHFKTSFFCFVFSVEHEISYFIGCFAEGSTEVLLEFDSEEILYVDFEKE
AVVFTGPSFFKANLSERIQGLTTYKNGKKNRIWCQLADQYFTAETEERDKDPPGEVLIYT
SAEVQPGVENTIICFVNGFYPPSIKVSWTKNGNPVSEGVSHSRYYPNKDQTFHQFSTLSF
TPSWTDVYSCTVEHPALESPKTVLWEPEFHHQHPNLDVFLAASLGLGLLGFAVGTCLIII
ALKRS
>M615B ENSORLG00000019376_ENSORLP00000024128_scf.615:38464-43568
TDMDFSSLCLLFLTLYSADGFLEYDMHRCLFNSSDMKDIEYIYSKHYNKEEVIRFSSSLG
KYVGYTKFGVMAAGYWNKDLEGLRERRADKERLCKTNIDLDYLNILTKSVKPSVMIESVT
SSGGHHPAMLVCSVYDFYPKYIKVSWLRDQEEVSSDVTSTTELEDGDWYYQIHSHLEYTP
RSGEKISCRVEHASLKDPLITDWDPSMPESEKNKLAIGASGLILGLVLSLAGFIYYKRKA
LGRSSLNRF
>M615A ENSORLG00000019409_ENSORLP00000024163_scf.615:87448-89094
FLLPLSLIKVMKIKLLLFLCGILSGTAEVFHEDFQITGCSDYDEEFMVNLDGEEIVYTDF
KKGTEVDAQPSFVGHITYAGGYGATVLELQGCRNDLEVIRRFTKDLPLKQDPPSNVVVYS
RNKVELGQQNTLICHVSGFYPAPVNVSWTRNGEKVSGSINVPRPSSDGTFSQISRLQFVP
QLGDIYSCSVEHPALPEVQTMIWDVEKSQPGIGPAVFCGLGLVVGLLGVATGTFFLIKGN
ECS
>M873B ENSORLG00000018856_ENSORLP00000023542_scf.873:60767-63324
MDSSSLCLLFLTMCSADAFLRYDVDRCVFNSTDLKDIEYIYSMYYNKKEFTRFSSSLGKY
VGYTEYGVKTAERANKDTSELSARKAQKETYCKHNIDNWYKNMLSKSVQPRVRVQSLAPS
GGHHPAMLVCSVYDFYPKTIRVSWLRGKEEVSSDVTSTAEMEDGDWYYQIHSHLEYTPRS
GEKISCKVEHASLKDPLVTEWDPSMPESERNKVAIGASGLILGLVLSLAGFIYYKRKARG
RILVPSS
>M873A ENSORLG00000018887_ENSORLP00000023574_scf.873:65205-67367
MKMKLLLFVCGILSGTAAVFHEDLAITGCSDSDGEDMYALDGEEVWYADFKKQTGVEPQP
PFVDHVSYPGGYEQAVANQQICRSNLKISRIAMKDLPLERDPPSNVVVYSRDEVELGEQN
TLICHVSGFYPAPVNVSWTKNGERVSGSINIPFPSSDGTFTQISRLPFVPQLGDIYSCSV
EHPALTEVQTKIWDVEKTQPGVGPAVFCGVGLAVGLLGVAAGTFFLIKGNECS

Medaka translated Genbank cDNA report sequences:

>FS518487.1_2 FS518487 OLTE Oryzias latipes cDNA clone olte28i23 5', mRNA sequence M873B match
SLTANTDMDSSSLCLLFLTMCSADAFLRYDVDRCVFNSTDLKDIEYIYSMYYNKKEFTRF
SSSLGKYVGYTEYGVKTAERANKDTSELSARKAQKETYCKHNIDNWYKNMLSKSVQPRVR
VQSLAPSGGHHPAMLVCSVYDFYPKTIRVSWLRGKEEVSSDVTSTAEMEDGDWYYQIHSH
LEYTPRSGEKISCKVEHASLKDPLVTEWGPVHAR
>BJ883759.1 BJ883759 MF01ASD cDNA Oryzias latipes cDNA clone MF01ASD033f06 5', mRNA sequence M873A match

MKMKLLLFVCGILSGTAAVFHEDLAITGCSDSDGEDMYALDGEEVWYADFKKQTGVEPQPPFVDHVSYPGGYEQAVAN
QQICRSNLKISRIAMKDLPLERDPPSNVVVYSRDEVELGEQNTLICHVSGFYPAPVNVSWTKNGERVSGSINIPFPSS
DGTFTQISRLPFVPQLGDIYSCSVEHPALTEVQTKIW
>BJ877464 MF01ASD cDNA Oryzias latipes cDNA clone MF01ASD014g19 5, mRNA sequence M5A match
ADSLVQLLTVLGFKMSLTSLLLITGAVCASSTTPRHMFHFIYGCYETDEVRVDLVVDDDT
IGYADFTKQEMVWCLPYVPPSGKDLEKEAFKFAKNSIANCHSVLAKAKKADHGTPLRQEP
PDLSIYTRYKAEEGVLDTLFCSANHFYPPTINFTWTKNGAEVTEGLLNLRFSHNKDGTFR
GISTLSFTPQRGDVYSCWVSHEALERPRIITWESR
>BJ891116 MF01ASD cDNA Oryzias latipes cDNA clone MF01ASD014g19 3, mRNA M5A match
DDDTIGYADFTKQEMVWCLPYVPPSGKDLEKEAFKFAKNSIANCHSVLAKAKKADHGTPL
RQEPPDLSIYTRYKAEEGVLDTLFCSANHFYPPTINFTWTKNGAEVTEGLLNLRFSHNKD
GTFRGISTLSFTPQRGDVYSCWVSHEALERPRIITWESRRRRSPARMFFCASLILCLAGI
GTGLYFFIKKPNYCCGCQ

Fugu (*Takifugu rubripes*) MHC class II sequences

Fugu Ensembl sequences:

>F402A ENSTRUG00000004342_ENSTRUP00000010322_scaffold_402:55188-57563
MMKMLLLILCCVVGASADSQHEDIRILGCSDLDGEFMYGLDSEEVWYADFSKGEGVDINP
PFIDPITYEEGAYSSAVADLQTCKTSLDITRKSLKDMPPERVAPTSPVIYTKEEVQLSQQ
NTLICFVTGFYPAPVNVSWTRNGEHVTQGTSINVPYPNKEGTFTQISRLAFVPQQGDIYS
CRVQHPALSGQDTRMWTVEVQQPGVGPAVFCGLGLTLGLLGVAAGTFFLIKGNECR
>F713A fuguDAA_scf 713:89-334
QHKDINIVGCSDLDGEFMYGLDSEELWYADFSKGEGVYGTPPFIDPFRYEEGVYSQAVTE
LQTCKTNLDSERKAYKDMPPET
>F7533B ENSTRUG00000017449_ENSTRUP00000044727_scaffold_7533:2656-4187
SRMSSSSLRVFLLFISLYTAAGGFQSYVVDSCDFNSTDLKDIEYTRSLYYNRVMYARFSS
RVGKFEGYTKYGLFQADYWNNQSSILEGLRETKESICQPNIKIDYSNILSKSVEPTVRVH
SVVPPAGGHPAMLVCSVYDFYPRYIKVSWQRDGEEVSQDVTSTDELADGDWFYQLHSHLE
YTPRSGEKISCVVEHASLKTPLVKDWGNHLSVCS
>F7533A ENSTRUG00000017444_ENSTRUP00000044708_scaffold_7533:430-1814
MMKMLLLILCCVLGVSADSQHKDIGIVGCSDLDGEFMYGLDSEELWYADFSKGEGVYGTP
PFIDPFRPTAAAVADLQTCKYNLDVDRKGNKDMPTETVAPTSPVIYTKEEVQLSQQNTLI
CFVTGFYPAPVNVSWTRNGEHVTQGTSINVPYPNKEGTFTQISRLAFVPQQGDIYSCRVQ
HPALSGLDTRMWTVEVQQPGVGPAVFCGLGLTLGLLGVAAGTFFLIKGNECR
>F7721A ENSTRUG00000000833_ENSTRUP00000002005_scaffold_7721:158-1461
MKMLLLILCCVLGVSADSQHKDIRITGCSDLDGEDMFGLDSEELWYADFSKGEGVDGTPP
FIDPVRFEGFYSTAVTNLQICKSNMDVVRKGNKDMPPETVAPTSPVIYTKEEVQLSQQNT
LVCFVTGFYPAPVNVSWTRNGEHVTQGTSINVPYPNKEGTFTQISRLAFVPQQGDIYSCR
VQHPALSGQDTRMWSRQADRQADRQAGR
>F7721B fuguDAB_Scf 7721:2626-3027_no enstrug name
MSSSSLRVFLLFISLYTAGGFQSYRVDRCDFNSTDLKDIEYSRSHYYNKLMYVRFRSSVG
KFEGYTKDGLIQADYWNNISSYLEQMRDEKERYCEPNIKVWYSNILSKS
>F8524B ENSTRUG00000002062_ENSTRUP00000004737_scaffold_8524:1050-3165
MSSSSLRVFLLFISLYTAGGFQSYRVDRCDFNSTDLKDIEYSRSHYYNKLMYVRFRSSVG
KFEGYTKDGLIQADYWNNISSYLEQMRDEKERYCEPNIKVWYSNILSKSVEPTVRVHSVV
PPAGGHPAMLVCSVYDFYPRYIKVSWQRDGEEVSQDVTSTDELADGDWFYQLHSHLEYLP
ACLSALLSVCLSETHLSVPTCLPSDPSMPESERNQIAIGASGLILGLILSLAGFIYFKRK
SRGRILVPNN

Fugu translated Genbank cDNA report sequences:

```
>CA846190 haa10g02.y5 Fugu UT11 adult fin Takifugu rubripes cDNA clone
IMAGE:6343467 5 similar to TR:Q31462 Q31462 MHC CLASS II PROTEIN.mRNA F7533B
match
MSSSSLRVFLLFISLYTAGGFQYYVVSRCSFNSTDLKDIEYSRSVYYNKLMYARFSSSVG
KFEGYTKYGLFQADYWNNQSSILEQVRDNKERYCQHNIKNWYSNILSKSVEPEVIVHSVV
PPAGGHPAMLVCSVYDFYPRYIKVSWQRDGEEVSQDVTSTDELADGDWFYQLHSHLEYTP
RSGEKISCVVEHASLKTPLVKDWDPSMPESERNQIAIGASGLILGLILSLAGFIYFKRKS
RGRILVPNN
>CA846902 hab89d12.y1 Fugu hgmpC adult gill Takifugu rubripes cDNA clone
IMAGE:6355654 5 similar to TR:Q31465 Q31465 MHC CLASS II ALPHA. contains MSR1
repetitive element; mRNA sequence. F402A and F7533A match
RYEEGVYSQAVTELQTCKTNLDSERKAYKDMPPETVAPTSPMIYTKEEVQLSQQNTLICF
VTGFYPAPVNVSWTRNGEHVTQGTSINVPYPNKEGTFTQISRLAFVPQQGDIYSCRVQHP
ALSGLDTRMWTVEVQQPGVGPAVFCGLGLTLGLLGVAAGTFFLIKGNECR
>AB453019 Takifugu rubripes mRNA for MHC class II alpha chain, complete cds
F7533A match
MMKMLLLILCCVVGASADSQHEDIRILGCSDLDGEFMYGLDSEEVWYADFSKGEGVDINP
PFIDPITYEEGAYSSAVADLQTCKTSLDITRKSLKDMPPERVAPTSPVIYTKEEVQLSQQ
NTLICFVTGFYPAPVNVSWTRNGEHVTQGTSINVPHPNKEGTFTQISRLAFVPQQGDIYS
CRVQHPALSGQDTRMWTVEVQQPGVGPAVFCGLGLTLGLLGVAAGTFFLIKGNECR
```

Tetraodon (*Tetraodon nigroviridis*) MHC class II sequences

Tetraodon Ensembl sequences:

```
>T19A ENSTNIG00000005593_ENSTNIP00000008293_Un_random:19583822-19591157
MKMLLLILCCILGVSADGQHEDIRIVGCSDFDGEYMHGLDGEELWYADFRRGEGVYAQPD
FIDPIKYEGFYSTAVAVLQICKFNLGIFRKGYKDMPRELDPPTSPMVYTRNEVQLTEPNT
LVCLVTGFYPAPVNVSWTRNGEEVTQGTSINVPYPNNEGTFTQISRLDFVPQQGDIYSCR
VQHPALSEADTRMW
>T19B ENSTNIG00000005592_ENSTNIP00000008294_Un_random:19597929-19598797
MASSALRVSLLFLGLSAAGAFEHYGLRRCDFTSAEPKDMEYSLSVYYNKHLMARFSSSVG
KFVGYDKYGQYQADYWNNQSSFLEAMRSSKQRLCQHNIPLWYSHILSKSVLKDVFVYSVA
PPAGGHPAMLVCSVYDFYPKKIKVSWRRDGQEVSHDVTSTDELADGDWFYQLHSHLEYTP
RSGEKISCVVEHASLKTPLVKDWDPSMPEAERNQIAIGASGLILGLILSLAGFIYFQRKS
RGRILVPTN
>T48B ENSTNIT00000004743_ENSTNIP00000004600_Un_random: 48,695,828-48,696,540
VLVYSVAPPAGGHAAMLVCSVYDFYPKKIKVSGRRDGQEVSHDVTSTDELADGDWFYQLH
SHLEYTPRSGEKISCVVEHASLKTPLVRDWDPSMPEAERNQYAIGASGLILGLILSLAGF
IYFQRKSRGRILVPTN
>T55A ENSTNIG00000000926_ ENSTNIP00000001278_Un_random:55779303-55780575
MKMLLLILCCVLGVSADGQHEDIRIVGCSDFDGEYMYGLDGEELWYADFRRGEGVYAQPD
FIDPLKYAEDPYSLAVATLQMCKFNLGIFRKGYKDMPRELVPPTSPMVYTRSEVQLTEPN
TLVCLVTGFYPAPVNVSWTRNGEEVTQGTSINVPYPNNEGTFTQISRLDFVPQQGDIYSC
RVQHPALSEADTRMWTVEVKQPGVGPAVFCGVGLTLGLLGVAAGTFFLIKVNQCR
>T61A ENSTNIG00000003745_ENSTNIP00000001966_Un_random:61621456-61622716
MKMLLLILCCVLGVSADGRHEDVHIRGCSDFDGEDMYGLDGEERWYADFKRGEGVYALPD
FVDPLKFPEGTYSQAVADQQICKINLGISRKGNKDMPRELVPPTSPMVYPSSEVQLTEPN
TLVCLVTGFYPAPVNVSWTRNGEVTQGTSINVPYPNNEGTFTQISRLDFVPQQGDIYSCR
```

```
VQHPALSEADARMWTVEVKQPGVGPAVFCGVGLTLGLLGVAAGTFFLIKGNQCR
>T61B ENSTNIG00000000531_ENSTNIP00000006345_Un_random:61617277-61620814
MASSALRVSLLFLGLSAAGGFEHYEVDLCDFTSAELKDIEYSRSLYYNKHLYARFSSSVG
TFVGFTKYGLHQADYWNNQSSYLDRLKEEKQRYCHRNIQNWYSHILSKSSHDVTSTDELA
DGDWFYQLHSHLEYTPRSGEKISCVVEHASLKTPLVKDWDPSMPEAERNQIAIGASGLIL
GLILSLAGFIYFQRKSRGRILVPTN
>T70B ENSTNIG00000004611_ENSTNIP00000007260_Un_random: 70,836,268-70,837,419
LKDPTVRVHLVAPPAGGHAAMLVCSVYDFYPKKIKVSWRRDGQEVSHDVTSTDELAD
GDWFYQLHSHLEYTPRSGEKISCVVEHASLKTPLVKDWDPSMPEAERNQIAIGASGLILG
LILSLAGFIYFQRKSRGRILVPTN
```

Tetraodon translated Genbank cDNA report sequences:

```
>CR731060.2 Tetraodon nigroviridis full-length cDNA T19A+T55A match
MKMLLLILCYVLGVSADGQHQDIKISGCSDFDGEFMYGLDGEELWYADFRRGEGVYPLPD
FIDPFRYDEGVYSLAVATLQVCKFNLGLFRKGYKDMPRELDPPTSPMVYTRNEVQLTEPN
TLVCLVTGFYPAPVNVSWTRNGEEVTQGTSINVPYPNNEGTFTQISRLDFVPQQGDIYSC
RVQHPALSEADTRMWTVEVKQPGVGPAVFCGVGLTLGLLGVAAGTFFLIKGNRSR
>CR732064.2 Tetraodon nigroviridis full-length cDNA T19A+T55A match
MKMLLLILCCVLGVSADGQHQDIKISGCSDFDGEFMYGLDGEELWYADFRRGEGVYPLPD
FIDPFRYDEGVYSLAVATLQVCKFNLGLFRKGYKDMPRELDPPTSPMVYTRNEVQLTEPN
TLVCLVTGFYPAPVNVSWTRNGEEVTQGTSINVPYPNNEGTFTQISRLDFVPQQGDIYSC
RVQHPALSEADTRMWTVEVKQPGVGPAVFCGVGLTLGLLGVAAGTFFLIKGNQSR
>CR682088.2 Tetraodon nigroviridis full-length cDNA T61A match
MKMLLLILCCVLGVSADGQHEDVHIKGCSDFGGEDMYGLDGEERWYADFKRGDGVYALPD
FVDPLKFVEGTYSQAVADQQVCKINLGISRKGNKDMPRELVPPTSPMVYPSSEVQLTEPN
TLVCLVTGFYPAPVNVSWTRNGEVTQGASINVPYPNNEGTFTQISRLDFVPQQGDIYSCR
VQHPALSEADTRMWTVEVKQPGVGPAVFCGVGLTLGLLGVAAGTFFLIKGNQCR
>CR727057.1 Tetraodon nigroviridis full-length cDNA T19B match
MASSALRVSLLFLGLSAAGAFKHYEVDLCDFTSAELKDIEFSRSVYYNKHLYARFSSSLG
KFVGFTKWGLRVADYWNNQSSYLDQLRGEKQRFCLHNIQVWYSNILSKSVLKDVLVYSVA
PPAGGHAAMLVCSVYDFYPKKIKVSWRRDGQEVSHDVTSTDELADGDWFYQLHSHLEYTP
RSGEKISCVVEHASLKTPLVKDWDPSMPEAERNQIAIGASGLILGLILSLAGFIYFQRKF
RGRILVPTN
>CR697461.3 Tetraodon nigroviridis full-length cDNA T19B match
MASSALRVSLLFLGLSAAGAFEHYWVSRCDFTSAELKDIEFSRSFYYNKHLNVRFSSSLG
KFVGYDKFGQHQADYWNNQTSFLEAMRDSKQRYCQHNIQNWYSNILTKSVLKDVLVYSVA
PPAGGHAAMLVCSVYDFYPKKIKVSWRRDGQEVSHDVTSTDELADGDWFYQLHSHLEYTP
RSGEKISCVVEHASLKTPLVKDWEPSMPEAERNQIAIGASGLILGLILSLAGFIYFQRKS
RGRILVPTN
>CR726114.2 Tetraodon nigroviridis full-length cDNA T19B match
MASSALRVSLPFLGLSAAGAFKEYTVDRCDFTSAELKDIEFTRSEYYNQHLVVRFSSSLG
KFVGFDKYGQYQADYYNNQSSILEAMRGEKQRYCLNHIPVWYSNILSKSVLKDVLVHSVA
PPAGGHAAMLVCSVYDFYPKKIKVSWRRDGQEVSHDVTSTDELADGDWFYQLHSHLEYTP
RSGEKISCVVEHASLKTPLVKDWDPSMPEAERNQYAIGASGLILGLNLFLAGFIYFQRKS
RGRILVPTN
```

Tilapia (*Oreochromis niloticus*) MHC class II sequences

Tilapia Ensembl sequences:

```
>O1A ENSONIG00000006325  GL831301.1: 1,431,629-1,433,771
MKMKELLLFLSCVLCVSANFLHEDIHIVGCSESNGEVMFGLDGEELWYADFENKIGVSPQ
```

PSFVDPISFGDGAYETAVINLYICKTNMQVSQEGMKDYPLNHDAPSGVMIYTRNEVELRV
TNTLICHVTGFYPGPVKVSWTKNGQKLTEGFSINVPYPNKDGTFKQIARLQFIPQQGDIY
SCTVEHLALKEPLTKIYNVEAPEKPKPSVGPAVFCGLGVIIGLLSAAGGTFFILKANKCT
>O1B ENSONIG00000006327 GL831301.1: 1,435,653-1,439,547
MASSFLCFTLLFISIHNADGFLEYIVDRCDFNSTELENMEYIYSHYYNKMEYIRFSSNVG
QYVGFTDFGVRLAEVWNNDTADLNLMRLKIATYCHHNIDINDRAILTKSVQPSVRIKSMT
PPSGQHPAMLVCSVYDFFPSKIKVSWLRDGQAVSSDITSTEEMPNGDWYYQTHSQLEYTP
RSGEKISCVVEHASLKEPLITDWDPSSDKSTSESTTLIAIGASILTLGLILLLAGFIFYR
WRARDPTLFSNE
>O9A ENSONIG00000019112 GL831209.1: 2,227,167-2,229,515
MDSSLMLLALASCVFTAHAAVHEETLFYSCSESGDGQVQLHYDGDQITYADFKKQHAVWT
APLLKDLETAILSWFFPLALDSRTRCEFYLDRAVRLDNSSMTLKAPAVVIYPMDEAETGK
NNTIYCHIRHFYPPSNNVTWTRNGIRVTEGVNLSNPYPQTDGTFNQLASFSFQPQSDDVV
ECSVQHQALSRPLITTWTLPMKPARSAAAWVCLSLGLLCIALGVIIFIISAKESLRHKLS
>O9B ENSONIG00000019113 GL831209.1: 2,230,917-2,233,060
AVCSLLLLLLLLRSKGVAEDEKYFHILKTCQFIEKSSQSDVHYQVQYWYNGLLQAFYNSS
TEKVVGFTHYGKVFADEVNKSPDYLKVRRNDLNYFCKVLGAVLYKDIQVKAVPPVSRLKS
VTSNSSEDSEVLVCSAYNFYPQQIRLSWLRDGVVIPDPPGMMITEMPGGEWRYQIHSYLQ
HKLNSGQNITCMIEHRGLQEPQLLKLERSEVQSNSVTLAWEGAILATGLFVLLASVVFHC
RKTHCIIFVGSF
>O18B1 ENSONIG00000019932 Scaffold GL831518.1: 4,104-13,309
MASSFLCLSLLFISLSTAGGFMHYSVDRCNFNSSEPQDIEYIRSYYFRKVEFTRFDSSVG
KYVGYTEYGVKNAEEWNKDKGNLAAMNAQKEVYCQKKIDNRYNAILPKSAKPYVKLHSTM
PLSSHHPAMLVCSVYDFFPSEIKVSWQRDGQEVTSDVTSTEEMADGDWYYQTHSHLEYTP
RSGEKISCVVEHASLEKPLITDWDPSMPESERNKLAIGASGLILGVILSLAGFIYYKRKA
RGRILVPTN
>O18B2 ENSONIG00000019937 GL831518.1: 312,391-316,936
MASSFLCLSILFISFSTADGFMEYHVDRCNFNSTDLKDIEYIYSCYYNKIEYARFSSSVG
KFVGFTKHGVYNADKWNNDPAELNNRRAQKERYCHNNIGVDYQVALTKSAKPYVKLHSMT
PASSHHPAMLVCSVYDFFPSKIKVSWLRDGQEVSSDVTSTEEMADGDWYYQTHSHLEYTP
RSGEKISCKVEHASLEKPLITDWDSSMPESERNKLAIGASGLILGLILSLAGFIYYRRKA
RGRILVPTN
>O18B3 ENSONIG00000019944 GL831518.1: 404,131-408,913
MASSFICLLFISLSTADGFKMYVVSRCDFNSTELKDIKYIRSYYYNKIEVTRFDSDVRKF
VGFTGFGVKQAKHFNSQPGRLATLKAEKERYCQHNIGIWYSNVLPKSDSEEADGDWYYQI
HSHLEYTPRSGEKISCKVEHASLEKPLITDWDSSMPESERNKLAIGASGLILGLILSLAG
FIYYRRKARGRILVPQH
>O18A1 ENSONIG00000019933 GL831518.1: 18,305-20,484
MKMKELLLFLSCVLSVSADVLHEDIGIVGCSDSDGEDMYGLDGEEVRYADFKKQEDINSL
PPFADLITCPGCYEQAVANQQVCKQNLQTLRKMMKDYPLEHDAPSAVMIYTRDEVEFGEK
NTLICHVTGFYPAPVNVSWTKNEQKVTERSSINVPYPNKDGTFTQISRLQFTPQQGDIYS
CTVQHLALTQPATKIYDVQASAQSDPGFGPAAFCGVGLTVGLLGVAAGTFFLIKGNECS
>O18A2 ENSONIG00000019940 GL831518.1: 317,998-320,025
MKMKELFLFLSCVLCVSADALHSDIAINGCSDSDGEFMYGLDGEEKWYADFNKHTGVDAP
PDFIGSFNYQEGAYENAVANQQICRANLKVDSKGMKDFPLEHDAPSGVMIYTRDEVEFGE
ENTLICHVTGFYPAPVNVSWTKNEQKVTQGSSINVPYPNKDGTFTQISRLEFTPQLGDIY
SCKVQHLALTQPLTKIYEVEASARSDPGVGPSVFCGVGLTVGLFGVAAGTFFLIKGNECS
>O18A3 ENSONIG00000019943 GL831518.1: 399,821-401,995
MKMKELLLFLSCVLCVSADPLHSDIAIGGCSESDGENMYGLDGEEIAYADFNKQEFIYPQ
PPFIDPITVPGGYESSVAFQHSCRENMKLFGKGMKDYPPEQDAPSAVMIYTRDEVEFGEK
NTLICHVTGFYPAPVNVSWTKNEQKVTQGSTINVPYPNKDGTFTQISRLQFTPQQGDIYS
CAVQHLALTQPLTKIYEVEASAHSDPGVGPSVFCGVGLTLGLVGVAAGTFFLVKGNECS
>O22A1 ENSONIG00000012437 Scaffold GL831322.1: 920,733-935,169
MKMKVWVLLLVLSSVPCVSADGLHETSSVSGCSDSDGEDVLILDDEVVWYADFYNQKGVD
SLPAFADHPHWKDGMYEQAVVNQQICRYNLKMARTAMKDFPKQRDAPSSVNIYTTLNVEI
GVQNTLICHVTGFYPAPVKVTWKKNIKKVVEGTSITVPLSNKDGSFSQTSKLDFVPKGGD
VYACIVEHMALTQPITKVYAVEDVEPILGPILGPAIFCGVGVTVGVFGVAAGIFLFIRGK

K
>O22B1 ENSONIG00000012441 Scaffold GL831322.1: 945,399-948,614
MDSFYFCFSLFFIILCRADGYEYYTSSRCLFNSTELRDIEYIRSYYYNKTEYVRFSSSVG
KFVGYTEYGVREADQWNKDQGQGSWTRKGQLAAMRAQKETYCKPKIDFWYQSVLTKAVKP
SVRLHSIPPVYLNPAILVCSVYNFYPKEIKVSWLRNGQQVSPDITSTVEFANADWLYQTH
SYMEYTPRSGEKISCMVEHASLKEPLVTDWVPSMSESKKLMIVGAFGLILGLLFFLAGVI
YTRMCSRGQSSTHTK
>O22A2 GL831322.1:1.002.299-1.002.538
LYEMNSVSGCSDTDGENVLILDDEVVWYADFNKQKGVDSLPAFADHPGFEGKYEEAVANQ
QICRQNLKMARLAMKDFPKE
>O22A3 GL831322.1 GL831322.1:1.234.580-1.234.813
LYEMNSVSGCSDTDGEDVLILDDEVVWYADFNKQKGVDSLPAFADHPGFEGKYEEAVANQ
QICRQNLKMARMAMKDFP
>O22A4 GL831322.1:1.279.567-1.279.812
LHETNSISGCSDTDGEDALILDDEVVWYADFNKHTGVDSLPAFADHPHWQEGMYEEAVAN
QQICRQNLKMARSAMKDFPKER
>O22A5 GL831322.1: 1,359,006-1,361,338
LSSVLCVSADGLHETNSISGCSDTDGEDALILDDEVVWYADFNKHTGVDSLPAFADHPHW
QEGMYEEAVANQQICRQNLKVARSAMKDYPKELDAPDSVEIYTIVNVEIGVQ
>O22A6 GL831322.1:1.368.491-1.368.730
LYEMNSVSSCSDTDGEDVLILDDEVVWYADFNKRKGVDSLPAFADHPSFEGKYEEAVANQ
QVCRQNLKVARTAMKDFPKE
>O22B2 GL831322.1:1.369.583-1.369.858
GYEYYTSSRCLFNSTELRDIEYIRSYYYNRIEYVRFSSSVGMFVGYTEYGVKEAEYFNND
PGQLAAMRAQKETYCTPNIDLWYRSVLTKS
>O29B1 ENSONIG00000006116 GL831229.1: 905,654-908,009
SLCEKIRLIATFIRDFVFVTMHTHNFLILPHVLLIFSTADAYFGYGVVRCLFTSKDDVVY
LAQVYLNKMLLGQYNSSLGKYVGYTEKTKEIADNLNKNKGFLEHEKKNKEKCRTNIPLVL
DILSRPVKPSVRLRLVESADSKHPGLFMCSAYNFYPKQIKLTWLRDGKEATSNVTSTDEL
PNGNWLYQIHSYIEISSKPGEKISCVVEHASLTEPKVIDWVPITEAGKNKIAVGAAGLLL
GLVFPIIGVIFYKRKTTDEMIVTTN
>O29B2 GL831229.1:925.993-926.199
LIRCLFTSKDDVVYLAQIYFNKVLLVQYNSTLGKYVGYTEKLKEVADELNKNERFLEQEK
KNEQECRTNIPLVFNVLSRP
>O29B3 GL831229.1:933.642-933.887
EQFYFNKVLECQYNSSVGKVVGYTKNGIEFADKLNGDKDFMKHEVWKSNLCKRNVQMFYD
GL
>O29A1 GL831229: 909.268- 909.463
DPPESILCLAEEVELGVENSLICFVNHFYPPSINFS*TKNGHPVSERVSFSRYVPNKDQI
FHQFSTLTFSLSEGDIYSCTAEH
>O29A2 GL831229.1:920028 - 920170
DPPESMLYPADEVELGVENSLICFVNYFYPPSINVSWTKNGHPVSEGVSLSRYFPNKDQT
FHQFSTLVFTPSEGDIYSCTV
>O29A3 ENSONIG00000006121 GL831229.1: 938,918-940,053
MKHAPLLILILNNFCVFSQIPHEMTYVVGCFVQGLTEVQFEFDGEEFLYADFPKEEIVYT
VPKFIFPDPSHVLVGLSILKDALDNRDWCLIITKIAAMEEKYPPEEKDPPEIVLFSSDKV
ELGVENSLICLVNHFYPPSINVTWTKNGHPVSTGVSLSRYFPNKDQTFHQLSTLTFTPSE
GDFYSCTVEHSALETPQTRIWEAEVTNSDQSLGPVIFCGVGLSLGLLGITVGVFFFVKGH
QRQ
>O31B1 ENSONIG00000007425 GL831231.1: 498,924-502,495
MASSFVCFTLLLISIHTADGFLEYMVERCKFNSTELKDMEYIYSHYYNKIEYIRFSSSVG
KYVGVTEFGENLAKHWNNDTEELNSTRSKIVTYCLNNIDIHDRAILAKSVQPRVRIKSKT
PLSGQHPAMLVCSVYDFFPTKIKVSWLRDGQEVYSDVTSTEVMPNGDWYYQTHSHLEYKP
RSGEKISCVVEHVSLSEPLITDWDPSSDKSTSESTPLTAIGASILTLGLILLLVGYIFYR
WKARGQTRTHTH
>O31B2 ENSONIG00000007445 GL831231.1: 1,171,738-1,174,377
FLEYIVDRCEFNSTELKDMEYIYSHYYNKIEYIRFSSSVEQYVGFTEYGVMLAEILNNNT

ALLNSSRQMITEYCYHNIDVHDQAIIAKSVRPRVRIKSKTPLSGQHPAMLVCSVYDFFPN
KIKVRWLRDEQEVTSDVTSTEVMPNGDWYYQTHSYLEYTPRSGEKISCVVEHVSLSEPLI
TDWESSSDKSTSESTPLTAIGASILTLGLVLLLAGFIFYRWK
>O31B3 ENSONIG00000007449 GL831231.1: 1,232,619-1,236,792
MASSFLRFTFLFISIHIADGFLEYIVDRCEFNSTELKEMEYIYSHYYNKIEYIRFSSSVG
KYVGVTDFGVNLAKRWNNNTALLNSTRSKIATYCLSNIGIHDRAIIAKSVRPSVRIKSKT
PLSGQHPAMLVCSVYNFFPRKIKVSWLRDGQKVTSDVTSTQEMPNGDWYYQTHSHLEYTP
RSGENISCVVEHASLKEPLITDWAHSDPPSDKSTPESTTLIAIGASILTLGLILLLAGFF
LYRWKARARLVVKTA
>O31A1 GL831231.1:463.509-463.778
VMIYTRNEVELRVKNTLICHVTGFYPAPVKVSWTKNGQITTGGSSINTPYPNKDGTFTQI
ARLQFIPQQGDVYSCTVEHLALTKPLTKIY
>O31A2 GL831231.1:505.586-505-849
APSCVMIYTRNEVEFTVKNTLICHVTGFYPAPVKVSWTKNGRITTGGSSINNPYPNKNGT
FTQIARLKFILHCTVEHLALTDPLTKT
>O31A3 ENSONIG00000007446 GL831231.1: 1,175,562-1,178,887
MKMKELLLFLSCVLCVSADSLHTDIHIVGCSDFNGEVMFGLDREELWYADFEKQMDVYPQ
PPFIDPISFGDGAYETAVSNLYTCRQLLQLSRQGMKDYPPLRDAPSGVMIYTRDEVELTV
KNTLICHVTGFYPAPVKVSWTKNEQITTEGSSINTPYPNKDGTFTQIARLKFIPQQGDVY
SCTVEHLALTKPLTKIYNVETPETPETPETPKPSVGPAVFCGLGVIIGLLSAAGGTFFIL
KANKAADQSDLSPLSMVILSYWQ
>O31A4 ENSONIG00000007453 GL831231.1: 1,242,793-1,244,465
MKMKELLLFLSCVVCVSADALHTDIHVIGCSDFNGEVMFGLDREELWYADFEKQMGVSPQ
PSFIDPISFGAGAYKTAVSNLNTCRQNLQITREGIKDFPPLRDAPSGVMIYTRDEMELRV
NNTLICHVTGFYPAPVKVSWTKNGRITTEGSSINTPYPNKNGTFTQIARLKFIPQQGDVY
SCTVEHLALTKPLIKIYNVETPETPEKPKPSVGPAVFCGLGVIIGLLSAAGGTFFILKAN
KSI
>O33A ENSONIG00000009902 GL831433.1: 1,722-3,127
EKYRLNMFLKIIIILSTAACVEAEGRHTLCHIYGCFDSSDTQICAAFDGDEVYYADFKKD
ELIWESKIPISFHLPDAYKYAVFYRSMCKNDIYRWKPDKSVIKKKEPPEFIIYPRDEVIT
GEDNTLICFINHFFPPSINIKWTKNDVEVTVEDPFIKTLSNPDGTAYVLSYLNFVPEKGD
IYSCTVEHEALKEPQTRFWEVETDEISKGPAVFCGLGLSLGLLGVFIGTYFFFKAGHSGP
AG
>O33B1 GL831433 5.113-5.340
STLVCSAYNFYPKQIRMMWMRNGQEVTTDVSYSDVMPDGDWYYQTHSYLEYIPTSGEKIA
CMVEHLGLSEPMFVVW
>O33B2 GL831433.1:16.120-16.443
DWYYQTHSYLEYIPTSGEKIACMVEHLGLSEPMFVVWPSLPVAQRTQIAVGLCGLVIGFV
IAISGFIYYNKKSAAYI
>O39A ENSONIG00000010744 GL831439.1: 301,122-309,267
MKMKELLLFLSCVLCVSAYGLHGDVHINGCSDFDAEDMYGLDGEEVAYADFNNHTEVYIL
PPFAEPMIYPGAYKQAVTELKVCRHNLKALRAVMKDYPSNNHAPSAVMIYTRDEVEFGEE
NTLICHVTGFYPAPVNVSWTKNGQKVTEGSSTNVPYPNKD
>O42A ENSONIG00000018452 GL831842.1: 89,157-98,104
MKMKELLLFLSCVLCVSAYGLHGDVHINGCSDFDAEDMYGLDGEEVAYADFNNHTEVYIL
PPFAEPMIYPGAYKQAVTELKVCRHNLKALSAVMKDYPSNNHAPSAVMIYTRDEVEFGEE
NTLICHVTGFYPAPVNVSWTKNGQKVTEGSSTNVPYPNKNGPFTQISRLQFTAQLGDIYS
CTVQHLALTEPLTKIYKVEASAQPDPGVGPSVFCGVGLTLGLFGVAVGTFFLIKGNECS.
>O49B GL832249.1: 577-1,128
MLVCSVYDFFPKYIKVSWLRDGQEVTSDVTSTEEMADGDWYYQTHSHLEHTPRSGEKISC
KVEHASLEKPLITDWDSSMPESERNKLAIGASGLILGLILSLAGFIYYRRKARG
>O50B GL831650 197.860-198.901
YQNIPSKIKVSWHRDGQEVTSDVTSTEEMEDGDWYYQTHCYLEYTPR
>O57B ENSONIG00000002259 GL831257.1: 1,608,728-1,609,986
LGVIIAILLTINYNPCVTVSSDEDYFQSRACCTFKGPHFEDMEYTRIISLNKIAVLEYNH
TRGSWIGFTPYTIEIAKFWNLNPFGTSEAKALCSTNLGYIQILSNVTDIPTIRVKSVKQH
SGGHPAMLVCSAFNFYPKQIRMIWLRNQKEVTTGVSYSEVMPDGDWYYQIHSYLEYTPTH

GEKITCVVEHLSVSEPILAIWGVLDPTLPVAARNKIIVGICGLLLGFVIVSSGFIYHKKK
SSGRVLIPV
>O57A ENSONIG00000002263 GL831257.1: 1,611,410-1,612,511
LYFFSQEHFASVHKIFLGSIKLCYTFGCFKSSDTQVCLTMDGDEVFYANFKNGSLIWDSK
IPTAFHVPYAYSQALYSQRQCRDFVHDDWKQHKTIMNTKAPPEIIIYPRDEVMKEEDNSL
ICLIKKFFPPSIKIKWTKNDIEVKVEDPFIKCLPNPDGTFYVFSHLDFVPEDGDIYSCSV
EHEALTEPLTKLWEVQTDEPSNGPDVFCGLGLSLGVIGVVAGAFLYVKGRQYQSTQNS
>O77B GL831677 200.297-200.562
VKLHSTTPLSSHHPAMLVCSVFDFFPSKIKVSWHRDGQEVTSDVTSTEEMEDGDWYYQTH
CYLEYTPR
>O79B1 GL831379.1:603.285-604.145
DGYELYNSDRCLFNSTELQDVEFIRSCYYNKIEFVRFSSDVGKFVGYTDFGMKNAEMWNK
DQGYLAAMRAQKETYCKHNIDLWYTNVLSKSVTPSVRLHSTMAPGCHHPAMLVCSVYDFY
PKLIKVSWLRNGQEITSDITSTEEFANGDWLYQVHSHLEY
>O79B2 ENSONIG00000009129 GL831379.1: 618,993-627,882
MASKFLCFTLLFINICTADGYECYSSTRCLFNSTELKDIEFITSYYYNKIEYVRFSSDVG
KFVGYTEYGVKNAEYWNKNQALLAQMRAQKETYCQPNIGIWYADVLSKSVTPSVRLHSTM
APGCHHPAMLVCSVYDFYPKLIKVSWLRNGQEITSDITSTEEFANGDWLYQVHSHLEYMP
RSGEKISCVVEHASLEKPLIINWVPPMPETERLIIVGVVGLILGLILFLAGVIYRRRSGV
CAPGLMLVPSS
>O79A1 ENSONIG00000009128 GL831379.1: 610,349-612,847
MKVWVLLLILSLVPCVSADDAHGSNFIIGCSDADGENVLILDDEAVWYADFNKQTGVNAL
PAFVDPISFPGFYEEAVADQQICKQNLMVTRKAMKDFPKELDPPSSLIIYTVLNVELGVQ
NTLICHVTGFYPAPVNVTWTKNEQKVTEGTNTSVPFPNKDGSFKQTSRLQFIPKQGDVYS
CTVEHVALIQPMTKFYGEKIQLSLFYIIQFHNNSDLKLLYILR
>O79A2 GL831379.1: 614,307-615.228
PPSSLIIYTVLNVELGVQNFLICHVTGFYPAPVNVTWTKNEQKVTEGTNTSVPFPNKDGS
FKQTSRLDFIPKQGDVYSCTVEHVALIQPMTKFYAVVNSDLILGPAMFCGVGLTVGVLGV
AAGIFLLIR
>O79A3 ENSONIG00000009132 GL831379.1: 630,011-633,534
MKMKLWVLLLVLSSVLCVSADALHGDNWISGCSDTDGEAVLILDDEAVWYADFNKQEGIY
PLPPFVDPITFPGQYEQAVANQQICRANLKVTRQAMKDVPKELDPPSSLIIYTVLNVELG
VPNILICHVTGFYPAPVNVTWTKNEQVTEGTSTSVPFPNKDGSFKQTSRLNFIPKQGDVY
SCTVEHVALTQPMTKFYDLVYSDLILGPAMFCGVGLTVGVLGVAAGIFLLIRATKCS
>O79A4 GL831379.1:677.714-677.994
DPPSCLVIYTLLHVELGVQNTLICHVTGFYPAPVNITWNKNEQKVTEGTSTSILFPNKDG
SFKQTSRLDLIPKKRDVYSCTVEHVGLTQPTTKFDG
>O80B ENSONIG00000005905 GL832180.1: 12,190-16,972
MASSFLCLLFISLSTVDGFMTYRLSRCDFNSTELKDIQFTDSYYYNMIEIIRFDSNVGKF
VGFTDFGVKTAETWNNIPARLASMRAQKGTYCKSNIDVRYHKLTSKSAQPTVKLHSTTPL
SSHHPAMLVCSVYDFFPSKIKVSWHRDGQEVTSDVTSTEEMEDGDWYYQTHCYLEYTPRS
GEKISCVVEHASLEKPLITNWDPSSSSFMPKIERYKLAIGASGLILCLILSLAGFIYYRQ
RARGRILVPTN
>O97B1 ENSONIG00000004432 - GL831197.1: 3,529,581-3,531,898
EDGYFMFADFCCVMHSRDPKTVEYLIDWYFNKEFTMQYNSTVGKWTGLTPAGLITASQFN
EDKFDVDQRKAEKQLTCVNNVDAVFNGVQENIAAPSMTLQEGDHTSTHDTILVCSVYDFY
PKHILVTWYLNGQEV
>O97B2 ENSONIG00000004434 GL831197.1: 3,534,451-3,536,032
RCQFLINLFFSFLDCSINGDGYFMFADFWCAVHSRDREEVEYLIDWYFNKEFTMQYNSTV
GKWTGLTPAGLITASRFNEDSADILQRKVEKQLICVNNVDAVFNITVENMAAPSMTLQEG
DHTSSHDTILVCSVYDFYPKHILVTWYLNGQEVTERVTCTVEHVSLKEPKSSYWDSSLKQ
SDRSFLTGGLCALLLGALILSLGLIYYRRKTSDPTFSN
>O97A ENSONIG00000004441 GL831197.1: 3,645,982-3,653,566
MALSVIALILSAGAACGYIQTQSHNFHYIYGCYESDDVRVDAVVDDDVVAYADFNKEEVV
WVLPHLPQPAKDFKKMAYGVAKASTAHCHSVLGKAKNADPGAPLRQDPPDIYIYTRYEGE
ASVMNTLFCVANHFYPPTINFTWTKNGVELTEGVANLRYRHNRDGTFHMISTLLFTPQPG
DHYICTVEHQAAKVPVSKSWELKENKMSRVSPEAVFFGVSVVLCLIGFGTGVFFFIKQPN

ECLQI
>O745B ENSONIG00000003902 GL831745.1: 25,751-37,458
MASSFLCLLFISLSTADGFKMYVLSRCDFNSTELKDMKYIRSYYYNKIEVTRFDSDVRKF
VGYTEYGVKKAETWNNDQAHMAAFRVEKERYCYTNIGIWYRNILSKSAKPYVKLHSTTPD
YSHHPAMLVCSVYDFFPSKIKVSWLRDGQEVTSDVTSTEEMADGDWYYQTHSHLEYTPRS
GEKISCKVEHASLEKPLITDWDSSMPESERNKLAIGASGLILGLILSLAGFIYYRRKAQG
RILVPTN
>O745A ENSONIG00000003904 GL831745.1: 44,977-46,516
MKMKELLLFLSCVLCVSADPLHSDIAVGGCSESDEENMYGLDGEELAYTDFNKKEEIYPL
PPFIDPITYGEDAYESSVAFQKTCRKNMKQFGEGMKDFPPEQDVPSAVMIYTRDEVEFGE
KNTLICHVTGFYPAPVNVSWTKNQQKVTQGSSINVPYLNKDGTFTQISRLQFTPQLGDIY
SCAVQHLALTQPLTKIYEVEASARSDPGVGPSVFCGVGLTLGLLGVAAGTFFLVKGNECS
>O779B GL831779 70180
YDFYPSEIKVSWHTDAQEVTSDITSTEEMTDGD*YYQIHSHLEYTPR
>O845A ENSONIG00000019001 GL831845.1: 36,104-40,196
MKMKVWVLLLVLSSVICVSADGLYEMNSVSGCSDTDGEDVLILDDEVVWYADFNKQKGVD
SLPAFADHPSFEGKYEEAVANQQICRQNLKMARTAMKDFPKQHEAPSSVAIYTTLNVEIG
VQNTLICHVTGFYPAPVNVTWNKNTKKVVEGTSITVPLSNKDGTFTQTSKLDFVPKGGDV
YACIVEHVALTQPLTKVYAVDNAEPILGPAIFCGVGLTVGVLGVAAGIYLFIRGTRCD
>O845B ENSONIG00000019004 GL831845.1: 41,863-44,745
MDSSFLCFSLFFIILCRADGYEYYTSSRCLFNSTELRDIEYIRSYYYNKTEYVRFSSSVG
KFVGYTEYGVREADQWNKDQGQGSWTRKGQLAAMRAQKETYCKPKIDFWYQSVLTKAVKP
SVRLHSIPPVYLNPAILVCSVYNFYPKEIKVSWLRNGQQVSPDITSTVEFANADWLYQTH
SYMEYTPRSGEKISCMVEHASLKEPLVTDWVPSMSESKKLMIVGAFGLILGLLFFLAGVI
YIRMCSRGQSSTHTK
>OA35A AERX01073735.2:6-251
VEIGVQNTLICHVTGFYPAPVNVTWNKNTKKVVEGTSITVPLSNKDGTFSQTSKLDFVPK
GGDVYVCIVEHVSLTAPLTKIY
>OA35B AERX01073735.2:3.469-3.735
GYEYYTSSRCLFNSTELRDIEYIRSYYYNRIEYVRFSSSLGMFVGYTEYGVKEADQWNND
PGQLAAMRAQKEIYCTPNIDLWYRSVLTK
>OA78B ENSONIG00000008338 AERX01073278.2: 2,515-4,762
MDSSFLCFSLFFIILCRADGYEYYTSSRCLFNSSELRDIEYVRSYYYNRIEYVRFSSSVG
MFVGYTEYGVREADQWNKDQGQLAVMRAQKETYCTPNIDLWYRSVLTKYVKPFVRLHWTK
PPVDHHPAILACSIYDFYPKYIKVTWLRNGQRVTSNTTTTEELPDADWFYQTHSYLEYTP
K
>OA78A ENSONIG00000008336 AERX01073278.2: 144-1,540
MCLCTDAPDSVEIYTIVNVEIGVQNTLICHVTGFYPAPVNVTWNKNTKKVVEGTSITVPL
SNKDGTFSQTSKLDFVPKGGDVYVCIVEHVSLTAPLTKIYEVDDVEPILGPAIFCGVGLT
VGVLGVAAGIYLFIRGRK

Tilapia translated Genbank cDNA report sequences:

>GR696948 cNOnST1-012_C05.ab1_c Tilapia adult stomach library Oreochromis niloticus cDNA 5 similar to H-2 class II histocompatibility antigen, A-R alpha chain in Mus musculus (P14436), mRNA sequence O845A match
MKMKVWVLLLVLSSVICVSADGLYEMNSVSGCSDTDGEDVLILDDGVVWYADFNKQKGVD
SLPAFADHPSFEGKYEEAVANQLICRQNLKMARTAMKDFPKRHEAPSSVAIYTTLNVEIG
VQNTLICHVTGFYPAPVNVTWNKNTKKVVEGTSITVPLSNKDGTFSQTSKLDFVPKGGDV
YACIAEHVALTQPLTKVYAVDNVEPILGPA
>GR667686 cNOnOE1019_D18.ab1_c Tilapia adult olfactory epithelium library Oreochromis niloticus cDNA 5 similar to H-2 class II histocompatibility antigen, A-B alpha chain in Mus musculus (P14434), mRNA sequence O29A3 match
MMSHHTTDMKHAPLLILILNNFCVFSQIPHEMTYVVGCFVQGLTEVQFEFDGEEFLYADF
PKEEIVYTVPKFIFPDPSHVLVGLSILKDALDNRDWCLIITKIAAMEEKYPPEEKDPPEI
VLFSSDKVELGVENSLICLVNHFYPPSINVTWTKNGHPVSTGVSLSRYFPNKDQTFHQLS

TLTFTPSEGDFYSCTVEHSALETPQTRIWEAEVTNSDQ
>GR671091 IIA cNOnOE1_014_G17.ab1_c Tilapia adult olfactory epithelium library Oreochromis niloticus cDNA 5 similar to HLA class II histocompatibility antigen, DP alpha chain in Homo sapiens (P20036), mRNA sequence O79A3 match
MKMKLWVLLLVLSSVLCVSADALHGDNWISGCSDTDGEAVLILDDEAVWYADFNKQEGIY
PLPPFVDPTTFPGQYGQAVADQQVCRANLKVTRQAMKDVPKELDPPSSLIIYTVLNVELG
VPNILICHVTGFYPAPVNVTWTKNEQKVTEGTSISVPFPNKDGSFKQTSRLNFIPKQGDV
YSCTVEHVALTQPMTKFYAVENSDLILGPAM
>GR702301.1_1 cNOnTE2014_G22.ab1_c Tilapia adult testis library Oreochromis niloticus cDNA 5' similar to RT1 class II histocompatibility antigen, A beta chain in Rattus norvegicus (P06341), mRNA sequence O80B match
GGKREARGETRSGRRSACQVN*TSARPTVKLHSTTPLSSHHPAMLVCSVYDFFPSKIKVS
WHRDGQEVTSDVTSTEEMEDGDWYYQTHCYLEYTPRSGEKISCVVEHASLEKPLITNWIP
PHPHSCLRQRGTSLPSAPQD
>GR627548 cNOn16-40D022_P07.ab1_c Tilapia juveniles 16-40 days post-fertilization library Oreochromis niloticus cDNA 5 similar to HLA class II histocompatibility antigen, DQ(W1.1) beta chain in Homo sapiens (P01919), mRNA sequence O57B match
XGGNMRIQNFYFYMFLSLFGLVSSDEDYFQSRACCTFKGPHFEDMEYTRIISLNKIAVLE
YNHTRGSWIGFTPYTIEIAKFWNLNPFGTSEAKALCSTNLGYIQILSNVTDIPTIRVKSV
KQHSGGHPAMLVCSAFNFYPKQIRMIWLRNQKEVTTGVSYSEVMPDGDWYYQIHSYLEYT
PTHGEKITCVVEHLSVSEPILAIWDPTLPVAARNKIIVGICGLLLGFV
>FF281534 AQAH-SP-0931 Spleen of Oreochromis niloticus infected with Streptococcus agalactiae Oreochromis niloticus cDNA clone SP1179 5 similar to MHC class II beta chain, mRNA sequence O79B2 match
MASKFLCFTLLFINICTADGYELYSSTRCLFNSTELKDIEFIRSYYYNRMELIRFDSDVG
KYVGYTEFGVKNAEMWNNDPGQLAAMRAQKETYCQYNVGIWYADVLSKSVTPSVRLHSTM
APGCHHPAMLVCSVYDFYPKWIKVSWVRNGQEITSDITSTEEFANGDWLYQVHSHLEYMP
RSGEKISCVVEHASLEKPLIINWVPPMPETERLIIVGAVGLILGLILFLAGVIYRRKSGD
TSLACRFSEKQAEDWDKDAAALSLWKPQKEMFFKPGSELCFTNVLLKSV
>GR679428.1 cNOnOV1024_K05.ab1_c Tilapia adult ovary library Oreochromis niloticus cDNA 5' similar to HLA class II histocompatibility antigen, DQ(6) alpha chain in Homo sapiens (P01906), mRNA sequence O97A match disrupted orf
MALSVIALILSAGAACGYIQTQSHNFHYIYGCYESDDVRVDAVVDDDVVAYADFNKEEVV
WVLPHLPQPAKDFKKMAYGVAKASTAHCHSVLGKAKKC*

Spotted gar (*Lepisosteus oculatus*) MHC class II sequences:

Spotted gar PreEnsembl sequences:

>501A1 MHCIIA   JH591501: 64.610-66.685
MSVGWKCLGLLVVMQGAATVAVEHTYEVAYFCQSENRKGSRNEVFDDDEMFHIDPDRKVD
EPRLPEFEKWAWNDSSLIQGALANLGICENHLKGVMKAIPDEPAVKVPPKPAVFPEEPVE
LGWPNTLICALNDFTPPTAQLRWLKNGQPVTSDVSSTDYIPLSSNKFAMFSYLSFTPQEG
DIYTCHVEHTALSEPVSVFWDAEVPTDSDASETAFCAIGLALGILGVVLGTVFLIKAIKF
HS
>501B1 MHCIIB   JH591501: 70.640-74.060
MRLVLLCSALVLCLTLSGADDFVYFFRSECHFSASDLRDLVYYVRSMTFNGKERVRFNSTV
GKYVGFDTWGEKQANYWNGQKDYIARLSADKDRFCKYNVQLMASGMQDRKIKPEVKIYPA
KTASQGHTHMLVCHAHGFYPRQIAVSWLRNGQPVSSDLTSMSFASDGDWTYQTLLYLEFT
PQGGETFECAVDHVALDGTLKLRWDPAAREAKRNKVIMGASGLVLGLVFAVIGVVYWKRK
TKGHHRLVSSERLVSCVGQVFSGGLVSCPGCALPCAQCLLG
>501A2 MHCIIA JH591501: 82.102-84.669
TSHIDINVIGCKSDDPVAQDEAQLDGDEMFYADFDKKEMILTLPAFADSFGVDPGWVQGA

IANRQICINNLEVAIKAENNPPENTDAPVNTIYPRDEVELGKPNTLICLANNFFPPPVKV
RWTKNDVDVSEQATLSRYYPNSDATFYQFSTLSFTPQLGDVYSCSVEHKALPEPKTRIWG
EERSALTSDAN
>501B2 MHCIIB   JH591501: 95.883-99.847
MLMCSALGFYPKQIKVSWLRDGQTVTSDVTSTEELADGDWYYQIHSHLEYTPRAGESIVC
RVEHSSFATPKELTWDPSMPEADRNKIIIGASGLVLGLVIAAAGGIYYKKKSG
>501A3 MHCIIA   JH591501: 120.683-125.781
MHGIAVGSIACREISEWKPGRPCQGGRDRAALGWIFRQDGDAPTAMVYPRDSVELGKPNT
LICSVTDFHPMGIGVTWTHNDRPVTEGVTQTTPLSGRDFSFKVFSFLPFTPRLGDVYSCQ
VQHSALPEPLARLW
>615A MHCIIA JH591615: 5.333-9.156
MLCAFLTAVLLGASGVLSQAQIRHLDRLLTFCVSNSTEAELEKEHDEDEIFYVDLETKQS
VQRLPEFAEKWHVGPEWPPFAQQEVETCRLYLAAKGEGFPEEKLDPPISRLYSENEVELG
VPNALICFITDFHPAPVKVSWTRNTEPVTQGFNVTQYYSNKDYSLRLFSYLSFTPQAGDV
YSCSVEHRALQEPLTRLWEVEVQSDSEAAETAVCGVGLTLGLLGVAAGTFFLIKGNKCN
>615B MHCIIB JH591615: 11.335-16.190
MDSPLHRLAVAVLVLGCTGSRRSVDGNMYQFVHDCEYNDHLEDFLYTRRDIFNKIEILRY
DSNIQTFVGYTPLGIKYAERFNQDKEYLAGLKDDLDNYCKHNAGVYKSTMTDRKVPPSVK
VSATKLLSSKHPTMLVCHVTGFYPQRITVTWLRDGLEIKTDVTSTDLLANGDWTYQVHSH
LELTPRAGETVACRVEHSSLERPLEVTWDPSMPESKKNKIVIGVSGLILGLIITAAGVIY
YKKKSSGRILVPSD

For spotted gar MHC class II we could not find cDNA reports in GenBank.
