# Supplementary material for: Comprehensive analysis of MHC class II genes in teleost fish genomes reveals dispensability of the peptide-loading DM system in a large part of vertebrates
Source: BMC Evol Biol. 2013 Nov 26;13:260. doi: 10.1186/1471-2148-13-260 (PMC4219347; doi:10.1186/1471-2148-13-260)
Supplement: Additional file 8: Text S2 — Atlantic salmon (Salmo salar) MHC class II sequences, transcripts and genomic regions. [file 1471-2148-13-260-S8.pdf]

Supplementary Text 2 (Text S2): Atlantic salmon (*Salmo salar*) MHC class II sequences, transcripts and genomic regions

Table of Contents		**Page**
Table TS2-1	Linkage group (LG) numbers, chromosome numbers, and GenBank contig sequence accession numbers of Atlantic salmon MHC class II loci	**2**
Text S2-1	Atlantic salmon MHC class II protein sequences used in this study	**3**
Text S2-2*A*	Expression pattern of Atlantic salmon MHC class II gene transcripts	**5**
Table TS2-2*A*	PKM values for Atlantic salmon MHC class II transcripts in various tissues	**5**
Table TS2-2*B*	Tissue distribution of human MHC class II EST reads	**5**
Text S2-2*B*	Atlantic salmon *DCB*, *DEA* and *DEB* tissue distribution analysis by RT-PCR experiments	**6**
Text S2-3	Investigation of potential polymorphism of Atlantic salmon *DCB*, *DEA* and *DEB*	**7**
Text S2-3*A*	Sequence alignment of Atlantic salmon *DCB* alleles	**8**
Text S2-3*B*	Sequence alignment of Atlantic salmon *DEA* alleles	**9**
Text S2-3*C*	Sequence alignment of Atlantic salmon *DEB* alleles	**12**
Table TS2-4	Genes situated in Atlantic salmon *DEA*/*DEB* (Ssal_scf_10) and *DFA*ψ/*DFB*ψ (Ssal_scf_137) genomic regions	**14**
Text S2-4*A*	Ssa1_scf_10: 411 kb scaffold sequence including *DEA*/*DEB* loci	**15**
Text S2-4*B*	Ssa1_scf_137: 428 kb scaffold sequence including *DFA*ψ/*DFB*ψ loci	**89**

Table TS2-1. Linkage group (LG) numbers, chromosome numbers, and GenBank contig sequence accession numbers of Atlantic salmon MHC class II loci

**Region**	**Chromosome**	**Genbank ID_ Salmon genome contig**	**MHCII genes**
LG6[1]	Chromosome 12	AGKD01360529.1_Contig_360694	*DAA*
	-"-	AGKD01083185.1_Contig_083194	*DAA*, *DAB*
	-"-	AGKD01084552.1_Contig_084561	*DAB*
LG5[2]	Chromosome 13	AGKD01021571.1_Contig_021574	*DBA*
	-"-	AGKD01084147.1_Contig_084156	*DBB*
DCA/*DCB*[2]	n.d.	AGKD01037528.1_Contig_037532	*DCA*, *DCB*
	n.d.	AGKD01028197.1_Contig_028200	*DCB*
DDA[2]	n.d.	AGKD01006140.1_Contig_006141	*DDA*
LG1[3]	Chromosome 2	Ssa1_scf_10<br>AGKD01038092.1_Contig_038096	*DEA*, *DEB*
LG12[3]	Chromosome 5	Ssa1_scf_137<br>AGKD01017119.1_Contig_017120	*DFAψ, DFBψ*

[1]First described by Moen et al. [(2008) A linkage map of the Atlantic salmon (*Salmo salar*) based on EST-derived SNP markers.*BMC Genomics* 9:223]
[2]First described by Harstad et al. [(2008) Multiple expressed MHC class II loci in salmonids; details of one non-classical region in Atlantic salmon (Salmo salar). *BMC Genomics* 9:193].
[3]A new Arachne (v3.9) assembly of 15,506,038 Sanger sequences (www.ncbi.nlm.nih.gov/Traces; as of June 2012; project_name: International Cooperation to Sequence the Atlantic Salmon Genome [Davidson WS, Koop BF, Jones SJM, Iturra P, Vidal R, Maass A, Inge Jonassen I, Lien S, Omholt S (2010) Sequencing the genome of the Atlantic salmon (*Salmo salar*) *Genome Biol* 11(9):403] was constructed (BFK, unpublished) and scaffolds Ssa1_scf_10 and Ssa1_scf_137 were identified as containing *DEA/DEB* and *DFAψ/DFBψ* genes. Data from these scaffolds were matched contigs from a previous assembly (GenBank accession AGKD01000000) in which Contigs 360694-006141 were identified as corresponding to MHC class II genes *DAA*, *DAB*, *DBA*, *DBB*, *DCA*, *DCB* and *DDA* while contigs 038096 (AGKD01038092.1), 007503 (AGKD01007502.1), 085515 (AGKD01085506.1), 082232 (AGKD01082223.1), 163744 (AGKD01163719.1), 017120 (AGKD01017119.1), 057643 (AGKD01057639.1) and 000853 (AGKD01000853.1) were found to contain *DEA*/*DEB* and *DFAψ/DFBψ* genes. This assembly represents an intermediate assembly of an ongoing project of which AGKD01000000 is the first initial public assembly. As assembly AGKD01000000 is an initial assembly of an ongoing project, there are some discrepancies that remain to be resolved. For example there are two separate contigs with *DAA*. Our current improved assemblies identify only one contig containing *DAA* and *DAB*. Scaffolds were mapped to the salmon genome (chromosomes and linkage groups) by blasting the sequences against a list of SNP and extended source sequences [Lien et al. (2011) A dense SNP-based linkage map for Atlantic salmon (*Salmo salar*) reveals extended chromosome homeologies and striking differences in sex-specific recombination patterns. *BMC Genomics* 12:615] and identifying 100% matches.

Text S2-1. Salmon MHC class II protein sequences used in this study

```
>Sasa-DAA*0101 (GenBank L77086_1) MHC class II alpha
MKTSVIVLILCWQVYAEHKVLHIDLVITGCSDSDGLDMYGLDGEEMWYADFNKQEGVVAL
PPFADPFTFPGFYEQAVGNQGVCKGNLAKCIKAYKNPEEKIDPPHSSIYPRDDVDLGVEN
TLICHVSGFFPAPVRVRWTRNNQNLTEGVRLSTPYPNADFTLNQFSSLPFTPEEGDIYGC
TVEHKGLAEPLTRIWEPEVIQPSVGPDVFCGVGLTLGLLGVAAGTFFLIKGNQCN
>Sasa-DAB*0101 (GenBank CAA49726) MHC class II beta
MSMSIFCVSLTLVLSIFSGTDGYFEQVVRQCRYSSKDLQGIEFIDSYVFNKAEYIRFNST
VGKFVGYTELGVKNAEAWNSDAAVLAVERGELERYCKHNADLHYSTILDKTVEPHVRLSS
VAPPSGRHPAMLMCSAYDFYPKPIRVTWLRDGREVKSDVTSTEELANGDWYYQIHSHLEY
TPRSGEKISCMVEHISLTEPMVYHWDPSLPEAERNKIAIGASGLVLGAILALAGLIYYKK
KSSGVL
>Sasa-DBA (GenBank EG757342, EU008541) MHC class II alpha
MSFEMNYSVIILILTGAVCTSAEIHHEIHFIFGCFESSDPAVGLEIDGDEVFYGDFNKNS
NTCLIADVVFTLPKFISITPEDKERACEYATISRVWCKDCIAWGKQSEPKIPKIKDAPES
TIYPRDEVELGVENTLICFVNDFFPPPVKVYWTKNEMEVTEGLSLSRYYPNKDGTFHQFS
SLSFTPQKEDVYICAVAHTALKEPKTREYKVSGSSAGPGPAVFCGVGLTLGLLGVATGIF
LIYKGKRATESQE
>Sasa-DBB (GenBank DY726096, EU008541) MHC class II beta
MYVLNCFSIHLLLLFSSLSEVVDSSDEDFAHDDAWCRFSSRDLHNMEYILEHHFNKILVA
QYNSTTERWTGYTAWGVISAEKWNEDPDEIPRRRTDMGVLCKPYANRIYNATEMFMVEPN
VTLRLEGPSSDSSLVCSVHFFYPKHIRVTWLRNGEEVTSDVTSTDVLANGLWSYQIQSYL
KYTPTTGERITCMVEHISQTEPKLYYWDPSLPKSEKNKIVIGVCGLLLGVVFVVAGLIYW
AKSTGRLLGLIGERDYGTCD
>Sasa-DCA (GenBank DW549478,DY704572) MHC class II alpha
MNLSVAIVVLTAVVCTSAEIPHETVYVLGCLEKTKVKAEAELQLDGEEVVYADFQSGQEV
WTLPEFLGPFSSSTVRNFYKNAVKGRRLCRDALALWIFEEKSPPEVKDAPESTIYPRAEE
ELGVENTLICFANHFYPPPVKVNWTKNGLEVTEGTSLSRYYPNEDGTFHQFSSLSFTPQE
GDVYGCTVKHTALEDPKTRFWEYEVREVSGSSAGPAVFCGVGLTLGLLGVATGTFLYVKG
QQFN
>Sasa-DCB (GenBank KC316031) MHC class II beta
(the N-terminus indicated in small font derives from genomic sequence at
GenBank AGKD01037528 and was confirmed by transcriptome reads)
msvlnlssihLLLLFSSLSGVDGYFGHFEMRCRFSSEDPRDIEYLLQVYGNKKLLGQY
NSTTEKCTVYTQWMKNFTETACKGPAFLSERREEMNKYCSSNVPVVYGYLLDKAVEPY
IRLRSVESFSTRHPAMLVCSAYDFYPKPIRVTWLRDGQEVTSNVTSTEELVNGDWTYQ
IHSHLEYTPTPGERITCMVEHFSLTEPKLYDWDPSLPGPERNKMVIGACGLLLGVVFI
AAGLIYYRKKSTEGRVLEPTMALPESYGTI
>Sasa-DDA AGKD01006140.1_Contig_006141 (GenBank DW557800) MHC class II
alpha
MMALVFMILLFMLSSTQGQEQHVFNVINTRSETEEFNMTIVVDDNEYLHVYLNNKEGVVT
LPEWGNYVDCPICVRVAESRRADLNNDIKLFNLETPEAKVPPEIKLYAKDEVKLGINNSL
VCFVNNFFPPPVQVKWTKNDENVPKGVKVGQYATNSDYTFYRFSTLTFEPQEGDIYTCIV
DHTALDEPLTRTWEFEVPPRASVGPAVFCGLGLTLGLLGVATGTFFLVKGTQCQ
>Sasa-DEA (GenBank KC316032) MHC class II alpha
MGCRVFLAFILGVCLLSHCQSKHLLRFLTFCQKNVPSDEEYDVEFDGDELFYVDSITY
QVERRLSEFAQQWTPDPGLPHEVYVSLGTCQYNIPRCIVGEKSPPEAIEVPTSHIYSQ
REVELGVPNTLICRVSDFHPTPVDVTWTRNEQPVAERTIIQTQYYSNEDFSFRIFSYL
SITPQEGDIYSCSVGHVSLQEPLTRIWEVEVHTDHQTVETAVCVGGVTLGVVGVATGV
WFIKKAKRSGWALRT
>Sasa-DEB (GenBank KC316036) MHC class II beta
(the N-terminus indicated in small font derives from genomic
sequence at GenBank AGKD01038092 and was confirmed by
transcriptome reads)
malcwVYWMAGLSVIQTWATPAGGYQFQGIVDCEYDDTIDNMIYFVKNIFDQKLTTIY
DSRVQKYVGFGEFGIRNADRYNSQAWKMAIRKAEVETICRYSAIFFKLSTLERIVPPI
VKVRLTKPSRYGELSMLECSVLGFYPQEVRVSWLRDGLETTTAVTSTDTLANGDWSYQ
LHSYLEFRPQRGESVSCMVEHPSLDEPLEVVWDTSGLDAKWFKMAIGVCSLFIGVAMA
IGGGVYYWWKNRSGFRRVNR
```

>Sasa-DFAψ (GenBank AGKD01017119) MHC class II alpha pseudogene (combination of encoded fragments)
MGCRVFLALILGVCLLSHCQS-
KHLLRFLTFCQKNITH-
KEYDVEFEDELFYVDPMTYRVERRLSAFAQQWTRDPGLPHEVNVSLGTCQYNIPCCIVGEN-
EAPTSHIYSQREVELGDPNTLICRVSDFHPTSVDVTWTRNEQPVGEGTVSLTQYYSNENFSFRIFSYLSITPQEG
DIYSCSVGHVSLQEPLTRIW-
EVEVHTDHQTVETAVCVGGVTLGVVGVAT-
KKAKRSGWALRT

>Sasa-DFBψ (GenBank AGKD01017119) MHC class II beta pseudogene (combination of encoded fragments)
AGLPVIHTWETPA-
GGYQFQGIVDCEYDTIDNMIYFVKNIFNQKLTIYGARVQKYMGFGEYGIRNADHYNS
QAWKMAIRKA*VETICPYSARFFKLSTLERI-
VPPTVKVCLTKTSRYGELSMLE-
DTSGLHTKWFKMAIGVCSLFIGVAMAIGGVYYWWKNR

Text S2-2*A*. Expression pattern of Atlantic salmon MHC class II gene transcripts

Expression of Atlantic salmon class II genes was estimated by transcriptome analysis and by gene-specific RT-PCR analysis. The transcriptome data, using tissues of a one-year old salmon (Table TS2-2*A*), agree fully with our previous RT-PCR analyses on *DAA*, *DAB*, *DBA*, *DBB*, *DCA* and *DDA* [Harstad et al. (2008) *BMC Genomics* 9:193-209] and our present RT-PCR analyses on *DCB*, *DEA* and *DEB* (Text S2-2*B*). They show that the classical *DAA* and *DAB* genes are expressed abundantly and ubiquitously, whereas the nonclassical genes are expressed at much lower levels and in some cases, especially for *DCA*, *DCB* and *DEA*, in a more tissue-restricted manner. In comparison, the tissue distribution of human nonclassical *DM* and *DO* genes is relatively similar to that of human classical class II genes and the relative expression levels of *DM* and *DO* in comparison with classical genes tend to be higher than the low nonclassical/classical expression ratios found in salmon (compare Tables TS2-2*A* and -*B*).

Table TS2-2*A*. RPKM values for Atlantic salmon MHC class II transcripts in various tissues

Tissues\ Gene	DAA	DAB	DBA	DBB	DCA	DCB	DDA	DEA	DEB	Total # reads
Brain	5.99	3.54	0	0.04	0	0	0.29	0	0	58939250
Eye	9.03	6.01	0	0	0.02	0	0.63	0	0.02	60380888
Gills	394.69	234.79	0.52	0.04	0.36	0	6.50	0.45	0.05	59793962
Gut	259.73	221.51	0.16	0.11	8.10	19.60	1.54	0	0.02	59806348
Head kidney	238.07	155.41	1.67	0.99	0.23	0	8.28	0	0.07	59084708
Kidney	104.31	61.50	0.89	0.25	0.29	0.04	4.25	0	0.05	61054936
Heart	15.61	8.36	0.14	2.61	0.05	0	0.76	0	0	58163180
Liver	15.11	9.22	0.25	2.28	0.02	0	0.43	0	0	58784272
Muscle	20.56	12.18	0.09	0.04	0	0	0.60	0	0	61426586
Nose	130.25	72.91	0.16	0.09	0.30	0	1.76	0.02	0.05	59545012
Pyloric caecum	41.36	26.77	0.18	0.12	7.75	12.84	1.11	0.15	0	61602874
Spleen	677.01	291.31	4.40	2.12	0.07	0.04	27.90	0.02	0.23	60203316
Contig length (bp)	708	738	740	783	735	789	705	744	708	

RPKM = Reads Per Kilobase per Million mapped reads. Mapping reads back to our unpublished Atlantic salmon reference transcriptome was done with CLC v 5.1.5 software. Reads were mapped with high stringency i.e. greater than 95% identity over more than 90% of the total length of the query read. The transciptome was based on analysis of tissues of a single one-year old individual and contained > 70,000 non-redundant contigs.

Table TS2-2*B*. Tissue distribution of human MHC class II EST reads

	DMA	DMB	DOA	DOB	DPA1	DPB1	DQA1	DQB1	DRA	DRB1
Blood	89	16	40	24	49	106	237	204	343	220
Brain	19	10	8	0	68	33	21	25	45	84
Intestine	43	25	4	12	81	64	103	81	142	301
Kidney	56	47	75	9	90	52	66	52	118	185
Liver	14	9	4	0	19	19	38	9	112	38
Lung	247	53	62	8	274	218	215	197	893	809
lymph node	178	156	200	133	468	791	601	390	1649	835
Muscle	47	9	0	9	9	75	9	28	75	141
Pharynx	834	761	0	49	1154	245	736	245	3167	687
Skin	9	9	14	0	23	23	18	14	251	460
Spleen	131	149	74	74	149	374	149	449	505	618
Thymus	75	37	25	25	62	188	100	62	100	313
Tonsil	646	176	58	352	2173	2350	1233	1527	2585	3466
Uterus	129	103	8	0	176	236	103	103	715	349

EST numbers according to "EST Profile Viewer" at Unigene, NCBI http://www.ncbi.nlm.nih.gov/unigene/
Numbers indicate gene-specific ESTs per million ESTs.

Text S2-2*B*. Atlantic salmon *DCB*, *DEA* and *DEB* tissue distribution analysis by RT-PCR experiments

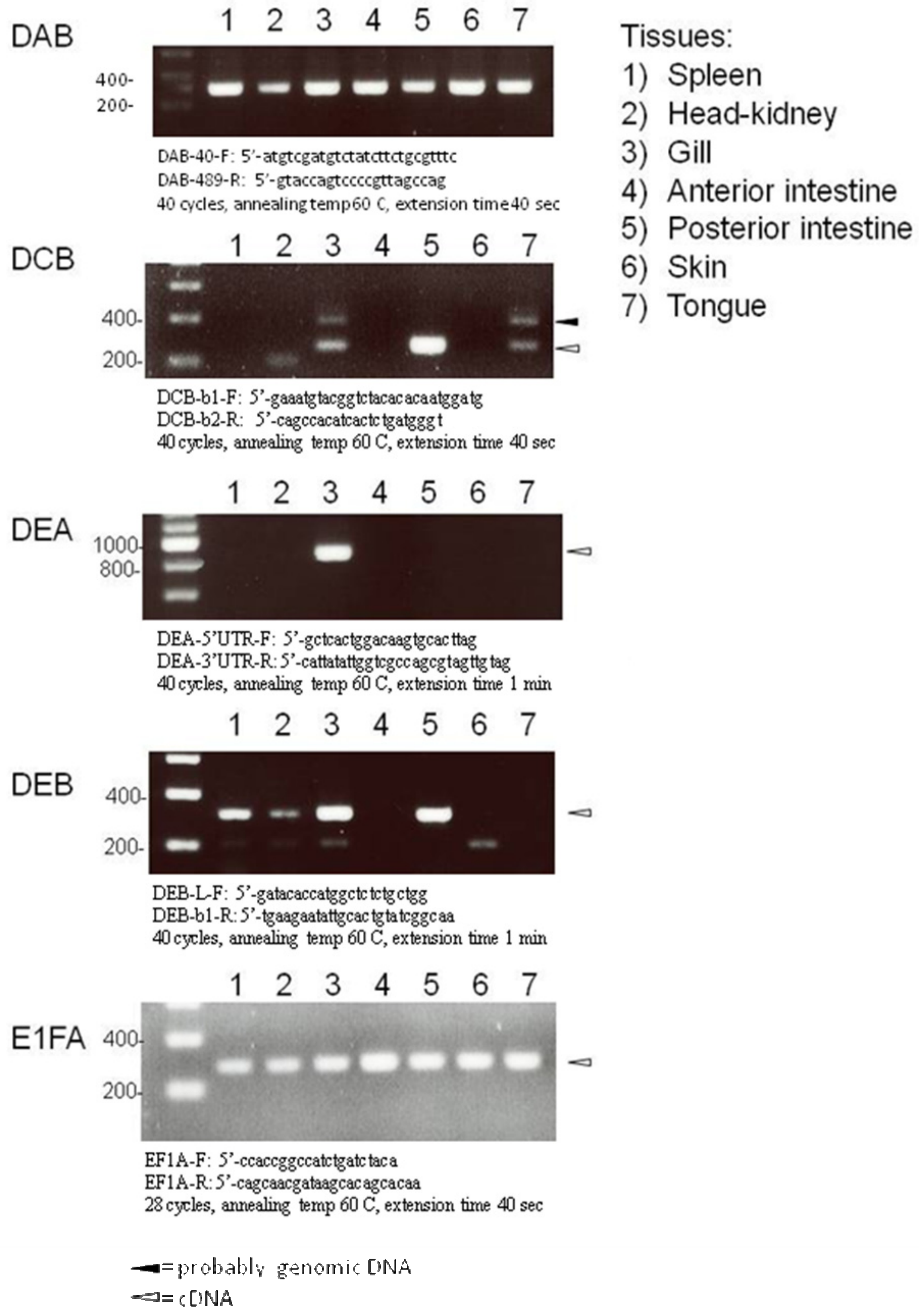

For RT-PCR analysis, RNA samples from tissues of three adult Atlantic salmon individuals from a Norwegian fish-farm preserved in RNAlater (Qiagen) were isolated using two-fold purification by TRIzol (Gibco), after which equal amounts of RNA were transcribed into cDNA using random hexamer primers and Superscript (Invitrogen). For analysis of tissue distribution, PCR was then performed using equal amounts of cDNA solution, ExTaq polymerase (Takara) and the primers and cycle numbers indicated in the figures in this paragraph. RT-PCR analysis of *DAB* and "housekeeping gene" *E1FA* served as positive controls. The figures show representative data obtained for a single salmon.

Text S2-3. Investigation of potential polymorphism of salmon *DCB*, *DEA* and *DEB*

Except for the amplifications shown above (Text S2-2*B*), also other *DCB*, *DEA* and *DEB* fragments were amplified from cDNA of the Atlantic salmon individuals from Norway in order to obtain more sequence information. For these amplifications ExTaq polymerase (Takara) and the following primer combinations were used:

(1) DCB-L-F (5'-TGTGCTGAATCTCTCGTCCATCC) +
DCB-3'UTR-R (5'-GTTTGTCACATCTGAAAATACCTTACAAAATCA)

(2) DEA-α1-F (5'-CAAACATTTGCTGCGGTTTTTGACC) +
DEA-3'UTR-R (5'-CATTATATTGGTCGCCAGCGTAGTTGTAG)

(3) DEB-L-F (5'-GATACACCATGGCTCTCTGCTGG) +
DEB-TM-R (5'-CTCCAATAGCCATGGCTACACCA)

(4) DEB-β2-F (5'-CTGTCCATGCTGGAGTGCAG) +
DEB-3'UTR-R (5'-ACACGCTCCAGAAGGTATATTTCACTG)

Furthermore, from genomic DNA of six Atlantic salmon individuals farmed in Canada, but originally derived from Norwegian stock, PCR fragments of *DCB*, *DEA* and *DEB* were amplified using ExTaq polymerase (Takara) and the following primer sets of which the primers are indicated in the below figures (Text S2-3*A*-to-*C*) with arrows:

(5) DCB-β1-F (5'-GAAATGTACGGTCTACACACAATGGATG) +
DCB-β2-R (5'-CAGCCACATCACTCTGATGGGT)

(6) DEA-α1-F (5'-CAAACATTTGCTGCGGTTTTTGACC) +
DEA-α1-R (5'-CTATTGCCTCTGGCGGGCTCTT)

(7) DEB-β1-F (5'-GTGGGTACCAGTTCCAGGGGATAGTTGAC) +
DEB-β1-R (5'-TGAAGAATATTGCACTGTATCGGCAA)

PCR fragments were cloned into plasmids, and for each individual multiple clones were sequenced. To account for possible PCR errors, sequences were only validated when obtained by independent amplifications.

In Texts S2-3*A* to-*C* the various (assembled) *DCB*, *DEA* and *DEB* sequences that we determined from the various Norwegian and Canadian salmon individuals are compared and provide an estimation of polymorphism. Only few different sequences were found, and they are not listed here per individual. Encoded amino acids are placed above the 2nd nucleotide of codons, and variable amino acids are shown below. Gen.=genomic; cDNA pred= cDNA sequence predicted from a genomic DNA sequence; TM= transmembrane domain; Cy= cytoplasmic tail. Polymorphic sites are marked orange. Alleles are numbered according to a nomenclature system described at http://www.ebi.ac.uk/ipd/mhc/fish/index.html. GenBank accession numbers are: *DCB*, KC316026-KC316031; *DEA*, KC316032-KC316035; *DEB*, KC316036.
Although there is some oligomorphism in *DCB*, this is highly different from the classical type polymorphism observed for the salmon *DAA* and *DAB* genes (http://www.ebi.ac.uk/ipd/mhc/fish/index.html). The DEA and DEB sequences are close to monomorphic, and their limited level of allelic variation is also found in many non-MHC molecules in fish [e.g., Guryev V, et al. (2006) Genetic variation in the zebrafish. *Genome Res* 16(4):491-497].

Text S2-3*A*. Sequence alignment of Atlantic salmon *DCB* alleles

```
                                                                          L  L
Sasa-DCB*0103 cDNA         1:                                           ACCTACTG  8
                                beta1-domain >
                            L  L  F  S  S  L  S  G  V  D  G  Y  F  G  H  F  E  M  R  C
Sasa-DCB*0103 cDNA         9:CTTCTCTTCTCCTCTCTGTCTGGAGTAGATGGCTATTTTGGACACTTTGAGATGAGGTGT  68

                            R  F  S  S  E  D  P  R  D  I  E  Y  L  L  Q  V  Y  G  N  K
Sasa-DCB*0103 cDNA        69:CGGTTCAGCTCCGAGGATCCCCGAGATATTGAGTATCTGCTGCAGGTCTACGGCAACAAG  128


                            K  L  L  G  Q  Y  N  S  T  T  E  K  C  T  V  Y  T  Q  W  M
Sasa-DCB*0103 cDNA       129:AAGTTACTGGGACAATACAACAGCACAACAGAGAAATGTACGGTCTACACACAATGGATG  188


                            K  N  F  T  E  T  A  C  K  G  P  A  F  L  S  E  R  R  E  E
Sasa-DCB*0101 gen.         1:AAGAACTTCACTGAAACGGCCTGCAAAGGCCCTGCTTTTCTGTCCGAAAGGAGAGAAGAA 60
                            K  N  F  T  E  T  A  C  K  G  P  A  F  L  A  E  R  R  E  E
Sasa-DCB*0102 cDNA         1:AAGAACTTCACTGAAACGGCCTGCAAAGGCCCTGCTTTTCTGGCCGAAAGGAGAGAAGAA 60
Sasa-DCB*0102 gen.         1:AAGAACTTCACTGAAACGGCCTGCAAAGGCCCTGCTTTTCTGGCCGAAAGGAGAGAAGAA 60
                            K  N  F  T  E  T  A  C  K  G  P  A  F  L  S  E  R  R  E  E
Sasa-DCB*0103 cDNA       189:AAGAACTTCACTGAAACGGCCTGCAAAGGCCCTGCTTTTCTGTCCGAAAGGAGAGAAGAA 248
Sasa-DCB*0103 gen.         1:AAGAACTTCACTGAAACGGCCTGCAAAGGCCCTGCTTTTCTGTCCGAAAGGAGAGAAGAA 60
                            K  N  F  T  E  T  A  C  K  G  P  A  F  L  A  D  R  R  E  E
Sasa-DCB*0201 gen.         1:AAGAACTTCACTGAAACGGCCTGCAAAGGCCCTGCTTTTCTGGCCGACAGGAGAGAAGAA 60


                            M  N  K  Y  C  S  S  N  V  P  V  V  Y  G  Y  L  L  D  K  A
Sasa-DCB*0101 gen.        61:ATGAATAAATACTGCAGCAGCAACGTTCCTGTGGTATATGGGTACTTACTAGATAAGGCA 120
                            M  N  K  Y  C  S  S  N  V  P  V  V  Y  G  Y  L  L  D  K  A
Sasa-DCB*0102 cDNA        61:ATGAATAAATACTGCAGCAGCAACGTTCCTGTGGTATATGGGTACTTACTAGATAAGGCA 120
Sasa-DCB*0102 gen.        61:ATGAATAAATACTGCAGCAGCAACGTTCCTGTGGTATATGGGTACTTACTAGATAAGGCA 120
                            M  N  K  Y  C  S  S  N  V  P  V  V  Y  G  Y  L  L  D  K  A
Sasa-DCB*0103 cDNA       249:ATGAATAAATACTGCAGCAGCAACGTTCCTGTGGTATATGGGTACTTACTAGATAAGGCA 308
Sasa-DCB*0103 gen.        61:ATGAATAAATACTGCAGCAGCAACGTTCCTGTGGTATATGGGTACTTACTAGATAAGGCA 120
                            M  K  K  Y  C  S  S  N  V  P  V  V  Y  G  Y  L  L  D  K  A
Sasa-DCB*0201 gen.        61:ATGAAGAAATACTGCAGCAGCAACGTTCCTGTGGTATATGGGTACTTACTAGATAAGGCA 120

                      Intron >
Sasa-DCB*0101 gen.       121:GGTAAGTGTATGAGTACATACTCAATGGTAGTGATAAGGAGGAGTACATACTAGATGAGA 180
Sasa-DCB*0102 cDNA       121:G----------------------------------------------------------- 121
Sasa-DCB*0102 gen.       121:GGTAAGTGTATGAGTACATACTCAATGGTAGTGATAAGGAGGAGTACATACTAGATGAGA 180
Sasa-DCB*0103 cDNA       309:G----------------------------------------------------------- 309
Sasa-DCB*0103 gen.       121:GGTAAGTGTATGAGTACATACTCAATGGTAGTGATAAGGAGGAGTACATACTAGATGAGA 180
Sasa-DCB*0201 gen.       121:GGTAAGTGTATGAGTACATACTCAATGGTAGTGATAAGGAGGAGTACATACTAGATGAGA 180
```

```
Sasa-DCB*0101 gen.      181:TAGATGAACATCATGAAAAGAGCACGAGGCAAGCCCTGAAGAACCCAGTATGTTCTTCCC 240
Sasa-DCB*0102 cDNA      121:------------------------------------------------------------ 121
Sasa-DCB*0102 gen.      181:TAGATGAACATCATGAAAAGAGCACGAGGCAAGCCCTGAAGAACCCAGTATGTTCTTCCC 240
Sasa-DCB*0103 cDNA      309:------------------------------------------------------------ 309
Sasa-DCB*0103 gen.      181:TAGATGAACATCATGAAAAGAGCACGAGGCAAGCCCTGAAGAACCCAGTATGTTCTTCCC 240
Sasa-DCB*0201 gen.      181:TAGATGAACATCATGAAAAGAGCACGAGGCAAGCCCTGAAGAACCCAGTATGTTCTTCCC 240

                      beta2 domain >
                                   V  E  P  Y  I  R  L  R  S  V  E  S  F  S  T  R  H  P
Sasa-DCB*0101 gen.      241:TCTCCAGTTGAGCCCTACATCAGGCTGAGATCAGTGGAGTCATTCAGTACCAGACACCCG 300
                                   V  E  P  Y  I  R  L  R  S  V  E  S  F  S  T  R  H  P
Sasa-DCB*0102 cDNA      122:-------TTGAGCCCTACATCAGGCTGAGATCAGTGGAGTCATTCAGTACCAGACACCCG 174
Sasa-DCB*0102 gen.      241:TCTCCAGTTGAGCCCTACATCAGGCTGAGATCAGTGGAGTCATTCAGTACCAGACACCCG 300
                                   V  E  P  Y  I  R  L  R  S  V  E  S  F  S  T  R  H  P
Sasa-DCB*0103 cDNA      310:-------TTGAGCCCTACATCAGGCTGAGATCAGTGGAGTCATTCAGTACCAGACACCCG 362
Sasa-DCB*0103 gen.      241:TCTCCAGTTGAGCCCTACATCAGGCTGAGATCAGTGGAGTCATTCAGTACCAGACACCCG 300
                                   V  E  P  Y  I  S  L  R  S  V  E  P  F  S  T  R  H  P
Sasa-DCB*0201 gen.      241:TCTCCAGTTGAGCCCTACATCAGCCTGAGATCAGTGGAGCCCGTTCAGTACCAGACACCCG 300

                             A  M  L  V  C  S  A  Y  D  F  Y  S
Sasa-DCB*0101 gen.      301:GCTATGCTCGTGTGCAGTGCCTACGACTTCTACTCCAA                       338
                             A  M  L  V  C  S  A  Y  D  F  Y  P
Sasa-DCB*0102 cDNA      175:GCCATGCTCGTGTGCAGTGCCTACGACTTCTACCCCAA                       212
Sasa-DCB*0102 gen.      301:GCCATGCTCGTGTGCAGTGCCTACGACTTCTACCCCAA <---------------------  338
                             A  M  L  V  C  S  A  Y  D  F  Y  P  K  P  I  R  V  T  W  L
Sasa-DCB*0103 cDNA      363:GCCATGCTCGTGTGCAGTGCCTACGACTTCTACCCCAAACCCATCAGAGTGACGTGGCTG 422
Sasa-DCB*0103 gen.      301:GCCATGCTCGTGTGCAGTGCCTACGACTTCTACCCCAA                       338
                             A  M  L  V  C  S  A  Y  D  F  Y  S
Sasa-DCB*0201 gen.      301:GCTATGCTCGTGTGCAGTGCCTACGACTTCTACTCCAA                       338


                             R  D  G  Q  E  V  T  S  N  V  T  S  T  E  E  L  V  N  G  D
Sasa-DCB*0103 cDNA      423:AGGGACGGACAGGAAGTGACCTCAAATGTGACTTCCACAGAGGAGCTGGTCAATGGGGAC  482

                             W  T  Y  Q  I  H  S  H  L  E  Y  T  P  T  P  G  E  R  I  T
Sasa-DCB*0103 cDNA      483:TGGACCTACCAGATCCACTCGCACCTGGAGTACACACCCACACCTGGAGAGAGAATCACC  542

                                                          Linker/TM/CY domains >
                             C  M  V  E  H  F  S  L  T  E  P  K  L  Y  D  W  D  P  S  L
Sasa-DCB*0103 cDNA      543:TGTATGGTGGAGCACTTCAGCCTCACTGAGCCCAAACTGTATGACTGGGACCCCTCCCTG  602

                             P  G  P  E  R  N  K  M  V  I  G  A  C  G  L  L  L  G  V  V
Sasa-DCB*0103 cDNA      603:CCTGGGCCTGAGAGGAATAAGATGGTGATCGGGGCCTGTGGACTGCTGCTGGGGGTGGTC  662

                             F  I  A  A  G  L  I  Y  Y  R  K  K  S  T  E  G  R  V  L  E
Sasa-DCB*0103 cDNA      663:TTTATAGCAGCTGGACTGATCTACTACAGGAAGAAATCTACTGAAGGACGAGTGTTGGAG  722

                                                               3'UTR >
                             P  T  M  A  L  P  E  S  Y  G  T  I  *
Sasa-DCB*0103 cDNA      723:CCGACCATGGCGCTGCCAGAAAGTTATGGCACCATATAGTGGTTCACACCAGTCTGCCTT  782

Sasa-DCB*0103 cDNA      783:CTGAAGGTTGTGATCTGAACCCACTTAATTAGTCTGGGTGCCAGTCTTTTTAGCTAACAT  842

Sasa-DCB*0103 cDNA      843:TCCACTCCTCGTAGCCTTTTCCGTGACATATGCCAAGCAGGTCGAGTAGTGGAGTGTCAG  902

Sasa-DCB*0103 cDNA      903:CTAAAAAGACTGGTACCCAGACTACCCACTTAATCATTCCAAGCATAATGTCCTCTGTCT  962

Sasa-DCB*0103 cDNA      963:CCCAGGCCTTTTGCATTGGAGGAGAGGAGAGGTTGCAGCCAGAAACTCCCAAGGCCCTTC  1022

Sasa-DCB*0103 cDNA     1023:TATAGGGATCACAATCACGACATTCAGATCAAGAGTTTACCACCGAGTGGCCAACGTGAT  1082

Sasa-DCB*0103 cDNA     1083:CAAATCTATGTCCACATCCTGTGCGTCTGAGACGTTAGATCTATGATA  1130
```

Text S2-3*B*. Sequence alignment of Atlantic salmon *DEA* alleles

Sasa-DEA*0101 cDNA pred is a sequence predicted from our Ssa1_scf_10 genomic sequence (Table TS2-1), while the Sasa-DEA*0102 and *0103 cDNA sequences were obtained from individuals from a Norwegian farm. In salmon individuals from a Canadian fish farm, for which genomic fragments between arrows were amplified and sequenced, sequences matching Sasa-DEA*0101 and *0102/*0103 were found. Although not shown in Text S2-2*B*, also splicoforms without the α2 exon were detected (Δα2).

```
                          Leader >
                                  M  G  C  R  V  F  L  A  F  I  L  G  V  C  L  L  S  H  C  Q
Sasa-DEA*0101 cDNA pred   1:-ATGGGGTGCAGAGTGTTCCTGGCTTTTATACTGGGTGTTTGTCTACTGTCACACTGCCA 59
                                  M  G  C  R  V  F  L  A  F  I  L  G  V  C  L  L  S  H  C  Q
Sasa-DEA*0102 cDNA        1:GATGGGGTGCAGAGTGTTCCTGGCTTTTATACTGGGTGTTTGTCTACTGTCACACTGCCA 60
                                  M  G  C  R  V  F  L  A  F  I  L  G  V  C  L  L  S  H  C  Q
Sasa-DEA*0102 cDNA Δα2    1:GATGGGGTGCAGAGTGTTCCTGGCTTTTATACTGGGTGTTTGTCTACTGTCACACTGCCA 60


                    alpha1 domain >
                                  S  K  H  L  L  R  F  L  T  F  C  Q  K  N  V  P  S  D  E  E
Sasa-DEA*0101 cDNA pred  60:AAGCAAACATTTGCTGCGGTTTTTGACCTTCTGCCAAAAAAACGTCCCCAGCGACGAAGA 119
                                  S  K  H  L  L  R  F  L  T  F  C  Q  K  N  V  P  S  D  E  E
Sasa-DEA*0102 cDNA       61:AAGCAAACATTTGCTGCGGTTTTTGACCTTCTGCCAAAAAAACGTCCCCAGCGACGAAGA 120
                                  S  K  H  L  L  R  F  L  T  F  C  Q  K  N  V  P  S  D  E  E
Sasa-DEA*0102 cDNA Δα2   61:AAGCAAACATTTGCTGCGGTTTTTGACCTTCTGCCAAAAAAACGTCCCCAGCGACGAAGA 120
                                                             F  C  Q  K  N  V  P  S  D  E  E
Sasa-DEA*0103 cDNA        1:                            TTCTGCCAAAAAAACGTCCCCAGCGACGAAGA 32
                                                             F  C  Q  K  N  V  P  S  D  E  E
Sasa-DEA*0103 cDNA Δα2    1:                            TTCTGCCAAAAAAACGTCCCCAGCGACGAAGA 32


                                  Y  D  V  E  F  D  G  D  E  L  F  Y  V  D  P  I  T  Y  Q  V
Sasa-DEA*0101 cDNA pred 120:GTATGACGTGGAGTTTGATGGAGATGAGCTCTTCTACGTTGACCCCATTACCTACCAGGT 179
                                  Y  D  V  E  F  D  G  D  E  L  F  Y  V  D  S  I  T  Y  Q  V
Sasa-DEA*0102 cDNA      121:GTATGACGTGGAGTTTGATGGAGATGAGCTCTTCTACGTTGACTCCATTACCTACCAGGT 180
                                  Y  D  V  E  F  D  G  D  E  L  F  Y  V  D  S  I  T  Y  Q  V
Sasa-DEA*0102 cDNA Δα2  121:GTATGACGTGGAGTTTGATGGAGATGAGCTCTTCTACGTTGACTCCATTACCTACCAGGT 180
                                  Y  D  V  E  F  D  G  D  E  L  F  Y  V  D  S  I  T  Y  Q  V
Sasa-DEA*0103 cDNA       33:GTATGACGTGGAGTTTGATGGAGATGAGCTCTTCTACGTTGACTCCATTACCTACCAGGT 92
                                  Y  D  V  E  F  D  G  D  E  L  F  Y  V  D  S  I  T  Y  Q  V
Sasa-DEA*0103 cDNA Δα2   33:GTATGACGTGGAGTTTGATGGAGATGAGCTCTTCTACGTTGACTCCATTACCTACCAGGT 92


                                  E  R  R  L  S  E  F  A  Q  Q  W  T  P  D  P  G  L  P  H  E
Sasa-DEA*0101 cDNA pred 180:AGAGCGGCGTCTGTCAGAGTTCGCCCAGCAGTGGACCCCTGACCCAGGACTGCCTCACGA 239
                                  E  R  R  L  S  E  F  A  Q  Q  W  T  P  D  P  G  L  P  H  E
Sasa-DEA*0102 cDNA      181:AGAGCGGCGTCTGTCAGAGTTCGCCCAGCAGTGGACCCCTGACCCAGGACTGCCTCACGA 240
                                  E  R  R  L  S  E  F  A  Q  Q  W  T  P  D  P  G  L  P  H  E
Sasa-DEA*0102 cDNA Δα2  181:AGAGCGGCGTCTGTCAGAGTTCGCCCAGCAGTGGACCCCTGACCCAGGACTGCCTCACGA 240
                                  E  R  R  L  S  E  F  A  Q  Q  W  T  P  D  P  G  L  P  H  E
Sasa-DEA*0103 cDNA       93:AGAGCGGCGTCTGTCAGAGTTCGCCCAGCAGTGGACCCCTGACCCAGGACTGCCTCACGA 152
                                  E  R  R  L  S  E  F  A  Q  Q  W  T  P  D  P  G  L  P  H  E
Sasa-DEA*0103 cDNA Δα2   93:AGAGCGGCGTCTGTCAGAGTTCGCCCAGCAGTGGACCCCTGACCCAGGACTGCCTCACGA 152


                                  V  Y  V  S  L  G  T  C  Q  Y  N  I  P  R  C  I  V  G  E  K
Sasa-DEA*0101 cDNA pred 240:AGTGTACGTGTCCCTCGGAACTTGCCAGTACAACATCCCCCGCTGCATTGTGGGAGAAAA 299
                                  V  Y  V  S  L  G  T  C  Q  Y  N  I  P  R  C  I  V  G  E  K
Sasa-DEA*0102 cDNA      241:AGTGTACGTGTCCCTCGGAACTTGCCAGTACAACATCCCCCGCTGCATTGTGGGAGAAAA 300
                                  V  Y  V  S  L  G  T  C  Q  Y  N  I  P  R  C  I  V  G  E  K
Sasa-DEA*0102 cDNA Δα2  241:AGTGTACGTGTCCCTCGGAACTTGCCAGTACAACATCCCCCGCTGCATTGTGGGAGAAAA 300
                                  V  Y  V  S  L  G  T  C  Q  Y  N  I  P  R  C  I  V  G  E  K
Sasa-DEA*0103 cDNA      153:AGTGTACGTGTCCCTCGGAACTTGCCAGTACAACATCCCCCGCTGCATTGTGGGAGAAAA 212
                                  V  Y  V  S  L  G  T  C  Q  Y  N  I  P  R  C  I  V  G  E  K
Sasa-DEA*0103 cDNA Δα2  153:AGTGTACGTGTCCCTCGGAACTTGCCAGTACAACATCCCCCGCTGCATTGTGGGAGAAAA 212
```

```
                                   alpha2 domain >
                               ________________
                                S  P  P  E  A  I  E  V  P  T  S  H  I  Y  S  Q  R  E  V  E
Sasa-DEA*0101 cDNA pred 300:GAGCCCGCCAGAGGCAATAGAGGTGCCCACCTCCCACATATACTCCCAGAGAGAGGTGGA 359
                                S  P  P  E  A  I  E  V  P  T  S  H  I  Y  S  Q  R  E  V  E
Sasa-DEA*0102 cDNA      301:GAGCCCGCCAGAGGCAATAGAGGTGCCCACCTCCCACATATACTCCCAGAGAGAGGTGGA 360
                                S  P  P  E  A  I
Sasa-DEA*0102 cDNA Δα2  301:GAGCCCGCCAGAGGCAATAG---------------------------------------- 320
                                S  P  P  E  A  I  E  V  P  T  S  H  I  Y  S  Q  R  E  V  E
Sasa-DEA*0103 cDNA      213:GAGCCCGCCAGAGGCAATAGAGGTGCCCACCTCCCACATATACTCCCAGAGAGAGGTGGA 272
                                S  P  P  E  A  I
Sasa-DEA*0103 cDNA Δα2  213:GAGCCCGCCAGAGGCAATAG---------------------------------------- 232


                                L  G  V  P  N  T  L  I  C  R  V  S  D  F  H  P  T  P  V  D
Sasa-DEA*0101 cDNA pred 360:GCTGGGGGTCCCCAACACACTCATCTGCCGGGTCAGTGATTTCCACCCAACACCCGTGGA 419
                                L  G  V  P  N  T  L  I  C  R  V  S  D  F  H  P  T  P  V  D
Sasa-DEA*0102 cDNA      361:GCTGGGGGTCCCCAACACACTCATCTGCCGGGTCAGTGATTTCCACCCAACACCCGTGGA 420
Sasa-DEA*0102 cDNA Δα2  324:------------------------------------------------------------ 324
                                L  G  V  P  N  T  L  I  C  R  V  S  D  F  H  P  T  P  V  D
Sasa-DEA*0103 cDNA      273:GCTGGGGGTCCCCAACACACTCATCTGCCGGGTCAGTGATTTCCACCCAACACCCGTGGA 332
Sasa-DEA*0103 cDNA Δα2  232:------------------------------------------------------------ 232


                                V  T  W  T  R  N  E  Q  P  V  A  E  R  T  I  I  Q  T  Q  Y
Sasa-DEA*0101 cDNA pred 420:TGTGACTTGGACCAGGAACGAGCAGCCGGTGGCAGAGAGGACCATCATTCAGACCCAGTA 479
                                V  T  W  T  R  N  E  Q  P  V  A  E  R  T  I  I  Q  T  Q  Y
Sasa-DEA*0102 cDNA      421:TGTGACTTGGACCAGGAACGAGCAGCCGGTGGCAGAGAGGACCATCATTCAGACCCAGTA 480
Sasa-DEA*0102 cDNA Δα2  320:------------------------------------------------------------ 320
                                V  T  W  T  R  N  E  Q  P  V  A  E  R  T  I  I  Q  T  Q  Y
Sasa-DEA*0103 cDNA      333:TGTGACTTGGACCAGGAACGAGCAGCCGGTGGCAGAGAGGACCATCATTCAGACCCAGTA 392
Sasa-DEA*0103 cDNA Δα2  232:------------------------------------------------------------ 232


                                Y  S  N  E  D  F  S  F  R  I  F  S  Y  L  S  I  T  P  Q  E
Sasa-DEA*0101 cDNA pred 480:CTACTCCAATGAAGACTTCAGCTTCAGGATCTTCTCCTACCTGAGCATCACACCTCAGGA 539
                                Y  S  N  E  D  F  S  F  R  I  F  S  Y  L  S  I  T  P  Q  E
Sasa-DEA*0102 cDNA      481:CTACTCCAATGAAGACTTCAGCTTCAGGATCTTCTCCTACCTGAGCATCACACCTCAGGA 540
Sasa-DEA*0102 cDNA Δα2  320:------------------------------------------------------------ 320
                                Y  S  N  E  D  F  S  F  R  I  F  S  Y  L  S  I  T  P  Q  E
Sasa-DEA*0103 cDNA      393:CTACTCCAATGAAGACTTCAGCTTCAGGATCTTCTCCTACCTGAGCATCACACCTCAGGA 452
Sasa-DEA*0103 cDNA Δα2  232:------------------------------------------------------------ 232


                                G  D  I  Y  S  C  S  V  G  H  V  S  L  Q  E  P  L  T  R  I
Sasa-DEA*0101 cDNA pred 540:GGGGGACATCTACTCCTGTAGTGTGGGTCACGTCAGCCTGCAGGAACCCCTCACCAGGAT 599
                                G  D  I  Y  S  C  S  V  G  H  V  S  L  Q  E  P  L  T  R  I
Sasa-DEA*0102 cDNA      541:GGGGGACATCTACTCCTGTAGTGTGGGTCACGTCAGCCTGCAGGAACCCCTCACCAGGAT 600
Sasa-DEA*0102 cDNA Δα2  320:------------------------------------------------------------ 320
                                G  D  I  Y  S  C  S  V  G  H  V  S  L  Q  E  P  L  T  R  I
Sasa-DEA*0103 cDNA      453:GGGGGACATCTACTCCTGTAGTGTGGGTCACGTCAGCCTGCAGGAACCCCTCACCAGGAT 512
Sasa-DEA*0103 cDNA Δα2  236:------------------------------------------------------------ 236


             Linker/TM/CY domains >
                                W  E  V  E  V  H  T  D  H  Q  T  V  E  T  A  V  C  V  G  G
Sasa-DEA*0101 cDNA pred 600:CTGGGAGGTTGAAGTGCACACGGACCACCAGACTGTGGAGACAGCAGTGTGTGTTGGAGG 659
                                W  E  V  E  V  H  T  D  H  Q  T  V  E  T  A  V  C  V  G  G
Sasa-DEA*0102 cDNA      601:CTGGGAGGTTGAAGTGCACACGGACCACCAGACTGTGGAGACAGCAGTGTGTGTTGGAGG 660
                                   E  V  E  V  H  T  D  H  Q  T  V  E  T  A  V  C  V  G  G
Sasa-DEA*0102 cDNA Δα2  321:-----AGGTTGAAGTGCACACGGACCACCAGACTGTGGAGACAGCAGTGTGTGTTGGAGG 375
                                W  E  V  E  V  H  T  D  H  Q  T  V  E  T  A  V  C  V  G  G
Sasa-DEA*0103 cDNA      513:CTGGGAGGTTGAAGTGCACACGGACCACCAGACTGTGGAGACAGCAGTGTGTGTTGGAGG 572
                                   E  V  E  V  H  T  D  H  Q  T  V  E  T  A  V  C  V  G  G
Sasa-DEA*0103 cDNA Δα2  233:-----AGGTTGAAGTGCACACGGACCACCAGACTGTGGAGACAGCAGTGTGTGTTGGAGG 287
```

```
                              V  T  L  G  V  V  G  V  A  T  G  V  W  F  I  K  K  A  K  R
Sasa-DEA*0101 cDNA pred 660:TGTGACTCTAGGAGTGGTTGGAGTAGCAACAGGAGTCTGGTTTATCAAAAAAGCCAAGAG 719
                              V  T  L  G  V  V  G  V  A  T  G  V  W  F  I  K  K  A  K  R
Sasa-DEA*0102 cDNA      661:TGTGACTCTAGGAGTGGTGGGAGTAGCAACAGGAGTCTGGTTTATCAAAAAAGCCAAGAG 720
                              V  T  L  G  V  V  G  V  A  T  G  V  W  F  I  K  K  A  K  R
Sasa-DEA*0102 cDNA Δα2  376:TGTGACTCTAGGAGTGGTGGGAGTAGCAACAGGAGTCTGGTTTATCAAAAAAGCCAAGAG 435
                              V  T  L  G  V  V  G  V  A  T  G  V  W  F  I  K  K  A  K  R
Sasa-DEA*0103 cDNA      573:TGTGACTCTAGGAGTGGTTGGAGTAGCAACAGGAGTCTGGTTTATCAAAAAAGCCAAGAG 632
                              V  T  L  G  V  V  G  V  A  T  G  V  W  F  I  K  K  A  K  R
Sasa-DEA*0103 cDNA Δα2  288:TGTGACTCTAGGAGTGGTTGGAGTAGCAACAGGAGTCTGGTTTATCAAAAAAGCCAAGAG 347

                                               3'UTR >
                              S  G  W  A  L  R  T  *
Sasa-DEA*0101 cDNA pred 720:GTCTGGTTGGGCCTTAAGAACATAG                                   744
                              S  G  W  A  L  R  T  *
Sasa-DEA*0102 cDNA      721:GTCTGGTTGGGCCTTAAGAACATAGGCAACAATCAGCTATCAGCGATACAGCAAGGGCTC 780
                              S  G  W  A  L  R  T  *
Sasa-DEA*0102 cDNA Δα2  436:GTCTGGTTGGGCCTTAAGAACATAGGCAACAATCAGCTATCAGCGATACAGCAAGGGCTC 495
                              S  G  W  A  L  R  T  *
Sasa-DEA*0103 cDNA      633:GTCTGGTTGGGCCTTAAGAACATAGGCAACAATCAGCTATCAGCGATACAGCAAGGGCTC 692
                              S  G  W  A  L  R  T  *
Sasa-DEA*0103 cDNA Δα2  348:GTCTGGTTGGGCCTTAAGAACATAGGCAACAATCAGCTATCAGCGATACAGCAAGGGCTC 407

Sasa-DEA*0102 cDNA      781:CATGCAACAATACCAAGGACAATTCTCATATTTAGCAAC                     819
Sasa-DEA*0102 cDNA Δα2  496:CATGCAACAATACCAAGGACAATTCTCATATTTAGCAAC                     534
Sasa-DEA*0103 cDNA      693:CATGCAACAATACCAAGGACAATTATCATATTTAGCAAC                     731
Sasa-DEA*0103 cDNA Δα2  408:CATGCAACAATACCAAGGACAATTATCATATTTAGCAAC                     446
```

Text S2-3*C*. Sequence alignment of Atlantic salmon *DEB* alleles

The *DEB**0101 cDNA pred is a sequence predicted from our Ssa1_scf_10 genomic sequence (Table TS2-1), which was corrected (1 nt was deleted) at the site of the downward arrowhead to be in frame and consistent with the detected cDNA sequences. In six salmon individuals from a Canadian fish farm, for which genomic fragments between arrows were amplified and sequenced, only sequences matching *Sasa-DEB**0101 were found.

```
                    Leader >
                             M  A  L  C  W  V  Y  W  M  A  G  L  S  V  I  Q  T  W  A  T
Sasa-DEB*0101 cDNA pred   1:ATGGCTCTCTGCTGGGTCTATTGGATGGCTGGACTTTCTGTCATTCAGACCTGGGCAACA 60
                                            V  Y  W  M  A  G  L  S  V  I  Q  T  W  A  T
Sasa-DEB*0102 cDNA        1:---------------GTCTATTGGATGGCTGGACTTTCTGTCATTCAGACCTGGGCAACA 45

                                 ---------------------------->
                     beta1 domain >
                             P  A  G  G  Y  Q  F  Q  G  I  V  D  C  E  Y  D  D  T  I  D
Sasa-DEB*0101 cDNA pred  61:CCAGCAGGTGGGTACCAGTTCCAGGGGATAGTTGACTGTGAATATGATGACACCATTGAC 120
                             P  A  G  G  Y  Q  F  Q  G  I  V  D  C  E  Y  D  D  T  I  D
Sasa-DEB*0102 cDNA       46:CCAGCAGGTGGGTACCAGTTCCAGGGGATAGTTGACTGTGAATATGATGACACCATTGAC 105


                             N  M  I  Y  F  V  K  N  I  F  N  Q  K  L  T  T  I  Y  D  S
Sasa-DEB*0101 cDNA pred 121:AATATGATCTACTTTGTGAAAAACATATTCAACCAAAAATTGACCACCATCTATGACTCC 180
                             N  M  I  Y  F  V  K  N  I  F  D  Q  K  L  T  T  I  Y  D  S
Sasa-DEB*0102 cDNA      106:AATATGATCTACTTTGTGAAAAACATATTCGACCAAAAATTGACCACCATCTATGACTCC 165


                             R  V  Q  K  Y  V  G  F  G  E  F  G  I  R  N  A  D  R  Y  N
Sasa-DEB*0101 cDNA pred 181:AGGGTTCAGAAGTATGTGGGTTTTGGGGAGTTTGGCATACGCAATGCAGATCGCTACAAC 240
                             R  V  Q  K  Y  V  G  F  G  E  F  G  I  R  N  A  D  R  Y  N
Sasa-DEB*0102 cDNA      166:AGGGTTCAGAAGTATGTGGGTTTTGGGGAGTTTGGCATACGCAATGCAGATCGCTACAAC 225
```

```
                             S  Q  A  W  K  M  A  I  R  K  A  E  V  E  T  I  C  R  Y  S
Sasa-DEB*0101 cDNA pred 241:AGCCAAGCTTGGAAAATGGCTATAAGGAAAGCAGAAGTGGAGACTATTTGCCGATACAGT 300
                             S  Q  A  W  K  M  A  I  R  K  A  E  V  E  T  I  C  R  Y  S
Sasa-DEB*0102 cDNA      226:AGCCAAGCTTGGAAAATGGCTATAAGGAAAGCAGAAGTGGAGACTATTTGCCGATACAGT 285

                                          beta2 domain >
                             A  I  F  F  K  L  S  T  L  E  R  I  V  P  P  I  V  K  V  R
Sasa-DEB*0101 cDNA pred 301:GCAATATTCTTCAAACTGAGCACTTTGGAAAGAATAGTGCCTCCCATTGTCAAAGTCCGC 360
                             A  I  F  F  K  L  S  T  L  E  R  I  V  P  P  I  V  K  V  R
Sasa-DEB*0102 cDNA      286:GCAATATTCTTCAAACTGAGCACTTTGGAAAGAATAGTGCCTCCCATTGTCAAAGTCCGC 345

                             L  T  K  P  S  R  Y  G  E  L  S  M  L  E  C  S  V  L  G  F
Sasa-DEB*0101 cDNA pred 361:CTGACCAAGCCCTCTCGCTACGGGGAGCTGTCCATGCTGGAGTGCAGCGTTCTAGGCTTC 420
                             L  T  K  P  S  R  Y  G  E  L  S  M  L  E  C  S  V  L  G  F
Sasa-DEB*0102 cDNA      346:CTGACCAAGCCCTCTCGCTACGGGGAGCTGTCCATGCTGGAGTGCAGCGTTCTAGGCTTC 405

                             Y  P  Q  E  V  R  V  S  W  L  R  D  G  R  E  T  T  T  A  V
Sasa-DEB*0101 cDNA pred 421:TACCCTCAGGAGGTGAGGGTGAGCTGGCTGAGAGACGGGCGGGAGACCACCACAGCCGTG 480
                             Y  P  Q  E  V  R  V  S  W  L  R  D  G  L  E  T  T  T  A  V
Sasa-DEB*0102 cDNA      406:TACCCTCAGGAGGTGAGGGTGAGCTGGCTGAGAGACGGGCTGGAGACCACCACAGCCGTG 465

                             T  S  T  D  T  L  A  N  G  D  W  S  Y  Q  L  H  S  Y  L  E
Sasa-DEB*0101 cDNA pred 481:ACCTCCACTGACACACTGGCTAACGGGGACTGGAGCTACCAGCTCCACTCCTACCTAGAG 540
                             T  S  T  D  T  L  A  N  G  D  W  S  Y  Q  L  H  S  Y  L  E
Sasa-DEB*0102 cDNA      466:ACCTCCACTGACACACTGGCTAACGGGGACTGGAGCTACCAGCTCCACTCCTACCTAGAG 525

                             F  R  P  Q  R  G  E  S  V  S  C  M  V  E  H  P  S  L  D  E
Sasa-DEB*0101 cDNA pred 541:TTCAGGCCTCAGAGAGGGGAGAGTGTAAGCTGCATGGTTGAGCACCCCAGCCTAGATGAA 600
                             F  R  P  Q  R  G  E  S  V  S  C  M  V  E  H  P  S  L  D  E
Sasa-DEB*0102 cDNA      526:TTCAGGCCTCAGAGAGGGGAGAGTGTAAGCTGCATGGTTGAGCACCCCAGCCTAGATGAA 585

                          Linker/TM/CY domains >
                             P  L  E  V  V  W  D  T  S  G  L  D  A  K  W  F  K  M  A  I
Sasa-DEB*0101 cDNA pred 601:CCACTGGAGGTGGTCTGGGATACCTCAGGCCTGGATGCTAAATGGTTTAAAATGGCAATA 660
                             P  L  E  V  V  W  D  T  S  G  L  D  A  K  W  F  K  M  A  I
Sasa-DEB*0102 cDNA      586:CCACTGGAGGTGGTCTGGGATACCTCAGGCCTGGATGCTAAATGGTTTAAAATGGCAATA 645

                             G  V  C  S  L  F  I  G  V  A  M  A  I  G  G  G  V  Y  Y  W
Sasa-DEB*0101 cDNA pred 661:GGGGTGTGTTCCCTTTTCATTGGTGTAGCCATGGCTATTGGAGGAGGAGTTTACTACTGG 720
                             G  V  C  S  L  F  I  G  V  A  M  A  I  G  G  G  V  Y  Y  W
Sasa-DEB*0102 cDNA      646:GGGGTGTGTTCCCTTTTCATTGGTGTAGCCATGGCTATTGGAGGAGGAGTTTACTACTGG 705

                                                   3'UTR >
                             W  K  N  R  S  G  F  R  R  V  N  R  *
Sasa-DEB*0101 cDNA pred 721:TGGAAGAACAGGTCAGGCTTTAGGCGTGTGAATAGATAA                      759
                             W  K  N  R  S  G  F  R  R  V  N  R  *
Sasa-DEB*0102 cDNA      706:TGGAAGAACAGGTCAGGCTTTAGGCGTGTGAATAGATAAGTCATTGGCAGCAGAGAACCG 765

Sasa-DEB*0102 cDNA      766:GAAAGGAAAGGCGGCCAAAGGAGGAATTGGCTTTGGGGGTGAC                  808
```

Table TS2-4. Genes situated in Atlantic salmon *DEA*/*DEB* (Ssal_scf_10) and *DFA*ψ/*DFB*ψ (Ssal_scf_137) genomic regions. Analyzed stretches of confirmed 411 and 428 kb regions of Ssal_scf_10 and Ssal_scf_137, respectively.

**Gene**	**Short name Ssa1_scf-10 (Ssa1-scf-137)**	**Ssal_scf_10**	**Ssal_scf_137**
Inactive phospholipase C-like protein 2	PLCL2	1.602 - 21.181	392.324 - 427.152
Inactive phospholipase C-like protein 2	PLCL2	51.359 - 51.646 (pseudo)	None
Cysteine-rich secretory protein-like	CRISP_L	58.675 - 62.766	None
Raftilin –like	RFTN	100.679 - 144.624 (pseudo)	None
Vitelline membrane outer layer protein 1	VMO1	153.552 - 157.956	None
MHCIIB	DEB (DFB)	158.800 - 160.528	258.882 - 259.877 (pseudo)
MHCIIA	DEA (DFA)	161.271 - 163.612	256.622 - 258.699 (pseudo)
Bromodomain containing 2	BRD2	171.995 - 177.278 (pseudo)	246.571 - 251.554
Transport-associated protein	TAP1	187.837 - 191.886 (pseudo)	230.485 - 237.656
NOTCH-like	NOTCH_L	194.568 - 200.065	224.757 - 229.063
Pre-B-cell leukemia homeobox 2	PBX2	200.909 - 206.709	218.364 - 223.704
G-protein signaling modulator 2-like	GPSM2_L	212.558 - 214.789	209.865 - 212.282
Deoxyribonuclease II-like	DNASE2_L	219.719 - 224.792	199.370 - 212.282
Ral guanine nucleotide dissociation stimulator-like 2	RGL2	227.464 - 240.837	184.391 - 196.696
Ral guanine nucleotide dissociation stimulator-like 2	RGL2	None	177.522 - 177.716 (pseudo)
PHD finger protein 1	PHF1	253.681 - 258.313	156.501 - 168.470
Synaptic Ras GTPase activating protein 1	SYNGAP1	281.804 - 292.762 (pseudo)	78.587 - 107.694
Synaptic Ras GTPase activating protein 1	SYNGAP1	325.393 - 352.011	64.244 - 74.707 (pseudo)
Smg-5 homolog, nonsense mediated mRNA decay factor like	SMG5_L	381.349 - 409.641	4.075 - 32.512

Pseudo= obvious pseudogenes.

Text S2-4*A*

>Ssa1_scf_10: 411 kb scaffold sequence including *DEA*/*DEB* loci

CCAAGGCTCTTTCCACTCCTATCAGCACACTGTAAACTAATCTATAGAGATCCTTCAGTTCCTCCAGCAGCTTCATGTGA
TGGTTGGGATATCCAGACCCTGCAGTCTCTGTCTGCCCTTAAACAGTAATACCATTAATACAACAGAAAACAGCACACTG
TAGAATGTTAATGAATGTTCATACGGTCTGTCATTGATTCTGACACCAGACACCATAAACGATTTGACGTAGTTTATTCT
GAAGGTTTCGGGAATTAACAGATAAGAGCAAAACCGGTATTCAGTTCAACTTTTCCCCCCTTCCATCTGTAAAAATGGTT
AACAACAGTTATAAATACAACTGTCACGACACTGAAGTTCATGAATGACTTTTTGTCCCAGTGTGGGCCCAGTTAAAACA
CTGGTGGTGAGATCCAGAGGTAGTGGTGGGTGGCTAAAGTTGACTAGGAGCAACACTGTTTCAGCACTGTGGAGAGAATG
AACAGCGGACAAATACAAAACTAGAAGCAACTGGTGGGGGAAAGCAGGTTAAGATGGATGGATGTGAGGAAGAGCGTCTC
TTTCCTCACCATCCATCCCCTTTGTTGACTGATTTGTAGCAATCATATATGAGGCACAGTCATATATGAAACCGTGTTCA
TGAATAGGGTGCTAGTTTGTTACCAGGACAGGTTGAGAAGCTATTATTACTTTGTACATCCCACTCAACCCTCTCACATG
GAATGGTGAAATGACACCAAAACGTAGGCGTTACTCTCTCCATGGTTCCTTCCCAAAGCAGTTGCTGGTAGTGCTTGAGA
CTCGACTGCCACCCTTGGACCACACACTGAGGTTTAAAGTCTCTATCCGTCCCTCTGTCTCGACAAAGGAACTCTCCAGT
GGGAAAGTGTAAAGGACACCTCAATTTCACATCTGCACAGACAAATAAAATACTTTTTTTTCCCATGGACGAGCCATTTC
CTTTACAAAATAAATCTTATTTCTCAAGAATACAATAATAAAATGAAGCACGTTGGAAAAAAAGTAACAGAACTCCATAT
ATACACATACTATTTTGGCAGACAAAAACATGAACAAGTTCATTATTGTAAACAGCTCTCTCCTCCCTTTTCCTCCCTTC
CTCTTCCTGTTTCCTATTACATTCACAGCTCTAGTTTCTGGTTGGTCAGAAACCCGTAAATTGTGCTTAAGAAATCCTTG
GACTGTTCAGAGTGGCTCTCCTAGGGTCAGACCCTGGGGTCTGCCTGTAAATAAGTGCACTGTCTCTACAGTAGGTCACT
GCTTTAAGACTGTCCCGTGGCTGCAAGTTCACGTGGCCCTCCTAGTTGACCCGCAACATCTTGCTGTTGAACGTTGAGGT
GGGCAGGAAGGGTTAAGGGCTTTTTGTGTGGTACCGGGTTAAGCAAAGGGAGGTTTGAGTGGGACTGGGGTGGGAATGGA
ATGGCTGTATACGGCGCTGGAATAAGAATAGTTGTGTGGCAACGGCACTAGAACAGTTATACTGTAGTTGGGTTTGTGTG
GGAATGCGGTAAGGAGATTTATGTATGCCACTAGGGTAGGGCAAGGGGCTTGGAGGTATGAAGTGTTTAGGGTGTAAGCC
TTCAGTTGTCCTCCTCAGAGAGCTCGTCCTCCCCCGTGGCTTTCTCGGGGATGGCGTCCAGGCTGCGTGGTCGGGTCCTG
GACTCGGAGCCTGCCGTGCTCCCTGATGGCCCGTCCTTACTGAGGTTACAGGTGAGGGCGGCATAGTGGATCTGCTTCAG
GTTCTCCTGGACCTCACTCTTTGCGTGTTTCAGCAGGTCCGCCTGGCCCTGGAGGGGATGCAGGGACATGACAGGAATGG
CAATATAGAGACAATGGAGCATGTATCAACAACTTCAATCTCCAGTTTAAACAGAAAGTCAAAGGGTGAAACCAATAATA
CCTTAAAAAGGTCACAAAGCGAGAGAGTAGTGTGGTACTAACTGTTTAATCTTAGTTGTATGCGGTCAGACATTGGAGTC
CTACATCTCCCCAACGCCCACAGAGCGTAACCGCTGCTTCTGTGTATAATGAATTATGCAGCGTAGCCAAACACTCACCA
GGCACCAACTCAATTTCCCGGGGACCATGTATAAATGATGAGCCCAAACGCTCATGAGGTGAGCATGCAATTAATGCATG
CAAATATTACAAATAAATATGCGGAAGGTCATTCCTCGATAATTACCCCTTGTATTGAGACAGGGATTAAATGAACGGCA
TGAAGACAAATACATCAGACTGAGTGTGTACAATCTAACTCAGACATAGGCTCTTATTCCACCGCGCTGCAGCTGAAAAA
CAAATGTCAATTTAAACCTAGCTACCTTATGTCCCTTCTGTAAACAAACTGTTTTTCTCATTTAGTGAAGCTCAACTGCA
TTGTGGAATCAGAATCTCTGAAGTGTTTGCTCGGAAGTAGTTTCTTGAATTTAAATTCATTGCCTACGCCTGTCCTCAAT
AAAACAAACATTAAGTTAAACCGCTTACATGTAACTAAATGTAATAGAAAATGTAATCTACGTAATCCCAAAAACGTATT
ATTTATCATGAGATAAACAATAACTAGTAATGCTGCATTCTCCAAGATTAAACTCTCACCTTTCTGGTAAAAATAGTTAC
ATTGCAGAGGTGTAGTCACTTTTGAGGACACAAGCTCAAGTACACAGATTTCCTATTCAACAAAACTGTATGAATAAGGC
TTGAAATGAATTTTTTTTCAAAACTATTAAGATTTCACCTTCCTTATAACTGTAAAATACATATTTGTATATTTAGTGTT
AGAGAAAATGTGCAAAGTTTCTAAAGGTCTCTCTTGATCAAAATGGCTGCCCTCATTGCTTTGAACATCTCTCAGAGCTC
TAGCTTGGCTTCCTGAACTCGTTAGGAACTCGCCTTTCATCTCATTTTAATATTGTCCATCTATGACAAAGTATTTTTTG
TTAATCTATTCATTATTTTTGAAATATCACATTTCCATAAGTATTCAGACCCATTACTCAGTACTTTGTTGAAGCGCCTT
TGGCAGCAATTACAGCCTCGAGGCTTCTTGGGTATGACGGTACAAGCTTGGCATACCTGTATTTGGGGAGTTTCTCCCAT
TCTTCTCTGCAGATCCTCTCAAGCTCTGTCAGGTTGGATGGGAAGCGTTGATGCACAGCTATTTTCAGGTCTCTCCAGAG
ACGTTAGATCAGCTTCAAGTCTGGGCAACTCAAGGACATTCAGAGACTTGTCCCGAAGCCACTCCTGCATTGTCTTGGCT
GTGTGCTTAGGGTCGTTGTCCTGTTGGAAGGTGAACCTTTGCCCCAGTCTGAGCGCTCTGGAGCAGGTTTTTATCAAGGA
TCTCTGTACTTTGCTCCGTTCATCTTTGCAACGATCCTGACTAGTCTCCCAGTCCATCGCTGAAAAACACCCCCACAGCA
TTATGCTGCCACCACCATGCTTCACCGTAGGGATGGTGCCAGGTTTCCTCCAGACGTGACCCTTTGCATTCAAGCCAAAG
AGTTCAGTATTGGTTTCATCAGACCAGAGAATCTTGTTTCTCATGGTCTGAGCTTCGTTAAGGGCCTTTTGGCAAACGGG
CTGTTATTTCCTTTTTTTGAGGAGTGGCTTCTGTCTGGCCACTACCATAAAGGCCTGATTGGTGGAGTGCTGCAGAGATG
GTTGTCCTTCTGGAAGGTTCTCCCATCTCCACAGACAAACTCTAGAGCACTGTCAGAGTGACCATCGGGTTCTGACCAAG
GCCCTTCTTCCCTGAATGCTCAGTTGGCCGGGCGGCCAGCGCTAAGAAGAGTCATGGTGGTTCCAAACTTCTTCCATTTA
AGAATGAGGGAAGCTACTGTGTTCTTGGGGACCTTCAATGCTGCAGAAATGTTTTGGTACCCTTCCCCAGATCTCTGCTT
CGACACAATCCTGTCTCGGAGCTCTACGGACAATTCCTTCGACCTCATGGCTTGGTTTTTGCTCTGACATGCACTGTCAA
CTGTGGGACCTTATATAGACAGGTGTGCTTTTCTAAATCATGTCCAATCAATTGAATTTACCACAGGTGGACTCCAAATC
AAGGAGTAGAAACATCTCAAGGATGATCAATGGAAACAGGATGCACCTGAGCTCAATTTTGAGTCTCATAGCAAAGGGTC
TGAATACTTGTGAAAATAAGGTATCTGTTTTTCATTTTTTATAAATTTGCAAACATTTCTAAAAACCTGTTTTCACTTTG
TCATTATGGGGTATTGTGTGTAGATTGCTGACGAATGTGAAAAAGGTCAAGGGGTCTGAATACTTTCCGAGGGCACTGTA
GGCTTCAGACAGGAGTTGACAGTCGGAAGAGCGAATTAAAATGGTAGGAGGGACATCCCTGCTGCAAGAGGTGTCAGATG
TCACATCCTGCTTCATACAGGCGAGATGAGACACTCCTGTGTCTGAGACTCAGGCCTACCTCTTCATGCAAACCATTTTT
GATCTACGGGTGTCCACAGGATCGATTTTATACGCAAATGGGTTTTATACGGGTTTACTGGGTAGCCTAAAATAAGATTT
AAACCATAAGATTAAAAACCTGTCCACCAAAAAAAAGATTTGCAAAAAACAAATTTACCTTCAAACTAGGGTGANNNNNN
NNNNNNNNNNNNNNNNNNNNNNNNNNNNNNNNNNNNNNNNNNNNNNNNNNNNNNNNNNNNNNNNNNNNNNNNNNNNNNNN
NNNNNNNNNNNNNNGGACAATGATAAAACACAAACACACATTAGATGATCATACGTTAAGTATTCAAGTCTCTTTGTCAG
TCTCTCAGTGTGCATACAGCACTAGAACTACCTGGTTCAGTTTTTGTTTTCATGGTCTATAAAAATTACACTAGTCTGAA
AACAGCTGCCCAGCAGACAACATGAGGACGGAAGCAAAGCTGGTGAGGTCAAGATGGAGTGAGAGACATCTTCTCTTGAG
AGTGTGAGAGAAAGAGAGAGCAAGACTAGCATGGGCAGGGCAGGGCAGACATGACATCCCCTCACTGTGGATTAGCATCA
AGGCGAGGAGCTCCCTTCCTCCTCTCCTTCCCCTCTTTCCTCCTCTCTCTCTCTCTGACGGAGTGGGGCTCTCCGTTAGG

ATACACAGACGAACATTGCCGTGTGTTTGATTTGAGTCAGGTTGCAGTCCACAACATAAGAGCAACAGCTGAGGGTCTGG
CCGTTAAGGTGCTCGGTAACGGGAGCTTGGTATACTACTAATAGCTTCACCAGACTCAGCTGATGGCACACACACACACA
CACACACACACACACACACACGCTGAAACGGTCAGACTGAGCATGCTCAGATCTATCACGGCGCTCTATCAGCTCTGGGT
CGGTTCCCAGCCCTGTTGGTGTTGTTTCCATGGCAACACCACATACCGTACATATGAGATATACGGTGGGTTGGATGGAT
GTTGATGATGATTCTGAGGAGGTCTGTGATATTGGCACTCCAGCAGTTAGCACATACCTGACCTAGATGAAGAGTCGAGA
CTGAGAAAACGGCCAAAGAAATGTTCACAAATTAGGCCCTGGCTGAGGCTAGTATTGTATTCTCTTACAGAGAATGCGTA
GGGCATAGATTTTTTTTTAAATTGGACCATACACTGTGCTAATAATCTGTTACTCCCAGCCTTTTTGATTTTGTTCACT
GTGAAACATCGTAATGATTCCGGAATGATTATCCCCCACCCTCAGGTGCTGTGCTGCTGTAACTACAGTCACTGTTGTTG
TACTCATGAAGCATGACTGTGCTGGACGCACACACACACACACACAACCAACTCTCAGCAGGGGTTGCTTAGCAACGCAG
TGTAGAGTTGGGGGTGTGTGGCGGGGCACTAATTCTGTCATTCTGAGCAAGCCCTTTCAAACTCAATTCTCGGCAAGCTT
ATGCTCGCTGACAGAGACTGTATGAAGCCACCAAAGGTTCTCAGTCACCTTTGACAGTCCATAACCGAGCGGCAGGTAGG
AAAGGGGTTTGCCGTGGCCAATGACAATACATCAGGGGTAAGCTTCTTTAGATGGTGTAATTTTTTTCCCTCGGATACCT
GGAGCTCAATAACTGTCAGTACAATGAACTTGGACCACAGTGCCCAGTCACAGGAAATATTCATGAAACCGTTTCCCCAA
ACTGTATTGTAGCCGAGGCTAAATCCCTGTTGGTATCAAACGGTGGCATACGTGTACATATGTTATAAAGATGATCTGC
CTGTTTACTAGCTTTATTAATAATGGATTGAAATCGCAAAAGCAACACATTGATGGATTGTCCCCAATCCTCCTGAATAG
ATGATGCATTTGACCCTGTAGAATATCACCTACTGTATTTAATGGCCATAGGTCATTACTGTAATGGGGTTTTTCCATTC
ATCTTATGTTCCACATTTTAGCTTGTTTTCTAGGCAGTTTGTAAAAGTTAACCTAGGTGGGATCTGTTATAAGCATTACT
CTGTCCAGACTAGGTGTCTTTCGTTCAGCTATTGAGTTGTCCCAGATGTTATTTGGTACCTGTGTGAATGTCAAGTATTG
CATTTGATCTGTGCACTTCAGACAGTAAGTAGTGAGCTTTGTTTATTGTTACCCAGGGGGTCTTTTGAGGCGGGTTGCTC
TCTCTGAGTGGTTATGATGGATTACACAGAACTCCAGTAGCTGTTGCTGGAATGTTCTGCTCACTGTCCGGAGGGAATAC
AGTTTGTATTGACCCATTTTCCACTCACACTCACAATACGTATAGATGAAATTATAACATGAATTTCTCGTTGCTTAGTT
TACCATTTGCTGTGTCCTATTTTGCAAAATGTTGGTGGTTGAATCCAGCCCAGTAGATTTGATATGGTGCCTGGTAGTAA
CCAAAGGCCTTGGGCAGCCAATGTGGAAGTCCATGTTTAACCTGTTTAGTTGTCATGTGTGCCCGTATTAACTAATAAAA
TAATACAAATTCATGAAGCATTTCCTACCTTCCAAGCCTATCGTTTTGGCTAACTGAGTGGCCTAGTAAGTACAATTTGA
AAGAATTTCTGTATTTGAGTACTCATGGAAATAATTGAGTGTTAGCGTGAAACATGTTAGCGTGAAACAGTGCTGAAGGC
AAAAAAAAGTTTGCGCTATGCTGGTTGTTCAGATACACAATTGAAAACCCAACTTAAAACATGTCCAATGCACTTCATAT
ACTCCTCAAAGAACACACACCTTACCCGAGTTTGAAATGTCAGTGCTTATAAATGTAACATAATAAATAGTCGCATTTGT
GAATTGAGTACTTGTATTCGAGTAATTAGTAAACGCAAACTTTAAAATCGAGTACTGTGTACGTGTGCCCATCCCTAGTT
ACTTTCAAGTTCATTTTCATAGACATTTCAAAGACTAGAGGAAAATAACCATACATTGACAAAGTGTGATGGGTTCATAT
CAACTTTGAAGAGGAATGTTTTCAGACTATGGACACAATATACTGTAATACTATAGATCTTACGAGCACCAAAAGTGAGC
CCAATTCCGTATGGCCCTTCTGCCTAGAGTAAGAGATGTTTTTGTTGTCTCTTTCAGCACCGTTCCTTTAGGCAGTTCAC
ATCTTAACTCTCAGACTACCAACGGCTAATGTCTCGGGCAGCAGCTTGAAGGCAGGTCATGTTGGTGTTGTGATGAATAC
TAAGAAGAGGAGATGACGGCAAGAAGCCATTGATTTGCGACACACACCTCAGTTAAAGAAGACACTCGTCCACCCCCTTG
ATGAGCCAAGACAACAGACTATGAATATTTTATGTGTCATCCATCTGGAAAGCAATAAATCCCAGCCCCCGGAACGATGG
CCTGTTTTTCCTCACTCGGCACTCCAAGCAAGTTACTCCATGATGAATGAAACATAAACAATGCTTAGATGGGAAACGTT
GCTGAAATGCGCTGCATTAGATTAAAGACAGACGTCTAAATGATCCCGTCACTGAATATGATTTTATTTGTACTGTAGTA
AATCTACAGGTCAATTTTATGCTGCTTTTAAAGCACACAATTTGGTTTTGCAGAGTTTGCACCACTAGAGCTCTTTCTGG
AAAAATCCACCTTGTCGAGTTCCCCTCATAATGGGGGAAGGAAGGAGAGGGTGAACATTCTGAAAGATTCTCAAGCCAAG
ACTCTCCCAACCTTGGAGTGAGATCTCTGCCAGCGTGTTCCATTGATGAGCTGCCAATCACCCTATCAAGGCTCCAAGCT
CCTTCCGATCCCCCACGCCCCAGAGCAGAATGGATTAACACTGCCCCTGTCCAAGTCAAGTCAACACATTATGCAGGGCC
AGAAGGCTATTCTCACAGAGACTCTCACCAGAGGAGCGGGATAAACAGTGCTAAGCGCACAGATATAATAAAATTGCAAG
AGCACACAAGCTGCAATATTCATATCAAAGTGCAAACGCAGAATTCTAACAATGAGATCAATAATTTTCCAATCAATTGC
TCAGACAAAAAAAAAAAAAAAAACGTATTTTTATCCATTCTGGTAAATTATTCCCTTTCAATTGTTATTAGATGGGCTTG
ATCTCATTCTGCACGTCTTGTAGCGGTGCGGTTCCTGTTGGAAACCGGGTATAAAAAGAGACAGCAGCCGCTTGCCAGTC
TTTGATGTTGTTCTGTTCAGAACCTGGGTGTTTACCACCTCAGCTGCAACCCTCCTAATTAGAGGAGCAGCAAGGCCGAC
GCCGTGAGAATCATCAAAAACCAGGCAGAACCAAAGCAGCGTTTCAAAGCAGCCGGGTCACAGAAATGAGCATGAATCAA
ATGTTAATAACTTATGTAACAGGGCCGGCATAATCAGTCCATATTTTTTGTTAGCTGCTGCTCTCTATGGCATACTTCAG
ACAGGGGGGTGATTGTATATGAAAGGAAGATGGGAAGATCTGAGGGACAGTGGATCTTTGCAGAGAAATGGGCTTTCATG
CTAAACATCGCTCTTTAGTCATCTCTTTCCCTCTCTTACCTGCTTTCTGGGTTAGCAGTATTCGGTCGTAGGCTCCCTCA
GAGTTCTCCAGCAGAGCCTTGCTGGTCTGGATCATCTGTCAGGAGAGGAGAAAGCAGACAGTCCAGTTCAACCTCTTTTT
CCCTTTACAGCAGCCTGGCATCCTGCGGTAGTGAGCAATCTTCATTTGTTTTGTTTTTGCAGAGTATGTATTCAGTGCGG
GGGAGAAAGGAGCCTAGGCCCTTATTACAGAGATAGAGGGCCAAGCAGCGGGAGTTGTGCTGAGGCACGGTAAAAAACAG
AGGGAGGAGAGTGTATTCGTGTATGTTATTGAAATTTGCAAGACAACTAGGGCATTGCATGTTACCATTATAAAATATGA
CGCTCCAGTCCTGTTTCAGAGTTGGTCTGAAACATGTGATTTCAAGAGTAGGTTATTATTGTTTATTACAGCAATCCGTT
GTAAATAGGAGAGGTCTTGTTCAGAGGCGTAACACAGCCTTATCTGTGGCATCACAGCTGGATAGCGGACCGCTAAATTT
AGCTCCCTGTCTTAATTGAGTGCACACTCTTCTGCCCTCCATGACTGTGTTGAAACTGTCATGGTGGCTCTGGTCACATA
TAGAGGCCCATTTGCACTTCTAATGCCTGGCCAAAAAATAAAACACCCCAAAATACATCCCTGGTGCATCGGGGGTATTT
CGGGAGGTTAATGACGCGTGTGTTTACTGTCATGGATCCTGGTAAATACCCACTGCACGACCCAGGAGACGTACACACGG
ATACGACCATCTGGATCTTTCTCCCTTATTACCGTCTCTCGGAGTGCAATTAATACGATCATCTGAAGGAAATGATATAA
CATGTAAAATTAGAATAGGCTTCTTAATGCGATTCTGGGGCGAGATGACACAATTACGGCAGCCTTCCGTTTCAANNNNN
NNNNNNNNNNNNNNNNNNNNNNNNNNNNNNNNNNNNNNNNNNNNNNNNNNNNNNNNNNNNNNNNNNNNNNNNNNNNNNNN
NNNNNNNNNNNNNNNNNNNNNNNNNNNNNNNNNNNNNNNNNNNNNNNNNNNNNNNNNNNNNNNNNNNNNNNNNNNNNNNN
NNNNNNNNNNNNNNNNNNNNNNNNNNNNNNNNNNNNNNNNNNNNNNNNNNNNNNNNNNNNNNNNNNNNNNNNNNNNNNNN
NNNNNNNNNNNNNNNNGGAGCTAAGGGGATCAGCAGCACTGTGACTGTGTGTCTGTGACAGCTTGTCCATGGTGGACTGC
TGGGCTGGGCGTTCCACTTCAGGCAGTGAAAATGGAGAACTCAAACAGTGACTCCAGCCCTCTACACCCAAGTGGGGATA
TTAGGAAATAAACCAATTAGAATATGTTCTTTGGTGATACATCAATTTGAATATTAGGAGTGATTTATACAATTATTTCA
AAGCCCTGACAATTAACAATATGGCCTATATATTTGAGGTGGGCATCAAACCTAGTTTTATGATGGCAATCCAGAGCATT
CCTACAGTCTGTGATGAGTGTAACACGTCGTGACTAAGCTGAGGGAGGCGTGTGGGGTGTTTGTGACAGTCCACAGCTGC
AGTAACAGGGCTGGAGGCTGTCAGGCACACTGATTACTTTACTAATGGGTGAAGAAATTACAGCCTTGGGTGACAGACAC

AGCAACACGGGGAGATATTTATAGTGGATTGAGAGGTAAGTGGCTGAACTCAACGCTGGCTTTGCTCGCTAAAGACTAGC
TGTGGTGATTTGTGTATTTAGTGGTGCTACTGGGAGTGTTTTACAAGGCTTCAAAATGGTAATAGTTATCATTAACAAAC
TAGTACTTTGTGACACATTGGTACTTCCCTACATTTTTTCTAGAAAAATCGACACAATACATTTACAACACGAAGAACAG
CACATTCAATTAAAGATAATTTCTGCAGAGGAGCACTGTGATGATCCTGGAAGAGTCCACATTTACTTTTCCTCTGCAAA
TAAAACAAGTAATGAATAGAAAAACCAGACAACCCCATAGTAGAATTGTGTATATCTATTTACCTAGTCGGCTTGACGTT
GTGTGTTCTATGAGAGAAATGTTCATCTCAAAGCGCATTCAAGTTATGAGTGCCACAGGGACGGAAACGTGCAATCTGAC
AAGCAGTCAATGCACAACAATTCTTAATTCAATAGTGGGGGAAAATCAAAGTTTTAAATGGCATTTGTTAAAAAGAGGTG
GATCCCGGCTTTCCATTCATACTTATTTATTTCTGAAATGCACCATTTTAAACCCAGAGTTTTTAGCAGCGGCATGGTTG
AGCACGATACCGCTCCGCAATTCACCTCGAGTGCACTGGGAGAGTGGACCAAACGTAACACGGGTCAATTGTAGGGTAAC
TTGGAGTGACCTAAATTGTGATGAGACAGATTACATACAGTTGAAGTCAGAAGTTTACATACACTTATGTTGGAGTCATT
AAAACTCATTTTTCAACCACTCCACAAATTTCTTGTTAAACTATAGTTCTGGCAAGTCGGTTAGGACATCTACTTTGTGC
ATGACAAGTAATGTTTCCAACAATTGTTTACAGACAGATTATTTCACTTATAATTCACTGTATCACAATTCCAGTGGGTC
AGAAGTTCACACACTGAGTTGACTGTGCCTTTAAACAGCTTGGAAAATTCCAGAAAATGATGTCATTTAGAAGCTTCTGA
TAGGCTAATTGACATCATTTGAGTCAATTGGAGGTGTACCTGTGGATGCATTTCAAGGCCTACCTTCAAACTCAGTGCCT
CTTTGCTTGACATCATGTAAAAATCCAAAGAAAATCAGCCAAGACCTCAGAAAAAAAAAAATTGTAGACCTCTACAAGTCT
GGTTCATCTTTGGGAGCAATTTCCAAACACCTGAAGGTACCACGTTCATCTGTACAAACAATAGTACCCAAGTATAAGCA
CCATGGGACCACGCAGCAGTCATACCGCTCAGGAAGGAGATGCGTTCTGTCTCCTAGACATGAACGTACTTTGGTGCGAA
AAATGCAAATCAATCCCAGAACAACAACAAAGGACCTTGTGAAGATGCTGGAGGAAACAGGTAGAAGGGTATCTATATCC
ACAGTAAAATGAGTCCTATGCCGACATAACCTGAAAGGCCACTCAGTAAGGAAGAACTGCTCCAAAACTGCCATAAAAAA
GCCAGACGGTTTGCAACTGCACATGGGGACAAAGATAGTACTTTTTGTAGAAATGTCCACTGGTCTGATGAAACAAAAAT
ATAACTGTTTAGCCATAATGACCATCGTTATGTTTGGAGGAAAAAGGGGGAGACTTGCGAGCCGAAGAACACCATCCCAA
CCGTGAAGCACGGGGGTGGCAGCATCATGTTGTAGGGGCTCTTTCCTCCAGGAGGGACTGGTGCACTTCACAAAATAGAT
GGCATCATGAGGAGGAAGACTATGTGGATATATTGAAGCAACATCTCAAGACATCAGTCAGGAAGTTAAAGCTTGGTTAC
AAATGGGTCTTCCAAATGGACAATGACCCCAAGCATACTTCCAAAGTTGTGGCAATTAAGGACAACAAAGTCAAGGTATT
GGAGTGGCCATCACAAAGCCCTGACCTCAATCCTATAGAACATTTGTGGGCTGAACTGAAAAATTGTGTGCGAGCAAGGA
GGCCTACAAACCTGACTCAGTTACACCAGCTCTGTCAGGAGGAATGGGCTAAAATCCACCGTACTTGTGAAAGGCTACCC
AAAACGTTTGACCTAAGTTAAACAATTTAAAGGCAATGCTACCAAATACTAACTGAGTGTATGTAAACTTCTGACCCACT
AGGAATGTGATGACAGAAATAAAAGCTGAAATAAATCATTCTCTACTATTATTCTGACATTTCACATTCTTAAAATAAAG
TGGTGATCCTAACTGACATAAGACAGGGAATATTTACTAGGATTAAATGTCAGAAATTGTGAAAAACAGTTTAAATGTAT
TTGGCTAAGGTTTATGTAAACTTATGACTTCAACTGTATTTGGTTGAGCAAGAATAGTGGCAACCAGGGAAAATGTTGGG
GGAATAAAATGTAACATGAAGTGGTTTCTGTGAGAAGCACAGGCAGGGGGCATGTCAGCGTTATTGTATTTATTCATTTT
GGAGTAGAATGTCTTTTTAAAAAGTCACCCAAACTGAGCTTCTCAGTCCTTCTACATAAAATGTACACGACTTGATCTTC
TTTCACTTCATATTTTCATCAGAATTGCTGAGGACACACAAGAGTCATTGTCTAACACTTAACTCATGATCCAAACTTCA
CTGACGCCTGCCCATGACTCTCAATGAGAGACACTTTTGAGTAAAGTTACACTACTCATCCGTTGGCAGGTTAGTTTGCA
CATTACCTTCATGCTCTGTGTGGGGGTTCTAAAGTCAAGAGAATGAGACATTATGAATACATTAGGTAAATTACATGCAA
TGCTATGCTAATGGTGTACTAATGCAGTCTGTAGCCTTTGACCTGTGTTCTACAGTGAATAATAAGCTTCACCAATACTT
CTCCAGGATTTATGCTTTGTGCACAGCACACTGCAAACATGTCTGACACAGAAAACAGCTGGTGTCAAATATAAATATCA
AAGTATGAATATTTTAAAGCGTATTCAATACGTATTCAGAGGAAATATGCTAGATTTGAGGATGAAATATCAGATGCTCC
TAAAGTGTTTTTCCATGGCCACGTTGGATAACCTAGCAGAGCTTTCATGTTGTTTTTATCTTACTGGCTGTATTGTAGCA
AATACAGTAGATAAGTAATGAAATATTCAAAATGGGGGACAAAGGTAATCCTATAACACTCTTGTATTATTAAGGTACAT
TGATTACGGTGAGGTGTAAGGCGTAATAGGTCTGTATTGAAATAGCCTCAATGACACATTATAAACTGGGTGTTTCCAGC
CCTGAATGCTGATTGGCTGACAGCCGTGGTATATGAGACCGTATACCATGGGTATGACAAAACAGTTATTTTTGCTGTTC
TAATTACGTTGGTAACCAGTTTATAATAGCAATAAGGCACCTCGGGAGTTTGTGGTATATGGCCAGTATACCACGGCTAA
GGTCTGTATCCAGGCACTCTGCGATGTGTCGTGCATAAGAACAGCCCATAGCCGTGGTATATTGGCCATATACAACAGCC
CCTCATGCCTTATTGGTTAATTATATAACTTCAGTCCATTGTCAACATTGATTAAATGACTTCATAAAAACAACCCACCC
CTGCCCTACTACCTAACCCTCTTACCCCCATGTTACCATTCAAATCCCACTAAACCCTCCTCTTGACTTGATTAGAGTCA
AACTCATTTTCTAGGTGTTTTGAGGTTGTTTTTACAACCAGCACATTAGTAGTGGTCTAAAATCCCAGATTTCAGGTCCT
GGTCCTAAAAAAAAGAACGTCCTCTATCAACTAACCTTATAAAACAAAGAAAAAATAACCAAACATGATACTGTTCAAAC
AAAAGTCCTCCTGTACTCACCATGTCATAGGTGGTGACCACCTTCTTCAGGACATCTGGTAGCTGGCCCTGGGGCTCCAT
GGTGGGGTACTGGTCCTTGAGGTTGAACACCAGGAGGGGGTTGTTGTCAGCCCCCGTCAGCCGGGAGGACAGGGCCAGGA
TGCACTGCTTCAGGTTGGCCACCGCCGAGAGGCCACACAGTTCCTTGAAGGGCACTATGGCATTCTGCAAAGGAACATGA
ACGCTTGGGTTGGTGTCCTGATGATGAACTAAAGACTTAGCATGTTATTGTATTGAAGACAAAGATCACTGGACAAAGTG
GAAGATAAAAAACAACTCAAGCTTTCATAAATGGACAAGATGCAGAATATTGTACTGCAGACTAGGGATGTGACAAATTA
AACAATTTTGTTAAAACGATAAGAAACATTCATCAAATTCCATGTGAATTCACAATGCAGATGTGCTGCTGCCAGCAACG
GTTTGGGTCTCACGGTGCGTCCCTTTCAGATGGTTAAGCATTAATGTACCTCAATTCCACCTCGCAATGTTTGCATTTCC
TTCTCAAAGTATTGCCATACAGGACTTCGCTGCTGTCGCTTGCCTTTCTCAGCCATTTTTCCAACTGTTAACGACAATGA
CGTAGTGGTGGAGAGTAGACTTCAAAATAACGTGTCGACTCCCATTTCTGAGTGTCCAGATTCAATTCCACTAGGAATCG
ACTCCTAAGATTGAGTATTTTGGGAATGGAGGAGACTGATGAGTTGCCGTACTTCCGAAAACACTTGCATCTTTCATAGT
GAGCTAACGAGGGACGGAGAGAGAGGGGAGGGGCACAGTACAGGCAGGTCGACCAACAGGCAGACAGAGTCAGAACTGTG
CACTTGGCCTAGGCTATTTATCGGCAATCAAAAGCTCTATCTCGCAATGCAATATCTCGCAATTTCTTGGCCTGCAATGT
CTCAACTTCAGTAAAGCAGGGCCTATCATCAATGAATAGGCTAGGATATTGAGATGAGAGAGATGCAACACGTCACTCTC
GCACTGTATCTGCACATATGCATGGGTTCACTTCACGCTGTTCACAGCGTGGTAGCCAGGGCAGCAAAACAGTAGTGAAG
TGCCTCGTGCTTCAAAGCTCTTAGTTGTTGAGGAAATTGACCAACTATGCTGTTTACTTTCTGGATCTACATAATTTCTT
TAACGTTAAAACATTCACACCCCTATTGCAGACAAAGTTGCAAAACAGTATTAGCCTCAAGGGTACTAGGGCATGAATGA
CAAGTGAATTATTACAGTTCAGAGTTCCCATTTGTTTTCATTTCAGTTTGGTTTTTTGTCATTTAAGATAAAGCTAACTG
CTGTGACAAAGTTCACTTGAGTAATTATCTCATTCAGCTGGTTCATTATGAATGAAGATCAGTGATTAGATGATTCCTAA
TGTTTTCAATTATCTAACGTAGAAAATACCAACTGAGCTCTGTTAAGTTACCTAAGTTAATGTGGCCTATGTAGTTACTA
ATTAACAGGCGAGAAGTACAAGTACAGAGAAACACCCTGTGATAGGAAGTGGTTACGAGTACTTCTTACCTCCAAGAACC
TTTGTTGGTTTTTGTTAGTGATTCGATCTCACACACACACACACACACACACACACACACACACACACACACACACACAC

ACACACACACACACACACACACACACACACACACACACACACACACACACACACACACACACACACACACACACACACACACAC
ACACACACACACACACACACACACACACACACACACACACACACACACACACACACACACATTCCTGTGAAAGGCCCAATTT
AAACAGTGTGTAAACAACCGGGAGGACAAACAGCCACAGAGAGAACGCCCAAGATGTGTTAGTCTCAGGGACAGTGGCCT
GTTATCACATAATGACTGCAATGTAAACAGAGCGACCAAAATAAACCCTAATACACAATGCATAATGCACCACAAAAGAC
CAACCACACAGTGGGGGAACAATCGACTATGCAACTTCCTTCTGGCACATTTCTCAGTATTGCTTCAGACCAGAAAAGAC
CAGCTGTTTGAAGTAGGCTAGCTCATGGGGGGCCCAGTGGGAACCTAGTGAGCACACAGGAATGCACAAAAGCAGATGTA
GGTGGATAGTCTGAACAGTCAAATTGTCATGTGGGTAGTGGGTAACAATAGCCTACTGTTCATCTTTTGGTCGATATATC
TTTCAGTTATGTCCTGTTTCAAGCAATAAAAGATTGATCATTGCTGCTAACAGTTTACTAAGTGCAAATTTGAAGTAAAA
CACCATATCCGTTTTTTGATATTTTGAAAATTGGTGACGCACTAGTCTCGTTTCCACTCCACTTCGACATAATTTGTGGT
AATCTGTCCTTTAGACAGCATACAGTAATCTCCTTCTAGAATTATTACTGACAAGCCCATAAGCGCCACAGTGCAGTATT
ACGTAACACGTTTTCCATTTTCTATATTAGAAGGTATGCTCATAAGCCACTACCTGGATATCTGAATCAACCCCAGGCCT
CAGTGCTCCAGTGGGAATGTCACCAGTCTGGCTCTGAGCTTCGAAGTCTCCTTGAGTCTGTCTCGACTGTTGTGCAATAC
ACATGGATTAGATCAAATCTGATTAAATCAAGTTTGGTGGCTTTGTTTTCTCTTGCCAAAAAGGCCTTTATTTTAGATGC
TACACAGTCTTTGTAGTAAATCTCACTGTGATCCCCGCTTGACACTAGGGGTCAGTGTTCATATTCTAACAAAAACATTC
CTATGTAGGCGGGTGAGTAAATAAACTTGGATTCAAACGAGTCATCCACGACTTTATTAAAGCATGCACATTCCCCCCAA
AATATGACATAATGTAGGCTAGCCTGGCCAATAGAAACTAGGAATAAATTGGTGATGTCTCACAAGTCAATCCTTAGGGA
TGGATCGAAATTTACACCATGGTTACTCAGGCCTACCTGTCTGACAGACGAAATTAGGCTGTAGGCGGCAATAGCCATAG
ATTTGTCAGTCAATTCCTCCACCCATCATGCATTCTTTAAATATCCTACCCTGTGTCAGTGAAGGGCTGTTAAGTTAAAA
CCAACACTCGAATTGAGAGGATATCTATTATTTTTTTAAATGGCAAAAAGCACAGGCCTACCCCTTGTTAGCTTAAGATT
TTGAAGAAAATTAAATGGGATCCGATTATATAAAGTGATCGATAACAGTGCTTTGGTCCGCAATGTTTTATGCTAGAAAC
AAGCTGTAAAATACCCAGGAAAGACGTGACTCCGATGCATCCTTTCTTTGACATTAAGAGGCTGAGCTACAACCTTCTAT
AGCCGATGAGACAAAACAGACTTTGATTGGGAAGTAATCTAATTCTGTCACTATCAATCGATTAAGCTGTTCACCATGTA
CAATAGAAGATGATTAAACCCAGACAGCTTTTAGGGAAACACTTCTGACCATCTCTTGTTGGGTTAAGCCACAGAAGAAA
AGTAGCCAGCAGTTAAAGCTTACATTTGTATGTGTCGGTAGACCTACTCTGTCTGGGGTGGAAAGGTAGGCCTACTCTGT
CTGGGGTGGAAAGGTAGGCCTACTCTGTCTGGGGTGGAAAGGTAGGCCTACTCTGTCTGGGGTGGAAAGGTAGGCCTAAC
TTTTAAAAGAACCACGATCAGATTCCTAATCATTTGCAAACAGGGACAGCTTTGTTGCAAACCAAGTACACCGATTTGGC
ATTGGAGAAATATGATAAGATGAACACATAAGCTATCGCTCAGTCCATTCTTAGCCTGCAATTTTGGTAATTTGTGTGGT
ATTAAAAAAGATTATGTAGAATTGTAGAATTGCCTGACATTTTTATAAAAAGGAAAATAAAATCTCAGACCTGCAAATAC
AATAATAGATTCATGCAATGCTTTTACTATAAAGGAGATCTTTCCACTTCTACTGTTGTCCATGGTGGTCTGTGAGCCCA
CAACTTTCTGGCCCTCAGCCCTGTGCGCTATAAAGAATTCCTGCGTTGCCATGTGATGCTCTGGTCAGTCCAAGACGTGA
GGGTAAACAGTAGACATGTTCTCTGCTATCCACTTTGTTTTAATTACTATTTTGGGTATAGGGGATGGTCACTTATAAAA
AATAAAAAATTACGTTCAGTAAGGGATTAGGTCAAATATATTTTTCATTTCAGAGAGGTCCGATTTTCTCCATGTAACCC
TTATTACAAATAATGTTCACTCCCTTACCTGCATGTTTTCTCTGAGGTCGGTGGCTTCCCTCAGTGGCAGTATGGCTGTC
TTGAAGATGTCGTCCACAGCCTTGATGACCAGCACTCTCATGGTGGCATACTCCCGACTTCTCTTGCCCTTACGCACAGA
CAGTCCCCTCTTGTGAGGCTTGCCCCCGCCCCGTCGGTTGGTGATGGCCACATGGACGAATAGCCAGGCGTGGGGAAGGA
CTTCCCCCGTTAGCGATTGTAACGGTATGTGCCGGAATCCTGGCTGGAGACACTCGAAGGGGATTGTGTACTGGCCGATG
AACTCGTCGCCAATGTAGTCATCATCAAGCACCACGAAGCGCACCATGGCCAACTCAGGTAGGTTTATCTGAAACTCAAA
GCTTTCGTCAAACAGTGGGTTCTCCCCATTCTGATTGACAGTTTTAGTCCTTTGCTCTGCACAATCAGCAGGGATGCCAT
GGATCTCCACATAGACATAAGGGTCAACAACATCTCCTTTGGAGCCTGAGCCTTTGGGTTTGGGGAAGTTTTGTCCACTT
ATGATCTTTATGTGCAAGAGCTGAGGAGACACACCAGGTACTGAGTCTTTGGTGTTGGCACTGAAATATGACACCTGCTC
CCTCATTATGGCTGGTCGCAGCACATAACCACAGTTCCCATTCTGACGGAACCAGCCAATGTTTAGGTCCATCATCAGGC
CGGGGGTCTGGTAGTTCATTGCCACGATCTGGCAACCCACACTTCCAGAAGTCCTGAGGGTTCATGTTACTGGAGTCGATC
CGCATTGGGCTGGGGTAGACTCTCGCCAATAATTTCTTGTTGTAGTTAACAAAGTCGCCTGGGAAGTCATTGGCACAGCG
ACTGGCAAAGACCTCATTGAACGAGCAAAGTTCCCAGTGCTTCTGGCTTTGGAAAGCTGTTGGGAAGTCTTTGAACGCAA
TCGATTTACACAACGTTACCAGGTCAGAGAGGTCCTTGGACAGCTGGAACTTTTTCAGTGGGATGCTCTGTTGTTCGTCG
GCTTCAATGTTCATTCTTTGGGACATCTCAGCCCCCTCATCCTCATCAGTCACTTCGCCCCTCTGAGCTAATGCAAGTCGT
GCCCAGCTTCTTCCCCTTCAGCAGGATCTTACACTTGAGGTCAAAGGGTGAGGGCAGGTAGCTGTCCTCAGGTGAAGGTG
GATCCATGTAAATCTTATCACCAAGAATCTTTTTCAAGTGCTGAAACATGACCTTCTGCTGCTTTAAGGAGCAGTGGTTC
TCCAGACACAATATCAGGGGAAAGTCAGATGCAACAAAGGCATATTTGTTGATGATGTCACAGACACTGCAGAAGACTAT
CTGCGAGGTCATTGTGTGGCCAGTGTAAATGACAGGCTCATTATCTGGTCCATCCCAGACATCTAGTTCTACACTACGAC
AGCCCATTTTGAGGGCACGGATGTACCCTGTAATGTCAGAGGGGCCTCTAAACTGATCCTCGATCAGGTATGTGTTGTGG
GAAGCGTTGATGTAATAGTGGGACAAGGGCTGGTTCATGTCCTGACACACTGTTTTTTGTTCAGGGTCAAAGATGTGGCA
CTCTGGCGACATAAGATAATTGGTAAACCCATCAAGGGAGAGCCAGCCCTTGAGCTGCCCCTCTTTGGAAGGTTCATACC
TCTGAATGACCTCCAGACTGGTGTCTTCACTTACTTGTGCCATACCCTGCTCAGCCTCCAGAAACATAGTTAAGTCCTTG
GTATCTAGAAATTCCTTGTTGCTAGAGAACTGGACAAGAAGGAAATACATTTCTGGTCTTGTGCAAAGGTCATGAAAGAC
TTCAATGAACTCCTCCTTAGTCACACCACATCCCACCGTATCTTTAGATTTGTGAAACTCTTTAAACTTGAGTTCAATCT
TAACGTTTTTGAGTCCAGGGTTTAGGTTTTTAATTAGCTGCACGGCAGCACATAGACTGATGTGCTTGCTGTTGTTTGCA
TCAGCCTGCTCAAAGAGGTCACCGAGCCAGGAGGAGCGCATGTTGTTCTGGTTGCTCTCAATCATATTCAGTGTGTGTTT
CCCATAGGATATCAGGTATCTAAGCCCGGTCACCCATATGTTGGCCATATCGGCAGAGTTTGCAACCAAGTCCAAAGACT
CATAGTTCTCCCCATAGATGATTGAGAAGGCACAGTCCTCTGATATCTGATCATATATTCCGTTGGTCCTAAACGTTTCT
GTGTTTTTCCCTGTCCGCACCTCTTTGATGGACTTGACATCAATTTTGGCTTTCTCTGACTCTTTTTTGGAGGGTTCCCA
CCTTAGAGACTGCATGTCCGCGTCCAGGAGGAAGTAGCGGTGGTAAACGCGGGAGTTGGAGCGGACCTTCTTCAGTTCAG
AGCCGTCCACCATGGCGTTGATGCAGTCGCTCGCACTGCTGATCTTCTTCTCAGTGGGCATGCTGCTGAACGAAACCGTC
TTCTTCCTCTCTTTGTGCAGCCTCGAGCCATCCTGAGGAAGAGAAAAGCAAAAACCTGAATACCTGTTCTGTAAACCAGT
AGAAAATAAGTAAATCACTTTAGATTTAGAGGTAAAAAGAGGGGCTGAATACTCTTTCAATCACTTCTCTGAGCTGGAAG
CAAAATATTTAATTTATGAGATATACTATAGGCCAACTCAAAAAGTGGGAGTATTCCTGACTGTATGACAAAGCATTTGC
GCATGTGTCTGCCTATTGAAATGGGTTGATTATTTCACTACCATTTGCTATGAAATCTGTTTAACAACTTAGCATTCCTT
GATGATTGGAGCTGCCTAATGTCAGTGTTTGACTTCTAACTGCAATTCTTTCGGAACAGCAGGTAGCCTAGTGGTTAGAG
TGTTGGGCCAGTGACCAAAAGGTTGCTGGATCGAATCCCCGAGCTGACAAGGTAAAAAAAATAAAAAAATCTGTCATTCT

GCCCCTGAACAAGACAGTTGGCCTACAGTGGGTTGTCAGTGTAAATAATTATTTGTTCTTAACTGACTAGCTTAGTCAAA
TAAAGGTTAAATGGAAAAAATAAAAACATTTAAAATTGTGAGCAATCTCTATTAAACAGTATTTTACCTTTCAGGCTTAA
CCCCAGAGAAACTATGTATTAACTAAGCACAGTTATGATTCATTAGTTTCAGAAGGACCAAAAAAAATTGATAATTTCTT
AAAATAGCCACGAAGAGCATTGTGAAGTATAAAACATAATGTTCAAATACTGGGCATAATGCAGTGGTATGGTAGCCTGC
TAATTCTCTTTGTCCATGTTGCTGCAGGCCTTTATGGTCTAGAGGTCATCAATGTTTCCTTTTCAGCCCTACCTCTCAGA
GCCCAGAGGGAGAGAACTATCGAACTAAACTTGGCTCAATAAAATAAAATAATTAAAACATTCCTGTGAAATGCGTGCTG
TTAAATTCTTGACTCTGGGGAAGCAAGTCACACTCCGATCAAAGAGGAAGCGGCCAGTATATGGGATATAATCAGTTGGG
ATAGGCAGTAGGGATGGGGATTTGAAATAATTTCACTATATGAATAGTATCATGAATTTTTTTCATTTATTTGGATATTC
TAAATACGTGTACGTTTTTAACCTGAGCACTGCTGCTGCGCGAGGGAGAGAGGCTCAAACAACATTTTATTGGTTGTGTA
CACAGATTTGCAGATGTTATCGTAGGTGCAGCGGAATGCTTGCGTTTCTAGCTCCAACAGTGCAGTAATACCTAGAAATA
AAAAATATAAAAAAGACAATCCAGAAAAATAAATATATCAGAACGAGCAATGTCAGAGACTGGAATATTAAGAAACTGGA
GAGGAGAAAGAAAGAGAGAGAGCGAGAGACATCTCGGCTACAGCCAAAACTAGCTAACTTGTAGCAGCCACAATATTATT
TATCATCCCATACAACAGTACCTGTCTTGACTGGAGTAGCCTAGAGAAGCTATAGTTAGCTAGGCTAAATGCCGGGCTTT
CTCCCCAACCCCATTCCGATAAAACAATATCCTACTTGTTGGTTGCTCGTTGTAGCCTACTCCTACTCCTTCTCATTTTG
CAAACTGTTAATTCCATAATTGTATTCCATCTTGCATTGAATTAGTGAACTCTCACTCCACTTGCTACATGTTGAGCCTC
TTTGCCACTTCGATTGGCCAACATAAACAACAAGCTACACATGATACTTGAAGGCTACCGGTCGGCTACCTGTCTTTTTA
AATGGGGCGCAGGCAGCACACCATTTTAAGATGAATTACATCCTCGAAGGAATGTTGCCAAGCTATCCTAGTGTCTAAAT
ATTTGACCTTGGATAAGTGCTTCAAACTGAAACTATAGGCTGTAGAGAGCTTAAAATAACTTTAGGGGGAACATAAACAA
TTGTTAAAAGAGCATGTTTCAGCATGGGTTAATTAATGCATCATAATTTCTCACTCTAATATACAGTATCTACTGCTGTA
CATATCATTCTTAGTATATCTTGTTTAAATTCATCAAGTGTACATGCATATAAATTGCATTTGGATTACTGTTACAGTGC
TATTCAGGTTCGTTCCGACATTTGTTGATATCTTGTTTGTTTTTTGGAGGATTATTTGACATTTTACTGCATTGTTAGGC
GCTAGTAACATAAGCATTTCACTGAACCCACTATTACATCTGCTAAACTGTGTACGCATCCAATAAACTGGTTTGATGAG
ATGCTAACAAAGAGGTCAAAGCAAAGTTGAGAGCAGCATACCATTTATGGTGCGTCACATGATGTGAAAAACACTGGTTG
TTGACATATCTTGTGTATACGTCTTTCCTAACTCCCCCCAACCCCTTCTATTTTTCTTGAGAACTGAGGACCGTTACCTT
GAAGGATCTCCTTCAACAATTGTGTTGCTCGACTTAAGGTGCCTGTCACAGTGCATTATGGGTGAACCACAATGAGTCCC
CCCCCTAAAAAGGATCTAGTAGTGTATTGGCCACGTGCGTGACATTTCCCAAGTAGTCCGTGTCATTGTGTTCCAGCACC
TTTATTTTCAACCTTTCCACATTCAAGCTGAACTAAATAAAACATGAATTCTGCATCAATTGACTAGCATCAGGTGAAGC
CTTCAATGCATGCTGAAAGTGCCTGACTTATGAATGAATAGATTCCATTTCCATTTGCTGATGAAAAGATTCATGACATT
ACTAAGCGGTTCCAATTTGTTTTCACCCAATATTTTGGATGCCTTTTTTTAAGGCCAATTGGAAGTTTAAAATGGGCATT
CAAAATACATAGACAAGCGTGCTGCTGGTTCTCGTGTAAGTTGTTTATACTGAAATAAACAGCATGCATATGGCGCTATA
GATGTGGGGAAGCCTTCAGCGCCCATCGTCCTGCAACGAACGCACTGCCATTCCTGATCAAATGGCCTGCGATGTTCAAT
GTCCCAAGGCCTGCCATGTGATCTTGTCCACGCTTGTGTGCACTTACTAAGAGTAATGGCTCTCATGAAAGTCTATGTGC
TTCACCCCCAGGGCAAAGTCCATTGAAAATGTATTACCTTTGGGTCAAGAAAAATCTGACTACCCTTTTTCTAGACAGAG
TTGAATGACAGACATTAACTGCTGAAAATGTAAGTTTATGATTGACTATGAGCACCACCAAATATATACGGAACAAAAAA
TATAAAACGCAACATGTAAAGTGTTGGTCCCATGTTTCATGAGCCAAAACAAAAGATCCCAGAAATTTCCCATACACACA
AGCTTATTTCGCTCAGATTTTGTGCACAAATTTGTTTATATCCCTGTGAGCATTTCTCCTTTGCCAAGATAATCCATCCA
CCTGCCAGGTGTGGCATATCAAGAAGCTGATTAAACAGCATGATCATTACACAGGTTCGTCACACAACACAATGCCACAG
ATGTCTCAAGTTGAGGGAGTGTGCAATTGGCATGCTGACTGCAGGAATGTCCACCAGAGCTGTTGCCAGAGAATTTAATA
TTAATTTCTCTACCATAAGTTGCCTCCAATGTCGTTTTAGAGAATTTGGTAGTACGTCCAACCGGCCTTACAACCGCAGA
CCAAGTGTATGACGTTGTGTGACGAGCGGTTTGCCGATGTCAACTTTATGAACAGAGTGCCCCATGCTGGCGGTTGGGTT
GTGGTATGGGCAGGCATAAGCTACAGATTGTATCAATGGTAATTTGAATGCACAGAGATACCGTGAAGAGATCCTGAGGC
CCATTGTTAGAATACTTAGAACATGTGAACAACTACAAATACCTAGGTGTCTGGTTAGACTGTAAACCCGCCTTCCAGAC
TCACATCAAACGTCTCCAATCCAAAGTTAAATCTAGAATTGGCTTCCTATTTCACAACAAAGCATCTTTCACTCATGCTG
CCAAACATACCCTGGTAAAACTGACCATCCTATCGATCCTCGACTTCGGCGATGTCATTTACAAAATAGCCTCCAACACT
CTACTCAACAAGCTGGATGCAGTCTATCACAGTGCCATCCGTTTTGTCACCAAAGCTCCATATACTACCCACCACTGCGA
CCTGTACGCTCTCGTTGGCTGGCCCTCACTTCATACTCGTCGCCAAACCCACTGGCTCCAGGTCATCATCATGCTAGGTA
AAGTTCCCCCTTATCTCAGCTCGCTGGTCACCATAGCAGCACCCACCTGTAGCACGCGCTCCAGCAGGTAGGTAGGTATA
TATATATATATATATATATATATATATATATATATATATATATATATATCTCTGATCACCCCCAAAGCCAATTCCT
CCTTCGGCCGCCTCTCCTTCTAGTTCTCTGCTGCCAATGATTGAAACGAACTACAAAAATCTCTAAAACTGGAAACACTT
ATCTCCCTCACTAGCTTGAAGCACCAGCTGTCAGAGCAGCTCACAGATTACTGCAGCTGTACATAGGCCATCTATAATTT
AGCCCAAACAACTACCTCTTCCCCTACTGTATTTATTTATTTATTTATTTTGCTCCCCATTATTTCTACTTTGCACTTTC
TTCCACCGCAAATCTACCATTCCAGTGTTTTACTTGCTATATTGTATTTACTTCGCCACCATGGCCTTTTTTGCCTTTAC
CTCCCTTATCTCACCTCATTTACTCACTTTGCAGCAGGTAGCCTAGTGGTTAGAGCATTGGACTAGTAACCGAAAGGTTG
CTACATCGAATCCCCGAGCTGACAAGGTTAAAATATGTCGTTCTGCCCCTGAACAAGGCAGTTAACCCACTGTTCCTAGG
CCGTCATTGAAAATAAGAATTTGTTCTTAACTGACTTGCCTAGTTAAATAAAGGTTAAAAAAATAATAATAATAAATGTA
AAACACTTATTTTTCTACTATATTATTGACTGTATGTTTGTTTTACTCCATGTGTAACTCTGTGTTGTTGTATGTGTCAA
ACTGCTTGCTTTATCTTGGCCAGGTCGCAATTGTAAATGAGAACTTGTTCTCAACTTGCCTACCTGGTTAAATAAAGGTG
AAATAAAAATAAATAAATACAATTGTTGTGCCATTCATCACCACCATCACCTTATGTTTCAGCATGATAATGCACAGCCA
TATGTCACAAGGATCTGTACACAATTCCTGGAAGCTGAAATGTCCCAGTTCTTCCATGGCCAGCATACTCACCACACATC
ACCCACTGAGCAGGTTTGGGATGCTCTGGATAGACGTGTACGACAGCGTGTTCCAGTTTCCGCCAATTTCCAACAACTTT
GCACAGCCACTGAAGAGTGGGACAACATTCCACAATCAAGTCTGATCAACTCTATGCAAAGGAGATTTGTCACGTTGTGA
GGCAATTGGTGGTCACACCAGATACTGACTGGTTTTCTGATCCACACCCTTACCTTTGTTAAGGTGTCTGTGACCAACTG
ATTCATATCTATATTCCCAGTCATGTGAAATCCATAGATTAGGGCCTAATAAATTCATTTCAAATGACTGATTTCCTTAT
ATGAAGTGTAACTCAACAAAATCTTTGCATTCTTTGCATTTATATATTTTTTGACAATGTATTCCTATCAACCATGTTGA
ATGCCAAGCTAGTTTGAGTAGAAATTCTAACATGCTTTGCTGATGAAGAAAATTAGAATCACATTTTTGTCAAAACAGTT
GCATAGCCTAGAGTTTCAGTTGATTTCGTTGTAACACTTGAGGTGTGGACACCAAATCTATTATGCTATAAGATTTAGCT
AAATAAGAATCACAGGCTGTGAGTACACTACAGACTTCATAGCCTACAAACAATAGTAGGCAAGGTCAAATATTTTCTTT
AGTACGGTATATGAAAGTGTTGACCCGTCTGATGCTGTGGTTCCTCATCCTGGTGAAAGAATAGCAACAGCATCCTGACA
TTTTATCTATTCTAGTCGGCTACTTCCTGCCCCTCATTCTCATCCTTGAGGTTGAAATGAGACAAAAGGGAAGGCCACAT

TGAGCCATGAATGCACTTTAAAAGACAGACAAGCCTCTCTGCAGGCAGGGCTAGAGCTTTTCTTTCATTCTATAGTCTGC
CGGTGTACACAGAGGATTAAAAAAGGTACAACTCAAAGGATTTCATGTTCAACACAGTGCTGGGAGACATGTTCAGAAAT
ATAGGACTTCGTCACAGAGAAATGAAAAGATCCTCCAATACACTCTATAGGCCTACAACTCAATTGTATGACCGGATTCC
AAAACGTAAACCATTGTAAACATGTCTGGGAGGTATGGTCCACAGATTGTAGCCTATGAATCTTCTACCTTTGAGAAAGC
TTCAGTATTTCATATGAATAAGTGGCGATTGTGATGAAGAACTACTGGTGAGCTATTTTGTTTAACACCATTACTGGCAG
AGGTCTAAACTTCACTGTAACCCCCACTCGTCTGGTCTTGTAGATAAATGAAATCAGCACATCTTTCTTTTACAACCTAT
ACATTAGCACATCTTTACACGTTATACATTAGCACATCTTTACACGTTATACATTAGCACATCTTTACACGTTATACATT
GCATAACAGCTCATCATAGCTGTTTTAACAAAGCCAGTCAGAGAGAAATTAGTTGAAAGAAGAAAAAAAAAAAAAAAAAA
AAAAGAATATCCTGTCATTATAAATTTGGCTTGGCTTTGTGCGTAAAAAAAAAAAAAAGAAGGAGGACTGTTGCATCACTA
TTTGTTTCAAAACGCCAAATTCTAGATTGAGAAGCGCACTATAAAAATATTGGCCAACACTGCATTAGATTTAAACAAAC
TCATCCACAAGCATATGCTGTGGATAAGGAAAAAACATTAATACAGTAGATTATCGCAATATTTTGCGATTTTATATTGA
AACTGTGACGCCAAGTATCGATTTGTAATGTCAGCGATCGCTAGTCAGTTGTACCTGCCCCAAAACTCTGGTATATCATC
CTATAACTAGTTCCCCATATTTTTCATTGTTTTAAATGGTGAGCCAACATGTTTTGTGTACTTTTACCTCTCATGGCTTT
CTACTGTCCCTCTGCAGCAGACATGTAGTGAGCAATGTGTTTGAAACATCAAATCACAATAAAATCACAGTATCGAATCA
CAACACATAGAACTGTGATACATAGAATCGCAACACATCGGCACCTAAGTATTGTGATAATATCTACAGTGAGGGAAAAA
GGTATATGATCCCCTGCTGATTTTGTACGTTTGCCCACTGACAAAGAAATTATCAGTCTATAAATTTAATGGTAGGTTTA
TATGAACAGTGAGAGACAGAATAACAACCAACAAAACAAGAAAAACACATGTCAAAAATGTTATAAATTGATTTGCATTT
TAATGAGGGAAATAAGTATTTGACCCCTCTGCAAAACATGACTTAGTACTTGGTGGCAAAACCCTTGTTGGCAATCACAG
AGGTCAGACGTTTCTTGTAGTTGGCCACCAGGTTTGCACACATCTCAGGAGGGATTTTGTCCCACTCCTCTTTGCAGATC
TTCTCCAAGTCATTAAGGTTTCGAGGCTGACGTTTGGCAACTCGAACCTTCAGCTCCCTCCACAGATTTTCTATGGGATT
AAGGTCTGGAGACTGGCTAGGCCACTCCAGGACCTTAATGTGCTTCTTCTTGAGCCACTCCTTTGTTGCCTTGGCCATGT
GTTTTGGGTCATTGTCATGCTGGAATACCCATCCACGACCCATTTTCAATGCGCTGGCTGAGGGAAGGAGGTTCTCACCC
AAGATTTTACGGTACATGGCCCCGTCCATCGTCCCTTTGATGCGGTGAAATTGTCCTGTCCCCTTAGCAGAAAAACACCC
CCAAAGCATAATGTTTCCACCTCCATGTTTGACGGTGGGGATGGTGTACTTGGGGTCATAGGCAGCATTCCTCCTCCAAA
CACGGCGAGTTGAGTTGATGCCAAAGAGCTCCATTTTGGTCTCATTTGACCACAACACTTTCACCCGGTTGTCCTCTGAA
TCATTCAGATGTTCATTGGCAAACTTCAGACGGGCATGTATACGTGCTTTCTTGAGAAGGGGGACCTTGCGGGCGCTGCA
GGATTTCAGTCCTTCACGGCGTAGTGTGTTACCAATTGTTTTCTTGGTGACTATGGTCCCAGCTGCCTTAAGATCATTGA
CAAGACCCTCCCGTGTATTTCTGGGCTGATTCGTCACCGTTCTCATGATCATTGCAACTCCACAAGGTGAGATCTTGCAT
GGAGCCCCAGGCCGAGGGAGATTGACAGTTCTTTTGTGTTTCTTCCATTTGCGAGTAATCGCACCAACTGTTGTCACCTT
CTCACCAAGCTGCTTGGCGATGGTCTTATAGCCCATTCCAGCCTTGTGTAGGTCTACAATCTTGTCCCTGACATCCTTGG
AGAGCTCTTTGGTCTTGGCCATGGTGGAGAGTTTGGAATCTGATTGATTGATTGCTTCTGTGGACAGGTGTCTTAATACA
GGTAACAAGCTGAGATTAGGAGCACTCCCTTTAAGAGTGTGCTCCTAATCGCAGATCGTTACCTGTATAAAAGACACCTG
GGAGCAGGAAATCTTTCTGATTGAGAGGGGGTCAAATACTCATTTCCCTCATTAAAATGCAAAACAATTTATAACATTTT
TGACATGGGTTTTTCTGGATTTGTGTTGCTGTTACTCTGTCTCCACTGTTCAAATAAACCTACCATTACAATTATAGACC
GATCATTTCTTTGTCAGTGGGCAAACGTACAAAATCAGCAGGGGATCAAATACTTTTTTCCCTCACTGTAAATCATGAGG
TCCCTGGGAATTCCCAGCCCTAGCTAGGAGAAACATTGTCTCGACATGAATAAACTCTTTAAAATGTTACTCGTGCCTTC
AATTCTTTTGGCATTTCCAGGAAAAGCCTTTATTTAACTAGGCAAGTCAGTTAAGAACAAATTCTTATTTACAATGATGA
CCTACCCCGGCCGAACCCTAACGACGCTGGGCCAATTGTGCGCTGCCCTATGGAACTCCCAATCCTGGCCGGTTGTGATA
CAGCCTGGAATCGAACCAGGGTCTGTAGAGCCCCAAGACATCTTTTGAGAAGGAAAGGAAAAGTGGTAGTTTTTGCTCTT
GGCAACTAGGATCATAGTCGTACATCCCGGCTAGCCTGGTTGTCATAAATGGTTAGTAACATTATAGCTTTGATACATCA
GGGCCTTTACATCTGCAAAATCTTAAAAGTCAAGCTTATCAGCAGGCAAAGTCTGTGTGGAATTTACGAGTTAGATTTAA
CCTAGTCTACAAATGCAGGTGAAAGTCTCAACCATGACATTATTCAGGGTTTGGCAGCATGAAGCCATGAAAACTGTATT
ATTCCTTGAAGCCATGGCATTCCAGAGACACATTCATCTCTTGTATCAACACGTCACATCTGGTAACAGGACTCTGGCCA
TGTTAATCGAACACTTTCTCACCCTTGCTCTGTGGTGTAATTGAAGAGAAGGCAGGCTGTACTTTAATGGGTACTAATCA
ATCTCAGGGCCCCACCACTTCATTTTTACCCCCTGCAGAGCCTAACCAGAGTTTCACATACATCTCTGTGCTGTGAGGAA
GGATCTAATCTAAAAGTAAATTATTCAAAATAAGTACATCACTCCAGACAGAAGTGCTTCTGGAGGAGCTTTCCTGAGGC
TTAGAAAAAGCCAAAATGCATGACTGAAGATAATCCATGTGATCTGGACAAGAATTTTGACTTTATCTTTGGAAGGAGTT
TTCATTCGTGTTGGCCTTTAGGTGTGGCTGCTAATCAGGATTTCCTCTTGCGCTCAGTGAGGACTGGCTTCAGCTCCCCA
AGCAAGAAAACGATACCACAGAACCCCCCCCCCCCCTTCCTGAAAATATAATTCTGCGTCTTATGTAATGGCCAAAGTC
AGCAGGGTGTTTTTAGTCACTTACACATGATTTCATTAAGACTCAAAATTATCCTGAAAGAGAAACATTTCTTGATGCTA
AAAGGAAATCCTCAAGAACATACTTTTTAGGGATGATAACGACAACCATACAATCTCCAATGTTTCAATTTAGCCTACTT
GCTCAGCAGAGATTCTTACTTTATTACCAGCATACTGGTCCACAATCCATTTCATTAATAGATTTTAACTGGATATTAAA
AATGTCCAATTTCTGAAGCAACACAAAATCAGTGTTTTGTTATTCTACGATGATGCTGTATCCAGACAATCCCAGGAGTC
TGCTGATCTGAGGTGCTGTCGTAATTAAGAATGACACAGGCCCAATTATCATCACGCGTAGGCCTGCGTTGGTAAAGCAA
CCATGCTGAGAGGTCCACAACTGACTCACTCTTGCGTGTTGGACAATTTGTGGAATCCAAATAGGTCATAAGTTGCAGAC
GCCAACCTCACCAGTGTATGAGAAATGATATTGAAATTCTCCCTTTATTTCCAGATACTGGATGTCCTGATTTCCTTTGC
AAATGTTTAGGCCTAATACATAGGCAGGTCAGATTCTGCAAAGGTCTACACCTGGAGTTCTTGTCACTTTGCACATAGAG
ATCGATTGTGGTTTTACTTTTCCCAGAAATACCCAGAGACATGATGTTCGATAAATTTAATAACAATAGGCCTAGCTAAA
CCTGTGCAGGGCTTAGCGGCATGGGGAGATTTGGAGAGTATTTTTGTCTCCCTGCATTACATTTGAGTGTGTTCAGCTGC
CAAAAACTCCCGTAGGATTCTGAATTCCCTAATAGACTGAAACCCCCTTTACTGGTTCAATGAGCAGTTCAGGACTGTCT
GTACGTCTGAAGAATTCACAGGGGACCTATTACAGTCCAGACCAAGTGCATTGTATAGAGCATCACACAAGAGACCAACC
ACGACTGCTGCATAATGTGATCATCAGTATTAGTCTACATCTATGCTAGGCTAAATACTATAGAATTCTCAACACCGATA
TTTGACTTTATAAATTATCATTCAAATGATAATGTTACCAAGGGACATTCAACAGCACTAAACTCCAGAAGTGCAGATAG
GTTGGCAGCTAAATGTGTAATCTGAAATCTTATTATAGCATAAAGGGGATATTGCTCATCATTTTGTCCCTTATCAGGCT
CTCGTCTCAATAACATCAAACGCTCAAAAGGGAAAAGAGCATTGGCTTTGATACCTGAGCCCAGACTGAACTTTCCCCCG
ACTACAATGTGAAGAAGCCTCCCTATGTGGAGAAAGCTCGTGCCCCCCACTCTGTGACTGCAACCTCATCTACCACTCTG
ACCTCCAAACACCAGGGTTCATTCACATCCACAGAGCAGATTGAGACCATAGTCACATTTTAAAGAGCACATCTGAAAAC
AGCCATTAGGAGTCAGCCATGCTGGCTTAGGTTTTACTATATAAAAGGGTAATTTAGATGCATTAATAGGCTAGGAGTCA
CAGCAAATATCATATATGTTTATGGTAGGCTATTTCCAGGGTTGACCACATTGGCAAACAGTGCTTTCAAGGATATTTCA

TATTTAGGAACATGGTGGGTGTTGGATGTGGGAAACATGGAGTAAAAGCCTGTTGTAGGTGGAAACTATGGACTGATGCT
GGATGTGGCTGTTATACCACTGAGGAACCTGACAAATGCCCAGCTGTTCACGTTGTAATTTGGTATCAGACCTGAGTCAA
ATCAACCATAAAATAGATAACTAGCATGGGAGCCATGGAGAGGATTTCCTGAATTATGTGTGCCTGAACTGNNNNNNNNN
NNNNNNNNNNNNNNNNNNNNNNNNNNNNNNNNNNNNNNNNNNNNNNNNNNNNNNNNNNNNNNNNNNNNNNNNNNNNNNNN
NNNNNNNNNNNNNNNNNNNNNNNNNNNNNNNNNNNNNNNNNNNNNNNNNNNNNNNNNNNNNNNNNNNNNNNNNNNNNNNN
NNNNNNNNNNNNNNNNNNNNNNNNNNNNNNNNNNNNNNNNNNNNNNNNNNNNNNNNNNNNNNNNNNNNNNNNNNNNNNNN
NNNNNNNNNNNNNNNNNNNNNNNNNNNNNNNNNNNNNNNNNNNNNNNNNNNNNNNNNNNNNNNNNNNNNNNNNNNNNNNN
NNNNNNNNNNNNNNNNNNNNNNNNNNNNNNNNNNNNNNNNNNNNNNNNNNNNNNNNNNNNNNNNNNNNNNNNNNNNNNNN
NNNNNNNNNNNNNNNNNNNNNNNNNNNNNNNNNNNNNNNNNNNNNNNNNNNNNNNNNNNNNNNNNNNNNNNNNNNNNNNN
NNNNNNNNNNNNNNNNNNNNNNNNNNNNNNNNNNNNNNNNNNNNNNNNNNNNNNNNNNNNNNNNNNNNNNNNNNNNNNNN
NNNNNNNNNNNNNNNNNNNNNNNNNNNNNNNNNNNNNNNNNNNNNNNNNNNNNNNNNNNNNNNNNNNNNNNNNNNNNNNN
NNNNNNNNNNNNNNNNNNNNNNNNNNNNNNNNNNNNNNNNNNNNNNNNNNNNNNNNNNNNNNNNNNNNNNNNNNNNNNNN
NNNNNNNNNNNNNNNNNNNNNNNNNNNNNNNNNNNNNNNNNNNNNNNNNNNNNNNNNNNNNNNNNNNNNNNNNNNNNNNN
NNNNNNNNNNNNNNNNNNNNNNNNNNNNNNNNNNNNNNNNNNNNNNNNNNNNNNNNNNNNNNNNNNNNNNNNNNNNNNNN
NNNNNNNNNNNNNNNNNNNNNNNNNNNNNNNNNNNNNNNNNNNNNNNNNNNNNNNNNNNNNNNNNNNNNNNNNNNNNNNN
NNNNNNNNNNNNNNNNNNNNNNNNNNNNNNNNNNNNNNNNNNNNNNNNNNNNNNNNNNNNNNNNNNNNNNNNNNNNNNNN
NNNNNNNNNNNNNNNNNNNNNNNNNNNNNNNNNNNNNNNNNNNNNNNNNNNNNNNNNNNNNNNNNNNNNNNNNNNNNNNN
NNNNNNNNNNNNNNNNNNNNNNNNNNNNNNNNNNNNNNNNNNNNNNNNNNNNNNNNNNNNNNNNNNNNNNNNNNNNNNNN
NNNNNNNNNNNNNNNNNNNNNNNNNNNNNNNNNNNNNNNNNNNNNNGGTCCCGAGGAGAGTTTGGGAAACACTGCACTAC
ACCAATCACTTCTTATTTCATAACGAACATACAGCTTTAGGAAAGCAGAATGACAAAGGGCCAAGCTGGGTTACCAATCA
GACAGGCTTTACAAAACAGAGACAGGCAGTCATAAGAGAACTGCTGCAGCAACAGGACGTCGATAGAGCCCCTGAAGCTA
CCCAAATCCACATTGATTGATTTAGCTCCAGTCAAGCCCCGATGCACTCCTCACACAGAGTGTGATTTGAGTGATGCAAT
CAATGGATAATACATCACTAGTGTGTGAGGAAGCTAAGCAACTTCAGACACTGTCACAGCTATTTTACCAAGAGGTTTGA
GTCCTTTAATCCATTTTTTTTCACATTAGAAAACATGTCCCCCGTCCATTTTATTATTGTTTATTAAAATAGCCAATATT
AAATCCGCCATCTTGATTTTGTTTAATTTGAGGGACCTAGGATTTTTAAGCTCAATTCCTGCAATTCTTTGTAGTTTAAC
AGGACTCATTACATATTTTAGGTAGGCTATTTAATGATGATCCTAATAATAAATAAGGAAAACAAAGTCTAGACCCTTTT
TCCCCAAATTTTATTCCGCTACCAAATATATTTTATTATAATTTATATGAACCCTCAATTTAATTATACATATTATTTTA
CAGAAAGTGAATAGATCGACTGATAGCGACAGAGTCACACATTGTATAGATGACTTCACAAGGAAAGTAAATCTATATAT
ATTTAGAAGGTCGTAGCTCAGCTTCTGAACATCAGACACAAACCAAAGACAACCGCTTGTTTCAAGCCTAAAGCGTTGCA
GAGATATGAATCGTTGTAGATCAGTTTATACAAATGATCATTTAAAACATGTTTTACCCCTCAAATCAAGGAAAGTCCTG
TGACCCACCAGGAAACTTTGGCGACCACCGGTGGGGGGCGAGACCCATATAAGGGCCTGACATTAACTATTTTAGATACA
AAAATCACTCATCCAAAAAGGACAAGTAGCACAATTTTTACATCACTGTAAGATGCAAGGAACCACTTCACAAAATGAAA
TGCATTACTATATTTTTTAAGATAGAGAATCAGACAAAAATGGAGGCTACACTCTGCCTACTGGCTTCTTTGCATATCCC
AGCCTGTCTCAAAATACAACACAACCCTTTTAAAGGCTCTCCTGAATGATGGTTGGCCACCCTTGGTAGCCTATAAAATA
TTGCAACATTACAATTAAAACCAAATACTTTTTATGCCTTGATAAAATAAGTCCCTCCAGGGATTGAAATTGGAGGCAAT
AAAATAATATGTATTTGGTTCAAAACAGGCTCTGGGGACAGTTCTGGGAGGACAGATCAGATCGTTTAGCTTAAGAAATG
TTGATGAGTTTTATATTTTCATATTATAAAGCACAACAGTGCACACATGGCTGTAGGCTACACGCGAATGTTCCAAAATG
CAATTAGAGGGAAGACGCCATTCTCAAAAGCTATGACTAGGATTATGCCTTTGGCTGCTGGACAATAAAAATTAAAAAAA
TGATTACATTTTTTTTTTTTAAAGAACATGAGAAAAATGCTGGCTTCAATAGCATGTTAAGTGTTTATAAATGTTGAGGG
AATTATTTTTTATCAAGGTCGAATTTGAAACAAGGTAATACATGCTTCATAAAATGACAAACATCCAGGTTTTAAACAAT
TATGATTAAATCGTTTTAAAATGCTGACCGCCTCCGCTCATATTTAAGTTGTCCCAGACAAGAGGACAAGCGTTAATGTC
GAGCCCTGCCCTAGTTGGGAACCCCTGCTTTAAATCTGACCAGAACCACATTTGTATTAAACTACAATGTGATGAGAAAC
AATAGCCAATATGGGAAATGAAAATGTACAATATCAAAATCACATCAGTTATAACATTGCTTTATGATGAGAAAGTGTGA
CCACTTTCATATTCAAAAACAGTATGGGACGTAAGGCAAAATGTGCAGTCATCGTATTGCATAGGGTAACGCTAGTTAGA
GGCAGTTTCGTTAGCCAATGCTAACTTCCTTCATACTGGACAGACAAAAATTGTATCCACGATTAATCTGACTCCTACCA
AAATCCCAAAGTATCACTTTAAAGAGGCAAGCAGCAATAAATCATAGGTGTCATGTCACACAATTTATATCCATGAAGGA
GAGTGAGCAGCTTCAATATAGTTCAGCATCTAATAAGGCATCTTTAATGAGACTAGCCTAACTCATCCTGCTGGAAGGCT
TCTTAACATCACAGTACTGCACTGTGAATAAAATTGGGCCCTACTGCTTAGTTTTTGCCAAGGGTATCAAGATGCCCAAA
GCCTGCATAAAATGTATTGTGATTACTGTTTTGCCGATTTAATTGGCTGCATAGCTGGAAGAGCCCCAAGGTCCAACAGG
AGAAGAGGACGGTGCTGGGGCAGAGGAAATGTGGTCAGGTCAAAGAAATCCATATGCCAATCATGAACAAATTAGGATGT
GAGAAGAGAAATCTTGTGAGATCAATAATTGTACTAAAGTTGTAGAGTACAATGCCAACGCGTCGTGACAGTGACTGCCT
TTCTAGCGTTCAATCACAGATCAAAAGATGCCGGCCGTGTTTGAGATCGACTAGCCTACATTGCAGTCGATGACTGCAGA
CTGAGCAAAATGTATGCTATCGAATAGTTATGTCATATCCTGGGCCAACTCTTGAAGATGGCAGGGGTATGGCAGGGGTC
AGTAACAGGTGTCCCGTGGGTAAAAACCGGCCTGTGAGTGATTTCCCCACCCCTGCAAAGATTTTTGTAAAAAAAAAAAA
ACAAGTCTAATAAAGGATTTCATTTTTTCTGCAAAAAATATTTTTTTTTTTTTTTTTTTAAATTCACCAGGAATTCAGT
AAACACTTCTACTAAGTATTCCCACAAATAAAAAGAGATGTGATCATGTCAATATAAAATATTTACAAAATTATTATTTT
CAAATATAATATATTTTTGGGCTTACTTTTTGTACATTTTGCAATGTACAAATAATTATAATTCTCTTCCGGACCCCGGA
ACATCCGCTCTGACAAAAATCAGACCACAGCTGGATCTAGTTGCTAATTCCATACCACTACACAATTCAAACAGACTTGT
GTTTAAAGTGAACAGGTGCTAAGATCTGTTAATAATTGAAGGTGTCAGAAATGTGTATACCTTTGTTATCAGGCTCTTAT
GCACTTCCCTTCAGTTAATGGTGTCTACTACAAACTTTAACCCTAGTAAAGCCACTTGAGTACTGCCAAGTCATCTGTGG
TCCCCCTGAAATAACAGGTTATAGCCGGTAGCTCTCCGTCAAGTCCCCAGCCCTGTAAATGAGTGACATCTTAGTGGATG
AATGAATAAATGGTACTGCAGGCTTGGTAAAGTGTAGTGAGTCGTCTGGAACAAATACAGCACAGTGAGCAACTTGAGAC
AGCACCCAGACCAGGAATGGTGACAGAAGATCTTTAATCAACGACAAAAAAAATACAGCGTTGGCTGATGCCATTGGATC
CCAACCAGAGCATGGCATTTTGGTAAATTAAGCAACACTCAAACATTGGACTTGGCACACAATATTTTAACTTAAAAGGT
AATGCCATAAGTCCTCATGGTGGATAACAGCATGTGGTTGGGTAAAATGCCAGTGTAATCCTGGTGTGGTTAAGCTGGTG
CTGAGAGACGGTACACACAAAAAAAAAAAAAAAAATGGCAGTAAGCCTGACTTTCCAATTTTCGAAAGGAAGTTGGTACA
TGGCGGATGGACCGTCTTCCAGTGGTGTGTATTCGTGGATGTGAAGGATAGCCAGACTTCGCCCAAAAAAATTACCAAGA
AAGAAAACAAAGCAAATAATTTAGAATCTTTCATCTCTGTGTTTCATAATTTTCCTTCAAATCACAGGAGGCTGAATGTA
TCTGAGAAAGTATCCGAATGATCGAAACAGCACCCCTCTGTCTCTGTAGGCCATCTATCTGATGCGGTCTTGGCTGAAAG

CGTATGACATTGTTGCAGCCCGTAGCATTGAATGCTAGGAAAGCCAGCAAGTATTTGGCCTCTCTTGATACATTTTTTTA
AATAAAATAATAGCCAATCAGTGTGAGTTCAACTGTGAATGGTCCTGGCGCACCCCCAATTTAAAAAATAAATGTTTAAA
AAATGTTTTAAAAAAAGAAATAAAAAAGTGTCGATGGAAGCCAGTTTGGGTTTGGCGTCACTCCTATCAAATCGCATTGA
GAGCAAACGTCATTGACAGAAACAACTTGAATTGTTGTGTTGTCCTCCGGTGGAAAGCTAGCTAAAATTGTCCCTTTCCT
AAATTAGCCATGGAAATAGGGATTTGGACTTACGGTATTTTATTAGGATCCCCATTAGCTGTTGCAAAAGCAGCAGCTAC
AGTTCCTGGGGTCCACACAAAACATAAAACAGAATGACATAATACAGAACATCATTAGACAAGAACAGAACTACATACAT
TTTTAAAAAGGCACACATAGTTTGTGAATGCATTGATATGTAGGCTATCTAGGTCAAATAGGGGAGAAGCGTTGTGCCGC
GAGGTGTTGCTTTATCTGTTTTTGAAACCATTTTTGCTGTTTATTTGAGCAATATGAGATGGAAGGAAGTTCCATGCAAA
ACTGTACGTATTCTTGAATTTGTTCTGGATTTTGGGACTGTGAAAAAACCCCTGGTGGCATAAGTGTGTGTGTCAGAGCT
GTGTGTAAGTTGACAAGGCAAACAATTTGGCATTTTCAACACATTAATGTTTCTTATAAAAAGAAGAAGTGATGCAGTCA
GTCTCTCCTCAACTCTTAGTCAAGATAGACTGGCATGCATAGTATTTATTATCAGCCCTCTGATTACAATTAAGAGCAAA
ACGTGCCGCTCTGTTCTGGGCCAGCTGCAGCTTAACTAGGTCTTTCCTTGCCGCACTGGACCACACAACTGGACAATAAT
CAAGATTAGACTAAATTAAAGCTTGCAGAACTTGCTTTTTGGAGTGTGGCGTCAAAAAAGCAGAGCATCCCTTTATTACG
GCTAGACCTCTCCCCATCTTTACAACCATTGAATCTATATGTTTTGACCATGACAGTTTACAATCTAAGGTAACACCAAG
TAATTTAGTCTCCTCAACTTGTTTAACAGCCACACCAGTTTTTTCCCCATCATTCTTTAGATCATTGATCTTGGCTTCAT
AATAAAGTTTGTTCTTTTTGTTGAGTTTAGTCCCATCATTTCTCAATTTGCAGTAAGTAAGCCAGTCAGATGTACAGCCA
GACTTATTAGCTACTCCTTTTGCCCCATCTCTTTCAACTGGTCAATCCATGGACCCTTAACAGTTCTAACAGTCAGTTTA
ACAGGTGCATATTTATCAATAATTGGAAGAAGCAAATTCATAAAGTGCAGTGTCTGGATGCTCCTCAAATCACATCAGAC
CAACAAATATTATCATCCACATAAGAGATACAGCAAAATCTTTTGTATGATCTCTTATACACTATTTTAGGCCCAGCTGT
TGGAACTTTGGCTTTCCTGGATATAGCCACTATATGGTGATCACTGCATCCAATGGGTATGGATGTAGCTTTAGAACAAA
GTTCTACAGCATTAGTAAAAATGTGATCGATACATGTGGATGATCTTGTTCTTGTAGTGTTTGTAAACACCCTGGTACGT
TGATTAATAACCTAAACCAGATTACAGGCACTGGTAACAGTAAGAAGCCTCCTCTTGAGTGGACAGCTTGATGAAAACCA
GTCAATATTCAGGTCCCCAAGAAAGTAGACCTCTCTGTTTACATCACATACACTATCAAGCATTTCACACATATTATTTA
GATACTGACTGTTAGCACTTGGTGGCCTATAGCAACACCCCAAAAGAAAAGGCTTTATATGTACAAAGTGAACCTGCAAC
CACAACACTTCAATAACACTTGACATAAGATCTTCTCTAAGCATTACAGAGATATGGCTCTGAATATATACAGCAACTCC
TACCCCCATACGCATTTCTGTCTCTTCGATAAATGTTATATCCTTGTATTGCTACTGATGTATCATCAAATGAATTATCG
AAGTGAGTCTCAGAAATGGCTAATATATGAATGTTATCTGATGTTAGCAAGTTATTGATTTCATGAACCTTATTTCTAAG
GCTACGTATATTAATATGGGCTATTTTCAGCCCTTTCCTGGGTAGCTTATCAGAGATAGACATAATATTGAAAAGAGCAA
ACAAAGCAAGATAAAAAAAAATATACAGTTGAAGTAGGAAGTTTACATACACTTAGGTTGGAGTCATTAAAACTCGTTTT
TCAACCACTCCACAAATTTCTTGTTAACAAACTATAGTTTTGGCAAGTCAGTTAGGACATCTACTTTGTGCATGACAGAA
GTAATTTTTCCAACAATTGTTTACAGACAGATTATTTCACTTATAGTTCACTGTATCACAATTCCAGTGGGTCAGAAGTT
TACATACACTACGTTGACTGTGCCTTTAAACAGCTTGGAAAATTCCAGAAAATGATGTTATGGCTTTAGAAGCTTCTGAT
AGGCTAATTGACATAATTTGAGTCAATTGGAGGTGTACCTGTGGATGTATTTCAAGGCCTACCTTCAAACTCCGTGCCTC
TTTGCTTGAGATCATGGGAAAACAAAAAGAAAATCAGCCAAGACCTCAAAAAAAACATTTAAAATCTGGTTCATACTTGG
GAGCAATTTCCAAATGCCTGAGGGTACCACATCCATCTGAACAAATAATAGTACGCAAGTATAAACACCATGAGACCACG
CAGCCGTCATACCGCTCAGAAAGGAGACGCGTTCTGTCTCCTAGAGATGAACCATTTGGCCATAATGACCATCGTTATGT
TTGGAGGAAAAAGGGTGAGGCTTGCAAACCGAAGAACACCATCCCAACCTTGAAGCACGGGGATGGCAGCATCATGTTGT
GGGGGTGCTTTGCTGCAGGAGGGACTGGTGCACTTCACAAAATAGATGGCATCATGAGGCAGGAAATTATGTGGATATAT
TGAAATCAACATCTCAAGACATCAGTCAGGAAGTTAAAGCTTGGTCGCAAATGGGTCTTCCAAATGTACAATGACCCCAA
GCATACTTCCAAAGTTGTGGCAACATGGCTTAAGGACAACAAAGTCAAGGTATTGGAGTGGCTATCACAAAGAACTGACC
TCAATCCTATAGAAAATTTGTGGGCAGAACTGCGTGTGTGAGCAAGGAGGCCCACAAACCTGACTCAGTTACACCAGCTC
GGTCAGGTGGAATGGGCCAAAATTCACCCAACTTATTGTGGGAAGCTTGTGGAAGGCTACCCAAAACGTTTGACCCAAGT
TAAACAATTTAACTTCTCTAGGCTAGGGGGCGGTATTTTCACGTCCAGATGAAAAGCGTGCCCAAAGTAAACTGCCTGCT
ACTCAGGCCCAGAAGCTAGGATATGCATTTTATTAGTAGGTTTGGATAGAAAACACTCTGAAGTTTCTAAAACTGTTTGA
ATGATGTCTGTGAGTATAACAGAACTACTTTTAAACAACAAATTGCATAGCGCTCTCATTCACGCACTTTTAAACACTAC
AGCCAAAATGGGAGCCACCTAGAAAAACTACAATTTCTGGCTAATTTTTCCAAAAACCAGCCTGAAACTCTTTCTAAAGA
CNNNNNNNNNNNNNNNNNNNNNNNNNNNNNNNNNNNNNNNNNNNNNNNNNNNNNNNNNNNNNNNNNNNNNNNNNNNNNNN
NNNNNNNNNNNNNNNNNNNNNNNNNNNNNNNNNNNNNNNNNNNNNNNNNNNNNNNNNNNNNNNNNNNNNNNNNNNNNNNN
NNNNNNNNNNNNNNNNNNNNNNNNNNNNNNNNNNNNNNNNNNNNNNNNNNNNNNNNNNNNNNNNNNNNNNNNNNNNNNNN
NNNNNNNNNNNNNNNNNNNNNNNNNNNNNNNNNNNNNNNNNNNNNNNNNNNNNNNNNNNNNNNNNNNNNNNNNNNNNNNN
NNNNNNNNNNNNNNNNNNNNNNNNNNNNNNNNNNNNNNNNNNNNNNNNNNNNNNNNNNNNNNNNNNNNNNNNNNNNNNNN
NNNNNNNNNNNNNNNNNNNNNNNNNNNNNNNNAAGGGTGACAGCCATTGTAAATAGTGGTATGCTAAATTCTATGGGGTG
GGAGGGTATGGTTAGTCCTCGGGGTGTCGCCTGCCATATCAGTTCGGATATACTCACAGACATTTTAACAGTTTTAGAAA
CTCTAGTGTTTTCTATCCAAATCTACCATATATGCATATCCTAGCTTCTGGGCGTGAGTAACAGGCGGTTTACTTTGGGC
ACGCTTTTCATCCGGACGTGAAAATACTGCCCCCTACCCAAGAGAGGTTAAGCCATTTTGCCACAACTTTGGAAGTATGC
GTCGGGTTATTGTCCATTTGGAAGACCCATTTGCGACCAAGCTTTAACTTTCTGACCCATGTCTTGAGATGTTGCTTCAA
CATCATTTTCCTGCCTCATGCTGCCATCTATTTTGTGAAGTGCACCAGTCCCTCCTGCAGCAAAGAACCCCCACAACATG
ATGCTGCAACCCCTGTGCTTCACGGTTGGGATGGCGTTCTTCACCTTACAAGCCTCCCTCTTTTTCCTCCAAACATAACG
ATGGTCATTATGGCCAAACAGTTCTATTTTTGTTTCATCAGACCAGAGGACCTTTCTCCAAAAAGTACAATATTTGTCCC
CATGTGCAGTTGCAAACCGTAGTCTGGCTTTTTTATGTCAATTTTGGAGCAGTGGTTTCTTTGAAGGTAGGCCTTGAAAT
ACATCCACAGGTACACCTCCAATTGACTCAAATGATGTCAATTAGCCTATCAGAAGCTTCTAAAGCCATAACATCATTTT
CTGGAATTTTCCAAGCTGTTTAAAGGCAAAGTCAACGTAGCGTATGTAAACTTCTGACCCACTGGAATTGTGATACAGTG
AAATAATCTGTCTGTAAACAATTGTTGGAAAAATGAATTATGTCATGCACAAAGTAGATGTCCTAACCGACTTGCCAAAA
CTATAGTTTGTTAACAATCAAATTTGTGGAGTGGTTGAAAAATGAGTTTTAATGACTCCAACCTAAGTGTATGTAAACTT
CCGATTTCAACTGTAGGCTGTAATATTGCTTTTTTTCTGGCTTCCCCAGTGATTATACCCACGCACCACTACTGCGGTCT
TCCCTAAAAACGTACTACTGCTCCCCTGATGAAAAACGGACCTATGACCAAGTAGCTTGGCATGAAAATGGGTTGGCTTT
CGAACTTTCTATTTGCCAACACATATTTTCCGGCTTTTCACTGACAGCAAATGTTGCGCAGCATACTAGGCCTAGCTTCT
TTCTTTTTAACAGAGGCAAAATATTGCCGCAGTGCTTAGTTCCTAACATTCTTGCAGCCATTGATATAGCATGGTGAAAT
ATGAATTACAAAAAATAAGATTAACAGTGGTGCACAGTGCATGTGTGACAATTATGTATGCCTACCTGCATCCAATGGCC

GATTACTGAAATAGCAATCATAAAATAGCCTTCCCAATGATAAGTAATTATAGGATTAATGACGCATTGCTCCTGCAATC
CATTTGATCCATGTTCACTTCATAGCTTTTAAGGCTGACCCCATTAAGTCGACTGGTCGATTGTTTGGTCGATAGGCTGT
TGGTCGACCAAGATTTCTTTTTAGTCAAGCAGTGGAAAATAAAATAAAAATATGTATGGCACACGAGACACTAGTCTGAT
TCACGCCTGTCTGAGTGGACTAATCCATTGCAGAGGCCGCAGGGATGGCACACCAGTATCACCAGTAGTACATTTACCAT
TAATTCCCACTATTTCTAATCTACAATGTTTGTTTGGTTAAGGTAATTTCTGTTAATGCATTCAATATATTATCACTACA
GTCTTACCGTCGTCCTTGTAGGAGTGGACACATTGTTTGCAGAGCACACAACCTAGGCTACACTGATTTCTGATTTATTT
CATTCCATTTACAAGTTGTCAATTTATTAATTGTCTTTTGTTTGTACTGTAGCGCTCCTGTCAATCTTGACTAAGGACAC
ACACCTAATTACACATAGAAGTAAGCCTATAGGCTACCTGGCCTGCACGCAAATGCAGGCCTATAAATATGCCCCTTTGT
CTGTTTTTACATCCATTGAAAATGCCAATAGTTCCTCAATGTAGCCTATTTGTGTCTCAGAAAGGGGAAGAAAAAAAAAT
GTCATGCCCCGATCCGGTGGAAATATCATAAAATAGGCCTACCTGATTACTTCTTATCCCGCAAATAGCCTACAGCTGTG
TGTCTCTCCGGAGCTCACTGGCACAGGAAACTGAGGGCCCAGAAAATTATAGGCAATGTTACTACTTCGCTAGCACGAGC
TTTGGGCCGGACCAAGCTAATAGTTGACAATGTTTCAAGTTCCTTGCAGATAGGCCATGCGTAGCCAAGGATAGGATATC
ATAGGATATTTATTTTTTAGCTAAATGAGAATGAGTGAGCTACAACAAACAACCAACAGGTATGCTGATTAATCTGAATT
ATGATGTTAATTGTTTACAAGGACAGGGAGCGCAGCATATTGTAGCCTCAATTACCTAAATAGCTAGCTGGCTAACTTAG
CTAGCTGAGCAATCTTCTCTACATTGATTTGATGCAGTCAAGTAGGACTGAGTGATATGGCCAAAATATATCACAGTATT
TAAAAAAAATATCATCATACCGCCCAATTTTTATGTTTTTGAATAATATGTATTATTTTGTGGATGTCCATCATCCATTT
CGTATGATATGTTCCAATTGCAATTTGTTGTGGCTAACGTTAGTTAAGTGGCTAGGGGCTAGAGTTAGGGGTCAAGGTTA
GGGCTAGGAGTTAGGTCAGGGTTAGCTAAAAGAGTTAAGGTTAGGGGAAGGGTTAGATAACATGCTAAGAAGTAACAATG
CTGTTAATTAGCAAAAATTGTCCTTGAGATTCACACACAACCTTTGGATTGCTAGATGTTAGGGGTCAAGGTTAGGGTTA
GCTAAGAGGGTTAAGGTTAGAAGAAGGGTTCACTAACATGCTAAGTAGTTGCAAAGTAGCTAAAAAGTAGTTGCAAAACA
GCACATTTTCCTAAAATGCTAAACATGTCCATGAGATTCGAACACACAACCTTTGCGTTGCTAGACATTCACGTTATACA
CCCACCCCGACCATACAAACCTTCCTTTAGTTTTTGCCTCAAGTAATCTTCCATCTTATGTAACCATAGCAAACATAACA
TATCATTACAATTTCCAGGATTTACACTTACCATGTTACATCTAGTCTATGAGACCAGTGACATTTCACATTCAACAGGG
TTTGGTTAACGCTAGGGTGCAAGAAAGATCAGGCATCATAAAACTGGTGTAGGTATGTGTCATGTAAACTACTCAATAGA
CAAATAACCCTGATCTTGAGAAAAGGGAAGAAAACTAATTGCTGCAAGGGATTCCTAAGCTAACTAAGTAGAAGTATAAG
GCTCCCCTTGAAATATGGAGCATCTGAATTGAAAGAGAACGTATTGCTACTCCTGTTCTGCCTTGACCAGATCAGAAGAT
CAATTTATTTGCAGTGGACAGTTGGAAAAATCCTAGGCCTACATTTTGCTTGGATGATCATTAAATCAGGGACATGCTCT
GAGCTGTGGTTTTGGCAACAAGATACAGAAGAGAATTGATAGCCATCAAACAATGGAGGGACCATGTTAAGGGAACTCTA
TGAAAAAATGATTTAGCTACAGGTTCATTCAAAAATCAGTCCTTTCCAGTGGTTGGTCAAAACTGAACAGATCATTTTAA
AAACTGTTAGGCCAAAGCTACATTATCATTCACATAATGTGTAGGCCGTCATTGTAAATAAGAATTTGTTCAACGGACTT
GACTAATTAAATAAATAAAAATCTGGATTTTGACACCTCAAGCAGTATGATACACAGTGGTTTTAATTGTGAGAGTCTAC
TTTTGATTATATTTTGAGCACATACTGTGACCTGACCCAAGCTAGGCCTATCTCCCCTCCACTCAGCAATACCACAACTT
TGGAAGCCAAGTTTCAGCCCACTGTGTGTGTGTTAATATGAGAAAGTGTTATTTTAAGTGCCAACCACAAGTTGTTTTTT
TGGGTTATCTGTACTTCACTATTTATATTTTTGAAGACTTTTTACTTCACTACATTCCTAAAGAAAATAATGTACTTTTT
ACTCCATACATGTCTCCCGACACCCAAAATTAAATTGAATGGCTAGCAGGACAGGAAAATTGTCAAATTCATGCACTTAT
CAAGAGAACATCCCTGGTCATCCCGATTGCCTCTGATCTGGCAGATTCAGTGTTTACGTGTGCCCCTGGCTATCCTCAAA
TAAAAAAATAAAAGCCAATTGTGCTGTCTGGTTTGCATAATTACTTACTAAGTATATTTCAGCAATTACATTTACTTTTG
ATACTTACGTATATTTCAAACCAAATACTTTTACTCAAGCAGTATTTTAATAGGGGACTTTCACTTGAGTAGTTTTCTTT
ACTTTAACTCAAGTATGACAAATCGGGTACTTCTTCCACCGCTGCCGACCACATCCTCTCGTCACTGCAGAAAAACTATT
CTGCTTTAGTAGAGAAATAGCTTATTATATTACTCAGGGGTTGGAACCGTTTTCAAAGAACATAAAAAAAGAACAAACCA
TTTTTAGGAACAGAAACAAAAGTGATATCTAGTGTTCCGGAACAGAACCATTATTTTCAAATCTTAAGAACAGTTTCATG
TTTTTCTTTGTTCCAGAAAGATTACCCATTTCTAGTGCATCATTCTGTAATAATTCATGTGATATCCTAAACACATTCCG
ATTCACTTCATGTCAAGCCCCCTCCATCCCCACATCACAGGAGGCATAATAATGGCCGGAACGAAGCAAATGGAATATCA
AACACTTGGAAACCACGCGTTTGATGTATTTGATACCATTCCACTTATTCTACTCCAGTCATTACCACAAGCCTGCACTT
ATCTGACCAATCAAACATGTAAACAAATATCTTATCCGTTTCACAAAAAGCTGCTCTCTCTGAAAATTGGTGGAGTAAAA
TGTAAAAACAAATGTTGATTTCAAAGGCTAAAACAAAAATGAAGGAAAACTTTCAATGTGTGCCAATGGGGATTTGCGCT
CCATAATACAGTTGAAAAATCAGTGTTCATGTTATGAGTGTGAGGACAAAAAGTGAAAGTGCCAGTCTTCTCATTGGAAG
TGCTCAATTAATTATACACTGAACAAAAAATATAAACGCAACATGTAAAGTGTTGGTCCCATGTTTCATGAGCTGAAAAG
ATCCCAGAAATGTTCGATACACACAAAAAAATTATTTAGCTCAAATTGTGCACAGATTTGTTTACATCCCTGTTAGTGAG
CATTTCTCCTTTGCCAAGATAATCAATTCACCTGACAGGTGTGGCATATCAAGAAGCTGATTAAACAGCATGATCATTAT
ACAGAAATACAGTGACGAGATCCTGAGGCCGATTGTCATGCCATTCATCCGCTGCCATCGCCTCATGTTTCAGCATGATG
ATGCACAGCCCCATATCGCAAGGATTTGTACACAATTCCTGGAAGCTGAAAATGTCCCAGTTCTTCCATGGCCTGTATAC
TCACCAGACATGTCACCCATTGAGTATGTTTGGGATGCTCTAGATCGACATGTAGGACAGCGTGTTCCAGTTCCCGCAAC
TTCGCACAGCCATTGATGAGTGGGACAACATTCCACAGGCCACAATCAACACCCTGATCAACTCTATGCGAAGGAGATGT
GTCACACTGCATGAGGCAAAAATGGTGGTCACACCAGATACTGACTGGTTTTCTGATCCATACGACTTCTTTTTTTTAAT
TTTTTTATTAAAGGTACTGTATCTGTGACCAACAGATGCATATCTGTATTCCCAGTCATATAAAATCTTTGAAATTGTTG
CATTTATATATTTTTGTTCAGTGTCATTTCTAAAAAGCACAGTGGTATCACTTTCTATTCACTGACATATCTGCAAGGCT
ACAGTGTAAGGTGGAAGTGGAGTGGCGTAATATGGCAGCACAGGTCTAAGACCTGCAGCAGAACCCACTGCTACTAATGG
GAGCTGTCCATTTTAGCTCTGGTCTGACAGGGCGAGCATGACTAGCCTGGAAATTTGCCCAACGCTGATTTGCATATGGA
CCAGTGTTGTATTATACCAACTTGTCAAGGCTGGCAGAGAAGGCTGTTATTGTGTAGTCTTACAGTCCCATATTGCATTT
GGGACATTGGTGGTAAGCCACGTGATCATTTTCTGACTGGATCCTTTGGCAGTTCTAACACGACGTGTGCATTAAGCTTT
GTTCAATAGCAATGAGAAGTTTGTCGACACAGAAGGTTTCCCAAATGTGGGCACCCTGGACCTACCAAGAGCTTAGTGAG
TGAAAGTATGTGCAGCAAATCTTTTGGGGGGAAATACAGAATTCTCTGCTCTCCAATACCTTTCAATAGACATAACTGAT
CTATTAATTTGTAGTTTTTTTTTAACCGTGAGTCACAGGAGTGGACATCAGATGAAATAAATAGTTCTTCACAGCTGGGA
AGTGAGACAGGTCCTTGTGTGGTGGTGAGAGATTACTTTACAAGGCAGAAATGTCAATGTTACCTTCATCAATCTCCAGG
CAGAGAGAGCGTCAAAGTTCAACGCTTTCAATATGCCAAGAAAGTGCTGCCCACAAAACCTGGCTTGAAAAAGAGCTAAC
TGGAAAGAACCATGTTCATTGCCGGCAAATCAACAATACCACAATGAAAAGGCTCACAATGAATAACATAGTTGATTGAG
ATTTTGGACAATAACAAGTACGAGCCTCATCTACAGTTGGTCCAGAAATTAGCTGATCAAAAGATATTGATGCAACTTCA
GTCACAGTGGGGACTATCCATTCATAAGGCTATACAGTTGACTTGCAGATATGATAAGAAGGCTAGAGCAGGGATGGGCA

ACTCCAGTCCTCAGGGGCCAAAGTGGTTGTCTCACTTTTCCCCCCAATCCCTAGCAAACACATTGATTAAACTAATTGCA
GTCTAAACTAAAATATCATTACTTGATTATTGGAGTCAGTTGTGTTAGCTGGGGCAAAAGGACTGGAGTTGCCCATCCCT
GGGCTAGAGTGTAAGCCATAAGCAGCTTAAAATCAAAAGAAGCATAGAAATAAGGAAAAGACCTACATAAGCCGTTTCCA
TTAACTTGTCCAGTGATTTTGTTGTTGACATTTAGAAAGTTTGCATAGAAATTGTAACAACTGCCTTTGTTTCTTCTGAA
CTGTCTTATGACAAACAGCAGGCTGTAATGATGTCACCCCACTGCCCCCAAAAAATGTAAACATTCGCAAAAAACTTGAG
TGCCGAATAAAAAAAATTCAAGTGGTTGAACTGTTTCCATAATCCATTTAGGGAAATTGCACGTTAATAAATGTGACAGT
TTGTATGCCTTCCCACCTAAAGTATGTTTCATGTCTCAGGTGGCCCACAAAGGCCAGCATGGATAGAATGCAAGAATTGA
CTCATATTTGCCATATTCTAATAATTCTCATGTGCCAACATATCGCCATGGTCTGTGCCATTGTGCAACTTGCACTCCTG
TAGATCTGAAATAATTGGGTTGTGAAACTTGCATCCAGCCAACCAGAAAAGCAAGGCGAAACAATTCAGGCATTCTTGAA
AGTCAAGACAATCCTTGTTCTGCAAAGAAACATTTTTATTTTTGCAAGCAGTAGACATTTAGAATTAAACTGTAGTGGAG
CTTTATAGTTATGCATGACATCACGTGCCCAGAGTACCTTAAATTATTCGTCAATAATCATTTGTTCTGAAAAACTGTTT
CCATCATAATTCCTCACAATAAAGAACGTTTGACAAATTACAAATCCACACATTGAATGGATAAAAAAAATGTTGTCAAT
CTTTAGAACATTTCTTCAATAGCTGCTGTTTCTATTACACATCGCAATGGTTTTCTTTGCGACATTGCTTTTATTGAATA
AACCTGGGTCAATGGAAACCTGCCTAATGAGCAGCAATAAGCTAAATACAAAAATGTTGTGAGTTTAGCAATGTGATAGC
CTCAGTTTGCAAGACCTAGGCTACACTTGAATGCAATTTATAGGCCTATTTAGGGATTTGTAGCCTTCACTTTCAGGGAA
AACAAGTCAAACGAACAACCTTGCAACACAGTCAATATGAGGAAATAGGTGAATGCATCATTTCCTTCATGAGGCCATCT
GTTGTTGTCTAATATAGAGGCCAACTGACAACACTATCGTAATGAAGACAACGGAATACAAAGCTGGGAAGTCTTAATGA
GGAAAAGCCTTGTCTCGTGATGAACTGCTTACAAAACCAATGTGTTCAGACAACAACGGCACCAATTTAATTTGGAAACC
GTTCTTAACCACAGGAATTGGCCCCGACTTTTCCCAAATGTGGAATGGCAACATATGGTAAAAGTCAAGCACTCAAGATT
GTTGTTTGAAAGATGTCGCAAACAGATCACTTTGGTCTCCAAATTCAGTTTACGCAAAGCATTCAAGCAATGTTTTCCAA
CAGAACAGAACGTACTTGTAACAACGCCTGTAGCCTAGACATTGTTGTCCCATACTAACTCCCTGTGTCTTCATATTAAG
GTCCCATCTTCAATTTCATAAATGAGTTAGTGAGGACTTGGCCTAGGATATTTTTAAACAATAGTATTCAAACTGATAAC
GGAGGTTTATCCCCTCCTAGTAGTAGCCTAGCCAGAATTCAGCTAAATCGCGCAGATATTTACCATGAGTTGACAACATA
GAAGTTACTTAATTTGCTCTGAATGGTGAATTGATACTTGCGATGAAATTCAGACGAGACCTATACTTTCACCTGACTGT
TTGCATTGTCAATGTGTGTGAACAGTGGTTTTTGGATTGCAAGTGGTCGTATGGTCGAGGTTGCTGAACCAATCAATGTG
CATAACATGTTACACTTCGTGGAAAATTAATCTAAGGATGCATCCACTATGCTAACCATCTATCCCTCTTCGCGTTTTC
AAATCCGTATACAAATATGATTTACCTTTATAATGCTGCTCCTGCGTGGTATTCCAGATTTGGAATCCAATCCGACAGAG
GAGTTGTTGGCTGTTTCAAGCCCTGGGCTGTCAGGTAACGTTACGGCGACAGATTGTGATCCAACCATGTCTGCCGGTAT
CCCACAGTCCCCATTGAAGACCGATACTTCGCACTGCGTCTTCCCGTCTGTTTTATCACTGGGCAAGGCCACCGAATCCC
CAGTATTCGATGGAGATGAGCCGTCGATCTCTCCAAACTCCGCCATAGGAACTCGACGGACACAGGCAAACCAAGCGTAG
CTGTCAAGCTGCAATGTAGGTGTTTCACTTGCAACGCTGTTGTATTTATCCAATGTCCTGGCTTGCTAGCTAGCTACCTC
GTTATAACATCTGCACAAAACCGTTCCGTATCATCTTTCGGTCCATATTTGACTCGTACCTAATGTCGCGTGCTCCATGG
GTTGTGCTCTGTAAAACAAAACGCATCATTAACCAGAGACACTGAATACCAATAGATTCAACTGTTTAATGCTAGACATT
GCATGCACACAAGAGGTCTAATAATGTGACTTTGAATGGTAGCTAACGTTAGTCAGCTAATTACATGCAAACAGTGCAAT
TGCATTGCAAAATTGTGTTTCACAATGGATGCATCAGCTAACGTTAGCTACATCGTCTCATTTGAAATGATGTCTGGTTA
AGGCATTCGATAGTTAGCAGCCTAGCTAAGCTTCGTTAGTCAACAAGTTAGCCATATTTACAATCGTCACGGATAGACGT
GGCTGGTTTGTTAGCTGCTGTGTAGATCTAGACTAGAGTTAATTTAGTTATAGCTAGTTAACGTAGTTAGACGTCCTTAC
TCGTTCATGTTACGTTAACTGACAAATTAGCCCACATTTCATACACTTGCCCATAGTTAGCTAACATTAACTAACCTGGC
AAGCTAACACTACAGCCAAATATGTTCTAACTAACCCTCATGTATCTGTGCTACAGCCACCCATGCCAACATAATTTCGT
GTTATCACACTTGCCTTGCTTGATAAAGTCTTGTAGAAAAAGAACAAAGTTCCATTCTGCTGTGCTATCCAGCTGCAACG
ACTCGACTACTGTTCAGTCTTGCATCACCACCAGTTGCTGCATTAGCTAACTTGCAATGTAGCTAGCTTGTAGTCACCGA
CTAAACCCAACTGTCAATACATTTGCTATCTAAATACAATTTATCTAGCGAGCTAACCGTTTAGGAAAAGTGAACGAACT
ACTTGTAACAGGCAGTAACGACAACGAACCTTCTGTCAATCAAACCTTACTCATAGAAAAAGGTAATCGATCAAAACCGG
TTCTCTATAATTACACTAGATGACTGACATGGGCACTGTTTTGAAGCCAACGCGCCTACGTCCTGGCATGCGCCCAACAC
AGTAAAAACAAAATCATTTTGGAAGCTATAGAAATGCAATTATTAATGTCTAAACAATTTTTTCCCACGTTTATTCTATT
ACAGACACCTTAATGCATACTTTACATATATATTATGTGAGTTAAACATACTTTATATATATATATATATATATATATAT
ATATATATATATATATATATATATATTTATTTATTTATTTTTCCATATATATATATATATTTTTTTCCATATTTTGTAACGT
TACAGCCTTATTCTAAAATTGATTGAATAATGTTTTTCCCTCATCAATCTACACACAGTACCCCATAATGACAAAATTAA
AACATGTTTGTAGAAATGTGTGCAAATTTATTAAAAACAAAAAACAGAAATACCTTATTTATATAAGTATTCAGACCCTT
TGCTATGAGACTCGAAATTGAGCTCAGGTGCATCCTGTTTCCATTGATCATCCTTGAGATGTTTCTACAACTTGATTGGA
GTCCACCTGTGGTAAATTCAATTGATTGGACATGATTTGGAAAGACACACAACTGTCTATATAAGGTCTCACAATTGACA
GTGCGTTCCAGAGCAAAAACCAAACCATGAGGACAAAGGAATTGGCCGTAGAGCTCCGAGACAGGGTGGTGTCGAGGCAC
ATATCTGGAGAAGGGTGCCAAAAAATGTCTGCAGTATTGAAGGTCCCCAAGAACACAGTGGCCTCCATCATTCTGAAATG
TAAGAAGTTTGGAACCAACAAGGCTCTTCCTAGAGCTGGCCGCCCGGCCAAACTAAGCAATCAAGGGAGAAGGGCCTAGG
TCAGGGAGGTGAACAAGAACCCGATGGTCACTCTGGCAGAGCTCGAGAGTTCTTCTGTGGAGATGGGACAACCTTCCAGA
AGGACAACCATCTGTGCAGCACTCCACCTTTATCGTAGTGTGGTCAGACAGAAGTCCCTGCTCAGTAAAAGGCACATGAC
AGCCCGCTTGGAGTTTGCCAAAAGTCACCAAAAGAACTCTCAGACCATGAGAAACAACATTATCTGGTCAGACCATCATA
GTCCCTGTGCCCAATAACACTGAGGTAACCTGCCTAAATGACTACCGACCCGTAGCACTCACGTCTGTAGCCATGAAGTG
CTTTGAAAGGCTAGTCATGGCTCACATCAACAACATTATCCCAGAAACCCTAGACCCACTCCATTTTGCATACCGCCCCA
ACAGATCCACAGATGCAATCTCTATTGCACTCCACACTGCTCTTTCACACCTAGACAAAATCAACACCTATGTGAGAATG
CTATTCATTGACTACATCTCAGCGTTCAACACCATAGTGCCCTCATAACTCATCACTAAGCTAAGGACCCTGGGACTAAA
CCTCCCTCTGCAACTGGATCCTGGACTTCCTGACGGCCACCCCATGTGGTAAGGGTAGGTAACAACACATCCGTCACGCT
GATACTTAACACGGAGGCCCCTCAGGGGTGCGTGCTCAGTCCCCTCCTGTACTTCCTGTTCACTCATGACTGTGTGGCCA
GGCACGACTCCAACACCATCATTAAGTTTGTCGATGACACAACAGTGGCCTGATCACCGACAATGACGAAACAGCCTATA
AGGAGGAGGTCAGAGACCTGGCCGTGTGGTGCCAGGTCAACAACCTCGAGAGCATCCTGACTGGTTGCATCACTGCCTGG
TATAGCAACTGCTCGGCCTCCGACCGCAAGGCACTATAGAGGGTAGTGTGTATGGCCCAGTACATCACTGGGGCTAAGCT
TCCTGCCATCAGGACCTCTATACCAGGCGGTGTCAAAGGAAGGCCCTAAAAATTGTCAAAGGCTCCAGCTACCCTTGTCA
TAGATTGTTCTCTCTGCTACTGCACGGCAAGCAGTAGTGGAGCTCTAAGTCTAGGTCAAAGAGGCTTCTAAACAGCTTCT
ACCCCCAAGCCATAAGACTCCTTAACATCTAATCAAATGGATGGCTGAAAACACAACCATCGCGGGATCTACGAATTTCC

GGCCAAGGGGGTCAATTTAAAAATCATCCCTTCCTCCCTTGCCCTGCATGTGTATACTCGTCAGACGTCATGATACGTCA
TCAGAAGTGTCCATTTAATTTGAGGGCTGAGGGGATAGGGTGTGTCTTTTAAGTGTTTGGACCGCAGTCAGTGTTTCCTC
CAACACATTGGTGCGGCTGGCTTCCGGGTTAAGCAAGCAGTATGTCAAGAAGCAGTGCAGCTTGGCAGGGTCGTGTTTCG
GAGGACGCATGACTCTCGACCTTCACCTCTCCCGAGTCCGTACCTTGGCTGCATTTCCAACTTGTCTGACGAGTATACAC
TTTCAGGGCAAGGGGCGAGGGAGGAAGGAATGATTTTTAAATGGGCCACCCTTGCCTGGAAATTTGTCACTTGTTCATCC
CGCAATTGTGTTTTCAGCCATCGGTAGCTTGTGGGTGCTTTATATGCGCTACAGTCAATTATGAGTGCATGTCTACTTAT
ATTAACTATGTAATTATTCATATACGCCTCCTGTATGATAGCTTGCAAATTAATTGGCTAATAAACGTTAGCCTGCCTTG
ATACTTCTGCCAAGCTACTTTCCAAAAGCTAACCAAACAAAAACATCATTACGTTTATGAGACCTGTTTGTGCATTAGTA
GCACAATTTCGAACTTATTTACTTTCGTTTTTTACTTACACTTGTATATTGACTCCATATTGACGTTGAGGTTTTAAGTT
TTGGCAGACTTTTCTTAGTGGATGAACAATCATCGCAAATTTAATTATGGGGCGTTTCAGGCCCTGAAGTGAACATAATT
GAAAAACGAGGGCTGAGGGGCTTACGTTGCAAACTTCCCTTGCTTGGCTAATCATTTGGACTGACGACAAATATGGCCAC
GGGGATTCTCCCAAGGGCATAAGGTGAGGGTAAGTAGACGAGGGTGTGTCTTTTATAAGCCCTTGTCCCAAGGTACTCCA
TACCGGAGTTGCAGCGATGGGACAAGACTGTAACTACCAATTGGATATCACGAAATTGGGGAGAAAAAGGTGTAAATATA
TATATATATTATTAAATCAACTTAAAAACAACTCCAACTGTATTTTGAATGAATATACTAAATTACAATTTAAAGCGTTT
CAAATTGAAGTACAGTTTTAATGAGTGTGTTGAAATTTAAAGATGGTGAACATATACTGTAGTATACTTGACTGAAAAAC
AATTAATTTAAAGTACACTTCTAATAAGTGTATTGAAGGTTGACGGTAGTATATTTATAGTATACTAAAAGCTTGAAAAA
ACTATTAATTTAAAGTACACTAATTTAAAGTACACTATTAATTTAAAGTGTGTTAGTGTAATGATATTATATATATAGTA
TACTTAAGATAAGAAAAATACATCTCGTATTTGAGGTATATTATTTTTAAAAAATTGTATATTTAAGTATACTTGTAATA
TATAAAAAAGATTACAAATATTTCACTTGGGATGTTAATCAGCCAATATTAAAATGTTGGATTTAATTCAGTGCTCTTGA
ATTTAATTCAAGATGCTGGATTGTGTCAATCCAAATTATTTCCTGGCTAAAAATATAGCATTTTACGGTCTGAAAAATGT
ATATGCTGTATTTATGTCAATTGTCAACAGATTGTCACATAACTTGTAGAAGACTAATTGTTCCTTCAATAGCATAGCCT
ATCCAGTTATTTAAACAGATAACAGTATAATGCATTGTTTTTCTTGTAGCAGCTCTTATTCTCTTTAGCTATTCAAAAAG
GATCCTTACAGTTTACGTTCTAATATTTTAGTCAAAGTTAATAATGTCTGAGTTTATGTGCAGGAAGGTATGCATTGCTG
AAGTTTATATAGCCTACACATGAGACATGAACATAAACAGTTAAATTGAACCTTTTTTTTAAATCAAATTCTAGACAATA
AATATACTACAGAATACCTTACCTCTATATCAGTGTGCCAGTTCCACCTTGTGTTGCCTTCTCAGGGTTCTTATTAGAAG
TCATGTCTTTTTTCTGATTAAGGATCAGTTCACATAAATCAATTGTTAAAAATACATATTGATCAGTTCCAGGATGTTGT
AAGGGACAGGAGAAGGGACGGTGTACACTAACTGTTCAAAAGTTTGGGTTCACTTAGAAATTGCCTTGTTTTTTAAAGAA
AAGCACTTTTTCTGGTCCTTTAAAATAACATCCAGGATGCNNNNNNNNNNNNNNNNNNNNNNNNNNNNNNNNNNNNNNNN
NNNNNNNNNNNNNNNNNNNNNNNNNNNNNNNNNNNNNNNNNNNNNNNNNNNNNNNNNNNNNNNNNNNNNNNNNNNNNNNN
NNNNNNNNNNNNNNNNNNNNNNNNNNNNNNNNNNNNNNNNNNNNNNNNNNNNNNNNNNNNNNNNNNTAGAAGGCCAGCAT
CCCGGAGTTGCCTCTTCACTGTTGACGTTGAGACTGGTGTTTTGCGGGTACTATTTATTGAAGCTGCCAGATGAGGACTT
GTGAGGCGTCTGTTTCTCAAACTAGACACTCTAATGTACTTGTCCTCTTGCTCAGTTGTGCACCGGAGCCTCCCACTCCT
CTTTCTATTCTGGTTAAAGCCAGTTTGCGCTGTTCTGTGAAGGGAGTAGTACACAGCATTGTACGAGATCTTCAGTTTCT
TGGCAATTTCTCGCATGGAATAGCCTTCATTTCTCAGGACAAGAATAGACTGACGAGTTTCATAAGGAAGTTCTTTGTTT
CATGCTGTTTTGAGCCTGTAATCGAACCCACAAATGCTGATGCTCCAGATACTCAACTAGTCTAAAGAAGGCCAGTTTTA
TTGCTTCTTTCATCAGGACAGCAGTTTTCAGCTGTGCTAACATAATTGCAAAAGGGTTTTCTAATGATCAATTTGCCTTT
TAAAATGATAAACTTGGATTAGCTAACACAACGTGCCATTGGAACACAGGAGTGATGGTTGCTGATAATGGGCCTCTGTA
CCCTTATGTAGATATTCCATTAAAAATCAGCCGTTTCCAGCTACAATAGTCATTTACAACATTAACAATGTCTACACTGT
ATTTCTGGACAAAATTGAGAAAAATGGACAAAAAATGTTTGCTTTTCTTTCAAAAACAAGGACATTTCTAAGTGACCCCA
AACTTTTGAACGGTAGTGTACATCAGGCCTGCAGGCGGTCATGTTATTCTGATACGGAATTTACTGTTGTACAACAGAGG
CATCTGGTGGTGACGCTGACTATCTGATAGTGAGTTGTAAAACCATTAACGCAGAGTCCATGAATTAATCTTCGTGATCA
TTTAATGTGATTAAGGAAAATAATAAGGGTTATATATTTCTGTCATGTGCAAACATTTAATGTTGATAGAGCAAATGGTG
AGCAGCATGAATCAACTTAAAGTTAAATGTGTTGAGGTATTACAGTTCTGTATAACCATATTACTGTGTTAGCAGAAATG
AAATGATCAAAATGTTCACTGTTACTGTAACTGATGGAAATATTGCTTCTCCAAATGTAGAGGATATTCTTATTCAACTT
CCATGAAAATCAAAAATGGATTTTCTTTGGTGAAATGGGTATGAAGACATTGAAACCAATGAAAGGGAACAAAATAAATT
GCAACGCTTCAAACTAACATTAGTTACTGTAGGTTAGACACCTTATGTTACCCCTGGAGTAAGAATCATGTTTTATCTGT
AGCTTAGACCTACAGTGATAATTAAGATATTAGTGATAGTTAAAATTTTTCAAATCAACTGACTTTACCAGAAGTACAGT
AGATAGATCAACCAGGTTGTATGTTTTTTTTTCTTCTTCTCATAGACAAGGAAACAACAGAGGAATCATTCAAAGTTATC
GTTCAGTGGACCATTTTGGAGAGGTCTGGTGGTTTCACTTCTTTGCTATTGGGATGATTTTGGAGGTGCACAGGCAGAGG
GCGGGACACCAGGCGTACACCAAAGGATTACTGCACCCGGCCTGTTTTTTCATGGCGGCACAGTTGACATACTTGTTTAT
GTAAGGACAGGGATTGGCTGAAATGACACATGAGAAGAAACATTGGGAGTCAATTCATTGTATGTTGACAGTTTATATTT
GGTGATTATTGTACATTGAACCGTGTGAACATGTTTTCAAAAGCTATCACTAACATTGAGTCATATTGGTAAGATTTTAG
ACCGCATGTATTTTCATCCCGTCTGATACTTACTGCAAAGCTTATTTTCGCACGAGTTGGGGCAGTCAGCACATGTAGCT
CCTTCCTTGTATGGAGCCACTCCTCTGTAGTTTCCCCTGATAGCCAAAGTATTGAAATGATCGCTGGATGATGCCTACCT
TCCACTACCAGTTACAGTAAATAGGGTGAAATTGTACACAGGTGTTAGGTGCATTTTGAGTGTGATTGGTGCAACATACG
CTCTGTAATACTGGCATCCATAGAAGTAGACTGAGCCAGGACACAGGGCCACACCACAGCCAACCTTGTATGAACTGTTC
CAAACCACCTATATGAAAGGAAACACAGTTTAGTGTTTTCAGAGTTTCGTTGGCGAATAACCATAATGAGACTTGTCGGT
AACTACACACAACTTTGACTACCATACAGCAAAGCAAATCAAATCACATTTTACTTGTCGCATACTCATATTTAGCAGAT
GTTATTTCAGGTGTAGCACGATGGTCCATAACAATATACACTGAGTATACAAAATGTTAAGAACTAAGAACACCGTCTTT
TTCCATGACACAGACTGACCAGGTGAATCCAGGTGAAAGTGATGATCTCTTATTTATGTCACTTGTTAAATCCACTTCAA
TCAGTGTAGATGAAGGGAAGGAGACAGGTTAAAGAATGATTTTTAAGCCTTGAGAATTGAGACCTGGATTGTGTATGTGT
GCTATTCAGAGGGTGAATGGACAAGACAAAATATTTAAGTGCCTTTGAACTGAGTATGGTAGTAGGTGCCAGGCACACCG
GGGTGCAACTCAATATTAGGAAGGTGTTCCTAATGTTTGATATACTCAGTGTATGCATAATACAATGACTGAAATTGCTA
CCAAAAAAATTGTAAATAAGTTAGTAATCTATTTTGTAAGTATACTCCTTTGGTGTCAATAGGTATATGTGATTAAATAT
CCCTGAACATGACAGAATAGGAACTGTAGTTTTAGTGTGTGTTTCCTATCTGACCCTAATTAGTGCTGGACATATAAAAA
ATACCCCATAGTCCATTGCGGGTCCTTTTGTACCTAAAGGTCCAGTGTTAAGATTGTATAGTGTTGACCATGCTATCTTT
AGCAACTGTTTTGGGTGTGGTCGAATCTGTTCAGAAGGTATGTTTATTTCCATATCGGTTTGTTTATTCAAATTTGACAG
CATGTGTATTACTTTGAAAGTTATGCCACTGCAGTTGTCTTTGATGTGTTTGGAGAGATGTTCATGAGGGTCCAAGCAGT
ATATTGAAACTGCCCTCCTTTAAGGTGACTGTTTAAGTTAACACATTTTGTATTTCTAAAATAGTTACTGTTCGTGTAGA

TATAGTGGCTAACCGCTCTTTTCATGCAGAAGTTGTTTGTCACTGTTTTCGGTTGTGAACATGGTCAGTTGTCACTGGAA
GCACTGTAGATATTTAGAAAATGATACAGCCATCCAGTGAAAGGAACTGAGGTGCGTTTTGCCTTGTTTTACTGTACATT
TTTTAAACAAGCAAACTTTTAGCTGATTCATTCACTTCTTGGTCTTTTTTTTTTATATCCACCTCAGACTGTTTCTCTAT
AAATGGGCCTATGAAACCTGTAATTTGATCAGATGTTACACGGTGCATTTGGAAAGTATTCACACCCCTTGACTTTTCCC
ACATTTTGTTATGTTACAGCCTTATTCTAAAATGTATAAAATATTTTTTCCCTCATCAATCTACACACAATACCCCATAA
TGGCAAATTATTAGCAACTTTATAAAATAAGAAACTGATATGACCTTTACATAAGTATTCAGACCCTTTAGTCAGTACTT
AGTTGAAGCACCTTTGGAAGCGATTACAGCGTTGCTGCACAGCTATTTTCAGGTCTCTTCAGAGATGTTCGATCGGGTTC
AAGTGCGGCCTCTGGCTAGGCCACTCAAGGACATTCAGAGACTTGTCCCGAAGCCACTCCTGCGTTGTCTTGGCTGTGTG
GTTTTCATTAAGGATCTCTCTGTACTTTGCTCCGTTCATCTTTGCCTTGATCCTGACTAGTCTCCCAGTCCCTGACACTG
AAAAACACCCCCACAGCATGATGCTGACACCACCAGCATGCTTCACCGTAGGGATGGTGCCAGATTTCCTCCAGATGTGA
CGCTTAGCATTCAGGCCAAAGAGTTCAATCTTGGTTTCATCAGACCAGAGAATCTTGTTTCTCATGGCCTGAGAGTCTTT
GGGTGCCTTTTGGCAAACTCATAGCGGGCTGTCATGTGCCTTTTATTAAGGAGTGGCTTTTGTCTGGCCACTCTACCACA
AAGGCCTAATTGGTGGAGTGCTGCAGAGATGGGAGAACCTTCCAGAAGGACAACCATCTCCACAGAGGAACTCTGGAGCT
CTGTCAGATTGACCATCGGGTTCTTGGTCACCTCTCTGACCAAGGCCCTTCTCTGGGTTGCCAGCTCTAGGAAGGGTCTT
GGTGGTTTCAAACTTCTTCCATTTAAGAATGATGGAGGNNNNNNNNNNNNNNNNNNNNNNNNNNNNNNNNNNNNNNNNNNN
NNNNNNNNNNNNNNNNNNNNNNNNNNNNNNNNNNNNNNNNNNNNNNNNNNNNNNNNNNNNNNNNNNNNNNNNNNNNNNNN
NNNNNNNNNNNNNNNNNNNNNNNNNNNNNNNNNNNNNNNNNNNNNNNNNNNNNNNNNNNNNNNNNNNNNNNNNNNNNNNN
NNNNNNNNNNNNNNNNNNNNNNNNNNNNNNNNNNNNNNNNNNNNNNNNNNNNNNNNNNNNNNNNNNNNNNNNNNNNNNNN
NNNNNNNNNNNNNNNNNNNNNNNNNNNNNNNNNNNNNNNNNNNNNNNNNNNNNNNNNNNNNNNNNNNNNNNNNNNNNNNN
NNNNNNNNNNNNNNNNNNNNNNNNNNNNNNNNNNNNNNNNNNNNNNNNNNNNNNNNNNNNNNNNNNNNNNNNNNNNNNNN
NNNNNNNNNNNNNNNNNNNNNNNNNNNNNNNNNNNNNNNNNNNNNNNNNNNNNNNNNNNNNNNNNNNNNNNNNNNNNNNA
CATCTCATGGATCTCCAGTGGAAACAGGATGCATCTCAATTTTCGAGTCTCATAGCGAAGGGTCTGAATACTTATGTAAA
TAAGGTATTTCTGTTTTTGGTTTTAATCACTTAGTCATTATGGGGTATTGTGTGTAGATTGGTGAGGAAATTGTTTTATT
TCATCCATTTTAGAAGGCTGTAACGTAACAATGTGGAAAAAGTCAAGGGGTCTGAATACTATCTGAAGGCACTTCATGTC
ATAATGTATCTGCATGTAAGATGAATGTTACCTGTGTGTAGTGGCCGATGCATTTACCATTGATAGATCCATTGGGATAG
GAGTAATCTATGACCTCACTGTGCCAGGCTGTAACTATTTCAGTCCAATTCTTAGAAGCGGAGGACATGAACAGGTTCTC
ACCCATTTCGTAGTCTGTTCAGAAGTAAAGCATGCATGGAAAATTTTACTAAATAGGTGTGTAACAGTGAAATCAGACTG
GAACAGTTGACTAAAATGTCTTTGAATGCAGGATTTAAGATGTCAACAAATGTAAGCTACCATAGAGAGTCACTGGTGAT
AAGAAATCATGACATTCAAAATATATTTGAGCTTAAGATCATGCTCACATTGATACCATTGCAGCGAAAGTCTTGAGTTG
CATGAATCAATAAAATATATTTGTCTTGGTTTGCTCTACAGCAGGGGAAAAACAGGAGATGTCAAGGAGAGACAAAGGGG
AAACCGTACCGCCAAGCATGCGACTGCTGGGTGGGCCATGAGCCATGGAGCAGGTGTCAACCCAAGCCTGAACACTGGCC
GCTGCCTCCTCGCTCCAATTCTGCAGATCCAATACTAACAGTTTTAGAATAAAGCCATCCACAAGACTGACATCCACATA
CTAGTGTCCAAGGAGTATTTTGTGGCCACTGTATTTTCTCTACCTGTACATAGACTAACCATATAGGCCTACCATAGGGC
TGCTCTATGAATTACATATCAGGGATTATATTCTTTATGACGGATTTAGGGGGATACTATGTTGGAAACATTGATTGTGA
GGTTTTGATACTGATGGAAAGTATATGAATCATGAAAGTGTACCATCTTGAGCATGTCTCTAGCAGTTGGCATAACCGCT
CTCCGGAAGGCGTTGTGTTGGTTAAGGATCTCAGCCTGAACTGCTGTGGTGTCTGGGCAAATTTCTATACATATATACAC
ACACACACACATTATTTTGTGGAATTGTATGCACATTACTTACTCCACGTGTAAATACTTCACTAAACCATAATACAAAG
CAACTATTTACTCCTAATATCTTTGCCAACATCTGGACAATGAGTCCCAAATCAATCCACTTTCTTTTGTAAAAACAACA
CGTGATGAATTAAACATTGTGTGTGATGACTAAATCCAGTTTAATTCATTTATAATCATGTTGGTAGAGGATAGTGAAAG
TGGACACACCCTTTGCGAAGTCTTCACATTTTGCTGCATCAAATTGAAAGGGATTACATTTTTTTCCTACAGTACAGATC
TACACAACCTACTCCACCTTTTTGAAGTAAAAGAAAGTTATAGAAAATGTAAACATACAACAACAAAAAACAAAGATGTT
TTATTTTTGCGTGTGTCTTCACACCCCAGAGTTAATACTTGGTGGAGCAGCCATTACAGATGTAAATCATTTAGAATCAT
ATTCTACCAACTTTACACAACTCTTAGGGCAGCATATGCCAATTTTTTTGTCAAGATTGCTCAAGAGCAGTAAATTTGGG
TTGAGAATCATTGATGGAAATCATATTTTAAACCTCTTTTCAAGCAAATTTTGTCNNNNNNNNNNNNNNNNNNNNNNNNN
NNNNNNNNNNNNNNNNNNNNNNNNNNNNNNNNNNNNNNNNNNNNNNNNNNNNNNNNNNNNNNNNNNNNNNNNNNNNNNNN
NNNNNNNNNNNNNNNNNNNNNNNNNNNNNNNNNNNNNNNNNNNNNNNNNNNNNNNNNNNNNNNNNNNNNNNNNNNNNNNN
NNNNNNNNNNNNNNNNNNNNNNNNNNNNNNNNNNNNNNNNNNNNNNNNNNNNNNNNNNNNNNNNNNNNNNNNNNNNNNNN
NNNNNNNNNNNNNNNNNNNNNNNNNNNNNNNNNNNNNNNNNNNNNNNNNNNNNNNNNNNNNNNNNNNNNNNNNNNNNNNN
NNNNNNNNNNNNNNNNNNNNNNNNNNNNNNNNNNNNNNNNNNNNNNNNNNNNNNNNNNNNNNNNNNNNNNNNNNNNNNNN
NNNNNNNNNNNNNNNNNNNNNNNNNNNNNNNNNNNNNNNNNNNNNNNNNNNNNNNNNNNNNNNNNNNNNNNNNNNNNNNN
NNNNNNNNNNNNNNNNNNNNNNNNNNNNNNNNNNNNNNNNNNNNNNNNNNNNNNNNNNNNNNNNNNNNNNNNNNNNNNNN
NNNNNNNNNNNNNNNNNNNNNNNNNNNNNNNNNNNNNNNNNNNNNNNNNNNNNNNNNNNNNNNNNNNNNNNNNNNNNNNN
NNNNNNNNNNNNNNNNNNNNNNNNNNNNNNNNNNNNNNNNNNNNNNNNNNNNNNNNNNNNNNNNNNNNNNNNNNNNNNNN
NNNNNNNNNNNNNNNNNNNNNNNNNNNNNNNNNNNNNNNNNNNNNNNNNNNNNNNNNNNNNNNNNNNNNNNNNNNNNNNN
NNNNNNNNNNNNNNNNNNNNNNNNNNNNNNNNNNNNNNNNNNNNNNNNNNNNNNNNNNNNNNNNNNNNNNNNNNNNNNNN
NNNNNNNNNNNNNNNNNNNNNNNNNNNNNNNNNNNNNNNNNNNNNNNNNNNNNNNNNNNNNNNNNNNNNNNNNNNNNNNN
NNNNNNNNNNNNNNNNNNNNNNNNNNNNNNNNNNNNNNNNNNNNNNNNNNNNNNNNNNNNNNNNNNNNNNNNNNNNNNNN
NNNNNNNNNNNNNNNNNNNNNNNNNNNNNNNNNNNNNNNNNNNNNNNNNNNNNNNNNNNNNNNNNNNNNNNNNNNNNNNN
NNNNNNNNNNNNNNNNNNNNNNNNNNNNNNNNNNNNNNNNNNNNNNNNNNNNNNNNNNNNNNNNNNNNNNNNNNNNNNNN
NNNNNNNNNNNNNNNNNNNNNNNNNNNNNNNNNNNNNNNNNNNNNNNNNNNNNNNNNNNNNNNNNNNNNNNNNNNNNNNN
NNNNNNNNNNNNNNNNNNNNNNNNNNNNNNNNNNNNNNNNNNNNNNNNNNNNNNNNNNNNNNNNNNNNNNNNNNNNNNNN
NNNNNNNNNNNNNNNNNNNNNNNNNNNNNNNNNNNNNNNNNNNNNNNNNNNNNNNNNNNNNNNNNNNNNNNNNNNNNNNN
NNNNNNNNNNNNNNNNNNNNNNNNNNNNNNNNNNNNNNNNNNNNNNNNNNNNNNNNNNNNNNNNNNNNNNNNNNNNNNNN
NNNNNNNNNNNNNNNNNNNNNNNNNNNNNNNNNNNNNNNNNNNNNNNNNNNNNNNNNNNNNNNNNNNNNNNNNNNNNNNN
NNNNNNNNNNNNNNNNNNNNNNNNNNNNNNNNNNNNNNNNNNNNNNNNNNNNNNNNNNNNNNNNNNNNNNNNNNNNNNNN
NNNNNNNNNNNNNNNNNNNNNNNNNNNNNNNNNNNNNNNNNNNNNNNNNNNNNNNNNNNNNNNNNNNNNNNNNNNNNNNN
NNNNNNNNNNNNNNNNNNNNNNNNNNNNNNNNNNNNNNNNNNNNNNNNNNNNNNNNNNNNNNNNNNNNNNNNNNNNNNNN
NNNNNNNNNNNNNNNNNNNNNNNNNNNNNNNNNNNNNNNNNNNNNNNNNNNNNNNNNNNNNNNNNNNNNNNNNNNNNNNN
NNNNNNNNNNNNNNNNNNNNNNNNNNNNNNNNNNNNNNNNNNNNNNNNNNNNNNNNNNNNNNNNNNNNNNNNNNNNNNNN

NNNNNNNNNNNNNNNNNNNNNNNNNNNNNNNNNNNNNNNNNNNNNNNNNNNNNNNNNNNNNNNNNNNNNNNNNNNNNNNN
NNNNNNNNNNNNNNNNNNNNNNNNNNNNNNNNNNNNNNNNNNNNNNNNNNNNNNNNNNNNNNNNNNNNNNNNNNNNNNNN
NNNNNNNNNNNNNNNNNNNNNNNNNNNNNNNNNNNNNNNNNNNNNNNNNNNNNNNNNNNNNNNNNNNNNNNNNNNNNNNN
NNNNNNNNNNNNNNNNNNNNNNNNNNNNNNNNNNNNNNNNNNNNNNNNNNNNNNNNNNNNNNNNNNNNNNNNNNNNNNNN
NNNNNNNNNNNNNNNNNNNNNNNNNNNNNNNNNNNNNNNNNNNNNNNNNNNNNNNNNNNNNNNNNNNNNNNNNNNNNNNN
NNNNNNNNNNNNNNNNNNNNNNNNNNNNNNNNNNNNNNNNNNNNNNNNNNNNNNNNNNNNNNNNNNNNNNNNNNNNNNNN
NNNNNNNNNNNNNNNNNNNNNNNNNNNNNNNNNNNNNNNNNNNNNNNNNNNNNNNNNNNNNNNNNNNNNNNNNNNNNNNN
NNNNNNNNNNNNNNNNNNNNNNNNNNNNNNNNNNNNNNNNNNNNNNNNNNNNNNNNNNNNNNNNNNNNNNNNNNNNNNNN
NNNNNNNNNNNNNNNNNNNNNNNNNNNNNNNNNNNNNNNNNNNNNNNNNNNNNNNNNNNNNNNNNNNNNNNNNNNNNNNN
NNNNNNNNNNNNNNNNNNNNNNNNNNNNNNNNNNNNNNNNNNNNNNNNNNNNNNNNNNNNNNNNNNNNNNNNNNNNNNNN
NNNNNNNNNNNNNNNNNNNNNNNNNNNNNNNNNNNNNNNNNNNNNNNNNNNNNNNNNNNNNNNNNNNNNNNNNNNNNNNN
NNNNNNNNNNNNNNNNNNNNNNNNNNNNNNNNNNNNNNNNNNNNNNNNNNNNNNNNNNNNNNNNNNNNNNNNNNNNNNNN
NNNNNNNNNNNNNNNNNNNNNNNNNNNNNNNNNNNNNNNNNNNNNNNNNNNNNNNNNNNNNNNNNNNNNNNNNNNNNNNN
NNNNNNNNNNNNNNNNNNNNNNNNNNNNNNNNNNNNNNNNNNNNNNNNNNNNNNNNNNNNNNNNNNNNNNNNNNNNNNNN
NNNNNNNNNNNNNNNNNNNNNNNNNNNNNNNNNNNNNNNNNNNNNNNNNNNNNNNNNNNNNNNNNNNNNNNNNNNNNNNN
NNNNNNNNNNNNNNNNNNNNNNNNNNNNNNNNNNNNNNNNNNNNNNNNNNNNNNNNNNNNNNNNNNNNNNNNNNNNNNNN
NNNNNNNNNNNNNNNNNNNNNNNNNNNNNNNNNNNNNNNNNNNNNNNNNNNNNNNNNNNNNNNNNNNNNNNNNNNNNNNN
NNNNNNNNNNNNNNNNNNNNNNNNNNNNNNNNNNNNNNNNNNNNNNNNNNNNNNNNNNNNNNNNNNNNNNNNNNNNNNNN
NNNNNNNNNNNNNNNNNNNNNNNNNNNNNNNNNNNNNNNNNNNNNNNNNNNNNNNNNNNNNNNNNNNNNNNNNNNNNNNN
NNNNNNNNNNNNNNNNNNNNNNNNNNNNNNNNNNNNNNNNNNNNNNNNNNNNNNNNNNNNNNNNNNNNNNNNNNNNNNNN
NNNNNNNNNNNNNNNNNNNNNNNNNNNNNNNNNNNNNNNNNNNNNNNNNNNNNNNNNNNNNNNNNNNNNNNNNNNNNNNN
NNNNNNNNNNNNNNNNNNNNNNNNNNNNNNNNNNNNNNNNNNNNNNNNNNNNNNNNNNNNNNNNNNNNNNNNNNNNNNNN
NNNNNNNNNNNNNNNNNNNNNNNNNNNNNNNNNNNNNNNNNNNNNNNNNNNNNNNNNNNNNNNNNNNNNNNNNNNNNNNN
NNNNNNNNNNNNNNNNNNNNNNNNNNNNNNNNNNNNNNNNNNNNNNNNNNNNNNNNNNNNNNNNNNNNNNNNNNNNNNNN
NNNNNNNNNNNNNNNNNNNNNNNNNNNNNNNNNNNNNNNNNNNNNNNNNNNNNNNNNNNNNNNNNNNNCCTTCTTGAATG
GGTGCAAACAGGTTGTGATTGCATGTGATTGAGGAATGTTACATTGTCCCAAGCAATCACAAAAGTCCTCAAGTTTCCTC
CCAGCTGTTCTCTTTTTGCTTTTGGCACCAGTTGTTGGTGGAGGTCATCTAAAAACAAAAGAAGGCTCTCACTGTTGTAG
GGACCAATCTGGCATTTCTGCAGGACCAAACCAGCGCTCGAGATTGCGGCACACATTGTGATATTTGCTCCTCTCTGACC
CGGCACATCAACGGTGGCCCTTTACCCGACAATGTTTCTTCCCCGCCGATGTGTTTTTGCCAGGTTAAACCCTGCCTCGT
CTACATAAATTATTTCACATGGGGTCTGATTAGCCTCCAACTCCATGACTCTCTGAAAGAAATCAAACACAGTATGTGTA
AATGCTTTTCAGTATGTCCTACTGTATGTACTGTAACCCATGTTTACATGTAGAATAGCATAGTGTCCTACTGTATGTAC
TGTAACCCATGTTTACATGTAGAGTAGCATAGTGTCCTACTGTATGTACTGTAACCCATGTTAACATGTAGAGTAGCATA
GTGTCCTACTGTATGTACTGTAATCCATGTTTACATGTAGAGTAGCATAGTGTCCTACCTTATGTACTGTAATCCATGTT
TACATGTAGAGTAGCATAGTGTCCTACTGTATGTACTGTAACCCATGTTTACATCAAATCAAATCAAATCAAATGTATTT
ATATAGCCCTTACATCAGCTGATATCTCAAAGTGCTGTACAGAAACCCAGCCTAAAACCCCAAACAGCAAGCAATGCAGG
TGTAGAAGCACGGTGGCTAGGAAAAACTCCCTAGAAAGGCCAAAACCTAGGAAGAAACCTAGAGAGGAACCAGGCTATGA
GGGGTGGCCAGTCCTCTTCTGGCTGTGCCGGGTGGAGATTATAACAGCACATGGCCTAGATGTTCAAATGTTCATAAATG
ACCAGCATTGTCAAATAATAATAATCATAGTAGTTGTCGAGGGTGCAACAAGTCAGTAACACAAGAGTAAGTGTCAGTTG
GCTTTTTCATAGCCGATCTTTGTGAGTATCTCTACCGCTCCTGCTGTCTCTAGAGAGTTGAAAACAGCAGGTCTGGGACA
GGTAGCACGTCCGGTGAATAGGTCAGGGTTCCAGCAGGTCTGGGACAACAGGTCTGGGACAGGTAGCACGTCCGGTGAAC
GGGTCAGGGTTCCATAGCTGCAGGCAGAACAGTTGGAACTGGAGCAGCAGCACGGCCAGGTGAACTGGGGACAGCAAGGA
GTCATCAAGCCAGGTAGTCCTGAGGCATGGTCCTAGGGCTCAGGTCCTCCGAGAGAGAGAAAGAGTAAGAGAGAATTAGA
GAGAGCATATTTAAATTCACACAGGACACCGGAAAAGACAAGAGAAATACTCCAGATGTGACAGACTGACCCTTGCCCCC
CGACACATAAACTACTGCTGCATAAATACTGGAGGCTGAGACAGGAGGGGTCAGGAGACACTGTGGCCCCATCTGATGAA
ACCCCCGGACAGGGCCAAACAGGTAGGATATAACCCCACCCACTTTGCCAAAGCACAGCCCCCACACCACTGGAGGGATA
TCTCCAACCACCAACATACCATCCCGGGACAAGGCCGAGTATAGCCCACAAAGATCTCCGCCACGGCACAGCCCAAGGGG
GGGGGCGCCAACCCAGACAGGAAGACCACGTCAGTGACTCAACCCACTCAAGTGACGCACCCCTCCCAGGGACGGCATGG
AAGAACACCAGTAAGCCAGTGACTCAGCCCCCGTAATAGGGGTAGAGGCAGAGAATCCCAGTGGAAAGAGGGGAAACCGG
CCAGGCAGAGACAGCAAGGGCGGTCCGTTGCTCCAGTCTTTCCGTTCACCTTCACACCCCTGGGCCAGACTACACTTAAT
CATAGGACCTACTGAAGAGATATGTCTTCAGTAAAGACTTAAAGGTTGAGACTGAGTCTGCGTCTCTCACATGGGTAGGC
AGACTATTCCATAAAAATGGAGCTCTCTAGGAGAAAGCCCTGCCTCCAGCTGTTTGCTTAGAAATTCTAGGAACAATTAG
GAGGCCCGCGTCTTGTGACCGTAGCGTACGTGTAGGTATGTACGGCAGGACCAAATCGGAAAGATAGGTAGGAGCAAGCC
CATGTAATAATTTGTAGGTTAGCAGTAAAACCTTGAAATCAGCCCTTGCCTTAACAGGAAGCCAGTGTAGGGAGGCTAGC
ACTGGAGTAATATGATCCAATTTTTGGGTTCTAGTCAGGATTCTAGCAGCCGTATTTAGCACTAACTGAAGTTTATTTAG
TGCTTTATCCGGGTAGCCGGAAAGTAGAGCATTGCAGTAGTCCAACCTAGAAGTAACAAAGGCATGGATTAATTTTTCTG
CGTCATTTTTGGACAGAAAGTTTCAGATTTTTGCAATGTTACGTAGATGGAAAAAAGCTGTCCTTGAAACAGTCTTGATA
TGTTCTTCAAAAGAGAGATCAGGGTCCAGAGTAACGCCGAGGTCCTTCACAGTTTTATTTGAGACGACTGTACAACCATC
CAGATTAATTGTCAGATTCAACAGAAGATCTCTTTGTTTCTTGGGACCTAGAACAAGCATCTCTGTTTTGTCCGAGTTTA
AGAGTAGAAAGTTTGCAGCCATCCACTTCCTTATGTCTGAGACACAGGCTTCTAGCGAGGGCAATTTTGGGGCTTCACCA
TGTTTCATTGAAATGTACAGCTGTGTGTCATCCGTATAGCAGTGAAATTTAACATTATGTTTTCGAATGACATCCCCAAG
AGGTAAAATATATAGTGAAAACAATAGTGGTCCTAAAACGGAGCCTTGAGGAACACCGAAATTTACAGTTGATTTGTCAG
AGGACAAACCATTCACAGAGACAAACTGATATCTTTCCGACGTGTAAGACCTAAACCAGGCCAGAACTTGTCCATGCAGA
CCAATTTGGGTTTCCAATCTCTCCAAAAGAATGTGGTGATCGATGGTATCAAAAGCAGCACTAAGATCTAGGAGCATGAG
GACAGACGCAGAACCTCGGTCTGAAGTCATTAAAAGGTAATTTACCACCTTCACAAGTGCAGTCTCAGTGCTATGATGGG
GTCTAAAACCAGACTGAAGCGTTTCGTGTACATTGTTTGTCTTCAGGAAGGCAGTGAGTTGCTGCGCAACAGCTTTTTCT
AAAATGTTTGAGAGGAATGGAAGATTCGATATAGGCCAATAGTTTTTTTATAATTTCTGGGTCAAGATTTGGTTTTTTC
AAGAGAGGCTTTATTACTGCCACTTTTAGTGAGTTTGGTACACATCCGCTGGATAGAGAGCCGTTTATTATGTTCAACAT
AGGAGGGCCAAGCACAGAAAGCAGCTCTTTCAGTAGTTTAGTTGGAATAGGGTCCAGTATGCAGCTTGAAGGTTTAGAGG
CCATGATTATTTTCATCATTGTGTCAAGAGATATAGTACTAAAACACTTTAGTGTCTCCCTTGATCCTAGGTCCTGGCAG

GGTTGTGCAGACTCAGGACAACTGAGCTTTGGAGGAATACGCAGATTTAAAGAGGAGTCCGTAATTTGCTTTCTAATGAT
CATGATCTTTTCCTCAAAGAAGTTAATGAATTCATCACTGCTGAAGTCAGAGCCATCCTCTCCTGGGGAATGCTGCTTTT
TAGTTAACTTTGCGACAGTATCAAAAAGAAATTTCGGATTGTTCTTATTTTCCTCAATTAAGTTGGAAAAATAGGATGAT
CGAGCAGCAGTGAGGGCTCTTCGATACTGCACGGTACTGTCTTTCCAAGCTAGTCGGAAGACTTCCAGTTTGGTGTGGCG
CCATTTCCGTTCCAATTTTCTGGAAGTTTGCTTCAGAGCTCGTGTATTTTCTGTATACCAGGGAGCTAGTTTCTTATGAC
AAATGTTTTTAGTTTTTAGGGGTGCAACTGCATCTAGGGTATTGCGCAAGGTTCAATTGTAGAGTAGCATAGTGTCCTAC
TGTATGTACTGTAACCCATGTTAACATGTAGAGTAGCATAGTGTCCTACTGTATGTACTGTAACCCATGTTAACATGTAG
AGTAGCATAGTGTCCTACTGTATGTACTGTAACCCATGTTTACATGTAGAGTAGCATAGTGTCCTACTGTATGTACTGTA
ACCCATGTTTACATGTAGAGTAGCATAGTGTCCTACTGTATGTACTGTAACCCATGTTTACATGTAGAGTAGCATAGTGT
CCTACTGTATGCACTGTAATCCATGTCTACATGTAGAGTAGCATAGTGTAAAACTCACTGTAGAACAAGACATATTGGAT
AGACACCATACCTGGACGTATTGGTGCCGGAGTTCCTTGACCCGCTCACTGTTCCTTTCAAAAGGAACTGTGTACAACTG
TTTCATTCCAACTCTGTGTTTGGTCAGAGTCCGAGAAATGGTAGTCAGGCTGATGCTTTCCACATTGTGAAATACCAGGT
TGTCCTCTACAATTCTGGTCTGAATTTCTCTCAGTTTTATTGCATTGTCAGCAATGACCAATCTTACAATGGCATGCTCT
TGGTCTTCAATGAGGAGCTTTCCTCTTCCCCAAGAGGGAGGAAGACATTGTGTCCTTCATAGGAACAAAAATACAACATA
TGTATTTGTATTGGCACTGGTGGTCTACAGTTACAGTGGGACATAGCAACAGTAATGATAGAAAAAGCCCTTGTGAAATG
CATTACTTACCTGTTGGTTTGTTGAAAATTCTGGATAATGGAAGCCACGGTTGACCGTCTGAGATTAGGCTGGACTCTTT
CACCAGTCTCTCTCAGTGATAGAACATGGTATTAGGCTGGACTCTTTCACCAGCCTCTCTCAGTGATAGACCATGGTATT
AGGTTGGACTCTTTCACCAGTCTCTCTGTGATAGACCATGGTATTAGGCTGGACTCTTTCACCAGCCTCTCTCAGTGATA
GACCATGGTATTAGGCTGGACTCTTTCACCAGCCTCTCTCAGTGATAGACCATGGTATTAGGCTGGACTCTTTCACCAGC
CTCTCTAAGTGATAGACCATGGTATTAGGCTGGACTCTTTCACCAGCTTCTCTCAGTGATAGACCATGGTATTAGACTGG
ACTCTTTCACCAGTTTCTCTCAGTGATAGACCATGGTATTAGGCTGGACTCTTTCACCAGCCTCTCTCAGTGACAGACCA
TGGTTTATCACATGGTCTTTGATGGTGGATCTTATTTCATCAATGACAACAGCTCTTACTCTCCTTTGAACACCACCACG
CATTCTTACTCCTCTCCCCCTACCTCTCCTTCTCCCTTGTCCTGGTCCAACTACTCCTCTGCCTGGTCCAACTACTTCTC
TGCCGGTCCAACTACTCCTCTGCCTGGTCCAACTACTCCTCTCCCTTGTCCAGACATTATTTTTTCTCTCTCTGTTCCAC
TGTGTTGTTCTGTTTCCACATTAAACAAAATTAATCCAAAGAGTCCCCTTTTTAGATCATGCATTTCTGTTTTACAGGTT
AGAAATGAAAGCACTTTATCTAGTTCCTGAACGAATCCAGATAATACCATGATTTATTTTTAATGAATAATGGTGAGTGA
GGTGGTTTGAGGCTATAACAAAACATGCTAACCTCTCACTTTCTTTTATCTGCAACACATTTATAATGCAATGAGAGAAT
ATGCCGAAACCAAGCACCCACGTCATGTCTTTTGATGTCATATCACCTTACCCTCATTCACTTCTGTCAAGAACACTTGT
GAAGTTTGTATATTTAACTGGGTGTTCCTCTTTACATACTATACATGTTTGGGTGAAGCAAATACGATGTATGCTCACTG
ACTTAAATATACTGAACAAAAGTATAAACGCAACGTGCAACAATTTCAAAGATTTTACTGAGTTACAGTTCATATAAGGA
AATCAGTCAATTGAAGTAAATTCATTAGACCCTAGTCTATGGATTTCACATGACTGGGAATACAGATATGCATCTGTGGG
TCATAGATTCCATTTAAAAAAAAGTAGGGGTGTGGATCAGAACCAGTCAGTATCTGTTGTGACCACCATTTGCCTCATGC
AGCACGACACATCTCCTTCGCATAAAGGGGATCAGGCTGTTGATTGTGGCCCGTCGAATGTTCTCCCACTCCTCTTCAAT
GGCTGTGTGAAGTTGCTGGATATTGGCGGGAACTGGAACACGCTGTAATACGTGTCGATTCAGAGCATCCCAAACATGCT
CAATGGGTGACATGTCTGGAGAGTATGTAGGCCATTTTATTAGTATTTTTTTATTTAACTTTTATTTAACTAGGCAAGTC
AGTTAAAAACAAATTATTATTTTCAATGACAGCTAAAGGTCTCCTGCAAGGACGGGGGCTGGGATTAAACATTTAAAATA
AATATAGGAAAAAAACACATTTCAAAACGAGAGAGACAACACAGCACTACATAAAGAGAGACCTAAGACAACAACACATT
ACAACACACCATGGTCGCAACACAACATGACAATAACATGGTAGCAGTACAAAACACAGGTCAAACGTTAATCTTTTTAT
AAGTAGGCAAGTCAGTTAAGAACAAATTCCAATAACAGCGGGTTACCTGCCTTGGTCAAGGGCAGAACGACAGATTTTTA
CCTTGTCAGCTCGGGGATTTGATCTTGCAACCTTTCGGTTACTAGTCCAATGCACTAACCACTAGGCTACTTGTCGCCCC
TTTATTGTGAAATACCAGGTTGTCCTCTACAATTCTGGTCTGAATTTCTCTCAGCTGTATTGCATTGTCAGCAATGACCA
ATCCTACAATGGCATGCTCTTGGTCTTCAATGAGGAGCTTTCCTCTTCCCCAAGAGGGAGGAAGAGATTGTGTCCTTCAT
AGGAACAAAAATACAACATATGTATTTGTATTGGCACTGGTGGTCTACAGTTACAATTAATCTCCTCCTTTCCCCCTTCT
GATAACCAGGTGGCGAATCGCATCTCTGCATGTCTGGCAGACATATCAGTGTGGATGACGGATCACCACCTCAAGCTGAA
CCTCGGCAAGACGGAGCTGCTCTTCCTCCCGGGGAAGGACTGCCCGTTCCATGATCTCGCAATCACGGTTGACAACTCCA
TTGTGTCCTCCTCCCAGAGTGCTAAGAACCTTGGCGTGACCCTAGACAACACCCTGTCGTTCTCCGCTAACATCAAGGCG
GTGACCCGATCCGGTAGGTTCATGCTCTACAACATTCGCAGAGTACGACCCTGCCTTACACAGGAAGCGGCGCAGGTCCT
AATCCAGGCACTTGTCATCTCCCGTCTGGATTACTGCAACTCGCTGTTGGCTGGGCTCCCTGCCTGAGCCATTAAACCCC
TACAACTCATCCAGAACGCCGCAGCCCGTCTGGTGTTCAACCTTCCCAAGTTCTCTCACGTCACCCCACTCCTCCGCACA
CTCCACTGGCTTCCAGTTGAAGCTCGTATCTGCTACAAGACCATGGTGCTTGCCTACGGAGCTGTGAGGGGAACGGCACC
TCCGTACCTTCAGGCTCTGATCAGTCCCTACACCCAGACAAGGGCACTGCGTTCATCCACCTCTGGCCTGCTGGCCTCCC
TACCTCTGCGGAAGCACAGTTCCTGCTCAGCTCAAAACTGTTCGCTGCTCTGGCACCCCAATGGTGGAACAAGCTCCCTC
ACGACGCCAGGACAGCGGAGTCAATCACTGCCTTCCGGAGACACCTGAAACCCCACCTCTTTAAGGAATACCTGGGATAG
GATAAAGTAATCCTTCTAACCCCCCCCCCCCCCCCCCCCAAGGATGTGTGTGCTTTGGGGACCTTTAACAGAATGTGACTG
GCAGAATGGGTGTTGTATGTGGAGGATGAGGGCTGCAGTATATATCTCAGATAGGGGGAGTAAGGCCTAAGAGGGTTTTA
GAAATAAGCATCAACCAGTGGGTCTTGCGACGAGTATAAAGAGATGACCAGTTTACAGAGAAGTATATACTGCAGTGACG
TGTCCTATAAGGAGCATTGGTGGCAAATCTGATGGCCGAATGGTAAAGAACATCTAGCCCTTGAGAGCACCCTTACAGTA
CCTGCCGATCTATATATTATGTTTCTGTAATCTAGCATGGGTAGGATAGTCATCTGAATCAGGGTTAGTTTGGCAGCTGG
GGTGAAAGAGGAGCGATTACGATAGAGGAAACCAAGTCTAGATTTAACATTAGCCTGCAGCTTTGATATGTGCTGAGAAA
AGGACAGTGTACCCTCTAGCCATACTCCCAAGTACTTGCATGAGGTGACTACCTCAAGCTCTAAAACCATCAGAGTTAGT
AATCACACCTGTGGGGAGAGGGGCATTCTTCTTACCGAACCACTTGACCTTTGTTTTGGAGGTATTCAGAACAAGGTTAA
GGGTAGAGAAAGCTTGTTGAACACCAAGAAAACTTTGTTGTAGAGCATTTAACACAACATCCGGGGAGGGGCCAGCTGAG
TATAAGACTGTATCATCTGCATATAAATGGATGAGAGAGATTCTTACTGCTTGAGCTATGTTGTTGATGTAAATTGAGAA
GAGCGTGGGGCCTAGGATTGAGCCTTGGGGTACTCCCTTGATGACAGGCAGTTACTGAGACAGCAGATTTTCTGACTTTA
CACACTACACTATTTGAAATGGTTAGTTAGCAAACCAGGACAAAGGCCCCTCAGAGACACCAATACTCCTTAGCTGGCCC
ACAGGAACGGAATGGTCTACCCTATCAAAAGCTTTGGCCAAGTCAATACAAATAGCAGCACAACATTGCTACGAATCAAG
GGCAATGGTGACATTATTGAGGACCTTTAAGGTTGCAGTGACACATCCATAACCTAAGCAGAGCAATTCCTCTTTTGGCC
GCTTTTCCTTCCAGTTCTCTGCTGCCAATGACTGGAATGAATTGCAAAAATCACTGAAGCTGGAGACTCTTACCTCCCTC
ACTAGCTTTAAGCACCAGCTGTCTGAGCAGCTCACAGATCACTGCACCTGTACATAGCCCATCTGTAAATATCCCATCCA

ACTACCTCATCCCCATACTGTATTTATTTATTTATCTTGCTCCTTTGCACCCCTGTATCTCTACTTGCACATTCATCTTC
TGCACATCAATCTCTCCAGTGTCTAATTGGTATATTGTAATTACTTCGCCACTATGGCCTATTTATTGCCTTACCTCCCT
TTTCTTACCTATATAGTATATAGATTTTCTTCAACTGTATTATTGACTGTGAGTTTTGTTTATTCAATGTCTAACTCTGT
GTTGTTGTATGTGTCGAACTGCTTTGCTTTTTTCTTGGGTAGGTCGCAGTTGTAAATGAGAACTTGTTCTCAACTGGCCT
ACCTGGCTAAATAAAGGTGAAATAAAATTAAAATAAATAAATAAAAAGAAACCAGATTGCATACCAGACAGAATACTGTA
GCGACCCGCACAGACAGCTGTGGGTTATGTGTTAGGCTATTAGTGGGTTGTGTTTACTTACCAGTGTTTGCTGGGTCATG
CCAATCAACCTGCTATCTGCCAATCACGGGAATGCCAGGAATGTTCTGATGCCGGGCATCCTGGTGGTTGGCGGAGTGGC
GTGGAGGGGGGTTGGGCAGGGGGATGGAGCATTGGAAGTTAAGACCGGGTTTAGCCATTGTTCGCTCTCTTACGTCTGGG
CTTCACAAGAGAAGGTCACGGTTGGTTTGTAGGGTACCTTTCATTTATTTGGCGTGGGCTACGGCCAAACAGTAGCCTGT
GTTAAGTTGGGTTAATAAACCGTCAATTCGTTAACTCCATCCTCTGTCTGGACAATTGTTCCTTTATGATCTAGTCAGGG
CATTACAATACTATAGACATCAAGAAAGCCAGTCAGTTGACAGGGCAAAATAGAAATAGGCCTCTAACAGTTAGGATCAG
CTTGATCTCTCCCTTTAAATAAAGGACGAACCGTGGCTGCCTTCCAAGCAATGGGAACCTCCCCAGAAAGGAGAGACAGG
TTAAAAAGGTTGGAGATAGGCTTGGCAATGATAGGGGCAGCAACCTTAACCTGTTGAGGACAGACGTTCTGCTAGCGGAA
CCCCGTTCCGCCTGCGGAACCCCTAGCCAACAGCCAATGGCATCGCATGGCGCGAAATACAAAACCAACTAAAATACCAC
AATTCAATTTTCTCAAACAATCAACTATTTTACACCATTTTAAAGATAAGACTCTCGTTAATCTAACCACATTGTCCGAT
TTCAAAAAGGCTTTTCAGCGAAAGCAAAACATTAGATTATGTTAGGAGAGTACCCAGCCAGAAATAATCACACAGCCATT
TTCAAAGCAAGCATATATGTCAAATAAACCCAAACCACAGCTAAATGCAGCACTAACCTTTGATGATCTTCATCAGATGA
CACTCCTAGGACATTATGTTATACAATACATGCATGTTTTGTTCAATCAACTTCATATTTATATCAAAAAACAGCTTTTC
ACATTAGCATGTGATGTTCAGAACTAGCATACCCACCGAAAACTTCCGGTGAATTTACTAAATTACTCATGATAAACGTT
CACAAAAAACATAACAATTATTTTAAGAATTATAGATACAGAACTCCTTCATGCAATCGCGGTGTCAGATTTTAAAATAG
CTTTTCGGCGAAAGCATGTTTTGCAATATTCTGAGTACATAGCTCGGCCATCACGGCTAGCTATTTTGACACCCACCAAG
TTTGGGGCTCACCAAACTCAGAATTACTATTAGAAAAATTGGATTACCTTTGCTGTTCTTCGTCAGAATGCACTCCCAGG
ACTTCTACTTCAACAACAAATGTTGTTTTGGTTCCAAATAATCCATAGTTATATTCAAATAGCTCCGTTTTGTTCATGCG
TTCAGGTCACTATCCGAAGGGTGACGCGCGAGCGCCTTTCGTGACCAAAAAATTCAAAATATTCCATTACCGTACTTCGA
AGCATGTCAAACGCTGTTTAAAATCCATTTTTATGCTATTTTTCTCATAAAATAGCGCTAATATTCCAACCGGGCAACGT
TGTATTCATTCAAAGGCTGAAAGAAAAAAATTGAGTAGTCTCGTGACCGCGCATCTCCAGTGTCACTGTCCCCAGGCTGA
CCACTTACAAATTCTCCTGCTGTTCTTCGCCCAGAGACAGCAGACACCCCATTCCACTTTCTGGCGGCTTTAGAGAGCCA
ATGGAAGCCTTAGAAAATGTCATGTTACAGCACATATGCTGTATTTTCGATAGAGATGCAACAGAAGGACAACAAATTGT
CAGACAGGGCACTTCCTGTATGGAATCTTCTCAGGTTTTGGCCTGCCATATGAGTTCTGTTATACTCACAGACACCATTC
AAACAGTTTTAGAAACTTTAGATTGTTTTCTATCCAAATCCACTAATATTATGCAGATTCTAGTTTCTGGGCAGGAGTAG
TAACCAGATTAAATCGGGTATGTTTTTTATCCGGCTGTGAAAATACTGCCACCTATCCCAAAGAAGTTAAAGAAGAAAGG
GTCTAAGCCATCTGACCCAGATGTTTTTTTTGGGTCAAGTTTCAGGAGCACCTCGGACTCAGTGACTGCCAGCAGGGAGA
AACTTTGTCAAGGGGCAGGGGAAAAAGAGGGAGAAGCATCGGGAATAGTCGCATCAGAAGAGGTGGGAGAGGAGGACATT
TTGGACGGTCAAGGAGGCATGGCTGAGTCAAATAGGAATCCTGACTTAATAAAGTGGTGATTAAAGAGCTCAGCAATGTG
CTCCTTGTCAGTAAAAACCACATCATCAACATTAAGGGACATGGGCATCTGTGAAGAGGAGGGTTTATTCTCCAGGTCTT
TAACCTTTTCCCAGAACTTCTTGGGGTTAGACCCACAGAGAGAGAACTGCTCCTTAAAGTAACTTTGGCCTTAGTGTACT
TATTACTCATTTGCCTGAACGAGAGCCAGTCAGCCTGAGTATGCGTGTGCCGAGCCTTTTGCCAAATGCAATTCTTGAGA
TGGAGTAACTCTGCAAGATCATGGTACAACCAGACCTTGAACTGTTTTTAATTCTCATTTTCTTAATGGGGGCGTGTTTG
TTAACAATACCACTGAAAATATAAAAAATAAAGGTCCAAGTGTCTGACAGAGGGGATCAAGCTGATTCTATACAATTTTA
CAGAGGCCAGTTCATGAAGGAAGGCTTGCTCCTTAAAGTTTTTTAGCAAGTGTCTATGACAAATCAGGACAGGTTGTTTC
ACTAAGCAGCCATTACAAACACAGGCCAGCAATTCAAATTGTTTTCGGACAGACTTGGTGGAGACAACCGAGCACTGAAG
GTGATTCTTGGTAAAGATTGCAACTTCCCCACATTTGGAAGATCTGTCTTGCCGAAAAAGGTTATAACCAGAAAGGTCAG
TATTCAAAACACTCTTCCTTAACCACGTCTCAGTAATGACCAACACATCTGGATTGGAGCTGTGAACCCACACTTTCAAT
TGATCCATTTTAGGTAATAAGCTTCTAGCGTTAATGTGCAGAAAACCCAGGCTTTTACGAGAGCAGAAATCAGTGAAGCA
GATATCAGAGCACAAGTCAGAATTGGGGCTAGCAACAGTAGATGGGCCAGGGTGTACATGCACATTTACAGATATCATCA
ACAGTAATACAATCAAGGCACGGCATAGTACAGGGAGAGCTCTGCAGTGTTGATTTATGACATCTGAATGTGCATCAGGT
GGCAACAAGATCATATTATACATCAGGTAACATGAATACAAAGCCAGTGAGAGGTGGTTAGAATAGGATGGGAGGCCAAA
AGTCTGTGTAACCAATAGAGAGTCAGTCCCAAGTGTGGGAACAAACAGTCTGTCCCACGGTTGGGTAAAGAAAGTTCGTA
GTTAACAAAGCATGCAGGAGTCATGAGGAAAATAGCAAAATGCATAAGAAAAAAATTATATATATAACCACTTGGGACTA
GCCATTGTAAGTTAAGAGTCATTCACCCCAACAGTGTACCATGGAAGAACTGGGTCATTTTCAGCTTCTGGAAATTGTTA
TATTTTTGGTGCTGATGTGGTAAGACAGTTTAAATAAAATCACAAGCCATTTTATAAGTTATATACAAGTCCTAAAATGG
ATGTACCAATCCCTGATTTCCCCTTTTAAATAATATTTCCTCCCTGACCCCCCACCCCCCCCTCTCACACATAGTTGTATG
ACTAGATATTAAACTATCCTCAGACTCTCAGACTGTACAGTTCCATTTGTCCAGTGTCTCTTGAACATTTCAGCCATGTG
ATGCTGTACCTTCAGTAATAAACACTAGAGGGCATTCTCACTTATAAAAAGGATGAGGCAGCTACACTCTCCTCCATATG
TATTTGGACAGCGAAGCCGAAATGTAAAATTTGGCTCTACATTTGTATTTTAGATTAAATGTTTAATGAGGCAACAGAAG
AGGATGTCACCTTTTTGAGGGTATTTTCATGCATATCTGCATGAAATATCTGCATATTACAGTTTAGAAATGAAAGTACT
TTGTCTAGTCGCTGAACACTTCTAAATGAATCCTGATAATGCCATGATTTCTTTTGAATGAATAGTGGTGAGTGAGAAGG
TTAGAGGCTATAACACCCAATAATGGGAGTTTAGCAACAACAAAAAAATGTGTGTTTGTGTGTGTGTGTGTGTGTGTGTGT
GTGTGTGTGTGTGTGTGGGGGGGGGGGGGGGGGGGTGATCTTTGTGCCTCTGTATCCTTCTCACATAATTATTAACAAATA
ATTCCTGATTATTCATAATCATGGTAGCATCCACGTTAATGTAGAAGTGTTCAGAAATATTTTATATTCTTACTTACAAT
GAAAGTGACTCCAAAATGGCAATACATTATTCACCAATCGACAACAGTATATCTTCTACTGCTACTTAAAGCTATTTTGA
CTCACATATACTGTACACTTGAATCTTAATTCCAACCCTCAGATGTCCACATGCTAAGCCATCTTTCCCAGAACACTACA
ATTTTCCTTTTTCCTTTTGCAATACATCCATCTATCTTACTGTGATGGATTAATGCGTCATTTCATTTGTATATCAGTTC
ATAAATTCGATGCCATGGTATTGGGACATCAAATCTCTTCCCAACTATCTTGAATTTTATGTGGCATAGTTGTCAAACCT
CTTGACCTTAAGTGAAACTGATACATTTTACGATTTATACTAGTCATTTTAAACCATGCATGGCTTTTCATTGGAGACAG
ACAAACCAATTCATTCCTATCTCTTTTGAGTAATTGCTTCTTCCATTTTTGTGGCAGAGATGTGATGAGTTGTTTTACTT
TTGTATTGAACATACTGTACATTTCCATACAACTTTGTTAATTGTTATTGTGCCAATTTTACTGTCCTTATTTACAATAT
CATTCATAATGATACCTTCCTTATACATTTTTTTCCAATAAATGGGTCTTTCCTCTATCGGTATAATGGAATTTAGCTGT
AATATTTGTTCTGCCTCTTCTGGAGGGTGAAATTGGAATTGTAACCAACTCTGTATGGCTTCATTTAAAAAGGAGGATAC

TTTAAATAGGGTTTCATTGTCAAGCCACCTGAAATGGTTGGTTTTAAATCTGTATAATGGCAAAAGGCCCTATTTTGAAC
ATTGGATGTGCCTCTCTTAGGCTACGTTTACACAGGCAGCTCAATTCTGATCTTTTTTTCACTAATTGGTATTTTGACCA
ATCAGATCAGCTTTGAAAAAGATCTGGTGTGAAAAGTTTGATGTGATTGGTCAAAAGACAAATTATTGCAAATAATATAC
ACTTGAGGTCTTGTGAGGCGTCTGTTTCTCAAACTAAACAGTCGAAAGTACTTGTCCTCTTGCTGAGTTGTACACCTAGG
CCTCCCACTCCTCTTTCTATTCTGGTTAGAAACAGTTTGCGCTGTTCTGTGAAGGGAGTAGTACACAGCGTTGTACCAGA
TCTTCAGTTTCTTGGCAATTTCTCGCATGGAATAGCCTTCATTTCTCAGAACAAGAATGGACTGATGAGTTTAAGAAGAA
AGTTTTTTGTTTCTGGCCATTTTCAACCTGTAATTGAACCCACATATGCTGATGCTCCAGATACTCAACTAGTCTAAAGA
AGGCCTGTTTTATTGCTTCTTTAAACAGCACAGCAGTTTTCAACAGTGCTAACATAACTGCAAAAGGGTTTTCTAATGAT
CAATTTGCCTTTTAAGTTGATAAACTTGGATTAGCTAACACAACGTGCCATTGGAACACAGGAGTGATGGTTGCTGATAA
TGGGCCTCTGTACGCCTATGTAAATATTCCATAAAAATCTGCCGTTTCCAGCTACAATAGTTATTTACAACATTAGCAAT
GTCTACACTGTATTTCTGATCAATTTGATGTTAATTTAATGGACCAAAAGATTGATTTTCTTTCAAAAACAAGGACATTT
CTAAGTGACCCCAAACTTTTGAACGGTAGTGTATATCAGAATTGGGCTGCCAGTGTAGACGCAGCCAGAGTTGACTGCTG
GAAAACCAGTTTGGATTTAAATATAATTTCGGTATAATGGGGGTTTTGAGAGGTTTAATGCCTTAATATTTAATTGCGTA
CATTGATACTTTTGTCTCTAATCCATACATTTTAAGCCCCTTAATATTATCATTAGCTCAGATTTTGATAACTAACAGTT
CAATAGCCATAATAAATAAGTATGGTGACAGAGGGCTTCCTTCTTTTACTCCTCTTGACAATTCAAAGTTCTCAGAGAAA
TAANNNNNNNNNNNNNNNNNNNNNNNNNNNNNNNNNNNNNNNNNNNNNNNNNNNNNNNNNNNNNNNNNNNNNNNNNNNNN
NNNNNNNNNNNNNNNNNNNNNNNNNNNNNNNNNNNNNNNNNNNNNNNNNNNNNNNNNNNNNNNNNNNNNNNNNNNNNNNN
NNNNNNNNNNNNNNNNNNNNNNNNNNNNNNNNNNNNNNNNNNNNNNNNNNNNNNNNNNNNNNNNNNNNNNNNNNNNNNNN
NNNNNNNNNNNNNNNNNNNNNNNNNNNNNNNNNNNNNNNNNNNNNNNNNNNNNNNNNNNNNNNNNNNNNNNNNNNNNNNN
NNNNNNNNNNNNNNNNNNNNNNNNNNNNNNNNNNNNNNNNNNNNNNNNNNNNNNNNNNNNNNNNNNNNNNNNNNNNNNNN
NNNNNNNNNNNNNNNNNNNNNNNNNNNNNNNNNNNNNNNNNNNNNNNNNNNNNNNNNNNNNNNNNNNNNNNNNNNNNNNN
NNNNNNNNNNNNNNNNNNNNNNNNNNNNNNNNNNNNNNNNNNNNNNNNNNNNNNNNNNNNNNNNNNNNNNNNNNNNNNNN
NNNNNNNNNNNNNNNNNNNNNNNNNNNNNNNNNNNNNNNNNNNNNNNNNNNNNNNNNNNNNNNNNNNNNNNNNNNNNNNN
NNNNNNNNNNNNNNNNNNNNNNNNNNNNNNNNNNNNNNNNNNNNNNNNNNNNNNNNNNNNNNNNNNNNNNNNNNNNNNNN
NNNNNNNNNNNNNNNNNNNNNNNNNNNNNNNNNNNNNNNNNNNNNNNNNNNNNNNNNNNNNNNNNNNNNNNNNNNNNNNN
NNNNNNNNNNNNNNNNNNNNNNNNNNNNNNNNNNNNNNNNNNNNNNNNNNNNNNNNNNNNNNNNNNNNNNNNNNNNNNNN
NNNNNNNNNNNNNNNNNNNNNNNNNNNNNNNNNNNNNNNNNNNNNNNNNNNNNNNNNNNNNNNNNNNNNNNNNNNNNNNN
NNNNNNNNNNNNNNNNNNNNNNNNNNNNNNNNNNNNNNNNNNNNNNNNNNNNNNNNNNNNNNNNNNNNNNNNNNNNNNNN
NNNNNNNNNNNNNNNNNNNNNNNNNNNNNNNNNNNNNNNNNNNNNNNNNNNNNNNNNNNNNNNNNNNNNNNNNNNNNNNN
NNNNNNNNNNNNNNNNNNNNNNNNNNNNNNNNNNNNNNNNNNNNNNNNNNNNNNNNNNNNNNNNNNNNNNNNNNNNNNNN
NNNNNNNNNNNNNNNNNNNNNNNNNNNNNNNNNNNNNNNNNNNNNNNNNNNNNNNNNNNNNNNNNNNNNNNNNNNNNNNN
NNNNNNNNNNNNNNNNNNNNNNNNNNNNNNNNNNNNNNNNNNNNNNNNNNNNNNNNNNNNNNNNNNNNNNNNNNNNNNNN
NNNNNNNNNNNNNNNNNNNNNNNNNNNNNNNNNNNNNNNNNNNNNNNNNNNNNNNNNNNNNNNNNNNNNNNNNNNNNNNN
NNNNNNNNNNNNNNNNNNNNNNNNNNNNNNNNNNNNNNNNNNNNNNNNNNNNNNNNNNNNNNNNNNNNNNNNNNNNNNNN
NNNNNNNNNNNNNNNNNNNNNNNNNNNNNNNNNNNNNNNNNNNNNNNNNNNNNNNNNNNNNNNNNNNNNNNNNNNNNNNN
NNNNNNNNNNNNNNNNNNNNNNNNNNNNNNNNNNNNNNNNNNNNNNNNNNNNNNNNNNNNNNNNNNNNNNNNNNNNNNNN
NNNNNNNNNNNNNNNNNNNNNNNNNNNNNNNNNNNNNNNNNNNNNNNNNNNNNNNNNNNNNNNNNNNNNNNNNNNNNNNN
NNNNNNNNNNNNNNNNNNNNNNNNNNNNNNNNNNNNNNNNNNNNNNNNNNNNNNNNNNNNNNNNNNNNNNNNNNNNNNNN
NNNNNNNNNNNNNNNNNNNNNNNNNNNNNNNNNNNNNNNNNNNNNNNNNNNNNNNNNNNNNNNNNNNNNNNNNNNNNNNN
NNNNNNNNNNNNNNNNNNNNNNNNNNNNNNNNNNNNNNNNNNNNNNNNNNNNNNNNNNNNNNNNNNNNNNNNNNNNNNNN
NNNNNNNNNNNNNNNNNNNNNNNNNNNNNNNNNNNNNNNNNNNNNNNNNNNNNNNNNNNNNNNNNNNNNNNNNNNNNNNN
NNNNNNNNNNNNNNNNNNNNNNNNNNNNNNNNNNNNNNNNNNNNNNNNNNNNNNNNNNNNNNNNNNNNNNNNNNNNNNNN
NNNNNNNNNNNNNNNNNNNNNNNNNNNNNNNNNNNNNNNNNNNNNNNNNNNNNNNNNNNNNNNNNNNNNNNNNNNNNNNN
NNNNNNNNNNNNNNNNNNNNNNNNNNNNNNNNNNNNNNNNNNNNNNNNNNNNNNNNNNNNNNNNNNNNNNNNNNNNNNNN
NNNNNNNNNNNNNNNNNNNNNNNNNNNNNNNNNNNNNNNNNNNNNNNNNNNNNNNNNNNNNNNNNNNNNNNNNNNNNNNN
NNNNNNNNNNNNNNNNNNNNNNNNNNNNNNNNNNNNNNNNNNNNNNNNNNNNNNNNNNNNNNNNNNNNNNNNNNNNNNNN
NNNNNNNNNNNNNNNNNNNNNNNNNNNNNNNNNNNNNNNNNNNNNNNNNNNNNNNNNNNNNNNNNNNNNNNNNNNNNNNN
NNNNNNNNNNNNNNNNNNNNNNNNNNNNNNNNNNNNNNNNNNNNNNNNNNNNNNNNNNNNNNNNNNNNNNNNNNNNNNNN
NNNNNNNNNNNNNNNNNNNNNNNNNNNNNNNNNNNNNNNNNNNNNNNNNNNNNNNNNNNNNNNNNNNNNNNNNNNNNNNN
NNNNNNNNNNNNNNNNNNNNNNNNNNNNNNNNNNNNNNNNNNNNNNNNNNNNNNNNNNNNNNNNNNNNNNNNNNNNNNNN
NNNNNNNNNNNNNNNNNNNNNNNNNNNNNNNNNNNNNNNNNNNNNNNNNNNNNNNNNNNNNNNNNNNNNNNNNNNNNNNN
NNNNNNNNNNNNNNNNNNNNNNNNNNNNNNNNNNNNNNNNNNNNNNNNNNNNNNNNNNNNNNNNNNNNNNNNNNNNNNNN
NNNNNNNNNNNNNNNNNNNNNNNNNNNNNNNNNNNNNNNNNNNNNNNNNNNNNNNNNNNNNNNNNNNNNNNNNNNNNNNN
NNNNNNNNNNNNNNNNNNNNNNNNNNNNNNNNNNNNNNNNNNNNNNNNNNNNNNNNNNNNNNNNNNNNNNNNNNNNNNNN
NNNNNNNNNNNNNNNNNNNNNNNNNNNNNNNNNNNNNNNNNNNNNNNNNNNNNNNNNNNNNNNNNNNNNNNNNNNNNNNN
NNNNNNNNNNNNNNNNNNNNNNNNNNNNNNNNNNNNNNNNNNNNNNNNNNNNNNNNNNNNNNNNNNNNNNNNNNNNNNNN
NNNNNNNNNNNNNNNNNNNNNNNNNNNNNNNNNNNNNNNNNNNNNNNNNNNNNNNNNNNNNNNNNNNNNNNNNNNNNNNN
NNNNNNNNNNNNNNNNNNNNNNNNNNNNNNNNNNNNNNNNNNNNNNNNNNNNNNNNNNNNNNNNNNNNNNNNNNNNNNNN
NNNNNNNNNNNNNNNNNNNNNNNNNNNNNNNNNNNNNNNNNNNNNNNNNNNNNNNNNNNNNNNNNNNNNNNNNNNNNNNN
NNNNNNNNNNNNNNNNNNNNNNNNNNNNNNNNNNNNNNNNNNNNNNNNNNNNNNNNNNNNNNNNNNNNNNNNNNNNNNNN
NNNNNNNNNNNNNNNNNNNNNNNNNNNNNNNNNNNNNNNNNNNNNNNNNNNNNNNNNNNNNNNNNNNNNNNNNNNNNNNN
NNNNNNNNNNNNNNNNNNNNNNNNNNNNNNNNNNNNNNNNNNNNNNNNNNNTACCGCTCAGGAAGGAGACGCGTTCTGT
CTCCTAGAGATGAACGTACTTTGGTGCGAAAAGTGCAAATCAATCCCAGAACAACAGCAAAGGACCTTGTGAAGATGCTG
GAGGAAACTGGTACAAATGTATCTATATCTACAGTAAAACGAGTCCTATATCGGCATAACCTGAAAGGCCACTCATCAAG
GAAGAAGCCACTGCTTCAAAACCGCCATAAAAAAGACAGACTACGGATTGCAACTGCACATGGGGACAAAAATTGTACTT
TTTGGAGAAATGTCCTCTGGTCTGATGAAACAAAAATGGAACTGTTTGGCCATAATGACCATCGTTATGTTTGGAGGAAA
AAGGGGGAGGCTTGCAAGCCGAAGAACACCATCCCAACCGTGAAGCACAGGGGTGGCAGCATCATGTTGTGGGGGTGCTT
TGCTGCAGGAGGGACTAGTGCACTTCACAAAATAGATGGCTTCATGAGGGAGGAAAATTATGGGGATATATTGAAGCAAC
ATCTTGAGACATCAGTCAGGAAGTTAAAGCTTGGTCGCAAATGGGTCTTCCAAATGGACAATGACCACAAGTATACTTCC
AAAGTTGTGGCAAAATGGCTTAAGGGCAACAAAGTCAAGGTATTGGAGTGGCCATCACAAAGCCCTGACCTCAATCCTAT
AGAAAATGTGTGGGCAGAACTGAATAAGCGTGTGCGAGCAAGGAGGCCTACAAACCTGACTCAGTTACACCAGCTTTGTC
AGGAGGAATGGGCCATAATTCACCCAACTTATTGTGGGAAGCTTGTGGAAGGCTACCCGAAATGTTTGACCTAAGTTAAA

CAATTTAAAGGCAATGCTACCAAATACACTCCATTAGTATGTAAACTTCTGACCCACTGGGAATGTGATGAAAGAAATAA
AGCTGAAATAAATCATTCTCTACTATTATTCTAACATTTCACATTCTTAAAATAAAGTGGTGATCCTAACTGACCTAAGA
CAGGGAATTTTGTACTAGGATTAAATGTCAGGAATTGTGAAAAACGGAGTTTAAATGTATATGGCTAAGGTGTATGTAAA
CTTCTGACTTCAACTGTATGTAATGTATTTCTATAAATGTTCCTTGGGATGGAAGTATGCTGTCATGGATATGCTTTTCA
TGCGGTTTTCAAATGTCAGGCCATGATCAAAGGTAACACAAGCTATTTTTAACAACAATTGAGAGAGTTCTGACATGTTC
ATAAGCTTGTTATGACCAAGAGAATCATGAGGCTATCATCTGCACAGCATGCAGGGCAAGGAAAATACATGTGTTTATTG
ATAACATTTCCAATGAGTAACGCAAAGGACATTTTAGAGCAGAATCTTGAATGCCAAATTTGTCATCTAGTTGGACAAAG
GAATGCTTCTCAGCCAATTTGTCAGCTCAAATGTGATGACTCACTTGTGATAAACGTGAGATTCCTTGTGATTTCTGCCT
TCAATGTAAGCTCCCCTTTTCTACTGACTTCTCCAGGGCAATGGCATCTTTCTCAAGGCATTGCCTCTGGTCCTTTATTT
CAGCAATTAGGCCAGAGACACACTGAGACACACAAATACGGATGGAATCGTACATGACGTACATCATTGTCCGTCAAAAG
TACAACTCAAGACAACTATTTTCAGACGATTCGCCGCCATCAAAAGATGGACCATCTCATTCGTTAACTGATGACTCCTA
TTGGAAAAGCATTGGTTTCATCTCAAATACTCAGACACAAAATACTCAGACAACAGTGTGTAGCCAATTCTGGTGGCAAT
ATCTGAACTGTCCAGCAATGTTTTTGCTGCTCCAGGAGAAAATAGTTTCCTCAATGATGCAAGAGCCCACATGTACCCAG
CAGACCCGATGCTGACCTCCCATAATTGATCTGTCCCATCCACCTGCACAGTGGTGTAAAGTACTTAAGTAAATATGCTT
TAAAGTACTACTTCAGTCGTTTTTGGGGGTATCTGTACTTTACTATTTATATTTGGACAACTTTTATTTTTACTTCACT
ACACTCCTAAAGAATACGATGTACTTTTTACTCCGTACATTTTACCTGACACCTAAAAGTACTCGTTACATTTTGAATGC
TTAGCTGGACAGGAAAATTGTCAAATTCACACACTTATCAACAGAACACGCCTGGTCATCTCTACTGCCTCTGATCTGGC
GGACTCACTGAACACAAATGCTTTATTTGCAAAGTGAGTGTTGTTGTTGAGTATCTGGCTATCTGTAAATATAAAAAACA
CATTGTGCCATCTGGTTTTTATAAGGAATTTGAAATTATTTATACTTTTACTTTTGATACTTAAGTATATTTTAGCAACA
TTTAATGTTGATACTTAAGTATAAAAACCAAATACTTTTAGACTTTTACCAAAGTAATATTTTACTGGGTGACTCACTTT
TTTCTATTAAATGAGCTTTACTTTTACTCAAGTTTGACAATTGGGTACTTATTCCACCACTGCACCTACATGAAGAATGA
CTTCCATGTTATGTTGCAATCACCTTCCAGTGTTGTTTTGCTTCAATGTTCGGTACTGTGAAAATCATGGGTTTCTGTGT
CAGTCACCTATCTCCAGAGCAGTGGTCCCTCTCACAGACTGGTGTGAATAATGCATATGCAGCTATAGGGGGCCTCGACA
GGGCCGTATTAAATATGTATAGTCACCACAGAAGTGCTGACACAACTTACCCACCAAAACATCCTCACACCCTGTACCCT
ACCCATAGTCAACCCCACCAAATTCTCAAGGAATATACTAGTATGTATAATATATTATATCTCTCTTTTAAATTACAATT
TTTATTTTCCATCCTTCTGTCTCTTTTTCCACTTTTACTCCTTGTACACCCGTTCTTGCTTCCTCACCTCTTCATTTCGC
TCTCTCTCCCTGGCATTTCCCACTTCTCCCTCCTTCCCTTTCGCCCTCTGTCTTTCCCCCAGCCACATGCTCAGATGGAT
TCTTCAGTCAGTAGACTGGAACTGGTGCGGCTCTCGGAGAAGCCTTGAGTGAGTGAAAGAGAGGGAGAGGGAAAGATAGA
GGGGTGGAGGGGCTGCGCCGAACAGAGGTTGGGTCAGACAATCACGAGAGAGAGAGAGAGAGAGAGAGAGAGAGAGAGGT
TGATATGTGACAGGATGTGGTTTCCCTGAGCTTGCAGTACATAGCCGATGCATACTTTGAGCATGACCTAAACGCACACG
CTCTTAGTGTTTGCTGCGCACGGTTAGCTAGTGTCTAGCGAGGATAGAACAAGCCGGCAGAGCTACTGATCCCATCCAGA
TTTGTGTCTTCTCGCTGTCTTTTTTTCCCCTCTTTGACCTGAAAGCCTCAGTTAGAGCTGTATCGGCAGGACCTGAGTGA
AGAATCGCAGAGGATCTGAGCGTGGACTGTTGCGCTGTTCTCCTGTCAGTAGAATGTGTGTGTTTGTTCGAGGAGCCGTT
CCAGTGGCTTTCTCTTGATCTCAATAGCGAACTTTCCAACTCCTGTAGTCAACAGCTTCTGATCAGGTAACTCCCTCTTC
TTTCTACTTTAACTTGCATGTGTTTACCGCTAGTCTACATCCTTATTTCTTAAGGCTTGAGAAAATAAATATGTAGCCGA
TAGGAAAAGACACTGATAAAGACAGACTGTACAACTCTTTGGACAGAAGTCACAGACAACTTAGTGTTCTAGTTGTTTAT
AGCGTAACAACCGTTCAGAGGCAGATTAATGAGGATGCATGTATCAGTCAAGGGATGCCACAAGTTTGGCTTGTGTGCAA
TGTCTTACAAGAAACTCCTGATCACCAACCCACACTCCTCCTATACACACACACACACATAAACACCCGTTCATGCTCAG
AGCAGTAATCATGCAGATCAATGAGCTGGTTGGTTTGAAGTCACATGTGCGGCACCAGAGGGGTGAGAATGCCAGAAATG
TCTACCTGGCTCCAATCACTGAACCCTGATGGTAATTCAAGATGTGCTTGACATACAGTATTTAACCTTAGCCTCTACTG
GAGAGAGAGAGTGAGAGTGTTTGCCAGTGTGAGGGAGGGACAAAGTAGAGAGTTGAAGGATGTGAGAAGGGATAAGAGGT
TGGGAGAAAGAAGAGGGCATGAGAGAAATGCACACAGACAGGAAGAGTAAGAAGGAATGAGAGAGAGAGAGAGAGAGAGG
GAGGGAGGGAGTGAGGAAGAGAGAGAGGAGGGAGTGAGTGAGGAAGAGAGAGAGTTCTGTGAGTGCTGTTTGAGTGAACT
GTTAATCATTACCAGCTGGTGGGTTGTGAGGGCTGGGCTGTTAACCCTTTATTGTTGCTGGGTCACGAGCTAGCACACAC
ACACACACACACACACACACACACAGGGAAACACTTTTCACCAGGAACCCCCAAATTACTGCATCAGACTAGCCACAACC
CAAATATAACTTGCCAGCTCTGAATGGGAAAGTCATTGTGGTAAATGCTCCATACTGTAGGCCTAATTTGAAACAGAAAA
TAATAGATGGGGCAAAAGGTGAAGCGAGGGAGCCGGAGCAGAAACAGCTTCATGATATAGTTACGTTGTCACATTGAATA
CTCGCCTGATATAATCTATAAGGCCTACATAGTAGGAACTCACTATTGTGTGATTCATAAAGTTGTTCATGTTTACAGAA
TTTCTGAAAATTGACTTCAGTCGTCCAGCTGCGTTGAAGTTCAAACTTCCATTTTAATCCAGTTCCCATATGTTTGCTAC
CGGACCAAGGTGTATAGATTTCTCTGCTACTGAGTTGAATTTGAATTGAATTGGCTTGAATTGAATTGACGAGTCTTGCT
TGATTTTAAACCTTCATTGATGTTCTACTCTCCACAGCATGTACAGTGCAAACATTCACAGAGGGAAAAGGGAAAACGTT
GACCACTAGTCTACAAGTTTACTGCATTTTACAGTAAAGTACTTGAACCTAGTTCTATCTTCCAATAAAACATAGTTTAA
ACAAGAACCAAAACTGAGAATTACTGAAATAATATTTCCATATTGAAAGATTCTACACACACAATCCCACACATACCGTA
TTTATCCCTATGTGCCTCCCTACAAGTACCTACCCACTGTATTTTAGGCCTTTTGGGTAAACAGCTTAGCCTGATTACTT
GCTGAAAGATGACAGGTAATTCTTTGCACTTCTCCTGACAGCGATGACAGTCTAAGTGGATTATACTCTTGCATAAGCAT
CACATCTATATCCTTTGAAATGCTTGGGTACATGATCTGTGGCAAAGATTGTTCCTTTTTCTTTCTTTAGGGATGAGCTT
TTGAAGAGACCAATTTATTCAAGCCTTTGGCATAGCCTTAAATGACTAAGAATGGATATTAAGAGAAGTCCCTTTGCTAG
AAGGAAAACATGGTGGTATCCAGTATTCACTTGTTTTGCAGTGGCTAAGCGAGGAAGAAAACTCCCCTTTGATACTGTAT
TTCTCAAAGGAAAATTGACTGGGTGGTCAAGAATTGCAGCAGTATTGATCAAGTGATGTTCAAGGACTTGTCGATACTGT
ATCTGAAAAGCAAAACTTGGCTTCAGTTGGCTGTAATAAAAAAAAATGCCCTGCCTCCAGAAAGTTCGCATGAAAACCAA
TGAATCGTTCCTTGAAATCTTTATCCCTATGTTTTGAGAAATAGAATATGACCAAATAATTCATGTTTCTATTATCAACC
GGCGGTTCCTCTTTCACATAGTTATTTTCCTTCTTGCTGTGTGCCATTCACTTTGGCCATGATCGGACTCATCCATTTGC
TATGTTTACCCAGGATAGATTTAGAAATGTGTTGAGTTCAGAGTATTGTATAAAGCCCGTGTGAATAGATCAGATTATAC
ATACTTTATCAAGATAATGACTGTCAAACACAGCAAATGCAAAATACAGAACTGAAAGTCAGAGACTGGGAGGAAATTAG
AGAGAAGACCTTACAGGCAGTGAGAGAAAGAGAAGGGAGAAGGGAGAGATGAGAGCTTAGCCCTTGGAGAGAGGAAAATA
GAGATATTGGTGTCTATTATTCTCCTGAGAGGGGAAGTAGTTCTAAAGGGGCTGGTTCCCATGGCAATAGCTCCCTACAG
TATGTGTGTCTGGGTGCAGCATGTGTAGCCGGCTGTGCCCAGGCCTTGAGACGATAGAGGGATGGCTTGGCTGGAACCAG
AGAGAACCAACCACCGGGCCAACCAGGACACTCAGCCTTGAAGCACTGGAGAGGCTCAAGCAGACGCTTGTTTACTACGC
TCTGTTTTTACCAGCACTTATCTCCCCTCATCTTTCAGCAAGGCTCTTTCTTTATATGTGTCATTATGTCTTGTCCTTGG

AGATTTCAATTGAATGTAATAAACTGTGTCTAGCTGTGGGTTTTCAAAAAAGCTATTCCAGTTTTCAGAGAAGGCCCAGT
ATTTGGGAATACGTTATGGTCATTGTTTTCATCTTAACTACAGGGCTTTCAAGCTATGTAATATTAGCACTGGTCTAATT
GTTTTTGTTGTTGTAGTATGGTCACCAACACAACCTAAATCCCAGCCCACCTCTGTTTTTCCTCTGTTGGGCCCCTTGCT
CCTATCCTCCTTGTCACTGTCCCGCCCAGGTGAGGAGCCAAGGAAGGAAGGATGTCCAAACAGGGAATGGCCCTTCTCCG
GGCCCCTGAGAGCACTCTCCCTCCTGAGTGGACAACACAACAGCTGCCAGGAAACTGTGCCTTGTGCAAGAACTGCTTTA
TTTGTCACATTGAAGAACCGGTGTAATCGGAGTTAATGTGGTAGACACAGGCAGAAGTCCGGGGGCAGAGACGGGGGCAA
TGTAAGAGATGGCCGTGTTTTAAAGAGCGACTGCCCCTGAAAAGCAACTTGGAAACTGCCTATGTGGCATCGATGGGAGT
CAAACATTTATTTGATTGTGAAAATTGACTACAAAGTGTAAATGGGATCATTTTGGTCGTAAAGTCAGTCTCGTCCAAAA
CAGAGATTTGTGAGATGATAGGAAAAAATTGGTACGTCATGGAATGAAGGGTACTGTCAATCTCCTTTGGGTTGGATACA
TTTCAACCCTGCCCACAGCTGGCAGCACCCAAGTAATCATCATTTTGAGGCACAGCTGCATGTGATCTGATCGTAATGCC
CTTGGTCAATTTGGTTTGACGTTCGATATCCCTATGGGGCTAGTTAGGGACCCGTAGTAAATTACCCAATTTACTGTACA
TGGGACAATATTTTAAAATGTCAACTATACATTTTAGAAATACAAAAATTGAAAGCATTTAATGAGAACTAAAATATTTG
CAACATTAAAATATTGTACCATTTGCATTGCGTAAGTTATTGACAGTCCCTAACTAGCCCCAGAGCTAGGCTGTCCAATG
TCAACTTTGCCACAGTTGCAAACCAGCCGGCTGAAGCTAATTGGCGAAAGAAGTTAGCTAGCACTAGCTAGCCTAGGCGA
CTTCAAGACAGAAGACCTGATTAGACTATTTCAAGTTATTTAGACGGATGAGTGACTAACTGTGCAGTGTTTTGGCAGAG
TGAATGTACATAAACTTGCCACATGCGAACAAACACAGAAGAGACACCCCCCCAAGACAACTCTTGTTTGCCTTCACTGC
ATTTCTTAATGACCAAGCTGAAAAACAAGGCCAAATCAGGAAGTTCAGCCCCCCTTTAAGTTTGGAGATGGCTAACTCTT
CCTCAAATCCAGTGGTCACCAACCTTTTCTGAGTCAAGATCACTTTCTAAGTCAAAATACAAGCCGAGATCTACCACTCA
GATTATTTTTTTTTTAAACATGACTTAAATAATGTAAGCCTATGCAACATTACCCAATTAAAACAGTTCTGTAGCATCGA
GGTTTGTGCAGTAAGCTATAGGCCCAATACATTGTCACTGCATATTGGCTATGCTTGAATTACCCTGCCAATGTTGTTCT
TCTCAGACCATTTTGGAATGATATTTAAAATTAGGTATATGATCACACTGGTAGTAGATCATTTGTTGTGTTACTTATGA
GGCACAGCAAAGTGAGCATGATCATTTGTTTATATTTTTTAATATTTTTTAAATTACTGGACTGAAGGTCAGTCTGAGGG
GAGGGAGGGAAGGAGCAGCGGTGATGCTGCCTCACCCGACTCATCGACCTCTGCTCTCCATCCCTCCACTGAGAAAAGGA
GACAGTCTTCCAGCTGATGGCGAAACTCAAGTCCACACTGCATTATTTTTGCCTCATACACCAATTCATGTTATTCCTAT
GACCAGAGAAAGTGAAATATTCCTCGTATTAAAAACGACAAGCCGCTAATAATAACAACACAAGCTTATTGGAACACTTT
TCTAACATTATACTCATTCATTGCAGCTGCAGTGCTGGTTGTAGCATGAGTGGAAGTATGGAACACGCGCATGTGACAAA
AGTGTTGAACAAAGTTTTGACAGTGCTGAATAAACATGAACTTAGTCAGCAGCTCTTTGCTGTATTTGTTGGTTTTGAAA
GTTTTGAAATCTCAAGAGTACCAACTTTGCTGCTTGTTCGAGGCTTCTTTTTACAGTCTATGGCTCGAGGAAACTGTGCA
GACACGGTTATCTGAGCTATCTGATTGGTCAGCGATTAGGTCTATAGGTGCACTTGATTTCTACCTTCAGACTTCCCGGC
ACTAGGGCTGCTGAATCACTGTGCGCCTACCGGCAACGGCGCAAAACACATGTAGAAAATAGGAACACAAGGCTTTAGTG
TTGGGTCTTTTACAGAAATGTTTGGTGATCAACTTAGAATCCGATCGACCGGTTGGTGACCACTGCTCTAATCCTTTCTT
GTCCAAAGAGATCTTTTCATAACAAGCTGAGCTTTGTCCTCAAGATTTGTATCTTTATATGACACTTCCAAGAAGACAAA
TTATTTTATTATTAGATAGCATTATGGAAAGACAATGAATCATCACTCTTGTTATTTTGTTGACCATATTTGGCAATATC
CAACCAAATAGTTTTTTACAGAATCATCATACATTATTAGCTCATGTAAGATGTTTCCTCCCATGCAGATATAACATAAA
TCCTGTGCTAAGCAACTGTGGAGAAGCTGGTGGTTATGAAGGATGTTTAACTATTGAATTTGATATTTATTAAGGATCCA
TCTTAGCTGCTGTCTTGGCAGCAGTTACTCTTCCTGGGGTCCAGCACCATTAAGACAACTACATACAGTTAAAAAATTAC
ATTTCATAACACTTTACCCAATACATTTAGTGTGTTTCTCTCAGGCCAGTGCTCCACTATCACATATTTACAATACAACA
TCCATGTGTACATGAAAAGTATCTTGTGAGGCTGAAATTGGGTAGAAGATACAAAATAGGTCCGTTTAGGAACCAAACAG
GTTCATGGCTAACAAACGTGACCTTCTACACTGAACAAAAATATAAATACAACATGTAAAGTGTTGGTCCCATGTTTCAT
GAGCTGAAATAAAATATCCTAGAAATGTTCCATACGCACAAAAAGCTTATTTCCCTGTTAGTGAGTGTTTATCCTTTGCT
AAGATAATCCATCCACCTGACAGGTGTGACATATCAAGAAGCTGATCAAACAGCATGATCATTACACAGGTGCACCTTGT
GCTGGGGACAATAAAAGGCCACTCTAAAATGTGCAGTTTTGTCACACAACACAATGCCACAGATGTCTCAAGTTTTTAGG
GAGTGTGCAATTGGCTTGCTGACTGCAGAAATGTCCACCAGAGCTGTTGCCAGATAATTGAATGTTCATTTCTCTTCCGT
AAGCCGCCTCAATGTCGTTTTATAGAATTTGGCAGTACGTCCAACCGGCCTCACAACCGCAGACCATGTGTAACCACGCC
AGCCCAGGACCTCCACATCCGGCTTCTTTGTATCAAATTTTATTTGTCACATGCGCCGAAGGTGTAGATCAGGGGTGGGA
AACTTCAGGCCTCGGGGGCCTGATTGGTGTCACACTTTTTCACCGTCCATAGCAAACGCAGCTGATTTAATCAAATTGCA
TTCTAAATTGAAGATCATGATTAGGTGATTACTGGAATCAGGTGTGTTAGCTGGGCCTGGGGCAAAACTGTGACACAAAT
CAGGCCCTCGAGGACTGGACCCTGGTGTAGACCTTACTGTGCAATGTTTACTTACAAGACCTTAACCAACAATGCATGGT
CTGAGACCAGCCACCCGGACAGCTGATGAAACTGTTGGTTTGCATAACCAAATAATTTCTGCTCAAACTGTCAGAAACCG
TCTCAGGGAAGCTCAACTGCGTGCACATCTTCCTCACCAGGGTCTTGACCTGACTGCAGTGGGCAACTCCTCACCATCGA
TAGCCACTGGCATGCTGGAGAAGTGTGCTCTTTGTGGATGAATCCCGGTTTCAACTGTACCGGGCAGTTGGCAGTATGGC
GTGGTGTGAGCCAGCGATATTTGCTGATGTCAACGTTGTGATCAGAGTGTCCAATGATGGAGGTGGGGTTATGGTATAGG
CAGGCATAAGCTACGGACAATGAACACAATTGCATTTTATCGATGGCAATTTGAATGCACAGAGGTACGGTGACAAGATC
CCAAGGCCCATTGTCGTGCCATATATCTGCCGCCATCACTCCAATTATTTTATGGTCTGCATACTCACCAGACATGTCAC
CCATTTAGCATGTTTGGGATGCTCTGGATCGACATTTACCACAGCGTGTTCCAGACCACAATCAACAGCCTGATCAACTC
TATGCCAAGGAGATGCGCTGTATGAAACAAATGGTGGTCACACCATATAATGACTGGTTTTCTGATCCACGCCCCTATCT
TTTTTTTATTAAGGTATCTGTGACCAACAGATGCATATGTATTCCCGGTCATGTGAAATCCATAGCTTAGGGCCTAATGA
ATTTATTTTAGTTGACTGATTTCCTTACATGAACTGTAAAATCATTGAAATTGTTGCAGGTTGCGTTTATATTTTGTTCA
GTATATATCTGTCCTATAAAAGTACAACCTGTAACAACAAACCCCCTTTGACTTCTAACATCTTGCCTTACAAAGTCTTAT
CGAGTAAAACCTCAAATGACATTAACTCCAAACAAAAGAAGGAAAGAAACGGTGAACCGATCGTTACTAATTCAGCAAAG
GATCAATGCCTTTCTGTTTTTCTGTTTAGACTGTTGGTGTAACCCAAACTCCCTCGCTGTCAGTCTAAAACTCAGAGGAG
ATTAACCAAAGCGGGCCTGTGGAGTCAGAGCTCAGACAGAGCGTGCTCAGAACATGCACCTCGACTCTGAGGAGAAGTTT
GAGAGGGAAAACAGTCACTCTGAAAATCATAAACATCTCACACTTCCGCTGTCAACTCTAAAAATACCCGGCCTCGCCTC
GCTCCCCCCGTCGGCCTCCAGGAGCTATACAAATAAGAGCCCCTCCGTTTTTCTAAAATGTTCAGCCCATTGTTTTTCTC
TCTTATACTTTTGTTCAGACTTCTGCCTCTCCTGTACACTACATCGTGGAGCCTCTCTCCGGAGTTCACTGGAACTTAAA
CAGAGCAGAGCACATCTGAGGTGAAAGAGAGAATATTAAAAACTATGGATATTTTACTGTAGTATTTGCCTGTTTTCTTT
GATGACAGAAAAATGTTGATGTCATTGAGCCGAGTTGCTTTAGTCCAGTCCTGGGGGGCTGTTTAGGAGAAATTGAACAC
TTTTAGGGAAATGGAAGATCAGTTTTTTGTTGTTGTTTCTTTTGGCTTTGAAATGTGTGCTCTTGATTTGAGAACTGAGG
CAGAAATTACACAATTCCCAGAGATGACTCACTGAGCTCTATTTTAAACATTTAAATCCAATTTTAAAACGTCTTTGAAA

CATATTTTCAAAAATACAAAACATCAGATCCTGTTCCTTGTATTCACTGGCGTACTACACGCCCTGCAGCCCCCGCAAGG
CAGGTCATAAGCCATGGCAAAATGGTTTAGAATGACAGGAAATAAGCTGTAAAACTGCTGAATTCTCTGACTCATGGCAA
AATGTGTAGAATAGCAGGACATTTGCTTAAAAAATGCTAAATTCTCTCAGCCTCATTGCAACATTTGTAGAATAGTATGA
GATGGTCTATAAAACTGCAGTATGCCGCTGCTTGTATTGACTTGAAAGCCATTGCTTGTAATGTTATAAATTATACATTT
TATGCACACTCATGAAATTGATCAAAAAGAGACATGAAGATTATAGATGATATGCACATTTCGTTCCATAATACAAACAC
GGATATCTATGTAGGTGTTGTCATGAGAGCCACTGTCTTCCACAACACGTTTGAATCTCTGCCATCTCCCCCTTCTTGTC
ACTTCTATTCAACAGGGTCTAATTACAGTGGCCTCTCTCTCCAGACTCTACTTGCCATGCATTACCTAACTCGTCTCTCT
AACGAACAATCTCCAACCCGCTACCCATTTCCTCTTTTTCCCTTGATTGATTCTCACCTTTTCATTCTTCCTGCTCACCT
CATTAACAGAGCACCGGTTCACCCCCCCCCCCCANNNNNNNNNNNNNNNNNNNNNNNNNNNNNNNNNNNNNNNNNNNNNNN
NNNNNNNNNNNNNNNNNNNNNNNNNNNNNNNNNNNNNNNNNNNNNNNNNNNNNNNNNCCCCCCCCCCCCCCCCTCCTATCTC
CACCACGGCCTCTTGGATTAGTACGCAGGCGGAGCCCGGAACGGTGTATAATTAGAGGGGAACATAATAAAGTGCGTTCC
TCATCAGCTGGGGGGCCGGTGCCCCCTGGACCACCATCCCACCCTCCCGGGTTTAATCAGCAGAGTACAATGGGATATTA
CCTGTGCCAGCCTGAGCCAATGGAGATGCAGCACCACTGTCACAAGCAATTTGGGTGACGCCCGGTGCACAGGCTCTTTA
GAGGCTCTTTCCCGTAGTCTTTCATGGTTGTCTCTGTTAATTTGTCCCCCAGGGAAAGAGGGCTCCGGAACCCATGAAAA
ATCAAGCTCATTTATGCCCTTTTTATTTATTTATTTATTTATTTGGAGCCACTTATCCAGCATGTTTTTCTTTATTGGTT
TTCCCTCTGAGACTCCCTTGGGGCCCCGTACGTGAGGGGTGTGTGTGTGATCGAGAGATTAATCCATCTCTCCCTAAATT
GGACATGTTCTATGCTTTGGGGACTGGTGTCCAGTTTCACACACACACGGGTATCAGGTAGCCTAGCAGTTAGAGCATTG
GGCTAGTAACCAAAAGGTTGCTAGTTTAAATCTGAGCCAACAAGGTGATAGCAGACCCTGGCCATGACCCCACTCTTCGA
GGGTGTCTCAGGGGGAGTTGGGATATGCAAAAAAACACATTTCCAATTCACACGTGTATTAATTAACACTAGTACATGTG
TGAAATAGGACAAATATAAGAACCCACATTATTAATATTATTGTTATTATTATTATTACACACATACACACACACACACA
CACACACACACACACACACACACACACACACACACACACACACACACACACAGATTCTTACAGAACCTCATTCTGCCCCA
TCTATTCCCTATGTAGGGTCCATACACACTCTGGGGAGATGGGCTGTGGACTGAGGAAGCTGAAGCCATCCGAGGAGAGC
AGTCCAGGGAAGATCTACTCCACCCTGAAGAGACCACAGGTGGAGACCAAGGTTGGCGTAGCCTACACATACCAATACGT
TGACTTCCTCGTAGGGAAAGGCGGTAAGACATGCAACTTTTCAGAGAATTTACTGGTTATTTCTAAGCCATTTGAAAATG
TATTAGTCACTGTTACAGTAAGAATACAAAATTATAATGTTCTCCAACTCTCTTTTACACACCAAAGATATCCCTAACT
ATCATATTATTAGGGGTGGCAGGTAGCCTAGTGGTTAGAGCATTGGGCCAGTAAACGAAAGGTTGCTAGATCGAATCCCC
GAGCTGACAAGGTACAAATCTGTCGTTCTGCCCCTGAACAAGGCAGTTAACCCACTGTTCCTAGGCCGTCATTGTAAATA
AGGATTTGTTCTTAACTGACTTGCCTACTTAAATAAAGGTTAAAAAAAAATATATATATTGTTTAAGTTCAGTACCAGAC
ATAAAACTGACAACCACAGAACACGTTCTCCACCTCTCACATCGTTAGATGGAAATACATTTCCCCACCATAATAGACTC
CCTCACACACCCCCAGGCAACAGAGGCCTTTCTTGTTGCAATTTGAGGTGTTTATGAGATGCAAGGTGGGTGTTGCTTCC
TGAGACCTGCTCTGCTCATTGCTCAGGGGCGGACCTATGGAAGCTGGGGCTAAATATAGGCATCACTTTTATTTCCCTT
CAAGAGAAGTTACAGTTCAAACAGTATGAATGGGGGTGGGCTGGTGGGAGATGCAGTCCTCTGGAGCTGGGAGAAGCTGG
GAGATGTAGTGTGCTGTGCGTCGCCTGTGACAGTCCATTAGCGAGTCACTCTGACGGGGAGAGAATGGGTACTAAGATAC
ACCATCTGGGTGTCTGGGGCTGTATGCGTGCGTCTCTCTCTCTTTCTCTGTCTCTCCCGCGCTCTTGCTCTCCCACTGTG
TCTGTCAGTCTCTCTCGCTCTTTCTCAGTTCTCTGCCTCGCTCTTTTTTTTAAAGCTGTGCCTGTTTGTCTCCTAGGACT
ATATTGAGTCTGTAGTTGCGTCCCTGTAGTGTTTGGGACTGTCTGAAAATCCTTACATTACCGAAACAGTTTTCCAGGGT
AACTGTTGGGGGATAACTTACTGTATGTAATTCTATTGTACCAGGCATGTGACTAGGCTACAGGTAAGTTCCACTGTTCC
TGCCACTGTTAGTGCTGTTTCCTAGAAAATACACAACATTTAATATGATCACAGAAGTGCACTATACGTACTCATGGCTG
TATATACTAACTAGCCTGTTTATTCCAAGTTGTAATGTAAATGTGGCCTCTGTGTATCCAGGTTCATGCTTTATTGTTCT
AAAAAGTGTTTTGGTCTCAGCAACAGGCTCTAACAAAACTCTTTCAACTAATCAAATCGTTTAGATAACTATGAGAATTG
AGGAGAAAATTAGGACATTAGGAAGATGTAAATGTTCTTGTTTATTTGTTTCCAATCATGTCCATAGCTCAATGTATCTC
TAAAACAGTGAGCATATAGCGCCGCCAGTTACTTTTAGGGGGTTTTCACATATGAAATTTGGTACCCTGGTTCGGTGTCC
TGGAGCATTTGGTAAGAGTTCTACAGAATGCGTTTTGCTTTCACAAGACCTGAAAATAACAGTTTCAAGATGCACATTTT
ACATAACGTTAGCGAGCTAGCTTGTTTTCAACGGGCAAAAAAGTTCCACACAACTCACGCTGCCGTGTCACAATATCTGA
TACTCTGGTTCTGGGTCTTGGACCCGCTACTCACACAGGTCCAGGATCTTTTGGGTTTCACACATGCTTTATAGCGCGGA
TCAGGGTACCAAATGCTCCAGAATCAGGATAATAAGGGGATATGTGAAGGCGGCCCTTAGATAAATGACCAAATGATAGG
GGGCCATGTTGAACCACATAAAGACCCGACCAAGATATGAAGAGCAATGTGTCATGAGCTTTTAATAAACTGCCACTGTA
AATACCAGGCAGCCTTAGCTAGCAAGCTATAACCCCATGTTTTATGTCAGTTTATTGCACACATTCACGAACACGGCCAT
GTTGCTACATTTCATTTACCTCAGAAAATGGCTTTGAAACAGAGAATGATTTGATATTAACACCATGACCCCATAGGGAA
AGGATAAGCCGAGGGCTGTGCTAACCGGTGTGGAAAGAAGCCATCTCACCGTACATACAGAGAAAGGGTCTCGGAGTGCT
TGGCACGTATTTTATTTCCAAAACTGTGATTTCAAATATTTAAAATGTCAAGAGGATGAAAAATTGAAAACACACTTATG
TTAGTCTTGAAATCAAATCCAGCTGACAGACACACACGCACGCACACACCATTTGATTTCATCTTGGCTCCAGACACATT
ATTTATATGCAAAACCCCCAGAGCCTGAAGTCCTCTTGTTAATATAAGCTTTTCTCACCGCTCCGTGTGACTGACGTGTA
GAAGCAGCGGCGTTCCGCCTGTGCTCTGATCGGAAGCAGGAATATGGAGCACGGAAATGAGAGGTCAGTGGAATTTTTGA
ACACCGTAGTGTGTTCTGAGTGGAGATTAACCCCTGGATGCTGGGAGCCAGAATTTATCCCAGTTCACCACTCCGAGAGG
GAGAATTGAACCGCGGTCGGTTCTCGGGGAAACGACATTGAAGTGAATTCACGGTCATCAGGTTTTACATTAGATTTCCA
TTATATTCTCACTGCATGGGTGAGGTGTTTGCATGGCTCGATTCCATCCAACATATTGTTTATAATGTAGAAACAGAAAA
GACACGCTTAGGGTAGAAGTTGACACCAACCACAGATCTAGGATCAGATGACTTTATACCCCCTAACCGTAACATTAGAG
GTATAACAAATGAATGAGCCTGTAGGCCTAATGATTATGGATATTTTCTATTCCAAGACATGCACATGTAGGATGCACAT
CCGCCTTAAAGTGTGAATGTAGATGATAGCGATGGAAACCTCTGGTGTTAATGGTCGGATTAGCATACTCTATCAATCAA
TGAACCACGGATGGCTTTGGGTAGCAAGTTGAATCTCGGTAACCAAATACACTGAGTGTACGAAACATGAAGAACACCTT
CCTAATATTGAGTTGCACCCTCCTGTTGCCCTCAGAACAGCTTCAACTCAGCGGGGCATGGACTCTACAAGGTGTCGAAA
GTGTTCCACAGGGATGCTGGCCCATGTTGATGCAAATGCTTCCCACAGTTGTGTCAAGTTGGCTGGATGTCCTTTGGGTG
GTGGACCATTCTTGATCCACACAGGAAACTGTTGAGCATGAAAAACCCAGCAGCGTTGCAGTTCTTGACGCAAACCGGTG
CGCTTGCCACCTACTACCATAGCCCGTTCATAGGCACTTAAATATTTTGTCTAGCCCATTAACCCTCTGAATGGCACACA
TACACAATCCATGTCTCAGTTGTCTCAAGGCTTTAAAATCCCTATTTAACCTGTCACCTCCCCTTAATCTACACTGATTG
AAGTGGATTTAACAAATTGCAAATTACAAGGGATCATAGCATTCACCTGGTCAGTCTATGTCATGGAAAGAGCAGGTGTT
CATAATGTTTTGTACACTCAGTGTATGTCATAAACTGTAAGTTTAACTATTCTGTAAATACTTCACATGTAAATACCTTT
TGAGGTTTCTCGACACAGGAGGCATAAGTGTACAGGTGTCCATACAGCAACAGATGATTGCTACTATGTAGTGCTGAATG

TTCCTTACTCTTGCTTTGGACCGCTCAAGCAGTCGAATACCAGCCTATTGTTTTGAAAAGAAAATGTGTTGGAGCAGCAT
TAAAAAGTCTGACATTGTAAAACTCAGCAGGTTCCTAATCATTAGGCTTCAAACAAGAGCAACTGCTGACAACGTTTTCA
TCAACAGGAAACGCAGATTTCTGTACATTTCCTTCCTCTCCCCCTCTCACTCTCTCTCTCTACATCCTATATCTGAGCCT
GTGTCTGAGGCAATATGTTTGAGATTAGCCTTGATTCTACAGGAGGAAATTCTTCTTATTCTGCAAATACTGTTTCTAAG
GGTCTGTCTTCTGTGGTGAACGGAACCTCATTTGCTATGTGAAATATGTGTACAGACAATTATGTTCCTTCTCCTTCGTG
GAGGCAAATAATCTGTTCATGATTTGGTTTTACTGGTAGCCCAGATATAAACCTGTCTTATTTAGCTTTCAGGAGAGTTC
TCTTTCTCTCTCTAGATATGTGTGGGGATTTCTGAGTTTGGGGTCTGTGTGCATTATGTGTTTATGTGTGTGTGTGTATA
TATATATATATATATATATATATATATATATATATATATATATATATATATTGTAAGAGTGTGTGTTTGAGTGCCTGTTTTT
ACCAACCCCATTTTCTATCCTAGCTTCCATCCTCTTCCCGCTACTCTGATACACAATACCTCCTATACGCCCCCTCACAG
CTTTATTGGGTGTTATTGTCCTGTTGCTCTGATCCAATATGACAGTATTGATTGTGTTGAAATGCAATCTAGGCATAGCT
AGCAGAGGAGTAAACAAGCTCAATGTTAACGAAACACCTCTGTCCCAGCCTTCACACAACCCAAATGAGGAACAAAGATA
TTTGTCTGCTGTAAAAAGAGGATAAATTGTATCCTCTCAGGGCTATTTTTGGACTTTATAAAGAAGGCATTGGGTTGTTG
CTCACCGTTCACAGGAAGTGTCCTCATTCTGGAGAATATTGACCTCACTTCCTGACAGCTAGGTTTGAACATTTCTGTTT
TCAAAGAAAGTCTTGGTTGAATGTTCATCTCAAGGTTCATGAGACCATTGGTTTTCTGTGAAAGCTTTCAATTGGAATGA
CAAGATACAGAATGTACTGAGAGTGTATGCATGTGCACACTTAAATGACAGAGCTGAACATGTACAGTTGTTTTTCATTC
ATCTTCATTCTCATTCAGTTAGTGGGATTCCAGGTGTCATTCAAAAAATGTTGCTTAATAATTGACATGCACAGCTCGAG
ACTGCCATGCTACTTACTGCTTATCCTACAGCCATTCTCTCCCATGAACCACCAAATGTAGAGCTTCAGACATTCTGGAG
CTGTTTGAACTCCTCTGAGCTGGCATGTTATGAATAGTCTGACTTTTCAAAAGCATATTTGAAGTTATATCTCATTAGAT
CGATGTAGATATGGGGGGAGGAGTTCTCTTCAGATTTCTGCTGTGAGAACCAACACAAGACTCCACTGAGGAATTATCAA
CCCTCAAATAAACCTGAAGGACTCAGCATCAATAACGGAATAGAGTAGCCTACCCACTAATGACTCATCCTCAGCGATCG
ACATACTTGGGAAAATGTTTGCTAAACGGGGTTCATTCGACTCCGTTGGGCTATCTAGGTTCTCGGAAAACCACACAGAA
GGAAACTATGGTATTGACAAAATGTCTTCTCTAAATAGTCATGTCAAGGTTTGAGCCCACCTCACCAACACACACTGGTT
GTGTGCGCTTTCTCCGAGAGCCTGTCTCTCATCCTGTGTAAACTGTCAATTGTACGTAGCACCAGTCATCTGTATCTGTT
ATCTTTAATCATATATTCAAATGTTTTTCACTCATTCTCCTGCATTTGACTTATTTCATAACTCATGGGAACACGGGTCN
NNNNNNNNNNNNNNNNNNNNNNNNNNNNNNNNNNNNNNNNNNNNNNNNNNNNNNNNNNNNNNNNNNNNNNNNNNNNNNNN
NNNNNNNNNNNNNNNNNNNNNNNNNNNNNNNNNNNNNNNNNNNNNNNNNNNNNNNNNNNNNNNNNNNNNNNNNNNNNNNN
NNNNNNNNNNNNNNNNNNNNNNNNNNNNNNNNNNNNNNNNNNNNNNNNNCGGCCTGGGGGGCGGCAGGTAGGCCTAGTGGTGT
GAGAGCGTTGGACTAGTGACCGAAAGGTTGCTGGATTGAATCCCTGAGCTGACAAGGAAAACATCTGTCGTTCTGCCCCT
GAACAAGGCAGTTAACCCACTGTTCCTAGGCATTCATTGAAAATAAGAATTTGTTCTTAACTGACTTGCCTAGTTAAATA
AAAATATGCCTCCCTAACTTATGTTACGGCTAATGCATCTCTATGGAAAATATGAGGAAGAGTAGAGTTATATATTAATG
CTATGTAAGAGAATGAGATGATTCACACCGTGTATGCAGGTGCAGACACGTTTTACAGTGCCGGCTGCACATAGACGTGG
ATCATCCACTCTATACAGTTGTGACACTTAGTGCCTTCTAAAAGTATTCACACCCCTTGACTTTTTCCACTTTGTTTTGT
TACAACCGGAATTTAAAATGGACTCAATTGAGATTGTGTGTCACACACAATACCCCATAATTTCATAGTGGAATTATGTT
TTTAGACATTTTCACAAATTCATTAAAAATGAAAAGCTGAAATGTCTTGAGTCAATTAGTATTCAACCCCTTTGTTATGG
CAAGCCTAAATAAGTTCAGGAGTAAAATTAGCTTAACAAGTCACATAATAACGTGCATGGACTCATACTCTGTGCGCAAT
AATAGTGTATGAAATGTATGCATTCACTACTGTAAGTCGCTCTGGATAAGAGCGTCTGCTAAATGACTAAAATGTAAATG
TAAAATGTTTTGAATGACTGACTACTTCATCTCTGTACCCCATCACTGTACCCCACACATACATACAAACTGTAAGGTCC
CTCAGTTGAGCAGTGAATTTCAAAAACAGATTCAACCACAAAGACCAGTAGGTTTTCCAATGCCTCGCAAAGAAAGGCAC
CTATTGGTAGATGGGTAAAAAAAAGCAGACGTTGAATATCCCTTTGAGCATGGTGAAGTTATTAATTACACTTTGGATGA
TGTATCAATACACCCAGTCACTAAGATACAAGCGTCCTTCCTAACTCAGTTGCTGGAGAGGAAGGAAACCGCTCAGGGAT
TTCACCATGAGGCCAATGGTGACTTTAAAACAGTTAGAGTTGAATTGCTGTGACAGGAGAAAACTGAGGATGGATCACCA
TGTTGTAGTTAGTCCACAATACTAACCTAAATTACAGAGTGTAAAGAAGGACGCCTGTACAGAATATTCCAAAACATTCA
TCCTGTTTGCAATAAGACACTAAAGTAAAACTGAAAAAAATGTGGCAATTTCTGAATACAAATCGTTATTTTGGGGCAAA
TCCAACAAAACACATCGAGTACCACTGTTCATATTTTCAAGCATGGTGGTAGCTGCATCATGTTATGGATATGCTTGTCA
TTGGCAATGACTAGGGAGTTTTTTAGGATAAAAAGAAACTGTAGGAAAACCTGGTTTAGTCTGCTTTCCAACAGACACTG
GGAGACAAATTCACCTTTCAGCAGGACAATAACCTAAAACATAAGCCAAATATACACTGGACTAGCTTACCAAGTTACAG
GCTTGAAAGTATATGGCACGACCTTGAAAATGTCTGTCTATTAATGATCATTCAACAACTTGACAGAGCTTGAAGACATT
TTTAAAGAATAATGTGAAAATATTGTACAATCTAGGTGTGCAAAGCTCTTAGAATTACCCAGAAAGACTCACAGCTGTAA
TCGCTGCCAAAGGTGATTCTAACATGTATTGACTCAGAGATGTATACTTATGTAAATTAGATTTCTGTATTTCATTTTCT
GTACATTTGCAAACATTTTTAAAAACATGTTTTACTTTGCCGTTATGGGGTGTATGTAGGTGGGGGAGAAAAAAATACAT
GTAATCCATTTTGAATTCAGGCTGTAACACAACAAAATGTGGAATAATTCAAGAGGTATGAATACTTTCTGAAGGCACTG
TATGTGGTCTCTTGAGGCCTGCACTTAGCTGAAAAACATGTAACACCTCATTGATAGTGTTAGGCTGCCATATTTTATCA
TCGAACATATTCAAGTTCACAGCCTTTGAGATGGGGCTTGTACTGAGCCCTGAGGTATACCAGTAGATTCAATACTACTG
CACTGAGGTGTTCTACTCCATTTGGGTTATTATTTTAAACATTTTTGACCTTTTATTTAACTAGGCATATTGAGGTATTG
AGTTGTGGTGGTGGTTCTCCGTACCCTGTGTACTCTTCCGTAAAAACAATGGAGGCACGAAGGGCACTACGTGAGATGTT
GAAACTGGTGATCCCAGAGGAATGCCGGGGGCCTAGGGCTGTTGGCATGTCATTATCAATGTGGGTACCCCCTTCCACAC
ACGTACAATACAGAAACACAGACACACACACACAACCTCCAACACCAACAGATAGACAGACCTCCCATACAGCCTTTACA
GTATCTCTCTCAAACAGTGCAATCTTTCACGACGTGGAAAGATATGATCCCCATAGTCATAGTGGTGGTGATTTATACAT
ACAGTACAAACCCAGACGGTAGGCATTATAGATGAGCCCTCTCTAAGACTGTTTTGGGGAGGGATACCAACGACCACAGC
TCCAGCTGTGAGAGTATTACTTCTTAGGAAAACAGTCTCATACATTATATAAACCAAGAACAGAAGTAAAGAAGATTATG
GACTTGAGAGTAAACAGAAATGCATTTGGTGCCAGGAACCTGTAAATAAGGAATGGAAATCTATCCTTGTTGAAGGGGTC
TAATCCTTATGTTAACCACAAGCTACTTTGTCAAATTCTAATCTCCATATTTTACAATTCTAATTTTGGATCTTACAATC
AATTTGCATCGAACGCTTCTCTTATTCAAGTGTGGCTTCTTTCAATGGAACCCTAATGTCAGAAAATGTTATGGAAATGG
ACTCCAACCTAAAGGGAGGAAGGAGAAACTCATGTACTGTTGTGGTGCCAGACTTTGGTTCAAATAGTATTTGAGATCTT
GAGTGTTTACTTGATCCTGCCCGGAGTGCCAGGTGGGCAGGTTTTTCACTTTTGGGATTTTTTTCTTCGATTGGTTCAAT
TGCACCAGGCAACCTCTCTCAAACACAGCTGAAGTATTTGAAAGATTCAGAATGCTATTTGAACCCAGGTCTGCTGTGGG
GCACCGCTGAATGTCGTCAATGGATTCATATGGGCATTGATGAACGCCTGCTCATTTAACACTGCTTGTCTCAAGAGCTG
CCCGTCTCCAAGCAACAGGGCTGTCAACGGATCTACTTTCTCAGAAAACTCGCTTCGGAGACTTCCTGTCAAGCAAATCT
GATGGAGGGAGAGACTTAATTCCAGAGAGACCTTTTTTCCTATTTATAAACCTTTTTACTAGCTGCACAAATTACAGGTC

ATTTTCCCTCCATCAAGTCGCCGAGCGCCTTTTCATTTAGCCGTCGTAACGTACAGAGTTTAGACATTTAAAGAGGGTGC
TTTTGATTAACTCTTTGTGCTTTTCCACTCTTTTATAGGCTTATGTGTGGTTGTGCTGAGATTGAAAGTAGCAGGGGATG
CATATGTACATGGTGATCTACAGATGGACGCTTAAATGCACTGTCTAGACTTAAAAGTACAATTAAATGAGCATTTTAGC
AGTCCTTGGAGACTCACTGCCTAGTCAAACGACACACTAAACCTAAGCTCCTCATCGATTATGCCTGCTTCAGACGGGTC
CAGTCTCTTGATGACCGCCTCCTCTTCTCATGATGCAGTCTGTTACGGTGCCACCGTGTAAGCTGCCATAAGACTGACAT
AACCTGTAACAACATCTAGCAGGCTAGCAGTGACTCTTCCTCCCTCTGGAACATGACCCCTGGCAGTAGTTCAGTTTTAA
ATGATTAGAATGTGTCATCTGTATCTGAGAAGGGAACAGGAGCAGCTTGTCTCCAGGTTATCTCCCACTTCGCTGGAGCA
CAAGGTGTGAATGCCTGTGTGTGTGCGCGTGAGCGAGCGAGCGAGCGTACGTGTGTGCGTGTCTGTCTCTTGCTGTGTGT
TTGAGAGATTGTGTGCTTACCTTGGATGGTGTGCCTAAGTCTTAGAGAGTGTGCATTTTTGCTTACCTCTGAGCGAGTGG
CGGCCAATGCATGGCACCAGCAGGCCTCTCACAATGACTCATTAGTTAGGTAATTAAGGCGCAAGGCTGTGCCCCCGGGG
CTCCACGGCGGAGCACTGTGAGCTAGGACTGCTAATGATACTGCTAGGCCCAGAGCAGAGTGATGTAGGGAAAGTATGGT
CCATAGGTTCTGCTCTCAACAACGGAGGGTGGTGGGGGGAAGAAAGGAGGAGAGGTGCGGACAGGGGACCTGTCGGCATT
TTCATTATGCGTCTATGAAAGCTAATGGCACAGGGCAGGGCCTGGAGAGGGGAGACGGGTCCAGAGGTTGTGCTCAGTGG
AAAGAAGGGTTTAGGGAGNNNNNNNNNNNNNNNNNNNNNNNNNNNNNNNNNNNNNNNNNNNNNNNNNNNNNNNNNNNNNN
NNNNNNNNNNNNNNNNNNNNNNNNNNNNNNNNNNNNNNNNAAGGACCTCATATTATGTAGTATTTATGTAGGCTTTATGAAC
TGCCGTAGACACCAGGTATTCCCACAATACACAACCATGTACTCCCGTTAGGGGAGAAGTAGAGAAATAGTGAAGGGGTG
GCGGCTATTGTCATTTTGAGCACAGTGCTACATTAGTCATTGTCATTTGGATGACATATTGTAGTATCCTAATCAAATTA
CAAGATGGCGGTCCCTTCCTGTATGGTTTCAGGATATTATACTCTACAGTATGCATCTTGTTTTGTCATGCAAAACACCA
CTTCCATACCCTGAACTCAAAGCAGCTAACGTTTTCAGTTGAATGATAATATAATCTTCAAGTTGTTTGAATATGATATT
GTCCATAAGCAAAATGAGTTATTAGTGTGGTAGGAAAATAAACAGCGCTTTAATTGTGATGAGGGAAAATGATGCTGAAC
AGCAGATATCAGGAAGAATGTTTAATTTTTCCCTGAGTACTAATAGCTAATTTATTCTGGGTCTATTTTTTTCACCATGC
TCAAAGGAATATTCAATGTCTGCTTTTTTTTTACCCATCTACCAATATACGCCCTTGTTTGCCAAGCATTGGAAAACCTC
CCTGGTCTTTGTGGTTGAATCTGTGTTTGAAATTCAACGCTCGACTGAGGGACCTTACAGATAATTCCACTTTGACAGTA
TGGGGTATTGTGTGTGGGCCAGTGACACAAAATCTACATTTAATTCATTTAAAATTCAGGATGTAAGACAACAAAATGTG
AAAAGTCTAGGGGTGTGGATGGCCAGGTGAAATATACAGCCCAGGTGAAATATACAGCGCATTCAGAAAGTATTCGGACC
CCTTTTCCACATTTGTTACGTTACAGCCTTATTCTAAAATGGATTAATTCATTTTTCCTCATCAATCTACACACAATAC
ACAATAATGACAAAGCAAAAACAGGTTTTTAGACATTTTTGCAAATTGATAAATAAAAAAACACTATCACATTTACACAA
GTACTCAGACCCTTTACTCAGTACTTTGTTGAAACACCTTTGACAGTGATTACAGCCTTGAGTCTTCTTGGGTATGATGC
AACAAGTTTGGCACACCTGTATTTGGCGAGTTTCTCCCATTCTTCTCTGCAGATCCTCTCAAGCTTTGTCAGGTTGGATG
GGGAGTGTCGCTGCACAGCTATTTTCAGGTCTCCCCAGAGATGTTAGATCGGGTTCAAGTAAAATAAAATAAAATTTTAT
TTTTCACATGCGCCGAATACAACCGGTACAGACCTTTACCGTGAAATGCTTACCAAGCCATGAGGTCGAAGGAATTGTCC
GTAGAGCTATTCAAGGACATTCAGAGACTTGTCCCGAAGCCACTCCTGTATTGTCTTGGCTGTGTGCTGAGGGTTGTTGT
CCTGTTGGAAGGTGAACCTTCACCCCAGTCTGAGGTCCAGAGTGCTCTGGAGCAGGTTTTCATCAAGGATCTCTCTGTAC
TTTGCTCCGTTCATCTTTCCCTCGATCCTAACTAGTCTCCCAGTCCCTGCCGCTGAATAACATCCCCACAGCATGATGCT
GCCACCACCATGCTTCACCGTAGGAATGGTGCCACGTTCCTCCAGACATGATGCTTGGCATTCAGGCCATAGGGTTCAAT
CTGGGTTTCATCAGACCAAAGAATCTTGTTTTTCATGGTCTGAGAGTCCTTTAGGTACCTTTTGGCAAACTCCAAGCAGG
CTGTCATGTCCCTTTTACTGAGGAGTGGCTTCCATCTGGCCACTCTACCACAAAGGCCTGATTGGTGGAGTGCCGCAGAG
ATGGTTGTCCTTCTGGAAGGTTCTCCCATCTCCACAGAGGAACTCTGGAGCTCTGTCAGAGTGGCCAACGGGTTCTTGGT
CACCTCCCTGACCTAGGCCCTTCTCCCACGATTGCTCAGTTTGGCCGGGCGGCCAGCTCTAGGAGGAGTCTTGGTGGTTC
CAAACTTCTTTAATATAAGAATGATGGATGCCACTTGGGGACCATCAATGCTGCAGAAATGTTTTGCTAACCTTCCCCAG
ATCTGTGCCTTGACACAATCCTGTCTCGGAGCTCTACGGACAATTCTTTCGACCTCATGGCTTGGTTTTGCTCTGACATT
CACTGTCAGCTGTGGGACCTTATATGGACAGGTGTGTGCCTTTCCAAATCATGTCCAATCAATTGAATTTACCACAGGTG
GACTCGTGTCAAGTTGTAGACACATCTCATGGATGATCAATGGAAACAGGATGCACCTGAGCTCAATTTCAAGCCTCATA
GCAAAGGGTCTGATACTTATGTAAATACGTTTTTAATTATACATTTGCAAAGATTTATAAACAAAAAAAATTTACTTGG
TCATTATGGAGTATTTTGTGTAGATTGATGAGGATTTTTATTATTTTTAATACATTTTAGAATAAGTCAAGGGGTCTGAA
TACTTTCCGAAAGCACTGTATATCACTATAATGGGTTGTGTAGTAGGACCAAGATGATTGAAAAGTATTTGAAACTTGTT
TGGATCTGTCACGTCCTGACCAGTATAGAGGATTATTTGTTATTGTAGTTTGGTCAGGACGTGGCAGAGGGTAGTTAGTT
TATGTATTCCGGGGTTTTGGGTTTCTTTTCTATGGGTTTTCTATCTGTTCTGTTTCTATGTTAGTTAATTGGGGTATGGA
CTCTCAATTGAAGGCAGGTGTTTTCTAGTTGCCTTTGATTGGGAGTCCTATATATTAGGGTGTGTTTGGTCTTGTTGTTT
GTGGGTAGTTGTTTTAGCACTGCTATACGTGTGTAGCCTGCTATACTGTTCCTGTCGGTCGTTGTTTTCTTGTTTTGTTC
GTGGTGTTCACATTATAATAAATTATGATGAGCACGCAATCCGCTGCATATTGGTCCACTTTGTCCGACAGCGATTTCAA
CATATCTTCTGACGAAGAGGAACGTGACAGGATCATCATGTTCTTGAGGATGACTCTCACACACAAGAAAATGCATTCAT
GTGGTCAATGTCAGGCAGTTGCCATGTAAAGAAGATCAACTAAACACATTTTTTTTTTAAATAGTATCCAACAGTTAAAC
TTATTTTCACCTAATATGGCCTGTGTGTGAACATAACCTGGAGAAGCAGATCATTTTTTTTTTTTTATTCCCACATTTTGT
TTTCTCCTCATTATTAAATGGGAGTCTTTTTTTTTTGTTTGGCTCTGTGCTAACAGAATGTTACAGAAAACCAATTTATT
GCCTGTCAGCCAGGAGCCTTTTCTTTCATTATTTGTCAAGGTATGATTATTTTCTTTCTTCTGTTTATGTAAGTTAAAAA
AAATCTGGGTAAACAAAAAAGGGAAAAGTAATTGGCTTCTTATTTGTTGATAACGTTAACCAGACTGCCTCAGTCTGACT
ATGGGCTTTTCAAACAAGGGCCCTCACATTTACGATATTAAACGCAAACATTGACTTGAGATATATACAGTGTGCTCCAA
AATTACTGGCACCCCTGACTGGCAATGAACAAACAATAATACTTAAAAAATATAAACAATATAATTATAGAGAGAAACTC
AAAATACCAACATGTGAGAAATACTGTACTTTATTCATGTTTCAATGGAACCAACCAAAACCATACAATTATTTAATTCA
AAATACATTTCCCCAAAATCAAGGTTTCATAATTATTGGCACCCCTCATTTAGTACAACCACCTCTGGCAAGGATAACAA
CATGGAGTCTTTTCCTGTAATGTTTGACAAGGTTAAGGTACACATTTGGAGGGATTTTGGACTACTCCTCTATGCAAATC
CTTTCAAGATCCTTCACATTCTTGGGTTTGCGCTTATCAACTGCCCTCTTCAACTCAGCCCACAGGTTTTCGATTGGATT
GCGGTCCAGCGACTGAGATGGCCATGGCACAACATTGATTTGTTGTAACAGAACCATTTCTGTGTGGATCTTGAGGTAT
GTTTTGGGTCATTGTCTTGTTGGAAAGTCCACCTACGGCCAAGTCCCAGCCTTCTGGCAGAGGCAACCAGATTGTCAACA
AAATTGCCTGATACTTGGTGGAATTCATTATGCCAACAATCTTAACCAGTGCCCCTGGACATCTGGAATTAAACAGCCCC
AAAACATCACTGACCCTCCACCATCTTTCACCGTGGGTATGAGGTGCCTCTCCTTGTATGCATCTCTGTTTCGACGCCAA
ACACGCCGATGCTGTATCTGACCAAAACGTTCAATGTTGGTCTCATCTGACCAGAGCACCTTCTTCCAGTCATAATTTAA
ATGACGTTTGGCAAACTCCAAGCCCTTGCGTCTTTCTGATTGAGAGGGGGTCAAATACTTATTTCCCTCATTAAAATGCA

AATCCTTTTATAACATTTTTGACATGCATTTTTCTGGATTTTTTGTTGTTGTTATTCTGTCTTTCACTGTTCAAATAAAC
CTACCATTAAAATTATAGACTGATCATTTCTTTGTCAGTGGGCAAACGTAAAAAATCAGCAGGGGATCAAATACTTTTTT
TCCTCACTGTACATACATACATACATACATACATACATACATACATACATACATACATACACACATATATACACTGCTCA
AAAAAATAAAGGGAACACTAACATAACACATCCTAGATCTGAATGAATGAAATAATCTTATTAAATACTTTTTTCTTTAC
ATAGTTGAATGTGCTGACAACAAAATCACACAAAAATAATCAATGGAAATCCAATTTATCAACCCATGGAGGTCTGGATT
TGGAGTAGAAAACCACACTACAGGCTGATCCAACTTTGATGTAATGTCCTTAAAACAAGTCAAAATGAGGCTCAGTAGTG
TGTGTGGCCTCCACGTGCCTGTATGACCTCCCTACAACGCCTGGGCATGCTCCTGATGAGGTGGCGGATGGTCTCCTGAG
GGATCTCCTCCCAGACCTGGACTAAAGCATCTGCCAACTCCTGGACAGTCTGTGGTGCAACGTGGCGTTGGTGGATGGAG
CGAGACATGATGTCCCAGATGTGCTCAATTGGATTCAGGTCTGGGGAACGGGCGGGCCAGTCCATAGCATCAATGCCTTC
CTCTTGCAGAAACTGCTGACACACTCCAGCCACATGAGGTCTAGCATTGTCTTGCATTAGGAGGAACCCAGGGCCAACCG
CACCAGCATATGATCTCACAAGGGGTCTGAGGATCTCATCTCGGTACCTAATGGCAGTCAGGCTACCTCTGGCGAGCACA
TGGAGGGCTGTGCGGCCCCCCAAAGAAATGCCACCCCACACCATGACTGACCGACCGCCAAACCGGTCATGCTGGAGGAT
GTTGCAGGCAGCAGAACGTTCTCCACGGCGTCTCCAGACTCTGTCACATGTGCTCAGTGTGAACCTGCTTTCATCTGTTA
AGAGCACAGGGCGCCAGTGGCGAATTTGCCAATCTTGGTGTTCTCTGGCAAATGCCAAACGTCCTGCACGGTGTTGGGCT
GTAAGCACAACCCCCACCTGTGGACGTCGGGCCCTCATACCATCCTCATGGATTCTGTTTCTGATTTTTTGACCAGACAC
ATGCACATTTTGTGGCCAATGGAGGTCATTTTGCCAGGGCTCTGGCAATGCTCCCTCATCCTGCTCCTCCTTGCACAAAG
GTGGAGGTAGCGGTCCTGAAGATGGGTAGTTGCCCTCTTACGGCATCATACAAGTCTCGTGATGTCCTGGCCTTTTTCGT
GGTAGGGCCTCCATGCTATGGACACTACGTTGACAGACACAGCAAACCTTCTTGCCACAGGTAGCATCGATGTGCCATCC
TGGATGAGCTGCATTACCTGAGCCAATTGGTTGGGTAGCAGACTCCGTCTCATGCTACCACTAGAGTGAAAGCACCGCCA
GCATTTAAAAGTGACCAAAACATCAGCCAGGAAGCATAGGAAATGAGAAGTGGTCTGAGGTCACCACCTGCAGAATCAGT
CATTTATTGGGGGTGTCTAGCTAATTGCCTATAATTTCCACATGTTGTCTATTCCATTTGCACAACAGTATGTGAAATGT
ATTGTCAATCAGTGTGGCTTCCTAAGTGGACAGTTTGATTTCACAGAAGTGTGATTGACTTGGAGTTACCTTGTGTTGTT
TAAGTGTTCCCTTTATTTTTTTGAGCAGTGTATATATATACATACATACACACACACACACACACACACACACACATACA
TTTAACCTGTCTGGGGTATGTGGGATGCTAGCATCCCACCCTTGGGACACACTATTCAACAGCTAGTGAAATAGCAGGGC
GGCAAATTCAAAACCACAAAAATCTCATAATTCAAATTTCTCAAACATACAACTATTATATCCCATTTTAAAGATACACT
TCTCGTTAATCCAACCACATTGTCCGATTACAAAAAGGCTTTACGGCGAAAGCATAACATTAGATTATGTTAGGACAGTG
CCGAGACAAGAAAAACCACACAGCCATTTTCCAAGCAAGGAGAGGCGTCACAAAAACCAGAAATACAGCTAAAATTAATC
ACTAACCTTTGATGATCTTCATCAGATGGCACTCATAGGACTACATGTTATACAATACATGTATGTTTTGCTCGATAAAG
TTCATATTTATATCCAAAAACCCTATTTTACATTGGCGTGTAATGTTCAAAAATGTTTTGCCTCCCAATACTTCCGGTGA
ATGAGCACATCAATTTACAGAAATACTCATCATAAACGTTGATAAAATATTCAACAGTTATTCAAAGAATTATAGATACA
CTTCTCCTTAATGCAACCGCTGTGTCAGATTTCAAAAAAGCTTTATGGCGAAAGCAAATTTTGCAATAATCTGAGTACAG
CGTTCAGACAACAACAAGCAATACAGATACCCACCATTTTGGAGTCAACTAAAATCAGAAATAGCATTATAAATATTCAC
TTACCTTTGATGATCTTCATCAGAATGCACTCCCAGGAATCCCAGTTCCACAATAAATGTTTGTTTTGTTCGATAAAGTC
CATCATTTATGTCCAAATACCTCCTTTTTGTTCGCGCGTTTATTTAGTCCACTACTCCAAATGCAGGAAGCACGCACAAA
ATGTCACGACGAAAAGTCAAAAAAAGTTATATTTTCGTTCGTAGAAACATGTCAAACGATGTTTACAATCAATCCTTAGG
GTCTTTTTATCATAAATCTTCGATAATATTCCAACCGGACAATAGCGTATTCATTACAGAGGAAAAAGAAGGAATGGTGC
GCCTGTGTGTCCGTGACTGCGCAGTAAACAACTCATTGGCCTCAAGCAGTCCACTTGTTGAGTCAGCTCTTATTCTCTCC
CCAGTCACAGTGGAAGCATGAAACAACGTTCTAAAGACTGTTGACATCTAGTGGAAGCCTTAGGAAGTGCAACATAAACC
CTAAGTCACTGTATACTGTATAGGCAATCACTTGAAAAACTACAAACCTCAGATTTCCACACTTCCTGGTTGGATGTTTC
TCAGGTTTTTGCCTGCCATATGAGTTGTGTTATACTCACAGACATAATTCAAACAGTTTTAGAAACTTCAGAGTATTTTC
TATCCAAATCTATTAATAATATGCATATCCTACCTTCTGAGCCTGAGTAGCAGGCAGTTTACTATGGGCACGCCTTTCAT
CTGGACGTCAAAATACTGCCCCCTACCCTAGAGAAGTTAAACTCCGTTTTTCACAATTCCTGACATTTAATCTTAGTAAA
AAGTCCCTGTCTTCTCACCACTTTATTTTAAGAATGTGAAATGTCAGAATAATAGTAGAGAGAATGATTTATTTATTTCA
GCTTTATTTCTTTCATCACATTTCCAGTGGGTCAGACGTTTACATACACTCAATTAGTATTTGGTAGCATTGCCTTTAAA
TTGTTTAACTTGAATCAAACATTTCGGGTAGCCTTCCACAAGCTTCCCACAATAAGTTGGGCGAATTTTTGGCCCATTCC
TCCTGACAGAGCTGGTGTAACTGAGTCGGGTTTGTAGGCCTCCTTGCTCGCACACGCTTTTTCATTTCTGCCCACAAATT
ATCTAGAGGATTGAGGTCAGGGCTTTGTGATGGCCACTCCAATACCTTGACTTTGTTGTCCTTAAGCCATTTTGCCACAA
CTTTGGAAGTATGCTTGGGGTCATTGTCCATTTGGAAGACCCATTTGCGACCAAGCTTTAACTTCCTGACTGATGTCTTG
AGATGTTGCTTCAATATATCCCCATAATTTTCCTGCCTCATGACGCCATCTATTTTGTGAAGTGCACCAGTCCCTCCTGC
AGCAAAGCACCCCCATAACATGATGCTGCCACCCCCGTGCTTCACGGTTGGGATGGTGTTCTTCGGCTTGCTAGCCTCTC
CCTTTTTCCTCCAAACATAACGATGGTCATTATGGCCAACCAGTTCTATTTTTGTTTCATCAGACAAGAGGACCTTTCTC
CAAAAAGTACGATCTTTGTCCCCATGTGCAGTTGCAAACCGTAGTCTGGCTTTTTTATGGCAGTTTTGGAGCAGTGGCTT
CTTCCTTGCTGACTGGCCTTTCAGGTTATGTCAATGTAGGACTCGTTTTAGTGTGGATATAGATACTTTTGTACCTGTTT
CCTCCAGCATCTTCACAAGGTCCTTTGCTGTTGTTCTGGGATTGATTTGCACTTTTCACACCAAAGTACGTTAATCTCTG
GAAGACAGAATTCGTCTTCTTCCTGAGCAGTATGACGGCTGCGTGGTCCCATGGTGTTTATACTTGCATACTATTGTTTG
TACAGATGAACGTGGTACCTTCAGGCGTTTGGAAATTGCTACCAAGGATGAACCAGACTTGTGGAGGTCTACAATTTTTT
TTCTGAGGTCTTGGCTGATTTCTTTAGATTTTCCCATGATGTCAAGCAAAGAGGCACTGAGTTTGAAGGTAGGCCTTGAA
ATAATTCCACAGGTACACCTCCAATTGACTCAAATGATGTCAATTAGCCTATCAGAAGCTTCTAAAGCCATGACATCATT
TTCTGGAATTTTCCAAGCTGTTTAAAGGCACAGTCAACTTAGTGTATGTAAACTTCTGACCCACTGGAATTGTGATACAG
TGAATTATAAATTAAATAATCTGTCTGTAAACAATTGTTGGAAAAATGAATTGTGTCATGCACAAAGTAGATGTCCTAAC
CGACTTGCCAAAACTATAGTTTGTCTAACAAGAAATTTGTGGAGTGGTTGAAAAACGAGTTTTAATGACTCCAACCTAAG
TATATGTAAACTTCCGACTTCAACTGTATATACATACATACATGCATACATACATACATACATACATACATACATACATA
CATACATACATACATATACTGCACAGAATCAAATCTGAATTTATCTACATAAAAAAGCGATTTTTTTTTAAATATGGACA
ACAGGGTCTTATTGCCAAATAAACACTCTCACAGTGTCACACATTTTTGTGAGACCTTGCATTGCCCTGCATTAGGCTTC
AAAGAGATGTGTGAATGATAAATATACATATTGGTTGTTCACCGTATAAAGGCTTTTGGATTAGATTTCAGGAGCAGCTC
TCATAGCGATTAGTGGCAACTTAAGAAGACCCCTATGGGTGTGTTCCATTAGCTCTTAANNNNNNNNNNNNNNNNNNNNN
NNNNNNNNNNNNNNNNNNNNNNNNNNNNNNNNNNNNNNNNNNNNNNNNNNNNNNNNNNNNNNNNNNNNNNNNNNNNNNNN
NNNNNNNNNNNNNNNNNNNNNNNNNNNNNNNNNNNNNNNNNNNNNNNNNNNNNNNNNNNNNNNNNNNNNNNNNNNNNNNN
NNNNNNNNNNNNNNNNNNNNNNNNNNNNNNNNNNNNNNNNNNNNNNNNNNNNNNNNNNNNNNNNGATGTTTTTCAGTTGC

AGAGACTGGGAGTCTAGTCGGGATCAAGGGAAAGATGAACGGAGCAAAAGTACAGAGAGATCCTTGATGAAAACCTACTC
CAGAGTGCTCAGGACCTCAGACTGGGGTGAAGGTTCACCTTCCAACAGGACAACGACCCTAAGCACACAGCCAAGACAAC
GCAGGAGTGGCTTCAGGAAAAGTCTCCCCCCCCCCCCCAGAACCGCTAGTGCCTGGAGCCCCCCAGGTGCACGTTTTGTT
TTTTGCCCTAGCACTACAGAGCAGATTCAAATAATCAAAGCTTGATGATGACTTGGTTATTCAGAACTGAGTTTGGGAAA
CCCTGATGTACTGTAAGACATACTATTGTGTTACAGTAGTGTACTATAGACTGGTAGAACAATTGAGTTATTATATTAAT
TCACAGACATGCACAGTTAAAAGGGAAATATATCAACCAAAGTAATTGGTCAATAGAAACGGGCACAGTACGTCTTTTTG
ATTTGTAGTTTGACTGGGAAAACACTGTAACTTCATCATTTCATTTGAGTGAACCCGTGTATATACCACCATCGATTCAA
CCACACATGCTAAAGTGAGAGTTACGCTAATATTGTCATTAGCATTTATTTGTTGATGTATTATACAGTGATATGAAGCT
GTGAGTATTTCTCTATGACCAAATGATCCTGACCTGACCTGACCGTGACCTCTCATATACTTTCCGTTCCGACGTTCCCA
TGGACTTTGCCTTTTTCTTCCCACTTTATGCCGCACTGATTCCAATTACCAGCAGTGACATTGCCACAACCATTTTCACA
TGTGACGGATCAAACAGACAGACAGCCAGACTCTGCACCCCTCCTCTCCACCTCCTTCCCTCCATCCTGCTCTTTGTCTT
GACTGTTTCCCACGCGGGCCAGCTGTTTGGACCGCTTTAAATGTCGCCTCATAGTGCGTTCTGGGAGCTGTGGATGGCTT
GGCTGAGCTGCTTGGATTGTGCTGATACGACTAGGCTTAAGGACAAAAGAAGAGAAAATACCCCCCCCCCCTTCCTTATC
CCCCCACGTCAAAATATCTGGTCACTCAGCGAGTCTACTATCTTGTTAATTCCAAGCGTCTGTCTTCACCACTCCTATTG
GGAGGTTAAGCCCTGTCGAGAAGGCATCAAAGCTCTCCTCAGAAAGCGGCTCGTATCATAGAGCTAAACAATCAGCTTTG
ATTGGATTTTTATCCTGTCTTATTAACTCTCAGACCAGGGAGCCAAAGCCAAAGGCATGATGCTAATTGCCAAAACAAAC
TGAAAATTCTCCGTGGATCAACACTGACATCCGGTCCTGCTTACGTTTGGGTTGTTGTACTGCAGTAAAGTATTATGACA
TTTGATAGGGATGTTTCTGTTTGGCAATAGGGTTGACCTCCCTGGTCATGGATGAGTTCAACTAGATTCCATTTGAATGT
AACCTTTTTGTGAGACTTTTCTTGGTTGTACATGATAATTCCCTGTGTATGTTTTAAAGTCCTAAAGAAATCCAAAAGCC
GAAACGCTTTGGTTCTACTTTTAACAATAAAGAAGCTTTTATTTATTGAATTCCATTGTCCTCCAAAAGTCGCTGAATCC
CCTCTCTGTTTATCACGTTTACCTGAATTTGAGTATCATTCACTGGACTGGACTACTACTACTACTGCAGTCATTACTAT
CACTACCATGACAAAACACCTGAACCAATCACACACGTGTTCTTCAGAAACTGTACCTTGTCTCGTTCCCTGTCTTTCAG
ATGGCTCATCTACACTGTGTCTTTCGTCTGTGCGGGAGCTCCCAGCCCAGCTCCAGGACCTGTACCAGCAGGGCTTCTCC
CTGGTTGCCGTTCACCCCTTCATTCATCCCTGTGGTTCCAACGCAGTCAGCCTCCAGCACCAGCTCTACAGGGCCGTGCT
GATCCGACTCGATGATGGGTACGAGTACATACACATTGCACTTATTCAGAGCTCCTACATTGACATAATTAGCGTAAGCC
TTATCCAGGGTCAATACATTTGTGTTTACGTAATATGTCATTACGTAATATGTATAGCAAATCAAAATGTATATTACCAC
ATGGATGTAGACATTGGTATTACAGAGGTTGTTTTGGGCTAGCTGTCACAGGGCTGTTGTTTTGGACGTTAGCAAGCAAA
TAATCTCTTTCCAAGCTTTTGATGCTCCGGCCAACATCTGGAATGGGCCAAAGTCACAGCTGCACCCCACATCTGCCCTC
CACCCGGGCCCCTGTCAGTGTTGCAAACCCTGGGTGAGAGTTAGGTTGGATCAGGGCATGGTTTTACTCCAAGTGTATGT
GAAGGGCGTGGCGGGGTGAGGCTGGTGGTGGTGGTTGCAGGCTGAAGCCCTGGCTGGCTTGCCTCGCGTGCCCCGTGTTC
TACCAGCTGGCTGCTATGAAGAACAGCGGTTTCATGCGTGTTTACTGAGAGTGTTAGGCCACATGCAGGTCTGTCTCTGG
GAATGACTGCCTGTGTGAAGTAGAACCAGACCCTCACTAGTCACTTTCCCCACAGAGAGGTTAGAGGGGCAGCAGTTAAA
CCTCTCACCCTCCCCTCTTAACAGTGTTTTATGGCCTGGTCTGTTCTCTCTTGTGGATCCAGAGCCCTGGGGTCGGACAG
TGACACCAGACTCCATAAATTACCGGACTCTCCCCTCGTTGAATTATGATCCAGGAATTCACCTCGAGATTCCTAAATAA
GTAATTCCAAACATGGACAAATAATTCCTGCACATTCCTCAGTATATTGGGGTTTAGACTCTTTGCCCTGTTGTTCTTGT
TTAACGCTTCCTGGTTGCCTAGTGTTGTCTTGAAAGGATCAAACCATTTGGCTGGTACAGGTGGAGGGTTTAGTTAGTTG
TAAAGCACAACAATCCATCCACTCAGGTGGCTCTTGCCTTCTTTCTAAAGACAGTCTGTGCCCATTAATGAGCTCCTAGG
GTCTAGGGAAATACATCACACCTCTCTCCCACTGTCTCTACACAAAAACCCACTCACAAACAAGTTCCAGACCGTAATCC
TATGCTGGGAGCGCATAAATATTGATTCTGTATGAAAAGGGATGGATGAATGGGCAGCAGCATGGGGTTTTTACACACCG
ACATGGTTGCCTTCCTCTGGAGGAAAAAATAGAATATGAGGAGAGGACTAGAAAGCGATAGAGAGAGAAAACAAGATCAC
AGCTGGAGTGTGAAAGAGCAGGAGTTAGAAAGGGGAAAGGGCTGCCATCCTTTGATGGCTCTGGCCAAACCGTCCTGCTT
TATCACAGTGTCTGTCACCGTGGAGACGGCTGCTCGCATAAGCACCAGCCCGGGGAAGGGGGGGGGGGGGGGGTAAAAAA
AATAAAAAAATCAACCAGGTAGCATCTTCCTTTCCTATTCCCTTGTCAGTCCCTTGAATCAGGGAGCAAAAACTGCAGAT
GACAGCGCAACCATTCATCACTCTCCCAACGCAAACAGACCCAAAGTGTCTTCTTCGCTCTGCAGTGCGCTATGCGTCAG
CTGGAGACAGACAGAAGGAGAGACAGACAGAGGGGGAGAGAGAGAGTGTGTGTGAGAGAGAGAGAGAGAGATGGAACA
TGGACCAGAACAAGAACAGCAGCTAGGCTATGCTTAGCAGCATCTGGATGTGCTGTGTTTCGTATGCACACAATGTCTCT
TTGGATACAATCCCAGACAATTTTCTACAGTATTAGCCTCAATTGTAAAGCATCAGTGAACAAGGATATAGCTGCACAGT
AGTGTGACATTGCAAAAGCTTCTAGAGCCAGGAATTACACAGGGGCAGGTAGTCTATATAGAGCTGGGAGCCAGTATTGT
TCATGTTAAACACTGTCACCTGGAACGACTATTCAGTTAAAGATACACTCTCAAACTTTTGTATCATTTCAGCCGGTAGT
TTTGAAAGTAGCGCTCACGAGCCAATTAGTGGTCCCCTTTTATTTGTGTACTATGTCATCAAATTGTGTACTACAGTACA
TCATTCATTTTGTATGATATTTTATGTCCTGCAAATGTTGTTTTTTTGCAATGATATGTTTTCAATCCAATTTGTACTAT
ACGTTACAAATTTGTTGTGCTTAAGATCTGGAGTGCATCTTTAAGTAGGCCATAACTTTAAAAGCCTGTTGTCTCTGACT
GTGATTTGTCGGTGGTGACCGTGGTAACCTGCTCAAATCTGCGGAACAGAGAGAATGGATAGGACCATTTTAATTTTCAA
ATGTCAACTGTCAAATTTCCCAATGTTCAATTTGGTTGCGGCGTAGCCGGGTTACAGTTCAGCAGGCAGTGTCTGATGGG
ATATATTCATCATCTATTGGATAATTGTAGAATTCACATGCGCTCGGGGAGAGAGCTGTATGGACCAGAAACCATTACAG
GGGATTCGTCCAATGAAATTATTCATGACTCTGTGAAATCTGACATTAGAAAAGATGTGCTATATTATTAAAGGCCAGGG
ATGAAAGATTGCTAATCACAGGTTCGATGGTGGATCCTGACCGAGCCGTTGTCATGGAGATTGTGATTTTTGAATTGCAC
TTTTGGCGCCCAATGTGCGGGCTTCTGGGTTTAAAGCTACATTCTGGGATTCAACAAACTTCCACACGCCCCACCATATC
AAGAATTAAAATAACCATTCCCAGATTCCAAACCCCAGACTGTGCCTTTTAATACTCCCTGTGGTCCCGGGACACAGAAC
CTTGAACGGTTGAACCTGACTGACAGTGCAGCTGAATAGATAGCAGCAGCAGCAGCAGCAGCAGCAGCAGCAGCAGCAGC
AGTCTACATAGAGAGTGGGAAGTCTGGGAGACGTACACCCAGGAGGAGGCTGCAGTCAGGCCAGCTGCAGGCTGGAGCCA
GGCATGCGGTTGACACTTACTCTAGCCACATGTGGCCTGGCGGTTGACAGCAGAGCTGTGGGGTTGAATGCAAGTTACCT
GTTTCACACCGAGAAAATGGACTGCAGGTGATTTTTCTAAGAAATCCGTGCCTCTAGAGAGCCGATGGGAGACTTTTTGA
ATGTACTTTTCTCCCACTTTGTCCTTTTTATGGATTTAAGCCCTTCTCAGGTATACAGATCCAATCTGCTTTTCATGATT
TCCAATAACAGAACGTGGAAAACGAATAGCAAGAATTCAGCGCTTTTCCTTTATATTGAGGCTTCACGGTGCTGTGAATT
GCACAGTAAGTTTGCAAAAACCTCTACCTGATTCAGAGCATCCAATGTGCAGATTTTCCTTGACACAGTTTGCTACTGAC
ATTTGATCTGGGATAAGTTGAATAATGATGGGGGACTGGTGTGACAGTGATGTCTGACTTCTGTGGGTTGTTGTTGACTG
TGAGATGACTCAGGTTCCCGCTGTGTTGTGAAGCCAGTGTGACAGAGAGAGAGCTAGGCCTGGGCTGCATGTAATAATGT
TTCAAGTCTCCTTACACACACACACACATACACACACAAATACAAGTATGTACTGTACACACACACACACACACGCGCAC

ACACGCTGTGGCGAGGGTGGAAGAGCCCCATCCGTGTTGTCAACAGGATGAGAAGTGAAGCAGAGCGATGGAAGCTGGGG
CATTGGCCTCCTCCAGGGCTAGCTGTGGGATTTCCTCCAGAGAAAATAGAAAAATACATATAAATAGATTCCATCCACCC
ACAATACTGCTAAGTGGAAAAACAAGACCAGATTTGTATATCATTGGGTTTCCTATCGCACCCTAACGTGCCCCTTTCAA
CCCTGATTTGTGTCTACGTTAAGTGCTTTTTTTTGTTGTCATACAGGCCAACAATTACATTAACAGGGCTCATATGTGAG
TTATTCATATGGATGGGCATAGATTCTTTGCATAAATCCATAAATACTTCATTTCCAAGGATTTCGATGTGCAACCATTT
CAGATATGTATTACTGAATGTGGTGAATGTCTGTGTCGTGTAAGACTGAAATGACTGTGGCTGAAATGTGTTGTTCCTCT
CTCTCCCGCCGTGTGGGCTGCTACCGCAGGTTGGAGAAGAGCCAGTCGAGCTGTGCTCCCTACCGTCTGCAGCTGGAGCA
GTGTCTGTCTACCGACCAGGTGCCCACCCCTGAGCTCATCCAGGGCTATGTCAAGAAGGTGAGCCTCAGATAAGGCCCTG
TCACACTACTGAGACGAGCTGAGCCAAGCCAAGCCGTACTGAGCTTGGCCTGGTTAGTGGTTAGGCATCCACAACTGTGC
TGAAAAGGACATCGACGTGAACGGAAAATATTAAGTCGGCACATTTTTGTTCGGGTGGCATGATAGTGTGAAAAGGGTAT
AGTGGACTCAGATCACAGGCACATCTGACCTTCCTATAGGTCCTAAAGACCCTGTGGGCAAAAACATCTTTGTTGTCTTT
ATCNNNNNNNNNNNNNNNNNNNNNNNNNNNNNNNNNNNNNNNNNNNNNNNNNNNNNNNNNNNNNNNNNNNNNNNNNNNNN
NNNNNNNNNNNNNNNNNNNNNNNNTGGAAACAGGGAAACAGGGTCACACGCCAAACAGTCAGAAAGTGAAAACTGGGACAT
GAAACCGACATGCTCTAAAATAGCATCAAAGAGCGTTGAGGTTATTTAAACTTAGCGCTGAGAACATTCCTGTGGGATCA
CAGGTGTGAGAGGAGGAAGTGATGGTATATAAAAAGACAGAGATTGGACAGACATCACAATGGTCAGTGAAATGAAGCAG
TATGAAGCTAACCACTCTCCAACTAAAAATCATGTTGCAGAGGAAGCTGGCCAACATGTTGGTTTGAGCATGTATTTATT
ATGAAATGTTAGTCCTGATTTAAATCGGTGTTGAGGAGGGTGGATTAGCAGAACAGATGCTGTCATGTTCAACGGTGGTT
CTGCACAGTGCACATTATCATTTAGGATTAAGGGGCCTGACCTTGTCTCTTGGACAGGCATCCATCAGGCCAGACTTTAA
AGGGACCCATTTGGAATGTCGTAATTGCGTTCAGGCGCTCGTTTACTCGTTAGTCGTCAATTTTCTCAAATGATTGGACC
ATACAAGGTATCGTCTCATAGCAGGCCTGTCACCTGATATTTTCCATGTGACTGTGGCCCGGCAGGTCACACACACCCTA
CCGTGTGTTATCGCCATCCAATTCTCAGGGTAAGTCAAGGGCGACCCTAAAATATTTGTTTAACCCTGATGCCCCTGATG
CTAAAGTTGTCGAGTCAGTTGCGTTGTGAAGTGGTTATCATGTGGGAATGATCTGATAGGTATGAATCCCCTGGATTATC
TTTAACTCCAGGTTATAACTCACCTTTAACCCTTTGCGTACAAAGCTAGCTGTCAAATGGATATAACACATGTCACAAAT
TGTTCAAAGTGTTGTTATAAAATGTATGTTATACAATTTACCACGATTTGTCTTCTCTTTAAATGCGTTACCATTGAGCT
GTCTGGTTTAGTCGGATGCATTAGTGCAGGGTCTCTGTCAGCTGTGCATAAATTACAAACGGTCTGTGACACATTGCAGA
CGCAGAGCATTTAGTGGAATGGTGAAGCTAGCAGTTCCTCAAAACCGCTGGCCTTGAACGAGTAAACATCACCTTTTAAT
ATTATGTGAAAACCTCATTTTAAATTCAGTCTTTTTGGGGGAAAACAAACAGAAAAATGAAGATTAGTGGAGCGACTTT
GTTGTAATGACTTGGCTTGGTTTTCAATACGTTTTGTACTTTCTCCAACGTATGCAGGGAGAGTTGTGATGCATTATTGT
CATTCAGTTGTGATTCTGGAGCTTTGAAGAGTGGAAAACGACAAGATTTGGGGAATTTTGTGGCCAGCTGTCACATCAGT
CAGATGTGGGAGAAAGGAAATGGTTATGAATGATTCATTCTTCCTGGGTGTCTGACTGAATGACTGACAGAGAACCAGAG
CAACTGAACCATGGTCCCCTCTGATGTAGTGTGTGTGTGTGTGTGTGTGTGTGTGTGTGTGTGTGTGTGTGTGTGTGTGT
GTGTGTGTGTGTGTGTGTGTGTGTGTGTGTGTGTGTGTGTGTGTGTGTGTGCGCGTCTTTTGTGAGTGTTCGTGTGTGTGGGAG
TCTAACACATTCTGGCCCTCCGTCTGGTTCACATGGGGATGGCAGATATTAGCATTAAAAAGAGAAGATTATGCAGCTCT
TGATTTAAATGGCTGCTGCCGAAACAACAGTCCATCCAAATGTGTATGCATTGGTCCATATTAAAGGAGGACCATTTCAA
TTGAGGCACAAGCCTTTGGTAGGGGATTGTGGGGAAATGATGAGTCAACTCTGTTATGTGTTATTAATCACCTCCGTTGT
GGGGAATGAGTCAACTCTGTTATGTGTCATAAATCACCTCTGTACGGGGTATCAACTCTGTCACGATATAAATCTCTGTT
GCGTGACATGTTTCATGTCGGCACAATCTATAGCGATGGAGATTGAGTGTTCTGTGTTCTCAGGGGTGGAGCTGCTAGCC
CTGTTCAACCACCCAATTGACCGTGAGGGCCAACTGAAGTACTACACTGGGAAGGTACCGCTGAGGGTTCAACTCCGTGA
CGAGGGGGTGAAAGGGGTGGAGGCCAACTGGCTGGATCACATGACCCAGCACTTCAACAACGGGGCCTCACTCGTTGATG
GATACTTCCACCTGGGCAACGACAACGGTAAACAAACTATCCAATGTCCACTTCATGTTTTTACACGCAGTACTTTTTAA
TGTAACATCTGTTCTGCTGTGGTATAGTACTAGCAAAGTAATTGGCCTCTTCACAATATCTCAGTACTTTTTTCATCCGT
CTGGCAAACCTCTTATTTTGTTCCACGTAAGTAAATAATGTAAAAAATAAATAATAATAATAAGACAAAGGAAATGATGA
CTTAGCTATTGCCAAAATATCCTGTCCCCTTTTCAAACGGTTCATTCTGTGCTGTTGTTGTTCTAGTAGTACTCTGTGCT
ACTCCTCTACTGCACATTGCACTCCAGCTCTAGACCCTGGGGCATGACACTTGATTCTGATGGTGACGAAAACCCCCAGC
CTGAGTAAATAAGAGTCATAATTCATTCATCTCCTAAAGTGAACGCATCAGCGCCTCTGAAACACAGATAATTAACCTGA
TGGCAAATGAGTGCCTCCTTGCATATTTTATCTCTTTAGAATGGGGTAATGAACCGCGTTATTATAATTCCTTATTTTCT
ATTAGGTTATTGTAATATATTTACCATTTTTATGTGTGGTTAAGATATGCATCTTATATTACAGCAAGAGCGTATTATAA
AAATTCATATTGGCCAGGCCGTGTTCTGTATTGATCTGGAAATGTCAGCTCTGTATCAGAAATAGCTTGTCAAGTGTACT
GTATAGGGAACGGCCTGTCAATGCATGCCTGCCAAACCCCTTTTCAAAACAGACAAGTTTGCCGAGATGAACGTTGTGTTC
GTGTGACCCTCTTGGGAACTGTGAACGGTAGGATATCAAAGCCAAGACAACATCACGTGTTTGTTGCTAATTACATCAAA
CGGAGGAGCGTCTGGTTTTAACAAGCCTGAGAACCCATGGCCGTGCAGATAAATTTAGCACCTCTTTGAGATCCATTTCT
GTATACGTTCGAGCATGAGAAGAAACTCTAGAGCAATGAGAAGAGACGCTTGAGACCGTCTCCTCTCTGTAAACATCAAG
GGCATTCTGGAACGTGAAGCAGCACAATATAAGATGGAGCGTGTCAGTGGCGATGACACTCGGTTTGATTGGAGATTATA
ATGACAGTCACAGCAGAGCACCAGAAGTGCAAAATAAGTGTCTCTGATTGTCATCATGTCCTCTGTCTCGGATGTAGTGT
AATTCAACTGTGTATCAATACTCTGCATGGTCCTGTTATGCCTTGTTTCCTGAAAGAGGTCTTGTTCTTCTTGTCGTTAA
GCTCTTCCTCTGAACTGTTTGGTAGTGCGTCTACTTTCCCAATGTCCTCCCACTTTACATGACATCACAGGCCTACCGAA
GTAACCGCCAGTGGAATAACCTATATATGTAACAACCCTAAAACAAGCCAAGCAAATGTGGTTACCGGTAGGCAGTTACC
CCTTTTAGGTTCTCTTTCAAAACAGAATTAACAATGCTAATAACTATGTCTGCCCCTGTACCTTCTAGTTACAGAGGAAT
CATCTCTCTGCGATGTAAAGTTTCCCTTCATTGTTCTGTACTTGTTTCGCTGTTGTTTACTCCTACAGTCCGACGCCTCT
TCGAATACATATTCATGAATTCCCCCTGCTCGGCTGGCCTTTTGCTAAACCCGGCGCTACCTTCCGACTGTGGCTGGCTG
TCGACAGCCTGCCTGGTATGGTCCAGGGGAATACCACACATGCTGTCTCTCCTCCTGCTCCTCTGCCCTCGCCCCCGAAG
AGATGGGTTTCCATGGTGAAGCTCACTGAGGAGGTGGGGAGGGGGATAAGTCCTGTTTAATATCCCGGCGTGGCTAGGTG
GGATGGAGGCGGGAGACTGGTGGGGAGTGGCTTCAGAGAGACCTCGCCGGCACGGAGGGTCGGATATTAGTTTATTATTG
AATTTGCGGATCGTGGCACTGGTAGGCGGCTCCTATGTAACGTTAACGGCCCCGCCACCCTGCAGCGTCATGAGAGGGGC
ATTAAAGATTAATACCAGGCCCAGCGTGTCACGGCGCCCATGCCGCCCAACGCCACGGCTGGAGTTCTTAGGGGGAGCAG
TTTGTTTACTGGTGAATTGATACTAATGGTGTTGATGGGGAGTTTTTCTTTGTTACGCTTAGCTTACGCTACCTCCGCCG
CAACAATGAGGATTTGAAGCTGGCGCTGTGAAACATTTGAGCTATTCATATGCATGACAACTAGCATAGTGCTTGGCTTT
CTATATTCAGGCAAAGCCGGTAGAGAGAGAGAGAGAGTTCCCCAGAATGAAATTCCAATAAGGTATTACTTCACAGTGAACA
GAATTGAGGAAAGTTTTGCTGGCAATTTTTATGGCCAAAGATTGGTTGTGAAATAGGGCTTGAAGGCCTGTGTGCGTGTG

TGTGTGTGTGTGTGTGTGTGTGTGGGTGTGCGTGATTGTGAGCAGTGGAGCAACAAAAAAAAAGATACCTTAATAGAAAA
TGACTCAAGTTAAAGTCAACCAGATAAAATACTACTTGAGAAAAAATCTAATATTTGGTTTTAAATATACTCAAGTATCA
AAACTAAATGTAATTGCTCAAATATACTTAAGTATCAATAGTAAAAGTATACATCATTTCAAGTTCCTTATATAAAGCAA
ACCAGACAGAACAGATGCAGTAGCGATGACCAGGGATGTTCTCTTGATAAGTATGTGTAATTTGGAGTCTTGCTAAGAAT
TAAAATTGTAACGAGTACTTTTGAATGTCCGGGAAAATTGATGGAGTAAAAAAATTTGTCAAAAATTGAAATAGTAATGT
ACATAATTGAGGAAGATACCCCACAGATATCCCATGTGCAGATACCCCACTTAAGTAGTACTTTAAAGTATTTTTACTTA
AGTACTTTACACCACTGACTGTGAGTGTGATTTTGGGTGAACATGCCTTTGTGTAAGGCAGGGTACAGTATTCATGTCAT
ATTCCTTAAAACCCTAGCTATAACCAGAAGTCTGCCCGGTAAGCACAGGTACGTGGACCCTAGCCTCCGAACCTTTGGCT
CATAGGAGGAAAGCCTTGGCCAATTCACACTAGAGGTCGACCGATTATGATTTTATTTTATTTTTTTACGCCGATACCGA
TTATTGGAGGACCAAAAAAAGCCGATACTGATTAATCGGCCAATTTTTTTTTAATTATTATTATTATTATTATTATTATT
TTTTTTTTTTATATATATTTGTAATAATGACAATTACAACAATACTGAATGAACAATGAACCCTTTTGTTTTAAATTAAT
ACATCAATAAAATCAATTTAGTCTCAAATAAATAATGAAACATGTTCAATTTGGTTTAAAAAATGCAAAAAACAAAGTGT
TGGAGAAGAAAGTAAAAGTGCAATATAAAAAGCTAACGTGTAAGTTCCTTGCTCAGAACATGAGAACATATGAAGCTGGT
GGTTCCTTTAACATGAGTCTTCAATATTCCCAGGTACGAAGTTTTAGGTTGTAGTTATTATAGGACTATTTCTCTCTATA
CCATTTGTATTTCATATACCTTTGACTATTGGATGTTCTAATAGGTACTTTAGTATTGCCAGCCTAATCTCGGGAGTTGA
TGGGCTTGAAGTCATAAACAGCGCAATGCTTCAAGCATTGCGAAGAGCTGCTGGCAAATGCAGTAAAGTGCTGTTTGAAT
GAATGCTTACGAGCCTGCTGCTGCCTACCACCGCTCAGTCAGACTGCTCTATCGAATATCAAATCATAGACTTAATTATA
ATATAATAACACACACAAATACGAGCCTTAGGTCATTAATATGGTCAAATCCGTAAACTATCATTTAGAAAACAAAATGT
TTATTCTTTCAGTGAAATATGAAACCATTCCGTATTTTATCTAACGGGTGGCATCCCTAAGTCTAAATATTGCTGTTACA
TTGCACAACCTTCAATGTTATGTCATAATTACGTAACATTCTGGCAAATTAGTTCGCAACGAGGCAGGTGGCCCAAACTG
TTGCATATACCCTGACTGCGTGCAATGAACGCAAGAGAAGTGACACAATTTACCTAGTTTAATATTGCCTGCTAACATGA
ATTTCTTTAAAATAAATATGCAGGTTTAAATACACTTCTGTGTATTGATTTTAAGAAAGGCATTGATGTTTATGGTTAGA
TACAGTCGTGCAACGATTGTGCTTTTTTCGCAAATTCGCTTTTGTTAAATCATCCCCCGTTTGGCGAAGTTGGCTGTCTT
TGTTAGGAAGAAATGGTCTTCACACAGTTTGCAACGAGCCAGGCGGCCCAGACTGCTGCATATACCCTGACTCTGTTGCA
CAGAAGAGAAGTGACACAATTTCCCTAGTTAAAAGAAATTCATGTTAGCAGGCATTATTAACTAAATATGCAGGTTTAAA
AATATATACTTGTGTATTGATTTTAAGAAAGGCGTTGATGTTTATGGTTAGGTACACATTGGTGCAACGACAGTGCTTTT
TTCGTGAATGCGCTTGTTAAATCATCACCCATTTGGCGAAGTAGGCTGTGATTCAATGATAAATTAACCGGCACCGCATT
GATTATATGCAACGCAGGACAAGCGTGTGTAGTTAACTAGTGATTATGTTAAGATGTATTGTTTTTTATAAGATACGTTT
AATGCTAGCTAGCACCTTACCTTGGCTCCTTGCTGCACTCGCGTAGCAGGTAGTCAGCCTGCCACGCAGTCTCCTCGTGG
AGTGCAATGTAATCGGCCATGATCGGTGTCCAAAAATGCCGATTACCGATTGTTATGAAAACTTGAAATCGGCCATTCCG
ATTAATTGGTCGACCTCTAATCCACACACATTAGTAACCCCACTCTTTCTATATCTCTCCTTCTCTCTCTGGATTCCTGT
CTGTCCCGTTTGAAAATATAGATAAGCTGTCACAGCCCCGTCTCCTGTTCTCCCCGGCAGTGCTAAGCATACATGTTTAA
TTCATGTCAGTAACAGACAGGACTTGGGGCAGGGTCATTCGGTTGCTTCTGCGGTCAACAAGGCTTTAGATTCCCCTCCG
TTTTTTTACTCCTCTGTGTCTCTCCTTCAAAGTCTATGTCAACCTAGCATCAGTCTCTTTGTCACTTAGTGCCTGTCTTC
AGTCCACTTCCCTGTCACTGGGCCCTAATTAGGTTGGTGCTCCGTGTCAGTCGGTGTCACGACTCACGCCGCCATTGTTA
CTATCTCTCAGTACATCTGTGTCACTCTTCGTCTTTCAGTTCACTCTCTCTGTGTCAGAGATGGTATGCGCACATCTCAC
TCAGTATGTTCAACCTCAGGTGCCTGGAGAGGGTAGGCTAGTCGATGAGATTACATTGTCCCATTGCGAATGAACACGTG
TTTCCCTCCAAGCTTCTGTTTCACTCACTACATTGTCAGTTAAGACTCAACTCAAAATGTTATGCTGTTCTTTGTGTTGG
CACTACATATCCTCCAAACCTCTCAACTCCACGGTAACTCAGTGTCTGGCAGCATCGTGCCAGGAGGCAGCGTGTTCCTC
CTAAAGCAAAGGGACTCGATCCCTGTGTGCATCTCACCTGAGTGATCCCAGTGCATCGGCAGGCCAACCCAATCTCCAAG
AGGGCTTTCCAATTACAAGTCAAGCAGGGAGACATCTGATGAACAATGGATGGGGGGGGGTGTAAAGGAGGGGTAGGGTG
GAGAGAGGGCGGGAGAGGGGGGAATGAACAACGACAATGTCTGTATTTGAAAGGAGCCAATAGATGTCTGAAAGATGTCT
TCAATTGAGCACGTCCAGCAGCCTTAAATGGGTTTTCTGGGGGGAAATAAAGGACCCTGTCATCCCTCCCTTTCCAGCCA
GCCCTCGTTCCCCCATTATTAGGGCAGAGTAATGAAATGTGGAGTGTCTCATGGTGGAGCCTGGGCCGCACAAACTCTAT
TAGTGTGTAAATGGTACAGGAGTAATGAGAAAGCACTCAGGCCAACTGAGAGCCCTGGTCTGAAGGGCAAGAGGTTCTGG
GACACTGCTCAATGGAATCAAAAGAAATGCGGGATGGAGAAGAAGCTCACCAGCTCGCTAATGTTCCTATCCCAATAATG
CCCAAACTGGGAATGTTACCCTTGTCCATGGGGTTCAAATGAAATGGAGTCTATAGAAAAGCTATTTTATGGTACCATGG
CCCTGTCTTTGTGTATTAGTCCTTACATGTAAATATCTACAATATGTAAAGCATATCATTGTTTTCAGATGAATCGTGTT
ATGCTTCAACAATCTGTCCATAATGTCGGTCTGTTCATCTATAATCTCTCCAAGCTTCTTCCCTGAAGGAAACACTCTCT
CTTGTTCTGTGATAGCTACATAATCCATATCTGGCCCTTGCTTACAGAAATGGTCCATGCTGCAGCCCTGCCTGGTAATA
AATGTCCCAGTGTCTGTGGGTTGCTGGTTGGGTGCGTAATGTCATTAACTTCCCTCTCCTCTTTGCACGGCCTAGAGGCC
TGGGAGCCAGAGAAACAGACACCCTCCATTCTGTAAACTCCTTCATGACCTTCCTCTCCATTTTCTACAGATTTGCAGCC
TAAGTCGGTGGAAAGCGTCTTCATCTTCCAGGAGGGGACGGAGGGCGTCGACCAGAGTGTGACCACCACGTACGATGCCA
TCGTTGTGGAGCAGTGGACTGTCATTGACGTGAGTGGGATTTTTACATGGCTCGGAGCGTTTCAGTGTTTAATGTGAGAG
TCGTCACATGGAAATATTCCCTCTGACCATGGTGGCTATGGCACATGTGTCACGGTGGCTTGATGGCTTATCTTATCTGC
AGCAAACAAGCCCTTATCTGGCCAGCGATTAAGTCCCCAGTTTGCGCAAATCCAAGTTGGAAATGGATTTCTGTCTACCT
CTGAAGGGTTTGAGGCTTTTCTCTCTGTGTGTGTGTGTGTGTGTGTGTGTGTGTGTGTGTGTGTGTGTGTGTGTGTGTGT
GTGTGTGTGTGTGTGTCACTCTAATCGAGTTTAGAGTTTGAGGCTGTCGAAGTGTGAAAATAGACTTTTGTCCCCTAAAT
GTGACTGGAAAATTGTCCAGTTCAGAGAGGAAGTCGGCTCACAAACACAGAATTTAACCCTTGAAATAAAAATGCTCTTA
ACCCTTTAAAATTACTACAAAAATTCAGACTACACAATATTCGCTGCCTTAAAATGTCATGTTTGTTCAGCGTCCCACAC
CACACAAAAACCTAGGCGCTTGTGTCTTGTGAAGGTTATACACTGTACGAGTATGTAGGCATATTTATGCTAATACTGTG
GTAAAGATACTGCAATAATGTCTTTATTAAAGACCAGCAATTATTTTATCAACTGCTGAGAAATAACATGGATTTTATAT
ACTTTTTTTATGCACCTTTGGAAGTTAATTGAAGGACAAAGCAACAATGTTTAACAACTTTAGATGTTCACATTATAGTT
CCAATGAATCCAATCAAATGTGCTGGCCAGGACTTATCAAAAGCAATTTTGCTAGACTAATCTCTCCTGAAGTTAGCTTG
ATAACACTATTTTACAAAGGATGATCAGACTTCATACCCGGCACATTCATTATGATAACTGCTTAAAATGTATATGCAAA
TTATCCTCACCGCTCATTGTCGTTTCGGTGCCAGAATAATTACTTAAATATTATGCAGAATATTGATTGGGATATTTTTC
CCCAGCTCTCTTGTTCCCATCTTGCTGAAAGACTGACAGGTGGTTGGATTTATGAGCAGCGAAACAGAAAACAGGCTGGT
TCACTGAGAATTCAATCAATATTGTTGTGTTGAAGCAGGAAGTGCAGCTTTTCTCAGAGATTTTGTTTTCGTATGGCACA
GAACATGTCAAAACTTCTGTTTTAATTGATAGCAAACTGCTAAAACGAATCTGACTTTTAACATAAATGAGGAGTTATTA

CAGTATTTCTTTGCGCAGTATTCACAATCACGTTGGTAGAATGTGGTAGGATTGGTACAGTGTAGATATGTCGAGCTGTT
TGTGGCCAAACTGTTTGTGGTCTTTAATCTAGAAACTCAGACCTTCCTCAAATGTCACTAATGTCACATTTCTGTGGGAG
AATGGGTCATTTCGACGGAAATGTATTTGTTACTTTACATTCACATTAGTCAATATGTATTCACCATATAAGAATGTATA
CGTTAGACCATTTAATCAAGAAAATTAAGGCGAAATAATAACTGTAGAATTTTGCGGTCTAATGGATGGACATTCTACTT
TAGCCAATCAGAAACATTCTTCACATTTTCTTCAACAAATGACTATGCTCTGATGAAGGTCGACTGACAAAGCTCGGCTA
CGATGAAAGACATTTGCATCTGACTGGAAGTGTGCAAAGACTTTTTCCTATTTCTACAATAAATGACTAAATGAACTGAA
TAGGACATACAGTAGATATGCATGTATAGGTTATTCATGTGGTGGACTCCATGCTGTATATAGGACTTAGCATGATTAAC
TGTTTAACACTCAATAATGTCATTCTAAATGCTCATGAAGATGCCTTACTACAAACAAAGATCATACCAAATACCTTCAT
CCATTACATTGAACAACAAGGGCATCATGTTTTCCCAGTGTTACCTCAGTTTCTCACAGGCTATATTACATTTAACCCAA
AAGTGCATGGCCATTATCCGGAGAGGCATTATACGTAGAACTCTACCAGTAACAATACTGTGTGCTTGAGGCAACAGTTA
ACAGCATTGACATTGTTGTCGTTCGTTCCTAGCAATTGTATAGATGCCTGAGCTTTGAAGAGCACTAATTCAGTCACCAG
AAACCAAATTATGTCAAAAAAAGATTAAAGACCAACAACAGAGTAAAGATGCCAATAAAGGAGAGTGAATGGAGAGTGCA
TTTTCAGACAGAGGACACTGTGGCTGTGTACGTGGGTGGGTGTTGAAAAACAGGGGTAGGGTTGTAGAGAATCTTAGGAC
CATGAATGTAATAAGAAATCTGAGGTGCTGGCTTAAGGCCGGTAGAAATCACTACAGAATGTCCGGTCAGAAGGATGAGG
CCGATGTCCAGGTTAGGGGGAGTTTAATGGATGTGTGTTATCTGAACTCATGGTTCAGATAACTCATGGTTCAGAACATG
GTTCAGATAACTCATGGTTCAGATAACTCATGGTTCAGATAACTCATGGTTCAGAACATGGTTCAGGTAACTCATGGTTC
AGATAACTCATGGTTCAGATAACTCATGGTTCAGAACATGGTTCAGATAACTGAGAATCATGTCCAGTGAGACCTGACAT
TAGCTACGGTTCTGAAAGGACAGCGGGGAGAAAGATAACTTAGGCTATAGCACTGCTATAATATGAACAACGTGGTGCCC
GTATAGCACTGCAATGTGTGCATGCATATGTCGTATGCGTGTCTTCATATCTGACACGTGCTGTGGTTTGTCAGTGAGGG
CAGATGCAGAAGAGATGGAAAACCACATACGCTTCCTGTGTGAGCAGAGGCGTTAGCGCACGTGTAGCGAGCGTGTGTCT
TTAACCCAAATCCCAAGTGCACTACACTGTCATACCAGCAATCAATCTAACAGAAATGCATGTATTTTGGTATGCATCAT
ACATAGTTCATCATCTGTTGTCAAGAGCTTGGGTAATCGTGGCTCTTTTGATGGGGTTTTAATGGTTTATGTGTTTATTC
ATGCCTACATTTATGCGTGTGTGTGTGTGTGTGTGATCGTACGATGTGACGATAATGGAATTTTGGGTAACGATTACTTGTC
ACGCAAATGGACACGGTTAGTGTCATAATTGTCCTTTTTGTTCAAATGTTTTGAGCATCATAATGCATCGGAATTATAGC
TACGACAAACCTAGTGAATACAACCCAGCTTTGGTAGCACCCCTCTCTCAGAAACAAATAGTTTAGACCTCTTGGAACTT
TTGGAAATGAAGCTTCTCCCAATGGTGCCGTTCTGCTCGTGTAAATGACTACCAACTTTACCCACTTCATGTAAAAATGA
AAAACTGCTATTGGGTAATTTATATCTACTTGAGTATGAAGTTGAATCCCCCTTTCTCTTAAAATGAATATGTATTAAGA
CTATTATGATAATTATTTTTGAAGTTCACAGTTATTGTCCATAATTGTCGGTTATACGATTATACGATAATTGTGCCAGC
CCTACTCGTACGTGTGCTTGTGCGTACGTGTATGCAAACACTATGTGTATAGTAGTATGTAATATCCATGTGTTTGCATT
CTCTCTTTAGGGCTTGCAGGTGAAAACAGACTATGTTCCCCTGCTCCAGTCTCTTGCCATGTACGGCTGGAGACTCACAT
GTGTCCTGCCTACACCCATCATCAAGACCAACAGGTAACTACTTTCACCTTCCAATACACACTCTCACTAGTATTGATTT
AGGGCCTGAATGACCACCATATCTCCTGGTTACCTAACTCTGGTATTGATTGGTCAACCTGAGTAACCCTCTGGAGATTA
AACTGCCTTCTCTTTGATTTCAAAGAGCACCCATCTCATTTAGACAAAGCGATGGTCAGCCCATATCACTGGTCAATTAA
CAGCTCAACTTAAATGGATTGTGAAATCAGTCAGAAGAACTCAACAGGATGTTCAACGAAAATACTAACTGACCCCAATT
GGCGGGTTCATATGTAACAGCGTGTGGTGCGAGACAGTTTAAACCCACAAATGTGTGTAACTGGAAAGTCCCTTCAGAGA
AATGTAACGGTTCTTCTGCTCAGCTTCTGCTGACTCATTCATCTTATATGAGGGCTATGCACGCACACATTCGCTAACAG
TCAACAGCTCAGTCATCTATCAGCAGTCACAACATGACTTCCAAGTCTTCAAAAATACTTGTCTCCCACCTGAAACCCCA
CTTTACACAGTCACAAGCGCTCATCATTATCACAAATCAGAACACGTCCAATCCAATATGTACCCATTGAATTGATTCTT
TAAGAATAAAACGTTTAAATAAATGTACTTGTGAGCTTAGTTCAACTGTCGTACCACATCAGCACCCAAAATATAAGCTT
GTTTTACTCCAATGTTTGTAAACAAAGTAAATGTAAACAAACACTGTATAGCCTAAAAACATGTTTAAAAGTATCATTTT
AATATCGTGGATGGTCAGTCCTTGCATCCATAGAATTTGAGTGGTAACATTTCTCCAGCCCCATCCCCCCCTCCCGCTTT
TTACTGAAACAATGTTGGGGAGAACGCTTTGTTATTGTTTCAACTACTGATTGCTGCTATTAGTCAGATTCATGAAATGT
GTTGTGTTATGCTTACGAAGACTCCAAGTATATGCTTAGAGTTCAACACGGTTGCAGTTAAATGTTTACCATGCGTACCC
TTGTCTCTGGGGGGTCGTGTTTGTGACCGTTCCGGAGCATAGCTGATTAATTACCGCTCTATTTTTATCCCTTGTTTATT
CATCACTCTTTATTTTGATCAACTCTCTCTCAAAATGCAACAGCACAACAGCGCAACATGGCTAATTAATCAGAGGGCCG
GCCTAGATAAAACCACACAGCAACGCTCATTTCTCTCCATCTCTGAAACACTTGTGTCTTTGTGCTCAGCAACGATGGCT
GTTGACCCATTTCAGATTAGCTTAGTCATTACACTTGTATTATGTTGACTCTTCCATTTTCACAACTATATTAAGCTAAT
GCACGTTGGGATACTGTCTGAGGATGAGCAAAGTGTACTTTCTCTGAAGCAGTCTACACAGAGTCTGTTATGCAAAGACC
TACTTACACACTAGTTAGCATCTAAAGTGAACATCCACCGGGACTCAAGGCTTTTTTAATCAACGTAACATGAATGTGAA
GGTGCACATGGTAATGTACAGTATATTTCTGCCACATGGAACATGTTACATTTGGTGTAAATCTGATGTTTGGACATCGT
AACTGTTTTAGTAAATAGGTAAGTGCTTCATAATACCATTATAAGAAATACTTAACATTACCCACAGGTAACCACTAAACA
TGATACACCATGTTCCCTACCACTGTACCAAAGAAAGTCTTGTTTGCTGGTATATGAAGTTTCTATTTTCAGTACTGCAG
ACACAGACAGCTATGAGAGGACTGTAGCAGCAAGACACAGACAGCTATGAGAGGACTGTAGCAGCAAGACACAGACAGCT
ATGAGAGGACTGTAGCAGCAAGACACAGACAGCTATGAGAGGACTATAGCAGCAAGACACAGACGGCTATGAGAGGACTG
TAGCAGTAAGACACAGACAGTTATGAGAGGACTGTAGCAGCAAGACACAGACAGTTATGAGAGGACTGTAGCAGCAAGAC
ACAGACAGCTATGAGAGGACTGTAGCAGCAAGACACAGACAGCTATGAGAGGACTGTAGCAGCAAGACACAGACAGCTAT
GAGAGGACTGTAGCAGCAAGACACAGACAGCTATGAGAGGACTGTAGCAGCAAGACACAGACAGCTATGAGAGGACTGTA
GCAGCAAGACACAGACAGCTATGAGAGGACTGTAGCAGCAAGGGGCCTGTAATTTTCAAACTGATCTGCTCCGTTCTACT
TTAACGGGCTTGGGCTTTTTCTGAGGACTCAGGTTGGCTTTTCTGCTTTCATTCAGCAGGAAGCTGTCCGGCAATCTGAC
AGCCTGGAAAATAGGTTATTGGAAATGCAGGGTAAGGAGGGAGGGATGCGAAATGGGGGTGTTTTAGATTGTCAGCCTCT
ACAGGAGATGTGGAAGCCCATTTGATGTTGTGGGGAAGATTTGCAGCCAGCTCTATACTGTTGTCACCCTATCATACATT
TCCGTTGTTCTTTATATGCCAAGTTAAGGCTCCAGCTTAACTCAAAGCTGATTCTTCCCCGTGTGTATAAGAGTACATCT
ATTTTAATGGATGAGGGATTCCAAGAGAGGAAGTGACAGAGAGAGCGATCTGGAGAGGGAGATCAGGGGATATACGAAAG
AGAGAAGGGAAAGGATGGAGAAAGCCTACTTGAAGCTCATACAAGAGGAAGAGGCAGAGAAGTAAAGTGAAGCAGACAGA
TAATTAGAGACAGCAGGCTAAAGTAGGAAACCGAGTCGATACGTGAGTGTCGGATGGAGAGATGGAGCAGAGTGAAGGTT
AATGCGTGAGAGCTGGAACGACAAGAGACGAACAAGAGAGAGCAAGCATGAGAGACAGGAAGCCCACTGACGACAGAATC
TCCTCGGTGGATTAGTCTGTCATCTGTACTGTATGGATTATCCATGAATTAACACAGAATGTGACGTGTGCATGGACAGA
GATTATTTTGGAGAAGTTTATGAGGACAAACATGTTTTGGGGAAGCCAGTAAACGTGGTCTCGTTCTCTTGGTGTCTGAG
CTCTCTCCTAAGCATAGAACATATTCACGTGGACATACAATATCGCTCCAAATTCCTTTTTGGTTTCTCGTATTAACAAG

AGACGACAGAAAGGGAAGAAAGCAAGGTTGACCACTGCTATGTAACTCACTTCTCACTGTGGTAGATGTCAGCTATTAGT
ATGGCATCAGGACTGACCATACTAGTCACACAAACCATTATAAGGTCTACCCTGGAGTGTGGATCGCTCCCCTTCAAGTG
GAACTGGAGCATTATAATACAAACTGCATTTTCCATTCCTCTCTTCCTTGCTCTGAAATGACCCCAAGTATTTCTAAAAG
AGCTTGTGGCAATGTGATTTGTGTGACATGGATTTCTTTATCGATGGTACAGGCATTTGTCCTCTACAAATAAAGTAACT
ATGAAGAAAATAGAAATGGTTTCCAAAGTTATTTGTATTTCAGTTGTAATTATATTCTCTCTACCTCCCTCTCTCCATAT
TCACCTCTGCCTTTTATCTCTCCGTCTCCCCATCTCTCCCTCTGCCCTAGTGATGGAAGTTTGGCCACCAAGCAGGTCCT
TTTTCTGCAGAGACCTGTCCTTCCCCGCAAGAGGGGGGAATCCAAGGTACGTGACCTCTCATTCCTCCCAGTGTTTGTCT
TGGCACATGTGGGTCGGCTTGCAGCTTGTTCACTAGCCAGAGCAGAGCTCCAAGGCCAACATCACCCCTCACCACCCCGG
CCCATACCACACTTGTTCTTGTTTTCACATAGATAAATATGCACACTGCCGGTCAAATGTGAATGAACCCCTTTCTTCTT
GAGATATGAGCCATTCTGTGGTAACTACGGCAATGCATGTTGAGAAAGTACTTTCCATTTGACCCTAGATAGTACTGCTA
TAACCTCTAGATTTGACCTTTGTCCTTTGTGGAAGGAATCAATGCTGATATTGCCTGTGACCTGAATTTAAACTCTGGTC
ACCATTGGCAACCAAACTGATGGGCAATAACCCTGGCAGAGATGAGCAGAGAACGATAATGTGGATGTTTGTGTGCTGGT
AACATGCTGTTTACCTCAGTATACTGTAAGTCTCAGCTAAAAGAGGGTCAATGGAGGCAATCTCAGAAATATTACCAGCC
ATATTTTCTTGTTTGGTCTGTACCCCTGAAGGCACCAATCAATTCCCTTGAAATTACCATTTCATTCCCCACAATGACAA
TCATTTTAATTTGTTCTTTCCTTGTTTGCTATTATGTCCATTTTCAACGGTCTGTCTGTCCGTCTGTAACATGGCCAATA
GAACAGCAGTTTAGAAATGTTCTTACACAAACATCCTGTCTTTCAGAAACTGATATTCAAAAGCAAGTCTAACAAGAACT
CTGTTAAAGATGCATCAAAAAACAAGAAGAAGAAGAAAAACAAGCAATCGGCAGCAGAGAAAGATCTGGAAGATTCCAAG
ATTCTTGACGACGGGGAAAAGGTGGTCAAAGAGAGCAGGAACGTGCAGGAAAACAAGGGGAACGAAGAGGAGACAGTGAA
AGAGACGGAGGTAGAGACTGTGTGTAAAGAGGCGGAGGCTCCCTGTGAAGTGGACGGAGAGTAAAAGGTTGAGGNNNNNN
NNNNNNNNNNNNNNNNNNNNNNNNNNNNNNNNNNNNNNNNNNNNNNNNNNNNNNNNNNNNNNNNNNNNNNNNNNNNNNNN
NNNNNNNNNNNNNNNNNNNNNNNNNNNNNNNNNNNNNNNNNNNNNNNNNNNNNNNNNNNNNNNNNNNNNNNNNNNNNNNN
NNNNNNNNNNNNNNNNNNNNNNNNNNNNNNNNNNNNNNNNNNNNNNNNNNAACGGAGAATGTTGCCATGCAACAAGACCC
ACCTGACCAGGAAGAACAGGTAGTGCAAAAGGCAGTAAAAGATAATGGACAGGTCAAATTGACCAATCAATGCCCTGAAG
AGAGTCAAAGGTCGGATGTGTAAACCTCTGCAGACATCACACCTAAGGGTTCTCCTACCCATCATTCATGTACCTACCAA
ACATAACCTGAGATAGGGGCTCCACAAGTCACCATTGAACAGCCTATATTGCTGCAACATCCTCCTACCATGTAATCACT
CTATCCTCTGCAGCAGACATCTCTCCACAATGGACACATCTCTACCACAGATGGACACGCAACAGTCTCTCTCACAGCCT
CAGACTGATCCTATACCTACACATAGTGAATGTACATAACTGAGAATGATGCGGAGGAGACTAATGTACGAGGGTCACTG
GATATAATGGACAAGTTGATGGGACCAATGGGGCTTGGGTCTGAAGAAACAGACTTTAACCAGGCAAACTCGCTTGTCCT
TATTTGTACAGTACTATAGTGGTACAGTTACTGTATGCCACATGGGTTTAGATGAGGAGCACTGTGGGAAATGTAGTGGC
TGTTGTTGCTACACGCAATCCAATGCAAGGTGAATGGAACTTGATTTACTTTTAGCCCGTAATGTAAAGTACTACAGATG
ATGCTGATAATCACTCTGATTCTTATAGTAGGTATGATACATTCGCCTTGCATTCCGTATGTGTCCATATCCAAGCCAAG
TAAAGTGTTTGATAATGGAGTAAATCTCCATTGAATGAATGTGTGTGGGTTGAGTGGAAGTGAAGTGTGATGAGAACCAA
ACTTGGAAATGTTGAGTCAACTTTGAGAGTTTACACACGTTTGATTTTAATAGCTTTTCCAATCAATGGTCATTAAAAAG
TACTTGGCAAGGTTATGGGCTGGGCCATGACTGGATGTTCAAGATAGAAACGGCGCACAGAGGAAGTAGAAAAACAAAAA
TTATTGAGCATTTTGGAATAGCTGTGTTCGAATTGCCATTTTCCATTGACATTGGCTTTAATATTTCACCTCAAGCTTTT
AAAGCCCCTCTTGACGGTGTCATTTCTGACAATACCAAAATCATAATGTAGTCTTTCAAGGTATCATGTTGCTTTCCATT
TTAAAGTGGTGCTGCGTCGATGCTGTGTGGAGAAATCATTAACCGGCTAATGGACTTTGTCAGCGGGGCGTCAGTCAAGT
CATACATTTATAATGGGGGTCACTTATGTGGCTATTGACAGTGGAAAATCTCACCTCTCAAAATAACAAATAGCACTTCA
TCTGGATTGTGATTTTCATAGGGATTGATTCTTCTTGTGCTGCAGTTGGTAAGCAAAGTCTGTAGAAACCTGTATGTTTA
CAGATGAATATAGGCTTGTCGTCATTTTCTAAATGTGGTATTTTAATGAATAACCGACTGTTGGTAATGTTCATCTCAAT
GGCAGATGTATTGTATAGAATTCCAAACTGTGACCAGGATAGCCTTATTATGATACTGGGCCTAAATCGTCAAGTACAAA
TGTCAGAAGTTTGACAAATATCAAGGTGGTTTCAGTGTTATAGCCTGAGAGGGTTGCTACAATCAGTGTTTGAAGGTAAT
ATGAAGGTAAGTTCAGTGTTCTCTGTTTTAGACTTGAACAGAAGGTAAGGGATTTGGCTTTACAAGGTGCTTTTGATTTG
AAGTTAGGCTCAGTGTATTCTGTCGTGCTAACGCTAGCTGAAGTGTGCTTTCTAACGCTCATACTGGAGCTAGCTACCTC
CTTAGGCATATTGGGGATTTGTAGCTATGGGGTCATCATTGCCAACTTTTTCCACCATATGGTCCATGCTGTGGGCGTGA
GGTCATTACTAATGGCCAAGTGGTCAGGGGTCCTGTTTGAACCATGTTTGATGGATTTTCCACAGCTGCTACTTGTTCGA
GTACAAAGGTGTTTGCCCACATGTCAAATAGCAGATTTGATATGAAGTCAGGATATTTTTTATTTTGTGATATTATCTGT
GGTATTGTTTTTTTCCAGCGACACTTCGCGTTAGGCGATTATGAGCTTTAATAAGCACTTGTTTTATTCAACATATTTTA
TTTAGCTTTGCGGGTCACCGCTAGAGAGAGAATAGAAATGACTTGTCCGTTTTAATATTTGTGAGACTAGGACAAATATG
AATAAGAAATGGAATTCTAAAACACCCCTCTGTGATAAATGAAGAGATTATCTGGAACCTGAGACAGATTTCCCCATTGG
CTTCTTATCAAGTCATTTTTTTTTCTCTTTAGATTTAAACTAATCGCTTTCTGCCCAGATATAAGATCATGTAGGACTGTC
ATATTTCCTTATTCATACATTGTTTATTATGTATTCATCAGTTCTGTTGTTCATACCAATTTGAGTCTTTTTTTTAATAT
ATATCTGTGATCCTTGTTCCTTTGTATATTATTTTACACTGAGATAATACTGCCTCTTCAAGTACAATGTACATTAAGTT
GTGGTAGGAAAGTGTGGGAAAATACAGCCAGGAACTATCAGGTGATCTCATGCATTGTATTTTAACACCTACAGCATTCC
GGTAGGACACCATTAACATGGACAACTGTAACCTCCGCGGAGTTGGGTCATATGACACCAGGTCCTGTATCCGTTTGTAT
TACCTGAGGGTCGTGAACTCCATGCCCTCATCTCTCGTGATAAGAGATGATTTGTACATTCGCTCAGCTTTGACATAAGT
AGACTCTGGGGAGAGTGGGTAGACTACATTTTCCCTGATACTGAAAAGCAAAGGATATAGAGAGAAAAGTGAAGCGGGAT
CTGGCACTTCATCAGTTAAATTATGCTGAATGTTGTTTACTTACCCTTCCGTGACGAGGGAGACCTGTGACTAACGTAGA
CCAACCTCAGCCTTGTCTAGAACAAAAGCCTCCAACCCACTCGTGAAAAAAAGGTAATCTACTGTTTCCTGTCTTTATGT
AACCATAATTGTCTAAAGCTCAGCAGTCAGTGGCAGTGCAATTGTTCTTGATAATGCTGTGCAAAATGTGTGTTCCAGGG
TTCATTTCCTGTGTGTTATGATGTGAAATGGACAGATTAGAAATGACAGAAACATGATACAGTAGGATGGCAATCTCTTT
ATGTTGTCACTTTAGCTTGCTCGTGGCCTCTGGAATTGTATTGTTAGGTTTCATCACCGATGTTACTGTTTATCACCTGC
GCTGCCATTTTGAATGTTGAGTGTCTTTGATCATCGGCTACCGTCACGCACTGCAGTGTTTGTAAAAACAAAGGTATTCT
TTGTGTTCACTTTTCATGGAAATGTCCACGTCCCTACTGTTCATGACTGTACAATTTGTATATTATAAAATTATTTATAT
TTAATGTGCACCTGTGGTGTTTTCTTTTTAGATGAATTTTGAAAACCAATGCATTTTGCCATGTTTGGGGTCATTCCTAA
ACTCAAATCATTACCCAACACTACACATTTCACTCATCAGTGGTCTTTCAATTTTCAAGTCTCTTGTAAAGATTTATGTG
CAACCTTCAATGTCTTAGATTCCGTAAACACAAAAATGTTCCATTAGATAGTAGAATCACATGGATGGTTGAACATGACA
TTGGCCAGTGAGAAAAACAAGTTGAGACATGTTTTGTTCAGGTTCAGCTCTAAGGGCTCAATTCAGACTCAGAAAATCTA
TTCCTTGTTTGATTTATTAAGCACTTCTCTGTAGTTGGTATTCATACTTCCCGTATGCAGGTGTGTAACAGGCTTTGCAG

GTGTGGCTACCTTGCACGCACTGAATAAATGTAATTCAACTGCTGAAAACCCTCTCACTTGCTGTCCAACAGATTTTCTC
ATGGAGTTTTCATTCAAAAGGGTTTTCAGTACATTTATCTAAAACCCATTAAATACGGTGTACCTTTTAATTCAAATTTT
CTCAACAGACACTGAGTTTGCAAAACATTGGGAACACCTTCCTAATACTGAGTTGCACCTCCTTTTACCCTCAGAACAGC
TTCAATTTGTCAGGGCATGGACTTTACAAGGTGTCAAGCGTTCCACAGGGATGCTGGCCCATGACTCCAATTCTTCCCAC
AGTTGTGTCAAGTTGGCTGGATGTCCTTTGGGTGGTGGACCATTCTTGATACACACGGGAAACATTTGTGCGTGAAAAAC
CCAGCAGCGTTGCAGTTCTTGACACACTCAAACCGGTGCGCCTGGCACCTACTACCATACCGCGTTCAAAGGCACTTAAA
TCTTTTTGGTCTTGCCCATTCAGCCTCTGAATGGCACACATACACAATCCATGTCTCGATTGTCTCAAGGCTTAAAAATC
CTTCTTTAACCTGTCTCCTCCCCTTCATCAACACTGATTTTAGTGGATTTAACAAATTCTATCAGTAAGGGATCATAGCT
TTCACCTGGTCAGTCTGTCATGGAAAGAGCAGGTGTTCCTAATGTTTTGTACACTCGGTGTATATTGAGAGAATTGGTTC
AGAATGGGAATTAATGAAAGAACCATCTTAATACACACATATTTATCTCCTTTCCGCCACCAGTTGGTCGATTTTAAAAT
ACTCTGATCTTGTATATTATTTAGGGTCATTTATATTCACAATCCCCTTATCCAAACGGCTTGCTCATTTACCTAGCTCT
TATCAATGTGTAAATTACAGTGCATGGAGAACGGTGTTATTTCTATCGGGGAAAAGAAAGTAGATGTTGTTCCACTTGG
AACCGACAGGTTGTTGGACCTTATTCATCGTTTCATCATGTGTAAAGGTGGCGTAATTATGAGGGAAATAATTCAAGGTC
AGAATTCGGCATATCTGTAGACACACCTCCACCACACCTTCATTTCTTACATCTTCACCCAAAACGAATAGCGGTTGAAT
AACATGCTATTCTCAAGTTCAAGGTCAGAATACCCGTAGAGCTAAAGGTGCATAAAAAGGGAATTACATTTTATTTACAC
ATGCTTAACTCGGGTCGGAATCGGCCCCCAAGTGAAGGGCTCTTGTAGGTTGGCCTTGGAGTGCTGAAAGAATGTAAAGA
GGTAAATGCAGAAAACATTAGTCCCTCACAGTCTACAGTATAAGGACTCCATTGTTGGATCAGGCTGGACCTGAGTGCTG
TAGCCATGCGCTTACGGACAATGTCATTTTCTTAGGCGGCACAAGGGGTAAAAAAACAGAACTATTGTTTAAAATCTGTC
ATATCTGCTTATTACATTTTCATTACAGTTGATCAAATGTATAGGCATTATGCTGCTGTATTGAAATACATTACGTGGGC
CCCATGTGTTCACAAATCATGGTAATGGAAAGCAAGCGTATTTCCGTAGAGTTTCACGGTCAGGGGTGATCTATACATCA
TGAGAGCTAGGAGCCCTGGGGAGAACAGGGTCATTATAATGTGGGCTATGAAACAACAACCACATTATTTCCATGTTGTC
CACTACAGGAAACGTTAATAGATCTTAAATGACCAAGGGGACGAGGTGGACCACTAGAGTGTCAAAAATCCTCATTTCCA
GCCTGCAGTTATAGTATTGAACAAATGTACTTTCGGCCTAAAGCGCTGTGGCCCATGGGGTTTACCACTAAGACCTGAAC
TGATGTTGGGTTTGGGATAAAACTGCTGAATGTGGCATACTGAATGTCTGGAGAATTTTGGTGATCTTGCATTAAGATGG
GAATTTATCAGACTAAATCTTTATATTTGTAAAATGATACGCCAAGGTTAATTTAAGGAATATCATTCTGTGCACAGAGG
GCCATAAAGGTTAATTGATTTACTGCAAACTGTAAGAGGATACCCAAACTGTCTACGCACCAGATCACTTCATCATTTC
TGTTCCTCTACATTGTGTATACTGTTATTTTTACTGGTTTTCATCTAGTTGGTTCTGTCATACCACTCAAACTCAACTCT
GGACCTCGCAGCCAGCACCATTGCTTTTTTTTCTCCATTGTTCCCCTCTAACTAGGAACTGATTTAGACCTAGGACACCA
GGTGGATGCAATTAATTATCCAGTAGAACAGAAAGCCAGCAGGCGCTGGACCTCATAGGGTAAGAGTTGAATACCGCTGT
GTCATACCATCCATCCACTGAAACATGTCAATTCACCTCTAAGATAACACTGCCATCTAGTGGTCATGTCAGTCACCTGC
TCATCTGGTTGGATGTACCTACCTCTGATGCTGCTCATGACTCAGCAAAGAAATGCATATTTTGTCTGCATTAAAACACT
TCATGGTCTTATAAGGACCCTTTCAAAGTATCAACATACATAAACTCAGCAAAAAAAGAAACGTCCTCTCACTGTCAACT
GCGTTTATTTTCAGCAAGCTTATGTGTAAATATTTGTATGAACATAAGATTCAACAACTGAGACACAAACCGAACAAGTT
CCACAGACGTGACTAACAGAATGGAATAATGTGTCTCTGAACAAAGGGGGGGTCAAAATCAAAAGTCAGTATCTGGTGTG
GCCACCAGCTGCATTAAGTACTGCAGTACTCCTTTTTATTTAACTAGGCAAATCAGTTAAGAACAAATTCTTATTTTCAA
TGAAGACCTAGGAACAGTGGGTTAACTGCTTTGGTCAGGGGCAGAACGACAGATTTTTTTTTTACCTTGTCAGCTCAGGG
ATTCGATCTTGCAACCTTTCGTTTACTAGTCCAACGCTCTAACCACTAGGCTACCTGCCGCTCCTCCTTATGGACTGCAC
CAGATTTGCCAGTTCTTACTGTGAGATGTTACCCCACTCTTCCACCAAGGCATCTGCCGGACATTTCTGGGGGGAATGGC
CCAAACCCTCACCCTCCGATCCAACAGGTCCCAGACGTGCTCAATGGGATTGAGATCAGGGCTCTTCGCTGGCCATGGCA
GAACACTGACATTCCTGTCTTGCAAGAAATCACGCACAGAACGAGCAGTATGGCTGGTGGCATTGTCATGCTGGAAGGTC
ATGTCAGGATGAGCCTGCAGGAAGGGTACCACATGAGGGAGGAGGATGTCTTCCCACAGCGTTCAGATTGCCTGCAATGA
CAACAAGCTCAGTCCGATGATGCTGTGACACACCGCCCCAGACCATGACGGACCCTCCACCTCCAAATCGATCCTGCTCC
ATAGTACAGGCCTCAGTGTAACGCTCATTCCTTCGACGATAAACACGAATCCGACCATCACCCCTGGTGAGACAAAACCG
CGACTCGTCAGTGAAGAGCACTTTTTGCCAGTCCTGTCTGGTCCAGCGACGGTGGGTTTGTGCCCATAGGTGACGTTGTT
GCCGGTGATGTCTGGTGAGGACCTGCCTTACAACAGGCCTACAAGCCCTCAGTCCAGCATCTCTCAGCCTATTGTGGACA
GTCTGAGCACTGATGGAGGGATTGTGCGTTCCTGGTGTAACTTGGGCAGTTGTTGTTGCCATCCTGTACCTGTCCCGCAG
GTGTGATGTTCGGATGTACCAATCCTGTGCATGTGTTGTTACACGTAATGTCTGCCACTGCGAGGATGATCAGCTGTCCG
CCCTGTAGCGCTGTCTTAGGCGTCTCACAGTACGGACATTGCAATTTATTGCCCTGGCCACATCTGCAGTCCTCATGCCT
CCTTGCAGCATACCTAAGGCACGTTCACGCAGATGAGCAGGGACCCTGGGCATCTTTCTTTTGGTGTTTTTCAGAGTCAG
TAGAAAGGCATCTTTAGTGTCCTAAGTTTTCATAACTGTGACCTTAATTGCCTACCGTCTGTAAGCTGTTAGTGTCTTGA
CGACCGTTCCACAGGTTCATTAATTGTTTGTGGTTCATTGAACAAGCATGGGGAACTGTTTAAACCCTTTACAATGAAGA
TCTGTGAAGTTATTTGGATTTTTACAAATGATCTTTGAAGACAGGGTCCTGAAAAAGGGATGTTTATTTTGCTGAGTTTA
TATAGATGACCTATGTCATCACTCTTTACTATCACTCCAATCTAACCCACACTAAATGAAAGTATTGCGTACCAAGAGGC
AATAAGTTTGAACTTAACTTGAGTTCACCAGAAGTTCACTCATTAGCATGAAAGAGAAACTCCAGAGCAGTGAATTATGG
CTCATTATCAGTATGCCTCATGTTTGAGTAGCTGGTGATAACCATTGCTGCGCTGCTTGATAAAGTCACACGGTCCTTTT
TCTCCACTTACACTGCGTTTGCAGATTACCCACTCACACATTAAACACAATTACCTCTGTGTACGCCAATCACCGCCTTA
TCGGACGCCGTCAGGCCTTTAAACTGTAAATTAATTAATAACCCTTGTCATTTCTTAATTAGCTCCCTTTCCAGGAGGAC
AAATGCGTGTGTCAGCACCTCCATTTTGTGCACACTTGTTATGACTTTGCTTTGAGTTTATTGGTTCATTAGAGACGCTC
TATGAAAACATGCTGTTCTCATGCTGATGAGGATGTCAGTGCGTATTGTGGAGTGATCTAGAGGGGTTCTTCAGCTCTGG
TGAGTGGGTTATATTCAGTCAGAGCTAATGTCATTTGTTTCAGCCTCACTGAACAAAGGAGCCATAAAGAGACAGTTGAA
TGATTTATTAGGTTTTAACCAGTGGTAGATTCAGATATTGGGTGTAGATTGGATTGAAGGGACCATGGGTCAGAGCCTGT
GTGTTGACAGGTCTGGGCTGGTTGGTTGGTTGAGATTGGTCCAGAAGAGGTATCTCCCTCAGATGATTACTGTGAGGATA
AGACAACAAAAAACACCAGAGGGTTAGAAAATGTGTGATATACTGTAGTGTTCCATAGCTGCCATGATATCAATATCATA
CAGAAGACCAACTCATTCAGCCCCTCCATGATAAATGATCCGGTATGACAACCATCTTATCTCACCTGCTGGAGTTTGAC
GCAACAGTGGAAGCGCACGTCATTGAGAGATGTGTCATCCAGGCCGTACTGGTATTCCTCCATCTTGGTCTCGATGCCAC
AGATTCCTCCATCACCACAATCCTGGCTCCAGAGGCTGTACTCGCCATAGTCCAGGCCGGCGCCTGTGAGGGTGGGGTTG
CTGCTGCAGCGGAACCTGATGTTGTTGGCGGCTGTGTCGTCACCGAACAGACTCTGGTGGGGCTCCACACGCAGCTGGAA
GGAAGTCAGCACACCCCTGGGGCAATAGTGAGGGTCCGACCAGTCACCAAAGCTGGGAGAGGAAGAGAGAGAGGCATACA
GAGGGAAGACGTGACATGGAATAATATTTCACGATCACAGCAGTCTGGAATGAGGTTTGACCATAGCTTTGAGACAGAGA

AAAGAGGCACATTATGTAGAGTGTAGAAATGGGACATGGAATAACATCTCACTGCAGTTTGAAGATGCAGGGTTTAAGTC
AATTTGTGTCTACCTTGAGCAATGAAATAGGACTGTTTTGTGTGCTGTTTGTTCTATACAAGACCAAGGTAATGTTACTT
AAGCATCACTCGGTAGCATGCATACAGTGCATAGTAAGTAATGGCTAACTTGGCTCGCTAGTACTATATAGTATCATACT
CAGATACAAATAACAGCAGCGCCATCTATTGGCTTGGCGACATCTCGTAACAAGGACGATAAGAAGCATGTTGTTAAGGA
TGACATCAGGATGCATTACAGCTTTGATTTTGTTCAACAGGTAAGCAACATTTACACCTTTATACTGATTAAGGACATAG
ATGGATAGATATACACAACAAAAAAACGATATATATGGGAGGCAGGCAAATACCTTTCACTGAGATGTGTAAAGACAGAT
TGACAGGGTTGAGACAATTAATTGTAATTTATCTTAATGTTATTTTGTTGTTTGCAGGTGCACTATCCTGACTCTATGCA
GCCAGGTCCCATCTACTTTTTAACTCCTCGCAAGTGTGGCTTGTTTGGTGTCTGCTGTGAAGGAATATCACAGGTCAACT
ACTTGATTGATGAAGGCTTGTCATCCAGCAAAGGCAGCAGCGCAGTCATCAACTACATGCACCGTTTCTTTACCAACTAT
CGAGTTGGGAAAACACATGTGGACCTGAATTGTGATAACTGCAGTGGCCAAAACAAGAACAAGTTTGTGCTCTGGTATTG
TACCAGGCAGACCATGCACAAGCTCCACCACAGTCTGGACCCAGACTGGTGCTTTGGCCTCATCAAGCAGCGCTTCAAAA
AGACCAGAATGAACACTGAGATTGCTGCTGTTGTGAAGGACAGCACTGTGACAGGGGTCAACATCCCAAACTGGTTGGAC
TGGAGGATGGTACGGTGCTGGTGGAAAGCTATGGCTGGCAACAACACCTGACTCAGATCAAGCAGTACCGGCACTTCAGG
TGAATATCATTTTCATTGCATTGTATGAGGTTATTCTTATCTAAACTTGGGAGGTGAATTGATGTTGTGCAAGGTTGTAA
TGTATTTTTAGTTATTTCTTGTTTTACAGCTTTGATGCTCTGGAGCCTGGTGTTGTCGCCAAGGAGAGTTCAGACTCAGT
CGGGACCAGGTTTCAGCTGCTGCGCAACGCTGACATCCTTCCTCCCATAGATGGTCTGACTGTACAAGCAGCACCTGGAC
TGGACACAGCTAGACAAACTTATATTTTTGAGAAGATCAGGGTGTTTTGCGACAAAGAGGCTATGGACATCACATGCCCT
GCACCAAAGTCAAGGACAGGACAGAAACAGACTCTCCGAATATAGATTCCCTTGTTCATGCGTGGTTCGGATGGTCCAGT
CATCAGCACTATCTGCAGTTCCTCTATTCAAATGACTCTCTCCTAACTACGTTGCTGCTACTATTTTTTATTATCCTGCT
GCTCAGCCACTTTACCCCTGATTGTATGCATATACTACCTCGTCTATCACTTTCGTTATTGATATGGTTTGTGTACATAC
TGTATATTTTATTGATATTGACACTATCCATTTATGCTATTGCAAGTAACCATTTGGCTGTACCTTTTACACCTTCTGTA
GAGACCCATCCACTTAGTAAAATATTGATATATATCATGTCGTAACATGATTTTGTGACATACAACAATATCATGTGGCC
GTATCATGTGTCCACCACACAAATATATGGAGCATGCATCTGTTTATGTACTGTACATGTGCAGCTCACTCAGGTTGCAT
GGCCTTAACTTATGTATGGAATACTATGTGATAAGTGCGTGTGTAACAGTATATAAAGTATGCTAATGTACTGGTGTTAA
GGCATTTTGGCAGTGGTGTAAAGTTCTTTAAAGTATTTTTTGGGGGTATCTGTACATTACTTATCATTTATATTTTTGG
AAACTTTTACTTCACTACATTCCTAAAGAAAATAATGTACTTTTTACTCCATACATTTTTCCTGATACCCAAAAGTACTC
GTTACATTTTTACAGGAAAATTGTCCAATTCACACACTTATCAAGAGAACATCCCTAGTCATCCCTACTGCATTTGATCT
GGCAGACTCACTAAACATAAATGCTTCGTTTGTAAATTGTCTGAGTGTGCCTCTGGCTATCTGTCAAAAATAAATTACAA
ATTGGGCCATCTGGTTTAATATAAGGAATTTGAAATTATTTATTATTTTTACTTTTGATACTTAAGTACATTTTAGCATTT
AATTTACTTGACACTTAAGTATTTTTAAAACTAAATACTTTTGCTCAAGTAATATTTTACTGGATGACTTACCTTAATAG
AAAATGACTGAAGTAAAAGTATGTTTACATTTACTCAAGTATGACAATTTTGTACTTTTATCACCACTGGATGTGGCTGG
GGGTTATAGAATTTATTTCACATGACCCATCAATTTAGACAAGTGTGATCTAACACTTATAAAATATTTTTATCTAGACA
CTTTGTTTACCTGGAATTTTTCCTTATTGTAGGCTACTACTTTTGGTCTTTACTACACTACACACTGTTTAGCACATGAC
CTCACATGTGAATCCTTAAAGAGATGGGTGGGGCTGATTTAAAGGGGTGTGAACAATGCTGAATGGGTGTAGACAAAGGA
GAGCTCTCCAGTAGCACCAAAACATTTATAGGCCCTTTTCTCAAAAGTGAGTTTATCAACTATCAAAGCAGAATTCCTTT
CCCATTGTTCCTCAAAAATGTAGTGTATGATATACCATTTTGTAGCTCTGAGTCTCTACTTTTTACTCATGTAAAAAAAA
ACATTTCAAATGTTGCTACATAAGACAGAATCTAGGTGTTGAGTCACATTTCATCACTATAGTTTGAAATGCGCTTTTAC
AGTTAAACCTGGGAAATATGATTATTTCCAATTGTGTCTACATTGAGCAATGTGATAAGCCGATATGAGGCATGTTGTTC
TAGTCTGTACATACAATCCAGTGTGAGACTCAATGGTGTACAAGAAGCTCCTATCTTCGTCCTTAGCGCAGATCAAGCGA
ATGCCATTCAGGGCAGTATCATCCATACCGTACTGTTCGGACTCCACCTGAGAGAAGGTTAATGGACATATGCTAGCATT
AGCTCAAACAGAAGAATGGTCTCGTAATACACAAATGTGTTGATCATGTTTGGTAACGGATCAACACCACAAGTACATTT
TGATTACATCAAATTTAGGATTTGAATTGTGTAGAAAATAATTCAAATAAATGGGCTATTTGAAAATAGGCTTTTTGGCC
ACAGGGGACTGGAATTTAATCCCTGAATTGACTAATTGAAAGAAATGCTTTCCAAACTGGTAGATGTTTGAAATGTATGA
AGGAGCCCTTGCTTACCCGGAGCATGAACCCAACGGCGAAGAACTTACCCGGAGCATGAACCCAACGGCAAAGAACTTAC
CCGGAGCATGAACCCAACGGCAAAGAACTTACCCGGAGCATGAACCCAACGGCAAAGAACTTGTCGGGACACATCTCAGG
CCAGGTCCAGTTCCCAAACTGCTCTCCATTGGGCACGGTGAGGAGGGAGGTGTAAGGCCTAGCGCTGTATTGGATACCAG
CACGCTCCACATAGGGCTGCTCCTCGGGCAATATAGCAAGGCATGACCCAAGGGCCAGCACAGCCAGGAAGGACAGAGCA
GTGAACAGAGGGGTAGTCATGTTGACAGAAGTCAGTCTCATCCACAGAAGGGATGGAACAAAGGCAGTGAACTCTTATAG
CCTTCCTCACTCACGCCTCCAAAATGGTGTCTCCATACTGTTATCAGTATTAATCATTTACAACACTGTGGTCTGGCCAT
GGGAAGAGGCAGGTGGTACTAGGACATAGAGTTAAAAATAACAATACTGTCAGTGACTTCAAAAGCAGCTGGTATAGTCT
CAATGAACTGTCAATAATTTGTCCTACCAAAGTATGACCCATGTTGTATATTAGTAATTTGACTGTTTACATGCTATGAC
GTAAAGAAAACGTTTAATTGTCAGAAACCAGGTCCAAAGCTGATTTTTTTTTTGGCGCGACAGAGTGAACCAAAAAAATAA
GGAACATATGTTAATAGTGGTCTTTATTATCAAAATAGATCAGTTAATTTACAATGATTTCAAAATGTCAATTTTCTTAC
ACCTCATATAAAGAGCCTTTCTGTTATCGCTGTGCATGTGCAGTCCCAAGGCAACTGAAGTGAATTGTTTTCATTTGACA
GGACTTGCATAATGAGTAGCTTTTTCTCCNNNNNNNNNNNNNNNNNNNNNNNNNNNNNNNNNNNNNNNNNNNNNNNNNNN
NNNNNNNNNNNNNNNNNNNNNNNNNNNNNNNNNNNNNNNNNNNNNNNNNNNATAGCCTCCAACACTCTACTCAGCAAATTGG
ATGCAGTCTATCACAATGCCATCCGTTTTGTCACTAAAGCCCCATATACTACCATCACTGCAACCTGCATGCTCTCGTTG
GCTGGCCCTCACTTCATATTCGTTGCCAAACCCACTGGCTACAGGTCATCTATAAGTCCTTGCTAGGTAAAGCCCCGCCT
TATCGCAGCTCACTGGTCACCATAGCAGCACCCACCCGTAGAACACGCTCCAGAAGGTATATTTCACTGGTCACCCCCAA
AGCCAATTCCTCCTTTGGCCGCCTTTCCTTTCCGGTTCTCTGCTGCCAATGACTGGAACGAAGTGCAAAAATCACTGAAG
CTGTAAATTGTAATTACTTCGCCACTACGGCCTATTTATTGCCTTACCTCCCTAATCTTACCTCATTTGCACACACTGTA
TTTTTTTGCAAAATATGTCAAACACAATATAGAAAAATGCCTTAGCCTTATCTATTCACACGCCTAAAGCCTGACCTGAA
ACAGTAAAATATGTCTACATTATGAAACTTCTTGAAATGGAAAATACAATACATACATAAAATCTCTGTCCATTGTGTGA
TAATGTTGTGACTTACCTGTTCTTCCACCAGTAGTAAACTCCTCCTCCAATAGCCATGGCTACACCAATGAAAAGGGAAC
ACACCCCATATTGCCATTTTAAACCATTTAGCATCCAGGCCTGAGGTATCTGGGAATGGAATTATGTAAAACAAGACATC
ACACTCTACCACAAGGCTGCTACCAATCCAGTAACTTCAAATCTATTGACATTTTCATGAAAACACTACTATAAGCAGAA
TGTTCCTCCCTCTTTCTGTACCCCTCTCCCCTACATTTCCATTCCAACTGTCCTCCCCTCCCTCCAGACCACAACACTCT
CTCTCTCTTCCCCAGGTCTCTCACCCCAGACCACCTCCAGTGGTTCATCTAGGCTGGGGTGCTCAACCATGCAGCTTACA
CTCTCCCCTCTCTGAGGCCTGAACTCTAGGTAGGAGTGGAGCTGGTAGCTCCAGTCCCCGTTAGCCAGTGTGTCAGTGGA

GGTCACGGCTGTGGTGGTCTCCCGCCCGTCTCTCAGCCAGCTCACCCTCACCTCCTGAGGGTAGAAGCCTAGAACGCTGC
ACTCCAGCATGGACAGCTCCCCGTAGCGAGAGGGCTTGGTCAGGCGGACTTTGACAATGGGAGGCACTGGGACATAGGAT
GACGTAGAAATGAAGTTGTCTGTCTGATGAAAGTTCCCGGCCTGAAACATCAACATTTTTAATAATGTTGCAAATTACTA
AGAAAGGAAAGAAACATAAAAATGACTTACCTATTCTTTCCAAAGTGCTCAGTTTGAAGAATATTGCACTGTATCGGCAA
ATAGTCTCCACTTCTGCTTTCCTTATAGCCATTTTCCAAGCTTGGCTGTTGTAGCGATCTGCATTGCGTATGCCAAACTC
CCCAAAACCCACATACTTCTGAACCCTGGAGTCATAGATGGTGGTCAATTTTTGGTTGAATATGTTTTTCACAAAGTAGA
TCATATTGTCAATGGTGTCATCATATTCACAGTCAACTATCCCCTGGAACTGGTACCCACCTGAAAAAAAAAATATATAT
AATTTGGACCGTTGTATCCCTTTCCATGTAGTTATGCAATGAGGTACACTCAAAAAATATGTTGTTGCTTATTGATTCAG
GTAAATCTCCTTATCATTGCCTTTGTGGAGTCGTTATACTGTATTGTTACAAAAGATCTGTCAACTTAATTTGATACATT
CTTCATAATATAGAGTATGTTAGACACATCATGCATTTCATCAAAAAATAGACTGACTCACCTGCTGGTGTTGCCCAGGT
CTGAATGACAGAAAGTCCAGCCATCCAATAGACCCAGCAGAGAGCCATGGTGTATCCAGTCACTCAGATAGAGACAAATA
ACTACACTGAAGTCTACCAGTTGAAGCAGCTATCATTGACAGGTACATCCCAATGTGTGACGAACAAAATTAAGCTCCAC
CTTGGTATCCAATAAGGTCCCCCCTTGCTATACTGCTGTAAACTGTTTCTGATATTGAGTGAAAATGGTTGTTTTCCCCT
CACCACTCAATTTTTTTTATATGTTTTGTACGGTAAGTGATAATCTGTGGAATGAGTTGAAGTTAATATCAGCCCTCACA
TGGACAGAATGAGATATTTCTGGTTCGGGTGGTGTGTTAGCAAAATATACATTTCCTCATATGGTTGCCCTGAGTCACTG
CATTATCTTTGTGCAACAGTAACCAACCACTTCCTTTTATACAAAAGTATAATAGCCAGGGCTAACCATGTGGCAGGTTT
ACAAACTGAGAAGTACACCTAAGGGAAGAAATCACCCACAAAGAATATAGTAACAAGTATTTCTTATTTTCATGTAATAC
AGAAATGTGACATTGAATAAATGTGTAGTGCCTACATTGCCAAAGCAAAAAAGAATGTCCCAATCTGAAAAACAATTAAG
GAGTATTTTATTAAGATTATTGATCAAATGTAGGAAATAGTGAACTGCATTATATTGGTCGCCAGCGTAGTTGTAGGTTG
CTAAATATGATAATTGTCCTTGGTATTGTTGCATGGAGCCCTTGCTGTATCGCTGATAGCTGATTGTTGCCTATGTTCTT
AAGGCCCAACCAGACCTCTTGGCTTTTTTGATAAACCAGACTCCTGTTGCTACTCCAACCACTCCTAGAGTCACACCTCC
AACACACACTGCTGTCTCCACAGTCTGGTGGTCCGTGTGCACTTCAACCTCTGTGGACATATAGTGAAGAAACAATTCAT
TTGTTAGTGTCCCTAATACAGTATCAATAACATCCCACCTTTCTCTGCGTCGTACCCTACAGTATAAGGTCACACCAGCA
GTGACTCACCCCAGATCCTGGTGAGGGGTTCCTGCAGGCTGACGTGACCCACACTACAGGAGTAGATGTCCCCCTCCTGA
GGTGTGATGCTCAGGTAGGAGAAGATCCTGAAGCTGAAGTCTTCATTGGAGTAGTACTGGGTCTGAATGATGGTCCTCTC
TGCCACCGGCTGCTCGTTCCTGGTCCAAGTCACATCCACGGGTGTTGGGTGGAAATCACTGACCCGGCAGATGAGTGTGT
TGGGGACCCCCAGCTCCACCTCTCTCTGGGAGTATATGTGGGAGGTGGGCACCTCTGTTGGGGGGGGGGATTCTAATTTG
GGCACTCCTTCATCCTCTCACCTCGGTGACCCGCAAACCGATGAGTGGTAGAGTGATCGAGGAGAGTGTCAATTCGTTAT
TATGCTGCATGAATTTCATCAAATAACCTGCACCCTTTTTTGTCCATTGTTTTGTGCGCTCCCGAGTGCGGATCAAATCA
AAGTTTATTTGTCAGTGTGCCGAATACAACAGGTGTAGACCTTAAAGTTAAATGCTTACTTAAAAATTGCACTGTTGGTT
AGGGCTTGTAAGTAAAAAAAAATTTTTTTTAAAATAGGTATTAGGTAAACAATAGATAAGAAAAAAAAAAAACAAAAATG
ACTGAAAATAACAGTAGCGAGGCTATATACAGGTGGTACCAGTACAGAGTCAATGTGCAGGGGCACCGGTTAGTCGGGCT
AATTGAGGTAATATGTACATGTAGGGATAGTGTTCACACCAGTCCAAACGAACTGAACTAAGGGGCTAAATGCACCAGAG
TTCGGAAAAAACGCACCAAACCAATTTGTTATATAAGACACCTTAGTCCACTGCGCCACCAGGATGGAGCTAGCATTTTA
AGGCATACAAAGCTGTTAATTTTAGTCTACTCAAACAGACCCCATTTCAAAGAAAACAAGTACTCATTAAGATCAGGTGT
GGTCAATTAATGGGCATGGCCATAACACCTGAACACACTTAACAAGATAGAGGATAGAGTGTTTTATTGACGCTGAGAAC
AGAATGTATATGTTTCTAAATAACATTATTAGAACGTTAAACATTTTCTTGTGGTTTTTAATGGAATGTCTTTTTAACAT
TCTCTGTACAATGAGAACGTGGCAAAATAGAACCATGAGGAAAAGTTATACTGAAGTACTGAAATTCCCACACAGGAATG
TTGTTTCTTAACCTTCTCTGAACTATTTGAGAACATTCCCAATATCAAACCACTTTGTCAAGTTCTTTAAATGTGCTGAG
AATGTTCCAAAGCCAAGCAACTATCCTGCACCATTCCCAGAAAGTTGTGGGAAGGTTGTATGCAAAATAACCATAGGACA
ACCACACTCTCACCGAGCTCTAAGAAACATTCTCAGAAATGGTTCTCAGATAATGTGTTAGCTGGGGTGTGACTTTGCAG
AAACAGCACTCACCTATTGCCTCTGGCGGGCTCTTTTCTCCCACAATGCAGCGGGGGATGTTGTACTGGCAAGTTCCGAG
GGACACGTACACTTCGTGAGGCAGTCCTGGGTCAGGGGTCCACTGCTGGGCGAACTCTGACAGACGCCGCTCTACCTGGT
AGGTAATGGGGTCAACGTAGAAGAGCTCATCTCCATCAAACTCCACGTCATACTCTTCGTCGCTGGGGACGTTTTTTTGG
CAGAAGGTCAAAAACCGCAGCAAATGTTTGCCTGTGTTCAATAACAGAAGTTGAGATGGAAAATGGCACATCTGACTGTC
TAGTACCATGCTTTGCCAAAAGGTACAAGAGATGTTGAACGCAATATAATTAATTTGGTTAGGCTTTTCTGTAAAATGTT
GTTTTAATAACAATGGGAACTGATATAAAGCAGCTGTCTGCATCTATGGGTTTACGGAATAGAATAACGGAACGCAGTAT
GGGTTAGCACTGCTAAAGGGATTCTTCCAAGCAAAATAATTCCCAGTCTGGTTCCTTTCTCTCTCTCATTCCTCTCTCTC
TCTCATATGAAAAGAGTAAAAGTGGACTTACTTTGGCAGTGTGACAGTAGACAAACACCCAGTATAAAAGCCAGGAACAC
TCTGCACCCCATCCTAAGTGCACTTGTCCAGTGAGCCTTATCAGTAATGTTCCGTTTCTTGTGTTGTACTCCAAACAGTG
CTGCTGAAGGTGACTTCCTCAATATCATCAGCCAATCATATCTTTTATCTGGATGACACTGACGTTATTACTAGAGGTCG
ACCGATTAATCGGAATGGTCCATTAATTAGGGCCGATTTCAAGTTTTCATAACAATCGGAAATCGGTATTTTTGGACACC
TTTATTTAACTAGGCAAGTCAGTTAAGAACACATTCTTATTTTCAATGTCGGCCTAGGAACGGTGGGTTAACTGCCTTGT
TCAGGGGCAGAATGACAGATTTTTACCTTGTCAGCTCGGGGATTCAATCTTGCAACCTTACGGTTAACTAGTCCAACGCT
CTAACCACCTGCTTTACATTACACTCCACGAGGAGCCTGCCTGTTACGCAAATGCAGTAAAAAGCCAAGGTAAGTTGCTA
GCTAGCATTAAACTTATCTTATAAAAAACAATCAATCAATCATAATCACTAGTTAACTACACATGGTTGATGATATTACT
AGTTTATCAGCCTGTCCTGCGTTGCATATAATCGCTTAGGTTCACGTTGGTCCAACGTGTACCTAACCATAAACATCAAT
GCCTCTCTTAAAATCAATACACAAGTATATATTTTTAAACCTGCATATTTAGTTAATATTGCCTGCTAACATTAATTTCT
TTTAACTAGGGAAAATGTGTCACTTCTCTTGCAACAGAGTCAGGGTATATGCAGAAGTTTGGGCCGCCTGGCTCGTTGCG
AACTGTGAAGACTATTTCTTCCTAACAAAGACAGCCGACTTTGCCAAACGGGGGATGATTTAACAAAAGCGCATTTGCGA
AAAAAGCACAATCGTTGCACGACTGTATCTAACCATAAACATCAATGCCTTTCTTAAAATCAATACACAGAAGTATATAT
TTTTAAACCTGCATATTTAGCTAAAAGAAATCCAGGTTAGCAGGCAATATTAACCAGGTGAAATTGTGTCAAACAAGGGC
ACTGCGTTCATCCACCACTGGCCTGCTGGCCCCCCTACCTCTGAGGAAGCACAGTTCCCGCTCAGCCCAGTCAAAACTGT
TCGCTGCTCTGGCACCCCAATGGTGGAACAAGCTCCCTCACGACGCCAGGACAGCGGAGTCAATCACCACCTTCCGGAGA
CACCTGAAACCCCACCTCTTTAAGGAATACCTAGGATAGGATAAAGTAATCCTTCTAACCCCCCCTCCCCCTTAAAATAT
TTAGATGCACTATTGTAAAGTGGTTGTTCCACTGGATATCATAAGGTGAATGCACCATTTTGTAAGTCGCTCTGGATAAG
AGCGTCTGCTAAATGACTTAAATGTAAATGTAAATGTGTCACTTCTCTTGCGTTCATTGCATGCAGAGTCAGGGTATATA
TGCAACAGTTTGGGCCGCCTGACTTGTTGCGAACTAATTTGCCAGAATTTTACGTAATTATGACATAACATTGAAGGTTG
TGCAATGTAACAGGAATATTTAGACTTAGGGATGCCACCCATTAGATAAAATATAGGACGGTTCCGTATTTCACTAAAAG

GAAAATGTCACGTCCTGACCATAGTAAGTTGTTATTTTCTATGGTAGAGTAGGTCAGAGCGTGACAGGGGGTGTTTGTCT
GTTTTTGTATTTCTATGTTCAGTTTCTAGTTTTGTATTTCTATGTTGGTGTTGTTTGGGTTGATCTCCAATTGGAGGCAG
CTGATCTTTGTTACCTCTAATTGGAGGTCATATTTATGTTGATGTTTGTCCCACCTGTGTTTGTGGGTGATTATCTTTTG
AGTAGTGTGTTTTCCTGTCTGCGTCACGGTTTGTTGTTTTTGTGTTTCAAGTATTTATGTATTGCATTTAGTTTCACGGA
ATAAATAAATATGTAGAACTACGAACACGCTGCATTTTGGTCCAATCCTTCAGACAGCCGTGACAGAAAAACGTTTTGTT
ATCGAGATAATTGTTTCTGGATTCGACCATATTAATGACCTAAGGCTCGTATTTCTGTGTGTTTATTATATTATAATTAA
GTCTATGATTTGATAGAGCAGTCTGACTGAGCGGTGGTAGGCACCAGCAGGCTCGTAAGCATTCATTCAAACAGCACTTT
CGTGCGTTTTGCCAGCAGCTCTTCGCTGTGCTTCAAGCATTGCGCTGTTTATGACTTCAAGCCTATCAACTCCCAAGATT
AGGCTGGTGTAACCGATGTGAAATGGCTAGCTAGTTAGCGGGGTGCGCGCTAATAGCGTTTCAAACATCACTCGCTCTGA
GACTTGGAGTAGTTGTTCGCCTTGCTCTGCATGGGTAACGCTGCTTCGAGGGTGGCTGTTGTCGATGTGTTGCTGGTTCG
AGCCCAGGTAGGAACGAGGAGAGGGACAGAAGCTATACTGTTACACTGGCAATACTAAAGTGCCTATAAGAACATCCAAT
AGTCAAAGGTATATGAAATACAAATCGTATAGAGAGAAATAGTCCTATAATTCCTATAATAACTACAACCTAAAACTTCT
TACCTGGGAATATTGAAGACTCATGTTAAAAAGAACCACCAGCTTTCATATGTTCTCATGTTCTGAGCAAGGAACTTACA
CGTTAGCTTTTTTACATGGCACATATTGCACTTTTACTTCTCCAACACTTTGTTTTTGCATTATTTAAACCAAATTGAAC
ATGTTTCATTAATTATTTGAGGCTAAATTGATTTTATTGATGTATTATATTAAGTTAAAATAAGTGTTAATTCAGTATTG
TTGTAATTGTCATTATTACAATTTTTTTTTTTTTTTTTTTAAATCGGCCGATTAATCGGTATCGGCTTTTTTTGGTCCTC
CAATAATCGGTATCGGCGTTGAAAAAATCATAATCGGTCGACCTCTAGTTATTACCATATCTATGGTTATTACATAGAAAA
CATAAGGCATTCTGAGCATGCATGGCACACCTTTCACACAACTAGGGTGTATCAAAGAGGTGAAACATGCAGAACTTTGC
AGATAGAAACTCAATAAATAGCGCTGAGACATGACTCCCTATTCTGCATTCTACACAATACATTTCTATCTGAATGATCA
GCATCGCCCTGACATAGCCCACGTCTGACTGCTGCCACCTACTGTTGAGTCATGATCATTACAGTGTCTGCCCTTTATTG
TTCGTTAAACTGTACATCCAACCGGCCTCACAACCGCAGACCACGGGTAACCACGCCAGCCCAGGACCTCCACATCCGGC
TTCTTCACCTGCGGGATCGTCTTAGACCAGCCACCCGGACATTGATAAAACTGTGGGTTTGCACAACCAAAGTATTTCTG
CAAAAACTGTCAGAAACCTTCTCAGGTAAGCTCGTCTGCGTGCTCGTTGTCCTCACCAGGGTCTTGACCTGACTGCAGTT
TGGCCTCATCACCGACTTCAGTGGGCAAATGTTCACCTTCGATGGCCACTGGCACGCTGGAGAAGTGTCTTCTTCACGGA
TTAATCCCAGTTTCAACTGTACCAGGCAGACAGCATGTATGGTGTTGTGTGGGCGAGCGGTTTGTTGTTGTGAAAAGAGT
GCCCGGTGGTGGCAGTGGGGTTATGGTATGGGCAGTCATAAACTACGGATAACGAACACAATTGCATTTTATCGATAGCA
ATTTGAATGCACAGCACAGGAGGTTGGTGGTACCTTAATTGGGGAGAACGGGCTCGTGGTAATGACTGGAGCGGAATCAG
TGGAACGGTATCGAATACACCAAACACATGGTTTCCAGCTGTTTGATGCCATTTCATTTTTTCAGTTCCGCTAATTATTA
TGAGCTGTCTCCCCTCAGCAGTCCTTCCATTCATCCAACGCCATCACCTCCTGTTTTCAGCACGATAATGCACAGTCCCA
TGTCGCAAGGTTCTGCACATAATTTCTGGAAGCTGGAAATGACCCAGTTCTTCCATGGCCTGCATACTCACCAGATGTCA
CCCATTGAGCATGTTTGGGATGCTCACGATCGACGTGTACAACAGAGTGTTCCAGTTCCCGCCAGTATCCAGAAACTTCG
CACAGCCATTGAAGTGGAGTGGGACTACATTCCACAGGCCACAATCAACAGCCTGAAAACGCGCGGCATGAGGCAAATTG
GTGGACACACCAGATACGGACTTGTTTTCTGACCCATACCAACATATGCATATCTGTATTCCCAGTGAAATCCATAGGCT
AGGGCCTAATTTATTTAAATTGACTGATTTTCAATTGACTGACTCCCTTATGTGAACTGTAACTCAGTAAAATCTTTGAA
ATTGTTGCATTCGGTGTGTATATATATATATATATATATATATATATATATATATATATATATACACACACACACACACAGT
GGATTTGGAAAGTATTCAGACCCCTTGACTTTTTCCCAATTTTGTTAGGTTACAGCCTTATTCTAAAATATATATATTTT
TTATAATCCTCAACAATCTACACACAATACCTCATAATGACAACGCAAAAACAGGTTTATCAATTTTACCAAATTTATAA
AATAAAAAAATACCTTATTTACATAAGTATTCAGACCCTTTGCTATGAGACTCAAAATTGAGCTCAGGTGCATCCTGTTT
CCATTGATCATCCTTGAGATGTTTCTACTCCTTGATTTGGAGTCCACCTGTGGTAAATTCAATTGATTGGACATGATTTA
GAAAAGCACACCTGTCTATATAAGGTCCCACAGTTGACAGTGCATGTCAGAGCAAAAACCAAGCCATGAGGTCGGAGGAA
TTGTCTGTAGAGCTCCGAGATAGGATTGTGTCGAGGCACAGATCTGGGGATTGGTACCAAAACATTTCTGCAGCATTGAA
GGTCTCCAAGAACACAGTGGCCTCCCTCATTCTTAAATGGAAGAAGTTTGGAACCACCATGACTCTTCTTAGCGCTGGCC
GCCCGGCCAACTGAGCATTCAGGGAAGAAGGGCCTTGGTCAGAACCCGATGGTCACTCTGACAGTGCTCTAGAGTTTGTC
TGTGGAGATGGGAGAACCTTCCAGAAGGACAACCATCTCTGCAGCACTCCACCAATCAGGCCTTTATGGTCGAGTGGCCA
GACAGAAGCCACTCCTCAAAAGGCATGACAGCCCGTTTGCCAAAAGGCCCTTAGCGACTCTCAGACCATGAGAAACAGAT
ATACCTTATTTACGTAAGTATTCAGACCCTTTGCTATGAGACTCGAAATTGAGCTCAGGTGCATCCTGTTTCCATTGATC
ATCCTTGATGTTTCTACTCCTTGATTTGGAGTCCACCTGTGGCAAATTCAATTGATTGGACATGATTTAGAAAAGCACAC
CTGTCTATATAAGGTCCCACAGTTGACAGTGCATGTCAGAGCAAAAACCAAGCCATGAGGTCGAAGGAATTGTCCGTAGA
GCTTCGAGACAGGATTGTGTCGAGGCACAGATATGGGGGAGAGTACCAAAAAGAAATATGCAGCATTGAAGGTCCACAAG
AACAGTGCCCTCCATCATTCTTACATAGGAGTTTGGAACTATCAAGGCTCTAACTAGAGCTGGCCGCCCGGCCAAACTGA
ACAATCAAGGGGGAGAATAGCCGTGGTCAAGGATGTGACCAAGATCCCAAAGGTCACTCTGACAGAACTCTAGAGCTCCTC
TGTTGAGATGGGAGAACCTTCCAGAAGTACAACCATCTGTGCAGCACCCCAACAATCAGTCCTTTGTGGTAGAGTGGCCA
GATGGAAGGCACTCCTTAGTAAAAGGTACATGACAGCCTGCTTGGAGTTTGCCAAAAGGCACCTAAAGACTCTGACCATA
AGAAACCAGATTCTCTGGTCTGATGACACTAAGATTGAACTCTTTGGCCTGAATGCCAAGTATCACATCTGGAGGAAACA
GGCACCATCCATACGGTGAAGCATGGTGGTGGCAGCATCATGCTGTGGGGATGTTTTTCAGCGGCACAGAATGGGACACT
AGTTAGGATCAAGGCAAAGATCAAAGTACAGAGTTCCTGGATGAAAACCTGCTCCAGAGCACTCTGGACCTCAGACTGGG
GAAAAGGTTCACCTTCCAACAGGACAACGACCCTAAGCATACAGCCAAGACAACGCAGGACTGGCTTCAGGACAAGTCTC
AATGCCCTTGAGTGGCCCAGCAAGAGCCTGGACTTGAACCCGATCGAACATCTCTGGAGACCTGAAAATAGCTGTGCAGC
AACGCTCCCCATCCAACCTAACAGAACTTGAGAGGATCTGCAAAGAATAGGGAAAAACTCCAAATACAGGTGTGCTAAGC
TTGTAGCGTCATACCCAATAAGACTCGACTGTATTCATTGCCAAAGGTTCTTCTAAGTACTGAGTCTAGGGTCTGAATAC
TTATGTAAATGTGACATTTTAAAATTATAAATTAGCAAATATTTCTAAAAAGCTGTTTTTGCTTTGTCATTATGGGGTAT
TGTGTGTAGATTGATTAAATTGTTTTCCTCATCAATTTTCAAACGATGCTAACGTAACAAAATGTGCAAAAGGTCAAGGG
TCCGAATACTTTCCATAGGCACTGTATAAATAGGGGTGTACTGTGTATACATATTACAAACAAATAGAAAAGGCTTTATA
GAAGGTCTCATTTTAATATATACAGTTTTCAAATGACTGGCATGCTTCTGAGCAGCAATCCAGTCAACCAGGAGGCAAAA
CAATGTTCAGGACATGTTCTGGAATAATAAAGCATGACTAAGTGGAGGATAAATAGAGTATAAAAGAAAAGCAAGACAGG
AAAATAGACTCACTGGTAGAAAGAAACATGATCATTTGACCACTGAAATACAGTACTATACATCTATATACACACACTTT
CCTTAAAGAGATCTAACAGCCTAGCTTCTATACATCTCATCTGGTGTATCTCTTGGTCCAAACTTCAACGTAGGATGTCT
GCTCTTGTATTCACGGTGATTTAACATCTAACCACCGGTTAGCTTACAAAGCGCATCATACTGTGACACTTTCTGTATTT
TGAAAGTTCTATGTTGAAAACTTGATGGCTGACATGCAAAACATTGTGGAACTGTATCAACAGTGGACTAACGAAACAAA

TACCAAATGATTGAGTGGAATCTTCCTTTAAGCACAAACAGAATTGCAGACTGATCAGTTTCTATGACTGCTGTGATTAA
GACTGCAGTGTCACCATCAAGAAACCTACTCCTTTCCTGAGAAATGACAACTGGGCTGTTTCTTAATGGTAAGTGCCATA
CATGTATGACAAATCTCACTGTCAAGAAAAAGAGTTTCAGTAGTTAGATCTCTTAAGAGGAACATATGGAATGGAAATAA
AACTCTCCTGCCTCTGAACTTTATGTCCAGCTGAATGTTGGTCTGTTAGCAGTAAGAACAGATGTAAAATGAAACACAGA
AAGCAACAACAAAAAGTACTGACTCGTGAACTGCAGCTATAAGACTTGACAGAAAAGACATAACTACCAGATCTGAAAAT
CAACCACTAGACAGTGACGGCCTCGTAGTGAGAGAACAGATAAGTGTAAGCATTACAATTCTCACTCCACATAGCTTTCT
GGGAGGGCAAGTGGGGTTTCTGCCATACTGCTTATACCCATTCAGTTCTTATCAATCTATGATTGCACATCAAGAAGTGT
CCAACATGGGGCTGGGCAAATCTCTCCACATCCTTTCACTGCTCCTCAGTCTCCCTCTTTGGGGGATCGCCATAAAATTC
TTCTTTATAAAAGAAACGTAGATAAATTGTACAAGTATTTACACAGGGAGGAGAGGGATAACAAGGCATTTGTGTTGGGG
GTCTTGAGCAGAGTCAGAGAGAGTTGGTCCGATGCGGTTTCTGTCTGAACATTCCGAGAGTGCCACTTCCTTCTCCATAG
CCATTGTAAGTGATAATTGACGCTTCTGCGCTGTTTATTTCTGATAGTTTCAAAAGCTATGAAGATGTCCAGTGAAAGCA
GATATGCAATTTGTTGACACCACAACACAGTAAAGACTTGGATATACATTATCCTGAATGGTCATTTCATAGGGAACTGT
CAGAGGCTCTCTCGTATTGTTACATTACCAACATTTCGAGAATAAAGGCTCTAACATGGCAGCTGTTCTTAAACCTGGCT
GAACTCTCAATATGTTTCTGGTCTTCAGTATCCTTAGTGACCTTCACGCGTCCTCCACCCTGCACACAAATCCCACCATC
ATCATTCATCTTGGGAGTGTTCGTGCTGGGGTGCCTTTCGAGGGAGGGGCACTCTTTTCCCTCCCTTGACTGCCCCAGGA
GACGTCGTCCTGGGGCTGCCGCATGTCCCTGAGCTCGACGGGGGTTCCCTCCCTCTCCCACTGTCTCATCAACCCGAGTC
TGATTCACTGGTGTCAGAGGAGGAGGATGACGAGGAGGAGGAAGAGTCTGAGCTGGAGCTGCTGCCGCTGAGGCGGGAGG
GCTGGGCGTGAGGCTCCACGCCACTGAGCTTCTCCGCTGCAAGAGAGACAAAGGGGGTAATAGTCTTAGCATGTATCTCA
ATGCTACATGCTACATGTGCAACAGCAGTTCTGCGAGCGTGTGGCTCTCACCCTTGGGTTTCTGGGGTTTCTTGCCAGAG
TTGAGCTGTCCGCTGACATCCTGCAACCTCCTCTCCAGCTCACTCTTCTTCTCCAGGGCCAGTTCCTCTCTGGACTTGCC
GGGGCCCCCTTTCTTCACTGTTACACACAGACAGGAAGACCGGTTCAAGGTTATATACTCAGCCCACACATACATATCCT
AACATTCCATAGTGAAAGACTGGTTACATTTGTATGGTATTAGTCAACCAGAGCGGTAGCATGTAGCGTTTTATCACCAC
AGCACCAGCTTGTAGCATCAGTTCTTTGTAGCATAACAAGGCATTAGCAAGTGTCTGCAGTGACAACATGATGCATTCAC
ACCATACTAGCTAACCACAGCTAGCATTAGCTATCACTATTTGTTCCCCCTCACCATAGGGCTTACGGGGCTTCTTCCTC
AGGCACGTCATGACGTAGCGCTCCAGCTCGCGCAGCGTCGACGGCTTGAGCGTCTCAAAGTCGATCTCGATCTCCTCGGG
GTTGGTGTCGCGCAGCGAGGGCTCGCGGGACTGGATGATGTGGACGACGCGGCCCAGCTTTTCGCCAGGCAGCTTGTTGA
TGTCCAGGCTCAGCTGGCGCTTCTCGTCGTACGACATGGGAGACGTCTCCTCCTCCTCCTCGGAGTCATAGTGGGGCAGC
ATGGAGAAGGCAGGCGGGGCGTAGTAGGGCATGGTAGCAGCCTGGGACTTTTTGGTGTTTCTAGGGAGAGGGAAGCTGTT
AGAGGCCATGCAGTAGTACTAGAGAAATTGTATGCAAACTTTTCCAGCATGGAGCCCATTTTTTTCACGCCCAAACTTGT
GGGGAACCAATTATACATTTTTTGAAATCATTTCTCTTGACCCACCCCAAATATAATGACAACCTACATTGACACATTTA
CATCAACAAATCACCTTCAATTCATTACATATTCATCTCTTATCAAAATGAACTTGATATACTGCCCCATACAATAATCT
GTACTCTGCAAACCCACTGCAGTTTCTAGAGAGACACATGCAGGTCAAGTTGGAAATTGGATTGTGATGAAAGAGAGGAA
GTAGAGAATAGTGTAAGAAACTTTAAAAAAGCGAAGCGAGGAAACAGAAAAGGTGGAAGAGGAAGCAATGTGAAAGTCTA
CAAAATGAGACTGAAACAAACAAGATAGAAGAAAGAAGACATGGATGACCGATGGAAAGGATGAGTAAAAGCAAAGAGAA
AGGTCGTGTTTGAGGTCAGTGTGTGCTTACTTGCTGCCCTTCTTCTTGCCGGGGCCTCTCTTGCCCTGTGAGGAGGCGGG
AGCTGAGCTCCTGGATTTACTCTTCGTAGTCCTCACCGGCTTGGCCGTCAGCATCTCCCAGTCCTCACCCCCTACCGCCG
CGCTGGGCCGTCCTCGGCTTCGCTTCTCCGGCTTCTTCTTCTTCTTCTCCTTCTTGTCTTTCTTCTCCCTCTTCTTC
TTGTTCTTGACGATTGGGCCCTGGGAGAGGGCTGCCAGCTGCTCGTGTACGGCTCGGAGCTGTCACCGGGGGGCAGACCA
GAGGTGGAGTGTTCAACTGGACAAACACACTTCACTTATATGGCTAGATCATGAAGGAATACTGTGATATTTTATTTAAC
CTTTATTTAACTAGGCAAGTCAGTTAACCTGTTAGGGCTAGGGGGCAGTATTGACACGGCTGGATAAAAAAAACATACCC
GATTTAATCTGGTTACCACTCCTACCCAGTAACTAGAATATGCATATACTTATTACATATGGATAGAAAACACCCTAAAT
TTTCTAAAACTGTTTGAATGGTGTCTGTGAGTATAACAGAACTCATTTGGCAGGCAAAACCCTGAGACATTTTCTGACAG
GAAGTGGATACCTGATGTGTTGTATTACCTTTAAACCTATCCCATTGAAAAACACAGGGGCTGAGGAATATTTTGGCACT
TCCTATTGCTTCCACTAGATGTCACCAGCCTTTACAAAGTGTTTTGAGTCTTCTGGAGGGAGATCTGACCGAACAAGAGC
CATGGAACGATGATGGCCCATTAGACACCTGGCGCGCGAGTTCATGTTGGGTACCCTCGTTCCAATACGTTATTAAAGAG
TATGCATTCGTCCACCTTGAATATTATTCATGTTCTGGTTAAAAAAGGCCCTAATGATTTATGCTATACAACGTTTGACA
TGTTTGAACGAACGTAAATATATTTTTTTCCCCTCGTTCATGACGAGAAGTCCGGCTGGCTTAGATCATGTGCTAACAAGA
CGGAGATTTTTGGACATAAATGATGAGCTTTTTTGAACAAAACTACATTCGTTATGGACCTGTGATACCTGGAAGTGACA
TCTGATGAAGAGAATCAAAGGTAATGGATTATTTACATAGTATTTTCGATTTTAGATCTCCCCAACATGACGTCTAGTCT
GTATTGCAACGCGTATTTTTCTGGGCGCAGTGCTCAGATTATTGCAAAGTGTGATTTCCCAGTAAGGTTATTTTTAAATC
TGGCAAGTTGATTGCGTTCAAGAGATGTAAATATATAATTCTTTAAATGACAATATAATATTTTACCAATGTTTTCTAAT
TTTAATTATTTAATTTGTGACGCTGACTTGACTGCCGGTTATTGGAGGGAAACGATTTCCTCAACATCAATGCCATAGTA
AAACGCTGTTTTTGGATATAAATATGAACTTGATAGAACTAAAAATGCATGCATTGTCTAACATAATGTCCTAGGAGTGT
CATCTGATGGAGATTGTAAAAGGTTAGTGCATCATTTTAGCTGGTTTTATGGTTTTGGTGACCCTGTCTTTGAATTGACA
AAACATTACACACAACTCTTGTAAATGTACTGTCCTAACATACTCTAAATTTATGCTTTCGCCGTAAAACCTTTTTGAAA
TCGTAAAACGTGGTTAGATTAAGGAGATGTTTATCTTTCAAAGGGTGTAAAATAGTTGTATGTTTGAAAAATTTGAATTT
TGACATTTATTTGGATTCAAATTTGCCGCTCTTGAAATGCACCTGCTGTTGATGGAGTGCACCACGGGTGGCACGCTAGC
GTCCCACCTAGCCCATAGAGGTTAAGAACAAATTCTTATTTACAATGACGGCCTACCATAGAGTGTAACAGTGAGAAAAT
ACAGCAAATAGCAGGCTTGTGAAAATACATGTTTGAACACAGAGAGTACTTGTATACCAAATGAGTGAAAGTATGCATAC
TAGCATGGATTTGTCTGAATATTAGAAAAACCTGTGTTGGAGAGAAATCTAAAACTCTTGATGCAGTCTGTGCTTGTGAT
GTGTAGATAAAAGCTGGCCTGTGTAAGGTATGTGGTGCAGTGTGTTCGGTGTGTGTGGTGCAGTGTGTTCGGTGAACGGG
GGGGGTTGAGTCTACCTGTGTACACACCTGGTCCTGTAACTCGGCCAGGCGGTGTGCTCGCTCCTCCTCGCTGTCCGAGC
TGCTCTCGCTCTCGCTGCTGGGCTCGCTCTCCGACGAGGAGGAGGAGGACGATGACGACGAGGAAGAAGACGAGGAGTCG
CCGCCGCCGTGAGCGTGAGTGCCCATGCCGCCGCCCATGCCACGGGACTTGGGAGGAGCCTGCTGAAGGGGCTCGTCGGG
CATCTTGGCGAAGCGGAACTCAAACACGTCCTGGATGGACAGATGGACAAACGGAGTGAGTGAGCTTGTTTTTTACTTCA
TTGTGGATCATGTCCCTTTTGTTTTGTTACACAATAATAACAGTTATCACAAGAAATAAAGTTGTAGGGACAGAACAGAG
AAATGGAGACCAAAACAATTTATAGATTAGTCATTAAGTAGACTTATCCAGTGACTTGCAGTCATTGCCTTCTGCCAAGG
TAGACTACCACATATCGCAGTCATTGCAAGTAAAAACCCCTGAGCATGCAGTGGACAGAGGTCAAGCAACAAAGGTGAAC
TAAGGGTTCCAGGGTGAATTGTGGTGACAGAACCGAGCTCACCTGCAGCTTGCGGGCCATGCCCACTACGTCATGGTCGG

GGGGGTTGTATTTGTAGCAGTTGGAGAACATGAGTCGGACGTCAGCGGAAAACTGCTGCGAATCCCTGTACTCCCGACTG
TCCATCTTCCTCTGAGAGAAAGATAGGAGAGAGAAAGACTGTTTTAAACTCCAACAGATAAAGGAGAGGACTCCAGATAG
GGATACGCCTCCTTAGTTTCATACTGACAGATATCCACACAAAACAAATCTGAGAAGATAGGTAATAATCCATGAACTGT
GTATTTCAGCCACATTCAGTTCTCATTGAGTCAAGGCAGTGCGACATCTCTATCTTGCAAGCTGACCTTGATGTTGCTGA
GGTCCATGGGGCACTTGATGATGTCGTGGTAGTCGTGCAGGCCCAGGGCCGAGGCGTCCACGGGCTTGTAGAAGGGCCAG
GCGTAGGCTGCGTGTTTCTTGGACAGCAGATCCTTCAGCACGCTGCTACAGTAACGCAGCTGTTGACTCAACTTGGCCCG
CCGTGGCGCCTTGGGCTGCACAGAGTCCGGCAGGTCTTTCTTGGGGGGCTTGATGGGGCGTCCACTGCCACCGCGCCGAA
CTAGGGTCTTCCCGCCTCCGGCCATCATGGGCTGGAGCAGAACGGGGCCTCCTGGGCCCATGGGTCCTCTAACCAGGCTG
GGAGGGGGGTCCATGTCCCCCAGGGAGGTGAGGGAGAGGGTGGAGGGGTCATGGCTCAGGCCTTGCCCGTGGCCTCCTTT
CCCAAGGCCCACCATCCGACCCCCTCCTACGGCCAGGGGCATGGTCATCATGGTGGAGGGAGTGGTGGTGTCCGCCTTCC
GTTTCACTCCCTTCTTCTGCACAGGGAGAGAAAAGAGTTATAGTATGGTATTCTGTAGCCAATATACAGTGTGAAGGCAG
CAGCAACAAGTGTGTAGCATCAATACAATGTACAGTACTATAGCAAGTTCCTATTCAGGTCTTAGCATCTTGAGTGAAGT
GTGGTGAGGTACCTTGGAGGTGGGCAGTGTGGGGGGCAGGCTCAGCAGCTGTGTGGGGGGCAGACTCTTGGGCAGCATGG
TCTGATGAGGGGTGTCTGAGTCACCCGTGTCAGAGGAGGAGGGGGAGTATGCTGACTGGGACACCGCAGGGACCTGGTGA
GCTTTCGATGCTGAAAGATGGGCAGAGAAATCAGGCTCAAGAACACACCCATAGNNNNNNNNNNNNNNNNNNNNNNNNNN
NNNNNNNNNNNNNNNNNNNNNNNNNNNNNNNNNNNNNNNNNNNNNNNNNNNNNNNNNNNNNNNNNNNNNNNNNNNNNNNN
NNNNNNNNNNNNNNNNNNNNNNNNNNNNNNNNNNNNNNNNNNNNNNNNNNNNNNNNNNNNNNNNNNNNNNNNNNNNNNNN
NNNNNNNNNNNNNNNNNNNNNNNNNNNNNNNNNNNNNNNNNNNNNNNNNNNNNNNNNNNNNNNNNNNNNNNNNNNNNNNN
NNNNNNNNNNNNNNNNNNNNNNNNNNNNNNNNNNNNNNNNNNNNNNNNNNNNNNNNNNNNNNNNNNNNNNNNNNNNNNNN
NNNNNNNNNNNNNNNNNNNNNNNNNNNNNNNNNNNNNNNNNNNNNNNNNNNNNNNNNNNNNNNNNNNNNNNNNNNNNNNN
NNNNNNNNNNNNNNNNNNNNNNNNNNNNNNNNNNNNNNNNNNNNNNNNNNNNNNNNNNNNNNNNNNNNNNNNNNNNNNNN
NNNNNNNNNNNNNNNNNNNNNNNNNNNNNNNNNNNNNNNNNNNNNNNNNNNNNNNNNNNNNNNNNNNNNNNNNNNNNNNN
NNNNNNNNNNNNNNNNNNNNNNNNNNNNNNNNNNNNNNNNNNNNNNNNNNNNNNNNNNNNNNNNNNNNNNNNNNNNNNNN
NNNNNNNNNNNNNNNNNNNNNNNNNNNNNNNNNNNNNNNNNNNNNNNNNNNNNNNNNNNNNNNNNNNNNNNNNNNNNNNN
NNNNNNNNNNNNNNNNNNNNNNNNNNNNNNNNNNNNNNNNNNNNNNNNNNNNNNNNNNNNNNNNNNNNNNNNNNNNNNNN
NNNNNNNNNNNNNNNNNNNNNNNNNNNNNNNNNNNNNNNNNNNNNNNNNNNNNNNNNNNNNNNNNNNNNNNNNNNNNNNN
NNNNNNNNNNNNNNNNNNNNNNNNNNNNNNNNNNNNNNNNNNNNNNNNNNNNNNNNNNNNNNNNNNNNNNNNNNNNNNNN
NNNNNNNNNNNNNNNNNNNNNNNNNNNNNNNNNNNNNNNNNNNNNNNNNNNNNNNNNNNNNNNNNNNNNNNNNNNNNNNN
NNNNNNNNNNNNNNNNNNNNNNNNNNNNNNNNNNNNNNNNNNNNNNNNNNNNNNNNNNNNNNNNNNNNNNNNNNNNNNNN
NNNNNNNNNNNNNNNNNNNNNNNNNNNNNNNNNNNNNNNNNNNNNNNNNNNNNNNNNNNNNNNNNNNNNNNNNNNNNNNN
NNNNNNNNNNNNNNNNNNNNNNNNNNNNNNNNNNNNNNNNNNNNNNNNNNNNNNNNNNNNNNNNNNNNNNNNNNNNNNNN
NNNNNNNNNNNNNNNNNNNNNNNNNNNNNNNNNNNNNNNNNNNNNNNNNNNNNNNNNNNNNNNNNNNNNNNNNNNNNNNN
NNNNNNNNNNNNNNNNNNNNNNNNNNNNNNNNNNNNNNNNNNNNNNNNNNNNNNNNNNNNNNNNNNNNNNNNNNNNNNNN
NNNNNNNNNNNNNNNNNNNNNNNNNNNNNNNNNNNNNNNNNNNNNNNNNNNNNNNNNNNNNNNNNNNNNNNNNNNNNNNN
NNNNNNNNNNNNNNNNNNNNNNNNNNNNNNNNNNNNNNNNNNNNNNNNNNNNNNNNNNNNNNNNNNNNNNNNNNNNNNNN
NNNNNNNNNNNNNNNNNNNNNNNNNNNNNNNNNNNNNNNNNNNNNNNNNNNNNNNNNNNNNNNNNNNNNNNNNNNNNNNN
NNNNNNNNNNNNNNNNNNNNNNNNNNNNNNNNNNNNNNNNNNNNNNNNNNNNNNNNNNNNNNNNNNNNNNNNNNNNNNNN
NNNNNNNNNNNNNNNNNNNNNNNNNNNNNNNNNNNNNNNNNNNNNNNNNNNNNNNNNNNNNNNNNNNNNNNNNNNNNNNN
NNNNNNNNNNNNNNNNNNNNNNNNNNNNNNNNNNNNNNNNNNNNNNNNNNNNNNNNNNNNNNNNNNNNNNNNNNNNNNNN
NNNNNNNNNNNNNNNNNNNNNNNNNNNNNNNNNNNNNNNNNNNNNNNNNNNNNNNNNNNNNNNNNNNNNNNNNNNNNNNN
NNNNNNNNNNNNNNNNNNNNNNNNNNNNNNNNNNNNNNNNNNNNNNNNNNNNNNNNNNNNNNNNNNNNNNNNNNNNNNNN
NNNNNNNNNNNNNNNNNNNNNNNNNNNNNNNNNNNNNNNNNNNNNNNNNNNNNNNNNNNNNNNNNNNNNNNNNNNNNNNN
NNNNNNNNNNNNNNNNNNNNNNNNNNNNNNNNNNTTAGGAAGGACCCTTCGCCAACACAGGCCCTTTGCCATCTGCTCCC
TCACCAATGCAATGAGGATTCTATCAATAGGCTGCCACTTTTGGTACAGCCCCGGAAACCCAGATCACTGGATGGGAAAT
CCCTCGGGCAGCATTGAGGGCAGTTAAACCTGCATTCATTGATAGAATAGTGACAACATGCAGGTTCTCCCTCTATTTAC
TTCCATCTTTTGTACTGTGTGGACCAGATTGACTATTTTATCCCAGGTCGCCCAGGGTTCACACAGCACGCACATGCCAT
CCCACTGACACACAAAGGGAAGCAAAGCTCTTGAGCTGATTTTCCATCTCAGGACTTACACACACCCCAACACAGCCTTG
TTAAGTTAAATGTTTGAAGAAAAGAGAAAGATCTGGCAATGCATTAATAATATTATTCCTTAATAACCCGGGAAAAAGAC
TGATAGTATTGTAAAGACCCTGGGTTTATAAGCGCGGATATCGACTCTGCCGCTCAAGCATGCTGTAATGAAACAGGCAG
GGAGTAGGTCTCGAACCCCCGATCTTCTAGCCCAGCGCGCTATCGACTGTGCCACGAAGGGTGCTTTAATATTATTTCGC
GTAATAATTACTTTATCTTCCTTCTACCCCTGTTTGACCCCTAACTTTTGGCTTTGGCAAAAAATATAGCAGATCCGGGT
GGCTATACATTCTGCTGCCATTTTCCATATTTTCCTATCTAGGGTTTCTTTATCAGTGCACCTATCGTTATTATTGTTGC
AGGCAAATCGCTTAAGAACCTATNNNNNNNNNNNNNNNNNNNNNNNNNNNNNNNNNNNNNNNNNNNNNNNNNNNNNNNNN
NNNNNNNNNNNNNNNNNNNNNNNNNNNNNNNNNNNNNNNNNNNTTCTGGCTAGAAGGTAGAGGGTTCGAGGCCCGCTCCC
TGCCTGTGTCGTTCCATTGGTATCAGAAGTGATCGGACCTTGCATCCACGACAGTGCGTATGCTTGGCCAGTGAGCGCGT
TCCTATAAGACGTAGAGTCGCAAGCAAGCGTGAGGACGCGCTCTTTGAAAGGAGGGAGTAGTGTAACGATCTGGGTTTAT
AAGCGCGGATATCGACTGCCGCTTGAGCAAGCTTTTGCACAGTCGATGGCGCGCTGGACTTCGGGCTAGAAGGTCGCGGC
ATGCTCCCTGCCAGTTTCATTACAGTATGTTGACCAGTTTACTGCTCTCTCTGAGTGTTTGTTATACTTAGCCTAATCTC
CCAAGTATTTTCGTTGAATTAACTTTGATTTGTGGGTGTTAACCGGTAATAGGCAATAATCAAATGCATTAGCTAGTTAG
ATTGTGATATGCTTTCATTCTTAACTGTGGCAATCATTTCAGCCGGCAGAGAGGAGAGGCTTCCGCGCTTATTTATAGTC
ATTGCGTGTGGTCAGTGTCGCCGCCCCGCCTTTTTTGAGCGTGTGTAAACACACCGACGCCAAAACAGACCCTCAGGATC
GTCAGTCAAAACCCTCAAACTCCTGTGACTCAAACCCTTGCACAACTATCCAATCAGCTCCTCGTGTTACTAGCTTGTTT
ATGTGAGACCCCATTTGTGGAAACGCTAACTTAACTGGCTAGCTTGGAACCATTGCATCGTTCGTAAACAACTGAATCCA
GCTAGCTACCAAGCTATTTAGCCAGGACGCTAACGTTTGCTGGCGAACATCAGAAAGTAAACAACCGGGTTTGGACAACT
GAATACGCTACAAATAAATTGCACATCTGCGGCCCTAGCTCAGTTTATTTCTCTGTCATTAAGATTTATAGCAAGAAAAT
GGCGGTGGCGCAGTGCCGCTCAATGAAGCGTTTCATCACCACAGCCAATTTTAAACAATCCCCCAGTACTAAGTTATTGA
GTAGGCTGACCCACACAATAAGTCAATATCCAACCTCACTGTATAGTTAACTATCGAAGGGGATGTTAGGAACAAAAATA
TCTCTATATTTACATCTTACTAGCCAACGAAATATTTTCATTGGTTCGTGTCAGAGTTGAGAAAAGACTAGTTAGCTAGC
TGATGCACCGACAGTGACTACTAGCTAACTAGGTTTAGCCATTCAGTCCCGATTCGTGTCAATTTTCAAGCCTGCAAAAT
CAGTTCCGACCCGAGCCTGAAGGTACAATGTTCATAAAGGTACCTGTCAAGGGGCGGGTTAACGGCCGTTTCCATTAGGA
TGGATCCGGATTCTTTTTATGTTCCAAATTTGTCCACTGTTATGTTTCCACCAAATAAACGCCGAACAGTTCTACAATGA
AATGGCGCCGAAAAGCTACCGCCTGCCGCTTTATAAACATATCACGTGGCGTTTTCGTGCAATTCCTGTCGGGGTTTCTC
GTAATACTTGCTTCAGATTGGCTACTCCGCATAGGTGCTAGATTATGTTTGGCTATCCACGCTGTCTGTAATTTAACATG

CCACACCCTGCAAGAAGAACATCCTGTTCCTTTAGCTTCAGACGGCTAGTCTAGCTAGTTAGATAAAAACAACAGATTTT
TTGTTTATATAATCGTCATCATCTTTACGTTTACGAAACCGAATGGTTATTAAGAGCTACTCTGAATTATAAATACAAAA
CAATTATTACCGGTGGTAGGTCTTCCTCATGTCATGACTGGGTACTATTTCCTAATGGTTAGCCCCAAGTAGCTAACGGT
AGCTAGCTAAAATAGCCAGAAAATCACCAGCCTTTTAGGCACACTGGCACCTAATGTATTGTCCCTCCAACCTTCATTCT
CCTTCCGTTCCGCTTTTGGGGTTACTATGGGCAGTTTTACTTGTGGGGTATATCAGCTGTATCCAATGATTTATATATAC
ACACACACATCATTGGCTGTATCAGCCTCAGTTATAGGTTTTATTCAGTCACTTGGTTGGAGCATAACTCCGTTTTTTTT
ATTTGAAGTGGAAGTTATGCAATCTGTCACATCAGTGGGAGTTTGGGGCAGTTCAGCCGTAACATGATGATGCCGACATT
TAAATAAATTGTTGTCAACATCTTGTCATGACATGGCAAATTTTTCAGACAGCTTGATTTTCTCAATTTGTTTCACATCA
TTAAAAAGGGATAGGCACTAGGCCCACTTGATGGGTCATTTATGACAAAACCTTTCGATATTTGCCAGTCATTTCAATGA
CTGTTTCACTAGTAAAGTGGAAAAACTCAGAAGTGAAATTAAAATATTGATCAGTGAACCATCATATTTTTGAATAAAAT
ATATAAGAATGAAAGAGAAGGATGGCTGTTTTGAATTTGGTCAAGTTAGTGTGGGAGAGGTGGGAAAACTATTGTTATCC
ATCAATAATGATGTGGGGCGGCAGGTAGCCTTGTGGTTAGAGCGTTGGACTAGTAACCGAAAGGTTGCAAGATTGAATCC
CTGAGCTGACAAGGTAAAAATCTGTTGTTCTGCCCCTGAACAAGGCATGTAACCTGCTGTTAGTAGGCTGTCATTGAAAA
TAAGAATTTGTTCTTAACTGACTTGCCTAGTAAAATAATAATGATAAGTCAACTGGTAAAGAAACCCTTGATGGGAAACT
ATTGAGAATGGTAGCAGAATGTATTGCCACCCCTATTTGCTATATCTTTTGCCAAAGCCTAAAGTAGTGTGTCAACAGGT
GTAGAAGGAAGCTAAAGTAATTCCACTGCCTAATAATAGTAAAGCACCCTTTGTTGGCTCTAACAGCCACCCACCACAGT
TTTGCTGCCTGCTCTTAGTAAACTGATTGAGAGAATTGTGTTTGACCTAATACTATGCTATTTTTCAGAGAACAAGTTAA
CTACTGACTTTCAGCATGCATATAGAGAAGGGAACTCAACTTGTACTGCACTGTCTCAGATGACAGATGATTTCTTAAAA
TAAATGGATAATAAGATGATAGTTGGAGCTGTATTGTTAGATTTCAGTGCAGCCTTTGATGTTATTGATCATAAATAGTT
ATTGAAGAAACTAATTTGCTTTGGCTTTAAATCACCTGCCAGCACATGGTTGGAGAGTTATTTATTCAATAGAACCCAGA
GAGTATTCTTCAATGGAAGCTTCTCTAGCATCAGATATGCACAGTACGGTGTACCTTAGGGCAGTTGCCTTGGGCCGTTA
CTCTTCTCTATTTAAAAAAATTATTTGCCACTGGTCTTCCACATGTATGTCTGTTATAAAACGATGTTCTGTGTTTTTGA
CACAAAAATCAACTGTACTAGTTGTTCAGACTCTGGTCTAGTCCTATCTTGATTAGTGTTCGGTAATATGGTCAAGTGCA
GCGAAGCCACAGCTGGCTCAAAACAGAGCAGCACGCCAATTCCAGATTGTCTGCATTATCAAATAACATTCAGCTCAGAC
ACCCATACATACCTCACAACATTTACATTTAAGTCATTTAGCAGACGCTCTTATCCAGAGCGACTTACAAATTGGTGCAT
TCACCTTATGATATCCAGTGGAACAACCACTTTAGAATAGTGCATCTAAATCTTTTAAGGGGGGGGGGGGGGTTAGAAGGA
TTACTTTATCCTATCCTAGGTATTCCTTAAAGAGGTGGGGTTTCAGGTGTCTCCGGAAGGTGGTGATTGACTCCGCTGTC
CTGGCGTCGTGAGGGAGCTTGTTCCACCATTGGGGTGCCAGAGCAGCGAACAGTTTTGACTGGGCTGAGCGGGAACTGTG
CTTCCTCAGAGGTAGGGGGGCCAGCAGGCCAGAGGTGGATGAACGCAGTGCCCTTGTTTGGGTGTAGGGCCTGATCAGAG
CCTGAAGGTATGGAGGTGCCGTTCCCTTCACAGCTCCGTAGGCAATCACCATGGTCTTGTAGCGGATGCGAGCTTCAACT
GGAAGCCAGTGGAGAGAGCGAAGGAGCGGGGTGACAAGACATGCCACCAACATGACACCAAATTCACGACAATGCACAGT
GTTATACAGAGCCAGTATTGCATGGAACTCCCATTCTCCAATTACTCAAGCTAACAGCAAAATGACCTTTAACCCTTCAT
GGTTTTGTTATTCACCCAATGTTTGCAGGTCTGATGGACCCACAACATTATTGGGTTTTTAAAACAATACAGCCATAATA
ATTTGCGTAAAAATACTTAATACTTAGATGTTGACATTTCAATTACAAACAATATAGACAGCATACATGGTTAATATTTG
CCATTTACCTCTGCTAGATCACATTTATCAATAAAAGCACTATTTGTTTTTAAAATATATAATCCAGACATGGGTGGAAT
AAATCATGTTTATCTTGAACAAATATAAATGAAACAAGGTTTTCATCACTATTGATGTTTATTGCTTTGAACTGAACAAA
TTGGAAGGACAAATTTTACATACAGTAAGGCCGGTCTACACACCACATGAGGGACAGAGTTTTACTGTTTGTGTGTTGCA
AATGTATTTCTTGCACTTGATGCATGTGTACTGTGTCTTCCTGTCCTTCTTGGGTCCACACACATCACAGCGCTTCTTGT
TGCTACCGGCTGCAATCTAGAATGAGGAAAACACTTAGTTCAGGAATTTTACTAACATCTCACTCACTCACTCACATATA
TAGTTACAGCAGGCCACGGTAGGAGAGTCAGACACACACACACATGCAACAACATCACTCACACTTACTTCCAGTATTGGAG
ATGTTGGTTCTGTGGGTTTGGCGGATGGGGCACCAGCATCCTCCTAAATCCTCCTCATGATGGCTGCAGAAGCTGGGGTC
CTTGGGATATGTTGCCTCTAGATTTGAGGTCTTACCAATGCCTTGCCCAGCACCTTGAGAAAGAGCTGTCTCCTCTGGAG
CTTCCCTCTGTTCCAATCTGGGTTCAACGCCATCCAGATGACAAAACGTGTTGTACGCCAAGATGTTGAAGAATATCACA
AGTGGCCAGCGTAGGGTTCTTATTTTGCAGTTGTAGCCAGTCACCAGCTTGTATAAAATGTCCACCCCTTCTTTTGTGGC
ATTGTAATCCATTATGATTTCTGGTTTTTGATGTTCCTGGCCACAGATTCTCACATCCCTATGCAGCGTACTCATGAGTA
CCACATTTTGGCCTTCATTTGGCATGAAGGACACTAGGGACGTGTTGGCCGTGAACACAAACTTAGGTCTGTTCCATTTA
TTCAACAGCTGAGGTGGGAGCTCTGGCTTGTTTTTTTGTACTGTTCCTACCATCGTCAGCTTCCTCTTGAGGACCTCCTG
CCCCAGCTTGTGCAAAGTAAAAAGGTTATCGCATGTGATGTTGTGGCTACGGAGTCCCTTTGTCACATCCAGGACAACTC
ACATCCCTTGGTTCTTCCCTTGGTTCTTCTCAGGGGCTCCTCCATCTGGCTTCCCCGTATACCCTTGCAAGTTCCACCCA
TATGATGAAGCGGCATCACAAGCAGCCCAGATCTTGATTCCATATTTTGCGGGTTTAGACAGTATGTACTGTCTGAAGGG
CATAAGCTGCTCATCAACAGTAACGTTGGGCCCAGGGTTGTAAAACACGGGAAGGTGGTCCACCCAATACAATAATGTAT
TGTAATAACATTGTGAAGGCACACAGCAAACCTTGTTTCATTTTTACTTGTTTTCACCAACACGCTTTTCCACACTTATC
CTCTGGTCCAGTGGACCTGAACACCACACAATGTAATATAAATGTGTAGAGGGGTGCACAGTGTACACTCAATGAAAATG
TGTTATTTTACATGTTTATCACAGAAAATGAGCCAAGGCCATTGAGTCTCAGGTAGAAAAAATAATGAATTGTATAATTT
ATTTTAGTAAACATTGAAAATGGGTCACACAGACCCAAACACCACACAAGGGTTAAAAAATAGATTAAACAACATGGCAT
GGATCAGCGGGGACTTTAAGGGGACAAGCACATGCAGACAAACATTATTTGTTTTACTTGAGTGAAGGAAAGCATCCTTC
ACTCATGCTGCCAAACATAACCTCATAAAACTGACTATGCTACCGATCCTTGACTTCGGCGATGTCATTTACAAAATAGC
CTCCAACACTTTACTCAGCAAATTGGATGCAGTCTATCACAGTGCCATCCGTTTTGTCACCAAAGCCCAATATACTACCC
ACCACTGCGACCTGTATGCTCTCGTTGGCTGGCCCTCGCTTCATATTTGTCGCCAAACCCACTGGCTCCAGGTCATCTAT
AAGTCTTTGCTAGGTAAATCCCCGCCTTATCTCAGCTCACTGGTCACCATAGCAGCACCCACCCGTAGCATGCGCTCCAG
ATGGTATATTTCACTGGTCACCCCCAAAGCCAATTCCTCCTTTGGCCGCCTTTCCTTCCAGTTCTCTGCTGCCAATGACT
GGAACGAATTGCAAAAATCACTGAAGCTGAGACTCATATCCCTCACTAACTTTAAGCATCAGCTGTCAGAGCAGCTCACA
GAGCATTGCACCTGTACATAGTCCATCTGTAAATAGCCCACCCAACTACCTCATCCCAATGTTATTTATTTTTTTGATCC
TTTGCACCCCAGTATCTCTACTTGCACATTCATCTTCTGCACATCTATCACTCCAATGTTTAATTGCTAAATTGTAATTA
CTTCGCCACTACAGTCTATTTTTTGCCTTACCTACCTAATCTTACCTCATTTGCCCACACTGTATATAGATTTTGTTCTA
TTGTGTTATTGACTGTACGTTTGTTTATTCCATGTGTAACTCTGGTGTTTGTGTCGCACTGCTTTGCTTTATCTTGGCCA
GGTCGCAGTTGTAAATGAGAACTTGTTCTCAACTGGCCTACCTGGTTAAATAAAGGTGAAAAAAATGTAATAATAATAAT
GGCACTTTAATGTTTACATATCTGGCATTACTCCTCTCATATGTATATACCGTACTTTATACCATCTATTGCATCTTGCC
TATGCTGCTCTGTCATTGCTCATCCATATATTTATATGTATATATTCTTATTCCATTCCTTTACTTAGATTTGTGTGTAT

TAGGTAGTTGTTGTGGAATTGTTAGATTACATGTTAGTTATTGCTGCACTGTCGGAACTAGAAGCACAAGCATTTTGCGA
CACTCGCAATAACATCTGCTAACCATGTGTATGTGATTTTTCCCATGACTGGACTTCCTCTCATTAACATATTTCATGGT
GAGAAAACGGTCTAAACAGATGCTTCAGCTTTATAGCCCCTGTGACGTGTGTGGGTTACCCCTTGTCTGTGCTGCTACTG
CAGTATCTGGCTGCTCCAGCAGTGTGTACGTTTGTGGACCTAAAACTATACTATTGAAATTGTTATGAAACTGCCTGTGT
TAAGCATCGTATTGCCTTCATTGGAAATACCCTCTAAACACTAACAGTATTGCTGGTGGTCAATGTATGCGATTCGGCTT
GGCCTTTAGCATCATTATCTAGGCCTGTAGTAAAGGAAAGAATAAGTGAAAGCATGACATTAGGCGTTTCCCACAATAAT
ACAGATCAACTGCAAAACGTACACATCATGCTGTTATTATGTAACCATTCCCCCACATTCAGATACTAAATGAAGAAATT
ATTTTAGAGAGCAGAATGTTTATTGTCATTAACACAGAACAGAAAAGGGCTCCTGTATTGTACTCCCTTGAAGACAATGT
ACACAGAAAACAAAACATTCTTTGGGATCCCTATTTATATATACTGCTCAAAAAAATAAAGGGAACACTTAAACAACACA
TCCTAGATCTGAATGAAAGAAATAATCTTATTAAATACTTTTTTCTTTACATAGTTGAATGTGCTGACAACAAAATCACA
CAAAAATAATCAATGGAAATCCAATTTATCAACCCATGGAGGTCTGGATTTGGAGTCACACTCAAAATTAAAGTGGAAAA
CCACACTACAGGCTGATCCAACTTTGATGTAATGTCCTTAAAACAAGTCAAAATGAGGCTCAGTAGTGTGTGTGGCCTCC
ACGTGCCTGTATGACCTCCCTACAACGCCTGGGCATGCTCCTGATGAGGTGGCGGATGGTCTCCTGAGGGATCTCCTCCC
AGCCCTGGACTAAAGCATCCGCCAACTCCTGGACAGTCTGTGGTGCAACGTGGCGTTGGTGGATGGAGCGAGACATGATG
TCCCAGATGTGCTCAATTGGATTCAGGTCTGGGGAACGGGCAAGCCAGTCCATAGCATCAATGCCTTCCTCTTGCAGGAA
CTGCTGACACACTCCAGCCACATGAGGTCTAGCATTGTCTTGCATTAGGAGGAACCCAGGGCCAACCGCACCAGCATATG
GTCTCACAAGGGGTCTGAGGATCTCATCTCGGTACCTAATGGCAGTCAGGTTACCTCTGGCGAGCACATGGAGGGCTGTG
CGGCCCCCCAAAGAAATGCCACCCCACACCATGACTGACACACCGCCAAACCGGTCATGCTGGAGGATGTTGCAGGCAGC
AGAATGTTCTCCACGGCGTCTCCAGACTCTGTCACGTCTGTCACGTGCTCAGTGTGAACCTGCTTTCATCTGTAAAGAGC
ACAGGGCGCCAGTGGCGAATTTGCCAATCTTGGTGTTCTCTGGCAAATGCCAAACGTCCTGCACGGTGTTGGGCTGTAAG
CACAACCCCCACCTGTGGACGTCGGGCCCTCATACCACCCTCATGGAGTCTGTTTCTGACCGTTTGAGCAGACACATGCA
CATTTGTGGCCTGCTGGAGGTCATTTTGCAGGGCTCTGGCAGTGCTTCTCCTGCTCCTCCTTGCACAAAGGCGGAGGTAG
CGGTCCTGCTGCTGGGTTGTTGCTCTCCTACGGCCTCCTCCACGTCTCCTGATGTACTGGCCTGTCTCCTGGTAGCACCT
CCATGCTCTGGACACTACGCTGACAGACACAGCAAACCTTCTTGCCACAGCTCGCATTGATGTGCCATCCTGGATGAGCT
GCACTACCTGAGCCACTTGTGTGGGTTGTAGACTCCATCTCATGCTACCACTAGAGTGAAAGCACCGCCAGCATTCAAAA
GTGACCAAAACATCAGCCAGGAAGCATAGGAACTGAGAAGTGGTCTGTGGTCCCCACCTGCAGAACCACTCCTTTATTGG
GGGTGTCTTGCTAATTGTCTATAATTTCCACCTGTTGTCTATTCCATTTGCACAACAGCATGTGAAATGTATTGTCAATC
AGTGTTGCTTCCTAAGTGGACAGTTTGATTTGACAGAAATGTGATTGACTTGGAGTTACATTGTGTTGTTTAAGTGTTCC
CTTTATTTTTTTGAGCAGTGTAGTTAGAACACAAATGTGGGAGAACAATTTAACAAAAATAAATGTAAAAATAAATACAA
AATGCTTTTGGGGCATAAAGAATACTTAAGTTACACTATAGTGCAAATAGAAAGTTGAACAAAGCACATATACATGACCA
TCTGAAGTTTGCCAATGAACATCTGAATGATTTAGAGGACAACTGGGTGAAAGTGTTGTGGTCAGATGAGACCAAAATGG
AGCTCTTTAGCATCAACTCAACTCACCGTGTTTGGAGGAGGAGGAATGCTGCCTATGACCCCAAGAACACCATCCCCACC
GTCAAACATGGAGGTGGAAACATTATGCTTTGGGGGTGTTTTTCTGCTAAGAGGACAGGACAACTTCACCGCATCAAAGG
GACGATGGACGGGGCCATGTACCGTCAAATCTTGGGTGAGAACATCCTTCCCTCAGCCAGGGCATTGAAAATGGGTCGTA
GATGGGTATTCCAGCATGACAATGACCCAAAACACACGGCCAAGGCAACAAAGGAGTGGCTCAAGAAGAAGCACATTAAG
GTCCTGGATGGCCTAGCCAGTCTCCAGACCTTAATCCCATAGAAAATCTGTGGAGGGAGCTGAAGGTTTGAGTTGCCAAA
CGTCAGCCTCGAAACCTTAATGACTTGGAGAAGATCTGCAAAGAGGAGTGTGACAAAATCCCTCCTGAGATGTGTGCAAA
CCTGGTGGCCAACTACAAGAAACGTCTGACCTCTGTGATTCCCAACAAGGGTTTTGCCACCAAGTACTAAGTCATGTTTT
GCAGAGGGGTCAAATACTTATTTCCCTCATTAAAATGCAAATTAATTTATAACATTTTTGACGTGTTTTTCTGGATTTTT
TTGTTGTTATTCTGTCTCTCACTGTTCAAATAAACCTACCATTAAAATTATAGACTAATTATTTCTTTGTCAGTGGGCAA
ACGTACAAAATCAGCAGGGGATCAAAAACTTTTTTCCCCTCACTGTACTCTCCTTTTCACGTCACTTTGGACATACAGTA
TGTGTTTATCAGTTAGTATAATCAATTAGTAAGCCAGCAACTGTATAAAAAATATATTTAAGAAAGGTGAAGTACAGGTA
ACTGCCAAAATAAAGAAATCACTTGAGTAAATGAGGGATACATAATATATTGAAAGCAGGTGCTTCCACCCAGGTGTGGT
TCCTGAGTTAATTAAGCAATGAACATCCCATCATGCTTAGGGTCATGTATAAATATGTCCAGCTGCCCATTATTTTGGCT
ACCATGGCTAGAAGAGATCTCAGTGACTTTGAAAGAGGGGTCTCAAAGCAGTATAGGGGGTTTAAATGGTGTGTGTGTCT
CAGTCACCAGATCTCAACCCAATTGAACAATTATGGGGGATTCTGGAGCAGCACCTAAGACAGCATTTTCCACCACCATC
AACAAAACAAGAAATTATTCAATTTCTCGTGAAAGAATGGTGTCAATCCCTCCAATAAAGTTCCAGACACTTGTAGAATC
TATGTCATATCACATTGAAGCTATTCTGGCGGCTAGTGGTGGCCCAAGGCCCTATTAAGACACTTTATGTTGGTATTTAA
TTTATTTAGGCAGTTACCTGTAGATTGCAATGAGATGTGACTCACCTGTCTGAGCTTTGTCAAAGAAAGGATCTCCTGC
TTCAGCACCGACTGGAAGACTAGGCCCAGGATGGAGGTGTGGATGCGACTCATGGTGATGTTGTAGATGAGATCACACAC
ACACACAGCACTGAATGATAGAGATGGGAGAAGGAGAACAAATAAACACGTTACTACAGTTTCATTACATAAATGCCCTA
TGAAAGTGATTAGGACACTTGACCTCAAAATTAAACAGGAGTATCAAACTGAGCATCAATTGGAAGGACTCAAAAGTATC
ATATCCCCAATTATTGATATATGCTGTACTATACCTCATGACTGTAATCAGAGACATGACCGTGAAGGCTTCAGGCTTGT
CTTCATTCATAATCAAGTCAGTCATTCGGCCGGTGTAGTGGGGAATAGCCATCTTACCAAGGGAAGAGATGACAAGACTC
AGCATGGCTCCAAAGTGTATGATATAAGGCTTCATGTATCCCATCAGTTTCTCTAGAGAGGCCCCAGTCTTCTTCTGTGG
TTCCTTGGTGGGCTTGGTAAGAAGAGAAGAGACGTAGCGTGTCCAGTACAACCATGCTACGACTTACGCAGTAGCCCTGA
AGTAGCTGAAAAACAAGTGATTTTAGTCTGTTTGGTTTATCAATTTTTAAAACAAAAAGGGTATATATAAATACACACAC
ACACACACACACACACACACATAGCAATATAAACTTGTCACCCACCACCTGCCAGGAGTGCCATCCCCATAGCTGCCCCA
GGATGGACTGGCCACATGCCCAGAGAAGGCTGATGTAGATTGGGCAGTGAAAGCAGAGGACACCCATGCTCTGGAGCCCC
TCGAAGCTGCTCATCCATAGGGGACTTCCAGGGAACGTGAAGGTGAGAAAGAGGTGGAGGCCGGCATGGAGCAGGCTTCC
ACCCCACAAGGTGATGAGGGGATGGGGGGATGNNNNNNNNNNNNNNNNNNNNNNNNNNNNNNNNNNNNNNNNNNNNNNNN
NNNNNNNNNNNNNNNNNNNNNNNNNNNNNNNNNNNNNNNNNNNNNNNNNNNNNNNNNNNNNNNNNNNNNNNNNNNNNNNN
NNNNNNNNNNNNNNNNNNNNNNNNNNNNNNNNNNNNNNNNNNNNNNNNNNNNNNNNNNNNNNNNNNNNNNNNNNNNNNNN
NNNNNNNNNNNNNNNNNNNNNNNNNNNNNNNNNNNNNNNNNNNNNNNNNNNNNNNNNNNNNNNNNNNNNNNNNNNNNNNN
NNNNNNNNNNNNNNNNNNNNNNNNNNNNNNNNNNNNNNNNNNNNNNNNNNNNNNNNNNNNNNNNNNNNNNNNNNNNNNNN
NNNNNNNNNNNNNNNNNNNNNNNNNNNNNNNNNNNNNNNNNNNNNNNNNNNNNNNNNNNNNNNNNNNNNNNNNNNNNNNN
NNNNNNNNNNNNNNNNNNNNNNNNNNNNNNNNNNNNNNNNNNNNNNNNNNNNNNNNNNNNNNNNNNNNNNNNNNNNNNNN
NNNNNNNNNNNNNNNNNNNNNNNNNNNNNNNNNNNNNNNNNNNNNNNNNNNNNNNNNNNNNNNNNNNNNNNNNNNNNNNN
NNNNNNNNNNNNNNNNNNNNNNNNNNNNNNNNNNNNNNNNNNNNNNNNNNNNNNNNNNNNNNNNNNNNNNNNNNNNNNNN

NNNNNNNNNNNNNNNNNNNNNNNNNNNNNNNNNNNNNNNNNNNNNNNNNNNNNNNNNNNNNNNNNNNNNNNNNNNNNNNN
NNNNNNNNNNNNNNNNNNNNNNNNNNNNNNNNNNNNNNNNNNNNNNNNNNNNNNNNNNNNNNNNNNNNNNNNNNNNNNNN
NNNNNNNNNNNNNNNNNNNNNNNNNNNNNNNNNNNNNNNNNNNNNNNNNNNNNNNNNNNNNNNNNNNNNNNNNNNNNNNN
NNNNNNNNNNNNNNNNNNNNNNNNNNNNNNNNNNNNNNNNNNNNNNNNNNNNNNNNNNNNNNNNNNNNNNNNNNNNNNNN
NNNNNNNNNNNNNNNNNNNNNNNNNNNNNNNNNNNNNNNNNNNNNNNNNNNNNNNNNNNNNNNNNNNNNNNNNNNNNNNN
NNNNNNNNNNNNNNNNNNNNNNNNNNNNNNNNNNNNNNNNNNNNNNNNNNNNNNNNNNNNNNNNNNNNNNNNNNNNNNNN
NNNNNNNNNNNNNNNNNNNNNNNNNNNNNNNNNNNNNNNNNNNNNNNNNNNNNNNNNNNNNNNNNNNNNNNNNNNNNNNN
NNNNNNNNNNNNNNNNNNNNNNNNNNNNNNNNNNNNNNNNNNNNNNNNNNNNNNNNNNNNNNNNNNNNNNNNNNNNNNNN
NNNNNNNNNNNNNNNNNNNNNNNNNNNNNNNNNNNNNNNNNNNNNNNNNNNNNNNNNNNNNNNNNNNNNNNNNNNNNNNN
NNNNNNNNNNNNNNNNNNNNNNNNNNNNNNNNNNNNNNNNNNNNNNNNNNNNNNNNNNNNNNNNNNNNNNNNNNNNNNNN
NNNNNNNNNNNNNNNNNNNNNNNNNNNNNNNNNNNNNNNNNNNNNNNNNNNNNNNNNNNNNNNNNNNNNNNNNNNNNNNN
NNNNNNNNNNNNNNNNNNNNNNNNNNNNNNNNNNNNNNNNNNNNNNNNNNNNNNNNNNNNNNNNNNNNNNNNNNNNNNNN
NNNNNNNNNNNNNNNNNNNNNNNNNNNNNNNNNNNNNNNNNNNNNNNNNNNNNNNNNNNNNNNNNNNNNNNNNNNNNNNN
NNNNNNNNNNNNNNNNNNNNNNNNNNNNNNNNNNNNNNNNNNNNNNNNNNNNNNNNNNNNNNNNNNNNNNNNNNNNNNNN
NNNNNNNNNNAGTACTGATTTAGGATCGGTTTAGCCTTTTTACCGTAATTAAAATGATTGCATGAACGGGGGGGGGAGCT
CCTATTCTGAGACGCTTTATGAATACAGGGCCTGGTTCATTTGAAAGATTACATCCTGAGACATTGAAATCAGTAGAAAA
ACTGACTGCAGACTCACACCAAGACCACAAACACACACACACACACACACACACACACACACACACACACACACACACAC
ACACACACACACACACACACACACACACACACACACACACACACACACACACACACACATCCCGCTCCTATTCTTTTCCA
CACAGTGGCAGGAAGTTGTCCACATATCCGCAGTGCTTCTGAAGAAGAATTAAAGAGAGGGAGTTGGGTGGGTGGTAGCA
GCACTGGAGGTTGGGGTGTACACTTTAGACATAAACTGTTCACACGGTCCAATTGCGAACGCGGTCTTCTCCAGAAGATA
CCCAGAGAGATACCTCACGACACTTTGGCGGAGTACAGACCTGCCGGACCTGCCATAGCTGGACCAAGGCATTGGACCTA
GTAACTGCACTGGTACAGCTCGGAGGATAGTGAGAGAGACTAGACTACTGCCGTCTCCATCCTCCTCTCTGGGACCTGCT
TGGCCCAATGATGCTGGTGCTGTGGACAGCTGTTCTGTTGGGTTTGAGCAGGTTGTGTCAAGGTGAGTCCATCTTCCAGC
TATTATCTTGTTGATATGATGTTAGATACCTATGAGAGTGAGGTGGGGTCTCTACTAAGTAATCGTCTAAAGAGAGGAAG
TCCTACATGGTACGTCCCAGCAACGAAACATACTTTTTTTTTTACATTGATGTTCTCACTTCTACGTTTTATAGATTTCT
GAGGATATATCATTAGGAGAAAATCCTGTGACGTACTTGACATATACTGCTCTCACATTCTTTCATCGTCACTTTAAAAG
ATGCTGCGTGAATGTGTCCCTGATTTAAAGATGTAACTAACCAAAAAGATTGTACTGCATTTCCTTGAATGTTCACTGCT
ATACAGTACACCATGTCTAATCCCAACTCTCCTGTGTTATAATTTAATTGTCCCTCACACACCTGCCAAGCCTCTCCATT
GTCCCACTCCATTACATTCCTCATCGCCAGGGTGACGACCTCCGACCTTCATCCAGTCCCAGCCCACTCCATTTCCTGTT
GCTATTCGCCTGTCCTGGAGACTTCCTGTTTGTGTCAGGCCTCCTATTTCCCGTTTCATGGTTCATGCCAAGGACACACC
TTTCATAGCCTGCCTGGGCTGCACGGTACCAGGCNNNNNNNNNNNNNNNNNNNNNNNNNNNNNNNNNNNNNNNNNNNNNN
NNNNNNNNNNNNNNNNNNNNNNNNNNNNNNNNNNNNNNNNNNNNNNNNNNNNNNTAGGCCTACACAATACACAGCTTGAA
AATGTAGGTCAAAGCACAGGTTCTTGCGTGATACAGTAATACAGTAGTGTAATTAATGGATGCTTTTATCCAAGGGATTC
AGTTTAAGTTAGTATATTAGTGTCAAGTGGAATCCAACCTTTGTGTGTGTGTGTGTGTGTGTGTGTGTGTGTGTGTGTG
TGTGTGTGTGTGTGTGTGTGTGTGTGTGTGTGATTCCTCTCCAAGTAGCAGCAGCATCTCCCGGTGGTGACCTCTGGCC
AAGTGTCCATCCGGACAGTGTAAGGCCAAGTTTGGAGATGGGTCATGTGATAAAGAGTGCACCGAGCCCGAGTGTCTGAG
AGACGGGTTCGACTGTCTGAGAGACAAAGGACACTGCAAGTAAGACACTGTAGCAGCAGGGAGATCTGGGCTGCATTTCA
TTCCAGGAAGATGTTCCATCCCCTCTTCCCTTTGTTCACTCTCCTTCATGGATCAGACAGGACTGGATATGTGAAAGCAA
TATGGTGACTTATGGTGACCCAGTAGTCTCATATTCATGGTGAGGGGAGTGAATAAGAGCAGAGGGGAGAGAACATCTTC
CTGGAATGAAACGCACCCTGCTCTTTACTCTTTATCACTCTCACTTTCCTTTTTGTACACATCCAGATGTTTCAGCTGGG
GTTCACGCTTTCTCTGCATCCCTCCAACTATCCCCCCTCTCAGTTCAGGCCACATCCACTACTGCCGAGACCACTATGCC
AACTCTTACTGTGACCAGGGCTGTGAAAGCGCCGCCTGTGGCTGGGACGGGAGTGACTGTCACAGGCACCACAGCCCTCT
GTGGGCTAAGGGCACCCTGCTCCTGCACACCCACGTCCCCCTGCAACACGGCACATTTTCCAACAGTTCCCTTCTCTGGG
CCCTCAGCACCCTCCTGCAGACGCCCCTCAAACTGCGCGGCACTGTGCCCCTTGACTCCAGCAAGGACCTCTTCACCTTT
AATCCCCAGCAGCTCGAACACCTGCTGGCTCAAGCTTCCTCGGATGACTCAAATGGGTAACTATAAAGAGATTATGATTT
TGCTTCCCCAAATGTTTTACCTTAACCTAGTCTCTTTTGTTAACATCCCATCAATGTCCTCACTCCCCATCTCTCTAAAA
CAGCTCTCTCCTATTCCTGCAAGTGGATAACAGGCCGTGCTCCCGTCTTCCTTCTACCTGTTTCCCATACGCCATCGAAG
CAGCTAACTTCCTACGGGCTGCTACGTCCTCGACTCGTGTGTCGGTCCCCTCTCACCCAGAATTAAAGGCCATTATTAGT
GTAAGAGGGGTTGGGGAGGAAATAGGAGGGAGAGAGGAGGACCCAGTTGAGGAAAAAGACGAAACTAATGGTATGTATCC
CGTTACGTGTGTGTGTGTGTGTGTGTGTGTGTGTGTGTGTGTGTGTGTGTGTGTGTGTGTGTGCGCGTGCGCGTGCGTGT
GTGTGTGTGTGTGTAAATGTGAGTACCACCTAATATCTCTGCACCCCTTCTCCGCACAGGAGCAACACCTCCATGGCTAT
GGGCTGTGATCGGCGTGGCAACGGGCCTGGTACTGGCTTTGGTCTTGATGGTTGTCTTGGCGATCAGAAGGGTGAGACGA
CGACGAGCGGAGAGGGAGGGAGGAGGAGAGAGGGTTAGGCACAGATCCACTGTCACTGAGAATGACAGCGGAGCCAATGT
GGCCAAGGCATGGGCACAGCACACACCCCACCGAGAGCAGAGGGGCAGGACAGGGAGAGAGAAAGACAGGAATGGGATAA
AGAAGAAGAAAGCAAAGGAGGCTGAGAAGAAAAGACGCAGAGATCCACTGGGGGAGGATGCCATTCGACTGCGGTGAGCG
AAGATGAATGATTATGGGAGAAGTTAGAACATGTTCATTCTAGTAAATCAACAGTGCTGTGATCAGATAACAGTTTGCTT
CATTGTAATGGGTTGCATGGCTTTCAGTTGTTTCCATTAGGGTCATTTTAATCTAACTGAACTTTTACTCTGTCTGTAAG
ATAAAACTGTTCTGTTGGACTACCTCTATTTTACACAATATTAGTGATACTGTAGTCATATCCCACGCACAAAGTACTGC
TATTCAGAGCATAAACGCATGATTTCAGGCCCCTGAAAAAGGACCTGGATATTGGAAGTGACACTGACTTTACCCAGAGT
TCTATGGAGGACATCAACACGTCCATCCGTGACCACCGAACGCAAGAGCAGAAGCACTACCGCAGCCCTCCAAGCCACCC
ACAATCACCCATACAACGTAAGATCACCTCCTGATCATGGTTAACATACTGCATTAATAGGCTTGTCAAATGATTCATTA
TCTCTTACATTCATTTGTCACAGAATAGTTTACTGTTTGCACAACGTAACTAAGCATGTAACTAAATTGACTTTCAGCTC
CACTTTTAGCTCCCCCAAGAGGATGGGAGAGAAATGCTGTCCCGTCTCCACATCACAGAACACCCAATCAAGTGAGTTCC
ATAAATATTCTACATAAATGTCAATATGTTTATTTACATTTTAGATAAGGCTGGTTATTATTTACAGTATTGTATTTTGA
CCGTACCAAACATTATTTTGACAGTCTGGTTCGGTTCAGTGGTGTGGTCCAGATGGGTCAGTGGTCCTGATTCGTGCAGT
CCGGAGTGGGCTTGACCGTGTGGTCCTGGAGCTGCTACGAGCTGGGGTGCCGGTCAACAACACAGACCACACTGGTATCA
CTTTACTCTCCCAATTTCCCAATTCTATTATTAACCAAAATGTAGGCTAGACGGTAAAATAAGTCAACTGAACTTCCCTG
TAGAGTGTCATGGGAGGATTATTGTGAAAATATATTTTAAAAATCAATATTGCGTGGAACAAATGTCCATAAATGTTATG
TAGCTTCCATAGGTGTAATAACATGCTTATAAACACACTGCCTCACATACATATTACTATATTGTATTACTTTGAGGGGT

TGAGTAACCAGTGTAACTGACCCCATCTCTCTCTGGTCCTCCCCATGTCGCGTCCCTTTTTGTGTCCCTTCTCTTCTCTT
CTGTTCTGGTAAAACCTCCATGGCTTCCCACTCTTCCTTTCCATTCCTCTCTACAGCACCCCTGCTGCCTCCCTCTCAAA
CCGATACAGAAGTTAATGTGCCCTAGTGCCCAGGCAGCGTGGATGTACCATATTAGCCTTCCTTGCTCTTCTCTCTCTCT
CTCACTCTCTCTCTCTCTCTCTCTCTCTCTCTCTCTCTCTCTCACACACCATCTCGGGCTGATTCCCTAGTCCCTCACCA
TCATTTGCATTCTCATTTCCTCCCTTTCAAAATCTCCCTCTTCCCCGTCTTTCACCCTTTGGGCTTGACTCTGTTGTATA
ATGTTTGTTCACTGGCTGACTTACTCCCTCTCTTTCTGTACTGAACTCCCACCTTCCTTCCCTCACCTTCCTCCACTTGA
AATGCACTCTCTCTCCCAGGGAGATCAGCCCTCCACTGGGCATGCTCAGTTAACCACCTCTCCTTGACAAGAACCCTCAC
TCGCTACGGTGCTGCGGTGGACCTACAGGACAACAAGGTACCAAGGCCAGCAGAAGCCTCTGCTTTTATGATTCTTCACG
TACTAGTATGAAGATTTAGGAAGAGAAATTCGAAGAAACTATATTCTGTGTAGAATTGTTAAACTTTAAGGTACTCCCAT
ATTATTGCTTCGACACTCCCATTCTCTGATATATACCACTCCCATTATCTGATAGGGTGAGACTGCTCTGTTCCTCTCCG
CCCTCCACGGTTGCTACGACACCGCCCGGTTCCTGCTCCTGAACGGTGCCAATCAGGATCTGTCTGATCGCAGAGGACGC
CGGGCACTGGATGTTGCCCGAGAGGGCATACATCATCAAGTCCTGGAGCTCCTATTGGCTCACAGGGTTCAGAGAGGGCC
TATTCCTATGGAGCAAGCCAATGATATGCTGTGGGATGAGCGTGCCTTGCTGTATAGCCAATGGGTGAATTCCCCTGGGC
TCCCTGGGAGAAGTGCCTCCTTCTCTGGTGTCATAGGGCATCGGGATATGTCTTCGCCTCCACCAAGGTAAAGTCTTGTG
GCATTTGGCACATCTACTCACAATAACATTTAACAATTTACTAATCTTGATAAGGCAAATTCATCACTGCCTACTCATAT
GATCGCCCTCTTCTTTTGACAGTGATTGGTCAATGGGCAGAGTGCAGTGCCCTTCCCCTCAGAACTGGCGGCCACAGCTC
AACCAATCAGTGACCGCATTGGTCACCCCAAGAATCATGGGGCGTCCCCCACGGCCAATCAGCACTCTGCAGGAAGTTAC
CTCAGAGGACGAAGATCGTGAAAGGCCCCAGGAAGTCCCGAGAGCAGCGACACCGCACTTCCTGTTACCCCAGCCTGCTC
CTCGACAGCGGTCCTTCTCCTGTACCCAGAATGCACTGCAGCGTCGCTCCAGTTCACAGCAGCCCGAACCAACTTATGTT
GCCTTATCGGAGAAAATTGCCAATGAGCCCATAGAAAGAGTGATCGTAGTCCCTCCTACAGATGCTCCCACCCAATCAAA
TCGTAGATCAATAGTCAATAGTAGCGATAACCCCAACAGGGCGGCACCAGAGATTGAGACAGCAAGTTTTAAAAAGGCAG
ACCAGAAAGCCCGAAGTGAGAAGCTAAATAACCACATGCCTGACTCTAAACAAACAGCTTTGTAAGGGAGAGGGCTCTGC
TAAAATTACCCCAAATACCATGTCAATCACGTGCTGTTTACATTTTCTGTAACAGGCTCCTTGCCCCTAAATAACCGCAG
ATTGACAGACGGGATATAAACTCAGTCAATAAATGAGTAACTTAATTTACTCTAAATATCTAAATCAATAAACTATGAAA
TAATTCATGTGCAATTGTTTTCTTCTGCCATATTAGGCAGTGACCTGTCTCTGCTGATATCAACCAATCAAGACAGACCA
ACTGACTGTGAATACAGAGTCTTTATTGTAAAAGAGGTAGAGAGAATACAGGTGTTTTTCACTCACTGTACAGAATGACA
GCACTGTTGATATACTCATTCAAACCGTTAATATAACCAGCACACACACTTCATTCATGATATAGACACACAGACAAACA
AGCCCAGTAGAAAAGAGAAAACACACACATTAAATCTTTGGATTTTCTCTATTGCCCCCATATCCTCTAAATGCAACTAT
ATCTGAGCTTTAAACGAAAATCCTAAAATATTTGTAATAACAGCATTAATAATATAGCCTAATAAAACAATGGTAATGCA
ATTTCCCAAGCCTCCCTGCCTACATACTACAGTAACATATAAAGTAGTGGAAAACAAAAACCCATGATCCCAATACAAAA
CATCACACACACACTGACACAAAAATCACACATTCAGCTCATACAAACATAATGAACAGTATCATTGGACAGTAAGAAAC
AGAAAAGACATACAAAAACTGCAGCCAAGTAAGAGTGATATCATTCTTTCAGTCTTTTCCGTTGTCAATCTTTCCATCTA
GGTCATTGACCCAAAGGGGGCGGGGCCATCAGTCAGAGTCCCCCGAGTGGTCGTTTCCCGGGGAAGTGGCAGAGGGGGGC
GTGTTTTGCCCTTGCCAGTTCCCATTAGTCTGCAAAACAGAAAAAGACATGGTGACTTGTAGTGGCATTAACCTACATTA
ACCGCTCTCCAACACAACTCCTCCCCAAGATTGTTGTTGTGAATGAGAATGTGCTCTCAATTAGCTACGAGCTAAAATAA
AGATGATATGTAACATGAAGTGAATGACAGTGAAGTTCCATACTTGTGCTCCCATGTAGACAGGCTGCTCTCCATTCATT
GGTGGAACACCCAGGAAAAGGTCTGGTCCAGTGAGTGAGAAGCCCCCCGACCCTGTGGAGGAACAAAATGGGAGATGTCT
TTAATAAGGGCTACGAGGCCTACAACACCAGTCTGGTCCCAGATCTGATCCTGTGGAGGAACAAAATGGGAGATGTCTTT
AATAAAGGCTACTAGGCCTACAACATCAGCCTGGTCCCAGATCTGATTGTGCTATAAAGCCGACTTCTATTGTTGGTCAA
GGAGTTGGCACGACAGCACAAAAGACCAATTCCGAATTAGGAAATTACGCAGGTCTTTCCTACGAAATTACGCAGGCCTT
TCCTACGCACTTCTCAGTAGTTGGTGTTCAGACGCATCTTATTCAGTTGCGTAACGGGCTTTGCAGACATAGTTCCCTTG
TGTGCTGATTAAGTGTAATTCAACTGCTGAAAACCCTCCCACTTGCTGGTCAATAGATTTTCTCGTGGAGTTTTCAGCAC
ATTTATTTTAAGCCATCCCTTTAAATATGGTATACATTTTAAGTTAGAATTTTGTTGACAGACTCGACAGAATATATCGA
GAGAACGGGTTCAGAATATTAGGGATCAATCAAAGAAAGTCATCTAAATATATGCATATTCGTCTCCGTTTCAGCACCAA
TTGGTAGAATTAAAAATAGAAAGCTGATAGACTGCAGATTATTTAGGGTTAGGAAAGTATTTATGAAAAACAGTGTTGGA
GAGCCTTTACACACGAAAGAAAACGGTTGTTTGATTCATTCTGAAAATCACCAAATTCAGTTTTTAACCCATGGTTATGT
TGGAAGATCAGCGTACGCAGAATTATATAATAGGGAGAATAGTGTACAATAGTAGACTATATCCTCCTCTATTTCCTGCAAT
AACCATGGAACTGTGTGATGACACAGCCAAGTATTGCGCCATGCGTTGGGTTCAAACTGTTACGGCATGAGCTGCGCTAC
GTTCCAGTACGATTTAATTAGCCTAAATGTAGTCATAGCTCTTTGTAGAGGTAGGTTTTGGGGATGGGGGTCAAGAGACC
AGAGGTGGCAGAGCAGGTTGGAGTGTAAAGTTGGAGCATAGTCTGAAGGTACGGAGGGGCAGTTCCCCTTGCTGCTCCGT
AACACAAAAACACTAACAACCCATCCATTTCTATTACCAATTGAACCATAAACTCAAAACGTTGGCGTGTATCACTAGGC
CTGGCTTCCTTTCCTCAACTCTTACCTGTTGAGTTGGGCGTGTGTGATGAGTCCTCGTTCTGTGTCGCCACGATGGCCGT
CTTCATGGCATAGATGTTGGCCTCCTCTTGGAACTTCCCGATGTTCTTCTTGTAGCGGATTCTCTTGTTGCCAAACCAGT
TGCATACCTGTTCAGTTAACGAGCCAGGAAGAGGCTGGTCAATGGAGTCAACTGTATATGGACGTATCAATGGGTGAGCT
AATAGTAAGTATATGACGCAAGGTTACCTGAGAGACAGTGATGCTACACTGTTTGGCTAGCTCTTCTTTGGCCTCTTCAC
TGGGGTAAGGGTTGGCGAGATGGGAGTAGAAATACTCGTTCAGGACTTCCGTCGCCTGCTTGCTGAAGTTACGTCTCTTA
CGCCTGACAACACAAAGGTGAGGGTGTTGAGAGTGGGCATACAACCCACGCAGAAGTGTACATTTCACAACTACAGTTAT
AACACACAATAGTCATTCACAACCTGCACTGTAATAACATGACTTTGCATTGGTATGTGACTGGTATGACCATGAACCAC
TCTTAATTTATGGAATGTATTATTCTTCATATAAAAATACTACCTGTACATGAATTGTCATTCACGATCATGGCAAGTTT
TGACAGGTGTTGTGTCACCACAATAACAGTGTTACACGCTGACCTGGCGTCGAGGAAGCGGGACCTCAGGATCATGACGG
CCTCGCAGGTGCTCTGCTTGAGTTGTGTCTGGATGGAGCTGAACTTGCGGTGGATGATGGCCACCATGCGTTCGATCTCC
CGCGGTGACACGGGTCGTGTACGCGACTGCTCCCTCAGCAGGTTCATCACGTGGGTAGTGAACTCACTACATGCCTACAG
GAGACACGGAGAGAGAGGGGGGAATTGGAGACAGATATGACAGTCAGTTGGGCTCAAAGGATGGAAGTCAACACTGACCC
CAGCACACANNNNNNNNNNNNNNNNNNNNNNNNNNNNNNNNNNNNNNNNNNNNNNNNNNNNNNNNNNNNNNNNNNNNNNNN
NNNNNNNNNNNNNNNNNNNNNNNNNNNNNNNNNNNNNNNNNNNNNNNNNNNNNNNNNNNNNNNNNNNNNNNNNNNNNNNN
NNNNNNNNNNNNNNNNNNNNNNNNNNNNNNNNNNNNNNNNNNNNNNNNNNNNNNNNNNNNNNNNNNNNNNNNNNNNNNNN
NNNNNNNAAGTACCTGGTCATATTCTCCATCTCAGTGTGATAGATGTTGCGAATCTGGCTCAACTTGCTTTTGTAGTCTG
AGTGTTCCAGTGAGCTGTCTGGCGACATCCCTCCAGAGCTGGTGGCTGCGGACACCGCCGCTGCCGCCCCGCCACCTTTC
TCCGGCCCAGCCACACCCTCCGCCAGCAGCATGTTGTCCAATCGCACCAGCTGTGGGTCCTGTGATTCTTCCTCCTGAGC

ATTTCGCATTGATAGGCCTGCAACAAGAGAGGTCTGTCAATCTGATTCATGGTAAATTAAGAGTTACAATGTCTCCTATT
ACACTGAACAAAAATATCAATGCAGCATGCAACAGTTTCAAAGATATTACTGAGTTACAGTTCATATAAGATAATTAGTC
AATTGAAATAAATTAATTAGGCCCCAATCTATGGATTTCACATGACTAGGAATACAGATAAGCATCTGTTGGTCACAGAT
ACCTTTAAAAAAAAAAGTAGGGGCTTGGATCGGAAAACTAGTCAGTATCTGGTGTGACCACCATTTGCCTCATGCAGCGC
CACACATCTTCTCACATTGAGTTGATCAGGCTATTTATTGTGGCCTCTGGAATGTTGTCCCACTCCTCTTCAATGGCTGT
GCGAAGTTGCTGGATATTGCTGGGAACTGGAAGACACTGTCGTACATGTCGATCCAGAGCATCCCAAACATGCTCAATGG
GTGACATTGTGCAGGCCATGGAAGAACTGGGACATTTTCAGCTTCCAGGAACTGTGTACAGATCCTTGCATTATCATACT
AAAACGTGATGATGGCGGCAGATGAATGGCACGACAATTCATTGCATTCAAATTTCCATAGATATAATATAAAAACAATT
ATAAAATATAATTGTGTTTGTTGTCCGTAGCTTATGCCTGCCCATTCCATAATCCCACCGCCACCATAGGACGCGCAGTT
CACAACGCTGACATCAGCAAACCACTCGCCCACACGACACCATTACACACTGTCTGCCATCTGCCCGAAACTGGGATTCA
TCTGTGAAGAGCACACTTCTCCAGCGTGCCATATCCATCGAAGGTGAGCATTTGCCCACTGAAGTTGATTACGCCGCCTA
ACTGCAGTCAGGTCAAGACCCTGGTGAGGACGACGAGCACGCAAATCAGCTTCCCTGAGACAGTTTTGACATTTTGTGGA
GAGATTATTCTGTTTCCCACAGTTTCATCACCAGTCCGGCTGGCTGTTCTCAGTCGATCCCGCAGGTGAAGAAGCCGGAT
GGTCTGCTTTGTGAGGCAGGTTGGATGTACAGCCAAATTCTCTAAAACAACATTCGAGGTGGCTTATGGTAGCAACAGCT
CTGGTGGACATTCCTGCAGTCAGCATACCAATTGCATGCTACCTAAAAAAAGATATCTGTGGCATTGTGTTGTGTGACAT
AACTGCACATTTTAGAGTGGCCTTTTATTCTCCCCCGCACAAGGTGCACCTGTGTAATGATCATGCTGTTTAATCAGCTT
CTTGATATGCCACACCTGTCAGGTGGATTGATTGTCTTGGCAAAGGAGAAATGCTCATTAACAGGGATGTAAACACATTT
GTGCACAAAAAAAAGCATGAAAATTTCAGGGATATTTTATTTCACCTCATGAAACCAACACTTTACATGTTGCATTTTCG
TTCAGTGTATATTAAAATATTCTCCCTCTGAGGTACAGACTTGGAGAAGCACTATGTGGATGAAACAAATACTGGAAGGC
TCATCTTGCGGCAAAATTTGAAAAGTCATCATACACAATACAACTTTGTTGAAAATTATAAATAGCTAGAGAATTTTCTA
ACTGTCATCTAACTGCGTGGATCATGCGCAACTCATTATTATGATGGAGCCAACATCTGCTTTCGTCACAGATGGCCAGA
AACTTTTAAACGAATTAGCAACAAGTTTTAAATCAAACAAAGGGTTTCAATTAGTGAAAATTAACTTACCTGTTTTCTCT
TTGATCTCGCATAAGACGCTGAACAAAGCTGGTTTCATTCGATGGCAATTCAATGCGTGTTTTCTGACGATTGAAAAAAA
AAGATCATTGTTATTAGCTAGCTAACTAGCTACTGTAGCTGTCTATCTAGTTTATCTAGCCAGCTATTTAGCAACGAGCC
ATTAGCACCAACAAAATATTTGTTAAGTTAGCTAGCTCCCTGCTTTCACACGCTTGATATAGCTAGCTAACGTTAACGAG
ATGACATTCATAACAACAAACATCGGCTGTTGTTCATGACCTTTTGACGAGTTCACTGCAGCCTGCTATGCTAGCGTTAG
CTACGCAATGGTATAGCTAGCTAGCAGCAGCTACAATATGTAGCTAATATGTTCACATTTTATTCGTAGAACAAATGTAA
CATGAAATGGATAATGTCAAGATTATGGCCACAGTCCGTTTATAATATATTGCTATTCACATACTACTAACGGTTATATT
TGCATGTAACACATTAGCTAGTTAGCAAACTAACAATAAAGCAATAGAGCGTAGCTAACTGGCTAGTTTTAGCTTAGCAA
GAATTCACCCCCTTCATCGAGTTAGCCACAAAAACTATTTCGGTTTTTATAAATTTCACAATAACAACAGGCGTGTAAAT
AGGCCAGGGGAAGAAGTGATAACCATTTAATAAGGCGTGCAAATGGCTTTATCATGAAACAAACCTAGTTAGCTTGCGAG
CAACGTTGTCTACCTAGCTAGCTTATTAAAAAACTTCAAAGTTGAAGAAAAAAAAAAACTTCTGAAAACTTTAGCTAACT
CTTGGCCATTGAGCAACTTTGCAGTAAGGTGTAGCTAAATCTGAATACGTTAGAAACAATTATGACTAACTTTGCTTGTG
CTTCGTCCAAACTTTGGTCGGTGATGGTCATGATTTGTTGCAATATGTCACCAATGTCTCTGCGGCTTTCATGTCCATTT
TCTGCGCCTTCGTGGCCCGTGTCTCCGTCGGAGCGGTGGTGGGAAGATGGATGAACCGAGTTTAGAGGGTGCATACCGGG
GTGAGAGCTCAGGCCAAGGCCCCGGCCGTTAGAGGGCCCGTTGCCAGTGAGAGGCTGCTGCTGCAACATCCTGAGCGGCA
CACAGTGGCGCGGACGAGTAATCACAGTCATACACCCTTCAAAACTCAAACACTAGAGCAGGCCAGCACTAGAGTCCCCT
AGCGATTGACAAAGAAAAAGTAAAGAACAAGAATGTTCATACAATTCGAATTGTACTCCAGACAGACCCCATAGGCTACT
GTACTAGGGATATTCATAGCTATGACTTTTAAACTAAGTAAGTCTTCTGAACAATCAGGTAGATGGATATAAGTATAAGG
CAACTGTGTGTGTAGGCCTATGTGTGTGTAAGAGAGAAAGAGAAACAAAACGTGATCTGCAAGTAGCCTAATACATTGAA
CATTTGCATCAATGATGGGGGAGGATCATCTTCTGATTTCATGTCAGCTAATTTAATAATGCATCAGCTTCATTTCTTAA
AATGTTTTATTTCACCTTTATTTAACCAGGTAGGCTAGTTGAGAACAAGTTCTCATTTGCAACTGCAACCTGGCCAAGAT
AAAGCGTAGCAATTCGACACATACAACAACACAGAGTTACACATGGAATAAACAAAACATACAGTCAATAATACAGTAGA
ACAAAAGAAAACAAAAAGTCTATATACAGTGAGTGCAAATGAGGTAAGTTAAGGCAATAAATAGGCCATGGTGGCGAAGT
AATTACAATATAGCAATTAAACACTGGAATGGTAGATGTGCAGAAGATGAATGTGCAACTAGAGATACTGGGGTGCAAAG
GAGCAGGATAAATAAATAAATACAGTATGGGGATGAGGTAGATGGGCTGTTTACAGATGGGCTATGTACAGGTGCAGTGA
TCTGTGAGCTGCTCTGACAGCTGGTGCTTGAAGCTAGTGAGGGAGATATGAGTCTCCAGCTTCAGAGGTTTTTGCAATTC
GTTCCAGTCATTGGCAGCAGAGAACTGGAAGGAAAGGCGGCCAAAGGAGGAATTGGCTTTGGGGGTGACCAGTGAGATAT
ACCTGCTGGAGCGCATGCTACGAGTGGGTGCTGCTATGGTGACCAGTGAGCTGCGATAAGGCGGGGCTTTACCTAGCATA
GACTTGTAGATAACCTGTAGCCAGTGGGTTTGGCGACGAGTATGAAGCGAGGGCCAACCAACGAGAGCGTACAGGTCGCA
AAACAAAACACTGTGATAGACTGCATCCAGTTTGTTGAGTAGAGTGTTGGTGGCTATCGAGGATCGGTAGGATGGTCAGT
TTTACGAGGGTGTGTTTGGCAGCATGAGTGAAGGATGCTTTGTTGCGAAATAGGAAGCTGATTCTAGATTTAATTTTGGA
TTGGAGATGCTTAATGTGAGTCTGGAAGGAGAGTTTACAATCTAACCAGACACCCAGGTATTTGTAGTTGTCCACGTATT
CTAAGTCAGAGCCGTCCAGAGTAGTGATGCTGGACGGGCGAGCAGGTGCGGGCAGTGATCGATTGAATAGCATGCATTTA
GTTTTACCTGCGTTTAAGAGCAGTTGGAGGCCACGGAAGGAGAGTTGTATGGCATTGAAGCTCGTCTGGAGGTTAGTTAA
CACAGTGTCCAAAGAGGGGCCAGAAGTATACAGAATGGTGTCGTCTGCGTAGAGGTGGATCAGAGAATCACCAGCAACAA
GAGCAACATCATTGATGTATACAGAGAAGAGAGTCGGCCCGAGAATTGAACCCTGTGGCACACACATAGAGACTGCCAGA
GGTCCGGACAACAGGCCCTCCGATTTGACACACTGAACTCTATCAGAGAAGTAGTTGGTAAACCAGGCGAGGCAATCATT
TGAGAAACCAAGGCTGTCGAGTCTGCCAATAAGAATGTGGTGATTGACAGAGTCGAAAGCCTTGGCCAGGTTGATGAATA
CGGCTGTACAGTAATGTCTCTTATCGATGGCGGTTATGATGTCGTTTAGGACCGTGAGCGTGGCTGAGGTGCACCCATGA
CCAGCTCTGAAACCAGATTGCATAGCGGAGAAGGTACGGTGGGATTCGAAATGGTCGGTAATCTGTTTGTTAACTTGGTT
TTCGAAGACCTTAGAAAGACAGGGTAGGATAGATATAGGTCTGTAGCAGTTTGGGTCTAGAGTGTCACCCCCTTTGAAGA
GAGGGATGACCATGGCAGCTTTCCAAACTTTTGGAATCTCAGACGATACGAAAGAGAGGTTGAACAGGCTAGTAATAGGG
GTTGCAACAATTTCGGCAGATAATTTTAGAAAGAGAGGGTCCAGATTGTCTAGCCCGGCTGATTTGTATGGGTCCAGATT
TTGCAGCTCTTTCAGAACATCAGCTATCTGGATTTGGGTGAAGGAGAAATGGTGGAGGCTTTGGCGGGTTGCTGTGGAGG
GTGCCGGGCAGTTGACCGTGGTAGGGGTAGCCAGGTGGAAAGCATGGCCAGCCGTAGAGAAATGCTTATTGAAATTCTCA
ATTATCGTGGATTTATCGGTGGTAACAGTGTTTCCTAGCCTCAGAGCAGTGGGCAGCTGGGAGGAGGTGCTCTTATTCTC
CATGGACTTTACAGTGTCCCAGAACTTTTTTGAGTTAGTACTACAGGATGCAAATTTCTGTTTGAAAAAGCTAGCCTTAG
CTTTTCTAACTGCCTGTGTATATTTGTTTCTAACTTCCCTGAAAAGTTGCATATCACGGGGGCTATTCAATGCTAATGCA

GAACGCCACAGGATATTTTTGTGCTGGTCAAGGGCAGACAGGTCTGGAGTGAACCAAGGACTATATCTATTCCTAGTTCA
ACATTTTTTGAGTGGGGCATGCTTATTTAAGATGGTGAGGAAGGCACTTTTAAAGAATAGCCAGGCATCATCTACTGACG
GGATGAGGTCAATGTCATTCCAGGTAACCCCGGCCAGGTCGATTAGAAAGGCCTGCTCGCAGAAGTGTTTTAGGGAGCGT
TTGACAGTGATGAGGGGTGGTCGTTTGGTCGCCGACCCATTACGGATGCAGGCAATGAGACAGTGATCGCTGAGATCTTG
ATTGAAAACAGCAGAGGTGTATTTGGAGGGCGAGTTAGTTAGGATGACATCTATGAGGGTGCCCGTGTTTATGGATTTGG
AGTTGTACCTGGTAGGTTCATTGATAATTTGTGTGAGATTGAGGGCATCAAGCTTAGATTGTAGGATGGCCGGGGTGTTA
AGCATGTCCAAGTTTAGGTCACCTAGTAGCACGAGCTCAGAAGATAGATGGGGGGCAATCAATTCACATATGGTATCGAG
GGCACAGCTGGGGGCAGAGGGAGGTCTATAGCAAGCGGCAACAGTGAGAGACTTGTTTCTGGAAAGGTGAATTTTTAGAA
GTAGAAGCTCGAATTGTTTAGGTACGGACCTGGATAGTAGGACAGAACTCTGCAGGCTATCTTTGCAGTAGATTGCAACA
CCGCCCCCTTTGGCAGTTCTATCTTGGCGGAAAATGTTATCGTTAGCGATGGAGATTTCAGGGAATTTGGTGGTTTTCCT
AAGCCAGGATTCAGACACGGCTAAGACATCCGGGTTGGCAGAGTGTGCTAAAGCAGTGAGTAAAACAAACTTTGGGAGTA
GGCTTCTAATGTTAACATGCATGAAACCAAGGCTTTTACGGTTACAGAAGTCAACAAATGAGAGCACCTGGGGAGTGGGA
GTAGAGCTAGGGCACTGCAGGACCTGGATTAACCTCCACATCACCAGAGGAACAGAGGAGAAGTAGGATAAGGATACGGC
TAAAGGCTATACGAACTGGCCGTCTAGCACGTTTGGAACAGAGAGTAAAAGGAGCAGGTTTCTGGGCATGATAGCATAGA
TTCAAGGCATAGTGTACAGACAAAGGTAAGGTAGGTTGTGAGTACATTGGAGGTAAACCTAGGCATTGAGTAATGATGAG
AGAGATATAGTCTCTAGAGACGTTTAAACCAGGTGATGTCATCGCATATGTAGGAGGTGGAACAACATGGTTGGTTAAGG
CATATTGAGCAGGGCTAGAGGCTCTACAGTGAAATAAGACAGTAATCACTAACCAGGACAGTAATGGACGAGGCATATTG
ATATTAGAGAGAGGCATGCGTAGCCAAGTGAACATATGGGTCCAGTGATTGGTTGGGCTGACTGGGACACGGCGATTCAG
ACAGTTAGCAGGCCGATGCTAACAGTTAGTAGGCCGGGGCTAAACAAGCTAGCAATTAGCAGACTGGGTTAGCAAGCAGG
CAGTTGGCAGACCGGGGCAGGCAAACTAGCAGTTAGCAGACCAGGACTAGCAAGTTAGCCTTTGGGGGACGTCGCGATGG
GGGTAAGTCTGTTTTTGCCTCTTCGTGCAGTGACGTCGATAGACCAGTCGTGGAATTAGTAGGGTCATAATTCATAATAA
GGTCACAGGTCTTTACCAATCAAATCATAAAAAATTCATCACATTTTATTTGTCACATGCTTCGTGAACAACAGGTGCAG
ACTAACAGTGAAAGTCTTACTTTTGTGCCCTTCCCAGCAATTTAGAGAGAAAAATATAAAAATAATAACACAATGAATAG
ATACACCATGAGTAACGATAACTTGGCTATATACACTGGGTACCAGTACCGAGTTGACATGCAGGGGTACTAGGAGGTAA
TTGAGGTAGATTTACATATAGGTAGGGGTAAAGTGACTAGGCAAAAGGGTAGATAATAAGCAGTAGCAGCTGTGTATGTG
ATGAGTCAAAAGAGTTTGAGTGTCAGCATGGTGGATGTGTTGTGGCCTACTCTCACCACATGGGACAATACAGACAATAT
AAGCACTCCATAATATTAACAGATTTGACAAATTAATTCACAGTGATAAAGTTTCATACATCAATGGGTGATACACAGCA
GAACACTTTTTAGAACTACAGAATAGTTCAGACAAGTCATATATGATCATACACAAATGAACAATTACCCTCCCCTCTGT
TTTCTCACTTCACCAAAAATAATCAACATCTCACATCTGACTATATCTCTCTCCCTCTCTCTCACACACTTTGTCTACTT
CTCCTTTAGCTGCAAGTCAACAGGAAGTATGCCAAACCCACAGTCCCTGTCAGAATAACAGAGTACCTGTTTCAACATGTT
GCTGTTTCTGACTGTATGCTGTTCTGCTGAAAATGCAACATGAGCAGAGCAAAACTTCCTAGTCATTTCCCCCTTTCTGA
TGTTCATCTCAAATTATGAGTTTTGGCTTTGTTAAAAACAAGGCTATTTGATAACATTGCACCACTTCACTTTTTACAGC
AATATTTCATAATGCCATGATGCAGAGTGATAGGGGACAAATAAATAGGGGTGGGTTGGGGTAAGAGGAACAGGAAGAGA
CTAGAGGGCTGACAGGCAGGCAGAGAGAGCGAAAGCGAGAGAGAGCTCGTCACGGTGATATGTGAAGCATAACTGATAGC
AATTAGTTGTCATGAAGAAAACTGCCATGTGAATCAGTGATGCCATACCTAATCAATGTGGACACAAGAAGATGATGTTT
ATCAGAAACTGAACTCCAGGAAAACACAGGTACGATAGACTTATTGAGAGTGTGGGTGGGTAAGGTAATGATTGGCAGGC
GGGCAAGTACAATCCAAGATAGGAATTCAGTGCCTGAAGAAGTGAGCAGGAAACCAGTCCTGGTTTTTCAGAAACACCCC
TCTTGAGATTATACCATATTATGCTTACAGTGGCTGTTCAGTCTGTATCACGCTAGAAGTCTTCTACATGTGTTGTTGGG
AGTTAACATTTTGATCAACTCTATTTGATTACCTAATACATTTTCTTAGAACCAGTTCCACAATCTTCATAATGTACAAT
AGAACATCAGTTGATATATTTGTCTTATCTAATTTTTTGTCAGGTTTACACTGAGACAGACGTAAATGGATGTTGTCATG
GAATACCCAGAGGGCAAAGTTGAAACAGCTGAAGAGGAGCACTTGGAGGATCTAATCATTATTAAGGAGGGGGATGTTGC
GGGAGGGATAGAGCCAATAACAGGCAAGAGCAACGATGAAGACTCCAGTGCGGAAGAGGATGTCACTGCCAGATATGGAC
AGACTGGAAAAGCCAACACTGAAAGTGGAGATGGCGACAAAAGAAAAAAGGATAACCTTATAAATCAAGCAAAAGGAGAG
ACTGGGACAGAGGAGGGGGTTGAGCACATTAAAGGGATGGATGAATTAGTAATGGATGGACCGAAAATACATGGCAAGAA
AGAGGGGATGGCGATGGTGGATAAAACAGAAAAATACAAAACAGCAGAACAAGATTTGGAGACGGACAAACCATCACAAG
GATTACAAGTTGGCACAAATAGACATCTTCCCTTTGACCAGTTGCCCAAAGATACGCTGAGCCCAGAGCATGCCCCAAAA
CAGGTTTATAACATTTGCTTTCACTAAAAAAGTACAGCAATTTCCCTTGTGCCCACAATTACAATAATTCCGTTTTTGGG
TTTTCTTTCACATATAGAAAAAGAACAAACAACATTCCAGTGGTGGAAGAATGAGGGGTTGATTTTGTGGTTACAGAATT
TGTAGGTGTGGAAATGCTAGCGTGTTTGAAATGAGTAGTGATACCCCCCATTTTAAAGTCCTTGATAATAACTACTCTTT
CTGCCACCTGTCTTTCCCAGGCTCAACGGTTAACCCCTTACTTCCCAGACGCGCTGTATGACCTGGTCTTTACTCTGCAA
GAAGGAAGACGACTCAATGACCAGCGGTGCTCGTTCAGAGGGAGGAGAAGGTGCCATTCTGAACCCAACACCTACATTCC
GGCCCACAGAGCCCACAGAGGTGATAGAATGTGAAAACAACTCCCTGTAATGATAACTAACTAAGTGACTGTGGTAAACA
TAACAACTGTTGCAATTAGCTTCCCTTGTTTCACCATTGACACGCTGACTCACTTCAAATGACAGAAGGGGGAAAGCCGT
AAATAACTGTACGGTACTAACATCCATTGAAGCTCTTCTATCATATAACTGTGGCAGGGACAGATATTAGGATGTCTTCT
GTACATTAGTAAAATATTGAAATAATATTGCTTGAATGATTAACTAGGACACTAAATGAACTAGGCCACTAAATGAACTA
GGACACTAAATGAACTAGGACACTAAATGAACTAGGACACTAAATGTAGCATGTCTCCACTCTCTTTTCTCCCCTTCCAT
TCCCAACAGTGCATTTCTCCTCAATGACCTCGCTGCAGAAAGATGAGTTCTTTGACTTGGTGGCTACTTCCCAAAGCCGT
CGGCTTGACGACCAGCGGGTGGAACTACATGACGTCCCACCCCTTAAGCCGAAAGCCAATAAGAAGAGGAGCAGCGTAAA
AGATACAAAGCCTAAAAAGTCTGCTCCAATTGCAGTGCAGAACGAGGACTTGTACAATATGATTCTCATCTCGCAAGTAA
GTAACTGTAATCACCCAAGCTGCCAAATGTCAATTTGACCAAAACATTATTTTGTTCTTGTCATAAGCAAAATATCATGT
AGGCATCATGTCATATGGTGTTACGTTGATATCATGTTACAGTGCTACATTGGCATAACAGATGAAGGATCTTAATTTTC
CTGCACACCAGGAAATGCAATGACTTGTGCTTTACATAAATTAACGGAAAACCCGCACTAACACAGTTATATTAACAGTA
TTTCCCTTTTCAAGTAGCTTAATTTTGGTCAGTTTATAGCCTAACCACCGATCATGCAACATCATGGAATAAACGTTCAA
ATGCTGTTGCTGCAGGATTATTTTACTGCAACAATACTGGTCAAATGATTATCCTACAGCTATGTCATGTGCTGTAACAT
GGGCATTGGGTATTATGTTGTATGGTGTAACAACATATCATGTCATATGGGGTATAAGCATCATGTTATGTGATGTTACG
TAGGTGTAATGTTTCATCTCCGTACCTCAGGTCCAGTGTAAAATAGGCATCAAACCATGTTGTGTTTCTGACTGTCTCTG
TACTTCAGGCCCAGGGTAGACTGGAGGAGCAGCGCAGTGTGGCCCCAGGGCCTATGGATGATGAGGACTTCTTTTCCTTA
CTGCTGAGTGTCCAGGGAGGACGCATGGAGGACCAGAGGACTGAGTTGCCTGGGATTCTGGGAACCTGAGAGATACACAT
GCTGTAGAGATAGGCTCAAATGGCTGAGAGCATGTGGTGTAGTCCTATAGAGCAATGGCTTTTGCTTCTTATTCACCTGG

CCAGACCAGGCATGACCATTTTGTTCTGTTATGGTCTGAGTTGACGGTATCATCTGTATCGAGGACTGCATCAGTAATTT
AATAAAAAACATGTTTGTGAATAATAGAGGCGTCAATTCAGTTGCATAATGCTGTAGAGACTAATTCCAATGTTTGTTGT
ATGATATTAAGCTTACAGAGTGGGCAGATTTGTATGTTGACTGCACAATAATTTGGATGATATTCAAAATGCCCCTACGC
TTTGGGGTGCTTTAATGTGTTTGTTGCAATATAAGGAAATTAGTTTTGCTCACCACAGCAGTGAACAAGTATGAATGAGA
GTGTTTGTGTTTCCATCTTATATTAGAATACAGCTCCTCATGAGAATAAAATAAGCGTTAAATGTTTGACCTGCATCAAG
AGTGTTTCTAGTTGTTCCCCGAGAAGGTTTACGATAAAGTCCCTCAGCCTTTGTCTACTGGACCCCCCAGAGATATTGAG
GGCCAATGCAAGAGCCAACACAGCATCAAAAGCCTGGGGGCCTCCAGGAAGCCTGCAGTCTTGTCAGACTCCAGTCCTTG
CCAACTGAGACGGCAACTTTCAGAGGAAGTCTTGAACTGTCTAGACTGGGGAAGGTGATAAATGATGAGGAAGAATACTT
ATTATGGCAGTTCCTCAAAGTGTGATTCAACATACAAAAGGCACTCGCACATTTGAGCTATCTTTATTGCAGGTTGAATA
AGATCAGAAAAAATAAGAAACCTGCACACTGCTGACGAAGACATTGAGGCCAATACGTAAAGCTTATTAAAGAGCAGTGA
TAATAGCAAGAGCAGTGAAAAGTTATCAAATCATATGACAACTGGCTGCTCGAAGGTCAAAATGCTAAGTATGCTCTGTG
GAACACAAGGAANNNNNNNNNNNNNNNNNNNNNNNNNNNNNNNNNNNNNNNNNNNNNNNNNNNNNNNNNNNNNNNN
NNNNNNNNNNNNNNNNNNNNNNNNNNNNNNNNNNNNNNNNNNNNNNNNNNNNNNNNNNNNNNNNNNNNNNNNNNNNNNNN
NNNNNNNNNNNNNNNNNNNNNNNNNNNNNNNNNNNNNNNNNNNNNNNNNNNNNNNNNNNNNNNNNNNNNNNNNNNNNNNN
NNNNNNNNNNNNNNNNNNNNNNNNNNNNNNNNNNNNNNNNNNNNNNNNNNNNNNNNNNNNNNNNNNNNNNNNNNNNNNNN
NNNNNNNNNNNNNNNNNNNNNNNNNNNNNNNNNNNNNNNNNNNNNNNNNNNNNNNNNNNNNNNNNNNNNNNNNNNNNNNN
NNNNNNNNNNNNNNNNNNNNNNNNNNNNNNNNNNNNNNNNNNNNNNNNNNNNNNNNNNNNNNNNNNNNNNNNNNNNNNNN
NNNNNNNNNNNNNNNNNNNNNNNNNNNNNNNNNNNNNNNNNNNNNNNNNNNNNNNNNNNNNNNNNNNNNNNNNNNNNNNN
NNNNNNNNNNNNNNNNNNNNNNNNNNNNNNNNNNNNNNNNNNNNNNNNNNNNNNNNNNNNNNNNNNNNNNNNNNNNNNNN
NNNNNNNNNNNNNNNNNNNNNNNNNNNNNNNNNNNNNNNNNNNNNNNNNNNNNNNNNNNNNNNNNNNNNNNNNNNNNNNN
NNNNNNNNNNNNNNNNNNNNNNNNNNNNNNNNNNNNNNNNNNNNNNNNNNNNNNNNNNNNNNNNNNNNNNNNNNNNNNNN
NNNNNNNNNNNNNNNNNNNNNNNNNNNNNNNNNNNNNNNNNNNNNNNNNNNNNNNNNNNNNNNNNNNNNNNNNNNNNNNN
NNNNNNNNNNNNNNNNNNNNNNNNNNNNNNNNNNNNNNNNNNNNNNNNNNNNNNNNNNNNNNNNNNNNNNNNNNNNNNNN
NNNNNNNNNNNNNNNNNNNNNNNNNNNNNNNNNNNNNNNNNNNNNNNNNNNNNNNNNNNNNNNNNNNNNNNNNNNNNNNN
NNNNNNNNNNNNNNNNNNNNNNNNNNNNNNNNNNNNNNNNNNNNNNNNNNNNNNNNNNNNNNNNNNNNNNNNNNNNNNNN
NNNNNNNNNNNNNNNNNNNNNNNNNNNNNNNNNNNNNNNNNNNNNNNNNNNNNNNNNNNNNNNNNNNNNNNNNNNNNNNN
NNNNNNNNNNNNNNNNNNNNNNNNNNNNNNNNNNNNNNNNNNNNNNNNNNNNNNNNNNNNNNNNNNNNNNNNNNNNNNNN
NNNNNNNNNNNNNNNNNNNNNNNNNNNNNNNNNNNNNNNNNNNNNNNNNNNNNNNNNNNNNNNNNNNNNNNNNNNNNNNN
NNNNNNNNNNNNNNNNNNNNNNNNNNNNNNNNNNNNNNNNNNNNNNNNNNNNNNNNNNNNNNNNNNNNNNNNNNNNNNNN
NNNNNNNNNNNNNNNNNNNNNNNNNNNNNNNNNNNNNNNNNNNNNNNNNNNNNNNNNNNNNNNNNNNNNNNNNNNNNNNN
NNNNNNNNNNNNNNNNNNNNNNNNNNNNNNNNNNNNNNNNNNNNNNNNNNNNNNNNNNNNNNNNNNNNNNNNNNNNNNNN
NNNNNNNNNNNNNNNNNNNNNNNNNNNNNNNNNNNNNNNNNNNNNNNNNNNNNNNNNNNNNNNNNNNNNNNNNNNNNNNN
NNNNNNNNNNNNNNNNNNNNNNNNNNNNNNNNNNNNNNNNNNNNNNNNNNNNNNNNNNNNNNNNNNNNNNNNNNNNNNNN
NNNNNNNNNNNNNNNNNNNNNNNNNNNNNNNNNNNNNNNNNNNNNNNNNNNNNNNNNNNNNNNNNNNNNNNNNNNNNNNN
NNNNNNNNNNNNNNNNNNNNNNNNNNNNNNNNNNNNNNNNNNNNNNNNNNNNNNNNNNNNNNNNNNNNNNNNNNNNNNNN
NNNNNNNNNNNNNNNNNNNNNNNNNNNNNNNNNNNNNNNNNNNNNNNNNNNNNNNNNNNNNNNNNNNNNNNNNNNNNNNN
NNNNNNNNNNNNNNNNNNNNNNNNNNNNNNNNNNNNNNNNNNNNNNNNNNNNNNNNNNNNNNNNNNNNNNNNNNNNNNNN
NNNNNNNNNNNNNNNNNNNNNNNNNNNNNNNNNNNNNNNNNNNNNNNNNNNNNNNNNNNNNNNNNNNNNNNNNNNNNNNN
NNNNNNNNNNNNNNNNNNNNNNNNNNNNNNNNNNNNNNNNNNNNNNNNNNNNNNNNNNNNNNNNNNNNNNNNNNNNNNNN
NNNNNNNNNNNNNNNNNNNNNNNNNNNNNNNNNNNNNNNNNNNNNNNNNNNNNNNNNNNNNNNNNNNNNNNNNNNNNNNN
NNNNNNNNNNNNNNNNNNNNNNNNNNNNNNNNNNNNNNNNNNNNNNNNNNNNNNNNNNNNNNNNNNNNNNNNNNNNNNNN
NNNNNNNNNNNNNNNNNNNNNNNNNNNNNNNNNNNNNNNNNNNNNNNNNNNNNNNNNNNNNNNNNNNNNNNNNNNNNNNN
NNNNNNNNNNNNNNNNNNNNNNNNNNNNNNNNNNNNNNNNNNNNNNNNNNNNNNNNNNNNNNNNNNNNNNNNNNNNNNNN
NNNNNNNNNNNNNNNNNNNNNNNNNNNNNNNNNNNNNNNNNNNNNNNNNNNNNNNNNNNNNNNNNNNNNNNNNNNNNNNN
NNNNNNNNNNNNNNNNNNNNNNNNNNNNNNNNNNNNNNNNNNNNNNNNNNNNNNNNNNNNNNNNNNNNNNNNNNNNNNNN
NNNNNNNNNNNNNNNNNNNNNNNNNNNNNNNNNNNNNNNNNNNNNNNNNNNNNNNNNNNNNNNNNNNNNNNNNNNNNNNN
NNNNNNNNNNNNNNNNNNNNNNNNNNNNNNNNNNNNNNNNNNNNNNNNNNNNNNNNNNNNNNNNNNNNNNNNNNNNNNNN
NNNNNNNNNNNNNNNNNNNNNNNNNNNNNNNNNNNNNNNNNNNNNNNNNNNNNNNNNNNNNNNNNNNNNNNNNNNNNNNN
NNNNNNNNNNNNNNNNNNNNNNNNNNNNNNNNNNNNNNNNNNNNNNNNNNNNNNNNNNNNNNNNNNNNNNNNNNNNNNNN
NNNNNNNNNNNNNNNNNNNNNNNNNNNNNNNNNNNNNNNNNNNNNNNNNNNNNNNNNNNNNNNNNNNNNNNNNNNNNNNN
NNNNNNNNNNNNNNNNNNNNNNNNNNNNNNNNNNNNNNNNNNNNNNNNNNNNNNNNNNNNNNNNNNNNNNNNNNNNNNNN
NNNNNNNNNNNNNNNNNNNNNNNNNNNNNNNNNNNNNNNNNNNNNNNNNNNNNNNNNNNNNNNNNNNNNNNNNNNNNNNN
NNNNNNNNNNNNNNNNNNNNNNNNNNNNNNNNNNNNNNNNNNNNNNNNNNNNNNNNNNNNNNNNNNNNNNNNNNNNNNNN
NNNNNNNNNNNNNNNNNNNNNNNNNNNNNNNNNNNNNNNNNNNNNNNNNNNNNNNNNNNNNNNNNNNNNNNNNNNNNNNN
NNNNNNNNNNNNNNNNNNNNNNNNNNNNNNNNNNNNNNNNNNNNNNNNNNNNNNNNNNNNNNNNNNNNNNNNNNNNNNNN
NNNNNNNNNNNNNNNNNNNNNNNNNNNNNNNNNNNNNNNNNNGTGTTTCTGTTATTAAATTCAATGATGAACACTAACTC
CGCTGCACCTTGGTCTACTCTCTCTCCTGCCAGCCATTACACAACTAGGCTGTTAGGTTAGCCTATATGTGCTGTGGATT
AATTTTCCACAGCACAGTGGAAATGTATCAACTCCATCCCTTAACTGTTCAACCATTGTTGACAACAGCAAGGAAAACAC
ACTTGTTCATTAAATATGTATCATACGTTTGCGGCTTGCTTTATGGTCATATATGCTCTGATTCCATTTAAATGAATAGA
TTTTCCCCATGTGTAAAAGGCAGAAGGGTGCGTCTGGTCCAGGGCGTTAATGCAGTTCCTCCACTTCATTAACTCGTGAC
CAAAAGCCCGCCCTGTGACATCATACTTCAAAACAAGGATGCGTCTCAAATATAGAAATGGAATAGTTCGCCACAATCCA
AGATCACGTGGATACGGCAAAGCTGTAGAATCAGCGCCCTTGTCAAATTCTTATCTCCTCAAAGGTAGGCTAAGTGTTGA
TGTAGAAATGACTTTTCAATCAACCTTACAGTGGGGCACATTGGGCATAGGCTATAGCAGGGGTATTCAACTCTTTCTTA
CCCTACGAGGTCCGGAGCCTGCTGGGTTTCTGTTCTAGCTGATCATTATGTCCGCAGACCTGGGTCTGAATCAGTCCCTC
CTGATTATAGGAGAACAAAGAAAACGCAGTGGAATTGGCTTCAAGGTCTGATTTTTGAGGAGGCTATACATTTCGTTTGC
ATGGGTTATGCTTCATCCTAATTGATTCTAAAGCAGTTGACAAATCAGTTAGATATCAAATCAATGCAAAATCGGATACT
TTTTCTTAGTTTAATTCTGAAACATTGTTACAACATTGTAACATAATTGTAACATCGACTGTCGGCTACTGTTGCAATTT
GTAATGCATCGCTGCGTGCTCTTGTTCTAAAGCCACCCCTTTTCCATTTTAGTCAACACATGTTGAAGTTGTCATTTTGG
CAATAGTTTTTCACAGTTCCTAAAACTATTTTGACCTCGTTGCTTTCCCTTCAGATGTTGTCACTCGTTGTCCTGATGAT

CCTGTTTCAACCAGAAGAGGCAGGCTCCTCACCTATCTCCTGTTACAATGATCAAGGGGAAGCTGTTGACTGGTGAGAAA
ACGGTTCTGGGCAGACTGAAGATTGAGCTGATATAACTTCCTTCTTCCCTTAGGTCTGTTAGCTACTTAGTCACAGACAG
TGTGCATAGCAAGGTATGGGGTTGCAGGTAGCCTAGTGGTTACAGCATTGGGCAGCTAACCAAAAAGTTGCTAGATCGAA
TCCCTGAGCTGACAAGGTAAACATTCTTTCGTTCTGCCCCTGAACAAGGCAGTTCAGGGGCACTGTTCCTAGGCCGTCAT
TGTAAATAAGAGTTTGTTCTTAACTGACTTGCCTGGTTAAATAAATAAAAAATGCTAAAACAACACACAGTACTGAGAAT
CAAATACTTTCTCTCAGGTTCTATCTCTACAAACTCCCCCATGTTGATCGTGGACTTCTCAACGGCGGACAGAGTTATCT
ACTGATGGACAAAGGGAGCGAGGGATGGACGGATGGAAACGTGTTGGTGAATGACAGCACTGGAGCCCTGGGGAGGACAG
TGGGCCAGCTGTATGAGCAGGGGAAGGTCTGGTTCCTTCTATTATTAATATCAGACCACATTTGAATGAACTGTGAAACT
TAAAGGGAAAATCCACTCAAAAACGATTAAAAAAATAAATAATAATTATGAGTTGTCTGTTGATACAGACCCAGAATGTT
TTTTCATGATGATGTGGCCGGTGACACGTTGTTTCTTGGAATTTAAATATCTTGAAAGTTGACTGCTGACTTGCAAACCA
TTTTGGGACTGAATCAGAAGTGGATTAATTAAAAAAAAAATACCAAAAGATAGTTTTTGAGTGGATTTTCCCTTTAAGTTT
CACTTGCAACACATAGCAGTATTTACCACTGCTGAGGTTTTATTTTTGGTAGCTGGCCCATCCCTTTTTCTTTGTCACAG
TGGAATGAGACAGAATGACACAACAAAATCGAAATGTGGATCTATAACTTTTTTGTGGGTTGTTTCTTTCACTGTTTAAT
TTTCACTAAGGTGGAATTGGAGTTTTCTTTTCACCCAGACAGCCATGATAAATAAATACAGCATATTACATGTTATCAAA
GAACAAACACAATTGGCACTATAACCCTTGTTTGTTTCTGAGTTTAGAGTGTGCAATATGCATGAAAGTGTATATCTTCT
GACGATGAGTGTGTGTACTTTCTTTTGCAGAATGATGACATTGCATACATCCTGTACAATGACCAGAGACCACCCGAGGA
GGGAGAAAGAGGATTTGGTTTCCTTAGTAGCCGAGGTGGCCACACTAAAGGTCAGGACAATGTAAACCTCAGCTGTATAC
TGTATACAGGCATTACATTATAATGTTAACCTCAGCACCCAGGCCTCTGGCCAGCTACTCCATTTGTAGCATGTTCATTT
TTTATTGTCTGACAAGAGAGTATGTACTGTTGTGAAATGACTCAGTTTTCAATGAGTGTGTTATCTCCCTCTCTCCATCT
CAGGTGTTGTCTTATTGGACAAGACACAGGGCTATTGGTTGGTCCACAGCACCCCTCATTTCCCCCCTGTAAAGGAAATA
GGGCAATATTCCTACCCAGGTAGTGGTGTACAGAACGGACAGAACTTCATCTGTGTGACCTACCCACTGGAACGCTTTCA
GGCCATTGGTGAGACTGTAATCCTGTAAAAGTCATTTGGAATACTATAAATTTACCTTAGAATTCTGAAAAAGTATCTTG
GATTATCCACCCTGTTATAATGCATGAAACTGTCAATTGTTTCATGAAGACACAAATGTGTTCTGTTTCATGCTGAGACA
GACTGTCTGTGCGTACAACCCGAGCCGACAAGGTGGAGAATCTGTTGACGTGCCCTTGAGCAAGGCACTTAACCCTAATA
TTCTCCAGGGGTGCCGTACTACTATTGCTGACCCTACAAACTAAGTGCTATGTCATAGTCATTCTCCACAGTAATAGTGG
GTCTCTGATGATAAAGTACTGCTGCCCCTATCACTGAAAAACCTGCATGCAATGAACTTAAAAATGACAATTAACTCATT
ATTACGCAACAGTTAATAAAGCCACATTAATGTTGACACAACAATTGTGAACCAAAAATAACATTTGCATAAACAATTTA
GGTCATTAGCATTTTAGCATATTGTAGTAGCAAGTGCCACTACAACCTGGCTAGGAAATGGTGCTGTTACCATAGTGACA
GATGGGGTTATCCTCTATGGCACTGTAACCATAGAGACCATACTCCTCCCGCTGTGAGATGTTTGCTCTATAAGTTTGAT
GTCTCTCGTGGAGTTATACTGAACAAAAATATAAACACAACATACAACATGTTTGGTGAGTATGCAGGCCATGGAAGAAC
TGGGACATTTTCAGCTTCCAGGAATTGTTTACAGATCCTTGTGACATGTGGCCGTGCATTATCATGGTGAAACATGAGGT
GATGGCGACGGATGAATGGCATGACAATCGGCCTCAGGATCCCATTGTTGGTATCTCTGTGCATTCAAATTGCCATTGTT
AAAATGCAATTGTGTTTGTTGTCCGTAGCTTATGCCTGCCGATACCATAACCCCACCGCCACCATGGGACACTATGTTCA
CAACGTTGACATCAGCAAACCGCTCGTCCACACAACGCCATACATGTGGTTTGCAGTTGTGAGGCTGGTTGGACGTAATG
CCAAATTCTCTAAAATTACGTTGGATGCAGCTTATGGTAGAGAAATGAACATTCAGTTATCTGGCAACAGCTTTGGTGGA
CATTCCTGCAGTCAGCATGCCAAATGCACACGCCCTCAAAACTTGAGACATTTGTGGCATTTTGTTGTGTGACAAAAGAG
AGGCATTTTATTGTCCCCAGCACAAGGTGCACCTGTGCAATGATCGTGCTGTTTAATCAGATTCTTAATATGCCACACCT
GTCAGGTGGATGGATTATCTTGGCAAAGGAGAAATGCTCACTAACAGGGATGTAAACAAATTTGTGCACAAAATTTGAGA
TTGAAAAAGCTTTTTGTGCATATGGAACATTTCTGGGGTCTTTTATTTCAGCTCATGAAACATGGGACCAACACTTCACG
TGTGTGTTTATATTTTTGTTCAGCTTAAGATGAGTGTCATTAATTGTGATGGGAGTTCTCAGTGGAGGCCAACTTCAATC
GTCCTCAAAGCATTGTCTTGTCTTAAAACATTTTCGATCTCAGCCACAGTCAGTGGTCTTGTATGTACAGGCGTTTGTCT
GCTACTCTGTATGAAAAATGTGAAAAGTCATCATACTAATAAGTCATGTTCTAGATTAAGATAGTACTTGTCTTGGAACT
TGGTGCCTATTTTTTTATTTTATTTAACCTTTATTTAACTAGGCAAGTCAGTTAAGAACAAATTCTTATTTACAATGACT
GCCAAACCCGGACGACGCTATTGAATCACACAGACAGACAAGACAGACAGATTGATCAGTCAATTCATCATTATTTGCTT
TACTTTTGACACAATTCCTTGATTGAAATGTTTGAAGTGTTCAGTTGAAGTGTTCAGTTGACATGTATTATGTTTTAAAT
TGCAGCGGAGCAGTTGCAGATCAACCAGCCCAATGTGTATGACTGTGGTGTCCCTGCTTCCTTGGCCTCTCTGGTGCCCG
CACTGGCTGACGTCTGTAACAACAGCCAAGGGCAGATGGCCTATAGCAGCTTACCGCACACCCAACCTGCATCCAATCGC
AGTGTGCCTCTCACCTCATTGGGTGGAACCGAGTTCATCAGCTTTGCCAAGGGGAGATCATTCAATAATGGTAAGGAACA
ATTTTGAAACTATTAATTTGTCATTTTTTTGTACTGTAAGAATGACTATTCATGTCATTACCGTTTTGTCATTGTGATTG
ACCCAACATTTAACCACAATATCCCCATTTCCCCTCCTCCTGTCTCCCATTAGATCTGTACCACTCCTGGGTGGCTCCCT
CCCTCCAGTCAGACCTCCTAGTCCAGTTCTGGATCCGCTCCACTGGCATCCTGCCCTCTGACTGCTCCCTGGGCTGGAAG
GTCCTGGACATCCAGCTTATCTCCCCAGGCGGCAGGATCACCTTCAAGGCCTCCAACGACCACTCCAAGTGGGCCGTGAG
TACCACCGGGGGTGGGTTGGATGGAGCTGCTGGCTGGGTGTGTGTGGGCGACATCAACCGCAACGAGGCTGAGGAGAAGA
GAGGGGGCGGGACGGTGTGTCTGCAGGACGCCACGGTGTGGAAGGCCTATCGGACAGCGGCGCTGGAGTGTGAGATGTGC
GGGGGTGGGACTAGCGGGTGTGATGTGGGCGTAGAGGATGCAAGGCACTACCAGGGAGATATGAATCAGTAAGCATCAGT
GTGGCACCAGTGTAAAGTTACTAGTATGCACACAGTTCACTGAGCAGCTAGGTTTATTAGCATTATTGAGCTCCGTGAAC
AAGGTACCCCATAGAATGTTGATTAATAAGGTACCGGCATTAAATTCACTTTTTTTTATGGCAGTTTTGGAGTGTTTCAT
AAATTGCCATGAATAGAAACATACTTGAAAGGGAAGTGGCACTCCTATAATCATTAAACAATGAGCATTTTGATACATTC
GATGTAGTTCTATAAGTTGCTAATGACTGTAGGCAATGTTGATTGTGATCTCAGGTATGTTTTTAGACATTTTATATGTC
TAAAAACCTTTTTTTTATGTGACAAATGATTAAAAAAAATGATTGTTAAAGTATGTGTAATGTTTTTTCTTTAATTCTCTT
TTACATTTTAGTCAAACGTTTGGTGACTTTTTCAGCTTTTAGCTTGAAACAGGCAAAGATACTGACTGTCACCTGTCCAA
ATAAAGAAAGTACTGTTGTTGCGGGGTGCACAAATATTTTGCATAATGTTCTTCATTAAACTTCCTCATCCACCAACAAG
CTAGATACTAAACCACAGTCTGAATCTTTTAGATGTACATCTTTGTACATTTATTTGGTAAGAATAGACACAGCAGAATT
ATAGCACACATCCGGGGAGTTGCATAGAAATTCCTCATATTGCCGTCACATCAAAATGCATTCATAGAAAAAGGAAATGT
ATCACACAAAAACAAATGGGGCATATGTAGTGCATAAACATGCCAGCTTATACAGCACTGTGAATGTACATCATATGTAC
TGTACATTCCAATGCATACATCACAGAGGCTTCATGTCAAATGGCACCTTATTAAGCGCACTACTTTTGACCAGGGTCCA
TGGGGCTCTGGTGAAAAGTAACACTCTATATAGGGAATAGGGTGCCATTTGGGACACTTTAAGTGGCTCAAGCACCAATG
AGAGAAAGGCACTAAACAATCAGTAGCTTCGCCTGGCGCTAACACTTCGTCAAATTACGTTACCCAGAATCCTCTCTCCT
CTCCCTGGAACTACCGTGTCTATGCTTCAGCCTTAGTAGAGACTTCAACAGCACTGGGTAGTATGACCAGAGTTCACAAG

AGTTCATCAGTTAGGGCCTTCCACAATCTGTTCCCCATGCGCCCTTATGCATACAGGTTTTCCTTCCTACTGGTCTCATC
CACATGGGATAGAAGCAGAGCCTTCTGCAAAACCCTCTACATTCAGTTCCTGGACTAAAGCCATTGCATAATGAAACCTT
ATAGCTGTCAGATCTCTATCAAACGTCAGAGAACATAACAATTAAACATCATTCAGTGATTGGTGAGAATAACTCACTAA
CCTGGCATGGGTCTTTGAGGACTGACTTTGGGAGCACTGGTTGAAGTACACTTCAGACAGACTTCCATTCCAACTAGCAG
CTAAACCCATTCAGAGACCAGCAATAAAGATTAGCTTATGCTGTATTGAAAAGTTTAGCATTAAGGTGCAATGGTAGACA
TCTTTTCAATACGCACAACATAGGTTAAGAGTTTGTGGGTTGATGTTGTTTTGCCTAATGGTTAGTACTCAAAATAGGAC
TTCTATATGGGAGTGGCAAGGCTGTATATGTATATGTGTTGTGTCTTACATAAGATACTACCGGAGCTATCATTCAATAT
GTATGGCACAAAACCCAAGTGAAATGATCATGTTTATCTTTGTAACGTCTTGGCCCATTTGAAATGGTTGCAATTTGGGG
CAATTTCATACATTTCCTGTTACTTCAAAATGAATAACATGTCATTCTGACACTTGTTGATTGCTCCTTGTGAACGTAAA
CACAATCATGACATCGTCAACACATAAAAAACATATGCATCCACAAAGTACATAGTTAAAACATTTTGATGAGCCCCCGT
GCTTTGCTATAATGAGAGGCTTCAGTGACTCTGTCTGTCACTTATTCAGATGCATTATGTCATAATGTCAGCCCAGTGCT
CTCCCCACCATTCATATTCTTCAAACGCCTTGCAATACTTAATATGTTGTCATGCTGCTTGTATCCACTCTCTATGGAGA
AATGGTCAACTTTGTTGTGGTCATGGTCAATTCAAGGAGGTCATATCGCCCAAATACTTTGATTTAATGAAGTTCTTTAC
TATCAACTCATCTCTATGATGAAACCTGAGGCACATTCCTCAAATACTCAAGATGATTTGGATTCCTCAGTTGCTCTTGG
TTACAAAGCTATTCCATTTGGTTAAATAGGAAATTAGATGCCAATGGTCAGAGAGACTTAAGGAACAGCACCATTCACCA
TCAGTTAAAGATTTGCACTGGTCTTGTTCTCGGTGTATATTTGAAGTAGTATTTATTGCCAACGAGAGAGACATGCACCC
TTCTACACTAATGGACTTCCCTGGTGTACACAAACAGGCAGCATATTTATTGAAATGTCCCAGGTAAGAACATGTTCATC
AAACAAGTTCAGACAGGTTTCAGGATACATTTTGCTTGAAACTCGGTCAGAATGTGTTTCCACTTTGGAAAAAGATTAGG
AAAGACAAGGCCACATCTCACTGAGATATCTGCCTGCATTCTTATCGTGACCTGTCCTTGAGAAGAACTAGCTACATTAT
GTCTATTTGGGAAAGAGTTGTTGTCAAAAAAACAGTCTCCGCCCCTTGGCTTTGATTCGAGGAAAGGTGGCACTGGTTTC
TGTGCTGATTGGTTGAGAGCGAAAAGGGGTGTTGCCTCCTTGTCTGCACAGCAGGAAGTCCACACTAGAGGAAGTCATGG
CATAGAAGACGTTTCCTGTGGCAGGGATGACCAACTCTACAGGGCAGAACAAGAAAAGTCATCACTACAATCAATATGAA
CAGAGAACTGTTAAAGTGACTTCGCATGGTCGGGTTGAGAAATGTCTTTACCTTTTCCCTCGGGCAATAGTTGGATGAGC
TCATATCTGGGTGCCTGTTTGGGGTCCTGACTGTGTTTTTCTAAGGCAGAACTGATCACAGTGGGCGTCTTGTCATTGCT
TGTCACCTGAGGAAACAGAGAAAAAGAACAACAATACCAGACAGCTTGACTCCTAGTTATAATATCCTGTGTACAACCTG
AGGAAATATTTTGGATTACTATCATTAACGGATATTTGTGGATTAAAGTAAATAGTCATGAGTGCAGGGTGCAGTGCAGT
GTATAATTTGATACCAAAAAAGGCAAATCTCTAAAAATATGCCATTATCTCGGCTCAGTACCAGTATGCTTCTGTACATA
TTCCCATCTTGTAAATCCATCCGTATCCTGACAATCCGCATTTCAGGCCCAGACCCTTTGTTGTTATTGGTGGGATTGTT
GCCACAGGAGACTGATCGACGATGTGATTTGACGGGAGTGGATGGTGTCAATGCAGACGGGATGGGGTCATTGGTGGGAT
GGGAGGAGTCAACATCCAGACAGGAAACTGAGGGGGATTTCATATGCTGAAATGATAATAGTGATGAAAATAATTTAAAA
AACATACTTACTGATATGACAAGATAAAAAGTGGCTGTTTCGTGAAGGCCAAAGCAAATAAAAAGACACAGAAGAGAATT
AAAAGATCAAATATTTTCCTCTTTTAAAAGTGCTCTTCTGTATGTTTTTACGACATGCATTTGTTTTAAAACAATCATAT
TTGATTACTAGGAAACAGCAATAGTTACTTAGCAATAACTACGAGTATTTGTACCTGAATAGTAATTACACGCTCTGCAA
TTCTAGCTGGCAATGCAAATATATTTTTGGGAAAGTACGTAAAATAGCTGTAAATATTAGGTCTTATTTTGTAATTACCT
TAGTGAATTTGGATAGAAAGTGACAGACGGGGGAGGGGAAGTCAAAATGACTGTCACTGTCAGCAACAAGGCTGGTCCTG
GAGGAATTCAGGCTGTAAGGAGGTGAGAGAATCATATCATGTATGTTCATCATGCACCGTAATCTGCATGACAACAACCT
ACAAAACATCTTACTCAACCCCTGAGAAATACATAGTAAATGTCCAAGTTTGGTTTTCAGCTCTTTCCAATGTTGTATGT
TAATTTACCAGCTCTTGTACTGTGTGAGGATATTTTTTATGGAAAAAAGTACGGAGTACTTACCCCCAGACCCCCCCAAG
TCAATTCTCCACAACTGTTTTCTTATGGCCACGTTACTACAAATTTGACAGTCAAATATGTGGACACCCCTTCAAATTAG
TGGATTCGACTATTTCAGCCACACCCGTTGCTGACAGATGTATAAAATCGAGCACACTGCCATGCGATCTCCATAGACAA
ACATTGGCAGTAGAATGGCCTTACTGAAGAGCTTAGTGACTTTCAATGTGACACCGTCATAGGATGTCACCTTTACAAAT
TTCTGTCCTGCTACAGCTGTAAGTACTGTTATTGTGAAGTGGAAACATCTAGGAGCAACAACGGCTCAGCCACGAAGTGG
TAGGCCACACAAGGTCACAGAACGGGACTGCCTAGTGCTGAAAAAAGCGAGGTCTATATAGAAATGGTTTGTCGAGATCG
GTGTGGAAGAACTTGACTGGCCTGCACAGAGCCCTGACCTCAACCCCATTGAACAGCTTTGGGATGAATTAGAACACCAA
CTGCGAGCCAGGCCTAACCGCCCAACATCAGTGCCCGACCTTACTAATGCTCTTGTGGCTGAATGGAAGCGAGTCCCCGC
AGCAATGTTTCCACATCTAGTGGAAAGCCTTCCTAGAAGAGTGGAGGATGTTATAGCAGCAAAGGCGGGACCAACTTCAT
ATTAATGCCCGTGATTTTGGAATGAGATGTTTGACATGCAGGTGTCCACATACTTTTGGGTCATGTAGTGTAGTATAGTT
CACAGAAAATGCTGACTGGCCATTCTTTTACAATAGTACTGAGCCCATATAACCAATGTATTTCACTATACATAGTGAAA
GGTATCTGCAGTGAATACTACCGGTATGTTGTGTGTAGAGTGTAATAATAGGCCTCATTACTTTGGGCACCGTGTGATGA
TCACTGTAGGGTTCAGGGTACGACCAGAGCTCGGTTCGCACGGCACCTCAATTTCATTGGACAGTCTGTAACTGCAGACA
TCATGTAATGTGATTAGCGATCATCAGAGTTGTAGTGTTCCGTTTGAAATAACATGTATGTACAAACTTGAATGGTTTGA
TTGTAAAATGAATAAAACCAAAAGATAATCACGGCTTACCTTTCTTCTTCATTTAGCGAGGGTACACTTTGGTACCACTG
TAGGAATGCCTCGTCAGTGATGAAAACACCGTTTTTACAGGAGGACTGCAGTAGTCGGATCTGAGCTAAAACCTCAAACT
CCTGCAAAGAGAAAAACCATTCATTATGAACATCAAATGACTCTCCAGTACTAATGGCACATTATCAGTTGAAATGCTAG
CAGTTAAACATGAATATGTGCATGGAGGCACTGCTCCAGAATTCAATACACTGCCACACATTTAATGAGAGGCACAACAG
AAAAGCAATTTATGAAGACCATCTGCTATGTTGAGCCAGAGGCTAATGAATTGGTCTGGATCCTGGAATAACTCAACATG
TGGACAATGGGTGATGAGGAATTTAAACAGCAGCAGTGCAAAGGCCCTGACCGCATCCCTTACTCGTCTCCTCTTGTCAA
AGTTGACGTAGCCGTTCTGAAATGGAGGAACGGGATATTATGTCACTGTCAATGCAACTTTTTGGTAAACCAACTACAAG
TTCACCACCAGGGCTTCTGACCAACTTCCTGTTGATGGACACATGATGGGGGATAAATTACTGTATATGAAGGGACTCAC
CTCTAGTCTGTCTTTGACGGCCGTGTCCAGCATGGTGAGGTCTGTGAGGAAGATCCCCAGGTAGGGTACTGTGCCCTGGG
CACTGGACTGTGTGAGAGAGGGGAGGGGTAGAGAATAATGAAAACCAAAAACAGAGGCATGGGGCTTCAGGCTTCCCAGA
CACAGATTAAGCCTACTCAGTGCATGCTCAACGGAGAAACTTAGTTAAGACCGGTGAATGTACTGTATATCAAGTTGTCC
TTACTTAAACATCACAGATTCCTATATTGAAGAACTACCAGCATTGCATTATGTTACATCAGCAAAGGGTTATTTGATGC
CCTTTGTACAGTACATTCCATGCTTCTTCCTTCAAGGCAAAATGATAATGAGCTTTGTGAGTGACTTCAAACTCGCTTAT
AAGAGCACTTACTCCTATCTGTCTCCTGTTGGTCATCTTGCCGTCCAGGTTGGCGAACTTCGAAGTGCCCTCCTGCAAAA
CACAGACAAACATCTAAGCATGATAATCTGGAACACAATGTAGAATTTAGAACCATGGCTATGTGCTCTAAAACCCTGTG
TAGTAACTGGATAGTAACTGGGTAGTAACTGGGTAGTAGGCCCTCAATTCCAGACAAACTGAGAGGGTAAGGAAGCAGCT
TACTTGGTAAATTATAAAAGCACAGCAGAAGGCAGTGTGATATTTTTCCAAAATCAAAGCCTGGAGTGAAGCTTTCAAAT
GAAAGCCCCGTCATATGTTTCGATGAATTCCTGTGTGGATGCCTTGGTGAAATTGCAGCTAATGTAAGGTATCGATGCAT

GCACAACGTATAAACAGTGAATGAAAATGCTGCTAATCCTACCGATTTCCAGACCCCCGTCAGAAACTCACAATGCATCC
TAAACACAATACATGTTTAGAAACGGGTGAATTTAGATTGACAGGACGTTGACATTGAGAGGCTGTAACCCCCAGATTAA
GCCTTCCTCTGGGTTAAAAGGCATGATTCATGATTCAAAGTGATTTTTAGCCCTGGAGTAGTACTTACTCTGGATCTGTG
TAGTGCAACCGCTCTTGAATGTATATGAATTTGAGACGCGTAAACACGATTGGTGTGATCTGTGACGACACTTCACATGA
GACCTGTCCTTAGTTCATATTGCTTGTATTATGGGATGTCTGTCAGTGCACTGTGTGTGTCTGTGTCTCCGTGTGTGTGT
GTGTGTGTGTGTGTGTGTGTGTGTGTGTGTGTGTGTGTGTGTGTGTGTGTGTGTGTGTGTGTGTGTGTGTGTGTGTGTGT
GTACATGCGTATGTACATACAGTACATGCGTGTTGTGTGTGTGTGTGTGTGTGTGCGTACGTGTGTGCAGTAACTCTCAA
TCACTGTGACTCACCTCTTTCAGCAGCTCCCTGCTCTGGGAGTAGTTGTCCTTCTCAGAGAAAATGTTGGACAGCTCCTC
GTATCTCCTCACTGCTTCCCTGTCAGACAGAGTCACATGACAACAACAATGAGCCATGCACCCAAAGAAGGTATTAGGGG
CATGGACTGACATTTCTCTGCTGTCTACTAGACCTTACTATAATCCACGTCCAAACGAATCAGCTAGCCATGATTAGTTT
AGCTACGATTGGGGAAATTATAACAAGATGAGATGATCAACTGCTGTAGTGTTTTGGAACGTTATATATGTTGACTANNN
NNNNNNNNNNNNNNNNNNNNNNNNNNNNNNNNNNNNNNNNNNNNNNNNNNNNNNNNNNNNNNNNNNNNNNNNNNNNNNNN
NNNNNNNNNNNNNNNNNNNNNNNNNNNNNNNNNNNNNNNNNNNNNNNNNNNNNNNNNNNNNNNNNNNNNNNNNNNNNNNN
NNNNNNNNNNNNNNNNNNNNNNNNNNNNNNNNNNNNNNNNNNNNNNNNNNNNNNNNNNNNNNNNNNNNNNNNNNNNNNNN
NNNNNNNNNNNNNNNNNNNNNNNNNNNNNNNNNNNNNNNNNNNNNNNNNNNNNNNNNNNNNNNNNNNNNNNNNNNNNNNN
NNNNNNNNNNNNNNNNNNNNNNNNNNNNNNNNNNNNNNNNNNNNNNNNNNNNNNNNNNNNNNNNNNNNNNNNNNNNNNNN
NNNNNNNNNNNNNNNNNNNNNNNNNNNNNNNNNNNNNNNNNNNNNNNNNNNNNNNNNNNNNNNNNNNNNNNNNNNNNNNN
NNNNNNNNNNNNNNNNNNNNNNNNNNNNNNNNNNNNNNNNNNNNNNNNNNNNNNNNNNNNNNNNNNNNNNNNNNNNNNNN
NNNNNNNNNNNNNNNNNNNNNNNNNNNNNNNNNNNNNNNNNNNNNNNNNNNNNNNNNNNNNNNNNNNNNNNNNNNNNNNN
NNNNNNNNNNNNNNNNNNNNNNNNNNNNNNNNNNNNNNNNNNNNNNNNNNNNNNNNNNNNNNNNNNNNNNNNNNNNNNNN
NNNNNNNNNNNNNNNNNNNNNNNNNNNNNNNNNNNNNNNNNNNNNNNNNNNNNNNNNNNNNNNNNNNNNNNNNNNNNNNN
NNNNNNNNNNNNNNNNNNNNNNNNNNNNNNNNNNNNNNNNNNNNNNNNNNNNNNNNNNNNNNNNNNNNNNNNNNNNNNNN
NNNNNNNNNNNNNNNNNNNNNNNNNNNNNNNNNNNNNNNNNNNNNNNNNNNNNNNNNNNNNNNNNNNNNNNNNNNNNNNN
NNNNNNNNNNNNNNNNNNNNNNNNNNNNNNNNNNNNNNNNNNNNNNNNNNNNNNNNNNNNNNNNNNNNNNNNNNNNNNNN
NNNNNNNNNNNNNNNNNNNNNNNNNNNNNNNNNNNNNNNNNNNNNNNNNNNNNNNNNNNNNNNNNTGAGTGAGTGAGTGA
GTGAGAGTGAAGTGAGGGAGGGACCGTGATTGAGGGAATAAGAGGTAGACAGTGAGTAAAGATTGCGTGAGAGAGAGACA
GAGAAAGAGAGAGACACTGAGAGAGACACCAAGAGAGAGAGAGACAGAGAGAGAGAGAGACACCAAGAGAGACAGTCAGA
AAGAGAGAGAGAGAGAGAGAGAGAGAGAGAGAGAGAGACAGCAAGGCATAGCCAGAGAGACTGCAAGTGGCAAACATGTCCA
GTATGAGTAAGAAAGGGTCAGAGAGACATAACAATAGAAAAAGAGGAACACAATTAAGACTGTAGTAAAGTGCATGTTCC
CATTATCTATACAAGGTTAGGAGGAGTAATAATACTGTAGGGGTCAGAGGTTTAAACCCACCGTTTCTATCTTGGTGAGC
TGCTCAGCGATCAGCCCAGCGGGGAAGCCCAGGATATTGGTGGGATCAAACTTAGTGGGGTCAGCAGGAAGGCTGGGGGT
GGTCCTATCTAAAAGACAGAGATGATTCAATACTAATCCCAATGCATGTAGGTCTATATTAACTGATACAAATGAAAGGC
TTATTCAGTCGGGTTGGCAGTGGAAAGAGACATTAGGGTGGTTGTTACAGAAGCAGGTTCCACCAGATTTGGATGGTAGA
CAGCAGACAGTGGGAATATAACGCTAAAGATCATTACAGAAAACTACTGGAAACAATTTGGAGAAGAGTTCTCTCCATAG
ATATCTACGGTTATGCCACCCACAAATCCTCATATAACCTCAGGTTGGCAGTGCCAGGACTGTCATAAACCCAGAGTGTA
TAGTGACCTGAACTAGAGTCAGGCAGTAGGGCCTGTTCACTGAGCTCCTCTGCCAGTCTGAGGAGGCGACCCCTGATCTC
TGTCCCAGAGGGGTCGGAGGGCAGCAGGGGGGCCAGGCGCAGCAGTAGACCAGGCTCTCCCAGAGAGCGGAAGTCCTCAG
GATACTCCGACAGCCACATACTGAACACCATACACACCGCTCTGGGAGAGGGAGAAAATATATACAAATGCATGAAGTGC
GCACACACACACACACACACACACACACACACACACACACACACACACACACACACACACACACACACACACACACACAC
ACACACACACACACACACACACACACACACACACACACACACACACACACACACAGGGGTGATATAGTATTAAACTCACC
TGTTGAAGGGTTGCCTAGTCTGACTCTTTGTACTTTCTCCTGGTGGATGTTGCAATCTGGAGAAAGAGGGAAGAAAAGAG
AAAAAAAAGCTGAATAAGAGACGATTTCTTCAGGGAAACGCAGAAGCATCACAACGTCTCCTTTCCTTCCCTTTCTACAG
CACTTCAAAAATGTTTTGTAACACTACATACAACCGGTGTGCAATGCATTGCTTCTGTCTTCCCTACCTGTCGATGAGGA
TGTCCAGCACCCTCTTTGTTGAAGTAAAGGAGCGATAGGTGGAGAGGAAGATGGTGATGAAGGAGGAGTCTCCCATGGCA
AAGGAGTGGAGGAGGTGCTGCACCAGTTTCTCCTCTGTTCCCGCTTTCACACACTGTGAACGAGAGCTGGACTGGAGAGA
GGGAGGGAGAGAGAGACAGAGAGAGAGAGAGAGAGAGAGAGAGAGAGAGAGAGAGAGAGAGAGAGAGAGAGAGAGAGAGA
GAGAGAGAGAGAGAAAGCAAGAGAGAGAGAGAGAGAGAGAGAGAGCGAGAGAGAGAGAGAGAGAAAGAGAGAGAAAGCGA
GAGAGAGAGAGAGAGAGACAGAGAGAGAGAGAGAAAGAGAGAANNNNNNNNNNNNNNNNNNNNNNNNNNNNNNNNNNNNN
NNNNNNNNNNNNNNNNNNNNNNNNNNNNNNNNNNNNNNNNNNNNNNNNNNNNNNNNNNNNNNNNNNNNNNNNNNNNNNNN
NNNNNNNNNNNNNNNNNNNNNNNNNNNNNNNNNNNNNNNNNNNNNNNNNNNNNNNNNNNNNNNNNNNNNNNNNNNNNNNN
NNNNNNNNNNNNNNNNNNNNNNNNNNNNNNNNNNNNNNNNNNNNNNNNNNNNNNNNNNNNNNNNNNNNNNNNNNNNNNNN
NNNNNNNNNNNNNNNNNNNNNNNNNNNNNNNNNNNNNNNNNNNNNNNNNNNNNNNNNNNNNNNNNNNNNNNNNNNNNNNN
NNNNNNNNNNNNNNNNNNNNNNNNNNNNNNNNNNNNNNNNNNNNNNNNNNNNNNNNNNNNNNNNNNNNNNNNNNNNNNNN
NNNNNNNNNNNNNNNNNNNNNNNNNNNNNNNNNNNNNNNNNNNNNNNNNNNNNNNNNNNNNNNNNNNNNNNNNNNNNNNN
NNNNNNNNNNNNNNNNNNNNNNNNNNNNNNNNNNNNNNNNNNNNNNNNNNNNNNNNNNNNNNNNNNNNNNNNNNNNNNNN
NNNNNNNNNNNNNNNNNNNNNNNNNNNNNNNNNNNNNNNNNNNNNNNNNNNNNNNNNNNNNNNNNNNNNNNNNNNNNNNN
NNNNNNNNNNNNNNNNNNNNNNNNNNNNNNNNNNNNNNNNNNNNNNNNNNNNNNNNNNNNNNNNNNNNNNNNNNNNNNNN
NNNNNNNNNNTATTACATTGACACCCCCACCTTTGTTTTTACACTGCTGCTACTCGCTGTTTATTATCTATGCATAGTCA
TTTTACCCCTGTCTACATGTACAAATTACCTCGACTAACCTGTACCCCCGCACATTTACTTGGTACCAGCACCCCCTGTA
TATAGCCTCGTTATTGTTATTTTATTGTGTTACTTTTTATTAAAAAAAATTTACTTTTGTTTATTTAGTATATATTTTCT
TAACTCTATTTCCTGAACTGCATTGTTGTAAATAAGCATTTCACGGTAAGGTCTACACCTGGTGTATTCGACAGTGGTGG
AAAAGTACCCAACTGTCATACTTGAGTAAAAGTAAAGATACCTTAATAGAAAATGACTCAAGTAAAAGTAAAAGTCACCC
AGTAAAATTTTACTTGAGTAAAAGTCTAAAAGTATTTGGTTTAAAATATACTTAAGTATCAAAAGTAAAAGAATACATCA
TTTCAAATTCCTTATATTAAGCAAACCAGATGGCAGCATTTTCTTGTTTCTTTTATTTACGGAAAGCCATGGGCACATTC
CAACATTCAGAAATCATTTACAAACAAAGTATGTGTGTTTGGTGACTCCGCCAGATCAGAGGCAGTAGAGATGACCTGGG
ATGTTCTCTGTTTAGTGAGTCCGCCAGATCAGAGGCAGTAGGGATGACCAGGGATGTTCTCTGTTTAGTGAGTCCTCCAG
ATCAGAGGCAGTAGGGATGACCAGGGATGTTCTCTGTTTAGTGAGTCCTCCAGATCAGAGGCAGTAGGGATGACCAGGGA
TGTTCTCTGTTTAGTGAGTCCTCCAGATCAGAGGCAGTAGGGATGACCAGGGATGTTCTCTGTTTAGTGAGTCCTCCAGA
TCAGAGGCAGCAGGGATGACCAGGGATATTCGGTTGATAAATGCGTCAATTGGACTATTTTCCTGTCCTTCTAAGCATTC

AAAATGTAACGAGTACTTTTGGGTATCAAGGAAAATGTATGGAGTAAAAAGTACATTATTTTCTTAAGGAATGTAGTGAA
GTAAAAGTAAAAGAAATCAAAAATATAAATAGTAAAGTAAAATAAAATACAGATACCCCCAAAAAAACGACTTAAGTAGTA
CTTTAAAGTATTTTTACTGAAGTACTTTACACCACTGGTATTCGGTGCATGTGACAAATAACATTTGATTTCAACTCTAG
CTAGCATAGACTACTAGCCTTTGTTAGCCTTTGTAGCTCTGTTTACCGGCACCTATAAACGCACTGTCTGCACACATATG
ACTATGCAACAGACAGAAGGAAGGTTGAGTAAATATATGGACTGCAAACCAGTTGAAGAGGATTTCTAGCCTTGGTTTAT
TCAGCTCAATGTACACAGAAAGAACATAGAACACAAGTCTTTTGGAGATGATTATTTGTGTGTTGTCATTAATGCTAGCC
TACAACAGGTCACGAAAAACAGAGAAGCCTTTGTACTGTAGGCCTAAAGTAGCACTGTGCATTCAACTAATCAGGGGGTT
ATGAACTAGGCTAACAGCACAGTCGCTGTTTCTAAGGGACTGCAGTTGTAAAAGTGAGGCTAGGTCTAGGTCTACTGTAG
GTCTACTGTAGGCCTCCTTTTCCATGGTATTGTGTTTTTGTCTCAAGCAGTAGGTCTAAGGTTAAGTTTACCTACAACTC
TTCCTGGTACAGTTAGCTTCAGTATAACCACTTAATTGGGACCATCCACATCAACCTCAACCAACCACAATCCCCACATC
TCAGTCTGTGAGTGGCGATGGAACAGAGGCACCAATGAGGATGTCTAATATTGTGGCATGTAACAACCCCTACAATGTTT
CTGTTCTACCAAATGCACAAAATCTGTGAATCTGTTGGAGTGCATTTTCCCCAACAATCAATACAAGGACTTGGCTGCTC
CGCTGTAAGGAAAAGCTGGCTCAGGGAGCAGATGATAATAATTATGGAAGTAAGTGGCTGAGGTTCATTCCATGAGAAAC
ATCTTGATAAGGAAATTTGCTTGACTCTCCTCAGATACTGTATCATTCAAATCTCCCATTTTGTCTGCTCATGTTTACAG
ATTTTGTCCAACGTTTTCTTTTGTTCGCCCACTTTTTCCATCGTTGCTAGTTTTAACTCAATGTTTATACTCTTTATGTG
GAACCTCTCTCATGATTCAACATGCTCCTTGTCTCTATATTTCCATATTTCTCTCCATAGAGGAAGTACATAATGAGCG
GAGACAAACACAGGAAGCAGGACGTGCTGTCGTGACGAGCTGCTATTGTTTCTCTTTCTCTTTCCCTCACAACCCGGCCT
CCTCTGACCACTACGATAGGCATTGGTGCAGGGGGGAAAGAGCCTCACAATCTCACCACCTTCACCCCCTGTCTTTACAC
AGTCTCAAGTTCTACTTCCGTCACTTGTTTGTCAACATCAGACATTTTGGGCTTTGTCCCTTTAAATGAGTATCTATGGT
TGTTATACAGTATGAACAAATAGGTACGTGAGCAAATGGGAGGCTATTGTTATGTGAAAACGTAATCCCACCACTGAAAC
CAATATTGTTGACTACTTTACACAGCAATACAATTGGGGCTCGTAGATATATCCTCTGAAGTTTTATTTAAAGATGCCTA
TTGTTTGTTTTTTTTAGCTCCCTTGCTCTCCCTCTCAGGCTCCCACCCCTTCCTCCCATCTTTCCCCGCTCTTCTTCTCA
CGCTTTCATCTGTGCATCCCATGGTACCCTCTCTACTCTTTAAAGTGAGCCCTGGTGGTATGCCAGAGCTGCTCTATTTC
CTTATATCAAAGTCTTATCTGAAATCTCTTTTAAATCTTTGGTTGATAAGATTGTTTTAACTANNNNNNNNNNNNNNNNNN
NNNNNNNNNNNNNNNNNNNNNNNNNNNNNNNNNNNNNNNNNNNNNNNNNNNNNNNNNNNNNNNNNNNNNNNNNNNNNNNN
NNNAGCCCCCGGGGAGTTTCTTGAGACTGCATGCTAAGTGAAGTCAAAGCCAATGAGACGAGCATCATGTGCACTACGAA
CTAAAGCCAAGTAGCAAGTGTATGAGAGTTAGTCAATGGGAAATGATGGAGTTAATTGATATGTAATTAAGAGACAATCA
ACTAAATGGCAATCACGCTTTCATCTTAAATTGACCTAATGCGGTCTGCAATTATTGGGTGAATATAATGATTTGTCAAA
TCAAAGTGATGAAATGACATTGGCCTAAGTGTCATAAATGAAGTAGTATAATACCTCACAATGGAAACAATTATCTGTGT
ACTTTAGGGTTGAAAAGGAGTAAAACCCAGACATCAGTGACAGAACAACAAGGCTGTGTGAGTAGAGTAGAATAACCCAG
AAATTGACTGCCACAACTAGTCTGATAAAACCTTTACAGCCTCTGTCTCAGCCAATGGAACATTAACAAAGGTTATACAT
CGTGAGAGAAACACGCGTGCTGTCAACCTGTCCAGGTTTGTACACTTTCAAAACAACTAACACAACCCTTTCTGTTTCAG
GCGAACTCAACTGGATATAAATAGTGAAGTATATTAGAAAATACATGTAATATGGTCATAAAAGTAATGCTCAAGAAAGC
AACATCTTAGCAAAAATGTTACAGTAGTCTACTTGGAAATCTCAAGACATGAGCATTTTATTCAATGCGTGGTGGTCAAA
CATCATATCATTCACTGGCATGTTTTGATGGAAAGCACCTCCTCTTTAGTGTCTGTCTTTGAAATCAAAATGACCAACCG
CATCTGCTGTTTACGCAAGGAACTGGTAGGAGAGCCCAGTCACCACGGTTTCACAGACCACTGCTCTAATACCCTAACCC
CCTACCCTGGTTGGTCTGAGGAATGGGATCAATTTTAAAGGGCAAGACTAAGAGGACAACCTGACTTAAAGCTGCAATAT
GTAATTTTGTGGGCGACCCGACCAAATTCACATAGAGTAAGTTATAGATCTGTTATTCTCATTGAAAGCAAGTCTAAAAT
GCTGTAGAGCTGTTCTATGTGAGCTATTTCTATGCTTCACTGTCTTCAGTTTCATTTGTTTTGTACACCAGCTTCAAACA
GCTGAAAATACAATATTTTGGGTTATTGAAAAGATTTTTCACAGCGGTTTAGATGGTGTAATGATTCTAATAGTTTGTTT
TTTCCCAAGCACTGCACAGCTGATTCAAATAATCAAAGCTTGATGGTGAGTTGGTTATTTGAATCAGCTATGAAGTGCTG
GGGCAAAAACCAAAACGTGCACCCGGGGAGGCAGGACTGAGTTTGGGAAACCCTGCTCTACACTATACTTGCTTGTTTTG
TCACATAAACTGAAAATAGGCAAACTATTAGAATTTTAGCAACCAGGAAATGGCAGAGCGATTTCTGCACAGTGCATCTT
TAAATTATATGTCACAGATGTGCTTCTGTATCAAATCAGCCATTTATTGGATTCACCATATCAGGTGAATTTCTTTTATA
TGAGACCACTGGAGAAAGTTTGCTATATGAGAGAACAAGAAACAGTGCCCCCCATAACTTTACAGGTATTAAAGTGCAAC
TGCACCTGTATTTCTCTGTCATCACAATGAAACTACTACTGCATTTTAATTGCTGTGAATGAGTTACTTACAGTACATGT
TCCTAATCAAGTTAATCAAATATAAAATCACTTCCAACCTCAGAGGAGTCCAGTCCAGTACTGTCAGTCACTTGCCCAGA
TTGGCTGCTGAGGGGAACATCGTCAGGCGCTAAAGGCCGGTAGCCAATGAGGGGTACATCCCTTGACTCCACCCCTGGCA
GGTCCATCCCACCTGTTCGCATACTACGAGGGATCATGGTGCCGACAGCTAGAGACACAAAATAAACAATAGGCACATAT
GGGGTTAATTGGTGATAATTTCAATTTTCAACTCAGAATATGTCATAGTTTATGGACATTAACCAACTCGGAAAGGCATA
GAAAAATAAATACAATAGTATATATACTGTACAATAAAAATAATTGTGTACCAAAGCTTTGGTGGTAGCAGTTCATTTGT
TTACATGATGATTTACATTTCACTGTGCCCTGTTCCAGCATTGAATTTGAATGCCAGCTCTTCCTTCCTTTTCCATTCTC
CCTCCCTGAGACATGGATTGCCATGATGATGGAAATCTAGAAAACACTGTTACACTGTAGGGCAGGAGGAGGATGTTGAC
ATGTACCACTATATATATATACTTAGAGAACGCAGATATTCATGTGATGATGCATTGCTGGTTCACATTAATGAAACTGTGT
CAGGACATTTTTTGCCAAGCAAGTGTGGTGTAGGCTACTGTATAGGAATGTACAGCATATGGATATGTCAGAAGACTTGC
TGTTCTTGTATGGTTAATGTCATTACTTCAGTAATGATGCTAAAGGTGTTTAAATAGGTGGAACAAACATTTTATGTTTC
ATCTTTATCTGTATAATTAAGGGTTTGCTATTGTTATTTTATTTTACTGCTGCTCTTTAATTATTTGTTACTTTATTTCT
TATTTTTGTAGGAATTTTTCTTAAAACTGCATTGTTGGTTAAGGGCTTGTCAGTAAGTATTTCACTGTAAGGTCTACCTG
TTGTATTCGGCGCATGTGACAAATACAATTTCATTTGATTTGGGCTACCTGTTATATATAGGTTTTTGTCTGTGAAATTA
AATACACTTTTCTACCCTACATGCACTGTTATACAATAGGCTTATATGGGACACCAATCAACTCAATCACATCATCTTTG
GAAACATGGTACTAAACTTTTAAGTGTATTTCAGTCTTAAGGGCATGCATATAATTAGGCCTAGTGTTATTAAAATATAT
ATATTGGTAATAGGAAAACATATCTGTAAATTGTGTGTATATACTGACTGTTAGTCAATAACTTCCATTCATGACATTGC
TGCCTTTTTGAGGAAGGCAACATTACATGTTGGTCTGCATGAGTGAGTCCACATTGTAACTAGGCTACAGATGGTCACAC
CCCAATTGGATATGTTCCCTTTCAATTCAACCAACCGATGGGTGGTGCTGTATTGATTTCTCAACAAAATTGTGTTCACC
TTCAATCATAGGAAACAAGACACTATAGGAATTAAACGGAATATGATGATAAAGTTTGCGTGTAAAGCCTTCGTTTCTTT
AGTAACCAAATACATAAGGTTTAGGCCTATTTATTGAAAGCCATATGATATTCAATGTCACGAGGGCCCTGCAATTAAAG
AGATGATGAATATACAAAGTGTCATTCATGGCTGTATCGTATGAACGAACTTACTGAATGTTGCGTGTCCAACTATGGTC
AGCTAAATTCCGCCAGCATTGGATATCTGTAGTGTCTGCCGCCGTGGAATATACGGTAACCGGTTAGTGGACGAATGTCG
CGCTCATCCGTTCCAGCACCGACGTCGGAAGAGAGTCTCTCTCCCCCTCTATCAGTATCTGTGTCCCCCTCTAGTTGCCT

GGCAAGCTTCTATCGGATGATAGCAGCACTGAGCACATTCGTGTGCAGCACACTCCCTCCCTGCTCTCTCTCTCTGTGCT
CTGACGGCGCAGTTCCTGTAGACTTGTGCTCAGCGCGTTTAACAACATAGCCTACACTTGTGTCGCTGGCTGAGAGAGGG
GGGAAAAGTGGTCATCTCGGAAATATAGTCCATGTGGACTGTGTGTGGTGTACAATATAAAAAATGTCAAGTCATCTATG
GGCTCTTAAAATTAGGGTTTAAGAACAAAACCAATAATTCCCTATGGAAATAGTCTATCTGCCACATTTTCTTTCCAATG
CCGACAGTAGGGGGAAACATTTCCAAAAGGAAATTGACCCGAACACTGCTGGAAGGCATTAGAGGGTGTCATGCCCACAG
GGGCTATAAGGGTATGTGCCCCCTCAGATTTGTCCTTTTCTTGTTTGTTTTTATTTTTCATGTGCACCGCATAGTAAAGA
TATGCTAGGCAGGAAGCTATATACAGTGCCTTGCGAAAGTATTCGGCCCCCTTGAACATTTCGAACTTTTGACACATTTC
AGGCTTCAAACATAAAGATATAAAACTGTAATTGTTTGTGAAGAATCAACAACAAGTGGGACACAATTATGAAGTGGAAC
GAAATTTATTGGATATTTCAAACTTTTTTAACAAATAAAAAACTGAAAAATTGGCCGTGCAAAATTATTCAGCCCCCTTA
AGTTAATACTTTGTAGCGCCACCTTTTGCTGCGATTACAGCTGTAAGTCGCTTGGGGTATGTCTCTATCAGTTTTGCACA
TCGAGAGACTGACATTTTTGCCCATTCCTCCTTGTAAAACAGCTCGAGCTCAGTGAGGTTGGATGAAGAGCGTTTGTGAA
CAGCAGTTTTCAGTTCTTTCCACAGATTCTCGATTGGATTCAGGTCTGGACTTTGACTTGGCCATTCTAACACCTGGATA
TGTTTATTTGTGAACCATTCCATTGTAGATTTTGCTTTATGTTTTGGATCATTGTCTTGTTGGAAGACAAATCTCCGTCC
CAGTCTCAGGTCTTTTGTAGACTCCATCAGGTTTTCTTCCAGAATGGTCCTGTATTTGGCTCCATCCATCTTCCCATCAA
TTTTAACCATCTTCCCTGCCCCTGCTGAAGAAAAGCAGGCCCAAACCATGATGCTGCCACCACCATGTTTGACAGTGGGG
ATGGTGTGTTCAGGGTGATGAGCTGTGTTGCTTTTACGCCAAACATAACGTTTTGCATTGTTGCCAAAAAGTTCGATTTT
GGTTTCATCTGACCAGAGCACCTTCTTCCACATGTTTGGTGTGTCTCCCAGGTGGCTAGTGGCAAACTTTAAACAACACT
TTTTATGGATATCTTTAAGAAATGGCTTTCTTCTTGCCACTCTTCCATAAAGGCCAGATTTGTGCAGTATACGACTGATT
GTTGTCCTATGGACAGAGTCTCCCACCTCAGCTGTAGATCTCTGCAGTTCATCCAGAGTGATCATGGGCCTCTTGGCTGC
ATCTCTGATCAGTCTTCCCTTGTATGAGCTGAAANNNNNNNNNNNNNNNNNNNNNNNNNNNNNNNNNNNNNNNNNNNNNN
NNNNNNNNNNNNNNNNNNNNNNNNNNNNNNNNNNNNNNNNNNNNNNNNNNNNNNNNNNNNNNNNNNNNNNNNNNNNNNNN
NNNNNNNNNNNNNNNNNNNNNNNNNNNNNNNNNNNNNNNNNNNNNNNNNNNNNNNNNNNNNNNNNNNNNNNNNNNNNNNN
NNNNNNNNNNNNNNNNNNNNNNNNNNNNNNNNNNNNNNNNNNNNNNNNNNNNNNNNNNNNNNNNNNNNNNNNNNNNNNNN
NNNNNNNNNNNNNNNNNNNNNNNNNNNNNNNNNNNNNNNNNNNNNNNNNNNNNNNNNNNNNNNNNNNNNNNNNNNNNNNN
NNNNNNNNNNNNNNNNNNNNNNNNNNNNNNNNNNNNNNNNNNNNNNNNNNNNNNNNNNNNNNNNNNNNNNNNNNNNNNNN
NNNNNNNNNNNNNNNNNNNNNNNNNNNNNNNNNNNNNNNNNNNNNNNNNNNNNNNNNNNNNNNNNNNNNNNNNNNNNNNN
NNNNNNNNNNNNNNNNNNNNNNNNNNNNNNNNNNNNNNNNNNNNNNNNNNNNNNNNNNNNNNNNNNNNNNNNNNNNNNNN
NNNNNNNNNNNNNNNNNNNNNNNNNNNNNNNNNNNNNNNNNNNNNNNNNNNNNNNNNNNNNNNNNNNNNNNNNNNNNNNN
NNNNNNNNNNNNNNNNNNNNNNNNNNNNNNNNNNNNNNNNNNNNNNNNNNNNNNNNNNNNNNNNNNNNNNNNNNNNNNNN
NNNNNNNNNNNNNNNNNNNNNNNNNNNNNNNNNNNNNNNNNNNNNNNNNNNNNNNNNNNNNNNNNNNNNNNNNNNNNNNN
NNNNNNNNNNNNNNNNNNNNNNNNNNNNNNNNNNNNNNNNNNNNNNNNNNNNNNNNNNNNNNNNNNNNNNNNNNNNNNNN
NNNNNNNNNNNNNNNNNNNNNNNNNNNNNNNNNNNNNNNNNNNNNNNNNNNNNNNNNNNNNNNNNNNNNNNNNNNNNNNN
NNNNNNNNNNNNNNNNNNNNNNNNNNNNNNNNNNNNNNNNNNNNNNNNNNNNNNNNNNNNNNNNNNNNNNNNNNNNNNNN
NNNNNNNNNNNNNNNNNNNNNNNNNNNNNNNNNNNNNNNNNNNNNNNNNNNNNNNNNNNNNNNNNNNNNNNNNNNNNNNN
NNNNNNNNNNNNNNNNNNNNNNNNNNNNNNNNNNNNNNNNNNNNNNNNNNNNNNNNNNNNNNNNNNNNNNNNNNNNNNNN
NNNNNNNNNNNNNNNNNNNNNNNNNNNNNNNNNNNNNNNNNNNNNNNNNNNNNNNNNNNNNNNNNNNNNNNNNNNNNNNN
NNNNNNNNNNNNNNNNNNNNNNNNNNNNNNNNNNNNNNNNNNNNNNNNNNNNNNNNNNNNNNNNNNNNNNNNNNNNNNNN
NNNNNNNNNNNNNNNNNNNNNNNNNNNNNNNNNNNNNNNNNNNNNNNNNNNNNNNNNNNNNNNNNNNNNNNNNNNNNNNN
NNNNNNNNNNNNNNNNNNNNNNNNNNNNNNNNNNNNNNNNNNNNNNNNNNNNNNNNNNNNNNNNNNNNNNNNNNNNNNNN
NNNNNNNNNNNNNNNNNNNNNNNNNNNNNNNNNNNNNNNNNNNNNNNNNNNNNNNNNNNNNNNNNNNNNNNNNNNNNNNN
NNNNNNNNNNNNNNNNNNNNNNNNNNNNNNNNNNNNNNNNNNNNNNNNNNNNNNNNNNNNNNNNNNNNNNNNNNNNNNNN
NNNNNNNNNNNNNNNNNNNNNNNNNNNNNNNNNNNNNNNNNNNNNNNNNNNNNNNNNNNNNNNNNNNNNNNNNNNNNNNN
NNNNNNNNNNNNNNNNNNNNNNNNNNNNNNNNNNNNNNNNNNNNNNNNNNNNNNNNNNNNNNNNNNNNNNNNNNNNNNNN
NNNNNNNNNNNNNNNNNNNNNNNNNNNNNNNNNNNNNNNNNNNNNNNNNNNNNNNNNNNNNNNNNNNNNNNNNNNNNNNN
NNNNNNNNNNNNNNNNNNNNNNNNNNNNNNNNNNNNNNNNNNNNNNNNNNNNNNNNNNNNNNNNNNNNNNNNNNNNNNNN
NNNNNNNNNNNNNNNNNNNNNNNNNNNNNNNNNNNNNNNNNNNNNNNNNNNNNNNNNNNNNNNNNNNNNNNNNNNNNNNN
NNNNNNNNNNNNNNNNNNNNNNNNNNNNNNNNNNNNNNNNNNNNNNNNNNNNNNNNNNNNNNNNNNNNNNNNNNNNNNNN
NNNNNNNNNNNNNNNNNNNNNNNNNNNNNNNNNNNNNNNNNNNNNNNNNNNNNNNNNNNNNNNNNNNNNNNNNNNNNNNN
NNNNNNNNNNNNNNNNNNNNNNNNNNNNNNNNNNNNNNNNNNNNNNNNNNNNNNNNNNNNNNNNNNNNNNNNNNNNNNNN
NNNNNNNNNNNNNNNNNNNNNNNNNNNNNNNNNNNNNNNNNNNNNNNNNNNNNNNNNNNNNNNNNNNNNNNNNNNNNNNN
NNNNNNNNNNNNNNNNNNNNNNNNNNNNNNNNNNNNNNNNNNNNNNNNNNNNNNNNNNNNNNNNNNNNNNNNNNNNNNNN
NNNNNNNNNNNNNNNNNNNNNNNNNNNNNNNNNNNNNNNNNNNNNNNNNNNNNNNNNNNNNNNNNNNNNNNNNNNNNNNN
NNNNNNNNNNNNNNNNNNNNNNNNNNNNNNNNNNNNNNNNNNNNNNNNNNNNNNNNNNNNNNNNNNNNNNNNNNNNNNNN
NNNNNNNNNNNNNNNNNNNNNNNNNNNNNNNNNNNNNNNNNNNNNNNNNNNNNNNNNNNNNNNNNNNNNNNNNNNNNNNN
NNNNNNNNNNNNNNNNNNNNNNNNNNNNNNNNNNNNNNNNNNNNNNNNNNNNNNNNNNNNNNNNNNNNNNNNNNNNNNNN
NNNNNNNNNNNNNNNNNNNNNNNNNNNNNNNNNNNNNNNNNNNNNNNNNNNNNNNNNNNNNNNNNNNNNNNNNNNNNNNN
NNNNNNNNNNNNNNNNNNNNNNNNNNNNNNNNNNNNNNNNNNNNNNNNNNNNNNNNNNNNNNNNNNNNNNNNNNNNNNNN
NNNNNNNNNNNNNNNNNNNNNNNNNNNNNNNNNNNNNNNNNNNNNNNNNNNNNNNNNNNNNNNNNNNNNNNNNNNNNNNN
NNNNNNNNNNNNNNNNNNNNNNNNNNNNNNNNNNNNNNNNNNNNNNNNNNNNNNNNNNNNNNNNNNNNNNNNNNNNNNNN
NNNNNNNNNNNNNNNNNNNNNNNNNNNNNNNNNNNNNNNNNNNNNNNNNNNNNNNNNNNNNNNNNNNNNNNNNNNNNNNN
NNNNNNNNNNNNNNNNNNNNNNNNNNNNNNNNNNNNNNNNNNNNNNNNNNNNNNNNNNNNNNNNNNNNNNNNNNNNNNNN
NNNNNNNNNNNNNNNNNNNNNNNNNNNNNNNNNNNNNNNNNNNNNNNNNNNNNNNNNNNNNNNNNNNNNNNNNNNNNNNN
NNNNNNNNNNNNNNNNNNNNNNNNNNNNNNNNNNNNNNNNNNNNNNNNNNNNNNNNNNNNNNNNNNNNNNNNNNNNNNNN
NNNNNNNNNNNNNNNNNNNNNNNNNNNNNNNNNNNNNNNNNNNNNNNNNNNNNNNNNNNNNNNNNNNNNNNNNNNNNNNN
NNNNNNNNNNNNNNNNNNNNNNNNNNNNNNNNNNNNNNNNNNNNNNNNNNNNNNNNNNNNNNNNNNNNNNNNNNNNNNNN
NNNNNNNNNNNNNNNNNNNNNNNNNNNNNNNNNNNNNNNNNNNNNNNNNNNNNNNNNNNNNNNNNNNNNNNNNNNNNNNN
NNNNNNNNNNNNNNNNNNNNNNNNNNNNNNNNNNNNNNNNNNNNNNNNNNNNNNNNNNNNNNNNNNNNNNNNNNNNNNNN
NNNNNNNNNNNNNNNNNNNNNNNNNNNNNNNNNNNNNNNNNNNNNNNNNNNNNNNNNNNNNNNNNNNNNNNNNNNNNNNN
NNNNNNNNNNNNNNNNNNNNNNNNNNNNNNNNNNNNNNNNNNNNNNNNNNNNNNNNNNNNNNNNNNNNNNNNNNNNNNNN

NNNNNNNNNNNNNNNNNNNNNNNNNNNNNNNNNNNNNNNNNNNNNNNNNNNNNNNNNNNNNNNNNNNNNNNNNNNNNNNN
NNNNNNNNNNNNNNNNNNNNNNNNNNNNNNNNNNNNNNNNNNNNNNNNNNNNNNNNNNNNNNNNNNNNNNNNNNNNNNNN
NNNNNNNNNNNNNNNNNNNNNNNNNNNNNNNNNNNNNNNNNNNNNNNNNNNNNNNNNNNNNNNNNNNNNNNNNNNNNNNN
NNNNNNNNNNNNNNNNNNNNNNNNNNNNNNNNNNNNNNNNNNNNNNNNNNNNNNNNNNNNNNNNNNNNNNNNNNNNNNNN
NNNNNNNNNNNNNNNNNNNNNNNNNNNNNNNNNNNNNNNNNNNNNNNNNNNNNNNNNNNNNNNNNNNNNNNNNNNNNNNN
NNNNNNNNNNNNNNNNNNNNNNNNNNNNNNNNNNNNNNNNNNNNNNNNNNNNNNNNNNNNNNNNNNNNNNNNNNNNNNNN
NNNNNNNNNNNNNNNNNNNNNNNNNNNNNNNNNNNNNNNNNNNNNNNNNNNNNNNNNNNNNNNNNNNNNNNNNNNNNNNN
NNNNNNNNNNNNNNNNNNNNNNNNNNNNNNNNNNNNNNNNNNNNNNNNNNNNNNNNNNNNNNNNNNNNNNNNNNNNNNNN
NNNNNNNNNNNNNNNNNNNNNNNNNNNNNNNNNNNNNNNNNNNNNNNNNNNNNNNNNNNNNNNNNNNNNNNNNNNNNNNN
NNNNNNNNNNNNNNNNNNNNNNNNNNNNNNNNNNNNNNNNNNNNNNNNNNNNNNNNNNNNNNNNNNNNNNNNNNNNNNNN
NNNNNNNNNNNNNNNNNNNNNNNNNNNNNNNNNNNNNNNNNNNNNNNNNNNNNNNNNNNNNNNNNNNNNNNNNNNNNNNN
NNNNNNNNNNNNNNNNNNNNNNNNNNNNNNNNNNNNNNNNNNNNNNNNNNNNNNNNNNNNNNNNNNNNNNNNNNNNNNNN
NNNNNNNNNNNNNNNNNNNNNNNNNNNNNNNNNNNNNNNNNNNNNNNNNNNNNNNNNNNNNNNNNNNNNNNNNNNNNNNN
NNNNNNNNNNNNNNNNNNNNNNNNNNNNNNNNNNNNNNNNNNNNNNNNNNNNNNNNNNNNNNNNNNNNNNNNNNNNNNNN
NNNNNNNNNNNNNNNNNNNNNNNNNNNNNNNNNNNNNNNNNNNNNNNNNNNNNNNNNNNNNNNNNNNNNNNNNNNNNNNN
NNNNNNNNNNNNNNNNNNNNNNNNNNNNNNNNNNNNNNNNNNNNNNNNNNNNNNNNNNNNNNNNNNNNNNNNNNNNNNNN
NNNNNNNNNNNNNNNNNNNNNNNNNNNNNNNNNNNNNNNNNNNNNNNNNNNNNNNNNNNNNNNNNNNNNNNNNNNNNNNN
NNNNNNNNNNNNNNNNNNNNNNNNNNNNNNNNNNNNNNNNNNNNNNNNNNNNNNNNNNNNNNNNNNNNNNNNNNNNNNNN
NNNNNNNNNNNNNNNNNNNNNNNNNNNNNNNNNNNNNNNNNNNNNNNNNNNNNNNNNNNNNNNNNNNNNNNNNNNNNNNN
NNNNNNNNNNNNNNNNNNNNNNNNNNNNNNNNNNNNNNNNNNNNNNNNNNNNNNNNNNNNNNNNNNNNNNNNNNNNNNNN
NNNNNNNNNNNNNNNNNNNNNNNNNNNNNNNNNNNNNNNNNNNNNNNNNNNNNNNNNNNNNNNNNNNNNNNNNNNNNNNN
NNNNNNNNNNNNNNNNNNNNNNNNNNNNNNNNNNNNNNNNNNNNNNNNNNNNNNNNNNNNNNNNNNNNNNNNNNNNNNNN
NNNNNNNNNNNNNNNNNNNNNNNNNNNNNNNNNNNNNNNNNNNNNNNNNNNNNNNNNNNNNNNNNNNNNNNNNNNNNNNN
NNNNNNNNNNNNNNNNNNNNNNNNNNNNNNNNNNNNNNNNNNNNNNNNNNNNNNNNNNNNNNNNNNNNNNNNNNNNNNNN
NNNNNNNNNNNNNNNNNNNNNNNNNNNNNNNNNNNNNNNNNNNNNNNNNNNNNNNNNNNNNNNNNNNNNNNNNNNNNNNN
NNNNNNNNNNNNNNNNNNNNNNNNNNNNNNNNNNNNNNNNNNNNNNNNNNNNNNNNNNNNNNNNNNNNNNNNNNNNNNNN
NNNNNNNNNNNNNNNNNNNNNNNNNNNNNNNNNNNNNNNNNNNNNNNNNNNNNNNNNNNNNNNNNNNNNNNNNNNNNNNN
NNNNNNNNNNNNNNNNNNNNNNNNNNNNNNNNNNNNNNNNNNNNNNNNNNNNNNNNNNNNNNNNNNNNNNNNNNNNNNNN
NNNNNNNNNNNNNNNNNNNNNNNNNNNNNNNNNNNNNNNNNNNNNNNNNNNNNNNNNNNNNNNNNNNNNNNNNNNNNNNN
NNNNNNNNNNNNNNNNNNNNNNNNNNNNNNNNNNNNNNNNNNNNNNNNNNNNNNNNNNNNNNNNNNNNNNNNNNNNNNNN
NNNNNNNNNNNNNNNNNNNNNNNNNNNNNNNNNNNNNNNNNNNNNNNNNNNNNNNNNNNNNNNNNNNNNNNNNNNNNNNN
NNNNNNNNNNNNNNNNNNNNNNNNNNNNNNNNNNNNNNNNNNNNNNNNNNNNNNNNNNNNNNNNNNNNNNNNNNNNNNNN
NNNNNNNNNNNNNNNNNNNNNNNNNNNNNNNNNNNNNNNNNNNNNNNNNNNNNNNNNNNNNNNNNNNNNNNNNNNNNNNN
NNNNNNNNNNNNNNNNNNNNNNNNNNNNNNNNNNNNNNNNNNNNNNNNNNNNNNNNNNNNNNNNNNNNNNNNNNNNNNNN
NNNNNNNNNNNNNNNNNNNNNNNNNNNNNNNNNNNNNNNNNNNNNNNNNNNNNNNNNNNNNNNNNNNNNNNNNNNNNNNN
NNNNNNNNNNNNNNNNNNNNNNNNNNNNNNNNNNNNNNNNNNNNNNNNNNNNNNNNNNNNNNNNNNNNNNNNNNNNNNNN
NNNNNNNNNNNNNNNNNNNNNNNNNNNNNNNNNNNNNNNNNNNNNNNNNNNNNNNNNNNNNNNNNNNNNNNNNNNNNNNN
NNNNNNNNNNNNNNNNNNNNNNNNNNNNNNNNNNNNNNNNNNNNNNNNNNNNNNNNNNNNNNNNNNNNNNNNNNNNNNNN
NNNNNNNNNNNNNNNNNNNNNNNNNNNNNNNNNNNNNNNNNNNNNNNNNNNNNNNNNNNNNNNNNNNNNNNNNNNNNNNN
NNNNNNNNNNNNNNNNNNNNNNNNNNNNNNNNNNNNNNNNNNNNNNNNNNNNNNNNNNNNNNNNNNNNNNNNNNNNNNNN
NNNNNNNNNNNNNNNNNNNNNNNNNNNNNNNNNNNNNNNNNNNNNNNNNNNNNNNNNNNNNNNNNNNNNNNNNNNNNNNN
NNNNNNNNNNNNNNNNNNNNNNNNNNNNNNNNNNNNNNNNNNNNNNNNNNNNNNNNNNNNNNNNNNNNNNNNNNNNNNNN
NNNNNNNNNNNNNNNNNNNNNNNNNNNNNNNNNNNNNNNNNNNNNNNNNNNNNNNNNNNNNNNNNNNNNNNNNNNNNNNN
NNNNNNNNNNNNNNNNNNNNNNNNNNNNNNNNNNNNNNNNNNNNNNNNNNNNNNNNNNNNNNNNNNNNNNNNNNNNNNNN
NNNNNNNNNNNNNNNNNNNNNNNNNNNNNNNNNNNNNNNNNNNNNNNNNNNNNNNNNNNNNNNNNNNNNNNNNNNNNNNN
NNNNNNNNNNNNNNNNNNNNNNNNNNNNNNNNNNNNNNNNNNNNNNNNNNNNNNNNNNNNNNNNNNNNNNNNNNNNNNNN
NNNNNNNNNNNNNNNNNNNNNNNNNNNNNNNNNNNNNNNNNNNNNNNNNNNNNNNNNNNNNNNNNNNNNNNNNNNNNNNN
NNNNNNNNNNNNNNNNNNNNNNNNNNNNNNNNNNNNNNNNNNNNNNNNNNNNNNNNNNNNNNNNNNNNNNNNNNNNNNNN
NNNNNNNNNNNNNNNNNNNNNNNNNNNNNNNNNNNNNNNNNNNNNNNNNNNNNNNNNNNNNNNNNNNNNNNNNNNNNNNN
NNNNNNNNNNNNNNNNNNNNNNNNNNNNNNNNNNNNNNNNNNNNNNNNNNNNNNNNNNNNNNNNNNNNNNNNNNNNNNNN
NNNNNNNNNNNNNNNNNNNNNNNNNNNNNNNNNNNNNNNNNNNNNNNNNNNNNNNNNNNNNNNNNNNNNNNNNNNNNNNN
NNNNNNNNNNNNNNNNNNNNNNNNNNNNNNNNNNNNNNNNNNNNNNNNNNNNNNNNNNNNNNNNNNNNNNNNNNNNNNNN
NNNNNNNNNNNNNNNNNNNNNNNNNNNNNNNNNNNNNNNNNNNNNNNNNNNNNNNNNNNNNNNNNNNNNNNNNNNNNNNN
NNNNNNNNNNNNNNNNNNNNNNNNNNNNNNNNNNNNNNNNNNNNNNNNNNNNNNNNNNNNNNNNNNNNNNNNNNNNNNNN
NNNNNNNNNNNNNNNNNNNNNNNNNNNNNNNNNNNNNNNNNNNNNNNNNNNNNNNNNNNNNNNNNNNNNNNNNNNNNNNN
NNNNNNNNNNNNNNNNNNNNNNNNNNNNNNNNNNNNNNNNNNNNNNNNNNNNNNNNNNNNNNNNNNNNNNNNNNNNNNNN
NNNNNNNNNNNNNNNNNNNNNNNNNNNNNNNNNNNNNNNNNNNNNNNNNNNNNNNNNNNNNNNNNNNNNNNNNNNNNNNN
NNNNNNNNNNNNNNNNNNNNNNNNNNNNNNNNNNNNNNNNNNNNNNNNNNNNNNNNNNNNNNNNNNNNNNNNNNNNNNNN
NNNNNNNNNNNNNNNNNNNNNNNNNNNNNNNNNNNNNNNNNNNNNNNNNNNNNNNNNNNNNNNNNNNNNNNNNNNNNNNN
NNNNNNNNNNNNNNNNNNNNNNNNNNNNNNNNNNNNNNNNNNNNNNNNNNNNNNNNNNNNNNNNNNNNNNNNNNNNNNNN
NNNNNNNNNNNNNNNNNNNNNNNNNNNNNNNNNNNNNNNNNNNNNNNNNNNNNNNNNNNNNNNNNNNNNNNNNNNNNNNN
NNNNNNNNNNNNNNNNNNNNNNNNNNNNNNNNNNNNNNNNNNNNNNNNNNNNNNNNNNNNNNNNNNNNNNNNNNNNNNNN
NNNNNNNNNNNNNNNNNNNNNNNNNNNNNNNNNNNNNNNNNNNNNNNNNNNNNNNNNNNNNNNNNNNNNNNNNNNNNNNN
NNNNNNNNNNNNNNNNNNNNNNNNNNNNNNNNNNNNNNNNNNNNNNNNNNNNNNNNNNNNNNNNNNNNNNNNNNNNNNNN
NNNNNNNNNNNNNNNNNNNNNNNNNNNNNNNNNNNNNNNNNNNNNNNNNNNNNNNNNNNNNNNNNNNNNNNNNNNNNNNN
NNNNNNNNNNNNNNNNNNNNNNNNNNNNNNNNNNNNNNNNNNNNNNNNNNNNNNNNNNNNNNNNNNNNNNNNNNNNNNNN
NNNNNNNNNNNNNNNNNNNNNNNNNNNNNNNNNNNNNNNNNNNNNNNNNNNNNNNNNNNNNNNNNNNNNNNNNNNNNNNN
NNNNNNNNNNNNNNNNNNNNNNNNNNNNNNNTGGTGTATTTATGAATTTATTAGTGTAATGACTCCATAGGGAAATGGCG
CCCCCACGTAGTTAGTGTAAAGTGAAGGGAAGCACAGCCAAATATCCGCACGTCAAAAGGAGTGAAATCTGAAAGGGTGC

AGGAGACATTGAAATCAATGAAGTGGCAAGGAAATGAATCAATATACATAAATTAACACTGTTGAGTGATCTCTCTCTTT
CTCCCCCTCTCTCTGATACTCACTCCTTTTCTCTCTTGCTTAAGTCTCAGTTGGAGTGGAAGAGGTTTGCTGTGTCTGTG
ATGCCCCGCCTCTTAAAGAACCTCTCATCAATTGTCTTAAATGTCGTCACGGTAAGGAAACACAGCATGTTATGTACTAA
TCATAGAGGTATTGTCAAATAATAACTTATGACGGATAGGCACAACTCATTTAAATAAAGAAAAAAAGAATGCCATCTTT
TTTTTCTTCAGGTTTTCTACCAGATAATATACTCTGAGTGTACAAAACATTAGGAACACCTTCCTAATATTGAGTTGCAC
CCTTTTTGCCCTCAGAACAGCCTCAGTTGTCAAGGGAAATGGACTCTACAAGGTGTCGAAAGAGTCCTACATAGATGCTG
GCCTATGTTGACTCCAATACTTCCCAGAGTTGTGTCAAGTTGGCTGGATGTTCTTTGGGTGGTGGACCATTCTTGATACA
CACAGGAAACTGTTGAGTGTGAAAAACCCAGCAGCGTTGCAGTTACTAATGTTTTATATACTCAAGAGTATAATATTTCA
TGAAAAGAGACTAGGAAAAGTCCTCCATCGGTAGCATATACTCAGCATGTGAATTACAGTAAACACTCTCTCCATTCCGT
AACAGGTTATCATCCTGAGTGCCACACTCCGTCCATTGAGCCGGAAGCTGACAGCAATTCTTGGATATGTCGGCAATGTG
TCTTTGCAGTGGCAACCAAGGTTAGACTGCTAATCTTTCTGTTTCACACACTGCCTCTTGGCTCAGGGAAAATATCAATT
TCAGACTTGTAACATTTTTTTTTATCTATCCAGAGGAAGCAATTTCAATACCAGTGTTGTGTTAAACAGTGTTGTCAATG
GATATATCTGAAGGACACCACAGAAATGTTACACTCTGTATTATTGTGCCTGGACATCTGACCAGTTCGTCAATGCAGAT
TTTTTTCAAGATTATACTGTATTATTATTATGGTCAGTAATTCATGTATGCTCCAATTCTGTACTGTAGGTGTTAGATGA
ATGAGCACTGTGGTTAAGGGCTAATTGTCCATCGGTCAATGTCCATATCTCATTGACCTGAAGATATTCTCAAGATATTA
TCTCACTCAGCTGACCTACAATAAAATGAGATGAACTGCTTTTGCTGGATAGTTCCCATCCAATGTCATCTATGTTGAAT
GTACTGACATGACCGGGAGTGGAGAGGTCGCTGTAGTGAATCGATTTGTCACCTTTTATTTCAAAAAGGGGGAGCCATCA
ANNNNNNNNNNNNNNNNNNNNNNNNNNNNNNNNNNNNNNNNNNNNNNNNNNNNNNNNNNNNNNNNNNNNNNNNNNNNNNN
NNNNNNNNNNNNNNNNNNNNNNNNNNNNNNNNNNNNNNNNNNNNNNNNNNNNNNNNNNNNNNNNNNNNNNNNNNNNNNNN
NNNNNNNNNNNNNNNNNNNNNNNNNNNNNNNNNNNNNNNNNNNNNNNNNNNNNNNNNNNNNNNNNNNNNNNNNNNNNNNN
NNNNNNNNNNNNNNNNNNNNNNNNNNNNNNNNNNNNNNNNNNNNNNNNNNNNNNNNNNNNNNNNNNNNNNNNNNNNNNNN
NNNNNNNNNNNNNNNNNNNNNNNNNNNNNNNNNNNNNNNNNNNNNNNNNNNTTTAGTCTTATCATTTCATTTTTACAGCG
GAGATGTAAAAACATTGGTGTCTGTCTCTCTGACATTTGCAACATTGTTTCAATATTCACATTGGATCTCCAGCTGTCCC
ATAGTAATGAACGAGTCCGGATGAGACAGACAGGAAGGCAGCTTTTCTCAGCCAGGTGAAAACATCAATCAGCATCATTT
GTATGGATATATATAAATAACTATCAATAGAAAACAGGTCAAACGAAACAAAGTGCAGCTAGTTTGCTGTTTTTCCATCT
TCAGTTTGAAGGGATTGTGTTAGCTGTGTTGTTGGCTAGCTCCTCTGAACAACAGTGTCCTAAAGAGAGCACACATTTTC
TATGCCAGGCGAAATCGCGCCTCATTAGCTCATTGTTATGGATGTACCCAAATAAATGTCACTAGAAAACAGTTTAAACA
AATGCAAATGCAGCTACTTTGCTGTTATTCCTGCTGCACTGTTTGACATGACTGTAAGTTAGCCGTAGTTAGCTAGCTAG
CAAGCAAGGGATAACAACGTTGCAAGTCAGTATTTCGATGGAACATTTAGAACAAACAACTGGGTCGCATCCATAGACAC
AGAACAAAAAGACTGAACGACCGGGTCGCGTCTCTGGGAACTGAACGAATGACCAGCCGGCTTGGGTAGCAACCCTAGAT
TTGTGTCGGGACTATATCTTGTGGAAGGATGAAATGGTATGAATACATTAATCCAAATAACGTTTATAATGAAAATATGT
CAATAATTATTTGAATATGTTGGTAACCCGTTGTATAAAAGTGATAATGCCCTCAAAGTCGGTGTTTGGAATATATATTG
GCACGGTTTGCCGGCCCTGGACTTTGTCTCGGTTCTAACAACACCCATACCAATATACCCTCCAAACACCAGCTTCTCTG
GCATTATCACTTAATAATAACATTTTATAATTAATGTTTAGCACATGTATTGGTCAATAAGCTTTACTATTAATTTTCCT
TACCAACAAAAACAAATAGAATATTCATGTTGGAACCCTGTATGTCCTTTTCACCATTATATAGCTCTACTGTCAAGACA
TAATGTGTTTTTCAATAGAGTATTGCAGCCTCTTGAAAAATAGATATTATACTGTGCTCTTGTCAGGTAGCCACCAGATG
TTAGCTATGTTTAGGCCATTGGGGAGTGTGGCTAATGTTTTTATTTATCAACTGCAGGTGGAACTTGAAAATGTTGCAGT
GTGGAAGTTGTGGTCAGTGGTTTCATGAAGCTTGCACGCAGTGTCTGACCAAGCCGTTGTTATATGGAGACAGGTGAGAC
AGAGTATTGTTATCTATAACCTTGTACTGCTTCTTTGCATTACCATTGTCAAACTATAACAGAGAGGTTATAAAACTGGC
AGTGGTAGGGCTAATGCTGTTAGTGTTAATTGCTTATAGCAGTGCAGTGTTGTTATTCTGCCTCTCTGGTGTCCAATGTA
ATGTGGTAGAGGTTTAAAATGAATTCAAATTAGTCCAGTCCATTACACCATTATTGTCACATTAACCATCTGTATTATCC
TACTGGTTTAATACCCATGATATATTTATTATATTACTCTTTGTTTCTGCGTAGGAAAGTAATGTTTGAGATTGATGGAG
AGGTAGAATGTGGTGGGTTGTTACATCTGCTAAACAGGTGATGGAAACTGGGAAGAATTTGCTGACAGTAGCAACCATGT
GCTATGTTATGTTTCCATTGACTTTTATATCTGGTCAATCACAGACAAAAATAGAATCATCTGTGTTCTATTTCTTTCTG
AAAGGTTTTATGAGTTCCAGTGCTCAGTCTGTGCAAGCGGACCAGAGACAATTCAACGGCTACCCATGAGCTGGTAAAGC
TATACAGTATCTGCTAAGAGAAGAATACATCATTTAAACTGCAGGGATTATCACACTGAAACAGTCTTGAAAACCAGTTT
CTTCTGACAGGTTTATTGAGTCATGTGGGTTACAGTATGTTAATTCCTCTTACTCAATAATGTAATGAAAAGCTTTGTGA
TGTGATAAATTCTGAACTAAGTGTTTTTTTTTCCTGGTGTGCTTGTTGTAAATACAGGGTAGATTTGGCCCATTTGGTGCT
CTACCACCTCTCCTTGTGCTGCAAGAGAAAATACTTTGATTTCGACCATGAAATCATGTCGTTTGCCAATGAGAATTGGG
ACTCTTTGCTTCTAGGCTCGGTATAAACATTTCCTCACTTACATGCTATGATGAAACGATGTGAAAGTGTTAGCTAGCCG
CATTGGTTAGCAGTCATGTGGTAATTATGTGCTTTGATTTCATTAGCTACATGCCATTGTTATTTAGCAGATTTTAAATG
GTAAATATAATTTGTATTTAATGTGGCTCTCAGGTACTGCAGTTGCATTTAAATCAGTCATACCCTTTTTTAATGAACTG
TTCACAAATCACATTAGTAACTAACTACGTTATCCATGGAAATGTTTACCTTTCGCCAATGAATGTCAATGTGTCTCTAA
ATGTGTTAACGTTTGTTTCAGGCTACCATCCACAGTGTTAATCAAACTCCTGATTTTCATTCCTCTCTGTCCCCCAGCTC
TCTGACACACCAAGGCAAGATCGCTGCCACAATCTGCTCAATGCTTTGAACTCCAACAAAGACAGGTGAGAGTCTGCATA
TGATGCGAAGAATAAACAGAAGCCACAGCTATGCGTGACCAGATTTATGATAGCGTTGCTTCTGGAATTGTACATTTGGG
TAAAGAAAATACAGTTAGTTAATTGTTTCTTAACTCTTTGATATTATCAATAGAGAACGATAAGAAAACATAGACAATAA
TGATAACTTGTTTTAAAAAGAACGATGACCTGACTTTACAAAGTTGTTGTCTACAAGTTTTGAAAGTCTTTTTCTCTGTG
ACGTCATCCAGGTTTGTCTCTGGTAAAGAGATCAAGAAGAAGTGTCTTTTTGGGCTCCAGGTCCGAGCTCCGCCTCC
CCTGACCTCTGATTCGTCCCCCCTCATCACTGACCCGCCCATCAACATCACCCAAAGGAGAAGGTCAGAGTAAGGACAAC
TTCTGTGATTTATTATTCTCTCTTCATCACAAACACAAGCCTCAGGACTCAACTCCTTATATGGACACATTCATTCTAGT
AATTAATTACACATTTACAATAGTGAAAAAGAGCGGCACCTTGTAGACGGTGGTCCTTCCAGCTGGATAGGAATAACTGT
TCCTAACTAAACAGGCACATGTGTGCCTCATTAAATCATGACGGGAAGGACTCACTTATTTCACTCTGGTGAATATGTTT
ATAACTAATATTTGTATCAATAAATACATCATCTATAGTGTGTGTGTGTGCATACGTGCGTGTGCGCATTCGCATGTGGA
CATTGATCATCTTGCTAAACATTCTCAACACCATCTTCTCTTTGATTCTCTATAGGCTTAAAATAAAAAACACATTTATA
CCAAAAGTTCAGACACACCTACTCATTCAAGGGTTTTTCTTTATTTTTACTATTTTCTACATTGTAAAATAATAGTGAAG
ACATCAAAACTATGAAATAACACATGGAATCAGGCAATAACCAAACGTGTTAAATTTTTTTTAAATAGATTTTATATTTG
AGATTCTTTAAAGTAGCCACCCTTTGCCTTGATGACAGCTTTGCACACTCTTGGCATTCTCTCAACCAGCTTCACCTGGA
ATGCTTTTCCAACAGCCTTGAAGGTGTTCCCACATATGCTGAGCACTTGTTGGCTGCTTTTCCTTCACTCTGCAGGTCCA

ACTCATCCCACACCATCTCAATTGGGTTGAGGTCGGGTGATTGTGGAGTCCAGGTCATCTGATGCAGCACTCCATCACTC
TCCTTCTTGGTCAAATAGCCCTTACACAATCTGGAGGTGTGTTGGGTCATTGTCCTGTTGAAAAACAAATGATAATCCCA
CTAAGTGCAAACCAGATGGGATGACATATCGCTGTGGTAGCCATGCTGGTTAAGTGTGCCTTGAATTCTAAGTAAATCAC
TGACAGTGTCAATAGCAAAGCACCCCCACACCATCACACCTCCTCCTCCATGCTTCACGGTGGGAACCACACATGCGAAG
ATCATCCGTTCACCTACTCTGCGTCTCACAACGACATGGCGGTTGGAACCAAAAATCTCAAATTTGGACTCATCAGACCA
AAGGACAGATTTTCACCGGTCTAATGTCCATTGCTCATGTTTCTTGGCCCAATAAATTCTCATATTCTGATTGATGGTCC
TTTAGTAGTGGTTTCTTTGCAGCAATTTGACCATGAAGGCCTGATTCACGCGGTCTCCTCTGAACAGTTGATGTTGAGAT
GTCTGTTACTTGAACTCTGTGAAGCATTTATGGGCTGCAATCTGAGGTGCAGTTAAATCGAATGAACTTATCCTCTGCAG
CGGAGGTAACTCTGGGTCTTCCTTTCCTGTGGCGGTCCTCATGAGAGCCAGTTTCATCATATCACTTCATGGTTTTTGCG
ACATCACTTGAAGAAAATTTAAAAGTTTTTGAAATGTTCCGTATTAACTGACCTTCATGTCTTAAATTAATGATGGACTG
TCATATCTCTTTGCTTATTTGAGCTGTTTTTGCCATAATATGGACTTGGTCTTTTAGCAAATAGGGCTATCTTCTGTATA
CCACCCCTACCTTGTCACAACAACTGATGGGCTGAAACGCATTAAAAGGAAAGAAATTCCACAAATTAACTTTTAACAAG
GCACACCTGTTAATTGAAATGCATTCCAGGTGACTACCTCATGGAGCTAGTTGAGAGAATGCCAAGAGTATGCAAAGCTG
TCATCAAGGCAAAGGGTGGCTACTTTGAAGAATCTCAAATTTCAAATATATTTTGATTTAACACTTATTTGGTTACTACA
TGATTCCATGTGTTATTTCATAGTTTTGATGTCTTCACTATTATTCCACAATGTAGAAAATAGTACAAATAAAGAAAAAC
CCTGGAATGAGTAGGTGTGTCCAAACTTTTGACTCATACTGTAGTTTTCTAAGTGAAAACATTGCACCACTCCTCAAGTT
ATTGACTGTGTCTCTGTGTTGTTGTGTGGAGCAGCCCATTGTCACTGCCCTGCCAGAGGAGAGCGGTGGGGCCAGAGTCA
CGCAAATCAAAGCGGCGCATCATGGAGACACAGGTCAGTGTTTCTGTCTCGAACCAGGCATCAAGGGAGAGATCAGCCAT
CAGTCGCCCCCTCACTGCATAGCAAAGAGCTGCCTGGCATTGGAATGACTTGTCAGTCATAGTCAGTCAGTGTGTGGAGA
AAAGATTCTCCTTATAGCCAGTGTTGTCTGCTTACATATTCTTCAAAGCCCAGCCTACCCCTCATACACTATTGAATTGA
AGCTGGTTTACGACTCGATCGAGTATGACAACATGTTATGGTCACGGGTTTAGAAATATATTTTTTAATATGAATAGAAA
ATACACATCCTTTTATACTACTGCTCAGGCTGGCTCCCTTATCCTGAGACAATAAATGTTATGGATCGGTCGTATTTCAG
CGGGCAGTACTTCCAGCAGACTGTTGTATCCTGAGCGGACATTAATCATCTGCCATCTTGGGGCAGGTTGGTTATCCCAG
GGAAAGTGAGAGGGGAAGGTACGCTGATATGCGGCATCTGGGAGCGCTTCCAAGTGCACTTTGCAGTCGATTACAGTTTG
GCCAGGTGGCACTGGCACTTTGACAGTGCAGCATTGACAGAGCACCTTGATTTAAAGGGACAGCACGCTAGTAAGCGGAT
AATGCTAGTATACCACTGCTGTAATTACTGTATATGTTGTGGGTCACAGTCCAGCATTGGAGGTTGACAGAGCTGAGGGT
GTGCTGAGAACTGAGAAAGCAAAGGATAGATGGGATAGAGTACTGATGCTTTTGTTGTTATGATAGTGAGCTCACACTAA
GAAATGCTACCTCAATCTCCACAATATGAATCTACTTATCATAGGAAATGGAGGTGACAGATATACACTATATATACAAA
AGTATGCGGACATGGCTATTTCAGCCAGACCTGTTGCTGAACATCACACAGCCATGCAATCTCCATAGACAAGCATTGGC
AGTAGAATGGCCTTACTTAAGAGCTCAGTGACTTTCAAATAGAGCAGCCCCTGTCAACTGTAAGTGCTGTTATTGTGAAG
TGGAAACGTCTAGGAGCAACAACGGCTCAGCTGCAAAGTGGTAGGCCACACAAGCTCACAGAATGGGACCTCCACGTGCT
GAAGCGTGTAGCGCGTAAAATCGTCTGTCCTCAGTTGCAACACTCACTATCGAGTTCCAAAATGCCTCTGGAAGCAGCGT
TGGCACAAGAACTGTTCGTTGGGAGCTTCATTAAATGGGTTTCCATGGCCGAGCAAATCACCATTCGCAAGGCCAAGCGT
CCGATGGAGTGTTACAAAGCTTGCCGCCATTTGACTTTGGAGCAGTGGAAACGCGTTCTCTGGAGTGATGAATCACATTT
CACCATCTGGCTGTCTGACGGACGAATCTGGGTTTGGCAGATTCCAGGAGAACTCGACCTGCCTCAATGCATAGTGCCAA
CTGTAAAGTTTGGTGGACGTGGAATAATGGTCTGGGGCTGTTTTTCATGGTTCGGGCTAGGCCCGAATGACATTCTAGAC
TACTCCGGGATGCTGGCCTTCTAGGCAGAGTTCCTCTGTCCAGTGTCTGTGTTCATTTTCCCATCTTAATATTTTCTTTT
TATTGGCCAGTCTGAGATATGGCTTTTTCCTTGCAACTCTGCCTAGAAGACCAGCATCCCAGGGTCGCCTCTTCCCTGTT
GACGTTGAGACTGGTGTTTTGCGGGTACTATTTAATGAAGCTGCCAGTTGAGGACTTATGAAGCGTCTGTTTCTCAAACT
AGACACTGAAGGCCTACCTTCAAACTCAGTGCCTCTTCTCTTGACATCATAGGAAAATCAAAAGAAATCAGCCAAGACCT
CAGAAAAAAAAATTGTAGACCTCCACAAGTCTGGTTCATCCTTGGGAGCAATTTCCAAATGCTTGAAGGTACCACGTTCAT
CTGTACAAACAATAGTACGCAAGTAAAACACCATGGGACCACGTAGCCGTCATACCGCTCAGGAAGGAGACACGTTCTGT
CTCCTAGAGATGAACGTACTTTGGTGCGAAAAGTGCAAATCAATCCGAGAACAACAGCAAAGGACCTTGTGAAAATGGTG
GAGGAAACAGGTACACAAGTATCTATATCCACAGTAAAACGAGTCCTACATCGACATAACCTGAAAGGCCGCTCAGCAAG
GAAGAAGCCACTGCTCCAAAACCGCCATAAAAAAGCCAGACTACGGTTTGCAACTGCACATGGGGACAAAGATCGTCCTT
TTTGGAGAAATGTCCTCTGGTCTGATGAAACAAAAATAGAACTGTTTGGCCGTAAGGACAATCGTTATGTTTGGAGGGAA
AAGGGGGAGGCTTGCAAGCCGAAGAACACCATCCCAACCGTGAAGAACGGGGGTGGCAGCATCATGTTGTGGAGATGCTT
TTCTGCAGGAGGGACTGGTGCACTTCACAAAATAGATGGCTGCATGAGGAGGAAAATGATGTGGATATATTGAAGCAGCA
TCTCAAGACATCAATCAGGAAGTTAAACTTGGTTGCAAATGGGTCTTCCAAATGGACAATGACCCCAAGCATACTTCCAA
AGTTGTGGCAAAATGGCCTAAGGACAACAAAGTCAAGGTATTGGAGTGGCCATCACAAAGCCCTGACCTCAATCCTATAG
ACAATTTGTGGGCAGAACTGAAAAAGCGTGTGCGAGCAAGGAGGCCTACAAACCTCACTCAGTTACACCAGCTCTGTCAG
GAGGAATGGGCCAAAATTCACCCAACTTATTGTGGGAAGCTTGTGGAAGGCTACCCAAAACGTTTGACGCAAGTTAAACA
ATTTAAAGGCAATGCTACCAAATACTAAATGAGTATGTAAACTTCTGACCCACTGGGAATGTGATGAAATAAATAAAAGC
TGAAATAAATCATTCTCTCTACTATTATTCTGACATTTCACATTCTAAAAATAAAGTGGTGATCCTAACTGACCTAAGGC
AGGGAATTTTTACTAGGATTAAGTGTCAGGAATTGTGAAAAACTGAGTTTAAACGTATTTGGCTGAGGTGCAAATCTTCC
GACTTCAATTGTAGATATTCCATTATAAATCAGCCGATATGTATCATTTGATACAGTATGTTAATAGACCTTGGTTTTTA
TTTAGATGTATTCACAAGCAGTATGAATTCTTCTATAAAGATAAGGACATGAACTGAAGTATCATGTCTGTGTGATTGGA
TTCATTGATTTGGCGTCAAAACACTAGCTAATCAGTGTTTCTCCTGTGTCCAGCCTTGTCCGCCCCCGGTCCCAGCCACC
CCAATAGATCTTGTGCCATGTTGCCATGGCTACGTAGGTGGAACTAACCTCTACAACTCCAGGAAGTCGGAGGAGGAGCT
GAATTTGGGGTTGGTGCTGTTATTGTATTGGTTACACTTTACATACTGTATAACAAGTAATAATGCATTGTATTGTATTT
ACAGTGTTAGCTTTTTTTTTACATTACATCCTCATTGATTTAGGCTACCCTTATCAAATCAAATTAAATTATGTTGGTCA
CATACACGTATTTAGCAGATGTTATTGCAGGTGTAGGAAATGCTTGTAATGCTTGTATTAGGCAGTATGTCAGTGGAAGT
CCATGCATTGTTGCTTTTTGTCCGTTTTTTTATTTTTTTTTTATGTCCCTGCTTTTTCCACAGCTTTCCACCTAAGCGGAT
GTTTGCCCTGTATCACCCCACCTATAACGAGCCCAGCCATCTCCAGAACCCTGCATCACTATAGGTCAGTATTAACCCCA
GCTCCTCCTGCTCTCCGGGGACTACCCTCAATCTAGACATCTAGAGAGGAAACCATAGCTGTGCCTGGATGACAATGGAA
AAAGTATGAATCGTGAAATATTAATTTCTCTCTCTCTCTCTCTCTCGCTCTCTCCAGTGCGGAAGATCCTTGTCGAGTG
CCGCCCCCCTGCCTCCCCTTCTCCCTGTCATCCTCCCACCATCACCACCACCACCACAACAACCACCACCACCAGCAGCA
GCACCACCAGCACCAGCCTGGCCAGAAGTTGGATCCCTGCCCCTCCCTCCTGCTGCGTGACGTCCCTTCCTATCCTGGCG
GTGTGGGGGGAGGGGCTGGGGGGGATGGGTCAGCTGTCAGGGGCTGGGGGGGCGGGGACGGGGTGAGGATTTTGGCGAGG

CGAGTAACGCCAGACGGGAAGGTCCAGTACCTCGTGGAGTGGGGGAACGTGAGTGTGTACTGAGAGAAAGTGAGAGATAG
ATGGGGGGGTGGAGGGAGGCAGAGAGGATCAACACACCAGAGTGCTTGGCAGGAAAGAGGGGATATTGGAGAGAGAGAGG
GAGAATGACTGTCGTGCAGGTGCCACATCAGAAATGTATGGCAACATCAAGTACAAGCAAAACAAAAACCCCACACAAGG
CAAATCCAGGTTGCTCAAACGATATCGCCTCCATTCTATATCATCACACTATGTGTTACTATATCTCCCTCTTTGCTAGT
GTTTCTCCTATATTTGTACTGTATACCTTTGGAATATGCCTTTGCATTTATCATGTTTACGTTTGAAGATTGTATATACA
GTATTTGATTATAGGCAATTTGTTACGTTACCTTTTGTCATACTTGATTTACATGATAGTATGTAATAGAAATATATTAG
GAGCCGCAACAGGGCTTTTGTTTTATAGTTAGATTTATATATTCACAATATATTATTATAGAATGATTTGATGTGGTTGC
ATAGTGCCTATCTGGATTGTGCATCTGCAGATTGAGAAGTTGACCTGAATCAAGGGATCAGGTGATTGACATGTATGATA
ACTATTCTATAAGGTTTTCTTCCTCATCCACCAGTTGCAATTGAAGAGTCACGACAATGTTGTGTAGGCTTTCGCACACA
GAAGTAGTGTAAAATACATTCCAAACAAATATTAAAACATCTCTTCAAGATTACTGATCAAAATCTTTGATCAGATAGCC
ATGGAAAATGGTCGCTCGTTGAGCCAGATTTGACTGTCAATTTGTTGAGGGGTGTTTTTGCCTTGTTTTAGGGAAGAGAA
GTCAACAAATTATGGTATTATGTGCAGGTGAAACTATTTGTACTGTTTTCCATTTTCAGGCCATAGACCGAGTCGTCAGC
TCCTATCTTCCAGGTTTACACTGAGGATGAGAGATAGCGTTACCTCTGGCCCACTTCCAAGCTGATACCTAAGCACTAGT
CTGGACACTGTAGATCTGACAAGATTGGATAGATCTGAGCAATATGGCTTAAGTTTCATCCGGTCTATCAGAGGGAAAGG
TGGAGCTTTCATCATATTTTTCATATATCCGATCCTTTCAGATGTACCCAAGAGTCTAAGGGGTTAGGGCTATGGGTTTG
TGTTTGATTTGGAACTGGGCCCCAGAGATCATAGTGGAACAGGAGGTTGTGTATAGAACTCTAGAATATCATAGTGCAAG
GTTCAGAGTTTATATTTATACAGATCCTTTATTTCTGCCTTTTGTGGGATAAAACACAGATAAGAGTAAATGTGTGTGTT
GTATCCCCCAGAAGACAGATTGTGTTACAGGTGTAGTTACAGTAAATACGTAAATAACACAATAGGCAGTTATACTGTTA
TAATCATGTCAACAGAAAACATGTTCAGTATTTTTTTTTTAAACCACAGCAAGATGTCGCCCGTTCCTCAGATTGAATGA
TGTGAAATATGTCATCTCTTACTTAAGCAATAAGGCCTGAGCGGGTGTGGTATATGGCAAATATACCACAGCCAAGGGCT
GTTCTTAAGCACAACCAAATGCGGAGTGCCTGGATACAGCCCATAGACATTGTATATTGGCCATATACCACAAACCCCAG
AGGTGCCTTATTGCTATTACAAACTGGTGAACAACGTAATTAGAACAACAAACAAGTAATTTTGTGTCATACACATGGTA
TACAGTCTGATATACCACATCTTTCAGCCAATCAGCATTCATGGCTCGAATGACCCGGTTTATAATAGTGAATAGATGAT
TGACGTAACATAGTAAATGTAAATCCTGAACACTCAAATGAGTATGATATGTTATGTTTGGTATAGTTAGATAAGACAGA
AGGTTACTTAAGGCAAAAAGGAAAGTAATGTGGATGGGTAGGAATATAACGCGAATGACTAACAACCCAAAGGTTGCGTG
TTCGAATCTCGTCACGGACTTTAGCATTTTAGTGCATTAGCAACTTTGCAACTACTTGCTACTTTGCAGCTACTTAGCAT
GTTAGCTAACCCATCCCCTAACCTTAACCCTTTAACTTAACTCCTCACCCTAACCTTAACCCCAACCCCTAGTGTAGTTA
ACGTTAGCCACAACAAGTTGGAATTCGTAAAACATCATACATTTAGCAAATTCCTACCATATTGTACAAATTGCAATTCC
TAACATATAATACAAAATCTACAAATTAATACATATCATACAAAATGTAACATAGCATACAAATTGGAGTGTCACAGATT
TACATTTACTATGTTACATCTACCCCTGAGTCTTGGTTGGATTGCAAAGGGTTTGGCACTTTCTAAACAGCAGTGCAGGT
CAAATGAACCTCCCAACTCAGTCCTTCCCATCCCAGTTTAGGATTTAGATAATTCTCAGTGAATTTGGCTGTAGTTGAAT
AGGAAGAGGTCTGTGGAACCATCAATTCGATGGTAATGAATGTATCTGTATGTTTTCTATGGTAAAGTATGTCTATAGAC
CCGTCAAGTGTTCGTTACTGTAGGTTATAGATGGAACTATACAAAATGACTTCAAAGGACTGGGGTAAATCTGATTTTGA
GTTGAGGAACAAGAAGCTAAGTCTTCTCTTTTTTTTTAATTTGTAGTCAAGTCCAAGTTTGGTTTGATGTATTTTTGGACT
TTTGGTCAAATTACGTTTATACATGTATAACCTAGGCTGACACAGCTAATGTAAAATGTAAAAAATTAAAAGTTGCCCTT
CACATCTTACTAATGTGCTTCAAGTGGTTTCTGTCCATTTCTGGGTTAACTTACAATNNNNNNNNNNNNNNNNNNNNNNN
NNNNNNNNNNNNNNNNNNNNNNNNNNNNNNNNNNNNNNNNNNNNNNNNNNNNNNNNNNNNNNNNNNNNNNNNNNNNNNNN
NNNNNNNNNNNNNNNNNNNNNNNNNNNNNNNNNNNNNNNNNNNNNNNNNNNNNNNNNNNNNNNNNNNNNNNNNNNNNNNN
NNNNNNNNNNNNNNNNNNNNNNNNNNNNNNNNNNNNNNNNNNNNNNNNNNNNNNNNNNNNNNNNNNNNNNNNNNNNNNNN
NNNNNNNNNNNNNNNNNNNNNNNNNNNNNNNNNNNNNNNNNNNNNNNNNNNNNNNNNNNNNNNNNNNNNNNNNNNNNNNN
NNNNNNNNNNNNNNNNNNNNNNNNNNNNNNNNNNNNNNNNNNNNNNNNNNNNNNNNNNNNNNNNNNNNNNNNNNNNNNNN
NNNNNNNNNNNNNNNNNNNNNNNNNNNNNNNNNNNNNNNNNNNNNNNNNNNNNNNNNNNNNNNNNNNNNNNNNNNNNNNN
NNNNNNNNNNNNNNNNNNNNNNNNNNNNNNNNNNNNNNNNNNNNNNNNNNNNNNNNNNNNNNNNNNNNNNNNNNNNNNNN
NNNNNNNNNNNNNNNNNNNNNNNNNNNNNNNNNNNNNNNNNNNNNNNNNNNNNNNNNNNNNNNNNNNNNNNNNNNNNNNN
NNNNNNNNNNNNNNNNNNNNNNNNNNNNNNNNNNNNNNNNNNNNNNNNNNNNNNNNNNNNNNNNNNNNNNNNNNNNNNNN
NNNNNNNNNNNNNNNNNNNNNNNNNNNNNNNNNNNNNNNNNNNNNNNNNNNNNNNNNNNNNNNNNNNNNNNNNNNNNNNN
NNNNNNNNNNNNNNNNNNNNNNNNNNNNNNNNNNNNNNNNNNNNNNNNNNNNNNNNNNNNNNNNNNNNNNNNNNNNNNNN
NNNNNNNNNNNNNNNNNNNNNNNNNNNNNNNNNNNNNNNNNNNNNNNNNNNNNNNNNNNNNNNNNNNNNNNNNNNNNNNN
NNNNNNNNNNNNNNNNNNNNNNNNNNNNNNNNNNNNNNNNNNNNNNNNNNNNNNNNNNNNNNNNNNNNNNNNNNNNNNNN
NNNNNNNNNNNNNNNNNNNNNNNNNNNNNNNNNNNNNNNNNNNNNNNNNNNNNNNNNNNNNNNNNNNNNNNNNNNNNNNN
NNNNNNNNNNNNNNNNNNNNNNNNNNNNNNNNNNNNNNNNNNNNNNNNNNNNNNNNNNNNNNNNNNNNNNNNNNNNNNNN
NNNNNNNNNNNNNNNNNNNNNNNNNNNNNNNNNNNNNNNNNNNNNNNNNNNNNNNNNNNNNNNNNNNNNNNNNNNNNNNN
NNNNNNNNNNNNNNNNNNNNNNNNNNNNNNNNNNNNNNNNNNNNNNNNNNNNNNNNNNNNNNNNNNNNNNNNNNNNNNNN
NNNNNNNNNNNNNNNNNNNNNNNNNNNNNNNNNNNNNNNNNNNNNNNNNNNNNNNNNNNNNNNNNNNNNNNNNNNNNNNN
NNNNNNNNNNNNNNNNNNNNNNNNNNNNNNNNNNNNNNNNNNNNNNNNNNNNNNNNNNNNNNNNNNNNNNNNNNNNNNNN
NNNNNNNNNNNNNNNNNNNNNNNNNNNNNNNNNNNNNNNNNNNNNNNNNNNNNNNNNNNNNNNNNNNNNNNNNNNNNNNN
NNNNNNNNNNNNNNNNNNNNNNNNNNNNNNNNNNNNNNNNNNNNNNNNNNNNNNNNNNNNNNNNNNNNNNNNNNNNNNNN
NNNNNNNNNNNNNNNNNNNNNNNNNNNNNNNNNNNNNNNNNNNNNNNNNNNNNNNNNNNNNNNNNNNNNNNNNNNNNNNN
NNNNNNNNNNNNNNNNNNNNNNNNNNNNNNNNNNNNNNNNNNNNNNNNNNNNNNNNNNNNNNNNNNNNNNNNNNNNNNNN
NNNNNNNNNNNNNNNNNNNNNNNNNNNNNNNNNNNNNNNNNNNNNNNNNNNNNNNNNNNNNNNNNNNNNNNNNNNNNNNN
NNNNNNNNNNNNNNNNNNNNNNNNNNNNNNNNNNNNNNNNNNNNNNNNNNNNNNNNNNNNNNNNNNNNNNNNNNNNNNNN
NNNNNNNNNNNNNNNNNNNNNNNNNNNNNNNNNNNNNNNNNNNNNNNNNNNNNNNNNNNNNNNNNNNNNNNNNNNNNNNN
NNNNNNNNNNNNNNNNNNNNNNNNNNNNNNNNNNNNNNNNNNNNNNNNNNNNNNNNNNNNNNNNNNNNNNNNNNNNNNNN
NNNNNNNNNNNNNNNNNNNNNNNNNNNNNNNNNNNNNNNNNNNNNNNNNNNNNNNNNNNNNNNNNNNNNNNNNNNNNNNN
NNNNNNNNNNNNNNNNNNNNNNNNNNNNNNNNNNNNNNNNNNNNNNNNNNNNNNNNNNNNNNNNNNNNNNNNNNNNNNNN
NNNNNNNNNNNNNNNNNNNNNNNNNNNNNNNNNNNNNNNNNNNNNNNNNNNNNNNNNNNNNNNNNNNNNNNNNNNNNNNN
NNNNNNNNNNNNNNNNNNNNNNNNNNNNNNNNNNNNNNNNNNNNNNNNNNNNNNNNNNNNNNNNNNNNNNNNNNNNNNNN
NNNNNNNNNNNNNNNNNNNNNNNNNNNNNNNNNNNNNNNNNNNNNNNNNNNNNNNNNNNNNNNNNNNNNNNNNNNNNNNN
NNNNNNNNNNNNNNNNNNNNNNNNNNNNNNNNNNNNNNNNNNNNNNNNNNNNNNNNNNNNNNNNNNNNNNNNNNNNNNNN
NNNNNNNNNNNNNNNNNNNNNNNNNNNNNNNNNNNNNNNNNNNNNNNNNNNNNNNNNNNNNNNNNNNNNNNNNNNNNNNN
NNNNNNNNNNNNNNNNNNNNNNNNNNNNNNNNNNNNNNNNNNNNNNNNNNNNNNNNNNNNNNNNNNNNNNNNNNNNNNNN

NNNNNNNNNNNNNNNNNNNNNNNNNNNNNNNNNNNNNNNNNNNNNNNNNNNNNNNNNNNNNNNNNNNNNNNNNNNNNNNN
NNNNNNNNNNNNNNNNNNNNNNNNNNNNNNNNNNNNNNNNNNNNNNNNNNNNNNNNNNNNNNNNNNNNNNNNNNNNNNNN
NNNNNNNNNNNNNNNNNNNNNNNNNNNNNNNNNNNNNNNNNNNNNNNNNNNNNNNNNNNNNNNNNNNNNNNNNNNNNNNN
NNNNNNNNNNNNNNNNNNNNNNNNNNNNNNNNNNNNNNNNNNNNNNNNNNNNNNNNNNNNNNNNNNNNNNNNNNNNNNNN
NNNNNNNNNNNNNNNNNNNNNNNNNNNNNNNNNNNNNNNNNNNNNNNNNNNNNNNNNNNNNNNNNNNNNNNNNNNNNNNN
NNNNNNNNNNNNNNNNNNNNNNNNNNNNNNNNNNNNNNNNNNNNNNNNNNNNNNNNNNNNNNNNNNNNNNNNNNNNNNNN
NNNNNNNNNNNNNNNNNNNNNNNNNNNNNNNNNNNNNNNNNNNNNNNNNNNNNNNNNNNNNNNNNNNNNNNNNNNNNNNN
NNNNNNNNNNNNNNNNNNNNNNNNNNNNNNNNNNNNNNNNNNNNNNNNNNNNNNNNNNNNNNNNNNNNNNNNNNNNNNNN
NNNNNNNNNNNNNNNNNNNNNNNNNNNNNNNNNNNNNNNNNNNNNNNNNNNNNNNNNNNNNNNNNNNNNNNNNNNNNNNN
NNNNNNNNNNNNNNNNNNNNNNNNNNNNNNNNNNNNNNNNNNNNNNNNNNNNNNNNNNNNNNNNNNNNNNNNNNNNNNNN
NNNNNNNNNNNNNNNNNNNNNNNNNNNNNNTGGCTCTCCTGTATTTTACACCCATGTTTAAATGTTTGCATCCCAATTCC
ATGGTTACTCAAAAGTCATTAGGCCTATGTGAGTATCACATGGACATTACATGTGAAGATTTTCTCTCTCTGTCTGTCTG
TCTGTCTGTCTGTCTGTCTGTCTGTCTGTCTGTCTGTCTGTCTGTCTGTCTGTCTGTCTGTCTGTCTGTCTGTCTGTCTG
TCTGTCTGTCTGTCTGTCTGTCTGTCTGTCTGTCTGTCTGTCTGTCTGTCTGTCTGTCTGTCTGTCTGTCTGTCTGTCTG
TCTGTACAGATGGACAGCATGGAGCACCAGCCAGCCACTGAGCCAACCCAGTCATGTCAATTTTACAGTAGTTTGATACA
GCAGCTCCTGTCAAGCATGGGCATTCAATTTAAAGAGACAGTGCACCACCAAAGCGCTGACTGCAGCACTCCTCTCATGT
GACATAAGGGGCTGTCTTACACCCTCCGCCAATCGAGCTGTGCCAGGAACCATCATCTGTTAGGCTATTACTGTACTGTA
TGCTCGAGATCTACAGTGTTCAATGATCTGTCAGGGGAAAAGCTGATTCATTTTCTGTTTGCGCCGGATTGAAGTGTTAT
ATTGAATTAATGCTAAAATTAGTAAATGATGCAACTCATTAGATACCATCTTGTCTGATGTCAATTCATTGCAGTGATTC
TGGCATTTTGTGAGTTAAAACCTCTTACATCTAGACGTTCCGCTAGCGGAACACCTGCTCCAATATCCAATGATGGGCGT
GGCGCGAAATACAAACTCCTTTAAAATCCGAAAACTTCAATTTTTCAAACATATTACTATTTTACAGCATTTTAAACACA
AGACTCTCGTTAATCTAACCACACTGTCCGATTTCAAAAAGGCTTTACAGCGAAAGCAAAACATTACATTATGTCAGCAG
AGTACCCAGCCAGAAATAATCAGATACCCATTTTTCAAGCTAGCATATAATGTCACAAAAAACAAAACCACAGCTAAATG
CAGCACTAACCTTTGATGATCTTCATCAGATGACAACCCTAGAACATTATGTTATACAATGCATGCATGTTTTGTTCAAT
CAAGTTCATATTTATATAAAAAAACAGCTTTTTTACATTAGCATGTGACGTTCAGAACTAGCATACCCCATGCAAACATC
CGGTGAATTTACTAAATTACTCACGATAAACGTTCACAAAAAACATAACAATTATTTTAAGAATTATAGATACAGAACTC
CTCTATGCACTCGATATGTCCGATTTTAAAATAGCTTTTCGGTGAAAGCACATTTTGCAATATTCTCAGTAGATAGCCCA
GCCATCACGGCTAGCCATTTAGACACCAACCAAGTTTAGCCCTGACCAAACTCCGATTTACTATTACAAAAGTTTGATTA
CCTTTGTTGTCTTCATCAGAATGCACTCCCAGGACTGCTACTTCAATAACAAATGTTGGTTTGGTCCAAAATAATCCATC
GTTATATCCAAATAGCGGCGTTTTGTTTGTGCGTTCTAGACACTATCCGAAATGGTAAATCAGGGTTACGAGCATGGCGC
AATTCGTGACAAAAGATTTCTAAATATTCCATTACCGTACTTCGAAGCATGTCAACCGCTGTTTAAAATCAGTTTTTATG
CCATTTTTCTCGTAAAAAAGCGATAATATTCCGACCGGGAATCTGCGTTTCGGTAAACAGAGGAAAGAAAACAAAGCATT
CGGTCGACGCGGGCACGAGCCTGAGTCTCACAGTACTGTAACCAGCCACTACCCAAATGCGCTACTTTGTTTCAACCAGA
GCCTGCAAAGCCATGATTCAGCTTTTTGCCGCCTTCTGAGAGCCCATGGCAGCCGTAGGAAGTGTCACGTAACAGCAGAG
ATCCTTTGTTATGGATAGAGATGGCAAAGAAGGCCAAGAAATGGTCAGACAGGGTACTTCCTGTACAGAATCTTCTCAGG
TTTTGGCCTGCCAAATGAGTTCTGTTATACTCACAGACACCATTCAAACAGTTTTAGAAACTTTGGAGTGTTTTCTATCC
AAAGCTAATAATTATATGCATATTCTAGTTTCTGGGCAGGAGTAATAATCAGATTAAATCGGGTATGTTTTTTATCCGGC
TGTGAAAATACTGCCCCCTATCCATAACAGGTTATTAAAGTGCCTGCTGTCCAAGTTTCGTGAAGTTGGGAATTCAAAAA
ATGCCAACTTCCCTCAAGACCTGAAAAGTCAACATTCAAGAACGTCAAGCGTTACTATGCCTCAGCCTTTTTCAGTGATG
CTAACAGCTAGCGTATACATGAAAGCAAAGACAATGTCGGTCGATATCCTCAAGGAAATATCGTATTCATAATTTCGGCC
CACAGACAAAGTCAATCAAAGTTAAACTGCCCCTCATTTGCTTGTATCTGCATACTGTTTAGCACATTCATGGCACAGTG
CAGCGGACGTACTGTACGAATAAACACAGGAGGTGGCGTAGAGCAGTTTGATGGCAAGATTATTTATGCATATTAACATT
TACTGGCTGATGCTTTAAGGTCTTTGGTTTTGCTCTAGGTCAACCAATGACTTGCCTCTATCATAAAAAGTGTTCATATT
GCACCATGGACCATGACGCTATACGGCACAGTGAGATAGGGTCCAGATTGAAACATGTTCCAGGTCATAAAGTATCATGC
CTTGTGTTAGATAACCATACTTAAGTTAAATTGTCCACTGCAGCATGTCTCATTCTACCAAAGCACAGTATAAAATGTAC
ATGGTTTATTACAAAGTACCACCGTGCTCCAATGTCCTTGATGGTGTCTGAGTGTGACATGATAAAGTGTCCTCTGGGTA
GTGTCAGCAAAAATGTTATTTTGAGGGCTGCCCCCCATAAGCCATGTTGTCTGGGGGGAAGGACATGTTTGTTACGGTGG
AAAGACCTTCAAATGATATTGTAATAATGCTATTAGCAGTAGTCCAAAAATTAAATCAGACAAACTGAATGTTTTTGCTA
CTGTAGTTTGCTTGCGTACTGGCTGAGCTCATTAATTATAATACAATTCACGTTTCCTGGCTCAAATTAAGATCCTACAT
CTGTAGTCTATGAATATAGGGTCATGTCAGGGAACTCTCTCCCCCTTGAACAGTTCTAGTACAGTGAGTTCAGGCATGCC
GCTAAGCCTTTGTCCACGGCTCCACTCCCTTACTATCTCTCTGTTCCCCAGCATTACTGACTGAACCTGGTGGTGAAAAG
GCCCAGATAGACCCCATCCACTAAGAGCCTTACTGCTGATTCTGAGGAAATCTACATAAGGATCCCATTAGAAATGAGTA
GCTGGGAGAAATGGTTGCTTAGTGCAGACCACACCATGGGAGGGGGGCCCACTTATTCAATTCCACCGCAAAGCCTGGAG
CCCAATGACTCAATGAGTTTGTGTTTAAAGGATGCTCGTCTTTTGATTGATCCTGTCAGGATTCACTCATGGCGAAAGGT
CAGGATGAGCTATCTCCTCTCCTAAGCCAATCGGAGGGCTGCCAATCCGGGATAATGATGTCCCTGCGGATGGTTGGCTC
ACAGGCCTTTCAGACATTATGTCATTTGATCCAACAGATTCTCATGATGCACGACAAGTTGTGCCTTCAGACCTGGAGAT
ATTCAACAGCTTACTGTACATATGTCACTATATTAGTAACAGACAAAAAAATAATATTAGTCATGGAGTCAAAATGGAGA
AGTTATTGGATGTTTGTAGGCACGATTGAATGGCATAAAAAAGCTGATTATGCCTAAAAACAGCAGGTTTCACTTGCATA
TGTATCATTAAAAAATAACATTTAGTTGTTAAAGGTGTGATGTTAGGTTATAATGTACAGGTAGGATCTTAATTTGATTA
CCCTGTTGCAGGAGAATTCTCCTGCCCACATTTCAGTTGAAGGCATTCAGTTGTACAACTGACTAGGTATCCCCCTTTCC
CTTTCCCTTTCCAAATGCAGGAAATGTAAAGCTTGTAGTGTATTTGACGTTTAAAAAGGCTTTTGTAATTTCCACTTAGA
AAATTTCCCTTACGAAAAATGCATCAACCCCAAAACCGGTTGCTGCAGGATTATTTTCCTGCTGTAGCAAACTGGCTCAA
ATTAAGATCCTTCATCTGTAAGATGAAACATGACTTTTATGTTTTTGATTTATACACATACTGTACATTAATATTATTTA
TGAGCAATTAAATTATTTGCACAGTATAGGGCTTTTTCAAACAACCCTCTCTGACCCTTTTCTGTCAAAGTATCTAAAAT
AAATGTCCTGTGATAGCTTTTGGGAGAGTATGAATTCCACACCGTGTCAATAGAGATGCTGATAGTACAGCCTGTGATAA
GTGCCCCCTAAAAGAACAGATTATTAAATCACTACTGTGGTGCCTCAAGCAGGATTACAAATTGGAGAGTTGGGCTCACA
CAGATAGTGCTCACTGAAGCCTTGTACCTGGGACCCAGAGTTACCATGAGAACTAGGAGATATTGCACTGTTGGGTAGAT
AATCAGTCACATGTTGAGATGTTCAGTCCCAGACATTTGTAGTCTATTTTCAGTGTTATGTGTACATTTCACACTTTCTT
GTAAGTCAAGGGGGTAACACGATGTAGGATGTCATAATGTATAGACTGCATACATATTATCCCTAGGTCAAATCAGGCCA

TGACAGTAGCAGATAACCTCTCATTACTCATTGATGTACTATAGAACTTGACATTTGAATGTTAGTTTGTTTTCTGATAT
GGCGTAAGGTTTAGGAGAGTTACAGTAGCCAGTCATGATGTCATAATGTGCTGACAGGACTTTACAGTCATCTTATTACA
GTGTTATAAAGGCAGACGGCTAAATCAAATGGGATCTCAAACCTTCAATATCATGCTAGCGCTGGGCTAATTTGTCCCCC
CCTCTCTTGTGATGCTAATAGGTGGCTGATATATGCAGTGTCAGTGTGCACTTTTACTGAAGGTGGTGAGAAAAGAGGAC
CGAATTTTCACGGGATGTACCTCTCATTCACTTCAGATGCACAGATACTGCAGAGAAGAATGGTGCATTTTAATTAGCTG
TAATCAAATACACTGGATATGCTGATATTCAAAAAACAGGGGCCATCGGACCCTGACATTTCACGAGTAGTCTATTACCA
TTTTAAAGTGTTTCACTATTACACATTTAGGGTAGTGTCTCTACTTCAGAAGAAAAAAATCATTTACAAGGCTCTCACGT
ATGTGTGTGTGTGTGTGCCGTGCATGTGCCGTGCATGCTTTTGTGTGTGTGTGTGTGTGTGTGTGTGCATGCGTGTTT
GTGAGCGTGCTTGTGTACAGATGTAGGATATTAATTTGATCACCCTGTTGCAGGACCACTTGTAGTGTATTTGAGGTTTA
AAAAGGCTTCTGAAGTTTGTAATTTCAGAGTTGATTTTCCCTTATGGGAAGATGTATCAACCCACATAATAATACACATT
TCCTGTCGCTGCAGGATTATTTTCCTGCTGTAGCAAACTGGTTCAAATTAAGATCCTACACATGTGTGTGCATGTGTGTT
TGTTTGTAGGTGTGTGTGTGTGTGTGTGTGTGTGTGTGTGTGTGTGTGTGTGTGTGTGTGTGTGTGTGTGTGTGTGTGTG
TGTGTGTGTGTGTGTGTGTGTGTGTGTGTGTGTGTGTGTGTGTGTGTGTGTGTGTGAGAGAGAGAGAGAGAGTCAGTGTT
TTTATTTGAGTGATTAACTTCTACTCTGAAGACCTCATGAAGGTTAGCGCGCTCACTCTGCATGCCTGGCGCTATGTTTA
GCATCTCATCTCCACCACATTTGAATCTTCACCACACAAAATATGACTGCATTTCAACTGTATTTGCTTCCAAAGAAAAT
TCTGGCATTCATGCAATGTTTATGTGATATGCCCTTTTGTTATTTCAAATAATGATTCATCTAAACTGGAGTGGATTGTT
GAGAGAGACTGGAAATGCAATCCACTTTCTAGTGTTTTTTATGTAAGTTTTCTTTTCAGGAGGAAAGCTCCTGTCCTAGT
GGTTTTCAGAGAGCCTATTTTACAGTTGTTGCTCTGTTTGGCATTTCAATGTGGAGTTGAGTTTCTCTGTATGAGAGTGG
AGATAATGGCAGAACATCCTGATCTCTTTGATCAGAGGCAGTGTCTAACTGGTAGAAATGGACTGCTGGGAGAGGAAGGT
GTGAGACACCTATGAAACACTTTTAATTGTAGCTTCTCGCTTCAATGTCATGTTTGAAATCTCCTTTCCTCCATGAATAT
ACTACACTGGAATTCACACTCACTTTAAAACACAGAACTGGCTTCAAAGTGTCTCACTTTGTTTGACCTACCGAGTGGTG
AGTGTTCATATTATCTAGGGGTGAAAAGTTGACTGGGATTGTTGTTGACTNNNNNNNNNNNNNNNNNNNNNNNNNNNNNN
NNNNNNNNNNNNNNNNNNNNNNNNNNNNNNNNNNNNNNNNNNNNNNNNNNNNNNNNNNNNNNNNNNNNNNNGAAAAAAAAT
TGTAGACCTCCACAAGTCTGGTTCATCCTTGGGAGCAATTTCCAAATGCCTAAAGGTACCACATTCATCTGTACAAACAA
TAGTACGCAAGTATAAACACCATCCGACCACTCAGCCGTCATACCGCTCAGGAAGGAGACGCATTCTGTCTCCTAGAGAT
GAACGTAGTTTGGTGCGAAAAGTGCAAATCAATCCGAGAACAACAGCAAAGGACCTTGTGAAGATGCTGGAGGAAACAGG
TACAAAAGTATCTATATCCACAGTAAAACAAGTCCTATATCGACATAACCTGAAAGACCGCTCAGCAAGGAAGAAGCCAC
TGCTCCAAAACCGCCATAAAAAAGCCAGATTACGGTTTGCAACTACACATGGGGACAAAGATCGTACTTTTTGGAGAAAT
GTCCTCTGGTCTGATGAAACAAAAATAGAACTGTTTGGCCATAATTACCATCGTTATTTTTGGAGGAAAAAGGGGGAGGC
TTGCAAGCCGAAGAACACCATCCCAACCGTGAAGCACGGGGGTGGCAGCATCATGTTGTGGAGGTGTTTTGCTGCAGGAG
GGACTGGTGCACTTCACAAAATAGATGGCTCCATGAGGAGGAAAGTTATGTGGATATATTGAAGCAGCATCTCAAGACAT
CAATCAGGAAGTTAAAGCTTGGTCGCAAATGGGTCTTCCAAATGGACAATGACCCCAAGCATACTTCCAAAGTTGTGGCA
AAATGGCTTAAGGACAACAAAGTCAAGGTATTGGAGTGGCCATGACAAAGCTCTGACCTCAACCCAATAGAAAATCTGTG
GGCAGAACTGAAAAAGTGTGTGCGAGCAAGGAGGCCTACAAACCTGACTCAGTTACACCAGCTATGTCAGGAAGAATGGC
CCAAAATTCATCCAATTTATTGTGGGAAGCTTGTGGAAGGCTACCCAAAACGTTTGACCCCAATTAAACAATTTAAAGCC
AATGCTACCAAATACTAATTGAGTGTATGTAAACTTCTGACCCACTGGGAATGTGATGAAAGAAATAAAAGCTGAAATAA
AAAAATTTCTCTACTATTATTCTGATATTTCACATTCTTAAAATAAAGTGGTGATCCTAACTGACCTAAGACAGGGAATT
TTTACTAGGATTAAATGTCAGGAATTGTGAAAAACTGAGTTTAAATGTATTTGGCTAAGGTGTATGTAAACTTCTGACTT
CAACTGTATATATATACAGTACCAGTCAAAAGTTTGGGCACACCTACTCATTCATGGGTTTTTCTTTATTTTTTACTATTTC
TACATTGTAGAATAATAGTGAAGACATCTTAACAATGAAATAACACATATGGAATCATGTAGTAACCAAAAAAGTGTTAA
ACAAATCAAAAATAGTCACAGCCTGGAGGTGTGTTGGGTTATTGTCCTGTTGAAAAACAAATCATAGTCCCACTAAACGC
AAACCAGATGGGATGGCATATCGCTGCAGAATGCTGTGGTAGCCATGCTGGTTAAGTGTGCCTTGAATTCTAAATATATC
ACTGACAGTGTCACAAGCAAAGCACCCCCACACTATCACACCTCCTCCTCCATGCTTCACAGTGCGAAACACACATGCAG
AGATCATCTCTTCACCTACTCTGCGTCTCACAAAGACACGGCGGTTGGAACCAAAAATCTCAAATTTGGACTCATCAGAC
CAAAGGACAGATTTCTGCCGGTCTACCACCCATTGCTCTTGTTTCTTGGCCTAAGCAAGTCTCTTTTTCTCATTGGTGTC
CTTTAGTAGTGATTTCTTTGCAGCAATTCGACCATGAAGGCCGGATTCACGCAGTCTCCTCTGAACAGTTGATGTTGAGA
TGTGTCTGTTACTTGAACTCCGTGAAGCATTTATTGGGCTGCAATCTGAGGTGCAGTGAACTCTAATGAACTTATCCTCT
GCAGCAGAGGTAACTCTGGGTCTTACTTTCCTGTGGCGGTCCTCATGAGAGCCAGTTTCATCATAGCGCTTGATGGTTGA
AGTTCTTGAAATTTTCCGCATTGACTGACCTTCATGTCTTAAAGTAATGGACTGTCGTTTCTCTTTGTTTATTTGGGCTG
TTCTTGCCATAATATGGACTTGGTCTTTTACCAAATAGGGCTATCTTCTGTATACCACACCTATCTTGTCACAACACAAC
ACATTAAGAAGGAAAGAAATTCCACAAATTAACTTTTATCAAAGCACAACTGTTAATTGAAATGCATTCCAGGTGACTAC
CTCATGATGCTGGTTGAGGGAATGCCAAGAGTATGCAAAGCTGTCATTAAGGCAAAGGGTGGCTACTTCGATGAATCTCA
AATCCCAAATATATTGTTGGTTTACTACATGATTCCATATGTGTTATTTCATAGTTTTGATGTCTTCACTATTATTCTAC
AATGTAGAAAATAGTAAAAATAAAGAAAAACCCTTGAATGAGCAGGTGTGTCCAAACTTTTGACTGGTACTGTATATAAA
GATCCCTCCATGCCCTGGCCTATATTCACGGCCTATATTTTGTGATTTGGTCAAATTCTTAATGATATGTGAGTGTATAT
GAAAACCCCTAGGGGGCTCAGTCACAATAGCCACTGTGACTGTGAGTCCATGAGTGAAAAGTGACTTAGAGGTTGAAATC
AAGGTTATGTAATCTCTCTTCATATCGTACAATGAATCATACCTTGGATTCCACCACTGTGATCCCCTTAATAAGCTTTG
CTTGTTTAGCAACTCATTTATATTTCAAAAGCAGCTTTATTCATAATTAAACTTGAATATAAAACATAAACTCAGCTGTA
TTCGCTACAATTAGACAGATTGCCTTTTGTGCTTGCCTTCCTACTTGGTAGGATTGTGAATACAGTAAATATTTGAGAAA
GCTTTTATTGTAAAATGAAGGTTCTGACAGGGTTGTTTGGCTGAAGTAGGCCTATTTTCAGGGTCGGAGTGGCTCTGTTT
TTGGCATAGTGTCAAATGGACAGCATGGCACGCTGCTGTGAGTGTGCCGACGATGCCGTGTCTCAGCCGAGTCAGGGCCT
GTTACATCATTCAACCTGCAGGGGCCTTCCTTTCTCAAACATGGGGAAATATGCAAAGGGTTAAAGTGTGCTGTCTGGAA
AGTGAAAAAGTAGACACTAGATAGCCATTGTTTGTACCATCTACAAGATGTATGGGTAAGGGTTAAATAAAGTGTGTTAG
AGGGTTTGACTCTGGGACTTGTACAATCTGCAGATATGATTGGTGTGAATTGTGAAAACAGTCTTGGTGTGAGGAGTGGG
GAATGTCACTTTGGCTAACCTCTCGTTGAGGGCTTGTTTCCGGCCTTGTTGGGGGCGTCTGTGAATTCACAACATAAGGC
TGTCTGGAGTGAGAATAGCGTTGAGATGTTTGCTTAACATTCTGGTTGAAATGTAAAAACGAGGCGAAGGAAAGAGATAT
CAAAAGTGTTTTGATGAAAACATTAACATAGCCCCTGACATAACTTAGAGGGAAAAGAGAAAGAAATATGAAAGAGGGGA
ACATGGAAAGAGACTGGAGGAAAGACGAATAGGAAGGTCAATACGAGAAAGACATGAAGTGATGGAGACAGATGAGAGAA
GGAGAAGCAGATGGATGGGCAGAGTGAGTAGGAGTGATAGAGAGACACACTAGGAAGTGGAGACTACGAAAGATAGTGAA

AAAGACAAACAGCCATGCCCTGGTTATTTCTGTGTGACCTAGCATCATGCTGACTGAAGCCAGAGCTTGGCTAATTGACT
TCAGCAGTTGTAGTGGATCACAAAGCTTATTTTGCTCTGTTTCATGCCACGGCCCTTCTGCTTCAGGACCCAGGGTCTAT
CTGAAGAGCTGCCTGTTTGTTACGGCCGTCTGGTCAGAGGGGGAACCTGGGGACGAACAAAGGCGTGTCCAGGCCAGAGG
GATTATGAAACTGATGGAATTAGCGATGTAGTATGAAGATATGGTCATATAGCACAGGGTTTTCTGAACTTTCTGGGTCC
GGGGACCCCTTTTGTGATAGCAAATTCATTAAGGACCGCCTTATAATCATAACACAACTCGCGGTGCGATAAAAATGGCA
TAAAAGGAGACGTTTTACTGTGCTGTCTGCAGTGCATGGCTGGCCGAACCAAGTCTCGGCCCCAGGGAAAGAACATTTAA
ATAGCCCGCCTCTTGGCATCGAAGATATATTTTTTCCGTTTTCAAGCGAATTTCATGCAATTCATTTTTTTTTATATCAT
AGGCAGAGAGAAAACTTTGCAGTTTTAAAGATTACATTAAAGAACATTCATGTTCAAAACAAAGAAGTATTGAGTTGAAA
AATGAATAATTGGTGGATATCCCTGTGAGCCTCCCAGGCCTTGATGCCCATGGTTAGTAAGAACCTACATTGTAGATTAA
TAATAATGACATTTTATTGTATTAGACCCTCTAAACGAATCCCTTGGCCAAAATGTGTTTCATCAGTTATTGTAACAATA
TTCATTAAAAAAAAGTTATTATTTATTTTATACATATTTAGAGATCTGGGCACGGCAGTACCTCTTCCGCAGACCCCATT
TTGGGAACCTCTGGTGTAGCAGACCCTTTCATCCAATCCTTTTATCTACATTTTAGGCTATGGTTACAAATCGTAATAAT
AATCCAACTCTGATTGGGGAGTTATTTACTGCGTAATGGACCAGTCCTAGATGAGTCTGTCCACTTCTTTCATTGAGCAT
TGTTGTTATGCGCGAAATGTGGACAACATGTGAAGAAGTCACATTGCATTTTCAGTGCTTTGAATTCAACTGATGCGGAC
GTAACAGCTTGCAAACATAAACAAGTTAACATCAACCATCTGACGACGGGCATGTTCTGGCATGTCCTCCACCGTCGAGT
TACCCTGACTGACCCCTCTCCCCTCTCTCTGTCCCTCTCCCCTTGTTCTCTCTCCTCCCCAGATGCGAGGTGTGCCGTCC
CCCCCTCCTATCGCCCCCATGCCCCTTCCTTTGCCGCTCCCTCCTACGACCGCCCCGACTGGAACCCCCGTCTCTGTGTG
ATCTCCGGAAATCAGCTCTACATGTTGGACCAGGAGGAGGTACTTACCTACTGCTGCACACACTATAGAAATACAATCCC
ACCCATCACCCACCCATTGACTTGAAAGGGGAATACCGTTCTAGGAGTAATATTTCTATCTATTCTATGGCTCACATTGC
TGTATCATACTGTATTAGACTGGGTTGTTTGACATGGCATGAGTGTCTACAACACTGTCATGACAGTTTCTAAAGCCACA
AAGTTTAATAAAGAATGACATATCCAAAAAAGGATATCACCACAATTGACCCTTTAGGCGTGTAACTTTATTGACAGANN
NNNNNNNNNNNNNNNNNNNNNNNNNNNNNNNNNNNNNNNNNNNNNNNNNNNNNNNNNNNNNNNNNNNNNNNNNNNNNNNN
NNNNNNNNNNNNNNNNNNNTACGCCTCTATACAAGTAGGTCACGTCTGGCTAAGATGGGCATTGGGCCCAGTAACCGAAAG
GTCGCTGGTTTGAATCCCCGAGCCATCAAAATACATATTTTGGTTGAATGCATTCCGTTGTGCAACTGACTAGGTATCCC
CTTTCCCATCTGAGTGCTGATCTAGGATCAGTATATATTTTAGATCATAACAGACATAACACGATTACATGACCAGGTGG
GACCAGATTCTAGATCAGCACTCCTACTCTGAGACACTTTAGGAATACGGGACTTTTTCAACCTGACTGTTTATTGCTAA
TTAAAAATATATATATATATACCTTAGATTACTTACTATATCATACTAAGTAGAGATTCAGCCTGCTAGCTAAGACCAGA
AGGGGGTACTATTCCTTCACTTTTGTACCTGTATCGCCTTTGGACAGGTCTCTTTTTAAATATAGACCTTGCAGACAGAT
TTACAGATTTATATCCAATAAATGAATCCTAAAACACAATTAAAACACATAGAGGTTTACTTACACACTTAAAGTCTAT
TACACTACAATAACAGTGTTCATAGTACATTTGATTGAGAGTATGAGTCTATTAAAGATGATGTAATGATGTAATGGAGT
ACTGTAGGGGTACTTGCAGGGTAGCTAGCTGCCAATATCTTTAATTCTCGTCTCTCAGAAGGAGGATAAAGAACCTTATG
ATCCAGATAAGGGCCTTTTCAGCTCTGGAGTAATGAGAAGAAGGCCCAGGTCAATTAATCAAAGGCTTATCCCTGTCTAA
TTGAGGCAGAAGCTATGAATGGTTTGAGACAGACCTGACGTGCCGATAGCCACCACATCGGAGAGAGGATGAGAGGCGAT
TAAATATTCATGCCTGTCACTCACCTCGTGCGGTGCGCGCGTCTCTCTCTCTCTCTCTCTCTCTCTCTCTCTCTCTCTCT
CTCTCTCACTCACTGCACCTCGTGCGGTGCGTGCGTCTCTCTCTCTCTCTCACTCTCCTTCTGCGGTGCGCGCGTCTCTC
TCGCTCTCTCTCTCTCTCTCTCTCTGTCTCATCCCCCCAGTTGCGAAATGGGTGTCCATCGGGTGATGCATGTCTAAAAA
ATGCCTACTACTCCTTGAGTCATGCTTTCCTCGTGCGGTGCGCGCGTCTCTCGTCTGCTGCATCTCTACTCTCTCTCTAC
TACTCACTCACCTCGTGCGGTGCGCGCGTCTCTCTCTCTCTCTCTCTCTCTCTCTCTCTCTCTCTCACTCACTCACCTCGTG
CGGTGCGCGCGTCTCTCTCTTTCTCTCTCACTCACCTCGTGCGGTGCGCGCGTCTCTCTCTCTCTCTCTCTCTCTCTCTC
TCTCTCTCTCTCTCTCTCTCTCTCTCTCTCTCTCTCTCTCTCTCTCTCTCACTCACCTCGTGCGGTGCGCGCGTCTCTCTCG
ACAGTAGTCTTTGGCTATCCCACAGACGTGCTCTCTCCTTTCTTTCCCAGGCAGCTCCATTGAGGTGCCACTTTGTTTTG
TGAATGACATCTGTCAAAAGACCCTTTTCTCTTCATTGCGACTTCTTCAGTCTCAACTAAGGGGCTGCTTGAGGGAATAA
CAGTGCTCTGGAGGAATATGTGAAAATATCTCANNNNNNNNNNNNNNNNNNNNNNNNNNNNNNNNNNNNNNNNNNNNNNNNN
NNNNNNNNNNNNNNNNNNNNNNNNNNNNNNNNNNNNNNNNNNNNNNNNNNNNNNNNNNNNNNNNNNNNNNNNNNNNNNNN
NNNNNNNNNNNNNNNNNNNNNNNNNNNNNNNNNNNNNNNNNNNNNNNNNCGCACCAGTGCCCTGTCCTCCAGTCATGTGCCC
AGGCATCGCTCTCCCTCCCACTGTGCCCCAGTGTTATGCAAGCAATCAGAGGGCACTGTTTCCTGCTAGCACAGCGCTAG
CACTAGCATGTTAGTATCCTCTCTCACTACTACTGAATCAAATTAGCATTAGCCTGCTAGGCTAATTCAAAATAAAGGGA
ATTAGAACAGGATTTGTTTCAGTATCAATGCATGGCCATTGTGTTGCATTCCCTGCCCATATGTTTGCAATGGCTTTGGC
AGTGTTGATACTGTAAATTAATTCATTAGCGCCCAAGCCAACAATGCAGCGCCGTGGTGGCTTTGCCTGTAGCTACTGTT
GTCACATTGCATAAAGCATGACTCAGGAGTAGTTAGCATTTTTACACATGCATTACCCTTGGGAACCCATTTGGAAACAG
GGGGGATGTGGCAATTATGTAATAATAAGGCACACATGGATGGAAGGAGTCATGAGGAAGAAGCTCCTTCATTTGACAAA
TAAGAAGGCTTAATGCACTATACATTGGCAAATTGTAATGTTATTTAACAAGCGCTTTACTGCCTTTTGGCATCTGAATA
ACCATTACGAGCAACTGAAGTAAGCCATTTTCTAAAGGTCACCCGTATACATATTAGGGGTTATACACTGAGTGTACAAA
ACATTAGGAACACCTTCATAATATTGAGTTTCACCCTTCTTTTGCCCTCAGAACAGCTTCAATTCATCAGGGCATAGACT
TTATAAAGTGTCGAAAGCGTTCCACAGGGATGCTGGCCCATGTTGTTGCCAATGCTTCCCAAAGTTGTGTGACGATGGCT
GTATGTCTTTTGGGTGGTGGACCATTCTTGATACACACAAGAAACTATTGAGCCTGAAAAACCCACGAGCGTTGCAGTTC
TTGACACACTCAAACCGGTGTGCCTGGCACCTACTACTATAACCCTTTCAAAGGCACAAATCTGTTGTCTTGTCCATTCA
CCCTCTGAATGGTACACATACACAATCCATGTCTTAATTCCTTCTTTAAAGTGTCTCCTCCCCTTCATCTACACTGATTG
AAGTGGATTTAACAAGTGACATCAATAAGGGATCATAGGTTTCACCTGGATTCACCTGGTCAGTCTATATANNNNNNNNN
NNNNNNNNNNNNNNNNNNNNNNNNNNNNNNNNNNNNNNNNNNNNNNNNNNNNNNNNNNNNNNNNNNNNNNNNNNNNNNNN
NNNNNNNNNNNNNNNNNGACGTTTCTCTTTGATTTTTATCACATGCCAGTAGCTGTGTCCAATGCCTGAGGCGAGGGAAC
ATCTGTCAGTGATAGGATGTTATTGAGAGACTTGAGGACAGACAGTGGTGTTGGCCAGCCTAACCATTTTCCATACTTCC
AGGGACACAGAGGCTAGTGTGTGCGTGTGTGTTTGTATGCGTGTGTGAATGCGTCTGTGTGTGTGCGTGTGTCGGGGGCA
GAGGTAGTGGACTCAGCCGCTGTTTGATCACGACTCCGTTACAAAGTAATGGATTCACACCTGCCGCTCCATTAACCCCC
CCCCCCAGACACACACACCACCCATATCCCCCACCCAGCCTACCCATCACAGCTGGGCCAGTAGTGACATATGGTCCAGG
CAGCCTGCAGCATTGCTCAACTCTCCAAGCCAGGCCCCCTCTCCTCTCCCTCTCACTCAGATCTATGGCTTTCATTCTCC
CTCTACTCACCCACAACCCACCATCCACCCCTCCTTGCAGTGCCTGAGCACAGTCATCTGTCTATTTCAGTCTTAGCCAC
TTCTGCTCCTACTCTTACCTCTGACTACAGTTTGCTGGATGCCGAAATCCAGTACAATTGACCTTCTTGTTAGCTATTAG
AATGACCTTATGAATGAGCTTATCTCTGACAACACTCAAGGGAGCCAAGGTCTGCATGCCAGTATTCAATCTACTTTTCA

ATTGACTGAAATGGATTGTTGACTCGCCCATACAAAAACCAGAATACCCAGAGACAGAAACTCCAGGACGGAAATTGGGA
AACACTGCTATACTACGTTACAATTACAGACGTTTATAGACAATTAGCATGCTTTCTTGCTCCCTGCAGGTTTCAATGCT
AGACCACTGCCCCTGTGTGAGTGTCAGTGCGGGCCCCACATGCCCTCTGTCTGGTCTGCCAGTGTTGTGTGCCAACCTGC
CTTGTGTGCCCGCTCATGAGCATGCCTACCAGGGACGGTGCCAAGGCCGTGTCAGGTCCCCAGCTGCAGCGCCACTGTCC
TAACCAGCTCCCTCTGGAGACAGATGGCTCATATTATTAACATGTTAGAGAGTATATCTGTGTTAGAAGGATATTCCTAG
AGTGTTATGGTCCAATAATGAAGTAAATTAGATTATATTGAGAAATAACCAAAAATAATGGGAATGTTATAAACAGATGT
TAAAATTTGGTCTGACGCATTTAACAGTGTGCTCGCAGTGTATCATAAAAAATATATAAACAGCAAAGCAAAGTCTTTCC
GTCCATATAATTGCTCAAATAAAAGGGCAGGCGATAAAGAAACAAGCTTTTATGCATGCGGGTGCTGGACTCATAAAGAT
TGGGTGTCTTATAATACATTTTTCATTCAGGAACTCATTCCGTAACTAGCAATATATCTGTCCTGAGCGTAAGATGCATC
TCACAACATACTCATTAGTATGCACTAAGTGGAAAATACGAGCATAATCAAATACACACTTGATTTATGGTGTGTGTATC
CCTGATTACCCTTTAAAAAAGAGGTAAACGTCAACAAAACGAATCCTGCCACCGATAGCGCTGGGAGATGTCTTCCGGTT
ACTGGGCTATGGGGAATTGTATGGAGTGCTGTGCTCTGTGCTGTGCATCCACTAGATATGTCTAGCGCAGTAGACTGCAT
GTTATGTATGTTGGGGGGGTGGGGGGGTGGGTACTGTCGCCTGCAGTGGGTGTATGTATGTATTACACTTTTATGTCATC
CATATTGTGCTGGAAGCCCTGCTACATGTCTATAATTTACAGCTGGTCATACGGTTAAACGTGGTCATGTAACCAGCCGG
TGACCATCACACACATACAGTACCAGTCAAAAGTTTGGACACACCTACTCATTCCAGGGTTTTTCTTTATTTGTACTATT
TTCTACATTGTAGAATAATAGTGAAAACAAAACTATGAAATAACACAAATGGAATCATGTAGTAACCAAAAAAGTGTTCA
ATCAAAATATATTTTATATTTGAGATTCTTCAAAGTAGCCACCCTTTGCCTTGACAGCTTTGCACGCTCTTGGCATTCTC
TCAACCAGCTTCATGAGGTAGTCACCTGGAATGCATTTCAATTAATAGGTGTGCTTTGTTAAAAGTTAATTTGTGGATTT
TCTTTCCTTCTTAATGCGTTTGAGACAATCAGTTGTGACAAGGTAGGGGTGGTATACAGAAGATAGCCCTATTTTGTATG
GGAAGAACAACTCAAATAAGCAAAGAGAAATGACAGTCCATCATTACATTAAGACATGAAGGACAGTCTATCCGGAAAAT
GTAAGAACTTTGAAAGTTTCTTCAAGTGCAGTCGTAAAAACCATCAAGCGCAATGATGAAACTGGCTCTCATGAGGACCG
CCACAGGAAAGGAAGACCCAGAGTTACCTCTGCTGCAGAGGATTAGTTCATTAGAGTTAAATGCACCTCAGATTGCAGCC
CAAATAAATGCTTCACAGAGTTCAAGTAGCAGACACATCTCAACATCAACTGTTCAGAGGAGACTGCGTGAATCAAGCCT
TCATGGTCTGAATTCGCACACACACACACAAATTCCTTATATTAAGCAAACCAGACAGCAAAATGTTCTTGTTTTTTACG
TTTACAGATAGCCAGGGGCACACTCCAACACTCAGACATAATTTACAAATGAAGAATGTGTGTTTAGTGAGTCCGCCAGA
TAAGATGCAGTAGGGATGACCAGGGATGTTCTCTTGATAAGTGTGTGAATGGACCATTTCCCTGTCCTGCTAAGCATTCA
AAATGTAACGAGTGCTTTTGGGTGTCAGGGAAAATGTATGGAGTAAAAATAAACTAATTCTCTTTAGGAATATAGTGAAG
TTAAAGTAGTCCAAACTATAAATAGTAAAGTAAAGTAGAGATACCCAAAAAACTACTTAAGTAGTACACACACACATCAG
TACTATACCATGGATAGCCATATCATATTTAGCTTATGTTGACTGGACTCAATTGTTTTTGGAATCTTTTAGTTGCCATT
GTATTCAACTAACCATTAGCATAGGTGATTTGATTCTGTTGAAATGTAGAAGTTAAAATGGTGCTGGAATAGTGGAGGCA
GCTCCTGTTTTCTTTGCGACTTGCGGTAACTCTCTGTGGTTCTAAATCATTTCCATTTCCATTTTAGTCATTTAGCAGAC
GCTCTTATCCAGAGCGACTTACAGTAGTGAATACATACATTTCATCCATTTCAAACATTTCATACATTTTTTCTCCGTAA
ATCAATAGTTGTTTAGTGGTCCAAAAATGTCAGAAACATTAACTGCTTGACCATGCTGTAGGTCATGTAACTGTTTGTTA
CATGCAAAATGCTTTGTGGACTATACTGGACAGGGGTTGCTCTCTGGTTTTGTGATGAAACAATGGTGTGGTTGAATTTA
TTCAATTTATTCAACCACTGTGTCTTCTTATTTTTTCAGCTTTAGGCCTATATATCACGGTGTCAAGGCATATGAACTAA
CAGGTTATACAGCAAACAATGTAATTATCACAACACATACCAGTAGGTTGTAATATGGCGATTTTTTCCTGGCTTGGCTT
CCCCAGTTATTTTTGCCCACGCACCACTACTGCTCAGTGATATACATTTCAAGATAGTATCCATTATGGTCTTCAGAAAA
TATAGCCTAATGCATTGTGCGTATTGACCTGACCTTGTCTCTCTGTGCATATGCAGGTACACCCTTTGTTGATGCGCGAG
CGGCGCTCCGAGTTGCACAGGAACAAGCTGCTGCGGAGGACAGTCAGCGTTCCTGTTGAGGGGAGGCACCATCCAGAGAT
GGGTGAGTAGGCCATGGGCCCTTCTATCTCTCTGAATTCTTTCGGATAGAAATACAGTCAATAGAATGGGCTTGGAACCT
CTGACCTTAAAACACAAATTTAATGTTGTACTTCCTGCTATTACTTCCTGCTTTACTCTTATGGGGACCCTCGCAATGGT
TGCCAGTCCACCCATTATGGCGTCATTGACTTGAATGGGGAGGCCCGTTCTACTCATTCTAGTTCTGTGTTTCTGACTCT
ACCTTTCTCTGCCTCTCTACTTCTTATATTTTGTGATTCTGTCTTTCTTACTGCCTCTCTGACTCGTTGTCTCTGCCTCG
TTGCCTCACTCCACATCTCTCACTCTGCCTCTCTGTACAACGTCTCAGCATTGATACACTCCCATCGTCCACATAGGGCA
ACTCTCCCCACAACAGTCTTGTGGGGATACTGCAATAGGCCACTCCCTCCATATTTGACATGAACTGTCCAGGTCTACTG
ACTCCAGAAGAGTGATGGTCATCACACTCTAAGCATGTCTATGGTATAACATATGTTAGCCAACAGCCTTCCATTGAGAA
CCATCTGCAGTGAGAGCCCATCCATCCTGAGGAGATGCCCATACTGAATGGGATGGATGCCCCCCAGGCGGCCAAGGCCC
CCCAGGCAGCCCATTAATGAGCTGATTAACAGAAATCTGTCATGCTGTCATCAGAATTCCACATTTACGGAGTCAGTGTC
AAGGGAACTGCTTGTGACACACTGACCCATTTCAGAAAGACTGTGACCTACTGCATAACTTGGAACTGAGATAAGTTAGT
GAGATCATCATTGGGATAATCCCAGAGTGAGAGTAGATTTGGATGCAAGCAAGTGAGTAGATTGGATACAAGCAGAGTAG
ATTGGATACAAGCAAGTGAGTAGATTGGATACAAGCAAGTGAGTAGATTGGATACAAGTGGATTGGATGGGATAGTGAAA
ATCTGTGTATAAAAAAAATCTTTGAAAATCCAATTTTGACAAGACTTCAAAAAAGAAAAATAGGACACAAAGGTATGGAA
TCAAAATGACAGGCTAAACTGTCCAATGATATGCAAGATGGAGATAGAGTATTGTTTGCTATGGGGGATGAGGTCCAATA
GACCTTCCGATGTTGTCCTCTACTCACTTAAGTGCTTCATTCCAGGGACACATACAGTACAAGTCATAGATTACTCTTGT
GTTACAAAAAAACAAGATTGAATTTGTATGCATCAAATGTGTTTATGTACTGTCTCTATACTTCAAACGAATTTTCATTT
ATCATCGTTTTGATAAATGAAGACACTTGACAACAATCCCAGCTGGGTCCTGATGTAATGGCCATCTCTGATGAAGACAT
TTGGCTCTGCATCTATGATTAGGGACGCATAGGATATTTCAATACGCTCTTTGGATTGGATACGGAAAGTTACTCAGACG
CAATTAGTCTACTTCCCCATCCACTTCCTTCTATTGCACCTTGAAAGCACGGAAATGGTTAAAGTTGGAGCAATCAACAT
GCAATTAAGGCATTCATCTTGGTAGATAATTGCAGTGTGCTGTAAATGTTTTATGCAAATATCTATTGTAATTAGAACTT
CAACCTAAACCCTATCCACAGAACTTCCAACCCAAAATGTTTGCTCTGTGCGGAGCTGTCCAAGGTACTGAAACAGCTAC
CAATCTTAGTGGATTTTAGCCCATGTTGGGCCCTCTGTCCATGGTGTTCAAACACCCTATGTACCCTGGACACATTTAGG
TACATATAAGGTACAGTAGTTACAATAAGGAGACAAAGGCCTCCCGAGGGGCGCAGCAATCTAAGGCACTGTATCGCAGT
GCTGAGGTGCCACTACAGCCTGGGGTTCAAGCAGCACAGCTTGGCGGGTCATTTTTTGAAGGATGCATGTCTTGACATTC
GCCTGTCCCGAGCCCGTTGGGGAGTTGCAGTGATGAGACAAGATCGGAACTACCAATTGGGGAGAAAAAAAATAGGTAAA
AAAAAATGAATTATGAAGAAAAAAAAATGAATAGGGAGACAGATTGTGTGGGGCAGTAGTACCAATAGTGGAGTATAGGGA
ATCTGGCTTGAATAGCAGTTGCTGCATTATGGTAGAGGTACTGTATCTATGCTATGTTGAATTGTTGCAATTACAGTAGC
ATGCATAATGAAAACTATAAACACTATAGAGTAAAAAGTGGTAGTATGTTGTGTACTGCTGCAAAATGTTAGATGCCACG
TTTCTGAGTCATTCAGCCATAAAGGGAAGTTATTGAAACTGTGTAGCTGGGGTTGTTTCTGTAATGTAGGGCGTTTGTGT
ATTTACTGGCAGGGTAAGTTACATTTGTGTTTACTTGTCATTTTGGGCAGCTCAAAGAGCAGGTCTGACCTACCACAAAA

CCCCTGTTTACCATATCAGATGTTAACCAAGTGAAACTCAGGCTGTTTCAAGCAGACCACCATAGTCCCTGTGCCAAGGA
AGCAAAGGTAACCTGCCTAAATTATTACCGCCCCGTAGCACTCACGTCGGTAGCCATGAAGTGCTTTGAAACGTTGGTCA
TGGCTCACATCAACAGCATCCTCCCGGATACCCTAGACCCACTCCAATTCGCATACCGCCCAAACAGATCCACTCCACAC
TGCTCTTTCCCACCTGGACAAAAATAACACCTATGTGAGAATGCTGTTCATTGACTATCCCTCAATGTGAGCAAGAGAAA
GGAGCTGATCGTGGACTACAGGAAAAGGCACGCTGAACAGGCCCCCATTAACATCGACGGGGCTGTAGTGGAGCGGGTCG
AGAGTGTTAAGTTCCTTGGTATCCACATCACCAACGAACCATCATGGTCCAAACACACCAAGACAGTCGTGAAGAGGGCA
CGACAAAACCTTTTCCCCCTCAGGAGACTGAAAATATCTGGAATGGGTCCCCAGATCCTCAAAAAGCTGCACCATCGAGA
GCAACTGCCCGGCATCTGACCGTAAGGCGATACAGAGGGTAGTGTGAACAGCCCAATACATCACTGGGGCCAAGCTTCCT
GCCATCCAGGACCTATATAATAGGCGGTGTCAGAGGAAAGCCCATAAAATTGTCAGAGACTCCAGTCACCCAAGTCATAC
ACTGTTTTTTCTGCTACTGCATGGCAAGCAGTACCGAGGTGCCAAGTCTAGGACCAAAAGGCTCCTGAACAGCTTCTACC
CCCAAGCCATTAGACCGCTGAACAACTAATCAAATGGCCATCGGGCTATTTACATTGACATATTATCTATGCATAGTCAC
TTCACCCCTACCTACATGTACAAATTACCTCAATTAACCTGTACCCCCACACACTGACTTGCTACCGGTACCCCCTGTAT
ATAGCCTCGTTATTGTATTGTGTTACTTTTTTCATTTTTACTTTAGTTTATTTGGTCAATATTTTCTTATCTCTTCTTGA
ACTGCACTGTTGGTTAAGGGCTTGTAAGTAAGCATTTCACGGTAAGGTCTATACTTGTTATATTTGGCGCATGTAACAAA
TAAAGTTTGATTTGATTTGATAGAGCAAGGTCAGTGAAGGGACTTGATTATGTAATATAATGAGAGAAACAATCCTTTTA
AATCTTTATTGGCACTGAAATGCACTCTCCTGATCCTATTGGGTCTCTTGCAATAACGTTCAATAACATTCAAAGAGGTG
CATTCTGGGCTAATAGAAGGAAAATATTTTTTTCTCTCTCTTATTAACCTCATCAAAATTGGATGTGATTAAATGTGTTA
CATTTTGAAATTGACTTTCACCACGGCTGCGCAGTCATTGAGAGAAATCCATGTTATCTGAATGAATTAACCTCAGGCTA
AATCACAACCATAATGTAGCTAGCTAACTGCTTATCTGATTGATAGCCTTTGGCTGAATGGAAATTCAATGTAAAAACAT
AACGTGTTGTTCGTATTTTCCCCCATATATAAACTTGTGTTCTCCGTCTTCTGGGACAACATTGTCATTTTGATATTTTA
TTTATAGGAGTTNNNNNNNNNNNNNNNNNNNNNNNNNNNNNNNNNNNNNNNNNNNNNNNNNNNNNNNNNNNNNNNNNNNN
NNNNNNNNNNNNNNNNNNNNNNNNNNNNNNNNNNNNNNNNNNNNNNNNNNNNNNNNNNNNNNNNNNNNNNNNNNNNNNNN
NNNNNNNNNNNNNNNNNNNNNNNNNNNNNNNNNNNNNNNNNNNNNNNNNNNNNNNNNNNNNNNNNNNNNNNNNNNNNNNN
NNNNNNNNNNNNNNNNNNNNNNNNNNNNNNNNNNNNNNNNNNNNNNNNNNNNNNNNNNNNNNNNNNNNNNNNNNNNNNNN
NNNNNNNNNNNNNNNNNNNNNNNNNNNNNNNNNNNNNNNNNNNNNNNNNNNNNNNNNNNNNNNNNNNNNNNNNNNNNNNN
NNNNNNNNNNNNNNNNNNNNNNNNNNNNNNNNNNNNNNNNNNNNNNNNNNNNNNNNNNNNNNNNNNNNNNNNNNNNNNNN
NNNNNNNNNNNNNNNNNNNNNNNNNNNNNNNNNNNNNNNNNNNNNNNNNNNNNNNNNNNNNNNNNNNNNNNNNNNNNNNN
NNNNNNNNNNNNNNNNNNNNNNNNNNNNNNNNNNNNNNNNNNNNNNNNNNNNNNNNNNNNNNNNNNNNNNNNNNNNNNNN
NNNNNNNNNNNNNNNNNNNNNNNNNNNNNNNNNNNNNNNNNNNNNNNNNNNNNNNNNNNNNNNNNNNNNNNNNNNNNNNN
NNNNNNNNNNNNNNNNNNNNNNNNNNNNNNNNNNNNNNNNNNNNNNNNNNNNNNNNNNNNNNNNNNNNNNNNNNNNNNNN
NNNNNNNNNNNNNNNNNNNNNNNNNNNNNNNNNNNNNNNNNNNNNNNNNNNNNNNNNNNNNNNNNNNNNNNNNNNNNNNN
NNNNNNNNNNNNNNNNNNNNNNNNNNNNNNNNNNNNNNNNNNNNNNNNNNNNNNNNNNNNNNNNNNNNNNNNNNNNNNNN
NNNNNNNNNNNNNNNNNNNNNNNNNNNNNNNNNNNNNNNNNNNNNNNNNNNNNNNNNNNNNNNNNNNNNNNNNNNNNNNN
NNNNNNNNNNNNNNNNNNNNNNNNNNNNNNNNNNNNNNNNNNNNNNNNNNNNNNNNNNNNNNNNNNNNNNNNNNNNNNNN
NNNNNNNNNNNNNNNNNNNNNNNNNNNNNNNNNNNNNNNNNNNNNNNNNNNNNNNNNNNNNNNNNNNNNNNNNNNNNNNN
NNNNNNNNNNNNNNNNNNNNNNNNNNNNNNNNNNNNNNNNNNNNNNNNNNNNNNNNNNNNNNNNNNNNNNNNNNNNNNNN
NNNNNNNNNNNNNNNNNNNNNNNNNNNNNNNNNNNNNNNNNNNNNNNNNNNNNNNNNNNNNNNNNNNNNNNNNNNNNNNN
NNNNNNNNNNNNNNNNNNNNNNNNNNNNNNNNNNNNNNNNNNNNNNNNNNNNNNNNNNNNNNNNNNNNNNNNNNNNNNNN
NNNNNNNNNNNNNNNNNNNNNNNNNNNNNNNNNNNNNNNNNNNNNNNNNNNNNNNNNNNNNNNNNNNNNNNNNNNNNNNN
NNNNNNNNNNNNNNNNNNNNNNNNNNNNNNNNNNNNNNNNNNNNNNNNNNNNNNNNNNNNNNNNNNNNNNNNNNNNNNNN
NNNNNNNNNNNNNNNNNNNNNNNNNNNNNNNNNNNNNNNNNNNNNNNNNNNNNNNNNNNNNNNNNNNNNNNNNNNNNNNN
NNNNNNNNNNNNNNNNNNNNNNNNNNNNNNNNNNNNNNNNNNNNNNNNNNNNNNNNNNNNNNNNNNNNNNNNNNNNNNNN
NNNNNNNNNNNNNNNNNNNNNNNNNNNNNNNNNNNNNNNNNNNNNNNNNNNNNNNNNNNNNNNNNNNNNNNNNNNNNNNN
NNNNNNNNNNNNNNNNNNNNNNNNNNNNNNNNNNNNNNNNNNNNNNNNNNNNNNNNNNNNNNNNNNNNNNNNNNNNNNNN
NNNNNNNNNNNNNNNNNNNNNNNNNNNNNNNNNNNNNNNNNNNNNNNNNNNNNNNNNNNNNNNNNNNNNNNNNNNNNNNN
NNNNNNNNNNNNNNNNNNNNNNNNNNNNNNNNNNNNNNNNNNNNNNNNNNNNNNNNNNNNNNNNNNNNNNNNNNNNNNNN
NNNNNNNNNNNNNNNNNNNNNNNNNNNNNNNNNNNNNNNNNNNNNNNNNNNNNNNNNNNNNNNNNNNNNNNNNNNNNNNN
NNNNNNNNNNNNNNNNNNNNNNNNNNNNNNNNNNNNNNNNNNNNNNNNNNNNNNNNNNNNNNNNNNNNNNNNNNNNNNNN
NNNNNNNNNNNNNNNNNNNNNNNNNNNNNNNNNNNNNNNNNNNNNNNNNNNNNNNNNNNNNNNNNNNNNNNNNNNNNNNN
NNNNNNNNNNNNNNNNNNNNNNNNNNNNNNNNNNNNNNNNNNNNNNNNNNNNNNNNNNNNNNNNNNNNNNNNNNNNNNNN
NNNNNNNNNNNNNNNNNNNNNNNNNNNNNNNNNNNNNNNNNNNNNNNNNNNNNNNNNNNNNNNNNNNNNNNNNNNNNNNN
NNNNNNNNNNNNNNNNNNNNNNNNNNNNNNNNNNNNNNNNNNNNNNNNNNNNNNNNNNNNNNNNNNNNNNNNNNNNNNNN
NNNNNNNNNNNNNNNNNNNNNNNNNNNNNNNNNNNNNNNNNNNNNNNNNNNNNNNNNNNNNNNNNNNNNNNNNNNNNNNN
NNNNNNNNNNNNNNNNNNNNNNNNNNNNNNNNNNNNNNNNNNNNNNNNNNNNNNNNNNNNNNNNNNNNNNNNNNNNNNNN
NNNNNNNNNNNNNNNNNNNNNNNNNNNNNNNNNNNNNNNNNNNNNNNNNNNNNNNNNNNNNNNNNNNNNNNNNNNNNNNN
NNNNNNNNNNNNNNNNNNNNNNNNNNNNNNNNNNNNNNNNNNNNNNNNNNNNNNNNNNNNNNNNNNNNNNNNNNNNNNNN
NNNNNNNNNNNNNNNNNNNNNNNNNNNNNNNNNNNNNNNNNNNNNNNNNNNNNNNNNNNNNNNNNNNNNNNNNNNNNNNN
NNNNNNNNNNNNNNNNNNNNNNNNNNNNNNNNNNNNNNNNNNNNNNNNNNNNNNNNNNNNNNNNNNNNNNNNNNNNNNNN
NNNNNNNNNNNNNNNNNNNNNNNNNNNNNNNNNNNNNNNNNNNNNNNNNNNNNNNNNNNNNNNNNNNNNNNNNNNNNNNN
NNNNNNNNNNNNNNNNNNNNNNNNNNNNNNNNNNNNNNNNNNNNNNNNNNNNNNNNNNNNNNNNNNNNNNNNNNNNNNNN
NNNNNNNNNNNNNNNNNNNNNNNNNNNNNNNNNNNNNNNNNNNNNNNNNNNNNNNNNNNNNNNNNNNNNNNNNNNNNNNN
NNNNNNNNNNNNNNNNNNNNNNNNNNNNNNNNNNNNNNNNNNNNNNNNNNNNNNNNNNNNNNNNNNNNNNNNNNNNNNNN
NNNNNNNNNNNNNNNNNNNNNNNNNNNNNNNNNNNNNNNNNNNNNNNNNNNNNNNNNNNNNNNNNNNNNNNNNNNNNNNN
NNNNNNNNNNNNNNNNNNNNNNNNNNNNNNNNNNNNNNNNNNNNNNNNNNNNNNNNNNNNNNNNNNNNNNNNNNNNNNNN
NNNNNNNNNNNNNNNNNNNNNNNNNNNNNNNNNNNNNNNNNNNNNNNNNNNNNNNNNNNNNNNNNNNNNNNNNNNNNNNN
NNNNNNNNNNNNNNNNNNNNNNNNNNNNNNNNNNNNNNNNNNNNNNNNNNNNNNNNNNNNNNNNNNNNNNNNNNNNNNNN
NNNNNNNNNNNNNNNNNNNNNNNNNNNNNNNNNNNNNNNNNNNNNNNNNNNNNNNNNNNNNNNNNNNNNNNNNNNNNNNN
NNNNNNNNNNNNNNNNNNNNNNNNNNNNNNNNNNNNNNNNNNNNNNNNNNNNNNNNNNNNNNNNNNNNNNNNNNNNNNNN
NNNNNNNNNNNNNNNNNNNNNNNNNNNNNNNNNNNNNNNNNNNNNNNNNNNNNNNNNNNNNNNNNNNNNNNNNNNNNNNN
NNNNNNNNNNNNNNNNNNNNNNNNNNNNNNNNNNNNNNNNNNNNNNNNNNNNNNNNNNNNNNNNNNNNNNNNNNNNNNNN
NNNNNNNNNNNNNNNNNNNNNNNNNNNNNNNNNNNNNNNNNNNNNNNNNNNNNNNNNNNNNNNNNNNNNNNNNNNNNNNN
NNNNNNNNNNNNNNNNNNNNNNNNNNNNNNNNNNNNNNNNNNNNNNNNNNNNNNNNNNNNNNNNNNNNNNNNNNNNNNNN
NNNNNNNNNNNNNNNNNNNNNNNNNNNNNNNNNNNNNNNNNNNNNNNNNNNNNNNNNNNNNNNNNNNNNNNNNNNNNNNN
NNNNNNNNNNNNNNNNNNNNNNNNNNNNNNNNNNNNNNNNNNNNNNNNNNNNNNNNNNNNNNNNNNNNNNNNNNNNNNNN
NNNNNNNNNNNNNNNNNNNNNNNNNNNNNNNNNNNNNNNNNNNNNNNNNNNNNNNNNNNNNNNNNNNNNNNNNNNNNNNN
NNNNNNNNNNNNNNNNNNNNNNNNNNNNNNNNNNNNNNNNNNNNNNNNNNNNNNNNNNNNNNNNNNNNNNNNNNNNNNNN

NNNNNNNNNNNNNNNNNNNNNNNNNNNNNNNNNNNNNNNNNNNNNNNNNNNNNNNNNNNNNNNNNNNNNNNNNNNNNNNN
NNNNNNNNNNNNNNNNNNNNNNNNNNNNNNNNNNNNNNNNNNNNNNNNNNNNNNNNNNNNNNNNNNNNNNNNNNNNNNNN
NNNNNNNNNNNNNNNNNNNNNNNNNNNNNNNNNNNNNNNNNNNNNNNNNNNNNNNNNNNNNNNNNNNNNNNNNNNNNNNN
NNNNNNNNNNNNNNNNNNNNNNNNNNNNNNNNNNNNNNNNNNNNNNNNNNNNNNNNNNNNNNNNNNNNNNNNNNNNNNNN
NNNNNNNNNNNNNNNNNNNNNNNNNNNNNNNNNNNNNNNNNNNNNNNNNNNNNNNNNNNNNNNNNNNNNNNNNNNNNNNN
NNNNNNNNNNNNNNNNNNNNNNNNNNNNNNNNNNNNNNNNNNNNNNNNNNNNNNNNNNNNNNNNNNNNNNNNNNNNNNNN
NNNNNNNNNNNNNNNNNNNNNNNNNNNNNNNNNNNNNNNNNNNNNNNNNNNNNNNNNNNNNNNNNNNNNNNNNNNNNNNN
NNNNNNNNNNNNNNNNNNNNNNNNNNNNNNNNNNNNNNNNNNNNNNNNNNNNNNNNNNNNNNNNNNNNNNNNNNNNNNNN
NNNNNNNNNNNNNNNNNNNNNNNNNNNNNNNNNNNNNNNNNNNNNNNNNNNNNNNNNNNNNNNNNNNNNNNNNNNNNNNN
NNNNNNNNNNNNNNNNNNNNNNNNNNNNNNNNNNNNNNNNNNNNNNNNNNNNNNNNNNNNNNNNNNNNNNNNNNNNNNNN
NNNNNNNNNNNNNNNNNNNNNNNNNNNNNNNNNNNNNNNNNNNNNNNNNNNNNNNNNNNNNNNNNNNNNNNNNNNNNNNN
NNNNNNNNNNNNNNNNNNNNNNNNNNNNNNNNNNNNNNNNNNNNNNNNNNNNNNNNNNNNNNNNNNNNNNNNNNNNNNNN
NNNNNNNNNNNNNNNNNNNNNNNNNNNNNNNNNNNNNNNNNNNNNNNNNNNNNNNNNNNNNNNNNNNNNNNNNNNNNNNN
NNNNNNNNNNNNNNNNNNNNNNNNNNNNNNNNNNNNNNNNNNNNNNNNNNNNNNNNNNNNNNNNNNNNNNNNNNNNNNNN
NNNNNNNNNNNNNNNNNNNNNNNNNNNNNNNNNNNNNNNNNNNNNNNNNNNNNNNNNNNNNNNNNNNNNNNNNNNNNNNN
NNNNNNNNNNNNNNNNNNNNNNNNNNNNNNNNNNNNNNNNNNNNNNNNNNNNNNNNNNNNNNNNNNNNNNNNNNNNNNNN
NNNNNNNNNNNNNNNNNNNNNNNNNNNNNNNNNNNNNNNNNNNNNNNNNNNNNNNNNNNNNNNNNNNNNNNNNNNNNNNN
NNNNNNNNNNNNNNNNNNNNNNNNNNNNNNNNNNNNNNNNNNNNNNNNNNNNNNNNNNNNNNNNNNNNNNNNNNNNNNNN
NNNNNNNNNNNNNNNNNNNNNNNNNNNNNNNNNNNNNNNNNNNNNNNNNNNNNNNNNNNNNNNNNNNNNNNNNNNNNNNN
NNNNNNNNNNNNNNNNNNNNNNNNNNNNNNNNNNNNNNNNNNNNNNNNNNNNNNNNNNNNNNNNNNNNNNNNNNNNNNNN
NNNNNNNNNNNNNNNNNNNNNNNNNNNNNNNNNNNNNNNNNNNNNNNNNNNNNNNNNNNNNNNNNNNNNNNNNNNNNNNN
NNNNNNNNNNNNNNNNNNNNNNNNNNNNNNNNNNNNNNNNNNNNNNNNNNNNNNNNNNNNNNNNNNNNNNNNNNNNNNNN
NNNNNNNNNNNNNNNNNNNNNNNNNNNNNNNNNNNNNNNNNNNNNNNNNNNNNNNNNNNNNNNNNNNNNNNNNNNNNNNN
NNNNNNNNNNNNNNNNNNNNNNNNNNNNNNNNNNNNNNNNNNNNNNNNNNNNNNNNNNNNNNNNNNNNNNNNNNNNNNNN
NNNNNNNNNNNNNNNNNNNNNNNNNNNNNNNNNNNNNNNNNNNNNNNNNNNNNNNNNNNNNNNNNNNNNNNNNNNNNNNN
NNNNNNNNNNNNNNNNNNNNNNNNNNNNNNNNNNNNNNNNNNNNNNNNNNNNNNNNNNNNNNNNNNNNNNNNNNNNNNNN
NNNNNNNNNNNNNNNNNNNNNNNNNNNNNNNNNNNNNNNNNNNNNNNNNNNNNNNNNNNNNNNNNNNNNNNNNNNNNNNN
NNNNNNNNNNNNNNNNNNNNNNNNNNNNNNNNNNNNNNNNNNNNNNNNNNNNNNNNNNNNNNNNNNNNNNNNNNNNNNNN
NNNNNNNNNNNNNNNNNNNNNNNNNNNNNNNNNNNNNNNNNNNNNNNNNNNNNNNNNNNNNNNNNNNNNNNNNNNNNNNN
NNNNNNNNNNNNNNNNNNNNNNNNNNNNNNNNNNNNNNNNNNNNNNNNNNNNNNNNNNNNNNNNNNNNNNNNNNNNNNNN
NNNNNNNNNNNNNNNNNNNNNNNNNNNNNNNNNNNNNNNNNNNNNNNNNNNNNNNNNNNNNNNNNNNNNNNNNNNNNNNN
NNNNNNNNNNNNNNNNNNNNNNNNNNNNNNNNNNNNNNNNNNNNNNNNNNNNNNNNNNNNNNNNNNNNNNNNNNNNNNNN
NNNNNNNNNNNNNNNNNNNNNNNNNNNNNNNNNNNNNNNNNNNNNNNNNNNNNNNNNNNNNNNNNNNNNNNNNNNNNNNN
NNNNNNNNNNNNNNNNNNNNNNNNNNNNNNNNNNNNNNNNNNNNNNNNNNNNNNNNNNNNNNNNNNNNNNNNNNNNNNNN
NNNNNNNNNNNNNNNNNNNNNNNNNNNNNNNNNNNNNNNNNNNNNNNNNNNNNNNNNNNNNNNNNNNNNNNNNNNNNNNN
NNNNNNNNNNNNNNNNNNNNNNNNNNNNNNNNNNNNNNNNNNNNNNNNNNNNNNNNNNNNNNNNNNNNNNNNNNNNNNNN
NNNNNNNNNNNNNNNNNNNNNNNNNNNNNNNNNNNNNNNNNNNNNNNNNNNNNNNNNNNNNNNNNNNNNNNNNNNNNNNN
NNNNNNNNNNNNNNNNNNNNNNNNNNNNNNNNNNNNNNNNNNNNNNNNNNNNNNNNNNNNNNNNNNNNNNNNNNNNNNNN
NNNNNNNNNNNNNNNNNNNNNNNNNNNNNNNNNNNNNNNNNNNNNNNNNNNNNNNNNNNNNNNNNNNNNNNNNNNNNNNN
NNNNNNNNNNNNNNNNNNNNNNNNNNNNNNNNNNNNNNNNNNNNNNNNNNNNNNNNNNNNNNNNNNNNNNNNNNNNNNNN
NNNNNNNNNNNNNNNNNNNNNNNNNNNNNNNNNNNNNNNNNNNNNNNNNNNNNNNNNNNNNNNNNNNNNNNNNNNNNNNN
NNNNNNNNNNNNNNNNNNNNNNNNNNNNNNNNNNNNNNNNNNNNNNNNNNNNNNNNNNNNNNNNNNNNNNNNNNNNNNNN
NNNNNNNNNNNNNNNNNNNNNNNNNNNNNNNNNNNNNNNNNNNNNNNNNNNNNNNNNNNNNNNNNNNNNNNNNNNNNNNN
NNNNNNNNNNNNNNNNNNNNNNNNNNNNNNNNNNNNNNNNNNNNNNNNNNNNNNNNNNNNNNNNNNNNNNNNNNNNNNNN
NNNNNNNNNNNNNNNNNNNNNNNNNNNNNNNNNNNNNNNNNNNNNNNNNNNNNNNNNNNNNNNNNNNNNNNNNNNNNNNN
NNNNNNNNNNNNNNNNNNNNNNNNNNNNNNNNNNNNNNNNNNNNNNNNNNNNNNNNNNNNNNNNNNNNNNNNNNNNNNNN
NNNNNNNNNNNNNNNNNNNNNNNNNNNNNNNNNNNNNNNNNNNNNNNNNNNNNNNNNNNNNNNNNNNNNNNNNNNNNNNN
NNNNNNNNNNNNNNNNNNNNNNNNNNNNNNNNNNNNNNNNNNNNNNNNNNNNNNNNNNNNNNNNNNNNNNNNNNNNNNNN
NNNNNNNNNNNNNNNNNNNNNNNNNNNNNNNNNNNNNNNNNNNNNNNNNNNNNNNNNNNNNNNNNNNNNNNNNNNNNNNN
NNNNNNNNNNNNNNNNNNNNNNNNNNNNNNNNNNNNNNNNNNNNNNNNNNNNNNNNNNNNNNNNNNNNNNNNNNNNNNNN
NNNNNNNNNNNNNNNNNNNNNNNNNNNNNNNNNNNNNNNNNNNNNNNNNNNNNNNNNNNNNNNNNNNNNNNNNNNNNNNN
NNNNNNNNNNNNNNNNNNNNNNNNNNNNNNNNNNNNNNNNNNNNNNNNNNNNNNNNNNNNNNNNNNNNNNNNNNNNNNNN
NNNNNNNNNNNNNNNNNNNNNNNNNNNNNNNNNNNNNNNNNNNNNNNNNNNNNNNNNNNNNNNNNNNNNNNNNNNNNNNN
NNNNNNNNNNNNNNNNNNNNNNNNNNNNNNNNNNNNNNNNNNNNNNNNNNNNNNNNNNNNNNNNNNNNTCTATAACCGAA
CTGGTTAAAACTCTGTTGTTGGCCGATTGTAGCTACAGCTAATGCCATGCTAACCTGAGTGCTACTACTAGCAGCAGTAA
ACCCAGGTATAATGGCTAAAACAAACATTGACTACCATGATATCCTGCAAAAAAGACGAGGCAGTTCAGTGTGTACTGCC
GCCACCAACAGGCAGTTCCCTTCTTGACGTCAAAGCCGTAATGTTTTGTTGGTATAGGAGTTTCTAATAAATGTAATTCA
AATTGCAAAATATGTGTCATTTTTTTTTTTCATCAACTTTTGCATAAAGAACTTTTGCATAAGATGGAATATCATCAATCT
ACATTGTTAGATTTGTGGAAATATATTGTGCAACCTTCCCTTTAATATGCGTAACAACCAGCAGTGTAGAGAGTGCTACT
ACACAGTAAATATGTATTCCTTGGGGCTGTATATTATTGAAAAATTAGGCAAATGTGATTTAAAGATAATGTTTTTATAA
TCAACATCTAAGCTACGATACATTTTTCAAGACGGGTCTTTTAGAATCCTCTATGTAATTGAAATGTTGACAAAAAATAG
TCCCCCTTTAATAACTGATTACTGATCAATTTATCAATTACTAGGTAACAGAAATGACATGTTAAGAAATGGAGTGAAGC
CCCATTGTAAATACCATGCATCAACTGGAAAATAGCTTTTTATGCAGAGCAGCTATGTAAATGTCGACGTTATGTAACAT
CAAGTGCTCACATATCCTGTGATATATCCTGAGGATGAAATAGAGCAATAACAATAATAACAGCAGTAGCAGCACAGCTC
CTGGTTTGACTGGCAGTTGTCCATGGGTCTCTTGTGTAGGCTGGTTTCCCTTCCTCAGCCCTGAGTGTGTTAGTCAGCTT
CCATACATTGGTTGGCTGTAGTCTTAAAAGACCTCATCTCTCTGCACTGAGACTAGGGTCAGGGATTTACTGCCACTTTC
TTCAACTTCAATTAAACTCACTCTCAAATAGCCACCTGTCCCTTTAAATAGCTGGGCATCGGCATGCAAATAAACCCCTG
TTTCAAAAAACACTGGGTCTACATTGATTGTTTACGAGGTTACCATGGACACAGCTCTGATTTTCCCACAGTCATGTGCA
TTACATTTTTTTATATTTTATTCTATCATCGTTGCTAGCCATGGTGACACCTAAAAGAAAACACATCAAATTCAAGCTAA

CAGTGAACAAATTCGCAGAGCAAAATTCAGGCGAGGCAACAGCCAGGCATTTTGGAATTGATCCAAAAAGAGTGAGAAAA
TGGCTGTCAAAAAGTTGAACTTCAACATTTGTCTGAGTTGGATCAGAAGAGGGCAAGACTGCAAGGTGGGGGGAGGGGAA
ATGTTAGCGAGGAGCTGGAGGTGGAAATCTGTCTCTTATAAGCAGCGGTTGTGTTCAGTGATTGAAGCAAATAAACACCT
GGGCTATTAATTGAAGTTTTACCTTAAATTTCTTTGATAGTTTCCTTCACCAAAGCCTGCTCTCCCCGAGGCCCAGAGGA
AAAGAAGATGCCAACTTGGTAGTAGTCATCTTCTTTTTGCTGAGCATGTGGATTGGGAGTCTTTGGGCGGTGCAGTGTGC
CTTTCAGTTTCACTTAGGAACCACTGGTGTAGATGAGAGTAGCCATTAACACAAACAATCATGCACACACAAATACACAC
AAATACACACAAACACACCAGTGGTGGACTGTAGGCAGAAATCTGTTGATGGCAGGGCCAGCAAAGATGTAGGCAGAGGT
GGAAAAAGTACTCAATTGTCATACTTGAGTAAAAGTAAAGATACCTTAAAAGTAAAAGTGAAAGTAACCCAGTAAAATAC
TCCTTGAGTAAAAGTCTAAAAGTATTAGTTTTAAATGTAGATAAGTACAGTAGTGGAAAAAGTACTCCATTGTCACACT
TGAGTAAAAGTACAAAGTAAAAGTAAATGCTATAAATCAAATGTCTTATATTTAGCGAACCAAACGGCACAATTTCCTTT
TTTATAACTTTTTTTTACAGCCAGGGCCAACTCCAACACTCAGACATCATTTACAAAGGAAGTATTTATGTTTAGTGAGT
CCACCAGATCAGAGGCAGTAGGGATGACCAGGGACGTTCTCACGATAAGTCTGTGAATTTGACCTTTCTCCTGTACTGCT
AAGCATTGAAATTGTAACTAGTTCTTTTGGGTGTCAAGGAAAATGTATGGAGTAAAAAGTACATCATTTTCTTTTGGAAT
GTAGTGAAGTAAAAGTAGTCAAAAATATAAATAGTAAAGTATAATAATAATAATTATAATAATAATTATAATAATAATAA
TAGATAGAGTTAGCCTAATTCACCCATTCTTTGTTTGATTTGGTCTACACCGTTTATGTTCTGGTTCAATTTGACCAGTA
CAGTAAGCGCAGTAAGTACATCAAGAACATAAATATATAAGTTTAACCTCCCAAAATGTATTTTCCAGTAGCTTAGAAAG
CAGCTTGTTTCTAGGACAAATCATTCAAAAGTTATACAGTGCCTTTGGAAAGTACACAGTGGCCTCCATCATTCTTAAAT
GGAAGAAGTTTGGAACCACCAAGACTCTTCCTAGAGCTGTGCGCCAGGCCAAATTGATCAATCGGGGGAGAAGGGTCTTG
GTCAGGGAGGTGACCAAGAACCTAAAGGTCAATCTGACAGAGCTCTGGAGTTCCTCTGTGGAGATGGGAGAACCTTCCAG
AGGACAACCATCTCTGCAGCACTCCACCAATCAGGCCTTTATGGTAGAGTAGCCAGACAGAAGCCACTCTTCAGTAAAAG
GCACATGACAGACTGCTTGGAGTTTGCCAAAATGCACCTAAAGATTCTCAGACCATGAGAAACCAGATTCTCTGGTCTGA
TGAAACCAAGATTGAACTCTTTGGCCTGAATGCCAAGCATCACATCTGGAGGAAACCTGGTAACATCCCTACGGTTAAGC
ATAGTGGTGGCAACATCATGCTGTGGGGATGTTTTTCAGGGGCAGGGAATGGGAGACTAGTCATGATCGAGGCAAAGATG
AACAGAGCAATGTACAGAGAGATCCTTGATGAAAACCTGCTCCAGAGCGCTTAGGACCTCTTGACAACACAGGAGTGGCT
TCGGGGACAAGTCTCTGAATCCTTGAGTGGCCCAGCCAGAGCCCGGACTTGAACCCAATCCAATATATTTAGAGAGACCT
GAATATAGCTGAAAGAGCTTGAGAGGATCTGCAGAGAAGAATGGGAGAAACTCCCCAAATACAGGTGTGCCAAGCTTGTA
GTCTCATACCCAAGAAGACTTGAGGTTGTAATCACTGCGAAAGGTGCTGCAACAAAGTACTGAGTAAAGGGTCTGAATAC
TTATGTGAATGTGATATGTTTCTTTAAATTTTTAATACATTTGCTAAAATGTCTAAAAACCTGTTTTTGCCTTGTCATTA
TCGGGTATTGTGTAGATTGATGAGGGGGAAAAAACGATTTAATACATTTTAGAATAAGGCTATAACCTAACAAAATGTGG
AAAAGGTCAAGGGGTCTGAATACTTTCCAAATGCACTGTATAGAGCAGGCCTGAGCAATTCCAGTCCTCGGGGGCCTGAT
TGGTGTCACACTTTTCCACCATCCCTAGCAAACACAGCTGATTTAAACTAATTGCATTCTAAACTGAAGATCATGATTAG
TTGACTATNNNNNNNNNNNNNNNNNNNNNNNNNNNNNNNNNNNNNNNNNNNNNNNNNNNNNNNNNNNNNNNNNNNNNNNN
NNNNNNNNNNNNNNNNNNNNNNNNNNNNNNNNNNNNNNNNNNNNNNNNNNNNNNNNNNNNNNNNNNNNNNNNNNNNNNNN
NNNNNNNNNNNNNNNNNNNNNNNNNNNNNNNNNNNNNNNNNNNNNNNNNNNNNNNNNNNNNNNNNNNNNNNNNNNNNNNN
NNNNNNNNNNNNNNNNNNNNNNNNNNNNNNNNNNNNNNNNNNNNNNNNNNNNNNNNNNNNNNNNNNNNNNNNNNNNNNNN
NNNNNNNNNNNNNNNNNNNNNNNNNNNNNNNNGGGCCTACCGAGTGGCAAAGCGGTCTAAGGCACTGCATTGCAGTGCTA
GAAGCGTTACTACAGACCCGGGTTCATTTCCGGGCTGTGCAACAACCAGCCGTGGCCCGGGCTAGGGGAGGGTTTGGCCG
GTGGGGCTTTACTTGGCTCATTGTGCTCTAGCGACTCCTTGTGGCGGGCCGGGTGCCTGCGGGTTGACCCCCGGTCACCA
GTTGAACGGTGTTTCCTCCGACACATTGGTGTGGCTGGCTTCTGGGTTAAGCTGGCGGGTGTTAAGGAGTGTGGTTAGGC
AAGTCATGTTTCGGAGGACGCATGACTCCACCTTCGCCTCCCTCGAGCCATTGGGGAGTTGCAGCGATGAGACAAGATCG
AAAATTGTGTAGAAAAAGGGGGTAAAATACAAAGAAACAAACAAAAAATTATAATTATGTGTTTTACAAGTTTGAAACCC
ATACTTAGGAAAGTAGATGAACGGTCATGGTGGAAGAATTACATTCATTTGTATCTTTGTGATTTTCAAACTCTAAGGGA
AGAGTTAAGTTGACAATAGTCTGGTGGGTGAGAATATTATCAATTGTCAAATTGTATATGAAGAATCTGATGAACAGTGC
AGCTTGGAATTGACATAGAGGCAGGGAAACGAAACATCCCAAAAAATGACTTTGCAGATTCTCCTGCAGAGTCACAAGCA
GGTAAGACGTGTTGACAGGGATTGGACTAGGAATGAATAATGGCCCTGGATTTTTGACTCAGACTGGCACACCAACACCC
CCCCAGCGATATACCACCACTACAATGGCACTGGCAGCAAGGTGGCAAGAATCTCTGGAACCATCAGACCAGTAAAATAA
ATAATACAGGAAGAAATGTGTTGGTGAAAAGACTGGTGAGTAGGCAAGTCATAGAATAAAATTAAAATAAACATATTTCC
TACCTCCTTAAATTTGCAATTTTTGCAGCAGCTCACTTCTCTCAGCAATGACATCTATAAACAGGTCACTTTCCAGGGCC
ATTAAAACATCTTGCTCAGTAGCCATGAGCATAAAAGCCTCAAGGTGCGGTCGAGAGAGTGCTCCTGAGCCTACTCTTGA
TGAATTTCAGAGTAGAGAAGCTCTGCTCGCAAGCTACCTGGGTTACTGACAGGGTAAGTAGGAACTTGTAAGCAAGGCCC
TGGATGTGGTATGCGTCGGTCAATATGTTGTACTGACTAAGAATACGGTAGCAACACACTGGACAGTCCTTTCAAGATGA
ACAGCTCTTGTTCACCATCTCCATCTTGTCTTCATTTCCTTCAAGTCCATGATCCTCCTTAGTCCTGGTTGTGTATTCTT
CAAATCCCGATTGTTTTAACCTAGTCCACTGTTCTGCCAGACTCATGAACTCACTCTGTAAATTGAAACACCGTTGCTCT
GCTATCAAATTTGATCAAACATTTGCTTAGTTCTTAAAGGGCTCTCTGTGGAAGGTCACTGGCACTGGATGTTAGCTGAC
AAAAGTTTTTAGGGTCCAGAAGAGCCATGTTGGCATACAAAGTGCCATTACCCAGAAATCGCCGATGGATGCTATCTATA
ACTGTATCCAGAATTTGATTATGGACACCAATCCTGTATGCACTCTCTGCCCCAGTAAAAGTCTCATCTTGTGCCATCTC
TCCAGGCATGGTTTTCTTCTTTCGAGCCCTTTTTGTTGGCAGAGTGGTCTCTAACTCCAGCTCTGTTTCCTCATCCCGTT
CCTGCAACTTCCTGTTGGCCCACTGAACAAATGTGTCTGCAGCTGCCTTCACTTTTTCTAAATCTCTTGCCATTCCTTTC
AGCTGCTCCTCTGTAGACACAACCATACGATGCGCAGACAGAATGTCCATTCCATTTGTTTGAAGATATTTTGAGACTGG
TGAGGTGACCTGAAATATTCTGAAGAATATCTGAGCTGTAAGTATTGTTTCATACTTCAGCAACCCCTCAATGTATCCTT
GTGCCTTGACCCGTGCAGTAGTGTTGATGTTTTTCTGATCTTATATGGTTGAAAGATTGAGAAGGACATCAACATACAGA
CCCTCATCTGGATTTCCAAAAGCTCCAAAGACCTTTAAGGCACTGTCTTTAGCCCACCAGCGTGTCTCTCCAATTGGAGC
AAGACGTCTGTGTCTCGGGTCCTTGCTCTCCTTCTCCCAAATGTTCATCCTTTGGTAGGATTCACGGAAGAGCACAGCTA
TATTGTTAATGAGAGAAAAAAGTGACCCACTTGCCAAGACACTCTGTTGTGTCTGCCAAAACAAGGTTCAAAATCTGNNN
NNNNNNNNNNNNNNNNNNNNNNNNNNNNNNNNNNNNNNNNNNNNNNNNNNNNNNNNNNNNNNNNNNNNNNNNNNNNNNNN
NNNNNNNNNNNNNNNNNNNNNNNNNNNNNNNNNNNNNNNNNNNNNNNNNNNNNNNNNNNNNNNNNNNNNNNNNNNNNNNN
NNNNNNNNNNNNNNNNNNNNNNNNNNNNNNNNNNNNNNNNNNNNNNNNNNNNNNNNNNNNNNNNNNNNNNNNNNNNNNNN
NNNNNNNNNNNNNNNNNNNNNNNNNNNNNNNNNNNNNNNTAATGAACAGGTAAATGGGGAATTGAAACCAGGTGTGTAAG
ACAAAGACAAAACAAATGGAAAATGAAAAGTGGATCGGCGATGGCAAGAAGGCCGGTGACGTCAACCGCCGAATGCCGCC

CAAACAAGGAGAGGGACCGACTTCGGCGGAAGTCGTGACACACACATGTACTTGATTGGTGGACTTTGCAGAGAGTAGGG
CAGAGAAGCCTTTATTCTGGCCTTGCATACTGCAGGCTCCATCAGTGGAATTGCCATTGCACTTGGTGATGTCAAGCTTC
ATGTTTTCCAGTACTTCACTCAGTACCTGGACAAAGTATTGTCCAGTGGATGCCTCACATTTCACCACAGCAACAAGTTA
CTCATGGATGACGTACGTCACATATCTGACTATGACAGAGCATTGATCTTGTGTTGTAATGTCCTGCGTAGTGTCAATCT
GGACTGAAAACATGCCAGCTTGGTGGCCTGCATTGTGTGTTGGATGGTGGTAATGACATTATCGATGGTAGTCTTTGATA
ATAGAGTGATTAGAGAGCCTCTGCCCCCTCTTGACCCAGACTCATGCAGTTTGTTGCTTTTCTCTATGCAGGCAGTGAGA
TGCTCTTTCATACACAGGTCGTACTTTCCCAGTAGAATGATAATCTCTAAAAAGTTGGCATGGTCAATGCTGCTGTCATC
TAGCGTATAAGCTGCTTCAGACTGCAGGCCTCTGTAGCTCAGATCCTTCTTGCCTATGACTTTAACAACATCTATAATGC
GCTCTAACACTTGCCTTCTTCTGTTCACTTGCTCTCTATGAGCAGACATCTGACTGCCGATGAACAGGGTCTCAATGCTA
CCTTTGGAGGACCTTAGAAAGTAGGCCTCAGCATAACTTCTATGCATAATGCTCTTCTCATGCTCTCCCACTCTCTGATG
GATATGCTTCGTCTGACATTCCATTCATGAAAGGGCTGTTCTCTGTGGGCTTTACAAATGCCTTACAAATTAAGCAGAAT
AAAGCATGGTTCACTTCACTGTCTGACGGCCACTTCCTTTTGGTCCCATCTTTACAGAAGAAAACCTTCTGCACCACAGA
CTTTTCACACTTTTGTTGGGGATGGAAACTAAAAAACATATCTAGGTCCCCTGACGTCGGCCTCTTAAAATAATCCCAAA
CTGGGGTGGCAGTGACATCCTGGCTCTGTCCACTGCAGATGCACTATCCTCAACCTGGATGAGTAATTAGTATAAATTAG
TATATTAGTATAATATTATAACATTAAATAAAAAGTATGATGTTAATATAATAGTATAATGTTAGTATAATATTAATATA
TTAATTATATAATGTTAGTAGATTCATATAATTTCATATAATAGTATACTATATAGCACTAGTGTAATATTAATTCCAAT
TGGGCAATACTGCAGTTTAAGAAATACACAACCAATATTTATAATTTTTGTAGGTTTTGTTCCATCATTAAATTCTTGTG
CAATAAAAATGGTCTACATTGGTGGTTAGTCTCTAATTTGTTAATGCTAAGCACTAATGTCAATAATCATAACCATAACA
ATGAGTTCAACATTTTGAAAGTTTGTTGTAGCGCGTCAACCTTTTTTTACTGTCCCGCCCCATCCAGGCTGTGATTGGTT
GGTTCACGAAAAGTGACATTGACGAGCGCTTGTTTCAACAAAAGGCACCTGATATAGCTAAATAGCCAGCTAGCCAAACC
CAAACATTCCTTATCTTATCCCTATTGTCTTCTGCCTTACCGCTTCAGCTACAAAACCTTGACTAACAGTTGAATGGGTA
TCGCTGGGCTCAATGGGCTGCTCTCTCTCCTCTCTGCTGCCAGTGCCTTCAGGCTCACTAGGCTGAGATGATTGACTGGT
AATGGCGCCAAATTAATAATTTGTTATTTTAAAACATTTGGCTGCATCAGCCTCAAGGGACTTCAACCTCTTCTCTCTCC
GCTTTTCAGCACCAACTTTACATTTTTTCTCTTTTTGTTTGTCCATCTTGCTCCACTGCACTATTTATCCAAATGTCACC
ACTTAACAGAGATGGGAAAGGGGCCCAGGACTGGACCAAAAGTAATTGGCTGGGAGAGCGAGGCTGACAGATGTAATACC
CAACCGCAGTGTCAGTGGGCCGGCAGCCCACTCGCCTGGGCCAGTCAGACACACAGTACTTGGTTATTTTTATTTAAGCG
GGGGCTGGGGTTATTTATATTTTGCGTTGCATCGGCCCCAATTGTCAAGCGGAAAATTCCGCTTGACAATGCTCCAGATG
GCCAGTCCGTCATTGCGTGTTGATGTCTATATAGTATCAGAAAGAGCAGATTCTGCAGTTTCCAAATGACCTATCCTCGC
CAAACAACTCTTAAAAATGACCCATTGAGCTGTTTTGAAAATACAACTTTTTGGCAGAATTACTGTGATTACTGTCAACA
CTCAAAGCACTTATATTTCAACTCAAAAGATGCCATTCTGTTTTTTTATGCATTTTCTTAAATTCATAATGTTCCTTAAG
ACCTCCAGGGGACCATGACACAACGTCCCAAGAACGTCTTATAAATGTCTTTGGGGACACCAGGAACTACACAAATCCTC
CAGGGGACCTGACACAATGTACTAAGAACGTTCAGGGGAACCTTCAGGGGTCAAATCTGGAGCTATAACAAGGTCCCCCA
GAGAACGTTACCTTGGGGCCATTGCACAACGTCCTAAGGACTAGTAAAATGAAAGTTATGAGCACGTCTGAGAAAGGTCC
TGAGTGACGTCCTGGGAACTAGACAAAGGTAACCCAAAGAACGTTCCCTTGGGATCATCACGCAACATTCTAAGAAGGTC
AATGGGATAACATTGCGCTGAGACCCTTTAGGGACCTAATAGGAATGTCACCGAATGTCCTCAGGTCATCTCCTGTTTGC
TGGGATATAGACTAGAACAGTGGTCACCAACCTTTTCTTTGTTGAGATCACTTTCTGAGTCAAAATGCAAGCCGAGTGCA
ATTCTCTGCTGGGCACGGGAGATCAAGTACGCATATAATGTAGCCTTTTGTGTGATGCAAATGAAATTATCTATAGCCTA
ACAGTGAATGGGAAAATAAGTACAGGGCAAAGTTATATTCCCAATGAAATGAACCTCCTAAAAATGATTATTTCATGCAT
TTGGCTATTTTCATATATAGTAAGAGAAGTCTGATTTTCTCCATGTAGGCCTTTATATTTCAAAACTCGAGTCTCAAGCT
AAGTCAGTGGCGACCCGTCATTCAGGGCAGGTGGGGGCAGAGCCTGTTTTGAGCCCCACCTGTTTAGTTAAAAAAATATA
TGTTTTGCATGTTATCTTGGCATTAATACGTGTCACATATCAGTTTACATACAATGTAAAAACAAATCGAGTGGATAAAG
CCGCATATAAACATGGTCTCTTTTTTTGCATTCTTGAGTAAGGCAGCTCCAAAATGCAGGTGTTTCAGCCTAGCTCAGTGC
TTTCTGTGGAGGGACAGCCAGTGGAAAATACAGAGTTTAGGGGTTGGTAATCTTCTCTAGTTGCGCCGTGATTGGCTCAG
TGTTCTGTCACTCATGGGGACACTATGTCATCTCAAAATCTACAGGGAGAGCTAGAAAATTCAAGCCCCCTTGGGTGCGG
CCATAGATTTACATTAGAAGTGCCCATCCAAGAAGGCTCAAGGTCATTGGCCACAGATAAAATGACATCAATCCTTTTCA
ATCCTTGTCTCATATGAAGAGAAATTATAGATAAAATGTATCGGTGCTCATCGGCCATTGGACATAAACATGACACAACA
AGTTGGAAATCACAAATTCAACAATGAGTGGTTTGGAAGGAATCAGTGGCTGACTGCAAGCATTGCAAAGCAGTCACTAT
TTTGCTTCCCCTGCCTGCTATTCGGTGGAGAGGGTGTGTGGTCCAAGTCTGGGTTTAAGGGTCTCTTTTTCAAGCTTAAA
AGGATAAACATTCACACACAACACCATGGGCCCAAAAAGGTTGAATACTCTGGCCATGCTGTCAACCCAGCATGACTTCT
GCCGTGTTCAAAACAACTTGAAATTGAACGTTTTGAACGGTTATCCAATACGGAATTTCAAGTCGGGAACTCGGGCCTCT
TTCTAGAGCGCCGAACTGAAGGTCACTGATGTCATGCCTGTTTTAGAGAAATGTCATCATCGAATATTGTAAGAGCTTTC
ATTGTCTGCTTATTCAGTATCCCCCCTTTATTTATCCTATGGTTGTGACATGGTGTTCAGGGAGAATACTGTAAGAATGG
CCCATGTTTTGAATTCTGTTGCTGTACATTTCAAAAGTTTTGATATACAGTTATATTGACAATGTCCGCCTTAGCTGGCT
CGTTAATGTTGTAATCAAAATTATGGATTGCCTTTTATCCGCACATCGTTCCCTTATGCTATGGTTTGTACATCTCAATT
GTCAGTGGATCCACATTGTTTAAGCAAGTCAGCCATATCCCAGCTATGTTTTTTTTTTTTATGCAGTAAATGAGGTTGAA
TGAANNNNNNNNNNNNNNNNNNNNNNNNNNNNNNNNNNNNNNNNNNNNNNNNNNNNNNNNNNNNNNNNNNNNNNNNNNNN
NNNNNNNNNNNNNNNNNNNNNNNNNNNNNNNNNNNNNNNNNNNNNNNNNNNNNNNNNNNNNNNNNNNNNNNNNNNNNNNN
NNNNNNNNNNNNNNNNNNNNNNNNNNNNNNNNNNNNNNNNNNNNNNNNNNNNNNNNNNNNNNNNNNNNNNNNNNNNNNNN
NNNNNNNNNNNNNNNNNNNNNNNNNNNNNNNNNNNNNNNNNNNNNNNNNNNNNNNNNNNNNNNNNNNNNNNNNNNNNNNN
NNNNNNNNNNNNNNNNNNNNNNNNNNNNNNNNNNNNNNNNNNNNNNNNNNNNNNNNNNNNNNNNNNNNNNNNNNNNNNNN
NNNNNNNNNNNNNNNNNNNNNNNNNNNNNNNNNNNNNNNNNNNNNNNNNNNNNNNNNNNNNNNNNNNNNNNNNNNNNNNN
NNNNNNNNNNNNNNNNNNNNNNNNNNNNNNNNNNNNNNNNNNNNNNNNNNNNNNNNNNNNNNNNNNNNNNNNNNNNNNNN
NNNNNNNNNNNNNNNNNNNNNNNNNNNNNNNNNNNNNNNNNNNNNNNNNNNNNNNNNNNNNNNNNNNNNNNNNNNNNNNN
NNNNNNNNNNNNNNNNNNNNNNNNNNNNNNNNNNNNNNNNNNNNNNNNNNNNNNNNNNNNNNNNNNNNNNNNNNNNNNNN
NNNNNNNNNNNNNNNNNNNNNNNNNNNNNNNNNNNNNNNNNNNNNNNNNNNNNNNNNNNNNNNNNNNNNNNNNNNNNNNN
NNNNNNNNNNNNNNNNNNNNNNNNNNNNNNNNNNNNNNNNNNNNNNNNNNNNNNNNNNNNNNNNNNNNNNNNNNNNNNNN
NNNNNNNNNNNNNNNNNNNNNNNNNNNNNNNNNNNNNNNNNNNNNNNNNNNNNNNNNNNNNNNNNNNNNNNNNNNNNNNN
NNNNNNNNNNNNNNNNNNNNNNNNNNNNNNNNNNNNNNNNNNNNNNNNNNNNNNNNNNNNNNNNNNNNNNNNNNNNNNNN
NNNNNNNNNNNNNNNNNNNNNNNNNNNNNNNNNNNNNNNNNNNNNNNNNNNNNNNNNNNNNNNNNNNNNNNNNNNNNNNN

NNNNNNNNNNNNNNNNNNNNNNNNNNNNNNNNNNNNNNNNNNNNNNNNNNNNNNNNNNNNNNNNNNNNNNNNNNNNNNNNNNNNNNNNNN
NNNNNNNNNNNNNNNNNNNNNNNNNNNNNNNNNNNNNNNNNNNNNNNNNNNNNNNNNNNNNNNNNNNNNNNNNNNNNNNNNNNNNNNNNN
NNNNNNNNNNNNNNNNATCAGCATGACTATAGATTTTCATTTCCCCCACATTTGGAGCATGTTTTCTCCCTCTGTTTCTTA
GCGTTTGGAGGTGAGTGTGTAGGATGTAGTTTATAGGGTCCTATGTGTCTTTTCTGTTCGCTGGCTTTGTTATGTGTCTA
TAACTCAAATGCTAGGTACTTGGAAAATGTTGCCATGGAAACTAAATAAAGAGCCCGGGTTTGGATCAGAAGGGACTGGG
TGAGAAATTGAATGAAATGGACCTATTGAGTCCCTTTTACGGATGGCTTTTTAATGCATAATTATAAATAAATATTTAAT
GTGGTCGATTATTGCGGAACACCATTAAGGGGTTCGTATAGGACACAAATAATACTGGTGCTGCTGCAAAACATCACACA
ATTCACGCTGCTCATTTGTCTCCACTTAAGGTAGAAGTTGCTCTAACAACAGATCTAAGATCAGATTACTAATCTCTTCA
TCATAACCTGGACCTTGTAGGGATGAAAAAAGATCTGACCCTATATCAGTGTTTAGAAGCAACTTCAACCCCACTCATCT
TGTCTCCACACTGAGCATGAAGTGCATGAAGTGTTATAGTAACACATGGACATGACTAAACAAGTGAGGATATGTGGTTA
CGTTTGACATGTATAAAATAATTAGAGCCCGTACACTCATAAGCTCCACCGTCAAGGTATGTTTGTGAATAAAGGTACGT
GGTAGAGCTTATGAGTGTGTGGGCTCCATTTCTTTTATACTGGCACTGCACCCCACTAAAGGATGGGTCAGTTCCTTACC
TTAACTCTTACCTTAATCATTTCAAATGTCAACTTCAATGGTGTCGGGACGTCCCAAGGATCCCGGGATAGCAGAGACCC
TAAAGGATGTACATTTGATCACACTTGACTACATTTTACATGTGTCTGACTCACCCATCATAATTCATACATGATGCTCT
TATGATTTCAATAGCATGATTCCCCGCTCCCTTCCTACTTCATAACATCTGATTTCAGTTCATACAAAAATGACTTACAG
CTGCTATGACACGTGGGAGTATACTTTCCCAATACCAAGTGACAAGCCAGAACAGATCCCAGATGATCTTGTGTACCATG
AACCATACACAACAGTGTTAGGTCCCTTGTCATTTATGTCTGCTTATGTCATTTCTAGAGCAGAAAATTGGAGCAGGAGG
AAATGTTTGTGGTAATTAAAAGTATGTGGGTGCCTGTTTCAAAAGGAATAGTCAAAAAAGAAAAAACAAGTGCAGGTTTC
TCCTTTTTCAGTATAGAACAGAAAATTGTCCATAGTATGTCGTCTCTGGTCAGATATATATTGTAGCTCTGTTACGCACA
AGCATACCATCTAGGATGAAGTATGCTAAAGCATTGGGCAAGTAGTATGCTTCTACACCTGCATTGCTTGCTGTTTGGGG
TTTTAGGCTGGGTTTCTGTACAGCACTTTGTGACATCAGCTGATGTAAGAAGGGCTTTATAAATACATTTTATTGAAATT
TGATTGATATGGTAAGTAGTACGCTAGTATGGTCATTGGAACGTAGCCCTACAGTACATTAGTGGAGGTTAGTATGCACT
ACACTGCACTACACTGTCTTAGATCCTTTCTTGTAAGTTTGCATATCACCAAGGAATTGGAACAAAGACTGTTGGCATTT
GTAAAGCTTTGAAATATCCACTTTTGGAGGTTAAACCGCAATGACTCAAAATCACAACACCGGAATACTGTTTCTTTGTA
GATAAAGCAATTAACACTTTATGTTTAAATGGGGCAGAAATCCTTGCTCAGCGATTGCAAAATTTGCCATAATGAGAAAG
AAAGTGGTGTAATAAATATAATGTAAACTTTATATTACTAAAATATTGTAGTGAAACGGATCATCAATCCTAGTAATATT
ATCATCATGTTTTGTTATACACAAAGCTCTTATGAATAACTGCGGCCTATCAGGTATATTTAAGGTTTTTGTTCATAATC
GTTACCAAACACGTGCATTCTTCTCTTAATTTACTGAGCTAAATGACACTCAGACGTTTGCCAAATTCTTGTTGTGTTGC
CTAGCAAGCGTTATGCTAAGGGCATCTCAAGGGTTCGTTCTGTTCGTACGGAAGCATCGGAACCTTTATTGTATAAACAC
TTTGAGTACAAACACTTTGACAATGTCAGGTTTGGCTTCACTCTGTGATTTGAGAGCTTTAAGTGAAGGCCAATGGTGGT
GAACAGCAGATACGGAATGACCTTGCTGAACTATTTCCNNNNNNNNNNNNNNNNNNNNNNNNNNNNNNNNNNNNNNNNNNN
NNNNNNNNNNNNNNNNNNNNNNNNNNNNNNNNNNNNNNNNNNNNNNNNNNNNNNNNNNNNNNNNNNNNNNNNNNNNNNNNNNNN
NNNNNNNNNNGTACCTTAGTAAGCAGTTGCTTATCAACAGATAGTTTGTTGATAGTATGACCATCTGTAGAGCATCTACA
GATGGATTACCTGGACGATCCAAATTAAGTGTGACTCAACCAGCCTTTACAGTCAGTCATACACACACAAAAAAAACATT
CAGAAGAATAATATTTGCTGTCTTTATGAATAATCTCTTTCACATAGTAGACTAAGCGTTGCAAAGAGTCAAGAAAACGA
CTTGGTAGATCTCCAAACAAATCAAAGGTTTTGCCCAATGAAATCCTCCATAACACCAATATTTTCAGATAATCTTATGA
ATCTAAATTGGCAACAAAGGACAACGTTTTCACCCTCAGGGAGAGAGCACGATGCCAAGTTGCTGACTGGCTCCAGCTTA
TCTAAATGTATAGCTAGCTAAAGCCACAGAGCGGTCACACCTTAGGACTGTTAGGAGGGATGCCAGCTGAATTTTCATTT
AGCTACTCAGACATGGCTACAGTCTACTTGCTCTTGGAAAACAAATTCGTTTTGGGAACAATTGTTCCAGGAGGATTTGA
TGCACCTCTTAGTTTATGTTGCATTTTGACTGGGGCTGTATTTGGAGTGCATACATAATGACTATTGTCCTGATATTTTC
CTAATTATGTGAACTGACCAGGAAAAGGTCACAATATAGTCAGGAAAAACTCTGGCCCCTAATAGTGATGTTTGTCTCTG
TCATAACAGATCATGACGCCAGGGTGACTAGTGCCTGTAGTTGTCCTAGTCAGGTTATGCGGTTAGTACGATAACTACCT
CAATACAGATGTAGGACCTTAATTTGAGCCGATTTGCTACAGCAGGAAAACAATCCTGGAGCAAGAGGAAATATTAATTA
TTATGTGGATTATAACTAATGGACATTTTTGTAGAGGTTTATACATTTTTCATGAGGGCAAATCAAGTCAGACATTTTTA
AGCGGAAATTACAAACTTAAGAAGCCTTTTTAAGCTGAAATATACTACAAGTAAAAATGTCCTGCTGTGCAGGAAAATTC
TCAGAATAAAAAGAGTGATCAAATTAAGATCCTGTGTGCCACCCATATTTCCTTTACAAAGGACAATGATGGACTAGCAG
TTGGTTCATTGGTCTTGGGATTTTCCGCTGTGTATAATGGACATACACCCTTTTGGATTTCATAACCCTCTGTGATTCGT
TGACTTGGTTATCATCACAATGAGGATCCTTCCCTAAGGCGCTCGCTCAGCTCAGTACGTGGGCAGAGGAAAGTAGCATG
ACAGCAGAGTCATGAGAAAAGATTGCCATTTTGAGAAGTAGAGAGAATGTAGAGACCAGATAAAGAGAAGGAATGCAGAT
AAATGAAAGGAGGATGAAGGAGAAGGATGGAGGAGACAAGATACATTACTAAGTAAAGGGAAAGACGGGATCATGTGAAA
ACGTGAAGAGTGAGGAGAGAAAAGAGAGAGCGATGAGAGGCTGGAGAGAGGAGAGGATAAATGCACTTTTGAATTATGTA
AAATAGCTGCGTGGGAGAGAGGAGGAGTGTGTGTAGTGTGTATGCCTGAGTATTTGTGTGTCTGTGTGATTGCAGAACCA
GGGACAGTGAGAGAAGGAGGGAAAGAAAGAGAGCGGATGAAAGAGAGAGGGAGAAGAGAGTGAGGGATAAAGAGAGAGAC
AGAATACGAGAGAGAGAGAGAGAGAGAGACAGAGAGAATAAGAGACAGGGAGAATATTTATAGCCTCAGATATGACTATGTT
ATCATTGATCTATCTCAAAAGAGGGTAGCAGAGAAAGGGATAGGGAGGGAAAGAAAGAAAGACTGAAATGAACTGGCCTC
AGACACTTTCTCGCTTTCATTCCTGCTCCCTTCTTTCTATGATACGGCATACATTATTATTATTTGTATATATACAGTTG
AAGTCAGAAGTTTACATACACCTTGGACAAATACATTTAAACTCAGTTTTCCACAATTCCTGACATTTAATCCTAGTAAA
AATTCCCTGTCTTAGGTCAGTTAGGATCACCACTTTATTTTAAGAATGTGAAATGTCAGAATAATAGTAGAGAGAATGAT
TTTTTTCTGCTTTTATTTCTTTCACCACATTCCCAGTGGGTCAGAAGTTTACATACACTCAATTAGTATTTGGTAGCATT
GCATTTAAATTGTTTAACTTGGATCAAATGTTTCGGGTAGCCTTCCATAAGCTTCCCACAATAAGTTGGGTGAATTTTGG
CCCATTCCTCCTGACAGAACTGGTGTAACTGAGTCAGGTTTGTAGGCCTTCTTGCTCGCACACACTTTTTCAGTTCTGCC
CACAAATGGTCTATAGGATTGAGGTCAGGGCTTTCTGATGGCCACTCCAATACCTTGCCTTTGTTGTCCTTAAGCTATTT
TGCCACAACTTTGGAAGTATGCTTGGGATCATTGTCCATTTGGAAGACCCATTTGCGACCAAGCTTTAACTTCCTGACTG
ATGTCTTGAGATGTTGCTTCAATATATCCACATAATTTTCCATCCTCATGAAGCCATCTATTTTGTGATGTGCACCAGTC
CCTCCTGCAGCAAAGCACCCCACAACATGATGCTGCCACCCCCGTGCTTCACGGTTGGGATGGTGTTCTTTGGCTTGCAA
GCGTTCCCATTTTTCTTCCAAACATAACGATGGTCATTATGGCCAAACAGTTCTATTTTTATTTAATCAGACCAGAGGAC
ATTTCTCTAAAAAGTATGATCTTTGTCCTCGTGTACAGTTGCAAACTGTAGTCTGGCTTTTTTGTGGCGGTTTTGGAGCA
GTGGCTTCTTCCTTGTTGAGCGGCCTTTCAGATTGTCGATATAGGACTCGTTTTACTGTGGATATAGATACTTTTGTACC
TGTTTCCTCCAGCATCTTCACAAGGTCCTTTGCTGTTTTTCTAGGATTGATTTGCACTTTTCGCACCAAAGTACGTTCAT

CTCTAGGAGACAGAATGCGTCTCCTTCCTGAGCGGTATGACAGCTGCATGGTCCCATGGTGTTTATACTTGCGTACTATT
GTTTGTACAGATGAACGAGGTACCTTCAGGCGTTTGGAAATTGCTCCCAAGGATGAACCAGACTTGTGGAGGTCTACAAT
TGGCTTGGCTGATTTCTTTTGATTTTCCTATGATGTTAAGCAAAGAGGCACTGAGGTTGAAGGTAGGCCTTGAAATACAT
CCACAGTTACACCTCCAATTGACTCAAATTATGTCAATTAGCCTATCAGAAGCTTCTAAAGGCATGACATCATTTTCTGG
AATTTTCCAAGCTGTTTAAAGGCACAGTCAACTTAGTGTATGTAAACTTCTGACCTACTGGAATTGTGATACAGTGAATT
ATAAGTGAAATAATCTGTCTGTTAACAATTGTTGGAGAAAATGACTTGTGTCATACACACAGTAGATGTCCTAACCGACT
TGCCAAAACTATAGTTTGTTAACAAGAAATTTGTGGAGTGGTTTAAAAACGAGTTTTAATGACTCCAACCTATGTGTATG
TAAACTTCCGACTTCAACTGTATATATATACACATAAACATTCATACATATACACGTACACTACTGTTCAAAAGTTTGTG
GTCACTTGGAAATGTCCTTGTTTTTGAAAGAAAAGCAAATGTTTTGTCCATTAAAATAACGTCAAATTGATCAGAAATTA
ATGTTGTAAGTGACTATTGAAATTAGAAACGGCTGATTTTTTATGGAATATCTACATACAGTGAGGGAAAAAGGTATTTG
ATCCCCTGCTGATTTTGTACGTTTGCCCACTGACAAAGAAATGACCAGTCTATAATTTTAATGGTAGGTTTATTTGAACA
GTGAGAGACAGAATAACAACAAAAAAATCCAGAAAAACGCATGTCAAAAATGCTAGAAATTGATTTGCATTTTAATGAGG
GAAATACGTTTTTGACCCCCTCTCAATCAGAAAGATTTCTGGCTCCCAGGTGTCTTTTATACAGGTAACGAGCTGATATC
AGAACAACATGAGGAGAGAAAGGGCTCCTCCAGTCTAGAGTTGACTGAGCAGAGTTACTGTAGTGTTGTTGTGTTGCTGC
TCTAATGATAATGATGTTTTCTCTGTGGAAGGTTAGAAAGTCAAGTGAACCATTGTACTGCTGTCTCCTATCTCCTGGCT
GTTCTTCCCTCACGTCTCACTCATTTCTCTCTTTTCAGACTGTATTGTGGACCCACCTCTCTGTTCTTTCTCTCTCTCTC
TCCCTCTCTCTCTCTCTCTCTCTCTCTCTCTTTTTTCTCTATCTGTGAGATCTCTCTCTGGGGGGGTGAGGGGATAATGT
GTATACACTCCTCTGTGTGGGCGGCGTATATCTCTCTCCACAAAGATATATATACAACTGTGTGTTATATTGTGTGTCTG
TGTGTACACGGTTTTCTCTGTGTGTGTGTGTGGCTGCCGCTCGGCTGCTGCTGCTGCGATTTGAGGTAGAAAATGTGAGG
ATAATGAGGGTGCGGCCATAAAAACTAGCAACATGGAAAGACAGCCAAATAAGCACAAACGAGCACAAAAATAGAATTAA
GCGGAAACAAGGAAACCAGTGGGTATGGTTAGAAATATTGGCAACGGAAAAGACGGGGGAAGACAAGGAGAGACGGCAAC
CAAGGAAAGCAGGCTAAAAGGGGGGAATAGGAGAAGACGCAAAAAAATAAAGAGAGCAANNNNNNNNNNNNNNNNNNNNN
NNNNNNNNNNNNNNNNNNNNNNNNNNNNNNNNNNNNNNNNNNNNNNNNNNNNNNNNNNNNNNNNNNNNNNNNNNNNNNNC
TCCACCATGGCCAAGACCAAAGAGCTCTCCAAGGATGTCAGGGACAAGATTGTAGACCTACACAAGGCTGGAATGGGCTA
CAAGACCATCGCCAAGCAGCTTGGTGAGAAGGTGACAACAGTTGGTGCGATTATTCGCAAATGGAAGAAACACAAAAGAA
CTGTCAATCTCCCTCGGCCTGGGGCTCCATGCGAGATCTCACCTCGTGGAGTTGCAATGGTCATGAGAACGGTGAGGAAT
CAGCACAGAACTACACGGGAGGATCTTGTCAATGATCTCAAGGCAGCTGGGACCATAGTCATCAAGAAAACAATTGGTAA
CACACTACGCCGTGAAGAACTGAAATCCTGCAGCGCCCACAAGGTCCCCCTGCTCAAGAAAGCATATATACATGCCCGCC
TGAAGTTTTCCAGTGAACATCTGAATGATTCAGAGGACAACTGGGTGAAAGTGTTGTGGTCAGATGAGACCAAAATGGAG
CTCTTTGGCATCAACTCAACTCACCATGTTTGGAGGAGGAGGAATGCTGCCTATGACCCCAAGGACACCATCCCCACCGT
CAAACATGGAGGTGGAAACATTATGCTTTGGGGGTGTTTTTCTGCTAAGGGGACAGGACAACTTCACCGCATCAAAGGGA
CGATGGACGGGGCAATGTACCGTCAAGTCTTGGGTGAGAACCTCCTTCCCTCAGCCAGGGCATTGAAAATGGGTCGTGGA
TGGGTATTCCAGCATGACAATGACCCAAAACACACGGCCAAGGCAACAAAGGAGTGGCTCAAGAAGAAGCACATTAAGGA
CCTGGAGTGGCATAGCCAGTCTCCAGACCTTAATCCCATAGAAAATCTGTGGAGGGAGCTGAAGGTTCGAGTTGCCAAAC
GTCAGCCTCGAAACCTTAATGACTTGGAGAAGATCTGCAAAGAGGAGTGGGACAAAATCCCTCCTGAGATGTGTGCAAAC
CTGGTGGCCAACTACAAGAAACGTCTGACCTCTGTGATTGCCAACAAGGGTTTTGCCACCACTAAGTTAGTTTCCCTCAT
TAAAATGCAAATCCATTCATAATATTTTTGACATGCGTTTTTCTGGATTTTTTTGTTGTTATTCTGTCTCTCACTATTCA
AATAAACCTACCATTAAAATTATAGACTGATAATTTCTTTGTCAGTGGGCAAACGTACAAAATCAGCAGGGGATCAAATA
CTTTTTTCCCTCACTGTAGGCGTACAGAGGCCCATTATCAGCAACCATCATTCCTGTGTTCCAATGGTACGTTGTGTTTG
CTAATCCTAGTTTATCATTTCAAAAGGCTAATTGATCATTAGAAAACCCTTCTGCAATTATGTTAGCACAGCTGAAAACT
GCTGTTCTGATTAAAGAAGCAGTAAAACGGGCCTTCTTTAGACTAGTTGAGTATCTGGAGCATCAGCATTTGTGGGTTCG
ATTACAGTCTCAAAATGGCCAGAAACACAGAAATTTCTTCTGAAACTCATCAGTCTATTCTTGTTCTGAGAAATAAAGGA
TATTCCATGCGTGAAATTGCTAAGAAACTGAAGATCTCGTACAACGCTGTGTACTACTCCTTTCACAGAACAGCGTAAAC
TGGCTCTAACCAGAATAGAAAGAGGAGTGGGAGGCCCCGGTACACAACTGAGCAAGAGGACAAGTACATTAGAGTGTCTA
GTTTGAGAAACAGACACCTCACAAGTCCTCAACTGGCAGCTTCATTAAATAGTACCCGCAACCCACCAGTCTCAACGTCA
ACAGTGAAGAGGCGACTCCGGGAACAGAGTTCCTCTGTCCAGTGTCTGTGTTCTTTTGCCCATCTTAATCTTTTCATTAT
ATTGGCCAGTCTGAGATATTACTTTTCTTTGCAACTCTGCCTAGAAGGCCAGCATTCCGGAGTCGGGATAATGGGCCTCT
GTACGCCTATGTAGATATTCCATTAAAAATCTGCCGTTTCCAGCTACAATAGTCATTTTCAACATTAACAATGTCCACAC
TGTATTTCTGATCAATTTGATGTTATTTTAANNNNNNNNNNNNNNNNNNNNNNNNNNNNNNNNNNNNNNNNNNNNNNNNNN
NNNNNNNNNNNNNNNNNNNNNNNNNNNNNNNNNNNNNNNNNNNNNNNNCAGTAAGAGATATGCTAACATGATTGCTA
TTGCTAGCTAGATAGCTTGCTAAAACAATTGACAAGCAACAAGCATCTTAAGATTTATAAGAAGATATTTGAGAAGTTTG
TAAAAAAGTTATAAAATCTTAAGGTATTATTGAGGAATTGCACTTATTGACTTCTTTTTTTTTTCTTAAGAAGCTTCTTA
AGTTTTTTCGTGACTTGTTTTGTGAATCTGGGCCCTGGTTGGTTAACACCAACACTCCAAACCCCTGGGTCCTCCTTCAA
CCCCCCTTCCACATTCCACTCTGTCCCTTAACATGTCACATTGTAAACCTGTGCCCAGTGGCCCATTGTGAAACATGGGT
AAATTATCTGCAATGTGACTGTACAAAACCTTATTTTTTCCTAGCCCTTCGAGACTGATAGGAACAATTGTCCTGGAACA
TCACCATTAGGGTCAGCGTGCAGTTTTGGTGCTTTGTGAAAGATCCTGGGGATGGGAATAGACAGACAGGTGATGTGGGA
TGGAATGCTAGTCTTGACGAGAAACATGCTGAGGAACACTTCAAGCCACTCCCCAAAGGACCATCTGGCTCTGCACCTCG
CATCATTGAGTTATCATGACAGATAACAGACATTTTTATGTCTTTAGGTATCTAGAGTCTTGACCTGACAGGACTTTTAT
CCTCCAGGGACTAGCTTAGCAGTTTCCCTCAAACTCTAAAAGGTATAAAAGTTCATCACAAACCCAGAGTCCTCAAACTA
AGTCTTAGTATAAAAATATGTAATAATCCACTCTGGCCGATGGATGGGTTTCACTGACCGAGTAGCTATTATTCAGCAAG
ATCCTGATCATTGTGAAGGGCATTATGTTTTCAATATCAATGAAAGTTCAAATGATTGGAGGAAGTGTTTAGATCTGATG
AGAAGTCCATAGGAAGCTGAAAACCGTAACCTGAAGCNNNNNNNNNNNNNNNNNNNNNNNNNNNNNNNNNNNNNNNNNNN
NNNNNNNNNNNNNNNNNNNNNNNNNNNNNNNNNNNNNNNNNNNNNNNNNNNNNNNNNNNNNNNNNNNNNNNNNNNNNNNN
NNNNNNNNNNNNNNNNNNNNNNNNNNNNNNNNNNNNNNNNNNNNNNNNNNNNNNNNNNNNNNNNNNNNNNNNNNNNNNNN
NNNNNNNNNNNNNNNNNNNNNNNNNNNNNNNNNNNNNNNNNNNNNNNNNNNNNNNNNNNNNNNNNNNNNNNNNNNNNNNN
NNNNNNNNNNNNNNNNNNNNNNNNNNNNNNNNNNNNNNNNNNNNNNNNNNNNNNNNNNNNNNNNNNNNNNNNNNNNNNNN
NNNNNNNNNNNNNNNNNNNNNNNNNNNNNNNNNNNNNNNNNNNNNNNNNNNNNNNNNNNNNNNNNNNNNNNNNNNNNNNN
NNNNNNNNNNNNNNNNNNNNNNNNNNNNNNNNNNNNNNNNNNNNNNNNNNNNNNNNNNNNNNNNNNNNNNNNNNNAACGCGA
GAGAGCTGGATGCATCAGGGACATCATAGTAGGGAGACTGTTATTTCTTTTCTATGCTTTTTCTTCATTGTGGTCTCTGT

CATTCCCTTTTCTGGCACTCCATATCTGTCTTCTCTTCAGTTCAGTGGCATTGTAGCATAATTGGTATGTGTGTGTCTAT
GTGTGTGCATGTATAAGTCAGTGTGGGCGGGCATGGCGGGAAAGGTCGTCAAGCGTCACGGACAATTCTATTCAGGTGTT
GCTCTGTGCCTTATGCATGTCCATCTGTAAATAGCCCGCCCAATCTACCTACCTCATCCCCATACTGTTTTTATTTACTT
TTCTGCTCTTTTGCACACCAGTATTTCTACTTGCACATCATCATCTGCTCATTTATCACTCCAGTGTTAATCTGCTGAAT
TGTAATTCTTCGCTACTATGGCCTATTTATTGCCTACCTCCTCATGCCTTTGGCACACACTATATAGACTTTCTTTTTTT
CTACTGTGTCATTGACTTGTTTATTGTGTTACTGGCTTGTTTATTGTTTATTCCATGTGTAACTCTGTGTTGTTGTCTGT
GTCACACTGCTTTGCTTTATCTTGGCCAGGTCGCAGTTGTAAATGAGAACTTGTTCTCAACTAGCCTACCTGGTTAAATG
AAGGTGAATTTTTTTTTTTTTTTTTTAAATGTGTGGCTGTCAATAAAAACGTAGCTTTGCTGTCCAAGAAATATAATGAT
AACACTATTGTCATTATGCTCAGATCTAATCTAATCCAGTGAAACTCTAATTGATTGGTAATGTGTTGACTTCTTCAGAT
GACAATTAGACCCATTGTGAGACTAGCATCGCATTGTTTTTAATGTGACCTCTTTTCAAGCTCACAGAACACAGACTTT
GTGAGGATAGACGGATTCCTTTGGCATGAAAAGCAGAATGGTTGCGTGCTGTTTCTCAACTTTTCGGAATTAGATATGCT
CGAGTGTATCTCACAAAAACTCCACCGGGCTTGCCGTTGTGAAAGATCTCTGGCTCTCTGTGCCCTCAATGACTGATTGT
ATTCCACTCCCCTTGTCACAGTTAGATTTGTTAGTGAGATTGCAGGGAGCTATAAACAGAGGGTTATCCCTTTAGTTGAA
GAAGTGTATGCCGACATTCAAATGAAAAATATTGACAGTGGTGGCATTTGTTTGGCCACATGGTGCCAGTTGCCTTGTTG
ACGTCATCACCCCCTAATCAAATCAAATCAAATTGTATTTGTCACATGCGCCGAATACAACAGGTAGACCTTACAGTGAA
ATACTTACTTACAAGCCCTTAACCAACAATGAAGTTTTAAGAAAAACAAATAATTAAGGAGCAGCAGTAAATAACAATAG
CGTGGCTATATAGAGGGGGTACTGGGGGGGTGGGGGGGGGGTGCAAATAGTCTGGGTAGCCATTTGATTAGCTGTTCAGG
AGCCTCATGGCTTGGGGGTAGAAGCTGTTTAGAAGCCTCTTGTTCCTAGACTTGGTGCTCCGGTACCACTTGCCATGCGG
TAGCAGAGAGAACAGTCTATGACTAGGGTAGCTGGAGTCTTTGACAATTTTAAGGCCTTCCTCTGTCACCGCCTGGTTAT
AGAGGTTCTGGATGGCAGGAAGCTTGGCCCCGGGGATGTACTGAGCCGTACGCACTACCCTCTGTAGTGCCTAATCAAAT
CAAATCGTATTTGTCACATGCTATGTAAACAACAGGTATAGACTAATAGTGAAATTCTTACTTACTGGCACTTCCCAACA
ATGCAGAGAAAAAAATGGAATAATAATAACACGAGGAATAAATACACCATAAGTAACGATAACTTGGCTATATACACGCA
GTACCAGCACTGAGTGGATGTGCAGGGGTAACAGGTAATTGAGGTAGATATGTACAGGATAAAGGTAGGGATAAAGTGAC
TAGGCAACAGGATATATAATAAACAGTAACAGTAGCATATGTGATGAGTCAAAAGAGATGCAAAAAAAGGATCAATGCAA
ATAGTTAAATAGTTAACCAATAGCTACATGGACTAGCTATTTAGCAGCCTTATGGTTTGGGGGTAGAAGCTGTTCAGGGT
CCTGTTGGTTCCAGACTTGATGCATCGGTACCGCTTGCTGTGCGAAAGCAGAGAGAACAGTCTATGACTTGGGTGGCTGG
AGTCTTTGACAATTTTTAGGGCCTTCCTCTGATAGGGAGCTCGGCCCCAGTGATGTACTGGGCCGTACGCACTGCCCTCT
GTAGCGCCTTGCGGTTTCCATACCAAGTGGTGATGCAGCCAGTCAAGATGCTCTCAATGGCGCAGCTGTAGAACTTCTTG
AGGATCTGAGGGTTCATGCCGAATCTTTTCAGCCTCCTGAGGGGGAAGAAGCGTTGTCTTGCCCTCTTCATGACTGTGTT
GGTGTGTGTAGACCATGATAATTCCTTAGTGATGTGGACACCGAGGAACTTGAAGCTATCGACCTGCTCCACTACAGCAC
CTTCGATGTAGATGGATGCGTGCTCGGCCCTCTGTTTCCTGTAGTCCACGATGATAGAATAGTCTCGAATGGACCTAATC
CAGTTTATTTATCCACTCTCTGACGTAGTCTCATCAGGTATCCTGTACACCAGCCTCTATAGCGGGCGTCTCCTCCTTCT
TCGGGTGTCAGGGATTTGGGACAGGCACGGGAAAATCAATATCTTTATTCTCCGACTCATTGAAGTAGAAGTCCTCGTCC
AAATCGAAGTTGGTGACAGCTGTTCTGATGTCCAGAAGCTCTTTTCAGTCATAGGAAATGATAGAGGAAACATTATGTAG
AAAAAAAAACACAGTTCAGAATGCTTTGTACCATAATGTATGGGACTCACTCTGCTCCTCTCACTGTGTCTCTGCTCCCC
ACCCAGAACGCGCCTGATGCAGAGCTTCAAAGAGTCCCACTCCCATGAGTCCTTGCTGTCTCCCAGCAGCGCAGCCGAGG
CGCTGGACCTCACCCTGGACGAGGACGCCATCATCAAGCCCGTCCACTCCAGCATCCTGGGACAGGAGTACTGTTTTGAG
GTGAGGCTGACACGACACACACACAATGAACCCAGATAGCGCTACTAATTAAACCTGGGTTCAAATACTACAAGAAGAGA
ACGTTCAAATCCCACCCATCTGCCACTCCAGGCAGGCTAAAGCAAACACTCAAAGTATTTGAAAGATTTTAAATAGCATT
TGAACCCAGGTCTGCCTACTATTGCTACTAATGCTACTAATGTTGGGTAGAAGTCTCTCGATTGGCATCAACATAAGGAC
TTATTGAATAAGGCTGTGATTTTTAAAATCTTGGTATCTGTGGAAAAGATATCAAAGAGAAAGCCAACAAGCGGCTCGAA
ATGAGCTCATGTCCCAAGTTGATTTGTCACAGAAATTTGTTGTGAAAATGGCTTTCACTGCATGGCTCACTTTCCCATAG
CCAACGTGTACATGACACAAGTCTCTGTGATTAAAGGACGTTGTTATGCCACTTGTCATCTTCTCCCAAGTTGCTAATGG
TTGTGGCTGTTGATGTTACCGTTGGTAACCTCAGGTGACTACGGCTTCGGGAACCAAGTGCTTTGCATGTCGCTCGGGTG
CAGAGAGAGACAAATGGATCGAGAATCTTCAGAGAGCTGTCAAACCCAACAAGGTGGGGAAACAAGGACGAGAAATTAAG
TTACTTCTCGTTCTGTCAGTGCTTCTCTCCTTCTCCACTGTCTTCAATATGTCTGTTTCAATATGAGCATTTCCTCATTT
TGTTTACTGTAACCTCCATGTGCTCTCTGTTTGTCTATGTCTGTAATGTATAGTCATCAAATACAGTACATATTAAAACA
TAGCACTTCTACACTATCAGAGTTAAATAAATAACTAAAAAGACGCAAACTCAGCACTTTAGATGCGTTTGATTAAAACA
GATCTTCTAATTTCAGCTACACTTGATCTCCTAGGTCTGCGTAATAGGCCCCTTCCTGTCCCGTCCCCCCCTCTCTCTTA
TCAAAAGAGACATTACGCGAGACAGATAAAACTGCTCTTGATGGCAGCAAGGGAGCTGCAGTTATTAAGGTAAACGGAGA
GGAAACATCAGCCCAAGTGGATTGACTCATCCAGATGCTGTGACTGCAGGGTTCCTGGCTGAATCATGGGGCTCCAAGGA
CTCGACACGAGGGCTCCGAAATATCCTCTGCACCCTCATGAATCTCCAACCAGATACATGATGGGAGAGATAAGACGCGG
TGTGATATCGATAGAGACCAGGATGTTCAATAGGTTTCGCAACCGTCGCTGGGCAGACAGACGCTCTTGATTCTTCTTCT
TATTGAAATTCATGATGAACGGTTGAAAGGGTTGCATCGGCGGACGTCCACATTTATTGTCAGGCGGCATACCAATGGAG
TACTTAAGAAATAACAACTTTGGAAAATTGCTGATATACTTAGCAATCTAAATTCGGTATTAAGAGCTGATCTGAGCCAG
GGCTGAGCAATCCAGTGGCATTTCAGACTAGTTGAAATGACTCACCCCTGAAAAAAAACATCACTCTGTGCAATCTGCAC
AGCATGCTCTCCTCCCATGGGGGATTTCAGACATAGTTGGCTCTTTGTAATCATGTTCAGAAAATGCAGCTCTCTGCTGC
AACATTACCAATCACACATCCACTTTCTGTCTCTCATGCCTCCAGTACATATGTATTATCTAATTTATCTCCATCTGTAT
GTGTGCATATTGTTCTTAGACTGTTCTTAACTAGTGGTGGAAAAAGTACTGAATTGTCAAACTTGAGTAAAAGTAAAGAT
ACCTTAATAGAAAATAACTCAAGTAAAAGTGAAAGTCACCCAGTAAAATACGACTTGAGAAAGTCTAAAAGTACACTGCT
CAAAAAAATAAAGGGAACACTTAAACAACACAATGTAACTCCAAGTCAATCACACTTCTGTGAAATCAAACTGTCCACTT
AGGAAGCAACACTGATTGACAATACATTTCACATGCTGTTGTGCAAATGGAATAGACAACAGGTGGAAATTATAGGCAAT
TAGCAAGACACCCCCAATAAAGGAGTGGTTCTGCAGGTGGGGACCACAGACCACTTCTCAGTTCCTATGCTTCCTGGCTG
ATGTTTTGGTCACTTTTGAATGCTGGCGGTGCTTTCACTCTAGTGGTAGCATGAGACGGAGTCTACAACCCACACAAGTG
GCTCAGGTAGTGCAGCTCATCCAGGATGGCACATCAATGCGAGCTGTGGCAAGAAGGTTTGCTGTGTCTGTCAGCGTAGT
GTCCAGAGCATGGAGGCGCTACCAGGAGACAGGCCAGTACATCAGGAGACGTGGAGGAGGCCATAGGAGGGCAACAACCC
AGCAGCAGGACCGCTACCTCCGCCTTTGTGCAAGGAGGAGCAGGAGAAGCACTGCCAGAGCCCTGCAAAATGACCTCCAG
CAGGCCACAAATGTGCATGTGTCTGCTCAAACGGTCAGAAACAGGCTCCATGAGGGTGGTATGAGGGCCCGACGTCCACA
GGTGGGGGTTGTGCTTACAGCCCAACACCGTGCAGGACGTTTGGCATTTGCCAGAGAACACCAAGATTGGCAAATTCACC

ACTGGCGCCATGTGCTCTTCACAGATGAAAGCAGGTTCACACTGAGCACGTGACAGACGTGACAGAGTCTGGAGACGCCG
TGGAGAACATTCTGCTGCCTGCAACATCCTCCAGCATGACCGGTTTGGCAGTGGGTCAGTCATGGTGTGGGGTGGCATTT
CTTTGGGGGGCCGCACAGCCCTCCATGTGCTCGCCAGAGGTAGCCTGACTGCCATAAGGTACCGAGATGAGATCCTCAGA
CCCCTTGTGAGACCATATGCTGGTGCGGTTGGCCCTGGGTTGTCTCCTAATGCAAGACAATGCTAGACCTCATGTGGCTG
GAGTGTGTCAGCAGTTCCTGCAAGAGGAAGGCATTGATGCTATGGACTGGCCCGCCCGTTCCCCAGACCTGAATCCAATT
GAGCACATCTGGGACATCATGTCTCGCTCCATCCACCAACGTCACGTTGCACCACAGACTGTCCAGGAGTTGGCGGATGC
TTTAGTCCAGGTCTGGGAGGAGATCCCTCAGGAGACCATCCGCCACCTCATCAGGAGCATGCCCAGGCGTTGTAGGGAGG
TCATACAGGCGCGTGGAGGCCACACACACACACTACTGAGCCTCATTTTGACTTGTTTTAAGGACATTACATCAACGTTG
GATCAGCCTGTAGTGTGGTTTTCCACTTTAATTTTGAGTGTGACTCCAAATCCAGACCTCCATGGGTTGATAAATTGGAT
TTCCATTGATCATTTTTGTGTGATTTTGTTGTCAGCACATTCAACTATGTAAAGAAAAAAGTATTTAATAAGATTATTTC
TTTCATTCAGATCTAGGATGTGTTGTTTAAGTGTTCCCTTTATTTTTGGAGCAGTATATTTAGTTTTAAATATAATTATG
TTTCAAAAGTAAATTAAATTGCTAAAATATACTTAAGTATCAAAAGTAAAAGTATAAATCATTTCAAATTCCTTATATTA
AGAAATCCAGATGGCACAATTGTGTTTTTTACATTTTTATTGACGAATAGCCAGGGGCACACTCCAACACTTAGACATAA
TTTACAAACGAAGCGTTTGTGTTTAGTGAGTCCGCCAGATCAGAAGCAGTAGGGATGACCAGGGCTGTTCTCTTGATAAG
TGTGTGAATTGGACCATTTTCCTGTCAAAATGTAACGAGTACTTTTGGGTGTCAGGGAAAATGTATGGAGTAAAAAGTAC
ATTATTTTCTTTTGGAATGTAAAGTAAAAGTTGTAAAAAATATGAATAGTAAAGTACAGATACCCCAAAAAACGACTTAA
GTTGTACTTTAAAGTGTTTTTACTGAAGTACTTTACACCACTGTTCTTAACCTACTCAACCCTCCCTTTCTCTTTGGCTT
TCTTTTTCTCTTTGGTCTTTTTCCCTTTGGTTTTCGTTCCTCCCTGTTCTGCTTTGTCCTTTTGCCACCCTCTCTTTGCC
CCTCTTCAGGACAACAGTCGGAGGGTGGACAACGTGCTGAAACTCTGGATCATCGAGGCACGGGAGCTTCCCACCAAGAA
GCGCTACTACTGCGAACTTTGTCTGGACGACATGCTTTACGCACGCACCACCAGCAAGCCCCGGACTGACACCGTGTTCT
GGGGCGAGCACTTTGAGTTCAACAACTTGCCAGCTGTCCGCAACCTACGCCTTCATCTCTACAAGGAGACTGACAAGAAG
AGACGCAAGGTGAAGGGACAACTCTCTCACCAGTGTGCAAAACAGTATGAAACTGGTTAAAATGACATTTTTGCAGACTG
GTTAAACCAGCTTGCCCAATGATTAAGATGGATGAATATACTTTAGTTTCATTACTATCTTATTTTTCTCCTCTTTCTTA
AAGGCCCCATAACTCTTGACCACAAAATCCCACATTTATTTCCCCTCTTCTATCAGGAAAAGAGCACATACCTGGGACTG
GTCAGTATCTCTATCTCCAGCATCACGGGCCGACAGTTTGTGGAGCAGTGGTACCCGGTCATCCAGCCTAGCATCCTGGC
CAAGGGGCAAGCAGGGGGCAAGATCATCAATGCCTCCCTACGCCTCAAGTCCCGTTACCAGACCATGAGTATCCTGCCCA
TGGAGCTGTACAAGGAGTTTGCCGAGTATGTTACCAATAACTACCGGACTTTGTGTGCGGTGCTGGAGCCTCTACTGAGC
GTGAAGAGTAAAGAAGAGGTGGCCTGTGCGCTGGTCCACATACTACAGAGCACGGGGAAAGCAAAAGTAAGTATTGGCAT
GAAACATTAAACAAGTTGTTACTTCAGTAGTACACGGTACAATGCAAGGTCATATTCCTGTATACACCTCACTGTATCCT
GTGTATATGCCAGATAAATGCTGTTTTTGATTTGATGGCATAATATTGATGGAATATGACTGCCAAATAGGTCAATGTCA
TAACAGTGTCCTTTAAATACATAGGATGTGGTGTTGCGCTGTAACCAGTGTAACATGTGTCATGCTAATGTAACCATCCT
ATTACTTGATGGCCCATGGTCTACCAATATGCCATGCAGATGAGCCATTGTTTACTTTACTGACCACACTGGATATTCAT
TCAGTCCTTGAAGTTGGGCAGTTCACGGTAGGACTCAACGCCATGCTGTGTAAGCAATATGCTGAACAGGCAAACATAGC
TTCCATTGGTCGATTACCAATAAAATGTATTATTCAATCACAAAACATTCCATAAAAATGTTTACCTCTCTTTTGGATTT
TTCCACAATCACAACTTCTACAACCTCAGGTTCCCTCGAAATCAGACTGTCTCCACCAGAATCTTTCTGAGAATGTTTTG
TTGTTGTTGTGTGATGTCACTGTACCGAATGTATAGCTAGATGATGCATTGTTTGGGGTGGTCCTAGGTTGAAAATGCTG
TCAACCACCTAGCATTATCATCTACAAACCAAACACACTATCATGTCAACAATCTGCATATTTAGCTTTACGTAACTAGC
TAACGTTAGCTAGTTGAAATAAAGGCATTGGGCATCTTGTGTGGTTTATGCAGTGTAGCTACATGATGTAGCTAGTTATC
GTTAGTTTCCAAATACAAAATATGTCAGCTAGCTAGCTAAGTAGCCATGTTAGCAAGCTTGAACATTGAACACACTAAGG
TTAGCAGCTAATGTAGCTAAAAAAACGTAGGCTAAAAACGCAATCCAACACTTACCTACCTGTCCATGACAATGGTGGCC
ATTTCGGGCATCACTCTTCCACCTTTCAAAATTAATTCCAATGTTTATCCGGTTTCTTGATCAGTCTTTTTTTCTCTTCT
TTTTCTCTTTTGGGGTCGAGTTGATTCAGATCCAAAAACCGAGAATGATGCTTGTTTCTGTCTACATTTTCTGAAAATGC
TGGCCCTGCTACTAGGACAGTTTATCAGCAAATAAGTTAGCTAGCTTGTTGGCTTCGTCTGCTCTGTGTTTGTTTCTTGC
GGCTGTAGCTTGCAAGTGACTTTCACTTCTTCTCCAGCTCTGGAGCATGGCAGCAGGGTCATTCGAGTAGGCAGGTTTTA
GTGCAAAGACACACCTTGCCCAGAAGGCCAGCCCAAGGCGGCTCAGGGCTCAATCCCCTTGTTTTCGTTCAAAGTGAAAA
CACAACAGAAGAAGTGGGGCGGCATGGGGGCGCCCATAACCTGCGGATTCTATCTTTTAGGCACTATAAACTACAAAATT
AACATTAGTTTGACTACTTCTTGGTTTTCTTACCAAACCATAAACCAAAAGAGATTGAAACAGAAGGAATATTAAACATA
ACAAATTACAATCTGACCTGAGGAAAGGCGTTATATCCTCAAAATTGAATTTGAGAGTACCCAATAGATTATAATTGAAT
GTTTTAAAAAGAAAGTCTATGCTATGTTGATGTTTGTTTTGTTTCTTCCCAGGATTTTCTTTCAGACATGGCGATGTGTG
AGGTAGATAGATTCATAGACCGAGAACACCTTATCTTCAGAGAGAACACTCTGGCCACCAAAGCCATAGAAGAGTATCTC
AAGCTGATTGGACATAAGTACCTCAAGGATGCAATAGGTGTGGCCTTCGAATGCTTCACATGAATCACTCAATACATGTT
GTTGAACTGTTTTTGCTGTGATGTATTCATTATCTTTCTGTGTTTGCCTAATGGTCAGTGATATTGTGTTTCTTTCAGGG
GAGTTCATAAGGGCGCTGTACGAATCAGAGGAGAATTGCGAGGTGGACCCCATGAGGACACCACCCTCTGTCCTTCCAGA
CCACCAAGCCAATCTCCGCATGTGCTGCGAACTGGCACTCTGCAAAATCGTCAACTCCCATTGGTCAGTAGTTGGCATAT
GAGTACTTACTGTTTTTTTCATTCAAGTAGGATTACTCCTGGGCTACAGCACCCAAATACTTATTTACAGAATTTTGATT
GTTGAACACACCATATTTGCATTCAGTAGTTGGCGTACACTGCCTTACTAAAGCAGAATATCCTATCAAATGAGAATAAC
ACGCTGTATATTTGTATCTCTCTACAGTGTGTTTCCTCGAGAGCTGAAGGAGGTGTTTGCCTCGTGGAGGGTGCGCTGTG
CAGAGAGGGGGAGGGAGGACATCGCAGACCGCCTAATCAGCGGCTCCCTTTTCCTGCGCTTCCTGTGTCCGGCCGTCATG
TCCCCCTCCCTGTTCAACCTGACCCAGGAGTACCCAGATGAGCAGACATCACGCACGCTCACCCTCATCTCCAAAGTGGT
GCAGAACCTGGCCAACTTCTCCAAGTCAGTTCATTCATTCACAACAATTTATAATGTTATGCTTATGCTAAAACTTCTGT
AATATTTTGAGTATTTTTCCGGGACACAGATTAAGCCTAGTCCTGGACTAAAAAGCATGCTCAATGTAGATTCTCCATTG
AAATGTCTTATTAGTTATTAATCCAGGATTGGGCTAGTTCTGTGTCTGGGAAACCAGCCAATGGTGTGTAATCTGAGTAT
ACAGCATTTTGACTCTTCTTCTTCTCTCTCTCCCTCAGGTTTGGTATGAAAGAGGAGTACATGTGCTTCATGAATGAGTT
CTTAGAGATGGAGTGGGGCTCCATGCAACAGTTCCTTTATGAGATCTCCAACCTGGACAGTGTGAGCAACGCAGGGGGCT
TCGAGGGCTACATCGACCTGGGCAGGGAGCTGTCCATACTGCACAGCCTCCTCTGGGAGGTCATGGCCCAGCTCAGCAAG
GTAGGGAGGATCAGGGTAAATATTAACAATCTAACCTCTACAATATATGACCAATAAAAAAAAGTAAAAGTTACAGATAA
TCATTATTGAATAACACTACAATGGTTAAGTCCAATTAGGAATCAGGACAATAAAATAGCTATGTATGGTCTTGACAACT
CAGTCATCTGAAAGTATGAGACAGTAGAATGAGTGGAATGATTAAGTGCACTTGCAATTTTCAAAGATAACATACTTAAA
ATGATCAACCACACTTTTTAACCATTTCAATGCTTTTCCTATTGTCATTGCTAACTTGTATTCATGTCAGTCTTCTAACC

```
CTAACCCACAGGATGCCATTATCAAACTGGGTCCCTTGCCCCGCCTCTTGAATGACATCAGCATGGCCCTGCGTAACCCC
CACCTGCAGAGGCAGCCCAGCCACCAGAACCAGACAGATAGAGTCCATGAGAGACAGACGGAGAGGCTGCTCTCACGACC
CAGCTTCAACAGGGGAGTCTCCTCGGAGTTCCAGAATCTCATGATGAAGGATCTCAACAGGTAGTGCCCTGGCTTTATTT
AATCCTATCGGCCTATTTCCTGGAAACAGGTTAAGTATAGTCCTGGACAAAAAAGCACTTTGAATGGAGAATTTCCATTG
AACATACTTTTTAGTCTATGATTAGGTTTAATCCGTGTTGGGGAAACCAGCCCTAGGGGGATGGTTGGTTGGTGCATTTG
TTAAAGTTTACCTTTCCTGCCATAATGCTNNNNNNNNNNNNNNNNNNNNNNNNNNNNNNNNNNNNNNNNNNNNNNNNNNN
NNNNNNNNNNNNNNNNNNNNNNNNNNNNNNNNNNNNNNNNNNNNNNNNNNNNNNNNNNNNNNNNNNNNNNNNNNNNNNNN
NNNNNNNNNNNNNNNNNNNNNNNNNNNNNNNNNNNNNNNNNNNNNNNNNNTGTGTTGTTGCCTTTCTTAAGCTCCATAGA
TATCACTCGCTTGCCTTCCCCTACATCTGGTGTGTCCGGTGGGGGGGTCATGCCGTCTCGTGCTCATCCTCAGCTGAGTT
TCCAAGAGCGTGATCACCCACATCGAGCCTCCAAAGATGTTTTCTATGTAACACGCCCTCCATTGGCCCGATCCAGCCCT
GCCTACTGCACCAGTAGCTCCGATATCACCGAGCCAGACCACAAGGTTAGTTGAATTCTAGAGTTTTGATAGCATTAAGA
TCTGTTAGGAGCCATGTTCTACAGGAAACTATGTAAACTGCACATCTCCTTTCCATGTGGTAGCCAAGTCTGGCAGACGA
CTGCAGGATAGTATGGCAGCTGTGCGCTCATAGGAAAATGATGCATGTACCTTAGTGGTAAGTCATGGGACATGCAATTC
AGGCATACAACCACCCCTCTTAATAGATAAAACACTTAATCAACTTATTGGCACTTTAGAATTGGGCACAGAAGGGTGTT
AGCTCTATCAATCTCGTCTAGGTGCTCAGTGTCAATAAAGTGTATATCTATGATGGACCTCCAGGACTCCACAATGAACA
GCGTGTCTAACCTGCAGTCGGTGGCCATGCTCAACTCCTCCCAGGCCTCCATCACCGGCATGGGCAGCTTCGGAGGGCTC
AGCAGCCAGGGTGGAGGAGGCCTGGGCTCGGGCCTGAGTGGCCAGCTCCGGGCCGGGGGCCGCCTCTCGGCCGGCTCTGG
GGGATCCAGCATGTCAGGGGGCCTCCGGCTGAGCCAGTTGAGCCAGATGGGCACCACCACCGACTCGCTGTCCCAGCAGC
AGCAGCAGCAGGCCGCCGCCCTGAGATACCCGCTCTCCTTCCAGAACCCCCTGTTTCACCTGGCCACCGGCACTGCAGAT
GGACCCCACCTTCACCACCAGCACAGCAGGGCCCATCTCCCGCCGCCCCTGCTCCTGGCCCCGGAGCCTGACCCCGGTGG
CTCGCACCAGGCCTACATCCCCCAGTTCGCCCACGGGGGGTTCTCCCGCAGCGAGGACCTGTCCACCCTGAGGACCGGGG
CAGGGCACCTGGGGCAGCCAGCCATTATCCACTCACACAGCTATAGTGACGACTACAGCAGGGCTGATTTCGCACGGCGG
CAACTGTCCATGCACGGGCAGGTATGATTTAACTTAAGGTCAAGAATGTGTTTACAATCATGTTCTACTTTTGTGCAAAT
TAGCCTTTTTGTCTCACCTACTTTATAATAATTTAAGTCAATATTTGTTTGTAGCCTACATAAATAGGATTAGCATAATG
TAAACATCTGAAACAGGTTTAAGACCAGGATAACTTGGAGTTCTCTAATTGTCCTTGTTAGGATAATCTTCAACAGCAAC
AGCAGANNNNNNNNNNNNNNNNNNNNNNNNNNNNNNNNNNNNNNNNNNNNNNNNNNNNNNNNNNNNNNNNNNNNNNNNNN
NNNNNNNNNNNNNNNNNNNNNNNNNNNNNNNNNNNNNNNNNNNNNNNNNNNNNNNNNNNNNNNNNNNNNNNNNNNNNNNN
NNNNNNNNNNNNNNNNNNNNNNNNNNNNNNNNNNNNNNNNNNNNNNNNNNNNNNNNNNNNNNNNNNNNNNNNNNNNNNNN
NNNNNNNNNNNNNNNNNNNNNNNNNNNNNNNNNNNNNNNNNNNNNNNNNNNNNNNNNNNNNNNNNNNNNNNNNNNNNNNN
NNNNNNNNNNNNNNNNNNNNNNNNNNNNNNNNNNNNNNNNNNNNNNNNNNNNNNNNNNNNNNNNNNNNNNNNNNNNNNNN
NNNNNNNNNNNNNNNNNNNNNNNNNNNNNNNNNNNNNNNNNNNNNNNNNNNNNNNNNNNNNNNNNNNNNNNNNNNNNNNN
NNNNNNNNNNNNNNNNNNNNNNNNNNNNNNNNNNNNNNNNNNNNNNNNNNNNNNNNNNNNNNNNNNNNNNNNNNNNNNNN
NNNNNNNNNNNNNNNNNNNNNNNNNNNNNNNNNNNNNNNNNNNNNNNNNNNNNNNNNNNNNNNNNNNNNNNNNNNNNNNN
NNNNNNNNNNNNNNNNNNNNNNNNNNNNNNNNNNNNNNNNNNNNNNNNNNNNNNNNNNNNNNNNNNNNNNNNNNNNNNNN
NNNNNNNNNNNNNNNNNNNNNNNNNNNNNNNNNNNNNNNNNNNNNNNNNNNNNNNNNNNNNNNNNNNNNNNNNNNNNNNN
NNNNNNNNNNNNNNNNNNNNNNNNNNNNNNNNNNNNNNNNNNNNNNNNNNNNNNNNNNNNNNNNNNNNNNNNNNNNNNNN
NNNNNNNNNNNNNNNNNNNNNNNNNNNNNNNNNNNNNNNNNNNNNNNNNNNNNNNNNNNNNNNNNNNNNNNNNNNNNNNN
NNNNNNNNNNNNNNNNNNNNNNNNNNNNNNNNNNNNNNNNNNNNNNNNNNNNNNNNNNNNNNNNNNNNNNNNNNNNNNNN
NNNNNNNNNNNNNNNNNNNNNNNNNNNNNNNNNNNNNNNNNNNNNNNNNNNNNNNNNNNNNNNNNNNNNNNNNNNNNNNN
NNNNNNNNNNNNNNNNNNNNNNNNNNNNNNNNNNNNNNNNNNNNNNNNNNNNNNNNNNNNNNNNNNNNNNNNNNNNNNNN
NNNNNNNNNNNNNNNNNNNNNNNNNNNNNNNNNNNNNNNNNNNNNNNNNNNNNNNNNNNNNNNNNNNNNNNNNNNNNNNN
NNNNNNNNNNNNNNNNNNNNNNNNNNNNNNNNNNNNNNNNNNNNNNNNNNNNNNNNNNNNNNNNNNNNNNNNNNNNNNNN
NNNNNNNNNNNNNNNNNNNNNNNNNNNNNNNNNNNNNNNNNNNNNNNNNNNNNNNNNNNNNNNNNNNNNNNNNNNNNNNN
NNNNNNNNNNNNNNNNNNNNNNNNNNNNNNNNNNNNNNNNNNNNNNNNNNNNNNNNNNNNNNNNNNNNNNNNNNNNNNNN
NNNNNNNNNNNNNNNNNNNNNNNNNNNNNNNNNNNNNNNNNNNNNNNNNNNNNNNNNNNNNNNNNNNNNNNNNNNNNNNN
NNNNNNNNNNNNNNNNNNNNNNNNNNNNNNNNNNNNNNNNNNNNNNNNNNNNNNNNNNNNNNNNNNNNNNNNNNNNNNNN
NNNNNNNNNNNNNNNNNNNNNNNNNNNNNNNNNNNNNNNNNNNNNNNNNNNNNNNNNNNNNNNNNNNNNNNNNNNNNNNN
NNNNNNNNNNNNNNNNNNNNNNNNNNNNNNNNNNNNNNNNNNNNNNNNNNNNNNNNNNNNNNNNNNNNNNNNNNNNNNNN
NNNNNNNNNNNNNNNNNNNNNNNNNNNNNNNNNNNNNNNNNNNNNNNNNNNNNNNNNNNNNNNNNNNNNNNNNNNNNNNN
NNNNNNNNNNNNNNNNNNNNNNNNNNNNNNNNNNNNNNNNNNNNNNNNNNNNNNNNNNNNNNNNNNNNNNNNNNNNNNNN
NNNNNNNNNNNNNNNNNNNNNNNNNNNNNNNNNNNNNNNNNNNNNNNNNNNNNNNNNNNNNNNNNNNNNNNNNNNNNNNN
NNNNNNNNNNNNNNNNNNNNNNNNNNNNNNNNNNNNNNNNNNNNNNNNNNNNNNNNNNNNNNNNNNNNNNNNNNNNNNNN
NNNNNNNNNNNNNNNNNNNNNNNNNNNNNNNNNNNNNNNNNNNNNNNNNNNNNNNNNNNNNNNNNNNNNNNNNNNNNNNN
NNNNNNNNNNNNNNNNNNNNNNNNNNNNNNNNNNNNNNNNNNNNNNNNNNNNNNNNNNNNNNNNNNNNNNNNNNNNNNNN
NNNNNNNNNNNNNNNNNNNNNNNNNNNNNNNNNNNNNNNNNNNNNNNNNNNNNNNNNNNNNNNNNNNNNNNNNNNNNNNN
NNNNNNNNNNNNNNNNNNNNNNNNNNNNNNNNNNNNNNNNNNNNNNNNNNNNNNNNNNNNNNNNNNNNNNNNNNNNNNNN
NNNNNNNNNNNNNNNNNNNNNNNNNNNNNNNNNNNNNNNNNNNNNNNNNNNNNNNNNNNNNNNNNNNNNNNNNNNNNNNN
NNNNNNNNNNNNNNNNNNNNNNNNNNNNNNNNNNNNNNNNNNNNNNNNNNNNNNNNNNNNNNNNNNNNNNNNNNNNNNNN
NNNNNNNNNNNNNNNNNNNNNNNNNNNNNNNNNNNNNNNNNNNNNNNNNNNNNNNNNNNNNNNNNNNNNNNNNNNNNNNN
NNNNNNNNNNNNNNNNNNNNNNNNNNNNNNNNNNNNNNNNNNNNNNNNNNNNNNNNNNNNNNNNNNNNNNNNNNNNNNNN
NNNNNNNNNNNNNNNNNNNNNNNNNNNNNNNNNNNNNNNNNNNNNNNNNNNNNNNNNNNNNNNNNNNNNNNNNNNNNNNN
NNNNNNNNNNNNNNNNNNNNNNNNNNNNNNNNNNNNNNNNNNNNNNNNNNNNNNNNNNNNNNNNNNNNNNNNNNNNNNNN
NNNNNNNNNNNNNNNNNNNNNNNNNNNNNNNNNNNNNNNNNNNNNNNNNNNNNNNNNNNNNNNNNNNNNNNNNNNNNNNN
NNNNNNNNNNNNNNNNNNNNNNNNNNNNNNNNNNNNNNNNNNNNNNNNNNNNNNNNNNNNNNNNNNNNNNNNNNNNNNNN
NNNNNNNNNNNNNNNNNNNNNNNNNNNNNNNNNNNNNNNNNNNNNNNNNNNNNNNNNNNNNNNNNNNNNNNNNNNNNNNN
NNNNNNNNNNNNNNNNNNNNNNNNNNNNNNNNNNNNNNNNNNNNNNNNNNNNNNNNNNNNNNNNNNNNNNNNNNNNNNNN
NNNNNNNNNNNNNNNNNNNNNNNNNNNNNNNNNNNNNNNNNNNNNNNNNNNNNNNNNNNNNNNNNNNNNNNNNNNNNNNN
NNNNNNNNNNNNNNNNNNNNNNNNNNNNNNNNNNNNNNNNNNNNNNNNNNNNNNNNNNNNNNNNNNNNNNNNNNNNNNNN
NNNNNNNNNNNNNNNNNNNNNNNNNNNNNNNNNNNNNNNNNNNNNNNNNNNNNNNNNNNNNNNNNNNNNNNNNNNNNNNN
NNNNNNNNNNNNNNNNNNNNNNNNNNNNNNNNNNNNNNNNNNNNNNNNNNNNNNNNNNNNNNNNNNNNNNNNNNNNNNNN
NNNNNNNNNNNNNNNNNNNNNNNNNNNNNNNNNNNNNNNNNNNNNNNNNNNNNNNNNNNNNNNNNNNNNNNNNNNNNNNN
NNNNNNNNNNNNNNNNNNNNNNNNNNNNNNNNNNNNNNNNNNNNNNNNNNNNNNNNNNNNNNNNNNNNNNNNNNNNNNNN
```

NNNNNNNNNNNNNNNNNNNNNNNNNNNNNNNNNNNNNNNNNNNNNNNNNNNNNNNNNNNNNNNNNNNNNNNNNNNNNNNN
NNNNNNNNNNNNNNNNNNNNNNNNNNNNNNNNNNNNNNNNNNNNNNNNNNNNNNNNNNNNNNNNNNNNNNNNNNNNNNNN
NNNNNNNNNNNNNNNNNNNNNNNNNNNNNNNNNNNNNNNNNNNNNNNNNNNNNNNNNNNNNNNNNNNNNNNNNNNNNNNN
NNNNNNNNNNNNNNNNNNNNNNNNNNNNNNNNNNNNNNNNNNNNNNNNNNNNNNNNNNNNNNNNNNNNNNNNNNNNNNNN
NNNNNNNNNNNNNNNNNNNNNNNNNNNNNNNNNNNNNNNNNNNNNNNNNNNNNNNNNNNNNNNNNNNNNNNNNNNNNNNN
NNNNNNNNNNNNNNNNNNNNNNNNNNNNNNNNNNNNNNNNNNNNNNNNNNNNNNNNNNNNNNNNNNNNNNNNNNNNNNNN
NNNNNNNNNNNNNNNNNNNNNNNNNNNNNNNNNNNNNNNNNNNNNNNNNNNNNNNNNNNNNNNNNNNNNNNNNNNNNNNN
NNNNNNNNNNNNNNNNNNNNNNNNNNNNNNNNNNNNNNNNNNNNNNNNNNNNNNNNNNNNNNNNNNNNNNNNNNNNNNNN
NNNNNNNNNNNNNNNNNNNNNNNNNNNNNNNNNNNNNNNNNNNNNNNNNNNNNNNNNNNNNNNNNNNNNNNNNNNNNNNN
NNNNNNNNNNNNNNNNNNNNNNNNNNNNNNNNNNNNNNNNNNNNNNNNNNNNNNNNNNNNNNNNNNNNNNNNNNNNNNNN
NNNNNNNNNNNNNNNNNNNNNNNNNNNNNNNNNNNNNNNNNNNNNNNNNNNNNNNNNNNNNNNNNNNNNNNNNNNNNNNN
NNNNNNNNNNNNNNNNNNNNNNNNNNNNNNNNNNNNNNNNNNNNNNNNNNNNNNNNNNNNNNNNNNNNNNNNNNNNNNNN
NNNNNNNNNNNNNNNNNNNNNNNNNNNNNNNNNNNNNNNNNNNNNNNNNNNNNNNNNNNNNNNNNNNNNNNNNNNNNNNN
NNNNNNNNNNNNNNNNNNNNNNNNNNNNNNNNNNNNNNNNNNNNNNNNNNNNNNNNNNNNNNNNNNNNNNNNNNNNNNNN
NNNNNNNNNNNNNNNNNNNNNNNNNNNNNNNNNNNNNNNNNNNNNNNNNNNNNNNNNNNNNNNNNNNNNNNNNNNNNNNN
NNNNNNNNNNNNNNNNNNNNNNNNNNNNNNNNNNNNNNNNNNNNNNNNNNNNNNNNNNNNNNNNNNNNNNNNNNNNNNNN
NNNNNNNNNNNNNNNNNNNNNNNNNNNNNNNNNNNNNNNNNNNNNNNNNNNNNNNNNNNNNNNNNNNNNNNNNNNNNNNN
NNNNNNNNNNNNNNNNNNNNNNNNNNNNNNNNNNNNNNNNNNNNNNNNNNNNNNNNNNNNNNNNNNNNNNNNNNNNNNNN
NNNNNNNNNNNNNNNNNNNNNNNNNNNNNNNNNNNNNNNNNNNNNNNNNNNNNNNNNNNNNNNNNNNNNNNNNNNNNNNN
NNNNNNNNNNNNNNNNNNNNNNNNNNNNNNNNNNNNNNNNNNNNNNNNNNNNNNNNNNNNNNNNNNNNNNNNNNNNNNNN
NNNNNNNNNNNNNNNNNNNNNNNNNNNNNNNNNNNNNNNNNNNNNNNNNNNNNNNNNNNNNNNNNNNNNNNNNNNNNNNN
NNNNNNNNNNNNNNNNNNNNNNNNNNNNNNNNNNNNNNNNNNNNNNNNNNNNNNNNNNNNNNNNNNNNNNNNNNNNNNNN
NNNNNNNNNNNNNNNNNNNNNNNNNNNNNNNNNNNNNNNNNNNNNNNNNNNNNNNNNNNNNNNNNNNNNNNNNNNNNNNN
NNNNNNNNNNNNNNNNNNNNNNNNNNNNNNNNNNNNNNNNNNNNNNNNNNNNNNNNNNNNNNNNNNNNNNNNNNNNNNNN
NNNNNNNNNNNNNNNNNNNNNNNNNNNNNNNNNNNNNNNNNNNNNNNNNNNNNNNNNNNNNNNNNNNNNNNNNNNNNNNN
NNNNNNNNNNNNNNNNNNNNNNNNNNNNNNNNNNNNNNNNNNNNNNNNNNNNNNNNNNNNNNNNNNNNNNNNNNNNNNNN
NNNNNNNNNNNNNNNNNNNNNNNNNNNNNNNNNNNNNNNNNNNNNNNNNNNNNNNNNNNNNNNNNNNNNNNNNNNNNNNN
NNNNNNNNNNNNNNNNNNNNNNNNNNNNNNNNNNNNNNNNNNNNNNNNNNNNNNNNNNNNNNNNNNNNNNNNNNNNNNNN
NNNNNNNNNNNNNNNNNNNNNNNNNNNNNNNNNNNNNNNNNNNNNNNNNNNNNNNNNNNNNNNNNNNNNNNNNNNNNNNN
NNNNNNNNNNNNNNNNNNNNNNNNNNNNNNNNNNNNNNNNNNNNNNNNNNNNNNNNNNNNNNNNNNNNNNNNNNNNNNNN
NNNNNNNNNNNNNNNNNNNNNNNNNNNNNNNNNNNNNNNNNNNNNNNNNNNNNNNNNNNNNNNNNNNNNNNNNNNNNNNN
NNNNNNNNNNNNNNNNNNNNNNNNNNNNNNNNNNNNNNNNNNNNNNNNNNNNNNNNNNNNNNNNNNNNNNNNNNNNNNNN
NNNNNNNNNNNNNNNNNNNNNNNNNNNNNNNNNNNNNNNNNNNNNNNNNNNNNNNNNNNNNNNNNNNNNNNNNNNNNNNN
NNNNNNNNNNNNNNNNNNNNNNNNNNNNNNNNNNNNNNNNNNNNNNNNNNNNNNNNNNNNNNNNNNNNNNNNNNNNNNNN
NNNNNNNNNNNNNNNNNNNNNNNNNNNNNNNNNNNNNNNNNNNNNNNNNNNNNNNNNNNNNNNNNNNNNNNNNNNNNNNN
NNNNNNNNNNNNNNNNNNNNNNNNNNNNNNNNNNNNNNNNNNNNNNNNNNNNNNNNNNNNNNNNNNNNNNNNNNNNNNNN
NNNNNNNNNNNNNNNNNNNNNNNNNNNNNNNNNNNNNNNNNNNNNNNNNNNNNNNNNNNNNNNNNNNNNNNNNNNNNNNN
NNNNNNNNNNNNNNNNNNNNNNNNNNNNNNNNNNNNNNNNNNNNNNNNNNNNNNNNNNNNNNNNNNNNNNNNNNNNNNNN
NNNNNNNNNNNNNNNNNNNNNNNNNNNNNNNNNNNNNNNNNNNNNNNNNNNNNNNNNNNNNNNNNNNNNNNNNNNNNNNN
NNNNNNNNNNNNNNNNNNNNNNNNNNNNNNNNNNNNNNNNNNNNNNNNNNNNNNNNNNNNNNNNNNNNNNNNNNNNNNNN
NNNNNNNNNNNNNNNNNNNNNNNNNNNNNNNNNNNNNNNNNNNNNNNNNNNNNNNNNNNNNNNNNNNNNNNNNNNNNNNN
NNNNNNNNNNNNNNNNNNNNNNNNNNNNNNNNNNNNNNNNNNNNNNNNNNNNNNNNNNNNNNNNNNNNNNNNNNNNNNNN
NNNNNNNNNNNNNNNNNNNNNNNNNNNNNNNNNNNNNNNNNNNNNNNNNNNNNNNNNNNNNNNNNNNNNNNNNNNNNNNN
NNNNNNNNNNNNNNNNNNNNNNNNNNNNNNNNNNNNNNNNNNNNNNNNNNNNNNNNNNNNNNNNNNNNNNNNNNNNNNNN
NNNNNNNNNNNNNNNNNNNNNNNNNNNNNNNNNNNNNNNNNNNNNNNNNNNNNNNNNNNNNNNNNNNNNNNNNNNNNNNN
NNNNNNNNNNNNNNNNNNNNNNNNNNNNNNNNNNNNNNNNNNNNNNNNNNNNNNNNNNNNNNNNNNNNNNNNNNNNNNNN
NNNNNNNNNNNNNNNNNNNNNNNNNNNNNNNNNNNNNNNNNNNNNNNNNNNNNNNNNNNNNNNNNNNNNNNNNNNNNNNN
NNNNNNNNNNNNNNNNNNNNNNNNNNNNNNNNNNNNNNNNNNNNNNNNNNNNNNNNNNNNNNNNNNNNNNNNNNNNNNNN
NNNNNNNNNNNNNNNNNNNNNNNNNNNNNNNNNNNNNNNNNNNNNNNNNNNNNNNNNNNNNNNNNNNNNNNNNNNNNNNN
NNNNNNNNNNNNNNNNNNNNNNNNNNNNNNNNNNNNNNNNNNNNNNNNNNNNNNNNNNNNNNNNNNNNNNNNNNNNNNNN
NNNNNNNNNNNNNNNNNNNNNNNNNNNNNNNNNNNNNNNNNNNNNNNNNNNNNNNNNNNNNNNNNNNNNNNNNNNNNNNN
NNNNNNNNNNNNNNNNNNNNNNNNNNNNNNNNNNNNNNNNNNNNNNNNNNNNNNNNNNNNNNNNNNNNNNNNNNNNNNNN
NNNNNNNNNNNNNNNNNNNNNNNNNNNNNNNNNNNNNNNNNNNNNNNNNNNNNNNNNNNNNNNNNNNNNNNNNNNNNNNN
NNNNNNNNNNNNNNNNNNNNNNNNNNNNNNNNNNNNNNNNNNNNNNNNNNNNNNNNNNNNNNNNNNNNNNNNNNNNNNNN
NNNNNNNNNNNNNNNNNNNNNNNNNNNNNNNNNNNNNNNNNNNNNNNNNNNNNNNNNNNNNNNNNNNNNNNNNNNNNNNN
NNNNNNNNNNNNNNNNNNNNNNNNNNNNNNNNNNNNNNNNNNNNNNNNNNNNNNNNNNNNNNNNNNNNNNNNNNNNNNNN
NNNNNNNNNNNNNNNNNNNNNNNNNNNNNNNNNNNNNNNNNNNNNNNNNNNNNNNNNNNNNNNNNNNNNNNNNNNNNNNN
NNNNNNNNNNNNNNNNNNNNNNNNNNNNNNNNNNNNNNNNNNNNNNNNNNNNNNNNNNNNNNNNNNNNNNNNNNNNNNNN
NNNNNNNNNNNNNNNNNNNNNNNNNNNNNNNNNNNNNNNNNNNNNNNNNNNNNNNNNNNNNNNNNNNNNNNNNNNNNNNN
NNNNNNNNNNNNNNNNNNNNNNNNNNNNNNNNNNNNNNNNNNNNNNNNNNNNNNNNNNNNNNNNNNNNNNNNNNNNNNNN
NNNNNNNNNNNNNNNNNNNNNNNNNNNNNNNNNNNNNNNNNNNNNNNNNNNNNNNNNNNNNNNNNNNNNNNNNNNNNNNN
NNNNNNNNNNNNNNNNNNNNNNNNNNNNNNNNNNNNNNNNNNNNNNNNNNNNNNNNNNNNNNNNNNNNNNNNNNNNNNNN
NNNNNNNNNNNNNNNNNNNNNNNNNNNNNNNNNNNNNNNNNNNNNNNNNNNNNNNNNNNNNNNNNNNNNNNNNNNNNNNN
NNNNNNNNNNNNNNNNNNNNNNNNNNNNNNNNNNNNNNNNNNNNNNNNNNNNNNNNNNNNNNNNNNNNNNNNNNNNNNNN
NNNNNNNNNNNNNNNNNNNNNNNNNNNNNNNNNNNNNNNNNNNNNNNNNNNNNNNNNNNNNNNNNNNNNNNNNNNNNNNN
NNNNNNNNNNNNNNNNNNNNNNNNNNNNNNNNNNNNNNNNNNNNNNNNNNNNNNNNNNNNNNNNNNNNNNNNNNNNNNNN
NNNNNNNNNNNNNNNNNNNNNNNNNNNNNNNNNNNNNNNNNNNNNNNNNNNNNNNNNNNNNNNNNNNNNNNNNNNNNNNN
NNNNNNNNNNNNNNNNNNNNNNNNNNNNNNNNNNNNNNNNNNNNNNNNNNNNNNNNNNNNNNNNNNNNNNNNNNNNNNNN
NNNNNNNNNNNNNNNNNNNNNNNNNNNNNNNNNNNNNNNNNNNNNNNNNNNNNNNNNNNNNNNNNNNNNNNNNNNNNNNN

NNNNNNNNNNNNNNNNNNNNNNNNNNNNNNNNNNNNNNNNNNNNNNNNNNNNNNNNNNNNNNNNNNNNNNNNNNNNNNNN
NNNNNNNNNNNNNNNNNNNNNNNNNNNNNNNNNNNNNNNNNNNNNNNNNNNNNNNNNNNNNNNNNNNNNNNNNNNNNNNN
NNNNNNNNNNNNNNNNNNNNNNNNNNNNNNNNNNNNNNNNNNNNNNNNNNNNNNNNNNNNNNNNNNNNNNNNNNNNNNNN
NNNNNNNNNNNNNNNNNNNNNNNNNNNNNNNNNNNNNNNNNNNNNNNNNNNNNNNNNNNNNNNNNNNNNNNNNNNNNNNN
NNNNNNNNNNNNNNNNNNNNNNNNNNNNNNNNNNNNNNNNNNNNNNNNNNNNNNNNNNNNNNNNNNNNNNNNNNNNNNNN
NNNNNNNNNNNNNNNNNNNNNNNNNNNNNNNNNNNNNNNNNNNNNNNNNNNNNNNNNNNNNNNNNNNNNNNNNNNNNNNN
NNNNNNNNNNNNNNNNNNNNNNNNNNNNNNNNNNNNNNNNNNNNNNNNNNNNNNNNNNNNNNNNNNNNNNNNNNNNNNNN
NNNNNNNNNNNNNNNNNNNNNNNNNNNNNNNNNNNNNNNNNNNNNNNNNNNNNNNNNNNNNNNNNNNNNNNNNNNNNNNN
NNNNNNNNNNNNNNNNNNNNNNNNNNNNNNNNNNNNNNNNNNNNNNNNNNNNNNNNNNNNNNNNNNNNNNNNNNNNNNNN
NNNNNNNNNNNNNNNNNNNNNNNNNNNNNNNNNNNNNNNNNNNNNNNNNNNNNNNNNNNNNNNNNNNNNNNNNNNNNNNN
NNNNNNNNNNNNNNNNNNNNNNNNNNNNNNNNNNNNNNNNNNNNNNNNNNNNNNNNNNNNNNNNNNNNNNNNNNNNNNNN
NNNNNNNNNNNNNNNNNNNNNNNNNNNNNNNNNNNNNNNNNNNNNNNNNNNNNNNNNNNNNNNNNNNNNNNNNNNNNNNN
NNNNNNNNNNNNNNNNNNNNNNNNNNNNNNNNNNNNNNNNNNNNNNNNNNNNNNNNNNNNNNNNNNNNNNNNNNNNNNNN
NNNNNNNNNNNNNNNNNNNNNNNNNNNNNNNNNNNNNNNNNNNNNNNNNNNNNNNNNNNNNNNNNNNNNNNNNNNNNNNN
NNNNNNNNNNNNNNNNNNNNNNNNNNNNNNNNNNNNNNNNNNNNNNNNNNNNNNNNNNNNNNNNNNNNNNNNNNNNNNNN
NNNNNNNNNNNNNNNNNNNNNNNNNNNNNNNNNNNNNNNNNNNNNNNNNNNNNNNNNNNNNNNNNNNNNNNNNNNNNNNN
NNNNNNNNNNNNNNNNNNNNNNNNNNNNNNNNNNNNNNNNNNNNNNNNNNNNNNNNNNNNNNNNNNNNNNNNNNNNNNNN
NNNNNNNNNNNNNNNNNNNNNNNNNNNNNNNNNNNNNNNNNNNNNNNNNNNNNNNNNNNNNNNNNNNNNNNNNNNNNNNN
NNNNNNNNNNNNNNNNNNNNNNNNNNNNNNNNNNNNNNNNNNNNNNNNNNNNNNNNNNNNNNNNNNNNNNNNNNNNNNNN
NNNNNNNNNNNNNNNNNNNNNNNNNNNNNNNNNNNNNNNNNNNNNNNNNNNNNNNNNNNNNNNNNNNNNNNNNNNNNNNN
NNNNNNNNNNNNNNNNNNNNNNNNNNNNNNNNNNNNNNNNNNNNNNNNNNNNNNNNNNNNNNNNNNNNNNNNNNNNNNNN
NNNNNNNNNNNNNNNNNNNNNNNNNNNNNNNNNNNNNNNNNNNNNNNNNNNNNNNNNNNNNNNNNNNNNNNNNNNNNNNN
NNNNNNNNNNNNNNNNNNNNNNNNNNNNNNNNNNNNNNNNNNNNNNNNNNNNNNNNNNNNNNNNNNNNNNNNNNNNNNNN
NNNNNNNNNNNNNNNNNNNNNNNNNNNNNNNNNNNNNNNNNNNNNNNNNNNNNNNNNNNNNNNNNNNNNNNNNNNNNNNN
NNNNNNNNNNNNNNNNNNNNNNNNNNNNNNNNNNNNNNNNNNNNNNNNNNNNNNNNNNNNNNNNNNNNNNNNNNNNNNNN
NNNNNNNNNNNNNNNNNNNNNNNNNNNNNNNNNNNNNNNNNNNNNNNNNNNNNNNNNNNNNNNNNNNNNNNNNNNNNNNN
NNNNNNNNNNNNNNNNNNNNNNNNNNNNNNNNNNNNNNNNNNNNNNNNNNNNNNNNNNNNNNNNNNNNNNNNNNNNNNNN
NNNNNNNNNNNNNNNNNNNNNNNNNNNNNNNNNNNNNNNNNNNNNNNNNNNNNNNNNNNNNNNNNNNNNNNNNNNNNNNN
NNNNNNNNNNNNNNNNNNNNNNNNNNNNNNNNNNNNNNNNNNNNNNNNNNNNNNNNNNNNNNNNNNNNNNNNNNNNNNNN
NNNNNNNNNNNNNNNNNNNNNNNNNNNNNNNNNNNNNNNNNNNNNNNNNNNNNNNNNNNNNNNNNNNNNNNNNNNNNNNN
NNNNNNNNNNNNNNNNNNNNNNNNNNNNNNNNNNNNNNNNNNNNNNNNNNNNNNNNNNNNNNNNNNNNNNNNNNNNNNNN
NNNNNNNNNNNNNNNNNNNNNNNNNNNNNNNNNNNNNNNNNNNNNNNNNNNNNNNNNNNNNNNNNNNNNNNNNNNNNNNN
NNNNNNNNNNNNNNNNNNNNNNNNNNNNNNNNNNNNNNNNNNNNNNNNNNNNNNNNNNNNNNNNNNNNNNNNNNNNNNNN
NNNNNNNNNNNNNNNNNNNNNNNNNNNNNNNNNNNNNNNNNNNNNNNNNNNNNNNNNNNNNNNNNNNNNNNAGAAGCATT
GTATGTATGTAGGCCTACATTCAGGCACACAAAGATATTGAGCATGTATACCTTACATGCATAGATACATCTATCATATA
ACCATACATACAGAACTCATACACTTAAATGTTTTTTCAGGTCCCATTCAGTATCAATTGAAACCATATCAACATACATT
ATACTGAGACTTCTGCATGTATGACCATGGATACTGTAAATCCCTAAATACCATAGATACAGCATCTACAGTACTGTAAA
AAAATATATTTGACTTACACATGGCTGTATAGTGTTTGTGATTTGAACAAAAATGTTAATCATGCAGATAGATGGATATT
GGTGCAGTTTATCTAAAGTGTTAAGATTTATAATTTTGCTGTGTGTCGTTGGTTTAACTTTTCATCTGCAGTAAATCATA
GGAGACAGAATGGTAAATGGTGTCGTTGTTTGCTAAAGTTAGAGCTTTGAGGGAGTCATTTTTTCAGATGCGATGTGGAA
CAAGAATTGTTCACTATCATAAGATTGCCTCTCTTTTGGCATTGTAGCAAGCATAATATTGGTAGTCTGTATAGATGTAT
AATATTACATCAGTATTACTTTGAAAGTTTTCATAGACATATAACTTGTCACTGTGCAATAAAGCCATTTGGATTGATAT
GGCAAAAAGCAGCACATAGTGAGGGTTTACTGTTGATGTACATATAATTGACTACATATCATGTGACCTTTGACTCACCC
GATCGCCTGCCTTATTTCTCTGTAACAGCCAATTCAGACATCATGACCTGTCCCTGACCTCATATTGACAAATCCCCACA
TTGTTTTGACTGTGTGTAAGACTTGACCTGACAAGCAAACTAGTTGAACTTGAAGTAAATAGATATACAGGTTTAGTTTT
GGTACATATCACCCATGTGAATCGCTGTCTGCCTTTTTTAGAATTCTACTGTATACTCATGTTACATTTCATTGTTTCTT
AGATCAGTGTCAATGTTTCTGTCAGTGCATACGCTTTTCCCTTTCTTATTTTTGTCAAATAAATCCCTGTACATTATTTA
TGTAATAAAACATCTAACACCAATGTCTGAAACTTCACCTGTGGATTTTTATTATTTATTCATGTTAAGCATCAATTTAG
TTGTAGTATGTTAAGCATAAACATGTTCAGAGACTGCTTTCTCTGCGGAAATGCTACAGCTTGATATGGTGGCAAATGAC
AATATGTTATCCTCTATCCAAACCAACCACACTTTTTGAAATAGTTTGACAAAAATTTGTGCTATGATTAAAATGTATGT
CTGTTTTTAGATTGTCTTATTGGCTCAGTTATTGCACCAATCACATTGGCTGTCTGGTCCACACTTTCCCTGTTGCCGCT
GCACAATGTCCTATAGAGTTCCTTCAGAAAGTATTCATACCCCTTGACTTATTCCACATTTTGTTGTGTTACATCCTGAA
TTCAAAATAGATTCAATATTTTTTTATCTCACCCATCTACACACAATACCACATAATGACAAAGTGAAAACATGTTTTAG
CTACTTTATTGAAATACAGAAATATGTAATTTACATAAGTATTCACAACCCTGAGTCAATATGCTGTATGTTAGACTCAC
CTTTGGCATTGATTACAGCTGTGAGTCTTTCTGTCTCTAAGAGCTTTGCACACCTGGATTGTACAACATTTGCACATTAT
TATAACAATTTTTCAAGCTCTGTCAAGCTGGTTGTTGATTATTGCTAGACAGCCGTTTTCAAGTCTTGCCATACATTTCC
ATGCCGATTTAAGTCAAAAATGTAACTAGGCCACTCAGGAACATTCAATGTTGTATTGGTAAGCAACTCCCGTGTATATT
TTACCTTGTGTTTTAGGTTATTGTCCTGCTGAAAGGTGAATTTGTCTCCAATGTCTGTCTGGAAAGCAGACTTAACCAGG
TTTTTCTCTAGGGTTTTGCCTGTGCTTAGCTCTATTCAATTTCTTTTATCCTAAAAAAACTCCCTAGTCCTTGCCGATGA
CAAGCATGCCCATAACATGATGCAGCCACCACTATGCTTGCAAATATGAAGAGTGGTACTCAGTGATGTGTTGGATTTGC
CCCAAACATAACGCTTTGTATTCAGGACTTAAAGTAAAAAAATGTAACATTTTTTGCAGTTTTATTGTCTGTTGTTGCTC
CAGTCTCAGTTTTCACTTATCACAACCATTAAACGCTGTAACTGTTTTAAAGTCACCTTTGGTCTCATTGTGAAATCCCT
GAGCGGTTTCCTTCCTCTCCGGCAAATGAGTTAGGAAGGATGCCTGTATCTTTGTAGTGACTGGGTGAATTGATACACCG
TCCAAAGTGTAATAATATGCTCAAAGAGATATTCAATGTCTGCTTTTTATTTTTTTTATCTACCAATAGGTGCCCCTCTT
TGCCAGGCATTGAAAAACCTGAATCTGTGTTTGAAATTCACTGCTCGACTGAGGGACCTTACAGATAATTGTATGTGTGG
GTACAGAGATGAGGTAGTCATTCAAAAATCTTGTTAAACAATATTATTGCACACAGTGAGTCCATGTAACTTATTATGTG
ACTTCTTAAGCACATTTTTACTCCTGAATTTATTTAGGCTTGCCATACGAAGGGGTTGAATACTTATTGACTCGAGACAT
TTCAGATTTTCATTTTTAATTCATTTGTAAACATTTCTAAAAACATCATTCCACTTTGACATTGTGGGGTATTGTGTGTA
AGCCAGTGACACATCTCAATGTAATCCATTTTAAATGTGGAAAAAGTCAAGGGTGTGAATACTTTTTGAAGGCACTGTAT

ATAATGTGTCATTCATTTAGTGTAATTAGTCTTCTCTCAGCATTCTGTTAATCTCAGGAGTTTAACGCTCTGAAGTTCAT
CCACTTTGTCAGCAATTTCACAGCTTTTCTGTGTGACTTCAAATTAAAACAACCATGTCGTTCAGGCGGAAAGTAGATGT
GATAAGATTGAAAGATAATGAAATATATAATGAGCAAAACAGAAGAATTTCATTACCTCAGAAATATCGACCAGGTAGGA
GTTAGGATGTCATATGTACCCTTGTTCATTGAATCTCCATCACCAAACAGCTGATCGCCATCTTGGATTTGACTTCATGA
TTGCAGTGACTTTTAATTGATCTTTCACCTTTTGTGTCTGTTGAAGAAAATATCAGAACCATTACCTTGAATGATGCAGA
TCTTGAAATTCCAAAGGCAAGTAATTAATGATCCATGTTCCCCTNNNNNNNNNNNNNNNNNNNNNNNNNNNNNNNNNNNN
NNNNNNNNNNNNNNNNNNNNNNNNNNNNNNNNNNNNNNNNNNNNNNNNNNNNNNNNNNNNNNNNNNNNNNNNNNNNNNNN
NNNNNNNNNNNNNNNNNNNNNNNNNNNNNNNNNNNNNNNNNNNNNNNNNNNNNNNNNNNNNNNNNNNNNNNNNNNNNNNN
NNNNNNNNNNNNNNNNNNNNNNNNNNNNNNNNNNNNNNNNNNNNNNNNNNNNNNNNNNNNNNNNNNNNNNNNNNNNNNNN
NAATAAATGCTGGAGCTGAAATATCAACCCAGATTCTGCGGTAGACATAACAGAGTAAACAAAAATCTGGGACACTCAAA
TTAGTATGATCGTTACGTATGGTATGTATTAATATGTGGATGTCCATCGTCCATTTCGTATGATATATTACAATTCATAA
AATATGCTACAAATTTGCAATACGTATGATATGTTATTAAGAATTCCAATTTGTTGTGGCTAATGTTAAGTAGGTGGCTA
GGTGGTTAAAGCTAACGTTAGCTAGGTGGCTATGTTAGCTAGGCTGGGGATTAGGGGTTAAGGTTAAGGTCAGGAGTTAG
GTGAAAGGGCTAGGGGAAGGGTTAGCTACCATGCTAAGTAGTTGCAAAGTAGCTATACGCTCAACCATCCACCCCGACCG
ACTACAATCCTTTAATTTTTGCGTTAAGTAACCTTCGGCCTGATGTAACCATACCAAACATAACATATCATACTCATTTG
AGTGTACAGGATTTTAGTTTACTTTTACATCTAGTCTGAGGCCAGGCTGGACGGTGCCTTTGGAAAGTATTCAGACCCCT
TGACCTTTTCCACATTTTGCTACATTACAGCCTTAATTGAATTTAAAACATTTTTTTAATCCTCAGCAATCTGCACACAA
TACCCCATAATGACAAAGCCAAAACAGGTTTTTAGAAATTTGTGCAAATGTATAAAACATTAAAAACATTAACTTATTTA
CATAAGTATTCAGACCTTTTGCTATGAGACTCGAAATTGAGCTCAGGTGCATCCTGTTTCCATTGATCATCCTTGAGATG
TTTCTACAACTTGATTGGAGTCCACCTTGATTGGAGTCCACCTGTGGTAAATTCTATTGATTGGACATCATTTGAAAAGG
CACACACCTGTCTGTCGGTCCCACAGTTGACAGTACATGTCAGAGAAAAAACCAAGCCTTGAGGTCGAAGGAATTGTCCG
TAGAGCTCCGAGACAGGATTGTGTCGAGGCACAGATCTGGGGAAGAGTACCAAAACATTTCTGCAGCATTGAAGGTCTAC
AAGAACACAGTGGCCTCCATTCTTAAATGGAATAAGTTTGGACCCATCAAGACTCTTCCTAGAGCTGGCCGCCCGGCCAA
ACTGAGCAATCGGGGGAGAAGGGCCTTGGTCAGGGAGGTGACCAAGAAACCGATGGCCACTCTGACAGAGCTCTAGAGTT
CCTCTGTGGAGATGGGAAAACCTTCCAGAAGGACAACCATCTCTGCAGCATTCCACCAATCAGACCTTCATGGTAGAGTG
GCCAGACGGAAGCCCCTTCTCAGTAAAAGGCACATGACAGCCTGCTTGGAGTTTGCCAAAAGGCACCAAAAGGACTCTCA
GAACATGAGAAACAAGATTGTCTGGTCTGATGAAACCAAGATTGAACTCTTTGGCTTGAATGCCAAGCGTCACATCTGAA
GGAAACCTGGCACCATCCCTATGGTGTAGCATGGTGGTGGCAGCATCATGCTCTGGCGATGTGTTTCAGCGGCAGGGATT
GGGAGACTAGTCAAGATCGAGGCAAAGATGAACGGAGCAAAGTACAGAGAGATCCTTGATGAAAACCTGCTCCAGAGCGT
TCAGGACCTTAGACTGGGACAAAAGTTCACCTTCCAACAGGACAACGACCCTAAGCACACAGCCAAGACAACGCGGGAAT
GGCTTCGGGACAAGTCTCTGAATGTCCTTGAGTGGCCCAGCCAGAACCCGGACGTGAACCCGATCGAACCTCTCTGGAGA
GACCTGAAAATAGCTGTACAGCAACGCTCCCCATCCAACCTGCCAGAGCTTGAGATGATCTGCAGAGAAGAATGGGAGAA
ACTCCCTAAATACAGGTGTGCCAAGCTTGTAGCATCATACCCAAGAAGACTTGAGGCTGTAATCTGAATACTTATAAAAA
TGTGATATTCCATTTATTTTATTTTTTTAGTAAAATAAAAACCTGTTTTTGCTTTGTCATTATGGGGTATTGAGTGTAGA
TTGATGGGGGGGAAAAACGATTTAATCAATTTTAGAATAAGTCTGCAACATAACAAAATCTAGAAAAGGTCAAGTGGTTT
GAATACTTTCCAAAGGCACTGTACAGTGTAGCACTAGATATTGGACATTTGGATTTATGTATTGTACAGATTTAAAATAC
ATATAGTGTATACATTAATGTATGTGACTCTTACACCACTCAGACAAATATGCCAACATTATTTGCACAAATTCAGTCAT
ATACGGAGACTTCCTAAACGTGTACCTCTTTGCATTAGGTAAATTGCTTACCCTCTGAATTCTCTGAAAACTCCAGCATC
AATGGTAGAATTCCTGACCAAAATGCATCAAAATGTTCAGCTGTTTAATTGCTCCATTTCAATGCTCCACTATTATCAAA
TGTTGGCATTTGCAAAACCTTGCAACTCCTTCATCCTTCTTCACATCCTTCATCCTTGTTCATCCCTCCACTACTTTTTT
ATTTCAATTTCCCTGTGTTGTTTTGGGATGATAAGCAAACTGTATGATCAGAATATTGATGGTAGTAATATATACTTAGG
TCTAGATTCAATCAGATTGACAGCCGAATAGCCGTTGTTTAGTGGTGTCGGAGGTGGAACTGCGTTAGAGCTGTCAAATC
CACAAGTGGCAACGAAACCACACTCAACGTAATCTGACAAATTCCATGCAGCCGCATTAGAGGTTTATGCAGCCGCGTTA
CCAATCAAACACTCAAATTAGTTTGATAGGCTTTACATCTCTATACATTAGCCTAACATCAATGGATTGACATTTGAGAG
TCATTATTTCTAACATCGATAGAGCCTACAATTTGTAGCGCCGTTTTGAACTTCTAACGCGGTAGGGATAACAAGTAAGG
GAGTGGTTTCAAAAGTACCCGCTCACCGATAGGTCAGCTCGCTGTAGATAAATAAAACATTTGCTATGCAGATGTCGGCC
AACGCTGGTTAACGATGGTTAATGCTCGATCTGATTGAATCCAGGCCTTAGTTAGTTCTAGTTATAACAAAAAGCTTGAC
AATTCAGATACTCTATTAGGTACACGTACAAACACTGATGGCTCTGCTGCAGTAATACATATAAGTAGGCTCTATTCCAA
GAACCAATATCAACAAGGTAACTGACTACAACCATATACAACAATACCCCTGCTCAAATCAACACAATCTCACCTACACA
CACTGCACGTGTCTCACAGAGGAACACGTTACAGTGGGTCATCAACATATCCATTCAATTCGACTCATCTCCCCTCCCAC
CACTCCCCAAAATAAATGACGGGAATTATGGGAAATCTCCGTATCTGACACAATCCTTTCCTGAAAATAAACAACTGGTG
TGCGTATATGTCAATGTGTGTGTTTCTTTGAGAGTGTGTTTATATATTCCTGTATGTATGTGTCCTGTATGTATATGGCA
CCTGAGCTCATAAGTTGATCCAATGTTCATGTATTGTCATTGTGAGTTTGATCAAGTGTGTCACCAGTACTGTATTGTTG
TTCTTTTTGCTCTGGGTCATACTTGGAGAGTTGTCTAGTGTTCTTTATATGTATGTCAGTCATCTACTGCTGTTATGGAA
TATTTGGTTGGTTTGTGCGTCTAATTTCCACAATGATATGCCTAGTTTGAAACAGCAATTTATTATTAATTTCCTATTCA
TGTTGCTGGGTAAGAACATTTGAGAATTGCGTACCAAAGCATATTAACAATGCACTTAACATTGCCCAATTCATCCACTA
TGTGTTGTGTTTTTGTCTACAATTTCCTAACTTTTATTAGTTGCAGCTTTTGTACAAACATAAACACCAAGTTGTTCAAG
TAAGCAAACTCTGCACATATGGATCTTTAACAGTGAGTGATGCACGTTTGTTAAGTTGACTGGATCGCTGCTTCTTTTAT
TAGCATAGGTTGGCTACCTAGCAGCTAGCTAACTTTCTTATCATAACATATTTGCACAATTTCCCAGTTGTATTGGCAGG
TCTGGCTAAACTTGGATAAATAATGTGAAAGGGTTTGTAGTAATTTTTGTAGAACTGGTTTGAAAGTAATCCAGCATTGG
TAGAGAAATACCACTTGTTCCCCTTGTTTCCATAACACATTTCCTGTTACTGTTGCTAGCACCAAGTAGGGAATGGGATT
AGCATGTATGCTAACGGTAATATCTGTTGATATTGGTTATCGCCGTGTGCTTTACAAGGTTCTGAGTGAGAAGAGAAAAG
GTCTGCCAGTTCAATTACACTTTTTTTGTTCTCTTTCTAGAGATGGAAACACCTGCTATATACAATGGACACACCCACCC
TGATATTTAATGCTAATTCGTTTTGAAAACAAATGGCCTGTTTCCGACTAATTTCTGTACTGGATGCAGATCATAAAACA
GTCTTGAAATACCTTTTTTCTGACTTTCACTTCTCCAGTGCTTCGTTCACATCTTTTAAGACTTCATAATCTTTGGTACA
ATCATATGAATGGTCTTTAATCCTTAATGTTCAATGAAGTTATTACACATTTGAGAAAATGTCTTGGCGTCTCAGTAGAG
TAAATGTCCATTCAGATTCTATTCGTGTAGAAATCTTTTAAATGATTCCACATTGCTGTTTTTACTTGGATACATTAGAC
ACCACAACCTCCATCAGTGCCAATAACCCAGCTATTTTCTTTCCTACTGCTCCAACACAGACTGCATTGAACAGGACTGG
ACATGATGACTTTCATTCAGAGCTTGATTTAATTAAGAACTGAAATCAATCTGGTATTTGCAATGACGTTATTCTGCGCC

AATGTAGGTAAGGATAGATAGCAAAACCAAACTTTGAGTTATTCGCTTATTACAAATTCCAACCCAGTGCTTTACAGATT
TGGTAAATTAATGAGCATCTATTGATAATTGGTCAATGGGTATGGGTTAGAAATTCTGATTGGTGGGTTAGACACAGTTG
AGATTTTATTCAAGAGCTCTTGAATACTAGCTTCTTATTCAAGCTTTGTAGATCAGTATTTAGTCGTTATGTTCCTTAAT
TCGCATCTAAAGTTCTAAGAATTACAGTAACCCATCTGCATGTTAAACTATTATAATAATGCAATACTATAATATTATCA
TCTATAATGTTTTATTTTAAAGTACTTATTACTATTTAAATGTTAAAGTACTTATAAGGTTCAATGACGTGTGGGAATCA
ACCACACACTTGACAAGGGAACTAAGGTGCATGAAAAAACAATATTGAATTTCTTCACATTTATTGTGGTCTTAGCTAAG
CTTTCGGTGAACGCTTTTTATTCTTTATTTATTTTCTTCTTTCTGTGAAAGTTTTGCTCTGACGAAGGTCAATTGACTGA
AAGCTTAGCAAAAACCATGCTAAACCTGAAATGTGCATGGATTTTAATGTGAGTAAGATTAATTAAGGTGACACAATTGT
ATCAAGTTATAGAGTCATAACTATTTGTGTCACCATGGTTTTCAAGCCTTTACTACAGTATATTTAAGCAATAAGGCACG
AGGGGGTATGGTATAAGGTCAATATACCACGGCTAAGGGCTTTTCTTAGCATGACCCAATGCAGAGTGCCTGGATACAGC
CCTTAGCCGTGGTATATTGGCCATACACCACAAACCCTCGAGGTGCCTTATTGCTATTATAAACTGGTTACCAACGTAAT
TGTATACGGTCTGATATGCCACGATTGTCAGCCAATCAGCATTCAGGGCTCGAACCACCCAGTTTATAATACAAATTAGA
ATGTATAGAAATTCCTTGAAATGAATCTCATTGGCTAAGATTGACACAACTCTCATTCTACTTGACTGCTCGGACTCCCT
TTGACACCCGTCCCTGACTAGTATCCCCTTCTAACTCAGCATCATACGAAGATCAAGACACTGACATGTTGGTCAACCCA
AGATTGTAATTCCTTCCACCTTTTCTTTTTCTGACTGTATCCCCTCTCCTATGCTGTCCATCTCTATTGGTGTACCTCAT
ACTTCTGCTCTGTAACTCATTTTCCTCTATTTCTCTTTCTCCGTTGTTCCTCCCATCCCTTGTCACTTATTTCTCTCCCT
CCCTTACTCCCTTTGCTTTCTCACACGATCCTTTCTTTCTTTACCTCCTGCTTTGATCCTTTCCTATCGGGACCCTCATC
ACCCCACACTATTTCCTCCATTCTTTTCTTTCTACATTCATTACTGATTCACCTATTTACTCTCTCCTCTACCCACCTGT
TTGTTCTTCACTGGCCTCCTCTTTTTATTTGATTATTCCTTGCTTGTTTATATCTCCCTCTCCTTCCATCCTCCCTGTTA
CCTTCTCTCTACACATTTCCTGCTTCATATATGACTCTTTATATTGCCTCACTCTCTCTTTCCTGGATTGGCTGACTCCT
TTACACCCTTGTCCATGCCTCAAATCCCCCACTGAATGCCCCTTCTCCCACTCCCATCTCTCCCCTCTCCTACTCCTCTT
CTCCCCTCCCTCTCTCTGTCTTGCTAGGGGGCCCAGCATTCCTCCCTGGGTTCAGCAGACCCCTGTGTGAATACTGAAGG
TGGAAGAGGAGGAGGAGGAGGAGAAAAAGAAGGATGGATGACCACTGATCCTGACCCTACTACCTCTTCCTCAGGCATCTCCT
CCACCGGCTTGCCCCGGAATGAGAATGGAGTCCCCTCCCCACCCGTAGACCCTCCTCCTCCCCCCACTCCTCATCTCCAC
CCCAGTCCACCCCAGAATGGAGAGCTCCGTGGCAACCCTACATCCTCCCCAGTGTCAAGCACGGAGGCAGCTGAGAGCAC
TGCAACAGCAGGACTGGGCATAGAGGAGAGGGAGCAGGTCAAAAACAACACAAAACCAAATCAGAGAGAGAAGACACAAA
TAAAAAAGTCTTGCCACATTTCCTTCCTTTTTTGAGGAGGATGAGATGACTGCAATCTTTCCCGTTTCTTTTATGTGTAC
CCAAACCAGAGAGAGAAGAAATGTGTGGGGGGCAGCAGTCATTGTACATTTAACTGTACAGTACATTCAGCATTATCACA
CAAGACAACAAAAGGGCTTTCCAATGGACCTCATTTCCGTCATGCTCAAACAGGGCTTTGGTCAGTATCTGTCTTGCAGG
ACAAGATAATGATACCTTTTAGTTATCGTTGACATTGTGGGATCCTCATGTACTGTTTCAATCGATCAAACTATAGAGCC
TATCAAACACTGGTATAGGCCTAATCACCAAACCAAGCAACTACGTGAGATTTTGCTTTGTGTGTTTGTTTAATCAAATA
TAGACCGTAAACAAACACTATATAATGTCTCACTCCCCAAAGACTTCTCAGTAGCCAATAGCATCCTACTATATACATGC
GTGTAGTATCCCATAATTCCATCGATAAAAGGTCTTGTCATTTCCTGTCCTGAAAACTCCAGCTCTCAAATTTCATTGGT
AGAGATGTGGTCATCTGGCTTTATAGTTTGGTTTCCTTAGTGCCTTTGAGTGTATTTTAACAGGGTTTTCAAATGACAGG
CGGAGATGATATTGACAGAAAGATTAGATTATTTGATGATAGTACATAAACAGTCTAATCAAGATCCCAAAATATCTATG
CTTTTTGAAATGCAGTCTAACTATCGAGTGCAGAACAAGCAGAATACAGTTTCATTTTTGGGATTTCACAGACATGGCCT
ATGGCCTAAGGAAAGTGTGTGATGTTTTCAAAAATGTGTTAATTAAGGGACTTCCCATTCGTGGACTGGTGCAATGTTTT
CTCTTCTCAATCTCAGAACCTATGTGTGATCAACTGGTGCTACAGACACAAACAGTGCCATTTCCAAATCAAGTGTGTGT
GTCATAGGACTGCCTCTCATGTTTCCCACTGTCTGTATGTCTGTCTCTCTCACTCTGTCTGTTATTGTCAAGGTAGCCTA
TTTCTTTTATGTATTTGTCTGTCTGTCTGTTATGTGTGTGTCGTGTCCCTATGTGTGCAATTGTTTAATGGAGTGGGAGC
TGGGTCAAATAGGGTGCTTAAACCAAATGAGACAATAGCAAAGTTGTCAGGTAAAAAGTTTACCAGCAATTTGAAGTGGT
TGAAAATATAAAAGGAATGCTATTTTTGTACCTTCAGTGTCCAGGCATATAACGTAGTCACTTGTAACACCTAGTACACT
TCCAGAATTGTTGTCATGGCTTTTTCTGTTTAGTACCACAAACTATTTATTTGATTCTTATTTTGGGGTAGCCAGTTAAG
TCGAGCCTTAAGTGCCTGGGTCTCAGATAATGACATTTTGGCCAGTAGTCAAACAGTGCTCAGCCAAATTTGTATGTGGC
ATAGACTTTCTCCGTGTCGTTTCGAGGGACTATTTTAAGTTAACGTACCATTCAGCTGAGCAATTTTGAGAAAACAAAGT
TTGTTTGGGGCATTGTTAGTAAAGAGTATTTCAAATCTAAAATATGTTACACAGTGGACTTAAAGGAATTGATTGCTTTG
TGTCAACTGTTCAGATATCACCTGTCAGTGAATACTGTTATACTGTGTTGTCATGTCCTCTTGTTTTGTGTATAACTTGT
CCATTGTAATCTTTGTTTAGATGTGAATTTGGGTTTATTTTGGCCCTTTCAGATTCACACTGGTGGTGCTTTATAACTAG
TTGGATAACTTTCCTTAGCTGGCTTAGCGTCTCAAGAGCATTAACATGCTTCAGACAGTCCTGGTTTTGCAGAACCCAAC
ATCAGTCTTATTTAACTCTTTTATTTGATTAAGAGTCAATTTACTAAGGGCCAAATCCTAACTTGATTTATATTTTTGCA
GTTCTCCCATTGGCATCATGCTTTACGAGTAAGTCTGAGTTTAACATGCAGATCTCAAGTTAGGATTCGGCACTTAGTGT
GTAGGGTCAAAATGCCTCCGCCAATGCTGTGTCGATCAGTACTAGGGGATTAGCCATAACGTAGAGTGGGTGAGATTCAG
AGATATGAGAGAGATTTAGCCATGAGTGTTGAATTAAAAGTGAACCAAATGTGATTATTAATAGGGAGAGTTTGATGTAG
TTAGCTTAAATCAGCTAATAGGGCTACCTTTCGAGTGCCAAAACCTTCATATCAGTAATAAGATGGCTTTTAAAGCAGCT
AGGGCTTTACGTCTGCATTGAGTAAAGATATGGGACTCTACAGGGTCACTGGTTGATATATGAGCATTGCCATTAATATA
CCTCAGAGACGCAGAATTGAGAGACTCAGGTAAACAAATAATTGCCTCAGAGCTGAACGCAAACTGACAGAGATTAATCC
AACTTTGGAGTGACTGCCATCAGATACAGTGGTTGGTCCATCTATTACAAGATCTATCCCACTGGGAAAAAACTGGTTGA
ATCAACAGTGATTCCACATCATTTAAACAAAAAGGCTAAATGTGATGTAGTTATGAATTTCATTAGGGACGTAAGGGAAT
GTAGTATTTTTTTTCACCTTACTTTTAACCTAAATCCAATGACATGGATTAAATTCACTTTAGTTGACAACTCAACCAAA
TGTAAATCAAAATTAGACTTTGAAATGACGTCTGTGCCCAGTGGGATGGCTATGATTGGATAGCAACTCATCAAGCACCT
CCCCGTTCCTTCTCTCTCTGTCTTCCTTTTCTCCTCTTAGCCAATCACGCAGCTTCGTGGAGGACAATCTGGAACCTAAT
TGGCTGAGGCAACATGGGCAGCCCGCATGGCCTGGTCATATATCAAGAGCGCTCTCTCGTGATGCGATTGGCTGACAAAG
GGAGAAGTTGGGCCATTGGTCCAATTCTCCTGACCTAAAGGATAGTCCAGAGCCACAGCTGACCATGTAAGGATCTGCTG
TGTTATTTACACTACTTTACTTGGGCCCTCTCTCTCTGACTGGATGGCTGGACAATCTGTATTTGATGCTTGTGATTAGA
TATTTGATTTTCTTTTAAAGACGTGCCAATCTCGGGAAATGGTTTTGTTGGCTCATCAAGCATGTGAAGAACAGCTATGC
AGATTGACATGATATTAACCTTTTGGGATTGAGCAGTAAATTAAATGAGGGAGAGCTACTGTGTGTAAGATGCATTTCAA
AGGTGCTGTAATTTTTCTTTCCTTACATTTTTCTAAGATCTCACAATTTGTTTCTTGCATTTTTCCTTCCTAACATTTTC
TACAGTCCACCCACGATCTACCCATTTCTCTTTCTATCTATTCTTAACCATTTTCAACCACCCACTTTATTTTCACCACT
TTTAGACTTTTCTGCTCCTGTTCATATCTCCCATGGTTTCGGTTTTGTTCTTTGTTCTTTATCTCTATTCGTTTTACATT

TAAAGGAAACTAATTATGCACAGCCTATTACAAGTGAAACTGCTACAAAACAAAAAATATTGCTTTGAAAAATGTAGACA
ATGCTATGATTTATATTTCTATAATAATGCTTATTCTTACCGTAGTACTACAAAAACTATTAATTGGAAATCTTCCTGCG
CAAATGTTTATTTCTTGATAATGACAAGACTCTAGTATCTAAAGACAGTCCAGAAACGATCAATTTTATATCAACTGATT
TTTCTTAAGACAATCTTATGGAACATTTCCCTTTTGTGGAGAAAGATTGCCCTGGGATTGTGACGGATAAAAACACAAAC
TGTTGTTTTACCTGCTCCCCTCACAATGATTTGCACGTTGCAGTGACCTGTACCCCTCCACTTCTCTCTTGTAATCCCTG
GAAAAGAGATATGAGGATGGATGTTGTTTCTCTACTTGACAATCGCTGGGGAGCTCTCCATTTTACAGACGATCTAAAAC
TCCTTAGAAAAGGACATCCTTACCATCACGTTTTTGTTTTCCTATTTCAGTCTCTGATGTAAGCTTAGTGGACCAGATGC
GAGTTTATCATGTTTTTCCAGTGTGGTGAAACCGAATTCTGGAGCCAAGTTTCTGTATAACTTCCATTTGATTTATTTGT
ATGGACTCCACTGACACAGAAGCTAAATAAAACCCTGTGCCCTCCTTCACCTCCGACCACTGACAAATGACACATCAAAC
GAGCGAGAGATTAGAGGTTAATACCTTTTAAGTGATTGAATGTGTGTGTATATACAGTGTGTGTACAGTTGTTGTGTATA
TACTGGATATGTGTGTGTATATATAGTATGTCTATGTGTATATACAGTTTGTTTGTATATGCAGGGTGTACAGTTTTTTC
CTCAATCCTCTAATCTCTCTCTCTCTCTCTCGTAAATGACGTATTTGACTCCTCAAAGAGAAATGTTGGAGTGGAGAGGT
TCACACTGGCAATTTAAAGGACAGCTGTCTGGGATGCACTTTCTGTGCAGACACTGACCAATCTGACACCTACACACCTA
ATTATGCCTCTCAAAACAATCTCTGGCTTGCACTCATTTTCTCATCAACTGAGGCATTTTTAAAAATTATATTTGGGTGA
CCAAGCAATGTTTGTACATATACAGTATGTAGAATAACTAAGATAATATTTCCAGTGACCTCCATCTCCTTTTTTTTTTA
ATTCCTGTAACTATTATGATTCTTCCTTGTTATTTGATTTAAGTTTTACGTTTTCTCTACATTAAATGTTTGCAATGGTT
GTCATTCAGATGTATATAATTATCCAGACAACCCTTATCTAATCTTTCCAATTTAGTTCCCCGAACTCTGCCTCTCATAG
ACCCCTCTGTCAGGCTGAACATTTGGTTCAGATCACACTGTTACTTGTGAAGCAACTCTTCCATGCAAGTAACACCATGT
TATTAGCCATTTCTTCAAGTTCTATTAAGTGTGCCTCCAACTCGCCAACCCTCCCTAACCCTTGAACTTTCAACCCCACA
GGTCACTCATAGTCATACCCCTTGACAAGCCATAGTCCCCCAACCCCTCTATTTTTGTTTTAAAAAGGACAGACATTCGG
GGACTTGTGTATATTAATGGAAAGAGTGATATTGAAGAGTGTATTGCCATTGTACATGGATTTGTTATGAATGTGGATAT
TCTGATTATTTAACTATTATTGTAATAATGATTATGATTATTGCCACTTTGATTTTGTTGATTCATTCCTTTGATGCGGT
CAATAACAATTCCTTCATTTCATTTTTGAATAAGCCGGAATTTATATTTTTCGGGTGGGTGAATCTTGGAGATAGAAATA
CTTAAATAAATGAATAATAAACAAATAATTAACTTCTTAAATTAAGATGTGATATGTATTTTTTTTTATCATTCCTACAG
AATAGTCACCACAGTGAAAAATATCTTAACTCTTACTAGGCCTGTTGGCCATTTAATGGTTTATGAGGCCTATTCTGGGG
TCCAAATCATGAGATATACTGTAGGATACAGAAACATCATTAGATTATCACTGGACAACCAAAGACGCAGTATGCCAGTC
AGATCATAGTAGTCGACGTTACCATAACCAATCATGTTTGCAAATATTATTTGCTGAGGCTGTTGGTGCATTTCATGTGA
GATAAAAGTGAAGACTAGTCTTTTTAGTAAGTATTCACACCCCTTGACTTTTTCCACATTTTGTTGTGTTGCAGCCTGAA
TATAACATGGATTCATTTGAGATGTGTCACTGGCCAACAAACAATACCCCAGAATGTCAAAGTGGAATTATGTTTTTAGA
AATGTATTCAACACCTTTGTTGTGAAAAGCCTAAATTATTTCAGGATTAAAAATGTGCTAAAGAAGTCACATAATAAGTT
GCAATAATAGTGTTTAACATATTTTTTTTATGACTACCTCATCTCTGTACCCCACACATACAAATGATTGGAAGGTCTCT
CAGTCTCTACAGTGAATTTCAAACAGAGATTCAACCAGAAGGACCAGGGAGATTCACCATGAGGCCAATAGTGTCTTTAA
AACAGTTACATAGTTTAACTGTGATATAAGAAAACTGAGGATGGATCAACAAATTCACCTTTAAGCAGGACAATAACCTA
AAACACAAGGCCAAATATACACTGGAATTGCTTACCAAGATGACATTGAATGTTCCCGAGTGGCCTAGTTACAGTTTTGA
CTTAAATCGGCTTGAAAATCAATGGCAAGGCTTGAAAATGGCTGTCTAGCAATGATCAACAACCAACTTGACAGAGCATG
AAGAGCATAATGTGCAATTATTCTACAATCCAGGTGTGCAAAGCTCTTAGAGACTTACCCAGAAAGACTCACAGCTGTAA
TCGCTGCCAAAGGTGATTCTAACATGTATTGAGTCGGGGGCGTGAATACACATGTAAATCTGATATTTCTGTATTTCATT
TTCAATAAATGTGCAACAATTGTTAAAAACATGTTTTCACTTTGTCATTATGAGTTATTGTGTGTAGATGGGTGAGAAAA
AAAAAAAATTCATTCATTTTGAATTTAGATTGTAACACAACAAAATGTGGAATAAGTCATGGGGTATGAATACTTTTGGA
AGGCACTGTATGTAGCCTGATACATCATCCGGTGATAGCTTGCTAGCTAGTCACCGTCTGACATAATACAATACAATTGT
TTGGAAAGGTCTTCTCAGGACCTCCGCAAGCCAGAATGTTGAATAATATTACATTTGAACTTACTCTACCGGTTGTCTTG
GTGGTTGGGCATTATGTTGAAAATTTGAGACAAAATATCCACAGAAAAGTGTTATTAAACTGACTTTTCTAAACCACCCC
CTGCTCGCATCATAAGCCATGACAAAATGTGTAGAATTGCAGGACCCCCGAAAACCAGAAAAATAGCAAACATTTTCAAG
GAACAGAAATAGAACATGGAACAAAAGTAATCCACACTGTTCCGGGACAGAACTGTTATTTTAAAAGCACAAAAACCAGT
TAATAACCTTCTTTTAAGTTGCAGGCATTTTTACTAGTCCCCCAAGAAAATGCCGCAAAGCGCCTATGCAAAACCCTCAC
TCTGTCACTCAGAAACTTATTCCAGTGTCTGCCTGCAAGCTGAAATCTTTGTGTGTGTGCATATTTAGGATACCTGCCCA
TCCCCCCTCCAAAGCATAGGCTACTGTACTGACGTTAGAATCGTGATTCAGAGGATAGGGAGATAGATTTTTAATTAGCT
TGGGAAGAATGGATTAACTTTTTAAATGCTAGTTAAGGATGCAATAGGCCTAGTTATAATGTTTCACAAAGGATTTATTA
ACTACAAAAAGGTAAGATGTGTTTTTAATTCTGGTGCCACTCTGCACACACAAGCTTGTTAGCTAGCTAGCTCAAGCTTG
TTAGCTAGCTAGCTCAAAGGACATTCAAAGTTCTTCCATAGAAGCCGGTCCTCTTAGGTATAATTCAGTGGACCTAATTC
AGATAATGCATGTCATAACAACATGCCCAGGACTTCAAGCCTCCTCCACCCCCCTCTCTCGCTCTTTTCCTTCCCCACCA
ACAAAATGTCAGTCGCATCTTGCACCATAGGCTACACTTGTCTGTCCATCACACATGTAAACAACTACCTTGCCTGCTCT
ATCCGCATTGATTGGTAAAGTAATTTAATAAGCTAAATGTAAAAAACTGTTGATATCACAGGTTAAAAAAGGAACAGTAA
GGAATGATATAAACCGGTACTTGTTGGGGGTTCGAGCTAGTTCAGAACTATATTTTGCTGGCTGGAACAGTGGAACGGAA
TGAAATAAATGTGGTTCTGTTCAGAACGAAACGATTGGAAAATAATTTTGGTTCCAACCCCTGGTTGTGGGGCCCCCTGA
AGGTAGGTGCCCAAAACCCCAGATGTGGTGATCCGGCCCTCAAGTACAGTTGAAATACATTTATTTCAACTGTACTTGAA
ATAAATACATTTAAACTACGTTTTTCACAATTCCTGACATTTAATGCCAGTAAAAAGTCCCTCTTAGGTCAGTTAGGATC
ACCACTTTATTTTAAGAATGTGAAATGCCAGAATAATAGTAGAGAGAATGACTTATTTCAGCTTTAATTTCTTTCATCAC
ATTCCCAGTGGGTGAGTTTACATACACTCAATTAGTATTTGGTAGCATTGCCTTTAAATTGTTTCATTTGGGTCAAATGT
TTCAGGTAGCCTTCCACAAGCTTCCCACAATAAGTTGGGTGAATTTTGGGCCATTCCTCCTGACAAAGCTGGTGTAACTG
AGTCAGGCTTGTAGGCCTCCTTGCTCGCACACACTTTTTTAGTCTGCCCACAAATTCTCTATGGGATTGAGGTCAGGGCT
TTGTGATGGCCACTCCAATACCTTGACTTTGTTGTCCTTAAGCCATTTTGCCACAACTTTGGAAGTATGTTTGGGGTCAT
TGTCCATTTGGAAGACCCATTTGCAACCAAGATTTAACATCCTGACTGATGTAATGAGATGTTGCTTCAATATATGCACA
TAATTTTCCTCCCTCATGATGCCATCTATTTTGTGAAGTGCAGCGAAGCACCCCCACAACATGATGCTGCCACCCCCGTG
CTTCATGGTTGGATGTTGCCACCCCCGTGCTTCAAGGTCGGCTTGCAAGCCTCCCCCTTTTTCCTCCAAACATAACGATG
GTCATTATGGCCAAACAGTTTTATTTTTGTTTCATCAGACCAGAGGACATTTCTCCAAAAAGTTCGATCTTTGTCCCCAT
GTGCAGTTGCAAACCGTAGTCTGGCTTTTATATGGCGGTTTTGGAGCAGTGGCTTCTTCCTTGCTGAGCAGCCTTTCAGG
TTATGTCGATATAGGNNNNNNNNNNNNNNNNNNNNNNNNNNNNNNNNNNNNNNNNNNNNNNNNNNNNNNNNNNNNNNNNN
NNNNNNNNNNNNNNNNNNNNNNNNNNNNNNNNNNNCCAAGAGGCACTGAGTTTGAAGGTAGGCCTTGAAATACATCCACA

GGTACACCTCCAATTGACTCAAATGATGCCAATTAGCCTATCAGAAGCTTCTAAAGCCATGACATCATTTTCTTGAATTT
TCCAAGCTGTTTAAAGGCACAGTCAACTTAGTGCATGTAAACTTCTGACCAACTGGAATTGTGATACAGTGAATTATAAG
TGAAATAATCTGTCTGTAAACAGTTGTTGGAAAAGTTACTTGTGTCATGCACAAAGTAGATGTCTTAACCGACTTGCCAA
AACTATAATTTGTTAACAGGACATTTGTGGTTCAAACACGAGTTTTAATGACTCCAACCTAAGTGTAGGTAAACTTCCGA
CTTCAACTGTATATGCATGCATTGTGTGCATGTATTTACATTGTGTTCTCAAACAAATCCTGAGAGCCACACTCAGACCC
GTGTTTGGAAGTCGATTTAAGGCCTACTTTCCTGTAGATATTGTGTCACGGTTGTCGTCGGTGAAGGAGGACCAAAACGC
AGCAGGTAAGTGCAGGCTCATCTTGACGTTTATTTATTTTCAAAATGAATGCCAAAATAACAAACCACGAGAATGAACGA
ACAACCAACAGTCTGGCAAAGCATAGGCTCAACACAGAACAATCACCCACAAATAACAAACACACCCTCATATATGGGAC
TCTCAATCAAAGGCAGATAGACAACACCTGCCTTCAACTGAGAGTCCCAACCCCAATTAACCAAACATAGAAACAGACAT
ACTAGACAAATCATAGAATACCTGAAAACCAAACAGTGCCCAAAAACCCCGGAATACTTAAACCAAATGCCCCTTCAACA
AAACACACCACCCCGAACCACATAAAACGAATACCCTCTGCCACGTCCTGACCAAACTACAATACCAATTAACCTTATAC
TGGTCAGGACGTGACATATTGGCTGTGTCCGAAATCTGTCTTGCCTACTACTTACTAAAACTGCATACTGTGTACTAATC
GTAGTACATACAATTTAGAACATACTGTTTGGTCAAATCAAATGCAGTAAGCAACACATTTCAACATTGTAGGCTCATCC
ATATTGAGAATGCTCATCTAGTGCGCATATGCACAATTGCGTTCCGCCTATCTGATTATCAGCTGTTATGAAACACACTT
GGGTCTCGAAAGACGATTCTCTTCCTCAATAGTGTGCAGAGAATTCAACTAGAGGAAAAGCCAAAATGAATATGACAGCA
TTTCCCAAACTCTGTCCTCGGGACCCCAAGGGGTGCACGTTTTGGTTTTTGCCCTAACACTACACACTTGATTCAAACGA
TCAAAGCTTGATGATTAGTTGATTATTTGAGTCAGCTGTGTGCCAGGGCAACAAAAAAAACAGGGTCTGGACAACCACGG
AGTATGAAATCCTGGCATTTAAAGTTGTCACGACTTCTACCGAAGTCGGTTCCTCTCCTTGTTCGGGCAGCGCTCGGCGC
TCGGCGTTGCCGGTCTTCTAGCCATCATCGATCCACTTTTCATTTTCCATTTGTTTTGTCTTGTCTTCTCACACAACTGG
TCTCAATTTCCCTCATTACATGTTGTGTATTTAACCCTCTGTTCCCCCCATGTCTTTGTGCAGAATTGTTTATTGTAAGT
GCATGTGCGCGACGGGTTTTGTACCCATTTGTTTGTGGTTCTGGTTACCGGTGGTTTTATGAATAAAACTGCTCTGTTGA
TTACCAAGTTTTGCTCTCCTGCACCTGACTTCCCTGCTGCCAGTTAGGCACTCCCTTACAAAAGTATACAAAAATTATCA
CATAGGCCTACTACAAAACCTTGTATTTCCCATACCCAAAATGCCTACTATTTAGCAAGAATGGGTATTCGGACATGACC
AATGTCTCAGATTTTTTATGGCATAAGGAAGTACTCAGCAGACACTAGGGCTGCGTTCAATATGACACAATGATGTGCAA
TGTTCAATTGAACGGAAAAGGTGCTGTTCTGAGTGACCAGTTGAAAAACGGGGTTGGGGAACCCTGTCAGCATGGCCACT
GCTATTTAAATACGTCATGCATTGTTCAAGCCAGACCCAGCCACACCCACGAAATGGAGCAAATAAAATAAAATCAACAT
TTATTGACCAAGTACACAGCTTTGTATATATACAGTGGGGGAAATAAGTATTTGATTCCCCTGCTGATTTTGTACGTTTG
CCCGCTGATAAAGAAAGGATCAGTCTATAATTTTAATGGTAGGTTTATTTGAACAGTGAGAGACAGAATAACAACAAAAA
TATCCAGAAAAACGCATGTCAAAAATGTTATAAAATGATTTGCATTTTAATGAGGGAAATAAGTATTTGACCCCCCTCTCA
ATCAGAAAGAATTCTGTCTCCCAGGTGTCTTTTATACAGGTAACGAGCTGAGATTAGGAGCACACTCTTAAAGGGAGTGC
TCCTAACCGCAGCTTGTTACCTGTAAAAATGACACCTGTCCACAGAAGCAATCAATAAATCAGATTCCAAACTCTCCACC
GTGGCCAAGACCAAAGAGCTCTCCAAGGATGTCAGGGACAAGATTGTAGACCTACACAAGGCTGGAATGGGCTACAAGAC
CATCGCCAAGCAGCTTGGTGAGAAGGTGACAACATTTGGTGCGATTATTCGCAAATGGAAGAAACACAAAAGAACTGTCA
ATATCCCTCGGCCTGGGGCTCCATGCAAGATCTCACCTCGTGGGGTTGCAATGATCATGAGAACGGTGAGGAATCAGCCC
AGAACTTCACGGGAGGATCTTGTCAATGATCTCAAGGCAGTTGGGACCATAGTCACCAAGAAAACAATTGGTAACACACT
ACGCCGTGAAGGATTGAAATCCTGCAGGCCCGCAAGATCCCCCTGCTCAAGAAAGCACATATACATGCCCGTCTGAAGTT
TGCCAATGAACATCTGAATGATTCAGAGGACAACTGGGTGAAAGTGTTGTGGTCAGATGAGACCAAAATGGAGCTCTTTG
ACATCAACTCAACTCGCCGTGTTTGGAGGAGGAGGAATGCTGCCTATGACCCCAAGAACACCATCTCCACCGTCAAACAT
GGAGGTGGAAACATTATGCTTTGGGGGTGTTTTTCTGCTAAGGGGACAGGACAACTTCACCGCATCATTTGACGGGGCCA
TGTACTGTCAAATCTTGGGTGAGAACCTCCTTCCCTCAGCCAGGGCATTGAAAATGGGTTGTGGATGGGTATTCCAGCAT
GACAATGACCCAAAACACATGGCCAAGGCAACAAAGGAGTGGCTCAAGAAGAAGCACATTAAGGTCCTTGAGTGGCCAAG
CCAGTCTCCAGACCTTAATCCCATAGAAAATCTGGGGAGGGAGCTGAAGGTTCGAGTTGCCAAACGTCAGCCTCGAAACC
ATAATGACTTGGAAAAGATCTGCAAAGAGGAGTGGGACAAAATCCCTCCTGAGATGTGTGCAAACCTGGTAGCCAACTAC
AAGAAACGTCTGACCTCTGTGATTGCCAACAAGGGTTTTGCCACCAAGTACTAAGTCATGTTTTGTAGAGGGGTCAAATA
CTTATTTCCCTCATTAAAATGCAAATCATTTTATAACATTTTTTACATGCGTTTTTCTGGATTTTTTGTTGTTATTCTGT
CTCTCACTGTTCAAATAAACCTACCATTAAAATTATAGACTGATCATTTCTTTGTAAGTGGGCAAACGTACAAAATCAGC
AGGGGATCAGATACTTTTTTCCCCCACTGTATGCAGTGCCTTCAGAAACTTTACAATTGTCTCATTCATCCCCCCTCCTC
TCTCCTGTAACTATTCCCCAGGTTGTTGCTGTAAATGAGAATGTGTTCTCAGTCAACTTACCTGGTAAAATAATGGTAAA
ATATTATTATTTTTTAAATATGCATACCCCATGACTTATTCCACATTTTGTTTTGGAACAGCCTGAATTGAATTTGGATT
AAATCTGTTTTTTTTCTCACCCATCAACACAAAACCCATAATGACAAAGTGAAAACACTTTTTCAGAAATGTTTGCACAT
TTATTGAAATTGAAATACAGACACATCTCATTTACATAAGTATTCACACTTCTGAGTCAATACATGTTCAAATCACCTTT
GGCAGCAATTACAGTTGTGAGTCTTTCTGGGTAAGTCTCTAAGAGCTTTGCACACTTGGATTGTAGAATATTTGCACATT
ATTCTTTTTAAAATTCTTCAAGCTCTATCAAGTTGGTTGTTAATCATTGCTAAACAGCCATTTACAAATCTTGCCATGGA
TTTTCAAGCCGATTTAAGTCAAAATTGTAACTAGGCCATTCAGGAACATTCAATGTCATCTTGGTAAGCAGCTCCAGTGT
ATATTTGGCCTTGTGTTTAAGGTTAATGTCCTGCTGAAAGGTGAATTTGTCTCCCAGTGTCTGTTGGAAAGCAGACTGAA
CAGTGTTTTCCTCGAGGATTTTGCCTGTGCTTAACTCTAATCCGTTTCTTTTCATCCTAAAAAACTCCCTAGTCCTTGCC
GATGACAAGCATACCCATAACATGATGCAGCCACCACCATGCTTGAATGTGTTGGATTTGCCCCAAACATAACACTTTGT
ATTCAGTATTACTTTAGTGCCTTGTTGCAAACAGAATGCATGTTTCGGAATATTTTTATTCTGTACAAGCTGCCTTATTT
TCATTCTGTCAATTAGGTTAGTATTGTAGAGTAACTACNNNNNNNNNNNNNNNNNNNNNNNNNNNNNNNNNNNNNNNNNN
NNNNNNNNNNNNNNNNNNNNNNNNNNNNNNNNNNNNNNNNNNNNNNNNNNNNNNNNNNNNNNNNNNNNNNNNNNNNNNNN
NNNNNNNNNNNNNNNNNNNNNNNNNNNNNNNNNNNNNNNNNNNNNNNNNNNNNNNNNNNNNNNNNNNNNNNNNNNNNNNN
NNNNNNNNNNNNNNNNNNNNNNNNNNNNNNNNNNNNNNNNNNNNNNNNNNNNNNNNNNNNNGTGGGTTACAGACATACTCA
ATCTCTCCCTATCACAGTCTGCTGTCCCCACTTGCTTCAAGATGTCCACCATTGTTCCTGTACACAAGAAAGGGAAGGCA
TTCCACAGTAATAACCTCATACCAATGGTCAAGCATGGGGTTGGTAGTGTGATGGTTTGGGGATGCTTTGCTGCTTCAGG
ACCTGGATGACTTGCCTTAATAGAAGGAACCATGAATTCTGGTCTGTATGAGTATATTCTACAGGAGAATGTCAGGCTAT
CCATCTGTGAGCTGAAGCTGAAGCGCAGCTGGGTCATGCAGCAAGACAATGATCCAAAACACACACAATCAAGTCTACATGG
AAATGGCTAAAAAGCAACACATTTGAAGTTTTGGAATGTTCTAGTCAAAGTCCAAATCCCAATTGAGATGATGTGGCAGG
ACTTGAAATGAGCAGTTCATACTTGAAAACCCACAAATGTCGCTGAGTTAAAGCAGTTCTGCATGTAAGAGAGGGACAAA
ATTCATCCACAGCGATGTGAGAGTCTGATCGACAACTACAGGAAGCATTTGGTTGCAGTCCTTGCAGCTAAAGGTGGCAC

AACCAGTTAGAGTGTAAGGGGGAAATTACTTTTTCCACACAGGACATGGGGTGTTGCATAACTTTGTTTATGAAATAAA
TTAAATAAGTATGTAATTGTTGTGTTATTTGTTCACTCAGGTTCACTTTATCTTTTATTTATCAGGTTTTGGTTGAAGAT
CTGATAACATTCCGTATCAAAAATATGAGAAAATTAGAAAGGGGGCAAATACTTTTTCACGCACTTGTCAAGTGTCAGTG
TGCAGCGGGTAGGACGGAGTCAGGCGCAGGACACAGAACTGAGTAAATAACTTCCTTTACTCGAATAAAACAACAAAATC
ATTCCACGCAGGGAAACACACCAGCTCACAGAAATCTCGACCACTTAACAATGAACAAACACGCACAAAAACCATGGGGA
AAACAGAGAGTTAAATATTGAAACTAGGTGTGTAAAACAAAGACGAAACAAATGGAAAATGAAAAGTGGATCGGCAATGG
CTAGAAGACCGGTGACGTCGACCGCCGAATGCCGCCCGAACAAGGAGAGGGATTGACTTTGGCGGAAGTCGTGACTAGGA
TGAGCCATGACTAGAATACAGTATATACATATAAAATGGGTAACACAGTATGTAAACATTATTAAAGTGACCAGTGTTCA
ATGATTCTATATACATAAGGCAGAGGTCTCTTAAGGTGCAGGGTAGTGTTCCTGGTGGAAGCCTGCTAGTCTAAGAAGTG
CAGGGTAGTGTTCCTGGCGGTATCTAAGAAGTGAGTGCTATGGGGCGATAGTCATTTAGTCCGGTTACCTTCCCTTTCTT
GGGTACAGGAACAATGGTGGACATCTTGAAGAAAGTAGGGACAACAGACTGGGATAGGGTACGATTGAATATGTCAGTAA
ACACTCCAGCCAGCTGGTCTGCACATGCTCTGAGGATTTGGCTTAGGATGCCATCTGGGTTGGCAGCCTTGTCAGGGTTG
ACATCATTGAATGTCTTACTCATATCGGCCACAGAGATCGGGAGCACACAGTCCTTGTCGGTGGCTCTGTGCTGTTTTCC
TCGAAGCAGGCAAGAAAGTGTTAAGCTTGATTGAGGCATCGGTGTCTGCGACGTACCTCAAAGTCTGTTCAAGAACATTG
AACATTTTGGGGAAACATGTCATTCAGTACTAACCGTCACGCAACGTAGCAAACATTTAATCAAACTGAACACACTTTAG
TAGAGCAGCCTAAATTCAAATTAAGTTTATGCAACAATCTTACGTAAAAGGACTGTTAGGTCTTCTGGAATCTACGACAA
GTGTGGAGATGTCCACACTCAGAAGCGTAGAGGCTAAAGTGTACAGTATGCTACACGTATGCCAAAGTTATGCATCTAGT
GTTGCAGACCAGTTTAAAACAAATGCAATCACTGCTTACCTGGTGTGTCGGTTCATGAAAAACGCACCCCAGCACACTAC
GTTATCAAATTACAGAGCCATAGCCAACCAGCAAAGTGGCCTAATTTAATTCCTAACGCTTAAAACTAAGCGGAAAAACA
GATAGGTCATTTAGCTACAAAACAAGAAGCTCCAGCCGATTCAAACTCTCAATGAACAGCATTAATGAACAGCACTTACC
TTTCTCTGTTGAAATAGATGTGATGATGGCATTCACGCTGGATCAGGTGATCCTTCGGTGATCGCCTTGTCTAGCAATCA
AAAACCAAAGAGAAACGTTTACTTGTAGCCTCTTTTTTATCAAAAACATAAACCCAGAATATTCAGTATTCATCCAAATC
ACCCCTATGGGACGGCAGCTCCTGGTCAGCCCAGCCCAAGCTCTTCTCTGTTCTGGCACCCGAAAGGTGGAACCAGCTTC
CCCCTGAAGCTACGACAGCAGTCTTCCGAAAACATCTGAACCCCCCCCCAAAAAAAAAATAGGAAAATATACAGTTGAAG
TCAGAAGAGTCATTAAAACTTGTTTTTCAACCACTCCACAAATTTCTTGTTAACTAACTAAAGTTTTGGCAAGTCGGTTA
GGACAACTACTTTGTGCATGACACAAGTAACTTTTCCAACAATTGTTTACAGATTATTTCACTGTATCACAATTCCAGTG
GGTCAGAAGTTACATACACTAAGTTGGCTGTGCCTTTAAACAGCTTGGAAAATTCAAGAAAATGATGTCATGGCTTTAGA
AGCTTCTGATAGGCTAATTGACATCATTTGAGTCAATTTTGATGTGTACCTGTGAATGTATTTCAAGGCCTACCTTCAAA
CTCAGTGCCTCTTTGTTTGACATCATGGGAAAATCTAAAGAAATCTGCCAAGACCTCAGAAAAAAATTGTAGACCTCCCC
AGTTCATCCTTGGGAGCAATTTCCAAACACCTGAAGGTTCCACGTTCATCTGTATAAACAATAGTACGCAAGTATAAACA
CCATGGGACCACGCAGTCGTCATACCACTCAGGAAAGAGATGCGTTCTGTCTCCTAGAGATAAACGTATTTTGGTGCGAA
AAGTGCAAATCAATCCCAGGACAACAGCAAAGGACCTTGTGAAGATGCTGGAGGAAACGGGTACAACAGTATCTATATCC
ACAGTAAAACAAGTCCTATATCGACATAACGTGAAAGGCCGCTCAGCAAGGAAGAAGCCACATAAAAAGCCAGACAACGG
TTTGCAACTGCACATGGGGACAAAGATCGTACTTTTTGGAGAAATGTCCTTTGGTCTGATGAAACAAAAATATAACTGTT
TGGCCATAATGACCATCATTATGTTTGGAGGAAAAAGGGGGAGGCTTGCAAGCATAAGAACACCATCCCAACCGTGAAGC
ACGGGGGTGGCAGCATGATGTTGTGGGGGTGCTTTGCTGCAGGAGGGACTGGTGCACTTCACAAAATAGATGGCTTCATG
AGGGAGGAAAATTATGTGGATATATTGAAGCAACATCTCAAGACATCAGTCAGGAAGTTAAAGCTTGGTCGTAAATGGGT
CTTCCAAATGGACAATGACCCCAAGCATACTTCCAAAGTTGTGGCAAAATGGCTTAAGAACAACAAAGTCAAGGTATTGG
AGTGGCCATCAGAAAGCCCTGACCTCAATCCCATAGAAAATTTGTTGGCAGAACTGAAAAAGCATGTGTGAGCAAGGAGG
CCTACAAACCTGACTCAGTTACACCAGCTCTGTCAGGAGGAATGGGTCAAAATTCACCCAACTTATTGTGGGAAGCATGT
GGAAGGCTACCTGAAACGTTTGACCCAAGTTAAACAATTTAATTAAAGGCAATGCTACCAAATACTAATTGAGTGCATGT
AAACTTCTGACCCACTAGGAATGTGATGAAAAAAAAAAAGCTGAAATAAATCATTCTCTCTGCTATTATTCTGACATTTAA
CATTCTTAAAATAAAGTGGTGATCCTAACTGACCTAAGACAGGGGATTTTTACTAGGATTAAATGTCAGGAGTTGTGAAA
AACTGAGTTTAAATGTATTTGGGTAAGGTGTATGTAAACTTCCGACTTCAACTGTATGCTGAAGAAAAATATAAACGCAA
CATGCAACAATTTCAAAGATTTTACTGAGTTACAGTTCATGTAAGGAAATCAGTCAGTTGAAATACTTTCATTAAACCCT
AATTATGGATTTCACATGACTGGGAATAGAGATATGCATCTGTNNNNNNNNNNNNNNNNNNNNNNNNNNNNNNNNNNNNN
NNNNNNNNNNNNNNNNNNNNNNNNNNNNNNNNNNNNNNNNNNNNNNNNNNNNNNNNNNNNNNNTGCCTCAGCAGGTGACA
ATTTTGCATAGAGTGATCAGCTGTTGATATGGCCTGGGAATGTGTCCCACTCTTCTTCAATGACTGTGCAAAGTTGCTGG
ATATTGGCGGGAACTCTTCAATCCAGAGCATCCAGAACATGCTCAATGGGGGACATGTCTGGTGAGTATGCAGGCCATGG
AAGAATTGTGACATTTCAGGTACCAGGAAGTGTGAACAGATCCTTGCGACAGGGGGCTGTGCTTTATCATGCTGAAACAT
GAGGTGATGGCGGTGGATGAATGGCAACATTGGAGGCAGCTTATGGTAGAGAAATGAACATTAAATTCTCTAACAGCTTT
GGTAGACATTCCTGTTGTCAGCATGCCAATTGCTTCTCCCTCAACTTGAGACATCTGTAACGTTGTGTGACAACTGCACA
AAGTGGCCTTTTATTGTCCCCAGCACAAGTTCCACCTGTGTCGTGATTATTGATATCTTAACAAAGGAGAAATGCTCACT
AACAGGGATGTAAACAAATTTGTGCACAACTTTTAAGAGAAATAAGCTTTTTGTGCATATATTTTATATCCGCTCATGAA
ACACTTTGCATGTTGCGTTTATATTTTTTGTTCAGTGTAAAAAAAAAAAAAAAAAAAGCGCCTTTTTTTGAACAAACACTT
AAGTCTTTTCCCTCTTACTAACTCTGACTTTTCTGATAGCTACTTTGAGGAAAAATGTACCTACCATGACTGTGATGTGG
TTATCCAACCTAGTTATCTTATTACTGCTGTTACGAATCCCTTTGGCCCAGCGGTCTAGGGGGGGGATGGATACGAGACC
CGTAACATAAATCACGCAAATTATAATCGTGACAAGTAACAGTGAGAACCAAAAAAACCACAGACAACTGAAATCTACCG
TCAAACTCAAAAGGTTTATTTTAAAAACACACGGTAAAGGGATGTGGGAAAAGGGGCTGAGTTGGACCCAATCAAAGAAA
CAATAATCCAAAAACACCCCTAAACTAGACAAACCTATTTCCACAACAGCTAACTAAAAATACAGAGGGTGGTCCGCCCA
GTTCTAACTAGGGTACTTAGACACAGTATTCCTACGGGTAATGTATGCCCATGGGCGACTTGTCTTGGTACCCCCTTTTC
CCACCAAACAAACAAAACAAAACAATACTCACAGGATTACCGGACAAATGTGACATGTAGTTGCAAAAACAAAAGAGATC
AATACAGAGAGAGACACAGGGAGATAGCGATTGGACACAAGCGCAATTGAGAGAATGAACACAAAGATTGATCTGGGGAG
AAAACAACTGACTGGGTTTTTAAACCAAGGGAAAGGGGCTGTGATAGGTTGAGGGAAAAGGAACAGGTGTCTTCTGATTG
GGGACTGATTGATGACTGATTGGGGAGTGATGATTGTCACCTGTGAGGAGGAGAAGGAGAGAAAAGAAATACAAATACAG
GATACAAACTACCTGTATCCGTAACAACTGCAGTGGGCTTAATCAGGGTCACACAGTGTTTCTTGTTGGTTTTAAACAAA
TCAACTTTGAAACAAAAGTATACACCTCACACACATGGTTATGGGCTTAAAAAAATAAGACACCTATACCATGTCCAATA
AAGAGTTGAAATGTATTCAATTTTGAGTTTGCATCCCCAAATTACACATTATTTACTGTACATCACAGAAGACTGAAATA
ATAACAACATTGTTTGACATAGAAACACCATATTATTATTTTTTTGGATTAATTATGAAAAAAATATGAATAACATTCCA

CCCTTGAGGCCACTAGGTCATTTGACTGCAGGAAAGGGGTATGATGAATGCACTAACTGTAAATCGCTCTGGATTAGAGT
GTGTGCTAAAGGCCTAATGTAAATTGGTTATGACTCTTATTTCATTTTCATGACAGTCTTCATCAATAACCATTGGTTAC
CCGGTTATTGTGCCATGCAGCCCTAATCATAAAAATATATATACAAACGTTTTACACTGCCAGGGATCACAAACCTACAC
AAACAAATTAATCATAATGACTGGCTGTCTTGCAATCTAGCTAGGTGTCCACTAGATTCTACAGCCACAAATTCAATTAT
TATTATTTATTTTTTTGCTTTTTAGTCTTAATTTAAGGTTGGGGTTATGCATACGGTTAGCAGTGTGGTAAAGGTTAGGG
TTAAGTCCTATTTTAATTAAGAGAAATTGTAGAAATGGCGGAGTTCATGACTTTGTGGCAACTAGTGACGGTCCCGCGCG
AACGTTTCATGAGAAGTCTCGCGCTATTCAGGCCGTCGCTGTTTAATGGCCGCCCTCTTACTCGAAGCAATGTCTGAAAC
GGGGAATAACACAAGGAACCCATCGGGGTGTGATAGCTGACCGGGAATTAACAAAAATACTGGGGCATTTCCTCTCCTAA
ACGGTTTCCGAACGGATGGGCAGTGTGGGGGGGATTGCCAAGCGACATAAGATACGAAAGCGATATAGGTGGCTAGCGGG
AGAGGAGAGAGCTGCTAACTAGCTAGTCAACACAGTTGTCACTGCCGGTGAGGCAAGCACCGAGCAACCCTGACAAGAGT
GGGTTTGACGAGGGTGGGAACACAGACTGTTGGACTGGAGCGTGTGACTCTTGGCACCAAATGCTAACATGAGTGGACCT
GGGCAGGATAGCGAGCCAGAGGCAAAAGTCCTCCTCATCAAGCGGCTATACAGGTGAGTCAAATTCCTTTTGCTGGGAAA
CATTACATTAGCTAACTAACAGTAGTAAAATAAATGGAAAGCTAACCTTAGCTAGTTTGCTAACTAACTAGCCATCTATT
CAACTTGCTAGTTAGCTAACTATCCAGTCACCTGTGTGTCTGTGACTTGCATCAGTTAACGTTAGCTAGTTAACTGTACT
TGTCACGAAGCCTTCGTCGAAGGACTATGACATTTTTACGAAGTAGTCGGACATTTTCGATAATATGGGTTTTAAATAAC
CAGATGTAGCCCAGTGGGAACTTATGGAGCGGATAGCTAACTAACGTTAGCTTCTAAACAACTGAGCATTAACAGTACAG
TAACAAGTTACTGTGCTATTAGCTAGCTAACTTATTCCTGTGCCAACTTGCTCTCTTAATAACGTTAGTTGGTTAACTAG
TTGTGACCGCTTCAGCTGTCGGTATTGCCACTACTAGTTACTTGATAGGCCTACCTTGTTTGCTAGCTAGCTACCTAGCT
AGTTAGTTATTTAACAGCTAAGATAACTAGAAGCGTCTTGCAGAACATCAACATATTAATAATGTCGACTGGCTACTTTT
AACATGCATAACTAGGCCAAGTTAGGGAGAAACACCATATTTCACAAGCCTCTTTATATTTACTGAACGTTACCTAGTTA
GGTAGCTGTAATGCCAACCATTATCTCAGTGTGAGACATGACATTTGATATTTTCCAGTTATAACTAGCTAGCTAACATT
AGCTCCTACATTGATAAGAATGAGTTTGCTCATTCCTCCATTGACCATTACCAGTTATTCACATGCCATAATAACTGTAT
CTTGCGTGCTCAGGGCAGTGGTTGAGTCGGTACACAAGCTGGATGTCATCATTGGCAGCAAGTCATCATACAGGGAGGTC
TTCAAGCCTGAAAACATCAGCCTTCGCAACAAGTAAGCATTTTACTCTACTCCAACCTTTGCCCTAAACACTAAGGCAAA
AGGCAAAGCACTAACCCCAACCTTGTCCATCTTCTGTGCCCAGGTTGAGGGAGCTGTGTGTGAAGCTGATGTTTCTCCAC
CCTGTGGACTATGGTCGCAAGGCTGAGGAGCTTCTGTGGAGGAAGGTCTACTACGAGGTCATCCAGGTCATCAAGACAAA
CAAGAAGGTACAGCATTTAAGGGTGTTTTCCCATATAGTCCTCTTTAAAATAACAAATCCCCGTCCTCTTTAAAGTGAAC
TTTGTGGTAAAAAAGAAGCTATAGAACTATGTTCTCTTTGTATTCACACTTCCCTTGCTTTTGAATGAAGACTCAACTCT
TTTGCCAGTTCACTTTATCCATGTTGTGGTCCAAGTCCTCTTTGCATTCAGATTGCTATATTTCAAAAGGAATCAAGATA
TTTTTTTCCAACATTCACTCAATGCACTCTGGGACAAGCCATTTCCATTCCTCGATCAGATTTTCCACCAATGCTCCTTA
TAGGACATATCACTGTGCTCTATACTCTTCTGTAAACAGGTCATCTCTGTATACCTGTTGCAAGACCCACTGGTTGATGC
TTATTTATATAACCCTCACTCCCCACTATCTGAGATATCTACTGCAGTCCTCATCCTCCACATACAACACCTGTTCTGCC
AGTCACATTCTGTTAAAGGTCCCCAAAGTGCACACATCCCTGGGTCGCTCCTCTTTTCAGTTCGCTGCAGCTAGCGACTG
GAACGAGCTGCAAAAAACACTCAAACTGGATAGTTTTATCTCCATCTCTTCTTTCAAAGACTCAATCATGGACACTCTTA
CTTGCAGTTGTGGCTGCTTTGAGGGATGTATGGTTGTCTCTACCTTGCCTCTGTGCTGTTGTCTTGTACCATGTTTTGTG
CTGCTACCATGTTGTGCTTCTGCCGTGTTCTTGCTACCATGTTGTTGTCACGTGGTGTTGCTACCATACTGTGTTGTCAT
GTGTTGCTACAATGCTATGTTGTTGTCTTAGGTCTCTCTTTATGTAATGTTGTGATGTGTGTGGTCCTATATTTTTAATG
CCAGTCCCTGTCCACGCAGGAGGCCTTTTGGTAGGCTGTCATTGTAAATAAGAATTTTGTTCTTAACTGACTTGCCTAGT
TGTTTAAAAAAATATATATATATATACACACACTCTGCAGATTAAAGAATGCTGTAATTGAAAATTGTTCACCCAATGTA
GGCTGCTGCCCCTTTAAGAGCTAAAATAGATTTGGACCGTATGTTGTGATTCTCACCAAATATGTTGTCTTCTCACACTT
CAAACCCACAATCAAATAAACAGCGTATGAAACGCTCTTATAGATCACATATTGCAAACAAATAATCTGAACTAAAAACA
TTTTGGAATATCTGTGGGTTCTTGACAAACGCTAATCAATATCCAAAAAACGGCACATTTTTTTTCTTCTGCAAACCTGC
ACATAATCTTCTCACTTGTGGCGCCATTGTGAGGGTTACGGCAGTGTTGCAGTAGTGTGTGTAATCACAAGCGAACCTGG
GGACCATTACCCTAAAAAATGGAACCGGAGCACGGAATTCAAGTGAACCGAACTCAAACCACCTCTGGAGACGGTCTCAG
TTCGCTTCGGGGGCTCTTTTGAGGGGTCCGAGTTCCTTTTGTAGTGTTAACATGGCACAAAACATTATGCAAACAGCACT
GAGTTCACAAATTTGCTAAAAGGGACCATGTGGGAAAACACCCTAAGTTATTGGGGAAATTTTATGACTGTGGCCAGTAC
AGTGCTTCTACACCTGAATTACTTGCTGTTTGGGGTTTTAGGCTGGGTTTCTGTACAGCACTTTGTGACATCAGCTGATG
TAAGAAGGGCTTAAAAATACATTTGATTGATACAGTGACTGCTGAGCTTTCTGACATGCCCACTTTCTTTCCTCATGCCC
TACACACAACCCTTTTATTTATTTCTAATCTCTTCTTATTACATGGCCCCGTCTTATATCTCCATGTCTTCTACAGCACA
TCCACAGTCGCAGTGCCCTGGAATGTGCCTACAGGACTCACCTGATAGCCGGGGTGGGCTTCTTCCAGCACCTGCTGCTC
TACATCCAGTCCCACTACCAGCTGGAAATGCAGGACTGCATCGACTGGACCCATGTCACCGACCCCCTCATCGGTGAGAC
ACACACACACACTTAGGTCAGCGGTATCTGTGATTGACACAGTGTATAAATGATTGTATTTGTTTTTGTAGGACGGAAGA
AGCCGGTATCAGCCACCCCTAAGGAGATGGAGTGGGCCCAAATGGCCTGCCATCGCTGCCTGGTGTACCTGGGAGATCTA
GGTAATACAAACTTTTAATCTTTCTCACCAAAGAGATTTGAGACAACCTTATTTGTATGACTATGGAATTGCATTCCCCA
AATGCAAGGTCACATTGATTTGAATGTACAGGAGAGGTTTACGGGAGGTTGTTTGAATGTTTTCTGCAGCCCGTTACCAG
AACGAATTGGCTGGGGTGGAGGCTGAGCAGCTGGCAGAGCGGTTCTACCACCAGGCCCTGTCTGTCACTCCGCACGTAGG
TGAGTTGACAACAAGGTGTTACCGGCCCTCCCTTTCACAGCACATTAAACACCGTTTTTTGTTTTACACTAACCGGTTTA
CTCACACCACCATCTACAAATGAATCACTCTTGTTATATTTCCCACATAGTGATGAACTCACACAGCACTTATTGATTGA
GCCTTATTATATCACTGGTCAAAGCTTTACGCAGCTCATAGTGATCTAGCTGAACTGTTAGCGGGCCATTTGGGCCAGGA
AGGGGGCATTCAGTAAATGTTAGCGGGCCAGGAGAGGGCATTCAGTCTCAAGAAATCACTGATCCACATTTCAAACGATC
TGTCTTTTTGAGAGGGCTTCAGAGGCCTTCATCAGTCGAACTGAAAGTCAACATTAAATGGCCTCTGCTAAAGGAAAGCG
ATGTGTTTAGTGTGCCATGATACTGACAAGTGTTTGATGTTTTGTTTTCAGGAATGCCTTTCAATCAGTTAGGTACACTG
GCGGGGAGTAAATTCTACAACGTGGAGGCCACCTATTACTACCTACGCTGGTGAGAGGAGACTTCAACATGTCTAGATTT
ACTGCAGCACATCCACAGTCTATCTATGACTGGTCTGTAGACTATGCTGTGCTATTGTTCTCTACCTCAGTGGGGCAATG
TAGTCACACTGCTGGGGAGAAATATATCTTCATTGCAACAATTCATATTTGGATCACATTCAGTAGACTCTAAATAGGAG
ACATGTTTTGAAATGGAAGAGGTTCTGAAACCAGTCTGTTAGAAGGCGGCCAACTTCGGAGCCAGAACATGTAAAAAAAA
AAGACTCACAACCAAGTGGTACCGGGATGATACCACTTGCAATACTCGTTAGTAGTGGTAAGGATACAAAACATGAAGCG
GATTTGAACTTCTTTAGGAAAACAGCCCCGATGTTGGAAAGAAACATTGTTGTCATCAGGTCACAATTATTTTCCAAGTT
ATAGCACACAATATTTTACATACAGCAGGTTTTTAAAGGACCAAAGAGTTGGGTCTGCTTCGTGTTTTCATTGCCATGGA

AAAAAATATTGCCATACTGGTATCGTCAAACCCCTACCACCAAGTACTGATCATGGTTTTCTCCTCTATGTTCCCTTGTT
TGAACAGTATACAGTCGGAGACTCCCTTCGAAGGGGCCTATGGGAATTTGAAGCGTCTGTTTGATAAAGCTGCTAAGATG
TACCACCAGGTGAAGAAACAGGAGATGAAGAAGCTGTCTCCCTCACGGCAAAGGTCAGTCTGGTTTCCTCAGACCACAGC
CCTGAAATGAAAAAACACTAAGTCAGCATTGAAACATGAATTGAGATAGTCCTCTTTGAGATCCTTACTGTTTCTTCAAC
TGTTTTCTCTCCAGATCCAAGGACATCAAGCAACTTCTAGTGAGCTTCATGTACCTGCAGAGTCTACTGCAGCCCAAGAA
CAGGTTAGGACTTTCACTATTCACCATTTAGGCCCTAGTCATTTATCTCAATAGGAACCTCAACATTTTGTCCATATATG
ACACAGCTATCTGATGATAATAACAATTGATTGAATTTTTATAACCCCCTCTGCTCAGTTTCAAAGGGCTTTCTATTGTC
TGTTGGGTACCGTGTTTGTAATGTCTTACCATTTCTCTCCTCCCTGTGAGTAGTCTGCTGGAGACAGAGTTGACATCTCT
GTGTCAATCGGTGCTGGAGGATTTTAACCTGGTGCTGTTCTACCTGCCCTCGCCGCCACACGGCCACGAGGCCACTTCCC
CCAGCGAGGAGGAGCATGAAGAGCACGACTCGCCCTGCGCCCTGCTCCCCGACGCCCTCGTCTTCAAGATGGTGGTCACC
TGCCTCATGGTGGTGCACAGCCTGAAGCGGGGAGGTCAGTAAGAATATAGATGTCATGTATTTTTTTTGAAAATCACTCT
TCAAACATTGCGCTCAAAGGATGTTGTCAGTAGGCATGCGAGGATAGTGACATACTGCGAGGGAGAAATAAGGGGATATG
TTGTATTGCAAAGTTGACATGTTGACTTGTTTGTGTAGGGTCCAAGCAGTACAGTGCGTCAATAGCCTTCACGCTGGCCC
TCTTCTCCCACCTGGTGAACCATGTCAACATCCGCCTGCAGGCTGAGCTGGAGGAGGGGGAGAGCCAGGTGCCCCCGCTG
CAGACCGACAACATAGGTGAGGACTACTCCCAGCTGACCTCATGATGCCTAGACACTATTCATGTTCTCAGTTAATCCTC
TGAAAAACAAATGGAACAATAATGACATTTGTTTGCTCCTAGATGACTCTGAGTTGAGAGACCCGTCTGACCCGTTGACC
TCGGAAGAGAGGCCCCTGCAAAACGGCTCCCTAGACCATGAGGGGGGCCACCGGAGCACCGTGGCCCAGAAAGCAGGCGT
CGGTGAGCGGAAGGCAGAGGAGGAGAAACGGAAGCAAAAGAAAAAGTACGCCCGCCTCTCCATGCTGCGCCGCCGCCGCT
GCGCCACCCGCAAAGAAGACGAGAGCGACCTGAGCGAGGGCTTCGAGAGCGACGAAGAGGAGGAAGAAGAACAGAGGGGC
TGCGTTGACTCTGGGGGCGGCGCCTCGGGACCCCCACTCACACCCTCCACGCTGGGAAAGGAGAGCAAGAGCGGGAGGGA
GCACCCGACAGAGGAGGGGACCTGGGAGAGCGCCTCGGAGGAAGACGAGGAGGGGAGCACGGCCTTTGACGTGGAGACTG
ACTCGGACATGAACAGCCAGGAGTCCCGCTCCGACCTGGAGGATATAGAGGACTCGGAAACCCCGGAGAACTTGGACACA
CCACCTCGGGGGGAGGTGCAGCCTGAAGACGACGATGAGGAGGAGGACGAGCAAGCCGATCCCAGGGAGGACGGAGACGG
GGACACCCCTCCGGCCACCAACGGCCCCCTCCTGCCCAGCGACCCCAGTATCAGCAGTAACCTCCAGGCCATGTCATCCC
AGCTGTTCCAGGCCAAGCGCTGCTTCCGCCTGGCGCCCACCTTCAGCAACGTGCTGCTGAGGCCCCACAGGTCCGCCACC
AACTCCACACCCAACCCCACCGACCCAGACACCGCCTCCACCACCCCCTCCCAGGAGACCCCTCCTCCCCCGGGGGACTC
ACCCTGCGACGCTGGCCCCGGCACTGCCAATGGAACCAACGACAATGGTCAGTAACCTGCCCACACACATTGACGCTGTG
TTGATTACTATGTGATTTTTGTATAGTGCAGGATATGATGAGTTTAAAGATTTAACTTTAGTATACCAATATAATTATGC
ACTTCTAGTTTGTTTTTAGTCTTTCCCAATGACTCCATGGAGTAACAGCACATCGCTAAAATATGCCCTTTTCTAACAGT
ACATTTGAGGTACCCCCTAAGAAAACTAGGGTGTAGATTAAACCCATGTTCATTTCTTTATTTGTCCATTCCCTGTAGAG
GTGGATTCCGATTCAGAGTGTAGCGTGCACAGCAACCAATCAGTACACAGCGAGAAGACCCTGTCGGAGAGACTGGAGAT
TCTGACCGATCAGGGGCTCATCCAGGTGGTCAAGGTGTTTGTGGATTGGCTGAGAACCAACACTGACATTATCGTCATGT
GTGCACAGGTAAAGGGGGTGGATTTTTCACTAACCTAGAACCACTTACAAACAGTATGCATTGACAAATCTAATTTGCAT
CAAATTGGTAAAATACAAGTTAGTCTTTGTATCAGCTGAGTCGGGACAAGGATTTATAGTCTTCATCATTGTTGGTGTTT
GTTTCACTGTCCATCTTCCGCTCAATGTAATTCCGTTACTCTGTCTGTAGAGTTCTCAGAGCCTATGGAACAGACTGTCT
GTGCTACTCAACCTGTTGCCTGACGGGAGCAAGATGCTGGAAGCAGGCGAGTTACTAAAATCACCTGTCCTTTTTATGGG
TTGTTAATCTGCGTTTACATTTTTTATTTAACTAGGCAAGTCGGTTAAGAACAAATTCTTATTTACAATGACAGCCAAAC
CCTAACCCGGGCGACGCNNNNNNNNNNNNNNNNNNNNNNNNNNNNNNNNNNNNNNNNNNNNNNNNNNNNNNNNNNNNNNN
NNNNNNNNNNNNNNNNNNNNNNNNNNNNNNNNNNNNNNNNNNNNNNNNNNNNNNNNNNNNNNNNNNNNNNNNNNNNNNNN
NNNNNNNNNNNNNNNNNNNNNNNNNNNNNNNNNNNNNNNNNNNNNNNNNNNNNNNNNNNNNNNNNNNNNNNNNNNNNNNN
NNNNNNNNNNNNNNNNNNNNNNNNNNNNNNNNNNNNNNNNNNNNNNNNNNNNNNNNNNNNNNNNNNNNNNNNNNNNNNNN
NNNNNNNNNNNNNNNNNNNNNNNNNNNNNNNNNNNNNNNNNNNNNNNNNNNNNNNNNNNNNNNNNNNNNNNNNNNNNNNN
NNNNNNNNNNNNNNNNNNNNNNNNNNNNNNNNNNNNNNNNNNNNNNNNNNNNNNNNNNNNNNNNNNNNNNNNNNNNNNNN
NNNNNNNNNNNNNNNNNNNNNNNNNNNNNNNNNNNNNNNNNNNNNNNNNNNNNNNNNNNNNNNNNNNNNNNNNNNNNNNN
NNNNNNNNNNNNNNNNNNNNNNNNNNNNNNNNNNNNNNNNNNNNNNNNNNNNNNNNNNNNNNNNNNNNNNNNNNNNNNNN
NNNNNNNNNNNNNNNNNNNNNNNNNNNNNNNNNNNNNNNNNNNNNNNNNNNNNNNNNNNNNNNNNNNNNNNNNNNNNNNN
NNNNNNNNNNNNNNNNNNNNNNNNNNNNNNNNNNNNNNNNNNNNNNNNNNNNNNNNNNNNNNNNNNNNNNNNNNNNNNNN
NNNNNNNNNNNNNNNNNNNNNNNNNNNNNNNNNNNNNNNNNNNNNNNNNNNNNNNNNNNNNNNNNNNNNNNNNNNNNNNN
NNNNNNNNNNNNNNNNNNNNNNNNNNNNNNNNNNNNNNNNNNNNNNNNNNNNNNNNNNNNNNNNNNNNNNNNNNNNNNNN
NNNNNNNNNNNNNNNNNNNNNNNNNNNNNNNNNNNNNNNNNNNNNNNNNNNNNNNNNNNNNNNNNNNNNNNNNNNNNNNN
NNNNNNNNNNNNNNNNNNNNNNNNNNNNNNNNNNNNNNNNNNNNNNNNNNNNNNNNNNNNNNNNNNNNNNNNNNNNNNNN
NNNNNNNNNNNNNNNNNNNNNNNNNNNNNNNNNNNNNNNNNNNNNNNNNNNNNNNNNNNNNNNNNNNNNNNNNNNNNNNN
NNNNNNNNNNNNNNNNNNNNNNNNNNNNNNNNNNNNNNNNNNNNNNNNNNNNNNNNNNNNNNNNNNNNNNNNNNNNNNNN
NNNNNNNNNNNNNNNNNNNNNNNNNNNNNNNNNNNNNNNNNNNNNNNNNNNNNNNNNNNNNNNNNNNNNNNNNNNNNNNN
NNNNNNNNNNNNNNNNNNNNNNNNNNNNNNNNNNNNNNNNNNNNNNNNNNNNNNNNNNNNNNNNNNNNNNNNNNNNNNNN
NNNNNNNNNNNNNNNNNNNNNNNNNNNNNNNNNNNNNNNNNNNNNNNNNNNNNNNNNNNNNNNNNNNNNNNNNNNNNNNN
NNNNNNNNNNNNNNNNNNNNNNNNNNNNNNNNNNNNNNNNNNNNNNNNNNNNNNNNNNNNNNNNNNNNNNNNNNNNNNNN
NNNNNNNNNNNNNNNNNNNNNNNNNNNNNNNNNNNNNNNNNNNNNNNNNNNNNNNNNNNNNNNNNNNNNNNNNNNNNNNN
NNNNNNNNNNNNNNNNNNNNNNNNNNNNNNNNNNNNNNNNNNNNNNNNNNNNNNNNNNNNNNNNNNNNNNNNNNNNNNNN
NNNNNNNNNNNNNNNNNNNNNNNNNNNNNNNNNNNNNNNNNNNNNNNNNNNNNNNNNNNNNNNNNNNNNNNNNNNNNNNN
NNNNNNNNNNNNNNNNNNNNNNNNNNNNNNNNNNNNNNNNNNNNNNNNNNNNNNNNNNNNNNNNNNNNNNNNNNNNNNNN
NNNNNNNNNNNNNNNNNNNNNNNNNNNNNNNNNNNNNNNNNNNNNNNNNNNNNNNNNNNNNNNNNNNNNNNNNNNNNNNN
NNNNNNNNNNNNNNNNNNNNNNNNNNNNNNNNNNNNNNNNNNNNNNNNNNNNNNNNNNNNNNNNNNCCTCAGTGTGACGT
ATGGTAATCTAGTGGGCCACATTTTAAGATATGACAACCACAATCTGCCAACCCAGCACATTAAATGGGATATGTGGAGT
CAACAGTTCTTTGAATGAAAAGATAAAGAGGGCGATTTTCAATGTGCTTAGCCTTTTATCCGGTGTGAAACTCTAGAGCC
AATGATACACAGGTCCTTCTATATCAATGTATCTCTCACGTAACCGATTTTCAAATGTGCTGGCTGTTGGGGCCCATATT
GTCCTTACTTCTTCAGATCTTAACAAAACAATGTTTCTAACCATAACCCACTCTGAATCTGTGATCCAGTAAACTAAGAA
TAGGCTGCGACCAATATGGGCGAAGACACTTGATGTTGTAAACCCCTCTTCTGTCCCCCTCTACCTCAGGTACATACGTT
GCCAGAAGGAGTCAGGGAGGAGTTTTGAGAGGGACAAACTGAAACGCCAGGACATTGAAGCCTGGTGAGTCTCTTCACAT
GGCCACCTCTCAATACTTAGCATTCTTATACAAATGCTTCAAAAGTGGTAAAAGATTTATGAAAGCTTTCTACCAGCAGT

AAAACACCTTCCCACTGTTAACTGTTTCCTTATGTGTCCTGATTTCTAGGCATCTATACAAGACGGTGGACAGCTGTCGT
CAGCTGACCGTCTCCCAGAGCAACGGAGACGAAGACACGGCTGGCATGGTCACTATTCTCACCGGTCACTCAGTGGAGGA
GCTGTGTACCCGCTCTGCGCCCATGAAGGTACACACATCACGAATACAATCACTCACTCAGTTCTTTAGGCTCTCCAGCA
ACCACTGTTGGTATGGACAGCTGGTCTGACTTCTGTGTATTTCTGGTGTATCCCTTGGGTTGTACAGTATTGCTATTAAT
ATTTCCCTGTTATAAACTACTGCCATTTTACCATGGTGTAGATCTTTGTAGAGAACCAGAGTTCTTTCTAAGAGAAGTTT
GGCATGTTCGACCTCCCCCATTCATCCTTACGTTGTATGTTCATTGGTGTTAGAAGAGTTGATGTCTTACCCTCCATTGT
CTGTGTCTCCTTGGTAAGTCGGCCATCCAGGCAGTGGCGACAGCAGGCATGGAGCTGAAGAACATTGTGGAGTTCTACCG
TCAGTGGAAGGAGATTGGCTGAGAGCGTCGCGGGGGCGAGGTCGGCATGGCGGACAGCTGATCACACACTCACTCCCTCT
CTCTCTCCCACTCTGTTGCTCTGTCTGTATCTGCGTCTCCAAGGAAAATCAACTCTAGGAAAGGATGTAAGGGGGGAGAA
TGAGAGAGAGAGAGGGTGGAAAAGTAAAGCAGTTGAAGGAAAGAAAAAGCGAGACACATGGGCAAAGGGGGCAATAAGAA
ACAGCAGAAGACCCCCAAACATAAAGAAGCGATCCCAGAGGTTGGTGAGAATTAAGTGGGGATGCTACGCACTTAAAGTG
CCCCTGGTTCACATCATAACCAGGCATATACTGTTTATGATAGAAATGAGTACATTACGTGTCGAGAGAGAACCACACAT
ACACATTCACGCTCAGTTAAATCCATTGGTTTAGATGTATAGAGCAGTGTTTCCCGTTTCCTCCTTTCCCTCTGTGTTGT
GAAGGTGGGACTATGTCAGCCCTATGGCTGGCTGGGTCTGGTCTGCTAAGGCAGGGGGGTGTGGGGTAAATCCCAGAGAG
AGAAAACGCATCTCTAGCGTGCCAAGCGTTGAGGCTATCGACTTTTCGGAGTCTCATAGCTCATAGGGTTCAGGTTAATG
GACGTACACTTCTCCAGGATTGTTTTCTTTCCTATCAGTTTTTTTGCAGTGTACTTTTTGTGTCCAGTGACTAATGTATT
GTGTTATCTGTGTATGATGAGGAGGAATGACGGGTTGTGCCCTGCTATTGTTTTAATTTGGATTTTATTTTTGTACCCAA
ATATACTTTATTTTGCGTTTTTGTAAGCTGTGCTGAATACACACAGGAATGAAGTGCAGGGGGAGGGATGGGGTTGTCTT
GTATCGTTTCCCTAAACCAGTCTGATAATCCTTCTGTTCTCAAAGAAATAGTCCTGACTTTTGTGTCATGGTGCTGTTTT
AGGAAGAGGTAGACTCGGGTGGGTCAGCTTTGTGCTGTTGCTAGACGTTAAGGAGAGCGGGGCCTGCGGCTGTCCCAATA
TGGCGGTACAAGGTGTTTGTGTTTTGACCCTTGTGAACTCCTGATATATCCCATCTACCATCATCACTAACTGTCCCATC
ATGCACAGTGCAAAGGAACTCTGTATATGTGTGTGGCCACGCATCAACAATAAAATGAACTCATTTTAAAATGTGTTTTC
TTGTTTTTACTCTCAAACCATTTACAGTGGCAACTTTTTCAAAGCTTTACACAATTCATGACAAGGCACAGGACTCTCTG
AATTGGGAAAGCAACCTATTTGAACTGAACATACTCATGGCTGCACCCTTATGACAATAATAGCCATTGTTTCAGTAAGT
TGAAGAACTAATCCAGAGATGACGACTGACCAACATTAAAATGAAATAGTGTGTATCAAATAGTCCGGGTAGCCATTTGA
TTAGCTGTTCAGAAGACTTATAGTTTGGGGGTAGAAGCTGTTGAGAAGCCTTTTGGACCTAGCCTTACTGCTCTGGTACC
GCTTGCTGTTTGGAACACATTTAATGTTTTTAAATGTATAGGTTTATTCCAAAGTTTCGAAACACCCTTACCTATTGAAA
TATGGTAGTTCTGTTCCACTTTCCTCTGAAACTTCTCATTCTGAACCCGTTCAGTGCAGCCTGGCTCATCCATCAGTCTC
GGTCCAGTGAGTAGGACTCGGTGTGCCATCTATCCTGCTCTCTTCTGTTGTGAGGTCTCGTGATCCTCACTACCCTGGAT
GCCTGGGCCTAATGCCCACCATTCTGCATGGCAGAGCGAGACCAATCCCCTGGCAGCGGGGTCAGCGCCACCCCAACAGA
AGATGGTTGGCACAGCACAGGGCTGGCCCACTTTCTGGTGGTGGGGAAATGAACAGTCTAGGGCCCGGTTTCCCTAAAGC
ATCTTAAGGCGAAGCAATGATGGCAAAAATGTGTGGAATTTTAGGAATTTGGCTTTAAAACTGAAAAATGTTCTCAGCCT
CATGGCAGCACTTTTTTCTTCTTCTCTGCCCCGTGGCAGAAAGTGAAGAATTGCAGGGAATTCACTTAAAAAAAGTAATA
ATAATTATCACAGTGTAGAATAGCATTAGGAAACAGCATTTTTTTTTCTTCTCTGCACCATGGCAAAATGTGTTGAAATG
CACATTTTGGCAGAGGTCAGTGCCCCGTGGCCCCCTTGCCTCCAAATGCGGTAGTCCGGCCCTGGGGGTGCTGAGGGAGG
ATAAAAAGTCCTAACAAAGCATTTTGTCAAAGTGCCTCTCAGTCCATGAAAAAAAGGCCCTGCTTTTCATGTAGATTTGG
TTCAGTTGGAATATTATGAAGTGACCTGCCAGCCTTGATTTACTGAGTGAGGATTTCTCAGGAATTTAAACTTTGCCTAT
ATGATCATAGTATTGAATAGGCTACAGATGAAAATCACCGTTGATATCACCCAATAGTCTTTGGAGCTGTTTCAAATATT
ATGTAATGTGAAAAACTTTGAAATGTGACATTTTGATTAAGAAAATGTTATATAACTGTCTGTGCATGTAAAATAGAATT
ATGCGATGATATGGGATGCCTTAAGGCTTGCTCTCTGCCTTGTCTCCTGATTCTCCATAACAATGTTTCTTTCTTGTCCC
CATCCAATCAAAACGTCGCTGTGTGCAGAGGGTGGGATTTCGACATTGGGGCTACGGGTTTGCGGTGTTTTTATTCGCCT
GTGAAGGAATGCAGGACATAGACGGTTTATACAGATTTACACCAGCCTCCAATGGTCAGGATAACCGGATTTACGATGGG
ACTATAGTTTATTACAAGCAACAATTGTTCATTTTGGTCAGTGACTACATTGCTCCAGTGAAGATTTGCGCTAATGAGCT
CACCGCCAATATAAAGCTGAAAGGTTTGACTTTGTTTCTTTCCTTTGCCGTAGTGTGGTTGTTACATTGTATAGGCTACT
GGATATGTATATTTTGTACATGTGCGCCACGAGCGTGTAGGAAACTGGAAATCCTGAAAATGTCTCCACGGAACCAGACT
TTTCAGCTATTATGCAAATTATTTCTGTAGCCTATGTATCTTGTCACTTAAAATATCATTTCACTTTTTGTTGGCATATT
TGCCTTTTTGTATTCATATTCAATTTATATTGATGAAATTGGCATTGTAGCCTGTCATGAGTGTGTAGGTGTTTTAGTCT
GAGGTGTGACAAATTTGACAGTCCTCCATTGCGCCCGAACAAAGGCAATTTGCCTATAACAATTTTATTACAATGTGATA
ACAATATAGTAGGCTATCCGACATGAAAATGAAGACCAAATAGTCCATGAAAGCAGATTAGAGATCCAGTACACCTCTTT
CTTGGAAAGACAACATGCGTTAACGCGCACGGGGGAACATTCTTGGTCTTGTCATTTTTGGCCCGCAGCACAGATAGTGT
TTTCTGTCTTGTTATTGCAACAGTCCACGTGAGCGCTTCTTCAGTCTTTCCAGGGTTCTTCTGGGCAGGGGCTGTGTAGG
GCTGTGCTACTAGAATAGACCAGACACCACCGGCAACCTTTCGGTTACTGGCCCAACGCTCTAACCACTAGGCTACCTGC
CACATCTCGGCACTTTTTAGGGAATATTGGCCCTCCACATGCCCGACTCCCACGGTAAGTGTTATTCTCTTGGGAAAAAT
AGATCCCAATCTCTCACCAGAAAGTTTCTCTCTGAGATTGTCTTTTTAGCCTGTGCCATAACTGAACATAACTGCTCATT
TATCTGTTTGTCTGTGATTGTTGTGTGTCTGCCTTTACCATTTCTCAGTATTAATGTCATATGAATGTGACTCCAGGCAT
TGTACATTCCTATGAAAAAGGCAAATTAATTTGAATAGGATTGAGTGCTCTTTGAGAAACTGTGTCCAATTGTTGGATCT
CGTTTCACTCATGAAACTATGCACTCAGCATTTCTTCTTTCTTTTGCAATGTTCATGTTGTAGACAACTTCAGAGCAATG
CATTGAATTTCACCCTCTGCCCTGCCTCTGCCCTGGTACAGGGTGTTAGTGCCATCTACAAAGATAGGGCGAGAGACATG
TAGGACAGCTACCTTGCCCTACCTAGGCTACATGAGTTAAACATTTCATAGACCAAATGTAGATCTCTTCCCTACATATC
TGGTTGGTCTGGTCTGCTGTTAATGCCACTCTATTGCGATGACAAGTTGTAACTTGTTCCATGTGACACTACGTTCTTCA
AGTTAATCACAGATTATCATAAGTGAAATTATTGGTTGACACCACACCTGCAGAAAGGAACTGTGTGACCCTGTTAAAAA
CATGTGAAATGCATTGTGAGATAATCATTCATCAGTTTGTAGATTGGTGAAAGCAAAGGTTCTGTTACGCTTTCAACAAT
TTATTCATGTTGGATCAGTGATTACAGGAAAGAAGCGACAAGAGAGGAAGGATGCCTTTAAGAAAACACAAATCTTACTC
TTACTGAACATAACAGATGATATTCAAATCCATGTATCACTAGCCTATAATAGTGTGAATTTAAAGTCCTCAACAGACAG
TGTTAATGAGTACTCCATCTGGGTCAATGGGAGGGTCTGATGGTAAGTTCAGCTATACATGGGGTTATTTGTTGGATTTC
TACCTTCTGCAGTACAGTACGGACATGAACTGGTGAATCCTGTTGTTCAGGGTACACTTGACCATCAGTCATATTGTGTG
TGTTGAGGTGATGTCTTCCATGTTCCATTACCAGTACAAACTGTACAGAATCATGCAGCTAGATGACAATGCAATGTTCT
ATGGGGCGAACGATGTGCCTTGTTCTTGTTTGCATAAAACTGACATGTGCCCATGTCAACATTGCTCCCCACATAGACTT
CTTATTGTTAGCTCCTGAGTTATAAAGTCTTCTATTGAGGAAGGTCATGTGACGAGATCACGAGCCAGGGAAAGCAATTC

AGGAGTTGGGCGAGAGAGTGGAACAGAAAGAGGGAAACGTGGGAGAGAAGCTGCGTATTCAGAAGAGGTGGGGTAAAGGG
GAGAGGGAGGACGGAGGGGGAAAAAAGTGAATATTTGTAGATTTTGAAACATGATGGTAGGGGAACTGGGGATGGTCACC
GCCACTGGCAGTCACTCTATCTAGAGTGCTTGTTTTCCCCTTGTTTGCTGTATGTGGCAACCATTACCATGCTCTTGCAC
ACAAACAAACAAAGACGGACACTCACTGACACTTAAACAAACACCTCTGAGTGTTTAACCTACTTCTCACATTCTTGTTA
CTATTTTGCACAATTGTATAGTATTTTAGATAGAAAGTCACGAATGATCAAGTCCTTGCCAATAAATTATTCAGAAGAGA
AATGTGTTGCTACTGTGCAGAACAGCCTTGTGAGTGGGTGGTGTAGTTGAGATTGGGTGGTAGTGAGCACATGAAAAGTG
GTTGGCATCACAGAGGTCCATGAGCAACACCTCCAAGACCTCCCGTTATTCGACTACAACAGAAATAGACCAACAGAATT
CCCATTCTATTAGTTGATGGAGACAGTGGAGGAGGATGAGGGGAAAACGGCTCATAATAATGTCTGTGACGGAACAAATG
GAATGACATCAAATGCATAGAAACCATGTGTTTGATACCGTTCCGCTGATTCCGCTCCAGCCGTTACCACGAGCCCGTCC
TCCCCAACCTCCTGTGGATGGAGAAGGAAATCTGAATGGGAAAGGACCGCACACTGTTAGACACACCCAGGAATCCAAAC
TGATATGCTCAGTTTCACCATTGTTTTAGAATCCAGGCTATACGTCTGTTATTCATAAGTTGTTTTACTGGTTGTGACAG
AACGTGCAGAGTTTTTTTTACAGTAAGCTGAGAACTATCATCTATGCACTCATTGCAGTGCGCTCATTGTCATGAGATCT
AATCAGATAGACCGGTTTGGACCATTACCCAATCATCACCTGGTCACTTAGCAGATCTTTCCCATAAACACGGACATGGA
TGCCAATAGTTTTGTGCTAGGCAATTCAGATTCCAACGAAGTTAACAAAATTGGGGTGTACCCTAAGCCAAAATATGTGG
ACATGTTTATTTTCCACTCTGCAGTGAGAGTATTTCTTACTCCCATGATTCCCTGACATAATTTTTACAGCTGATTAGCA
GTGAGGCTGCTGGGAAGAGTCAGTTTGGGAGGGTACTGAGGGAGAGGACAGCTGGAGTCAAGACGAGTCCACTTAGAATT
GAAGATGACTTCATAGTGGGGTGGGTTAGGATTAGGAATAATGCATTTAGGTTTGTTTTCTTCGCCACAGTTTCTCTGCT
GCAGAGAGGAAGCCAGATCTAGGATCAGGTTCTCCCTGTTCATTCAATGTTATTCATTATGATCTAAGAGGCAGCACTTA
GGCAAAATCAGCAATCCTACTCTGAAACACTTCCTGAATATGGCCACAGATGTTTAGTCATCTATGTCTAAGTTCTATTA
CATTATACAGGCAACATTTTGACCCTATAACTAAGTCTTCATCAGGACCAAATTTACAACATGAACTTGCTTCTTGGGAT
AGCTCAATTAGCAGAATAAGGATGTGTGCATACAGGTGGGCATACGGGTAGTGCCATCCCAAGGAGCACACGAATCCCCC
CCCCCCAACAATGGAGGCAGTGGCGTAATCTCTGAGTGTACAAAACATTAGGAGCACATTACTAATATTGAGTTACACCC
CTTTTGCCCTCAGAACAGGCTCAATTCGCCAGGGCTTGGACTCTACAAGGTGTCAAGCGTTCCACAGAGATGCTGGCCCA
TGTTGACTCCAATGCTTCCCACAGTTGTGTCAAGTTGGCTGGATGTTCCATTGTTGATACACACAGGAAACTTTTGAGTG
AAAAACCCAGCAGTGTTGCTCCTGGCAATTACTACCGTACCCTCTGAATGGCACGCACACAATCCATGTCTCAATTGT
CTTAAGGCTTAAAAATCCTCCTTTAACCCGTCTCCGCCCCTTCATCTACACTGATTGAAGTGGATTTAACAAGTGACGTC
AATAAGGGATCATAGCCTTCACCTGGATTCACCTAGTCAGTTTATGTCATGGAACGAGCAGGTGTTCCTAATGTTTTGTA
CACAGTGTATATCATATCAATATGTGCAACCCACTATCGAAGCTTATTGTGAAATAGACACTATTTACTTGTATTGCAAC
GTCATACAGAATTCAATATAATATAATTCAAATTGAGGAGGCAGTAATTGGTAGCACGCTAGACTTTGAAGTGACTTGAG
TGAATCTGTGAGGAACAGGAAGTTAGTGATGTGCAGGTCACAAATTATAATTTTTTGAACAACCTTTTTTACTGACTCGA
GAGATGTGTTCCTAATGTTTTGTGCACTCAGTGTACGTTTTAAATGAAGACTTTGCTTATTGAAATCAGCGGCACGCTTT
GCAAACCACACACTGATACGCACAGCCTTGTATTTACCCAGCCTTTTATTTATCCAAATCATTGGCACCTCGACATCACC
CCTCTTTCATTGATAGGAGTCTCATGCATGCAAATGATTCACCTTCTGTTCTCAGCCCTGAACCCAGTGACCCCACTAGC
ATGGTTCAATGTCAGACACAAGCTATTGTCTTGTCTTTTTCGCTCTCTTTCTGTGTGTGTGTGTGCACATTTCCATGAAT
AAGAACTTCCAGTTCAGCATGTGTGTGTATTGAGCAGAATTGCTACTCATTTTCATAGAGCCCAGGCCAGTGTGTAACAG
TCGGCCCTTGGCCCTGCTCTCTCCAGACAGGGCCTCAGTCACAAAGCACGCATTGTCCCAGTAAATGTGATAAGGCCTGC
AGCTGGGCCACCATGCAGTGTCCTGTTTCCTCTCTCTGTCTCTGTCTCCCCTCACACACTGTCACATCACAGAGCCTGAG
GTATGCACCATACTGACAGGGCAACCTCCAGGTATTATTTCACCCAGCCAACACTGAGATAAGTGCCCTGACTCTGACAA
ATCATCAGACCAAAGCAACTTCCATTCTGAGCCTTAGCATAGGGCTTATATGAGTCTGTTGTTGCAACTGTACTCTCACA
AAGGAAGCCGTATGGATGGTGATCACAGGTTGTTTCTGGCACTTTAATTGGGGAGGACGGGCTCGTGAAAATGGCTGGAG
CGGAATTTAGCTTGGTGGTGCTCTTTAACATACACTCTTAGAGGAAAAGCTGCTATCAAGAGCCTAAAAGGGTTCTTCGG
CTGTCCCCATAGGAGAACCCTTTTGGAACCCTTTTTTCTGAGTGTACTTAAGTCAATTTCTACAGTGAAGTGCCATAAAC
AGAAAAGAAGCTGTGCAAATCAGTTGATTGTCATCATGTCATATTATTGCATACAATCCATCCTGTTTGCTTGTTCAGCA
CATACATGTTCTGCTGTTTGTGTGTGTGCCTCAGTGGCTATCACTCAATATTTCCTGTAGTGATGGGAATTTCGACGCTT
TTTACTGACTCGGATCGTTTCGACTCGTTCAGTCAAAAGTATGAATCTTTCGACTCATTTCGTTGTTTTTATTTAGTAAT
GCCCAGAGCGCGCAGGGCCCCCTACCGGCCAACGATTAACTAAAAACTTGACCGGCTTATGATTCTACAAGCCTCTCGTT
CACCATAAGGGGCTTATTGGAGCTGTCATTTGTGACTGAGACTTACTAAAGTGTGCTTCAAACAATGTACATTTGTGATA
GCTAGGCATACAACCAGACTAGATTGTGAACGACTCTTTGCGGAATGACCTGCCTGCCTGCCGAGAGAGAAGCAGGCCCC
CAAACGATTCTTCCTCGATTTTGTTGAGTAGAGGCAGGCCGTTGATGCTTCTACAAAGCTGTGTAGCTTTTCAATAATCA
ATTAATGACATTCATTATTACACCGTGAGTTCAAATACCAGTTCTAAACCTGTATTCTTCTTCTCTATGCTGCAGCATGA
TCTGTTTGCGTGCGCACACATATGATAGACAGTTGTTGGTGGTAGCAGGTCTCTGGAACCGCTGAGCCGGAGAAACTGGT
ATTAATCCTGCTGTAGGATTCATCGTGGCCAGCAGAGCAGATCAAACGAACCACTAAAAAGGAACGAGTCACTCTGAAAA
ATGTATCATGACTCTCGAGTCAGGAAAAAAGGTTGTTAAAATAATAATCATTTGTGACCTTGCCCATTCAGAGGGTGAAT
GGGCAAGACAAAAGATTTAAGTGCTTTTGAACAGGGTGTGGTAGTAGTAGGTACCAGTCACACCGGTTTGAGTGTGTCAA
GAACTGCAACGCTGCTGGGTTTTTCACGCTCAACAGTTTCCCATGTGTATGAACAATGGTCCACGACCCAAAGGACACCC
AGCCAACTTGACACAACTGTGGGAAGCGTTGGAGTCAACATGAGCCAGGAAAGCTTTCAACACCTTGTAGAGTCCATGCC
CTGACAAATTGACGATGTTCTGAGGGCAACAGGGGGTGTAATATATTCCTAATATTTTGTTCACTCAGTGTATTTCCATT
TAGCGTATCTGAAATACCGTTGAGTTAGAAAGAGACAGGGAGATATGAGAAATTTAAATATCAGTAAGTAGGGCAAAATG
AAACTTCCTCAACAGAAACTCTCAATAGATGAAGACAGATTTGCATCAAAACTAGATTTTTTTTTTTTTTTTTTGCCAGC
TGAAAAATGGCAAGCAAAGCATGCCTGCAACATTGAACATGGTGTGAGTTGCTGGGTCCACAAACATAAGGCTCCTATTG
ACAATCTCCCTTGTTATCTGAACAGAAGGAGCCCTCTGTTCAATACTACATGGCAGGAACATGTTATTGTCATGCTAGTT
TTGTTAGCTAACAGCCCACCATAAGACACCATCTCAGTGCAAGAGGCGTCAATGCAGTACCTGGTTCGAATCCAGGCTGC
ATCACATCTGGCCGTGATTGGGAGTCCCATAGGGCGTCGTCTGGGTTTGGCCGGGGTAGACCGTCATTGTAAATAATAAT
TTGTTCTTAACTGACTTGCCTAGTTAAATAAAGGTTTAATCAAAAATAATAATGTGACAATGCTAACCACTGTCTAAATC
TGAGTCCATGTCTGGGGACGTCTAGAGAAGGTTGACACAATGGAAGAGTCCAGTCAACAAGGACTTTATGACAAAAGAAG
AAATACTCATTTTGATGGAACCAATTGAAAATTCTATTGAATAGTTTTGTATTGGTTTGTAACTTGAAATCAAGTGGAAA
CAGAGTGGACTATTCCCCAACCGATTCACATACAGTGGATGTGTCATGTGTGATATGTAAATGGTTATATAGCAGTCCTC
ACACAATATATCTGAGTGTATGTCAGTGTAATTTCTAGAGAATTATATCTAACAGACATTTTCTTCCCTTTCTGTCTCTG
CAGTGTTTCCTTTGTGGTGGGGGGGTGGGGTGGCCAACGGTGGTGGGGGCTGGGCCCAGGGCTGGGGCTGGGACCGCTGT

ATGCAATGGGAACCAGGGCTTGGAGAGGTGGCCCAGGCCCCAGCGTTTGACTGCCCGGTCTGGTGTGTCCTCTGCCAGGG
ATGCAGGGCTAGGCTCAGGGAGGCCTGCCAGAGAGGGAGGGGGGCCATCAGACTGCCCACAGACATGCTTAGCATCAAAC
TCCCCCAGCTCTTCGACATCCACCAAGTCCCCAAGGTAAGAGTGGCCAGACTGAAAGAGAAACCTTTCACAACCATTTCA
CAAACCTTCTGAGGTTTCACTGACTAAATGTTCATGTTTTCCTAAATATAGGATATGGTGTCGAGATCAGGATTGTGTCA
TATAGTTCCCAGAGGGCTGTTTGCGCTTATGTTCAACCCAGTCCTTGCTCCATCTTCACCCGGGTCCGTATTCACAAAGT
GTCTCAGAGTTGCAGTGCTGATATAGGATCAGTTTAGCCTATTAAATTATGAAGTGACGCCCGACAACCTGCCCGCTCGA
CCCCATCCCCTCCTCCCTTCCCCAGACCATCTCTGGAGACCTTCTCCCATTCCTCACTTCCCTCATCAACTCATCCCTGA
CCACTGGCCACGTCCCCTCCTCCCTTCCCCAGACCATCTCTGGAGACCTCCCATTCCTCACTTCCCTCATCAACTCATCC
CTGACCACTGCATCCCCTCCTCCCTTCCCCAGACCATCTCTGGAGACCTCCCATTCCTCACTTCCCTCATCAACTCATCC
CTGACCACTGGCCGTGTCCCCTTCTCCCTTCCCCAGACCATCTCTGGAGACCTTCTCCCCTTCCTCACTTCCCTCATCAA
CTCATCCCTGACCACTGGCTGTGTCCCCTCTGACTTCAAAATGGCCTGAGTCGCTCCCTTCCTCAAGAAACCAACACTCG
ACCCCTCTGATGTCAAGAACTGCAGACCTGTTTCCCTTCTTTCTTTTCTTTCTAAAACACTTGAGCGTGCTGTCTCTGAC
CAACTCTTTCACTATCTCTCTCAGAACCATCTTCTTTATCCTAACTAGTCAGGCTTCAAGACGGGTCACTCAACGGGTCA
CTCAACCAAGACTGCTTTCCGCACTGCCAAAGCTGACTTTCCCTCCTCTGTTCGCATCCTCCTAGATCTATCCACTGCCT
TTGACACCGTGAACCATCAGATCCTCCTCTCCACCCTCTCAGGGCTGGGCGTCTCAGGCTCTGCACACTCTTGGATTGCA
TCCTACCTGGCAGGCGGCTCCTACCAGGTGACTGTGTCTGCACCATGTACTCTCACTATTGGTGTCGCCCAGGGCTCGGT
TCTAGTCCCTCTCTTGTCTTCTCTCTATACACCAAATCACTTAGCAGATATCTCAGCTTGGATGTCGGCCCACCTCAAGC
TCATCCTCGACAAGACAGAGCTCCTCTTCCTCCAGGGGAAGGCCTGCCCCCGCTCCAAGACCAATCTATCACGGTTGACA
ACTCCATGGTGTCCCCCTCCTAGAGTGCAAAGAACCTTGGCAGAGACCTGTTGTTCTCTGCAAACATCAAAGCAGTGACT
TGCTCCTGCAGGTTCATGCTCTACAGCGTTAGTAGAGTATGACCCTATCTCACAAAGAAAGCGGTGCAGGTCCTAATCAG
TCTCCCGTCTGGACTACTGCAAATCGCTGTTGGTTGCACTCCCCGCTTGTGCCATAAAAAACCTGCAATTTATCCAGAAT
TCCGCAGCCCACACATTTTTTGTAAATAAAAAATTAAAGAAAGATTGCCTCTACTAGCACTGACTTTGCTGATAGCTACT
AAAAAAAGTACTTACTATGACTGAGATATGTGGTTCTCCCACCTAGCTATCTTACAATGAATGCTATCTTAAGATGAACG
CAACTGTAAGTCACACTGGATAAGAGTGTCTGCTAAATTACTAAAATGCCAATGTAAGTGTTGATCAGCATTGCTGCTCT
AAGACACTTTTTGAAATACAGGCCCAGATGTACCAGACTAGGAGGACAGGTCTTGATGTCCTCCAGTTGTTTGTTAGATG
ATCTCTATAGTAGCCTATGTACTGTGTATCACAGTGTTTTGCCTTCAGGTGTTCCGGGAGGATGGCATCATCTCGGGATA
CCGTCACCCCCAAAGCTCCGCCCTGGACTGCATCCTTAGCAGCTTCCAGATGACCAATGAGACAGTCAACATCTGGACCC
ACTTCCTGCCCACATGGTATGTACACCAATGCAAACACTCTGAATGAAGACAAGGAGATCAGATCAATAGATAGCGGGAG
GTATTCTGTAACAATTAGGGCTGGTTTAGACTGGAAAACATACGTATCAGATTGATGTCTTCCTGTCTTCGTATCAAATC
TTCCGATCACTCCCCCATTTCATTTCAAATGGAACTGACGGTGTTGCGACGCGTCTCATTTCCCAGGTACTTCCTGTGGC
GCTTCAGCGTCCTGTGCTCCACACTGAACTTCCTGTCGGAGAGCTACACCTGGCCGCTGCTGGTGTACATGCTGCTAATC
TGCGTGTACCCCTTCACCTCCAGCTGTGCCCACACCTTCAGCACCATGTCGCCCGAGGCACGCCACATCTGCTACTTCTT
CGACTACGGCGCACTCAGCCTCTACAGCCTGGGTAAGGCCCGCTGCATGAAGTCTGACTGTCTGCACTTCACAGTTAGAC
TATTAGTGCTTCCTTTCCCTTTAGTTATTTCTTTTTGGTTACATTTCCTCCAGTACCAAGACTGCCAACTCTCACAGTAG
GCTACTGCTTCCCGTAATCCAAACTGGTGCAATCGCACAGGGTACAGGCCACATGCAATCTTTCAAGTTCCAAAAAGTAT
CGGTGAACTTGGGCAATCAGTTGCATGATTAGAACTTTATATACTACTTCCCACATTTGTAACGACTGTTTGTGTGGTTA
CTTCATTGTTCGTGTATGTTTGATTTGGCGCTGTGTGTGTCATGTTGCTGTGCTGTCTGGGCTTGTCAGATTCACATTAA
TTAAAGTTTTTGGCCCACAAAACCTCATAATAATACATGTCTTGTTCAAGTATCAAATCAAATCAAATGTTATTGGTCAC
ATATACATATTTAGCAGATGTTATTGCGGGTGTAGGAAATGCTTGTTTTCCTAGCGTCAACAGTGCGGTGGTATCTAACA
ATTCACAATAATACACACAAATCTAAAAGTAAAAGAATGTAATTAAGAAATGTTAAAAAATTTGGACGAGCAATGTCGGA
ATGGCATTGACTATAATACAGTAGAATAGAATACAGTATATACAGTTGAAGTCGGAAGTTTACATACACCTTAGCCAAAT
ACATTTAAACTCAGTTTTTCACAATTCCTGACATTTAATCCTAGTAAAATTCCCTGTCTTAGGTCAGTTAGGATCACCAC
TTTATTTTAAGAATGTGAATTTTCAGAATAATTGTAGAGAGAATGATTTATTTCAGCTTTTATTTCTTTCATCACATTCC
CAGTGGGTCAGAGGTTTACATAACTCAATTAGTATTTGGTAGCATTGGCTTTATATTGTTTAACCTGAGTCAAACGTTTC
GGGTAGCCTTCCACAAGCTTCCCACAATACGTTGGGTGAATTTTTTGCCCATTCCTCCTGACAGAGCTGGTGTAACTGAG
TCAGGCTTGTAGGCCTCCTTGCTCACACACACTTTTTCAGTTCTGCCCACAAATGTTCTATAGGATTGAGGTCAGGGCTT
TCTGATGGCCACTCCAATACCTTGACTTTGTTGTCGTTAAGCCATTTTGCCATAACTTTGGAGGTATGCTTGGGGTCATT
GTCCATTTGGAAGACCCATTGGTGACCAAGCTTTAACTTCCTGACTGATGTAATGAGATGTTGCCTCAATTTATCCACAG
AATTTTCCCCCCTCATGATGCCATCTATTTTATGAAGTGCACCAGTCCCTCCTGCAGCAAAGCACCCCCACAACATGATG
CTGCCACCCCTGTGCTTCACGGTTGGGATGGTGTTCTTTGGCTTGCAAGCCTCCCCCTTTTTCTTCCAAACATAACGATG
GTCATTATGGCCAAACAGTTCTATTTTTGTTTCATCAGACCAGAGAACGTTTCTCCAAAAAGTACGATCTTTGTCCCCAT
GTGCAGTTGTAAAACCGTAGTCTGGCTTTTTTAATGGCGGTTTTGGAGCAGTGGCTTCTTCCTTGCTGAGCAGCCTTTCA
GGTTATGTCGATATAGGACTCGTTTTACTGTGGATATAGATACTTGTGTACCTGTTTCCTCCAGCATCTTCACAAGGTCC
TTTGCTGATGTTCTGGGATTGATTTGCACTTTCGCACCAAAGTACGTTCATGTCAAGGAGACAGAACGCGTCTCCTTCCT
GAGTGGTATGACGGCTGCGTGGTCCCATGATGTTTATACTTGCGTATTATTGTTTGTACAGATGAACGAGGTGCCTTCAG
TCATTTGGAGGTCTTGGCTGATTTCTTTTGATTTTCCCATGTCAAGCAAAGAGGCACTGAGTTTGAAGGTAGGCCTCGAA
ATACATCCACAGGTACACCTCCAATTGACTCAAATGATGTCAATTAGCCTATCAGAAGCTTCTAAAGCCAAGACATAATT
TTCTGGAATTTTCCAAGCTGTTTAAAGGCACAGTCAACTTAGTGTATGTAAACCTCTGACGCACTGGAATTGTGATAGAG
TGAATTATAAGTGAAATAATCTGTCTTTAAACAATTGTTGGAAAAATTACTTGTGTCATGCACAAAGTAGTTGTCCTAAC
CGACTTGCAAAAATTTTTTGTTTGTTAACAAGAAATTTGTGGAGTGGATGAAAAACGAGTTTTAATGGCTCCAAAATAAG
TGTATGTAAACTTCCGACTTCAACTGTACATATAAGCTGAGTAAAGCACTATGCAAATAAGTTCGACAGCTCAATACCAA
CAGTGAAATCAGAGTTTGTTTTCTTTCACAATTCTTTCACCTTGGAATAATGTTTGTCTTTTGTTTTAGACATCCAGCCT
GAGGGTTGCTCTTCCAACAGAGAAGCCATACAGGGCTACTTACAAACAGAAATCATGTCGCACCAGATTGCACTGTCAGA
TTTAGGTCAACAGACAGCATAGATTAAAATATCAGCCTATGTTTCCTCTCGGATCCAGCATNNNNNNNNNNNNNNNNNNN
NNNNNNNNNNNNNNNNNNNNNNNNNNNNNNNNNNNNNNNNNNNNNNNNNNNNNNNNNNNNNNNNNNNNNNNNNNNNNNNN
NNNNNNNNNNNNNNNNNNNNNNNNNNNNNNNNNNNNNNNNNNNNNNNNNNNNNNNNNNNNNNNNNNNNNNNNNNNNNNNN
NNNNNNNNNNNNNNNNNNNNNNNNNNNNNNNNNNNNNNNNNNNNNNNNNNNNNNNNNNNNNNNNNNNNNNNNNNNNNNNN
NNNNNNNNNNNNNNNNNNNNNNNNNNNNNNNNNNNNNNNNNNNNNNNNNNNNNNNNNNNNNNNNNNNNNNNNNNNNNNNN
NNNNNNNNNNNNNNNNNNNNNNNNNNNNNNNNNNNNNNNNNNNNNNNNNNNNNNNNNNNNNNNNNNNNNNNNNNNNNNNN

NNNNNNNNNNNNNNNNNNNNNNNNNNNNNNNNNNNNNNNNNNNNNNNNNNNNNNNNNNNNNNNNNNNNNNNNNNNNNNNN
NNNNNNNNNNNNNNNNNNNNNNNNNNNNNNNNNNNNNNNNNNNNNNNNNNNNNNNNNNNNNNNNNNNNNNNNNNNNNNNN
NNNNNNNNNNNNNNNNNNNNNNNNNNNNNNNNNNNNNNNNNNNNNNNNNNNNNNNNNNNNNNNNNNNNNNNNNNNNNNNN
NNNNNNNNNNNNNNNNNNNNNNNNNNNNNNNNNNNNNNNNNNNNNNNNNNNNNNNNNNNNNNNNNNNNNNNNNNNNNNNN
NNNNNNNNNNNNNNNNNNNNNNNNNNNNNNNNNNNNNNNNNNNNNNNNNNNNNNNNNNNNNNNNNNNNNNNNNNNNNNNN
NNNNNNNNNNNNNNNNNNNNNNNNNNNNNNNNNNNNNNNNNNNNNNNNNNNNNNNNNNNNNNNNNNNNNNNNNNNNNNNN
NNNNNNNNNNNNNNNNNNNNNNNNNNNNNNNNNNNNNNNNNNNNNNNNNNNNNNNNNNNNNNNNNNNNNNNNNNNNNNNN
NNNNNNNNNNNNNNNNNNNNNNNNNNNNNNNNNNNNNNNNNNNNNNNNNNNNNNNNNNNNNNNNNNNNNNNNNNNNNNNN
NNNNNNNNNNNNNNNNNNNNNNNNNNNNNNNNNNNNNNNNNNNNNNNNNNNNNNNNNNNNNNNNNNNNNNNNNNNNNNNN
NNNNNNNNNNNNNNNNNNNNNNNNNNNNNNNNNNNNNNNNNNNNNNNNNNNNNNNNNNNNNNNNNNNNNNNNNNNNNNNN
NNNNNNNNNNNNNNNNNNNNNNNNNNNNNNNNNNNNNNNNNNNNNNNNNNNNNNNNNNNNNNNNNNNNNNNNNNNNNNNN
NNNNNNNNNNNNNNNNNNNNNNNNNNNNNNNNNNNNNNNNNNNNNNNNNNNNNNNNNNNNNNNNNNNNNNNNNNNNNNNN
NNNNNNNNNNNNNNNNNNNNNNNNNNNNNNNNNNNNNNNNNNNNNNNNNNNNNNNNNNNNNNNNNNNNNNNNNNNNNNNN
NNNNNNNNNNNNNNNNNNNNNNNNNNNNNNNNNNNNNNNNNNNNNNNNNNNNNNNNNNNNNNNNNNNNNNNNNNNNNNNN
NNNNNNNNNNNNNNNNNNNNNNNNNNNNNNNNNNNNNNNNNNNNNNNNNNNNNNNNNNNNNNNNNNNNNNNNNNNNNNNN
NNNNNNNNNNNNNNNNNNNNNNNNNNNNNNNNNNNNNNNNNNNNNNNNNNNNNNNNNNNNNNNNNNNNNNNNNNNNNNNN
NNNNNNNNNNNNNNNNNNNNNNNNNNNNNNNNNNNNNNNNNNNNNNNNNNNNNNNNNNNNNNNNNNNNNNNNNNNNNNNN
NNNNNNNNNNNNNNNNNNNNNNNNNNNNNNNNNNNNNNNNNNNNNNNNNNNNNNNNNNNNNNNNNNNNNNNNNNNNNNNN
NNNNNNNNNNNNNNNNNNNNNNNNNNNNNNNNNNNNNNNNNNNNNNNNNNNNCCATTCTGGGAATTGCCTGTATATTT
ACATGATTATTTTTGTAAATGTTTTAGATGAGGAAAAATGATTAATTACATCCCCAACATCGTATAATCATTTCTTGACA
TAATAGCACTTACATGTACCACGCTAGACCTAACACACACGTCATTATTTTATCATGTAGCTCTGTATCCAGTATGTAGG
GGAGACCAGGGTTGGTTGTCACAGTGCTTATAACTCCCAAACTAGAGGGCGCTGTGTAATCATTTTAAGATTAAAATGTG
TGAATTCTTTGAAAGAACTAATCCACCTGGTCAATTTATGTCATCATAACAAAACATTGAGGTGAGGGGATTTTTTTCAT
AGGTGAAAGTTAAAAAAAAGAATAATAAAGAAATATGTCAGTATTCTTTTAAATAAATGTTTGAATTATGGGAGTATACA
TGTAAAAAAAATATTATAGGGAGAGCTACATTCCAAACCTATTTCTGAGTTCCTATGTCAAGCCATGTGGTAACTATCAT
CCTAATTAGCATTGGGTGCTGTTGCTATCGACCTCCCGTCCCAATAAACCTGAACACCTTTGGCCGGCATATAAAAATGT
GTTGGGAATAATATACAGTTTAAGTCGGAACTTTACATACACTTAGGTTGGAGTCATTAAAACTCGTTTTTCAGCCACTC
CACAAATTTCTTGTTAATCAAACTATAGTTTTGGCAAGTCGGTTAGGACATCTACTTTGTGCATGACACAAGTAATTTTT
CCAACAATTGTTTACAGACAGATTATTTCACTTATAATTCACTGTATCACAATTCCAGTGCGTCAGAAGTTTACATACAC
TAAGTTGACTGTGCCTTTAAACAGCTTGGAAAATTCCAGAAAATGATGTCCTGGCTTTAGAAGCTTCTGATAGGCTAATT
GACATCATTTGAGTCAATTGGAGATGTGGATGTATTTCAGGCCTACCTTCAAACTCAGTGCCTCTTTGCTTGACATCATG
GGAAAATCTAAAGAAATCAGCCAAGACCTCCAAATGACTGAAGGCACCTCGTTCATCTGCAAGTCTGGCTCATCCTTGGG
AGCAATTTCCAAACGCCCGAAGGTACCACGTTCATCTGTACAAACAATAGTACCCAAATATAAACACCATGGACCCCGC
AACCGCCATACCGCTCAGGAAGGAGACGCGTTCTGTCTCCTAGAGATGAACGTACTTTGGTGCAAAAAGTGCAAATCAAT
CCCAGAACAACAGCAAAGGACCTTGTGAAGATGCTGGAGGAAACGAGTACAAAAGTATCTATATCCACAGTAAAACGAAT
CCTATATCGACATAACCTGAAAGGCTGCTCAGCAAGGAAGAAGCCACTGCTCCAAAACCGCCATAAAAAAGCCAGACTAC
GGTTTGCAACTGCATATGGGGACAAAGTTCGTACTTTTTG

Text S2-4*B*

> Ssa1_scf_137: 428 kb scaffold sequence including *DFA*ψ/*DFB*ψ loci

TTGTTACCCCAACACTTTGATTATCGTCTGACCATGAATAAAACAACCATTTGGTTCAAGAGCTCAGTTTCCAATTGTCT
ACCATCTTACAGTACATGTTTTTATATTATTTCGGAAGGATGAACAAATACAGACTAATTGTACCTAAACGTTTTAGCCT
GCAAAAGCACCTTTCATTGTGTGGAGGTCCGTAATCAATTACCTGAATTACACACAGCAGTACTTTCTCACTGGAACTCT
CACCACACACATCAGTTACTGGAAGAATAAAGTTTTATATGACCTTAGGTCCCCCCCAACAAACAAACACACTTTGGATA
TATTACAGCAGCCTCTCTTCACCCCACACTTTTTTTTCTCCCTCCCTTTTCTCCATATCCACTCCCTTAGGAGTGCTAAT
GGGTTGTGAGTAATGTTATATCGCTGGCTCTCTCCCTCTCTTTAGTGTATTGGAAATGAAAACACTTGTTAGGCTCATGC
TGGACCACTGTTTCCTATGACGTCTTACAGGCAGAGACTGGACTATACCTTCAACCCCCTATAGAGATATACTTTCCACT
GCCCATCTCCTTCCAGGACTAGGGCCAAGTGAGTCTCCCTCATTATCACTCCTCCTTTTTAAGATACCCTAATACACACT
CTTTCCAAGGAAAAGAGAGCCTCTATTCCTCATCTGAGGTTTGAGAGTCCAATATGTCAGCATCTTCACCATTGACTGAG
AGAAAAAAATAACCTCAAATTGCCTCAAAACAAGATAAGCAGTGTTTTTTTGTTGTATGTTATCGCAATTTGGTAGAATC
TGTCAAAATAACTATATTTTCCACTCTTGTAGATGGGGTGGGCTCCATACTTGTTGGCTACACAGGCAAAAAAATAATCC
TTGTTCAATCATGCCACCTACTGCTCACATTCATACATAAACCAATAGTTTGTTCCCTCTTGCAGTAGAACATTGGTTGT
TTTACAGTAGATCATTGCTCAGTGAAAGGTTGTCATCTAGTGTATTGGAAAATAACTGCAGTAATCCCCTATACAGTCCA
ACATTTCAACTGATACAGTTGTTATACACAGTCGACAGACAAACACATTAATGCTGTCAAATATGTGGTAAGTAATTTTT
ATTAGAGAAATGATTCCAGTGATGAATACACTCTAATAAGGCAGCATTGACACAATTTTAGTAACAAGATAGCAAACAAT
GAAATATTTTTTTTCTTCAGTATTTTCCCCAGGAACTATATTAAGTTCTCCACACAAAACACAGCATATATGCTAAATTA
AAATAAAAGTTGAATTAAAGGTAGTATAAAAGGAAAATAGGCACAGACATTCATTCAAACCGCTTCTTCCCTCTTTGCTA
AAACCTAGCACTCGTCAGGAGTCACTGGAAGTTCCTTGGTGTGTGGTTTGTTGTGTCATCCAACCAAATCTTTACATAGC
TTCCTATTTTACTTTCTGTAACATGCCATTCAATATGAACACACTTTGGGAATCATCATATTAACTTTATGAAAGGATC

GACGTAAACAAACAGACGCATTTGAACTAATAGGTCATCACTTTTTTTTCAACGCTAATCTAGTTTGAACGAAATTAGAT
TTCAATGACAAAAGCATAACAGAATGTTTAGAAATGTAGTTGAACGTTCAAATATTCAACAACTTAAAGGACAACTGCAG
GTATATCAGCACTACAATATGTCAATTCAGATACAGTTATGGTCGATATGGTCGGCCACATGCTAGTCACAACCCACTTC
CAAGAAACAAGTTGTAAGATGATGAAAGCACCATACCAAAATCAACAATGGAAAACTGAGTGGTATAAGAATGATGAAAA
GGGCAAATATATAGGTCTCTCTGTGTTCAATACACTAACCCTCAAAACAAAAATTGGTTTTCAAACAATAGGGCTCTCGT
TGGAGTAAAATTAAGTGTGTGTATGTGTGTTTGCCTATACTGCCTGTACACTCTGCTCGACCTGCAACACAGTCCATTGC
CCTCACTGACACAATATCACTTTCTATTCCCTCATAACAACCCAGAGCCTTTAGGGGAGACTTGGGTTGGTTGTCACACG
TTTTACTCTGGTGTGTATTTCTCAGCACCAGTATATATAACAAGACTCTTTTCAACATTAACAGAAAGACCAAGGACTTG
GGTTGAAATAGGGATCCTATTAGTATGTCTTATTCCAACATGGGAGTAATAAGCCATCAAACACACTCTGCCCACTTGTG
ACAACCAGCCCCATCGCAGGGTTGGTTGTCACACAGTATAAGGTTCACAGGAGGTTGGTGGCACCTTAATTGGGGAGGAC
GGGCTCGTGGTAATGGCTGAAGTGGAATCAGTGGAATAATATCAAATATATCAAACAAATGGTTTCCATGTGGTTGATGC
CATTCCATTCGCTCTGCTTCACCCATTAGTTTGCGCCGTCCTCCCCTAGGCAGTCTCCACTGATGGGATTGGTTGTCACA
ATGTTGGCTAGTAGTTTATTCCTTTTGTAAAAGGAAATTAAGCAATATAGTTTACAGCGTCTTAGAAATAGCCTTTCTTT
TATCAAATGATATTCCTAACAAAATGCAGGATAACATATGACATTTTGAGTCAATTTGTGTGTGCGTGTGTTGCATACAC
GTGTACATTTGTTGGTGTGTGACAGAAAAAATATGTTTGGGAGAGAAAGGCAACAAACCCAGATAGCAGAGAAACCTCTG
GCCAATGGATGTACACATGATGAATCGGGAAGACGCAGTTTAGACATCGTTTGCTAATTGGCAAGACGTGTCACAGATGT
TTTTAGAATACCTCCATTCTAAATTCTACAGTTCTACATAGTATATATGTCGGCCAGGCATCAAGACTCTATTCTGATGT
CTCGGCGACATCTTCCCAATGTGCTAACGCTCTCCACCCTACATATATTATTCCCAACACATTTTGATATGCCTTGAAGG
AAGGTTTATAGCAACAGAACCCAATGCTAATTAAGATGCTAGTTACCACATGACTTGACCTAGGAACTCAGAAATAGGTT
TGGAATGTAGCTCTCCCTATAATATTTTTTTACATGTATACTTCCATAATTCACACATTTTTTTTTTTTTATTCCATCTT
AAAAAAAACAACTTTCACCTATGAATTAAAAAAATACAAAAATACCATCACCTAAATGTTTTGTTATGATGACATGAATC
GACCAGGTGGATTAGTTCTTTCAAAGAATTCACACATTTTAATCTTAAAATGATTACACAGCGCCCTCTAGTTTGGAAGT
TATAAAGCACTGACAACCAACCCTGGTCTCCCCTGCATACTGAATACAGAGCTACATGATAGAATAATGACATGTGTGTT
AGGTGTAGTGTGGTACATGTAAGTGCTATTATGTCAAAAAAATGATCATATGATGTTGGGGATGTAATTAATCATTTTTC
CTAATCTAAAACATTTACAAAAATAATCATGCAAATATACAGGTAATGCCCAAAATAAAGAAAACACTTCAGTAAATGAG
GGATACATAGTATATTGAAAGCATGTGCTTCCACACAGGTGTGGTTCCTGAGTTAAGCAATTAATATCCCATCATACTTA
GGGTCATGTATAAAAATGCCCTATTGCCCATTATTTTGGCTACCATGGCTAGAAGAGATCTCAGTGACTTTGAAAGAGAG
GTCTCAAAGGAGCACAGGGGGCTTAAAGGGTCTGTGTGTGTCTCTTAGTCACCACATTTCAACCCAACTGAACACTTATG
GGAGAATGTGGAGCGGCGCCTGAGACAGCGTTTCCACCCCTTCAACTAAACACCAAATTATGGAATTTCTCGTGGAAGA
ATGGTGTCGCATCCCTCCAACAGAGTTCCAGACACTTGTAGAACCTATGCCAAGGCATAGAAGCTGTTCTGGCTCGTGGT
GGCCCAACACCCTATTAAGACACTTTATGTTGGCGTTTCCTTTATTTTGGCAGTTACCTGTACATATACACTATGCTTTA
CAACTGAACAGAAACAGTAAGTGCTGTTATATATACTACAAGGATTAAATGCTCAAGAGATACCACACTCTTTTTCAAGG
ATACATGTAAACAATAGCCACTGACAATGTTATATCAGCATGGGGTGAGTGGTGTATTGGTTTGCGATAAACCTGCAATG
ACCACCACGATACCACATGAATACGACAGTGCCAAGACCCTTTGACCTCCACTGACCACATCCACATGCCTGGGAGATGA
GGAGACATCACTCATTCAGTTCTGTTGTGCACCATCATCTAAGATTTTTTCCATCAGATAGCTGTTATAAACAAGATCAT
TTAGTATGTCCTATTGATGTTACAATGATGGGTATGTTTTAGGGCGAATTTCAGTAATTCCCTGACATTTAAACAGCATG
TAGTGTTTCAAAAAATGTATCCAATGACCATTAATGTATGTTGTAGATGTAACAAATAAGAGTACTCCAAAGAACAATTA
AAGTGACTGAGCATTCACAGGAACACAAACTGCGAAGAACCATGGAGTTTCCATGGAAACGTACCTCATGTAAAATGGCA
CCTGTCTTTTAATCATAGACATGGTACAGTGCTGTAGCTTTCAGGTACACGCTTACAAAAAAGGGTTTCAAAAGGGTCCT
TTGGCTGTCCCCATAGGAGAAACCTTTTTGGTTCCAGACAGAAATATTTTGGGTTCCATGTAGAACCCTCTGTGGAAAGG
GTTTTACAAGGAACTCAAAAGGGTTCTACCTGGAACCAAAAAGGGTTCTTTAAAGGGTTCTCCCTACGGGGACAGCCGAA
GAACCCTTTTAGGTTCTAGAAAGCACCTTTTTTTCTGAGAGTGTAGACGTTTAGAGCAGGCAATCTGAGAGAGCACTACA
CTGGTCTGTAAATGTATTCAAGTCATAGTGGAGATTTAAATGAGATGGTATTGAATATTAAAACAAAATGATGGAATCAT
ACTAAGTAGTCCAGTTAGTGATGAGAATCACGTAACGGATAGTCATACTTTATATTAAATATGGGTTTTGAGATGTTCTA
ATAATGGCTTACAAATACATAGGACAATTGAAAAACAGTAGCTAAATACTTATATATTAATATGAAAATTCCCTGCAAAT
GTACAGTTAAAGATGTTTGGGATTTACACAAACCAGAAATAATGTTACGAGTACTTAGGTAACATTTGGGTCACTATTTA
TAAAAGAAGGCACTTTTATGTGTTGTTAGACATCACATATGCTTCATACAACCCAGATCCTTTCAAGTATAAGTGAAAAG
AAGTCGGCATATGATACTTTATCTCAAAAAACCTCCACAACTTTAAACAGTAAACAATGATACTGAATAACTGAAAGGCT
TCATACAAATAGCTGGGTATTCGGCTTCAAAGTAAACAGTGAACACATTAATAAACTCCTAAACAAACCTTATAATGACG
TTAGAATAACATGTAGAAAAACCATCCTGTGATAATAATCCAATGGGTTTATACAAATACTACCTGGTTTGAAAAGGTAT
AATACAACTTCTTATGGTTATTACTTCCAAAGTTCACAACTGGGAGAATGGGTTTAGCCAACCCCTTTTCATTTGAACATA
TTGGTTTGGTCACTGGTTGTGGGGAAGTGNNNNNNNNNNNNNNNNNNNNNNNNNNNNNNNNNNNNNNNNNNNNNNNNNNN
NNNNNNNNNNNNNNNNNNNNNNNNNNNNNNNNNNNNNNNNNNNNNNNNNNNNNNNNNNNNNNNNNNNNNNNNNNNNNNNN
NNNNNNNNNNNNNNNNNNNNNNNNNNNNNNNNNNNNNNNNNNNNNNNNNNNNNNNNNNNNNNNNNNNNNNNNNNNNNNNN
NNNNNNNNNNNNNNNNNNNNNNNNNNNNNNNNNNNNNNNNNNNNNNNNNNNNNNNNNNNNNNNNNNNNNNNNNNNNNNNN
NNNNNNNNNNNNNNNNNNNNNNNNNNNNNNNNNNNNNNNNNNNNNNNNNNNNNNNNNNNNNNNNNNNNNNNNNNNNNNNN
NNNNNNNNNNNNNNNNNNNNNNNNNNNNNNNNNNNNNNNNNNNNNNNNNNNNNNNNNNNNNNNNNNNNNNNNNNNNNNNN
NNNNNNNNNNNNNNNNNNNNNNNNNNNNNNNNNNNNNNNNNNNNNNNNNNNNNNNNNNNNNNNNNNNNNNNNNNNNNNNN
NNNNNNNNNNNNNNNNNNNNNNNNNNNNNNNNNNNNNNNNNNNNNNNNNNNNNNNNNNNNNNNNNNNNNNNNNNNNNNNN
NNNNNNNNNNNNNNNNNNNNNNNNNNNNNNNNNNNNNNNNNNNNNNNNNNNNNNNNNNNNNNNNNNNNNNNNNNNNNNNN
NNNNNNNNNNNNNNNNNNNNNNNNNNNNNNNNNNNNNNNNNNNNNNNNNNNNNNNNNNNNNNNNNNNNNNNNNNNNNNNN
NNNNNNNNNNNNNNNNNNNNNNNNNNNNNNNNNNNNNNNNNNNNNNNNNNNNNNNNNNNNNNNNNNNNNNNNNNNNNNNN
NNNNNNNNNNNNNNNNNNNNNNNNNNNNNNNNNNNNNNNNNNNNNNNNNNNNNNNNNNNNNNNNNNNNNNNNNNNNNNNN
NNNNNNNNNNNNNNNNNNNNNNNNNNNNNNNNNNNNNNNNNNNNNNNNNNNNNNNNNNNNNNNNNNNNNNNNNNNNNNNN
NNNNNNNNNNNNNNNNNNNTCTGGAAGTGGGTACCCACCACGGCACAGATGTGGAAAAGCTGGTGACTGTGGCCTGAGAG
AGGAGAGAGAGAGAAGGTGAGAGAGATCAATGAGAGAGAGAGTCATTAAAAATGCTTGAATTTGATCATACTGCAGTGAACA
AGGATTTACCAGGTTTGCTCAAAGAATTGTCACTGTTCAAATATTTGCCTACAGTGCCATCACGGACACAGTAGAAATGA
CACTGATATGCGCCAACAATAACTGGCCCCAGGCCAGACTAGCTAAAGCTTGCTCAATAACTGATATAATCTCCCAATTC
CAGTTGCCATGGTAACCGGGCGTTGGGGTGGACGTACCGAAGTAGTCGAAGCGCCCCGGGGCCAGCCTCTCTGGGAGGTG

TGAGGCAAACAGGAAGCAGGTGAGGAAGGCGAAGAGGAGGTGGTAACAGTGACTGGACAGGGCCTCACTGTGACAGCAGC
TGCCCCCGCAGCACAGCAGCAGCTACACACACACACACACACACACACACACACACACACACACACACACACACACACAC
ACACACACACACACACACACACACACACACACACACACATATACAAACCCACATAAGATCATTGGCACAAACAGCGGATGAA
GGGATTAAAAGCTTTAACAGCATGTGGTCAAATCTTTATTTATTCTACACAAAATCAGTTTCTAAGTTAATGAGCCAAAA
GTCCTGGTCAATGTATTAAAGAGAGGCATTAATCGGATCTAAACTGTGACATAACAAACAGAATACAACCCAATGACTGT
ACAAGCAGACTTTCTGTGAACTCCGTCTACACCTTCTGAAAATACACTCACTCTGTAAAATAGAGGGATGTTGTCAAAGA
TGAATGGAAAGACAAATGCCCCTGTCCGGAGAACTTTACTGCGGTGTGGAAACTGCATCTCCAGGAATCTATAAAAAAAA
GGGAGGGAGGAAAGTTACAATGGTTTCAACGACCAGGAGAGTGATGCATCATGGGTAGGATACCGTATTGGCTGCCTACC
TAGAATAGCAGGACAGGCTGGTGCAGAATACTGTGTTGCAGATGGCGATGGGCACAAAGTGTTGGTGCAGCCAGCTGTTG
ACCCAGCAGTCAGGCATCACATATGACCCATAGGTTATGGCACAACCTGAACACAAACACACAAAAACACTGAAAGGTTA
AAAAAAAACATACGATATCTGAAACTAAATCTACCATGCATTTACCCACCATACACTCACCAAGCACTTAAAATCATTAG
AAATGGCAACGCTCATAACATATCAATTAGGCTTTAATAACCTACTAATGAAACATTGGCCTTGCACTTGCTGTATACAT
GCTTCAACACCAGTTTTAATCCCAATGATGTACTTCCAATAAATCTAGAGGCTGTTTTAAATAAGCTTGCCTGCAGAACA
CCATCTACAGTAAACATTTGCAATTCTTATCCCAGAAACGTCTGGACATTTAGGCTGGGTATGTTTTGGCAGTAAGGAGA
ATTGTGGTAGATTAAGGATCATTTACATTTACGTCATTTAGCAGACGCTCTTATCCAGAGCGACTTACAAATTGGTGCAT
TCACCTTATGATATCCAGTGGAACAACCACTTTACAATAGTACATCTATATCTTTTTTTTCTCTTCTTTTTTTTTTGCAG
GGGCCTCATATCCATTAATATGGATTTGAAAAGGCACGTTTTCAAAGTAAGAGACAAAGTTTATGTTTGAAAGAGAGGGA
CAAGTCTATCACACACACCCATACAAGCAAACACACACCCAAGAAAAGCACATGTATTCACAAGCAGAATGCAAATACCC
AAGTATGTTAATTAGCTGAGCAAGCACGTGGACACAGACATGAATCCATGGAAAATGTACATGTGAAGGTGTAGAATAGT
GGTATGCACACATCCATCAAAACTTTTCCCAGATGCTGGATCCGAGAGGAAAACATATGCTGATATTTAATCTATGCTGT
CTGTTGACCTAAATCTGACAGTGCAATCTGGTGCGACATGCTTTCTGTTTGTAAGTAGACCTGTATGGCTTGATGAGCAA
CCCTCAGGCTGGATGTCTTAAATGAAAGGCAAACATTATTCCAAGGTGAAAGAATTGTAAAAGAAAACAAACTCTGATTT
CACTGTTGATATTGAGCTGTCGAACTTGTTCACATACTGCTTTACTCTGCTCATATGTATATACTGTATTCTATGCTACT
GTATTTTAGTCAATGCCCCTCCGACATTGCTCGTCCTAATTTCTTAATATTTCTTAATTCCATTCTTTGACTTTTAGAGT
GTATTGTTGTGAATTGTTAGATACTACCGCACTGTTGGAGCTAGGAACACAAGCATTTCGCTACACCCGCAATAACATCT
GCTAAATACGTGTATGTGACCAATAACATTTGATTTGATTTGATACTTGAACAGGACATGTATTATTATGAGGTTTTGTG
GGCCAAAAAAAACTTAATTACAGTGAATCTGACAAGCCCAGACAGCACAGAAACACAACACACACAGCGCCAAATCAAACA
TACACAAACAATGAAGTAACCACACAAACAATGAAGTAACCACACAAACAATGGTTACAAATGTGGGAAGAAGTATATAA
AGTTCTAATCGTGCAACTGATTGCCCAAGTTCGACGAGACTTTTTGGAACTTGAAAGATTGCACGTGGCCTTTACCCTGT
GGGATTGCACCAGGTCGGATTAGTGGAAGCAGTAGCCTACTGTGAGAGTAGGCAGACTTGGTACTGGAGGAAAAGTAACC
AGAAAAAAAATAACTAAAGGCAAAGGAAACACTAATAGTCTAACTGTGAAGTGCAGACAGTCAGACTTCATGCAGCGGGCC
TTACCCAGGCTGTAGAGGCTGAGTGCGCCGTAGTCGAAGAAGTAGCAGATGTGGCGTGCCTCGGGGGACATGGTGCTGAA
GGTGTGGGCACAGCTGGAGGTGAAGGGGTACACGCAGATCAGCAGCATGTACACCAGCAGCGGCCAGGTGTAGCTCTCCG
ACAGGAAGTTCAGTGTAGAGCACAGGACGCTGAAGCGCCACAGGAAGTACCTGGGAAATGAGATGCGTCGCAACACCGTC
AGTTCCAATTCATATGAAATGGGGGAGTGATCGGAAGATTTGATACGAAGACAGGAAGACACCAATCTGATGCGTATGCT
TTCCAGTCTAAACCAGCCCTAATTGTTACAGAATACCTCCCGCTATCTATGGATCTGATCTCCTTGTCTACATTCAGAGT
GTTTGCATTGGTGTACATACCATGTGGGCAGGAAGTGGGTCCAGATGTTGACTGTCTCATTGGTCATCTGGAAGCTGCTA
AGGATGCAGTCCAGGGCGGAGCTTTGGGGGTGACGGTATCCCGAGATGATGCCATCCTCCCGGAACACCTGAAGGCAAAA
CACTGTGATACACAGTACATAGGCTACTATAGAGATCATCTAACAAACAACTGGAGAACACCAAGACCTGTCCTCCTAGT
CTGGTACATCTGGGCCTGTATTTCAAAAAGTGTATTTGAGCAGGAGTGCTGATCAACACTTGTATTGGCATTTTAGTAAT
TTAGCAGACACTCTTATCCAGTGTGACTTACAGGTGCATTCATCTTAAGATAGCTAGGTGGGACAACCACATATCTCAGT
CATAGTAGGTACCTTTTTTAGTAGCTAACAGCAAAGTCAGTGCAAGTAGGGGGAAAAAGTCAAGTGCGAGTTTTAGTTCA
CGAAAGGCAATCTTTATTTTATTTTTTATTTACAAAAATGGGTTAAAGATTCAGTCTGTGAAACGTCTGAAGGTTTGTGA
AATGGTTGCGAAAGGTTTCTCTTTCAGTATGGTCACTCTTACCTTGGGGACTTGGTGGATGTCGAAGAGCTGGGGGAGTT
TGATGCTAAGCATGTCTGTGGGCAGTCTGATGGCTCCCCTCCCTCTTTGGCAGGCCTCCCTGAGCCTGGCCCTGCATCCC
CGGCAGAGGACACACCAGACTGGGCAGTCAAACGCTGGGGCCTGGGCCCCCTCTCCAGGCCCTGGTTCCCATTGCATACA
GCGGTCCCAACCCCAGCCTTGGCCCCAGCCCTGGGCCCAGCCCCCACCACCGCTGGCTACCCCACCCCCCCACCACAAAG
GAAACACTGCAGAGAGAGAAAGGGAAGAAAATGGCTGTTAGATATAATGCTCTAGAAATTACACTGACATACACTCAGAT
ATATTGTGTGAGGACTGTTACATACCCATCAGAACTAGAAATGTGTTTACATATCACACATGACACATCCACTGTATGTG
AATTGGTTGGGGAATAGTCCACTCTGTTTCCACTTGATTTCAAATTACAAACCAATACAAAAACATTCCATAGAATTTTC
TCATGGTTCCATCAAAATGAGTATTCCTTCTTTTGTCATAATGTCCTTTTTGACTGGACTCAACACAGTCTTCCATTGTG
TCAACATTCTCTAGACGTCCCCAGACATGGACTCAGATTTAGACAGTGGTTAGCATTGTCACAATATGGTGGGTTGTTAG
CTAACAAAGCTAGCATGACAATAACATGTTCCTGCTATGTAGTATTGAACAGAGGACTCCTTCTGTTCAGATAGCAAGGC
AGATTGTCAATAAGAGCCTTTTGTTTATGAAGCCAGCAACCCACACCATGTTCAATATTGCAGGCATGCTTTGCTTGCCA
TTTATCAGCTGGCAAAACAACAAAAAACGCGTGTTGGTTTGATGCAAATCTGTCATCATCTATTGAGAGTTTCTGTTGAG
GAAGTTTCATTTTGCCCTACTTACTGATATTTAAATCACTCATCATCTCTCACTGTCTCTTTATAACTGAAAGGTATTTC
AGATACCCTTTATGGAAATACACTGAGAGTACAAAATATTTGGAACACCTTCCTAAAATTGAGTTGCACCCGCTTTTGCC
CTCAGAACAGCCTCAATTCGTCGGGGCATGGACTCTACAAGGTGTCGAAAGCATTCCACAGGGATGCTGGCCCATGTTGA
CTACAATGCTTCCCACAGTTGTGTCAGGTTGGCTGGATGTCCTTTGGGTCGTAGACCATTCTTGATACACACGGGAAACT
GTTGAGCGTGAAAAACCCAGCAGCAAATCGGTGCGCCTGGTACCTACTACCACACCCTGCTCAAAAGCACTTAAATATTT
TGTCTTGCCCATTCACCCTCTGAATGGCACACATACACAATCCATGTCTCAATTGTCTCGAGGCTTAAAAATCCTTCTTT
AACCCGTCTCCTCTCCTTCATCTACACTGACATCAAGTGACATCAATAAGGGATCACAGCTTTCACCTGGATTCACCTGG
TCAGTCTATGTCATGGAAAGAGCAGGTGTTCCTAATGTTTTGTAAACAGTGTATATCATATCAATGTGTGCAAATCACTA
TCAAAGCTTACTGTGAAATAGACACAAATGACTTGGGTTGCAAAGTCATACCGAATTCAATATAATATAATTCAAATGGA
AGAGGCAGTAATTGGGAGCACGCTAGACTTTGAAGTGACTGATGTGAATCTGTGAGGAACAGGAAATTCGTGATGTGCAG
TTCACAAATTATTTTTTAACAACCTTTCCAACTGACTCGAGAGTCATGATACATTTTTCAGAGTGACTCGTTCCTTTTAG
TCGTTCGTTTGACCTGCTCTGCTGGCCACGATGAATCCTACAGCAGGATTCATACGCATTTCTCCGGCTCAGCGGTTCCA
GAGGCGTGCTCCGACCACCAGCTGTCTGTCATATGTGCGTGCGGGAAAATAGATCATGCTGCATGTGCAGCATAGAGAAG
AATAATCCATGTTTAGAACTGGTATTTGATCTCATGGTGTAAGAATGAATGTAATTAGTTGATTTTTGAAAACTACACAT

CTTTGAAGAAGCCTAAACAAGCCTGCCTCTGCTCAACAAAATCAGTTCGTGAACAGCAGTTCGTGAACGATATCTTCCTC
TCCGCAGTCAAGTCATTCCGCAAAGAGTCGTTCACAATCTAGTCTGGTTGTATGCCTAGCTATCACAAATGTACATTGTT
TGAAGCACACTTTAGTAAGTCTCAGTCACAAATGACAGCTCCAATAAGCCCCCTGTGGTGAACGAGAGGCTTGTAGAATC
ATGAGCCTTTCAAGTTTTTAGTTAATCGTTCGCCGGTAGGGGGTCCTGCGCGCTCTGGGTATTACTGAATCAAATGAACG
AAATGAGTCGAAAGATTCATACTTCTGACTGAACGAGTCGAAACGATTTGAGTCAGTAAAAAGAGTCAAACTTCCCATCA
ATACAGGAAATACTGTGTGATAGCCACTGAGGCACACACAAACAGCAGAGCATGTATGTGCTGAACAAGCAAACAGGATG
GATTAACATGCCAGGAGAGGAAGGATCATGATGTCTACCTGGAACCAAAAAGGGTTCTTCAAAGGGTTCTCCTATAGGGA
AAGCCGAAGAACCCTTTTAGGTTCTTGATAGCACCTTTTTTTCTAAGAGAGTATGTTAAAGAGCACCATCAAGCTAAATT
CCGCTCCAGCCATTATCACGAGCCCGTCCTCCCCAATTAAGGTACCAGAAACCTCCTGTGGCCACCATCCATACGGTTTC
CTTTGTGAGAGTACAGCTGCTACAACAGCCTCATATAAGTCCTATCCTAAGGCTCAGAACGGAAGTTGCTCTGGTCTGAT
GATATGTCATAGTCAGAGCACTTATCTCAGTGTTGGCTGGGTGAAATATTACCTGGAGGTTGCCCTGTCATTATGGTGCC
TACCTCAGGCTCTGTGGTGTGACAGTGTGTGAGGGGAGACAGAGACAGGGGACAGGACATTGCATGGTGGCCCAGCTGCA
GGCCTTATCCTATTTACTGGGACAAAGTGTGCTTTGTGACTGAGGCCCTGTCTGGAGAGAGCAGGACCAAGAGCCGACTG
TTACACACTGGCCTGGGCTCTATGAAAATGACTAACCATTCTGCTCAACACACACACACAATATTATATTGAATTCTGTA
TGACATTGCAATCCAAGTAATTACTGTATATTTCACAATAAGCTTTGATAGTGGGTTGCACATATTGATACACTGAGTGT
ACAAAACACTAGGAACACCTGTTCTTTCCATGACATAGACTGACATATTGATGTCANNNNNNNNNNNNNNNNNNNNNNNN
NNNNNNNNNNNNNNNNNNNNNNNNNNNNNNNNNNNNNNNNNNNNNNNNNNNNNNNNNNNNNNNNNNNNNNNNNNNNNNNN
NNNNNNNNNNNNNNNNNNNNNNNNNNNNNNNNNNNNNNNNNNNNNNNNNNNNNNNNNNNNNNNNNNNNNNNNNNNNNNNN
NNNNNNNNNNNNNNNNNNNNNNNNNNNNNNNNNNNNNNNNNNNNNNNNNNNNNNNNNNNNNNNNNNNNNNNNNNNNNNNN
NNNNNNNNNNNNNNNNNNNNNNNNNNNNNNNNNNNNNNNNNNNNNNNNNNNNNNNNNNNNNAGATCATAATGAATACCAT
GAAAGAACAGGGAGGACCTGATCCTTGATCCGACCGTAAATACGGTCCCTGGCTTTTTTTATGCAGCAGAGAAACTGTGG
TAAAGAAACAAGCCTAAAAAATTATTCTTAATCCTAACCCACCCCACTATGAGGTCATCTTTAATTCTAAGTGGACTCAT
CTTGACTCCAACTGTCCTCTCCCTCAGTACTCTCCCAAACTGACTCTTCCCAGCAGCCTCATTGCTAAACAGCTGTAAAA
ATGTCAGGTGATCATGTGAGTAAGAAATACTCTCACTGCAGAGTGGAAAATAAACATGTCCACATATTTTGGCTTTGGGT
ACGCCCCAATTTTGTTAACTTCGTTGGAATCTTAATTGTCTAGCACAAACTATGTCCGTGTTTATGGGAAAGATCTGGTA
AGTGACCAGGTGATGATTGGGTAATGGTCCAAACCGGTCTATCTGATTAGATCTCATGACAATGAGCGCACTGCAATGAG
TGCATAGATGATAGTTCTCAGCTGACTTTTAAAAGAACTGCACGTTCTATCACAACCAGTAAAACTACTTATGAATAACA
CAGATTTATAGCCTAGAATCTAAAACAATGGTGAAACTGAGCATATCAGTTTGGATTCCAGGGTGTGTCTAACTGTGTGC
GGTCCTTTCCCATTCAGATTTCGTTCTCCATCCACAGTTGGTTGGGGAGGACGGGCTCGTGGTAATGGCTGGAGCGGAAT
CAGCGGAATGGTATCAAACACATGGCTTCTATGCATTTGATGCCATTCCATTTTTACTCCATTCCAGACATTATTAGAAG
CCATTATCCCCTCAGCAGCCTCCACTGTCTCCATCAGTAGTAGAATGGGAATTCTGTTTGTCTATTTCTGGTATAGTCGA
ATAACGGGAGGTCTTGGAGGTGTTGCTCATGGGCCTCTGTGATAACAACCACTTCTCATGTGCTCACTACCACCCACTCT
CAACTACACCACCCACTCACAAGGTTATTCTGCACAGTAGCAACACATTTCTCTTCAGAATAATTGATTGGCAAGGACTT
GATCATTTGTGACTTTCTATCTACAATACTATACAATTGTGCAAAGTAGTAACAAGAAAGTGAGAAGTAGGTTAAACCCT
CTGTCAGAGGTGTTTGTTTAAGTGTCAGTGAGTGTCTGTCTTTGTTTGTTTGTGTGCAAGAGCATGGTAATGGTTACCAC
ATACAGCAAACAAGGGGAAAACAAACACTCTAAACAGTGACTGCCAGTGGCGGTGACCATCCACAGTTCCCCTACCAACA
TGTTTCACAATCTACAAATATTCACATTATTCGCTCCTTCCTCCCTCTCTCCTCTTCACCTCCCCTTTACCCCTCTCCAC
CTCTTTTGAATATGCAGCTTCTCTCCCACTTTTCCCTCTTTCTGTTCCCACTCTCTCGCCCAACTCCTGAATTCTGAAAT
TCTTTCCCTCGCTCGTGATCTCGTCACATGACCTTCCTCAATAGAAGACTTTATAACTCAAGAGCTGACGATAAGAAGCC
TATGTGGGGAGTATGTTGACATGGGCACGTGTCAGTTTTATGTAAACAAGAACAAGGCACATCGTTCGCCCCATAGAACA
TTGCATTGTCATCTAGCTGCATGATTCTGTACAGTTTGTACTGGGAATGGAACATGGAAGACATCACCTCGACACACACA
AACAATATGACTGACGGTCAAGTGTACCCTGAACAACAGGATTCACCGGTTTCTGTCCGTACTGTACTGCAGAAGGTATA
AATCCATCAAATAAATCTGTGTATAGCTGAACTAACTATCAGACCCTCCCACTGACCTAGATGGAGTACTCATTAACACT
GTCTGTTGAGGACTTTTAATTCCACTATGCCACTATGNNNNNNNNNNNNNNNNNNNNNNNNNNNNNNNNNNNNNNNNNNN
NNNNNNNNNNNNNNNNNNNNNNNNNNNNNNNNNNNNNNNNNNNNNNNNNNNNNNNNNNNNNNNNNNNNNNNNNNNNNNNN
NNNNNNNNNNNNNNNNNNNNNNNNNNNNNNNNNNNNNNNNNNNNNNNNNNNNNNNNNNNNNNNNNNNNNNNNNNNNNNNN
NNNNNNNNNNNNNNNNNNNNNNNNNNNNNNNNNNNNNNNNNNNNNNNNNNNNNNNNNNNNNNNNNNNNNNNNNNNNNNNN
NNNNNNNNNNNNNNNNNNNNNNNNNNNNNNNNNNNNNNNNNNNNNNNNNNNNNNNNNNNNNNNNNNNNNNNNNNNNNNNN
NNNNNNNNNNNNNNNNNNNNNNNNNNNNNNNNNNNNNNNNNNNNNNNNNNNNNNNNNNNNNNNNNNNNNNNNNNNNNNNN
NNNNNNNNNNNNNNNNNNNNNNNNNNNNNNNNNNNNNNNNNNNNNNNNNNNNNNNNNNNNNNNNNNNNNNNNNNNNNNNN
NNNNNNNNNNNNNNNNNNNNNNNNNNNNNNNNNNNNNNNNNNNNNNNNNNNNNNNNNNNNNNNNNNNNNNNNNNNNNNNN
NNNNNNNNNNNNNNNNNNNNNNNNNNNNNNNNNNNNNNNNNNNNNNNNNNNNNNNNNNNNNNNNNNNNNNNNNNNNNNNN
NNNNNNNNNNNNNNNNNNNNNNNNNNNNNNNNNNNNNNNNNNNNNNNNNNNNNNNNNNNNNNNNNNNNNNNNNNNNNNNN
NNNNNNNNNNNNNNNNNNNNNNNNNNNNNNNNNNNNNNNNNNNNNNNNNNNNNNNNNNNNNNNNNNNNNNNNNNNNNNNN
NNNNNNNNNNNNNNNNNNNNNNNNNNNNNNNNNNNNNNNNNNNNNNNNNNNNNNNNNNNNNNNNNNNNNNNNNNNNNNNN
NNNNNNNNNNNNNNNNNNNNNNNNNNNNNNNNNNNNNNNNNNNNNNNNNNNNNNNNNNNNNNNNNNNNNNNNNNNNNNNN
NNNNNNNNNNNNNNNNNNNNNNNNNNNNNNNNNNNNNNNNNNNNNNNNNNNNNNNNNNNNNNNNNNNNNNNNNNNNNNNN
NNNNNNNNNNNNNNNNNNNNNNNNNNNNNNNNNNNNNNNNNNNNNNNNNNNNNNNNNNNNNNNNNNNNNNNNNNNNNNNN
NNNNNNNNNNNNNNNNNNNNNNNNNNNNNNNNNNNNNNNNNNNNNNNNNNNNNNNNNNNNNNNNNNNNNNNNNNNNNNNN
NNNNNNNNNNNNNNNNNNNNNNNNNNNNNNNNNNNNNNNNNNNNNNNNNNNNNNNNNNNNNNNNNNNNNNNNNNNNNNNN
NNNNNNNNNNNNNNNNNNNNNNNNNNNNNNNNNNNNNNNNNNNNNNNNNNNNNNNNNNNNNNNNNNNNNNNNNNNNNNNN
NNNNNNNNNNNNNNNNNNNNNNNNNNNNNNNNNNNNNNNNNNNNNNNNNNNNNNNNNNNNNNNNNNNNNNNNNNNNNNNN
NNNNNNNNNNNNNNNNNNNNNNNNNNNNNNNNNNNNNNNNNNNNNNNNNNNNNNNNNNNNNNNNNNNNNNNNNNNNNNNN
NNNNNNNNNNNNNNNNNNNNNNNNNNNNNNNNNNNNNNNNNNNNNNNNNNNNNNNNNNNNNNNNNNNNNNNNNNNNNNNN
NNNNNNNNNNNNNNNNNNNNNNNNNNNNNNNNNNNNNNNNNNNNNNNNNNNNNNNNNNNNNNNNNNNNNNNNNNNNNNNN
NNNNNNNNNNNNNNNNNNNNNNNNNNNNNNNNNNNNNNNNNNNNNNNNNNNNNNNNNNNNNNNNNNNNNNNNNNNNNNNN
NNNNNNNNNNNNNNNNNNNNNNNNNNNNNNNNNNNNNNNNNNNNNNNNNNNNNNNNNNNNNNNNNNNNNNNNNNNNNNNN
NNNNNNNNNNNNNNNNNNNNNNNNNNNNNNNNNNNNNNNNNNNNNNNNNNNNNNNNNNNNNNNNNNNNNNNNNNNNNNNN
NNNNNNNNNNNNNNNNNNNNNNNNNNNNNNNNNNNNNNNNNNNNNNNNNNNNNNNNNNNNNNNNNNNNNNNNNNNNNNNN

```
NNNNNNNNNNNNNNNNNNNNNNNNNNNNNNNNNNNNNNNNNNNNNNNNNNNNNNNNNNNNNNNNNNNNNNNNNNNNNNNN
NNNNNNNNNNNNNNNNNNNNNNNNNNNNNNNNNNNNNNNNNNNNNNNNNNNNNNNNNNNNNNNNNNNNNNNNNNNNNNNN
NNNNNNNNNNNNNNNNNNNNNNNNNNNNNNNNNNNNNNNNNNNNNNNNNNNNNNNNNNNNNNNNNNNNNNNNNNNNNNNN
NNNNNNNNNNNNNNNNNNNNNNNNNNNNNNNNNNNNNNNNNNNNNNNNNNNNNNNNNNNNNNNNNNNNNNNNNNNNNNNN
NNNNNNNNNNNNNNNNNNNNNNNNNNNNNNNNNNNNNNNNNNNNNNNNNNNNNNNNNNNNNNNNNNNNNNNNNNNNNNNN
NNNNNNNNNNNNNNNNNNNNNNNNNNNNNNNNNNNNNNNNNNNNNNNNNNNNNNNNNNNNNNNNNNNNNNNNNNNNNNNN
NNNNNNNNNNNNNNNNNNNNNNNNNNNNNNNNNNNNNNNNNNNNNNNNNNNNNNNNNNNNNNNNNNNNNNNNNNNNNNNN
NNNNNNNNNNNNNNNNNNNNNNNNNNNNNNNNNNNNNNNNNNNNNNNNNNNNNNNNNNNNNNNNNNNNNNNNNNNNNNNN
NNNNNNNNNNNNNNNNNNNNNNNNNNNNNNNNNNNNNNNNNNNNNNNNNNNNNNNNNNNNNNNNNNNNNNNNNNNNNNNN
NNNNNNNNNNNNNNNNNNNNNNNNNNNNNNNNNNNNNNNNNNNNNNNNNNNNNNNNNNNNNNNNNNNNNNNNNNNNNNNN
NNNNNNNNNNNNNNNNNNNNNNNNNNNNNNNNNNNNNNNNNNNNNNNNNNNNNNNNNNNNNNNNNNNNNNNNNNNNNNNN
NNNNNNNNNNNNNNNNNNNNNNNNNNNNNNNNNNNNNNNNNNNNNNNNNNNNNNNNNNNNNNNNNNNNNNNNNNNNNNNN
NNNNNNNNNNNNNNNNNNNNNNNNNNNNNNNNNNNNNNNNNNNNNNNNNNNNNNNNNNNNNNNNNNNNNNNNNNNNNNNN
NNNNNNNNNNNNNNNNNNNNNNNNNNNNNNNNNNNNNNNNNNNNNNNNNNNNNNNNNNNNNNNNNNNNNNNNNNNNNNNN
NNNNNNNNNNNNNNNNNNNNNNNNNNNNNNNNNNNNNNNNNNNNNNNNNNNNNNNNNNNNNNNNNNNNNNNNNNNNNNNN
NNNNNNNNNNNNNNNNNNNNNNNNNNNNNNNNNNNNNNNNNNNNNNNNNNNNNNNNNNNNNNNNNNNNNNNNNNNNNNNN
NNNNNNNNNNNNNNNNNNNNNNNNNNNNNNNNNNNNNNNNNNNNNNNNNNNNNNNNNNNNNNNNNNNNNNNNNNNNNNNN
NNNNNNNNNNNNNNNNNNNNNNNNNNNNNNNNNNNNNNNNNNNNNNNNNNNNNNNNNNNNNNNNNNNNNNNNNNNNNNNN
NNNNNNNNNNNNNNNNNNNNNNNNNNNNNNNNNNNNNNNNNNNNNNNNNNNNNNNNNNNNNNNNNNNNNNNNNNNNNNNN
NNNNNNNNNNNNNNNNNNNNNNNNNNNNNNNNNNNNNNNNNNNNNNNNNNNNNNNNNNNNNNNNNNNNNNNNNNNNNNNN
NNNNNNNNNNNNNNNNNNNNNNNNNNNNNNNNNNNNNNNNNNNNNNNNNNNNNNNNNNNNNNNNNNNNNNNNNNNNNNNN
NNNNNNNNNNNNNNNNNNNNNNNNNNNNNNNNNNNNNNNNNNNNNNNNNNNNNNNNNNNNNNNNNNNNNNNNNNNNNNNN
NNNNNNNNNNNNNNNNNNNNNNNNNNNNNNNNNNNNNNNNNNNNNNNNNNNNNNNNNNNNNNNNNNNNNNNNNNNNNNNN
NNNNNNNNNNNNNNNNNNNNNNNNNNNNNNNNNNNNNNNNNNNNNNNNNNNNNNNNNNNNNNNNNNNNNNNNNNNNNNNN
NNNNNNNNNNNNNNNNNNNNNNNNNNNNNNNNNNNNNNNNNNNNNNNNNNNNNNNNNNNNNNNNNNNNNNNNNNNNNNNN
NNNNNNNNNNNNNNNNNNNNNNNNNNNNNNNNNNNNNNNNNNNNNNNNNNNNNNNNNNNNNNNNNNNNNNNNNNNNNNNN
NNNNNNNNNNNNNNNNNNNNNNNNNNNNNNNNNNNNNNNNNNNNNNNNNNNNNNNNNNNNNNNNNNNNNNNNNNNNNNNN
NNNNNNNNNNNNNNNNNNNNNNNNNNNNNNNNNNNNNNNNNNNNNNNNNNNNNNNNNNNNNNNNNNNNNNNNNNNNNNNN
NNNNNNNNNNNNNNNNNNNNNNNNNNNNNNNNNNNNNNNNNNNNNNNNNNNNNNNNNNNNNNNNNNNNNNNNNNNNNNNN
NNNNNNNNNNNNNNNNNNNNNNNNNNNNNNNNNNNNNNNNNNNNNNNNNNNNNNNNNNNNNNNNNNNNNNNNNNNNNNNN
NNNNNNNNNNNNNNNNNNNNNNNNNNNNNNNNNNNNNNNNNNNNNNNNNNNNNNNNNNNNNNNNNNNNNNNNNNNNNNNN
NNNNNNNNNNNNNNNNNNNNNNNNNNNNNNNNNNNNNNNNNNNNNNNNNNNNNNNNNNNNNNNNNNNNNNNNNNNNNNNN
NNNNNNAAACTCCGCCCTGACTCCTTCTGGCAACGTATGTACCTGAGGTAGAGGGGGACGGAAGAGGGGTTTACAACATC
AAGTGTCTTCACCCATATGGGTCGCAGCCTATTTTTAGTTTACTGGAGCACAGATTCAGAGTGGGTTATGGTTAGAAACA
TTGTTTTGTTAAAATCTGAAGTAGTAAGGGCAATATGGGCTCCAACAGCCAGCTCATTTGAAATCGGTTACGTGAGACAC
ATTGATATAAAAGGAACAATGACCTGTGTATCATTGGCTTTAGGGTTTCACACCGGATAAAAGGCTATAAGCACATTGAA
AATCGCCCTCTTTATCTTTTCATTCAGAGAACTGTCAACTCAACATATCCCATTCAATGATAACAGGCTATGCTTCTGAC
ACAAGAACTGGCTTCCGTCGTGCTGGGTTGGCTGATCTTAAAATGTGGCCCACTATATTACCATACGTCACACTTGAGGG
TTGAGACAGACACATACCTGTTGCCTTTACGGAACTCAGACTCCAGGAAGCGGATACCGTCTCTCGCACCAGCATTCTCC
TTCTTCAGGTGGTCAAGGCCATCAATCACTGAGGCAGATAAGTCAACCATCATGTTTTAACTTTGGTATCTATTCATTTC
ATGCTCTAACTAGAAAGACAAACACAGCTTCAGTCGTCCCCTCGGGAACTTACAAAACACAGCCAGCAAATAGCCAATGT
CGAAACAATATAGCAGGGCTATTCAACTGGCTGCCAGAAAGACAGATCAGGCCCGTTTATCAATTTAGCATGGGCTTCTG
ATCAATTCTGAAGAGTATATATATTTTTTAAAATCCTGCAACAAATTTTCTTTGGGGATGTTTAGAGCCAAAATGAGCCC
TTGTTCAATATTCTCCAATGGGGGAATCTGTTTCCCTGTAGTTCCGCCCATTAAATGCTGTCAATCAATATCACCCAACA
TACCAGGTACAGCCAATCAGAGCTGCTAACAGCGGTACAGGGTCTGTCTGCACGCCTATCGGCTTTTTCACAGCAGATGA
GATCACTACACTGCCTGATAAGTGCCAAGCATATTCTGGTGTAAATCAGCAGTGAGTTAAACACCAGACTCAGCCATGGT
TATCATAAACTTTATCTAATCAACATCTAGCCTGCAACATCACTTGTTTCGTGGTCTGCTGAGCATCATGATCTACTTTT
ACATAATGATGTTCAGTGGCTAAGCACGGGTAATAATGCTGTCAAGAGAGCTGTCAAGAGAGCAGCCTGTGTTAGCATAG
GGAGGAGATAGTTGCTTTTCTCCGTGATTGACGCCACAAGAAAGCCAACACATCTAGTGAAAATGCTTGATGTTCGTTTT
TCAGTGACATCTTCAATCAATTGAAAGGATTTAATTTGAGAGTACAGGGGAGGGATAAAACAGTTGACATGATTGAAAAA
CGTGTCAATAAAAAAAATACACATGTTATTTTCTCCTCTGATTTGATTTCCCTACACAACTATATTCAGTTATCATCAGT
ACAGATCGCTCCCGTGATGTCAGAGTTTTAAGATAAACTGAAAGCTGACTTTGCAACAAGATTTGAGGAATTTAAGTCCC
CACAGGTGGTGCAGTTTGTTACAGGGAGTCGTTCTCTACCGATGCGGGGGGAGAGTGACTTCTCCGCACAAGTTAGTCAA
CTGAGATATGACATGCATAAAAAGGTTTCCAAGTAAAAAAAACCCAGAAAAGAACAAAATGATAAAGAAATATAAAATAG
AACAGAATATGATGCTGGTCAACTGAGATATGCCATGCCTAAAAGGTTCACTATGTGTAAACAAAAACAAAAGATGAAGC
TATGTCTAAAAACGGACAGGAAAGTAGAAATGAATGTGATGCAGGTCAACTGTGTTACAAGTACAAATGTGTTCCGTTAT
TTAAACAATTTCATGCACTGAATTAGGCTTATCTTAAAGCACTTAAAATGTACTTTTAATAAAATGTGCTTTATAAGAGT
AATATAGAAGATGTCTGGCCCCCCTGATTCAAATCTGGACCCCGTATACTTCAATAACCCTGCAATATAGAAATCATATT
TATCCCAATTTTTATCTCCATGCAATTCTGGTGAGAAACCTTTAAATTATGACTGCATACAAACAAACAGACTTATCCAG
TCTAACACTGGGTTTATTCACTCAGACAGGGCATGGCTGTGCTCACCTGTGCGGGGGATGATGATGATGAAGCAGCCACT
GCCAGCCAGATGTCTGAGGAGGTTGAGGTGCTGGCACAGGGCGGCAGAGTCGGGTACCAGGTACGGAGACATGGATGACT
GANNNNNNNNNNNNNNNNNNNNNNNNNNNNNNNNNNNNNNNNNNNNNNNNNNNNNNNNNNNNNNNNNNNNNNNNNNNNNN
NNNNNNNNNNNNNNNNNNNNNNNNNNNNNNNNNNNNNNNNNNNNNNNNNNNNNNNNNNNNNNNNNNNNNNNNNNNNNNNN
NNNNNNNNNNNNNNNNNNNNNNNNNNNNNNNNNNNNNNNNNNNNNNNNNNNNNNNNNNNNNNNNNNNNNNNNNNNNNNNN
NNNNNNNNNNNNNNNNNNNNNNNNNNNNNNNNNNNNNNNNNNNNNNNNNNNNNNNNNNNNNNNNNNNNNNNNNNNNNNNN
NNNNNNNNNNNNNNNNNNNNNNNNNNNNNNNNNNNNNNNNNNNNNNNNNNNNNNNNNNNNNNNNNNNNNNNNNNNNNNNN
NNNNNNNNNNNNNNAAGCACACAGGCCAAAAACAATGCAGGAGTGGCTTCCGAGACAAGTCTCTGAATATCACTTGAGTG
GCTCAGCCAGAGCCCGGACTTGAACCCCCTCCCCCTCCCCCCTTTTGCCCTCAGAACAGCCTCAATTCGTCAGGGTGTGG
ATTCTACAAGGTGTCAAGTGCATTCCATATGGAGGCTGGCCCATGTTGACTACAATGCTTCCCACAGTTGTGTCAATTTG
```

TCTGGATGTCCTTTGGGTGGTGGTCCATTGTTGATACACAGAGTAAACTGTTGAGCGTGAAAAACCCAGCAGAGTTGCAG
TTCTTGACACAAACTGGTGCCCCTGCCATCTACCATATCCCGTTCAAAGGCACTTAAATCTTTTGTCTTGCCCATTTACC
CTCTGAATGGCACACAGACACAATCCATGGCTCAATTGTCATGAGACTTAATCTTTCTTTAACCTGTCTCCTCCCCTTCA
TCTACACTGATTGAAGTGGATTTAACAAGTGACATCAATAAGGGATCATAGCTTTCACCTGGATTCACCTGGTTAGTCTA
CAGTACGTCATGGAAATGGAATGTTCCAAATGTGTTGTACACTCAGTATAGTGTCAGATAGCTAGATTGATAGTGTTTGG
TTTATGATAGAATGGAATGTTCCAGAAGAAAGACCTGTAGTCGGAGCTGAGCCATGTCTCTCATCAAGCGGTTTCGTCGA
GCCTCCTCCTCTGCCTAGACACACAAACGCACAAACAGTGTTGAAATAGATAAGTTTGCAGTCATTTGAGCTTCACAAGT
GAAACAAATACTGAAACTAGAGCGACTGCTGCTGCTTTGTTCTCATTGGTTCATTCAACTGCCGCTCACCATGCGGATCT
GGGCCTTGGCCTGCTGCACCAGGTTGTCCTGCTCCGATTGGCTGATGCTGGTGAAGATGCCCGCCTCCGGGTTGAAGTGC
AGCACATTGCATTGGAGATTAGTGAGGAAGTGGCCAAAGCTACGGATGCAGCACACGCGCACCACACACTGAGATGGGGA
GAGACAGAGAGAGATCAAGTGAGATAGTAAAGTAGAGAGGGGGCAGCAAAACAATGAGGGTGTCAAAGGGAAGCTAGGTG
TGTGTACCTCCTGTACGGGGCTGAGAGAAGGCCTGGGGCGGGTGTAGTCGAGGCGGCGGTGGGCCAGGTTGAGGGCAGGT
AGGTGTCGCAGGGCCAGGTCCTCAGGCAGCAGCAGGGACTGGACCAGGCCAGGCTGCTCACACTCACTCAGCAGCTCCAT
CACCTCAGTGTTCAGACCCAGCTCTAAAATACACACACAGAGAGAGAGAGATTAAGAAGTGACTTCTTGTCACATGTGCT
CCACAGAGACACATTTGGAATTTCATCCCTGAAAAGATGGGTTAAAATAACCAAAGGAGAGGAAAAGAGAACAGATGGAA
TGAGAAATAATGTGAGAGGGGGGAGAGAAGAATTTGGCCAACTCAAGCCTCCATATCGGGCTCCCGAGTGGCACAGCGGT
CTAAAGTACTGCATCTCAGTTCTAGAGGCATCACTACAGACCATGGTTTGACTCCTGGCTGTATCACAACAGGCCATGAT
TGGGAGTCCCATAGGGCGTCGCACAATTGGCCCAGCGTCATCCGGGTTAGGCAGTCATTGTAAATAAGAATTTGTTCTTA
ACTAACTTGCCTAGTTAAATAAAGGTTAAATAAAAATAAAAAAAAACGCAGATTAACAACCCATAAAAAGGACAGGTGCA
TTTAGTAACTCGCCTGCTTCCAGCATCTTGCTCCCATCAGGCAGCAGGTTGAGTAGCACAGACAGTCTGTTCCATAGGCT
CTGAGAACTCTACAGACAGAGTAACGGAATTACATTGAGCGGAGGACGGACAGTGAAAAACACCAACAATAATGAAGACT
ATAAACCCTTGCCCCGAGTCAGCTGATACAAAGATTAACTTGTATTTTACCAATTTAATGCAAATTGGATTTTTCAATGC
ATACTGTTTGTAAGTGCTTTTAGGTTACTGAAAAATCCACGCCCTTTACCTGTGCACACATGACGATAATGTCAGTGTTG
GTTCTCAGCCAATCCACAAACACCTTGACCACCTGGATGAGCCCCTGATTGGTCAGAATCTCCAGTCTCTCTGACAGGGT
CTTCTCGCTGTGTACCGATTGGTTGCTGTGCACGCTACACTCTGAATCGGAATCCACCTCTACAGGGAATGGACAAATAA
ATAAATAAACATGTGTTTAATCTACACCCTAGTTTTCTTAGGGGGAACCTCAGATGTACTGTTATAAAATAGTATATTTT
AGCGATGTGCTGTTACTCAATGTAATCTTTGGGAAAGACTAAAAACAGATTAGAAGTACATCTTTATATTGAAGTTAATC
TTTAAAAAAAACAATAATAATCATATCCTGCACTATACAAAAATCACATAGTATTCAACTAGAGGTCGACCGATTAATCG
GAATGGCCGATTAATTAGGGCCGATTTCAAGTTTTCCTAACAATCGGCAATGCTCTGTAAGGGTCGCAGCTTTTGTGGAA
CGATGGGTAATGATGCTTCGAGGGTGGCTGTTGTCGATGTGTTCCTGGTTCGAGCCCAGGTAGGGGCGAGGAGAGGGACG
GAAGCTATACTGTTACACTGGCAATACTAAAGTGCCTATAAGAACATCCAATAGTCAAAAGGTACATGAAATACAAATGG
TAGAGAGAGAAATAGTCCTAGAATTCCTATAATAACCTAAAACTTCTTAACTGGGAATATTGAAGACTCATGTTAAAAGG
AACCACCAGCTTTCATATGTTCTCATGTTCTGAGCAAGGAACTTAAACGTTAGCTTTTTTACATGGCACATATTGCACTT
TTACTTTCTTCTCCAACACTTTGTTTTTGCATTATTTAAACCAAATTGAACATGTTTCATTATTTATTTGAGGCTAAATT
GATTTGATTGATGTATTATATTAAGTTAAAAGAAAAGTGTTCATTCAGTATTGTTGTAATTGTGATTATTACAAATAAAT
AAATATATTTATTTATTTGTACTTTTTTTTTGGTAATATAAAAATTGTCCGATTAATCGGTATCGGTTTTTTTGGGTCCTC
CAATAATCGGTATCGGCGGCGAAAAATCATACTTGGTCGACCTCTATAATCAACACAGCGTCAATGTGTGAGGGCAGGTT
ACTGACCATTGTCGTTGGTTCCGTTGGCAGTGCCCGGGCGGGTGTCGCAGGGTGAGTCCCCCGGGGGAGGAGGGGTCTCC
TGGGAGGGGGTGGTGGAGGGGGCGTCTGGGTCGGTGGGGGTGGTGTTGGTGGCGGAGCTGTGGGGCCTCAGCAGCACGTT
GCTGAAGGTGGGCGCCAGGCGGAAGCAGCGCTTGGCCTGGAACAGCTGGGATGACAAGGCCTGGAGGTTACTGCTGATAC
TGGGGTCGCTGGGCAGGAGGGGGCCATTGGTGGCCGGAGGGGTGTCCCCGTCACCGTCCTCCCTGGGATTGGCTTGCTCC
TCATCATCTTCTCCGGGCTGCACCTCCCCCCGAGGATGGGTGTCCAAGTTCTCCGGAGTTTCTGAGTCCTCTATATCCTC
CAGGTCGGAGCGGGACTCCTGGCTGTTCATGTCCGAGTCAGTCTCCACGTCGAAGGCCGTGCCACCCTCCTCGTCTTCCT
CCGAGCCGCTCTCCCAGGTCCCCTCCTCTGTCGGGTGCTCCCTCCCTCTTTTGCTCTCCTTCCCCAGCGTGGCGGGGGTG
AGTGGGGGTCCCGACGAAGCACCCCCGGAGTTGGCGCAGCCCCTCTGCTCTTCTTCCTCCTCTTCATCACTCTCGAAGCC
CTCGCTCAGGTCGCTCTCGTCTTCTTTGCAGGTGGCGCGGCGGCGGCGCAACATGGAGAGGCGGGCGTACTTCTTTTGCT
TCTGTTTCTCCTCCTCCGCCTTCCGCTCACCGACGCCTGCTTTCTGGGCCACAGTGCTCCGGTGGCCCCCCTCGTCCTCC
TCATGGTCCAGGGAGCCGTTCTGCAGGGGCCTCTCCTCCGAGGTCAACGGGTCAGACGGGTCTCTCAACTCAGAGTCGTC
TAGGAGCAAACAAATATCATTATCGTTCCATTTGTTTTTCAGAGGATTAACAGAACATGAATAGTGTATTGGCAGCATGA
GGTTAGCTGGGAGTGATGTTCTCACCTGTGTTGTCGGGCTGCAGCGGGGGCACCTGGCTCTCCCCCTCCTCCAGCTCAGC
CTGCAGGCGGATGTTGACATGGTTCACCAGGTGGGAGAAGAGGGCCAGCGTGAAGGCTATTGACGCACTGTACTGCTTGG
ACCCTACACAAACAAGTCGACATATCAGCTTTGCAATACAACGAATCAACATATCCCCTTATTTCTCCCTTGCAGTATGT
CACTATCCTCGCATGCCTACCCACTCAGCTCTACTTACAACACCCTATGCGCATAATGTTTGAAAAGCGATTTGTAAAAA
AAGAAGAAGAAAAAAAAAAAAGACATCTATATTCTTACTGACCTCCCCGCTTCAGGCTGTGCACCACCATGAGGCAGGTG
ACCACCATCTTGAAGATGAGGGCATCGGGGAGCAGGGCGCAGGGCGAGTCGTGCTCCTCAAGCTCCTCCTCACTGGGGGA
AGTGGCCTCGTGGACATGTGGCGGCGAGGGCAGGTAGAACAGCACCAGGTTAAAATCCTCCAGCACCGACTGACACAGAG
AGGTCAGCTCTGTCTCCAGCAGACTACTCACAGGGAGGAGAGAAATGGTAAGACATTACAAACACGGTACCCAACAGACA
ATAGAAAGCAGGTTGAGACTGACCAAACAGGGTTATAAAAATTCAATCAATTGTTATTATCATCAGATAGCTTTGTCATA
TATGGACAAAATATCAAGGTTCCTATTAAGATAAATGACTAGGGCCTAAGTGGTGAAAAGTGAGTCCTAACCTGTTCTTG
GGCTGCAGTAGACTCTGTAGGTACATGAAGCTCACCAGAAGTTGCTTGATGTCCTTGGACCTGGAAAGAAAACAGTTGAA
GAAACAGTCCTCTTTGGGATTGTTACTATCTCAATACCTGTTTCAATGCTGACCTAGTGATTTGTCATTTCAGGGCTGTG
GTGTGAGGAAACCAGACTGACCTTTGCCGTGAGGGAGAGAGCTTCTTCATCTCCTGTTTCTTCACCTGGTGGTACATCTT
AGCAGCTTTATCAAACAGACGCTTTAAATTCCCGTAGGCCCCCTCGAAGGGAGTCTCCGACTGTATACTGTTCAAACACG
GGACCATAGAGGAGAAAACCATGATCAATACTTGGTGGTAGGGGTGTGACGATACCAGAATCGCAATTTTTTCCCCATGG
CAATGAAAACACAAAGCAGACGCAACTCTTTGGTCCTTTAAAAACCTTCTGTATGTAAAATAGTGTGTGCTATAGCTTAG
AAAACAAATAATTGTGACTGGATGACAACAATGTTTCTTTCCAAAATCAGGGCTGTTTTCTGAAAGTTCAATCCGCTTTG
TGTTTTGTAAACCCACCACTACTAACGAGTATTGCAATACTGGTATCGTCCCGGTACCACTCGGTGGCAAGTCTTTTTTT
TAACGTGTTCTGGATCCGAAGTTGGGCTTCTTCTCCTAACAGACTGATTTCAGAACCTCTACCATTTCAAACATGCTATT
TAGAGCCTACTGATGTGATCCAAATATGAATTGCTGCTATGAAGATATTTCTCCCCAGCAGTGTGACTACATTGCCCCAC

TGAGGTAGAGAACAATAGCACAGCATAGTCTACAGACCAGTCATAGATAGACTGTGGATGTGCTGCAGTAAATCTAGACG
TGTTGAAGTCTCCTCTCACCAGCGTAGGTAGTAATAGGTGGCCTCCACATTGTAGAATTTACTCCCCGCCAGTGTACCTA
ACTGATTGAAAGGCATTCCTGAAAACAAAACATCAAACACTTGTCAACATCATGGCACACTAAAGACATTGCTTTCCTTT
AGCAGCAGCCATTTAATATTGACTTTCAGTTCGACTGATGAAGCCCTCTGAAGCCCTCTCAAAAAGACAGAACGTTTCAA
ACGTGGATCAGTGATTTCTTGAGACTGAATGCACTCTCCTGGCCCGCTAACAGTTACTGAATTTCCCGTTCCTGGCTCAA
ATGGCCCGCTAACCATTCAGCTAGATCACTATGAGTTGCGTAAAGCTTTGACCAGTCATATAATAAAGCTCAATCAATAA
GTGTTGTGTGAGTTCATCACCATGTGGGCAATATAACAAGAGTGATTCATTTGTAGATGGTGGTGTGGTGTGAGTAAACT
GGTTAGTGTAAAAACAGTGGTTAATGTGCTGTGAAAGGGAGGGCCGGTAACACCTTGTTGTCAACTCACCTACGTGCGGA
GTGACAGACAGGGCCTGATGGTAGAACCGCTCTGACAGCTGCTCAGCCTCCACCCCAGCCAACTCGTTCTGGTAACGGGC
TGCGGAAAACATTCAAACAACCTCCCGTAAACCCCTCCTGTACATTCAAATAAACGTGACCTTGGATTTGGGGAATGATA
TTCCATGGTCATACAAATAAGGTTGTCTCAAATCTCTTTGGTGAGAAAGATTAAGTTTGTATTACCTAGATCTCCCAGGT
ACACCAGGCAGCGATGGCAGGCCATTTGGGCCCAGTCCATCTCCTTAGGGGTCGCTGATACCGGCTTCTTCCGACCTACA
GAAACAAATACAATAATTTATACACTGTCAATCACAGATACCGCTGACCGGAGTGTGTGTGTGTCTCACCGATGAGGGGG
TCGGTGACATGGGTCCAGTCGATGCAGTCCTGCATTTCCAGCTGGTAGTGGGACTGGATGTAGAGCAGCAGGTGCTGGTA
GAAGCCCACGCCGGCTATCAGGTGAGTCCTGTAGGCACACTCCAGGGCACTGCGACTGTGGATGTGCTGCAAAAGACAGA
GAGATAAAAAGGGGGCCAGGTAATAAGAAGAGATTAGAAATAAAAGTTACGGGTTGTGTGTAGGCCATGAGGAAAGATAA
AGTGTGCATGTCTGAAAGCTGAGCAGTCACTGTACTGGCCACACAGTCAACATTTCCCCAATAACTTAGGGTGTTTCCAC
ACATAGTCCCTTTTAGCACATTTTTGTGAACTCAGTGTGGCTTGCATAATTTTTTGTTCAGTGTTAACACTCCAAAAGGA
ACTCAGACCCCTCAAAAGAGCCCCCGAAGCAAACTGAGACCATCTCACTTGAATTCTGTGGTCCGGTTCCATTATTTAGG
GCAATGCGCCCCCAGGTTCGCTTGTGATTACACACACTACTGCAACACTGTTTTCCCTGCCATAACCCCTAACATTGGTG
CCACAAGAGAGAAGATTATGTGCAGGGTCGAGGGGAAAAAAAATGTGTGCCATTTTTGGATATTGATTAGCGATTGTCAA
GGACACGCAGACATTCCAAAATGTGTAGTATTTTTAGTTCAGATTATTTGTTTGCAATATGTGATCTATATGAGAGTTTC
ATACACTGTTTATTTGATTGTGGGGTTGGAAGTGTAAGAAAACATATTTGGTGAGAATCAACATAACGTCCAAATCTATT
CTAGCGCTTTAAGGGGCGGCTGCCTACATTGGGTGTTCAATTTTCAATTACAGCATTATTTAATCTGCAGTTATTCTTCC
ACTATTTATTCTGTTACCAATAATAATTACATGTTAATAGTATCTTCTGATTACAATTATTATGTCTCATCTTTTAACTA
AATGCAATATATTAGATTCTGCAATGTAAGTAGGTATATCTGTCCAATAACATATTTTGATTAAATGTATTATTTGTGAT
GTGTGATGATAGATAGATAGATAGATAGATAGATAGATAGATATTTTAAACAACTAGGCAAGTCAGTTAAGAACTAATTCTTAT
TTACAATGACGGCCTACCAAAAGGAAAAAGGCCTCCTGCGGTGACGGGGGTTGTCATTAAAAATATAGGACAACACACAC
ACCATGACAAGAGAGACACCCACAACACTACATGAAGAAAGACCTAAGACAACATAGCATGGCAGCAACACATGACAACAC
AGTATGGTAGCAACATAACGTGACATGGTAGCAGCACAAAACATGGCACAAGACAACAGCACAGAGGCAAGAAGGTAGAG
ACAACGATACATCACATGAAGCAGCCACAACTGTCAGTAAGTGTCCATGATTGAGTCTTTGAATGAAGAGATGGAAATAA
AACTGTCCGGTTTGAGCGTTTTATGCAGCTCGTTCCAGTCGCTAACTACAGCGAACTGAAAAGAGGAGCGACCCAGGGAT
GTGTGCGCTTTGGGGACCTTCAACAGAATGTGACTGGCAGAACGTGTGTTGTATGTGGAGGATGAGGGCTGTAGTAGGCA
TCTCAGATACAGGGGAGTGAGGGTTTTATAAATAAGCATCAACCAGCGGGTCTTGCGACGGGTATACAGAGATGACCAGT
TTACAGAGGAGTATAGAGTGCAGTGATGTGTCCTATAAGGAGCATTGGTGGCAAATCTGATGGCCGAATGGAAGTCGCTT
GTCCCAGAGCGCATTGAGGGAATGTTGGAAAAAGATCTTGGGTCATTTCTAAATATAGCAATGTGAATGCAAAAGAGTAG
AGTCCTCATTCAAAAGCAAGGGAAGTGTGAATACAAAGAGAACAGAGTTTTTAGCTTCTTTTTTACCACAAAGTTCACTT
TAAAGAGGACGGAGATCGGTTATTTTAAAGAGGACTATATTGGAAAACACCCTTAAATGCTGTACCTTCTTGTGGGTCTT
GACGACCTGGATGACCTCGTAGTAGACCTTCCTCCACAAAAGCTCCTCAGCCTTGCGACCATAGTCCACAGGGTGGAGAA
ACATCAGCTTCACACACAGCTCCCTCAACCTGGGCACAGAAGACAGATGAGGTTGGGGTTAGTGCTTTGCCTTTTGCCTT
AGTGTTTAGGGCAAAGGTTGGAGTAGAGTAAAATGCTTACTTGTTGCGAAGGCTGATGTTTTCAGGCTTGAAGACCTCCC
TGTATGATGACTTGCTGCCAATGATGACATCCAGCTTGTGCACCGACTCAACCACTGCCCTGAGCACGCAAGAGACAGTT
ATTATGGCAGGTGAATTACTGGTAATGGTCATTGGAGGAATGAGGAAACTCATTCTTATCAATGTAGGGAGCCAACATTA
GCTAGCTAGCTATAACTAGAAAATATAAACTGTAATACATGTTTCACACTGAGATAATGGTTGACATTATAGCTTACTAA
CTAGGTACAGTGAGGGGAAAAAAGTATTTGATCCCCTGCTGATTTTGTACATTTGCCCAGTGACAAAGAAATGATCAGTC
TATAATTTGAATGGTAGGTTTATTTGAACAGTGAGTGCTTCTAATCTCAGCTTGTTACCTGTATAAAAGACCTGTCCACA
GAAGCAATCAATCAATCAGATTACAAACTCTCCACCATGGCCAAGACCAAAGAGCTCTCCAAGGATGTCAGGGACAAGAT
TGTAGACCTACACAAGGCTGGAATGGGCTACAAGACCATCGCCAAACAGCTTGGTGAGAAGGTGACAACAGTTGGTGCGA
TTATTCGCAAATGGAAGAAACACAAAAGAACTGTCAATCTCCCTCGGCCTGGGGCTCCATGCAAGATCTCACCTCGTGGA
GTTGCAATGATCATGAGAACGGTGAGGAATCAGCCCAGAACTACACAGGAGGATCTTGTCAATGATCTCAAGGCAGCTGG
GATCATAGTCACCAAGAAAACAATTGGTAACACACTACGCCATGAAGAACTGAAATCCTGCAGCGCCCGCAAGGTCCCCC
TGCTCAAGAAAGCACATATACATTCCCGTCTGAAGTTTGCCAATGAACATCTGAATGATTCAAAGGACAACTGGGTGAAG
GTGTTGTGGTCAGAGGAGACCAAAATGGAGCTCTTTGGCATCAACTCAACTCGCCGTGTTTGGAGGAGGAGGAATGCTTC
CTATGACCCCAAGAACACCATCCCCACCGTCAAACATGGAGGTGGAAACATTATGCTTTGGGGGTGTTTTTCTGCTAAGG
GGACAGGACAACTTCACCGCATCAAAGGGACAATGGACGGGGTCATGTACCATCAAATCTTGGGTGAGAACCTCCTTCCC
TCAGCCAGGGCATTGAAAATGGGTCGTGGATGGGTATTCCAGCATGACAATGACCCAAAACACATGGGCAAGGCAACAAA
GGAGTGGCTCAAGAAGAAGCACATTAATGTCCTGGAGTGGCCTAGCCAGTCTCCAGACCTTAATCCCATAGAAAATATGT
GGAGGGAGCTGAAGGTTCGAGTTGCCAAACGTTAGCATCAACCTTAATGACTTGGAGAAGATCTGCAAAGAGGAGTGGGA
CAAAATCCCTCCTGAGATGTGTGCAAACCTGGTGGCCAACTACAAGAAACGTCTGCCCTCTGTGATTGCCAACAAGGGTT
TTGCCACCAAGTACTAAGTCATGTTTTGCAGAGGGGTCAAATACTTATTTCCCTCATTAAAATGCAAATCAATTTATAAC
ATTTTTGACATGCGTTTTTCTGGATTTTGTTGTTGTTATTCTTTCTCTCACTGTTCAAATAAACCTACCATTAAAATTAT
AGACTGATCATTTCTTTGTCAGTGGGCTAACGTACAAAATCAGCAGGGGATCAAATACTTTTTCCCCTCACTGTAGGTAA
AGCTAAAGAGGCTTGTGAAATATGGTGTTTCGCCCTAACTAGGCCTAGTTATGCATGTTAAAAGTAGCCAGTCAACATTA
TTCATGTGGTGATGTTCTGCTAGACGCTTCTAGTCAGCTGCTACATAACTAACTAGCTAGGTAGGTAGCAAACAAGGTTG
GCCTATTAGCTAACTAGTACTGTGACAATACTGACAGCTGAAGCTATCACAACTTAGTTAAACAACTAACGTCATTTAGA
AAGCAAGTTGACACAGGAATAAGTTATCTAGTTAATAGCACAGTAACTAGCACAGTAACTTGTCAGTGCTCAGTTGTCTA
GAAGCTAACGTTAGTTAGCTAATTATCTATCCGCTTCACAAGTTCCCACTGTGCTACATCTGGTTATTTTAAACCCATAC
TATCGAAAATGTCCGACTACTTCGTAAAAATGTCATTGCCCTTCGACGAAGGCTTCGTGACAAGTACAGTTAACTAGTTA
ACGTTAGCTGATGCAAGTCACAGACAGACAGGTGACTGGGTAGTTAGCTAACTAGTTAGTTGAATAGATGGCTAGCTAGT

TAGCAAACTAACGTTAGCTAAGGTTAGCTATCCATTTATTTTACTACTGTTAGCTAGCTAACGTAATGTTTCCCAGCAAA
AGGCCTTTAACTCACCTGTATAGCCGTTTGATGAGGAGGACTTTTGCCTCCGGCTCGCTATCCTGTCCAGGTCCACTCAT
GTTAGCATCTGGTGCCAAGAGTCACACGCTCCAGTCCAACAGAGTCTGCGTTCCCACCCTCTTCAACCCCACTCTTGTCA
GGGTTCTCCCGGCTTGCCTCACCGGCAGTGACAACTGTGTTGACTAGCTAACGTTAGATAGCAGCTCTATCCTCTCCCGC
TAGCCACTATGTCGCTTTCGTATCTTATGTCGCTTGGCAAACTGCCCATCCGTTCAGAAACCGTTTACGAGAGGGAAAGC
CCCAGTATTTGTGTTAATTCCCGGTCAGCTATCCCACCCCGATGGTTTCCTTGTGTTATTCCCCGTTCCAAACATCGCTA
CGAGTAAGAGGGCGGCCATTAAACAGCGACCGCCTGAACAGCGCGAGACTTCGCGTGAAACGTTCGACCTGCGTTCGCGC
CGGACCGTCACTAGTTACCACAGCCACAAAGTCATGAACTCCGCCATTTCTACAATTTCTCTTAATTAAAATATGACTTT
ACCTTAACCCTAACCTTTACCACACTGCTAACCTTATGCCTAACCTTGAATTAAAACCAAAAAGCAAAAAAAAAAAAAA
AGAAGGGAATTTGTGGCTGTAGAATCTAGTGGAAACCTAGCTAGATTGCCAGACAGCCAGAGTCATGGTAAATTTGTTTG
TGTACGTTTGTGATCCCTGGCAATGTAAAACGTTTGTATATATTTTTTGGTGATTAGGGCTGCATGGCACAATAACCGGG
TAACCAATGATTATTGATGAAGACTGTCATGAAAATGAAATAAGAGTCATAACCACCTAAAAAAAACCACCTGACTGAAG
ACTGGACTGCTGGGCATTTGTGCAGTTGACTTCGTTTCTGGACTCAAAAACTTGATGAAACTTTTGTTGCACCGTGCTGA
AACAATCAAGGTGTTGTAGCAAACAAATCTTTGGTTGCCTAGGCAATTTACATTAGTCATTTAGCAGACACTCTTATCCA
GAGCGACTTAAAGTTAGTGCATTCATCGTACCCCTTTCCTGCAGTCAAATGACCTAGTGGCCTCAAGGGTAGAATGTTAT
TCATATTTTTTTCATAATTAATAATAAAAAAATGAAAATCTGGTGTTTCTATGCCAAATAGTTTTGTTATATTTCAGTCT
TCTGTGATGTATATAAAGTGTAATATTGGGATGCAAACTCAAAATTGAATACATGTCAACTATTTATCTGACAGGTTACA
GGTGTCTTCTTTTTTTAAGCCCATGAACATGTGTGTGAGGTGTATACTTTTGTTTCAAAGTAGATTTGTTTAAAACTAAT
AAGAAACACTGTGTGACCCTGATTTAGCCCACTGCAGTATTAAGATAGCTAGGTTGGACAACAATATATCACAGTCATTG
TAAGTACATTTTTCCTTAATAAAGTAGCTATCAGAAAAGTCTGAGTTAGTAAGAGGGAAAAGAGTTAAGTGTTAGTTCAC
AAAAGGCTTCTTTTTTGTTTTTACACAGAACAAAAATATGGGCTAGCGGTTGGCTGATGTCAACGTTGTGAACAGAGTGC
CCCATGGTGGCGGTGGGGTTATGGTATGGGCAGACATAAGCTACGGACAACAAACACAATTGCATTTTTTCTATGGCAAT
TTGAATGCACAGAGATACTGTGACAAGATCCTGAGGCCAAATGTCGTGCCGTATAATCGTCGCCATCACCTCATGTTTCA
GCATGATAAAGCACAGCCCCATGTCGCAAGGATCTGTACACACTTCCTGTTACCTGCATACTCACCAGACATGTCCCCCA
TTGCGCATGTTCTGGATGCTCTGGATCAATGTGTTCCCACCAATGTCCAGCAATTTCGCACAGCCATTGAAGAGGAGTGG
GACAACATTCCACAGGCCATAATTAACAGCGAAGGAGATATNNNNNNNNNNNNNNNNNNNNNNNNNNNNNNNNNNNNNNN
NNNNNNNNNNNNNNNNNNNNNNNNNNNNNNNNNNNNNNNNNNNNNNNNNNNNNNNNNNNNNTGGACTTTTGACTATAACA
TTCCCGAAATGTAATTTTTTTTTGCTTTTTTAGCCATTTTTATGTAGACTAGATTGTGTGTATTGGATCATTGTCTTGCT
GCATGACGCAGCTGCACTTCAGATTCAGCTCACAGATGGATGGCCAGACATTCTCCTGTAGAATATAGTGATACAGAGCA
GAATTCATGGTTCCTTCTGGTCACGTCATCCAGGAACAGCAAAGCATCCCTAAACAACACTACCACCACCATGCTTGACC
ATTGGTATGAGGTTCTTAATGTGGAATGCAATGTTTGGTTTTTGCCAGGCATAATGTGACCCAGTTATGCTTTTGAATCA
TCTGTCCATAGAACATTCTTCCAAGAGTCTTGATGATCATCAAGGTGCTTCCAGCTGTTTTTGGCAAACGTCAGTCAACT
TTTAGGATGACATGGGTCCCATGATGCTTGGCGAAAACCAAACACTGCATTCCAATACCTTGCAAAGGGCACCTAGTGGT
AGATGGGTAAAAAAAAAAAAGCATACATTAAATATCCTTTTGAGCATGAAGTTATTAATTCCATCTCAGATGGTGCATCAA
TACACCCAGTCACTACAAGACTTTAAAAGAGAGTTAATGGCTGTGATAAGAGAATTGAGGATGGGACATTGTAGTTACTC
TAATTGACGGAGTGAAAAGGCAGTTTGTACAGAATAAAAATATTCCCAAACATGCATTCTGTTTGCAACAAGGCACTAAA
GTAATACTGAATACAAACTGTTAAGTTTGGGGCAAATCCAACACATTCAAGCATGGTGGTGGCTGCATCATGTTATGGGT
ATGCTTGTCAGAGAAACGGATTAGAGCTAAACAGGCAAAAGCCTAGAGGAAAACCTGGTTCAGTCTGCTTTCCAGACAGA
CACTGGGAGACAAATTCACCTTTCAGCAGGACAATAACTTAAAACACAAGGCCAAATATACACTGAAGCTGCTTACCAAG
ACATTGAATGTTTTTGAGTGGCCTGGTTACAGTTTTGACTTAAATCAGCTTGAAAATCTGTCAAGATTTGAAAATGGCTG
TTTAGCAATGATCAACAACCAACTTGATAGCGCTTGAAGAATTTTAAAAAGAATAATGTGCAAATATTCTACAATCAAGG
TGTGCAAAGCTCTCACAGCTGTAATTGCTGCCAAAGGTGATTTTAACGTGTACTGACTCAGGGGTGTGAATACTTGTGTA
ATGAATACTCAAGGAGAAAAAGGTGTAGATACATGCGCAGAGCGCGGCAGGTGTTTATTTAGTCTTCACAAAAGGCAGGA
ATCGTGGTCACAGGCAGGCAATGGTCATACACAGGTAGGCAAATCAAAACTAGCACTGAAGGCTGTAACTAGTTCTCACA
AATGAGCTAGGAAAAAGCTTAGCCGGGTCAAAACGAACAATACCTCACAAAGGCACAAACAGAATGAACTGATCTAAATA
AGGAGCTGATGAGACCAGGTGAGTAACTAACACAGCTGAAATCAATGAACAAAAATGAAAGACAGGGCTACGTTCAAGAA
GACAAAGAAACAGAACACAAGGTTGACTAAGAAAACACAGAACCTTACAGTAATACACACACCACCTTGTATGGCTGAGT
GAACCTCCCACTGTACCGGTCCCATAATGCAAGAGGAAGGAAGAAAGGTGTAGGGTCTGAGTTATTGGTCAGAAGGTCAA
ACTGGCGGGAGAGAGCATCAGCTTTTACGTTTTTCTTTCCAGGGATGTAAGTAACATGAAATGGAAGTGTGTGAAGAGAG
CCCAGCGGGCTTGTCTGGAGTTTAACCTCTTGGCCCCTCTGATATATTCCATTACGATGATCTGTTAGGACGAGAAAGGG
ATGTTGTGCGCCCTCCAACCAGTGGTGCCACTCCGAGGGCCAGCTTGATGGTGAGGAGTTGTCTGTTCCCCACATCGTAA
TTCCTCTCAGCGGAGGATAACTTCCTGAGAAGTAGGCACAGGGATGTGATTTGGGAGGTGTTCCCACCCGCTGAGAGAGT
ATGGCTCCTACTTCGGAGGCATCCACCTCCAGGGTGAAAGGAAGGGTTGGATCAGGTTGGCGAAGGACAGGAGCTGAGGT
GAAGAGGCGTTTCAAGTTGTTGAAAGCAGTCAGAGCAGTATCAGTCCATTGACGGGTCTGGGTCTTTTGGCTGATAAGGA
CACTGAGGGGAGCGACAGTAGAACTGAAGTGCCGAATGAGTCGGCGATAGTAGTTGGAGATCCCAATGAAACGTTGGAGT
TCCTTAAAGGTGGTGGGTTTGGGCCAGTTACTGACGGCGGTGACTTTGTTCTCATCCGTGCTGACCCCTCCAGGTGTCAG
TACGAAACCGAGGAAGTTTACCAAGGTTACATGGAAGGTGCTCTTTTCAGTCTTCACATAAAGGTTATGGTTAAGGAGCC
GTTGAAGAACCTTTTGCACATGCTGTATGTGTTCCTTCAGGGAGTTGGAGTAAATCAGGATGTCATCAATGTAGACGATG
AGAAAGCTATTGATCATATCCTGTAAAACCTCATTCATAAAGGATTGGAAGACGGCGGGGGCATTAGTAAGGCCGTAGGG
CATGACCAGGTATTCGTAGAGGCCTCATGCGGTGATAAAGGCAGTCTTCCATTCGTCGCCTTCACGTATACAGACAAGCT
TATATGCACTTCGCAGGTCGAGTTTTGTATAGATGTTGGCGGGGCCCACTTGTTCAAGGGTTGGGCTGGGACCATCGTCC
CCTGGGCGATGTGGTGGCAGAGTGGAGGCCTTGATTTTGCTGAAGACATTGCTGTACTGTGAGTATTCAGATGCACGGAA
GAGGAGCATGGCCCAACTGCATTGGCTCTGAAAGACTAAGACGGGATGACGGAATCATGGAAAGAGAAGGTTTCGGGTTC
CTGGGTGGCAGAGTTCTTCGGCGATGAGGTGGTCGATGGAGATACGAATGTATTGATTTAAATCCTGAAGGTCACCACTA
CAGGCCAGCTCCGCCTGAAGTTCCCTGTTGAGCCCTCTTCGGTAGATTGTAAGTAAAGTAGCCGCACTCCATCCACTCCC
TGCAGCCATGGTGCAGACCTCGAGGGCATACTCGGCAGCTGAATTGCATCCTTGTTGAAGTTCTATGAGGAGGTCTCCTA
TAGGACGACCGGAAGGAGAGTGATCGAATACCTCTTTTAAGAGGGTGTGGAAATGGTTCTGTGCTGTTGGCAGTCCATAT
GGCGGTGGCCCAATCTAGGGCTTTGCCTGTGAGTAGAGACACAACAAAATCCACCCTGCTCTTGTCGGTGGCGAAGTTAG
TCAGGTTGTGCTCAATGTATTTGCTGTATTTAAATAAGGAGCTGATGAGACCAGGTGAGTAACTAACACAGGTGAAATCA

ATGAACAAAAATGAAAGACAGGGCTACGTTCAAGAACACAAAGAAACAGAACACAAGGTTGACTAAGAAAATAAACACAG
AACCTTACATTATGCAAATGAGATATTTCTGTATTTCATTTTCAATAAATGTGCAAATATTTCTGAAAACATGTTTTCAC
TTTGTCATTTTGGGTTCTTTGTGTTGATGGGTGAGATTTTTTTTTATTGAATCCAATTTCAATTCACGCTGTAACAAAAA
AAAAGTGGAATAAGTCAAGGGTTATGAATAGTTTCTGAAGGAACTGTATACAAAGCTGTGTACTTGCTCAATAAACATTT
ATTTTATTTTATTTGCTCCGTTTGGTGGGTGTGGCGGGGTGTGGCTTGAACAATAAGTGATGTATTTAAAGGGCAGTGGC
CATGCTGACAGCGTTCCCCAACCCCGTTTTTCAACTGGTCGCTCAGAACAGCACCTTTTCCGTTTAATTGAACATTGCAC
ATCGTTTTGTCATATTGAACGCAGCCCTAGTGTCTGCTGAGTACTTCCTTATGCCATTGAAAATCTTAGACATTGGTCGT
CCTTATGTCATTGAAAATCTGAGACACTGGCCGTGTCTGAATACCCATACTTGCTAAATAGTAGGCATTTTGGGTATGTG
AAATATAACGTTTTATAGTAAGCCTATGTGAAATAAGAATACATTTAGTATGCTTTAAAATGCCAGGATGTCATACTCCA
AGGTTTCCTAAACTCGGTCCTTGGGACCCCGTTTTTTGTTGTTGTTGCCCTGGCACTACACAGTTGATTCAAATAATCAA
CTAATCATCGAGCTTTGATAATTTGAATCAGCTGTGTAGTGTTAGGGCAAAAACCAAAACATGCACCCCTTGGGGTCCGG
AGGACAGAGTTTGGGAAACGCTTTCATATTCTTTTAGGCTTTTCCTCTAGTTGAATTCTCTGCACACTATTGAGGAAGAT
AATCTGCTTACTGCATTTGATTTTACTAAACAGTACGTTCTAAATAGTATGTACTATGATTAGTAAACAGTATGCTGTTT
TAGTAAGTAGTAGGCAAGACAGATTTCGGACACAGCCAATATCCACAGGAAAGTAGGCCTTAAATCGACTTCCAAACGCG
GATCTGTGTGTGGCTCTCATGGTTTGTTTGAGCAAACAATGTAAATACATACACACAATGCATGCATATACTAGCGGGGC
CGGATCCCCACATCTGGGGTCTTGGGCACCAACCTCCAGGGGGCCCCACAACCAGGGGTTGGAACCAGAATTATTTTCCA
ATTCTGAACAGAACCACAATTTTTTTGTTCCGTTCCACTGTTCCAACCAGCAAAATAAAGTTCTGAACCAGTTCAAACTC
CCCAAAACTACCGGTTTATATCGTTCCTTACTGTTCCTTTTTTAACCTGTGATATCAACAGTTTTTTACATTTAGCTCAT
TAAATTACTTGACCAATCAATAAGGATAGAGCAGGCAAGGTAGTTGTTTACATGTGTGATGGACAGACAAGTGTAGACTA
TGGCGCAAGATGCGACTGACATTTTGTTGGTGGGGAGGGGAGAGAGCGAGAGAGGGTAGTGAAGGAGGCTTGAAGTGCTG
GGCATCTTGTTATGACATGCATTATCTGAATTAGGTCCACTGAATTATACCTAAGAGGGCCGGCTTCTATGAAAGAACTT
TGAATGTCCTTTGAGCTAGCTAGCTAACAAGCTTGAGCTAGCTAGCTAACAAGCTTGTGTGTGCAGAGTGGCACCAGAAT
TAAAAACACATCTTGCCTTTTTGTAGTTAATAAATCCTTTGTGAAACATTCTAATTAGGCCATTTGAATCCTTAACTAGC
ATTGAAAAAGTGAATTGATTCTTCTCTAGCTAATAAAAAATCTCTCTTATCTTCTGAATCACGATTCTAACAACAGTACA
GTAGCCTATGCTTTGGAAGGGGGATGGGCAGGTAGCCTAAATACACACACTTACAAAGATTTTCAGCTTGCAGGCAGACA
CTGGAATAAGTTTCTGAGTGACAGAGTGAGAGCTTTGCATAGGCGCTTTACTGCGTTTTCTTGTGGGACTTCACTGTGTG
CACAATTATTAGGCAAGTCAGTATTTTGACCATATCATCATTATTATGCATATTTCCCAACTCCAAGCTGTATAAACTGG
AATGCTTATTGGATTTAAGCATATCAGGTGATGTGTATTTGTGTAATGAGGGAGGGTGTGGCCTAAGGAGATCAACACCC
TATATCAAGGTGTGTAGAATTATTAGGCAGCTTCTTTTCCTCAGGCAAAATGGGCCAAAAAAGAGATTTAACTGACTCTG
AAAAGTCAAAAATTGTAAAAAGTATTTCAGAGGAATGCAGCACTCTTGAAATTGCTAAGATATTGGGGCGTGATCACAGA
ACCATCAAACGTTTTGTTGCAAATAGTCAACAGGGTCGCAAGAACCGTGTTGAGAAAAAAAGACGCAAATTAACTGCCAA
AGATTTGAGAAGAATCAAACGTGAAGCTGCCAGGAACCCATTATCCTCCAGTGCTGTCATATTCCAGAACTGCAACCTAC
CTGGAGTGCCCAGAAGTACAAGGTGTTCAGTGCTCAGAGACATTTCCAAGGTAAGGAAGAATGAAACCCGACCACCACTG
AACAAGACACATAAGTTGAAACATCAAGACTGGGCCAAGAAATATCTGAAGACAGATTTTTCAAAAGTTTTATGGACTGA
TGAGATGAGTGACTCTTGATGGACCAGATGGATGGGCCCGTGGCTGGATCAGTAACGGCAAGGAGTTCCACTTCAAGACG
CCAGCAAGGTGGAGGTGGGGTACTGGTATGGGGTGGTATTATTAAAGATGAGCTAGTTGGACCTTTTTGGGTTGAAGATG
GACTCAAACTCAACTCCCAAACCTACTGCCAGTTTTTAGAAGACACTTTCTTCAAGCAGTGGTACCGGAAAAAGTCTGCA
TCTTTCAAGAAGTCAGCATCTTTCTAAGGCTAGCCAGTAAAGGCCTTAAAGATGAAAGAATAATGACATGGGCACCCCTT
CCTCACCTAACCTAAACCCTATTGAGAACTTGTGGTCTCTTCTTAAACAGACGATTTACGGTGAAGGAAAACAGTACACC
TCTCTGACCAGTGTCTGGGAGGCTGTGGTTGCTGCTGCACAAAAAGTTGATCGTCAACAGATCAAGAAACTGACAGACTC
CATGGATGGAAGACTTATGACTGTTATTGAATAGAAGGGTGGCTATATTGGTCACAGATTTTGTTTGTTGAAATGTTTAT
TTATACATTTTGAGTTGTTTGTTTATTATTCTCACTTTAACAGATGAAAATAAACAAGTGAGATGGGGAACTTTTTGTTT
TTCATTTAGTTACATAATAATTCTGCACACTAATAGTTGCCCAATAATTGTGCACACATAGATATTCTCCAAAGAAAGCC
AAAGCCTCACTTTTACTTTCTTAAATATTCAGGTTTGAGGTTTATTCAGGTTTGTAGTTGTTCAAAAAATAAAATTAATC
CTCAAACATACAACTTGCCTAATAATTGTGCACACAGTGTAGTAAAAATGCCTGCAACTTAAAAGAAGGTTATTAACTGG
GTTCTTGTGCTTTTAAAATAACAGTTATGTTCCGGAATAGTATGGATTACTTTTGTATGTTTTATTTTGTTTCTTGAAAA
TGTTTGCTATTTTTCTGGTTTTCGGGGGTCCTGCAATTCTACACATTTTGTCATGGCTTATGATGTGAGCAAAAGACAGT
TTAATAACACTTTCCTGTGGATATTTTGTCTAAAATTTCCAGCATAATGACCAACCACCAAGACAACCAGTAGAGTAAGT
TCAAATGTAATAGTATTCAACATTCTGGCTTGCAGAGGTCCTGAAAGGACCTTTCCAAACAATTTTTTTGCATTATGTCA
GACGGGGACTAGCTAGCAAGCTAGCACCGAATGATGTATCAGGCTACATACAGTGCTTTCCGAAAGTATTCATACCCCAT
GACTTATTCCACATTTTGTTGTTACAATCTCTTTTTTTTTTTCTTCCAATACAATCTTTTTTCTCTCACCCATCTACACAC
AACTCATAATGACAAAGTGAAAACATGTTTAAAAATTGTTGCACATTTATTGAAAATGAAATACAGAAATATCAGATTTA
CATGTGTATTCACGCCACTGACTCAATACAAGTTAGAATCATGTTTGGCAGCGATTACAGCTGTGAGTCTTTCTGGGTGT
GTCTCTAAGAGCTTTGCACACCTGGATTGTAGAATATTTGCACATTATTCTATTTGAAATTCTGCAAGCTCTGTCAAGTT
GGTTGTTGATCATTGCTAGACAGCCATTTTCAAGTCTTGCCATTGATTTTTCAAGCCAATTTAAGTCAAAAATGTAACTA
GGCCACTCGGAACATTCAATGTCATCTTGGTAAGCAATTCCAGTGTATATTTGGACTTGTTTTAGGTTATTGTCCTGCTG
AAAGGTTAATTTGTCTCCCAGTGTCTGTTGGAAAGCAGACTGAACCAGGTTTTCCTCTAGGATTTTGCATATGCTTAGCT
CTATTCCATTTCTTTTTATCCTAAAAAAACGCTCTAGTCCTTGCTGATGACAAGCATAACCATAACATGATGCAGCCACC
TCCATGCTTGAAAATATGAAGAGTGGTACTCAGGGATGTGATGTACTTGCCCCAAAGATAACACTTCGTATTCAGGACAT
AAAGTTCATTTCTTTGCCACATTGTTTTTTTACAGTTTTAGTTTAGTGCCTTATTGCAAATATTTTTATTCTGTACGGGC
TTCCTTCTTTTCACTCTGTCATTTATGTTAGTATTGCGGAGTAACTACAATGGTGTTGATTCATCATCAGTTTTCTTATA
TCACAGCTATTGAACTATGTAACTGTTTTAAAAACACTATTGGTCTCATGGTGAATCTCCCTGGTCCTTCTGGTTGAATC
TCTGTTTGAAATTCACTGCAGAGACTGAGGGACCTTACAATCATTTGTATGTGTGGGGTACAGAGATGAGGTAGTCATTA
AAAAATAATGTTAAACACTATTATTGCAACTTATTATGTGACTTGTTAAGCACATTTTTAATCCTGAAATAATGTAGGCT
TGCCACAACAAAGGGGGTTGAATATATTTCTAAAAACATAATTCCACTTTGACATTCTGGGGTATTGTGTGTAGGCAGTGA
CACATCTCAAATTAATCCATGTTTTATTCAGGCTGCAGCACAACAAAATGTGGAAAAAGTCAAGGGGTGTGAATACTTAC
TAAAAAGACTAGTCTTCATTTTTATCTCACAGGAAATGCACCAACAGCATCAGCAAATATTTGCAAACATGATCGGTTAT
GGTAATGTCGACTACTATGATCTGACTGGCATACTGCGTCTTTGGTTGTCTAGTGATGATCTAATGATGTTTCTGTAACC
TACAGTATATCTCATGATATGGACCCCAGAATAGGCCTCATCAACCATGAAATGGCTAATAGGCCTTTGTAAGAGTTTCA

AGATATTTTTCACTGTGGTGACTGTTCTGTAGGAATTATACAAAAAATACATATCACATCTTAATTTAAGAAGTTAATTA
TTTGTTTATTATTCATTTATTTAAGTATTTCTATCTCCAAGATTCACCCACCCGAAAAATATAAATTCCGGTTTATTCAA
AAATGAAATGAAGGAATTGTTATTGTCCGCATCAAAGGAATGAATCAACAAAATCAAAGTGGCAATAATCATAATCATTA
TTACAATAATAATTAAATAATCAGAATATCCACATTCATAACAAATCCATTTACAATGGCAATACACTCTTCAATATCAC
TCTTTCCATTAATATACACAAATCCCCGAATGTCTGTCCTTTTTAAAACAAAAAAAGAGGGGTTGGGGGACTATGGCTTG
TCAAGGGGTATGACTATGTGTGACTTGTGGGGTCGAAAGTTCAAGGGTGAGGGAGGGTTGGCGAGTTGGAGGCACACTTA
ATAGAACTTGAAGAAATGGCTAATAACATGGTGTTACTTGCATGGAAGAGTTGCTTCACAAGTAACAGTGTGATCTGAAC
CAAACGTTCAGCCTGACAGAGGGGTCTATGAGAGGCAGAGTTCGGGGAACTAAATTGGAAAAATTAGATAAGGGTTGTCT
GGATAAATATATAAATCTGAATGACAACCATTGCAAACATTTAATGTAGAGAAATGTAAAACTTAAATCAAATAACAACG
AAGAATCATAATAGTTACAGGAATTCAAAGAAAAGGAGATGGAGGTCACTGGAAATATTATCTTAGTTATTCTATATACT
GTATATGTACAAACATTGCTTGGTCACCCAAATATAATTTTTAAAAATGCCTCAGTTGATGAGAAAATGAGTGCAAGCCA
GAGATTGTTTTGAGAGGCATAATTATGTGTGCAGGTGTTCAGAGGTGTCAGATTGGTCAGTGTCTGCACACAAAGTGCTT
CCCAGACAGCTGTCCTTTAAATTGCCAGTGTGAACCTCTCCACTCCCCAACATTTATTCTTTGAGGAGTCAAATACGTCA
TTTAAGAGAGAGAGAGATTAGAGGATTGAGGAAAAATCTGTACACCCTGCATATACAAACAAACTGTATATACACATAGA
CATACCATATATACACACACATATCCAGTATATACACAACAACTGTACACACACTGTATATACACACACATTCAATCACT
TAAAAGGTATTAACCTCTAATCTCTCGCTCTTTTGACGTGTCATTTTTCAGTGGTCAGAGGTGAAGGAGGGCACAGGGTT
TTATTTAGCTTCTGTGTCAGTGGAGTCCATACAAAGAAATCAAATGGAAGTTATACAGAAACTTGGCTCCAGAATTCGGC
CTCACCACGCTGGAAAAACATGATAGACTCGCATCTGCTCCACTAAGCTTACATCAGAGACAGAAATAGGAAAACAAAAA
CGTGACGTTAAGGATGTCCTCTTCTAAGGAGTTTTAGATCGTCTGTAAAGTGGAGAGCTCCCCAGCGATTGTCAAGTAGA
AAAAACAACATCCATCCTCATATCTCTCTTCTAGGGATTACAAGAGAGAAGTGGAGTGGTACAGGTCACAGCAACGTGCA
AATCATTGTGAGGGGAGCAGGTAAAACAACAGTTTGTGTTTTTATCCGTCACAATCCCAGGGCAATCGTTCTTCACAAAA
GGGTAATGTTCCATAAGATTGTCTTAAGAAAAATCAGTTGATATAAAATGTATTATTTCTGGACTGTCATTAGATACTAG
TCTTGTCATTATCAAGAAATAAACATTTGCGCAGGAAGATTTCCAATGAATAGTTTTTGTAGTACTATGGTAAGAATAAG
CGTTATTATAGAAATATAAATCATAGCATTGTCTACATTTTTCAAAGTTTTAATTGAAGCAATATTTTGTTTTGTAGCAG
TTTCACTTGTAATAGGCTGTGCATAATTAGTTTCCTTTAAATGTAAAACTAATAGAGATAAAGAAGAAAGAACAAAACCA
AAACCATGGCAGATATGAACAGGAGCAGAAAAAGTCTAAAAGTGAACATAAAACGGAGGTGGGTGGTTAAGAATAGATAG
AAAGAGGAATGGGTAGATAGTGGGTGGACTATAGAAAATGGTAGGAAAGAAAAATGAAAGAAATTGTGAGATCTTAGAAA
AATGTTAGGAAAGAAAAATTACAGCACCTTTGAAATGCATCTTACACACAGCAGCTCTCCCGCTTTCATTTGACTTACTG
CTCAATCCCAAAAGGTTTATATCATGTCAATCTGCATAGCTGTTCTTCACATGCTTGATGAGCCAACAAAACCATTTCCC
GAGATTGGCACGTCTTTAAAAGAAAATCAAATATCTAATCACAAGCGTCAAATACAGATTGTCCAGCCATCCAATCAGAG
AGAGAGAGAGCCCAAGTAAAGTAGGGTAAATAACACAGCAGATCCTTACATGGTTAGCTGTGGCTCTGGACTATCCTTTA
GGTCAGGAGAATTGGACCAATGGCCCAACTTCTCCCTTTGTCAGCCAATCGCATCACGAGAGAGCGCTCTTGATATATGG
CCAGGCCATGCGGGCTGCCCATGTTGCCTCAGCCAATTAGGTTCCAGATTGTCCTCCACGAAGCTGCGTGATTGGCTAAG
GGGAGAAAAGGAAGACAGAGAGAGAAGGAACGGGGAGGTGCTTGATGAGTTGCTATCCAATCATAGCCATCCCACTAGGC
ACAGACGTCATTTCAACGTCTAATTTTGATGTACATTTGATTGAGTTGTCAACTAAAGTGAATTTAATCAATGTCATTGG
ATTTAGGTAAAAATTTGGGTGAAAAAATACTAAATTCCCTAACGTCCCTAATGAAATTCCATAACTATTAGCCTTTTTGT
TTAAATGATGTGGAATCAACGTTGATTCAACCAGTTTTTGCCCAGTGGGATAGATCTTGTAATAGATGGACCAACCACTA
TATCAGATGGCAGGCACTCCAAAGTAGGATTAATCTCTTTGTCAGTTTGTGTTCTGCTCTGAGGCAAATATTTGTTTACC
TGAGTCTCTCAATTCTGCATCTCTGAGGTATATTAATGGCAATGCTCATATATCAACCAGTGACCCTGTATAGTCCCATA
TCTTTACTCAATGCAGATGTAAAACCCTAGCTGCTTTAAAAGCCATTTTATTACTGATATGTTTTGGCACTCGAAAGGTA
GCCATATTAGCTGACTTAACACAAGTTAACTACAACAAACTCTCCCTATTAATAATCACATTTGGTTAACTTTTAATTCA
ACACTCATAGCTAAATCTCTCTCATATATCTGAATCTCATCCACTTTACATTATGGCTAATCCCCTAGTACTGACCGACA
CAGCATTGGCGGAGGGCATTTTGACCCTATACACAAAGTGCCGAATCCTAACTTGAGATCTGCATGTTAAACTCAGACTT
ACTCGTAATGCATGACGCCAATGGGAGAACTGCAAAAATATAAATCAAGTTAGGATTTGGCCCTTAGTAAATTGACTCTT
AATCAAATAAAAAAGTGAAATAAGACTGGTGTTGGGTTCTGCAAAACCAGGACTGTCTGAAGCATGTTAATTCACTTGAG
ACGCTAAGCCAGAGTAAGGAAAGTTTTCCAACTAGTTATAAAGCACCACCAGTGTGAATCTAAAAGGGCCAAAATAAACC
CAAATTCACATCTAAACAAAGATTACGATGGACAAGTTACACACAAAACAAGAGGACATGACAACACAGTATATCTGAAC
AGTTGACAAAGCAATGAATTCCTTTTAGTGCACTGTGTAACATGTTTTAGATTTGAAATACTCTTTACTAACGATGCCCC
AAACAATCTTTGTTTTCTCAAAATGTTGTTACATTAGCATTCATCATGCTCAGCTGAATGGTACGTTAACTTAAAATAGT
CCCTCGAGACGACATGAAGAAAGTCTATGCCACATAAAAATTTGTCTGAGCGCTGTTTGACTACTGGCCAAAATGTCATT
ATCTGAGACCCAGGCACTTAAGGCTAGACTTAACTGGCTACCCCAAAATAAGAATCAAATAAATAGTTTATGTGGTACTA
AACAGAAAAAGCCATGACAACAATTTTGGAAGTGTACTAGGTGTTACAAGTGACTACGTTATATGCCTGGACACTGAAGG
TCCACAAATAATATCCCTTTGATATTTTCAACCACTTAAAATTGCTGGTAAACTTTTTACCTGACAAGTTTGTTATTGTC
TCATTTGGTTTAAGCACCCTACTTGACCCAGCTCCCACTCCATTAAACAATTGCACACATAAGGACACGACACACACATA
ACAAACAAACAAAGACAAATACATTAAAGAAATAGGCTACCTTGACAATAACAGACAGAGTGAGAGAGACAGACATACAG
ACAGTGGGAAACATGAGAGGCAGTGCTATGACACACACACTTGATTTGGAAATGGCACTGTTTGTGTCTGTAGCACCAGT
TGATCACACATAGGTTCTGAGATTGAGAAGAGAAAACATTGCACGAGTCCAGTCCACGAATGGGATGTCCCTTAATTAAC
ACGTTTTTTGAAAACATCACACACTTTTCTTAGGCCATAGGGCATCTCTATGAAATCCCCAAAATGACACCGTATTCTGC
TTGTTCTGGACTCTGCAGTTAGTCTGTATTTCAAACAGCATAGTTATTTTTGGATCTTGATTAGACTGTTTATGTACTAT
CATCAGTTAAAATAATCCTTCTGTAAATATCATCTCTGCCTGTTATTTGAAAACCCTGTTAAAATACACTCAAAGGCACT
AAGGAAACCAGACTACAAAGCCAGATGACCACATCTCTACCAATTCAATTTGAGAGCTGGAGTTTTCAGGACAGGAAATG
ACTAGACCTTTATCGATGGAATTATGGGATACTACACGCATGTATATAGTAGGATGCTATTGGCTACTGAGAAGTTTTTG
GGGAGTGAGACGTTATATAGTGTTTGCTTACTGTCTTTATTTGATTAAACAAACACACAAAGCAAAATCTCATATAGTCG
CTTGGTTTGGGGATTAGGCCTATACCAGTGTATATGATAGGCTCTATAGTTTGATCGATTGAAACAGTACATGAGGATCC
CACAATGTCAACGATAACTGAAAGGTATCATTATCTTGTCCTGCAAGACAGATACTGACCAAAGCCCAGTTTGAGCATGA
TGGAAATGAGGTCCATTGGAAAGCCCTTTTGTTGTCTTGTATGATAATGCTGAACGTACTGTACAGTTAAATGTACAATG
ACTGCTGCCCCCCACACATTTCTTCTCTCTCTGGTTTGAGTACACATAAAAGAAACGGGAAAGATTGCAGTCATCTCATC
CTCCTCAAAAAAGGAAGGAAATGTGGCAAGACTTTTTTATTTGTGTCTTCTCTCTCTGATTTGGTTTAGTGTTGTTTTTG
ACCTGCTCCCTCTCCTCTATGCCCAGTCCTGCTGTTGCAGTGCTCTCAGTTGCCTCCGTGCTTGTCACTGGGGAGGATGT

TGGGTTGACACGGAGCTCTCCATTCTGGGGTGGACTGGGGTGGAGATGAGGAGTGGGGGGAGGAGGAGGGTCTACGGGTG
GGGAGGGGCCTCCATTGTCGTTCCGGGGCAAGCCAGTGGAGGAGATGCCCGAGGAAGAGATAGGGTCAGGATCAGTAGTC
ATCCTTCCTTCTTTTTCTCCTTCTTCTCCTCCTCCTCCTCCACCTTCAGTATTCACACAGGGGTCTGCTGAACCCAGGGA
GGAATGCTGGGCCCCCTAGCAAGACAGAGAGAGGGAGGGAGAAGAGGAGTAGGAGAGGGGAGAGATGGGAGTGGGAGAAG
GGGCATTCAGTGGGGGATTTGTGGCATGGACAAGGGTGTAAACGAGTCAGCCAATCCAGGAAAGAGAGAGTGAGGCAATA
TAAAGAGTCATATATGAAGCAGGAAATGTGGGGAGAGAAGGCAACAGGGAGGATGGAAGGAGAGGGAGTATGGGTGATAT
AAACAAGCAAGGAATAGTCAAATAAAAAGAGGAGGCCAGTGAAGAACAAACAGGTGGGTAGAGGAGAGAGTAAATAGGNN
NNNNNNNNNNNNNNNNNNNNNNNNNNNNNNNNNNNNNNNNNNNNNNNNNNNNNNNNNNNNNNNNNNNNNNNNNNNNNNNN
NNNNNNNNNNNNNNNNNNNNNNNNNNNNNNNNNNNNNNNNNNNNNNNNNNNNNNNNNNNNNNNNNNNNNNNNNNNNNNNN
NNNNNNNNNNNNNNNNNNNNNNNNNNNNNNNNNNNNNNNNNNNNNNNNNNNNNNNNNNNNNNNNNNNNNNNNNNNNNNNN
NNNNNNNNNNNNNNNNNNNNNNNNNNNNNNNNNNNNNNNNNNNNNNNNNNNNNNNNNNNNNNNNNNNNNNNNNNNNNNNN
NNNNNNNNNNNNNNNNNNNNNNNNNNNNNNNNNNNNNNNNNNNNNNNNNNNNNNNNNNNNNNNNNNNNNNNNNNNNNNNN
NNNNNNNNNNNNNNNNNNNNNNNNNNNNNNNNNNNNNNNNNNNNNNNNNNNNNNNNNNNNNNNNNNNNNNNNNNNNNNNN
NNNNNNNNNNNNNNNNNNNNNNNNNNNNNNNNNNNNNNNNNNNNNNNNNNNNNNNNNNNNNNNNNNNNNNNNNNNNNNNN
NNNNNNNNNNNNNNNNNNNNNNNNNNNNNNNNNNNNNNNNNNNNNNNNNNNNNNNNNNNNNNNNNNNNNNNNNNNNNNNN
NNNNNNNNNNNNNNNNNNNNNNNNNNNNNNNNNNNNNNNNNNNNNNNNNNNNNNNNNNNNNNNNNNNNNNNNNNNNNNNN
NNNNNNNNNNNNNNNNNNNNNNNNNNNNNNNNNNNNNNNNNNNNNNNNNNNNNNNNNNNNNNNNNNNNNNNNNNNNNNNN
NNNNNNNNNNNNNNNNNNNNNNNNNNNNNNNNNNNNNNNNNNNNNNNNNNNNNNNNNNNNNNNNNNNNNNNNNNNNNNNN
NNNNNNNNNNNNNNNNNNNNNNNNNNNNNNNNNNATTTATACAACCATCTCTTAGGTTACTACTGTAGGCACATTTAACA
CTTTCTCTTAACCCATGGTGCCATTAAAACATTGCTGATATAGTCACACTATAAACCGTCTTGCTTTAATAAAATGCCCT
GGTCTATTAAATATTGAGTACCACGGTATCACCGGCGTGTTTTGGACTTCTAACCCCTTTAATACATTGTGTGTTTGAGA
CTGCTGGGTTGTACCATTGGATTTGTCTATTTCTTCTGCATCCACTGGTACCAATCATTTGGGGCTCCCGAGTGGCGCAG
TCTAAGGAACTGCATCTCAGTGCAAGAGGTGTCACTACAGTACCTGGTTTGAATCCAGGCTGCATCACATCCGGCCGTGA
TTGGGAGGCCCCTAGGGCGGCGCACAATTGGCACAGAATCATCCGGGTTTGGCCGGGGTAGGCCGTCATTGTAAATAAGA
ATTTGTTCTTAACTGACTTGCCTAGTTAAATAAAGGTTAAATTAAAAAAAACGTATGTAAAGTCATAACGGTTTGAGCTA
TAAACTAGTATGACATATCAAGACTCTCACGAACACATACAGTGAGGGAAAAAAGTATTTGATCCCCTGCTGATTTTGTG
CGTTTGCCCACTGACAAAGAAATGATCAGTCTATAATTTTAATGGTAGGTTTATTTGAACAGTGAGAGACAGAATAACAA
CAACAAAATCCAGAAAAATGCATGTCAAAAATGTTAGAAAATGATTTGCATTTCAATGAGGGAAATAAGTATTTGACCCC
TCTGGAAAACATGACTTAGTACTTGGTGGCAAAACCCTTGTTGGCAATCACAGAGGTTAGACGTTTCTTGTAGTTGGCCA
CCAGGTTTGCACACATCTCAGGAGGGATTTTGTCCCACTCCTCTTTGCAGATCTTCTCCAAGTCATTAAGGTTTCGAGGC
TGACGTTTGGCAACTCGAACCTTCAGCTCCCTCCACAGATTTTCTATGGGATTAAGATCTGGAGACTGGCTAAGCCACTC
CAGGACCTTAATGTGCTTCTTCTTGAGCCACTCCTTTGTTGCCTTGGCTGTGTGTTTTGGGTCATTGTCATGCTGGAATA
CCCATCCACGACCCATTTTCAATGCCCTGGCTGAGGGAAGGATGTTCTCACCCAAGATTTGACGGTACATGGCCCCGTCC
ATCGTCACTTTGATGCGGTGAAGTTGTCCTGTCCCCTTAGCAGAAAAACACCCCCAAAGCATAATGTTTCCACCTCCATG
TTTGACGGTGGGGATGGTGTTCTTGGGGTCATAGGCAGCATTCCTCTTCCTCCAAACACGGCGAGGTGAGTAGATGCCAA
AGAGCTCCATTTTGGTCTCATCTGACCACAACACTTTCACCCAGTTGTCCTCTGAATCATTCAGATGTTCATTGGCAAAC
TTCAGACGGGCATGTATATGTGCTTTCTTGAGCAGGGGGACCTTGCGGGTGCTGCAGGATTTCAGTCCTTCACGGCGTAG
TGTGTTACCAATTGTTTTCTTGGTGACTATGGTCCCATCTCCCTTGAGATCATTGACAAGATCCTCTGTGTAGTTCTGGG
CTGATTCCTCACCGTTCTCATGATCATTGCAACTCCACGAGGTGAGATCTTGCATGGAGCCCCAGGCCGAGGGAGATTGA
CAGTTCTTTTGTGTTTCTTCCATTTGCGAATAATCGCACCAACTGTTGTCACCTTCTCAACAAGCTGCTTGGCGATGGTC
TTGTAGCCCATTCCAGCCTTGAGTAGGTCTACAATCTTGTCCCTGACATCCTTGGAGAGCTCTTTGGTCTTGGCCATGGT
GGAGAGTTTGGAATCTGATTGATTGCTTCTGTGGACAGGTGTCTTTTATACAGGTAACAAGCTGAGATTAGCAACACTCC
CTTTAAGAGTGTGCTCCTAATCCCAGCTCGTTACCTGTATAAAAGACACCTGGGAGCCAGAAATCTTTCTGATTGAGAGG
GGGTCAAATACTTATTTCCCTCATTAAAATGCAAATCAATGTATCACATTTTTGACATGCGTTTTTCTGGATTTTTTGTT
GTTGTTATTCTGTCTCTCACTGTTCAAATAAACCTACCATTAAAATTATAGACTGATAATTTCTTTGTCAGTGGGCAAAC
GTACAAAATCAGCAGGGGATCAAATACTTTTTTCCCTCACTGTGGCTACTTTGCAACTACATAGCATGTTAGCTAACCCT
TCCTCTGCTCTTTCACCTTACTCCTGACCTTAACCTTAACCCCTAGCCTAACTAACATTAGCCACCTAGCTAACGTTAGG
GTTAGCCACCTACTTATAACGTTAGCCACAACAAACTGGAATTCTTAACATATCATACATATTGCAAATTTGCAGCATAT
TTTATGAATTGTAATATATCATACGAAATGGATGATGGACATCCACATATTAATACATGCCATATCAAACATAACGATCA
TACTAATTTGAGTGTCCCAGATTTTTGTTTACTATGTTATGTCTACCCCTGACTCTGGGTTGATATTTCAGCTCCAGCAT
TTATTGATTTTATATTACAGTAAAGCTCGAAATTATTCAAATTGCTGTTGACAAAATCAAAATAACCAAAATAATGTTGA
ACAATAGGGTAAAGAAAAGAAAGACTATTGCTTGTTGATAATACGTGGTGATTTTCTGTATAAGACATGTCTGTGTAAGT
CAATAAGTACTTCTGTTGGTGCTGCATACGGTCAGTGACGCACATACAGTACAGTGGAGGAATCAAAGTGAAGAGGCAGA
ATGTATGGATGGAGAGAGGCAGTACCACTGATGTGTATGCTGGTTGGTTTATTACCTTTGTACTCGGGGTGGAACGGGCG
CGAGACGGGCNNNNNNNNNNNNNNNNNNNNNNNNNNNNNNNNNNNNNNNNNNNNNNNNNNNNNNNNNNNNNNNNNNNNNN
NNNNNNNNNNNNNNNNNNNNNNNNNNNNNNNNNNNNNNNNNNNNNNNNNNNNNNNNNNNNNNNNNNNNNNNNNNNNNNNN
NNNNNNNNNNNNNNNNNNNNNNNNNNNNNNNNNNNNNNNNNNNNNNNNNNNNNNNNNNNNNNNNNNNNNNNNNNNNNNNN
NNNNNNNNNNNNNNNNNNNNNNNNNNNNNNNNNNNNNNNNNNNNNNNNNNNNNNNNNNNNNNNNNNNNNNNNNNNNNNNN
NNNNNNNNNNNNNNNNNNNNNNNNNNNNNNNNNNNNNNNNNNNNNNNNNNNNNNNNNNNNNNNNNNNNNNNNNNNNNNNN
NNNNNNNNNNNNNNNNNNNNNNNNNNNNNNNNNNNNNNNNNNNNNNNNNNNNNNNNNNNNNNNNNNNNNNNNNNNNNNNN
NNNNNNNNNNNNNNNNNNNNNNNNNNNNNNNNNNNNNNNNNNNNNNNNNNNNNNNNNNNNNNNNNNNNNNNNNNNNNNNN
NNNNNNNNNNNNNNNNNNNNNNNNNNNNNNNNNNNNNNNNNNNNNNNNNNNNNNNNNNNNNNNNNNNNNNNNNNNNNNNN
NNNNNNNNNNNNNNNNNNNNNNNNNNNNNNNNNNNNNNNNNNNNNNNNNNNNNNNNNNNNNNNNNNNNNNNNNNNNNNNN
NNNNNNNNNNNNNNNNNNNNNNNNNNNNNNNNNNNNNNNNNNNNNNNNNNNNNNNNNNNNNNNNNNNNNNNNNNNNNNNN
NNNNNNNNNNNNNNNNNNNNNNNNNNNNNNNNNNNNNNNNNNNNNNNNNNNNNNNNNNNNNNNNNNNNNNNNNNNNNNNN
NNNNNNNNNNNNNNNNNNNNNNNNNNNNNNNNNNNNNNNNNNNNNNNNNNNNNNNNNNNNNNNNNNNNNNNNNNNNNNNN
NNNNNNNNNNNNNNNNNNNNNNNNNNNNNNNNNNNNNNNNNNNNNNNNNNNNNNNNNNNNNNNNNNNNNNNNNNNNNNNN
NNNNNNNNNNNNNNNNNNNNNNNNNNNNNNNNNNNNNNNNNNNNNNNNNNNNNNNNNNNNNNNNNNNNNNNNNNNNNNNN
NNNNNNNNNNNNNNNNNNNNNNNNNNNNNNNNNNNNNNNNNNNNNNNNNNNNNNNNNNNNNNNNNNNNNNNNNNNNNNNN

NNNNNNNNNNNNNNNNNNNNNNNNNNNNNNNNNNNNNNNNNNNNNNNNNNNNNNNNNNNNNNNNNNNNNNNNNNNNNNNN
NNNNNNNNNNNNNNNNNNNNNNNNNNNNNNNNNNNNNNNNNNNNNNNNNNNNNNNNNNNNNNNNNNNNNNNNNNNNNNNN
NNNNNNNNNNNNNNNNNNNNNNNNNNNNNNNNNNNNNNNNNNNNNNNNNNNNNNNNNNNNNNNNNNNNNNNNNNNNNNNN
NNNNNNNNNNNNNNNNNNNNNNNNNNNNNNNNNNNNNNNNNNNNNNNNNNNNNNNNNNNNNNNNNNNNNNNNNNNNNNNN
NNNNNNNNNNNNNNNNNNNNNNNNNNNNNNNNNNNNNNNNNNNNNNNNNNNNNNNNNNNNNNNNNNNNNNNNNNNNNNNN
NNNNNNNNNNNNNNNNNNNNNNNNNNNNNNNNNNNNNNNNNNNNNNNNNNNNNNNNNNNNNNNNNNNNNNNNNNNNNNNN
NNNNNNNNNNNNNNNNNNNNNNNNNNNNNNNNNNNNNNNNNNNNNNNNNNNNNNNNNNNNNNNNNNNNNNNNNNNNNNNN
NNNNNNNNNNNNNNNNNNNNNNNNNNNNNNNNNNNNNNNNNNNNNNNNNNNNNNNNNNNNNNNNNNNNNNNNNNNNNNNN
NNNNNNNNNNNNNNNNNNNNNNNNNNNNNNNNNNNNNNNNNNNNNNNNNNNNNNNNNNNNNNNNNNNNNNNNNNNNNNNN
NNNNNNNNNNNNNNNNNNNNNNNNNNNNNNNNNNNNNNNNNNNNNNNNNNNNNNNNNNNNNNNNNNNNNNNNNNNNNNNN
NNNNNNNNNNNNNNNNNNNNNNNNNNNNNNNNNNNNNNNNNNNNNNNNNNNNNNNNNNNNNNNNNNNNNNNNNNNNNNNN
NNNNNNNNNNNNNNNNNNNNNNNNNNNNNNNNNNNNNNNNNNNNNNNNNNNNNNNNNNNNNNNNNNNNNNNNNNNNNNNN
NNNNNNNNNNNNNNNNNNNNNNNNNNNNNNNNNNNNNNNNNNNNNNNNNNNNNNNNNNNNNNNNNNNNNNNNNNNNNNNN
NNNNNNNNNNNNNNNNNNNNNNNNNNNNNNNNNNNNNNNNNNNNNNNNNNNNNNNNNNNNNNNNNNNNNNNNNNNNNNNN
NNNNNNNNNNNNNNNNNNNNNNNNNNNNNNNNNNNNNNNNNNNNNNNNNNNNNNNNNNNNNNNNNNNNNNNNNNNNNNNN
NNNNNNNNNNNNNNNNNNNNNNNNNNNNNNNNNNNNNNNNNNNNNNNNNNNNNNNNNNNNNNNNNNNNNNNNNNNNNNNN
NNNNNNNNNNNNNNNNNNNNNNNNNNNNNNNNNNNNNNNNNNNNNNNNNNNNNNNNNNNNNNNNNNNNNNNNNNNNNNNN
NNNNNNNNNNNNNNNNNNNNNNNNNNNNNNNNNNNNNNNNNNNNNNNNNNNNNNNNNNNNNNNNNNNNNNNNNNNNNNNN
NNNNNNNNNNNNNNNNNNNNNNNNNNNNNNNNNNNNNNNNNNNNNNNNNNNNNNNNNNNNNNNNNNNNNNNNNNNNNNNN
NNNNNNNNNNNNNNNNNNNNNNNNNNNNNNNNNNNNNNNNNNNNNNNNNNNNNNNNNNNNNNNNNNNNNNNNNNNNNNNN
NNNNNNNNNNNNNNNNNNNNNNNNNNNNNNNNNNNNNNNNNNNNNNNNNNNNNNNNNNNNNNNNNNNNNNNNNNNNNNNN
NNNNNNNNNNNNNNNNNNNNNNNNNNNNNNNNNNNNNNNNNNNNNNNNNNNNNNNNNNNNNNNNNNNNNNNNNNNNNNNN
NNNNNNNNNNNNNNNNNNNNNNNNNNNNNNNNNNNNNNNNNNNNNNNNNNNNNNNNNNNNNNNNNNNNNNNNNNNNNNNN
NNNNNNNNNNNNNNNNNNNNNNNNNNNNNNNNNNNNNNNNNNNNNNNNNNNNNNNNNNNNNNNNNNNNNNNNNNNNNNNN
NNNNNNNNNNNNNNNNNNNNNNNNNNNNNNNNNNNNNNNNNNNNNNNNNNNNNNNNNNNNNNNNNNNNNNNNNNNNNNNN
NNNNNNNNNNNNNNNNNNNNNNNNNNNNNNNNNNNNNNNNNNNNNNNNNNNNNNNNNNNNNNNNNNNNNNNNNNNNNNNN
NNNNNNNNNNNNNNNNNNNNNNNNNNNNNNNNNNNNNNNNNNNNNNNNNNNNNNNNNNNNNNNNNNNNNNNNNNNNNNNN
NNNNNNNNNNNNNNNNNNNNNNNNNNNNNNNNNNNNNNNNNNNNNNNNNNNNNNNNNNNNNNNNNNNNNNNNNNNNNNNN
NNNNNNNNNNNNNNNNNNNNNNNNNNNNNNNNNNNNNNNNNNNNNNNNNNNNNNNNNNNNNNNNNNNNNNNNNNNNNNNN
NNNNNNNNNNNNNNNNNNNNNNNNNNNNNNNNNNNNNNNNNNNNNNNNNNNNNNNNNNNNNNNNNNNNNNNNNNNNNNNN
NNNNNNNNNNNNNNNNNNNNNNNNNNNNNNNNNNNNNNNNNNNNNNNNNNNNNNNNNNNNNNNNNNNNNNNNNNNNNNNN
NNNNNNNNNNNNNNNNNNNNNNNNNNNNNNNNNNNNNNNNNNNNNNNNNNNNNNNNNNNNNNNNNNNNNNNNNNNNNNNN
NNNNNNNNNNNNNNNNNNNNNNNNNNNNNNNNNNNNNNNNNNNNNNNNNNNNNNNNNNNNNNNNNNNNNNNNNNNNNNNN
NNNNNNNNNNNNNNNNNNNNNNNNNNNNNNNNNNNNNNNNNNNNNNNNNNNNNNNNNNNNNNNNNNNNNNNNNNNNNNNN
NNNNNNNNNNNNNNNNNNNNNNNNNNNNNNNNNNNNNNNNNNNNNNNNNNNNNNNNNNNNNNNNNNNNNNNNNNNNNNNN
NNNNNNNNNNNNNNNNNNNNNNNNNNNNNNNNNNNNNNNNNNNNNNNNNNNNNNNNNNNNNNNNNNNNNNNNNNNNNNNN
NNNNNNNNNNNNNNNNNNNNNNNNNNNNNNNNNNNNNNNNNNNNNNNNNNNNNNNNNNNNNNNNNNNNNNNNNNNNNNNN
NNNNNNNNNNNNNNNNNNNNNNNNNNNNNNNNNNNNNNNNNNNNNNNNNNNNNNNNNNNNNNNNNNNNNNNNNNNNNNNN
NNNNNNNNNNNNNNNNNNNNNNNNNNNNNNNNNNNNNNNNNNNNNNNNNNNNNNNNNNNNNNNNNNNNNNNNNNNNNNNN
NNNNNNNNNNNNNNNNNNNNNNNNNNNNNNNNNNNNNNNNNNNNNNNNNNNNNNNNNNNNNNNNNNNNNNNNNNNNNNNN
NNNNNNNNNNNNNNNNNNNNNNNNNNNNNNNNNNNNNNNNNNNNNNNNNNNNNNNNNNNNNNNNNNNNNNNNNNNNNNNN
NNNNNNNNNNNNNNNNNNNNNNNNNNNNNNNNNNNNNNNNNNNNNNNNNNNNNNNNNNNNNNNNNNNNNNNNNNNNNNNN
NNNNNNNNNNNNNNNNNNNNNNNNNNNNNNNNNNNNNNNNNNNNNNNNNNNNNNNNNNNNNNNNNNNNNNNNNNNNNNNN
NNNNNNNNNNNNNNNNNNNNNNNNNNNNNNNNNNNNNNNNNNNNNNNNNNNNNNNNNNNNNNNNNNNNNNNNNNNNNNNN
NNNNNNNNNNNNNNNNNNNNNNNNNNNNNNNNNNNNNNNNNNNNNNNNNNNNNNNNNNNNNNNNNNNNNNNNNNNNNNNN
NNNNNNNNNNNNNNNNNNNNNNNNNNNNNNNNNNNNNNNNNNNNNNNNNNNNNNNNNNNNNNNNNNNNNNNNNNNNNNNN
NNNNNNNNNNNNNNNNNNNNNNNNNNNNNNNNNNNNNNNNNNNNNNNNNNNNNNNNNNNNNNNNNNNNNNNNNNNNNNNN
NNNNNNNNNNNNNNNNNNNNNNNNNNNNNNNNNNNNNNNNNNNNNNNNNNNNNNNNNNNNNNNNNNNNNNNNNNNNNNNN
NNNNNNNNNNNNNNNNNNNNNNNNNNNNNNNNNNNNNNNNNNNNNNNNNNNNNNNNNNNNNNNNNNNNNNNNNNNNNNNN
NNNNNNNNNNNNNNNNNNNNNNNNNNNNNNNNNNNNNNNNNNNNNNNNNNNNNNNNNNNNNNNNNNNNNNNNNNNNNNNN
NNNNNNNNNNNNNNNNNNNNNNNNNNNNNNNNNNNNNNNNNNNNNNNNNNNNNNNNNNNNNNNNNNNNNNNNNNNNNNNN
NNNNNNNNNNNNNNNNNNNNNNNNNNNNNNNNNNNNNNNNNNNNNNNNNNNNNNNNNNNNNNNNNNNNNNNNNNNNNNNN
NNNNNNNNNNNNNNNNNNNNNNNNNNNNNNNNNNNNNNNNNNNNNNNNNNNNNNNNNNNNNNNNNNNNNNNNNNNNNNNN
NNNNNNNNNNNNNNNNNNNNNNNNNNNNNNNNNNNNNNNNNNNNNNNNNNNNNNNNNNNNNNNNNNNNNNNNNNNNNNNN
NNNNNNNNNNNNNNNNNNNNNNNNNNNNNNNNNNNNNNNNNNNNNNNNNNNNNNNNNNNNNNNNNNNNNNNNNNNNNNNN
NNNNNNNNNNNNNNNNNNNNNNNNNNNNNNNNNNNNNNNNNNNNNNNNNNNNNNNNNNNNNNNNNNNNNNNNNNNNNNNN
NNNNNNNNNNNNNNNNNNNNNNNNNNNNNNNNNNNNNNNNNNNNNNNNNNNNNNNNNNNNNNNNNNNNNNNNNNNNNNNN
NNNNNNNNNNNNNNNNNNNNNNNNNNNNNNNNNNNNNNNNNNNNNNNNNNNNNNNNNNNNNNNNNNNNNNNNNNNNNNNN
NNNNNNNNNNNNNNNNNNNNNNNNNNNNNNNNNNNNNNNNNNNNNNNNNNNNNNNNNNNNNNNNNNNNNNNNNNNNNNNN
NNNNNNNNNNNNNNNNNNNNNNNNNNNNNNNNNNNNNNNNNNNNNNNNNNNNNNNNNNNNNNNNNNNNNNNNNNNNNNNN
NNNNNNNNNNNNNNNNNNNNNNNNNNNNNNNNNNNNNNNNNNNNNNNNNNNNNNNNNNNNNNNNNNNNNNNNNNNNNNNN
NNNNNNNNNNNNNNNNNNNNNNNNNNNNNNNNNNNNNNNNNNNNNNNNNNNNNNNNNNNNNNNNNNNNNNNNNNNNNNNN
NNNNNNNNNNNNNNNNNNNNNNNNNNNNNNNNNNNNNNNNNNNNNNNNNNNNNNNNNNNNNNNNNNNNNNNNNNNNNNNN
NNNNNNNNNNNNNNNNNNNNNNNNNNNNNNNNNNNNNNNNNNNNNNNNNNNNNNNNNNNNNNNNNNNNNNNNNNNNNNNN
NNNNNNNNNNNNNNNNNNNNNNNNNNNNNNNNNNNNNNNNNNNNNNNNNNNNNNNNNNNNNNNNNNNNNNNNNNNNNNNN
NNNNNNNNNNNNNNNNNNNNNNNNNNNNNNNNNNNNNNNNNNNNNNNNNNNNNNNNNNNNNNNNNNNNNNNNNNNNNNNN
NNNNNNNNNNNNNNNNNNNNNNNNNNNNNNNNNNNNNNNNNNNNNNNNNNNNNNNNNNNNNNNNNNNNNNNNNNNNNNNN
NNNNNNNNNNNNNNNNNNNNNNNNNNNNNNNNNNNNNNNNNNNNNNNNNNNNNNNNNNNNNNNNNNNNNNNNNNNNNNNN
NNNNNNNNNNNNNNNNNNNNNNNNNNNNNNNNNNNNNNNNNNNNNNNNNNNNNNNNNNNNNNNNNNNNNNNNNNNNNNNN

NNNNNNNNNNNNNNNNNNNNNNNNNNNNNNNNNNNNNNNNNNNNNNNNNNNNNNNNNNNNNNNNNNNNNNNNNNNNNNNN
NNNNNNNNNNNNNNNNNNNNNNNNNNNNNNNNNNNNNNNNNNNNNNNNNNNNNNNNNNNNNNNNNNNNNNNNNNNNNNNN
NNNNNNNNNNNNNNNNNNNNNNNNNNNNNNNNNNNNNNNNNNNNNNNNNNNNNNNNNNNNNNNNNNNNNNNNNNNNNNNN
NNNNNNNNNNNNNNNNNNNNNNNNNNNNNNNNNNNNNNNNNNNNNNNNNNNNNNNNNNNNNNNNNNNNNNNNNNNNNNNN
NNNNNNNNNNNNNNNNNNNNNNNNNNNNNNNNNNNNNNNNNNNNNNNNNNNNNNNNNNNNNNNNNNNNNNNNNNNNNNNN
NNNNNNNNNNNNNNNNNNNNNNNNNNNNNNNNNNNNNNNNNNNNNNNNNNNNNNNNNNNNNNNNNNNNNNNNNNNNNNNN
NNNNNNNNNNNNNNNNNNNNNNNNNNNNNNNNNNNNNNNNNNNNNNNNNNNNNNNNNNNNNNNNNNNNNNNNNNNNNNNN
NNNNNNNNNNNNNNNNNNNNNNNNNNNNNNNNNNNNNNNNNNNNNNNNNNNNNNNNNNNNNNNNNNNNNNNNNNNNNNNN
NNNNNNNNNNNNNNNNNNNNNNNNNNNNNNNNNNNNNNNNNNNNNNNNNNNNNNNNNNNNNNNNNNNNNNNNNNNNNNNN
NNNNNNNNNNNNNNNNNNNNNNNNNNNNNNNNNNNNNNNNNNNNNNNNNNNNNNNNNNNNNNNNNNNNNNNNNNNNNNNN
NNNNNNNNNNNNNNNNNNNNNNNNNNNNNNNNNNNNNNNNNNNNNNNNNNNNNNNNNNNNNNNNNNNNNNNNNNNNNNNN
NNNNNNNNNNNNNNNNNNNNNNNNNNNNNNNNNNNNNNNNNNNNNNNNNNNNNNNNNNNNNNNNNNNNNNNNNNNNNNNN
NNNNNNNNNNNNNNNNNNNNNNNNNNNNNNNNNNNNNNNNNNNNNNNNNNNNNNNNNNNNNNNNNNNNNNNNNNNNNNNN
NNNNNNNNNNNNNNNNNNNNNNNNNNNNNNNNNNNNNNNNNNNNNNNNNNNNNNNNNNNNNNNNNNNNNNNNNNNNNNNN
NNNNNNNNNNNNNNNNNNNNNNNNNNNNNNNNNNNNNNNNNNNNNNNNNNNNNNNNNNNNNNNNNNNNNNNNNNNNNNNN
NNNNNNNNNNNNNNNNNNNNNNNNNNNNNNNNNNNNNNNNNNNNNNNNNNNNNNNNNNNNNNNNNNNNNNNNNNNNNNNN
NNNNNNNNNNNNNNNNNNNNNNNNNNNNNNNNNNNNNNNNNNNNNNNNNNNNNNNNNNNNNNNNNNNNNNNNNNNNNNNN
NNNNNNNNNNNNNNNNNNNNNNNNNNNNNNNNNNNNNNNNNNNNNNNNNNNNNNNNNNNNNNNNNNNNNNNNNNNNNNNN
NNNNNNNNNNNNNNNNNNNNNNNNNTAGACACTCCTCTTTGGGTAGCCGTATCTTTTTGTGTCTCCCTTTTTTCTGGAGC
CAGTTGGTGGTCGCTGAGCCTGTATTTGGTCAGGATCTGTCTCTGTTTTGTATCTCTGACAGAGTAGAGATTATCCCTCC
CTCCCTCCCTCCCTTTTTCTAGGGGCATTTGGTACTGACACACCTGTCATTAGGAAGACTGGCTGGGCCTGGATGGAGAG
AGTCAGTGGAGGAGAGAAAGGGCTGACCCCACAGGAAGGAGGGCAGATGGAGATTGATGGAGGGAGAGCGAGAGGGAGAG
GGAGAAAGAGGAAAGAGGGATGGGGGGCCGCAGGCATATGCATCACACATGTGCACAAACGATTCACTTCTCTCCTCGCA
TGCAAGCCTTTCCTTTTGGGAGAGCAGATCAATTCTTGTCTAGTCTGTCTCTGCCTCTAGGAGTTTGTGTGCCTGTGTGT
GTCTCTGCGTGTATGTGTATGTGCATAGTGATCTACCCCACTATGGAAAAAGCTAAAAATCCCCCTCTGCCCCACATCAT
CCCTCACTCCGTCTCTCCTTCCTTCCCTCTCCTCCCTCCTTCTGTTTCAGAAGGACCTGTAAGTAATGGAGTGTGTTTGG
TTGCTCAGCCTGGGGCAGGATGTTAAGTCCAGAGCAGAATAAAGCTGCTGTTGAATTTGTGTGTGTGTGTGTGTGTGTGT
GTGTGTGTGTGTGTGTGTGTGTGTGTGTGTGTGTGTGTGTGTNNNNNNNNNNNNNNNNNNNNNNNNNNNNNNNNNNNNNN
NNNNNNNNNNNNNNNNNNNNNNNNNNNNNNNNNNNNNNNNNNNNNNNNNNNNNNNNNNNNNNNNNNNNNNNNNNNNNNNN
NNNNNNNNNNNNNNNNNNNNNNNNNNNNNNNNNNNNNNNNNNNNNNNNNNNNNNNNNNNNNNNNNNNNNNNNNNNNNNNN
NNNNNNNNNNNNNNNNNNNNNNNNNNNNNNNNNNNNNNNNNNNNNNNNNNNNNNNNNNNNNNNNNNNNNNNNNNNNNNNN
NNNNNNNNNNNNNNNNNNNNNNNNNNNNNNNNNNNNNNNNNNNNNNNNNNNNNNNNNNNNNNNNNNNNNNNNNNNNNNNN
NNNNNNNNNNNNNNNNNNNNNNNNNNNNNNNNNNNNNNNNNNNNNNNNNNNNNNNNNNNNNNNNNNNNNNNNNNNNNNNN
NNNNNNNNNNNNNNNNNNNNNNNNNNNNNNNNNNNNNNNNNNNNNNNNNNNNNNNNNNNNNNNNNNNNNNNNNNNNNNNN
NNNNNNNNNNNNNNNNNNNNNNNNNNNNNNNNNNNNNNNNNNNNNNNNNNNNNNNNNNNNNNNNNNNNNNNNNNNNNNNN
NNNNNNNNNNNNNNNNNNNNNNNNNNNNNNNNNNNNNNNNNNNNNNNNNNNNNNNNNNNNNNNNNNNNNNNNNNNNNNNN
NNNNNNNNNNNNNNNNNNNNNNNNNNNNNNNNNNNNNNNNNNNNNNNNNNNNNNNNNNNNNNNNNNNNNNNNNNNNNNNN
NNNNNNNNNNNNNNNNNNNNNNNNNNNNNNNNNNNNNNNNNNNNNNNNNNNNNNNNNNNNNNNNNNNNNNNNNNNNNNNN
NNNNNNNNNNNNNNNNNNNNNNNNNNNNNNNNNNNNNNNNNNNNNNNNNNNNNNNNNNNNNNNNNNNNNNNNNNNNNNNN
NNNNNNNNNNNNNNNNNNNNNNNNNNNNNNNNNNNNNNNNNNNNNNNNNNNNNNNNNNNNNNNNNNNNNNNNNNNNNNNN
NNNNNNNNNNNNNNNNNNNNNNNNNNNNNNNNNNNNNNNNNNNNNNNNNNNNNNNNNNNNNNNNNNNNNNNNNNNNNNNN
NNNNNNNNNNNNNNNNNNNNNNNNNNNNNNNNNNNNNNNNNNNNNNNNNNNNNNNNNNNNNNNNNNNNNNNNNNNNNNNN
NNNNNNNNNNNNNNNNNNNNNNNNNNNNNNNNNNNNNNNNNNNNNNNNNNNNNNNNNNNNNNNNNNNNNNNNNNNNNNNN
NNNNNNNNNNNNNNNNNNNNNNNNNNNNNNNNNNNNNNNNNNNNNNNNNNNNNNNNNNNNNNNNNNNNNNNNNNNNNNNN
NNNNNNNNNNNNNNNNNNNNNNNNNNNNNNNNNTTTGTTATGAGGTTTGGGAATCGCTTACCTTTCAGAGGTTGTAGAGT
ATCACTGCTATTTTCTGGATTCTGATAATTAGCGGGTATTGGACTAATTCTGCTCTGCAAGCATTATTTGGTGTGTTACG
TTGAACTCAGCAGATATTTTTTCATAATTCTGGATGCAGAGCCTCAATTTGGTGTTTGTCCCATTTTGTGAATTCTTGGT
TGGTGAGCGAACCCCAGACCTCACAACCATAAAGGGAAATGGTTTCTATAATTGAATGAAGTATTTTTTGCCAGATCCTA
ATTGGTATGTTGAATTTTATGTTCCTTTTGATGGCATAGAAGGCCCTTCTTGCCTTGTCTCTCAGCTTGTTTACAGCTTT
GTAGAAGTTGCCTGTGGCGCTGATGTTTAGGCCGAGGTATTTATAGTTTTTGTATTTGTGGTCCTGACAACTGGACCTTT
TTTGGAACACCATTATTTTTGTCTTACCAAGATTTCATCTGTGCAGAAGATCCTTGTTTGGGGAGAGAAGCACCAGATAA
TCAGCAAACAGTAGACATTTGACTTCAGATTATGTTTTAATGCCCTCGCCAATTTGTTGATAGCTTAAGCTGCATCCCTG
TCTCACCCCACGGCCTTGTGGAAAGAAAGTTGTGCTTTTTGCCTATTTTAACCGCACACTTGTTTGTGTACATGGATTTT
CTCCCAACACCCCTTTCCATCAATTTGTATAGCAGAGCCTCATGCCAAATTGAATCAAAAGCTTTTTTGAAATCAACAAA
CCAAGAGAAGACTTTGCCTTTGTTTTGTTTGTTTGTCAATTAGAGTGTGCGGGGTGAATACGTGGTCTGTCGTATGGTAA
TTCCGTAAAAAGACAATTTAACATTTGCTCAGCATATTGTTTTCACTGAGCAAATGTACGAGTCTGCTGTTAATGATAAA
GCAGAGGATTTTCCCAAGGTTGCTGTTGATGCATATCCCACGGTAGTTATTGGTGTCAAATTTGTCTCCACTTTTGTGGA
TTGGGGCGATCTGTCCTATGTTTCAAATATTGGGGAAAATGACAGAGCCAAGGATGATGTTAAAGAGTTTAAGTATAGCC
AATTTTAATTTCTGGTCTGTATATTTTATAATTTCATTGAGGATACATCAACACCACAGGCCTTTTTGGTTTGGAGGGTT
TGTATTTTGTTCTGTAGTTCAGTGTCCAGTGGTTTCTGGTAGTCTTTAATAGTTGATTCTAAGATTTGTATTTGATCATG
TATATGTTTTAGCTGTTTGTTCTTAGTTATAGGGCCAAAAAGATTGGAAGGTTTATCCATACATGTCACGTTTTTGAAAA
TGAGCGGACCAAGGCGCAGCGTGAGTATAGTTCCACATATTTTTAATCTCCGTGAAACAAACAAAACAATAAAGAAACAA
TGAAACGTGAAGTCATGACGTGCACACAGGCAACCGAACACAAACAATATCCCACAAAACACAGGTGGGGAATTGTCTAC
CTAAATATGATCCCCAATCAGAGGCAACGATAAACAGCTGCTTCTAATTGGGAACCATATTAGCACCAACATAGAAATAG
ACATACTAGATCACCCCCTAGTCACGCCCTGACCTACTACACCATAGAGAACCAAGGGCTTTTCTATGGTCAGGGCGTGA
CACCTAACTCCTACCTGGTCAATATTTCTGAGGTAATGAAATTCTTCTGCTTTGCTCATTATAGATTTCATTGTATTCAA
TCTTATCACATCTACTTTCCACCTGAATGACATGGTTGTTTTCATTTAAAGTCATCCAGAAAAGCTGTGAAAAATTGCTG
ACAAAGTGGATGAACTTCAGAGTGTTAAACTCCTGAGATGAACAGAATGCTGAGAGAGGACTAATTACACAAAAGGAATG
ACACATTTATATACAGTGCCTTCAAAAAGTATTCACACCCCTTGACTTTTTCCACATTTAAAATGGATTACATTGAGATT

GTGTGTCACTGGCTGGGGTTACCCCATAATGCCCCATAATGTCAAAGTGGAATTATGTTTTTAGACATGTTTACAAATGA
ATTAAAAATGAAAAGCTGAAATGTCTTGAGTCAATAAGTATTCAACCCCTTTGTTATGGCAAGCCTAAATAAGTTCAGGA
GTAAAAATGTGCTTAAGAAGTCGCATAATAAGTTACATGGACTCACGGTGTGCAATAATAGTGCTTAACAAGATGTTTGA
ATGACTACCTCATTTCTGTACCCCACACATACAGATAAGTTGTGCAGTAAATATCAAACACAGATTCAACCACAAAGACC
AGGGAGGTTTTTCAATGCCTCACAAAGAGGGGCACCTATTGGTAGATGGTTTAAAAAAAAAAAAGCAGACATTGAATATCC
CTTTGAGTATGTTTAAGTTATTAATTACACTTTGGACGGTGTATCAATACACCCAGTCACTACAAAGATACAGGCATCCT
TCCTAACTCAGTTGCCGGAGAGGAAGGAAACCGCTCAGGGATTTCACGATGAGGCCAACAGTGACTTTAAAACAGTTATA
GTTTAAAGACTGTGATAAGCGAAAACAGACTGGATCAACAACATACAATAAAACTGGCCAAAAATGTGGCAAAGAAATTA
ACAAGGCAAATATACACGGGATTTGCTTACCAAGACAACATTGAATGTTCCTGAATGGCCAAGTTACATTTGACTTAAAT
CGGCTTGGAAATGTATGGCAAAACTTCAAAATGGCTGTCTAGCAATAATCAACAACCAGCTTGACAGAGCTTGAAACATT
GTTATAATAACATGCAAATCTTGTACAATCCAGGTGTGCAAAGCTCTTAGAGACTTACCCAGAAAGACTCACAGCTGTAA
TCGATGCCAAAGGTGACTCTAACATACAGTATTTTGACTCAGGGTTGTGAATACTTATGTAAATTACATATTTCTGTATT
TCAATAAAAATGCTAAAATAAAAAAAAACTGTTTTCACTTTGTCATTATTGTGTGTAGATGGGTGAGAATTTTTTTTAAA
ATCCATTTTTAATTCAGGATGTAACACAACAAAACGTGGAATAAGTCAAGGGGTATGAATACTTTCTGAAGGAACTGTAT
AGGACATTATGCAGTGGCAACAGAGAAAGTGAGGACCAGGCAGCCGATGTGATTGGTGCAAAAACTGAGCAAATAAGATG
AGCTAAAAACAGAAATAGCACAGATTTTAGTCAAACTATTCCAAAATTAGTGGTTGGTTTGGACAGAGGATAACATATCA
CCCATTGTCATTTGCCACCATATCAAGCTGTCGCAATTCCGCAGAGAAAGCATTCTCTGAATATCTTTATGCTTAACATA
CAACTAAATTGATGCTTAACATTAATAAATAATGAAAATCCACAGGTGAAGTTTCAAACATTGGTGTTAGATGTTTTATT
ACATAAATAATGTACAGGGATTTATTTGACAAAAATAAGAAAGGGAAAAGGAATCAAACGTATGCACTGACTGAAACATT
GGCACCGATCTAAGAAACAATGAAATGTAACATGAGTGTACAGTAGAATTCTAAAAAAGGCAGACAGTGATTCACATGGG
TGATAAGTATCAAAACTAAACCTGTATATCTATTTACTTCAAGTTCAAGTAGTGTGCTTGTCAGGTCAAGTCTTACATGC
AGTCAAAACAATGTGGCTATTTGTCAATATGAGGTCAGGGACAGGTCATGATGTCTGACTTGTCTGTTACATAGAAATAA
GGCAGGCGATTGGGTGAGTCAAAGGTCACATGATATGTAGTCAATTGTATGTACATCAACAGTAAACTCTCACTATGTGC
TGCTATTTGCCATATCAATCCAAATGACTTTATTGCACAGTGATAAGTTATATCTCTATGAAAACTTTCAAAGTAATACT
GATGTAATATTATACATCTATACAGACTACCAATATTATGCTTGCTACAATGCCAAAAGAGAGGCAATCTTATGATAGTG
AACAATTGTTGTTCCACATCGTATCTCAAAAAATGACTCTCTCAAAGCTCTAACTTTAGCAAACGACGACACCATTTACC
ATTCGGTTTCCTATGACTTACTGCAGATAAAAACTTAAACCAACGACATACAGCAAAATTATAACAGATATAAATCTTAA
CACTTTAGATAAATGGCAGCAATATCCATCTGGCTACACGATTAACATTTCTGTTCAAATACACTATACAGCCATGTGTA
AATCAAAGATATAAGTTATTAACTATTTTTACAGTACTGTATCTATGGTATTTAGGGATTTACAGGATCCATGGTCATAC
ATGCAGAAGTCTCAATATAATTTATGTTGATATGGGTTCATTTGATACTGAATGGGACCTGAAAAAACCTTTAAGTGTAT
GAGTTCTGTATGTATGGTCATATGATAGATGTATCTATGCATGTAAGGTATACATACTCAATATATTTGTGTGCCTGAAT
GTAGGCCTACATGCATATATGTAATGCTTCTGTTCATAAATGCTAAATGTAATCTATTATTGCACATCTCGTAATGCTAA
AATATTGCTATTTCAATAGATTAAAATAGTTAATTACATTGGTAATATCATAACAAAATGTACATATAGAATCTTAAAGT
ATCCTTATTGGTATGGCTGGTACAGTACTGGTACTTCAGTAGGGTATTGATAACATTTAGATAAAGTGTGTTACACTAGC
GTGTCTGATATTCTTTGATATAATATAATCTGTCATATTCCTCTTAAGAAACATAACTGATACTCTAGAGGCACCAAGAT
AAATCGCAAAAAAAAGGCTTTCAAAGAATATCTGAAGTCGTTTCTAAGAAGAATGATGAATGACATTTGAAAAGCAATGAT
AGAAGTTTGGGTTTTGTGAAATGTATGGTTAATTATCCTTTTGGAGGACAGTGGATATAACTACTTGAGCCACTGAAATT
GCTTGAAACATACTTTCTACCATCAATAATTAAAAAAAAACGCAAATAGTATCTCATTCATTGACAGCATATGAATCGGT
CTTAAAACCTTGCAGCCGTCCACTACTTGTAGAATCCTCTAGATTACCTTGTTAACTGTAACAATGCCATCACCCAAACT
AACTTCAAAGGGGAAGGAGAACTTGTCATTTAGATGCTAAATTAGGTTTACTCTGTGTTCTCTTTACACATCATAAGATA
GTATTTCCCTATCAAACTGTGGTCGCAGTTTGTAAAGATTGGCATTGTTCATCCTCATGTCAAGCTGTTATGGTGATGAC
AACAAGATGTCATGTACTACCTTAGTCTTTGTTTCATGCATTTTTATAAAAAACACCATTATGGCCTCATACTGTATAGG
GCAGATAGAAGTCATATGATGTCACCTTTCAGTCCATTATTGTGCTTTTCGATTCAATCTAGGTGAATCCCTCTCAGTTT
ACGATTATCGGAATAATCACCACAGATGACACAGGTAAACTACTGAAGGAAAAAAACTGTTTGATATACTGGCAATAAAA
TGGCTGCCAAATAGTGCAATAGCACAAAGGGCCTAAACATGTCTTTTGGGTTCTTTATGCACCAAGTTATGGGGTTATGG
ATGGGATTGGTTCCATTAAGTACAGCCAGGGACTGTCTCGGTCTTCATTCCATTTCTTCTTCCCTGGAATGGATTAAAAG
TAGCAAAGTTCCCTGTGACACATATCTAACTATCATTTGCAGATTATTAGAAGTGTTGGACAAAAGCAGAACATTTACAC
AAATAGTTTCCTCTCTCAAGTTCCTCATCTCAAGTCAATTTGTCAACATTAGTCAGAAGAAGAATGTTCAATGAACATTT
TCCTACTAACTGGGTGAAAACCAATTGCCTAAAGTATTTGCATACATACATGAATTATTCATATGAATGGATTTCACTGT
TGACCTCTCACCTTTCAAATGAATGTGCAGTTAGTTTGAAGCTGTCCCATATATATATATATATATATATATATATATAT
AGCTCTCGAGTGGCACAGTGGTCTAAGGCACTGCATCTCAGTGCAAGAGGCGTCACTACAGTCCCTGGTTCGAATCCAGG
CTGTATCACATCTGGCCGGGATTGGGGGTCCCATAGGGCAGCGCACAATTGTCCGAGTTTGACTGGGGTAGGCTATCATT
GCAAATAAGATTTTGTTCTTAACTGACTTGTCTAGTTAAATAAAAGTTACATTTGACTTTTTTTAAATTAAAATATCCCT
GCATACATTTGAGTGTGCAGGTTGAGGCCTCCCCATTATTTCTCCATACATGAATGTATGTATTTCATAACTATACATTA
TCTAGACTCTCATATACTGTATATTCGTCAATATTCCCCAGATTATTTCCAAAAATCAGCACCTGCCTAAAGAAGATTGT
TGGATATTTCTTGGATAAATGTAATCAGACAGTTTGTTCTCAACAATACATTAATTTATTTTCTTCACTCCATTTTAGCT
CTCTCCACACCCGTCTTCTCTTTCTCTCTACATCTCCATTCTCCTTCACCACCCCTCTTTTTACCTGTTGATTATTCCTT
TAATTTGGGAGTCTTTCTCCACTTGCTGTTGCCGTAGCCTCCTCTCGCTGTCCTCCAGGCGACTCTGGTATTGCGAGAGG
ATCTTATTGGTCTGCTGCTCCTGGGAAAGGAGCCTCTTCTCATACTCCTCCAGCTTCCTGTGGGACATGATCAGTCGCTC
CTTTAGGGAATGGATCTCCTCCTCGTATTCCTTTACCTGAGACAGACAGAGAAAAGAGAACGCATTGGAAATAGAAAATG
GTTGAGCAATTGTCATACACGTCGACAAGTCACCATGGAATATGGAGGCAACCACATTGTTGTCCAGTGTTCACTAAAGT
CTAAGGCACACCGTTTATCCCTATTCACTTGCTCACTATACACAAAGATCTAGCTATGTCTGTTATGAAAATAACTCCAG
AAGGGAGTATATTGAAAATATAAAAATAGTTAAGCAACTGTCATACTGTTTATTAGCAACACACGGCTAAGCAACCAACA
AATGTTCTTGGGGGGTTGACTGAACACACCTTCACTCGGACAAAGAAATGTGGTAATGAGGAGTCTGCGGTCAAATATGAA
TATGAGAGCTAAAAGTTTGTTTATTTTCAATGACTCACTGTTGTTCAAGTTCATCCTCAGCTGTTTCTAAATTATTCATA
AATAAGTGTCTCGGTACCATCTGCTCTCCACTGATAGACTTGACTTGCACTTCCCTGACATCTGCACTGTGCATTAAGAA
GGAGATATGACTCAGATCAGTGTTACGGTTCTCTAGTCACAGTTTGCTGTTGAATATGCTGCTAAGTCGAATTCTGCATT
CCTTTGAGGCATCGTTTTGTTACTGTCGATTTCCTAGAAGATTGGGATTTCAATGCCAAAGCTACAGTATATTGTTAAGA
CAATTATCTACTGTTTTTTAAATATACACATTGCAAATGTCAAATTTAGGCTACCATTCTTTGTATTGCAAAAGCTGTGG

TGGGAGAGATGCATCTACCCTGTCTAAGCGAGACTCATCCATGCTTTTGGAGTACTCCTTCAGCTTGAACTCCTCACGGT
CGATGCGGTTAGCCTCCAGAGTAATGTCAGCGGAGAGGTGAGGCATGTTGGAGACCCAGGCGACTGTTCTCTCATTGGCC
GGGGTCGGGGGGTTAAGGGTCGAGGGCGACTGGCCAGAACCCTGTGGAAAAAGACAAAAAGCAGATATGAAGAATATGTA
CCAGTACTAGCAGTGGCGTGTATTTATGGGTGCCAAGGGAAGCCAGACATTCACCAACAATTGAACAAGAAAAGATAAAA
TCATTTATCTTTCGTCTCTCTGTGTTTCATAATTATCCTTCAATTCGCAAGAGGCTGAATATATCTCACTGGAGAAAGCA
TCCGAGCGAGCAAAACACTGCCCCTCTGTCTCTGTATGTGTAGGCCATCTATCTGATGCTGTCTGGGCCAAAAGAGTATG
ACATTGTTGCCGGACGTAGCATTGAATGCAAGGGAAGCCAACGAGCATTTGGCTTTCCTTGATATTATTATTATTTTTAA
ATAATAGCCAATCAGCGTTGCGCTAAACTGAGTGAGCTCAACTGTGAATGGTCCTGGCGCATAAAATATACATGTCAAGG
GAAGCCAGTTTGGATTTGGCATCACTCCTATCAAATCGTATCGAGAAACAACTTGAATTGTTGAATCTCGTTGTGTAGTT
GTCCTCCGGTGGCGAGCTATCTAGCTAAAGTAGGCCCTTTCCTAAATTGGCCATGGATGGAGATAGGGATTTGGACTTGT
GGTTTTACTTAATTCTCCGAACTGGCCAATGCTTATGACGGCAATTCTGAACCAACTATTAATTCATACATTGTGCCCCT
GGCCTGAGAGGATGGAAGTTCAATATGTAGCTAGATGTAGAAGGCTAATGTTGATCATGAAAGAAAGTTAGACTAGCGAG
CAAGCATTTTAGCCAGGTAGCCTAGGACAACAAAAAATAAAAGCATGTACTGTATGACAGAGTGAGAGACTATTTTATCA
ACATGAAAGAGAGGAGGATGGCATTGGCGTTTCTCTACAAGTAGGGTGAGTCAACATGTGTTTTAGACTTGCACAAACTC
GCATGCACGCACACATAGGAATCAGAACCATGGACAGCCACATTATATTTAGCCTACGTTGATTGGACTAAATTGTTTTT
GGTATCTTTTAGTTGTCACTGTAGTAGACTAAGCATAGGTGATATGATGATATTGAAATGTTTTCTCTTCATTCAAAGAC
TCAATCAGGGACACTCTTACTGACAGTTGTGCTTGCTTGATGTATAGTCTCTACCTTCTTTCCCTTTGTGCTGTTGTCTG
TGCCCAATAATGTTTGTACCATGTTTTGTGCTGCTACCATGTTGTTATTATGTTGTGTTGCTACCATGCTGTGTTGCTGC
CTTGCTATGTTGTTGTCTTAGGGCTATCTTTATGTAGTGTTGTGTTGTCTCGTCGTAATGTGTGTTTTGTCATATATATA
TATATATATATATATATATATTTATTTATATATATATACTGCAAAAAAAAATAAAGGGAACACTTAAACAACACAATGTAAC
TCCAAGTCAATCACACTTCTGTGAAATCAAACTGTCCACTTAGGAAGCAACACTGATTGACAATAAATTTCACATGCTGT
TGTGCAAATAGAATAGACAACAGGTGGAAATTATAGGCAACAAGCAAGACACCCCCAATAAGGACTGGTTCTGCAGGTGG
GGACCACAGACCACTTCTCAGTTCCTGTGTTTCCTGGCTGATGTTTTGGTCACTTTTGAATGCTGGTGGTGCTTTCACTC
TAGTAGTAGCTTGAGACGGAGTCTACAACCCACACAAGTGGCTCAGGTAGTGCAGCTCATCCAGGATGGCACATCAATGC
GAGCTGTGGCAAGAAGGTTTGCTGTGTCTGTCAGCGTAGTGTCCAGAGCATGGAGGCGCTACCAGGAGACAGGCCAGTAC
ATCAGGAGACGTGGAGGAGGCCGTAGGAGGGCAACAACCCAGTAGCAGGACCGCTACCTCCGCCTTTGTGCAAGGAGGAG
CAGGAGGAGCACTACACGAGCCCTGCAAAATGACCTCCAGCAGGCCACAAATGTGCATGTGTCTGCTCAAACGGTCAGAA
ACAGACTCCATGAGGGTGGTATGAGGGCCCGACGTCCACAGGTGGGGGTTGTGCTTACAGCCCAACACCGTGCAGGACGT
TTGGCATTTGCCAGAGAACACCAAGATTGTCAAATTCGCCACTGGCGCCCTGTGCTCTTCACAGATGAAAGCAGGTTCAC
ACTGAGCACATGTGACAGACGTGACAGTCTGGAGACGCCGTGGAGAACGTTCTGCTGCCTGCAACATCCTCCAGCATGAC
CGGTTTGGCGGTGGGTCAGTCATGGTGTTGGGTGGCATTTCTTTGGGGGGCCGCACAGCCCTCCATGTGCTCGCCAGAGG
TAGCCTGACTGCCATTAGGTACCGAGAGGAGATCCTCAGACCCCTTGTGAGACCATATGCTGGTGCGGTTGGCCCTGGGT
TCCTCCTAATGCAAGACAATGCTAGACCTCATGTGGCTGGAGTGTGTCAGCAGTTCCTGCAAGAGGAAGGCATTGATGCT
ATGGACTGGCCCGCCCGTTCCCCAGACCTGAATCCAATTGAGCACATCTGGGTCATCATGTCTCGCTCCATCCACCAACG
CCACGTTTCACCACAGACTGTCCAGGAGTTGGCGGATGCTTTAGTCCAGGTCTGGGAGGAGATCCCTCAGGAGACCATCC
GCCACCTCATCAGGAGCATGCCCAGGCGTTGTAGGGAGGTCATACAGGCACGTGGAGGCCACACACACTACTGAGCCTCA
TTTTGACTTGTTTTAAGGACATTACATCAAAGTTGGATCAGCCTGTAGTGTGGTTTTCCACTTTAATTTTGAGTGTGACT
CCAAATCCAGACCTCCATGGGTTGATAAATTGGATTTCCATTGATTATTTTTGTGTGATTTTGTTGTCAGCACATTCAAC
TATGTAAAGAAAAAAGTCTTTAATAAGATTATTTCTTTCATTCAGATCTAGGATGTGTTGTTTAAGTGTTCCCTTTCTTT
TTTTGAGCAGTATATATATATATATATATATATATATATATATATATATATATATATATATATATAGTTGAAGTCGGAAGTTTAGA
TATACCTTAGCCAAATACATTTGAAATCAGTTTCACAATTCCTGACATTTAATCCTAGTAAAAATTCCCTGTCTTAGGTC
AGTCAGGATCACCATTTCATTTTAAGAATGTGAAATGTCAGAATAATAGTAAAGAGAATTATTTATTTCAGCTTTTATTT
CTTTCAAATTCCTTTGGGTCAGATGTTTACATGCACTCAAAAAGCTGGTGTAACTGAGTCAGGTTTGTAGGCCTCCTTGC
TTGCCTACGCTTTTTCAATTCTGCCCACAAATTTTCTATAGGATTGAGGTCAGGACTTTTTGTTGGCCACTCCAATACCT
TGACTTTGTTGTCCTTAAGCCATTTTGCCACAACTTTGGAAGTATGCTTGGGGTCATTGTTCATTTGGAAGACCTATTTG
CGACCAAGCTTTAACTTCCTGACTGATGTCTTGAGATGTTGCTTTAATATATCCACATAATCTTCCTCCCTCATGATGCC
ATCTATTTTGTGAAGTGCACCAGTCCCTCCTGCAGCAAAGCACCCCCACAACATGATGCTGCCACCCCCGTGCTTCACGG
TTGGGATGGTGTTCTTCGGCTTGCAAGCTTCCCCCTTTTTCCTCCAAACATAATGATGGTCATTATGGCCAAACAATTCT
ATTTTTGTTTCATTTCTCCAAAAAGTATGATCTTTGTCCCCATGTGCAGTTGCAAACCTTATTCTGTTTTTTTTTATGGC
GGTTTTGGAGCAGTGGCTTCTTCCTTGCTGAGCGGCCTTTCAGGTTATGCGATATAGGACTCGTTTTACTGTGGATATAG
ATACTTTTGTATCTGTTTCCTCCAGCATCTTCACAAGGTCCTTTGCTGTTGTTCTGGGGTTGATTTACACTTTTTGCACC
AAAGTACGTTCATCTCTATGAGACAGAACGCATCTCCTTCCTGAGCGGTATGACAGCTGCGTGGTCCCATGGTGTTTATA
CTTGCGTACTATTGTTTGTACAGATGATAGGGGTACCTTCAGGCATTTGTAAATTGCTCCCAAGGATGAACCGGACTTGT
GGAGGTCTACAATTTTTTTTCTGAGGTCTTGGCTGATTTCTTTTGATTTTCCCATGATGTCAAGCAAAGAGGCACTGAGT
TTGAAGGTAGGCCTTGAAAATACATCCACAGGTTACACCTCCAATTGACTCAATGATGTCAATTAACCCTATCAGAAGCT
TTCTAAAGCCATGACATCATTTTCTGGAATTGTCCAAGCTGTTTATAGGGCACATTCCACCTAATTTTATGTAAACCTGN
NNNNNNNNNNNNNNNNNNNNNNNNNNNNNNNNNNNNNNNNNNNNNNNNNNNNNNNNNNNNNNNNNNNNNNNNNNNNNNNN
NNNNNNNNNNNNNNNNNNNNNNNNNNNNNNNNNNNNNNNNNNNNNNNNNNNNNNNNNNNNNNNNNNNNNNNNNNNNNNNN
NNNNNNNNNNNNNNNNNNNNNNNNNNNNNNNNNNNNNNNNNNNNNNNNNNNNNNNNNNNNNNNNNNNNNNNNNNNNNNNN
NNNNNNNNNNNNNNNNNNNNNNNNNNNNNNNNNNNNNNNNNNNNNNNNNNNNNNNNNNNNNNNNNNNNNNNNNNNNNNNN
NNNNNNNNNNNNNNNNNNNNNNNNNNNNNNNNNNNNNNNNNNNNNNNNNNNNNNNNNNNNNNNNNNNNNNNNNNNNNNNN
NNNNNNNNNNNNNNNNNNNNNNNNNNNNNNNNNNNNNNNAAAAAAAATGGTGCTGGAATAGTTGAGGCAGCTCCTGTTTTCTTTG
CGTCTTGTGGTAACTTTCCGTGGTTCTAAATCAATAGTTGTTTAGTGGTCCGAAAATGTCGGAAACATTAACTTGATCAT
GCAACTGTTTGTTACATGCAATACGCTTTGTGGACTTCACCAGGTGTGGTTGAATTTATTCTACCACTGTGTCTTCTTAC
TATCTCGGCCTTAGGCCTTTATATCACTGTCGCAAGGCATATGAACTAACAGGTTATAGAGCAAACAAAGCAATTATCAC
AACACATAAGTTGTAATATGGCTTTTTTTTCCTGACTTCCCCAGTGATTTACCCATGCACCACTACTGTGTACTACACA
AATGAGGGGGTGACCTTTGGCCCTATTTAACCATACAGGTCACTGGGAGTCTGGGGTTGGTCAGAAACAACTGTGCAAGA
GATAGCATGGTTGAACTGTAATTGAGAGGTGTGTTGGAATTGTTATATAATGCTCTTTTCAGAGTGAGAGAAAATAACAG
TGACAGCTATGAGCTTGTTGTCAGATTTCTAGGGTAGATTGAATTACACAATAGAGTACAATAGCCTGCATACCTGTTTG

CTGATGGAAGGTTTAAGCAGGTGCTGTTGCTGTGGTTGTTGCTGTTGCTGTTGCTGCTGCTGCGATTGCTGTATGGTCGT
TGTAGTGCCTCCCTGTGGCCCCTGGGGGCCCTGGCTCTCCCTGACAGAGCTCTGCTGGTGTGGCAGTCCAGGGGCTGTGT
TGTCTTTGACCGACAGCTGTTGCCGCGGTGCCTGTTGCCGACCTCCGTAGCCCGACTCTGGCGATAGGTTGGCACTGGGC
GGTCGTGTCTTAGCGGATGGTGTGGGTGCAGCAGCCGCACTGACAGACAGCTGATGGGAGGTCTGGGATTTGACACGTTG
AGCGGAAGGCGGGGCGATGGAGCTCTGGCGCACGGGCTGGACTGTGGAGGGGGGTGTGGCCAGGGAGGAATGGGAGGTAC
CAGTCTGAGAGGCCATGCCCATCTGCTGTTGAAGATTATCCTAACAAAGACAATTAGAGAACACCAAGTTATCCTGGCCT
TCCAACTGTTTCAGATATTTACATTATGCTAATCCTATTAATGTAGGCTACAAACAAATGTTGACTTAAATTATTATCAT
GTAGGAGAGACAAAAAGGCTAATTTGCACAAAAGTAGAACATGATTGTAAACACATTCTTGACCTTAAGTTAATCCATAC
CTGCCCGTGCATGGACAGTTGCCGCCGTGCGTAATCAGCCCTGCTGTAGTCGTCGCTATAGCTGTGTGAGTGGATAATGG
CTGGCTGGCCCAGGTGCCCTGCCCCGGTCCTTAGGGTGGACAGGTCCTCGCTGCGGGAGAACCCCCCGTGGGCGAACTGG
GGGATGTAGGCCTGGTGCGAGCCACCGTGGTCAGGCTCCGGGGCCAGAAGCAGGGGCGGCGGGAGATGGGCCCTGCTGTG
CTGGTGGTGAAGGTGGGGTCCATCTGCAGTGCCGGTGGCCAGGTGAAACAGGGGGTTCTGGAAGGAGAGCGGGTATCTCA
GGGCGGCGGCCTGCTGCTGCTGCTGCTGGGACAGCGAGTCGGTGGTGGTGCCCATCTGGCTCAACTGGCTCAGCCGTAGG
CCCCCTGACATGCTGGATCCCCCCGAGCCGGCCGAGTGGCGGCCCCCGGCCCGGAGCTGGCCGCTCAGGCCCGAGCCCAG
GCCTCCTCCACCCTGGCTGCTGAGCCCTCCGAAGCTGCCCATGCCGGTGATGGAGGCCTGGGAGGAGTTGAGCATGGCCA
CCGACTGCAGGTTAGACACGCTGTTCATTGTGGAGTCCTGGAGGTCCATCATAGATATACACTTTATTGACACTGAGCAC
CTAGACTAGATTGATATAGCTAACACCCTTGTGTGCCCAATTCTAAAGTGCCAATACGTTGATTAAGTGTTTTATCTATT
GAGAGGGGTGGTTGTATGCCTGAATTGCATGTCCCATGACTTACCACTAAGGTACATGCATCATTTTCCTATGAACGCAC
AGCTGCCATACTATCCTGCAGTCGTCTGCCAGACTTGGCTACCACATGGAAAGGAGATGTGCAGTTCACATAGTTTCCTG
TAGAACATGGCTCCTAACAGATCTTAATTACATTTAATGCTATCAAAACTCTAGAATTCAACTAACCTTGTGGTCTGGCT
CAGTGATATCGGAGCTACTGGTGCAGTAGGCAGGGCTGGATCGGGCCAAGGGAGGGCGGGTTACATAGAAAACGTCTTTG
GAGGCTCGATGTGGGTGATCGCGCTCTTGGAAACTCAGCTGAGCATGAGCACGAGACGGCATGACCCCCCCACTGGACAC
ACCAGATGTAGGGGAAGGCAAGCGAGTGATATCTATGGAGCTTAAGAAAGGCAACAACACATGGCAACAGATTGGAGAAG
ACGATGACAAGGTTATAGGTGTATTACAAGTTTGGCACAACCCAACTACAGCCTTATGGCGGGAAAGGCAAAACTTTAAC
AAACGCACCAACCAACCAACCATCCTCCTAGGGCTGGTTTCCCCAACACAGATTAAACCTAATCCTAGACAAAAAAGTAT
GTTCAACGGAAATTCTCCATTCAACGTGTTTTTTTGTCCAGGACTATACTTAATCTGTTTCCAGGAAATAGGCCCATAGG
ATTAAATAAAGCCAGGGCACTACCTGTTGAGATCCTTCATCATGAGATTCTGGAACTCCGAGGAGACTCCCCTGTGGAAG
CTGGGTCGTGAGAGCAGCCTCTCCGTCTGTCTCTCATGGACTCTATCTGTCTGGTTCTGGTGGCTGGGCTGCCTCTGCAG
GTGGGGGTTACGCAGGGCCATGCTGATGTCATTCAAGAGGCGGGGCAAGGGACCCAGTTTGATAATGGCATCCTGTGGGT
TAGGGTTAGAAGACTGACATGAATACAAGTTAGCAATGACAATAGGAAAAGCATTCAAATGGTCAATTTTTTAAAAGTAT
CTTATCCTTGAAAATTGCAAGTATACTTAATCATTCCATTCATTCTACTGACTCATACTTTCAGATTACTGGTGTGTCAA
TACCACACATAGTTATTTTATTGTACTGATTCCTAATTGGACTCAATCATTATTGAATAACACCACATTAGTTATCTATA
ACTTGTATAACTTTTTTTATTGGTCATATATTGTAGAGGTTATATTGTTAATATTTACCCTGATCCTCCCTACCTTGCTG
AGCTGGGCCATGACCTCCCAGAGGAGGCTGTGCAGTATGGACAGCTCCCTGCCCAGGTCGATGTAGCCCTCGAAGCCCCC
CGCGTTGCTCACACTGTCCAGGTTGGAGATCTCATAAAGGAACTGTTGCATGGAGCCCCACTCCATCTCTAAGAACTCAT
TCATGAAGCACATGTACTCCTCTTTCATACCAAACCTGAGGGAGTGAGAGAGAGAGAGAGAAGAAGAGTCAAAATGCTGT
ATACTCACAGAGATTACATCATTGGCTGGTTTCCCGGACACAGAATTAGCCCACCCTGGATTAATAACTAATTAGCACTT
TCAATGGAGAATCTACATTGAGCAAGCTTTTTAGTCCAGGATTAGGCTTAATCTGTGTCCCGGAAAAATACTCAAAATAT
TACAAAAGTTTTAGCATAAGCATAACATTATAAGTTGTAGTGAATGAATGAACTGACTTGGAGAAGTTGGCCAGGTTCTG
CACCACTTTGGAGATGAGGGTGAGCGTGCGTGACGTCTGCTCGTCTGGGTACTCCTGGGTCAGGTTGAACAGGGAAGGGG
ACATGACGGCCGGACACAGGAAGCGCAGGAAAAGGGAGCCACTGATTAGGCGGTCTGCGATGTCCTCCCTCCCCCTCTCC
GCACAGCGCACCCTCCACGAGGCAAACACCTCCTTCAGCTCTCGAGGAAACACACTGCAGAGAGATACAAATATTCAGTG
TGTTATTATCATTTGATAGGATATTCTGTTTTAGTCAGGCAGTGTATCCCAACTACTGAATGCAAATCTGGTGTTCAACA
ATCAAAATTCTGTAAATAAATCTTTGGGTGCTGTGGCTCAGGAGTAATCCTACTTAAATGAAAAAAAGAGTACGCACTCA
TATGTCATCTACTGACCAATGGGAGTTGACGATTTTGCAGAGTGCTAGTTCGCAGCACATGCGGAGATTGGCTTGGTGAT
CTGGAAGGACAGAGGGTGGTGTCCTCATGGGGTCCACCTCGCAGTTCTCTTCTGATTCGTACAGTGCCCTTATGAACTCC
CCTGAAACAAGCACAATATCACTGACCATTAGGCAAACACAGAAAGATAATGAATATATCACAACAAGAACAGTTCAACA
ACATATATTGAGTGATTAATGTGAAGCATTCGAAGGCCACACCTATTGCATCCTTGAGGTACTTATGTCCAATCAGCTTG
AGGTACTCTTCTATGGCTTTGGTGGCCAGAGTGTTCTCTCTGAAGATAAGGTGTTCTCGGTCAATGAATCTATCTACCTC
ACACATCGCCATGTCCGAAAGAAAATCCTGGGAAAAAACAAAACGTCAACATAGCATAGACTTTCTTTTTAAAACATTCA
ATTATAATCTATTGGGTACTCTCAAATTCAATTTTGAGGATATAACGCCTCTTCTCAGGTCAGAATGTAATTTGTTATCT
CTTTTGGCTTATGGTTTGGTAAGAAAACCAAGAAGTAGTCAAACTAATGTTCATTTTGTAGTTCATAGTGTCTTAAAGAT
AGAATCCGCAGGTTGTGGGCGCCCCCATGCCGCCCTACTTCTTCTGTTGTGTTTTCACTTTGAACGAAAACAAGGGGATT
GAGCCCTGAACCGCCTTGGGCTGGCCTTCTGGGCAAGGTGTGTCTTTGCACTAAATCCTGTCTACTCGAACGACCCTGCT
GCCATGCTCCGGAGCTGGAGAGGAAGTGAAAGTCACTTGCAAGCTACAGCCGCCAGAAACAAATACAGAGCAGAGGAAGC
CAACATGCTAGCTAACTTGTTTGCAGATAAACTGTCCTGGTAGCAGGGTCAGCATTTTCAGAAAATGTAGACAGAAACAA
GCATTATTCTCTTCTTGGATCTGAATCAACTCGACCCCAAAAGAGAAAAAGAAGCGAGAAAAAGACTGCTCATGACCAAT
CCAGGAACCGGATAAATATAGGAATTAATTTTGAAAGGTGGAAGAGTGATGCCGAAATGGCCACCATTCTCATGAACAGG
TAGGTAAATGTTGGGTTGCGTTTTTAGCCTGTGTTTTTTAGCTACATTAGCTGCTAACCTTAGTGTGTTCAATGTTCAAG
CTTTCTAACATGGCTACTTAGCTAGCTAGCCGACATCTTTTGTATTTGGACTGTTTTGTACTCAAAGAATCTAATGATAA
CTAACTACATCATGTATACACTGCATAAACCACACAAGATGCCCAATGCCTTTATTTCAACTAGCTAACGTTAGCTAAGT
TACATAACGCTGGATATGCAGATCGTTGACATGATAGTGTGTGTGGTTTGTAGATGATAATGCTAGCTAGCTAGATGGTT
GACAGCATTTTCAACCTAGGCACACCCAAACAATGCATCATCTAGCTATACATCCGGTACAGTGACATCACACAAACATT
TATTTCTCAGGGAGATACTGGTGGAGATTTCGAAAGGAACCTGAGGTTGTAGAAGTTGTGGTTGTGGAAAAATCCAAAAG
GGAGGTAAAAATATGCTATGGAATGTTTTGTTATGTTTTGTGATTCAATAATAATTTGATTGGTAATCGACCAATGGAAG
CAATGTTTGCCTGTTCAGCACATTACCTACATAGCATGGCATTGAGTCCTACCATGAACTGCCCAACTTCAAGGACTGAA
TGAATATCCAGTGTGGTCAGTAAAGTAAACAATGGCTCGTCTGCATGACATATTGGTAGAACATGGGCCATCAAGTAATA
GAATGGTTACATTAGAATGACACATGTTACACTGGTTACATCGCAACACCACATCCTATGTATTTAAAGAATACGGTTAT
GGCACTGATCTATTTGGCAGTAATGCCCCCCCCCCCCCCCCCCATTTAGCAGCCGAAGCCAACAAATCCTGGTGATTTGG

GGGGGTTGAATTAATATAAGGGGGAATNNNNNNNNNNNNNNNNNNNNNNNNNNNNNNNNNNNNNNNNNNNNNNNNNNNN
NNNNNNNNNNNNNNNNNNNNNNNNNNNNNNNNNNNNNNNNNNNNNNNNNNAATCAAAAACAGCATTTATCTGGCATATACACA
GGATACAGTGAGGTGTATACAGGAATATGACCTTGCATTGTACCGTGTACTACTGAAGTAACAACTTGTTTAATGTTTCA
TGCCAATACTTACTTTTGCTTTCCCCGTGCTCTGTAGTATGTGGACCAGCGCACAGGCCACCTCTTCTTTACTCTTCACG
CTCAGTAGAGGCTCCAGCACCGCACACAAAGTCCGGTAGTTATTGGTAACATACTCGGCAAACTCCTTGTACAGCTCCAT
GGGTAGGATACTCATGGTCTGGTAACGGGACTTGAGGCGTAGGGAGGCATTGATGATCTTGCCCCCTGCTTGACCCTTGG
CCAGGACGCTAGGCTGGATGACAGGGTACCACTGCTCCACAAACTGTCGGCCCGTGATGCTGGAGATAGGGATACTGACC
AGTCCCAGGTATGTGCTCTTTTCCTAATAGAAGAGGGGAAATAAGGCATAAATCAGAGAAACTTAATGTGGGATTTTGTG
GTCAAGAGTTATGGGGCCTGGGCCATTAAGAAAGAGGAGAAGAAGACAATAGTAAATTAAACTAAAGTATATTTATTCAT
CTTAATCATTGGGCAAGTTGCTGTCATTAAACCCCAGCAGTGCTTTAACCAGTCTGCAAAAATGTAATTTTAACCAGTTT
TGTACACTGGTGAGAGAGTTGTCCCTTCACCTTGCGTCTCTTCTTGTCAGTCTCCTTGTAGAGATGAAGGCGTAGGTTGC
GGACGGCTGGCAAGTTGTTGAACTCAAAGTGCTCACCCCAGAACACGGTGTCAGTCCGGGGCTTGCTGGTGGTGCGTGCG
TAAAGCATGTCGTCCAGACAAAGTTCGCAGTAGTAGCGCTTCTTGGCGGGAAGCTCCCGCGCCTCAATGATCCAGAGTTT
CAGCACGTTGTCCACCCTCCGACTGTTGTCCTGAAGAGGGGCAAAGAGAGGGTGGCAAAAGGACAAGGCAGAACAGGGAG
GAAAGAAAACCAAAGGGAAAAAGACCAAAGAGAAAAAGAAAGCCAAAGGGAGAGGGAGGGTTGAGTGGGTTAAGAACAGT
GGTGTAAAGTACTTAAGTAAAAATACTTTAAAGTACTACTTAAGTAGTTTTTTGGGGTATCTGTATTTCACTATTTATAT
TTAACTTCACTACAGTACATTCCAAAATAAAATAATGTACTTTTTACTCCATACATGTACCCTGACAACCAAAACTACTC
GTGACATTTTGACATGAAAATGGTCCAATTCACACACTTATCAAGAGAACATCCCTGGTCCTCCCTACTACCTATTTTCT
GGCGGACTCACTAAACACAAATGCTTAGTTTGTAAATTATGTCTGAGTGTTGGAGCGTGCCCCTGGCTATTCGTCAATAA
AAAAAATACAATTGTGCCGTCATGATTGCTTAATATAAGGAATTTGAAATGGTTTATACTTTTACTTTTGATACTGAAGT
ATATTTGAGCAATTTCATTTACTTTTGATACTTAAGTATATTTAAAACGAAATACTTTTACTCAAGTCGTATTTTACTGG
GTGACTTTCACTTTTACTTGAGTCATTTTCTATTAAGGTATTTTTACTTTTACTCCAGTATGACAATTCAGTACCTTTTC
CACCACTGATTAAGAACAGTCTAAGAACAAAATGCACACATACGGATGGAGATAAATTAGATAACACATATGTACTGGAG
GCATGAGAGACAGAAAGTGGATGTGTGATTGGTAATGTTGCAGCAGAGAGCTGCATTTTCTGAACATGTTTACAAAGAGC
CAACTATGTCTGAAATCCCCCCATGGGAGGAGAGCGTGCTGTGCAGATTGCACAGAGTGATGTTTTAGTCAGGGGTAAGT
CATTTTAACTAGTCTGAAATGCCACTGGATCGCTCAGCCCTGGCTTAGCTCAGCTCTTAATACCGAATTTAGATTGCTAA
GTATATCACCAATTTTCCAAAGATGTTATATCTTAAGTACTCCATTGGTATGCCGCCTGACAATAAATGTGGACGTCCGC
CGATACAACCCTTTCAGCCGTTCATCATGAATTTCAATAGGAAGAAGAATCGAGAGCGGCTGTCTGCCCAGCGACGGTTT
GCGAAACTTAATGAACATCCTGGTCTCTATCGACATCGCACCTCGTCTTATCTCTCCCATCATTTATCTGGTTGGAGATT
CATGAGGGTGCAGAGGATATTTTGGAGCCCCGTGTCGAGTCCTTGGAGCCCCGTGATTCAGCCAGGAACCCTGCAGTCAC
AGCATCTGGATGAGTCAATCCACTCGGGCTGATGTTTCCTCTCGGTTTGCCTTAATAACTGCAGCTCCCTTGCTGCCATC
AAGAGCAGTTATCTGTCTCGCGTAATGTCTCTTTTGATAAGAGGGGGGGGGGGGGGGGGGCTATTATGGAGACCTAGGAG
ATCAAGCTGAGCTGAAATTAGAAGATCTGTTTTAATCAACCGCATCTAAAGTCCTGAGTTTGCGTCTTTTTAGTTCTTTA
TTTAACTCTGATGATAGTGTAGAAGTGCTGTGTTTTAATATGTACTGTATTTGATGACTATACATTACAGATATAGACAA
ACAGAGAGCACATGGAGGTTACAGTAAACAAAATGAGGAAATGCTCATATTGACACAGACATATTGAAGACAGGGGAGAA
GGAGAGAAGCACTGACAGACAGAGAAGTAACTTAATTTCTCGTCCTTGTTTCCCCACCTTGTTGGGTTTGACAGCTCTCT
GAAGATTCTCGATCCATTTGTCTCTCTCTGCACCCGAGCGACATGCAAAGCACTTGGTTCCTGAAGCCGTAGTCACCTGA
GGTTACCAACGGTAACATCAACAGCCACAACCATTAGCAACTTGGGAGAAGATGACAAGTGGCATAACAACGTCCTTTAA
TCATAGAGACTTGTGTCATGTACACGTCGGCTATGGGAAAGTGAGCCATGCAGGGAAAGCCGTTTTCACAACAAATTTCT
GTGACAAATCAACTTGGGACATGAGCTCATTTCGAGCTGCCTGTTGGCTTTCAGTTTGATATCTTTTCCACAGATAACTA
GATTTTAAAAAATCCCAGCCTTATTCAATAAATCCTTTTGTTGATTCCAATGGAGAGACTTCTACCCAACATTAGTAGCA
TTAGTAACAATAGTAGACAGACCTGGGTTCAAATGCTATTTGAAATCTTTAATATGTGTTCATGTGTTTGCTTTAGCCTG
CCTGGAGTGGCAGATGGGCAGGATTTGAACTTTCTTTTCTTCTATTGGTTCCATTGCACCAGGCAAGCTCAATCAAGCCC
AGCAAAGTGATTTGAAATCATTTCAATTAGTATTTGAACCCAGGTTTAATTAGTAGCGCTATCTGTGTTCATCGTGTGTG
TGTGTGTCGTGTCAGCCTCACCTCGAAACAGTACTCCTGTCCCAGGATGCTGCTGTGGACGGGCTTGATGATGGCGTCCT
CGTCCAGGGTTAGGTCCAGCGCCTCAGCTGCGCTGCTGGGAGACAGCAAGGACTCATGGGAGTGGGACTCTTTGAAGCTC
TGCATCAGGCGCGTTCTGGGTGGGGAGCAGAGACACAGTGAGAGGAGCAGAGTGAGTCCCATACATTACACAGCATCAAG
GATACAGTACACAGCATTCTGAACTGTGGTTATAAAGGGTAACTCTCCCACAACCAAACAGCCACATATACACTACCGGT
CAAAAGTTTTAGAACATGTCACTGTCATGCCCTAATCTGTTTCACCTGTTCCTGTGATTGTCTCCACCCCCTCCAGGTGT
CGCTTATTATCCCCAGTCTATTTATCCCTGTGTTTCCTGTCTCTCTGTGTCAGTTCGTCTTGTATGTTTAGTCAAGTCAA
CCAGCGTGTTTTTCCCGTACTCCTTTTGCTATTCTCTTTTTCTAGTTCTCCTGGTTTTGACCCTTGCCTGTTTCTGGACT
CGCCTGCCTGACCTTTCTGCCTGCCTTGACCACGAGCCTGTCTGCCACTCTGTACGTCCTGGACTCTGATCTGGTTTTGA
CCTTTTGCCAACCATTCTCTTGCCTACCCCTTTGGATTATTAAACATTGTAAAACTCCAACCATCTGCCTCCTGTGTCTG
CATCTGGGTCTCGCCTTGTGCCTTGATTGTCACGTCTACTCCTGCTCCTCCCCTCCGGCATTCGACGTCGCCCGTATACT
AACCACCGATCCTAGGATCCATCATTACGCACACCCACCATCAAACATTACGTGCACCTGCACGTCATCATGAAGCATCA
CTGACTCCAACAATGGAAGCAGCAGGAAAACAATATCTCCCAGACGGTCGACAAGCAGGGATACCTTCTACGTCAACAGC
ATGACCAGCTGGCACAAATGGGGACGGCTATGGAAGAGGTTCTTCGCAGTGTGCGACATCTCGAACATACCCGGGAGGCG
TTGCCGTCAACTAGCGGAGGATACTCTACAACCAGTCGACCCAGCGAGCCAGGACAACAGCCCATTCAGCAACCCACTCA
GGTCAGCGATGACCGTTTGTTCCTCCCGGATAAATTGACGGCACCCCATCTAAATGCCGTGGCTTCCTACTTCAGTGCTC
CCTCTACTTTACGCACCAGATGGGAGCCCCACCACCGAGAGTTCCAAGGTTGCCACGGTTATTTTTCTGCTACCTGAGCG
GGCGTTGCAATGGGCCACAGCTGTCAGGAGAGAGGAGGGAAGGAGCGGGAGTAGATGTGACAGTACCCCACCCCCTCGAT
TCCTTCCACGTAGTGAGTTGTGGGCGTACTCTGCCCGTCACACCTCCACTGGTTTGAGCCCCTTCCAGTGTGTTCTGGGT
TTTCAGCCGGACTGAAGCTCCTGCAGTTGATGAGTGGTTCAGGTGCGAGAAAGTGTGGAGCGATGCCCACGTGAGACTCC
AGCGTGCCGTCCACCGTCAAAAGGAGCAGGCGACCGCCCCCGCCCCCCCCGTTCCTCTACCAGGAACCTCCCACTCCGCC
TGTCCTGCAAGAAGCTGAGCCCCCGGTTTGTTGGGCCATTCAAGGTTCTCCGGAGGGTCAACGAGGTGACGTATAGGTTA
CAGCTTCCCAATGACTACCGTATCTGCATCACCACCTGGTATGGAAACGGCTTGGCTTCCAACCGCAAGGCGCTACAGAG
GGCAGTGCGTACGGCCCAATACATCACTGGGGCCGAGCTCCATGCCATCCAGGACCTCTATACCAGGCGGGATCAGAGGA
AGGCCCTAAAAATTGTCAAACACTCCAGCCACCCAAGTCATAGACTCTTCTCTCTGCTTTCGCACGGCAAGTGGTACCGA
TGGACCAAGTCTGGAACCAACAGGACCCTAAACAGCTTCTACCCCCATGCCATAATACTGCTAAATAGCTAGTCCGTGTA

GCTATTGGTTAACTATTTCACTATCTGCATTGACACTTTTTTTGCATCTCTTATGACTCATCACATATGTTACTGTTTAT
TATCTATCCTGTTGCCTATTCACTTTATCCCTACCTTTATCCTGTACATATCTACCTCAATTACCTCATACCTCTGCACA
TCGACTCAGTACTGGTACTGCGTGTATATAGTCAAGTTATCGTTACTCATGGTGTATTTATTCCTCGTGTTATTATTTTT
CAATTTTTTTCTCTGCATTGTTGGGAAAGGCCTGTAAGTAAGCATTTCACTGTGAGGACTACACCTGTTGTTTACGAAGC
ATGTGACAAATACAATTTGATTTGATTAGGGGGATGATTAAGTCAACAAGGCAACTGGCACCATGTGGCCAAAAAAATGC
AACCACTGTCAATATTTTTCATTTAAATGTCGGTACACACTTCTTGCACTAAAGGGACTACCCTCTGTTGATAGCTCCCT
ACAATCTCACTAACAAATCGAACTGTGACAAGGGGAGTGGAATACAATCAGTCATTGAGGGCACAGAGAGCCAGAGATCT
TTCATAACGGCAGGCCCAGTGGAGTTTTTTTGAGATACACTTGAGCATATCTGATTCTGAAAAAATTAGAAACAGCACGC
AACCATTCTGCTTTTCATGCCAAAGGAATCCGTCTGTACTCACAAATTCTGTGTTATGTGAGCTTGAAAAGAGGTCACAT
CAAAAAACAATGCGCTGCTAGTCTCACAATGGATCTAATTGTCATCTGAAGAAGTAAACACATTAGCATTCAATTAGAGT
TTCACTGGATTAGATTAGATTTGAGCATAACCACAATAGTGTTATCATGATAGTTCTTAGACAACAAAGCTATATTTCTA
TTGACAGCCATACTCTACATGCATAAGGCACAGAGCAACACGTGCATAGAATTGTCCATGACGCTTGCCCGCCATGCCCG
CCCACACTGACTTATACATACAGACACACACACACCGATTATGCCACAATGCCACTGAACTGAAGAGGAGACAGATACAG
AATGACAGAAAGGGGAACGACAGAGACCACAACACAGAAAAGCAGAGAAAAGAAAGTAACAGTGTCCCTACTATGATGTC
CCTGATGCATCCAGCCCTCTCACGTTCTCCCTGTGCCACTGTTTTTGTGGACATTCACTCATAATGTGCATCAGCACATA
GAATACTTACAGTGGAGTGGGAAAGAGAGAGAGAGAGAGAGACAGTGGCGGTTCTTGACCATTTCAACTGGGGGGGCCAA
GCTGGGGCCAGTTGTACTGTTAGAGGGGCCAGTTACATTAGACGTTAATGTTGTCATATCGTTTTCTTCACTGCATTTCA
GGCATTATCAGGCAAAAGACCATGAATAATCGTCATCGTTGCCACTGTCTAATAACGGATGTAAAAAAAGAACGATAGCA
AAAATTAGTTCCTTAAAAATTATTTCATACTCCACATTTAGGGGGGCTACAAGGGGGTCCAAAATTGTTGTCACAGGGGC
ACTGGCCCCCCCCTGGCCCCCTGCCCAGAACCGCTAGTGGAGAGAGAGTGAGAGAAAGATAAGAGAGAGATGTGGAGAGA
GAGATAAAAATAGATCCAAAGAAATGAGTCAGAATGCGAGAAATAAAGGACAGTGGGGGAGGTCGAGAAAGGGAGGGCGA
GAGGGCGAGAGAGACAGAGGGAGAGAGAAGGAGAGGGGGAAGACGGTGGGAGAGAGAACCTCTCAGAGGAAATCAAGTTA
TGTAAACTGTGGGTACTTAAGAAAAGAAGGGAGTGACAGGGTCGATGAGAAAACAGAGAGAGAGACTAAGAGAGATATAG
TTGAAGTCGGAAGTTTACATACACCTTAGCCACATACATTCAGTTTTTCACAATTCCTGACAGTTAATCCTAGTAAAAGT
TCCCTGTTTTAGGTCAGTTAAGATCACCACTTTATTTTAAGAATGCGAAATGTCAGAATAATAGTAGAGAGAATTATTTA
TTTCAGCTTTTATTTCTTTCATCACATTCCCAGTGGGTCAGAAATTTACATACACTCAATTAGTATTTGGTAGCACTGCC
TTTAAATTGTTTAACTTGGGTCAAACGTTTCGGGTAGTCTTCCATAAGCTTCCCACAATAAGATGGGTGAATTTTGGCCC
ATTCCTCCTGACAAAGCTGGTGTAACTGAGTCAGGATTGTAGGCCTCCTTGCTCGCACACGCTTTTTCAGTTCTGCCCAA
AAACTTTCTATGGAATTGAGGTCAAGGCTTTGTGATGGCCACTCCAATACCTTGACTTTGTTGTCCTTAAGCCATTTTGC
CACAACTTTGGAAGTATGCTTGGGGTCATTGTCCATTTGGAAGACCCATTTGCGACAAGTTTTAACTTCCTGACTGATGT
CTTGAGATGTTGCTTCAATATATCCACATCATTTTCCTCCCTCATGATGCCATCTATTTTGTGAAGTGCACCAGTCCCTA
CTGCAGCAAACCACCCAGACAACATGATGCTGCCACCCCCGTGCTTCACGGTTGGGATGGTGTTCTTCGGCTTGCGAGCG
TCTCCCGTTTTCCTCCAAACATAACGATGGACATTATGGCCACACTCTATGTTTGTTTCATCAGACCAGAGGACATTTCT
CCAAAAAGTCCCCATGTGCAGTTGCAAACCGTCGTCTGGCTTTTTTATGGCGGTTTTGGAGCAGTGGCTTCTTCCTTGCT
GTGCGGCCTTTCAGGTTATGTCGATATAGGACTCGTTTTACTGTGGATATAGATACTTTTGTACCTGTTTCCTCCAGCAT
CTTCACAAGGTCCTTTGCTGTTGTTCTGGGATTGATTGCACTTTTCGCACCAAAGTACGTTCATCTCTAGGTGACAGAAC
GCATCTCCTTCCTGAGCAGTATGACGGCTGTGTGGTCCCATGGTGTTTATACTTGCGTACTATTGTTTGTACAGATGAAC
GTGGTACCTTCAGGCGTTTGGAGATTGCTCTCAAGGATGAACCAGACTTGTGGTTCTACAAAAAAAATTCTGAGGTCTTG
GCTGATTTCTTTTGATTTTCCCTTGATGTCAAGCAAAGAGGCACTGATTTTGAAGGTAGGCCTTGAAATACATACAGAGG
TACACCTCCAATGGACTTAAATGATGTCAATTAGCCTGTCAGAAGCTTCTAAAGCCATGACATAATTTTCTGGAATTTTC
CAAGCTGTTTAAAGGCACAGTCAACTTAGTGTATGTAGACTTCTGACCCACTGGAATTGTGATACAGTGAATTCTAAGTG
AAATAATCTGTCTGTAAAGAATTGTTGGAAAAGTTACTTGTGTCATGTACAAAGTAGATATCCTAACCGACTTCCCAAAA
CTATAGTTTGTTAACAAGAAATTTGTGGAGTGGTTGAAAAACGAGTTTTAATGACTCCCACCCTAGTGTATACAAACTTC
CGACTTCAACTGTATGTCCGTTCTGTGGCCATACGGCTGTCTGATGGTTGCCATGGAGAGGTCAGGACTTTTTTTATTAA
ATCTTCCATTGGTATTCTCCATGTATTAAGCTGTCATAAATAATGTACTATGTAAAATGGGAGTGCAGCAGCACTGAAAG
GGGCAGTTGTGAAACAAACACTTACTGTCAAGGCTTACTGGTTTCATTAAAGCTAGGAGTAATAGTAAAAGCACAGTACA
TGCCAGATTGTCCTTGAAAGGTATCCAAAATAGACACAAGAAAGCATCTTTAAGAGTCCCACATACATGTGGACACACAG
GTAGGAGTCTACACACACACACACTTAGACAAGTTAAGTGCCTGCCTGACCTGCTCCGGAACAGGGAGAGCAGGGAACAC
ACACACACCCTCACAGAAACACACACAGACACACACACACACACACACACAGACACACACATAGACACACACAGAGAC
TCGTACCTGTCCTGGTCGGCACTACGGAAGCGGGGGAGGATCATGTGTCGGAAGCTGCTGGTGCGGTCCAGTTTGGGCTG
ACTCTTAGCCCTCTTGATAGAGCCCTTCAACCTTCTACTGAGGAAACTCTGAACAGGGCAGCCATGGAGAGAGGGAGGAG
GAGGTGAAGAATGAGAGGGTGTGAAACAGGGATGGATAGAGGGACAGAGGGAGGGAAGGGACGCAGGAAAGGAGATAGAT
CAGAGTTGTAGAGAGGGGGAATAGGGTGAGTAAGGAAAGATCGATATTTGGTGCCCAAAATGGATTGGAAGAAGAACAGA
GGACATATGTGATTTAGTGAATGACAGATGCATGAAGGGGAGAGCAACAGAAAGATTNNNNNNNNNNNNNNNNNNNNNNN
NNNNNNNNNNNNNNNNNNNNNNNNNNNNNNNNNNNNNNNNNNNNNNNNNNNNNNNNNNNNNNNNNNNNNNNNNNNNNNNN
NNNNNNNNNNNNNNNNNNNNNNNNNNNNNNNNNNNNNNNNNNNNNNNNNNNNNNNNNNNNNNNNNNNNNNNNNNNNNNNN
NNNNNNNNNNNNNNNNNNNNNNNNNNNNNNNNNNNNNNNNNNNNNNNNNNNNNNNNNNNNNNNNNNNNNNNNNNNNNNNN
NNNNNNNNNNNNNNNNNNNNNNNNNNNNNNNNNNNNNNNNNNNNNNNNNNNNNNNNNNNNNNNNNNNNNNNNNNNNNNNN
NNNNNNNNNNNNNNNNNNNNNNNNNNNNNNNNNNNNNNNNNNNNNNNNNNNNNNNNNNNNNNNNNNNNNNNNNNNNNNNN
NNNNNNNNNNNNNNNNNNNNNNNNNNNNNNNNNNNNNNNNNNNNNNNNNNNNNNNNNNNNNNNNNNNNNNNNNNNNNNNN
NNNNNNNNNNNNNNNNNNNNNNNNNNNNNNNNNNNNNNNNNNNNNNNNNNNNNNNNNNNNNNNNNNNNNNNNNNNNNNNN
NNNNNNNNNNNNNNNNNNNNNNNNNNNNNNNNNNNNNNNNNNNNNNNNNNNNNNNNNNNNNNNNNNNNNNNNNNNNNNNN
NNNNNNNNNNNNNNNNNNNNNNNNNNNNNNNNNNNNNNNNNNNNNNNNNNNNNNNNNNNNNNNNNNNNNNNNNNNNNNNN
NNNNNNNNNNNNNNNNNNNNNNNNNNNNNNNNNNNNNNNNNNNNNNNNNNNNNNNNNNNNNNNNNNNNNNNNNNNNNNNN
NNNNNNNNNNNNNNNNNNNNNNNNNNNNNNNNNNNNNNNNNNNNNNNNNNNNNNNNNNNNNNNNNNNNNNNNNNNNNNNN
NNNNNNNNNNNNNNNNNNNNNNNNNNNNNNNNNNNNNNNNNNNNNNNNNNNNNNNNNNNNNNNNNNNNNNNNNNNNNNNN
NNNNNNNNNNNNNNNNNNNNNNNNNNNNNNNNNNNNNNNNNNNNNNNNNNNNNNNNNNNNNNNNNNNNNNNNNNNNNNNN
NNNNNNNNNNNNNNNNNNNNNNNNNNNNNNNNNNNNNNNNNNNNNNNNNNNNNNNNNNNNNNNNNNNNNNNNNNNNNNNN
NNNNNNNNNNNNNNNNNNNNNNNNNNNNNNNNNNNNNNNNNNNNNNNNNNNNNNNNNNNNNNNNNNNNNNNNNNNNNNNN

NNNNNNNNNNNNNNNNNNNNNNNNNNNNNNNNNNNNNNNNNNNNNNNNNNNNNNNNNNNNNNNNNNNNNNNNNNNNNNNN
NNNNNNNNNNNNNNNNNNNNNNNNNNNNNNNNNNNNNNNNNNNNNNNNNNNNNNNNNNNNNNNNNNNNNNNNNNNNNNNN
NNNNNNNNNNNNNNNNNNNNNNNNNNNNNNNNNNNNNNNNNNNNNNNNNNNNNNNNNNNNNNNNNNNNNNNNNNNNNNNN
NNNNNNNNNNNNNNNNNNNNNNNNNNNNNNNNNNNNNNNNNNNNNNNNNNNNNNNNNNNNNNNNNNNNNNNNNNNNNNNN
NNNNNNNNNNNNNNNNNNNNNNNNNNNNNNNNNNNNNNNNNNNNNNNNNNNNNNNNNNNNNNNNNNNNNNNNNNNNNNNN
NNNNNNNNNNNNNNNNNNNNNNNNNNNNNNNNNNNNNNNNNNNNNNNNNNNNNNNNNNNNNNNNNNNNNNNNNNNNNNNN
NNNNNNNNNNNNNNNNNNNNNNNNNNNNNNNNNNNNNNNNNNNNNNNNNNNNNNNNNNNNNNNNNNNNNNNNNNNNNNNN
NNNNNNNNNNNNNNNNNNNNNNNNNNNNNNNNNNNNNNNNNNNNNNNNNNNNNNNNNNNNNNNNNNNNNNNNNNNNNNNN
NNNNNNNNNNNNNNNNNNNNNNNNNNNNNNNNNNNNNNNNNNNNNNNNNNNNNNNNNNNNNNNNNNNNNNNNNNNNNNNN
NNNNNNNNNNNNNNNNNNNNNNNNNNNNNNNNNNNNNNNNNNNNNNNNNNNNNNNNNNNNNNNNNNNNNNNNNNNNNNNN
NNNNNNNNNNNNNNNNNNNNNNNNNNNNNNNNNNNNNNNNNNNNNNNNNNNNNNNNNNNNNNNNNNNNNNNNNNNNNNNN
NNNNNNNNNNNNNNNNNNNNNNNNNNNNNNNNNNNNNNNNNNNNNNNNNNNNNNNNNNNNNNNNNNNNNNNNNNNNNNNN
NNNNNNNNNNNNNNNNNNNNNNNNNNNNNNNNNNNNNNNNNNNNNNNNNNNNNNNNNNNNNNNNNNNNNNNNNNNNNNNN
NNNNNNNNNNNNNNNNNNNNNNNNNNNNNNNNNNNNNNNNNNNNNNNNNNNNNNNNNNNNNNNNNNNNNNNNNNNNNNNN
NNNNNNNNNNNNNNNNNNNNNNNNNNNNNNNNNNNNNNNNNNNNNNNNNNNNNNNNNNNNNNNNNNNNNNNNNNNNNNNN
NNNNNNNNNNNNNNNNNNNNNNNNNNNNNNNNNNNNNNNNNNNNNNNNNNNNNNNNNNNNNNNNNNNNNNNNNNNNNNNN
NNNNNNNNNNNNNNNNNNNNNNNNNNNNNNNNNNNNNNNNNNNNNNNNNNNNNNNNNNNNNNNNNNNNNNNNNNNNNNNN
NNNNNNNNNNNNNNNNNNNNNNNNNNNNNNNNNNNNNNNNNNNNNNNNNNNNNNNNNNNNNNNNNNNNNNNNNNNNNNNN
NNNNNNNNNNNNNNNNNNNNNNNNNNNNNNNNNNNNNNNNNNNNNNNNNNNNNNNNNNNNNNNNNNNNNNNNNNNNNNNN
NNNNNNNNNNNNNNNNNNNNNNNNNNNNNNNNNNNNNNNNNNNNNNNNNNNNNNNNNNNNNNNNNNNNNNNNNNNNNNNN
NNNNNNNNNNNNNNNNNNNNNNNNNNNNNNNNNNNNNNNNNNNNNNNNNNNNNNNNNNNNNNNNNNNNNNNNNNNNNNNN
NNNNNNNNNNNNNNNNNNNNNNNNNNNNNNNNNNNNNNNNNNNNNNNNNNNNNNNNNNNNNNNNNNNNNNNNNNNNNNNN
NNNNNNNNNNNNNNNNNNNNNNNNNNNNNNNNNNNNNNNNNNNNNNNNNNNNNNNNNNNNNNNNNNNNNNNNNNNNNNNN
NNNNNNNNNNNNNNNNNNNNNNNNNNNNNNNNNNNNNNNNNNNNNNNNNNNNNNNNNNNNNNNNNNNNNNNNNNNNNNNN
NNNNNNNNNNNNNNNNNNNNNNNNNNNNNNNNNNNNNNNNNNNNNNNNNNNNNNNNNNNNNNNNNNNNNNNNNNNNNNNN
NNNNNNNNNNNNNNNNNNNNNNNNNNNNNNNNNNNNNNNNNNNNNNNNNNNNNNNNNNNNNNNNNNNNNNNNNNNNNNNN
NNNNNNNNNNNNNNNNNNNNNNNNNNNNNNNNNNNNNNNNNNNNNNNNNNNNNNNNNNNNNNNNNNNNNNNNNNNNNNNN
NNNNNNNNNNNNNNNNNNNNNNNNNNNNNNNNNNNNNNNNNNNNNNNNNNNNNNNNNNNNNNNNNNNNNNNNNNNNNNNN
NNNNNNNNNNNNNNNNNNNNNNNNNNNNNNNNNNNNNNNNNNNNNNNNNNNNNNNNNNNNNNNNNNNNNNNNNNNNNNNN
NNNNNNNNNNNNNNNNNNNNNNNNNNNNNNNNNNNNNNNNNNNNNNNNNNNNNNNNNNNNNNNNNNNNNNNNNNNNNNNN
NNNNNNNNNNNNNNNNNNNNNNNNNNNNNNNNNNNNNNNNNNNNNNNNNNNNNNNNNNNNNNNNNNNNNNNNNNNNNNNN
NNNNNNNNNNNNNNNNNNNNNNNNNNNNNNNNNNNNNNNNNNNNNNNNNNNNNNNNNNNNNNNNNNNNNNNNNNNNNNNN
NNNNNNNNNNNNNNNNNNNNNNNNNNNNNNNNNNNNNNNNNNNNNNNNNNNNNNNNNNNNNNNNNNNNNNNNNNNNNNNN
NNNNNNNNNNNNNNNNNNNNNNNNNNNNNNNNNNNNNNNNNNNNNNNNNNNNNNNNNNNNNNNNNNNNNNNNNNNNNNNN
NNNNNNNNNNNNNNNNNNNNNNNNNNNNNNNNNNNNNNNNNNNNNNNNNNNNNNNNNNNNNNNNNNNNNNNNNNNNNNNN
NNNNNNNNNNNNNNNNNNNNNNNNNNNNNNNNNNNNNNNNNNNNNNNNNNNNNNNNNNNNNNNNNNNNNNNNNNNNNNNN
NNNNNNNNNNNNNNNNNNNNNNNNNNNNNNNNNNNNNNNNNNNNNNNNNNNNNNNNNNNNNNNNNNNNNNNNNNNNNNNN
NNNNNNNNNNNNNNNNNNNNNNNNNNNNNNNNNNNNNNNNNNNNNNNNNNNNNNNNNNNNNNNNNNNNNNNNNNNNNNNN
NNNNNNNNNNNNNNNNNNNNNNNNNNNNNNNNNNNNNNNNNNNNNNNNNNNNNNNNNNNNNNNNNNNNNNNNNNNNNNNN
NNNNNNNNNNNNNNNNNNNNNNNNNNNNNNNNNNNNNNNNNNNNNNNNNNNNNNNNNNNNNNNNNNNNNNNNNNNNNNNN
NNNNNNNNNNNNNNNNNNNNNNNNNNNNNNNNNNNNNNNNNNNNNNNNNNNNNNNNNNNNNNNNNNNNNNNNNNNNNNNN
NNNNNNNNNNNNNNNNNNNNNNNNNNNNNNNNNNNNNNNNNNNNNNNNNNNNNNNNNNNNNNNNNNNNNNNNNNNNNNNN
NNNNNNNNNNNNNNNNNNNNNNNNNNNNNNNNNNNNNNNNNNNNNNNNNNNNNNNNNNNNNNNNNNNNNNNNNNNNNNNN
NNNNNNNNNNNNNNNNNNNNNNNNNNNNNNNNNNNNNNNNNNNNNNNNNNNNNNNNNNNNNNNNNNNNNNNNNNNNNNNN
NNNNNNNNNNNNNNNNNNNNNNNNNNNNNNNNNNNNNNNNNNNNNNNNNNNNNNNNNNNNNNNNNNNNNNNNNNNNNNNN
NNNNNNNNNNNNNNNNNNNNNNNNNNNNNNNNNNNNNNNNNNNNNNNNNNNNNNNNNNNNNNNNNNNNNNNNNNNNNNNN
NNNNNNNNNNNNNNNNNNNNNNNNNNNNNNNNNNNNNNNNNNNNNNNNNNNNNNNNNNNNNNNNNNNNNNNNNNNNNNNN
NNNNNNNNNNNNNNNNNNNNNNNNNNNNNNNNNNNNNNNNNNNNNNNNNNNNNNNNNNNNNNNNNNNNNNNNNNNNNNNN
NNNNNNNNNNNNNNNNNNNNNNNNNNNNNNNNNNNNNNNNNNNNNNNNNNNNNNNNNNNNNNNNNNNNNNNNNNNNNNNN
NNNNNNNNNNNNNNNNNNNNNNNNNNNNNNNNNNNNNNNNNNNNNNNNNNNNNNNNNNNNNNNNNNNNNNNNNNNNNNNN
NNNNNNNNNNNNNNNNNNNNNNNNNNNNNNNNNNNNNNNNNNNNNNNNNNNNNNNNNNNNNNNNNNNNNNNNNNNNNNNN
NNNNNNNNNNNNNNNNNNNNNNNNNNNNNNNNNNNNNNNNNNNNNNNNNNNNNNNNNNNNNNNNNNNNNNNNNNNNNNNN
NNNNNNNNNNNNNNNNNNNNNNNNNNNNNNNNNNNNNNNNNNNNNNNNNNNNNNNNNNNNNNNNNNNNNNNNNNNNNNNN
NNNNNNNNNNNNNNNNNNNNNNNNNNNNNNNNNNNNNNNNNNNNNNNNNNNNNNNNNNNNNNNNNNNNNNNNNNNNNNNN
NNNNNNNNNNNNNNNNNNNNNNNNNNNNNNNNNNNNNNNNNNNNNNNNNNNNNNNNNNNNNNNNNNNNNNNNNNNNNNNN
NNNNNNNNNNNNNNNNNNNNNNNNNNNNNNNNNNNNNNNNNNNNNNNNNNNNNNNNNNNNNNNNNNNNNNNNNNNNNNNN
NNNNNNNNNNNNNNNNNNNNNNNNNNNNNNNNNNNNNNNNNNNNNNNNNNNNNNNNNNNNNNNNNNNNNNNNNNNNNNNN
NNNNNNNNNNNNNNNNNNNNNNNNNNNNNNNNNNNNNNNNNNNNNNNNNNNNNNNNNNNNNNNNNNNNNNNNNNNNNNNN
NNNNNNNNNNNNNNNNNNNNNNNNNNNNNNNNNNNNNNNNNNNNNNNNNNNNNNNNNNNNNNNNNNNNNNNNNNNNNNNN
NNNNNNNNNNNNNNNNNNNNNNNNNNNNNNNNNNNNNNNNNNNNNNNNNNNNNNNNNNNNNNNNNNNNNNNNNNNNNNNN
NNNNNNNNNNNNNNNNNNNNNNNNNNNNNNNNNNNNNNNNNNNNNNNNNNNNNNNNNNNNNNNNNNNNNNNNNNNNNNNN
NNNNNNNNNNNNNNNNNNNNNNNNNNNNNNNNNNNNNNNNNNNNNNNNNNNNNNNNNNNNNNNNNNNNNNNNNNNNNNNN
NNNNNNNNNNNNNNNNNNNNNNNNNNNNNNNNNNNNNNNNNNNNNNNNNNNNNNNNNNNNNNNNNNNNNNNNNNNNNNNN
NNNNNNNNNNNNNNNNNNNNNNNNNNNNNNNNNNNNNNNNNNNNNNNNNNNNNNNNNNNNNNNNNNNNNNNNNNNNNNNN
NNNNNNNNNNNNNNNNNNNNNNNNNNNNNNNNNNNNNNNNNNNNNNNNNNNNNNNNNNNNNNNNNNNNNNNNNNNNNNNN
NNNNNNNNNNNNNNNNNNNNNNNNNNNNNNNNNNNNNNNNNNNNNNNNNNNNNNNNNNNNNNNNNNNNNNNNNNNNNNNN
NNNNNNNNNNNNNNNNNNNNNNNNNNNNNNNNNNNNNNNNNNNNNNNNNNNNNNNNNNNNNNNNNNNNNNNNNNNNNNNN
NNNNNNNNNNNNNNNNNNNNNNNNNNNNNNNNNNNNNNNNNNNNNNNNNNNNNNNNNNNNNNNNNNNNNNNNNNNNNNNN
NNNNNNNNNNNNNNNNNNNNNNNNNNNNNNNNNNNNNNNNNNNNNNNNNNNNNNNNNNNNNNNNNNNNNNNNNNNNNNNN

NNNNNNNNNNNNNNNNNNNNNNNNNNNNNNNNNNNNNNNNNNNNNNNNNNNNNNNNNNNNNNNNNNNNNNNNNNNNNNNN
NNNNNNNNNNNNNNNNNNNNNNNNNNNNNNNNNNNNNNNNNNNNNNNNNNNNNNNNNNNNNNNNNNNNNNNNNNNNNNNN
NNNNNNNNNNNNNNNNNNNNNNNNNNNNNNNNNNNNNNNNNNNNNNNNNNNNNNNNNNNNNNNNNNNNNNNNNNNNNNNN
NNNNNNNNNNNNNNNNNNNNNNNNNNNNNNNNNNNNNNNNNNNNNNNNNNNNNNNNNNNNNNNNNNNNNNNNNNNNNNNN
NNNNNNNNNNNNNNNNNNNNNNNNNNNNNNNNNNNNNNNNNNNNNNNNNNNNNNNNNNNNNNNNNNNNNNNNNNNNNNNN
NNNNNNNNNNNNNNNNNNNNNNNNNNNNNNNNNNNNNNNNNNNNNNNNNNNNNNNNNNNNNNNNNNNNNNNNNNNNNNNN
NNNNNNNNNNNNNNNNNNNNNNNNNNNNNNNNNNNNNNNNNNNNNNNNNNNNNNNNNNNNNNNNNNNNNNNNNNNNNNNN
NNNNNNNNNNNNNNNNNNNNNNNNNNNNNNNNNNNNNNNNNNNNNNNNNNNNNNNNNNNNNNNNNNNNNNNNNNNNNNNN
NNNNNNNNNNNNNNNNNNNNNNNNNNNNNNNNNNNNNNNNNNNNNNNNNNNNNNNNNNNNNNNNNNNNNNNNNNNNNNNN
NNNNNNNNNNNNNNNNNNNNNNNNNNNNNNNNNNNNNNNNNNNNNNNNNNNNNNNNNNNNNNNNNNNNNNNNNNNNNNNN
NNNNNNNNNNNNNNNNNNNNNNNNNNNNNNNNNNNNNNNNNNNNNNNNNNNNNNNNNNNNNNNNNNNNNNNNNNNNNNNN
NNNNNNNNNNNNNNNNNNNNNNNNNNNNNNNNNNNNNNNNNNNNNNNNNNNNNNNNNNNNNNNNNNNNNNNNNNNNNNNN
NNNNNNNNNNNNNNNNNNNNNNNNNNNNNNNNNNNNNNNNNNNNNNNNNNNNNNNNNNNNNNNNNNNNNNNNNNNNNNNN
NNNNNNNNNNNNNNNNNNNNNNNNNNNNNNNNNNNNNNNNNNNNNNNNNNNNNNNNNNNNNNNNNNNNNNNNNNNNNNNN
NNNNNNNNNNNNNNNNNNNNNNNNNNNNNNNNNNNNNNNNNNNNNNNNNNNNNNNNNNNNNNNNNNNNNNNNNNNNNNNN
NNNNNNNNNNNNNNNNNNNNNNNNNNNNNNNNNNNNNNNNNNNNNNNNNNNNNNNNNNNNNNNNNNNNNNNNNNNNNNNN
NNNNNNNNNNNNNNNNNNNNNNNNNNNNNNNNNNNNNNNNNNNNNNNNNNNNNNNNNNNNNNNNNNNNNNNNNNNNNNNN
NNNNNNNNNNNNNNNNNNNNNNNNNNNNNNNNNNNNNNNNNNNNNNNNNNNNNNNNNNNNNNNNNNNNNNNNNNNNNNNN
NNNNNNNNNNNNNNNNNNNNNNNNNNNNNNNNNNNNNNNNNNNNNNNNNNNNNNNNNNNNNNNNNNNNNNNNNNNNNNNN
NNNNNNNNNNNNNNNNNNNNNNNNNNNNNNNNNNNNNNNNNNNNNNNNNNNNNNNNNNNNNNNNNNNNNNNNNNNNNNNN
NNNNNNNNNNNNNNNNNNNNNNNNNNNNNNNNNNNNNNNNNNNNNNNNNNNNNNNNNNNNNNNNNNNNNNNNNNNNNNNN
NNNNNNNNNNNNNNNNNNNNNNNNNNNNNNNNNNNNNNNNNNNNNNNNNNNNNNNNNNNNNNNNNNNNNNNNNNNNNNNN
NNNNNNNNNNNNNNNNNNNNNNNNNNNNNNNNNNNNNNNNNNNNNNNNNNNNNNNNNNNNNNNNNNNNNNNNNNNNNNNN
NNNNNNNNNNNNNNNNNNNNNNNNNNNNNNNNNNNNNNNNNNNNNNNNNNNNNNNNNNNNNNNNNNNNNNNNNNNNNNNN
NNNNNNNNNNNNNNNNNNNNNNNNNNNNNNNNNNNNNNNNNNNNNNNNNNNNNNNNNNNNNNNNNNNNNNNNNNNNNNNN
NNNNNNNNNNNNNNNNNNNNNNNNNNNNNNNNNNNNNNNNNNNNNNNNNNNNNNNNNNNNNNNNNNNNNNNNNNNNNNNN
NNNNNNNNNNNNNNNNNNNNNNNNNNNNNNNNNNNNNNNNNNNNNNNNNNNNNNNNNNNNNNNNNNNNNNNNNNNNNNNN
NNNNNNNNNNNNNNNNNNNNNNNNNNNNNNNNNNNNNNNNNNNNNNNNNNNNNNNNNNNNNNNNNNNNNNNNNNNNNNNN
NNNNNNNNNNNNNNNNNNNNNNNNNNNNNNNNNNNNNNNNNNNNNNNNNNNNNNNNNNNNNNNNNNNNNNNNNNNNNNNN
NNNNNNNNNNNNNNNNNNNNNNNNNNNNNNNNNNNNNNNNNNNNNNNNNNNNNNNNNNNNNNNNNNNNNNNNNNNNNNNN
NNNNNNNNNNNNNNNNNNNNNNNNNNNNNNNNNNNNNNNNNNNNNNNNNNNNNNNNNNNNNNNNNNNNNNNNNNNNNNNN
NNNNNNNNNNNNNNNNNNNNNNNNNNNNNNNNNNNNNNNNNNNNNNNNNNNNNNNNNNNNNNNNNNNNNNNNNNNNNNNN
NNNNNNNNNNNNNNNNNNNNNNNNNNNNNNNNNNNNNNNNNNNNNNNNNNNNNNNNNNNNNNNNNNNNNNNNNNNNNNNN
NNNNNNNNNNNNNNNNNNNNNNNNNNNNNNNNNNNNNNNNNNNNNNNNNNNNNNNNNNNNNNNNNNNNNNNNNNNNNNNN
NNNNNNNNNNNNNNNNNNNNNNNNNNNNNNNNNNNNNNNNNNNNNNNNNNNNNNNNNNNNNNNNNNNNNNNNNNNNNNNN
NNNNNNNNNNNNNNNNNNNNNNNNNNNNNNNNNNNNNNNNNNNNNNNNNNNNNNNNNNNNNNNNNNNNNNNNNNNNNNNN
NNNNNNNNNNNNNNNNNNNNNNNNNNNNNNNNNNNNNNNNNNNNNNNNNNNNNNNNNNNNNNNNNNNNNNNNNNNNNNNN
NNNNNNNNNNNNNNNNNNNNNNNNNNNNNNNNNNNNNNNNNNNNNNNNNNNNNNNNNNNNNNNNNNNNNNNNNNNNNNNN
NNNNNNNNNNNNNNNNNNNNNNNNNNNNNNNNNNNNNNNNNNNNNNNNNNNNNNNNNNNNNNNNNNNNNNNNNNNNNNNN
NNNNNNNNNNNNNNNNNNNNNNNNNNNNNNNNNNNNNNNNNNNNNNNNNNNNNNNNNNNNNNNNNNNNNNNNNNNNNNNN
NNNNNNNNNNNNNNNNNNNNNNNNNNNNNNNNNNNNNNNNNNNNNNNNNNNNNNNNNNNNNNNNNNNNNNNNNNNNNNNN
NNNNNNNNNNNNNNNNNNNNNNNNNNNNNNNNNNNNNNNNNNNNNNNNNNNNNNNNNNNNNNNNNNNNNNNNNNNNNNNN
NNNNNNNNNNNNNNNNNNNNNNNNNNNNNNNNNNNNNNNNNNNNNNNNNNNNNNNNNNNNNNNNNNNNNNNNNNNNNNNN
NNNNNNNNNNNNNNNNNNNNNNNNNNNNNNNNNNNNNNNNNNNNNNNNNNNNNNNNNNNNNNNNNNNNNNNNNNNNNNNN
NNNNNNNNNNNNNNNNNNNNNNNNNNNNNNNNNNNNNNNNNNNNNNNNNNNNNNNNNNNNNNNNNNNNNNNNNNNNNNNN
NNNNNNNNNNNNNNNNNNNNNNNNNNNNNNNNNNNNNNNNNNNNNNNNNNNNNNNNNNNNNNNNNNNNNNNNNNNNNNNN
NNNNNNNNNNNNNNNNNNNNNNNNNNNNNNNNNNNNNNNNNNNNNNNNNNNNNNNNNNNNNNNNNNNNNNNNNNNNNNNN
NNNNNNNNNNNNNNNNNNNNNNNNNNNNNNNNNNNNNNNNNNNNNNNNNNNNNNNNNNNNNNNNNNNNNNNNNNNNNNNN
NNNNNNNNNNNNNNNNNNNNNNNNNNNNNNNNNNNNNNNNNNNNNNNNNNNNNNNNNNNNNNNNNNNNNNNNNNNNNNNN
NNNNNNNNNNNNNNNNNNNNNNNNNNNNNNNNNNNNNNNNNNNNNNNNNNNNNNNNNNNNNNNNNNNNNNNNNNNNNNNN
NNNNNNNNNNNNNNNNNNNNNNNNNNNNNNNNNNNNNNNNNNNNNNNNNNNNNNNNNNNNNNNNNNNNNNNNNNNNNNNN
NNNNNNNNNNNNNNNNNNNNNNNNNNNNNNNNNNNNNNNNNNNNNNNNNNNNNNNNNNNNNNNNNNNNNNNNNNNNNNNN
NNNNNNNNNNNNNNNNNNNNNNNNNNNNNNNNNNNNNNNNNNNNNNNNNNNNNNNNNNNNNNNNNNNNNNNNNNNNNNNN
NNNNNNNNNNNNNNNNNNNNNNNNNNNNNNNNNNNNNNNNNNNNNNNNNNNNNNNNNNNNNNNNNNNNNNNNNNNNNNNN
NNNNNNNNNNNNNNNNNNNNNNNNNNNNNNNNNNNNNNNNNNNNNNNNNNNNNNNNNNNNNNNNNNNNNNNNNNNNNNNN
NNNNNNNNNNNNNNNNNNNNNNNNNNNNNNNNNNNNNNNNNNNNNNNNNNNNNNNNNNNNNNNNNNNNNNNNNNNNNNNN
NNNNNNNNNNNNNNNNNNNNNNNNNNNNNNNNNNNNNNNNNNNNNNNNNNNNNNNNNNNNNNNNNNNNNNNNNNNNNNNN
NNNNNNNNNNNNNNNNNNNNNNNNNNNNNNNNNNNNNNNNNNNNNNNNNNNNNNNNNNNNNNNNNNNNNNNNNNNNNNNN
NNNNNNNNNNNNNNNNNNNNNNNNNNNNNNNNNNNNNNNNNNNNNNNNNNNNNNNNNNNNNNNNNNNNNNNNNNNNNNNN
NNNNNNNNNNNNNNNNNNNNNNNNNNNNNNNNNNNNNNNNNNNNNNNNNNNNNNNNNNNNNNNNNNNNNNNNNNNNNNNN
NNNNNNNNNNNNNNNNNNNNNNNNNNNNNNNNNNNNNNNNNNNNNNNNNNNNNNNNNNNNNNNNNNNNNNNNNNNNNNNN
NNNNNNNNNNNNNNNNNNNNNNNNNNNNNNNNNNNNNNNNNNNNNNNNNNNNNNNNNNNNNNNNNNNNNNNNNNNNNNNN
NNNNNNNNNNNNNNNNNNNNNNNNNNNNNNNNNNNNNNNNNNNNNNNNNNNNNNNNNNNNNNNNNNNNNNNNNNNNNNNN
NNNNNNNNNNNNNNNNNNNNNNNNNNNNNNNNNNNNNNNNNNNNNNNNNNNNNNNNNNNNNNNNNNNNNNNNNNNNNNNN
NNNNNNNNNNNNNNNNNNNNNNNNNNNNNNNNNNNNNNNNNNNNNNNNNNNNNNNNNNNNNNNNNNNNNNNNNNNNNNNN
NNNNNNNNNNNNNNNNNNNNNNNNNNNNNNNNNNNNNNNNNNNNNNNNNNNNNNNNNNNNNNNNNNNNNNNNNNNNNNNN
NNNNNNNNNNNNNNNNNNNNNNNNNNNNNNNNNNNNNNNNNNNNNNNNNNNNNNNNNNNNNNNNNNNNNNNNNNNNNNNN
NNNNNNNNNNNNNNNNNNNNNNNNNNNNNNNNNNNNNNNNNNNNNNNNNNNNNNNNNNNNNNNNNNNNNNNNNNNNNNNN
NNNNNNNNNNNNNNNNNNNNNNNNNNNNNNNNNNNNNNNNNNNNNNNNNNNNNNNNNNNNNNNNNNNNNNNNNNNNNNNN

NNNNNNNNNNNNNNNNNNNNNNNNNNNNNNNNNNNNNNNNNNNNNNNNNNNNNNNNNNNNNNNNNNNNNNNNNNNNNNNNNNNNNN
NNNNNNNNNNNNNNNNNNNNNNNNNNNNNNNNNNNNNNNNNNNNNNNNNNNNNNNNNNNNNNNNNNNNNNNNNNNNNNNNNNNNNN
NNNNNNNNNNNNNNNNNNNNNNNNNNNNNNNNNNNNNNNNNNNNNNNNNNNNNNNNNNNNNNNNNNNNNNNNNNNNNNNNNNNNNN
NNNNNNNNNNNNNNNNNNNNNNNNNNNNNNNNNNNNNNNNNNNNNNNNNNNNNNNNNNNNNNNNNNNNNNNNNNNNNNNNNNNNNN
NNNNNNNNNNNNNNNNNNNNNNNNNNNNNNNNNNNNNNNNNNNNNNNNNNNNNNNNNNNNNNNNNNNNNNNNNNNNNNNNNNNNNN
NNNNNNNNNNNNNNNNNNNNNNNNNNNNNNNNNNNNNNNNNNNNNNNNNNNNNNNNNNNNNNNNNNNNNNNNNNNNNNNNNNNNNN
NNNNNNNNNNNNNNNNNNNNNNNNNNNNNNNNNNNNNNNNNNNNNNNNNNNNNNNNNNNNNNNNNNNNNNNNNNNNNNNNNNNNNN
NNNNNNNNNNNNNNNNNNNNNNNNNNNNNNNNNNTCCTTTAGGTACAAGCAGCTTGAACGAAAAGGTACAATCATGCTATTTG
TGGGGAACCCCTGGCTAAGATCACCATGTTTACAGCAGCTTTGACGAGCCCCACAGTGAAAAACAATTAAATTCAACCCT
CATTTACTGCCGTTTTTAAAAACATAGCTGGGATATGGCTGACTTGCTTAAGCAAATGTGGTTTCTACTGACAATTGAGA
TGTACAAGCCATAGCATAAGGGAACGATGAGCGAATAAGAGGCAATCCTTAATTTTGATTAAGACATTAACGAGCCATCT
AAGACGGACGTTGTCAATATAACTATATATCAGCACTTTTGAAATGTACAGCGACAGAATTCAGAACATGGGCCATTCTT
ACAGTATTCTCCCTGTACACCAAGTCAGAACCGAAGGATAAATAAAGGGGGCATACTGTATAAGCAGACAATGAAAGCTC
TTACAATATTCGATGACGACATTTCTCTAAAACAGGCTATAGGCTACATGTGCACCACCAAGTCAGAACAGTAGGCCAAG
TCATGAGGGGGAAAGGGACCAAATGATTAAGGTGATGCACATGGGCTACTAACAGCTTACTACACAACATACACTTACTA
TTACTTTCTTAGCTGCAGTATACAATACAATACAAATACAATACAAAATTATTACATAATTTATGCAGCAGCAAGACATT
TTAGGACTCACCATTGTGTTGTGCTCACTTGAACAGGAAGGTGGCGCAGCAGTCCTTACTGTGGGCAAATTTTGTCATGA
AACTTTGTCATCAAAGTCTGGCATTCTCTAGATTTATGGTGCTTTCAAGACAACTGGGAACTCGGAAAAAAACAAGATTG
AATCATGACATCTTCAGGTCAGAGCTCTAGAAAGAGGCCTGAGTTCCCGACTGGAATTCCGTATTGGATGACCGTTCAAA
ACTTATTTTCCCAGTCGGAGCTCGTTTTTTCAGAGTTCCCAGTTGTCAAGAACACACTGAAGTCTGAGATTTCCCAGTTC
TGCGTTTCAAGTTGTTTTGAACGCGGCAGAAGTCATGCTGGATTGACAGCATGGCCAATGTATTCAACCTTTTCTGGCCT
ATGGTGTTGTGTGTGAATGTTTATCCTTTTAAGCTTTGAAAAGAGACCCTTAAACCCAGACTTGGACCACACACCCTCTC
CACCAAATAGCAGGCAGGGGAAGCAAAATAGTGATTGCTTTGCAATGCTTGCAGTTAGCCACTGATTCCTTCCAAACCAC
TCATTGTTGAATATGTTGTTGTGTAATGTTTATGTCCAATGGCCGATGAGCACCGATACATATTATCTATGATTTCTCTT
TATATGACAAGGATTGAAAAGGATTTACCAGTAGATTGTTGATGTGATTCATGATAATGACTGTTTTCTCTAACTTGCTA
GTTAAGATTTTGAAAGTATGATGTTGACATGATCAGTCCAATCAAAGCTACTGTAGATATAACGTGATTTGATGTCATTT
TATCTGTGGCCAACGACCTTGAGCCTTCTTGGATGGGCACTTCTAATGTAAGCCTATGGCAGCACCCAAGGGGGCTTGAA
CTTTCTAGCTCTCCCTGTAGATTTTGAGATTAAGTAATGTCACCATGAGTGACAGAACACTGAGCCAATCACGGCGCAAC
TAGAGAAGATTAACAACCCCTATGCTCTGTATTTTCCGCTGGCTGCCCCTCCACAGAAGCAACTGTGCTAGGCTGAAACA
CTTGCATTTTGGAGCTGCTTTACTCAAGGAAGCAAAAAAGAGTCCATGTCTGCATGCAGCTTTATCAACTCAATACATTT
ATTTTTACATTGTTTGCAAACTGATATGTGACACATTAATGCCTAAATAACAAGCAAAACAGGCAAACAATATATATTTT
TCTTTTAAAATAAACAGGTGGGTCTCAAAACAGGCTCTGCCCCACCTGCCCTGAATGACGGGTCGCCACTGACCTAGCTT
GAGACTCGAGTTTTGAAATATAAGGGCCTACAGTACATGGAGAAAATCAGACCTCTCTTACTATATATAAAAATAGCCAC
ATTTCTTCCCAGTCAATGTAAATAGCCTATCATTTTTAGGAAGTTAATTTCATTGGAAATATAACTTTGCCCTGTACTTA
TTTTCCCATTCACTGTTAGGCCCTATATACTACAGATAATTTAATTGACATCACACAAAAGGCTACATTGTAGGCGCACT
TGATCACCTGTGCCCAGCAGAGAATTGAACTCAGCTTGCATTTTGACTCAGAAAGTGATCTCAATAAAGAAAAGGTTGGT
GACCACTGTTCTAGTCTATATCCCAGCAAACAGAAGATGACCTGAGGACATTCGGCTGCATTCCCATTAGGTCCCTAAAG
GGTCTCAGTGTGATGTTATCCCATTGACGTTCTTAGAACGTTGCGTGATGGTCCCAAGGGAGAATTATTTGGGGTACTTC
TGTCTAGTTCCTTGTACGTTCCCAGGACGTCACTGAGGACCATGTCAGGACCTTTCTCGGACGTTCTCATGACTTTCATT
TTACTAGTCCTTAGGACGTTGTGCAATGGTCCCAAGGGAACGTTCTCTGGGGGACCTTTTTATAGTACCAGGTTTGACTC
CTGAAGGTTCCCCTGAACGTTCTTAGTACGTTGTGTCATGGTCCCTTGGAGGATTTGTGTAGTTCCTGGTTGGACCCGGG
GACATCCCCAAAGACATTTTTAAAATGTTCTTTGGACGTTGTGTCTTAAGGAACATTATGCATTTAAGAAAATGCATAAA
AAAAACAGAGTGGCATATTTTGAGCTTAAATATAAGTGCTTTGAGTGTTGACAGTAATCACAGTAATTCTGCCAAAAAGT
TGTATTTTGAAAAACAGCTCAATGGGTCATTTTTAAGAGTCATTTGGAAACTGCAGAATCTGCTCTTTCTGATACTATAT
AGACATTAACACGTCTTACCTGCCTATGACTCTAGGATAATTTTAAAAAGTAACTTTTTGGGACTCTGTTTCATTTCCCT
GCCTCTATGTCAATTTCAAGCTGCACTGTTCATCAGATTCTTCATATAAAATGTAACAATTGATAATATTCTCACCCACA
AGATTATTGTCAATTTAACGCTTCCCTTAGAGTTTGAAAATCACAAAAATACTATTTTACGTAACTCTTCCACCATGACC
CTTCATCTACTTTCCTAAGTACGGGTCTCAAACTTGTAAAACACATACATCTTTTTTAGTTTTTTTGTATTTTACGCCCT
TTTTCTCCCCAATTTTCAATCGTGTGTCATCGCTGCAAATCCCCAATGGGCTCGGGAGAAGCAAAGGTGGAGTCATGCGT
CCTTGGAAACATGACTCACCTAACCGCGCTCCTTAACACCCGCAAGCTTAACCCGGAAGCCAGCCACACCAATGTGTCGG
AGGAAACACCTTTCAACTGGTGACCGGGGTCAGCCCGCAGGTACCCGGCCCGCCACAAGGAGTCGCTAGAGAATGATGAG
CCAAGTAAAATCCTCCCCTAACCCGGGTGACGCTGGGCTAATTGTGCGCCGTCCTATAGGACTCCCGGCCAGTTGTGGCA
CAGCTCGGAAATGAACCCGGGTCTGTAGTAACGCCTTAGAATTCTGCGCCACTCGGTAGACCCCATAATTACATGTTTTA
CAAATAAATTAGACCCGCTACCGGAATAGTGACATCCATGATGTTCTGGGTCAATTTGACCCACGCAATTTGACCTTGAC
CTCGGTCTATACAGTGCCTTCGGAAAGTATTCAGACCCCTTGCCTTTTACAAATGTTGTTAGGTTACAGGCTTATTCTAA
AATGTATTAAATTGTTTTTTTTCCTCATCAATCTACACCCAATACCTAATATTGACAAAGCGAAAACCTTTTTTTTATTT
TTTTTTTACATTTTAGCAAATGTCTTAAAAATAAAAAACATAACACATTCACATAAGAATTCAGAGCCTTTACTCAGTAC
TTTGTTTAAAACCTCTTCAGCGTCTGATCCCATTAGCGGGATCAAAATCCAGCGAAAAATCATATCGCCAATTAGCATTA
AAAAAATCATAATTTAAAAAAATATATATTAATATATATTTTTTTTTATAGATACACCTCTCCTGAATCGACCCACGTCG
TCCGATTTCAAAAAGGTTTTACAGGAAAAGCAAATCATTAGATTATGTTAGATGAGTCCATCGTAAAAAGCAGCAACATA
GCCATTTTCCGACCAACCACTTGCATCACAAATAACCAAAAAACAGCTAAATGCAGCACTAACCTTTTACAAACTTCATC
AGATGACACACCTAGGACATCATGTTACACAATGCATGCATTCTTTTGTTCAATAAAGGTCATATTTATATATAAAAACA
GCATTTTACATCGGCGCGTAACGTTGACTGACTATTTTCCCCTCAAATGCGTCCCGGGAAACAATGCTACAATTCCCTAA
ATTACTATTCGAAAACGATTTTTTAAATTTATATTGTCAATCTAACATTTATAGATGAATATCTCTTGAGAACACCTGTC
ATACCAGATTTTAAATTAACTTTACTGGGTAATCACACTTTGCGATAAAAGGAGATGCGATACACAGAAAATGAGCTAAT
AAACCGAGCTCGGCGCCATCTTGGAACAATCGCATGTCACATCTCATCTTGTATAATATTGTCAATAATCGCCTACCTTT
AGTTGTCTTCATCAGAAAGCACTTCCAGGAATCCCAGGTCCACAACAGATGTATTTTCGGTCGAAAAAGTACATCATTTA
TGTTCCATTAGCTTTCTGTTGTTAGCGCGTCTGAACGGCACAGCCGTTTCGAAATAAAACATTTTTTTCCCATTTAGGTT
CGTTCAAACATGTCAAACGTTGTAAAACATTAATCTTCAGGGCCTGTAACACCAAGAGAGCCAATAAGATTCGAGGGGGA

TGATTTCATTGTGTTTCAAACCATTTCCAAAGGTGGGGGTAGACATGGCCGCCGGCGTCATAATGGTGATGGCCCATCCC
CTGTGACCAATTTCCAACGTGTCTCATTCACTGAGTTTCGACTGTATAAGGCTCAAACCACTGTGAAAAGACTGGTGACA
TCTAGTGGAAGCAATAGGAAGTGCTCACTGAACCATTGTTAACGGTGTGATTAATAGGGAAAGATGAGAAGTCGAGTCCA
CAATTCTGAATTCCACTTCCTGTTTCAATCGGTCTCGGGGTTTTGACTGCCATAGGAGTTCTGTTATACTCACAGACACC
ATTCAAACAGTTTTAGAAACTTTAGGGTGTTTTCTATCCATATATAATAAGTATATGCATGTCCTAGCTTCTGAGTTTGA
TTAGTAGGCCGTTGAAAATGGGAACGAATTTTTTTCAAAATGCGCTGTGGCGCCCCCTATCCTAGGGTATCCTCAAGAGC
ACCTTNNNNNNNNNNNNNNNNNNNNNNNNNNNNNNNNNNNNNNNNNNNNNNNNNNNNNNNNNNNNNNNNNNNNNNNNNNN
NNNNNNNNNNNNNNNNNNNNNNNNNTGGTCTGAGAGTCTTTAGGTGTCTTTTGGCAAACTCCAAGCAGGCTGTCATGTGC
CTTTTACTGAAGAGTGGCTTCTGTCTTGCCACTCTACCATAAAGGCCTGATTTGTGGAGTGCTGCAGAGATGGTTGTCCT
TCTGGAAGGTTCTGCCATCTCCACAGAGGAACTCCAGAGCTCTGTCAGATTGACATTCAAGTTCTTGGTCTCCTCCCTGA
CCAAGGCTCTTCTCCCCCGATTGCTCAGTTTGGCCGGGCGGCCAGCTCTAGGAAGAGTCTTGGTGGTTCCAAACTTTTTC
AATTTAAGAATGATGGCGGTCACTGTATTCTTGCGGACCTTCAACGCTGCAGAAATGTTTTGGTACCCTTCCCCAGATCT
GTGCATCAACACAATTCTGTCTCAGAGCTCGACAGACAATTCCTTTGACCTCATGGCATGGTTTTTGCTCTGACATGCAC
TGTCAACTGTGGGACCTTATATAGACAGGTGTGTGCCTTTCCAAATCATGCCCAATCAATTGAATTTACCACTGTTGGAC
TCCAATCAAGTTGTAGAAACATCTCAAGGATGATCAATGGAAACAGGATGCACCTGAGCTCAATTTTGAGTCTGATAGCA
AAGGGTCTGAATACTTATGTATTTGCTGAAATGTGTGGCGATGCCCAGCTATTTAAAGGGACAGGTGGCTATTTGAGACT
GAGTTTAATTGAAGTTGAAGAAAGTTGCAGTAAATCCCTGACCCTAGTCTCAGTGCAGAGAGATATTTTGCAGAGGGAAG
TATTTTAAGACAACAGCCAACCAATGTATGAAAGCTGACTTACACACTCAGGGCTGAGGAAGGGAACCCAGCCTACACAA
GAGATCCATGGACAACTGCCAATCAAACCAGGAGCTGTGCTGCTACTGCTGTTATTATTGTTATTGCTCTATTTCATCCT
CAGGATATATCACAGGATATGTGAGCACTTACATAATGTCGACATTTGCATAGCTGCTCCGCATAAAAAGCTATTTTCTA
ATTTAGGCATGGTATTTTCAATGGTGCTTCACTCCATTTCTTAACATGTCATTTCTGGTACCTTGTAATTGATAAATTGA
TCAGTAATCATTTATTAAAGGGGGATTTTTTTTGTCAAAATTTCAATTACAAAGAGGATTCTAAAAGACCCGTCTTGAAA
AAATGTATTGTAGCTAAGATGTTGATTATAAAAACGTTATCTTTAAATCACATTTGCCTAATTTTTCAATAATATACAGC
CCCAAGGAATACATATTTACTGTGCAGTAGCACTCTCTACTCTGCTGGTTGTTACGCTTATTAAAGAGAAGGTTGCACAA
TATATTTCCACAAATCTAACAATGTAGATTGATATTCCATCTCATACAAACGTTTATAAAAAGAAAATGTATACACTTAT
ACACACATTTTCGCAATTTGAATTACATTTATCAGAAACTCCTATACAAACAAAACATTACGGCTTTGACGTCAAGAAGG
GAACTGTTGGTGGGGGCAGTACACACTGATCTTTTTTGCAGGATATCATGGTAGTCAATGTTTGTTTTAGCCATTATACC
TGGGTTTACTGCTGCTAGTAGTAGCACTCAGGTTAGCATGTCATTAGCTATAGCTACAATCGGCCAACAACAGAGTCTTA
GCCAGTTCGGTTATAGATGTTCAATGTCTATTATGATGGTAAAGGTTTATCTTGATTTTGTGTAGCAGATTTTGTGTAGC
AGATTTTGTGTAGCAGGTTTTGTGTATCAGCGAGAAATGCTGATGTGCTGAACTGCTAATAAGTGACGTGTTTCAAGACT
GCTAGTGGTTAGCTAGTAGGGGTAAGCCAGGGGTGGAAAGGGGGCTATGCAAAAGGCCAGAGAGCTGTATTAGTGTTTAT
TTTTTTAATGAGCGGATACAGAGTAAAAAAAAATCACAGGATATGATCAGATGATAAATTGGCTTTATTCAGAGTGCCAAA
ATATATTTTTCTAACAATAGTAGGACTCTGTATGATGTGGGTATTCACCATCTTATGTAGGCAGTGGTTTCACTCCTACT
CCTGTAGTGCTCCTCTTCATGTTACATTTCCGAACGCTTTTCCGACCTGACTTCGGTCAAATTCGATTAAAATATAAATT
TCTCAGAAAATACAAATGTAAAGTGATAGGGTATAAAACAAATCAGAATGTTTTAAAATAAATTGAATTCTACAATCTTC
CCTTTAAGTAAAATATAACTTGACTACTTGTTCCACATGAAGTAGCTTTACAGTGAGGGAAAAAAGTATTTGATCCCCTG
CTGATTTTGTACATTTGCCCACGGACAAAGAAAGGATCAGTCTATAATTTTAATGGTAGATTTATTTCAACAGTGAGAGA
CAGAATAACAACAAAAAATTCCAGAAAAATGCATGTCAAAAATATTATAAATTGTTTTGCATTTTAATGAGGGAAATAAG
TATTTGACCCCGTCTCAATCAGAAAGATTTCTGGCTCCCAGGTGTCTTTTATACAGGTAACGAGCTGAGATTAGGAGCAC
ACTCGTAAAGGGAGTGCTCCTATCTCAGCTTGTTATCTGTATAAAAGACACCTGTCCACAGAAGCAATCAATCAGATTCC
AAACTCTCCACCATGGCCAAGACCAAAGAGCTCTCCAAGGATGTCAGGGACAAGATTGTAGACCTACACAAGGCTGGAAT
GGGCTACAAGACCATCGCCAAGCAGCTAGGTGAGAAGGTGACAACAGTTGGTGCGATTATTCGCAAATGGAAGAAACACA
AAAGAACTGTCAATCTCCCTTGGCCTGGGGCTCCATGCAAGATCTCACCTCGTGGAGTTGCAATGATCATGAGAACAGTG
AGGAATCAGCCCAGAACTACAAGGGAGGATCTTGTCAATGATCTCAAGGCAGCTGGGACCATAGTCACCAAGAAAACAAT
TGGTGACACACTACGCCGTGAAACGCTGAAATCCTGCAGCGCCCGCAAGGTCCCCCTGCTCAAGAAAGCACATATACATG
CCCGTCTGAAGTTTGCCAATGAACATCTGAATGATTCAGAGGACAACTGGGTGAAAGTGTTGTGGTCAGATGAGACCAAA
ATGGAGCTCTTTGGCGTCAACTCAACTCGCTGTGTTTGGAGGAGGAGGAATGCTGCCTATGACCCCAAGAACACCATCCC
CACCGTCAAACATGGAGGTGGAAACATTATGCTTTGGGGGTGTTTTTCTGCTAAGGGGACAGGACAACTTCTCCGCATCA
AAGGGTGGATGGACGGGGCCATGTACCGTCAAATCTTGGGTGAGAACCTCCTTCCCTCAGCCAGGGCATTGAAAATGGGT
CGTGGATGGGTATTCCAACATGACAATGACCAAAAACACATGGCCAAGGCAACAAAGGAGTGGCTCAAGAAGAAGCACAT
TAAGGTCCTGGAGTGGCCTAGCCAGTCTCCAGACCTTAATCCCATAGAAAATCTGTGGAGGGAGCTGAAGATTCGAGTTG
CCAAACGTCAGCCTCGAAACCTTAATGACTCGGAGAAGATCTGCAAAGAGGAGTGGGACAAAATCCCTCCTGAGATGTGT
GTAAACATGGTGGCCAACTACAAGAAACGTCTGACCTCTGTGATTGCCAACAAGGGTTTTGCCACCAAGTACTAAGTCAT
GTTTTGCAGAGGGGTCAAATACTTATTTCCCTCATGAAAATGCAAATCAATTTATAAAATTTTTGACATGCGTTTTTCTG
GATTTTTTTGTTGTTATTCTGTCTCTCACTGTTCAAATAAACCTACCATTAAAATTATAGACTGATCATTTCTTTGTCAG
TGGGCAAACGTACAAAATCAGAAGGGGATCAAATACTTTTTTCCCTCAGTGTACGTCATTTCGAAGAAGTGTGGGAACAG
TGATGTAAACTGTTGCCAGAAAACAGTATTGATTCTAGCTTAGCGGGCATACAATTCCATGAACCATCTCTCTCTGCCCC
TTTCCCCAGTGAATGTTCCCCTGCTCATGTCCATGAACCGTTAAACAAAGCTACAGTAGCGCTCCATTTGTATTCATTCG
TTGTTCCAACATTAAGACAGCAAGATGACTCAGCAAATCTCTCCAAAGACAAGCGGCAGCTTAGGGGTGGATTTGCAGTT
CCCTCAGTGACAGACTCACAGGCTCCCTGTCATCCTGCACTGTTAATTTTATATAGTCTCACTAAAACAAACAATACTGT
TGTTGATACTGCTACAGTACTACGTATGCTTCATGCTGCAATTAAAACATATTGAAGTGTCCTTTAGTATTCCCATGTGG
TAGCAAACAGAGAGTAGCATAATTGGGGTTGGCTAATGGGTTCGTATTGCGTAATGTTGCAGTAGATTTTTGGTGTCAAC
GGAGGCTGAATTGCTCAAAACAAAGATAAATCTGAATAACAGACTCACTAATCTAATATATAATGCTAGAGAATACAAAC
TACGCACATGCATGGTAACTGTACTGAGGTTGTTGATGGGTTGGCAGGTTAGCGGTAGCTGCTGGAGTATGCTGCAAACA
TGAACCACTTCTGAGTTCCACTCTTACCCTGACAGTTGTTTTGTCCCTGGTGGAACCTCAAAGACATTGCATGTTGTTTT
AATGAGTTAAAAGGCTGGGGAAAATCCATAATTTATACTATTTATTATGATTCATTTTCTGAAAGTTATTGGAAGAGAAA
TTAGTTCAGTTATACACGCTAGTGTTGTACCTTTAAGATGGGATGTGGAACAACTCCTATAAATAAAATATCAAAATGAC
AATGTTGTCCCAGTAGACGGAGAACAAAAGTTTATAATGGAGGAAAATACGAACAACACGTTGTGTTTTTACATTGAATT
TCCATTCAGCCAAAGGCTATCAATCAGATAAGCAGTTAGCTAGCTACATTATGTTTGTGATTTAGCCTGAGGTTAATTCA

TTCAGATAACATGGATTTCTCTCAATGACTGAGCAGCCGTGGTGAAAGTCAATTTCAAAATGTAACACATAACATCAAAG
GCCAACTTCATTAATGCAGATAATCACATCCAATTTTGAGGAGGTTAATAAGAGAGAGCGAAAACAATATATTTTCCTTC
TATTAGCACAGAATGTACTCTTTGAATGAACGTTATTGCAAGAGACCCAATAAGACCAGGAGCATGCACTTCAATGCCAT
TAAAGATTTAAAAGGATTGTTTCTCTCATTATATTACATAATCAAGTCCCTTTGCTGACTTTGCTCTATCAAATCAAACT
TTATTTGTCACACGCGCCGAATACAACAAGTGTAGACCTTACAATGAAATGCTTACTTACAAGCCCTTAACCAACAGTGC
AGTTCAAGAAGACTTAAGAAAATATTTACCAAATAAACTAAAGTAAAAAATAATAAGTAACACAATAAAATAACAACAAT
GAGGCTATATACAGGGGGTACCGGTACCGAGTAAGTGTGCGGGGGTAGAGGTTAGTTGAGGTAATTTGTACATGTAGGTA
GGGATGAAGTGACTATGCATAGATAATAAACAGCGAGTAGCAGCAGTGTACAAAACAAGTGGAGAGGGCGGGGTCAATGT
AAATTGTCCGGTGGCCATTTGATGAATTGTTCAGCAGTCTTATGGTTTGGGGTAGAAGCTGTTGAGGAGCATTTTGGCCC
TAGACTTGGTGCCTCGGTACCGCTTGCCGTGTGGTAGCAGAGAAAACAGTCTATGACTTGGGTGACTGGAGTCTCTGATA
TTTTTATGGGCCTTCCTCTGACACCGCCTATTATATGGGTCCTGGATGGCAGGAAGCTTGGCCCCAGTGATGTACTGGGC
TGTACGCACTACCCTCTGTAGCGCTTACGGTCAGATGCCGAGCAGTTGCCATACCAGGCAGTGATGCAACCGGTCAGGAT
GCTCTCGATAGTGCAGCTGTATACATTTTTGAGGATCTGGGGACCCATGCCAAATTTTTTCAGTCTCCTGAGGGGGAAAA
GGTGTTGTCGTGCCCTCTTCACGACTGTCTTGGTGTGTTTGGAACATGATAGTTTGTTGGTGATGTGGACACCAAGGAAC
TTGAAACTCTCGACCCGCTCCACTACAGCCCCGTCGATATTAATGGGGGCCTGTTCGGCCCGCCTTTTCCTGTAGTCCAC
GATCAGCTCCTTTGTCTTGCTCACATTGAGGGAGAGGTTGTTGTCCTGTCACCACACTGCCAGTTCTCTGACCCGCTCCC
TCATTGTCGTGATCATGCCTACCACTGTTGTTTCATCAGCAAACTTAATGATGGTGTCGTGGTTGGCCACGCAGTCGTGG
GTGAACAGGGAGTACAGGAGGGGACTAAGTGCACACCCCTGAGGGGCCCCAGTGTTGAGGATCAGCGTGGCAGACGTGTT
GTTGCCTACCCTTACCACCTGGGGGCGGCCCGTCAGGAAGTCCAGGATCTAGTTTCAGAGGGAGGTGTTTAGTCCCAGGG
TCCTTAGCTTAGTGATGACCTTTGTGGGCACTATGGTGTTGAACGCTGAGCTGTAGTCAATGAACAGCATTCTCACATAG
GTGTTCCTTTTGTCCAGGTGGGAAAGGGCAGTGTGGAGTGCGATTGAGATTGCGTCATCTGTGGATCTGTTGGGGCGGTA
TGCGAATTGGAGTGGGTCTAGGGCATCTGGGAGGATGCTGTTGATGTGGGCCATGACCAGCCTTTCAAAGCACTTCATGG
CTACCAATGTGAGTGCTGCAGGGCGGTAATCATTTAGGCAGGTTACCATCGCTTCCTTGGGCACAGGGACTATGGTGGCC
TGCTTGAAACAGCCTGAGTTTCACTTGGTTAACGTCTGATATGGTAAACAGGGGTTTTGTGGTAGGTCAGACCTGCTCTT
TGAGCTGTTCGAAATGACAAGTTAACACAAATGTAATTTACCCTGCAAGTAAATACAGAAACATGCAACAGTACAGAGAC
AACCCCAGCTACACAGTTTCAATAACTTCCCTTTATGGCTGAATGGCTCAGAAACGTGACATCTAACATTTTGCAGCAGT
ACACAACATACTAGAGGTCGACCGATTAATCGGAATGGCCGAATAATTAGGGCCGATTTCAAGTTTTCATAACAATCGGT
AATCGGTATTTTTGGCCACCAATTTGCCGATTATTATATATATTTTTTGTTTGTTATTTACATATTTTTTTATTTTTTTA
TTTTTTACATCTTTATTTAACTAGGCAAGTCAGATTAAGAACACATTCTTATTTTCAATGACGGCCTAGGAACGGGGGTT
AACTGCCTTGTTCAGGGGGGATTCGGGGATTCGTTTTTGCAACCTTCTGGTTACTAGTCCAACGCTCTAACCACCTGCCT
TACATTGCACTCCACGAGGAGCCTGCATGGCAGGCTGACGACCTGTTACGCGAGGGCAGCAAGAAGCCAAGGTAAGTTGC
TAGCTAGCATTAAACTTATCTTATAAAAAACGATCAATCTTAACATAATCACTAGTTAACCACACATGGTTGATGATATT
ACTAGTTTATCTAACGTGTCCTGCGTTGCATATAATCGATGGGGTGCCTGTTCATTTCTCATCGAATCACAGCCTACTTC
GACAAACAGGTGATGATTTAACAAGGGCATTTGCGAAAAAAGCACTGTCGTTGCACCAATGTACCTAACCATAAACATCA
ATGCCTTTCTTTAAAATCAATACACAAATATATATTTTTAAACCTGCATATTTAGTTAATATTGCCTGCTAACATGAAAT
TGTGTCACTGCTCTTGCGTTCATTGCCTGGCTCATTGCGAACTAATTTGCCAGAATTGTACGTAATTATGACATACCATT
GAAGGTTGTGCAATGTAACAGGAATATTTAGACTTAGGGATGCCACCCATTAGATAAAATACGGACCGTTTCCATATTTC
ACTGAAAGAAAAAAACGTTTTGTTTTCGAGATGATAGTTTCCGGATTTTACCATATTAATGACCTAAGGCTTGTATTTCT
GTGTGTTTATTATATTATAATTAAGTCTATGATTTGATAGAGCAGTCTGACTGAGCGGTGGTAGTCACCAGCAGGCTCGT
AAGCATTCATTCAAACAGCACTTTTGTGCGTTTTGCCAGCAGCTCTTCGCAATGCATTGCGCTGTTTATGACTTCAAGCT
TGTCAACTCCCAACATTAGGCTGGTGTAACCGATGTGAAATGGCTAGCTAGTTAGCGGGTGCGCGCTAATAGCGTTTCAA
ACGTCACTCACTCTGAGACTTGGAGTAGTTGTTCCCCTTCCTCTACATGGGTAAAGCTGCTTCAAGGGTGGCTGTTGTCG
ATGTGTTCCTGGTTCGAGCCCAGGTAGGAGCGAGGAGAGGGACGGAAGCTATACTGTTACACTGGCAATACTAAAGTGCC
TATAAGAACATCCAATAGTCAAAGGTATATGAAATACAAATGGTATAGAGAGAAATAGTCCTATAATTCCTATAATAACT
ACAACCTAAAACTTCTTACCTGGGAATATTGAAGACTCATGTTAAAAGGAACCACCAGCTTTCATATGTTCCCATGTTCT
GAGCAAGGCACTTAAACGTTAGCTTTCTTACATGGCACATATTGCACTTTTACTTTCTTCTCCAACACTTTGTTTTTGCA
TTATTTAAACCAAATTGAACATGTTTCATTATTTGAGGCTAAATTGATTTTATTGATGTATTATATTAAGTTAAAATAAG
TGTTCATTCAGTATTGTTGTAATTGTCATTATTACAAAAAAAAAATAAAAAAAAATGGCATCGGCTTTTTTTGGTCCTCCA
ATAATCGGTATCGGCGTTGAAAAATCATAATCAATCGACCTCTACAACGTACAACCACTTTTTACTCTATAGTGCTTATA
GTTTCCATTATGCATGCTACTGCAATTGCAACCATTTAACATAGTATAAATACAGTACCTCCACCATAATGCAGCAACTG
CTATTCAAGACAGATTCCTTATACTCCACTATTGGCACTACCACCCCACACTGTCTGTCTCCCTATTCATTTTTTTCCCT
TCATTTTTTTTTTTTTTTTACCCATTTCTTCCCCCCAATTGGTAGTTACGATCTTGTCTCATCACTGCTACTCCCCAGCGG
GCTCGGGACAGGCGAAGGTCAAGACATGCATCCTTTGAAACATGACCCGCCAAGCCACGCTTCTTGAACCCCAGGCTGTA
GTGGCGCCTCAGCGCTGCAATACAGTGCCTTAGATTGCTGCACCACTCGAGAGGCCTTTGTCTCCCTATTGTAACTACTG
TACCTTATATGTACCTTAAATATGTCCAGGGTACATAGGGTGTTTCAACACCATGGACAGAGGGCCCAACATGGGCTAAA
ATCCACCAAAGATGAGAGCTGTTTCAGTAACCTTGGACAGCTCCGCACAGAGCAAAGATTTTGGGTTGGTCTGTGTGGCT
GCATGCAGAACTTCTGTGGATAGGGTTTATGTTGAAGTTCTAATTACAATAGATATTTGCATAAAACATTTACAGCACAC
TGCAATTATCTACCAAGATGAATGCCTTAATTACATGTTGATTGCTCCAACTTTAACCATTTCCGTGCTTTCAAGGTGCA
ATACAAGGAAGTGGATGGGGAAGTAGACTAATTGTGTCTGAGTAACTTTCAGTATCCAATCCAAAGAGCGTACTGAAGTA
TCGTATGTGTCCCTAATCATAGATGCAGAGCCAAATGTCTTCATCAGAGATGGTCATTACATCAGGACCCAGCTGGGATT
GTTGTCAAGTGTCTTCATTTATCAAAACGATGATAAATGAAAATTCACGTTTGAAGTATAGATACAGTACATAAACACAT
TGGATGCATACCAAGATGGATACAAATTCAAGCTTGTTTTTTTTATAACACAAGAGTAATCTATGACTTTTACTGTATGT
GTTCCTTGAATGAAGCACTTAAGTGAGTAGAGGACAACATCGGAAGGTCTATTTGACCTCATCCCCCATAGAGCATTGTT
TGCTATCTCGATCGGACAGTTCAGCCTGTCATTTTGATTCCATGCCTTTGTGTCCTTTTTTTCTTTTTTGAAGTCTTGTC
AAAATTGGATTTTCAAAGATTTTTTTATACCGGTCAAACGTTTTAGAACACCTACTCATTCAAGGGTTTTTCTTTATTTT
GTACTACTTTCTACATTGTAGAATAATAGTGAAGACATCAAAACGATGAAATAACACATATGGAATCATGTAGTAACCAA
AAAAGTGTTAAACAAATCTAAATATATTTTACATTTGAGATTCTTCAAATAGCCACCCTTTGCCTTGATGACAGCTTTGC
ACACTCTTGGCATTCTCTCAACCAGCTTCATTAGTTAGTCACCTGGAATGCATTTCAATTTGGTAAAAGACCAGTCCCTA
TTATGGGAAGAACAGCTCAAATAAGGAAAGAGAAATGACAGCCCATCATTATTTTAAGACATGAATGTCAGTCAATGCTG

AAAATTTCAATGACATCTTCAAGTGAAGTCACAAAAAACATCAAACACTATGATAAAACTGGCTCTTACGAGCCTCAGAA
ATTGCAGCCCAAATAAATGCTTCACAGAGTTCAAGTAACAGACACATCTCAACATCAACTGTTCAGAGGAGACTGTGTGA
ATCAGACCTTCATGGTCGAATTGCTGCAAAGAAACCACTACTAAAGGATACCAATAATAAGAAGAGACTTGCTTGGGCCA
AGAAACACATTAGACCGGTGGAAATGTGTCCTTTGGTCTGGAGTCCAAATTTGAGATTTTTGGTTCCAACCGCCGTGTCT
TTGTGAGACGCTGTATGGGTGAACGGATGATCTCCGCATGTGTATTTCCCACCGTAAAGCATGGAGGAGGAGGTGTTATG
GTGTGGAGGTGCTTTGCTGGGTTCACAGTCTATGATTTATTTAGAATTCAAGGCACACTTAACCAGCATGGCTATCACAG
CATTCTGCAGCGATATACCATCCCATCTGGTTTGGGCTTAGTGGGACTATCATTTGTTTTTCAACAGGACTATGACCCAA
CACGCCTCCAGGCTGTGTAAAGGCTATTTGACAAAGAAGTAGAGTGATGGAGTGCTGCATCAGATGACCTGGTCTCCACA
ATCTCCCGACTTCAACCAAATTGATATGGTTTGGGACGAGTCGGACCGCAGTGTGAAGGAAAAGCAGCCAACAAGTGCTC
ATGCACTTGTTGCACAAAAATAATATTTTTGCAGGAATCTCCTTGCGAATGATTTTGCCGAAGATTGTATCTCCGCCGGC
CTGGGCACCTGAGCTTTTGCTATCTATGCTATGTGGGAACTCCAAGTGAAGCTGGTTGAGAGAATGCCAAGAGTGTGCAA
AGCTGTCATCAAGGCAAAAGGTGGCTATTTGAAGAATCTCAAATATAAAATATATTTTGATTTGTTAAACACTTATTTGG
TTACTATATGATTCCATATGTGTTATTTCATAGTTCTGATGTTTTCACCATTATTCTACAATGTAGAAAATAGTAAAAAT
AGGTGGTGTTCTAAAACTTTTGACTGGTAGTGTAGATTATCCTTATCCCATCCAATCCACGTGTATGCAATCTACTCTCA
CTTTAGGATTATCCCAATGATGATCTCACTAAGTTATCTCAGTTCCAAGTTATGCAGTAGGTCACAGTCTTTCTGAAATG
GGTCACTGTGTCGCAAGCATTTCCCTTGACACTGACTGGGGGGCCTTGGCCGCATGGGGGGCATCGATCCCATTCAGTAT
GGGCATCTCCTCAGTGTGGATGGGCTCTCACTGCAGATGGTTCTCAATGGAAGGCTGTTTGATAACATGCTATATCATAG
AGATGCTTAGAGTGTGATGAGCATCACTCTTCTGGAGTCAGTAGGCTTGTGTAGCCTGTGGCGTTATAGGAGAGTATTGC
TGTGTCAGACCTGAACAGGTTCATGTCAAATATGGAGTGAGTGGCCTATTGCAGTATCCCCACAAGACTGTTGTGGGGAG
ATTCGCCCTATGTGGACAATGGGAGTGTATCAATGCTGAGACATACATGAGGCAGAGAGGCAGAGTGAGAGAGGTGGAGT
AAGGCAAAGAGGCAGGCAGAGACAATGAGTCAGAGAGGCAGTAAGAAAGACAATCACAAAATATCAGAAGTAGAAAGGCA
GAGAAAGGTGAGAGCAGCCCATGAAAGGTAGAGTCAGAAACACAGAACTAGAATGAGTAGAACGGCCCTCCCATTCGAGT
CAATGACGCCATAATGGGTGGACTGGCAGCCATTGCGAGGGTCCCCATAAGAGTAAAGCGGGAAGTAAAAGCAGGAAGTA
CAACATTACATTTGTGCTTTATGGTCAGAGGTTCCAAGCTATTGACTGTATTTCTATGGTCAGAAAGAATTCAGAGAGAT
AGAAGGGCCCATGGCCAACTTACCCATCTCTGGATGGTGCCTCCCCTCAACAGGAACGCTGACTGTCCTCCGCAGCAGCT
TGTTCCTGTGCAACTCGGAGCGCCGCTCGCGCATCAACAAAGGGTGTACCTGCATATGCACAGAGAGACAAGGTCAGGTC
AACACACACGCACAATGCATTAAGCTATATTTTCAGAAGACCATAATGGATAGTATCTTGAAATGTCTATCACTGAGCAG
NNNNNNNNNNNNNNNNNNNNNNNNNNNNNNNNNNNNNNNNNNNNNNNNNNNNNNNNNNNNNNNNNNNNNNNNNNNNNNNN
NNNNNNNNNNNNNNNNNNNNNNNNNNNNNNNNNNNNNNNNNNNNNNNNNNNNNNNNNNNNNNNNNNNNNNNNNNNNNNNN
NNNNNNNNNNNNNNNNNNNNNNNNNNNNNNNNNNNNNNNNNNNNNNNNNNNNNNNNNNNNNNNNNNNNNNNNNNNNNNNN
NNNNNNNNNNNNNNNNNNNNNNNNNNNNNNNNNNNNNNNNNNNNNNNNNNNNNNNNNNNNNNNNNNNNNNNNNNNNNNNN
NNNNNNNNNNNNNNNNNNNNNNNNNNNNNNNNNNNNNNNNNNNNNNNNNNNNNNNNNNNNNNNNNNNNNNNNNNNNNNNN
NNNNNNNNNNNNNNNNNNNNNNNNNNNNNNNNNNNNNNNNNNNNNNNNNNNNNNNNNNNNNNNNNNNNNNNNNNNNNNNN
NNNNNNNNNNNNNNNNNNNNNNNNNNNNNNNNNNNNNNNNNNNNNNNNNNNNNNNNNNNNNNNNNNNNNNNNNNNNNNNN
NNNNNNNNNNNNNNNNNNNNNNNNNNNNNNNNNNNNNNNNNNNNNNNNNNNNNNNNNNNNNNNNNNNNNNNNNNNNNNNN
NNNNNNNNNNNNNNNNNNNNNNNNNNNNNNNNNNNNNNNNNNNNNNNNNNNTTTTGGTTCCAACCGCCGTGTCTTTGTGAGACG
CTGTATGGGTGAACGGATGATCTCCGCATGTGTATTTCCCACCGTAAAGCATGGAGGAGGAGGTGTTATGGTGTGGAGGT
GCTTTGCTGGGTTCACAGTCTATGATTTATTTAGAATTCAAGGCACACTTAACCAGCATGGCTATCACAGCATTCTGCAG
CGATATACCATCCCATCTGGTTTGGGCTTAGTGGGACTATCATTTGTTTTTCAACAGGACTATGACCCAACACGCCTCCA
GGCTGTGTAAAGGCTATTTGACAAAGAAGTAGAGTGATGGAGTGCTGCATCAGATGACCTGGTCTCCACAATCTCCCGAC
TTCAACCAAATTGATATGGTTTGGGACGAGTCGGACCGCAGTGTGAAGGAAAAGCAGCCAACAAGTGCTCATGCACTTGT
TGCACAAAAATAATATTTTTGCAGGAATCTCCTTGCGAATGATTTTGCCGAAGATTGTATCTCCGCCGGCCTGGGCACCT
GAGCTTTTGCTATCTATGCTATGTGGGAACTCCAAGTGAAGCTGGTTGAGAGAATGCCAAGAGTGTGCAAAGCTGTCATC
AAGGCAAAAGGTGGCTATTTGAAGAATCTCAAATATAAAATATATTTTGATTTGTTAAACACTTATTTGGTTACTATATG
ATTCCATATGTGTTATTTCATAGTTCTGATGTTTTCACCATTATTCTACAATGTAGAAAATAGTAAAAATAGGTGGTGTT
CTAAAACTTTTGACTGGTAGTGTAGATTATCCTTATCCCATCCAATCCACGTGTATGCAATCTACTCTCACTTTAGGATT
ATCCCAATGATGATCTCACTAAGTTATCTCAGTTCCAAGTTATGCAGTAGGTCACAGTCTTTCTGAAATGGGTCACTGTG
TCGCAAGCATTTCCCTTGACACTGACTGGGGGGCCTTGGCCGCATGGGGGGCATCGATCCCATTCAGTATGGGCATCTCC
TCAGTGTGGATGGGCTCTCACTGCAGATGGTTCTCAATGGAAGGCTGTTTGATAACATGCTATATCATAGAGATGCTTAG
AGTGTGATGAGCATCACTCTTCTGGAGTCAGTAGGCTTGTGTAGCCTGTGGCGTTATAGGAGAGTATTGCTGTGTCAGAC
CTGAACAGGTTCATGTCAAATATGGAGTGAGTGGCCTATTGCAGTATCCCCACAAGACTGTTGTGGGGAGATTCGCCCTA
TGTGGACAATGGGAGTGTATCAATGCTGAGACATACATGAGGCAGAGAGGCAGAGTGAGAGAGGTGGAGTAAGGCAAAGA
GGCAGGCAGAGACAATGAGTCAGAGAGGCAGTAAGAAAGACAATCACAAAATATCAGAAGTAGAAAGGCAGAGAAAGGTG
AGAGCAGCCCATGAAAGGTAGAGTCAGAAACACAGAACTAGAATGAGTAGAACGGCCCTCCCATTCGAGTCAATGACGCC
ATAATGGGTGGACTGGCAGCCATTGCGAGGGTCCCCATAAGAGTAAAGCGGGAAGTAAAAGCAGGAAGTACAACATTACA
TTTGTGCTTTATGGTCAGAGGTTCCAAGCTATTGACTGTATTTCTATGGTCAGAAAGAATTCAGAGAGATAGAAGGGCCC
ATGGCCAACTTACCCATCTCTGGATGGTGCCTCCCCTCAACAGGAACGCTGACTGTCCTCCGCAGCAGCTTGTTCCTGTG
CAACTCGGAGCGCCGCTCGCGCATCAACAAAGGGTGTACCTGCATATGCACAGAGAGACAAGGTCAGGTCAACACACACG
CACAATGCATTAAGCTATATTTTCAGAAGACCATAATGGATAGTATCTTGAAATGTCTATCACTGAGCAGTAGCGGAGCG
TGGGTAACATTTCTGGAGAAGCCAAGCCAGGAAAAAAAAATCCTTATTACAACCTAGATATTGTGATAATTACCTAGCTTG
CTCTATAACCTGTTAGTTCATATGCCTTGACACTGTGATTTATAGGCCTAAGGCTGAAACAATAAGAAGACACAGTGGCA
GAATAAATTCAACCACAACTTTGTTTCATTAAAAAAACAGAGAGCAACATCTGTCCGGTGAAGTCCACAAAACATATTGC
ATGTAACAAACAGTTACATGACCTACAGCATGGTCAAGCAAGTTCATGTTTCTGACATTTTCGGACCACTAAACAACTAT
TGATTTAGAACCACGGAGAGTTACCTCAAGTTGCAAAGAAAACAGGAGCTGCCTCCATTATTCCAGCACCATTTTAACTT
CTACATTTCAACAGAATCAAATCACCTATGCTAATGGTTAATTTAATACAGTGACAACTAAAAGATACCAAAAACAATTA
AGTCCAGTCAACATAAGCTAAATATGATGTGGCTATCCATGGTACTGATTTGTGTGTGTGTGTGTGTGTGTGTGTGTGTG
TGTGTGTGTGTGTGTGTGTGTGTGTGTGTGTGTGTGTGTGTGTGTGTGCTGTACGTCATGTTTGTTCATTAATAGAGTTGTT
TGTTGGGGTGAATAACAAGGCTGTTTGGGGTTGTTTCTGGTTTCTTGTATTTTGGGGTAGCTGTTTCTGGTAGTTTCCTG
TATTTCGCCGTAGCTGTTTCTGGTAGTTTCTTGTATTTTGGGGAAGCTGTCCAGTATTTCTCTCCGCTCATTGCATTAAT

ATTTTTTTCATTGTGATTTATCTGGGGGTGAACCCCTACTTCCTGCAGCTATGGTTTCTGGTATTTAAGGGTTTATTCTG
GTATTTTGGGGTTGCTGTTTGGGGTTGTATCTGACATTTTGCGCGTAGCTGTTTGGGGTTGTTTCTGGTATTTTCAGGTA
GCTATTTGGGGTAGTTTCACAGGTGCGCTGACAGACATGGCAGCTCTGCTTCTAGCTCCTAAGCAACTTTGCAGTATTTT
GTTTTTTGTATGTTATTTCTTACATTATTAGCCCATAACGTTTTTTTTTTGTGTTATTGCATACAGCCGGAAATAACTTT
TAGATAGCAGCGCGGCGCGGCGCGGCGGTAATCCTTTGTTTGTATCCCCCANNNNNNNNNNNNNNNNNNNNNNNNNNNNN
NNNNNNNNNNNNNNNNNNNNNNNNNNNNNNNNNNNNNNNNNNNNNNNNNNNNNNNNNNNNNNNNNNNNNNNNNNNNNNNN
NNNNNNNNNNNNNNNNNNNNNNNNNNNNNNNNNNNNNNNNNNNNNNNNNNNNNNNNNNNNNNNNNNNNNNNNNNNNNNNN
NNNNNNNNNNNNNNNNNNNNNNNNNNNNNNNNNNNNNNNNNNNNNNNNNNNNNNNNNNNNNNNNNNNNNNNNNNNNNNNN
NNNNNNNNNNNNNNNNNNNNNNNNNNNNNNNNNNNNNNNNNNNNNNNNNNNNNNNNNNNNNNNNNNNNNNNNNNNNNNNN
NNNNNNNNNNNNNNNNNNNNNNNNNNNNNNNNNNNNNNNNNNNNNNNNNNNNNNNNNNNNNNNNNNNNNNNNNNNNNNNN
NNNNNNNNNNNNNNNNNNNNNNNNNNNNNNNNNNNNNNNNNNNNNNNNNNNNNNNNNNNNNNNNNNNNNNNNNNNNNNNN
NNNNNNNNNNNNNNNNNNNNNNNNNNNNNNNNNNNNNNNNNNNNNNNNNNNNNNNNNNNNNNNNNNNNNNNNNNNNNNNN
NNNNNNNNNNNNNNNNNNNNNNNNNNNNNNNNNNNNNNNNNNNNNNNNNNNNNNNNNNNNNNNNNNNNNNNNNNNNNNNN
NNNNNNNNNNNNNNNNNNNNNNNNNNNNNNNNNNNNNNNNNNNNNNNNNNNNNNNNNNNNNNNNNNNNNNNNNNNNNNNN
NNNNNNNNNNNNNNNNNNNNNNNNNNNNNNNNNNNNNNNNNNNNNNNNNNNNNNNNNNNNNNNNNNNNNNNNNNNNNNNN
NNNNNNNNNNNNNNNNNNNNNNNNNNNNNNNNNNNNNNNNNNNNNNNNNNNNNNNNNNNNNNNNNNNNNNNNNNNNNNNN
NNNNNNNNNNNNNNNNNNNNNNNNNNNNNNNNNNNNNNNNNNNNNNNNNNNNNNNNNNNNNNNNNNNNNNNNNNNNNNNN
NNNNNNNNNNNNNNNNNNNNNNNNNNNNNNNNNNNNNNNNNNNNNNNNNNNNNNNNNNNNNNNNNNNNNNNNNNNNNNNN
NNNNNNNNNNNNNNNNNNNNNNNNNNNNNNNNNNNNNNNNNNNNNNNNNNNNNNNNNNNNNNNNNNNNNNNNNNNNNNNN
NNNNNNNNNNNNNNNNNNNNNNNNNNNNNNNNNNNNNNNNNNNNNNNNNNNNNNNNNNNNNNGTGTATAGGAGATGTTGT
TCCCATTGTGACTAATAAAACTGAAAGCGTGAAACCACCGCATCTAACCACTACAAGGTGACTGGGAATATGGTCGAATA
CAAACAGTGTAGTTATTCCCTCCGTAAGGCAATCAACAGGCAAACTGTCAGCACAGTCGCAATTCAATGGCTCAGACACG
AGAAGTATGTGGCAGGCTCTACAGACAATCACAGATTGCAAAGGGAACACCATCCACATCACGGACACAAACGTATTGCT
CCCGCACAAGCTAAACACCTTTTTCGCATGCTTTGAGGATAACACAGTGCCACCGACGTGGCCAGCTCCCAAGGACTGTG
GGCTCTCATTCTCCGTGGCCGGCGTGAGTAAGACATTTAAGCGTGTTAACCCTTGCAAGGCTACCGGCCCAGACGGCATC
ACTATCTGCGTCCCCAGAGCATGTGCAGACCCGTTGGCTGGATTGTTTACGGACTTGCTTCAAGATGTCAACCATTGTTC
CTTTACCCGAGAAAGCAAAGGTAAGTGAACTAAATGACTATCGCCCTGTAGCACTCACATCTGTTATCAAGAACTACTCT
GAGAGGCTAATTAACTTTTTTAGGATAGGGGACAGCATTTAGAATTTTGGATGAAAAGGGTGCCAAAAGTAACATTCCCC
CTTCTCATGCCCAGAAGCTCGGATGTGCCTCTAATCGTCATAGTAAAACTAAAACTCGACTTCCCCCCCCCCCACACACA
CACACGCACACACCCACTCTGACACACAGCACTTTCTGGTTGCGAGGGGGAACACCTCAGCGCTATTTATAGCTTGCGTG
GCAGTGTTTGATTGGCAGAGGGGTGGGGGGGGGCTGGTTAGGACAGTGGCGCTGCAGCTGGGGACTTGACACGGCCTTGG
CACTGTCCCCGGTAGCCATGCTCATGAGTGGGCACACAAGGCAGGTTGGCACACAACACTGGCAGACTAGAAAGAGGGCA
CATAGGGCCGAACTGACAATCACACAGGGGCAGTGGTCTAGCATTGAAACCTGCAGGGAGCAAGAAAGCATGCTAATAAG
GATTGCGTTGCTAATATGTAAACGACTATAATTGGAATGAGAATCTAATGCAGTATAGCAGTGTTTCCTAATTTCTGTCC
TTGAGTTTCTGTCTCTGGGTATTCCGGTGTTTGTTGGGCGAGTCAACAATCCATTTCATTCAATTGAAAAGTAGATTGAA
TACTGGCATGCAGACCTAGGCTCCCTTGAGTGTTGTCAGAGATAAGCCCATTCATACGCGAGGTAATTCTAATAGCTAAC
AAGAAGGTCAATTGTATTAGGTTTCGGCATCAGGCAAACTGTAGTCAGAGGCAAGAGTAGGAGCAGAAGTGGCTAAGACG
GAAATAGACAGATGACTGTGCTCAGGCACTGCAAGGAGGGGTGGATGGTGGGTGGNNNNNNNNNNNNNNNNNNNNNNNNN
NNNNNNNNNNNNNNNNNNNNNNNNNNNNNNNNNNNNNNNNNNNNNNNNNNNNNNNNNNNNNNNNNNNNNNNNNNNNNNNN
NNNNNNNNNNNNNNNNNNNNNNNNNNNNNNNNNNNNNNNNNNNNNNNNNNNNNNNNNNNNNNNNNNNNNNNNNNNNNNNN
NNNNNNNNNNNNNNNNNNNNNNNNNNNNNNNNNNNNNNNNNNNNNNNNNNNNNNNNNNNNNNNNNNNNNNNNNNNNNNNN
NNNNNNNNNNNNNNNNNNNNNNNNNNNNNNNNNNNNNNNNNNNNNNNNNNNNNNNNNNNNNNNNNNNNNNNNNNNNNNNN
NNNNNNNNNNNNNNNNNNNNNNNNNNNNNNNNNNNNNNNNNNNNNNNNNNNNNNNNNNNNNNNNNNNNNNNNNNNNNNNN
NNNNNNNNNNNNNNNNNNNNNNNNNNNNNNNNNNNNNNNNNNNNNNNNNNNNNNNNNNNNNNNNNNNNNNNNNNNNNNNN
NNNNNNNNNNNNNNNNNNNNNNNNNNNNNNNNNNNNNNNNNNNNNNNNNNNNNNNNNNNNNNNNNNNNNNNNNNNNNNNN
NNNNNNNNNNNNNNNNNNNNNNNNNNNNNNNNNNNNNNNNNNNNNNNNNNNNNNNNNNNNNNNNNNNNNNNNNNNNNNNN
NNNNNNNNNNNNNNNNNNNNNNNNNNNNNNNNNNNNNNNNNNNNNNNNNNNNNNNNNNNNNNNNNNNNNNNNNNNNNNNN
NNNNNNNNNNNNNNNNNNNNNNNNNNNNNNNNNNNNNNNNNNNNNNNNNNNNNNNNNNNNNNNNNNNNNNNNNNNNNNNN
NNNNNNNNNNNNNNNNNNNNNNNNNNNNNNNNNNNNNNNNNNNNNNNNNNNNNNNNNNNNNNNNNNNNNNNNNNNNNNNN
NNNNNNNNNNNNNNNNNNNNNNNNNNNNNNNNNNNNNNNNNNNNNNNNNNNNNNNNNNNNNNNNNNNNNNNNNNNNNNNN
NNNNNNNNNNNNNNNNNNNNNNNNNNNNNNNNNNNNNNNNNNNNNNNNNNNNNNNNNNNNNNNNNNNNNNNNNNNNNNNN
NNNNNNNNNNNNNNNNNNNNNNNNNNNNNNNNNNNNNNNNNNNNNNNNNNNNNNNNNNNNNNNNNNNNNNNNNNNNNNNN
NNNNNNNNNNNNNNNNNNNNNNNNNNNNNNNNNNNNNNNNNNNNNNNNNNNNNNNNNNNNNNNNNNNNNNNNNNNNNNNN
NNNNNNNNNNNNNNNNNNNNNNNNNNNNNNNNNNNNNNNNNNNNNNNNNNNNNNNNNNNNNNNNNNNNNNNNNNNNNNNN
NNNNNNNNNNNNNNNNNNNNNNNNNNNNNNNNNNNNNNNNNNNNNNNNNNNNNNNNNNNNNNNNNNNNNNNNNNNNNNNN
NNNNNNNNNNNNNNNNNNNNNNNNNNNNNNNNNNNNNNNNNNNNNNNNNNNNNNNNNNNNNNNNNNNNNNNNNNNNNNNN
NNNNNNNNNNNNNNNNNNNNNNNNNNNNNNNNNNNNNNNNNNNNNNNNNNNNNNNNNNNNNNNNNNNNNNNNNNNNNNNN
NNNNNNNNNNNNNNNNNNNNNNNNNNNNNNNNNNNNNNNNNNNNNNNNNNNNNNNNNNNNNNNNNNNNNNNNNNNNNNNN
NNNNNNNNNNNNNNNNNNNNNNNNNNNNNNNNNNNNNNNNNNNNNNNNNNNNNNNNNNNNNNNNNNNNNNNNNNNNNNNN
NNNNNNNNNNNNNNNNNNNNNNNNNNNNNNNNNNNNNNNNNNNNNNNNNNNNNNNNNNNNNNNNNNNNNNNNNNNNNNNN
NNNNNNNNNNNNNNNNNNNNNNNNNNNNNNNNNNNNNNNNNNNNNNNNNNNNNNNNNNNNNNNNNNNNNNNNNNNNNNNN
NNNNNNNNNNNNNNNNNNNNNNNNNNNNNNNNNNNNNNNNNNNNNNNNNNNNNNNNNNNNNNNNNNNNNNNNNNNNNNNN
NNNNNNNNNNNNNNNNNNNNNNNNNNNNNNNNNNNNNNNNNNNNNNNNNNNNNNNNNNNNNNNNNNNNNNNNNNNNNNNN
NNNNNNNNNNNNNNNNNNNNNNNNNNNNNNNNNNNNNNNNNNNNNNNNNNNNNNNNNNNNNNNNNNNNNNNNNNNNNNNN
NNNNNNNNNNNNNNNNNNNNNNNNNNNNNNNNNNNNNNNNNNNNNNNNNNNNNNNNNNNNNNNNNNNNNNNNNNNNNNNN
NNNNNNNNNNNNNNNNNNNNNNNNNNNNNNNNNNNNNNNNNNNNNNNNNNNNNNNNNNNNNNNNNNNNNNNNNNNNNNNN
NNNNNNNNNNNNNNNNNNNNNNNNNNNNNNNNNNNNNNNNNNNNNNNNNNNNNNNNNNNNNNNNNNNNNNNNNNNNNNNN
NNNNNNNNNNNNNNNNNNNNNNNNNNNNNNNNNNNNNNNNNNNNNNNNNNNNNNNNNNNNNNNNNNNNNNNNNNNNNNNN
NNNNNNNNNNNNNNNNNNNNNNNNNNNNNNNNNNNNNNNNNNNNNNNNNNNNNNNNNNNNNNNNNNNNNNNNNNNNNNNN
NNNNNNNNNNNNNNNNNNNNNNNNNNNNNNNNNNNNNNNNNNNNNNNNNNNNNNNNNNNNNNNNNNNNNNNNNNNNNNNN
NNNNNNNNNNNNNNNNNNNNNNNNNNNNNNNNNNNNNNNNNNNNNNNNNNNNNNNNNNNNNNNNNNNNNNNNNNNNNNNN
NNNNNNNNNNNNNNNNNNNNNNNNNNNNNNNNNNNNNNNNNNNNNNNNNNNNNNNNNNNNNNNNNNNNNNNNNNNNNNNN

NNNNNNNNNNNNNNNNNNNNNNNNNNNNNNNNNNNNNNNNNNNNNNNNNNNNNNNNNNNNNNNNNNNNNNNNNNNNNNNN
NNNNNNNNNNNNNNNNNNNNNNNNNNNNNNNNNNNNNNNNNNNNNNNNNNNNNNNNNNNNNNNNNNNNNNNNNNNNNNNN
NNNNNNNNNNNNNNNNNNNNNNNNNNNNNNNNNNNNNNNNNNNNNNNNNNNNNNNNNNNNNNNNNNNNNNNNNNNNNNNN
NNNNNNNNNNNNNNNNNNNNNNNNNNNNNNNNNNNNNNNNNNNNNNNNNNNNNNNNNNNNNNNNNNNNNNNNNNNNNNNN
NNNNNNNNNNNNNNNNNNNNNNNNNNNNNNNNNNNNNNNNNNNNNNNNNNNNNNNNNNNNNNNNNNNNNNNNNNNNNNNN
NNNNNNNNNNNNNNNNNNNNNNNNNNNNNNNNNNNNNNNNNNNNNNNNNNNNNNNNNNNNNNNNNNNNNNNNNNNNNNNN
NNNNNNNNNNNNNNNNNNNNNNNNNNNNNNNNNNNNNNNNNNNNNNNNNNNNNNNNNNNNNNNNNNNNNNNNNNNNNNNN
NNNNNNNNNNNNNNNNNNNNNNNNNNNNNNNNNNNNNNNNNNNNNNNNNNNNNNNNNNNNNNNNNNNNNNNNNNNNNNNN
NNNNNNNNNNNNNNNNNNNNNNNNNNNNNNNNNNNNNNNNNNNNNNNNNNNNNNNNNNNNNNNNNNNNNNNNNNNNNNNN
NNNNNNNNNNNNNNNNNNNNNNNNNNNNNNNNNNNNNNNNNNNNNNNNNNNNNNNNNNNNNNNNNNNNNNNNNNNNNNNN
NNNNNNNNNNNNNNNNNNNNNNNNNNNNNNNNNNNNNNNNNNNNNNNNNNNNNNNNNNNNNNNNNNNNNNNNNNNNNNNN
NNNNNNNNNNNNNNNNNNNNNNNNNNNNNNNNNNNNNNNNNNNNNNNNNNNNNNNNNNNNNNNNNNNNNNNNNNNNNNNN
NNNNNNNNNNNNNNNNNNNNNNNNNNNNNNNNNNNNNNNNNNNNNNNNNNNNNNNNNNNNNNNNNNNNNNNNNNNNNNNN
NNNNNNNNNNNNNNNNNNNNNNNNNNNNNNNNNNNNNNNNNNNNNNNNNNNNNNNNNNNNNNNNNNNNNNNNNNNNNNNN
NNNNNNNNNNNNNNNNNNNNNNNNNNNNNNNNNNNNNNNNNNNNNNNNNNNNNNNNNNNNNNNNNNNNNNNNNNNNNNNN
NNNNNNNNNNNNNNNNNNNNNNNNNNNNNNNNNNNNNNNNNNNNNNNNNNNNNNNNNNNNNNNNNNNNNNNNNNNNNNNN
NNNNNNNNNNNNNNNNNNNNNNNNNNNNNNNNNNNNNNNNNNNNNNNNNNNNNNNNNNNNNNNNNNNNNNNNNNNNNNNN
NNNNNNNNNNNNNNNNNNNNNNNNNNNNNNNNNNNNNNNNNNNNNNNNNNNNNNNNNNNNNNNNNNNNNNNNNNNNNNNN
NNNNNNNNNNNNNNNNNNNNNNNNNNNNNNNNNNNNNNNNNNNNNNNNNNNNNNNNNNNNNNNNNNNNNNNNNNNNNNNN
NNNNNNNNNNNNNNNNNNNNNNNNNNNNNNNNNNNNNNNNNNNNNNNNNNNNNNNNNNNNNNNNNNNNNNNNNNNNNNNN
NNNNNNNNNNNNNNNNNNNNNNNNNNNNNNNNNNNNNNNNNNNNNNNNNNNNNNNNNNNNNNNNNNNNNNNNNNNNNNNN
NNNNNNNNNNNNNNNNNNNNNNNNNNNNNNNNNNNNNNNNNNNNNNNNNNNNNNNNNNNNNNNNNNNNNNNNNNNNNNNN
NNNNNNNNNNNNNNNNNNNNNNNNNNNNNNNNNNNNNNNNNNNNNNNNNNNNNNNNNNNNNNNNNNNNNNNNNNNNNNNN
NNNNNNNNNNNNNNNNNNNNNNNNNNNNNNNNNNNNNNNNNNNNNNNNNNNNNNNNNNNNNNNNNNNNNNNNNNNNNNNN
NNNNNNNNNNNNNNNNNNNNNNNNNNNNNNNNNNNNNNNNNNNNNNNNNNNNNNNNNNNNNNNNNNNNNNNNNNNNNNNN
NNNNNNNNNNNNNNNNNNNNNNNNNNNNNNNNNNNNNNNNNNNNNNNNNNNNNNNNNNNNNNNNNNNNNNNNNNNNNNNN
NNNNNNNNNNNNNNNNNNNNNNNNNNNNNNNNNNNNNNNNNNNNNNNNNNNNNNNNNNNNNNNNNNNNNNNNNNNNNNNN
NNNNNNNNNNNNNNNNNNNNNNNNNNNNNNNNNNNNNNNNNNNNNNNNNNNNNNNNNNNNNNNNNNNNNNNNNNNNNNNN
NNNNNNNNNNNNNNNNNNNNNNNNNNNNNNNNNNNNNNNNNNNNNNNNNNNNNNNNNNNNNNNNNNNNNNNNNNNNNNNN
NNNNNNNNNNNNNNNNNNNNNNNNNNNNNNNNNNNNNNNNNNNNNNNNNNNNNNNNNNNNNNNNNNNNNNNNNNNNNNNN
NNNNNNNNNNNNNNNNNNNNNNNNNNNNNNNNNNNNNNNNNNNNNNNNNNNNNNNNNNNNNNNNNNNNNNNNNNNNNNNN
NNNNNNNNNNNNNNNNNNNNNNNNNNNNNNNNNNNNNNNNNNNNNNNNNNNNNNNNNNNNNNNNNNNNNNNNNNNNNNNN
NNNNNNNNNNNNNNNNNNNNNNNNNNNNNNNNNNNNNNNNNNNNNNNNNNNNNNNNNNNNNNNNNNNNNNNNNNNNNNNN
NNNNNNNNNNNNNNNNNNNNNNNNNNNNNNNNNNNNNNNNNNNNNNNNNNNNNNNNNNNNNNNNNNNNNNNNNNNNNNNN
NNNNNNNNNNNNNNNNNNNNNNNNNNNNNNNNNNNNNNNNNNNNNNNNNNNNNNNNNNNNNNNNNNNNNNNNNNNNNNNN
NNNNNNNNNNNNNNNNNNNNNNNNNNNNNNNNNNNNNNNNNNNNNNNNNNNNNNNNNNNNNNNNNNNNNNNNNNNNNNNN
NNNNNNNNNNNNNNNNNNNNNNNNNNNNNNNNNNNNNNNNNNNNNNNNNNNNNNNNNNNNNNNNNNNNNNNNNNNNNNNN
NNNNNNNNNNNNNNNNNNNNNNNNNNNNNNNNNNNNNNNNNNNNNNNNNNNNNNNNNNNNNNNNNNNNNNNNNNNNNNNN
NNNNNNNNNNNNNNNNNNNNNNNNNNNNNNNNNNNNNNNNNNNNNNNNNNNNNNNNNNNNNNNNNNNNNNNNNNNNNNNN
NNNNNNNNNNNNNNNNNNNNNNNNNNNNNNNNNNNNNNNNNNNNNNNNNNNNNNNNNNNNNNNNNNNNNNNNNNNNNNNN
NNNNNNNNNNNNNNNNNNNNNNNNNNNNNNNNNNNNNNNNNNNNNNNNNNNNNNNNNNNNNNNNNNNNNNNNNNNNNNNN
NNNNNNNNNNNNNNNNNNNNNNNNNNNNNNNNNNNNNNNNNNNNNNNNNNNNNNNNNNNNNNNNNNNNNNNNNNNNNNNN
NNNNNNNNNNNNNNNNNNNNNNNNNNNNNNNNNNNNNNNNNNNNNNNNNNNNNNNNNNNNNNNNNNNNNNNNNNNNNNNN
NNNNNNNNNNNNNNNNNNNNNNNNNNNNNNNNNNNNNNNNNNNNNNNNNNNNNNNNNNNNNNNNNNNNNNNNNNNNNNNN
NNNNNNNNNNNNNNNNNNNNNNNNNNNNNNNNNNNNNNNNNNNNNNNNNNNNNNNNNNNNNNNNNNNNNNNNNNNNNNNN
NNNNNNNNNNNNNNNNNNNNNNNNNNNNNNNNNNNNNNNNNNNNNNNNNNNNNNNNNNNNNNNNNNNNNNNNNNNNNNNN
NNNNNNNNNNNNNNNNNNNNNNNNNNNNNNNNNNNNNNNNNNNNNNNNNNNNNNNNNNNNNNNNNNNNNNNNNNNNNNNN
NNNNNNNNNNNNNNNNNNNNNNNNNNNNNNNNNNNNNNNNNNNNNNNNNNNNNNNNNNNNNNNNNNNNNNNNNNNNNNNN
NNNNNNNNNNNNNNNNNNNNNNNNNNNNNNNNNNNNNNNNNNNNNNNNNNNNNNNNNNNNNNNNNNNNNNNNNNNNNNNN
NNNNNNNNNNNNNNNNNNNNNNNNNNNNNNNNNNNNNNNNNNNNNNNNNNNNNNNNNNNNNNNNNNNNNNNNNNNNNNNN
NNNNNNNNNNNNNNNNNNNNNNNNNNNNNNNNNNNNNNNNNNNNNNNNNNNNNNNNNNNNNNNNNNNNNNNNNNNNNNNN
NNNNNNNNNNNNNNNNNNNNNNNNNNNNNNNNNNNNNNNNNNNNNNNNNNNNNNNNNNNNNNNNNNNNNNNNNNNNNNNN
NNNNNNNNNNNNNNNNNNNNNNNNNNNNNNNNNNNNNNNNNNNNNNNNNNNNNNNNNNNNNNNNNNNNNNNNNNNNNNNN
NNNNNNNNNNNNNNNNNNNNNNNNNNNNNNNNNNNNNNNNNNNNNNNNNNNNNNNNNNNNNNNNNNNNNNNNNNNNNNNN
NNNNNNNNNNNNNNNNNNNNNNNNNNNNNNNNNNNNNNNNNNNNNNNNNNNNNNNNNNNNNNNNNNNNNNNNNNNNNNNN
NNNNNNNNNNNNNNNNNNNNNNNNNNNNNNNNNNNNNNNNNNNNNNNNNNNNNNNNNNNNNNNNNNNNNNNNNNNNNNNN
NNNNNNNNNNNNNNNNNNNNNNNNNNNNNNNNNNNNNNNNNNNNNNNNNNNNNNNNNNNNNNNNNNNNNNNNNNNNNNNN
NNNNNNNNNNNNNNNNNNNNNNNNNNNNNNNNNNNNNNNNNNNNNNNNNNNNNNNNNNNNNNNNNNNNNNNNNNNNNNNN
NNNNNNNNNNNNNNNNNNNNNNNNNNNNNNNNNNNNNNNNNNNNNNNNNNNNNNNNNNNNNNNNNNNNNNNNNNNNNNNN
NNNNNNNNNNNNNNNNNNNNNNNNNNNNNNNNNNNNNNNNNNNNNNNNNNNNNNNNNNNNNNNNNNNNNNNNNNNNNNNN
NNNNNNNNNNNNNNNNNNNNNNNNNNNNNNNNNNNNNNNNNNNNNNNNNNNNNNNNNNNNNNNNNNNNNNNNNNNNNNNN
NNNNNNNNNNNNNNNNNNNNNNNNNNNNNNNNNNNNNNNNNNNNNNNNNNNNNNNNNNNNNNNNNNNNNNNNNNNNNNNN
NNNNNNNNNNNNNNNNNNNNNNNNNNNNNNNNNNNNNNNNNNNNNNNNNNNNNNNNNNNNNNNNNNNNNNNNNNNNNNNN
NNNNNNNNNNNNNNNNNNNNNNNNNNNNNNNNNNNNNNNNNNNNNNNNNNNNNNNNNNNNNNNNNNNNNNNNNNNNNNNN
NNNNNNNNNNNNNNNNNNNNNNNNNNNNNNNNNNNNNNNNNNNNNNNNNNNNNNNNNNNNNNNNNNNNNNNNNNNNNNNN
NNNNNNNNNNNNNNNNNNNNNNNNNNNNNNNNNNNNNNNNNNNNNNNNNNNNNNNNNNNNNNNNNNNNNNNNNNNNNNNN
NNNNNNNNNNNNNNNNNNNNNNNNNNNNNNNNNNNNNNNNNNNNNNNNNNNNNNNNNNNNNNNNNNNNNNNNNNNNNNNN
NNNNNNNNNNNNNNNNNNNNNNNNNNNNNNNNNNNNNNNNNNNNNNNNNNNNNNNNNNNNNNNNNNNNNNNNNNNNNNNN
NNNNNNNNNNNNNNNNNNNNNNNNNNNNNNNNNNNNNNNNNNNNNNNNNNNNNNNNNNNNNNNNNNNNNNNNNNNNNNNN

NNNNNNNNNNNNNNNNNNNNNNNNNNNNNNNNNNNNNNNNNNNNNNNNNNNNNNNNNNNNNNNNNNNNNNNNNNNNNNNN
NNNNNNNNNNNNNNNNNNNNNNNNNNNNNNNNNNNNNNNNNNNNNNNNNNNNNNNNNNNNNNNNNNNNNNNNNNNNNNNN
NNNNNNNNNNNNNNNNNNNNNNNNNNNNNNNNNNNNNNNNNNNNNNNNNNNNNNNNNNNNNNNNNNNNNNNNNNNNNNNN
NNNNNNNNNNNNNNNNNNNNNNNNNNNNNNNNNNNNNNNNNNNNNNNNNNNNNNNNNNNNNNNNNNNNNNNNNNNNNNNN
NNNNNNNNNNNNNNNNNNNNNNNNNNNNNNNNNNNNNNNNNNNNNNNNNNNNNNNNNNNNNNNNNNNNNNNNNNNNNNNN
NNNNNNNNNNNNNNNNNNNNNNNNNNNNNNNNNNNNNNNNNNNNNNNNNNNNNNNNNNNNNNNNNNNNNNNNNNNNNNNN
NNNNNNNNNNNNNNNNNNNNNNNNNNNNNNNNNNNNNNNNNNNNNNNNNNNNNNNNNNNNNNNNNNNNNNNNNNNNNNNN
NNNNNNNNNNNNNNNNNNNNNNNNNNNNNNNNNNNNNNNNNNNNNNNNNNNNNNNNNNNNNNNNNNNNNNNNNNNNNNNN
NNNNNNNNNNNNNNNNNNNNNNNNNNNNNNNNNNNNNNNNNNNNNNNNNNNNNNNNNNNNNNNNNNNNNNNNNNNNNNNN
NNNNNNNNNNNNNNNNNNNNNNNNNNNNNNNNNNNNNNNNNNNNNNNNNNNNNNNNNNNNNNNNNNNNNNNNNNNNNNNN
NNNNNNNNNNNNNNNNNNNNNNNNNNNNNNNNNNNNNNNNNNNNNNNNNNNNNNNNNNNNNNNNNNNNNNNNNNNNNNNN
NNNNNNNNNNNNNNNNNNNNNNNNNNNNNNNNNNNNNNNNNNNNNNNNNNNNNNNNNNNNNNNNNNNNNNNNNNNNNNNN
NNNNNNNNNNNNNNNNNNNNNNNNNNNNNNNNNNNNNNNNNNNNNNNNNNNNNNNNNNNNNNNNNNNNNNNNNNNNNNNN
NNNNNNNNNNNNNNNNNNNNNNNNNNNNNNNNNNNNNNNNNNNNNNNNNNNNNNNNNNNNNNNNNNNNNNNNNNNNNNNN
NNNNNNNNNNNNNNNNNNNNNNNNNNNNNNNNNNNNNNNNNNNNNNNNNNNNNNNNNNNNNNNNNNNNNNNNNNNNNNNN
NNNNNNNNNNNNNNNNNNNNNNNNNNNNNNNNNNNNNNNNNNNNNNNNNNNNNNNNNNNNNNNNNNNNNNNNNNNNNNNN
NNNNNNNNNNNNNNNNNNNNNNNNNNNNNNNNNNNNNNNNNNNNNNNNNNNNNNNNNNNNNNNNNNNNNNNNNNNNNNNN
NNNNNNNNNNNNNNNNNNNNNNNNNNNNNNNNNNNNNNNNNNNNNNNNNNNNNNNNNNNNNNNNNNNNNNNNNNNNNNNN
NNNNNNNNNNNNNNNNNNNNNNNNNNNNNNNNNNNNNNNNNNNNNNNNNNNNNNNNNNNNNNNNNNNNNNNNNNNNNNNN
NNNNNNNNNNNNNNNNNNNNNNNNNNNNNNNNNNNNNNNNNNNNNNNNNNNNNNNNNNNNNNNNNNNNNNNNNNNNNNNN
NNNNNNNNNNNNNNNNNNNNNNNNNNNNNNNNNNNNNNNNNNNNNNNNNNNNNNNNNNNNNNNNNNNNNNNNNNNNNNNN
NNNNNNNNNNNNNNNNNNNNNNNNNNNNNNNNNNNNNNNNNNNNNNNNNNNNNNNNNNNNNNNNNNNNNNNNNNNNNNNN
NNNNNNNNNNNNNNNNNNNNNNNNNNNNNNNNNNNNNNNNNNNNNNNNNNNNNNNNNNNNNNNNNNNNNNNNNNNNNNNN
NNNNNNNNNNNNNNNNNNNNNNNNNNNNNNNNNNNNNNNNNNNNNNNNNNNNNNNNNNNNNNNNNNNNNNNNNNNNNNNN
NNNNNNNNNNNNNNNNNNNNNNNNNNNNNNNNNNNNNNNNNNNNNNNNNNNNNNNNNNNNNNNNNNNNNNNNNNNNNNNN
NNNNNNNNNNNNNNNNNNNNNNNNNNNNNNNNNNNNNNNNNNNNNNNNNNNNNNNNNNNNNNNNNNNNNNNNNNNNNNNN
NNNNNNNNNNNNNNNNNNNNNNNNNNNNNNNNNNNNNNNNNNNNNNNNNNNNNNNNNNNNNNNNNNNNNNNNNNNNNNNN
NNNNNNNNNNNNNNNNNNNNNNNNNNNNNNNNNNNNNNNNNNNNNNNNNNNNNNNNNNNNNNNNNNNNNNNNNNNNNNNN
NNNNNNNNNNNNNNNNNNNNNNNNNNNNNNNNNNNNNNNNNNNNNNNNNNNNNNNNNNNNNNNNNNNNNNNNNNNNNNNN
NNNNNNNNNNNNNNNNNNNNNNNNNNNNNNNNNNNNNNNNNNNNNNNNNNNNNNNNNNNNNNNNNNNNNNNNNNNNNNNN
NNNNNNNNNNNNNNNNNNNNNNNNNNNNNNNNNNNNNNNNNNNNNNNNNNNNNNNNNNNNNNNNNNNNNNNNNNNNNNNN
NNNNNNNNNNNNNNNNNNNNNNNNNNNNNNNNNNNNNNNNNNNNNNNNNNNNNNNNNNNNNNNNNNNNNNNNNNNNNNNN
NNNNNNNNNNNNNNNNNNNNNNNNNNNNNNNNNNNNNNNNNNNNNNNNNNNNNNNNNNNNNNNNNNNNNNNNNNNNNNNN
NNNNNNNNNNNNNNNNNNNNNNNNNNNNNNNNNNNNNNNNNNNNNNNNNNNNNNNNNNNNNNNNNNNNNNNNNNNNNNNN
NNNNNNNNNNNNNNNNNNNNNNNNNNNNNNNNNNNNNNNNNNNNNNNNNNNNNNNNNNNNNNNNNNNNNNNNNNNNNNNN
NNNNNNNNNNNNNNNNNNNNNNNNNNNNNNNNNNNNNNNNNNNNNNNNNNNNNNNNNNNNNNNNNNNNNNNNNNNNNNNN
NNNNNNNNNNNNNNNNNNNNNNNNNNNNNNNNNNNNNNNNNNNNNNNNNNNNNNNNNNNNNNNNNNNNNNNNNNNNNNNN
NNNNNNNNNNNNNNNNNNNNNNNNNNNNNNNNNNNNNNNNNNNNNNNNNNNNNNNNNNNNNNNNNNNNNNNNNNNNNNNN
NNNNNNNNNNNNNNNNNNNNNNNNNNNNNNNNNNNNNNNNNNNNNNNNNNNNNNNNNNNNNNNNNNNNNNNNNNNNNNNN
NNNNNNNNNNNNNNNNNNNNNNNNNNNNNNNNNNNNNNNNNNNNNNNNNNNNNNNNNNNNNNNNNNNNNNNNNNNNNNNN
NNNNNNNNNNNNNNNNNNNNNNNNNNNNNNNNNNNNNNNNNNNNNNNNNNNNNNNNNNNNNNNNNNNNNNNNNNNNNNNN
NNNNNNNNNNNNNNNNNNNNNNNNNNNNNNNNNNNNNNNNNNNNNNNNNNNNNNNNNNNNNNNNNNNNNNNNNNNNNNNN
NNNNNNNNNNNNNNNNNNNNNNNNNNNNNNNNNNNNNNNNNNNNNNNNNNNNNNNNNNNNNNNNNNNNNNNNNNNNNNNN
NNNNNNNNNNNNNNNNNNNNNNNNNNNNNNNNNNNNNNNNNNNNNNNNNNNNNNNNNNNNNNNNNNNNNNNNNNNNNNNN
NNNNNNNNNNNNNNNNNNNNNNNNNNNNNNNNNNNNNNNNNNNNNNNNNNNNNNNNNNNNNNNNNNNNNNNNNNNNNNNN
NNNNNNNNNNNNNNNNNNNNNNNNNNNNNNNNNNNNNNNNNNNNNNNNNNNNNNNNNNNNNNNNNNNNNNNNNNNNNNNN
NNNNNNNNNNNNNNNNNNNNNNNNNNNNNNNNNNNNNNNNNNNNNNNNNNNNNNNNNNNNNNNNNNNNNNNNNNNNNNNN
NNNNNNNNNNNNNNNNNNNNNNNNNNNNNNNNNNNNNNNNNNNNNNNNNNNNNNNNNNNNNNNNNNNNNNNNNNNNNNNN
NNNNNNNNNNNNNNNNTATACATGAATGGGACTCGTCTGTGTGTGTGTGTGTGTGTGTGTGTGTGTGTGTGTGTGTGTGT
GTGTGTGTGTGTGTGTGTGTGTGTGTGTGTGTGTGTGTGTGTGTGTGTGTGTGTGTGTGTGTGTGTGTTTGAGTGTGA
ATTACTGTGACTGCAAAACCACAGTGATCCACATTCACAGGTAACAATGCTGCTGTATTTATTTGCCCACGTCTATCACC
TATCATGATGTGTTCTCCCGTCTCTCTCTGTTTGCATATCCTGTCTTATGTCACCATGTGATGGGTGCTCCACTGAGAGA
AAGCAGGTCAAGCCTTTGGTCCTCAGAGATAAATAATAATTATAGTGATCTGTCCTCAATCCAGAGATAGACTAAGAACT
GTGTCATTGTTATATTACCCAAAATGGCAATCTCTCAAACTCCTACACAGCTTAAGTCATCTCGTCTGACAGACAGAGGA
GTGACGGGGAGTCTGACTGGGAGGCCCTGTGTTGCCCTGGGTGGAATTATGAAAGAACCTTCTGATTGAGAAAAAGCCAG
AGGTGTCTTGGATCAGAGGTATCTGGGGTATTGGCACCGTTTTGCTTCCTTGATTTTTACAGATCAACGCAATGATGTCT
GAGTAGGAATACACAGAAACCATCTAAATGGAATTTAGTTATGGGTTTTCTGCTTTCACTTCATCACCAAGATGTCTGTC
ATGTTCAGTGGGGTTTGAAATGATTGCCACCCTTGATAAAGATGACCAAAAATGACTGTATAAAATAAATAACTCAAATA
CTGAAAAGGGAAATGATATTGTTTTATACTAATACAGTTGTTCCGAGAAATATATTTTGTTTAACAAGTAATAAAAAACA
ATCTCAAAAAAGGTAGGGCTCAAATTATCAACACCCCTGTTTTCTATACCGTTCAATACCTCACCTTGCGAGGACAACGC
AATGCCTTTTTCAAAAAATGATTTATGAGATTGGCGAACACATTGGGAGGGATTTTAGACCATCCCTCCATACAGAATCT
TTCCAGATCCTTGATATCCTTCGTCTACGCTTATGGACTGCCCTCTTCAATTCAATCCACAGGTTTTCAATGGGGGTCAT
GTCCGGAGACTGAGATGGCCTTTGCAAAATGTTGATTTTGTGGTCAATGAACAAGTTCTTTGTTGATTTTGATGTGTGCT
TGGGGTTATTGTCTTGCTGTAAGATCCACTTGCTGGAATATCCTATTGCGGCAAAGTTTCAGCCTCCTGGCAGAGGCAAT
CAGGATTTTGGCTGAAATGTTCTGGTACTGTGTGAAGTTCATGATTTTTAAGAAAGATTTTTAAGCCTTGAGACAATTAA
GAAATGGATTGTGTAAGTGTGCCATTCAGAGGTTGAATGGGCAAGACAAAAGATTTAAGTTCCTTTGAACGGGGTATGGT
AGTAGGTGCCAGGTGCACCGGTTTTCCCGTGTGTATCAAGAATGGCCCACCACCCAAAGGACATCCAGTCAACTTGACAC
AACTGTGGGAAGCATTGGAGCCAACATGGGCCAGCATCCCTGTGGAACGCTTTCAACACCTTGTAGAGTCCATGCCCCGA
CGAATTAAGACTGTTCTGAGGGCAAAGGGGGTGCAATTCAATATTAGGAAGGTGTTCTTAATGTTTTGTACACTCAGTGT

ATATAGTTCGATATCTTTTCTGCATGCTTATCTGGTTTTGGTCATTTAAGTTGACACTGAAACTGTTTTTCTATCCAATA
AATATCCAATACATTTTCTACACAAAAAACTCCTGTAATTAGCTCCAATGACTGTAATTTCCTCCATATTAAAGGGATAG
TTTGAGATTTTGGCAACAAAGCCTTTTATATACTTTCCCAGAGTCCAGCTAGCATGCTAACTGTTCCTGTAGACTTCCAG
TCATTGAACTTTTTCAGACTGGACACAGAAACATAAAATTGGTACACATGATGGTATTCTGTTGCAGCTAGCAAAAATTC
ACAAAATTATAATTTTTCACATTAAAAAATCCCCAAAATGATTTTCACAAAAAAATATATTTTTTTAAAATCGCAGACCC
CCTGCAGCACCTCTGCGGACCCTAGTTTGAAACCCCCTGAATTAAGCAACTGCACAGAGCTCACCTCAGGGTTTTCTACA
GTACACAACGCTAACACATTAACATGAATGGACATATGCCTTCATGGCAAACAAACGTCCTAATCTCAACAGTATCTACC
TGCAGCAGGCTACCAGAACAGAGCAGCCCGTACTGCCAGATTCTATTTATTAATATCATTACTTTGAATTAGGTTTTAAA
TGAGAAGTGTAAGGGATGAGATAGTAAGACAAACCCTAACTGAGTCCCAGACCCTCTTAGACTTGTCTGTGTATGTTACC
GCATATTATCTCATCCCTTGTTGCACTAAAACAAATACAGTAAAATTGTACTTGGGCGTCCAAGACCACTGTCTGTTTCA
GTTATAAATACTGCAATACCTTCATTCATTATGTAAGTCAGCTTTTCCTATTTTTGAGTATTCTCCTCATCTTTCTCCAC
ATGTTCAAATGCTCCTACATTCAGAGGGAGCAATGACATCATCATCACCCCAATTACCTTACACAATCCCTTCCCCTGAC
TGAGCGATCTGATTGATTCAATCAACACAAACGGTTATACAACTTCCTGTGTCATCAAGGTATCGGTGGTCATGTGGTGA
CACATTTGCTGTCTGGGTGACAGTAGCCGTACAGAAGCTACTTAGCTGAAAGGACAAGAAAGGGGACTTGAGCAGACAAG
ATTAACTTGTGTATCTGTAGCGATCAAATGCATGCCAAATAAATCACTTTTTGCTCAATAAAGTAGTCTCCCTCTGTCTC
AATCAACAACTAACATTATTTTCCATTCAAAAAACTTTAGTGACTTGGATAACTGATCTCAATTGTATATTCCCCCAAAA
ACATAATTTAGATATGTATCTGGTCACCATTACTGCAACTTCAATCAGGTAAATACGATATGTTTTTACTGTTGTTAAAG
TCCCTTGTGCCCCATGTATGCCCGAGGTCTACAAAATAAGTGTAATTTAACTGTATCTTCCTTCTGATCAAGTTGCACAA
ATATATAGTTGTGTGTCTTTTTTACTCTCTCATGTGAAAAAGGTCAACTGTAGAACCAAATAAACAAAACTTGTTTTGAC
AGCAAAGCTGTTGTAGAGCACTGATACTCTACCTCTGCATTCAGCACCACTGAAGTGGACAGCGACTGAATCGGTTTTCT
GAAGTTCAGCCCCACATGTCAGTGGAAAGCGACTGTGTATAGTCAATGGCAGTGTGGAATGATGGTAAGGCCATTATACT
TTAACCTAGAAAGGAACCTGTACTGAAGAAAAATATAAACGCAACATGCAACAATCAATTGAAAAAAATGTATTGGTCCC
TAATCTACGGAATTCACATAACTGGGAATACAGATATGCATCTCTTGTCACAGATACCTAAGGGGCGTGGATAAGATAAC
CAGTACCACCATTTGCCTCATGCAGCCGTTTGCCTCATGCAGACATACTGCCAAATTCTCTAAAATGACGTTGGAGGCGG
CTTATGGTAGAGAAATTAACATTAAATTCTTTGGTAACAGCTCTAGTGGACATTCCTGCAGTCAGCATGCCAATTGCACG
CTCTCTCAAAATGTGAGATATCTGTGGCATTGTGTTGTGTGGCAAAACTGCACATCTCAGAGTGGCATTTTATTGTCCCC
AGTACAAGGTGCACCTGTGTGATGATCATGCTGTTTAATCAGCTTCTTGATATGCCACACCTGTCAGGTGGATGGATTAT
CTTGGCAAAGGAGAAATGCTCACTAACAGGGATGTAAACAAATTTGTGCACAAAATTATAGAGAAATTCCATACGTTTTT
GTGCGTATGAAAATGTCTATTTCAGCTCATGAAACACGGGACCAACACTTTACATATTGCATTTATATTTTTGTTCAGTC
CATATAACCCTGTGTGTCATGTTCTTCATCTCAAACCAGGGGTTATCCAGCTCTGACATCTCGCTTGCTTGGTCAACACA
ATCTCAGTCAACTTTTCACCCCTAGCTAATATGAACACTCACCACTCGCTAGGTCAAACAAACTGAGACACTTTGAAGCC
AGTTCTGTGGTTAAAGTGAGTGTGAATTCCAGTGTAGTATCTTCATGCAGGAAAGGAGACTTCAAACATGGCATTGAAGC
GAGAAGCTACAATTAAAAGTGTGTCATAGGTGTCTCACACTTTCCTCTCCCAGCAGTCCATTTCTACCAGTTAGACTCTG
ATCAAAGAGATCAGGATGTTCTGCCATTATCTCCACTCTCATACAGAGCAACTCAACTCCACATTCAAATGCCAAAAAGA
GCAACAACTGCAAGATAGACTCTCTGAAAAACACTAGGACAGGAGCTTTCCTCCTGAAAAGAAAACTTACATAAAAAACA
CTAGAAATTGGATTGCATTTCCAGTCTCTCTCAACAATCCACTCCAATTTAGATGATTCATTATTTGAAATAACAAAAGG
GCATATCACTTAAACATTGCATGAATGCCAGCAAATACAGTTGAAATGCACTTATATTTTGTGTGGTGAAGATTCAAATG
TGGTGCAGATGAGATGCTAAATATAGCGACAGGCATGCAGAGTGAGCCTGCTAACCTTTATGAGATCTTCAGAGTAGAAG
TTAATCACTAAAATAAAAACACACACAGACTCTCTCTTTCTCTCTCTCTCTCTCTCTCTCTCACACACACACACGCACAC
ACGCACAGACACACGCACACGCAAACACACATACGCACATGTGTAGGATCTTAATTTGAGCCAGTTTGCTACAGCAGGAA
ATGTGGATTATTATGTGGATTATAATCAATGGACAATATTGAATGGGTTGATACATTTTCCGTAAGGGCAAATCAAGTTT
GAAATTTCAAAGTGGAAATTACAAACTTCAGGAGCCTTTTTACCTCAAATACACTACAAGTGCTCTGTACACAAGCACAC
TCACAAACACGCACACACACACACAAAAGCATGCACGGCACATGCATGGCACACACACACACACACACACACACACACAC
ACATTCTTGTGAGCCTTGTAAAATGACTAATCTTTTTCTGAAGTAGAGACACTACCCTAAATGTGTAATAGTGAAACACT
TTAAAATGGTAATAGACTACTCTGAAATGTCAGGGTCCAATGGCACCGTCGTTGAGTATCAGCATATCAGTGTATTTGAT
TACAGCTAATTAAAATGCACCATTCTTCTTTGCAGTATCTGCACATCTGTGAATGAGAGGTACATCGCGTGAAAATCCGG
TCCTCTTTTCTCACCACCTTCAGTAAAAGTGCACACTGCATATATCAGCCACCTATTAGCGTCACAAGAGAGGAGGGACG
AATTAGCCCATCGCTAGCATGGTATTGACGGTTTGAGAGCACATTTGAATTAGCCGTCTGCCTTTATAACACTGTAATAA
GATGGCTGTAAAGTCCTGTCAGCACATTATGACGTCCTGACTGGCTACTGTAACTTTCCTAAACCTTACGCCATATCAGA
AAACAAACTAACATTCAAATGTCAAGTTCTATAGTATATCAATGAGTAATGTGAGGTTGTCTGCTACTGTCATGGCCTGA
TTTGACCTAGGGATAATATGTATGCAGTCTATACATTATGACATCCTACATCAAGTGTTACTTGACTTACAAGAAAGTGT
GAAATGTACACATAACACTGAAAATAGACTTCAAATGTCTGGGACTGAACATCTCAACAGGTGAGTGATTAACTACCCTA
CAGAGCAATACCTCTTGGTTCTCATTGTAACTCTGGGTCCCAGGTACGTACAAGGCTTCAGTGAACATTATCTTTGTGAG
CCCAAATCTCCAATTTGTAATCCTGCTTGAGGCACCACAGTAGTGTTTTAATAATCTGTTCTTTTAGGGGGCACTTATCA
CAGGCTGTACTATCAGCATCTCAAATGACACGGTGTGGAATTCAGACTCTCCCGAAACCTATCCCAGGCCATTTATTTTA
GATACTTTGACGGAAAAGGGTCAGAAAGGGTTGTTTGAAAAAGCCCTATACTGTGCCGATAACTGAATTGCATATAAATA
ATATTAATGGACAGTATGGGTATAAACCAAAAACATAAAAGTAATGTTTCATCTTACAGATGTAGGATCTTAATTTGAGC
CAGTTTGCTACAGCAGGAAAATAATCTTGCAGCAGCTGGAAATTTGAATTGTTATGTGGATTATAGTTAATGGACATTTT
TGTAGGGGTTGGTGCATTTTTCGTAAGGGAAAATCAAGTCTGAGATTTCTAAGTAGAAATTACAAATGTCTTTTTAAACC
TCAAATACACTTCAAGCTTGACATGTCCTGTATTTCAGGAACGTTCTCCTGCAACAGGCTGATCAAATTAAGATCCTACC
TGTACATTACAAACTAACATCACACCTTTAACAACTAAATGTTATTTTTTAATGATACATCTGCAAGTGAAACCTGCTGT
TTTTAGTTATAATCAGCTTTTTATGACATTCAATCGTCCCTACAAACATCCAATAACTGGTCCATTTTGACTCCATGACT
AATATAATTTGTTCTATTACTAATATAATGACATATGTACAGTAAGTTGTTGAATATCTCCAGGTCTGAAGGCACAACCT
GACTGTGCATCATGAGAATCCCTGGAATCCAATGACATAATGTCTGGAAGGCCTGTGAGCCAACCATTATCCCGGATTGG
CAGCCCTCTGATTGGCTTAGGAGAGGAGATAGCTCATCCTGACCTTTTGCCATGAGTGAATCCTGACAGGATCAATCAAA
AGACGAGCATCCTTTAAACACAAACTCATTGAGTCATTGGGCTCCAGGCTTTGCGGTGGAATTGAATAAGTGGGGCCCAC
TCCCATAGTGTGGTCTGCATTAAGCAACCATTTCTCCTAGCTACTCATTTCTGATGGGATCCTTATGTAGATTTCCTCAG
AATCAGCAGTAAGGCTTTTAGCGGATGGGGTATGTCTGGGCCTTTTCACCACCAGGTTCAATCAGTACTGCTGGGGAACA
GAGAGATAGTGAGGGAGTGGAGCCGTGAACTGAGGCTTAGGGGCATGCCTGAACTCACTGCACTAGAACTGTTCAAGGGG

GAGAGAGTTCCCTGACATGGCCCTATATCCATAGACTACAGATGTAGGATCTTAATTTGAGCCAGGAAATGTGAATTAAA
TGTGTATTATAATTAATGAGCTCAGCCAGTACTCAAGCAAACTACAGTAGCAAAAAACATTCGGTTTGTCTGATTTCATT
TTTGGACTACCCCTATTAGCATTATTACAATATCATTTTAAGGTCTTTCAACCACAACAAACATGTCCTTCCCCCCAGAC
AACATGGCTTATGGGGGGCAGCCCTCAAAATAACATTTTTGCTGACACTACCCAGAGGACATTTTATCATGTCACACTCA
GAAACCATCAAGGACATTGGAGCACTGTGATACCCTGTAATAAACCGTGTACATTTTATACTGTGCTTTGGCTGAATGAC
ACATGCTGCAGTGGACACTTTCACTTAAGCATGGATATCTAACACAAGGCACGATACTTTATGGCCTGGAACATGTTTCA
ATCTGGACCCTATCTCGCTGTGCCGTATAACATCATGGTCCACGGTGCAATATGAACACTTTTTATGATAAAAGGCAAGT
CGTTGGTTGACCTAGTACAAAACCAAAAACCTTTAATAAGCATCTGCCAGTAAATGATAATATGCGTAAATAATCTTGCC
ATCAAACTGCTCTACGCCACCTCCTGTGCTTATTCATACGTTCGCTGCACTGTGCCATGAATATGCTAAACAGTATGCAG
ATACAAGTAAATGAGGGGCAGTTTAACTTTGATTGACTTTACGTCTGTGGGCCAAAATTATGAATACGATATTTCCTTGA
GGATACCGAACAACACTGTCATTGCTTTCATGTATACGCTAGCTGTTAGCATCGCTGATAAAGGCTGAGGCATAGTCACG
CTTGACGTTCTTGAACGTTGACTTTTCACGTCTCGAGGGAAGTGGNNNNNNNNNNNNNNNNNNNNNNNNNNNNNNNNNNN
NNNNNNNNNNNNNNNNNNNNNNNNNNNNNNNNNNNNNNNNNNNNNNNNNNNNNNNNNNNNNNNNNNNNNNNNNNNNNNNN
NNNNNNNNNNNNNNNNNNNNNNNNNNNNNNNNNNNNNNNNNNNNNNNNNNNNNNNNNNNNNNNNNNNNNNNNNNNNNNNN
NNNNNNNNNNNNNNNNNNNNNNNNNNNNNNNNNNNNNNNNNNNNNNNNNNNNNNNNNNNNNNNNNNNNNNNNNNNNNNNN
NNNNNNNNNNNNNNNNNNNNNNNNNNNNNNNNNNNNNNNNNNNNNNNNNNNNNNNNNNNNNNNNNNNNNNNNNNNNNNNN
NNNNNNNNNNNNNNNNNNNNNNNNNNNNNNNNNNNNNNNNNNNNNNNNNNNNNNNNNNNNNNNNNNNNNNNNNNNNNNNN
NNNNNNNNNNNNNNNNNNNNNNNNNNNNNNNNNNNNNNNNNNNNNNNNNNNNNNNNNNNNNNNNNNNNNNNNNNNNNNNN
NNNNNNNNNNNNNNNNNNNNNNNNNNNNNNNNNNNNNNNNNNNNNNNNNNNNNNNNNNNNNNNNNNNNNNNNNNNNNNNN
NNNNNNNNNNNNNNNNNNNNNNNNNNNNNNNNNNNNNNNNNNNNNNNNNNNNNNNNNNNNNNNNNNNNNNNNNNNNNNNN
NNNNNNNNNNNNNNNNNNNNNNNNNNNNNNNNNNNNNNNNNNNNNNNNNNNNNNNNNNNNNNNNNNNNNNNNNNNNNNNN
NNNNNNNNNNNNNNNNNNNNNNNNNNNNNNNNNNNNNNNNNNNNNNNNNNNNNNNNNNNNNNNNNNNNNNNNNNNNNNNN
NNNNNNNNNNNNNNNNNNNNNNNNNNNNNNNNNNNNNNNNNNNNNNNNNNNNNNNNNNNNNNNNNNNNNNNNNNNNNNNN
NNNNNNNNNNNNNNNNNNNNNNNNNNNNNNNNNNNNNNNNNNNNNNNNNNNNNNNNNNNNNNNNNNNNNNNNNNNNNNNN
NNNNNNNNNNNNNNNNNNNNNNNNNNNNNNNNNNNNNNNNNNNNNNNNNNNNNNNNNNNNNNNNNNNNNNNNNNNNNNNN
NNNNNNNNNNNNNNNNNNNNNNNNNNNNNNNNNNNNNNNNNNNNNNNNNNNNNNNNNNNNNNNNNNNNNNNNNNNNNNNN
NNNNNNNNNNNNNNNNNNNNNNNNNNNNNNNNNNNNNNNNNNNNNNNNNNNNNNNNNNNNNNNNNNNNNNNNNNNNNNNN
NNNNNNNNNNNNNNNNNNNNNNNNNNNNNNNNNNNNNNNNNNNNNNNNNNNNNNNNNNNNNNNNNNNNNNNNNNNNNNNN
NNNNNNNNNNNNNNNNNNNNNNNNNNNNNNNNNNNNNNNNNNNNNNNNNNNNNNNNNNNNNNNNNNNNNNNNNNNNNNNN
NNNNNNNNNNNNNNNNNNNNNNNNNNNNNNNNNNNNNNNNNNNNNNNNNNNNNNNNNNNNNNNNNNNNNNNNNNNNNNNN
NNNNNNNNNNNNNNNNNNNNNNNNNNNNNNNNNNNNNNNNNNNNNNNNNNNNNNNNNNNNNNNNNNNNNNNNNNNNNNNN
NNNNNNNNNNNNNNNNNNNNNNNNNNNNNNNNNNNNNNNNNNNNNNNNNNNNNNNNNNNNNNNNNNNNNNNNNNNNNNNN
NNNNNNNNNNNNNNNNNNNNNNNNNNNNNNNNNNNNNNNNNNNNNNNNNNNNNNNNNNNNNNNNNNNNNNNNNNNNNNNN
NNNNNNNNNNNNNNNNNNNNNNNNNNNNNNNNNNNNNNNNNNNNNNNNNNNNNNNNNNNNNNNNNNNNNNNNNNNNNNNN
NNNNNNNNNNNNNNNNNNNNNNNNNNNNNNNNNNNNNNNNNNNNNNNNNNNNNNNNNNNNNNNNNNNNNNNNNNNNNNNN
NNNNNNNNNNNNNNNNNNNNNNNNNNNNNNNNNNNNNNNNNNNNNNNNNNNNNNNNNNNNNNNNNNNNNNNNNNNNNNNN
NNNNNNNNNNNNNNNNNNNNNNNNNNNNNNNNNNNNNNNNNNNNNNNNNNNNNNNNNNNNNNNNNNNNNNNNNNNNNNNN
NNNNNNNNNNNNNNNNNNNNNNNNNNNNNNNNNNNNNNNNNNNNNNNNNNNNNNNNNNNNNNNNNNNNNNNNNNNNNNNN
NNNNNNNNNNNNNNNNNNNNNNNNNNNNNNNNNNNNNNNNNNNNNNNNNNNNNNNNNNNNNNNNNNNNNNNNNNNNNNNN
NNNNNNNNNNNNNNNNNNNNNNNNNNNNNNNNNNNNNNNNNNNNNNNNNNNNNNNNNNNNNNNNNNNNNNNNNNNNNNNN
NNNNNNNNNNNNNNNNNNNNNNNNNNNNNNNNNNNNNNNNNNNNNNNNNNNNNNNNNNNNNNNNNNNNNNNNNNNNNNNN
NNNNNNNNNNNNNNNNNNNNNNNNNNNNNNNNNNNNNNNNNNNNNNNNNNNNNNNNNNNNNNNNNNNNNNNNNNNNNNNN
NNNNNNNNNNNNNNNNNNNNNNNNNNNNNNNNNNNNNNNNNNNNNNNNNNNNNNNNNNNNNNNNNNNNNNNNNNNNNNNN
NNNNNNNNNNNNNTGTTCTTGTTTTGGTATTCTAGGTTTTCTATGTTGTGTATTTCTTTTTTTGGCCGGGTGTGGCTCTC
AATCAGGAACAGCTGTACATCGTTGTTTCTGATTGAGAGTCATACTTAGGGAGCTTGTTTTCACCTGTCTGTTTGTGGGT
AGTTGTTTTTGCACTGCTTTTAGTTAGCCTGCAAAACTGTTCCTGTCGTTCTTTGTTTTCTTGTTTTTTCATATAAATAA
ATTATGATGAACACGCAACCCGCTGCGCCTTGGTCTTTACATTACGACACCTGTGACAGGTCCCATCTATTGTAGTGGAA
AGAGTATAGCCTGTACATTCCTATTGACATGGGTTTTGAAGGTATAATACTACTGTGCACACATTTTTCTGGCAGTGATC
TCTCAAGAGTGGGCTCGCATACAGTAGCGTAACATCTCACCAATATTATGCAACCAACTTCTATTGCTGTGCTCACACAA
GAGGCTGCTGGGCTATATCCGCTTGTGATGTGTTAGAAGATGACCAAAAGCATCATATAACACAGTTTGAACTGGCCATT
CTCTTTGAATGACTGATAACTTTAAGACATTCATGTATTGGCTGCTGAACATGAGTTGGGTTTGAATTTTGTCAATGCAC
AACCAGTACAGCAGTACGGCAGTGTCCTTACTGTACCCAGTGTTACAAGACATCACACCTCAGCAGCCTCAAGTCTCTCG
CTCTCTCTCCTTCACCCCCCTCTCTCTCTCTATCACTCTCTCTCTCCCTTTACCTCTTTCTCTCACTCTCTCTCTTTCCA
CTCTCTCTCTTTCCCCTCTCTCTCTTTCCCCTCTCTCTCTGTCACACACATATACATTCTCCTCACACAAGATTTGCAGT
TGCTGTACAGAGATAATTTGGTAATAGGGACTGGACTCAGTGTACCATACAACCCTTGGATCTCTCAAAGTCCCACCAAA
CTTTCAAGTCACCATAACATTTTACACATACAGTCAATCAACAAGATATATTACCCCAAACAAAGAATTTGAAGGCTGAA
TGATTGAAGGGGCTCACGTTGCACTCACGTTCACACAGCATTAGTCATGGTTGCGTGTCACAACTCCAAGGGAACTGACT
TGAACATACATTTTCATGGACCATTTTCCTCCACTACAAACTCATTAAATATTGAGTCTCCAAAACAAGCAAATAAGTAC
CCCTTCAGGCTCCTTACTGCACACATTGAATAGATATGGCTCAATACTAGAGGGGCAAAGTTACAGTGTCATGGAGATAC
AGTAGGCTACCGGTGACCATGATCTAAAGACACAGGGAAATGGACTTGCAAGAATGTGATACCAGCAGGCATGCACACCT
CAGCACATTGCANNNNNNNNNNNNNNNNNNNNNNNNNNNNNNNNNNNNNNNNNNNNNNNNNNNNNNNNNNNNNNNNNNNN
NNNNNNNNNNNNNNNNNNNNNNNNNNNNNNNNNNNNNNNNNNNNNNNNNNNNNNNNNNNNNNNNNNNNNNNNNNNNNNNN
NNNNNNNNNNNNNNNNNNNNNNNNNNNNNNNNNNNNNNNNNNNNNNNNNNNNNNNNNNNNNNNNNNNNNNNNNNNNNNNN
NNNNNNNNNNNNNNNNNNNNNNNNNNNNNNNNNNNNNNNNNNNNNNNNNNNNNNNNNNNNNNNNNNNNNNNNNNNNNNNAGT
CTCTCGGCATGCAACAAAAATACCACACACCAGTCAGAAAATAAGGAATACGCTATTCACCATTGGAGTCGATGGTAGCC
AATATTCCACTGCTGTTCAGAAATTGAAATTATAATCCACAACCACCGAAAGCCATTGAAAATGTACCCCCGTTAATAAA
AATATATGAACAAATATATCGCACGCACCGTTATCGAGGTGTGGGGGAAAACACGAGAGAGAGAGAGAGAGAGAGAGAGA
GAGAGAGAGAGAGAGAGAGAGAGAAAGGGGGCGGATGAGTGAGTGCGCGCGTGTGTGCGCTTTCCATTATAGCTATAGCC
TATCTATGCATTGTATGCTCCATGTTTGCTCCTTGAAGTTCAAGAAGCAATGATGGCTAAAATCGCCTTATTGAATACGG

CCCCTTCTACCGCTATCAAACGCGTACGCCACCGCACGCACAGTCACACGCAAACGTGCGCACACACGAACACACTACAC
CGATTGGTGCTGCTGCCGATAACGCGGTAGCCTACATTACAGTTCGCTATAATTCTCTCGTTTTGGTGGTAGGCTATAAA
CTAGACAATTGGGAAGTCATAAACTTATTTTGGGCTGTAGGCCTATGTCTGGTGACATGGTCAATGGGAATATTCTCAAG
GGGTAGGCTAAATAAATGGTTTTCGACAATGACTTCCACCAACTCATCTCAAATGTCAAAACGAACCACTTTTTTTTTTA
TTTGCCTGTAGGCTCTACATTCAGAGCTAAATGGTTGCATCTTTATTCCTGAACTTTTTTGTTCCATTACAAAGATTAAC
GGGCAAAATTATTGATAAGGCTATTGATCATTTTTAGCGACACATTTGAAGTTGCTGGTGTCCTAAAAGGAACACAGTTA
AAAGTAGTTATACATGTGCATTGTATTCGTCCAATGTACACATGCACAAATTGATATGGAGCCTACCTACACATTATCAG
GCTCAAGAATGGCATCACGTGTGCAGTTGGAAGCTGATGATTTCCTCTTGGATCCAATAGTGGTCCATAGAGCCAACATG
GCGTCCTGATCGGCTCAGTTGCTTCACCTGCCTGCACAGTACGGTGTCAAACTTGGATTATGGCTACCACCTGGCGGTAG
ACTCTTGCAGCTGCAGTTGGAGACTGAACAGTAATGCACTTTAATCTTTCCTTTACACCTCCTCCTAGTGGGAAATGTAA
GTAAGTACATGCATTACACATAATTACACCCAAAGCTTTAATGCTGAATATACATTTCCGCCTAACGACCTATGTAAAGG
CCAATAGTGATCTGCTAATTAGAATGACACCATCTATACAGAGTAACATACAGTTACTGCAGTATGCATCACCATAGCCC
TCTAAGATTCAGTAGGCTATCTGAAATGTCACTGCAATGAATCAACTGCTCTTGACATGCCATGGCAAAGAGATTCTTGG
CAACGGAGCCATAAGGACTAGCCAGTCGGTTTGCCTGGCAGAAGGGCTATTGATTAATCACAAAATACCTACATGTGAAT
GGGGAGTGGAGTCTATTGCTATACCAATAGAATAAAATGGAATAGAACAGAAAATAACTCTCTGAAGTGCAATTCATCAT
ATATTATTATTGGCTAGCCTACTGTAAATACAAATAGGCATATACATATTGTAAGTTAAACAAGAAATGGACAGAAAACA
CTCAAAGCACATTGGTAAGATGTGAAGGGCAACTTTTAATTTTTTAAATTTTACATTGGCTGTATCAGCCTAGGTTATAC
ATGTATAAACGTAATTTGACCAAAAGTCCAAAAATACATCAAACCAAACTTGGACTTGACTACAAATAAAAAAAAGAGAA
GACTATAGAGACTCTCTTAGCTTCTTATTCCTCAACTCAAAATCAGATTTACCCCAGTCCTTTGAAGTAATTTTGTATAG
TTCCATCTATAACCTACAGTAACGAACACTTGACGGGTCTATAGACATACTTTACCATAGAAAACATACAGATACATTCA
TTACCATCGAACTGATGGTTCCACAGACCTCTTCCTATTCAACTACAGCCAAATTCACTGAGAATGATCTAAATCCTAAA
CTGGGATGGGAAGGACTGAGTTGGGAGGTTCATTTGACCTGCACTGACATTTAGAAATGTGTCAAACCCTTAGCAATCCA
ACCTGGACTCAGGGGTAGACGTAACATAGTAAACGTAAATCTGTGACACTCCAATTAGATATGTAATAATTTGTGGATGT
CCATTATCCATTTCATATTATATGTTAGGAATTGCAATTTGTACATGTTACGAATTTGCAAAACGTATGATATATTACGA
ATTCCAACTTGTGGCTAACGTTATCTAGGTGACTAACGCTAAAGTTAGCTAGGTGGCTAATGTTAACTACACTAGGGGTT
AAGGTGAGGAGTTAAGTTAAAGGGTTAAGGTTAGGGCTAGGGGATGGGTTAGCTAACATGCTAAGTAGGTGCAAAGTAGA
TGAAAAGTAGTAAGTAGTTGCAAAGTTGCAAATAAACTAAAATGCTAAAGTCCGTGATGAGATTCGAACACGCAACCTTT
GGGTTGCTAGTCATTTGCGTTATATGCCTACCCATCCACTCTGACCAACCACATTACTTTCATTTTTGCCTTAAGTAACC
TTCTGTCTTATCCAACCATACCAAATGTAACATATCATACTAATTTGAGTGTTCCGGATTTACATTTACTATGTTACGTC
TAGTCTATGAGACCAGGCTGAGCAATCATCTATTCACTATTAGAAACCGGGTCATTCGATCCCTGAATGCTGATTGGCTG
AAAGATGTGGTATATCAGACCGTATACCACAGGTATGACACAAAACTACTTGTTTGCTGTTCTAATTACGTTGATAACAA
GTTTATAATAGCAATAAGGCACCTCTGGGGTTTGTGCTATATGGCCAATATACAATGGCTATGGTCTGTATCCAGGCACT
CCGCATTTGGTTGTGCTTAAGAACAGCCCTTAGCCGTGGTATATTTGCCATATACCACACCCGCTCAGGCCTTATTGCTT
AAGTAAGAGATGGGATATTTCACATCATTCAATCTGGGGAATGGGCGACATCTTGTTGTGGTTTTAAAAAAATGACTGAA
AATGGTTTCTGTTGACATGATTATAACAGTATAACTGCCTATTGTGTTATTTACTGTAACTACACCTGTAACACAATCTG
TCTTCTGGGGGATACAACACACACATTTACTCTTATCTGTGTTTTATCCCACAAAAGGCAGAAATAAAGGATCTGTATAA
ATATAAACTCTGAACCTTGCACTATGATATTCTAGAGTTCTATACACAACCTCCTGTTCCACTATGATCTCTGGGGCCCA
GTTCCAAATCAAACACAAACCCATAGCCCTTACCTCTTAGACACTTGGGTACATCTGAAAGGATCGGATATATGAGAAAT
ACGATGAAAGCTCCACCTTTCCCTCTGATAGGCCGGATGAAACATAAGCCATATTGCTCATATCTATCCAATCTTGTCAG
CTCTACAGTGTCCAGACTAGGGCTTAGGTATCAGCATTAAAGTGGGCCAGAGTTAACGCTATCGCTCATCCTCAGTGTAA
ACCTGGAAGATAGGAGCTGACTCCTCTGTCTATGACCTGAAAATGGAAAACAGTACAAATAGTTTCACCTGCACATAATA
CCATAATTTGTTGACTTCTCTTCCCTAAAAGAATGTAAAGCACCCCTCAACAAATTGACAGTCAAATCTGGCTCAACAAG
CGGCCATTTTCCATGGCTATCTGATCAAAGATTTTGATCAGTAATCTTGAAGTGTTTTGATATTTGTTAGAAATGTATTT
TACACTAATTCTGTGTGTGAAAGCCTAAACAACATTGTCGTGACTCTTCAATTTGCAATTGGTGGATGAGGAAGAAAACC
TTACAGAATAGTTATCATACATGTCATACATTTCAATCACCTGATCCCTAGATTCAGGTAAACTTCTCATTCTGCAGATG
CACAATCCAGACAGGCACTATGCAACCACATCGTCAAATCATTCTATAATAATATATTGTGAATATATAAATCTAACTAT
AAAACAAAAGCCCTGTTGCGGCTCCTAATATATTTCTATTACATACTATCATGTAAATCAAGTATGACAAAAGGTAACGT
AACAAATTGCCTATAATCAAATACTGTATATACAATCTTCAAACGTAAACATGATAAATGCAAAGGCATATTCCAAAGGT
ATACAGTACAAATATAGGGGAAACACTAGCAAATAGGGAGATATAGTAACACGTAGTGTGATGATATAGAATGGAGGCGA
TATCGTTTGAGCAACCTGGATTTGCCTTGTGTGGGGTTTTTGTTCTGCTTGTACTTGAAGTTGCCACACATTTCTGATGT
GGCACCTGCACGACAGTCATTCTTTCTCTCTCTCTCCCCAATATCCCCTCTTTGCTGCCAAGCACTCTGGTGTGTTGACC
CCCCCCCCATCTCTCTCTCACTTTCTCTCAGTACACACTCACGTTCCCCCACTCCACAAGGTACTGGACCTTCCCGTCTG
GCGTTACTCGCCTCGCCAAAATCCTCACCCCGTCCCCGCCCCCCCAGCCCCTGGCAGCTGACCCATCCCCCCCGCCCCCT
CCCCCCACGCCGCCCGGATAGGAAGGGACGTCACGCAGCATGAGGGAGGGGCAGGGATCCAACTTCTGGCCAGGCTGGTG
CTGGTGGTGCTGCTGGTGGTGGTGGTTGTGGTTGTTGTGGTTGTGGTGGTGATGGTGGGAGGATGACAGGGAGAAGGGGA
GGCAGGGGGGCGGCACTCGACAAGGGTTTTCCGCACTGAAGAGAGAAAGAGAGGTGAATATTTCACGATTCATACTTTTT
GCCATTGTCATCCAGGCACAGCTATGGTTTCCTCTCTAGATGTCTAGATTGAGGGTAGTCCCCGGAGAGCAGGAGGAGCT
GGGGTTAATACTGACCTATAGTGATGCAGGGTTCTGGAGATGGCTGGGGCTCCGTTATAGGTGGGGTGATACAGGGCAAA
CATCCTCTTAGGTGGAGAGCTGTGGAAAGAGCAGGGATATAATAAAAACAATTTTAAACGGACAGAAAGCAACAATGCAT
GGATTTCCACTGACATACTGCCTAATACAAGCATTTCGCTACACCCGCAATAACATCTGCTAAACACGTGTATGTGACCA
ATAATTTGATTTGATAAGGGTAGCCTAAATCAGTGAGGATGTAATGTAAAAAAAACAGTAACACTCTGTAAATACAGTAC
AATGCATCATTACTTGTTATACAGTATGTAAAGTGTAACCAATACAATAACAGTACCAACCCCAAATTCAGCTCCTCCTC
CTTCCTGGAGTTGTAGAGGTTAGTTCCACCTACGTAGCCATGGCAACATGGCACCAGATCTATTGGGGTGGCTGGGACCG
GGGGCGGACAAGGCTGGACACAGGAGAAACACTGATTAGCTAATGTTCTGACGCCAAATCAGTGGATCCAAACACACATT
AAACATGATACTTCAGTTCATGCCCTTTCATGTTATCTTTATAGAAGAATCCATACTGCTTGTGAATACATCTACATAAA
AACCAAGGTCTGTTAACATACTGTATCATTTGATACATATCATTTAAAAAATGTAATTGCATTTCACTTTTAAAATGGCC
GGACGCTGGAACTTTGTTAGTACAAAGGGCTAGTCTTTCCAAAAGGAAATATTTTCACGTGACAGCTTTAAACCTGCATT
ACAATTCCACATCACACATTTGTATTGTATGCCGCGAACACACTGCTGCAGTAGCTTTGAAATTATTTACAGTATGGTAT
TGCTATTGTATCACGATTCACTTAATTATCATCACTTAATATGTCTCATCAAGGCATAGCATGATCCATGTCCTGCATGA

TCAAAAGTATGTGGACACCTGCTCATCGAACATCTCAGTCCAAAATCATGGGCATTAATATGGAGTTGGTCCTCCCTTTG
CTGCTATAACAGCCTCCACTCTTCTGGGAAGGCTTTCCTCTAGATGTTGGAACATTGCTACGGAGACTTGCATTAGTGAG
GTCGGGCAATGGTGTTGGGCAATTAGGCCTGGCTCTGTGCAGGCCAGTCAAGTTCTTCCACACCGATCTCGACAAACCAT
TTCTGTATGGACTTCGCTTTGTGCATGGGGGCATTGCACATGCTGAAAGAGGAAAGGGCCTTGCCCAAACTGTTGCCACA
AAGTTGGAAGCACAGAATAGTCTAGAATGTCATCGTATGCGCGTTAAGTTTTCCATTCACTGGAAAAAAATGTCTTGAAC
CATGAAAACAGTCCCAGACCATTATTCCTCCTCCACCAAACTTTACAGTTGTCATTATGCATTTGGGCAGGTAGCGTTCT
CCTGGCATCTGCCAAACCCAGATTCGTCCGGCATACAGCCAGATGGTGAAGCGCGATTCATTACTCCAGAGAACGCGTTT
CCACTGCTCCAGAGTCAAATGGCAGCAAGCTTTGTAACACTCCATCGGACGCTTGGCATTGCGAATGGTGATCTGCTCGG
ACATGGAAACCCATTTCATGAAACTCCTGATGAACAGTTCTTGTGCCAACGCTGCTTCCAGAGGCAGTTTGGAACTCGGT
AGTGAGTGTTGCAACCGAGGCCAGACAATTTTTACGTGCTACAAGCTTCAGCACTCGGAGGTCCGGTTCTGTGAGCTTGT
GTGGCCTACCACTTCGCGGCTGAGCCGTTGTTGCTCCTAGATGTTTCCACTTCACAGTAACAGCACTTTCAGTTGACCAG
GGCTGCTCTAGTAGGGCAGAAATTTGACAAACTGACTTGTTGGAAAGGTGGCATCCTGTGACGGTGCCACAATGAAAGTC
ACTGAGCTCTTAAGTAAGGCCATTCCACTGTCAATGTTTGTCTATAGAGATTTCATGGCTGTGTGATGCTCAGCAACAGG
TGTGGCTGAAATAGCTGTGTCCACATACTTTTGTATATACATAGTGTATATCTGTCACCTCCATTGCCAATGATAAGTAG
ATTCATATTGTGGAGACTGAGGTAGCATTTCTTACAGTGTGAGTTTGCTACCATAACAACAAAAGCATCAGTACTCTATC
CCATCTATCCTTTGCTTCCTCAGTTCTCAGCACACCCTCAGGTCTGTCAACCTCCAATGCTGGACTGTGACCCACAACAT
ATACAGTAATTACAGCAGTGGTATACTAGCATTATCCCCTCCCCTTACTAGCGCGCTGTCCCTTTAAATCGAGGTGCTCT
GTCAATGCTGCACTGTCAAAGTGCCAGCGCCACCTGGCCAAACTGTAATCGACTGCAAAGTGCACTTGGAAGCGCTCCCA
GATGCCGCATATCAGCGTACCTTCCCCTCTCACTTTCCCTGGGATAACCAACCTGCCCCAAGATGGCAGATGATTCATGT
CCGCTCAGGATACAACAGACTGCTGGAAGTGCTGCCCGCTGAAATACGACCGATCTATAACATTTATTGTCTCAGGATAA
GGGAGCCAGCCTGAGCAGTAGAATAAAAGGATGTGTATTTTCTGTTCATATTAAAATATATATTTCTAAACCCGTGACCA
TAACATGTTGTCATATTCGATCGAGTCATAAACGGTAAACCAGCTTCAATTCAATAGTGTATGAGGGGTAGGCTGGGCTT
TGAAGAACATGTAAACAGACAACACTGGCTATAAGGAGAACCTTTTCTCCACACACTGACTGACTGTGACTGACAAGTCA
TTCCAATGCCAGGCAGCTCTTTGCTATGCAGTGAGGGGGTGGCTGATGGCTGATCTCTCACTTGATGCCTGCTTCGAGAC
AGAAGCACTGACCTGTGTCTCCATGATGCGCCGCTTTGATTTGCGTGACTCTGGCCCCACTGCTCTCCTCTGGCAGGGCA
GTGACAATGGGCTGCTCCACACAACAACACAGAGACACAGTCAATAACTTGAGGAGTGGCGCAATGTTTTTACTTAGAAA
ACTACAGTACCAGTCAAAAGTTTGGACACACCTACTCATTACAGGGTTTTTCTTTATTTTTACTATTTTCTACATTGTAG
AATAATAGTGAAGACATCAAAACTATGAAATAACACATATGGAATCATGTAGTAATCAAAAAAGTGTTAAACAAATCAAA
ATATATTTTATATTTGAGATTCTTCAAAGTAGCAGCCCTTTGCATGAGACGCCGAGTAGGTGAATGGATGACCCTTGCAT
GTGTGGTTCCCACCGTGAAGCATGGAGGAGGAGGTGTGATGGTGCTTTTCTGGTGACACTGTCAGTGATTTATTTAGAAT
TCAAGGCACACTTAACCAGCATGGCAACCACAGCATTCTGCAGCGATATGCCACCCCATCTGGTTTGCGCTTAGTGGGAC
TATCATTTGTTTTTCAACAGGACAATGACCCAACACATCTCCAGGCTGTGTAAGGGCTATTTGACCAAGAAGGAGAGTGA
TGGAGTGCTGCATAAAATGACCTGGCCTCCACAATCACCTGACCTCAACCCAATTGAGATGGTTTGGGATGAGTTGGACC
GCAGAGTGAAGGAAAAGCAGCCAACAAGTGCTCAGCATATGTGGGAACACCTTCAAGGCTGTTGGAAAAGCATTCCAGGT
GAAGCTGGTTGAGAGAATGCCAAGAGTGTGCAAAGCTGCCAACAAGGCAAAGGATGGCTACTTTGAAGAATCTCAAATAT
AAAATATATTTTGATTTGTTTAACACTTTTTTTGGTTACTACACGATTCCATATGTGTTATTTCATAGTTTGAGGTCTTC
CATATTATTTCACAATGTAGAAAATAGTAAAAATAAAGAAAAACACTGGAATGAGGAAGTGTGTCCGACCTTTTGACTGG
TACTATATGTCAGGGTATAAATGTGGTGTTTTTTATTTTAAGCCTATAGAGAATAAAAGAGAAGATGGTGTTGAGAGTGT
TTAGCAAGAGAATGAATATCCACATGCGAATGTGCACACACACATATGCACACACACTATAAATTATGTATTTATTCATA
AAAATCTTCGTTATAAACATATTCACCAGAGTGAAATAAGTGAGTCCTTCCCTTAAATGATTTAATGAGGCACACAGGTG
CCTGTTTAGTTAGGAACAGTTGTTCCTATCCAGCTGGAAGGACCACCGTCTACAAGGTGTTGCTCTTTTTCACTATTGTA
AATGTGTAATTAATTACTAGAATGAATGTATCCATATAAAGAGTTGGGTCCTGAGGCATGTGTTTGTGATGATGAGAGAA
TAATAATCACAGAAGTTGTCCTTACTCTGACCTTCTCCTTTGGGTGATGTTGATGGGCGGGTCAGTGATGAGGGGGGACG
AATCAGAGGTCAGGGGAGGCGGAGCTCGGACCTGGAGCCCAAAAAGACACTTCTTCTTCTTGATCTCTTTACCAGAGACA
AACCTGGATGACGTCACAGAGAAAAAGACTTTCAAAACTTGTAGACAACAACTTTGTAAAGTCAGGTCATCGTTCTTTAT
TGTCTATGTTTTCTTATCGTTCTCTATTGATAATATCAAAGAGTTAAGAAACAATTATCTAACTGTATTTTCTTTACCCT
TATGTACAATTCCAGAAGCAACGCTATCATAAATCTGGTCAAGCATAGCTGTGGCTTCTATTTATTCTTCGCATCATATG
CAGACTCTCACCTGTCTTTGTTAGAGTTCAAAGCGTTGAGCAGATTGTGACAGCGATCTTGCCTTGGTGTGTCAGAGAGC
TGGGGGACAGGGAGGAATGAAAATCAGGAGTTTGATTAACACTGTGGATGGTAGCCTGAAACAAATGTTAACACATTTAG
GAGACACATTGACATTCATTGGTGAAAGGTAAACATTTCCATGGATAACGTAGTTAGTTACTAATGTGATTTGTGAACAG
TTCATTAAAAAAAGTGTATGACTGATTTAAATGCAACTGTAGTACCTGAGAGCCACATGAAATACAAATGATATTTACCA
TTTCAAATGTGCTAAATAACAATGACAAGTAGCTAATGAAATCAAAGCACATAATTACCACATGACTGCCTAAAATTACG
CTAGCTAACACTTTCACATCGTTTCATCATAGCACGTAAGTGAGGAAATATTTATACCGAGCCTAGAAGCAAAGAGTCCC
AATTCTCATTGGCAAACGACATGATTTCATGGTCGAAATCAAAGTATTTTCTCTTGCAGCACAAGGAGAGGTGGTAGAGT
ACCAAATGGGCCAAATCTACCCTGTATTTACAATAAACACACCAGGAAAAGACAGTTAGTTCAGAATTTTTCACATCACA
AAGCTTTTCATTACATCATTGAGTAAGAGGAATTAACATATTGTAACCCACATGACTCAATAAACCTGTCATAAGAAACT
GGTTTTCAAGGGTGTTTCAGTGTGATAATCCCTGCAGTTTAAATTATGTAATCTTCTTTTAGCAGATACTGTATAGCTTT
ACCAGCTCATGGGTAGCCGTTGAATTGTCTCTGGTCCGTTTGCACAGACTGAGCACTGGAACTCATAAAACCTTTCAGGA
AAAAAAAAGGAAACACAGATGATTCTATTTTTGTCTGTGATTGACCAGATATAAAAGTCAATGGAAACATGACATAGCAC
ATGGTTGCTACTGTCAGCAAATTCTTCCCAGTTTCCATCACTTGTTTAGCAGACGTAACAACCCACCACATTCACCTCAA
ACATTATTTTTCTGTGCAGAAACAAAGAGTAATATAATATATCATGGGCATTAAACCAGTAGGATAATACAGATGGTTGA
TGTGACAATAATGGTGTAGCGGACTGGACTAATTTTAATTCATTTTTACCTCTACCACGTTACATTGGACACCAGAGAGG
CAGAATAACAACTGCACTGCTATTAGCAATAAACACTAACAGCATTAGCCCTACCACTGCCAGTTGTATAACCTCTCTGT
TATGTTTTGGACAATGGTAACGCAAAGAAGCAGTACAAGGTTATAGATAACAATACTCTGTCTCACCTGTCCCCATATAA
CAACGGCTTGGTCAGACACTGTGTGCAAGCTTCATGAAACCACTGACCACAACTTCCACACTGCAACATTTTCAAGTTCC
ACCTGCAGTTGATTAATAAAAACATTAGCCACACTCCCCAATGGCCTAAACATAGCTTACATCTGGTGGCTACCTGACAA
GAGTACACTATATTATGTATTTTTCAAGAGGCTGCAATACTCAACTGAAAAAACACATTATGTCTTGACAGTAGAGCTAT
ATAATGGTGAAAAGGACATANNNNNNNNNNNNNNNNNNNNNNNNNNNNNNNNNNNNNNNNNNNNNNNNNNNNNNNNNNNNNNNNN
NNNNNNNNNNNNNNNNNNNNNNNNNNNNNNNNNNNNNNNNNNNNNNNNNNNTTGGAGGATATATTAGCATGGGTGTTGTTA

GAACCGAGACGAAGTCGAGGGCCGGCAAACCATGAAAATATATCCTCCAAACACTGGCTTTGAGGGCATTATCACTTTAA
TACAACTAGTTAACAACATATTCAAATAATGATTGACATATTTTCATTATAAACGTTATTTTGATTCATTTATTCATACT
ATTTCATCCTTCCACAAGATATAGTCCCGACACAAATCTAGGGTTGATACCCAAGCTGGCTGGTCGTTCATTCTATCGGT
TTGGTTGCCAGAGAAGCGACCCAGTCGTTCAGTCTTTTTGTTCTGTATCTATGGATGCGACCCAGTCGTTGGTTCTAAAT
GTTCCATTGAAATACTGACTTGCAACATTGTTATTGTTGCTTGCTAGCTAGCTAACTACGGCTAACTTACAGTCATGTCA
AACAGTGCAGCCAGAATAAGAGCAAAGCAGCTGCATTTGCGTTTGTTTAAGCTGTTTTCTAGTGACATTTATTTGGATAC
ATCCATAACAATGAGCTACTGATGCGAGATTTCGCCTGGTATAGAATATGTGTTCTCTCTTTAGGACACTGTTGTTCAGA
GGAGCTAGCCAACAACACAGCTAACACAATCACTTCAAACTGAAGCTGGAAATACTGCAAACTAGCTGCACTTTCTTTCG
TTTTACCTGTTTTCTAATTTCTATTGATTTCTATTGATATATATATATATATATATATATATATATATATAAATTATGCTGA
TTCATAATTTCAACTGGCTGAGAAAAGCTGCCTTGCATGTCTGTCTCATCCCGACTCCCAACATGTTTGTTACTATGGGA
CAACTGGAGATAGAATTTGAATGTTGAAACAATGTTGCAAATGTCAGAGAGACAGACACCAAGGTTCATACAAATCTCCG
CTGTTGAAAACTAAATGTTAGTCTAAAAGAAATGTGAGATAATGCCTAGATGCTTTTTATAGTGGAGATCAGTGGATTGT
GCAGTCAGATGGAACAGAGTAAATAGGCATTTTAACATCATAGACTTAGCCGGTGGTAACTTGTAGAATAGACACCATCT
GGAATGCGGTTTTAACCAATCAGCATTCATGATTAGACCCACCCGTTGTATAACATAATTTATCTACTGAGGGCATCTGT
TAGAAGCGATAAATTGTCACTCACTCTCCAGGTCCGGCACAGTAGCAGTAACACTGCTGCTGGTTGGTCAGATGCTGTGG
GTCCCAGTCTAAAGATGACAGCTGATAGGGCAGCCGAAGTTTCATGATTTGCATGAGCCGGGCAAACCGTCCTCGTTTGA
TGGCTCCCCCTCTCTGTAAGACAATTTTGACAAATCGATTCACTACAGCGACCTCTCCACTCACGGTCATGTCAGTACAT
TCAAGATAGATGACATTGGATGGGAACTATCCAGAAAAAGCAATTCATCTCATTTTATTGTAGGTCAGCAGAGTGAGATA
ATATCTTAAGAAAAGTCAGGTCAATGAGATTGACATTGACCGATGAGCAATTAGCCCTTAACCACAGTGCTCCCTCATCT
AACACCTACAGTACAGAATTGGAGCATAAATGAATTACTGACCATAATGACTATAATACAGTATAATCTTGAAAAAATCT
GCATTGACGAACTGGTCAGATGTCCAGGCACAATGTGGAGAAGTATCTAATCCCTATGGTGAATAATACAGAGATGTAAC
ATTTCTGTGGTTTCCTTCAGATATATCCATTGACAACATTGTTTAACACAACACTGGTATTGAAATCCTCTGGATAGATT
TAAAAAAAAATGACAAGTCTGAACTGGATATTTTCCCTGAGCCAAGAGGCAGCGTGTGAAACAAAGATTAGCAGTCTAAC
CTTGGTTGCCACTGCAAAGACACATTGCCGACATATCCAAGAATTGCTGTCAGCTTCCGGCTCAATGGACGGAGTGTGGC
ACTCAGGATGATAACCTGTTATGGGATGGAGAGAGTGTTTACTGTAATTCACATACTGAGGATATGCTACAGATGGAGGC
CTTTTCCTTGTCTCTTTTCATGAAATATTATACACTGAGTGTACAAAACATTATGAACTGCAACGCTTCTGGGTTTTTCA
CGCGCAACAGTTTCCTGTGTGTATCAAGAATGGTCCACCACCCAAAGGACATCCAGCCAACTTGACACAACTGTGGGAAG
TATTGGAGTCAACATGGGCCAGCATCTATGTGGGACGCTTTCGACACCTTGTAGAGTCCATTTCCCCAGACAACTGAGGC
TATTCTGAGGGCAAAAGGGTTGCAACTCAATATTAGGAAGGTGTCCTAATGTTTGGTACACTCAGTGTATATTATNNNNN
NNNNNNNNNNNNNNNNNNNNNNNNNNNNNNNNNNNNNNNNNNNNNNNNNNNNNNNNNNNNNNNNNNNNNNNNNNNNNNNN
NNNNNNNNNNNNNNNNNNNNNNNNNNNNNNNNNNNNNNNNNNNNNNNNNNNNNNNNNNNNNNNNNNNNNNNNNNNNNNNN
NNNNNNNNNNNNNNNNNNNNNNNNNNNNNNNNNNNNNNNNNNNNNNNNNNNNNNNNNNNNNNNNNNNNNNNNNNNNNNNN
NNNNNNNNNNNNNNNNNNNNNNNNNNNNNNNNNNNNNNNNNNNNNNNNNNNNNNNNNNNNNNNNNNNNNNNNNNNNNNNN
NNNNNNNNNNNNNNNNNNNNNNNNNNNNNNNNNNNNNNNNNNNNNNNNNNNNNNNNNNNNNNNNNNNNNNNNNNNNNNNN
NNNNNNNNNNNNNNNNNNNNNNNNNNNNNNNNNNNNNNNNNNNNNNNNNNNNNNNNNNNNNNNNNNNNNNNNNNNNNNNN
NNNNNNNNNNNNNNNNNNNNNNNNNNNNNNNNNNNNNNNNNNNNNNNNNNNNNNNNNNNNNNNNNNNNNNNNNNNNNNNN
NNNNNNNNNNNNNNNNNNNNNNNNNNNNNNNNNNNNNNNNNNNNNNNNNNNNNNNNNNNNNNNNNNNNNNNNNNNNNNNN
NNNNNNNNNNNNNNNNNNNNNNNNNNNNNNNNNNNNNNNNNNNNNNNNNNNNNNNNNNNNNNNNNNNGTCTGTCCTCCGC
GACGTCGTTTCGTCATGTTCCCGTGCCTTTGCTCTCTTTTCTCCACCTATTGATGCTATTAAGCGTGTTTGTCAGAGCAG
CACGCGAATCAACCCGCGAGAGACGCCATCATCCCTGCCTCTTTCTCGACCGACTTGTTGTGACGCAGACATAAAACACC
GATAGAGTGGGAGGGAGATGGGGGGACCGGGTCAACGGCGGCGGGAACACCTTAACATCCGAAGAAATTGCCTCTAAACC
GTTATGACAAGGCAAGCTGTGTTTTCTGAAGAATTAACTAAATTGTGATAAAAGCCAGTTAAAAATCAGCCGACGACGTA
GTCGCTGGAGTTTAATCGGGCAATTAGTGGAAGTTTACATACCTCATTAATTGGTACTTCCCAAAGAAGGGTGCTGCAAG
TTTGGAGTCCTGGAGAAAGTTTTAGAAGGGCCAGTGGAAGCTGCGCTCACACGTGTTATTCACTTTTCTTTTTTCTTACC
ATTCAGTAAATTGCACAATAAATAGTTTCCTTGCTATAGTAAGCATTTTTATTTCCTTCCAGCTAAAATAACACTATAAT
CATAATGTAATCATAAATTATCTAATTTCTTAATATTACTACACATTTTGATTATATAGCCTCGGCTGTAATATACAATA
TGTATAATAAACATTGTGATTGTTTATATAGAGTTACTTTGTGCAGTCTTTGTGGTCCCCTTGCCAGATAAGCCTATTTT
CAGGTTTTGGACACTATACAAAGTTAGCCTATTAGGTCTAAGTAATTAAGGATCATTTAAGGCGTGGATTACTTTGAGCA
CTTGTCACTGTAGTCATCAAAGGGAATTGTTGAATGTGATTTACAATAGTCTATAGTTGTCCTATACATTTCCTATTAAT
ATACATATTGTGTGTGAAATATTGATTATCTATGATCAATCGTCTATTCTTTACGGCTTGATAATATATACCTACGCTCA
ATAATGTCATCATCACTTGTCACTGCTCACTATCACCATGAATATCACTGGTCACACAAGCTTTCAATTCTCACGACAAT
TGGGTGATAACATAACATGGTAATAACCAATATCAGAATGAGTAACAATTGTACTTATTTACCATTGGTTAATTATGAGC
CCTAGAAATTGCACACATGTTCATGTATATCATTTATGAACGGCAGATTAAAGGGCATTTAAAAGTGTCATACCATAACT
GAGCACTGACAATTCTATGAGTAGGCAACATAGCCTATAATTTAATAGTTAAAACAACAAGCGATGAGGTGTTTCATATG
TTATTTTTTTATCTTTAGATTTTATGTATTTGTTCATTTGCTAGTTTTGTCATATGAATGTATCAAGCATATGGTAATAT
TGCGCTCAAAATGGCCACTGGCATTTCTAAAGGATGACAATGAGTTTCTCATTGGCTACACTAGATGGCGAGCACACTAC
ACCTTCTAATGCGCTGCTTTGTCAGTATTGGAGTGTAGTGGTAGGCGAATAAAAATGCAGTGGTGGCATTTAGCTGAACT
ACAGTTCAGTTCACATAACTGTGTGATGACAGAGATATTTTCAAATGTATCATTAGCATTTGGATGAGGATCACTTTTAA
TAATGGCAAAACGGAATTATGCTTCTAGCAGTCTCACAGCAAGATTCACTGTTTACCGAGTAACGATAGTGAGCCCGTTA
GCCCAACTTAAGTATCATCACAGAAGAGAAAAAGGACCCCTATTCATGTTAATACGGGGCTTGTGCAGAGGTCATAAGGG
TGACAACTTGATGCCTTTCATGTGCTCTCCCTTTTGGAATAAAGATGCCTCTCACACACACAGCCGGGGCTTTGTGAGCG
TTTCTGTCATGAGACAGAGAGGAGGGGAAGCAGACAGTCATGAGCTTCTGATAAAAACTAGGCAACGCTCAGTGTGGCTA
GGGTCTACAGAAGGGTTTAATTAAGAATATTAGAGTAAGCTTACAGAGAAAGAAGGAAACAAGCACCATTATTAAGATAA
TGTGCTACTACATCCAGGGGCGTCATGGACAATGTTCCCTCTAATTGAGCACTGAGCAAATTTCAGGTCTGATGAGCACA
AACTTGAACATTGTGAACATTTACTATGAGCCCTGAGGCTGTACCCGCTTGAAGTTACAGTTTTAACAGTGGCCAAGTAG
GCTACTGTGGCTATTTGAGTGGCCTACCATCAAAAACAACGAAGAAAAAGCATCCCATAACATATTAACATGGAAATAGC
TGTTTAATCATTCAGCCTATAGTAGCAGCCAATGTGTGCAGTTCAGTGTAGGCCTACATTCCATGAGACTTCTGAAAAAA
AACATACAGGGCTTGACATTAACCTGTTTATCCACTTGTCATTCAGACAAGGAGGTGACTGAAAATGTTGTTGTATTGTT
TGATGCAAGAAACCACTTTACAAAATAAAATGCATTATTATTCCCATACCGTTATTACAGAGAATCAGACAAATTATGCT

ATCTTCTGCCTATTGGCAACTTAGCTTATTCAAGCTTATACAACACTGTCCCTTTAAGACTAAAAAATGCTGTTTGCCTG
ACTAGCTTTTCAAAGAAGTCTAGAATTTTACACGTTTCGTGCTCTTGTAGGAAGCAATCACTCCCCTATTGCTGACTACA
AATGATCTATAACTGTGCGAACTCACTAACTAGCAAAGGATATTAACAAAATGTGCTCACATGGCTACTTGCAACTCTCG
CCTTGATCTCAAAACAAGCACATCTACTCACGATCGCTCATGCTGTAAACACAGTTCAGTTCAAAGTAAATGGCACAGAT
CCATATATGGAATTGTCTATTTGCACATAGGCCTACTGCAGCTCTGATTGGTTATGCCGCACAGGTCTGTTTAGAGTACG
GGCTGAGTCATACGTGTCAATGTAATAGAATCCTACTCTGATGCGTTCTGCCTACAACAAAATCTCTTGCATAGTTAGTT
TTACTATGTTTCTTTTTGTTTTGGTATGTTGCATTGAAAGTGGCTAATATTGCATTGATTCAATCACCAATGCCACAGTA
AAGGGACATATTAATAGTGTTAACTAACAGGGAAAACCCTTCTCTCCATGGGCTCATATTTCTTCTGCCTGCGTGGCAGT
CTCAGGGATACTGCAAGCCTGCACACACGCAGCTTAGAGGGAACTTTGGTCATGAGGGAACTTTGGTTATGCACCCATTA
TTATAGAGGGGGTTGTCCCCCCCCCCATGAGTTGGAGCATTTTGCATTTTTCAAACACCTGAAAATGTTTTTCCTGCAAT
CTAGACTATGTTAAAAAAAAAAAAAAATTCTGAATAGGTTATCTAAGCATACCTCTTTATCTATTCAATTGCTATTTTAAT
GAACTATGCACATTGATTCTTCAAGACCTTTTATACCTTAGGAGTTTAGTACAACTACCACACTGGTATATAGTATCGTA
GCCCTAAATATGCTTCTCTGCATCTCCACTAAAATCTGGGTAAAAGATTGAAAGGAATTTGAGTCATTAGTAATTGCCTG
CTTTTCTAAAGTCTAGCAACCTTGCTAGCAGGCATGCCAGCTAAGATAGCTAGACAAGCTTCTCTGACTTGATTAATAGC
CTGAAATGGCTTGGTAGCTACCCATGCATACCCCACAAGACATGCCACCAGAGATCTCTTCACAGTCCCCAAATCAAGAA
CAGACTATGGGAGGCGCACAGTACGACAAAGAGCCATGACTACATGGAACTCTATTCCATATCAAGTAACTCATGCAAGC
AGTAAAATGTAATTAAAAAAACTGATAGAAATACACCTTATGGAACAGTGGGGACTGTGAAGAAACACAAACACTGGCAC
AGGCACACATACACATGATAAGATATGCACTCTACACACGGATGTTGAATTGTAAATATGTGATAGTGGAGTAGTCGCCT
GAGGGAACACACTAAATGTATTCGGTAAACTGTTATGAATTATGCAATATTTTAAATTGTATATAAATGCCTTAATGTTG
CTTAACGAACAGTTCTTGTGCTGACGTTGCTTCCAGAGGCAGTTTGGAACTCGGTAGTGGGTGTTGCAACCGAGGACAGA
CAATTTTTACGCGCTACGCGCTTCAGTACTCAGACGTCCCGTTCTGTGAGCTTGTGTGGCCTACCGCTTTGTGGCTGAGC
TGTTGTTGCTCCTAGATTTTTCCAATTCACAACCGGGGCAGCTCTAGCAGGGCAGAAATTTGACAAACTGACTTGTTGGA
AAGGTGGCATCCTATGACGGTGCCACGTTGAAAAACACTGATCTCTTCAGTATGGGCCATTCTATTGCCACTGTTTGTCT
ATGGAGATTGCATGGCGGTGTGCTCGATTTTATACACCTGTCAGCAACGGGTGTGGCCGAAATAGCCGAATCCACTCATT
TGAAGTGGAGTTAACATTCTTTTGGCCATGTAGTGTATCTAGCTGGCTAGCGAAAGCCAACTTCATAAAATTGCTAAGTG
GCTAGTATTACAGAGAAATAAAACATTTTTGCTTTATTTATTCATCAAGAAGGAATTAATTTTTCAAACATTCCTCCTAC
GCGTCCATCCCAGATAAAGTCAAAGGAAACGCTACGTGTGTTACTCTACACTCTCTCTCTCCTCCTCCACTGACGATACT
GACTACACACACACTAGAGCAGGGTTCCTTAACAGGACGAATTTGGACCGTGAGTGATTTTATTTGTCCCCCCCCCCCAA
GTTTTCAAACCAAAAAATAAAAACCTTTCTTTGCTTTTTTTGGGGGGGGGGGGGCATAATAGACTGTAAAACACTGGCAA
ATCAGCTCCAAGTGATTTTAATTTTGGAAATCTGTTCAAAGGTATTACCATGCATAATGGAGAGATATATGTGATGGCAT
GCAAATGTAAGCAAGGTTTTAAATTAAAATGTTTTAGTCAAATATAATATCTATTTGGGCTTCTTACGGTCAATTTGCAG
TCTAGATTATATTTTTAATTGTGTTCTGGCTCCCTGACCATCCGCTCAAGAAAAAATCGTCCCGCGGCTGAATCTAGTTG
ATGATCGCTGAACTAGAGTATCCAGCTTTCTGCTTAGCATACCTTTACTCTGTGGTGCACCTTGGAAGGAACCTTTTTTT
ACATGAAAAAAAAAAGAGGACAAATCTGAAGGGGCACATACCCTTGTGGCCCCTATGGGCATGACACACTGTAATGCCTTC
CAGCTGTGTTTGGGTCAATTTCCTTTTGGAAACGTTTTCCCCTACTGTCAGCATTGAAAAAAAAAATGTGGCAGATAGCCA
AAAGAAAATGTAAAAAAAAATCCAGCAGATAAAATTTTAGGTTATATCACGTGTGTGTGTGTGTGTGTGTGTGTGTGTGTG
TGCTTTCCATAGGGAATTATTGGTTTTGTTCTTAAAATAAACCCTAATTTTAGGAGCCCATAGATGACTTGACATTTTTC
ATATTGTACACCAACACACAGTCCAAATAGACTATATTTCCGAGACGACCACTTTTCCCCCCTCTCTCAGCCAGTGAGAC
AAGTGTAGGCTATGTTGCTAAATGCGCTGGGCACACGTCTACAGGAACTGCGCTGTCGGAGCACAGAGAGCATTGAGGGA
GTGTGCGGCACACGAATGTGCTCAGTGCTGCTATCAGCACTAGAAGCTTGACAGGCGACCAGAGGGGGACACAGATACTG
ATAGAGTGGGTGCGAGACTCTCTTCCGACGTCGGTGCTGGAACGGATAGGCGCGACATTCGTCCACTAACCGGTAACCGT
ATATTACACGGCGGCAGACACTACAGCTATCCAATGCTGGCGGAATTTAGCTGACGGTAGTTGGACACGCAACATCCAGT
AAGTTCGTTCATACGATGTAGCCATGAATGACATTTTGTAATTTCATCATCTCTTTAATTGCATTGAATATATATGGCCT
TCTACAAATAGGAATAAACCTTATGTACTTGGTTACTAAAGAAACAAATGCTTTACACGCAAACTTTATCATCATATTCC
GCTTAACCCCTATAGTGTCTTGCTTCCTATGATTGAAGGTGAACATAATTTGGTTGAGAAGTCAATACAGCACCACCCAT
CGGTTGGTTGAATTGAAAGGGAACATATGCAATTGGGGTGTGACCATCTGTAGCCTAGTTACAGTGTGGACTCACTCATG
CAGACCAACATGTAATGTTTGCCTTCCTCAAAAAGGCAGCAATGTCATGAATGGAAGTTATTCAGACCGCTGAATAACCG
TTGGTTGGTTATGATGACAAACTAACAGTCAGTATATAGACACATTTTACAGATATGTTTTCCTTTTACCAATATATATA
TTTTTTAATAACGCTAGGCTTAATTATATGTAGGCCCTCAAGACTGAAATACACTTAAAAGTTTAGTACCATGTTCCCAA
AGATTATGTGATTGAGTTGATTAGTGTCCCATCTAAGCCTATTGTACAACAGTGCATGTAGGGTAGAAAAGTGCATTTCA
TTTCACAGACAAAAAGCTATGTATAACAGGTAGCCTAAATCAAATCAAATCAAATCAAATTGTATTTGTCACATGCGCGG
AATACAACAGATGTAGACCTTACAGTGAAATGCTTACTTACAAGCCCTTAACCAACAATGCAGTTTTGAGAAAAATACCT
AAAAGAATAAGAAATAAAGTAACAAATAATTAAAGAGCAGCAGTAAAATAAAATAACAATAGCAAACCCTTAATTATACA
GATAAAGATGAAACATAAAATGTTTGTTCCACCTATTTAAACACCTTTAGCATCATTACTGAAGTAATGACATGAACCAT
ACAAGAACAGGAAGTCTTCTGACATATCCATATGCTGTACATTCATATACAGTAGCCTCCACCACACTTGCTTGGCAAAA
AGAATTCTGAAACAGTTTCATTAATATGAATCAGCAATGCATCATCACATGAATATCTGCTGTCTCTAAATATATATAGT
GGTACATGTCAACATCCTCCTCCTGCCCTACAGTGTAACAGTGTTTATTAGATTTCCATCATCATGGCAATCCACATGTC
AGGGAGGGAGAAGGGAAAATGCAGGAAGAGCTGGCATTCAAATTCAATGCTGGAGCAGGACACAGTGAAATGTAAATCAT
CATGTAAACAAATGAACTGATACCACCACAGCTTTGGTACACAATTATTTTTATTGCACACTATATATAAACCATTGTGT
TTATTTCTTTATGCCTTTCCGAGTTGGTTAAAGTCCATGAACTATAAAATGATGTTGGTCCTGTGCCATTGACTCTCATT
TTCTGAGTAGAAAATTGAAATTATAACCAATTAACCCCATAAGTGCCTATTGCTTATTTTGTGTCTCTAGCCGTCGGCAC
CATGATCCCTCGTAGTATGCGAACAGGTGGGATGGACCTGCCAGGGGTGGAGTCAAGGGATGTACCCCTCATTGGCTACC
GGCCTTTAGCGCCTGACGATGTTCGCCTCAGCAGCCAATCAGGACAAGTGACTGAGAGTGCTGGACTGGACTCCTCTGAG
GTTGGAAGTGATTTTATATTTAATGAACTTGATTAGGAACATGTACTGTCAGTAACTCATTCACAGCAATTAACTGCAGT
AGTAGTTTCATTGTGATGATGGATACATCAAGGTGCAGTTGCACTTTAATATCTGTAAAGTTATGGAGGGCACTGTTTCC
TGATCTCTCATATAGCAAATTTTCTCCAGTGGTCTCATATTAAATCAATTCACCTATGCGGAATCCAATAAATGGCTGAT
TTGATACAGAAACTGGGTCCTGGGGCCCCCCTGGGTGCACGTTTTGGTTTTTGACCTAACACTACATAGCTGATTCAAAT
AACTAACTCACCTTCAAGCTTTAATTATATGAATCAGCTGTGCAGTGCTAGGGAAAAAAAACAACTATTAGAATCATTCT
ACCATCTAAACCGCTGTGAAAAATCTTTTCAATAACCAAAAATATTGTATTTTCAGCTGTTTGAATCTGACTACAAAACC

GAAAGTAAAAGTCTTAAAAATGAAACTTAAGACCGGGAAGCATAGAAATAGCACACATAGAACATCTCTACGGCATTTTA
GACTTGCTTTCAATGAGAATAACAGATCTATAACTTACATTTCTCTGTGAATTTGGTCGGTCACCCAAAAAGTTACATAT
TGCAGCTTTAAGTCAGGTTGTCCTCTTAGTCTTGATCACATTCCTCAGACCAACCAGGGTAGGGGGTTAGGGTATTAGAG
CAGTGGTCTGTGAAACCGTGGTAACAGGGCTCCCCTACCAGTTCCTTGAGTAAACAGCAGATGCGGTCGGTTGGTTTTCA
AAGAACCTCAGGGATTTCAGTCATTTTGATTTCAAAGACAGATACTAAAGAGGAGGTACTTTCCATCAAGACATGCCAGT
GAATGAGATGATGTTTGACCACCAAGCATTGAATAAATGCTCATGTCTTGAGATTTCCAAGTAGACTACTGTAACATTTT
TGCTAAGATGTTGCTTTCATGAGCATTACTTTTATGACCATATTACATGTATTTTCTAATATAATTCACTATTTATTTAT
ATCCAGCTGAATTCCCCTGAAACAGAAAGGGTTGTGTTGGTTGTTTTGAAAGTCTGCAAACCTGGACAGGTTGACAGCAT
GCGTGTTTTTCTCACGATGTATAACCTTTGTTTATGTTCCATTGGCCGAGACAGAGGCTGTAAAGGTTTTATCAGACTAG
TTGTGGCAGTCCATTGCTGGGTTATTCTATACTCACACAGCCTTGTTGTTCTGTCACTAATGTCTGGGTTTTACTCCTTT
TCAACCCTAAAGTACGCAGATAATTGTTTCCATTGTAAGTTCTTGCCTCTTTTATTGGAAAGAATGTGTTTGTGAGATAT
TATACTACTTCATTTATCAATCAGTCAAATGTATTTATAAATCCCTTCTTACATCAGCTGATGTCACAAAGTGCTGTACA
GAGACCCAGCCTTAAACCCCAAACAGCAAGCATTGCCGGTGTAAAAGCATGGTGGCTAGGAAAAACTCCCTAGAAAGGCC
AGAACCTAGGAAGAAACCTAGAGAGGAACCAGGCTATGAGAGGTGGCCAGTCGTCTTCTGGCTGTGCTGGGTGGAGATTA
TAACAGAACATGGCCAAGATGTTCAAATGTTCATAGATGACTAGCAGGGTCAAATAATAATAATCACAGTGGTTGTCGAG
GGTGCAACAGGTCAGCACCTCAGGAGTAAATGTCAGTTGGCTTTTCATAGACGATCATTCAGAGTATCTCTACCGCTCCT
GCTGTCTCTAGAGAGTTGAAAACAGCAGGTCTGGGACAGGTAGCACGACTGGTGAACAGGTCAGGGTTCCATAGCCGCAG
GCAGAACAGTTGAAACTGGAGCAGCAGCACGGCCAGGTGGTCTGGGGACAGCAAAGAGTCATCAGGCCAGGTAGTCCTGA
GGCATGGTCCAAGGGCTCAGGTCCTCCGAGAGAGAGAGCGAGAGAAAGAGAGAAAAAGAGAGAAAGAGAGAGATATTTAG
AGAGAGCATACTTCAATTCACACTTCAATTCACATGGACACCAGATAAGACATGAGAAATACTTCAGATATAACAGACTG
ATCCTAGCCCCCCGACACAAACTATTGCAGCATAAATACTGGAGGCTGAGACAGGAGGGGTCGGGAGACACTGTGGCCCC
GTCCGACGATACCCCCGGACAGGGCCAAACAGGCAGGATATAACCCCACCCACTTTGCCAAAGTTCAGCCCCCACACCAC
TAGAGGGATAACTTCAACCACTAACTTACCATCCTGAGACAAGGCCGAGTATAGCCCAGAAAGATCTTCGCCACGGCACA
ACCAAAGGAGCGGCGCCAACCCGGAGAGGAAGATCACGTCAGTGACTCAACCCACTCAAATGACGTACCCCTTCTAGGGA
CGGCAGGGAAGAGCACCAGTAAACCAGTGACTCAGCCCCTGTAATAGGGTTAGAGGCAGAGAATCCCAGTGGAGAGAGGG
GAACCGGCCAGGCAGAGACAGCAAGGGCAGTTCGTTGCTCCAGTGCCTTTCCGTCCACCTTCACACTCCTGGGCCAGACT
ACACTCAATCATAGGACCTACTGAAGAGATGAGTCTTCAATAAAGACTTAAAGGTTAAGACCGAGTCTGCGTCTCTCAAA
TTGGTAGGCAGACCATTCCATAAAAATGGAGCTCCACAGGAGAAAGCCCTGCCTCCAGCTGTTTGCTTAGAAATTCTAGG
GACAGTTAGGAGGCCTGCGTCTTGTGACCGTAGCGTACGTGTAGGTATGTACAGCAGGACCAGATAGGTAGGAGTAAGCC
CATGTAATGCTTGTAGGTTATCTGTAAAACCTTGAAATCAGCCCTTGCCTTAACAGGAAGCCGGTGTAGAGAGGCTAGCA
CTGGAGTAATATGATCACATTTTTGGGGAGGTTCTAGTCAAGATTCTAGCAGCCGTGTTTAGCACTAACTGAAGTGTATT
TAGTGCTTTATCCGAAAGTAGAGCATTGCAGCAGTCTAACCTAGAAGTGATAAAAACATGGATTAATTTTTTGGCATCAT
TTTTGGACAGAAAGTTTCTGATTTTTGCAATGTTACGTAGATGGAAAAAAGCTGTCCTTGAAACAGTCTTGATATGTTCG
TCAAAAGAGAGATCATGGTCCAGAGTAACGCCGAGGTCCTTCACAGTTTTATTTGAGATGACTGTACAACCATCAAGATT
AATTGTCAGATTCAAAAGAAGATCTCTTTTTTTTTCTTGGGACCTAGAACAAGTATCTCTGTTTTGTCCGAGTTTAAAAGT
AGAACATTTGCCGCCATCCACTTCCTTATGTCTGAAACACAGGCTTCCAGGGAGGGCAATTTTGGGGCTTCACCGTGTTT
CATTGAAATGTACAGCTGTGTGTCATCCGCATGACAGTGAAAGTTAACATTATGTTTCCAAATGACATCACCAAGAGGTA
AAATATATTGTGAAAACAATAGTGGTCCGAAAACGGAGCCTTGAGGAACACCGAAATTTACAGTTGATTTGTCAGAGGAC
AAACCATCCACAGAGACAAACTGATATCTTTCCGACAGATAAGATCTAAACCAGGCCAGAACTTGTCCGTGTAGACAATT
TGGGTTTCCATATCTCCAAAAGAATGTGGTTATCGATGGTATCAAAAGCAGCACTAAGGTCTAGGAGCACGAGGACAGAT
ACAGAGCCTCGGTCTGACACCATTAAAATGTAATTTACCACCTTCACAAGTGCAGTCTCAGTGCTATGATGGGGTCTAAA
ACCAGACGGAAGTGTTTCAAATACATTGTTTGTCTTCAGGAAGGCTGTGAGTTGCTGCGCAACAGCTTTTTCTAACATTT
TTGAGAGGAATGGGAGATTCGATATAGGCCGATAGTTTTTTATATTTTCCGGGTCAAGGTTTGGCTTTTTCAAGAGAGGC
TTTATTACTGCCACTTTTAGTGAGTTTGGTACACATCTGGTGGATAGAGAGCCGTTTATTATGTTCAACATAGGAGGGCC
AAGCACAGGAAGCAGCTCTTTCAGTAGTTTAGTTGGAATAGGGTCCAGTATGCAGCTTGAAGGTTTAGAGGCCATGATTA
TTTTCATCAATGTGTCAAGAGATTTAGTACTAAAAAACTTGAGTGTCTCCCTTGATCCTAGGTCCTGGCAGAGTTGTGCA
GACTCAGGACAACTGAGCTTTGGAGGAATACACAGATTTAAAGAGGAGTCCGTAATTTTCTTTCTAATGATCATGATCTT
TTCCTCAAAGAAGTTCATGAATTTATCACTGCTGAAGTGAAAGCCATCCTCTCTTGGAGAATGCTGCTTTTTAGTTAGCT
TTGCGACAGTATCAAAAATACATTTTGGATTGTTCTTATTTTCCTCAATTAAATTGGAAAAATAGGATGATCGAGCAGCA
GTGAGGGCTCTTCGATACTGCACGGTACTGTCTTTCCAAGCTAGTCGGAAGACTTCCAGTTTGATGTGGTGCCATTTCCG
TTCCAATTTTCTGGAAGCTTGCTTCAGAGCTCGGGTATTTTCTGTATAGCAGGGAGCTAGTTTCTTATGACAAATGTTTT
TGTTTTTAGGGGTGCGAATGCATCTAGGGTATTCTGCAAGGTTAAATTGAGTTCCTCAGTTAGGTGGTTAACTGATTTTT
GTACTCTGACGTCCTTGGGTAGGCGGGGGGAGTCTGGCAGTGCATCTAGGAGTCTTTGGGTTGTCCGAGAATTTATAGCA
CGGCTTTTGATGATCCTTAGTTGGGGTCTGAGCAGATTATTTGTTGCGATTGCAAACGTAATAAAATGGTGGTCCGATAG
TCCAGGATTATGAGGAAAAACATTAAGATCCACAACATTTATTCCACGGGACAAAACTAGGTCCAGAGTATGACTGTGGC
AGTGAGTAGGTCCGGAGACATGTGGGACAAAACCCACTGAGTCGATGATGGCTCCGAAAGCTTTTTGGAGTGGGTCTGTG
GACTTTTCCATGTGAATATTAAAGTCACCAAAAATTTGAATATTATCTGCCATGACTACAAGGTCCGATAGGAATTCAGG
GAACTCAGTGAGGAACGCTGTATATGACGCAGGAGGCCTGTAAACAGTAGCTATAAAAAAATGATTGAGTAGGCGGTGAG
GCCTCATTTAACACAGTACATTCATCAGGCTTCATCAGGCTTAAGCCATGTTTCAGTCAGGCCAATCACATCAAGATTAT
GATCAGTGATTAGTTCATTGACTATAACTGCCTTGGAAGTGAGGGATCTAACATTAAGTAGCCCTATTTTGAGATGTGAG
GTATCACAATCTCTTTCAATAATGGCAGGAATGGAGGAGGTCTTTATTCCAGTGAGATTGCTAAGGCGAACACCGCCATG
TTTAGTTTTGCCCAACTTAGGTCGAGGCACATACACGGTCTCAATGGGGATAGCTGAGCTGACTACACTGACTGTGCTAG
TGGCAGACTCCACTAAGCTGGCAGGCTGGCTAACAGCCTGCTGCCTGGCCTGCACCCTATCTCATTGGGGAGCTAGGGGA
GTTAGAGCCCTGTTTATGTTCGTAGATAAGATGAGAGCACCCCTCCAGCTAGGATGGAGTCCGTCACTCCTCAACAGGCC
TGTTTGTAGCTGAGTACCAGAAAGAGGGCCAATTATCTACAAATGTTATCTTTTGGGAGGGGCAGAAAACAGTTTTCAAC
CAGCGATTGAGTTGTGAGACTGCTGTAGAGCTCATCACTCCCCCTAACTGGGAGGGGGCCACAATTTTATGTCACTTAGG
CCAATGTAATTTCATCACTTTGATATGACAAATCATTATATTCACCCAATAATTGCAGACCGTATCAGTTCAATTTAAGA
TGAAAACATGATTGCCATTTATTTGATTGTCTCTTAATTACATGTCAATTAACTCCATAATTTTCCACTGACTAACTCTC
ATACACTTGCTACTTGGCTTTAGTTCGTAGTGCACATGATGCTCGTCTCATTGGCTTTGACTTCACTTAGCATGCAGTCT

CAAGAAACTCCCCGGGGCTATAAAAAGAAGTCGCACTGACTCAGGCACCAGCTGCAGTGGTAACTTCCCTCTTGTTCTGT
TTGTGTCACATGACATGACCACTGTACTATGTGTCTCGTGTTAAATAATCTATGGGTGGCAACCAGCCGGTGTGTCTATG
TGTTGTGTGTTGTACAGCTGCCACATCCCCCACTCCACTCAGATATCATAGGCCGGGTTTCAGAGGACATTCTATCAAGT
GGAATTGAATCAAGCCACTTGTTCACTCTTTAATGAGGGAAACTTAGATGCATGTGAATGGATTTAGACAATGGGGTCAG
GCCTCACGATGACAATCCATCCCCCCCTGCAGGCAATTAAACTTTTCCACACAGGGCAACATGTTCTTTAGGTCAGGTTT
CATAGATATGAGCTACCAGCATCAACATTTGTTTTGAAGCTATAGCTTGGTGGAATAATTTCAGTAGTATTTCCGCTTAT
CTAAAAATGGGGCCTATGGTTTGTCATATAGTGCTGTGGTGACCCATACAGTCTACTGAATATTCATTCTGAACAAAAAA
AACGAAGTGTCTTGCCAATATGACCCTGATAGTTAAAACAACCTTATGAACCAAAGATTTAAAAGAGATTTCAGATACGA
CTTTGATATAAGGAAATAGAGCAGCTCTAGAATACCACCAGGGCTCACTTTAAAGAGTAGAGAGGGTACCATGGGATGCA
CAGATGAAAGCGTGAGAAGAAGAGCGGGGAAAGATGGGAAGGAAGGGGTGGGAGCCTGAGAGGGAGAGCAGGGAGCTAAA
AAAAAACAATAGGCATCTTAAAATAAAACTTCTGAGGATATATTTACGAGCCCCAATTGTACTGCTGTATGAAGTAGTCA
ACAATATTGGTTTCAGCGGTGGGATTACATTTTCACATGACAATACGCTCCCATTTGCTCACGTACACATTTGATCATAC
TGTATAACAACCATAGATACTTATTTAAAGAGACATAGCCCAAAATGTCTGATGTTGACGAACAAGTGACGGAAGCAGAA
CTTGAGAATGTGTAAAGACAGGGGGTGAAGATTGAGAGATTGTGAGGCTCTTCCTGCTTTCCCCCTCCCCTGCACCGATG
CCTATTGTAGTGGTCAGAGGAGGCCGGGTTGTGAGGGAAAGAGAAAGACAAAAAATAGCAGCTCGTCACAACAGCACATC
GTGCATCCTGTGTTTGTCTCCGCTCATTATGTACTTCCTCTATGGAGAGAAAAGATGGAAATATAGAGACAAGGAGCATG
TTGAATCATGAGAGAGGTTCCACATAAAGAGTATAAACATTGAGTTAAAACTAGTAGCAATGGAAAAAGTGGGCGAACGA
AAAAAAACATTGGACAAAATCTGTAAACATGAGCAGACAAAATGGGAGATTTGAATGATGCGGTATCTGAGGAGAGTCAA
GCAAATTTCCTTATCAAGATGTTTCTCATGGAATGAACCTCAGCCACTTACTTCCATGATTATTATCATCTGCGCCGTGT
GCCAGCTTTTCCTTACAGCGGAGCAGCCAAGTCCTTGTATTGATTGTTGGGTAAAAATGCACTCAAACAGGTTCAAAGAT
TTTGTGCATTTAGTAGAACAGAAACACTGTAGGGGTTGTTACATGCCACAATATTAGACATCCTCATTGGTGCCTCCCTG
TTCCATTGCCACTCACAGACTGAGATGTGGTGATTGTGGTTGGTTGAGGTTGACACGGATGGTCCCGAGTAAGTGGTTCT
ACTGTAGGTAACTGTACCAGGAAGAGTTGTGGGTCAACTTAACCTTAGACCTACTGCTTGAGACAAAAACATAATACCAT
AGAAAAGTAGGCCTACAGTAGACGGATGTTAAAAATGTAAACAAGTTGTATTTTGCCTCACTTTTACAACTGCAGTCTCT
TATAAACAGCGACTGTGCTCTTAGCCTAGTTCATAACACCCTGATTAGTTGAATGCACAGTGCTACTGTAGGCCTACAGT
ACAAAGCCTGTAGTCACTGTTTTTCGTGACCTGACGTAGGCTAGCATTTTCCTGTAGTCCACGATCAGCTCCTTTGTCTT
GCTCACATTGAGGGAGATGTGATTGTCCTGGCACCACACTGCCAGGTCTCTGACCTCCTCCCTGCAGGAGGTTAACATGC
TAACAGCCAGACCTAGCTTCTACTAGTGGTAAAAGGCTGTAGCCTGAACCAGTCTATCATTGTGAGGCTGAAAGTCTTCA
CCTGTGTGTCAACATGTGCTCACCCGCTAGTGTCGTTAGCCCCTTATGTAAAAAGGGCGTGCTTCCTGCTTTATTAGTTG
AATAGGAACATTTATGAAGTCATTAGCATATGTCACATATTAATAGATCAGTGTCAGAAGATGTAATGATCCTCAGAGCA
GGTTTGTTGAAATGTTGATAATGTAGGATGAGTTTAGTTAATTAGGTCTCCCTCTAAATTTACTCTATTTATTGTGGGAA
GAGATATAGTCAAACAGGTTTAGTTACAAATGTAGGTCATTCACACAGGGAAAGAGAGTGGGAGACGGGGAAGGAGGGAT
TGAAAGAGAAAAGGTATTGTTCAGCCAATAGCTGCAGGCCTTCTCATTGTGTGTAGGCTATGTGAGTCCCCTGTGTGTTC
AACAGGTGAGTATAATATTATATCATCCCTCTGTCTGTCTGTCTGTCTGTCTGTCTGTCTGTCTGTCTGTCTGTCTGTCT
GTCTGTCTGTCTGTCTGTCTGTCTGTCTGTCTGTCTGTCTGTCTGTCTGTCTGTCTGTCTGTCTGTCTGTCTGTCTGTCT
GTCTGTCTGTCTGTCTGTGAGTGAGTGAGTGTATACGTGTGCATACGTGTGCATACGTCTATCTCTGTGTGTGTTATGGG
TGTGTCCATGTGACGCAGGCACACAGAGGGGGTGGCCCTACCTAATGACGGCTTCAGGTGTGTTTATGTGTGTTAAGTCC
GAGAGCCAGACAAACTGAAAGACAAAGACAAACAATCCCTCGGTCATTGAAAAACGACGGTCAAGGAGAAAATAGGAAAC
TAAAAAGACCGATGAAAGAAAGTGGACAAGATGGTGTTTGTGGCTTTCTTGTGAAGTTCTCAAAGAAGCTAAAGGCTTGT
AGCCTGTCTGGACTTGTCGAGGCAAAGGTAACTTGTGTTTTTCTGGGTCAATGTCAATCAGGTACAGATAGGGGGGAAAG
AGAAAGAGCAACAGACATATGAGATCAACAACAGAAACATGCTGCAGTAGGTAGACCAACATGTTCTGAACCTATGTCAC
GATAATAGACGGACTATGGATTTTTTTATTCATCCTTTGTTAAACTAGGCAAGTCAGTTAAGAACAAAATCTTATTTACA
ATGAAGGCCTACCCTGGCCAAACCCTATCCCGGACGACGCTGGGCCAATTCTCCCCCGCCCTACGGGACTCCCAATCACG
TCCGGTTGTGATACAGCCTGGAATCGAACCGGGGTCTGTAGTGACGCCTCTAGCACTGAGATGCAGTGCCTTAGACCCCT
GTGCCACTCGGGAGCCCGAAATAAGATCCTTTCGCTACATTAACTGAGCATGGACCTTCGGTACTCCAATTGATTTGAAT
GAGAAAGTGGTTACTGAAGGTGAATACAGAAGGCACCTCCGCATGTCAAAAAAGGAAACTGATAGACACGTTATAGGATA
CAGCAGTTGTTTTATAACTGTGATGTCTGGTCAGTGGTCTATATGACCTGCTTTGTGCTTAATGCATTTGCACGTATATG
TTGACCTGGATATTGCTTATCATCATATTGTGGTTGGATCTCAACTGAAGGGGGAAGTTAATGCAAAAGGAGGCAGTGCA
ATGCCTTAAATAGCTTTTAAACGTGTTGACGTTGTTGTGGTTTACATGTTGAATGATTCACATATGACCAGTCGGACTTC
TGTTTGGGTCACTGGTATGGATTGTTGATGTCAGACTAACGATGAGGTCACGGTGTTTTCTCATGCATTTTTCAGCATGG
AAAATCACATTTTCATGGGTGTTAATGGCAGGGGAAGTGTAAACTGATATATAGGTTCAGCTGCATTTACAATTGACTGT
GGATTGTTTTGTGTTAAGACGTACTCCACTTACAACCTTATCAGATTCTGGTGATATTTCACCCATGTTTGTTTGTTTTC
TCTTTTCAACCCACTCAGATATTTTCTGTTAAAACTGCTTTGCCCTTTGAAACTCCTAAATATTTTGTGTTTATTATTGT
ATAATTCTAATAACATAATATGCTTCATGGGCACGTCGGGGCTTTATGTTAAAACCCAAGGTTCTGGTCATGTGACAGCA
CTTATTTTTATTGGATGTTATTGTATTCCACAGGGGGAGACTTCCCCCATGAAGACAACTTGGTACTGTCCTCTGGATTT
GGTAGGTCAATGCAACTTGAGATAAGCATGAATAACTAGATTTTGGTGACAGTGGGTGATTTCTGCGAGAGTATGAATGA
TGCACTTTTTTTTCTGAATCATACTCTTAATCATACTCTTTCTCTCTTCCTCAGTCTACTGTGTTGGAGGAGGAAGAGGA
CGGAGTGATATACAATGTGGTGGTGAAGCAACAGCATGGCGCCCCCACCAGTGCAACAGTGAGAATACATACCATTATAC
AATGCACAGAAATTACAGTATAATTACACCTTACATCCTTACACGTTATCTTTGTAAGTCTGTATTGTCATGGTAATATT
GTTATGAATCTCTCTCTCTCTCCCTCCCTCCAGTCCAGCTCTCGTTCGCAGTGTGTGAAGGCGGGAACAGAGGAGAAGTT
GGTGCAGCACCTCCTCCACTCCTTCGCCATGGGAGACTCCTCCTTCATCACCATCTTCCTCTCCACCTATCGCTCCTTTA
CCTCTACCAAGAGAGTGCTGGACATCCTCATCGACAGGTGGGGTAGACAGAGGGAACAATGCATTGCACACCAGTTGTAT
GTAGTGTTACAAAACGTTTTTGAAGTGCTGTAGAAAGACCAAGGGAAGGAAAGGAGACATTGTGATGCCTCTGCGTTGCC
CTGAAGAAACCATCTCTTATGCAGCTTTTTTTTCTCTCAAAGGAACTTTCGTCTCTATTTTTTTCTCTTTTCTTCCCTCTT
TCTCCAGATTGCAACATCCTCCAGGAGAAAGTACAAGGAGTCAGACTAGGCAACCCTTCAACAGGTGAGTCTGATACTAT
ATGATCCCTCTCACGTGTGTGTGTGTGTGTGTGTGTGTGTGTGTGTGTGTGTGTGTGTGTGTGTGTGTGTGTGTGTGTGT
GTGTGTGTGTGTGTGTGTGAGTGTGCGTGGGTCAACCCCAAAGAAAAAGAAGACACTCGCAAACCCAAANNNNNNNNNNN
NNNNNNNNNNNNNNNNNNNNNNNNNNNNNNNNNNNNNNNNNNNNNNNNNNNNNNNNNNNNNNNNNNNNNNNNNNNNNNNN
NNNNNNNNNNNNNNNNNNNNNNNNNNNNNNNNNNNNNNNNNNNNNNNNNNNNNNNNNNNNNNNNNNNNNNNNNNNNNNNN

NNNNNNNNNNNNNNNNNNNNNNNNNNNNNNNNNNNNNNNNNNNNNNNNNNNNNNNNNNNNNNNNNNNNNNNNNNNNNNNN
NNNNNNNNNNNNNNNNNNNNNNNNNNNNNNNNNNNNNNNNNNNNNNNNNNNNNNNNNNNNNNNNNNNNNNNNNNNNNNNN
NNNNNNNNNNNNNNNNNNNNNNNNNNNNNNNNNNNNNNNNNNNNNNNNNNNNNNNNNNNNNNNNNNNNNNNNNNNNNNNN
NNNNNNNNNNNNNNNNNNNNNNNNNNNNNNNNNNNNNNNNNNNNNNNNNNNNNNNNNNNNNNNNNNNNNNNNNNNNNNNN
NNNNNNNNNNNNNNNNNNNNNNNNNNNNNNNNNNNNNNNNNNNNNNNNNNNNNNNNNNNNNNNNNNNNNNNNNNNNNNNN
NNNNNNNNNNNNNNNNNNNNNNNNNNNNNNNNNNNNNNNNNNNNNNNNNNNNNNNNNNNNNNNNNNNNNNNNNNNNNNNN
NNNNNNNNNNNNNNNNNNNNNNNNNNNNNNNNNNNNNNNNNNNNNNNNNNNNNNNNNNNNNNNNNNNNNNNNNNNNNNNN
NNNNNNNNNNNNNNNNNNNNNNNNNNNNNNNNNNNNNNNNNNNNNNNNNNNNNNNNNNNNNNNNNNNNNNNNNNNNNNNN
NNNNNNNNNNNNNNNNNNNNNNNNNNNNNNNNNNNNNNNNNNNNNNNNNNNNNNNNNNNNNNNNNNNNNNNNNNNNNNNN
NNNNNNNNNNNNNNNNNNNNNNNNNNNNNNNNNNNNNNNNNNNNNNNNNNNNNNNNNNNNNNNNNNNNNNNNNNNNNNNN
NNNNNNNNNNNNNNNNNNNNNNNNNNNNNNNNNNNNNNNNNNNNNNNNNNNNNNNNNNNNNNNNNNNNNNNNNNNNNNNN
NNNNNNNNNNNNNNNNNNNNNNNNNNNNNNNNNNNNNNNNNNNNNNNNNNNNNNNNNNNNNNNNNNNNNNNNNNNNNNNN
NNNNNNNNNNNNNNNNNNNNNNNNNNNNNNNNNNNNNNNNNNNNNNNNNNNNNNNNNNNNNNNNNNNNNNNNNNNNNNNN
NNNNNNNNNNNNNNNNNNNNNNNNNNNNNNNNNNNNNNNNNNNNNNNNNNNNNNNNNNNNNNNNNNNNNNNNNNNNNNNN
NNNNNNNNNNNNNNNNNNNNNNNNNNNNNNNNNNNNNNNNNNNNNNNNNNNNNNNNNNNNNNNNNNNNNNNNNNNNNNNN
NNNNNNNNNNNNNNNNNNNNNNNNNNNNNNNNNNNNNNNNNNNNNNNNNNNNNNNNNNNNNNNNNNNNNNNNNNNNNNNN
NNNNNNNNNNNNNNNNNNNNNNNNNNNNNNNNNNNNNNNNNNNNNNNNNNNNNNNNNNNNNNNNNNNNNNNNNNNNNNNN
NNNNNNNNNNNNNNNNNNNNNNNNNNNNNNNNNNNNNNNNNNNNNNNNNNNNNNNNNNNNNNNNNNNNNNNNNNNNNNNN
NNNNNNNNNNNNNNNNNNNNNNNNNNNNNNNNNNNNNNNNNNNNNNNNNNNNNNNNNNNNNNNNNNNNNNNNNNNNNNNN
NNNNNNNNNNNNNNNNNNNNNNNNNNNNNNNNNNNNNNNNNNNNNNNNNNNNNNNNNNNNNNNNNNNNNNNNNNNNNNNN
NNNNNNNNNNNNNNNNNNNNNNNNNNNNNNNNNNNNNNNNNNNNNNNNNNNNNNNNNNNNNNNNNNNNNNNNNNNNNNNN
NNNNNNNNNNNNNNNNNNNNNNNNNNNNNNNNNNNNNNNNNNNNNNNNNNNNNNNNNNNNNNNNNNNNNNNNNNNNNNNN
NNNNNNNNNNNNNNNNNNNNNNNNNNNNNNNNNNNNNNNNNNNNNNNNNNNNNNNNNNNNNNNNNNNNNNNNNNNNNNNN
NNNNNNNNNNNNNNNNNNNNNNNNNNNNNNNNNNNNNNNNNNNNNNNNNNNNNNNNNNNNNNNNNNNNNNNNNNNNNNNN
NNNNNNNNNNNNNNNNNNNNNNNNNNNNNNNNNNNNNNNNNNNNNNNNNNNNNNNNNNNNNNNNNNNNNNNNNNNNNNNN
NNNNNNNNNNNNNNNNNNNNNNNNNNNNNNNNNNNNNNNNNNNNNNNNNNNNNNNNNNNNNNNNNNNNNNNNNNNNNNNN
NNNNNNNNNNNNNNNNNNNNNNNNNNNNNNNNNNNNNNNNNNNNNNNNNNNNNNNNNNNNNNNNNNNNNNNNNNNNNNNN
NNNNNNNNNNNNNNNNNNNNNNNNNNNNNNNNNNNNNNNNNNNNNNNNNNNNNNNNNNNNNNNNNNNNNNNNNNNNNNNN
NNNNNNNNNNNNNNNNNNNNNNNNNNNNNNNNNNNNNNNNNNNNNNNNNNNNNNNNNNNNNNNNNNNNNNNNNNNNNNNN
NNNNNNNNNNNNNNNNNNNNNNNNNNNNNNNNNNNNNNNNNNNNNNNNNNNNNNNNNNNNNNNNNNNNNNNNNNNNNNNN
NNNNNNNNNNNNNNNNNNNNNNNNNNNNNNNNNNNNNNNNNNNNNNNNNNNNNNNNNNNNNNNNNNNNNNNNNNNNNNNN
NNNNNNNNNNNNNNNNNNNNNNNNNNNNNNNNNNNNNNNNNNNNNNNNNNNNNNNNNNNNNNNNNNNNNNNNNNNNNNNN
NNNNNNNNNNNNNNNNNNNNNNNNNNNNNNNNNNNNNNNNNNNNNNNNNNNNNNNNNNNNNNNNNNNNNNNNNNNNNNNN
NNNNNNNNNNNNNNNNNNNNNNNNNNNNNNNNNNNNNNNNNNNNNNNNNNNNNNNNNNNNNNNNNNNNNNNNNNNNNNNN
NNNNNNNNNNNNNNNNNNNNNNNNNNNNNNNNNNNNNNNNNNNNNNNNNNNNNNNNNNNNNNNNNNNNNNNNNNNNNNNN
NNNNNNNNNNNNNNNNNNNNNNNNNNNNNNNNNNNNNNNNNNNNNNNNNNNNNNNNNNNNNNNNNNNNNNNNNNNNNNNN
NNNNNNNNNNNNNNNNNNNNNNNNNNNNNNNNNNNNNNNNNNNNNNNNNNNNNNNNNNNNNNNNNNNNNNNNNNNNNNNN
NNNNNNNNNNNNNNNNNNNNNNNNNNNNNNNNNNNNNNNNNNNNNNNNNNNNNNNNNNNNNNNNNNNNNNNNNNNNNNNN
NNNNNNNNNNNNNNNNNNNNNNNNNNNNNNNNNNNNNNNNNNNNNNNNNNNNNNNNNNNNNNNNNNNNNNNNNNNNNNNN
NNNNNNNNNNNNNNNNNNNNNNNNNNNNNNNNNNNNNNNNNNNNNNNNNNNNNNNNNNNNNNNNNNNNNNNNNNNNNNNN
NNNNNNNNNNNNNNNNNNNNNNNNNNNNNNNNNNNNNNNNNNNNNNNNNNNNNNNNNNNNNNNNNNNNNNNNNNNNNNNN
NNNNNNNNNNNNNNNNNNNNNNNNNNNNNNNNNNNNNNNNNNNNNNNNNNNNNNNNNNNNNNNNNNNNNNNNNNNNNNNN
NNNNNNNNNNNNNNNNNNNNNNNNNNNNNNNNNNNNNNNNNNNNNNNNNNNNNNNNNNNNNNNNNNNNNNNNNNNNNNNN
NNNNNNNNNNNNNNNNNNNNNNNNNNNNNNNNNNNNNNNNNNNNNNNNNNNNNNNNNNNNNNNNNNNNNNNNNNNNNNNN
NNNNNNNNNNNNNNNNNNNNNNNNNNNNNNNNNNNNNNNNNNNNNNNNNNNNNNNNNNNNNNNNNNNNNNNNNNNNNNNN
NNNNNNNNNNNNNNNNNNNNNNNNNNNNNNNNNNAGAGGAGACAGATAGGGGTAAGTGCTCTTATAAGCGAGTTTGAAG
TCTCTCACAAAGCTCATAATAATTTTTCCCTTGAAGGAAGAAGCATGGAATATACTGTACAAAGGGCATCAAATAACCCT
TTGCTGATGTAACGTAATGCAATGCTGGTATTTCTTCAATATAGGAATCATTGATGGTTAAGTAAGGACAACTTGATATA
CAGTACATTCACCGGTCTTAACCCAAGGTTCTCCACTGAGCATGCATTGAGTAGGCTTCATCTGTGTCTGGGAAGCCTGA
AGCCCTATGCCTCTGTTTTTGGTTTTCATTATTCTCCACCCCTCCCCTCTCTCACACAGTCCAGTGCCCAGGGCACAGTT
CCCTACCTGGGGATCTTCCTCACAGACCTCACCATGCTGGACACGGCCGTCAAAGACAGACTAGAGGTGAGTCCCTTCGT
ATACAGTCATTTATCCCTCATCATGTGTCCATCAACAGGAAGTTGATAATAGGCTCTGGTGGTGAAGTTGTAAAAAAAAA
AAAAAGTTGCATTGACAATGACATAATATCCTGTTCCTCCATTTCAGAACGGCTACATCAACTTTGACAAGAGGAGACGA
GTAAGTGACGCAATCAGGGCCTTGCGCTGCTGCTGTTTAAATTCCTCATCACCCATTGTCCACATGTTGGATCCAGACCA
ATTCATTAGCCTCTGGCTCAACATAGCAGATGGTCTTCATAAATTGCTTTTCTGTTGTGCCACTCATTAAATGTGTGGCC
GTGTATTGAATTCTGAAGCAGTGCCTCCATGCCCGTATTAATGTTTAACTTCTAGCATTTCAACTGATAATGTGCCATTA
GTACTGGAGAGTCATTTGACGATAATGATTTGTTTTTCTCTTTGCAGGAGTTTGAGGTTTTAGCTCAGATCCGACTACTG
CAGTCTTCCTGTAAAAACTGTGTTTTCCTCACTGACGAGGCATTCCTACAGTGGTACCAAAGTGTACCCTCGTTAAATGA
AGAAGAAAGGTAAGCCGTGATTATCTTTTGGTTTTATTCATTTTACAATCAAAACATTCACGTTTGGACATACATGTTAT
TTCAAACGGAACTCTACAATTATGATGATCGCTAATCACATCACATGATGTCTGCAGTTACAGACTGTCCAATGAAATTG
AGGTGCCGTGCGAACCGAGCTCTGGTCGTACCCTGAACCCTACAGTCATCATCACACGGTGCCCAAAGTAATGAGGCCTT
TTATTACACTCTACACACAACATACCGGTAGTATTCACTGCAGATACCTTTCACTATGTATAGTGAAATACATTGGTTAT
ATGGGCTCAGTACTATTGTAAAAGAATGGCCAGTAAGCATTTTCTGTGAACTATACTACACTACATGACCCAAAAGTATG
TGGACACCTGCTCGTCAAACATCTCATTCCAAAATCATGGGCATTAATATGGAGTTGGTCCCCCCTTTGCTGCTATAACG
TCCTCCACTCTTCTGGGAAGGCTTTCCACTAGATGCTGGAACATTGCCACAAGTCGGGCACTGATGTTGGGCGATTAGGC
CTGGCTCGCAGTTGGCGCTCCAATTCATCCCAAAGGTGTTCAATGGGGTTGAGATCAGGGCTCTGTGCAGGCCAGTCAAG
TTCTTCCACACTGATCTCGACAAACCATTTCTGTATGGACCTCGCTTTGTACACGGGGGCATTGTCATGCTGAAACAGGA
AAGGGCCTTCCCAAAACTGTTGCCACAAAGTTGGAAGCACAGAATTGTCTAGAATGTCACTGTATGCTAAAATGTTAAGA
TTAAGAAACCCATTTCAGGAAGCTCCCAATGAACAGTTATTGTGCTGACGTTGCTTCCAGAGGCAGTTTGGAACTCGGCA

GAGAGTGTTGCAACCGAGGACAGACAATTTTTTGAGCACTTGACAGTCCGGTTCTGTGAGCTTGTGTGGCCTACAACTTC
GTGGCTGAGCCGTTGTTGCTCCTAGATGTTTCCACTTTACAATAACAGCACTTACAGTTGACCGGGGCAGCTCTAGCGAG
ACAGAAATTTGGTGAACTGACTTGTGGGAAAGGTGACATCCTATGACAGTGCCACATTGAAAGTCACTAAGCTCTTCAGT
ATGGGCCATTCTACTGCCAATGTTTTCCGAAAGAGATTGCATGGCAGTGTGCTCGATTTTATACACCTGTCATCAACGGG
TGTGGCTGAAATAGCCGAATCCACTCATTTGAAGGGGTGTCCACATACTATATACTGTATATATAGTGACTATCTTGACT
ATTTGACTGTCAAATTTGTAGTAATGTGGCCATAAGTAATCAGTTGTGGAAAATTGACTTGGGGGGGTCTGGGGGTAAGT
CCTGCGCACTTTTTTTCCATTAAAAATATCCTCACACAGTGCAAGAGCTGGTAAATGAACATGCAACATTGGAAAGAGCT
GAAAGCTAAGCTTGTACATTTACTATGTATTTCTCAGGGGTTGAGTAAGATGTTTTGTAGGTTGTTGTCATGCAGATTAC
GGTGCATGATGAACATACATGATATGATTCTCTCACCTCCTTACAGCCTGAATTCCTCCAGGACCAGCCTTCTTGCTGAC
AGTGACGGTCTTTTTGACTTCCCCTCCCCCGTCTGTCACTTTCTTTCCAAATTCACCAAGGTAATTACAAAATAAGACCT
AATATCTCCAGTTATTTTACGTACTTTCCCAAAAGTATATTTGCATTGCCAGCTAGAATTGCAGAGCATGCAATTACTAT
TCATGCGCAAATACTCGTAGTTATTGCTAAGTATTGCTGTTTACTAGTAATCAAATATGATTGTTTTAAAACAAATGTCG
TAAAAACATACAGAAGAGCACTTTTAAAAGAGGAAAATCTTTGATCTTTTAATTCTCTTCTGTGTCTTTTTATTTGCTTT
GGCCTTCATGGAACAGCCACTTTTTATCTTGTCATATCAGTAAGTATAATGTTTTTATTTATTATTTTCATCACTATTAT
TATTTCAGCATATGAAATCCCCCTCAGTTTCCTGTCTGGATGTTGACTCCTCCCCTCCCACCAATGACCCCATCCCGTCT
GCATTGACACCATCCACTCCCATCAAATCACATCGTCGATCAGTCTCCTGTGGCAACAATCCCACCAATAACAACAAAGG
GTCTGGGCCTGATATGCGGATCGTCAGGATACGGATGGATTTACAAGATGGGAATATGTACAGAAGCATACTGGTACTGA
GCTGGGATTATGATATATTGAGTTGAGATTTGATTAGAGATATTTGCCTTTTTTTGGTATCAAATTATACACTGCACTAC
ACCCTGCACTCATGACTATTTACTTTAATCCACAAATATCCGTTAATGTAAGTAATCCAAAACATTTCCTCAGGTTGTAC
ACAGGATCTTATAACTAGGAGTCAAGCTGTCTGGTATCATTGTCGTTCTCCTTCTCTGTTTCCTCAGGTGACAAGCAATG
ACAAGACGCCCACTGTGATCAGTTCTGCCTTAGAAAAACACAGTCAGGACCCCAAACAAGCATCCAGATATGAGCTGATC
CAACTATTGCCAGAGGGAAAGGGTAAAGACATTTCTCAACCCGATTATGCGAAATCACTTTAACAGTTCTCTGTTCATAT
TGATTGTAGTGATGACTTTTCTTGTTCTGCCCTGTAGAGTTGGTCATCCCTGCCACAGGAAACGTCTTCTATGCCATGAC
TTCCTCTAGTGTGGACTTCCTGCTGCGCAGACAAGGAGGCAACACCCCTTTTCGCTCTCAACCAATCAGCACAGAAACTA
GTGCCACCTTCCCTCGAATCAAAGCCAAGGGGCGGAGACAGCTCTTTCCCAAATAGGCTATATCTGAGACATAATGTAGC
TAGTGCTTCTCAAGGACAGGTCACGATAAGAATGCAGGCAGATATCTCAGTGAGATGTGGCCTTGTCTTTCCTAATCTTT
TTCCGAAGTGGGAACACATTCTGAGCGAGCTTGTTCTAGCAAAATGTATCCTGAAACCTGTCTGAACTTGTTTGATGAAC
GTATTCTTACCTGGGACATTTCAATAAATAGGCTGCCCGTTTGTGTACACCAGGGAAGTCCATTAGTGTAGAAGGGTGCA
TGTCTCTCTCGTTGGCAATGCATACCACTGCAAATATACACCTAGAACAAGACCATTGCAAATCTTTAACAGATGGTGAA
TGGTTCTGTTCCTTAAGTCTCATTGGCATCTAATTTCCTATTTAACCAAATGGAATAGCTTTGGAACCAAGAGCAACTGA
GGAATCCAGATCATCTTGAGTATTTGAGGAATGTGCCTCAGGTGCCATCCTAGAAATGAGTTGATAGTAGAGAACTTCAT
TAAATCAAAGTAGTTTGGGCGATATGACCTCCTTGAATTGACCATGACCACAACAAAGTTTTACATTTCGCCATAGAGAG
TGGATACAAGCAGCATGACGACATATTAATTATGCAAGGCGTTTCGAGAATGTGAATGATGGGGAGAGCACTGGGCTGAC
ATTATGACATAATGCATCTGAATAAGTGACAGACAGAGTCACTGAAGCCTCTCATTATAGCAAAGCACGGGGGCTCATCA
AAATGTTTTAACTATGTACTTTGTATGTATATGTTTTTTTATGTATGGACGATGTCATGATTGTGTTTACATTCCCAAGG
AGCTATCAACAAGTGTCAGAATGACATGTTATTCATTGTGAAGTAACATGAAATGTATGAAATTGCCTCAAATTGCAACC
ATTTCAGATGGGTCAAGACGTGTCAAAGATTTACATGATCATTTCGCTTGAGCTTTGTGCATACATATTGAATGATACTT
CTGGTAGTATCTTATGTAAAATACAATAACTTTACATTTACAGCCTTGCCACTCCCATATAGAATATAGTCATATTTTGA
GTACTAACCATTAGGCGTAACAGCATCAACCCACAAACTCTAACCTAAACAATTCATGTGAAGACACTAATGAAAGTCCA
GACTATGTTGTGCGTATTGAAAAGATGTCTACCATTGCACCTTAATGCTATACTTTTCAATACAGCATAAGCTAATCTTT
ATCAATGGTCTCTGAATGGGTTTAGTTGGTAGTTGGAATGGAAGTCTGTCTGAGGAGTATTTCAACCAGGGCTCCCAAAG
TCAGTCCTCAACGACCCATGCGAGGTTAGTGAGGGATTCTGACCAATCACTGAATGATGTTTAATTGATATTTTCTCTGA
CGTTTGATAGAGATCTGACAGCTATAAGGTTTCATTATGCAATGTCTTTAGTCCAGGAACTGAATGTAGAGGGTTTTGCA
AAAGGCTCTGCTTCTATCCCATGAGGATGAGACCCGTAGGAAGAAAAACCTGTATGCATATGGACGCATGGGGGACAGAT
TGTCGGAGGCCCTAACTGATGAACTCTGGCCAGTGCTGTTGAAGTCTCTACTAAGGCTGAAGCATAGATTCAGTAGTTCC
AGGGAGAGGAGAGAGGATTCTGGGTAACGTAATTTGATGAAGTGTTAGCGCCAGGCTAAGCTACTGATTGTTTAGTGCCT
TTCTCTCATTGGTGCTTGAACCACTTAAAAGTGTCCCAAATGTCACCCTATTCCCTATATAGTGTATTACTTTTCACCAG
AGCCCTATGGGACCTGGTCAAAAGTAGTGGGCTTAATAGGGTGCTATTTGACATGAAGCCTCTGTGATATATGCATTGGA
ATGTACAGTACATATGATGCACATTCACAGTGCTGTATAAGCTGGCATGTTTATGCACTACATATGCCCTGTTGTTTTTG
TGTGATACATTTCCTTTTTCTATGCATTTAGATGTGACAGTAATATGAGGAATTTCTATGCAACTCCCCGGATGTGTGCT
GTAATTCTGCTGTGTCTATTCTTATCAAATAAATGTACAAAGATGTACATATAAAAGATTCAGACTGTGGTTTAGTATCT
AGCTTGTTGCTGGATGAGGAAGTTTAATGAAGAACCTTTTGTGCACCCCGCAACAGTACTTTCTTTATTGGGACAGAAGA
CAGTCAGTATCTTTGCCTGTTTCAAGCTAAAAGCTGAAAACGTCACTAAACGTTTGACTAAAAAGTAAAAGAGAAATAAA
GAAAAAACATTATACATACTTTAACGATCATTTAAAAAATTACAAAATTGTTACATAAAAAAGGTTGAAAACAGACGCAT
ATAAAATGTACCTGAGATCACAATCAACATTTCCTACAGTCATTAGCAACTTATTAGCATTTCCTACAGTCATTAGCAAC
ACAGAATGTAGCAAAGTGCTCATTGTTTAATTATTATAGGAGTGCCACTTCCCTTTCAAGTATGTTTCTATTCATGGCAA
TTTATGAAACACTCCAAAGCTGCCATAAAAAAGTGAATTAGATGCTGATACCTTATTAATCAACATTCTATGGGGTACCT
TGTTCATGGAGCTTAATAATACTAATACACCTAGCTGCTCAGTGAACTGTGTGCATACTAGTAACTTTACACTGGTGCCA
CACTGATGCTTACTGATTCATATCTCCCTGGTAGTGCCTTGCATCCTCTACGTCCACATCACACCCGCTAGTCCCTCCCC
CGCACATCTCACACTCCAGCGCCGCTGTCCGATAGGCCTTCCACACCGTGGCGTCCCGCAGACACACAGTCCCGCCCCCC
CTCTTCTCCTCAGCCTCGTTCCGGTTGATGTCGCCCACACACACCCAGCCAGCAGCTCCATCCACCCCACCCCCGGTGGT
ACTCACGGCCCACTTTGAGTGGTCGTTGGAGGCCTTGAAGGTGATCCTCCCGCCTGGGGAGATAAGCTGGATGTCCAGGA
CCTTCCAGCCCTGGGAGCAGTCAGAGGGCAGGATGCCAACGGAGCGGATCCAGAACTGGACTAGGAGGTCTGACTGGAGG
GAGGGAGCCACCCAGGAGTGGTACAGATCTAATGGGAGACAGGAGGGGAAATGGGGATATTGTGGTTAAATGTTGGGTCA
TTCACAACTACAAAATGGCAATGACATGAATAGTCATTCTAACCATGAAACAACACTTCAACAGTTTAAAAAATGACAAA
CGAATAGTTTCTAAATTGTTCCTTACCATTATTGAATGATCTCCCCTTGGCAAAGCTGATGAACTCGGTTCCACCCAATG
AGGTGAGAGACACACTGCGATTGGATGCAGGTTGGGGGTGCGGTAAGTTGCTATAGGCCATCTGCCCTTGGCTGTTGTTA
CAGACGTCAGCCAGTGCGGGCACCAGAGAGGCCAAGGAAGCAGGGACACCACAGTCATACACATTGGGCTGGTTGATCTG
CAACTGCTCTCCTACAATGTAAAACATAATACATGTCAACTGAACACTTCAAATATTTCAATCAACGAATTGTGTAAAAA

GTAAAACAAATCATTATGAATTGACTGGTCTGTCTGTCTGTCTGTCTGTCTGTCTGTCTGTCTGTCTGTCTGTCTGTCTG
TCTGTGTGATTCAATAGGCACCAAGCTCCAAGACAAGTACTATCTTAATCTATGACATGACTTATTAGTATGATGACTTT
TCAAATTCTTCAAACAGAGTAGCAGACAAATGCCTGTACGTATAAGACCACTCACTGATTGTGGCTAAAATGAGATTGAA
AATGTTTTAAGACAAGACAATGCTTTGAGGATGATGGAAGTTGGCCTCCACTGAGAACTCCTATCACAATTTAATGACAC
TCATCTTAAACTGAACAAAAATATATATGCAACACATGAAGTGTTGGTCCCATGTTTCATGAGCTGAAATAAAAGATCCC
AGAAATGTTCCATACGCACAAAATGCTTTTTTCTCTCAAATGTTGTGCACTGTTAGTGAGCGTTTCTCCTTTGCAAAGAT
AATCCATCCACCTGACAGATGTGGCATATCAAGAAGCTGATTAAACAGCATGATCATTACACAGGTGCACCTTGTACTGG
GGACAATAAAATGCCACTCTAAGATGTGCAGTTTTGTCACACAACACAATGCCACAAATGTCTCAAGTTTTAAGGGAGCG
TGCAATAGGCATGCTGACTGCAGGAATATCCACCAGAGCTGTTGCCAGATAATTGAATGTTCATTTCTCTACCATAAGCG
GCATCCAACATCATTTTAGAGAATACGTCCAACCGGCTTCACAACTGCAGACCACATGTATGGCGTTGTGTGGGCGAGCG
GTTTGTTGATGTCAACGTTGTGAACAGAGTATCAAATCAAATCAAACTTTATTTGCCACGTGCCGAATACAACAAGTGTA
GACTTTACTGTGAAATGCTTACTTACAAGCCCTTAACCAACAGTGCAGTTCAAGAAGAAGAAAATATTTACCAAGTAGAC
TCAAATAAAAAGTAATAATAAAAGTAACACAATAAGAATAACAATAACGAGGCTATATACAGGGGGCACCGGTACCGAGT
CAGTGTGCAGGGTTACAGGCTAGTTGTGGTAATCTGTACATGTAGGTGGAGACGAAGTGACTATGCATAGGTAACATACA
AACAGCGAGTAGCAGCAGTGTACAAGGAGGGAGGTGTCAATGTAAATTGTCCTGTGGCGATTTTTATGAATTGTTCAGCA
GTCTAATGGCTCGGGGGTAGAAGCTGTTGAGGAGCCTTTTGGTCCTAGACTTGGCGCTCCGGTATCGCTTGCCGTGCGGT
AGCAGAGAAACCAGTCTATAACTTGGGTGACTGTGGAGTCTCTGACAATTTTATGGGCTTTCCTCTGACACCGCCTATTA
TATGGGTCCTGGATGGCAGGAAGCTTGGCCCGAGTGATGTACTGGGCCGTTCGCACTACCCTCGGTTGCGCCTTACGCCT
TATCCCATGGTGGCGGTGGGGTTATGGTATGGGCAGGCATAAGCTATGGACAACAAACACAATTGCACTTCAACAATGGC
AATTTGAATGCACAGAGATACCATGACGAGATCCTGAGGCCCATTGTCATACCATTCATCCACCGCCATCACCTCATGTT
TCAGCATGATAATGCACGGCCCCATGTCACAAGGATCTGTAAACTATTCCTGGAAGCTGAAAATGGCCCAGTTCTTCCAT
GGCCTGCATACTCACCAGACATGTTGTGTTTATATTTCTGTTCAGTACAACTGCACAAGAGACATGAAACTTACAGAGCA
AACATCGCACAGTGGGAGGATTATGGTCTCTATAGTTACAGTGCCATAGAGGATAACCCTATCTGTCACTATGGTAACAG
CACCATTTGCTAGCCAGGTTGTAGTGGCACTTGCTACAACAATATGCTAAAATGCTAATGACCTAAATTGTTTATGCAAA
TGTTCTTTTTGGTTCACAATTGTTGTGTCAACATTAATGTGGCTTTATTAACTGTTGCGTAATAATGAGTTAATTGTACA
TTTTTTGTTCACTGCATGCAGGTTTTCAGTGATAGGGGCAGCAGTACTTTATCATTAGAGACCCACTATTACTGTGGAG
AATGATTATGACATAGCATTTAGTTGGTAGGGTCAGCAATAATAGTACGGCACCCCTGGGGAAAATTAGGGTTAAGTGCC
TTGCTCAAGGGCACGTCGACGGATTTCCCACCTTGTCGGATCGGGTTGTACGCACAGAGAGTCTGTCTCAGCATGAAACC
GTCGCATGAAATAGAAACAATTCACAGTTTCATGCATTATAACAGGGTGGATAATCCAAGATCCTTTATCAGAATTCTAT
GGTAAACCTATAGTATTCCAAATGACTTTTACAGGATTACAGTCTTACCAATGGCCTGAAAGCGTTCCAGTGGGTAGGTC
ACACAGATGAAGTTCTGTCCGTTCTGTACACCACTACCTGGGTAGGAATATTGCCCTATTTCCTTTACAGGGGGGAAATG
AGGGGTGCTGTGGACCAACCAGTAGCCCTGTGTCTTGTCCAATAAGACAACACCTGAGACGGAGAGAGGGAGATAACAGT
CACTCATTGAAAACGGAGTCATTTCACAACAGTACTTTCTTGACAGACAAAAACATTAACATACAACAAATGGAGTAGCT
GGCCAGATACCTGGGTGCTGAGATTAACATTACAATGTGATGCCTGTATACAGTATACAGCTGAGGTTTATATTGTCCTG
ACCTTTAGTGTGGCCACCTCGGTTACTAAGGAAACCAAACCCTCTTTCTCCCTCCTCGGGTGGTCTCTGGTCATTGTACA
GGATGTATGCAATGTCAGCATTCTGCAAGGGAAAGTACACACACTCATCATCAGAAGATATACACTTCCATGGATATTGT
ACACTCTAAACTCAGAAACAAGGGTTATAGTGTCAATAGTGTTTGTTCTTTGATAACGTCATATGCTGTATTTATTTATC
ATGGCTGTCTGGGTGAAAAGAAAACTCCCATTGCACCTTAGTGAAAATTAGACAGTGAAACAAACAACCCACAAAAAGTT
ATAGTTCCACATTTTTATTTTTGTGTCATTCTGTCTCATTCCACTTTGACCCCAAAAAATTCAGCAGTTGTAAATACTGC
TATGTGTTACTGCAAATACAACTGTTAAGGGAAAACACTCAAAAACTATCTTTTGGTATTTTTTTCATTAGTCCACTGTT
GATACAGTCCCAAAACGGTTTACCATGTCAGCAGTCAAGTTTCGAGATATTGAACTTCCAAGAAACAAAATGTCACCGGC
CATATCATCATGAAAAAAACATTTTGGACCTGTATCAACAGATGGTTTTTGAGTGGATTTTCCCTTTAAGTTTCACAGCT
CATTCAAATGTGGTCTGATATTTATACTAGAAGGAACCAGACCTTCCCCTGCTCATACAGCTGGCCCACAGTCCTCCCCA
GGGCTCCAGTGCTGTCATTCACCAACACGTTTCCATCCGTCCATCCCTCGCTCCCTTTGTCCATCAGTAGATAACTCTGT
CCATCGTTGGGAAATTCACGATCAACATGGGGGAGTTTGTAGAGATAGAACCTGAGAGAGAGCAACCGAGAGAGAGAGAA
GGAGAGATAGAGTTTGATTCTCAGTACTTTGTGTTGTTTTAGCATACCTTGCTATGCACACTGTCTGTGACTAAGTAGCT
AACAGACCTAAGGGAAGAAGGAGGTCATATCAGTTCAATCTTCAGTCTGCCCAAAGCTGTTTTCTCACCAGTCTACAGCT
TCCCCTTGATCATTGTAACAGGAGATAGGTGAGGTGCCTCCCTCTCCTGGTTGAAACAGGATCAGCAGGACAACAAGTGA
CAACATCTGAAGCAAAAGCAATGAGGTCAAAATAGTTTCGATGTTACAATTATGTTACAATGTTGTTACAAATGTATCAG
AAGTAAACTAACAAAAAGTATCCGATTTTTCATTGAGTTGATATCTAATTGATTTGTCAACTGGTTTAGAAAGAATTAGA
AAGAAGTATAACCCATGCAAACGAAACTTATATCCCCGTTAAACTCAACTCTGGACCTCGAAGCCAGTTCCACTACGTTT
TTTAGTTCCCCTCTAATCAGGGGGGACTGATTCAGACCCAGGTCTGCGCAAATAATGATCAGATAGAACAGAAATCCAAC
AGGCTCCGGACCTCGTAGGAAAAGAAAGAGTTGAATACACCTGCTATAGCCTATACCAAATGTGCCCCACTGTAAGGTTT
ATTGAAAAGTCTAATTTCTACATCAACACTTATCCTACCTTTGAGTAGATAAGAACTTGACAAGTACGCTGATTCTACAG
CTTTTCTGTATCCGCGTGATCTTTGATTGTGGCGAATTATTCCATTTCTAAAGTCGAGACGCATCCTTGTTTTGAAGTAT
GATGTCACGGGGCGGGCTTACGGTCACGAGTTAATGACAATAGTTTGCATTAGCTACCCTTCTGTTAGCTCTGTTCCAGG
GCGCCTTCAAAGCCTAGTGGAGGAACTCGCACTAACGCCCTGGACCATAGGCACCCTTCTGACTTTTACACATGGGGAAC
AACCGAGGTGAAGACAGTTTGAGGTTGGATATTCGACGGATATTCGCCTGTAGTTTTCCTTACGTCAACCAACACCAGTT
AGGGACCGTCAACATAGTGACAAATCAAATTTTTCAAATTTCAATAACTCTTTAACCATTATTCCCAAAACTTTCAAAAC
TGGTTGTAGCTAACCCGATGGCATAGACACACATTGCACACACTTCTTTTTGTCAATTTACATCTCGCACTATTTAAAAT
AATTTATTTACATTTTTGAGTTTCAATAGCTCATTGGTCATATGACTTAGGGACTTCAAACAAGGTTCAGAATGTCCACT
GACTACCCTTCTGTATTCCACACCCTTTAGTTTGTCCATTTTCATCTCACACGTTTTTACATGAATTTTTCTACATTTAA
AATTCAATATCTCCTTGGTCATGTGACTTAGTGACTTCAAACAAGGTTCAGAATGTCCACTCAGTGGGCCTACACATTGC
ACATCCTTTAGTTTGTCCATTTTCATCTCACACGTTTTTACATTAATTATTCTACATTTAACATTTCAATAACTCCTTGG
TCATGTGACCTAGTGACTTCAAATAAGGTTCAGAATGTCCACTGCCTACCCTTCTATATTGTACACCCTTTAGTTTGTCA
ATTTTCATCTCACATGATTTTACATGAATTTTTCCAAATTTAGAATTTCAATAGCTCCTTGGTCATGTGACCTACTGACC
TCAAACAAGGTTCAGAATGTCCACTGACTATACCTTCATATCGCACACTCTGATGTTTGTCTACTTTCATCTCATGCTAT
TAGACTTAAATCTGTAAGCTTTGTAGGCCTTCATTTTACATGGGTGTTAAAAAAATGATGTTAACAAATGTTCCATCCTC
GCAATAACTATCTTTCACATGGACACTGAAAAGATCCGCAAATGGCTGTATGTGGGATGGATCAAGGACAGGACAGGTGT

TCAAACAGAGGTGCTTAAAGGAAGGCGCACTGGTAGGCCTCATGAAAAACAATGTTTCATATGCTCTTTTTAATCACAGT
AATAGGCAGTGATTTGTCAGTCAGAGTGAGCTTGATAACGTGAAAGAATCCTTTTCATAGCATCACTTTCATATCCTGTA
CATTAAGACAAATCCTTCTACGTTGAGTATTAGAACGCAGTTTACACAACCTGATAATGACTTGATATTTGAGTGCATTC
ACAACAAGCCACCTGCAGACAACTTACAAAATGCAATATTCCATTTGAGGGTTTGTTTTTCTGATGAAAATAGTTATTCG
ATACATGGCCTACTATTTCTAACTGGAAAAATTAATTCAAAGTCTTGTTTGAGTAAAATCCTTTTTCCAAAATTGATTTA
GGTACATTTTTGTGAAGATGTATGAGGGTTGTGATATGGGTCATTTAATAGTAAAATCATTTAAGGGATCAATACATTTC
TATATTGCTTCCCCAGTATCTTCATGAACCATCAGGCCAAGTGTTTTTGGTTGACCCTGCTGGACAGAAAGAGAAGGAGG
AATCGGAACACGCTGCATTCAAATTCAGGTCTTAGTTATTCCAATGTACTGGATCTAATTCACATTAGCATTTTACATAC
ATTCATAAGTGTAATAATTTGTTATGACATATTGTGAAAAACTCTCTTGGCTGCTGGCTCTGTCACTTTCAGACATGCGC
AGAGCAGACTGAAAGGGGAAGGATAGCTCTTGACTTGAACCACAGGGGTTCTCTGTAAAGAGAGAGTAATCCAAAAGGCA
CAGACACACCCCTTGACTTTCAATTGGCACCGCTGACCTGTATGTATTCTCTCCTGATTGGCTCTGTGGTGCACAGTCTG
GCAGTCTGACTAAAGTGCCAGAGGTATGAGGAGAGACATCCTCCTTTGTATTTATGGGAACAAATATTTCCTAACACTTT
GGATCCTATTTTTGGACAGTTAGAAAACACAATGCATCAATGCAAAACATTTTTTATTACTAATTACTGTCCATGAGTAA
CCTCAACCTTGTGGGTCTGTGGGTGTTTGGGCCAATAAAGTGCCAACTCAATTCTACCAATGACTAGTCTCTCATGCCCC
TATGAACGGGGACACAGGGAAATAGTTTTTAATCTAAACCAGCCCTTTTTTACTGGAGTACACTAACCCTAATTGGGGCT
CTTTTCTTGACACACAGTAGCAGTTTACCAGATAAGAAGTAAAGAAGATCTATCTTCAGAGTTTGTACTGTTTGGTGACC
TGAACTGGGATGTGCTTAACACCCCGGCCGTCCTACAATCTAAGCTAGATGCCCTCAATCTCACCCAACTGATCATGGAA
CCTACCAGGTACAACCCCAAATCTGTAAACTCGGGCACCCTCATAGATATCTTCCTGACCAACCTGTCCTCTAAATACAC
CTCTGCTGTCTTCAACCAGGATCTCAGCGATCACTGCCTCATTGCCTGCATCCGTAATGGGTCTGCGGTCAAACGACCAC
CCCTCATCACAGTCAAACACTCCCTAAAACACTTCAGCGAGCAGGCCTTTCTAATCGTCCTGGAAAGATATTGACCTCAT
TCCGTCAGTAGAGGATGCCTGGTTATTTTTTTAAAGTGCTTTCCTCACCATCTTAAATAAGCTTGTCCCATTCAAAAAAT
GTTGAACCAGGAACAGATATAGCCCTTGGTTCACTCCAGACCTGACTGCCCTTGACCAGCATAAACACATCCTGTGGCAT
ACTGCATTAGCATTGAATAGCCCCCGTGATATGTAACTTTTCAGGGAAGTTAGGAACCAATATACACAGGCAGTTAGGAA
AGCAAAGGCTAGCTTTTTCAAACAGAAATTTTCATCCTGTAGCACAAACTCAAAAAAGTTCTGGGACACTATAAAATCCA
ATGAGAATAAGAGCACCTCCTCCCAGCTGCCCAATGCACTGAGTCTAGGAAACACTGTCACCACCGATAAATCTATGATT
ATTGAGAATTTAAATAAGCATTTTTCTACGGCTGGCCTTGCTTTCCACCTGACTACCCCTACCCTGGTCAACAGCTCTGC
ACCCCCCACAGCAACTTGCCCAAGCCTCCCCTATTTCTCCTTCACCCAAATCCAGATAGCTGATGTTCTGAAAGAGCTGC
AAAATCTGGATCCCTACAAATCAGCTGGGCTAGACAATCTGGACCCTCTCTTGCAACCCCTATTACTAGCCTGTTCAACC
TCTCTTTCGTATCGTCTGAGATCCCCAAAGATTGGAAAGCTGCCGCGGTCATACCCCTCTTCAAAGGGGGAGACACTCTA
GACCCAAACTGTTACAGACCTATATCTATCCTACCCTGCCTTTCTAAGGTCTTCGAAAGCCAAGTTAATAAACAGATCAC
CGACCATTTCGAATCCCACCGTACATTCTCCGCTATGCAATCTGGTTTCCGAGCTGGTCATGGGTGCACCTCAGCCACGC
TCAAGGTCCTAAACGATATCATAATCGCCATCGACAAAAGACAATTTCCCCAATTTCGTGGTATCCAATTGGTAGTAGTT
ACAGTCTTGTCTCATCGCTGCAACTCCCGTACGGACTCGGGAGAGGCGAAGGTCGAGAGCCATGCGTCCTCCAAAACACA
ACCCAACCAAGCCGCACTGCTTCTTGACACAATGCCCATCCAACCCGGAAGCCAGCCGCACCAATGTGTCGGAGGAAACA
CCATACACCTGGCGACCGTGTCAGCGTGCACTGCGCCCTGCCCGCCACAGGAGTCGCTATGTTTGAAGTCAGTAGGTCAC
ACGACCAAGGAACTATTGAAATTTTACATTTAGAAAAATTAATGTAAAACCATGTGAGATGAAAATGGACAAACTAAAGG
GTGTGCAATGTGTAGTCCCACTCAGTGGACATTCTGAACCTTGTTTGAAGTCAGTAGGTCACATGACCAATGAGCTATTG
AAATTTGAATGTAAAAATAGTTTGTACTTTGTTCACGCTCCCTAACTATTTCAACTAGGAAAACAAAATGCTGCTCACAT
TTCCACATGTTTATTAGCTTTGGAAAGCGAATTAACGCTCAAATCATGAAAATAAGACTTGTGCAATGTTGTGCGCAATT
TTTCCAACATCATGACACTTATTTGGTGTCTGTGGCTCAAGTGGTTTGAAAGCTATTGAGGTTTGAAAGTGCGAATATCC
GTTGAATATCCAACTTGAAACTGTCTTTACCTCGGTTGGGGACCATCTATTTATTTAAATGGAAGAGCATATATGACCAT
AAAGCAAGCCGCAAACGTATGATACATACTTATTGGATACATATTTAATGAACAAGTGTGTTTTCCTTGCTGTTGTCAAC
AATGGTTGAACAGTTTAGGGATGGAGTTGATACATTTCCACTGTCCTGTGGAAAATTAATCCACAGCACATATAGGCTAG
CCTAACAGCCCAGTTGTCTAGAAAAGTTAGTTTCTGTTAACAGCACTAGTGCATTGCAATAGTGTAATGTAAAGGCAGAT
GTCTTTCGTTTGATAAATTAATAATGAATATTGAGTGGTCCAGTAGACAAAGACTTTATCGTAAGCGTTCTCGGGGAACA
ACTAGAAACACTCTTGATGCAAGTCAAACATTTAACACATAGTTTATTCTCGTGAGGAGCTGTATTCAAATATAACATGG
AAACACAAACACTCTCATTCATACTCATTCACTGCTGTGCTGGGCAAAACGAATTCCCTCATATTGCGATAAACACATTT
AAAGCACCCCAAAGCTTAGGGGCATTTTGAATATCATCCAAATTATTGTGCAGTCAACATACAAATCTGCCCACTCTGTA
AGCTTAATATCAGACAACAAACATTCGAATTAGTCTCTATAGCATTATGCAGCTGAATTGACACCTCTATTATTCACATA
CAATTTTTTTGTTAAATTACTGATGCAGTCATCGATACAGATGGTACTGTCAGCTCAGACCATAACAGAACAAAATGGTC
ATGCCTGGTCTAGCCAGGTGAATAAGAAGCAAAAGCCATTGCACTATAGGGCTACACCACATGCTCTGAGCCATTTGAGC
CCATCTCTCCAGCATGTGTATCTCTCAGGTTCCCAGAATCCCAGGCAGCTCAGTCCTCTGGTCCTCCATGCGTCCTCCCT
GGACACTCAGCAGTAAGGAAAAGAAGTCTTCATCATCCATAGGGCCCGGGGCCGCACTGCGCTGCTCCTCCAGCCTACCC
TGGGCCTGAAGTACAGAGACAGACAGAAACACCACATGGCTTGATGCCTATTTTACACTGGACCTGAGGTACGGAGACGA
GACATTACACCTACGTAACACCATATGACATGATAAGTTGTTACACCATACAACATAATACCTAATGCCCATGTTACAGC
ACATGACACACAGGTGTAGGATATTCATTTGACCAGTAAAATAATCCTGCAGCAACAGCATTTGAATGTTTAGTCCATAA
CGTTGCATGATCGGTGGTTAGGCTATAAACTGGCCAAAATTAAGCTACATGAAAAGGACAATACTGTTAATATAACTGTG
TGTTAGTGCGGGTTTTTGTTCATTTATGTAAAATCACGAAGCTCATCTGCATTTTCTGGGGGGCAGGAAAATTCTCAGCA
ACAACAGAGTGATCAAATGAAGATCCTTCATCTGTAATGACAATGTAGCACTGTAACATGATCTCAATGTAACACCATAT
TTACATTTACATTTGACATTTTAGTCATTTAGCAGACGCTCTTATCCAGAGCGACTTACAGTAGTGAATGCATACATTTC
ATACATTTTTTTTCTCCGTACTGTATGACATGATGCCCACATTATATTTTGCTTATGACAAGAACAAAATAACATTTTGT
TCAAATTGACTACTGGCAGCTTGGGTGATTACAGTTATTATAATTTTTTATTTAACCTTTTTTTAATTAGACAAGTCATT
TGAGAACACATTCTTAATTATCATGATGGCCAGTTACTTACCTGCGACATGAGAATCATATTGTACAAGTCCTCGTTCTG
CACTGCAATTGGAGCAGACTTTTTGGGCTTTGTATCTTTTACGCTGCTCCTCTTCTTATTGGCTTTCGGCTTAGGGGGTG
GGGTGTCATGTAGTTCCGCCCGCTGGTCGTCAAGCCGACGGCCTTGGGAAGTAGCCAGCAAGTCAAAGAACTCATCTTTC
TGCAGCGAGGTCATTGAGGAGAAATGCACTGTTGGGAATGGAAGGGGAGAAAAGAGAGTGGAGACATGCTACATTTAGTG
GCCTAGTTCATTTGGTGTCCTAGTTAATCATTCAAGCAATATTATTTCAATATTTCACTAATGTACAGAAGACATCCTAA
TATCTGTCCCTGCCACAGTTATATGATAGAAGTGCTTCAATGGATGTTGGTACCGTACAGTTATTTACAGCTTTTCCCCT
TCATGAGTCAGCGTGTTTATGGGGAAACAAGGGAAGCTAATTACAACAGCTGTTATGTTTACCACAGTCAGTTAGTTAGT

TATCATTACAGGGAGTTGTTTTCACATTCTATCACCTCTGTGGGCTCTGTGGGCTGGACTGTAGGTGTTGGGTTCAGAAT
GGCACCTTCTCCTCCCTCTGAACGAGCACCGCTGGTCATTGAGTCGTCTTCCTTCTTGCAGAGTAAAAACCAGGTCATAC
AGAGCGTCTGGGAAGTAAGGGGTTAACCGTTGAGCCTGAGAAAGACAGGTGGCAGGTGGCAGAAAGAGTAGCTATTATCA
AGGATTTCAAAATGGGTGGTATCACTACTCATTTCAAACACGCTGGCATTTCCACACCTACAAATTCTGTAACCACAAAA
TGAACCCCTCATTCTTCCACCACTGGAATGTTGTTTGTTCTTTTTATATATGTGAAAGAAAACCCTTCTACATCAAAAAC
TAATGCTACATCAAAATGTTTAGACTGGTATTCGGTCACTAACGGAATTATTGTAATTGTGGGCACAAGGGAAATTGCTG
TATTTTTTTGTGAAAGCAAATGTTATAAACCTGTTTTTGGGCATGCTCTGGGCTCAGCGTATCTTTGGGCAACTGGTCAA
AGGGAAGATGTATATTTGTGCCAACTTGTAATCCTTGTGATGGTTTGTCCGTCTCTTGTTCTGCTGTTTTGTATTTTTCT
GTTATATCCACCATCACCATCCCCTCTTTCTTGCAATCTATTTTCTGTCTATCCATTACCAATTCATCCATCCCTTTAAT
GAGCTCAACCCCCTCCTCTGTCCCAGTCTCTCCTTTTTCTTGATTTATTTGGTTATCCTTTTTTCTTTTGTCTCCATCTC
CACTTTCAGTGTTGGCTTTTCCAATCTGTCCATGTCTGGCAGTGGCATCCTCTTCCACACTGGAGTCTTCATCGTTGATC
TTGCCTGTTATTGGCTCTGTCCCTCCCTCAACAACTCTCTCAACATCCCCCTCCTTAATAATGACTAGATCCTCCAAGTG
CTCCTCTTCAGCTGTTTCAACTTTGACCCCTGGGTATTCCATGACNNNNNNNNNNNNNNNNNNNNNNNNNNNNNNNNNNN
NNNNNNNNNNNNNNNNNNNNNNNNNNNNNNNNNNNNNNNNNNNNNNNNNNNNNNNNNNNNNNNNNGATTGTGTAACTGGT
TGTAAGAAAATGTTTTAGGTAATCTAAAAGAGTTTCTTAAAATGTTAACTTGCAACAACACATGTAGAAGACTTCTAGTG
TGACACAGACTGAACGGCCACTGTATCTGTAAGCATAACAGTATAATCTCAAGAGGGGTGTTTATGAAAAACCAGTACTG
GTTTCCTGCTCACTTCTTTAAGGCACTAAATTCCTATCCTGGATTGTACTTGCCTGCCTGCTAATCATTACCTTACCCAC
CCACGCTCTGAATAAGTCTATCGTACCTGTGTTTTCCTGGAGTTCAGTTTCTGATAAACATCATCTTCTTGTGTCCACAC
TGATTAGGGATGGCATCACTGATTCACGACAGTTTTCTTCATGAGAACTAATTGCTATCAGTTATGCTTCACATATCACC
GTGACGAGCTCTTTCTCGCTCTCTCTTTCTCTGCCTGCCTGTCAGCCCTCTAGTCTCTTCCTGTTCCTCTTACCCCAACC
CACCCCTATTTATTTGTCCCCTATCTCTCTGCGTCATTGCATTATGAAATATTACTTTAAAAAGTGAAGTGGTGTGATGT
TATAGCCAGTGGTGCGATGTTATCAAATAGCCTTGTTTTTAACAAAGCCAAAACTCATTATTTGAGATGAACATCAGAAA
GGGGGAAATTACTATGAAGTTTTGCTCTGCTCATGTTGCATTTTCAGCAGAACAGCATACAGTCAGAAACAGCAACATGT
TGAAACAGGCCCACTGTTTTTCTGATAGGGGCTGTGGTTTGACATACTTCCTGTTGACTTGCAGCTAAAGGAGAAGTAGA
CAAAGTGTGTGAGAGAGAGAGAGAGAGAGGGAGAGAGTCAGATGTGAGATGTTGATTCTTTTTGGTGAAGTGAGAGAACA
GAGGGGAGGGTAATTGTTCATTTGTGTGTATTCATATATGACTTGTCTGAACTATTCTGTAGTTCAAAAAGTGTTCTGCT
GTTTATCATCCATTGATGTATAAAACTTGATCACTGTGAATTAATTTGTCAAATTTGACAAATCTGTTAATATCATGGAG
TGCATGTATTGTCCCGTGTGGTGAGAGTAGGCAACAACACATCTGCCACGCTGACCCTCAAACTCTTTTGACTCATCACA
TACACAGCTGCTACTGCTTATTATCTGTCCTCTTGCCTAGTCACTTTACCCCTACCTACAGTTGAAGTCGGAAGTTTACA
TACACCTTAGCCAAATACATTTAAACTCAGTTTTTCACAATTCCTGACATTTAATCCTAGTAAAAATTCCCTGTCTTAGG
TCAGTTAGGATCACCAGTTTATTTTAAGAACTTGAAATGTCAGAATAATAGTAGAGAGAATGATTTATTTCAGCTTTTAT
TTATTTCATCACATTCCCAGTGGGTCAGAAGTTTACATACACTCAATTAGTATTTGGTAGCATTGCCTTTAAATTGTTTA
ACTTGGGTCAAACGTTTCAGGTAGCCTTCCACAAGCTTCACACAATAAGTTGGGTGAATTTTGGCCCATTCCTCCTGACA
GAGCTGGTTTAACTGAGTCAAGTTTGTAGGCCTCCTTGCTCACACACGCTTTTTCAGTTCCGCCTATACATTTTCTATGG
GATTGAGGTCAGGGCTTTGTGATGGCCACTCCAATACCTTGACTTTGTTGTCCTTAAGCCATTTTGCCACAACTTTGGAA
GTATGCTTGGGGTCATTGTCCATTTGGAAGATCCATTTGCGACCAAGCTTTAACTTCCTGACTGATGTCTTGAGATGTTG
CTTCAATATATCCACATCATTTCCCTACCTAATGACATTTATTTTGTGAAGTGCACCAGTCCCTCCTGCAGCAAAGCACC
CCACAACATGATGCTGCCACCCCCGTGCTTCACAGTTGGGATGGTGTTCTTCGGCTTGCAAGCCTTCCCCTTTTTCCTCC
AAACATAACAATGGTCATTATGGCCAAACAGTTCTATTTTTGTTTCATCAGACCAGAGGATGTTTCTCAAAAAATATGAT
TTTTGTCCCCATGTGCAGTTGCAAACTGTAGTCTGGCTTTTTTATGGCAGTTTTGGAGCAGTGGCTTCCTCCTTGCTGAG
CGGCCTTTCAGGTTATGTCAATATAGGGCTCATTTTACTGTGGATATAGATACTTTTGTACCTGTTTCCTCCAGCATCTT
CACAAGGTCCTTTGCTGTTGTTCTGAGATTGATTTGCAATTTTCGCACCAAAGTACGTTCATCTCTAGGAGACAGAACGT
GTCTCCTTCCTGAGGGGTTGTAACGGTTGTTGTCGGTGAAGGAGGACCAATATGCAGCAGGTACGTGAATACTCATCTTT
AATTAAAATAAAGGAGTAAAGCATCCACTTAAAAATTCAAACAAAGTAACAACAGCTACAGTTCTGCAGGCTAGAAACGC
AGTGCAAAAAACAACCACCCACAACCCCAAAGAAAAACACACACACCTATATAGGACTTCCAATCAAAGGCAACTATACA
CACCTGGCTTCAATTGGAAGTCCCAATCATCCACCCAACATTTAACCAATACAAACATGCCACGTCCTGACCCCAAAACT
AAAACACTAGCTCCATCTGCTGGTCAGGACGTGACAGGGGTATGATGGCTGCGTGGTCCCATGGTGTTTATACTTGCGTA
CTATTGTTTGTTCAGATGAACGTGGTACCTTCAAGCATTTGGAAATTTCTCCCAAGAATGAACCAGACTTGTGGAGGTCT
ACAATTTTTTTCTGAGGTCTTGGCTGATTTCTTTTGATTTTCCCATGATGCCAAGCAAAGAGGTAGGTTTGAAGGTAGGC
CTTGAAATACATCCACAGGTACACCTCCAATTGACTCAAATGGTGTCAATTAGCCTATCTAAAGCCATGACATAATTTTC
TGGAATTTTCCAAGCTGTTTAAAGGCACAGTCAACTTAGTGTATGTAAACTTCTGACCCACTGGAATTGTGATACAGTGA
ATTATATGTGAAATAATCTGTCTGTAAAGAATTGTTGGAAACGTTACTTGTGTCATGCATAAAGTAGATATCCTAACCGA
TTTGCCAAAACTATAGTTTGTTTACAAGAAATTTGTGGAGTGGTTGAAAAACGATCTTTAATGACTCCAACCTAAGAGTA
TTTAAACTTCCGACTTCAACTGTATGTGTAAATATTCCTCAGTTACCTCCTAGTAACCTTTGCATGTCAACTCAACAGGA
TTCAATTCTCGGGCCGACTCTTTTCTCTGTATACCGTAAATTCCGGACTATAAACTGCAATTTTATTCCCAGGCTTTGAA
CCTCGCGGCTTAAACAATGACGCGGCTAATGTATGGATTTTTCCCGCTTTCAAATTTTTTTCTTCCAAAAAAACACATTC
TGTGACGTGCTCAGTTTTTTGGCGGCATAAAGCTTTCATTAGACCAATGAAATTGCCGAACGGGTTAAGGTCTTAAGGTC
AAACAACTTTTTTGTTTACTGTTTAGATTAAATCGAGCGCTCTCAAACTTCCCATCATTCTGATTATGGTAGTCATTTTG
TCACCCTCATCATGGCAAAGACACGGAGAAATGCATATGATGCAGCTTTCAAGTTGAAGGCGATTGATCTGGCTGTTGGA
AAAGGAAATAGAGCTGCTGCATGGGAGCATGGTCTTAATGAGTCGATGATAAGACGTTGGAAACAGCAGCGTGAGGAATT
GACTCAGTGCAAAAAGACAACTTACTACAAATGTTTTATTTTTTGTTACAAGTCGTGTTTCGTTAAAACCTATTTATTTT
TGTTACAAGCCGTGTTTCGTTAAAGCCTATTTATTTTTGTTACAAGCCGTGTTTCGTTAAAGCCTGTGTAAAGTTCATTT
GTTTCAATGTAGCAGTAGGCACCTGCGGCTTATAGACATGTGCGGCTTATTTATGTTAAAAATAAAAATAAAAATGTAAT
TCAGTGGGTGCGGCTTATATTCAGGTGCGCTTAATAATAGTCCGGAAATTACGGTATATCAATGATGTCACTCTTGCTGC
AGGGGATTCCCTGATCCACTTCTACGCAGACGACACCATTCTGTATACATCTGGCCCTTCTTTGGACACTGTGTTAACAA
ACCTCCAAACGAGCTTCAATGCCATACAACACTCCTTCCGTGGCCTCCAACTGCTCTTAAACGCTAGTAAAACTAAATGC
ATGCTTTTCAACCAATCGCTGCCGGCATCCGCCCGTCCGACTAGCATCACTACTCTGGACGCTTTTGACTTAAAATATGT
GGACAACTACAAATACCTAGGTGTCTGGCTAGACTGTAAACTCTCCTTCCAGACTCATATTAAACATCTCCAATCCAAAA
TGAAATCTAGAATCGGCTTCCTATTTCGCAACAAAGCCTCCTTCACTCACGCCGCCAAACATATCCTCGTAAAACTGACT

ATCCTACCGATCCGGCGATGTCATTTACAAAATAGACTCCAACACTCTACTCAGCAAACTGGATGCAGTCTATCCCAGTG
CCATCCGTTTCGTCACCAAAGCCCCATATACCACCCACCACTGCGACCTGTATGCTCTCGTCGGCTGGCCCTCGCTACAT
ATTCGTCGTCAGACTCACAGGCTCCAGGTCATCTATAAGTCTTTGCTAGGTAAAGCTCCGCTTTATCTCAGCTCACTGGT
CATGACAACAACACCCACCCGTAGCACGCGCTCCAGCAGGTATATCTCACTGGTCATCCCCAAAGCCAACACCCCCTTTG
GCCGGCTTTCCTTCCAGTTCTCTGCTGACAATGACTGGAACGAATTGCAAAAATCGCTGAAGTTGGAGACTTATATCTCC
CTCACTAACTTTAAACATCAGCTATCTGAGCAGCTAACTGATCGCTGCAGCTGTACACAGCCCATCTGTAAATAGCCCAT
CCTATCTACCTACCTCATCCCCATATTGTTTTTATTTACTTTTTTCTCTTTTGCACACCAGTATTTTCTACTTGCACATC
ATCATCTGCACATCTATCACTCCAATGTTAATTTGCTAAATTGTAATTACTTCGCTACTATGGCCTATTTATGGCCTTAC
CTCCTCATGCCATTTGCACACATTGTATATAGACTTTTTTATTTTATTTCTTCTATTGTGTTATTGACTGTACGTTTGT
TTATTCCATGTGTAACTCTGTGTTGTTGTTTGTGTCACACCACTTTGCTTTATCTTGGCCAGGTCGCAGTTGTAAATGCG
AACTTGTTCTCAACTGGCCTACCTGGTTAAACAATGTGAAATAAAAAATAAATAAATAAACTCTGTACTGGTAACCTGTG
TATATAGCCAAGTTATCATTACCCATGGTGTATTTATTCCTTGTGTTATTATTTTTCTATTTTTGTCTCTACATTGTTGG
GAAGGGCCCGGAAGTAAGAATTTCACTGTTAGTCTACACCTGTTGTTTACGAAGCATGTGACAAAGGTCCCGAGAGTAAG
GATTTCACTTTTAGTCTACATCTGTTGTTTACGAAGCATGACAAATAAAAAGTGATGAAAAAAATTGTTGATGATTTGAT
TGGTAAAGACCTGTGACCTTATTATGAAGCTGATGCATTATTACATTAACTGATATGAAATCAGAAGATGATCCCTCCCC
CCATCATGGTGCAAATGCTCAATGTATTAGGCTACTTGCAGATTGAAAGACCCCCCTCTTCTCTCTTTTTCTGTTTCTCT
CTCTTTTACACATAGGCCTACACACACCGTTGCCTCATAGTTATATCCATCTACCTGATTGTTCATAATAATTACTTAGT
TTAAAAGTCATAGCTATGAATATCCCTAGTACAGTAGCCTATGGGCTCTGTCTGGAGTACAATTCGAATTTTATGAACAT
TCTTGTTCGAGACTTTTTCTTTGTCAATCGCTAGGGGACTCTAGTGCAGGCCTGCTCTAGTGTTTGAGTTTTGAAGGGTG
TGTGACTGTGATTACTCGTCGGCGCCACTGTGTGCCGCTCAGGATGTTGCAGCAGCAGCCTCTCACTGGCAACGGGCCCT
CTAACGGCCGGGGCCTTGGCCTGAGCTCTCACCCCGGTATGCACCCTCTAAACTCGGTTCATCCATCTTCCCACCACCGC
TCCGACGGAGACACGGGCCACGAAGGCGCAGAAAATGGACATGAAAGCCGCAGAGACATTGGTGACATATTGCAACAAAT
CATGACCATCACCGACCAAAGTTTGGACGAAGCACAAGCAAAGTTAGTCACAATTGTTTATAACTTAATCAGATTTAGCT
ACACATTTCTGCAAATTTGCTCAATGGCCAAGAGTTAGCTAAAGTTTTCAGAAGTTTTCAATTTGATCTACTTTGAAGTT
TTTTAATAAGCTTGCTAGGTAGCCAACGTTGCTAGCTAGCTAACTGGCTTTGTGTCATGATACGTTGTTTATTAAATAGT
TATTACTTCTTCTCCTGGCCTATTTACACGCATGTTGTTGTTGTGAAATGTATAAAAACCGAAGTTGTTTTTGTGGCTAA
CTCGATGAAGAGGGTGAATTCTTGCTAAGCTAAAACTGGCCAGTTAGCTAGCTATGCTCTATTGCTTTATTGTTAGCTTG
CTAATGTGTTACTACTAACGCGTTAGCCGGTTAGATTTACATGTTAGTGAATAGCAAGATATTATAAACGGATTGTGGCC
ATAATCTTGGCATTATCCATTTCATGCTACATTTGTTCTACGAATAAAATGTTAACATATTAGCTACATATTGTAGCTGC
TGCTAGCTAGCCATAGCATTACGTAGCTAACGTTAGCATAGCAGGCTGCAGTGAACTCCAGTCAAAAGGTCACGAACAAC
AGAGCCGATGTTTGTTGTTGTGTATGACATCTAGTTAACGTTAGCTAGCTATATAAAGCGTGTGAAAGCAGGGAGCTAGC
TAACTAAACAAATATTTTGTTGGTGCTAATGGCTCGTTGCTAAATAGCTGGCTAGATATACAGCTACAGTAGCAACGTTA
GTTAGCTAATAATAATAATGTTTTTTTCCAATCGTCAGAAAACACGCATTGAATTGCCATCGAATGAAACCAGCTTTGTT
CAGCGTCTTATGTGAGATCAAAGAGAAAACAGGTAAGTTCATTTTCACTAATTGAAACCCTTTGTTTGATTTTAAACTTG
TTGCTAATTCGTTTAAAAGTTTCTGGCCATCTGTGATGAAAACAGAAGATGATGTTGACTCCATCACAATAATAATGAGT
TGCGCATAATCCATGCAGTTAGATGACAGTTAGACAATTCTCTAGCTATTTTACATTTTCAACAAAGATGTATTGTGTAT
GATGACTTTTCAAATATTGCCGCAAGATGACCCTTCCTTTATATGTTTCATCCACAAAGTGCTTCTCCAAGTCTGTACCT
CAGAGGGATAATATTTGAATATACACTGAACAAAAATGTAAACGCAACATGTAAAGTGTCCCATGTTTCATGAGCTGAAA
TAAAATATCCCAGAAATTTTCCATATGCATTTTTGTGCACAAATTTGTTTACATCCCTGTTAGTGAGCATTTCTCTTTGC
CAAGATAATCAATCCACCTGACAGGTGTGGCATATCAGGAAGCTGATTAAACTGCATGATCATTACACAGGTGCACCATG
TGCGGAGGACAATAAAAGGCCACTCTAAAATGTGCAGTTATGTCACAGATAACTCTAGGTAGTGTGCAATTGGTATGCAG
ACTGCAGGAATGTCCACCCGAGCTGTTGCTAGAGAAATGAATGTTCATTTCTCTACCATAAGCCGCCTCTAACGTTGTTT
TAGAGAATTTGGCGGTACATCCAACCAGCCTCACAAAGCAGACCATGTGTAACCACGCCAGCCCAGGACCTCCACATCCG
GCTTCTTCACCTGTGGGATCGTCTGAGACCAGCCATCCGGACTGGTGATGAAACTGTGGGAATGCACAACAGAAGAATTT
CTTGACCTGACTTCAGTTCGGCAGCGTAATTGACTTCAGTGGGCAAATGCTCACCTTCGATGGATATGGCACGCGGGAGA
AGTGTGCTCTTTGCGGATGAATCCCGGTTTTCACTGTACCGGGCAGATGGCAGACAGTGTGTATGGCGTCATGTGGGCAA
GTGGTTTGCTGATGTCAGCGTTGTGAACAGAGCACCCTATGGTGGCGGTGGGATTATGGTATGGGCTACGAACAACGAAC
ACAATTGCATTTTATCTATGGAAATTTGAATGCACAGAGATACCGTGACGAGATTCTGAGGCCCATTGTTGTGCCATTCA
TCTGCCGCCATCATCTCATGTTTCAGCATGATGATGCAAGACCTCATGTCACAAGGATCTGCACACAGTTCCTGGTAGCT
GAAAATGTCCCAGTTCTTCCATGGCCTGCACAATGTCACCCATTGTTTGGGATGCTCTGGATTGACGTGTACGACAGTGT
TTTCCAGTTACTGCCAATATTCAGCAACTTCACACAGCCATTGAAGAGGAGTGGCACAACATTCCAGAGGCCACAATAAA
CAGCCTGATCAACTCAATGTGAAGGAGATGTGTCACGCTGCATGAGGCAAATGGTGGCTACACCAGATTCGGTCAGGCTG
GTTTTCATATCCACGCCCCTACTTTTTTTGTATATCTGTATTCCCAGTCATGTGAAATCCATAGATTAGGGCCTAATGAA
TGTATTTCAATTGACATATTACCTTATATGAACTGTAACTCAGTAATATCTTTGAAATTGTTGCATGCTGCATTGATATT
TTTGTTCAGTGTAATAGGAGACATTGTAACTCTTAATTTACCATGAATCAGATTGACAGACCTCTCTTGTTGCAGGCCTA
TCAATGCGAAATGCTCAGGAGGAAGAATCACAGGACCCACAGCTGGTGCGATTGGACAACATGCTGCTGGCGGAGGGTGT
GGCTGGGCCGGAGAAAGGTGGCGGGGCGGCAGCGGCGGTGTCCGCAGCCACCAGCTCTGGAGGGATGTCGCCAGACAGCT
CACTGGAACACTCAGACTACAAAAGCAAGTTGAGCCAGATTCGCAACATCTATCACACTGAGATGGAGAAATATGACCAG
GTACTTTTCTAGTTGTTGCTTTTTTCAAGCAGGGGGGCAGTCCCAATCCTTTTGTATTGTTTTTTTGCATTATTGAAGAG
ATAGTAAAGTGTTGGAAAGACGGTTAAGATAGAGGGAGGGTGAGTCAAAGAGCAGTGGGCCGGATTCAAACTCATATCGA
CTCTGACATACACGTGTGCTGGGGTCAGTGGCTGTACTTGATTTGAGGTGGAGGGACTTGACACTTTGCACACACTGAAA
ATTACACCCTTTCGCGAAGTAAACTAAAATGAGGTGATGTTTTAATTTAGGTGAGATAATTGAACGTGAACATAAATGAC
CGACGTCAACAGTGTCGGCCCGCTGTTGACTTCCATCCTTTGAGCTCAACTGACGGTCATATCTGTCTCCAATCCCCCCT
CTCTCTCGCTCTCCGTGTCTCCTGTAGGCATGTAGTGAGTTCACTACCCACGTGATGAACCTGCTGAGGGAGCAGTCCCG
AACGCGGCCGGTGTCACCGAGGGAGATCGAGCGCATGGTGGCCATCATCCACCGCAAGTTCAGCTCCATCCAGACACAAC
TCAAGCAGAGCACCTGCGAGGCCGTTATGATCCTGAGGTCCCGCTTCCTCGACGCCAGGTCAGCGTGTAACACTGTTATT
GGGGTGACACAACACCTGTCAAAACCTGCCATGACAGTGAATGACAATTCATGTACAGGTAGTATTTTTAATGAAGAGTA
AAACATTCCATAAATGAACAGTGGTTCATGGTCATACCAGTCATATTTACAGTGCATAATCATGTTATTACAGTGCAGGT
TGTGAATGACTATTGTGTGTCAATATCAGTTGTTACTGTAGTTGTGAAATGTATACTTCTGCCTGGGTTGCATGCCCACT

CTCAACACACTCACCTTTGTGTTGTCAGGCGTAAGAGACGTAACTTCAGCAAGCAGGCGACGGAAGTCCTGAACGAGTAT
TTCTACTCCCATCTCGCCAACCCTTACCCCAGTGAAGAGGCCAAAGAAGAACTAGCCAAACAGTGTAGCATCACTGTCTC
TCAGGTAACCTTGTGTCATAAGTATAAGTATATGCGTCATATATTTACTATTAGCTCACCCATTGATACGTCCATATACA
GTTGACTCCATTGACCAGCCTCTTACTGGCTCGTTAATTGAACAGGTGTGCAACTGGTTTGGCAACAAGAGAATCCGCTA
CAAGAAGAACATTGGGAAGTTCCAAGAGGAGGCCAACATCTATGCCATGAAGACGGCCATCGTGGCGACACAGAACGAGG
ACTCTCCACACACGCCCAACTCAACAGGTAAGAGCTGAGGAAAGGAAGCCAGGCCTAGTGATACACACTGTTTTGAGTGT
ATGGTTCAATTGGTAGTAGAAATCGATGGGTTGTTAGTGTTTTTGTGTTACGGATCAGCAAGGGGAACTGCCCCTCCATA
CCTTCAGACTATGCTCAAACCTTACACTCCAACCCGCTCTGTCACCTCTGGTCTCTTGACGCTCCCACTCATCCCATTCA
AAGCTCTTTTCTTTTCATAAATACTTTCCTACCCTAAATAATCTACAAGATCAGAGTATTTTTTGAAATCTACCAATTGG
TGCTGAAACGGAGACGAATATGCATATATTTAGATGTCTTTCTTTGATTGATCCCTAATATTCCGTACCAATTCTCTCAA
TATATTCTGTTGAGTCTGTCAACAAAATTCGAACTTAAAGGTACACCTTATTTAAAGGGATGGCTTAAAATAAATGTGCT
GAAAACCCTATTGAATGAAAACTCCACAAGAAAATCGGTTGACCAGCAAGTGGGAGGATTTTCAGCGGTTGAATTACATT
TAATCAGCGCATGCAAGGGAACCATGTCTGCAAAGCCCGTTACGCAACTGAATAAGATATGTCTGAATTACAACTACTGA
GAAGTGCGTAGGAAAGGCCTGCGTAAGAAAGGCGTCATTTTCTAAGTCGGAATTGGGCCTTTTGTGCTGTTGTGCCAACT
CCTTGCCCAACAATAGAAGTCGGTTTTACAGCGCAATCAGATCTGGGACCAGGCTGATGTTGTAGGCCTAGTAGCCCCTA
TTAAAGACGTCTCCCCCTTTGTTCCTCCACAGGATCGGGGGGCTTCTCACTCACTGGACCAGACCTTTTCCTGGGTGTTC
CACCAATGAATGGAGAGCAGCCTGTCTTCATGGGAGCACAAGTATGGAACTTCACTGTCATTCACTTCATGTTACATATC
ATCTTTATTTTAGCTCGTATCTAATTGAGAGCACATTCTCATTCACAACAACAACCTTGTGGAGGAGTTGCATTGGAGTG
GAGCGGGGTTAATGTAGGTTAATGCCACTACAAGTCACCACGTCTTTTTCTCTTTGGCAGACTAATGGGAACTGGCAAGG
GCAAAACACTCCCCCCTCTGCCACTTCCCCAGGAAACGACCACTCAGGAGACTCTGACTGATGGCCTCGCCCACTTTGTG
TTGATGACATAGATGGAAAGATTGACAACGGAAAAAGACTGAAAGAATGATATCACTCTTACTTGGCTGCAGTTTTTGTA
TGTCTTTTCTGTTTCTTATTGTCCAATGATACTGTTCATTATGTTTGTATGAGCTGAGTGTGTGTGTGTGTGTGATGTTT
TGTATTGGGATCATGGGTTTTGTTTTCCACTACTTTATATGTTACTGTGGTATGTGGGCAGGGAGGCTTGGGAAATTGCA
TTACCATTGTTTTATTAGGCTATATTATTAATGCTGTTACAAATATTTTAGGATTTTCGTTTAAAGCTCAGATATAGTTG
CATTTAGAGGATGTGGGGGCAATAGAGAAAATCCAAAGATTTTGTGTGTTTGTTTTCTCTTTTCTACTGGGCTGTTTTGT
CTGTGTGTGTCTATATCATGAATGAAGTGTGTGTGCTGGTTATGTTAACAGTTTGAATGAGTATATCAACAGTGCTGTCA
TTCTGTACAGTGAGTGAAAAACACCTGTATTCTCTCTACCTCTTTTACAGTAAAGACTCTGTATTCACAGTCAGTTGGTT
TGTCTTGATTGGTTGATATTAGCATAAGAGAGACAGGTCACCTCCTAATATGGCAGAATAAGAAAAAATTGAACATTATT
CGTTTATTGATTTAGATATTTAGAGTAAATTAAGTTACTCATTTATTGACTGAGTTTATTTCCCGTCTGTCAATCTACTG
TTATTTAGGGGAAATGGCCCTAAATGGCCTTTTACAGAAAATGTAAACAGCACATGATTGACATGATGGTATTTGGGGTA
ATTTCAGCATGATATCTCACAGCAGAGCCCTCTCCCTTACAAAGCTGTTTGGTTAGAGTCAGGCATGTGGTTATTTAGCT
TCTCACTTCGGGCTTTCTGGTCTGCCTTTTTAAAACTCGCTGCCTCAATCTCTGGCGCTGCCCTGCTGGGGTTGTCGCTA
CTATCCACTATTGATCTGCGATCTGATTGGGTGGGAGCATCTGTAGGAGGGACTATGGTCACTCTTTCTATGGGCTCATT
GGCTATTTTCTCTGATAAGGCAACATATGTCGGTTCGGGCTGCTGTGAACTGGAGCGACGCTGCAGTGCATTCTGGGTAC
AGGAGAAGGACCGCTGTCGAGGAGCAGGCTGGGGTAACAGGAAGTGCGGCGTCGCTGCTCTCGGGACTTCCTGGGGCCTT
TCGCGATCTTCGTCCTCTGAGGTAACCTCCTGCAGAGTGCTGATTGGCCGTGGTGGACGCCCCATGATTCTTGGGGTAAC
CAATGCGGTCGCTGATTGGTTGAGCTGTGGCCGCCAGTTCTGAGGGGAAGGGCACTGCACTCTGCCCATTGACCAATCAC
TGTCAAAAGAAGAGGGAGATCATACGAGTAGGCAGTGATGAATTTGCCTTATCAAAATGAGTAAATTGCTAAGTGTTATT
GTGAGTAGATGTGCTAAATGCCACAAGACTTTACCTTGGTGGAGGCGAAGACATATCCCGATGCCCTATGACACCAGAGA
AGGAGGCACTTCTCCCAGGGGAATTCACCCATTGGCTATACAGCAAGGCACGCTCATCCCACAGCATATCATTGGCTTGC
TCCATAGGAATACGCCCTCTCTGAACCCTGTGAGCCAATAGGAGCTCCAGGACTTGATGATGCATGCCCTCTCGGGCAAC
ATCCAGTGCCCGGCGTCCTCTCCGATCAGACAGATCCTGATTGGCCCCGTTCAGGAGCAGGAACCGAGCGGTGTCGTAGC
AACCATGGAGGGCGGAGAGGAACAGAGCAGTCTCACCCTATCAGATAATGGGAGTGGTATATATCAGAGAATGGGAGTGT
CAAAGCAATAATATTAGAGTACCCTAAAGTTGAACCATTCTACGCAGATTTAACTGAACATAGTTTCTTCGAAGTTCCTC
TTCCTAAATCTTCATACAAGTATGTGAAGAATCATAAAAGCAGAGGCTTCTGCTGGCCTTGGTACCTTGTTGTCCTGTAG
GTCCACCGCAGCACCGTAGCGAGTGAGGGTTCTTGTCAAGGAGAGGTGGTTAACTGAGCATGCCCAGTGGAGGGCTGATC
TCCCTGGGAGAGAGAGAGTGTATTTCAAGTGGAGGAAGGTGAGGGAAGGAAGGTGGGAGTTCAGTACAGAAAGAGAGGGAGT
AAGTCAGCCAGTGAACAAACATTATACAACAGAGTCAAGCCCAAAGGGTGAAAGATGGGGAAGAGGGAGATTTTGAAAGG
GAGGAAATGAGAATGAAAATGATGGTGAGGGACTAGGGAATCAGCCCGAGAGGGAGAGAGCGAGAGAGAGAGAGAGAGAG
AGAGAAAGAGAAGAGCAATGAAGGTTAATATGGTACATCCACACTGCCTGGGCACTAGGGCACATTAACTTCTGTATCAG
TTTGAGAGGGAGGCAGCAGGGGTGCTGTAGAGAGGAATGGAAGGGAAGAGTGGGAAGCCATGGAGGTTTTACCAGAACAG
AAGAGAAGAGAAGGGACACATGGGGAGGACCAGAGAGAGATGAGGTCAGTTACACTGGTTACTCAACCCCTCAAAGTAAT
ACAATATAGTAATATGTATGTGAGGCAGTGTGTTTATAAGCTCGTTATTACACCCATGGAAGCTACATAACATTTATGGA
CATTTGTTCCATGCAATATTGATTTTTAAAATACATTTGCACAATAATCCTCCCATGACACTCTACAGGGAAGTTTCGTT
TACTTGGTTTATTTTACCGTCTAACCTACATTTTGGTTAATAATGTAATTGGGAAATTGGGAGAGTAAAGTGATACCAGT
GTGGTCTGTGTTGTTGACCGGCACCCCAGCTCGTAGCAGCTCCAGTACCACACGGTCAAGCCCACTCCGGACCGCACGAA
TCAGGACCACTGAACCATCTGGACCACACCATTGAACCGAAGCAGACTGTCAAAATAATGTTTGGTACGGTCAAAATATA
ATACTGTAAATAATAACCAGCATTATCTACAATTTAAATAAACATATTGACATTTATGTAGAATATTTATGGAACTCACT
TGATTGGGTGTTCTGTGATGTGGCGACGGGACAGCATTTCTCTCCCATCCTCTTGGGGGAGCTAAAAGTGGAGCTGAACG
TCAATTTAGTTACATGCTTAGTTACGTTGTGCAAACCGTAAATTATTCTGTGACAAATGAATGTAAAGGAATAATGAATC
ATTTTACAAGCCTATTAATACAGTATGTTAACCATGATCAGGTGATCTTACGTTGTATGGGTGATTGTGGGGGGCTGCGG
TAGTGCTTCTGCTCTTGCGTTCGGTGGTCACAGATGGACCTGTTGATGTCCTCCATAGAACTCTGGGTAAAGTCAGTGTC
ACTTCCAATATCCAGGTCCTTTTTCAGGGGCCTGAAATCATACGTTTATGCTCTGAATAGCAGTACTTTGTGCAGACTTA
GTGATATGACTACAGTATCACTAATATTGTGTAAAATAGAGGTAGTCCAACACAACAGTTTTATCTTACAGACAGAGTAA
AACTTCAGTTAGTTTAAAATGCCCCTAATGGAAACAACTGAAAGCCATGCAACCCATTACAATGAAGCAAACTGTTATCT
GATCACAGCACTGTTGATTTACAAGAATGAACATCCTCTAACTTCTCCCATAATCATTCATCTTCGCTCACCGCAGTCGA
ATGGCATCCTCCCCCAGTGGCTCTCTGCGTCTTTTCTTCTCAGCCTCCTTTGCTTTCTTCTTCTTTATCCCATTCCTGTC
TTTCTCTCTCCCTGTCCTGCCCCTCTGCTCTCGGTGGGGTGTGTGCTGTGCCCATGCCTTGGCCACATTGGCTCCGCTGT
CATTCCCAGTGACAGTGGATCTGTGCCTAACCCTCTCTCCTCCTCCCTCCCTCTCTGCTCGTCGTCGTCTCACCCTTCTG

ATCACCAAGACAACCATCAAGACCAACGCTATCACATGACCCATCTCCAAACTTGGCCTTACACTGTCTGGACGGACACT
GGGCCCAGGGGTCACCACCAGGAGACACTGCTGCTACATGGGGAGAAATCACACACACACACACACACACACACACACAC
ACACACACACACACACACACACACACACACACACACACACACACACACACACACACACACACACACACACACACACACAC
AAAGGTTGGATTCCCACTTGATACTAATATACTAACTTAAACTGAATCACTTTGGATAAAAGCATCCATTAATTACAATA
CTGTATTACTATATCAAGCAAGAACCTGTGGTTTGACCTACATTTTCAAGCTGTGTATTGTGTAGGCCTAGGTGTATCTA
TCACCACCAAGCATTCATTGTTAACAATGCTTGGTACCGTGCCAGCCAGGCAGGTTATGAAAGGTGTGTCCTTGGCATGA
ACCATGAAACGGGAAATAAGTGGCCTGACACAAACAGGAAGTTTCCAGGAGAGGCGAATAGCAACAGGAAATGGAGTGGG
CTGAGACTGGATGAAGGTCAGAGGTTGTCACCCTGGCGATGAGGAATGTAATGGAATGGGACAATGGAGAGGCTTGGCAG
GTGTGTGAGGGACAAAAAAATCATAGCACAGGAGAGTTGGGATTAGACATGGTGTACTGTATAGCAGTGAACATTCAAGG
AAATGCAGTACAATCTTTGACTCTTATGCAAGCAAAGCACTTTTTGGTCAGTCATATCTTTAAATCAGTGACACATTCAC
GCAGCATCTTATAAAGTGATGATGAAAGGATGTGAGAGCAGTATATGTCAAGTACGTCACAGTATTTTCTCCTAATGATA
TATCCTCAGAAATCTATAAAACATAGAAGTGAGAACATCAATGTAAAAAAAAAGTATGTTTTGTTGCTGGGAAGTATCAA
AATAAAGAAAGTCGCATACTCCATGTATAAACTCCCATCAACTTAATGGGTATTTATCAACGTTTCGGCATCACTGTGCA
TTCCTCAGGGTTGCTAGGAAGTACCATGTAGGACTTCCTCTCTTTAGACGATTACCTAGTAGAGACTCTACCTCACTCTC
ACAGGTATCAAACATCATATCAACAAGATAATAGCTGGAAGATGGACTCACCTCGACACCACCTGCTCAAACCCAACAGA
ACAGCCGTCCACAGCACCAGCATCATTGAGCCAGGCAGGTCCCAGAGAGGATGGAGACGGCAGTAGCCTACTCTCTCTCA
CTATCCTCCCAGCTGTACCAGTGCGGTTACTAGGTCCAATGCCTTGGTCCGGCTATAGCAGGTCCGGCAGGACTGTACTC
CGCTACAGTGTTGTGGGGTATCTCTCTGGGTCTCTTCTGGAGAACTTACTGTCTCACTGCGCTCCTGACTGCGTTCGCAA
TGGGACCATGTGCACAGTTTATATCTAAAGTGGTACACCCCAACTTCCAGTGCTGCTACCACCTACCTCCCTTTCTCTAA
TTATTTTTCAGAAGCAATGCGGAAATATGTGGACAACTTCCTGCTACTGTGTGGAAAACAATAGGAGCGGGGTGTGTGTG
TGTGTGTGTGTGTGTGTGTGTGTGTGTGTGTGTGTGTGTGTGTGTGTGTGTGTGTGTGTGTGTGTGTGTGTGTGTGTGTG
TGTGTGTGGTCTTTGTGTGAGTCTGCAGTCTGCTTTTCTACTGATTATAATGCCTCAGGATGTAATCTTTCAAATGAACC
AGGCCCTATATTCATGAGGCAATCTAAGTCCCCCCGTTCATGCAATCATATTAATTACGTTAAAAAAGGCAACACATTAT
GAATACAGGCCCTGGAGTAGGCCTGCATATATCACTTTTTTAACTTCCCATGCAGGAGCATGCTCTCTGCATTTAAAAAA
AAATCATCTTATCTACTGCAAACGACAAATAAAACTATTTCAAAATCACAAAGAAGAGATAATAACCTCCAGGAATGTGG
TGGGTCTTAAAATAGACAGATATAGCTGCAGGCCCATATGTCATCTGTCTCAAATGAAAGGATGGATCCAGGAACAGGAA
ACTATGAGGTTCTGCCATTCATCCTTAACCTCAGGACTCATCAATTGTGCCAAGAAACAGCCTCACAGCAGGTGGTACAA
CAAAGATTGTGTGACTTTTCAAGTTTGGGGGGGGGGGGGGGGGTCTATTTCAGGCTAAGACATACTACTTACTCATTATC
TGGGCAAAAACCTAAAATCGTTTTTTTTTATATGATGAGTTTATATATATAACCATTAGGCTGTTGTGCATTGTCGAGGG
AAATATGCAATAGATGATCATGGAAATAATGTTTCAATGATGGTAGTGATAATGATTACGATTATCATTGTGTAGGTCAT
CAAGCTTTCCACACGATGTCGCTATTCTATATTAGATTCCTTTCAAGTTCAAAGGTGAATCAGCCAGTAAGTAGTTTACT
GTACAACATTTTCATATATACCAAGCCCTTACACAGCAATCACCTACTCGTTTCACATGAAGTATAGCATAGTATAATTA
ATCTAAAATCGGCCTTTGCCAATGTCAAGAGGGGGGCGGGTCGTTCAAAAGGGACTTTCCAGTTTTATTTACTTTCGGTTT
CTATTATGAAGCACAATCATCAACTCGAACGTGGGGTCAAGTCCAGTCGCAGGCTGCTGCTGCATAACTAGAATAATAAT
AATATTGCCCTTTATATGATATGAGTGTGTAACATAGAACATAAGCTAGATCTATTCTTAATAGATATAGGCTATCGTTT
CCTTGACATTCAGAAGAAATTGCAACCTCAATCGTAAGAGCCCACATGCAAACGTGAGTAGTCTATATTTTTAAAAAGCT
ATTTAAACGTGTTATATTGTGTTATATATTACAGCATGTATTGTAATGTGTTGTTACAATTCGTCATATCCATCAAGAAG
CCTAAACAACTTTGAAATTCACCTAATTGAAAGTGAAAGTAAAAATGAAGCATAAGATAAGAGTTGAATACACACTCTAG
CACCCAATGCTGTCCCTTATTAGTCGGTCAGCTGTTTTTCATTAAATGGTCAAACTCCCTCCAATATTTTCAGATAATCT
ATGTCCCAGGGGGTTAATTGGGATTTCGTGCGAACTAATTCCCAAATTGCCACTTTTTCCATAATACGCTATAACAGATC
TATGTGCACATTTCATGTTCCATATGCTATCCCACTTAGTCCTCCTCAATTTAACCCCTGGATATGATGTTCCTCAACCT
CATTATCCGTCTCTCCCAACCTCCTGTTCTAGAGCCTGTTCAGAATGCCGAAAATGAACTTCTCTCCGCTGCTCTTCCTG
TGCATGGATGTGTGTGTGGTGCAGACAGTCCGCTTGGCCCAACTCTCCCCTCTTCTCCTCCACCATCCCATCATCACCTT
GTGGGGTGGAAGCCTGCTCCGTGCCGGCCTCCACCTCTTTCTCACCTTCACATTCCCTGGAAGTCCCCCATGGATGAGCA
GCTTCGAGGGGCTCCAGAGCATGGGGGTCCTCTGCTTCCATTGCCCACTCTACATCAGCCTTCTCTGGGCATGTGGCCAG
TCCATCTTGGGGCAGCTATGGGGATGGCATTCCTGGCAGGGGGTGGGTGACAAGTCTATATTGCTATAATTATGTTAATA
TATATACCCTTTTTGTTTTAAAAATTGACAAACCACACAGACTAAAATCACTTGTTTTTCAGCTACTTCAGGGCTACTGC
GTAACAGTCGTAGCATGTTTGTACTGGACACGCTACGTCCCTTCTCTTCTTACCAAGCCCACCAAGGAACCACAGAAGAA
GACTGGCGCCTCTCTAGAGAAGCTGATGGGATACATGAAGCCTTATATCATACGCTTTGGAGCCGTGCTGAGTCTTGTCG
TCATCTCTTCCCTTGGTGAGATGGCTATTCCCCACTACACCGGCCGAATGACAGACTGGATTATGAATGAAGACGAGCCT
GAAGCCTTTACTCACGCCATCATGGTCATGTCTCTGATTACAGTCATGAGGTATAGTACAGCATATATCAATAATTGGTG
ATACGATACTCTCGAGTCCTTCCAATTGATGCTCAGTGTCTGATACTGCTGTTTCATTTGAAGGTTTCAAGTGTAATAAT
CACTTCCACAGGGCATTTATTGTAATGATACTGTAGTAACGTGTTTATTTGCCCTCCTTCTCCCATCTCTATCATTCAGT
GCTGTGTGTGAGTTTGTGTGTGATCTCATCTACAACATCACCATGAGTCGTATCCACACCTCCATCCAGGGCCTAGTCTT
CCAGTCGGTGCTGAAGCAGGAGATTGCTTTCTTTGACAAAGCACAGACAGGTGAGTCACATTTCATTGCAATCTACAGGT
AACTGCCTAAATAAATGAAACACCAACATAAAGTGTCTTAATAGGGCGTTGGGCCACAACTAGCCACCAGGATAGCTTCA
ATGTACCCTGGCATAGATTCTACAAGTGTCTGGAACTTTATTGGAGAGATTGGCACCATTCGTCCACGAGAAATTCCATA
ATTTCTAATAGGGATGCACCGATATGACATTTTTGCCCGATACCAATATCCAATATTTTCCTTACCAAAAAAAAAAAAAA
CATACCGATAGCCGATATTTAACATTTTAGCAGCCTTTTAAGCATTCTAGTACAGTTAAATAGTTGAAACCAAGTGTCAT
ATGTTCATTTGTTCAGTTAGGAACAGATTGTTTAATAACTTGTAAATTAGAAAAATGACACATCCATGAATCTATTTTAT
CGCACAACAGATATTCATGACACAAATATTTTAGCTGCCCCGCATTATAAAGATGGTTGCTGGTGGCTCTTCTTACTATC
CAATCTTGGATAAACTTAAATGCTTTGAAAGAAATATTGGTTTAAACTAAAATGCAGTTCATAATTTTAGATTTAACTGC
TTGAAGAAATGACTGTCTCTAGGCTGTCATAAACTGCTATAAATGGCACAACTTAAACTTCAAATGTGCTCAACTGACTT
GACTAGTAAAATGAGAACAAATGTATACCACATCATCTCATCTTTCATTTAGGAAATAAAAACAAAAAACAAAACTGCTT
TGTTTTAAATTTAAATAAAATCTTGACCGATACCAGCCATGAATGAACAACAGAAAGCCATTGGCCTTAGTAAATATTTA
TCCAGTGACTCTAGATGGCAGGGTTACAATATGACTGTCCATTTACAATTTTAACATTTGGGGGAGGTTTTTCTTCACAA
AGAGAACCTGCTCGGCTTTGTCACAAGAGAGTCGTTTCTTTTATCGTCTACAATGTGTGAAGCTGCACTGAAAAGGCGCT
TACTGTCCACACTAGTACAGGGAGCACCAAGGTACTTGCGCGCAGCTTTAGCTAGGATGGGGAAACGGGGCTTGTTACTT
CTCCAGTGTCCAAGTGCATCTTCATTCCTGGGAATAGTAGGTTCTGTCATGCAAATGTTGGCCGATGTAGTAACAGAAAA

TGAAAATACAATGCGTCCGTCTCTCTCTCTCTCTCTCTCTCTCTCTCTCTCTCTCTCTCACACACACACACACTCACGCA
CACACACACACACACAACAAAAAGTTATTTTGTTGGCATTTACGCATGTCCTCATTACCAGTAAAATATAATCAAAACCT
ATTTCTTTCACGTACTTGCTGTGCTGTTTTGTTGTTCATTTGTTCAGTCCTTTCATTCTCAACCAGGATTTCATCATACA
TGTCAAGCAGGGAAGTTTCAGCCCTGTCTGTCCGTGGCCTCTTCTGTCGTGGGTCCTCTTCTTCGGTGCGCACTGTCACT
GTGTCCGTTTCCATCTTGTCCATCTGTGTCAGTAACATTTCATGTAAACCCTGTTTCTTGTCTGCATCGAAGTAGTGGTC
CTTGTACCTAGCATCGAGTATGTTGGCGACACAGTAAAGAGGCTCAGAGAGAATGCCACCAAATCGCTTGTTCACAGCGT
CTAGTAGAGTATTTTTGAAAGTTTTAACCCCACTGTCTGTGTCGGAATTTCTGTTGAGCAGGCGTTTCAATGCCATGACA
GAGGGTATTACGTCTGCTGCAGATGCAGTTGAAGAACTTATTTCTCGAGTCAGTTGTTCGAATAGAGCTAGGACTGGGTT
CATGTTTTCAATGAGAGTTCACTGGTTCATACTGATTGTTGCAGGTAACTCATAGACAGCTGCATACATGCCAAGGACTC
GTTTTTGTTCCAGCAGGCTCTCCATCATGTAAAAAGTACTGTTCCATCTGGTGGAAACGTCTTGCTTAAGCCTTTTCGTA
TTCACTCCAAGCTTCTCCTGAATTACTTGCAGGTATGCTAGCTGTGAGTGTTTAAAATGACCCACTATCTTCCTACCTGT
TGCCACTGTGTCAGAAATGCTGCGTTGGGCCAAAACACCTTCGTTCACAGCCAGTTGCAGCGTGTGTGCCATGCATGGCA
AACTGACGACTCCGCATTCTTCCAAAGCCTTTGTCATGTTATGTGCATTATCACGTAGCACAGCGTGTACTTTGTTCTTG
GGGATTTTCCAAATTTCAAACATTTTCTCAAATGCCATTGAAATGGCAGCAGCGGGATGAGAACCAGCACATTCTTGAGC
ATATGGCTTTCCTCAATACTAAATCCTCGTCGACCCACTGTGCTGTCAGACTCAGCATGCTCATGGGGCTGACGTCGCTG
GTACAAATGCCAGTTGTGAAGCTAATAGCAGCGACGCCCATAGCAAGTTGCTCGTGGATGTGCGATTCAACAATACTGTG
TAATTCCGGTAGGGCAACATCTGAAAAATAGCATCTACTTGGTAGTGTGTACCGGGGCTCGAGGTGCTCGACCAGTCGGT
GAAAGCCAACATCATCCACAACAGAGAACGGTTGATTATCAAGGGCAATGAATTCCATTATCTTAGCATTAGTGGATTTC
GCCTTTGAGTTGTCTCGCTGAAATTTTCTTACTCTTTCAAATGACTGCTCAACTTTATCTTTTTTTTCTGCTTTTGTTCC
AAGTAGTCGCTGAACGTCTGGGGGCGATGCACTTTCAAATTAGTTATTAGGTTTGTGGTATTAAAAGATTTCACTTTCTC
CCCTCCTCGGGAAATAATAGCAGCACAAACGTTGCATATAGTCTTTTTGTTATCTACCTTTGAAACTTCAAAATAGATTC
ACACCGCAGACATTGTGGCCTAGGTTAGGAATGCTGTGTTGAAAATGTAGCGCTGTATTTTTCGTGGCATCATTATGTCA
CCTACCTACGTTATGTAGGTATGCACTTCAGCTTTGACATCGGCGTTAAACTAGACATCGGCCGATGTCGATGTTGGCAT
TTTTGGCTAAAATCGGCCAATTCCAATATTCTCACCGATATATCGTGCATCCCTAATTTCTTGTTTTGTTGATGGTGGTG
GAAAACGCTGTCTTATGTGTGCCCAATTGGGTTGAGATCTGGTGACTGAGACACACACATACCCTTTAAACCTTCTATGC
TCTTTGAGACCCCTCTTTCAAAGTCACTGAGATCTCTTCTTCTAGCCATGGTAGCCAAAATAATGGGCAGCTGGACATAT
TTATACATGACCCTAAGCATGATGGGATGTTCATTGCTTAATTAACTCAGGAAACACACCTGTGTGGAAGCACCTGCTTT
CAATATACTATGTATCCCTCATTTACTCAAGTGAATTCTTTATTTTTATCAGGTTTGAAGGTAAGACCCAGATACAGACT
GTGTCGGAGGAACAATGTTTATTACAGCAACAGGGCAGGCAAACGACAGGTCAAGGCAGGCTGGGGTCGATAACCAGAGT
AGTGGGGGAAAGGTACAGGACGGCAGGCAGGCTCACGCAGATGTTGGTAGTCCAGAGGTGGGGCAAAGGTACAGGACGGT
AGTCAGGGTCAGGGACAGGCAGAGTGGTCAGGCAGGCAGAGACCAGAAAGTCAAGGGTCAAAACCAGGAGGGCGCGAAAA
AGAAAGACTGGGGAAAAGCAGGAGCTGAGACAAAATACCCCAGGTATTTTGAAGTGAAAAGGAGAGTATCTTAATAAACA
CAAGTGATCATGTCAAGAGTATTGTGCAGGTCTTTCTTTCTTCAAAGTGAAGAGGATACCTTTATGCTATATATTTATGC
TTTACGCTATATATATGATATATATTTGCACACTGTCCCCTCCAAGGGGCTAACTTAACCTATGACCTTTGTTCCAGGTG
ACCTTGTGTCACGCATCACCACGGACACCAACGACATGAGTGAGTCTCTGAGTGAGAAGTTGAGTCTGCTGATGTGGTAC
TTCATGCGGGTCACTTTCCTCTTCCTCTTCATGCTCAATCTCTCCTGGAAGCTGTCACTCTTCACCGCTATGGGGCTTCC
TATCATCTGGGTCATACCAAAGATATCCGGCAAGTGGCACCAGGTACAGGAAATCAGACCTTTTCTAACGTATTGTAGTG
GATGTAGCCTGGTCCCAGATCTGTTTGCGTCTTCTTGACAACATGTATGGGGTGACAATGACAATAAGAGTTGGCTAGAC
AGCATAAACAGATCTGGGACAAGGCTATAGTGGGGGTAGTTTTGTGAACTACTGAACCCTCCCATAATGAGTAACATTGT
CTGAGAGTTGCAGTTTTGTGTTTGCATACATCTTGACTCTACAAGTTACAGTCACCAAAAATAGCACTACAGAGCAGACA
GAACGTTACCTGACCAATCAGAAGGTAACTGTGAATATTGTTTTTGTCCCAGGAGCTCGGTGCGAAGGTTCAAAAGTCAC
TAGCCCAGGCCAATGACGTTGCCACGGAGACCTTCTCCTCTATGAAGACAGTACGCAGCTTTGCCAACGAGGAGGGTGAG
ACGGAGCGCTACAGGAAGAGGCTGGAGAAAACCTACTCTCTGAATAAAGAGGAAGCAGCAGCCTACGCAGCCTCAACCTG
GACCAATAGTGTGAGTCTCAGCTTCAATTGTAAACGGTCACAGGGTGAGAGCAATTTATCCAAATTCAGTTCCAAAGAGC
TTGGATTGCACATTGACTCATGAGGGTACATGAATTTGTCTGTAATAAGGGCCCTGAGTTTTTCCTGGCCTTGTGAACTG
ACAAGGGAAAGCTCTGGGCAGTTGTTATAGACTATAACTACTGTAGGTCCCAGAGTTGTTCCTGGTTACATGATCTGGAA
AACTCTGGGCCCTAGTCATAATCAATAGTAACCCCATCTGTCTCCCTCTCAGATGTCAAGCCTGTTCCTGAAGGTCAGTA
TCCTGTACTATGGCGGTAGTCTGGTGACAGGGGGGACCGTCAGCAGTGGGGACCTGGTGGCCTTTGTGCTCTACGAGCTC
CAATTCTCCTCTGCTGTGGAGGTAAGGCCGAGATTAACAATCTCCATCCAATCTACCCGCAGCCATTTTATGTTTAATGC
CTACTGCCAAATCCAAACAGACCTAAATGTACACGCCCTAACAAGGCACTGCCAAGTTGTTCTACAGTAAAATAGGGTTC
AAAATGATTTATTAGTATCACAACATTTTAATCACAACTAGAGTTTTGAGTAGCAACATTTTGACAGTCACTTACTAGTG
TTGCTTTTACACGTCCTGTCTGACAGGCCGTGATGTCGTATTACCCAAGTGTGATGAGGGCAATCGGTGGCTCTGAGAAG
ATATTTGAGTATGTGGACCGGCAGCCCCAGGTTCCTCCAGAAGGCACCCTCGCCCCACAGAATCTTGAGGGACATGTTCA
GTTCAAGAATGTCACATTTACCTACCCTACCAGGGAAGATACACCTGTGCTCAAGGTATCTACAGTATATTTACAGTATA
CTGTATACACATATACTGGATATGAATTAAAATGACANNNNNNNNNNNNNNNNNNNNNNNNNNNNNNNNNNNNNNNNNNNN
NNNNNNNNNNNNNNNNNNNNNNNNNNNNNNNNNNNNNNNNNNNNNNNNNNNNNNNNNNNNNNNNNNNNNNNNNNNNNNNN
NNNNNNNNNNNNNNNNNNNNNNNNNNNNNNNNNNNNNNNNNNNNNNNNNNNNNNNNNNNNNNNNNNNNNNNNNNNNNNNN
NNNNNNNNNNNNNNNNNNNNNNNNNNNNNNNNNNNNNNNNNNNNNNNNNNNNNNNNNNNNNNNNNNNNNNNNNNNNNNNN
NNNNNNNNNNNNNNNNNNNNNNNNNNNNNNNNNNNNNNNNNNNNNNNNNNNNNNNNNNNNNNNNNNNNNNNNNNNNNNNN
NNNNNNNNNNNNNNNNNNNNNNNNNNNNNNNNNNNNNNNNNNNNNNNNNNNNNNNNNNNNNNNNNNNNNNNNNNNNNNNN
NNNNNNNNNNNNNNNNNNNNNNNNNNNNNNNNNNNNNNNNNNNNNNNNNNNNNNNNNNNNNNNNNNNNNNNNNNNNNNNN
NNNNNNNNNNNNNNNNNNNNNNNNNNNNNNNNNNNNNNNNNNNNNNNNNNNNNNNNNNNNNNNNNNNNNNNNNNNNNNNN
NNNNNNNNNNNNNNNNNNNNNNNNNNNNNNNNNNNNNNNNNNNNNNNNNNNNNNNNNNNNNNNNNNNNNNNNNNNNNNNN
NNNNNNNNNNNNNNNNNNNNNNNNNNNNNNNNNNNNNNNNNNNNNNNNNNNNNNNNNNNNNNNNNNNNNNNNNNNNNNNN
NNNNNNNNNNNNNNNNNNNNNNNNNNNNNNNNNNNNNNNNNNNNNNNNNNNNNNNNNNNNNNNNNNNNNNNNNNNNNNNN
NNNNNNNNNNNNNNNNNNNNNNNNNNNNNNNNNNNNNNNNNNNNNNNNNNNNNNNNNNNNNNNNNNNNNNNNNNNNNNNN
NNNNNNNNNNNNNNNNNNNNNNNNNNNNNNNNNNNNNNNNNNNNNNNNNNNNNNNNNNNNNNNNNNNNNNNNNNNNNNNN
NNNNNNNNNNNNNNNNNNNNNNNNNNNNNNNNNNNNNNNNNNNNNNNNNNNNNNNNNNNNNNNNNNNNNNNNNNNNNNNN
NNNNNNNNNNNNNNNNNNNNNNNNNNNNNNNNNNNNNNNNNNNNNNNNNNNNNNNNNNNNNNNNNNNNNNNNNNNNNNNN

NNNNNNNNNNNNNNNNNNNNNNNNNNNNNNNNNNNNNNNNNNNNNNNNNNNNNNNNNNNNNNNNNNNNNNNNNNNNNNNN
NNNNNNNNNNNNNNNNNNNNNNNNNNNNNNNNNNNNNNNNNNNNNNNNNNNNNNNNNNNNNNNNNNNNNNNNNNNNNNNN
NNNNNNNNNNNNNNNNNNNNNNNNNNNNNNNNNNNNNNNNNNNNNNNNNNNNNNNNNNNNNNNNNNNNNNNNNNNNNNNN
NNNNNNNNNNNNNNNNNNNNNNNNNNNNNNNNNNNNNNNNNNNNNNNNNNNNNNNNNNNNNNNNNNNNNNNNNNNNNNNN
NNNNNNNNNNNNNNNNNNNNNNNNNNNNNNNNNNNNNNNNNNNNNNNNNNNNNNNNNNNNNNNNNNNNNNNNNNNNNNNN
NNNNNNNNNNNNNNNNNNNNNNNNNNNNNNNNNNNNNNNNNNNNNNNNNNNNNNNNNNNNNNNNNNNNNNNNNNNNNNNN
NNNNNNNNNNNNNNNNNNNNNNNNNNNNNNNNNNNNNNNNNNNNNNNNNNNNNNNNNNNNNNNNNNNNNNNNNNNNNNNN
NNNNNNNNNNNNNNNNNNNNNNNNNNNNNNNNNNNNNNNNNNNNNNNNNNNNNNNNNNNNNNNNNNNNNNNNNNNNNNNN
NNNNNNNNNNNNNNNNNNNNNNNNNNNNNNNNNNNNNNNNNNNNNNNNNNNNNNNNNNNNNNNNNNNNNNNNNNNNNNNN
NNNNNNNNNNNNNNNNNNNNNNNNNNNNNNNNNNNNNNNNNNNNNNNNNNNNNNNNNNNNNNNNNNNNNNNNNNNNNNNN
NNNNNNNNNNNNNNNNNNNNNNNNNNNNNNNNNNNNNNNNNNNNNNNNNNNNNNNNNNNNNNNNNNNNNNNNNNNNNNNN
NNNNNNNNNNNNNNNNNNNNNNNNNNNNNNNNNNNNNNNNNNNNNNNNNNNNNNNNNNNNNNNNNNNNNNNNNNNNNNNN
NNNNNNNNNNNNNNNNNNNNNNNNNNNNNNNNNNNNNNNNNNNNNNNNNNNNNNNNNNNNNNNNNNNNNNNNNNNNNNNN
NNNNNNNNNNNNNNNNNNNNNNNNNNNNNNNNNNNNNNNNNNNNNNNNNNNNNNNNNNNNNNNNNNNNNNNNNNNNNNNN
NNNNNNNNNNNNNNNNNNNNNNNNNNNNNNNNNNNNNNNNNNNNNNNNNNNNNNNNNNNNNNNNNNNNNNNNNNNNNNNN
NNNNNNNNNNNNNNNNNNNNNNNNNNNNNNNNNNNNNNNNNNNNNNNNNNNNNNNNNNNNNNNNNNNNNNNNNNNNNNNN
NNNNNNNNNNNNNNNNNNNNNNNNNNNNNNNNNNNNNNNNNNNNNNNNNNNNNNNNNNNNNNNNNNNNNNNNNNNNNNCC
TTTTAGCTGTTCCCAAAACTGAAACGTTACGAACACAGTGCACTGCGTGTTATAGACTTGACGCTTAGCGAAAGATAAAA
ATAATTGCATTGTTTAGGAATGCACGGTCGAATTGAGTCTGTCTCGGCACATGCGCACTTCACAGAGGCGTACCCTAACG
AAAATATGCAAATACATGATATAACGTGCCAATCGGATCTCGCTAGCTCGTGCTTGGCTTTGCCCCCTTGTTCTGCGCAC
TATTTCATTTGCTTACACTGTAAATGACTTAACTATCTTGGGTTAGTTATAAATATCTTTGGTACCGGGTCCATCACAGC
TAAACTGTCGTAACACCATAGCAACAGTATTTGTTTATTTGTTTCCCCATTAGCTTTTGCTGAGGCAGAAACTATTCCAT
GGGGTCAAAAAAAACACAAAACATGAGAAGTAACAAAACACTAATACACTGATAGACAGTAGCACACATTTTAACACAAA
GATATATAAACAATACAATTATAATAATTCAAACAATACAAGTAAAACAAATAATTTGTGTGTGCATGTGTTTGTCAACG
CCGATCCACGACTTTGTTTATTCTTTTTTTAAAAGGTAATTTTGCTGTTAGCTTGAGTAATTGGAAAATGGAAGGGAGTT
CCATGCAGTCCTGGCTCTGTGTAATACTGTGCATAGTTGTGAATTCGTTTTGGATTTGGGGACTGTGAAGAGACCCCTGG
TGGCATGTCTTGTGGGTATGTATGGGTGTCTGAGCTGAATGTTATTTGATTATGCAGACAATCTGGATTTGGCGTATTGC
TCTGTTTTGAGCCAGCTGTGTCTTTGCTAGGTCTTTCTTTGCTGCACTTGACCATATTACCAGGCACTAATCAAGATGGG
ACAAGACCTGAGTCTGAACAACGAGTACAGTGATTTTTGAGTAAAAAACGCAGAACATCTAACATCTTTTCATAACAGAC
ATACATGTGTAAAACCAGTTGCAAATAATTTTTAAAATAGGGAAGAGTAATGGCTCAATGCAACTGCCCTAAGGACACT
GTACTGTGATGCTAGAGAAGCTTCCATTGAAGAACACTCTCTGTGTTCTATTGGATAAATAACTCTCCAACCATGTGATT
TCAGGTGATGTAAAGCCAAAGCAAGTGATGTTATTGATCATAAATAGTTATTGAAGAAACTCAAAGGCTACACTGAAAAT
AATACAGCTCCAACTATCATATTATCAATGTATTTTAAGCAATCATCTGTTATCTGAGCCAGTGCAGTACAAGTTGAGTG
CCCTTCTCTATGCATGCTGAAAGTCAGTAGTTAACTTGTTCTCTGAAAAATAGCATTGTATTTGGTCAAACACAATTCGC
TCCATCAGTTTACTAAGAGCAGGCAGCAAAACTGATTGGGCGGCTATTAGAGCCAAAAAAGTCTGTTTTACTATTTTTAG
GCAGTGGAATTACTTTAGCTTCCTTCTACGCCTGTGGACACCCTATTTTAGACTTTGGTTAAAGATATAGCAAATAGGGG
TGGCAATACCATTCTCAATATTTTCTCATCAAGGTTTTCTATACCTGTTGGCTTATCATTATTGATGATAACAATAGTTT
TTCCAACTCTCCCACAATAACTTGAACAAATTCAAAACAGCCATCATTATTATACATTTTATACAAAAATATGATGGTTC
ACTGATCAATGTTTTCATTTCACTTCTGAGTTTTTCCACTTTACTAGTGAAACAGTCATTGAAATGATTGCCAATATCAA
AAGGTTTTGTTATAAATTACCCATCAACTTCAATGAATGAATGCGGAGGATTAATTGGGTATTCTGCCCATGTAATTTAA
GGTACTCCAAAGTCTTTTTCCACTGTGTTTTATGTAATTTATCTTGGTTTGGTAGTTTCTTCTTTTTGTAAATTTCCCAA
TTTACAGTATGTCAACGAATCAGCTGATCAGCCTGACTTGTTTGCCACCTCTTTTACACCATTTCTTTGAACCATACCAT
TTTTCAATTCATCATCAATCCAGAGGGCTCCAACAGTTCTCACATTTAATTTCTTAACAGCTGCATCTGGATTCACTTCT
TCATACACATCAGACCAACATACACTTTTTTTACATCTTCAACAAAATTGTCCTGAGAAAACACTTTGTGTGATCTTTTA
TAAATTACTTTAGGCACAACCTTTGGCTTTCCTTGTTATTGCCACAATGTTATGGTCACTACAGCCAATGGGAACTGATA
TTGCTTTGGAGCAAAGCCCTGCAGCATTATTGAAGATATGACCAATACAAGTGGATGTCACAGATCCAACACTATTGGTA
TGCACTCTAGTTGGTTGAGTAATTAGCCTGAGTCATATTACAGGCATTAGCCACAGTTAGAAGCTTCCCCTTGAGAGGAC
AGCTTCCCCTTCAGAGCAACCAGAAAAATGATTATCTGTTAACATCGTACACCATCAAGCATTGAGCACATATTTAGATG
AGGCAGATGAACTTGCAATCTTCTTATGGTCCATCTGTAGCTTTTTGGTGATTTTTCTCACTTTCTCCATCAAGGTCTCA
AGTGGAGACTGGAACGCCATCCACAACAATGTTATTCCTCTTGGATTGTCACTCTAGATAGTCATCTGTAATAATGATTC
ACATACAGTGCTGTCATCATCTCATGTGCTGTCATCATCTTGCTGCAGTCAACTCGCATTGCTAAAATGCCATTGATTTA
TTTAACCAGGCAAGTCAGTTAAGAACAAATTCTTATTTACAAAGACTGCCAATTGTGCGCCACCCTATAGGACTCCCAAT
CACAGTCGGTGATTGAGAGCAGCATGGATTCAATGCAGCCTGGATTCGAACCAGGTACTGCAGTGACCCCTCTTGCACTG
AGATGCATTGTCTTAGACCATTGTGCCCATTGACTGCGAAACCTTCAGTCTACCATAAGGACAAAGCCTGATTTTCCTTT
CCTAATTAATCATGTGACTTTTGGCTAAATGTCAGGTTTAAAAACATTCACTTTCAACTTAACCTTTATTTAACTAGGCA
AGTCAGTTACGAACAAATTCTTATTTACAATGACAGCCTACCGGGGAACAGTGGGTTAACTGCCTTGTTCAGGGGCAGAG
TGACAGATATTTACCTTGTCAGCTCATGGATTCAATCCAGCAACCTTTCGGTCACTGGCCCAACGTTCTCACCACTAGGT
TAGCTGCCACCCCCAACTTTAATAGCCCTGGAAAAATATATTTCAATGTAAAAGCTTGTAGGCTACTCTAGCATATATGT
TCAATAGTGCAACTCATGGGAAAACCCTGGCCTAAACTTTATGATAAACTGGCACATCCAGGGTTAGAATTATAGTATTG
ACTCTGGGCCAAGGCCCCTGCCAAACTTTAGAGTTGCAATGTATTGGATGCCCCATAGACCATCAACATTGTTGGGAGGG
ATGTCCACAGTAATATTTGTCATGTGGCTGATTTTGGGGTACGCAACCTCCCCTGGGTGCATCTTAAAAGTCTAGCAGCC
TCAGTCCCCACTCCATCTGAAAATCAGAAAATATCCCTGAAGCCAAGCCAACTGCAGGACCCGTTTGCAGNNNNNNNNNN
NNNNNNNNNNNNNNNNNNNNNNNNNNNNNNNNNNNNNNNNNNNNNNNNNNNNNNNNNNNNNNNNNNNNNNNNNNNNNNNN
NNNNNNNNNNTGCCATGCAACATGACAAGATGTTGACAACAACTTCTTTAAATTTCAGAATCATCATGTTATGGCTGAAC
TGCCCCTGATGTGACAGATTGCATAACTTCCACTTCAAATAAAAAACCTGAGTTATACTCCAACGTAGTGACTGAATACA
ACCTACACCTGAGGCTGATACAGATGTGTACATATATATATATATATATATATATATATATATATATATATATATATATA
TATATATATATATAAATAAATAAAAACTTGGATACAGCTGATATACCCCACAAGAAAAAGTAGGTGCCTAAAAGGCTGTT
GATTTTCTGGCTATTTTAGCTAGCTACCGTTAGCTACTTGGGGCTAAACATCGGGAAATAGTACGCAGTCATGACATGAG
GAAGACCTACCACCGGTAATAATTGTTTTGTATTTAGAATTCGGAGTAGTTCGTAATAAGCATTCGGTTTCGTAAAGATG

ATAACGGTTATATAAACAAATCATCTGTGTTGTTTTTATCTAACTAGCTAGATTAGCCGCCTGAAGCTAAAGGAACAGGA
TGTTTTTCTTGCAGGGTGTGGCATGTTAAATTACAGACAGCGTGGATAGCCAAACATAATCTAGCGCCTATGCGGAGTAG
CCAATCGGAAGCAAGTATTACGAGAAACCCGACAGGAATTGCACGGAAACGCCACGTGATATGTTTATAAAGCGGCAGGC
GGAAGCTTTTCGGCGCCATTTCATTGTAGAACTGTTCGGCTTTTATTTAGTGGAAACATAACAGTGGACAAATTTGGAAC
ATAAAAAGAATCCGGATCCATCCTGATGGAAACGGCCGTTAACCCGCCCCTTGACAGGTACCTTTAGGAACATTGTACCT
TCAGGGTCGGGTCGGAACTGATTTTGCAGGCTTGACTATTAACACGAATCGGGACTGAATGGCTAAACCTTGTTAGCTAG
TATTCACTGTCGGTGCATCAGATAGCTAACTAGTCTTTTCTCAACTTTGACACCGAACAATGAAAATATTCCGTTGGCTA
GTAAGATGTAAATATAGAGATGTCTTTGTTCCTTACGCCCCCTTAGTAAGTTATGTAGTGAGGATGGATATTGACATATT
GTGGGTCAGCCTACTCAATAACTTAGTGCTGGGGGGATTGTTTAACATTGTCTGTGGTGATGAAACGCTTCATTGAGAGG
CACTGCACCACCGCCATTTTCCTGCTATAAATCTTATGACAGAGAAATAAACTGAACTAGGGCCGCAGATGTGCAATTTA
TTTGTAGCTTATTCAGTTGCCCTAACCCGGTTGTTTACTTTTTGATGTTCGCTAGCAAACGTTAGCGTCCTGGCTAACTA
GCTTAGTAGCTAGCTGGATTGTTGTTTACAAACGATGCGATGGTTCCAAGCTAGCCAGCTAAGTTAGCGTTTCCAGAAAT
GGGGTCTCACATAAACAAGCTAGTAACAAGAGGAGCTGATTGGATAGTTGTGCAAGGGTTTCAGTCACAGGAGTTTGAAG
GTTTTGACTGACGATCCTGAGGGTCTGTTTTGGCGTCGGTGTGTTTACAAACACTCATAAAAGGCGGGGCGACGACACTG
ACCCCACGCAATGACTACAAGCGCAGAAGCCTTGCCTCTGCCGGCTGAAATGATTGCCACAGTTAAGAATGAAAGCATAT
CACAATCTAAATAGCTAATGCCTTTGATTATTCCCTATTACCGGTTAACACCCACGAATCCAAGTTAATTCAACGAAAAT
ACTTGGGAGGTTAGGCTAAGTATAACAAACATGCATAGAGAGAGCAGTAAACTGGTCAACATACTATCTATCCGTCGTTT
TCCAGGTTATCAAGGAATAATATTACTAATGTGTTGACAGATACTCTGCAGCTCTTTCCCTTTTCTTCAAAGGGGTTAAC
TTAACAAAGCCATGTTGTGTTGGCGTGTAAGTCCTGAGAGGAAAAATCAGCTCATGAGCATTGCCTACTTTTGTGTCAGT
GGGATGGAATGTGCGTGCTGTGAGCCCTGGGGTGACCGGGATAAAAATCATCCAAATCTGGTCCACACAGTGCAAAAGAT
AAAAGCGAATAGAGGGAGAACCTGCATGTTTTCACTATTCTATCACAGAATGCAAGTTTAACTGCCTTAAGTGCTGTACA
GTACTGATGTTTAGTACAGTAGTGCTTCAATGCTGCCCCAGGGATTTTCCCATCCAGTGATCTGGGTTGCCGGGGCTGTG
CCAAAAGTGGCAGCCTATTGATAGAATCCTCATTGCATTGGTGAGGGAGCAGATGGTGAAGGGCCTGTGTTGGCGAAGGG
TTCCTTCCTAATGCTTTCCTGCTTGAATTGCATAGCCTGCACCTTAAAGTTTGTTGTCATGAAGTTTGGCTTCCTGTGGG
GCAATGATCAAATATCAAGCACATTGTAGCTTACTACTTTTGAAATGCCAGAGCTGAGGGATAATTTTAGCATATGAATG
AGTTTTCTGCCCAAATGTATCTCTCCCCTAATACATCCCTTTCTGTCTTCCCACGGGCTGTCCTGTCTATCCCACTACTT
CAACCTCTCCCACCCTCTGCTCCTCTCTCCTGGTCAGCTGCTCTCTGGGAGTGGTGGGGCTGTCTGGCGGCGGGATGGAG
CACCATGCCTCGGGCCCAGGCAAGCGCATGCGGAAGCCCTCGCTGCTGTATGAAGGCTTTGAGAGCCCCCAGCTGCCGCA
CGCGCCGCCCCCTCCCCCGGTCCAGCCCCCCGTCAGGGACCCCGGCCGGCAGGGCTGCATGACCAACCAGCTGCAGTTCC
TCCAGAAGGCTATGATCAAGTCCCTGTGGAGGCACCATTTTGCCTGGCCCTTCCATGAGCCAGTGGATGCCTTCAGACTC
AACCTCCCAGTGAGTTACACTGTTTGTCTATAAACAAGTGTAGAGATTAGTGGTCACCAACACTGGTCCTAGAGAGCCAT
AGGGGTTGCAAGCTTTTGTTACAACCCACCACCATGTAGGCCTACACCTATGAGTGTTTGTCCATAGTTGACATCCTTGT
CACACCACCATTCAACAGTCATTACTCTCTTTTTCTATATTCTGTTCTTTCTTGCTCTACTAGGATTATCACAAGATCAT
CAAGCAGCCCATGGACATGGGCACCATCAAGAAGCGTCTGGAGAACAACTACTACCGCAGCGCCAGTGAGTGCATGCAGG
ACTTCAACACCATGTTCACCAACTGCTACATCTACAACAAGGTATGGCCATAATATTTACATGTGTAGGTCAGGCTAATG
CATCGATGACAACGGACCGAATATGTTTATCACAAGGTCTTTCCATATTCACAGCAACTGTAGATGTACCGGTGTTAACT
GGATGTTGATTCTAAAGTGCCTGTGGCCTCTTCTCCTCTCCCAGCCCACAGATGACATAGTGCTGATGGCCCAGTCCCTG
GAGAAGGTGTTCCTGCAGAAGGTGGCCCAGATGCCCCAGGAGGAGATTGAGCAGCCCCCTCCTGCCCCCAGGGGCAAACC
GGGCAAGCCTGGCAAAGGGGGCCGCAAGTCCAACGGTGAGTCACTTTGGAGAATGTGGTAGGGAAAAGAACGCAAGGGTA
ATGTTCAACGAAGAGTTGGTGAATGTACTGTCTCGTTTGGTTAAATATGAGTTGGAACAACCTTGTTTAACGAGTAGTGC
ACTGTACATTTTTGGCCATCATATTCTTGGTAGTATTACCATTATGTGTGTGTTCTTGTGTATGATTTTTCTCTGTCCAT
CTTTCAGCTTCGAAAGCTCACCAGGTCCCTGCGGTGTCCCAGTCAGCATACTCCCCCTCCTCCTCTGACACGGGTGACTC
AGACACCCCCCCTCAGACCATGCTGCCCAAGAGTCTGCCCCCCACACAGCTGTTGAGCCTGCCCCCCACACTGCCCACCG
CCAAGGTATCACACCTTACTTCACTAAGGCCTGAATAGGAACTTGCTATAGTACTGTACATTGTATTGATGCTACACACT
TGTTGCTGCCTTCACACTGTATATAGGCTACAGAACCTCCATTGATAAACAGAATACCATACTGTAACTTCTCTCTCTCT
GTGCAGAAGAAGGGAGTGAAGCGGAAGGCGGACACCACCACTCCCTCCACCATGATGACCATGCCCCTGGCCGTAGGAGG
GGGTCGGATTGTGGGCCTGGGGAAAGGAGGCCACGGGCAAGGCCTGAGCCACGACCCCTCCAACCTCTCCCTCACCTCCC
TGGGGGACATGGACCCCCCTCCCAGCCTGGTTAGAGGACCCATGGGCCCAGGAGGCCCCGTTCTGCTCCAGCCCATGATG
GCCGGAGGCGGGAAAACCTTGGCTCGGCGCGGGGGCAGTGGACGCCCCATCAAGCCCCCCAAAAAGGATCTGCCGGACTC
TGTGCAGCCCAAGGCGCCGCGGCGGGCCAAGTTGAGCCAACAGCTGCGTTACTGTAACAGTGTACTGAAGGATCTGCTGT
CCAAGAAACACGCGGCCTACGCCTGGCCCTTCTACAAGCCCGTGGACGCCTCGGCCCTGGGCCTGCACGACTACCACGAC
ATCATCAAGTGCCCCATGGACCTCAGCAACATCAAGGTCAGCTTGCAAGATAGAGATGTCGCACTGCCTTGACTCAATGA
GAACTGAATATGGCTGAAATACACAGTTCATAGATTACCTCATATCTTCTCAGATTTGTTTTGCGTGGATATCTGTCACA
GTATGAAACCAGGGAGGCTTATCCCTATCTGGAGTCCTCTCCGTTATCTGTTGGAGTTTAAAACAGTCTCTTTTCTCTCC
TATCTTTCTCTCAGAGGAGGATGGACAGTCGGGAGTACCGGGATTCGCAGCAGTTTTCCGCTGACGTCCGACTCATGTTC
TCCAACTGCTACAAATACAACCCCCCCGACCATGACGTAGTGGGCATGGCCCGCAAGCTGCAGGTGAGCTCGGTTCCGTC
ACCACAACTCACCCTGGAACCCTTAGTTCACCTTTGTTGCCAGACCTCTATGTCCACTGCATGCTCAGTGGGTTTTACTT
GCAATGACTGCGATATGTGGTCGTCTTACCTACCGTGGCTGAACGCACTGACTGCAAGGCACTCTGGATAAGTCTACTTA
ATGACTAATATGTCAATCGTTTTGGTCTCCATTTCTCTGTTCTGTCCCTACAACTTTGTCTCTCGTTATAACTGTTATTA
TTGTGTAACAAAACAAAAAGGGACATGATACACAATGGAGTAAAAAACAAGCTCTCACTCACTCCGTTTTGTCTGTCTGT
CCATCCAGGATGTGTTTGAGTTCCGCTTCGCCAAGATGCCCGACGAGCCCCTCCAGCAGGCTCCTCCCAAGTCCCGTGGC
ATGGGCGGCGGCATGGGCGCTCACGGCGGCGGTGACTCCTCGTCTTCGTCGTCGTCCTCCTCCTCCTCGTCGGCGAGCGA
GCCCAGCAGCGAGAGCGAGAGCAGCCCCAGCTCGGACAGCGAGGAGGAGCGAGCACACCGCCTGGCCGAGTTACAGGACC
AGGTGTGTACACAGGTAGACTCAACCCCCCCCCCGTTCACCGAACACACTGCACCACACACACACCTAACACGCTGTACC
ACACACCTAACAGGCCTGCTTTCATCTACACATCACAAGCATAGACTGCATCAAGAGTTTAGACTTCTCTCCAACACAGG
TTTTTCTAATATTCTGACAAATCCATGCTCGTATACATACTTTCACTCATTTGGTGTACATGTTTATACTAGTACTCTGT
CTGTGTTCAAACATGCATGTACATAAGCCTGCTATTTGCTGCATTTTCTCACTATTACACTAAGGTAGGCTGTCATTGTA
AATAAGGATTTGTTACCAACTGACTTGCCTAGTTAAAAAAACAAAAAAACATTGGCGTATTCCTTCATGACCTAACCATA
TAAGTGAAGTGTATTTGTCCAGTTGAACACTCCACCTCTGGTCTGCCCCCCGGTGACAGCTCCGAGCCGTACACGAGCAG

CTGGCAGCCCTCTCCCAGGGCCCAATCGTCAAGAACAAGAAGAAGAGGGAGAAGAAAGACAAGAAGGAGAAGAAGAAGAA
GAAGAAGCCGGAGAAGCGAAGCCGAGGACGGCCCAGCGCGGAGGTAGGGGGTGAGGACTGGGAGATGCCGACGGCCAAGC
CGGTGTGGACTACGAAGAGTAAATCCAGTAGCAGGAGCTCAGCTCCCGCCTCCTCACAGGGCAAGAGAGGCCCCGGCAAG
AAGAAGGGCAGCAAGTAAGCACACACTGACCTCAAACATGACCTTTCTCTTTGCTTTTACTCAACTTTTCGATCTGTGAT
CCATGTCCTCTATTTCTCCTACCTTGCTTGTTTCCGTCTCTCATTTTGTAGACTTTCACATTGCTTCCTCTTTCCACCTT
TTCTGTTTCCTCGCTTTGCTTTTTGAAAGTTTCTTACACCATTTTATACTTCCGCTCTTTCATCACATTCCAATTTCCAA
CTTTACCTGTTTGTCTCTCTAGAAACTGCAGTGGGGTCGCAGAGTACAGATTATTGTATGGGACCGTATATCAAGTTCAT
TTTGATAAGAGATGAAAATGTAGTTAATTGAAGGTGATTTGTTGATGTAAAAATGTAAATGTACCGAAATGTAGGTTGTC
ATTATATTTTGGGTGGGGGTCACGATAAATTGTGACAAAAAATGATAATTGGGTCCCCACAAGTTCGAGCGTGAAAAAAA
ATGTCTCCCTGCTGGAAAGGTTTGCATACAACTTCTTTAGTACTACTCCACGGTCTCTAACCCCCCCCTTTTTTTAGAAA
CACCAAGAAGTCCCAGGCTGCTACCATGCCTTACTACGCCCCGCCTACTTTCTCCATGCTGCCCCACTACGACTCCGAGG
AGGAGGAGGAGACGTCTCCCATGTCGTACGACGAGAAGCGCCAGCTGAGCCTGGACATCAACAAGCTGCCCGGCGAAAAG
CTGGGCCGCGTCGTCCACATCATCCAGTCCCGCGAGCCCTCGCTGCGCGACACCAACCCCGAGGAGATCGAAATCGACTT
TGAGACGCTCAAGCCGTCGACGCTGCGCGAGCTAGAGCGCTACGTCATGACGTGCCTGAGGAAGAAGCCCCGTAAGCCCT
ATGGTGAGCGGGAACAGATAGCGATAGCTAATGCTAGCTTTGGTTAGCTAGTATGGTGTGAAAGGGAATGCATCATGTTG
TCACTGAAGACACTTGCTAATGTTATGCTACAAAGAACTGATGCTACGAACTGGTGCTGTGGTGATAAAATGCTATATGC
TAGCGCTCTGGTTGACTAATACCATACAAAAGGAACCAGTCTTTCACTATGGAATGTTTTTATATGTATGTGTGGGCTGA
GTATGTAACCTGTCCTCCTGTCTGTGTCTAACAGTGAAGAAAGGGGGCCCCGGCAAGTCCAGAGAAGAACTGACCCTGGA
GAAGAAGAGGGAGCTGGAGAGGAGGCTGCAGGATGTCAGCGGACAGCTCAACTCTGGCAAGAAACCCCAGAAACCCAAGG
GTGAGCACCACAGACACGCGGAGCTGCTTTTGCACATGTAGCATTTAGCATTGAGATAAATGCTAATCCTATTCCCACTT
TGTCTCTCTCTCGCAGCGGAGAAGCTCAGCGGCGTGGAGCCCCATGCCCAGCCCTCCCGCCTCAGCGGCAGCAGCTCCAG
CTCAGACTCTTCCTCCTCCTCGTCATCCTCCTCTTCTGACACCAGTGAATCAGACTCGGGTTGATGAGACAGTGGGAGAG
GGAGGGAACCCCCCCGTAGAGCTCAGGGACATGCAGCAGCCCCAGGACAAGGTCTCCTGGGACGGTCAAGGGAGGGAGAG
AGTGCCCCTCCCTCGAAAGGCACCCCAGCACGAACACTCCAAGATGAATGATGGTGGGATTTGTATGCAGGGTGGAGGAT
GCGTGAAGGTCACTAAGGATACTCAAGACCATAAACATAGAGTTCAGCCAGGTTTAAGAACAGCTGCCATGTTAGCGCCT
TTATCCTCAATGTTGGTAATGTAACAATATGAGAGCACCTCTGACCGTTCCCTATGAAATGACCATTCAGGATAATGTAT
ATCCAAGTCTTTACTGTGTTGTAGTGTCAACAAATTGCATATCTGCTTTCAGACATCTTCATAGGTTTTGAGACTATCAG
AAATAAACAGCACAGCGGAGAAGCGTCTATCACTTAGCAACGGCTATGGAGGAGGAAGTGGCTCTCAGAACGTTCAGACA
GAAACCGCATCGGCCCAACTCTTTGACTCTGCTCAAGACCCCCCAACACAAATGCCTTGTTATCCCTCTCATCCCTGTGT
AAATACTTGTACAATTTATCTACGTTTCTTTTATAAAGAAGAATTTTATGGCGATCCCCCAAAGAGGGAGACTGCGGAGT
AGTGAAAGGATGCGAGATGTGCCCAGCCCAATGTTGGACACTTCTTGATGTGCACTCATAGATTGATAAGAACTGAATGG
GTATAAGCAGTATGGCAGAAACCCCACTTGCCCTCCCAGAAAGCTATGTGGAGTGAGTATTGTAATGCTTACACTTATCT
GTTCTTTCACAACGAGGACGTCACTGTCAAGTGATTGATTTTCACATCTGGTAGTTATGTCTTTTTTTTGTCAAGTCTTA
TAGCTGCAGTCACGAGTCAGTACATTTTTGTTGCTGTCTGTGTTTCATTTTACATCTGTTCTTACTGCTAACAGACCTAC
ATTCAGCTGGACATAAAGTTCAGAGGCAGAAGGAGAGTTTTATTTCCATTCCATATGTTACTCTTAAGAGATCTAACTAC
TGAAACTGTTTTCCTTGACAGTGAGATTTGTCATACATGTACGGCACTTACCATTTAAGAAACAGCCCATGTGTCATTTC
TCAGGAAAGGAGTAGGTTTCTTTATGGTGACACTGCAGTCTTACAATCACAGCAGTCATAGAAACTGATTGGTCTTCAGT
TCTAAGCAATTCCGTTAACTCTATGCTTAAAGGAAAATAACAATCAATCATTTGGTATTTGTTTCGTTAGTCCACTGTTG
ATGAAGTTCCACAATGTTTTGCATGTCAGATATCAAGTTTTCAACAGAACTTTCAAAATACAGGAAGTGTCACAGTGTGA
TGCGCTCTGCAAGCTAACTGTTGGATGTTAAATCCTCATGTATACGGGAGTAGACGTCCTAAGTTGAAGTTTGGACCAAG
AGATACACCAGATGAGATGTACAGAAGCTAGGTTGTTAGATATCTAAGGAAATTGTGTGTGTGGGTGTATATATATATAT
ATTACTGTATTTCAGTGGTCAAATGATCATGTTTCTTTCTACCAGTGAGTCTTTTTTTCCCTGTCTTGCTTTTCTTTTAT
ACTGTATTTATCCTCCTCTTAGTCATGCTTTATTCCAGAACATGTCCTGAATGTTGTTTTGCATCCTGGTTGACTAGATT
GCTGCCTCAGGAGCATGCCACTCATTTGAAAACTGTATATATTAAAATGAGACCTCTTCTATAAAGCCTTTTATATTTCT
TTGTGATAATGTATACGCAGTACACCCCTATTATTTATACAGTGCCTATGGAAAGTATTCCGACCACTTGACCTTTTACA
CATTTTCTTACAGTACAGCCTTATTCTAAAATTGATGAGGGAAAACAAACAATTTAATCAATCTACACACAATACCCCAT
AATGACAAAGCGGTTTTTAGAAATGTTTGCCAATTTATAATAATGAAAGAAAAAAAAATCACATTTACATAAATATTCAG
ATCCTTTACTCAGTACTTTGTTGAAGCACCTTTGGCAGCAATTACAGCCTCAAGTCTTCTTGGGTATGACGCTACAAGCT
TTGCACACCTGTATTTGGGGAGTTTCTCCTATTCTCTGCAGATCCTCTAAAACTCTGTCAGGTTAGATGGGGAACATTGC
TGCACAGCTATTTTCAGGTCTCCCCAGAGATGTTTGATCGGGTTCAAGTCCAGACTCTGGCTGGGCCACTCAGGGACATT
CAGAGACTTGTCCCGAAGCCACTCCTGCATTGTCTTGGCTGTGTGCATAGCGACGTTGGCCTGTTGGAAGGTGAACCTTC
GACCCTAGTCTGAAGTCCTGAGAGCTCTGGAGCAGGTTTTCATCAAGGAACTCTCTGTACTTTGATCTGTTTGTGTCAAA
GGAATTTTTCTATCCCAATGATAATTAACAATCAATAAGCCTTGACTAATTCCCAAGGTTTGTAAGACAATGGGTTAATA
ATTAGGCAAGGACTCAGCTTTCTGCAAAAGGTTCGTACAGTTTATTCAGAGAACGTTCTGCCCAACATTAACAAAGAATT
TCATTTTATCCTCTCTCCGCTTACGCACACACCTCCATAGCAACAGTAGATAACCTACGCACACACATACACACAAACAG
TAGGTGAACAGTATGCCTCCCTTGACTTCCCACCACTGTTAATCACTATCCAGCGCTGCCTGTTCCTTTCCCCCGAGATT
AGGGGGATCTTGAAGGCTACTCCTGGTCTCTACACACACATTTCTCTCTCTTCCGGGTCTCTACTAGACCTTATGGAACT
ATCGTTCAGTACTTTAATCATTCATTACCCATGCTACATAACTACTAATTAATCATGGATTATTGATTCATTTACCTTTA
TTAGATAATTCTCTTATCAGTTTGTCTTTGCCTCAATCCTGACTAGTCTGCCATTCCCTGTCGCTGAAAAACATCCCCAC
AGCATGATGCTTTACCGTAGGGAGGGTGCCATGTTTCCTCCAGACTTGACACTTGGCATTCAGGCCAAAGAATTCAATAT
TGGTTTCATCAGACCAGAGAATCTGGTTTCTTATGGTCTGAGAGTCTTTAGATGCCTTTTGGAAAACTCCAAGTGGGCTG
TCATGTACAGTTGAAGTCGGAAGTTTACATACACTTAGGTTGGAGTCATTAAAACTCATTTTTCAACCACTCCACAAATT
TCTTGTTAACAATCTATAGTTTTGGCAAGTCGGTTAGGACATCTACTTTGTGCATGACACAAGGAATTGTTCTAACAATT
GTTTACAGACAGATTATTTCACTTATAATTCACTGTATCACAATTCCAGTGGGTCAGAAGTTTACATACACTAAGTTGAC
TGTGCCTTTAAACAGCTTGGACAATTCCAGAAAATGATGTTTAACGATTTTACTGAGTTAGAGTTCATACAAGGGAGTCA
GTCAATTGAAATAAATTAGGCCCTAACCTATGGATTTCACTGGGAATACAGATATGCATCTGTTAGTATGGGTCAGAAAA
CCAGTCCGTATCTGGTGTCCACCATTTGCCTCATGCTGTGCGTTTTCAGGCTGTTGATCGTGGTCTGTTGTCCCACTCCT
CTTCAATGGCTGTGCCAAGTTGCTGGATATTGGCGGGAACTGGTACACGCTGTCGTACACGTCGATCGAGAGCATCCCAA
ACATGCTCAACGGGTGACATCTGGTGAGTATGCAGGCCATGGAAGAACTGGGACATTTCCAGCTTCCAGGAATTATGCAG

AACCTTGCGGCATGGGGCTAGGCATTATCATGCTGAAACATGAGGTGATGGCGTTGGATGAATGGCAGGACAAAGGGCCT
CGGGATCTCGTCGCGGTATCTCTATCAGTGGAGGCTGCTGAGGGGGAAACGGCTCATAACAATGGGCGGAACTGTGAAAA
TGGAATGGCATCAAACACCTGGAAACCGTGAGTTTGATGTATTTGATACCATTCCACTGATTCCGCTCTAGTCATTACCA
CAAGCATGTTCTCCCCAAATGAAGGTGCCACCAACCTCCTGTGCTGTGCATTCAAATTGCTAGCAATAAAATGCAATTGT
GTTTGTTATCCGTAGCTTATGCCTGCCCATACCGTAACCCCACTGCCACCATGGGGCACTCTGTTCACAACAACAAACCG
CTCGACCACAGGACGCCATACATGCTGTCTGCCTGGTACAGTTTAACCTGGGATTAATCCGTGAAGAAGACACTTCTCCA
GCATGACAGTGGCCTTCAAAGGTGAGCATTTACCCACTGAAGTCGGTGACGACGCCAAACTGCAGTCAGGTCAAGACCCT
GGTGAGGACAACGAGCACGCAGATGAGCTTCCCTGAAGGTTTCTGACAGTTTGTGCAGAATTACTTTGGTTGTGCAAACC
CACAGTTTCATCAATGTCCAGGTGGCTGGTCTCAGACGATCCCGCGGGTGAAGAAGCCAGATGTGGAGGTCCTGGGCTGG
CGTGGTTACCCGTGGTCTGCGGTTGTGAGGCGGGTTGGACGTACAGTATAACTAAGGGCAGACACTAATGATCATGACTC
CATAGTAGGTGGCAGCAGTCAGATGTGGGCTATGTCAGGGCTATACTGATCATTCAGATAGAAATGTTTTGTGTAGAATA
GGGAGTCATGTCTCAGATCTATTTATTGCGTTTCTATCTGCAAAGTTCTGCATGTTTCACCTTTGATACACGCTAGTTGT
GTGTAAGGTGTGCCATGCATACTCAGAATGCCTAACGTGTTCTATGTAGTAACCATAGATATGGTAATAAACAGTGTCAT
CCAGATTAAAGATTTGATTGGCTGATGATATTGAGGAAGTCACCTGACACCTTCAGCAGGACTGTTTGGAGTACAACACT
AGAATCGGAACAAAAACATTGGTACATTACTGATGAGCCTCACTGGACAAGTGTACTTAGAATGGGGTGCAGAGTGTTCC
TGGCTTTAATACTGGGTGTTTGTCTACTGTCACACTGCCAAAGTAAGTCCACTTTCACTCATTTCATATGAGAGACAGGA
ATGTGATTAGAGGAAGGAAGGAAGCAGACTGGGCATTATTTTGCTTGCAAGAATCACTTTAGCAGTGCTAACCCATACTG
TGTTCCAGTATTCTATTCCGTAAACTCATAGATGCAGACAGCTACTTTATATCAGTTCCCATTCTTATTAAAACAACATT
TACTGAAAGGCGTAACCAAATTAATTATATTGCATTCAACTACTCTCGTACCAGAGGTCAAGATTTGTCATGTTTGTCGT
TTTCCATCTCAACTTGTGTTACTGAACACAGGCAAACATTTGCTGCGGTTTTTGACCTTCTGCCAAAAAAACATCACTCA
TACAAAGAGTATGACGTAGAGTTTGAGGATGAGCTCTTCTACGTTGACCCCATGACCTACCGGGTAGAGCGGCGACTGTC
AGCGTTCGCCCAGCAGTGGACCCGTGACCCAGGACTGCCTCACGAAGTGAACGTGTCCCTCGGAACCTGCCAGTACAATA
TCCCCTGCTGCATTGTGGGAGAAAATTTGGGGCGGCAGGTAGCCTAGTGGTTAGAGCGTTGGGCCGTGTAACTGAAAGGT
TGCAAGATTGAATCCCCGAGCTGGCAAGGTATGAATCTGCCCCTGAACAAGACACTTAACCCACTGTTCCTAGGCCGTCA
TTGTAAATAAGAATTTTTTCTTAACTGACTTGCCTAGTTAAAATAAGGTTTACCTTAAAAAAAAAAAAAAATTTTAGAGTC
CACCGGAGGCATTAGGTGAGTGCTGTTTCTGTAAAGTCACACCCCAGCTAGCACATCATTATCTGTTTCTTAGAGCTTGG
TGAGAGTGTGGTTGTCCTATGGTTATTTTGCATATAACCTTACCACAACGTTCTGGGAATGGTGCGGGGTAGTTGCTTGG
CTTTGGAACATTCTCAGCACATTTAAGGAACTTGACGAAGTGGTTTGATATTGGGAATGTTCTCAAATAGTTCAGAGAAG
GTTAAGAAACAACATTCTTCTGTGTGAATTTCAGTACTTCAGCATAACGTTTCCTCATGGTTCTATTTAAAGTCATGTTC
TCAAAGTTCAGAGACTGTTACAAAAACGTTCCATAAAAACCACAAGAAAATGTTTGTAACGTTCTAATAATGTTATTTAA
AAGCATATACATTCCGTTCTCAGCGTCAATAAAAGGCTCTATCCTCCATCTTAAGTGTGTTCAGGTGTTATGGCCGTGCC
CATTAATTGGCCACCCCTGATCTTAATGAGTGCTTCTTTCCTTTGAAGTGGGATCTGTTTGAATAGACTAAAATGAACAG
CTTTGTATGCCTTAAAATTCTAGTTCCACCCTGGTGGCGAAGTGGACTACGGTGTCTTGTACAGCAAATTGGTTTGGTAT
GTTTTTTTATGAACTCTGGTGCATTTAGCCCCTTAGTTCAGTTCGTTTGGACTGGTGTGAACACTGTCCTCACTCGGGAG
CGCCCAACAGAGGCTCCCACCTCCCACATATACTCCCAGAGAGAGGTGGAACTGGGGGACCCCAACACACTCATCTGCCG
GGTCAGTGATTTCCACCCAACATCCGTGGATGTGACTTGGACCAGGAACGAGCAGCCGGTGGGAGAGGGGACCGTCAGTC
TGACCCAGTACTACTCCAATGAAAACTTCAGCTTCAGGATCTTCTCCTACCTGAGCATCACACCTCAGGAGGGGGACATC
TACTCCTGTAGTGTGGGGCACGTCAGCCTGCAGGAACCCCTCACCAGGATCTGGGGTGAGTCACTGCTGGTGTGACCTTA
TACTGTAGGGTATGACACAGAGAAAGGTGGGATGTTGTTCATACTGCATTAGGGACACTAACACATCAATTGTTTCTTCA
CTATATGTGTCCACAGAGGTTGAAGTGCACACGGACCACCAGACTGTGGAGACAGCAGTGTGTGTTGGAGGTGTGACTCT
AGGAGTGGTGGGAGTAGCAACAGGAAGCCAAGAGGTCTGGTTGGGCCTTAAGAACATAGGCAACTATCAGCAATACACCA
AGGGCTCCATGCAACAATACCAAGTACAATTATCATATTCCGCAACCTACAACTGTGCTGGCGACAATATGATGCAGTTC
ACTATTGTGAGGGCAATTTCTGTTAAACCTTACTAATGCTGTTGTACTAAAATATGTCTTTAAGCCTAGGCATTGGGCTT
GGCTGGACTTCCTGTCATTCACACCTGGGAAACACCAGCAGGTGAGTCAATCCAATTTTTTTTGATGAAATGCATGATGT
GTCTAATATATTATGAAGAATGTATCAAATGAAGTTGACAGATCTTTTGTAACAATACAGTATAACAACTCCACAAAGGC
AATGATTAGGAGATTTTTCCTAAATGGATCCATGTTGTAAGCAACAACATATTTTTTGAGATTACCTCTTTGCACAATGA
CATGGAAAGGGATACAGCAGTACAAATTAATTATTTGTTTCAGGTGGGTACCAGTTCCAGGGGATAGTTGACTGTGAATA
TGACACCATTGACAATATGATCTACTTTGTGAAAAACATATTCAACCAAAAATTGACCATCTATGGCGCCAGGGTTCAGA
AGTATATGGGTTTTGGGGAGTATGGCATACGCAATGCAGATCACTACAACAGCCAAGCTTGGAAAATGGCTATAAGGAAG
GCATAAGTGGAGACTATTTGCCCATACAGTGCAAGATTCTTCAAACTGAGCACTTTGGAAAGAATAGGTAAGTCATTTTT
ACGTTTCTTTCCTTAGTAATTTGCACGTGCTGTACATTAAGTCAAGTTTGATAAAATATTTGTTTGCAAAAATGTTGCTG
TTTCGGTCTGTGGACCTCCATCAGACAACTTAATTTCTACGTCATCCTATGTCCCAGTGCCTCCCACTGTCAAAGTCTGC
CTGACCAAGACCTCTCGCTACGGAGAGCTGTCCATGCTGGAGTGGATTAGAGAGAGATATCTCTGCTTATAGCAGTGTTT
TTTTTAAATAAATGTCAATAGATTTTGAAGTTACTGGATTGGTAGCAGCTTTGTGGTAGTGTGATGTTTTACATAATTCC
ATTCCCAGATACCTCAGGCCTGCATACTAAATGGTTTAAAATGGCAATAGGGGTGTGTTCCCTTTTCATTGGTGTAGCCA
TGGCTATTGGAGGAGTTTACTACTGGTGGAAGAACAGGAAAGTCACAACATCACACAATGGACAGAGATTTTATGTAACT
ATAAATCTATTGGATTATTTTTGATTTCAAGAGGTTTCATAATGTAGACATATTTTACTGTTTCAGGTCAGGCTTTAGGC
GTGTGAATAGATAAGGTAAGGCATTTTTCCATATTGTGTTTGACATATTTTGCAATAAATAAACATGTCTGATATATATA
CTGCTGGAATCTTTACATTCCATACATCAAAGCAGAGAATTGCATAGACAATCCTTGTGCACCCAAATGTCACATTTGAA
CTTTTTTTTCTTCTTTCCAGTAGGTCAATTGACTGCTTCGATATGCATGGCATAAGAGCGAACTCTTTCTTTGCCCAAAA
TGCCACTGCTTAACAAAGCAGAAATAATACTTTCATTCTTTACCATACTATTCAAATTGTGTCCATATGATGGAATCAGN
NNNNNNNNNNNNNNNNNNNNNNNNNNNNNNNNNNNNNNNNNNNNNNNNNNNNNNNNNNNNNNNNNNNNNNNNNNNNNNNN
NNNNNNNNNNNNNNNNNNNGGTATGACATGGGTATTCAACTCTTAGGTCCGATGCCTGCTGGCTTTCTGTTCTGCGGGAT
AATTAATTGCATCCACCTGGTGTCCCAGGTCTAAATCAGTTCCTGGTAAGAGGGGAACGATGGAAAAAAAGCAATGGGGC
TGGCTACGAGGTCTAGAGTTCAGTTTGAGGGGTATGACAGAACCAACTAGATGAAAACCAGTAAAAATAACAGTATACAC
AATGTAGATGAACAGAAATTATTAAGTGATCGGTTTGAATGTCCTCTTACAGTTTGCATTAAATCAATTAACCTTTATGG
CCCTCTGGGCACAAAATGATATTCCTTTAATTAACCTTGGCGTATCATTATATAAATATTTAGTCTGATAAATTCCCATC
TTAGAACAAGATTACCAAAATTCTCCAGACATTCAGTATGCCACATTCAGCAGTTTTATCCCAAACCAAACATCAGTTCA
GGTCTTAGTGGTAAACCCCATGGCCACAGCGCTTTAGGCCATAAGTACAGTTGTTCAATACTACAACTGCAGGCTGGAA

ATGAGGATTTTTGACACGCTCTAGTGGTCCACCTCGTCCCCTTGGTCATTTAAGATCTAATAATGCTTCCTGTAGTGGAC
AACATGGATATGATGTGGTTTCATAGCCCTCCTTATAATGACCCTGTCCTCCCCAGGGCTCCTAGCTCTCATGATGTATA
GATCACCCCTGACTGTGAAACTACGTAAATACGCTTGTTTTCACAAGCACCATTACCATGAGTTGTGAACACATGGGGCC
CACGTAATGTATTTAATACAGCAGCATAATGCATATACATCTGATCAACTGTGACCCCTTCTGGTACTGAAAATGTAATA
AGTAGATACGACAGATTTTAACCATAGTTATGTTCGGGGGNNNNNNNNNNNNNNNNNNNNNNNNNNNNNNNNNNNNNNNN
NNNNNNNNNNNNNNNNNNNNNNNNNNNNNNNNNNNNNNNNNNNNNNNNNNNNNNNNNNNNNNNNNNNNNNNNNNNNNNNN
NNNNNNNNNNNNNNNNNNNNNNNNNNNNNNNNNNNNNNNNNNNNNNNNNNNNNNNNNNNNNNNNNNNNNNNNNNNNNNNN
NNNNNNNNNNNNNNNNNNNNNNNNNNNNNNNNNNNNNNNNNNNNNNNNNNNNNNNNNNNNNNNNNNNNNNNNNNNNNNNN
NNNNNNNNNNNNNNNNNNNNNNNNNNNNNNNNNNNNNNNNNNNNNNNNNNNNNNNNNNNNNNNNNNNNNNNNNNNNNNNN
NNNNNNNNNNNNNNNNNNNNNNNNNNNNNNNNNNNNNNNNNNNNNNNNNNNNNNNNNNNNNNNNNNNNNNNNNNNNNNNN
NNNNNNNNNNNNNNNNNNNNNNNNNNNNNNNNNNNNNNNNNNNNNNNNNNNNNNNNNNNNNNNNNNNNNNNNNNNNNNNN
NNNNNNNNNNNNNNNNNNNNNNNNNNNNNNNNNNNNNNNNNNNNNNNNNNNNNNNNNNNNNNNNNNNNNNNNNNNNNNNN
NNNNNNNNNNNNNNNNNNNNNNNNNNNNNNNNNNNNNNNNNNNNNNNNNNNNNNNNNNNNNNNNNNNNNNNNNNNNNNNN
NNNNNNNNNNNNNNNNNNNNNNNNNNNNNNNNNNNNNNNNNNNNNNNNNNNNNNNNNNNNNNNNNNNNNNNNNNNNNNNN
NNNNNNNNNNNNNNNNNNNNNNNNNNNNNNNNNNNNNNNNNNNNNNNNNNNNNNNNNNNNNNNNNNNNNNNNNNNNNNNN
NNNNNNNNNNNNNNNNNNNNNNNNNNNNNNNNNNNNNNNNNNNNNNNNNNNNNNNNNNNNNNNNNNNNNNNNNNNNNNNN
NNNNNNNNNNNNNNNNNNNNNNNNNNNNNNNNNNNNNNNNNNNNNNNNNNNNNNNNNNNNNNNNNNNNNNNNNNNNNNNN
NNNNNNNNNNNNNNNNNNNNNNNNNNNNNNNNNNNNNNNNNNNNNNNNNNNNNNNNNNNNNNNNNNNNNNNNNNNNNNNN
NNNNNNNNNNNNNNNNNNNNNNNNNNNNNNNNNNNNNNNNNNNNNNNNNNNNNNNNNNNNNNNNNNNNNNNNNNNNNNNN
NNNNNNNNNNNNNNNNNNNNNNNNNNNNNNNNNNNNNNNNNNNNNNNNNNNNNNNNNNNNNNNNNNNNNNNNNNNNNNNN
NNNNNNNNNNNNNNNNNNNNNNNNNNNNNNNNNNNNNNNNNNNNNNNNNNNNNNNNNNNNNNNNNNNNNNNNNNNNNNNN
NNNNNNNNNNNNNNNNNNNNNNNNNNNNNNNNNNNNNNNNNNNNNNNNNNNNNNNNNNNNNNNNNNNNNNNNNNNNNNNN
NNNNNNNNNNNNNNNNNNNNNNNNNNNNNNNNNNNNNNNNNNNNNNNNNNNNNNNNNNNNNNNNNNNNNNNNNNNNNNNN
NNNNNNNNNNNNNNNNNNNNNNNNNNNNNNNNNNNNNNNNNNNNNNNNNNNNNNNNNNNNNNNNNNNNNNNNNNNNNNNN
NNNNNNNNNNNNNNNNNNNNNNNNNNNNNNNNNNNNNNNNNNNNNNNNNNNNNNNNNNNNNNNNNNNNNNNNNNNNNNNN
NNNNNNNNNNNNNNNNNNNNNNNNNNNNNNNNNNNNNNNNNNNNNNNNNNNNNNNNNNNNNNNNNNNNNNNNNNNNNNNN
NNNNNNNNNNNNNNNNNNNNNNNNNNNNNNNNNNNNNNNNNNNNNNNNNNNNNNNNNNNNNNNNNNNNNNNNNNNNNNNN
NNNNNNNNNNNNNNNNNNNNNNNNNNNNNNNNNNNNNNNNNNNNNNNNNNNNNNNNNNNNNNNNNNNNNNNNNNNNNNNN
NNNNNNNNNNNNNNNNNNNNNNNNNNNNNNNNNNNNNNNNNNNNNNNNNNNNNNNNNNNNNNNNNNNNNNNNNNNNNNNN
NNNNNNNNNNNNNNNNNNNNNNNNNNNNNNNNNNNNNNNNNNNNNNNNNNNNNNNNNNNNNNNNNNNNNNNNNNNNNNNN
NNNNNNNNNNNNNNNNNNNNNNNNNNNNNNNNNNNNNNNNNNNNNNNNNNNNNNNNNNNNNNNNNNNNNNNNNNNNNNNN
NNNNNNNNNNNNNNNNNNNNNNNNNNNNNNNNNNNNNNNNNNNNNNNNNNNNNNNNNNNNNNNNNNNNNNNNNNNNNNNN
NNNNNNNNNNNNNNNNNNNNNNNNNNNNNNNNNNNNNNNNNNNNNNNNNNNNNNNNNNNNNNNNNNNNNNNNNNNNNNNN
NNNNNNNNNNNNNNNNNNNNNNNNNNNNNNNNNNNNNNNNNNNNNNNNNNNNNNNNNNNNNNNNNNNNNNNNNNNNNNNN
NNNNNNNNNNNNNNNNNNNNNNNNNNNNNNNNNNNNNNNNNNNNNNNNNNNNNNNNNNNNNNNNNNNNNNNNNNNNNNNN
NNNNNNNNNNNNNNNNNNNNNNNNNNNNNNNNNNNNNNNNNNNNNNNNNNNNNNNNNNNNNNNNNNNNNNNNNNNNNNNN
NNNNNNNNNNNNNNNNNNNNNNNNNNNNNNNNNNNNNNNNNNNNNNNNNNNNNNNNNNNNNNNNNNNNNNNNNNNNNNNN
NNNNNNNNNNNNNNNNNNNNNNNNNNNNNNNNNNNNNNNNNNNNNNNNNNNNNNNNNNNNNNNNNNNNNNNNNNNNNNNN
NNNNNNNNNNNNNNNNNNNNNNNNNNNNNNNNNNNNNNNNNNNNNNNNNNNNNNNNNNNNNNNNNNNNNNNNNNNNNNNN
NNNNNNNNNNNNNNNNAGCAGGCGCAACTATACCAGCTGCTTTTGAAGTCACTGCCAGTATTGCTATTTTTAACTCAAAGT
CCTAGTACCACCTGCCTCTTCCCATCCCCTGATAACAGTATGGAGGCACCATTTGGAGGCGTGAGTGAGGAAGGTTATAA
GAGTTCACTGTCTTTGTTCCATCCCTTATTTGGATGAGACTGACTTCTGTCAACATGACTACCGCTCTGTCCTTCCTGGC
TGTGCTGGCCCTTGGGTCACGTCTACCTATCTTGCCTGAGGAGCAGCCCTATGTGGAGCATGCTGGTATTCAATACAGCG
CTAGGCCTTACACCTCCCTCCTCACCGTGCCCAATGGAGAGCAGTTTGGGAACTGACCCTGGCCCGAGATGTGTCCCGAC
AAGTTCTTCGCCGTTGGGTTCATGCTCCGGGTAAGTTCTTCGCCGTTGGGTTCATGCTCCAGGTAAGCAAGTGCTCCTTC
ATACATTTCAAACACATCTATCCGTTTGGATAGCATTTCTTTCAATTAGCCAATTTTGACCAGTCCTCTGTGGTCAAAAT
GCCTATTTTTGGACAGCCCAAAAAAATAAGATACAGGTTTATCTGTAAAAGCGCATTTCAAACTATAGTGATGATGACAT
AAACTCAGCAAGAAAATAAACGTCCTCTCACTGTCAACTGCGTTTATTTTCAGCAAACTTAACATGTGTAAATATTTGTA
TGAACATAAGATTCAACAACTGATACATAAACTGAAGTTCCACAGACGTGACTAACAGAAATGGAATAATGTGTCCCTGA
ACAAAGGGCGGGTCAAAATCAAAAGTAACAGTCAGTATCTGGTGTGGCCACCAACTGCATTAAGTACTGCAGTGCATCTC
CTTCTCATGGACTGCACCAGATTTGCCAGTTCTTGGTGTGAGATGTTACCCCACTCTTCCACCAAGGCACCTGCAAGTTC
CCAGACATTTCTGGGGGGAATGGCCCTAGCCCTCACCCTCTGATCCAACAGGTCCCAGACGTGCTCAATGGGATTGAGAT
CTGGGCTCTTCGCTGGCAGAACACTGACATTCCTGTCTTGCAGGAAATCACGCACAGAATGAGCAGTATGGCTGGTGGCA
GGGAGGTCTGCAGGAAGGGTACCACATGAGGGAGGAGGATGTCTTCCCTGTAACGCACAGCGTTGAGATTGCCTGCAATG
ACAACAAGCTCAGTCCGATGATGCTGTGACACACCGCCCCAGACCATGATGGACCCTCCACCTCCAAATCGATCTCGCTC
CAGAGTACAGGCCTCGGTGTAACGCTCATTCCTTTGACGATGAACGCGAATCCGACCATCACCCCTGGTGAGACAAAACC
GTGACACGTCAGTGAGGAGCATGTCCAGAGACGGTGGGTTTGTGCCCATAGGCATGTCTGGTGAGGACCTGCCTTACAAC
AGGCCTACAAGCCCTCAGTCCAGCCTTTCTCAGCCTATTGTGGACAGTCTGAGCACTGATGGAGGGATTGTGCGTTCCTG
GTGTAACTCAGGCAGTTGTTGCCATCCTGTACCGCAGGTGTGATGTTCGGATGTACCGATCCTGTGCAGGTGTTACACGT
GGTCTGCCACTGCAAGGGCGATCAGCTGTCTGTCTTGTCTCCCTGTAGCGCTGTCTTAGGCGTCTCACAGTACGGACATT
GCAGATGTGGCCAGGGCAATAAATTGCAATCTTCATGCCTCCTTGAAGCATGCCTAAGGCACGTTCACGCAGATGAGTAG
GGACTCTGGGCATCTTTCTTTTGGTGTTTTTCAGAGTCAGTAGAAAGGCCTCTCTAGTGTCCTAAATGTTCATAACTGTG
ACCTTAATTGTCTACCGTCTGTAAGCTGTTAGTGTCTTAATGACCGTTCCACAGGTGCATGTTCATTAATTGTTTATGGT
TCATTGAACAAGCATGGGAAACCGTGTTAAAACCCTTTACAATGAAGATCTGTGAAGTTATTTGGATTTTTACGAATTCT
TTGAAAGACATGGTCCTGAAAAAGGACGTTTGTTTTTTTGCTAAGTTCATTTCATTGTCCAAAGGAGACATAAATGGGCT
TATCAGTTAAACCCTGCATCTTCAAACTGTGGTGATATGACATGTTATTCCATGTCATGTTTCTACTCTTGCTATAATGT
GCCTCTTTTCTCTGTCTCACCGCTATGGTCACCGCACATGAAGTGTTTAAATGCAGACAAAATATGCATTTGTTTGCTGA
GTCACAAGCAGCATCAGAGGTCAGTACATCCAACCAGATGAGCAGGTGACTGTCATGACCACTAGATGGCAGTGTTATCT
TAGAGGTGAATTGACATGGTTCAGTGGATGGATGGTATGACATGGGTATTCAACTCTTAGGTCCGATGCCTGCTGGCTTT

CTGTTCTGCGGGATAATTAATTGCATCCACCTGGTGTCCCAGGTCTAAATCAGTTCCTGGTAAGAGGGGAACGATGGAAA
AAAAGCAATGGGGCTGGCTACGAGGTCTAGAGTTCAGTTTGAGGGGTATGACAGAACCAACTAGATGAAAACCAGTAAAA
ATAACAGTATACACAATGTAGATGAACAGAAATTATTAAGTGATCGGTTTGAATGTCCTCTTACAGTTTGCATTAAATCA
ATTAACCTTTATGGCCCTCTGGGCACAAAATGATATTCCTTTAATTAACCTTGGCGTATCATTATATAAATATTTAGTCT
GATAAATTCCCATCTTAGAACAAGATTACCAAAATTCTCCAGACATTCAGTATGCCACATTCAGCAGTTTTATCCCAAAC
CAAACATCAGTTCAGGTCTTAGTGGTAAACCCCATGGGCCACAGCGCTTTAGGCCATAAGTACAGTTGTTCAATACTACA
ACTGCAGGCTGGAAATGAGGATTTTTGACACGCTCTAGTGGTCCACCTCGTCCCCTTGGTCATTTAAGATCTAATAATGC
TTCCTGTAGTGGACAACATGGATATGATGTGGTTTCATAGCCCTCCTTATAATGACCCTGTCCTCCCCAGGGCTCCTAGC
TCTCATGATGTATAGATCACCCCTGACTGTGAAACTACGTAAATACGCTTGTTTTCACAAGCACCATTACCATGAGTTGT
GAACACATGGGGCCCACGTAATGTATTTTAATACAGCAGCATAATGCATATACATCTGATCAACTGTGACCCCTTCATGG
TACTGAAAATGTAATAAGTAGATACGACAGATTTTAAACAATAGTTATGTTCGGGGGTTTTTTACCCCTTGTGCCCCCTA
AGAAAATTACATTGTCTGTAAGCGCATAGCTATAGCACTCAGGTCCAGCCTGATCCAACAATGGAGAGTCCTTATACTGT
AGACTGTGAGGGACTGAAGCTTTCTGAATTTACCTCTTTACATTCTTTCAGCACTCCAAGGCCAACCTACAAGAGCCCTT
CACTCGGGGTCCGATTCCGACCCGAGTTAAGCTCGTGTAAATGGAATGTAATTCCCTTTTTATGCACCTTTCTCTCTACG
GGTATTCTGACCTTGAACTTGAGAATAGCATGCTATTCAACCGCTGTTGGGTGGGGATTAAATAAATGAAGGTGTGGTGG
TGTGTCTACAGATATGCCGTATTCTAACCTCGACTTAATTCCCTCATAATTACACCATCTTTACACATGATGAAACAATG
AATAAGGTCAAGCAACCTGTCGGTTCCAAGTGGAACAACATCTACTTTATTTTTCCCCCCCGATAGAAATAACACCATTC
TCCATTCACTGTAATTTACAAATTGATAAGAGCTAGGTAAATGAGTAAGCCGTTTGGATAAGGGGATTGTAAATATAAAT
GACAATCGTTTCCTAACCCTAAATAATACACAAGATCAGAGTTTTTTTAAATCGACCAACTGGTGGTGGAAAGGAGATA
TATGTGTGTATTAAGATGGTTCTTTCATTAATCCCCATTCTGAACCAATTCTCTCAATTACACTGAGTGTACAAAACATT
AGGAACACCTGCTTTTTCCATGACAGACTGACCAGCTGAATCCAGGTGCACCGGTTTGAGTGTGACAAGAACTGCAACGC
TGCTGGATTTTTCATGCTCAAATGTTTCCTGTGTGTATCAAGAATGGTCAAGCTAACTTGACACAACTGTGGGAAGCATT
GGAGTCAACATGGGCCAGCATCCCTGTGGAACACTTGACACCCTGTAGAGTCCATGCCCTGATGAATTGAAGCTGTTCTG
AGGGTAAAAAGAGGTGCAACTCAATATTAGGAAGGTGTTCCCACATTCTTTGCACACTCAGTGTCTGTCGAGTAAATTTG
AATTAAAGGTACACCATATTTAATGGGATGGCTTTAGATAAATGTACGGAAAACCCTTTTGAATGAAAACTCCACGTGAA
AATCTGTTGGCCAACAAGTGGGAGGGTTTTCAGCAGTTGAATTACATTTATTCAGTGCGTGCAAGGTAGCCACACCTGCA
AAGCTTGTTACACACCTGCATACGGTAAGTATGAATACCAACTACCGAGAAGTGCTCAAAAAATCAAAAAAATAATCGAT
TTTCTGAGTCTGAATCGGGCCCTTAGAGCTGAACCTGAACAAAACATGTCTCAGCTTGTTTTTCTCACTGGCCAATGTCA
TGTTTAACCACCCATGTGGTTCTAAGACATTGAAGGTTGCACATAAAGCTTTTACAAGAGACTTGAAAATTGAAAGACCA
CTGATGAGTGAAATGTGTAGTGTTGGGTAATGATTTGACTTTAGGAATGACCCCAAACATGGCAAAATGCATTGGTTTTC
AGAATTCCCCTAGAAACATAAAACACCACAGGTGCACATTAAATATAAATAATTTTATAATATACAAATTGTACAGTCAT
GAACAGTAGGGATGTGGACATTTCCATGAAAAGCGAATACAAAGAATACCTTTGTTTTTACAAAGACTGCAGTGCGTGAC
GGTAGCCGATGACCAAAGACACTCAACATTCAAAATGGCAGCGCAGGTGATGAAAACAGTTACATCGGAGATGAAACCTA
ACAATACAATTCCAGAGGCCACGAGCAAGCTAAAGTGACAATATAAAGAGATTGCCCTCCTACTGTATCATGTTTCTGTC
ATTTCTAATCTGTCCATTTCACATAATAACACACAGGAAATGAACCCTGGAACACACATTTTGCACAGCATTATCAAGAA
CAATTGCACTGCCACTGACTGCTGAGCTTTAGACAATTATGGTTACATAAAGACAGGAAATAGTAGATTACCTTTTTTCC
ATGAGTGGGTTGGAGGCGCTTGTTCTAGACAAGGCTGAGGTTAGTTAGTCACAGGATGAGGGATCGTCAAGGAAGAGTAA
GTAAACAACATTCAGCATCATTTAACTGATGAAGTGCCAGATCCTGCTTCACTTTCTCTCCATATCCTTGTTTTTCAGTA
TCAGGAAAAATTTTGTCTACCCACTCTCCCCAGGGACTACTTAGGGCAAAGCTGAGCAAATGCACAATCACAAATCATCT
CTTATCACGAGAGATGAGGGCATGGAGTTCACGACCCCCCAGGTAACACAAACGGATACAGGACCTGGTGTCATTTGACC
CAACTCTGTGGAGGTTACAGTTGTCCATGTTAATGGTGTCCTTCCAGAATGCTGTAGGTGTTAAAATACAATGATGTAAA
GAATGAGATCACCTGATAGTTCCTGGCTGTATTTTTCCACATTTTCCTACCATAACTTGATGTACATTGTACTTGAACAG
GCAGTATTGTCACTGTGTAAAATAATATACAAAGGAGCAAGGCTCACAGATATATAAAAAAAAAGACTCATATTGGTATG
AACAACAGAACTTATGAACACATAATAAACAATGTATGAATAAGCAAATATGACAGTCCTACATGATCTTATATCCGGGC
AAAAAAGCGATTTGTTTAAATCTAAAGAGAAAGAATGACTTGATAAGAAGCCAATGGGGAAATATGTCTCAGGTTCCAGA
TAATCTCTTCATTTATCACAGAGGGGTGTTTTAGATTTATATTTCTTACTCATATTCGTCCTAGCCACACAAATATTAAA
ACTGACAAGTCATTTCTATTCTCTCTCAAGCTGTGACCCACAAAGCTAAATAAAACATGTTGAATACAACAAGTGCTTAT
TAAAGCTCATAATCGCCTAAAGCGAAGTGTCGCTGGAAAGAAACAATACCACAGATAATATCACAAAAAAAAAGAAAAAT
GATCCTGACTTTATATCAAATCTGCTATTTGACATGTGGGCAAACTCCTTTCTACTTGAACAAGTAGCAGCTGTGGAAAT
TCCATCAAACATGGTTCAAACATAACCCCTGACCACTTGGCCATTAGTAATGACCTCACGCCCACAGCATGGACCATATG
GTGGAAAAAGTTGCCAATGACGACTCCATAGCTACAAATCCCCAATATGCCTAAGGAGGTAGCTAGCTCCAGTATGAGCG
TTTGAAAGCACACTTCAGCTAGCGTTAGCACGACAGAATACACTGAGCCTAACTTCAAATCAAAAACAGAAACTTACCCT
TTAAATCAACAACACCTGTTAAAGCCAAATTCCTTACCTCCCGTTCAAATCTAAAACAGAAAACACTGAACTTACCTTCA
AACACTGATTGTAGCAACCCTCTCAGGCTATAACACTGAAACCAACTTCAAACTTCTGACATTTGTACTTGACGATTTAG
GCCCAGTATCACAATAAGGCTATCCTGGTCACAGTTTGGAATTCTATTCTATACAATACATCTGCCATTGAGATAAACAT
TTCAGAACAGTCGGTTATTCAGAAAAATACCACATCTACAAAACGGCGACAAGCCTATATTCATCTGTAAACATACAGGT
TTCTACAGACTTTGCTTAGCAACTGCAGTACAAGAAAAATCCATCCCTATGAAAATCACAATCCAGATGAAGTGCTATTT
GTTATTTTTCGAGGTGAGGTTTTCCACCGTCAATAGCCACATAAGTGACCCCCATTATAAATGTATGACTTGACTGACAC
CCCACTGACAAAGTCCATTAGCCGGTTAATGATTTCTCCACACAGCATCGATGCAGCACCACTTTAAAATGAAAAGCAAC
ATGATACCTTGAAAGACTACATTATGATTTTGGTATGGTCTGAAATGACACCGTCAAGAGGGGCTTTAAAAGCTTGAGGT
GAAATACTAAAGCAAAGTCAATGGAAAATGGCAATTCGAACACCGCTATTCCAAATTGCTCAATATTTTTTTTTTACTTC
TTCTATGCTCCGTTTCTATCTTGAACACCCAGTCATGGCACAGCCCATAACCTTGCCCTCATACTTGCCAAGTACTTTTT
AATGACCATTGATTGGAAAAGCTATTAAAATCAAACGTGTGTAAACTCTCAAAGTTGACTCAACATTTCCATGTTTGGTT
CTCATCACACTTCACTTCCACTCAACCCACACACATTCAATGGAGATTTACTCCATTATCAAACCCTTGACTTGGCTTGG
ATATTGACACATTCAGAATGCAAGGCGAATGCGTCATACCTACTATAAGAATCAGAGTGATTATCAGCATCATCTGTAGT
ACTTTACATTATGTGCTAAAAGTAAATCAAGTTCCATTCACCTTGCATTGGATTGCCTGTCGCAACAGCCACTACATTTC
CCACAGTGCTCCTCATCTAAACCCATGTGACATACAGTTACTGTACCACTATGGTACTGTACAAATAAGGACAAGCGAGT
TTGCCTGGTTAAAGTCTGTGTTTCTTCAGACCCAAGCCCCATTGGTCCCATCAATTTGTCCATTATATCCAGTGACTCTC
GTACATTAGTATCCTCCGCATCATTCTCAGTTATGTACGTTCACTACGTGTAGGCATAGGATCAGTCTGAGGCTGTGAGA

GAGACTGTTGTGTGTCCATCTGTGGTAGAGATGTGTCCATTGTGGAGAGCTGTCTGCTGCAGAGGATAGAGTGATTACAT
GGTAGGAGGATGTTGCAGTAATTTAGGCTGTTCCAGGGGGTAATGGTGAGCCCCTATCTTAGGTTATGATGTTTCGTAGG
TACATGATTGATGGGTAGGAGAACCCTTAGGTGTGATGTCTGCTGTGGTCTGAAGAGGTTCACACATCTGACCTTTGACT
TTCTTCGGGGCATTGATTGGTCAATTAGAAAGGAAACCATCCTTTACTGCCTTTTGCACCTCCTGCACTAAATGTTCATC
CTGGTCAGGTGGGTCTTGTTGCACGTCAACATTCTCAGTTTCTCCATTCTCTTCCCCTCCATTTGTCTCTTCCTTAGTCT
CAACCCCATTTTCCTTCACTTCCATTTCAGCTCCTCCCCCTTTCTCCTCTTTCTGCCCTTGCTCTTTGGTCTCCACCTCC
ACCTCTGAACCCTTGGTAGTTTCGGTTTCATCATCAACTGCCACCCCATTCTCTTTCTCTTCCACGGTCTCCCTCTCTCC
ATCTCTTTCCTCCTCAGTCTCTGCCCCCGCTTCTTCTCCCTCCTGCACTCTCCCCTCTTTGTCGCTCTTGTCATCTGTTC
CTGCAGCTCCAACTCTCTCCTCCCCAGTTTCTACCCCGCTTTCTACCCCTTCCTCAGTTTCTGCCTCAACTTTTTTCTCT
TCCTCCACCTCACAGGGAGCCTCACCCTCTTTACACACAGTCTCTACCTCCGCCTCTTTCACTTTCTCCTTTTTGTTCCC
CTTGTTTTCCTTCACGTTCCTCTCCTCTTTGAGCACCTTTTCCCCATCGTCAAGAATCTTGGAATCTTCCAGATCTTTCT
CTGCTGCCGATTGCTTGTTTTTCTTCTTCTTCTTGTTTTTTGACGCATCTTTAACAGAGTTCTTGTTGGACTTGCTTTTG
AATATCAGTTTCTGAAAGACAGGATGTTTGTGTAAGAACGTTTCTCTAAACTGCTGTTCTATTGGCCATGTTAAGGATGG
ACAGACAGACCGTTGGAAATTGACATAATAGCAAACTTGGAAAGAACAAATTAAAATGATTCTTATTGTGGGGAATGAAA
TTGTAATTTCAAGGGAATTGATTGGTGCCTTCAGGGGTACAGACCAAACAAGAAAATATGGCTGGTAATATTTATGAGAT
TGCCTCCATTGACCCTCTTTTAGCTGAGACTTACAGTATACTGAGGTAAACAGCATGTTACCTGCACACAAACATTCACA
TTATCATTCTCTGCTCATCTCTGCCAGGGTTATTGCCCATCAGTTTGGTTGCCAATGGTGACCAGAGTTTAAATTCAGGT
CACAGACAATATCAGCATAGATTCATTCCACAAAGGACAAAGGTCAAATCTAGAGGTTATATCAGTACAATCTAGGATCA
AATGGAAAGTTATTTCTCAACATGCATTGCCGTGGTTACCACAGAATGGTTCATATCTCAGGAAGAAAGGGGTTCATTCA
CATTTGACCGGCAGTGTGCATATTTATCTATGTGAAAACAAAAAGAAGTGTGGCATGGTGAGGGGTGATGTTGGCCTTGG
AGCGCTTCTCTGGCTAGTGAACAAGCTGCGAGTCGACCCACATGTGCCAAGACAAACACGGGGAGGAATGAGAGGTCACA
CACCTTGGATTCCCCCCTCTTACGGGGAAGGACAGGTCTCTGAAGAAAAAGGACCTGCTTGGTGGCCAAACTTCCATCAC
TAGGGCAGAGGGAGAGATAGGGAGACGGAGAGATAACAGGCAGAGGGAGAGATGGGGAGACGGAGAGATAACAGGCNNNN
NNNNNNNNNNNNNNNNNNNNNNNNNNNNNNNNNNNNNNNNNNNNNNNNNNNNNNNNNNNNNNNNNNNNNNNNNNNNNNNN
NNNNNNNNNNNNNNNNNNNNNNNNNNNNNNNNNNNNNNNNNNNNNNNNNNNNNNNNNNNNNNNNNNNNNNNNNNNNNNNN
NNNNNNNNNNNNNNNNNNNNNNNNNNNNNNNNNNNNNNNNNNNNNNNNNNNNNNNNNNNNNNNNNNNNNNNNNNNNNNNN
NNNNNNNNNNNNNNNNNNNNNNNNNNNNNNNNNNNNNNNNNNNNNNNNNNNNNNNNNNNNNNNNNNNNNNNNNNNNNNNN
NNNNNNNNNNNNNNNNNNNNNNNNNNNNNNNNNNNNNNNNNNNNNNNNNNNNNNNNNNNNNNNNNNNNNNNNNNNNNNNN
NNNNNNNNNNNNNNNNNNNNNNNNNNNNNNNNGGAACAATGATGTATGTAAATGTAAATATGTTCTATGCTTAGGAGAGA
GCTCAGACACCAAGAGAAAGAGACCATGTTTACTGGCTTCCCGAAAACATGGCTGTCCTCATAAACTTCCCCCAAATAAT
CCCTGTCCATGTACATGTCACACTCTGTGTTCATTTATGGATAATCCATACAGTACAGATGACAGACAGACTAATCCACC
GAGGAGATTCTGTCATCAGTGGGCTTCCTGTCTCTCGTGCTTGCTCTCTCTCCTGTTCGTCTCTCTTCGTTCCATCTCAC
ATCAATAACGTTCCCTCTGCTTCTCTCCTTCATCTTTCCATCCGATATGCACGTATCGACTCCGTTTCTTACTTTAGCCT
GCTGTCTCTAACTATCTGTCTCCTTCACTTTACTTCTCTGCTTCTTCTTCTTCTTCTGTGAGCTTCATGTAGGCTTTCTC
TATCCTTTCCCTTTTCCCTTCTCTCTTTTGTATATCCCCTGATCTCCCTCTCCAGATCGCTCTCTCTGTCACTTCCTCTC
TCGAAATCTCTCATCCGTTAAAACATATGTACTCTTATACACACGGGGAAGAACCAGCTTTGAGTTAAGCTGGAGCCTTA
ACTTGGCATATGAAGAACAACGGAAATGTATGATAGGGTGACAACGGTATAGCGCTGGCTGCAAATCTTCCCCACAACAT
CAAATGGGCTTCCAGATCTCCTGTAGAGGCTTACTATCTAAACCACCCCCCATTTCCCATCCCTCTCTCCTTACCCTGCG
TTTCCAACAACCTATTTTCCAGGCTGTCATATCGCCGGACAGCTTCCTGCTGAATGAAAGCGGAAAAGCCAACCGGAGTC
CTAAGAAAAAGCCCAAGCCCATTAAAGTAGAACGGAGCGGATGAGTTTGAAAATTACAGGCCCCTTGTTGCTACAGTCCT
CTCATAGCTGTCAGTGTCTGCAGTATTGGAACAGAAACTTCTTATACCAGCAACAAGACTTTCTTTGGTACAGTGGTAGG
GAACTTGGTGTATTATGTTTAGTGGTTACCTGTGGTTGTTCACATCAGCTTTATTCATGTTTTCTGTTGAGTCATTTCTT
ACAACGGTATAATGAAGCACTTACTTATTTACTAAAACAGTTACGATGTCCAAACATCAGATTTACACCAAATATAACAT
GTTTCATGTGGCAGAAATATACTGTACATTACCACGTGCACCTTCACATTCATGTTACGTTGATTAAAGAAGCCTTGAGT
CCCAGTGGATGTTCACTTTAGATGCTAAGTAGTGTGTACGTAGGTCTATGCAGATCAGACTCCATGTAGTCTCCATTGGA
AGAAGTGCAATTTGCTCATCCTCAGACAGTATCCCAACGTGCATTAGCTTTAATATAGTTGTAAAAATGGAAGAGTCAAC
ATAATACAAGTGTAATGAACAAGCTAATCTGAAATGGGTCATCAGCTATCGTTGCTGTGCACAAAGAGACGAGTGTTTCA
GAGATGGAGAGAAATGACCGTTGCTGTGTTGTATCTAGGCCGGCTCTCTGATTAATTAGCCATGTTGCGCTGTTGTGTTG
TTGCATTTTGAGAGAGAGGGATACAAATAAAGAGTGATGAAAAAACAAGGGATGAAAATAGAGCGGTAATTAATCAGCTA
AGCTCTGGAACGGTCACAAACACGACCACCAGAGACAAGGTTACGCAAGGTAAACATTTAACTGCAACCGTGTTGAACTC
TAAGCATATACTTGGAGTCTTCATAAGCATGACATAACACATAACTCTGACTAATAGCGGTAATCAGTAGTTGAAACAAT
AACAAAGCGTTCTCCCACCACTGTTTCAGTCAAAAACGGGAGCATGGGGTTGGAGAAATGTTACACTCAAATTCTATGGA
TGCAAGGACTGACCATCCATGATATTAAAATGATCGTTTTAACCATGTTTTCAGGCTATACAGTGTTTGTTTGCATTTAC
TTTGTTTACAAACATTGGAGCAAAACAAGCTTATATTTTGGGTTCTGATGTGGTACGACAGTTGAACTAAGCTCACAAGA
CATTTATTTATAAGTTATGTTCTTCAGGAATCAATGCAAGGGGTACATATTGGATTGGACGTGTTCTGGACTTGTGATAA
TGATGAGCGCTTGTGACTGTGTAAAGTGGGGTTTCAGGTGGGAAACAAGTATTATTGAAGACTTGGAAGACATGTTGTGA
CTGCTGATAGATGACTGAGCTGTTGACCGTTAGCGAATGTGTGCGTGCATAGTCCTCATATAAGATGAATGAGTCGGCAG
GGGCTGAGCAGAAGAACCGTTACATTTCTCTGAAGGGATTTTCCAGTTACACACATTTGTGGGTTTAAACTGTCTCGCAC
CACACGCTGTTACGAATGAACCCGCCAATTGGGGCCAGTTAGTATTTTCGTTGAACAACCTGTTGAGTTCTTCTGACTGA
CTTCACAATCCATTCCACAATCCACAATCCATGAGCTGTTAATTGACCAGTGATATGGGCTGACCATCGCTTGGTCCAAA
TGAGGTGGGTGCTCTTCGAAATCAAAGAGAAGGCAGTTTAATCTCCAGAGGGTTAATCAGGTTGACCAATCAATACCAGA
GTTAGGTAACCAGGAGATATGGTGGTCATTCAGGCCCTAACTCAATACTAGTGAGAGTGTGTATTGGAAGGTCAAAGTAG
TTACCTGTTGGTCTTGATGATGGGTGTAGGCAGGACACACGTTAGTCTCCAGCCGTACATGGCAAGAGACTGGAGCAGGG
GAACATAGTCTGTCTTCACCTGCAAGCCCTAAAGAGAGAATACAAACACATGAATATTACATACTCCAGTTATACACAAA
CACTATACACACGCATGCATACACGTACGCATAAAGACAAGTACGAGGAGGGCTGGCACAATTATCGTATAATCCTATAA
CTGACAATAACTGTGAACTTCAGAAATAATTATCATAATAGTCATAATGCATTTTCATTTTAAGAGAAGGGGGGATTCAA
CTTCATATTCAAGTAGATATAAATGACTCAATAGCAGAAGTGGGTAAAGTTGGTAGTCATTTTACGAGCAGAATGGCACC
GTTGGGAGAAGCTTCATTTCCAAAAGTTCCAAGAGGTTTTTGAGACAGGGATGTTACCAAAGCTGGGTTGTACTCATTAG
GTTTGTCGTGGCTATTTTTCAGAGATGCATTACGATGCTCAAAACATTTTAACAAAAATGACAATTATGACAATAACTGT

GCCCATTTGCATGACAAGTAATCTTTACCCAAAATTCCAAAGTATGAAAACACACACGCAGGCAGACAGGCACACACACG
CATAAACGAATGCATGCATAAACACATAAACCATTAAAACCACATCAAAAGAGTCACGATTACCCAAGCTCTTGACAACA
GATGATGAACTATGTATGATGCATACCAAAATACATGCATTTCTGTTAGATTGTTCGCTGGTATGACAGTGTAGTGCACA
TGGGATTTGGGTTAAAGACACATGCTCGTCGCACGCACACATGCGCTAACGCCTCTGCTCACACAGGAAGTGTGTGTGGT
GTTCCATCTCCTCTGCATCTGCCCTCACTGACAAACCACAGTATGTGTCAGATATGAAAAGACAGAATAACAACAAAAAT
ATCCAGAAAAACGCATGTTAGAAATGTTATAAATTGATTTGCATTTTAATGAGGGAAATAAGTATTTGACCCCCTCTCAA
TCAGAAAGATTTCTGGATCCCAGGTGTCTTTATACAGGTAACGAGCTGAGATTAGGAGCACACTCTTAAAGGGAGTGCTC
CTAACCGCAGCTTGTTACCTGTATAAAAGACACCTGTCCACAGAAGCAATCAATCAATCAATCAGATTCCAAACTCTCCA
CCATGGCCAAGACCAAAGAGCTCTCCAAGGATGTCAGGGACAAGATTGTAGACCTACACAAGGCTGGAATGGGCTACAAG
ACCATCGCCAAGCAGCTTGGTGAGAAGGTGACAACATTTGGTGTGATTATTTGCAAATGGAAGACACACAAAAGAACTGT
CAATATCCCTCGGCCTGGGGCTCCATGCAAGATCTCACCTCGTGGAGTTGCAATGATCATGAGAACGGTGAGGAATCAGC
CCAGAACTACATGGGAGGATCTTGTCAATGATCTCAAGGCAGCCGGGACCATAGTCACCAAGAAAAATATTGGTAACACA
CTACGCCGTGAAGGACTGAAATCCTGCAGCGCCCGCAAGGTCCCCCTGCTCAAGAATACATATACATGCCCATCTGAAGT
TTGCCAATGAACATCTGAATGATTCAGAGGACAACTGGTGAAAGTGTTGTGGTCAGATGAGACCAAAATGGAGCTCTTTG
ACATCATCTCAACTCGCCGTGTTTGGAGGAGGAGGAATGCTGCCTATGACCCCAAGAACACCATCTCCACCGTCAAACAT
GGAGGTGGAAACATTATGCTTTGGGGGTGTTTTTCTGCTAAGGGGACAGGACAACTTCACCGCATCAAAGGGACGATGGA
CGGGGCCATGTACTGTCAAATCTTGAGTGAGAACCTCCTTCCCTCAGCCAGGGCATTGAAAATGGGTCGTGGATGGGTAT
TCCAGCATGACAATGACCCAAAACACACGGCCAAGGCAACAAAGGAGTGGCTCAAGAAGAAGCACATTAAGGTCCTGGAG
TGGCCTAGCCAGTCTCTAGACCTTTATCCCATAGAAAATCTGTGGAGGGAGCTGAAGGTTCGATTTGCCAAACGTCAGCC
TCGAAACCTTAATGACTTGGAGAAGATCTGCAAAGAGGAGTGGGACAAAATCCCTCCTGAGATGTGTGCAAACCTGGTGG
CCAACTACAAGAAACGTCTGCCCTCTGTGATTGCCAACAAGGGTTTTGCCACCAAGTACTAAGTCATGTTTTGCAGAGGG
GTCAAAGAATTATTTCCCTCATTAAAATGCAAATCATTTTATAAAATTTTTGACATGCATTTTTCTGGATTTTTTTGTTC
AAATAAACCTACCATTAAAATTATAGACTGATAATTTCTTTGTAAGTGGGCAAACGTACAAAATCAGCAGGGGATCAAAT
ACTTTTCCCCCCCACTGTATGTATATATATATATATATATACATATGTCATATGCGTGCACACATTGTAGTGCTATATGG
GCATCTACAGTACAGACTGAGGGCAGTGGGTCCCAAACCAACAGATAGACAAAACAACTGTAACCACAAACAAGTCTTAA
GCCCAAGCCAGTGTGAATTGGCCAGGACCCATTGTTCGAGAAACACTGCAGTAGGGTAGAGAGGGCTCTGCTGAAACTGT
TATTTACATTGGCATTTGCAGTCCCAGCATCCCTCCGAAAATCCAGAGCTGTGAGAGCTGTGTTTTAGGGTTTGTGTTGG
AACCTATGCCAGAAATGGACACATTTTCAAAGCCATAAAAGCAAAGTAAATTGGGAAATAAGTTTTGTTTAATGGCCCTC
TTCATTCCTGAGCGTAACTGAGACTCGAAAGAGTGAATCATGTTCTGGAAGATTCCCTCCCCCTGATCTCACCATTGTCA
CCTTGCTGCATCAGCACATGAGAAACGGAACCCGGCGCCTTAGCAGTAACTCTGTGTTCTGATTTGTGTGTTAAATATAA
TCTATATATAAAAACAGGGTGGGGTGCAAGCTAGGGTGGTAAAGGGCTACATTATCAAGAGTGGAGAACGGTGAGGTAGG
GGCTAAGATTTGAAAACAGAACAGCGTTATAGAGTAAAACTAGCAGGGTGGGGTTGAGTTCAACTCTTGGGTTCAAAGTT
CACTAAGAGTTGGATGAAAGAAAACAGAGCTCTATTGTTTTGAGGTAATTCTCCACCGTTTCTATTTGATAACACATGAC
AGACATAGCTCCCTTTTTGCAATAAGTCTAATTCTACCTTAATGTTTTTACAGGCCGTAGTCTGGTCTATCAGCCCAGGG
AAATGATCTAGGCTACTCCTCTAGCCTCCTAGCTGTCACTTTGTCTTTGGACCGTGCTGCACTTCTTCCACCACAATCTC
ATTATATTTTTATGGACAGGGTATCCTGAAGTGAGGCTTAAAAGTCCCTAATCTGCATGTCAATCATTGAGAGATTGAGA
AATTTTTGAAAACCAAAAGCTGTATTGAAGACTAATCACACCATATACTGTATAATAATAATAATAATAATAATAATAAT
AATAATAATCAACATAGTCTCAATAGAATATATCAACATTTTGACATTTAATGTAGTTAGCCTAGATGTCAACCAAGCTG
ACGTATTAGTCACATATTTCGGCGAGCAGAATTCTCTGGGAACTTCCTCATCCACAAGCCATGAGCATAATTTCCACATC
CCTCATCAAACTAGTCTGACCATAGGAGTGGACTCATACAGGGAAAGTGAAAATTGGTCTCTGTATCTAGGTCAGCTCTT
GTCTCGCCTCGGCTTCAGAGGAAAAATAAATCATTGGCAAAGGATACACTTTAATGATCCCTTCAGTGGTCTCCCTGCCA
CACCGGCTGCTACATGACTATTGACATGCCTTGGTTTACTTTTGACCAGTATGTGTTAAAATACATATTTCAAGCAATAG
CCAACTAACAAGCAATATCTATGCTTAGATAATCTTTAAAAAGTTATGCAAGTTATTACTAATTTCCCCAAGACCCATTG
ATGCTGTTGAATGAAAACAGAAACTTTTCAGGAAATGGAGAAAAAAAAACGGCCATATTTTCTCATTAATGAACATACAA
TTATGAAACCTCAGAAGCTATTTTGAATACATGTTATTGGTCCTAGAGTCATGAAATGTTCAGAATTAACTGAGGGGAGT
TACTGCTTTCAATACATGTAAAATAAAAAAATGCAGAATTGTAGGTATGAGGTATTCATCTGACAGCGATGTATAATTTA
GTCTATTTATGCTATTCAACATTTTGCAACACCCACTGCATGCTAATTGGCTGCTCAAATCGGGCAGCACCAATAGGGTT
GATGGCATATTTCAGCTCATTACATCAATTAAGTCAAGCTTAGCACCATGACAGACAAATGTTGGGAGAACTAGAGTCTA
GACTACAAGTTAACATGAGAGGGCCTCTGACCGATGACACCTGTTAAAATGGCCAGCTTCAGTCAGCTGATTCTAGCCAT
CCTTCATGGTTTGCTCTAGTCGGGCAGCTTAGTCTCCTATTCAGAGGCCCCCCACTTTTGTTAGTGTCTTATTGGCCAAG
GTCATGCCAAATATTTATCACCACATCTTGTTCAGCCTATCAGCAGCCTTAATTTCCGGGACTTCTGTTTCAGTTGGTCT
ATCATCAGAAACGTGCTTTCCCCATCCTGATGAGCACTTCCACAGTTTCCCTCCTGCTGAAGTGTTCCTTCTGTGGACTG
AATCCCTGTAGGTCGGATGTTTAATTGTGCGTGCATCTAAACGTAAGTGCCTTGAGATCTGTGTCATGTTATGTGGAGCT
AAGTTTAGTGGTTTATTGTGTACAATTACCATGTGTTGATCTTGTTAGCTAGCTCGCGCTCCCACTAGCACTACCGTTAG
CATTTTGCATTGGAATGCGTGAGCATTTTATATATATATATATATATATATATATATATATATATATAAATACACACATAAC
TCATGCTCATTGTATTTATATTATGTATACATTTCAACATTACTGTAATATTTATTTGAGCACATTTGCCTGAGTTTCAT
GTGTACAAGTCCAATAGGTTGTTTAGTCTATTGCCATGGGACTAGGCCTTATGCCTGCTGTAGTGCATAGCCCTGGCTGT
TATATACTTACCTGTGCTAGCTTATACAGCCATGTTGTTCATATTATAGTAGTGCTATAGCCTAAGTTTTCTTTCTCCGC
TTCCCTGTAAGAACCATAGTTAATATCCGTTCTCACTGGACATGATTCTCAGTTATCTGAACCATGAGTGGTCAGATGTG
TGTGTGATGTGTGTGTAAATGTGGACATCTTCTTCCATTAAACTCCTCCTAACCTGGACATCCGCCTCATCCTTCTGACC
GGACATTCTGTAGTGGTTACTACCGGCCTTAAGCCAGCACCTAAGATTTCTTATTACATTCATGGTCCTTCAATTCTGTA
CAACTCTACCCCTATTTTTCAACACCCACCCACACACCCACACACCCACGTACACAGCCACACAGACCCACATCTAAAGT
GTCCTCTGTCTGAAATTCACTCTCCAAAGATCCTTTATTGGCATCTTTACGCTATGTTGTTGGTCTTTAATCCTATTTTA
ACACAATTTGGTTTCTGGTGACTGAAGTAGTGCTCTTCAAAGTTCAGGCATTTATACAATTGCTGGGAACGAATTACAAC
AATGTCAATGCTGTTAACTGGTGCCTCAAGCACACAGTATTGTTACTGGTAGAGTTCTATGTTTAATGCCTCTCCGGATA
ATGGCCATGCACTTTTGGGTTAAATGTAATATAGCCTGTGAAAAACTGAGGTAACACTGGGAAAACATGACGCCCTTGTT
GTTCAATGTAATGGAGGAAAGGCATTTAGTATGACTTTTTTTAAGGCATCTTAATGAGCATTTAGAAAACCATTATTGAA
TGTTAAACAGTTAAGTCCTATATATGTTACACTCATAGCCATGTTGTGAATAGATGCAGTGTAGAGGTCAATGGAACTCG
ACCAAAACAATGGAAATTGCCATGGAAACGTGTGGGGGAAAGATGCCGTTGTGGAAAGATTCCGAATGTCCTCATTGCCC

CCCTGCAAAATTAGCATACATTTAGGCTATTGTTTATATGTGTATTTCACACTATGTTGACGTAAAAATTGGAGTATAAT
GGTTGTATGGATGTCAACCCCAATACATGTTCATGTCATCGTCAACAATGAACTGCGTTGCAGTTAAAAATGACTTTGAC
TACCACCACCGATTTCTGTGTGGTACTTTAGCTATTATACCAGCTTATACGAACAGGAGTTAGCATTAAGCAGTCACTTC
TTCTAAACCTGAAAAGGAACAACTTCTAAATGTTATGCAGCAAATACAGCCAACTATATCCAAATCGGACATTAGTAACC
ACATTGTGGTCCTGTTACAATACATCAATCATGACGGATTTGACGAATATCCACTTTGTTAGATTTATTGAACGTGCACC
AATCCGGCGCCATTCTATCCCTGTGGTCTGAGTTCTATTGACCTCTACCACATCTATGCTATATGAAGTTGCCAAGAATG
TGTTTATGACACATTTTACAACGGATGGGTCTAATCCTGAATGCTGATTGGTTAAAACCTCATTCCAGCCGGTGTCTATT
CCACAAGTTACCCCCGGCTAAATCTATGACGTTAAAATGCCTATTTACTCTGTTTCATCTGACTGGGAAATCCACTGTCT
CATCAGCCCAGCCAGGCAATTTATAAACTTGATCTCCACTATAAAAAGCATCTAGACATTACTTAACATTTAGTTAACAG
CGGAGATTTGTATAAACCTTCCCATTTGTCTCTCCAACATTTGTAACATTGTTTCAATATTCAAGTTCGATCTCCAGCTG
TCGCATAGTAATGAACTTGTTGGGAGTCAGGATGAGACAGACAGGCAGCTTTTCTCAGCCAGTCGAAGTCATGAAAAGGG
CCACATTTTTATGGATATATACAAAGAAATATCAATAGAAAACAGGTACAAATAAATAAAATACAGCTAGTTTGCAGTCT
TTCCAGCTTCAGTTTGAAGTGATTATGTTAGCTGTCATGTTGGCTAGCCCCTCTGAACAACAGTGTCCTGATCATGCCTC
ATTAGCTCATTGTTATAGATGTGGCTTGCGAAAGTATTGAACCCCCCCTTGGCATTTTTCCTATTTTTTTGCATTACAAC
CTGGAATTAAAATGGATTTTTGGGGTGTTTGTATTATTTGATTTACACAACATGCCTACCACTTTGAAGATGCAAAATAT
TTGTTTTGTGTGAAACAAACAAAAAAATAAGACAAAAAAACTGAACTTGAGTGTGCATAACTATTCACCCCCCCCAAAGTC
AAAACTTTGTAGAGCCACCTTTTGCAGCAATTAGAGCATCAAGTCTCTTGGGGTATGTCTCTATAAAGCTTGGCACATCT
AGCCACTGGGATTATTGCCCATTCTTCAAGGCAAAACTGCTCCAGCTCCTTCAAGTTTAATGGATTCCGCTGGTGTACAG
CAATCTTTAAGTCATACCACAGATTCTCAATTGGATTGAGGTCTGGGCATTGACTAGGCCATTCCAAGACATTTAAATGT
TTCCCCTTAAACCACGCGAGTGTTGCTTTAGCAGTATGCTTAGGGTCATTGTCCTGCTGGAAGGTGAACCTCTGTCCCAG
TCTCAAATCTCTGGGAGACTGAAACATTTTCCCCTCAAGAATGTCCCTGTATTTAGGGCCATACATCATTACTTTAATTC
TGACCAGTTTCCCAGTCTCTGACAATGAAAAACATCCCCACAATGTGCTACCACCACCATGCTTCACTGTGGGGATGTTG
TTCTCAGGGTGATGAGAGGTGTTGGGTTTGTCCCAGACATAGCGTTTTCCTTGATGGCCAAAAATCAAAATGTTTGTCTC
ATCTGACCAGAGTACCTACTTCAATATCTTTGGGGAGTCTCCCACATGCCTTTTGGCAAACGCCAAACGTGTTTGCTAAT
TTCTTCTTTAAGGAATGGCTTTTTTTCTGGCCACTTGTCCGAAAAGCCCAGCTCTGTGGAATGTACGGCTTAAAGTGGTC
CTATGGACAGATACTCCACTCTCCACTGTGGAGCTTTGCAGCTCGTTTAGGGTTATCTTTGGTCTCTTTGTTGCCTCTCT
GATTAATGCCCTCTTTGCCTGGTCTGTGAGTTTTGGTGGGCGGCCCATTCTTGGCAAGTTTGTTATGGTGCCATATTCTT
AAAACATTTTAATAATGGATTTAATGGTGCTCCGTGAGATGTTCAAAGTTTTGGATATTTTTTATAACCCAACCCTGATC
TGTACTCAGAAGAGGTGTGTATATATATATATATACTGAGATCATGTGACAGATCATGTGACACTTAAATTGCACAAAGTTG
GACTTTATTTAACTAATTATGTCACTTATGAAGGTAATTGGTTGCACCAGATCTTATTTAGGGGCTTTATAGCAAAGGGG
TGAATACATATGAACGCACCACTTTTCCGTTTTTTTTTATTTATTATATATATATATATTTTACTATTTTGTGTATGTCCATT
ACATGAAATCCAAATAAAAATCCATTTAAATTACAGGTTGTAATGCAACAAAATAGGAAAAACGCCAAGGGGTTGAATAC
TTTTGCAAGGCATTATACACAAATAAATGTAACTAGAAAACAGCTTAAACAAATGCAAATGCAGCTACTTTGTTGTTGTT
TTGACTGCACTGTTTGACGTGACTCTAAGTTAGCTGTAGTTGGCTAGCTAGCAAGCAAGCGATAAGAACGTTGCCAGAAC
GAACGACTGGGTCACGTCCATAGATACAAAACAAAAAGACTGAACAACTGGGTCGCATCTCTGGCAACCGAACCGATAGA
ACGAATGACCAGCCTGCTTGGGTAGCAACCCTAGATTTGTGTCGGGACTATACCTTGTAGAAGGATTAAATAGTATTTAA
AAAATTCAGCGAAATAAAGTTTTTAATACAAATATGTAAATCATTATTTCAATGTGTTGGTAACCCGTTGTATAAAAGTG
ATAATGCCCTCGAAGTCAGTGTTTGGAGGATATATTGTGGGCTCCCGAGTGGCGCAGTGGTCAAATGCACTGCATCTCAG
TGCAAGAGGCGTTACTACAGTCCCTGGTTTGAATCCAGGCTGAATCACATCCGGCTGTGATTGGGAGTCCCATAGGGCGG
TGCACAATTGGCCCAGTGTCATCTGGGTTTGGCTGGGGTAGGCCGTCATTGTAGAATAAGAATTTGTTCTTAACTGACTT
GCCTAGTTAAATAAAAGGTTAAATATATATATTTTTAAACAACACCAATGCCTAAATGTAAATATATCCTCCAAATATTGGC
TTTTCGGGAATTATCACTTAAGTATAACACTTCATAAAATCTCAAGTAATGTACATTTTAGTCATTTAGCAGACTCTCTT
ATCCAGAGCGACTTACAGTAGTGAATGCATACATTTCTTTCATGCATTTTTCTTTTTTTTTTGTACTGGCCCCCCGTGGG
CATTGAACCCACAACCCTGGCATTGCACACACCATGCTCTACCAACTGAGCCACAGTATAACCCAAATGTTGACCACATT
GCACCCACATGCATTCCAAATCCCCAAAGGACACAGTTAGGCATTAAATATTAGCAGTAGTGCTATCATAAAGTAATATTC
ACACACATCCACTTCCTGTTGTTTTCAGTTGTCATTCATGTTATTTGTTAGATAATTTATCTAGAAACTGAGAATGTTAG
AAATACACTAACATGCCGGTATACTAAGTAGTTCATTCCCTTCCATCAATAAGAACTAATTACAAGGTTGCAAAATCTAT
AAAGAATATTTCTCAGAGGTCTCAAGAACAGGAACTTTTTGTTGCAGCTTTAAGAGCTCAATGATAAGGGGAGGCATTGC
ATTATATTCAAGGCTGAACTTTCATATTGATTTTCCTTTTGATAGGAGAGACGGGAGCACAGTCCTAATTTGAATTTCAT
TGTGTAGGAAATTATGTCTAGTTATTCATTTTGCGGGCTCCATGAAAATCAACTGTATGTCCTATTCAGTTCATTTAGTC
ATTTATTGTAGAAATAGGAAAAAGTCTTCCAGTCAGATGCAAATTTCTTTCATTGTAGCCGAGCTTGGTCAGTCGGCCTT
CATCAGAGCATAGTCATTTATTGAAGAAAATGGGAAGTATGTTTCTGATTGGCTAAGGTAGAATGTCCATCCATAGACCT
ACGCAGAATTCTGAGCATCTGTACAGTTATTATTTAGCCTTACTTTTCTTGATTAAATGCTGTAACGTATACATTCTTAT
ATGGTCAATACATATTGACTAATATGAATTTAAAGTACCAAATAAATTGCTGTCGAAATGACCCCATCTCCCACAAAAAT
GTGAAATTAGTGACAATTGAGGAAGGTCTGACTTTCTAGATAACAGACCACAAACAGTTTGGCCACAAACAGCTCTACAT
ATCAACACTGTACTAATCATACCACATTCTACCAACGTGATTGTGAATACTGCGCAAAAAATATACTATAATAACAGATTT
GTTTTAAAAGTTTGCTATCAATTAAAACAGAAGTTTGACATGTTCTATGCCATACGAAAACAAAATCTCTGAGAAAAGCT
GTACTTCCTGCTTCAACCCAACAATATTGATTGAATTCTCAGTGAACCAGCCTGTTTTCTTGTTTTCGTGGCTCATAAAT
CCAACCACCTGTCAGTCTTTCAGCATGACGGGAACGAGAGCGCTGAAGAAAAATATCCCAATCAATATTCAGCATAATTT
TTCAGTGTAATTATTCTGGCACCGAAACGACAATGAGCGGTGAGGATAATTTGCATATACATTTTAAGCAGTTATCATAA
TGAATGTGCCAGCAATGAAGTCTGATCATCCTTTGTAAAATAGTGTTATCAAGCTAACTTCAGGAGAGATTAGTCGAGCA
AAATTGCTTTTGATAAAGTCCTGGCCAGCACATTTTGATTGGATCCATTGGAACTATAATGTGGAACATCTAAAGTTGTT
ACACATTGTTGCTTTGTCCTTCAATGAACTTCCAAAGGTGCTTAAAAAAGTATATAAAATCCATGTTATTTCTCAGTGGT
TGATAAAATAATTGCTAATCTTTAATAAAGACATTATTGCAGTATCTTTACCACAGTATTCACATAAAGATACCTACATA
CTCCATGATTTTCAAAATTGCTCAGTGTATAACCTTCACAAGACACAAGCGCCTAGGTTTTTGTGTAGTGTGGGACACTG
ACGAACATTACATTTTAAGGCGGCGAATATTGTGTAGTCTGAATTCTTCAGTCATTTTAAAGGGTTAAGAGCATTTTGAT
GTCATGTGTTAAATTCTGTCTTTGTGAGTCGACTTCCTTTCTGAGCTGGACGATGCTCCAGTCACATTTAGGGGACATAA
GTCTATTTTCACACTTCGACAGCCTCAAACTCTAAACTCTGACAGTGATACACACACACACACACACACACATAGAAAAA
AAAGCCTCAAACCCTTCAGAAGTAGACAGAAATCCATTTCCAACTTGGATTTGCGCAAACTGGGGATTTAATGGCCGGCC

AGATAAGGGCTTTTTTGCTGCAGATAAGACAAGCCACTGTGACACGTGCCATAGCCACCATGGTTAGAGGGAATATTTTC
ATATGATGACTCTCACAGTAAAATGTAAGGACAAACAGCTCCAACCTTGGAAAACACTCAAATACTCAGAGCCATGTAAA
AAGCCCACTCACATCAATGACAGTCCACTGCTCCACAACGATGGCGTCGTACTTGGTGGTCACGCTCTGGTCGCCGCCCT
CCGTCCCCTCCTGGAAGATGAAGATGCTTTCCACTGACTTAGGCTGCAAATCTGTAGAAAATGGAGAGGGAGGCCATGAA
GGAGTTAGTATTTACAGAATGGAGGGTGTCTGTCTGTGTCTGTCTCCAAGGCCTCTAGGCTGTGCAAAGAGGAGAGGGAA
GTTAATGACATTATGCAGCCGACCAGCAACCCACACACACTGGGCCATTTATTACCAGGCAGGGCTGCAGCATGGACCAT
TTGTATAAGCAAGGGCTATGGATTATGGATTATGTAGCTATGACAGAACAAGAGAGAGGGAAGAGAGGGGGAGAAGAATG
TTTCCTTCAGGGAAGAAGCTTGGAGAGATTATAGATGAACAGACAGGTATTATGGACAGATTGTTGAAGCATAACACGAT
TCATCTGAAAACAATGATATGCTTTACATATTGTAGATATTTACATGGAAGGACTATTAGACCAAGACAGGGCCATGGTA
CCATAAAATTGCTTTTCTGTAGACTCTATTTCATTTCAACCCCATGGATATGGGTAACATTCCCAGTTTTGGCACTATTG
GTATAGAAACATTGGTGAGCTTCTTCTCCATCCCGCATTTCTTTTGATTCCATTGAGCAGTGTGCTAGAACCTCTTGCCC
TTCAGACCAGGGCTCTCAGTTGGCCTGAGTGCTTTCTCATTACTCCTGTACCATTTACACCCTAATAGAGTTTGTGCGGC
CCAGGCTCCACCGTGAGACACTCCACATGTCATTACTCTGCCNNNNNNNNNNNNNNNNNNNNNNNNNNNNNNNNNNNNNN
NNNNNNNNNNNNNNNNNNNNNNNNNNNNNNNNNNNNNNNNNNNNNNNNNNNNNNNNNNNNNNNNNNNNNNNNNNNNNNNN
NNNNNNNNNNNNNNNNNNNNNNNNNNNNNNNNNNNNNNNNNNNNNNNNNATCTATCAAACATCTATTGGCTCCTTTCAATAC
AGACATGGTCGTTGTTCATTCCCCCCCTCTCCCGCCCTCCCTCCATCCTTCCTCCACCCTACCCCTCCTTTACATCCCCC
CCATCCATTGTTCATCAGACGTCTCCCTGCTTGGCGTGTAATTGGAAAGCCCTCTTGGAGATTGTGTTGGCCTGCCGATG
CACTGGGATCACTCAGGTGAGATGCACACAGGGATTGAGTCCCTTTGCTTTAGGAGGAACACGCTGCCTTCTGGCATGAT
GCTGCCAGACACTGAGGCACCGTGGGGTTGAGAGGTTTGGGGGACATGTAGGGCCAACACAAATAACAGCATAACATTTT
GAGTTGAGTCTTAAATGTAGTGAGTAAAACAGAAGCTTGGAGGGAAACACGTGTTCATTCGCAATGGGACAATGTAATCT
CATCGACTAGCCTATCCTCTCCAGGCACCTGAGGTTGAACATACTGAGTGAGATGTGCGCATACTATCTCTGACACATAG
AGAGTGAACTGAAAGACAAAGAGTGACAGACGTACTGATAGATAGTGACAATGGCGGCGTGAGTCGTGACACCGACTGAC
ACGGAGCACCAACCTGACTAGGTGACAACCAGTGACAGGGGTGTGGACTGAAGACAGGCACTAAGTGAGAAAGACTGATG
CTAGGTTGACATAGACTTTGAAGGAGAGACACAGAGGAGTAAAAAACAAGGAGGGGAATCTAAAGCCTTATTGACCCGCA
GAAGCAACCGAATGACCCTGCCCCAAGTCCTGTCTGTTACTGACATGACTTAAACATGTGTGCTTAGCACTGCCGGGGAG
AACAGGAGGGCTGTGACAGCTTATCTATATTTAAAAACGGGACAGGCAGGAATCCAGAGAGAGGAGAGATATAGAAAGAG
TGGGGTTACTAATGTGTGTGGATTGGCCAGGGTTTCCTCCTATGAGCCAAAGGTTTGGAGGCTGGGAGCCACGTACCTGT
GGTTACCGGGCAGACTTCTGGTTGTAGCTTGGGTTTTGAGGAATATGACATGAATAATGTACCCTGCCTTACACAAAGGC
ATATACTTCCAAAATCACACTCACAGTCAGTGGTGTAAAGTACTTAAGTAAAAATACTTGAAAGTTCTACTTAAGTCGTT
TTTTGGGGGGGTATCTGAACTCTATTTTTGACAACTTTTACTCCACTACATTCTATTTTACTCCACTACATTCTCCATCA
ATTTTTCCAGACATTCAAAAGCACTCGTTACATTTTGAATTCTTAGCAAGACTCCAATTCACACACTTATCAAGAGAACA
TCCCTGGTCATCCCTACAGCCTCTGTTCTGTCGGACTCACTATACACAAATGCTTTGTTTGTAAATTATGTCTGAGTGTT
GGAGTGTGCTGCTGCCTATTCGTAAAAAAACAAGAAAATCGTGCCGAATGGTTTGCTTTTGTCACGGGTGTCATAGGGTA
GACACCAAGACGCAGCGGGAAAATATATACTCATCTTTTTATTTGGTGAAGAAGGGAAACACATATAACGTATACAAAAA
CACAAACAAACTGGACAGTCTTGTCAGGCAAACAGCTAAACAAGAACAATCTCCCACAAAACTAACAGGTGAAAACAGGC
GACCTAAGTATGACTCTCAATCAGCAACAACGATGTACAGCTGTTCCTGATTGAGAGTCACACCAGGCCAACACAGAAAT
ACAACACTAGACCAGCCCCCTAGAAATACAATACAAGAACATACCCAACAACCCGGAACACATAAATCAAACACCCCATC
TTACATAAATACATATCTAATAAACCCTGAACCACTTAAATCAAATACCCTTTCTACATAGATACACCACTAAACCCAGA
AACACTTTACACAAAATAACCCTCTATATAAACACATAACCAAAAACATGTACAACAAATACCCTCTGCCACGTCCTGAC
CAAACTACAATAACAAATAGGCCCTTTACTGGTCAGGACGTGACAGCTTTATATAAGGAACTTGAAATGATTTACACTTT
TACTATTGATACTTAAGTATATTTTAGCAATTACATTTAGTTTTGTTACTTAAGTATATTTAAAACCAAATACTATTAGA
CTTTTTCTCAAGTAGTATTTTACTGGGTGACTTTCACTTTTACTTGAGTCATTTTCATTTAAGGTACCTTTTACTTTTAC
TCAAGTATGCCAATTGGGTACTTTTTCCACCACTGCTCACAGTCACGCACATGCACAAACACGCACACAAACACACTGGC
CTTCAAGCCCTATTGCACAACCAATCTTTGGCCATAAAAATTGCCAGCACAACTTTCCTCCAGATTCTTTTCACTCTGAA
GTAATACCTTATTGGAATTTCATATTCTGGGGAACTCTCTCTCTCTCCACCGGCTTTGCCTGAATATTGAAAGCCAAGCA
CTATGCTAGCTGTCATGCATATGAATAGCTCAAATGTTTCACAGTGCCGGCTACAAATCCTCATTGTCGCGGCGGAGATA
GCGTAAGCTAAGTGTAACAAAGAAAAACTCCCCATCAACACCATTAGTGTCAATTCACCAGTAAACAAACTGCTCCCCCT
CAGAACTCCAGCCGTGGCGTAGCCGTGTAGGGCTGCAGGGGTGCTGTGACACGCTGGGCTGGGTATAAATCTTTAATGCC
CCTCTCATGACGCCGCAGGGTGGCAGGGCCGTTAACGTTACACAGAAGCAGCCTGCCAGTGCCACGGTCTGCGAATTCAA
TAACAAACTAATATCCGACCCTCCGTGCCGGCGAGGTTTCTTTCTCTCTGAACCCACTCCCACCAGTCTCCCGCCTCCAT
CCCACCTAGCCACGCCGGGATATTAAACAGGACTTATTCCCCCCCCCCCTCCCACCGCACCGCCTCCTCAGTGAGCTTCA
CCATGGAAACCCATCTCTTCAGGGGCGAGGGCAGAGGAGCAGGAAGACACACTGTATGTGTGGTATTCCCCTGGACCATA
CCAGGGAGGCTGTCGACAGCCAGCCACAATCGGGAGGTAGCGCCGGGTTTAGCAAAAGGCCAGCCGAGCAGGGAGAATTC
ATGAATATGTATTCGAAGAGGCGAAGGACTGTAGGAGTAAACAACAGCGAAACAAGTTCAGAACAATGGAGGGAAACTTT
ACATCGCAGAGAGATGATTCCTCTGTAACTAGAAGGTACAGGGAGATGCATAGTTATTAGCATTGTTAATTCTGTTTTCA
AAGAGAACCTAAAAGGGGTAACTGCCTACCGGTAACCAAATTTGCTTGGCTTGTTTTAGGGTTGTTACATATATAGGTTA
TTCCACTAGCGGTTACTTTGGTAGGCCTATGATGTCATGTAAAGTGGGAGAACATCGGAAAAGTAGCACTACCAAACAGT
TCAGAGGAAGAGCTTAACAACAAGAACAACAAGGCCTGTTTCAAAAAACATCAAAAGGGGAATTGAAAAAAAAAAACAAGG
CACAAGAAGATCATGCAGAGTACTGGTACACAGTTGTATTTCACAGGGGACATTATGACAATCAGTGACACTTATTTTGC
ACTTCTGGTGCTCTGCTGCGACTGTCATTATAATCTCCAATCAAACTGAGTGTCATCGCCACTGACACGCTACATCTTTT
ATTGTGCCGCTTCACGTTCCAGAATGCCCTTGATGTTTATAGAGCTTAGATGGTCTCAAGCGTCAGGGAGAAGAGTTTCT
TCTCATACTCTAATGTATTCAGAAATGGATCTCAAAGAGGTGCTAAATTTGGAGTTGTCTGCACGGCCATGGGTTCTCAG
GCTTGTTAAAACCAGACGCTCCTCCATTTTGATGTAATTAGCAACAAACACATGATGTTGCCTTGGCTTTGATATCCTAC
CGTTCACAGTTCCCAAGAGGGTCACACGAACACAACGTTCATCTAGACAAACCTGTCTGTTTTGAAAAGGGTTTGGCAGG
CATGCATTGACAGGCTGTTCCCAATACAGTGCACTTGACAAGCTATTTATGATAGAGAGCTGACATTTTCCAGATCAATA
CAGAACACGGACAAGCCAATATTTATTTTTTAGAATACACTCATTCTGTAATAAAAGATGCATATCTTAACCACACATAA
AAATGGTAAATATATTACAATAACCTACTAGAATAAAGGAATTATAATAACGCGGTTCATTACCCCATTCTAAAGAGAGA
AAATATGCAAGGAGGCACTCATTTGCCATCAGGTTAATTATCTGTGTTTCAGAGGCGCTAATGCGTTCACTTTAGAAGAT
GAATGAATTATGACTCTTATTTACTCAAGCTTAGGTTTTTCGTCACCATCAGAATTAAGTGGCATGCCCCAGGGTCTAGA

CACAGAGTGCAATGTGCAGTAGAGGAGTAGTACAGCACACTACCAGAACAGCAACAGCACAGACTGGACAATTTGAAAGG
GGACAGGATATGGAGCCATTTTGGCAATAGCTAAGTCATCATTTCCTTTGTCTTATTATTATTAATTGTTTACATTATTT
ACTAACTTTGAACAAAATAAGAGGTTTGACAGACGGATGAAAAAATGACTGAGTACTTTCCATAACTAAACTCAGCAAAA
AAAGAAAAGTCCTCTCACTGTCAACTGCGTTTATTTCCAGCAAACTTAACATGTGTAAATATTTGTATGAATATAAGATT
CAACAACTGAACAACAACTGAACAAGTTCCACAGACATGTGACTAACAGAAATGGAATAATGTCTCCCTGAACAAAGGAC
TTCACCAGATTTGCCAGTTCTTGCTGTGAGATGTTACCCCACTCTTCCACCAAGGCACCTGCATTTCTTGGGAGAATGGC
CCTAGCCCTCACCCTCCGAGCCAACAGGTCCCAGACGTGCTCAATGGGATTGAAATCCGGGCTCTTCGCTGGCCATGGCA
GAACACTGACATTCCTGTCTTGCAGGAAATCACGCACAGAATGAGCAGTATGGCTGGTGGCATTGTCATGCTGGAGGGTC
ATGTCAGGATGAGCCTGCATGAAGGGTACCACATGAGGGAGGAGGATGTCTTCCCTGTAACGCACAGCGTTGAGATTGCC
TGCAATGACAACAAGCTCAGTTCGATGATGCTGTGACACACCGCCCCAGACCATGACGGACCCTCCACCTCCAAATCGAT
CCCGCTCCAGAGTACAGGCCTCGATGTAATGCTCATTCCTTCGACGATAAACGCAAATCCGACCATCACCCCTGGTGAGA
CAAAACCACGACTCCTCAGTGAAGAGCCCTTTTTGCCAGTCCTGTCTGGTCCAGCGACGGTGGGTTTGTGCCCGTAGGCG
ATGTTGTTGCTGATGATGTCTGGTGAGGACCTGCCTTACAACAAGCCTACAAGCCCTCAGTCCAGCCTCTCTCAGCCTAT
TGCGGACAGTCTGAGCACTGATGGAGGGATTGTGCGTTCCTGGTGTAACTCGGGCAGTTGTTGTTGCCATCCTGTACCTG
TCCCGCAGGTGTGATGTTCGGATGTACCAATCCTGTGCAGGTGTTGTTACACGTGGTCTGCCACTGCGAGGACGATCAGC
TGTCCGTCCTGTCTCCTTGTAGCGCTGTCTTAGGCGCCTCACAGTACGGACATTGCAATTTATTTCCCTGGCCACATCTG
CAGTCCTCATGCCTCCTTGCAGCATGCCTAAGGCACATTCACGCAGATGAGCAGGGACCCTGGGCATCTTTCTTTTGGTG
TTTTTCAGAGTCAGTAGAAAGGCCTCTTTAGTATCCTAAATCTTCATAACTGTGACCTTAATTGTCTACCGTCTGTAAAC
TGTTAGTGTCTTAACGACCGTTCCACAGGTGCATGTTCATTAATTGTTTATGGTTCATTGAACAAGCATGGGAAACAGTG
TTTAGACCCTTTACAATGAAGATCTGTGAAGTTATTTGGATTTTTACGAATTATCTTTGAAAGACATGGTCCTAAAAAAG
GGACGTTTCTTTTTTTGCTGAGTTTACTTTACTAGAACGATACCATAGCAGAACAGATGTTACATTGAAATGTAATGTGT
GTAAAAACACAAAGTGGACGTTGGATAGTTTGTTTACCGTTGTCGTTGCCCAGGTGGAAGTATCCATCAACGAGTGAGGC
CCCGTTGTTGAAGTGCTGGGTCATGTGATCCAGCCAGTTGGCCTCCACCCCTTTGACCCCCTCGTCACGGAGTTGAACCC
TCAGCGGTACCTTCACAGTGTAGTACTTCAGTTGGCCCTCACGGTCAGTTGGGTGGTGGAACAGGGCTAGCAGCTCCACC
CCTGAGAACACAGAACACTCAATCTCAATCTCTTTAGATTGTGCTGACATGAAACATGTCATGCAACAGAGATTCATAAC
GTGACAGAGTTGATACTTTGTAACAGAGGTGATTTATAACACTTAACAGAGTTGACTCATACCCCATAACAGAGGTGATT
TATGACACATAACAGAGTTGACTCATACCCCCATAACAGAGGTGATTAATAACACATAACAGAGTTGACTCATCATCATT
TTTCCACAATCCCCTCCCAAAGGCTTGTGCCTCAATTGAAATGGTCCATTAATATGGACCAATGCATACAAATTTGGATG
GACTGTTGTTTTGGCAGCAGCCATTTAAATCAATTGCGGCATAATCTTCTCTTTTTAATGCTAATATCTGCCATTCCCAT
GTGAACCAGACGGAGGGCCAGAATGTGTTAGACTCCCACACACGAAGACTCACAAAAGATGCACGCACGCACGCCTCACA
CACACAGAGTGCATCAGAGGGGACCATGGTTCAGTTGCTCTGGTTTTCTGTCAGTCATTCAGTCAGACACCCAGGAAGAT
TGAATCATTTATATCCATTTCCTTTCTGCCACGTCTGACTGATGTGACAGCTGGCCACCAAATTCCCAAAATCCTGTCGT
TTTCCACTCTTCAAAGCTCCAGAATGACAACTGAATGACAGTAATGCATCACAACTGTCCCTGCATACGTTAGAGAAACT
ACAAACTTATTGAAAACCAAGCCAAGTCAATACAACAAAGTCACTCTACTAATCTTCATTTTTCTGTTTTTTTTCCTCCC
AAAAGAGTGAATTTAAATGAGGCTTTCACATGATATTAAAGTTGATGTTTACTCGTTCAAGGCCAGCGGTTTTGAGGAAC
TGCTGGCTTCACCATTCCACTAAATGCTCTGTGTCTGCAATGTGTCACAGACCGTTTATAATTTATGCATAGCTGACAGA
CCTTGCACTAATGCATCCGACTAAACCAGACAGCTCAATGGTAACGCATTTAAAGATGAGATAATTGTGGTAAAATGTGC
AACATACATTTTATAACAACACTTATAACAATTTGCGGCATAGGTTATTTCCATTTGACAGCTAGCTTTGTATGCTAGGT
TAAGTCTGAGTTACGGTAGAGGTATGACCTGGAGATAAAGATAATCCAGGGTCCATACCTATCAGATCATTCCCACATGA
TGACCACTTCACAACGCAACTGTGTCGACAATTTCAGCATCAGGGGCATCAAAGTTAAACAAATATTTGAGGATCGCCCT
TGACTTGCCCTGAGATTTGGATGGCGATAACACAAACAGTAGGGTGTATTGGCTATTGAGACCTGCCAGGTCACAGTCAC
ATGGAAAATGGCAGACCTGCTATGAGACGATACCTTGTGTGGTCCAATCATTTGAGAAAATCGACAACTAACGAGTAAAC
GAGCGCATGAACGCAATTACAACATTCCAAATGGGTCTCCTTTTAAAGTCTGGTCTGATGGATGCCTGTCCAAGTCACAA
GGTCAGGCCCCATAATCCTTACTGGTAATGTGTACTGTACAGAACCACTGTTGAACATGACAGCATCTGTTCTGCTAATC
CACCCTTCTCAACACTGATCTAATCAGGACTAACATTTCATAATAAATACATGCTCAAATCAACATGTAGGCCAGCTTTC
TCTGCAACGTGATTTTTAGTTGGAGAGTGGTTAGCTTTATACTGCTTCATTTCTAAGAGGTAACGGCAATAATGTGAGTG
ACCATTGTGATGTTTGTCCAATCTCTGTCTTTTTTTATACCATCACTTCCTCCTCTCACACCTGTGATCCCACAGGAATG
TTCTCAGCACTAGTTTCAATAACCTCAACGGTCTTTGATGCTATTTTATAGCACGTCGGTTTCAAGTCCCAGTTTCACTT
CTGACTGTTGGCTTGTGACCCTGTTCCCTGTTCCACATTCTGCTGTTCAGGAGGATTATGGGGGCAAAGCCACCCAGAAC
TGAGACTTTGAGTCAATTTCAGCAGAGAGCATTAGCGAAGAGGACAAGGGCACTTGGCACATGGTCACACACCATGCTGG
CTATGCTGTGGTGGTGGTGGTGGTGTGAACTGTGGAGGTTTGGGGAGGAGGGAGGAGGCTGAGTGAGGTAGGGGGGGGGA
TTATCTCACTGCAGTACACACTTGAGGGACGTCCAGATGGACCAGGGTGCTGCGTCTACAGGGAGCCAAGTATGTATGGG
CACACACGCACAGAGGGGAGCAATCAACACACATGCACATTTTATGAGCACGATTTCCAACTGGGGTTGAAAGAGAATGT
GTGTGAGAGAGAGAGAAGAGCTGTCCATCTGGTATTCTAAGATGCTGAAATATGCTAATTTAATCCAACTTTATGCTGCA
CCACAGTCTGAGGAAATTGAAGAACCATTTATGCGGTTTCAAGTTGAATCTTGCCTGGCATACTGTATACAAATCCAATT
ACATAGTAACAAATGGAAATCTGACAATACTCACCATTGGGTCTCTGAGTGTGACGGCTTGGTTTCTCCTTCAGTTCTAT
GACGTTTGTCTTGTTATTGTTGTGTGTCAGTGTGTCTTCCACTATAACATCCTGATCATCATCTTGCTTTCTCTCTGTGG
ATGGTGTTATGGGAAGAGAGGACCTCCCATCATGGTTGCTTTCCCCGACAGTGGTCTCACTCCCCCCAGTGTCCCTAGTC
TGGTCCTCCCCCTCCCCACCCTTACATGAGGAGCCTTGGCCCTCATCATCCCCAGTGTCCATCGCCTGCTCCTGGGCTCC
TGGTTCTCCATTGTTCCGAGGGCTAGTGGGGCTTCGGCTGCTCAGGGAGCCTAGCATGGAGCTGGGGCTGGAGTGGAGGG
AGAGGGAGGGCGTCTCTGGTTCTCTGGTCTGGGTACCTCGTACCCCACACGGCTCCTGGATGAAGCCCACAAACATGACC
CCCTGGTCTGCAGCATCCTGGATCTGCTGGAGAGACAGACAGCAAACAACCGCCTTAGAGAGATCCTTGGACGACCTGAC
TTTTACAAATACAACTTAATAATACCAACCTTTCGTACTGTATGCTCTGTTATGATGGACTGTTATTTAAGATCATGTTG
GCTAATCATGAGTCGATCAATGTTGTAGCTTGTCTTCAATGGACTTTCTAAAAAATGTGTGATAATCTTGAAACACAATC
TAGAACATAAGCACACATTGCTAAGTCATACCATTATAGATCCATAGCTATTTTTTCACCCTGAATTTCCCCCATTTCCT
CCCTATTTTATCAACCAAATGGATTCCAGGGGAACTGTTTACATACCTGGTATTATCATAGAACTATAGAGGCACTCCCC
CTCCCTCCACCCCTCTTCCCAGCATCAGCCATTCAAATTGAACTAGAGCTGATTGGATTGCAATGGCTCCAGCCCTGGCA
GGACTGCATCCCAAATCAAGCATTGCTTTTCCCATTTCATTGTCATCTGGCATTTTATGTCAATATTGGGATTTTCCATC
AAAATGAAATTGAAATGTTAACTGTCAAGCAGTGTTAATGATGCCATTCTCCACTCACATTCTGTCACAAGATCCAGCCA

GAGGCAGTACATTCTGCTATCTGCAGCCGGGGACCAGGAGAATCCAAATCAAATCAGCACGGGTGCCATATCACAGGTTA
TCCTCTATGAAATGAAACTGTAAAACAGTCACATCGTTTCCTAGCTCCATTGCTTAATATCTATTGAACGTTCACTCAGC
ACTTGCACATTTTTATTCCCAGACAAATTGCAAAATGGAATTGCCAAGACGAAAATAAAAATATTGTATCTCATTTGAGG
GTAACGTTGCCAGTCAATGAGGGAGAGGGATCATTTCTACTGTGTGTGTGTGTGTGTGTGTGTGTGTGTGTGTGTGTGTG
TGTGTGTGTGTGTGTGTGTGTGTGTGTGTGTGTGTGTGTGTGTGTGTGTGTGTGTGTGTGTTATCGTGGGTCGACTCAGGTG
TGAAGGTAATGGATAGCCTTTGAAATGGTCCGCATTGAATGGAAAATCACACTGCTGTGTATTGCACATGCTCAGGTCAG
AGAAACAGGTAAACGTCATATGTTTCTGTTACATTGCCATTTATAATGACAAGTCTCTTTGCTTTTAATAGACGTCTGTG
ATATCTCCTAATATAATGTGGTTATGGCCATCAGCTTGCTTGAGAGTGGTTAGTGCTAACATGTCTTCACTGCAGAATAA
GAGCAGCCCTCTGTTAAGACTTTCATTTTCCAGTTCTACATAGGCTACATATTTCCTATTTTCCAGATATTAGAGAGAGT
GCTCCAGGAGAGGAGAGTTTGAAGGTCAGAATGAAACTCTGGTCCTCAGGCGACTGGCCGGCTGAATTGATACTCCCCAA
TGTTTTATTAAAACTAAGGACCGTCTCGTCTACTGGCTGTGGAAAGCGAGTCGAGATAAAGAAGAAACCCAACAGATAAG
TTTTTGCCCACAGCGCCTTTACGACTTATAGGCTGGTCAGTTGTGTCTGTGATCTGAGTCCACTATACCCTTTTCACACT
ATCATGCCACCTGAACAAAACTGTGCAGCCTTAATATTTTCGTTCACATCGATGTCCTTTTCAGCACAGTTGTGGATGCC
TAACCACTAACCAGGCCAAGCTCAGTACGGCTTGGCTTGGCTCAGCTCGTCTCAGTAGTGTGACAGGGCCTTATCTGAGG
CTCACCTTCTTGACATAGCCCTGGATGAGCTCAGGGGTGGGCACCTGGTCGGTAGACAGACACTGCTCCAGCTGCAGATG
GTAGGGAGCACAGCTTGACTGGCTCTTCTCCAACCTGCGGTAGCAGCCCACACAGCGGGAGAGAGAGGAACAACACATTT
CAGCCACAGTCACTTCAGTCATGCACGATACAGACATACACCACATTCTGTAATACATATCTGAAATGGTTGCACAGAGA
AATCCTTGGAAATTAAGTATTTATGGATTTATGAAAATCATTTATTAATAAATCGCATATGAGCCGTGTTAATGTAATTG
TTGGCCTGTATGACAAAAAAAAAGAAGAAGCACTTAACATAGACACAAATCAGGATTGAAAGGGGCACATTAGGGTGGGA
TAGGAAACTCAATGCAATACAATTCTTGTCTTGTTTTTCCACTTATCAGTATTGTGGGTGGATGGAATCTATTTATATGG
GTCTGGATTTTTCTATTTTCTCTGGAGGAAATCCCACAGCTAGCCCTGGAGGAGGCCAATGCCCCAGCTTCCATCGCTCT
GCTTCACCTCTCATCCTGTTGACAACACGGATAAGACCCTTCCACCCTCGCCACAGCATGTGTGTGTATGTACAGTACCT
ACCTGTGCGTGTGTGTGTGCGTGCGTGTGTGTGTGTGCGCGTGTGTGTGCGCGTGTGTGTGTGCGTGTGTGTGTGCGTGT
GTGTGTGTGTGTGTGTGTGTGTGTGTGTGTGTGTGTGTGTGTGTAAGGAGAGTTTAAACATTATCACATGCAGCCCAGGCCT
AGCTCTCTCTCTGTCACACGGACTTCACAACACAGCGGGAACCTGAGTCATCACTCACAGTCAACAACACCCCACAGAAG
TCAGACATCAATGTCACGCAAGTCCCCCATCATTATGCAACTTATCCCAGATCAAATGTCAGTAGCAAACTGTGTCAAGG
AAAATCTGCGCATTGGATGCTCTGAATCAGGTAGAGGTTTTTGCAAACTTACTGTGCAATTCACAGCACCGTGAAGCGCC
AATGTAAAGGAAAACCTTTGAATTCTTGCTATTTGATTTCCACGTTCTGTTATTGGAAATCATGAAAAGCAGATTGGATC
TGTAAACTTGAGGAGGGCTCAAATCCATAATGGACAAAGTGGGAGAAAATAACTAGTCTCCCATCGCCTCTCTTGAGGTA
AGGACTTCTTAAAAAAATAGCCCCATCACCTGCAGTCCATTTTGTCAGTGTGAAACAGGTTACTTGCATTCAACCCCATA
GATCTGCTGTCTACCGCCAGGCCGCTAGAGTAAGTGTCAAACGCATGCCTGGCTCCAGCCTGCAGCTGGCCTGACTGCAG
CCTCCTCCTGGGTGTACGTCTGCCGGACTTCCCACTCCTTATGCAGACTGCTGCTGCTATCTATTCAGCTGCACTGTCAG
TCAGGTTCAACCGTTCAAGGTTCTGTATCCCGGACCACAGGGAGTATTAATGGCACAGTCTGGGATTTGGAATCTGGGGA
TGGTTATTTTAATTCTTGATATGGTGGGCCGTGATGAAGTTTTTTTGAAACCCAGAATGTAGCTTTAAACCCAGAAGCCC
TCACATCGGGCGCCAAAATCGCAATTCCAAAAATCACAATCTCCATGACAACGGCTCGGACAGGATCCACCATCGAACCT
GTGTTTAGCAATCTTTCATCCCTGGCCTTTAATAATATAGCACATCTTTTTCTAATGTCAGCTTTTCACAGAGTCATGAA
TAATTTCTTTGGACGAATCCCCTCTAATGGTTTCTGGTCCATACAGCTCTCTCCCCGAGCACATGTGAATTCTACAATTA
TCCAATAGATGATGAATATATCCCATCAGACACTGCCTGCTGAACTGTAACCCCGCTACGCCGCAACCAAATTGAACATT
GGGAAATTTGACAGTTGACATTTGAAAATTAAAATGGTCCTATCCATTCTCTCTGATCCGCAGATTTGAGCAGGTTACCA
CGGTCACCAGCGACAAATCACAGTCAAAGAGACAACAGGCTTTTAAAGTTATGGCCTACTTAAAGATGCACTCCAGGATC
TTAAGCACAACAAATTCGGAGCGTATAGTACAAATTGGATTGAAAACATATCATTGCAAAAAAAAATCGACAACATTTGC
AGGACATAACATATCATACAAAATGAATGACGTACTGTAGTACCCAATTTGATGACATAGTACACAAAAAAAAGGGACCA
TTTTTGGCTCGTGAGCGCCACTTTCAAAACTACTGGCTGAAATTATACAAGTTTGAGAGTTTATCTTGAACTAAATAGTT
GTTCCAGGTGACAGCGTTTAACATGAGCAATACTGGCTCCCAGCTCTATATAAGCTACCTGCCCCTGTGTAATTCCTGGC
TCTAGAAGCTTTTGCAATGTCACACTACTGTGCAGCTATACCCTTGTTCACTGAGGCTTTACAATTGAGGCTAATACTGT
AGAAAATTGGCTGGGATTGTATCCAAAGAGACATTGTGTGCATACTAAACACAGCACATCCAGCTGCTGCTAAGCATAGC
CTAGCTGCTGTTCTTGTTCTGGTTCTCACACACACACACTATCTCTCCCCCTCTGTCTGTCTCTCCTTCTGTCTGTCTCC
AGCTGACGCATAGCGCACTGCAGAGCGAAGAAGACACTTTTGGGTCTGTTTGCTTTGGGAGAGTGATGAATGGTTGCGCT
GTCATCTGCAGTTTTCGCTCCCTGATTCAAGGGACTGACAAGGGAATAGGAGAGGAAGATGCTACCTGGTTGATTTTTTT
GGTTCTTTCTCTTTTATCCCCCCCCTCCCCCCTGGGGCTGGTGCTGATGTGAGCAGCCGTCTCCACGGTGACAGACGCTG
AGATAAAGCAGGACGGTTTGGCCGGAGCCATCAAAGGATGGCAGCCCTTTCCCCTTTCTCTCTTTCTAACTCCCGCTCGT
TCACACTCCAGCTGTGATCGTGTTTTCTCTCTCTACCGCTTTCTATTCCTCTCCTCATATTCTTTTTTTTTCCTTCAGAG
GAAGGCAACCATGTCAGTGTGTAAAAACCATGCTGCTGCCCATTCATCCATCCCTTTTCATACAAAATCAATATTTATGT
GCTCCCAGCATAGGATTAGTGTCTGGAACTTGTTTGTGAGTGGGTTTTGTGTAGAGACAGTGGGAGAGGGGTGAGATGTA
TTTCCCTAGACCTTAGGAGCTCATTAATGGGCACAGGCTGTCTTTAGAAAGAAGGCAAGAGCCACCTGATTGGATGGATT
GTTGTGCTTTACAACTAACTAAATCCTTCACCTGTACCAGCCAAATGGTTTGATCCTTTCAAGACAACACTAGGCAACCA
GGAAGCACAAAACAAGAACAACAGGGCAAGGAGCCTAAACCCCAATATACTGAGGAATGTACAGGAATTATTTGTCCATG
TTTGGAATTATTTATTTTAGGAATCTTGAGATGAATTCCTGGATCATAATTCAACGAGAGGAGAGTCCAGAGATTTATGG
AGTGTGGCGTCACTGTCCGACCCCAGGGCTCTGGATCCACGAGAGAGAACAGACCAGGCCATAAAACACTGTTAAGAGGG
GAGGGTGAGAGGTTCAACTGCTGCTCTAACCTCTCTGTGGGGAAAGTGACTAGTGAGTGTCTGGTTCTACTTCACACAGG
CAGTCATTCCCAGAGACAGACCTGCATGTGTCCTAACACTCCCCCGAAAAAAACGCATGAAACCGCTGTCCTTCATAACA
GCCAGCTGGTAGAACACGGGGCATACGAGGCAAGCCAGACAGGGCTTCAGCCGGAAACCACCACTACCAGCACCACCCCG
CCATGCCCTTCACATACACTTGGAGTAAAACCATGCCCTGATCCAAGCTGACTCTCACCCAGGGCTCGCGACACTGACAG
GTGCCCGGGTGGAGGGCAGACAGGGGGGGGGGGGGGGGGCAGCTGTGACTTTGGCCCATTCCACATGTTGTCCGGAGCAT
CGAAAGCTTGGAAAGAGATTATTTGCTTGCTAATGACCAAAACAACAGCCCTGTGACAGCTAGCCCAAAACAACCTCTGT
AATACCACTGTCTACATCCATGTAGTAATATACATTTTGATTTGCTGTACATATTACGTAATGACAAAGCTTACACAAAC
ACAAATGTATTGACCCTGGATAAGGCTTATGCTAATTGTGTCAATGTAGGTGCTCTGAATAAGTGCAATGTGTATGTACT
CGTACCCATCATCGAGTCGGATCAGCACGGCCCTGTAGAGCTGGTGCTGGGGGCTGACTGCGTCGGAACCACAGGGATGA
ATGAAGGGGTGAACGGCAACTAGGGAGAAGCCCTGCTGGTACAGGTCCTGGAGCTGGGCTGGGAGCTCCCGCACAGACGA

AAGACACAGTGTAGATGAGCCATCTGAAAGACAGGGAACGAGAAAAGGTACAGATTCAGAAGAACGTGTGTGGTTGCTTC
AGGTGTTTTGTCATGGTAGTGGAAGTTATAGTGATAGTACTGACTATAGTAGTAGTAGTAGTAGTAGTAGTAGTAGTCCA
GTGAATGATACTCAAATTCAGGTAAACGTGATAAACAGAGAGGAGATTCAGCAACTTNNNNNNNNNNNNNNNNNNNNNNN
NNNNNNNNNNNNNNNNNNNNNNNNNNNNNNNNNNNNNNNNNNNNNNNNNNNNNNNNNNNNNNNNNNNNNNNNNNNNNNNN
NNNNNNNNNNNNNNNNNNNNNNNNNNNNNNNNNNNNNNNNNNNNNNNNNNNNNNNNNNNNNNNNNNNNNNNNNNNNNNNN
NNNNNNNNNAAAATCCTCTGTCCCCTCAGGCCAGTAACTGGTTGGCTAAAAATATCCCCACATTTTCTCAATCACACTCT
GTCCTACTCCGACACAATACAGGAGCTCCCAGAGCTAAAAGGCATGACAGAGAAAAGAAATAGGCCACCCTCTCAAAATG
TGTGGAACATTTTTTTTCTGCCCTACTGCCGTTGGAAATGTAATTAATCCTTCTGTGATCTGTGATTTTATTTTCCTGTT
TTGGTGATCCACAAACTCTTTGCCCTTTCCCTTGAGGCTTGGTTCTATGCTGTGTATGTATGCTGGGTCTGTGAGATGGT
GGATATATAATAAAGTGACTAGAATTCTCTCTGCTTACTTTTTGAACACTCCATTGAGATGAGTAAATATAGAATGTTTT
TCATTAAGAGCCAATGGAAACACACCCATAGGGGACTTCTTAAGTTGCCTCTAATGGCTATGAGAGCTGATCCTGAAATC
GAATCCAAAAGCCTTCATACGGTGACCAACCGATATGTATATTTAACATTCACACGTCTCTTTTGAAGCCTAATGCAGGG
TAATGCGAGGTCTCACAAAAATGTGTGATGCGGTGAGAGCGTTTATTTGGCGATAAGACCCTGTTATCCATACAGAAACC
CATTTTTTTAAGTAGATAAATTCAGATTTGATTTTGTGCAGTTTATGTATATCTCAAGTCAATGTTTGCGTTTAATATCG
TAAACGTGAGGGCCCTTGTTTGAAAAGCCCATATTCAGACTGAGGCAGTCTGGTTAATGTTATCAACAAATAGGAAGCCA
ATTTCTTTTCCCTTTTTTGTTTACCCAGAAAATAAATTAACTTACATAAACAGAAGGAAAAAAATGAATCGTACCTTGAC
AAATAATGAAAGAAAAGGCTCCTGGCTGACAGCCAATAAATTGGTTTTCTGTCAAATTCTGTTAGCACGGAGTCATCAAA
CAAAATACACCCATTTAAGAATGAAGAGAAAACAAAATGTGGGATTTTTTTTTAAATGATCTTCTTTTCCAGGGTATGTC
CACACACAGCCATATTAGATGAAAATAAGTTTAACTGTTGGATCCTATTTTTGTAGAAATAAAAAGTTATTAGTTTAGTT
GATCTTCTTTACATGGCAACTGCCTGGCATTGATCACATGTGCACATTAATACATTTTCTTGTGTGTGAGTCATCCTCAA
GAACATGATGATCCAAACAAGTTTCAAATACTTTTCAATCATCTTGGTCCTACTACACAATACATTATAGTGATATACAG
AGCTTTCGGAAAGGATTCAGACCCCTTGACTTATTCTACAATGTATTAAATTAAAACAATTCCTCAGCAATCTACACAAA
ATACCCCATAATGACAAAGTGAAAAAAAGAACAAAAAAACCTTATTTACATAATTATTCAGACCCTTTGCTATGAGACTC
GAAATTGAGCTCAGGTGCATCCTGTTTCCATTGATCATCCATGAGATGTTTATACAACTTGATTGGAGTTCACCTGTGGT
AAATTAAATTGATTGGATATGATTTGGAAAGGCACACACCTGTCTATATAATGTCCCACGGTTGACAGTGAATGCCAGAG
CAAAAAACATGCCATGAGATCGAAGGAATTGTCCATAGAGCTCCAAGACAGGATTGTGTTGAGGCACAGATCTGGGGAAG
GGTAGCAAAACATTTCTGCAGCATTGAAGGTCCCCATGAGCACAGTGGCATCCATCATTCTTAAATGTAAGAAGTTTGGA
ACCACCAAGACTCTTCCTAGAGCTGGCTGCCCGGCCAAACTGAGCAATCGGTGGAGAAGGGCCTAGGTCGGGAGGTGACC
AAAAACCTGATGGCCACTCTGACAGAGCTCCAGAGTTCTTCTGTGGAGATGGGAGAACCTTCCAGAAGGACAACCATCTC
TGCAGCACTCCATCAATCAGGCCTTTATGGTAGAGTGGCCAGACAGAAGCTACTCCTCAGTAAAACGGACATGACAGCCC
GCTTGGAGTTTGCCAAAAGGCACCTAAAGTACTCTCAGACCATGAGAAACAAGATCTCTGATCTGATGAAACCAAGATTG
AACTCTTTGGCCTGAATGCCAAGCGTCGTGTCTGGAGGAAACCTGGCACTATCCCTACGGTGAAGCATGGTGGTGGCAGC
ATCATGCTGTGGGGATGTTTTTCAGTGGCAGGGATTGGGAGACTAGTCAGGATCAAGGAAAAGATGAACGGAGCAATGTA
CAGATATATCCTTGATGAAAACCTGCTCCAGAGCTCTCTGGACCTCAGACTGGGGCAAAGGTTCACCGTCCAACAGGACA
ACAACCCTCAGCTCACAACCAAGACAACGCAGGAGTGGCTTCGGGACAAGTCTCTGAATGTCCTTGAGTGGCCCAACCAG
AGCTTTACGCACAATTTCTTCGACCTCATGGCTTGGTAAGCATTTCAGGGTAAGAGCTGCACCTGTTGTATTCGGCAAAT
GTGAAAAATAAAATTGTATTTTATTTTACTTGAACCCGATCGAACATGGAGAGACCTGAAAATAGCTGTGCAGCGATGCT
CCCCATCCAACCTGACAGAGCTTGAGAGGATCTGCAGAGAAGAATGGGAGAAACTCCCCAAATACAGGTGTGCCAAGCTT
GTTGTATCATACCCAAGAAGACTTGAGGCTGTAATCGCTGCCAAAGATGTTTCAACAAAGTACTGAGTAAAGGGTCTGAA
TACTTATGTAAAATGTGATATTTCAGTGTTTTTTTATTTATCAATTTGCAAACATTTCTAAAAACCTGCTTTTGCTTTGT
CATTATGGGGTATTGTGTGTAGCTTGATGAGGAAACATGAATGAATTCATTTTAGAATAAGGCTGTAACGTACCAAAATG
TGGAAAAGGTCAAGGGGTCTGAATACTTTCCGAATGCACTGTATATTTCACCTAGGCTGTATATTTCACCTGGGTATTCA
CACTCCTTGACTTTTTTTTAAACATTTTGTTGTCTTACAGCCTGAAATGTAAATGGATTAAATTTAGATTTTGTGTCACT
GGCCTACACACAATACCCCAAAATGTCAAAGTGGAAATATCAGTCGAGCAGTGAACTTCAAACACAAATTCAATCACATA
GACCAGAGAGGTTTTCCAATGCCTGGCAAACAAGGGCGTCTATTGGTAGATGTGTGGAAAAAAAGCAGACATTGAATATC
ACTTTAATAAATGATGAAGTTATTAATTACACTTTGGATAGTGTATCAATACACCCAGTCACTACAAAGATACAGGCGTC
CTTCCTAACTCAGTTGCTGGAGAGGAAGGAAACCGCTCAGGGATTTCACAATGAGGCCAATGGTGACTTTAAAACAATTA
CAGAGTTTAATGGCTGTGAAAGGAGAAAATTGAGGATGGATCAACAACATTGTAGTAACACTGTCATGACCCATATATTT
ACACCTGATGTGACATACAGTATATTGCGTTATTTTATGGCTGGTTATGACGCATTCATACGTTTCAAAATCTACATTTA
TTCAAATGTGTCAGATGTAATCACTGCCAAAGGTGATTCTAACATGTATTGACTCTGGGGTGTGAATACTTATGTAAATG
AGATATTTCTCAATTTCATTTTCAATACATTTGCAAACATTTCTAAAAACATGTTTTTACTTTGTCATTATGGGGTATTG
TGTGTAGATAAATATTTAATCAATTTTGAATTCAGGTTGTGACACAACAATGTGGAATAAGTCAAGGGGTATGAGTAACT
TTTTGATGGCACTGTAAATGTGACATATTATACAACGGTGGGTCTAATCCTGAATGCTGATTGGTTAAAAACGCATTCCA
GCCGGTGTCTATTCCACAAGTTACCACCGACTAAATCTATGATGTCAAAATGCCTATTTACTCTGTTCCATCTGACTGCG
CAATCTGCTGTCTCATCAGCCCAGCCAGGCAATTTATAAACTTGATCTCGACTATAAAAAGGATCAAGACATTATCTCAC
ATTTATTTTAGACTAACATTCAGATTTCAACAGCGGAGATTTGTATAAACCTTGCTGTCTCTCCAACATTTGCAACATTG
TTTCAATATTCAATTTCGACCTCCAGCTGTCCCATAGTAATGAACGTTTTGGGAGTCGGGGTGAGACAGACAGGAAGGTT
TTCTCAGCCAGTCAAAATCATGACTCAGCATCATTTTTATGGATATATAAAAATAACTATCAATAAAAAGCAGGTCAAAC
GAAGTGGAGCTAGTTTGAAGTCTTTCCAGCTTCAGTTTGAATTGATTGTGTTAGCTGTGTTGTTGGCTAGCTCCTTTGAA
CAAATGTCCTGACGAGAAAGCACATTTTCTATGTCAGGTGAAATCGTGCATCATCAGCTCCTTATGAATGTATCCACATA
AATGTCACAGCTTAAAGAAACGTTAAACAGCTTAAACAAATGCAAATGCAGCTACTTCGTTGTTATTCTGGCTGCACTGT
TTAACGTGACTGTAAGTTAGCCGTAGTTGGCTAGCTAGCAAGCAAGGGATAAGAACGTTGCCAGCCAATATGGCAATGGA
ACATTTAGAATGAATGCCTGGGTCGCGTCCGTAAACACAGAACAAAAAGACTGAACGACTGGGTCGCATTTCTGGTCACT
GAACCGATAGAAGGAACAGCTTGGGTAGCAACCCTAGATTTGTGTCAGCGCTATTTCTTGTGGAAGGGTGAAATTGTATG
AATAAAATCATAAAAATATATTATTGAATGAAAATGTCAATCATGATTTTAATATGTTGGTAACCCGTTGTATAAAAGTG
ATTATGCCCTCGAAGTCAGTGTTTGACTTCGTCTCGTGCCTAACACCCGTGCCAATATATCCTCCAAACACTGGCTTCTT
GGGCATTATCACTTAATTATATCATGCGAGATATATGTAATCAAAGGTGACATTCAACTAATGTCAGCCCAGGTGAAATA
TACTCTATCTACAATGGGTAGATGTCACTATTTTGACATCCTCTAATGATACAATGTTGCAGCGCCAACATGTTTACAGT
ATATTAGCATCATACACCCCACTTGCCATACAACATTAAGACATGTATAGACTCTGACATCCCTTATAATGACACCGGCT

CAATTGTTTATTCGCCCTCTACCATCAGTTCCATGTAAGCACCGTTGAGCTCGGCTGTTGCACTTCCATCAGTCAATGTG
TTAAAAATGAAATGAGTAAATAGGAAATTAATAAGCTGAGAGATTTGAGCGTGTGGGCTCCACCGACGCTCGGTACTCCG
CCGCTGGGGGACATGGTTCAATACGATTTGCATGAAACTTGAGTGGGGGAAAATAGACCCGGAATAAATTAGCTATTAGG
ACTCAGGGAAAAGTGAAATGTTCTTCCTGCTATCTGCTGTTCAGCATCATTTTCCCTCATCACAATTAAAGCGCTGTATA
TTTTTCTACCACACTAATAACTCATTTTGCTTATGGACAATATCATATTCAAACTGATGTACAAACAACTTGAAGATTAT
AGTATCATTTCATTCAATTGAAAAAGTTAGCTGCTTTGAGTTCAGGGTATGGGAAGTGGTGTTTTGCATGACAAAACGAG
ATGCGTACTGTGGAGTAGAATATCCTGAAACCATACAGGAAGGGACCTCCATATTGTAATTTGATTAGGATACCACAATA
TGTCATCCAAATGACAATGACTAATGTAGCACCGTGCTCAAAATGACAATAGCCGCCACCCCTTCACTATTTCTCCCCCA
ACGGGAGTACATGGTTGTGTATTGTGGGAATACCTATGGGGCCTACGGGAGTTCATAAAGCCTTACAAATCACCACAGAA
GACCATTTAAAATCTGTGTTAACCCACCCTTGCAGTATACTGTAAGTATCCAAAGTACTCATACCCTCATCTGTGTCCAA
TTAAAATACTCTGTCTCCCTGAACCCTTCTTTCCACTGAGCACAACCTCTGGACCCGTATCCACTCTCCAGGCCCTGCCC
TGTGCCATTAGCTTTCATAGACGCATAATGAAAATGCTGGCAGGTCCCCTGTCCGCACCTCTCCCCATTTCTTCCCCCCA
CCACCCTCCTTCGTTGAGAGCAGAACCTATGGACCATACTTTCCCTCCATCTCTCTCTGCTCTGGGCCTAGCAGTATCAT
TAGCAGTCCTAGCTCACAGTGCTCCACCGTGGAGCCCCGGGGGCACAGCCTTGTGCCTTAATTACCTAACTAATGAGTCA
TTGTGAGAGGCCTGCTGGTGCCGTGCATTGGCCCGCCACTCGCTCAGAGGTAAACAAAAATGCACACTCTCTAAGACTTA
GGCACACCGACCCCCGGTAAGCACACAATCTCTCAAACACACACAAAGAGACAGACACGCACACACATGTACGCTTGATC
TCTCACACGCACACACACGGGCATTCACACCTTGTGCTCCGGCGAAGTGGGAGATAACCAGGAGACAAGCTGCTCCTGTT
CCCTTCTCAGATACAGTGTTGAAGATGACCACATTCTAATCATTTAAAACTGAACTACTGCCAGGGGTCATGTTCCAGAG
GGAGGAAGAGTCACTGCTAGCCTGCAAGATGTTGTTACAGGTTATGCAAGTCTTATGACAGCTTACACAGTGGCACCGTA
ACAGACTGCATCATGAGCAGAGTAGGCAGTCATCAAGAGACTGGACCCGTCCGAAGCAGGCATAATCGATGAAGAGCTTT
GGTTTAGTGTGTCGTTTGACTAGGCAGTGAGTCTCCAACAAGGACTGCTAAAATGCTCATTTAATTGTACTTTTGAAGTC
TAGACAGTGCATTTAAGCATCCATCTGTAGATCACCATGTACATATGCATCCCCTGCTACTTTCAATCTCAGCACAACCA
CACACAAGCCTATAAAAGAGCGGAAAAGCACAGAGTTAACCAAAATCTCCCTCTTTAAATGTCTAAAGACTGTATGTTGT
GACGGCTAAATGAAAAGGCGCTCGGCGACTTGATGGAGGGAAAACTACCTGTAATTTGTTCAGCCTGTAAAAAGGTTTAT
AAATAGGAAAAAAGGGCTCTCTGGAATAAAGTCTCTCCCTCCATCAGATTTTCTTGACAGGAAGTCTCCGAAGCAAGTTT
TCTGAGAAAGTAGATCCGTTGACAGCCCTGTTGCTCGGAGACGGGCAGCTCTTGAGAGACAAGCAGTGTTAAATGAGCAG
GCGTTTATCAATGCCCATATGAATCCATTGACGACATTCAGCGGTGCACCACAGCAGACCTGGGTTCAAATAGTATTCTG
AATCTTTCAAATACTTTAGCTATGCATGACAGAGGTTGCCTGGCCGCAATTGAACCAATCGAAAATGTTTAAAAAAAAGT
GAAAACCCTGCCCACCTGGCAATCCAGGCAGGATCAAGTAAACACTCAAGATCTCAAATACTATTTGAACCCAAGTCTGG
CTCCACAACAGTACATGAGTTTCTCCTTCCTCCCTTTAGGTTTGAGTCCATTTCCATAACATTTTCTGACATTAGGGTTC
CATTGAAAGAAGCCACACTTGAATAAGAGAAGTGTTCAACGCCAATTGATTGGAAGATCCAAAATTAGAATTGTAAAATA
TGGAGATTAGAATTTGACAAAGTAGCTTGTGATTAACATAAGGATTAGACCCCTTCAACAAGGCTAGATTTCCATTCCTT
ATTTACAGGCACCTTGTTCCTGGCATCAAATGCATTTCTGTTTACTCTCAAGTCCATAATCTTCTTTACTTCTGTTCTTG
GTTTATATAATGTATGAGAATGTTTTGGGGAGGGATACCAACGACCACAGCTCCAGCTGTGAGAACATTTCTTCTTAGGA
AAACATGGGGAATCTGTCTTAGTGAAAAACGAAATAGTTTTTATGTTATCCTCCAGGGTTCATCTATAATGCCTACCGTC
TGTGTTTGTACTAGATGTATAAATCACCATCACCATCACTATGACTATGGGGATCATATCTTTCCATATAGAGAGATATT
ATAAACCACAAGGCTGTATGGTAGGTCTGTCTGGGTACCTGTGGGTGGCTTAGGTTGTGTGTGTGTCTGTTTCTGTATTG
TACGTGGGTGGAAGACGGTACCCACATTGATAATGACATGCAAACAGCCCTAGGTCCCCAGCATTCCTCTGGGATCACCA
GTTTAAACATCTCATGTAGTGCCCTTGGTACGGAGTATGGAGAACCACCACAACTGCAACTCAATACCTAAAGCCTAGTT
AATTAAAAGGTCCAACATTTTAAATAATAACCCAATGGAGTAGAACACCTCAGTGCAGTAGTAGACAATGTAGTGGTATA
CCTCAGGGCTGAGTACAAGCCCCATCTCAAAGGCTGTGAACTCGAATATGTTTGATGATAAAATGTGGCAACGTAAACTA
TCAATGAGGTGTTACATGTTTTTCAGCTAAACGCAGGCCTCTTGAGAAACCACATGCAGTGCCTTCAGAAAGTATTCATA
CCTCTTGAATTATTCCACATTTTGTTGTGCTACAGCCTGAGTTCATAATGGATTACATTTATTTTTTCTCCCCCATTTAC
ATACACCCTATAATGACAAAGTGGAACATGTTTTTAGAAATGTTTGCAAATGTACAGAAAATAAAATACAGAAATCTAAT
TTACATAAATATACACACCCCTGAGTCAATACATGTTAGCATCGCCTTTGGCAGCGATTACAGCTGTGAGTCTTCTTGGG
TAATTCTAAGACATTTGCAAACCTAGATTGTACAATATTTTCATATTATTCTTTAAAAATGTCTTCAAGCTCTGTCAAAT
TGTTGAATGATCATTGCTAGACAGTCATTTTCAAGACTTGCCATATACTTTCAAGACGATTTTAAGTCAAAACTGTAACT
AGGCCACTCAGGAACATTCAATGTCATCTTGGTAAGCTAGTCCAGTGTATATTTGGCTTATGTTGTAGGTTATTGTCCTG
CTGAAAGGAGAATTTGTCTCCCAGTGTCTGTTGAAAAGCAGGCTAAACCAGGTTTTCCTCCAGTTCTTTTTATCCTAAAA
AAACTCCCTAGTCATTGCCGATGACAAACATACCCATGACATGATGCAGCTACCACCATGCTTGAAAATTTGAACAGTGG
TACTCGGTGATGTGTTTTGTTGGATTTGCCCCAAACATAACGATTTGTACTCAGAAATTGCCCCAAAACATTCTGTTTTA
CTTTCGTGTCTTATTGCAAACAGGATGCATGTTTTGGAATATTTTTATTCTGTACAGACTTCCTTCTTTTCACTCTGTAA
TTTAGGTTAGTATTGTGGAGTACTACAATGTTGTTGATCCATCCTCAGTTTTCTCCTATCACAGCAATTAAACTCTAACT
GTTTTAAAGTCACCATTGGCCTCATGGTGAAATCCCTGAGCGGTTTCCTTCCTCTCCAGCAACTGAGTTAGGAAGGATGC
CTGTATCTTAGTGACTGGGTGTATTGATACACTATACAAAGTGTAATTAATAACTTCACCATGCTCAAAGGGATGTTCAA
TGTCTGCTTTTTCTTACCCATCTACCAATAGTTGCCTTTCTTTGCGAGGCATTGGAAAACCTACTGGTCTTTGTGGTTGA
ATCTGTGTTTGAAATTCATTGCTCGACTGAGGGACCTTACAGATAATTGTATGTGCGGGGTACAGAGATGAGAGAGTCAT
TCAAAAAGCATGTTAAACACTATTATTACACACAGAGTGTGAGTCCATGCAAGTTATTATGTGACTTGTTAAGCTAATTT
TACTCCTGAACTTATTTAGACTTGCCATAACAAAGGGGTTGAATACTAATTTACTCAAGACATTTCAGCTTTTAATTTTT
AATACATTTGTAAAAATGTCTAAAAACATAATTCCACTATGAAATTATAGGGTATTGTGTGTAAGCCAGTGACACACAAT
CTCAATTTAGTCCATTTTAAATTCCGGCTGTAACACAACAAAATGTGGAAAAAGTCAAGGGGTGAGAATACTTTCTGAAG
GCACTAAGTGTTTTCACAACTGTATAGAGTAGATTATCCACTTCTATGTGCAGCCGGCATTGTAAACATGTGTCTGCACC
TACACACATGGTGTGAATCATCTCATTGTCTTACATAGCATTAATAAATAACTCTACTCTTCCTCATATTTTCCATAGAG
ATGCACTAGCATTTACCATAACAAAAGTTAGGGAGGCATATGGGAAACAAGAGGACCCCAAACTCTAACTGATTCCCCTG
ACTGAGTCTGAAGGGGCCAGTGGGATACGTCAAGAGACACTATCCTAAATTAATAAACAGATACTCTAATCATCCACACC
CTCTTCAGTAGTCATTAACTCCCATTACAAAAAGCCCTGTACTCACACCTCAGTCTCCCAGCAGATGGGAGCTCCACCAG
ACAACAGGTGGTAGATCGTCATGGAAACCATTCCCGGCACGATAATGATTGAACCCATGGAAACAATGCCTGGGACCTCG
CTAACAACTCAGACGTATGAGTGAAATACGAGACAGATCTGCAATAATTACGGAATGTGTCTATGATCTTTATTAGAATT
AATTGCCTACAGTGCCTTGCGAAACACCTCCATCTTTTGTTTCTAAATGACCAGAATATATTGCCATAGACATAGAGGAT

TATTATATATATATAATTGACGCTGTGATACCTACATACTTCACAAGCGATGTACTAAATTCTTGAAAGACAAGACCCGT
GTTCCCATGAATTATGAAATAAGTAAAATGCAGGAGAATGACTGAAAACATATGAATATATTACTAAAGAGAACAGATAG
AGACGACTAGTGATTGACTCAGTTTACACAGTATGAGAGACAGNNNNNNNNNNNNNNNNNNNNNNNNNNNNNNNNNNNNN
NNNNNNNNNNNNNNNNNNNNNNNNNNNNNNNNNNNNNNNNNNNNNNNNNNNNNNNNNNNNNNNNNNNNNNNNNNNNNNNN
NNNNNNNNNNNNNNNNNNNNNNNNNNNNNNNNNNNNNNNNNNNNNNNNNGATAGTATTTAAATAACAAAAAACCAAAATAC
ACACGTATTGGCACCTAGTTCTGGGGCGTGCTCGGGCACCCCCCCCCCCCCCCCATATCTACATCGATCTAATAAGATAT
AACTTAAAATCTGCTTTTGAAAAGTCCGATTTTTCATAACACGCCAGATCAGAGGAGTTCAAACAGCTCCAGAATGTCAA
CCACTGATTAGTGGTTGATGGGAGAGAATGGCTGTAGGATAAGCAGGAAGTAGCATGGCAGTCTCGAGCTGTGCATGTCA
ATTATTCGGTGACATTTTTGGAATGACAACTGGAACTCCACTAACTGAATGAGAATGAAGACGAATGAAAAACAACTGTA
CATGTTCAGATCTGTCATTTAAGTGCGCACATGCATACACTCTCGGTACATTCTGTATCTTGTCATTCCAATTTCAAGCT
TTCACAGAAAACCAATGCCTCATGCACCTTGAGATGAACCTTCAACCGAGACCTTTGAAAACAGAAATGTTCAAACCCAG
CTTTCAGGAAGTGAGGTCAAGATTCTCCAGAATGAGGACACTTCCTGTGAACAGTGAGCAACAACCCAGTGCCTTCTTTA
TAAAGTCCAAGAACAGCAATGAGAGGATACAATATATCCTCTTTTTACAGCAGACAAATATCATTGTTCCTCATTTGGGT
TGTGTGAAGGCTGGGTCAGAGGTGTTTGGTTAAATTTAGCTTGTTTACTCCTCTGCTATCTATGCCTAGATTGCATTTCA
ACACAATCAATACTGTCATATTGGATCAGAGCAACAGGATAATAAGACTCAATAAAGCTGTGAGGGGCGTATAGGGGGTA
TTGTATATCAGAGTAGCGGGAAGAGGATGGAAGCTAGGATAGAAAATGGGGTTGGTAAAAACAGGCATTCAAACACACAC
ATACAACATGCGCGTGCATACACACACACACACACACACACACACACACACACACACACACACACACACACACACACACA
CACACACACACATTGTTACAGTATATATACAAACACATAATGCACACAGACCCCACACTCAGAAATCCCCACACACCTCT
AGAGAGAGAATGAGAACTCTCCTGAAAGCTAAATAAGACAGGTTTATATCTGGGCTGCCAGTAAAACCAAATCATGAACA
GACTATTTGCCTCCACGAAGGAGAAGGAACATATTTGTATGTACACGTATTTCACATAGCAAATGATGTTCCGTTCACCA
CAGAAGATAGACCCTTAGACACAGGAAACAGTATTTGCAGAATAAGAACAATCCTCCTGTAGAATCAAGGCTAATCTCAA
ACATATTGCCTCAGACACAGGCTCAGATACAGGAGGTAGAGAGAGAGGGGGAGAGGAAGGAACTGTACAGAAATCTGCGT
TTCCTGTTGATGAAAACGTTGTCAGCAGTTGCTCTTGTTTGAAGCCTAATGATTAAGAACCTGCTGAGTTTTACAATGTC
AGACGTTTTAATGCTGCTCCAACACATTTTCTTTTAAAAACAATAGGCTGGTATTCAACTGCTTGAGTGGTCCAAAGCAA
CAGCAAGGAACATTCAGCACCAAATAGTAGCATAGTAGCAATCATCTGTTGCTGTATGGACACCTGTAAACCTATGCCTC
CTGTATCGAGCAACCTCAAACGGTATTTATATGTGACGTATTTACAGAATAGTTAAACATCCTACTTACAGTGTATGACA
TCCACTGAGTGTACAAAACATTATGAACACCTGCTCTTTCCATGACATAGACTGACCAGGTGAATGATATGATCCCTTAT
TGATGTCACTTGTGAAATCCACTTCAATCAGTGTAGATTAATGGGTGGTGACAGGTTAAAGAAGGATGTTTAAGCCTTGA
GAAAACTGAGACATGGATTGTGTATGTGTACCATTCAGACAGTGAATGGGCAAGACAAAATATTTAAGCGCCTATGAACG
GGCTATTGTAGTAGGTGCTGCCGGGTTTTTCAAGCTCAACAGTTTCCTGTGTGTATCAAGAGTGGTCCACCACCCAAAGG
ACATCCAGCCAACTTGACACAACTCTGGGAAGCATTTGCATCAACATCGGCCAGCATCCCTGTGGAAGGCTTTTGACACC
TTGTAGAGTCCATGCCCCAACGAATTGAGGCTATTCTGACAGAACAAGGGGGGTGCAACTCAATATTAGCAAGGTGTTCT
TAATGTTTTGTACACTCAGTGTATTTGGTTACTGATTTAACCTAGTAGGAGCTGTCTGCTATCCAAAGCCTTCCGTGGTT
TATTGATTGACAGAGTATGCTAATCTGACCAGTAACATCAGAGGTTTCCATCGCTATCACAACATCTACAGTTGAAGTCG
GAGGTTTACATAGACCTTAGCCAAATACATTTAAATTCAGTTTTTCACAATTCCTGACATTTAATCCTAGTAAAAATTGT
CTGTCTTAGGTCAGTCAGGATCACCACTTTATTTTAAGAATGTGAAATGTCAGAATAATAGTACAGATAATGATTTATTT
CAGCTTTTTATCTGTCATCACATTCCCAGTGAGTCAGACGTTTACAAACACTCAATTAGTATTTGGTAGCATTGCCTTTA
AATTGATTAACTTGGGTCAAATGTTTTGGGTAGCCTTCCACAAGCTTCCCACAATAAGTTGGGTGAATTTTGGCCCATTC
TTCCTGACAGAGCTGGTGTAATGGAGTCAGGTTTGTAGGCCTCCTTGCTCGCACATGCTTTTTCAGTTCTGCCAACAAAT
TGTCTATAGGATTGAGGTCAGGGCTTTGTGATGGCCACTCCAATACCTTGACTTTGTTCCCCTTAAGCCATTTTGCCACA
ACTTTGGAAGTATGCTTGGGGTCATTGTCGATTTGGAAGACCCATGTCAGGAAGCTTTAACTTCCTGACTGATGTCTTGA
GATGTTGCTTCAATATATCCACATCATTTTCCTCCCTCATGATGCCATCTATTTTGTGAAGTGCACCAGTCCCTCCTGCA
GCAAAGCACCTCCACAACACCCCCGTGCTTCACGGTTGGGATGGTGTTCTTCGGCTTGCAAGCCTCCCCCTTTTTCCTCC
AAACATAACGATGGTCATTATGGCCAAGCAGTTCTATATTTGTTTCATCAGACCAGAGGACATTTCTCCAAAAAGTACGA
TCTTTGTCCCCATATGCAGATGCAAACCATAGTCTGGCTTTTTTTTGGTGGTTTTGGAGCAGTGGCTTCTTCCTTGCTGA
GCGGCCTTTCAGGTTATGTCAATATAGGACTTGTTTTACTGTGGATATAGATACTTTGTACCTGTTTCCTCCAGCATCTT
CACAAGGTCCTTTGTTGTTGTTCTGGGATTGATTTGCACTTTTTGCACCAAGGTACGTTCATCTCTAGGAGACAGAGCAC
GTCTCCTTCCTGAGCGGTATGACGGCTGCGTGGTCCCATGGTGTTTATTCTTGTGTACAATTGTTTGTACAGATGAACGT
GGTACCTTCAGGCGTTTGGAAATTGCTCCCAAGGATGAACCGGACTTATGGAGGTCTACAAAATTTTTTTTCTGAGGTTT
TGGCTGATTTCTTTAGATTTTCCATGATGTCAAGCAAAGTGCCACTGAGTTTGAAGGTAGGCCTTGAAATACATCCAGAG
GTACACCTCCAATTGACTCAAATGATGTCAATTAGCCTATCCAAAGCTTCTAAAGCCAAGCCATGAATTTTCCAAGCTGT
TTAAAGGCACAGTCAACTTAGTGTATGTAAACTTCTGACCCACTGGAATTGTGATACAGTGAATTATAAGTGAAATATTC
TGTCTGTAAACAATTGTTGGAAAAATGACTTGTGTCATGCACAAAGTAGATGTCCTAACCGAATTGGCAAAACTATAGTT
TGTTAATAACAAGACATTGTGGAGTGGTTGAAAAACGAGTTTTAATGACTCCAACCTAAGCATATGTAAATTTCCAACTT
CAACTGGGGTGGCAGGTAGCCTAGTGGTTAGAGCTTTGGACTAGTAACCGGAAGGTTGCTAGATCAAATGCCCGAGGTGA
CAAGGTAAAAATCTGTGGTTCTGCCCCTCAACAAGGCATTTAACCCATTGTTCCTAGGCTGTCATTGTAAATACGAATTT
GTTCTTAACTGACTTGCCTAGTTAAATAAAAAAGTGTGCATGTCTTGGAATATAAAATACCCATAATCATTAGTAGGCTT
ACAGGCTCATTCATTTGTCATACCTCTAATGTTAGGATTAGGGGGTATAAGGTCATGGGATCCTAGATCTGTGGTTGGGG
TCAATATCTACCCTAAGTGTGTCTTTTCTGTTTAGGTCATATCTACATTATAAACAACATGTTGAATGGAATCGAGCATG
TTTCATCTGTGCCATCCAAACACCTCACCCATGCAGTGAGAATATAATGGAAATCTAAAGTAAAACCTGATGACCGTGAA
TTCACTTCAATGTCATTTCCCCGAGAACCGACCGCTGTTCAATTCTCCCTCTCGGAGTGGTGAACTGGGATAAATTCTGG
CTCCCCGCATCCAGGGGCTAATCTCCACTCAGAACACACTACGGTGTTCTAAAATTCCACTGACCTCTCATTTCCGTGCT
CCATATTCCCGCTTCCGATCAGAGCACAGGCGGAACACCGCTGCTTCTACACGTCAGTCACACGGAGCGGTGAGAAAAGC
TTATATTAACAAGAGGACTTCAGGCTCTGGGGGTTTTGCATATAAATAATGTGTCTGGAGCCAAGATGAAATCAAATGGT
GTGTGCGTGCGTGTGTGTGTCTGTCAGCTGGATTTGATTTCAAGGACAAAATCTCCGTAGCTATATGCTGTCTACTGGAA
ACTAACAAAGTATGTTTTCAATTTTTCATCCTCTTGACATTTTAAACATTTGAAATCACATTTTCGGAAATAAAATACGT
GCCAAGCACTCCAAGAACCTTTCTATGTGTGTACGGTGAGATGGTTTCTTTCCACACTGGTTAGCACAGCACTCAGTTTG
TCCTTTCCCTATGGGGCCATGGTGTTAATATCAAATAATTCTTGCCTCTTTGTTTCAAAGCCATTTTCGAAGGTAAATGA
AAGGTAGCAGCATGGCCATGTTCGTGAATGTGAGAAATAAACTTACATAAAACTTGGGGTTATAGCTTGCTAGCTAAGGC

TGCCTGGTATTTACAGTGGCCGTTTATTAAAAGCTCATGACACATTGCTCTTCATATCTTGGTCCGGTCTTTATGTGGCT
CAACATGGCCCCCTGTCATTTGGTAATTTAAGGGCGCTTTCACATATCCCCTTATGATCCTGATCCTGGAGCATTTGATA
CCCTGATCCGCGCTATAAAGCATGTGTGAAACGCAAACGAACCCTGGCTGAAATGAATCCTGGACCTGTGTGAGTAGCGG
GACCAAGATCCAGGACCAGAGTACCAGATATCGTGACACAGCATCTTGCTAATCGCACCCTGAAACTGGTATTTTCAAGT
CTTGTGAAAGCAAAACGTATTCTGTAGAATTGCTCCAGGAAACCGAACCAGGGTGCTCCAGGAAACCGAACCAGGGTACC
AAATTACATTTGTGAAAACACCCTAAAAGTAACTGGCAGTGCTATATAGTGGCTGTTTTAAAGATACATGGACATGATTG
GAAACAAATAAACACGAACATTTGTAATCTTCCCATGTCCTAATTTTTGATTAGTTGAAAGTATTTGGTTAGAGCTTGTT
GCTGAGACCAAAACACTTTTTAGAACAAGGAAGTATGAACCCGGATGCACAGAGGCCACATTTACATTACAAAGGCCTTA
TACCCAAGTAATTGGAATAAACAGGCTAGTTAGTATATACAACCATGAGGACGTATAGTGCACTTCTGTGATCATATTAA
ATGTTGTGTATTTTGTAGGAAACAGCACTAACATTTGCAGGAACTATGAAACTCCCTGTAGCCTAGTCACATGCCTGGTA
CAATTTAATTACATACAGTAAGTTATCCCCCAACAGTTACCCTAAAAAACTGCTTAGGTAATGTAAGGATTTTCAGACAG
ACCCAAACACTATAGGGACACAACTACACAGACTCAATATAGTCCTAGGAGACATAAACAGACACAGCTTTAAAAAAGAG
TGAGGCTGAGAACTGAGAAAGAGCGAGCGAGAGACAAACAGACACAGTGGGAGAGAGAGAGAGATAGCGTGGGGGGGGGA
GAGAGAGAGAGAGAGAGAGAGAGAGAGAGAGAGAGAGAGAGAGAGAGAGAGAGAGAGAGAGAGAGAGAGAGAGAGAGAGA
GAGAGAGAGACGCACGCATGCAGCCCCAGACACCCAGATGGTGTATCTTAGTACCCATTCTCTCCCCGTCAGATTGACTA
GCTAATGGAGTCTAATAAACTGTCACAGCCAACACACAGCACACTACATCTCCCAGCTTCTCCCGGCTCCAGAGGTCTGC
ATCTCCCACCAGCCCACCACCGTTCACACTGTTTGAATTGTAACTTCTCATGGAGGGAAATAAAGTGATGGCTATATTTA
TCCCCAGCTTCCATAGGTCTCTGCTGCTCGCCCCATATACCCGCCCCTGAGCCATGAGCAGAGCAGGTCTCAGGAAGAAA
CACCCCACCTTGCATCTCATCAACACCTCAAATTGCAACAAGAAATGGCTCTGTGGGTTGGGGGTGTGTGAGTAGGTGGG
AGAAGGCGATGGAGTCTATTATGCTGGAGGAATGTATTTCCATCTAACTAAGTGTGAGGCGGAGGGCAACATGTTCTGTG
GTTGGAGTGCATGTTGTGTTTGTCAGTTTAATTTCTGTTACTGAACTTGAACAATATGATGGACAATATGATAGTTAAGC
ATATCTTTGGTGTGTAAAAAGAGAGTTGGGAAACATTATAATTTGGTATTCTTACTGTAACAGTGATTAGTACATTTTTC
AAACGGCTTAGAAATAACCACTGAATTACATGTCTTACCATCTTTCCCTACGAGGAAGTTGACGTATTGGTATGTGTAGG
CTATGCCCCACCTTGGTCTCCACCTGTGGTCTCTTCAGGGTGGAGTAGATCTTCCCTGGACTGTTCTCCTCTGATGGCTTC
AGCTTCCTCAGTCCACAGCCCATCTCCCCAGAGTGTGTGGACCCTACATAGGGAATAGATGGGGCAGAATGAGATTCC
TGTGAGTGTGTGTGTGTGTGTGTGTGTGTGTGTGTGTGTGTGTGTGTGTGTGTGTGTGTGTGTGTGTGTGTGTGTGTGTG
TGTGTGTGTGTGTGTGTGTGTGTGTGTGTGTGTGTGTGTGTGTGTGTGTGTGATAATAATAAGGTGGGTTCATATATTTG
TCCTATTTCACACATGTACAAGTGTTTATTAATACACGTGTGAATTGGAAATTCGTTTTTTGCATATCCTAAATCCCCCT
GAGACACCCTCGAAGAGTGGGGTCACGGCCAGGGTCTGCCATCACCTTGTTGGCTCTGGGANNNNNNNNNNNNNNNNNNN
NNNNNNNNNNNNNNNNNNNNNNNNNNNNNNNNNNNNNNNNNNNNNNNNNNNNNNNNNNNNNNNNNNNNNNNNNNNNNNNN
NNNNNNNNNNNNNNNNNNNNNNNNNNNNNNNNNNNNNNNNNNNNNNNNNNNNNNNNNNNNNNNNNNNNNNNNNNNNNNNN
NNNNNNNNNNNNNNNNNNNNNNNNNNNNNNNNNNNNNNNNNNNNNNNNNNNNNNNNNNNNNNNNNNNNNNNNNNNNNNNN
NNNNNNNNNNNNNNNNNNNNNNNNNNNNNNNNNNNNNNNNNNNNNNNNNNNNNNNNNNNNNNNNNNNNNNNNNNNNNNNN
NNNNNNNNNNNNNNNNNNNNNNNNNNNNNNNNNNNNNNNNNNNNNNNNNNNNNNNNNNNNNNNNNNNNNNNNNNNNNNNN
NNNNNNNNNNNNNNNNNNNNNNNNNNNNNNNNNNNNNNNNNNNNNNNNNNNNNNNNNNNNNNNNNNNNNNNNNNNNNNNN
NNNNNNNNNNNNNNNNNNNNNNNNNNNNNNNNNNNNNNNNNNNNNNNNNNNNNNNNNNNNNNNNNNNNNNNNNNNNNNNN
NNNNNNNNNNNNNNNNNNNNNNNNNNNNNNNNNNNNNNNNNNNNNNNNNNNNNNNNNNNNNNNNNNNNNNNNNNNNNNNN
NNNNNNNNNNNNNNNNNNNNNNNNNNNNNNNNNNNNNNNNNNNNNNNNNNNNNNNNNNNNNNNNNNNNNNNNNNNNNNNN
NNNNNNNNNNNNNNNNNNNNNNNNNNNNNNNNNNNNNNNNNNNNNNNNNNNNNNNNNNNNNNNNNNNNNNNNNNNNNNNN
NNNNNNNNNNNNNNNNNNNNNNNNNNNNNNNNNNNNNNNNNNNNNNNNNNNNNNNNNNNNNNNNNNNGCCTCAGTTCCAA
AATCAAGAGCACACATTTCAAAGCCAAAAGAAACAAAAAAACAGATCTTCCATTTCCCTAAAAGTGTTCAATATCTCCTA
AACAGCCCCCCAGGACTGAACTCTAAAGCAACTCGGCTGAATGACATCAACATTTTTCTGTCATCAAAGAAAACAGGCGA
ATACTACAGTAAAATATCCATAGTTTTTAACATTATCTCAGATGTGCTCTGTCTAAGTTCCAGTGAACTACGGAGAGAGG
CTCCACAATGTAGTGTACAGGAAAGGCAGAAGAAGTCTGAACAAAAGGAGAAGAGAGAAAAACAAAAGGCTGAACATTTT
AGAAAAACGTAGGGGCTCTTATTCGTATAGCTCCTGGAGGCCAACGGGGGGGGGAGTGAGGCGAGGCCAGGGTATTTTTA
GAGTTGACAGAAGAAGTGTGAGATGCTTATGATTTTCAGAGTGACTGTTTTCCCTCTCAAACTTCTCCTTAGAATCGAGG
TGCATGTTCTGAGCATGCTCTGTCTGAGCTCTGCCTCTACGGGCCCACTTTGGTTAATCTCCTCTGCTTTTCAGACTGAC
AGCGAGGGAGTTTGGGTTACACCAACAGTCTAAACAACGGCTCGATATTGTCAGAAAAACAGAAAGGCATTGATTCTTTG
CTGAATTAGGAATGATCGGTTCACTGTTTCTTTCCTTCTTTTGTTTGGAGTTAATTGCATTTGAGGTTTTACTCGATAAG
ACTTTGTAAGTCAAGATGTTATAGGAGTCCAAAGGGTTTGTTGTAACTGGATGTACTTAAATAGGACGGATATACACTGA
ACAAAATATAAACGCAACATGCAACAATTTAAACGATTTTAGTTCATATAAAGGAAATCAGTCAATTTAAATAAATAAAT
TAGACCCTAAGCTGTGGATTTCACATGACTGGGAATACATATGCATCTGTTGGTCACAGATACCTTAATAAACAAGGTAG
GGGCGTGGATCAGAAAGCCAGTCAGTATCTGGTGTGACCACCATTTGCTTCATGCAACGCATCTCCTTCGCATTGAGTTG
ATCAGGCTGTTGATTGTGGTCTGTGGAGTGTTGTCCCACTCCTCTTCATATTGGCGGGGACTGGAACACGCTGTGGTAAA
CGTCGATCCAGAGCATCCCAAAAGTGCTCAATGGGTGACATGTCTGGTGAGTATGCAGGCCNNNNNNNNNNNNNNNNNNN
NNNNNNNNNNNNNNNNNNNNNNNNNNNNNNNNNNNNNNNNNNNNNNNNNNNNNNNNNNNNNNNNNNNNNNNNNNNNNNNN
NNNNNNNNNNNNNNNNNNNNNNNNNNNNNNNNNNNNNNNNNNNNNNNNNNNNNNNNNNNNNNNNNNNNNNNNNNNNNNNN
NNNNNNNNNNNNNNNNNNNNNNNNNNNNNNNNNNNNNNNNNNNNNNNNNNNNNNNNNNNNNNNNNNNNNNNNNNNNNNNN
NNNNNNNNNNNNNNNNNNNNNNNNNNNNNNNNNNNNNNNNNNNNNNNNNNNNNNNNNNNNNNNNNNNNNNNNNNNNNNNN
NNNNNNNNNNNNNNNNNNNNNNNNNNNNNNNNNNNNNNNNNNNNNNNNNNNNNNNNNNNNNNNNNNNNNNNNNNNNNNNN
NNNNNNNNNNNNNNNNNNNNNNNNNNNNNNNNNNNNNNNNNNNNNNNNNNNNNNNNNNNNNNNNNNNNNNNNNNNNNNNN
NNNNNNNNNNNNNNNNNNNNNNNNNNNNNNNNNNNNNNNNNNNNNNNNNNNNNNNNNNNNNNNNNNNNNNNNNNNNNNNN
NNNNNNNNNNNNNNNNNNNNNNNNNNNNNNNNNNNNNNNNNNNNNNNNNNNNNNNNNNNNNNNNNNNNNNNNNNNNNNNN
NNNNNNNNNNNNNNNNNNNNNNNNNNNNNNNNNNNNNNNNNNNNNNNNNNNNNNNNNNNNNNNNNNNNNNNNNNNNNNNN
NNNNNNNNNNNNNNNNNNNNNNNNNNNNNNNNNNNNNNNNNNNNNNNNNNNNNNNNNNNNNNNNNNNNNNNNNNNNNNNN
NNNNNNNNNNNNNNNNNNNNNNNNNNNNNNNNNNNNNNNNNNNNNNNNNNNNNNNNNNNNNNNNNNNNNNNNNNNNNNNN
NNNNNNNNNNNNNNNNNNNNNNNNNNNNNNNNNNNNNNNNNNNNNNNNNNNNNNNNNNNNNNNNNNNNNNNNNNNNNNNN
NNNNNNNNNNNNNNNNNNNNNNNNNNNNNNNNNNNNNNNNNNNNNNNNNNNNNNNNNNNNNNNNNNNNNNNNNNNNNNNN

NNNNNNNNNNNNNNNNNNNNNNNNNNNNNNNNNNNNNNNNNNNNNNNNNNNNNNNNNNNNNNNNNNNNNNNNNNNNNNNN
NNNNNNNNNNNNNNNNNNNNNNNNNNNNNNNNNNNNNNNNNNNNNNNNNNNNNNNNNNNNNNNNNNNNNNNNNNNNNNNN
NNNNNNNNNNNNNNNNNNNNNNNNNNNNNNNNNNNNNNNNNNNNNNNNNNNNNNNNNNNNNNNNNNNNNNNNNNNNNNNN
NNNNNNNNNNNNNNNNNNNNNNNNNNNNNNNNNNNNNNNNNNNNNNNNNNNNNNNNNNNNNNNNNNNNNNNNNNNNNNNN
NNNNNNNNNNNNNNNNNNNNNNNNNNNNNNNNNNNNNNNNNNNNNNNNNNNNNNNNNNNNNNNNNNNNNNNNNNNNNNNN
NNNNNNNNNNNNNNNNNNNNNNNNNNNNNNNNNNNNNNNNNNNNNNNNNNNNNNNNNNNNNNNNNNNNNNNNNNNNNNNN
NNNNNNNNNNNNNNNNNNNNNNNNNNNNNNNNNNNNNNNNNNNNNNNNNNNNNNNNNNNNNNNNNNNNNNNNNNNNNNNN
NNNNNNNNNNNNNNNNNNNNNNNNNNNNNNNNNNNNNNNNNNNNNNNNNNNNNNNNNNNNNNNNNNNNNNNNNNNNNNNN
NNNNNNNNNNNNNNNNNNNNNNNNNNNNNNNNNNNNNNNNNNNNNNNNNNNNNNNNNNNNNNNNNNNNNNNNNNNNNNNN
NNNNNNNNNNNNNNNNNNNNNNNNNNNNNNNNNNNNNNNNNNNNNNNNNNNNNNNNNNNNNNNNNNNNNNNNNNNNNNNN
NNNNNNNNNNNNNNNNNNNNNNNNNNNNNNNNNNNNNNNNNNNNNNNNNNNNNNNNNNNNNNNNNNNNNNNNNNNNNNNN
NNNNNNNNNNNNNNNNNNNNNNNNNNNNNNNNNNNNNNNNNNNNNNNNNNNNNNNNNNNNNNNNNNNNNNNNNNNNNNNN
NNNNNNNNNNNNNNNNNNNNNNNNNNNNNNNNNNNNNNNNNNNNNNNNNNNNNNNNNNNNNNNNNNNNNNNNNNNNNNNN
NNNNNNNNNNNNNNNNNNNNNNNNNNNNNNNNNNNNNNNNNNNNNNNNNNNNNNNNNNNNNNNNNNNNNNNNNNNNNNNN
NNNNNNNNNNNNNNNNNNNNNNNNNNNNNNNNNNNNNNNNNNNNNNNNNNNNNNNNNNNNNNNNNNNNNNNNNNNNNNNN
NNNNNNNNNNNNNNNNNNNNNNNNNNNNNNNNNNNNNNNNNNNNNNNNNNNNNNNNNNNNNNNNNNNNNNNNNNNNNNNN
NNNNNNNNNNNNNNNNNNNNNNNNNNNNNNNNNNNNNNNNNNNNNNNNNNNNNNNNNNNNNNNNNNNNNNNNNNNNNNNN
NNNNNNNNNNNNNNNNNNNNNNNNNNNNNNNNNNNNNNNNNNNNNNNNNNNNNNNNNNNNNNNNNNNNNNNNNNNNNNNN
NNNNNNNNNNNNNNNNNNNNNNNNNNNNNNNNNNNNNNNNNNNNNNNNNNNNNNNNNNNNNNNNNNNNNNNNNNNNNNNN
NNNNNNNNNNNNNNNNNNNNNNNNNNNNNNNNNNNNNNNNNNNNNNNNNNNNNNNNNNNNNNNNNNNNNNNNNNNNNNNN
NNNNNNNNNNNNNNNNNNNNNNNNNNNNNNNNNNNNNNNNNNNNNNNNNNNNNNNNNNNNNNNNNNNNNNNNNNNNNNNN
NNNNNNNNNNNNNNNNNNNNNNNNNNNNNNNNNNNNNNNNNNNNNNNNNNNNNNNNNNNNNNNNNNNNNNNNNNNNNNNN
NNNNNNNNNNNNNNNNNNNNNNNNNNNNNNNNNNNNNNNNNNNNNNNNNNNNNNNNNNNNNNNNNNNNNNNNNNNNNNNN
NNNNNNNNNNNNNNNNNNNNNNNNNNNNNNNNNNNNNNNNNNNNNNNNNNNNNNNNNNNNNNNNNTTCTCTTAATATCCA
TTCTAAGTACTTTAAGGCTATGCCAAAGGCTTGAATAAATTGGTCTCTTCAAAAGCCCATCACTAAAGAAAGAAAAAGGA
ACAATCTTTGCCACAGATCATGTACCCAAGCATTTCAAAGGACATAGATGTGATGCTTATGCAAGAGTATAATCCATTTA
GACTGTCATCGCTGTCAGGAGAAGTGCAATAAATTACCCGTCATCTTCCAGCAAGTAATCAGGCTAAGCTGTTTACCCAA
AAGGCCTAAAATACGGTGGGTAGGTACTTGTAGGGAGGCAAATAGGGATAAATACAGTATGTGTGGAATTGTGTGTGTGT
GTAGAATCTTTCAACTTGTGGCTAGGATGTGGGGATTGAGCTTGGGCCAATTCCGGTGTGAGTTATCTGGCCCAAATGTA
TATAATACTTGGGGCTCGGGCCGATTCCGATGTGAGTTATCTGGGCCAAATATATTACTTGAGGCTTGGGCCGATTGGGG
CTACTCTCTGGCAGAGGATTACACGGGCTGCTTTATATAATTAACATTTCATTATTTATTTTTCCATATTTTCTCATTGT
GTCTGTGATCAAAAAAATACAATTAACAATTAAATGAAATTCTTTAAGAGTCCTGTGTAGTATTTTAGTATTTTGATACT
TTGTGCATTAAAATCTTTGTGGCTGGAGCCTCCTGAGTGGCACAGCAGTCTAAGACACTGCATCGCAGTGCAAACTGTGT
TGCTACAGATGCTGGTTCGAGACCCGTGCCGGCTGCGGCCGGGAGACCCATGAGGCGATGAACAATTGAGTTAGGGGAGG
GTTTGGCCGGCCGGGATGTCCTTGTCCCATCGCGCTGTAGCGACTCCTGTGGTCGGCCAGGCACATGCATGCTGAAACGG
TCACCTGGTGTATGGTGTTTCCTCCGACACAAAATGTTTTTTTGTGGATGGGTATAATTTGTATGTATAAAATGTATAAT
AGTAATATTTCAAATGACTAAATCGGGTGATTTTGTATACATTTATTTAAATTTGCATCGGGCGGCTTCTGGGGAGATTC
TGGTAAGATGCCGGCAAGGTCAGCCGAATTCCGGTAAAAGGAAACGGCCCGATGGTACTTGGGCCAATTCTGGACAGTCA
TTAATTTTGATTCCAGGCCGAGTCCGACACCCGATTCCAGACGATTCAATCAGTTCCAGACCCCAGGAAGAGGGCTGCTT
CTGGGCCGATTCCTCATTGCTAGCTGGGATACTGTAAAATGCAGTAAACTTGTAGACTAGTGGTCAACTTTCTCCTACTC
CCTCTGTGAATGTTTGCACTGTACATGCTGAGGAGAGTAGAACATCAATTAAGGTTTAAAATCAAGCAAGACTTGCCAAT
TCTATTCAACCCAACTCAATTCAAATTCAACTCAATACCAGATAAATCTCTACACCCTGGTCCAGTAGCAAACATATGGG
AACTGGATTAAAATGGAAATTTGAACGTCAACACAGCTGGACGACAGAAGTCAATGTTCAGAAAATCTGTGAACATGATC
TACTTTATGAAACACACAATAGTGAGTTCCTACTATGTAGGCCTTATAGATTATATCTGGCGTGTATTAAATTTAACAAC
GTAATTATGTCATGAAAGTGTTTTTGCTCCGGCTCCCTCGCTTCACCTTTTGCCCCATCTATTATTCTCTGTTTCAAATG
AGGCCTACAGTGTGGAGCAGATACCACAATGACTTTCCCATTCAGGGCTGGCCAGTTATATTTGGGTTGTGGCTAGTCTG
TGATGCAGTCATTTGGGGGTTCCTGGTGAAAAGTGTTTCCCTGTGTGTGTGTGTGTGTGTGTGTGTGTGTGTGTGTGTGT
GTGTGTGTGTGTGTGTGTGTGTGTGTGTGTGTGTGTGTGTGTGTGTGTGTGCTAGCTCGTGACCCAGCAACAATAAAA
GGGTTAACAGCCCAGTCTTTACAACCAACCAGCTGGAACTGATTAACAGTTCACTCAAACTCTCTCTCTCTTCCTCACTC
ACTCCCTAACGCTCTCTCTCATTCCTTCTCACTCTCATTCTTCCTGTCTGTGTGCATTCCTCTCTCATGCCCTCTTCTCC
TTTCTCCCAACCTCTTATCCCTTCTCACATCCTTCAACTCATTCACTCTACTTTGTCCCTCCTCGCTCTTTCTTTCTCTC
ACACTGGCAAACACACACACACACTCTCTCTCTGCAGTAGAGGCTAAGGTTTAATACTGTATGTCAAGCACATCTTGAAA
TATCATCAGGGTTCAGTGATTGGAGCCAGGTAGACATTTCTGGCATTCTCACCCCTCTGGTACCACACTTCCAGTTCTCT
GCTGCCAATGACTGGAACGAATTGCAAAAATCACTGAAGCTGGAGTCTTATATCTCCCTCAGTAACTTTAAGCATCAGCT
GTCAGAGCAGCTTACCAATCATTGCACCTGTACATAGCCCATCTGTAAATAGCCCACCCAACTACCTCATCCCCATATTG
TTATTTATTTTTTTGCTCCTTTGCACCCCAGTATCTCTACTTGCACCCCAGTATCTCTACTCTACTTCCAGTGTTAATGT
TAAATTGTAATTATTTCATGACTATGGCTTATTTATTGCCTAACCTCGCTAATCTTACTAAATTTGCACACACTGTATAT
AGATTTTTCTATTGTGTTATTGACTGTACGTTTGTTTATCCCATGTGTAACTCTGTGTTGTTTGTGTCGCACTGCTTTGC
TTTATCTTAGCCAGGTCGCAGTTGTAAATGAGAACTTGTTCTCAACTGGCCTACCTGGTTAAATAAAGGTGAATCAAAAT
TTAAAAAATACATTACATTTTTTGAACTTCAAACCAACCAGCTCATTGATCTGCATGATTACCGCTCTGAGCATGACCAG
GTGTTTGTGTGTGTGTGTGTGTGTGTGTGTGTGTGTGTGTGTGTGTGTGTGTGTGTGTGTGTGTGTGTGTGTGTGTGTGT
GTGTGTGTGTGTGTGTGTGTGTGTGTGAGTGTGGGTTGGTGATCAGGAGTTTCTTGTAAGACATTGCACCCAAGCCAAAC
TTGTGGCTTGCGGCATCCCTTGACTGATGCATTCATCCTCATTAATCTGCCTCTGAACGGTTGTTATGCTATAAACAACT
AGAACACTAAGTTGTCTGTGGCTTCTGTCCAAAGAGATGTAAAGTCTGTCTTTATCAGTGTCTTTTCCTGTAGGCTACAT
ATTTATTTTCTCAAGCCTTGAGAAATAAGGTTGTAGACTAGTGGTAAACACATGCAAGTTAAAGTATAAAGAAAAGGGAG
TTACCTGATCAGAAGCTGTTGACTACAGGAGTTGGAAAGTTCACTGTTGAGATCAAGAGAAAGCCACTGGAACGGATCCT
CGAACAAACACACACATTCCACTGACAGGAGAACAGCACCGCGCAACAGTCCACGCTCAGATCCTCTGCGATTCTTCACT
CAGGTCCTGTCGATACAGCTCGAACTGAGGCTTTCGGTCAAAGAGGGAAAAAAAGACAGCAAGAAGACTCAAATCTGGAT
GGGATCAGTAGCTCTGCCAGCTTGTAATATCCTCGCCAGAGGCTCACTAGCTAACGGTGCGCAGCAAACACTTTGAGTGC

GTGCGTTTAGGTCATGCTCAAAGTATGCCTCGGCTATGTTCTGCAAGCTCAGGGAAACCACATCCTGTCACATATCTACC
TCCTCTCTCTCTCTTTCTCTCTTTCTCTCTTGTGATTGTCTGAAGCGATGGAGCGCTCTGACCCAACCTCTGTTCGGCGC
AGCCCCTCCACCCCTCTATCTTTCCCTCTCCCTCTCTTTCACTCACTCAAGGCTTCTCCGGGAGCCGCACCAGTTCCAGT
CGACTGACTGAAGAATCCATCTGAGGATGAGGCTGGGGGAAAGAGGGAGGGCGAGAGGGAAGGGGGGGGGGGGAATGCCA
CAGAGAGAGAACGAAATGAGGAGAGGTGAGGAAGCAAGAAAGGGTGTACAAGGAGTACAAGTGTAGAAAGAGCTAGGGAG
ACAGAAGGATGGAAAATAAAAATAGTAATTTCAAGGAGAGATATAATGTATTATACATACTAGTATATTCCTTGAGAATT
TGGTGGGTAAGTTGTTGTGTCAGCACTTCTGTGGTGACTATACATATTTAATACGTTCCTGTCGAGGCCCCCTATAGCTG
CATATTCATTATTCACACCAGTCTGTGAGAGGGACCACTGCTCTGGAGATAGTTGACTGACACAGAAAACAGTGATTTTC
ACAGAACCGAACATTGAAGCAAAACAACACGGGAAGGCGATTGCAACGTAACACGGAAGTTATTCTTTATGCAGGCGCAG
TGTGTCACGACTTCCGCCGAAGTCGGTCCCTCTCCTTGTTCGGGCGGCGTTCGGCGGTCGACGTCACCGGCTTTCTAGCC
ACCGCCGATCCACTTTTCATTTTCCATTTGTTCTGTCTTTGTCTTACACACCTGGCTTCACTCAACCAATTACTTGTTTA
TTATTTAACCCTCTGTTCCTCATGTTGTGTTTGTGAGTGATTGTTTATTTGTATTCAGTCCGTCTTTGTGGACTCATATG
TTACTTTGTATATTTGCCTTTTTTGAGTAAAGTACATTTATTACTCAACTCTGCTGTCCTGCGCCTGACTCTCTACACCA
GCTACACACAGGACCCTTACAGAATCACACACCCCATAGAATGGAGTCAGCAGGAGCAGGTTCCCTCCCTGTTCCAGTGG
AGGAGCGCGTTCAGCAGCACGCGACCATGTTGCAATGTCTGGATACCGCCATGGATCACGTGCTGCAGACGATGGATCGT
TGGGAGAGAGGAGGTTTCCCAGCACCTCCACCAGCCCCACTACAGCCGGGCCGGCCCACTGTCCACCCCTCTTTCACCCA
GTCCCAGCGGGATTCGACTCGCGCTCCCAAGGGAGTATGATGGGACGGCTGCCGGATGCCAGGGGTTTCTACTCCAGCTG
GAGCTCTACCTGGCGACCGTCCACCCGGCTCCTTCGGGGCGTGAGAGCGTGTCCGCCCTCGTCTCCTGCCTCTCAGGCAA
AGCCCTGGAGTGGGCCAACGCCGTACGGAGTGAGGGAGACGCGGAGTTGGACCATTACGCAGAGTTCACCCGTCGCTTTC
GGGCAGTGTTCGACCACCCGCCTGAGGGTCGAGCGGTGGGTGAACGTCTGTTCCACCTGAGGCAGGGGACGAGGAGTGCT
CAGGATTTCGCATTGGACTTCCGGACCCTGAAACGACAGGGCCTTGATCGATCACTACCGGTGCAGTCTGCGCGAGGATG
TCCGTCGGGAGTTGGCCTGTCGAGACAGCACCGTCACATTGGACCAGCTGGTGGACCTGTCCATCCGACTGGACAACCTG
CTGACTGCCCACGGCCTGTCAGTTCCATCCCCCAGCACCTCCACTCCGATGCCCATGGAGTTGAGAGGTGCTGCACTTAG
GGCGAAAGGAGGAGGGGCCAATCCCTGCACCATCTGTGGCCGCAGAGGGCACACTGCTGGTCAGTGCTGGCGGGGTTCCT
CAGGGAGTCGAGGCAGCAGGCAGTGCACTATCGTGTCACCCCAGGTGAGTCGGCACCAGGCTCACCCAGAGCCCCCTGTT
GCTCAAATGTATGTGTTTATTTCATTTCCTGAGTTTTCCCCGCATTCCCAGCATAAGGCACTAGTAGATTCAGGCGCAGC
TGGGAATTTTATTGACCGTTCATTTGCCCATAGTTTAGGGATCCCCCTTGTTCCTGTAGATATGCCCTTCCCTGTGCACG
CCCTAGATAGTCGACCATTCGGGTCAGGGCTGATTAGGGAGGCCACCGCTCCACTAGACATAGTTACGCGGGAGGGTCAT
AAGGAGAGAATCAGTCTCTTCCTTATTGATTCTCCTGCGTTTCCCATGGTGCTGGGCTTACCCTGGTTGGCCTGTCATGA
CCCTACTATTTCGTGGCAACAGAGGGTTCTCAAGGGGTGGTCACGAGAGTGCTCAGGGAGGTGTGTGGGGGGTTCCATCG
GTGCGACTACGGTGGAAAGTCCAGACCAGGTCTCCACCGTGCGCGTCCCCTCAGAATATGCCGATTTGGCTCTCGCCTTC
TGTAAGAAGAGGGCGACTAAATTACCACCTCATCGATGGGGGGATTGTGCGATAAATCTCCTGGTAGACGCAGCACTTCC
CAGGAGTCACGTGTATCCTCTGTCACAGGAGGAGATGGTGGCTATGGAAACATATGTCTCCGAATCCCTGGGGCAGGGAT
ACATTCGGCCCTCCCCTCCACCTGCTTCCTCGAGTTTCTTTTTTGTGAAGAAGAAGGATGGGGCTCTGCGCCCGTGTATT
GACTATCAAGGTATCAATCAGATCACTGTGAGGTACAGCTACCCGCTGCTGTCCGCGACCGTTTGATCTATTGGGCCCAT
GTGTCACCCTCCTCTGGTCATCCTGGCAATGGTCGGACGGTGCATTGCCTTAGTAGGAAGTACTGGTGGCACACCTTGGC
CAAGGACGTGAGGGTTTATGTTTCCTCCTGCTCGGTGTGCACCCAGTGCAAGGCTCCCAGGCACCTGCCCAGAGGGAAAT
TACAACCCCTACCCGTTCCACAATGGCCGTGGTCGCACCGGTCGGTGGACTTCCTTATGGATCTTCCTCCCTCATGGGGC
AACACCATGATCCCGGTTGTTGTGGATCAGTTTTCTAAGTCCTGCCGTCTCCTCCCTTTGTCCGGTCTCCCTACGGCCCT
GCAGACTGTGGAGGCCCTGTTCACACACGTCTTCCGGCACTATGGGGTGCCTGAGGACATAGTTTCTGATCGGGGTCCCC
AGTTCACGTCCAGGGTCTGGAGAGCATTCATGGAATGTCTGGGGGTCTCGGGCAGCCTCACCTCAGGTTTCCACCCCGAG
AGTAACGGGCAGGTGGAGAGAGTAAACCAGGATGTGGGTAGGTTTCTGCGGTCATATTGCCAGGACCGGCCGGGGGAGTG
GGCGGCGTTCATCCCCTGGGCAGAGATGGCCCAAAACTCACTCCGCCACTCCTCCACTAACTTCTCTCCTTTCCAGTGCG
TACTGGGGTATCAGCCGGTTCTGGCACCTTGGAATCAGAGCCAGATCGAAGCTGCTGCGGTGGACAAATGGTTTAAGCGC
TCGGAGGAGACCTGGGACGCCGCCCATGTGCACCTACAATGGGACGTGAGGCGGCAAAAAGCAAGCGCCGACTGCCACCG
CAGCGAGGCCCCGGTGTTCGCACCGGGGGATCGGGTCTGGCTCTCGACCCGAAACCTGCCCCTCCGCCTGCCCTGCCGGA
AGCTGGGTCCGTGGTTTGTGGGGCCATTTAAAGTCCTGAGGAGGCTGAACGAGGTATGCTACAGGTTACATCTTCCCTCA
GATTATCATATTAATCCCTCGTTCCATATGTCTCTCCTCAGGCTGGTGGTGGCTGGTCCACTCCAGGAAGCTCAGGTGCG
GGAGGTTCCTCCACCCCCTCTGAACATCGAGGGGGCCCCGGCATATGCTGTTCGAGCCATCCTGGACTCGAGGCGTCTGG
CGAGGGGCCTTCAGTACCTCGTGGAGTGGGAGGGGTACGGTCCGGAGGAGATTGCTGGGTGCCGGTGGAGGACGTCCTGG
ACCCTTCGTTGCTGCGGGATTTCCACCGTCTCCATCCGGATCGCCCTGCGCCTCATCCTCCGGGGCGTCCCCGAGGTCGG
TGTCGGCGCGCTGCTGGAGCCGCACGTCAAGGGGGGGTACTGTCACGACTTCCTCCGAAGTCGGTCTCTCTCTTTGTTCG
GGTGGCGTTCGGCGGTCGACGTCACCGGCTTTCTAGCCACCGCCGATCCACTTTTCATTTTCCATTTGTTCTGTCTTTGT
CTTACACACCTGGCTTCACTCAACCAATTACTTGTTTATTATTTAACCCGCTGTTCCTCATGTTGTGTTTGTGAGTGATT
GTTTATTTGTATTCGGTATGTCTTTGTGGGCTCATATATTACTTTGTATATTTGCTTTTTTTGAGTAAAGTACGTTGATT
ACTCAACTCTGCTGTCCTGCGCCTGACTCTCTACACCAGCTACACACAGCACCCTTACACAGTGATGGAAAAAAGTACCC
AATTGTCATACTTGAGTAAAAGTAAAGTTACCTTAATAGAAAATGAAAAAGTGAAAGTCACCCAGTAAAATATTACTTGA
GTAAAATTCTAAATTAATTTGGTTTTAAATATTCTTAAGTATCAAAATTAAATGTAATTGCTAAAATATCCTTAAGTATC
AAAAGTAAAAGTATAAATCATTTAAAATTCTTTATATTAAGCAAACCAGATGGAACAATGTGTTTTTTAAATTTACAGAT
AGCCAGATACTCAACAATGCTCACTTTGCAAACAAAGCATTTGTGTTCAGTGAGTCCACCAGATCAGTGGCAGTAGAGAT
GACCAGTGGTGTGAATTTGACAATTTTCCTGTCCTGCTAAGCATTCAAAATGTAACGAGTACTTTTAGGTGTCAGGGAAA
ATGTATGGGGTAAAAAGTGTATACTTTTACTTCACTTCTAAAGAAAATTAAAAGTTGTCCAAAATATATATATTAAAGTA
CAGATACCCTCAAAAACGACTTAACGTAGTACTTTAAAGCATTTTTCTTAAGTACTTTACACCACTGTGCAGTTGGATGG
GACAGATCAATTATGGGACATCAGCATTTGGTCTGCTGTGTACATGTGGGCTCTTGCATCATTGAGGATACTATTCTCTC
CTGGAGCAGCAAAAACATTGCTGGACAGTTCAGATATTGCCGCCAGACTTGGCAACACACTGTTGTCTGAGTATTTCAGA
TGGAACCAATGCTTTTCCAATGGGAGTCATTTGTTAATGAATGAGATGTGCCTCCATTAAAAGACAGACCATCTCTCGAA
AATAGTTCAGTTGAGTTGTACTTTTGGACAATGATGTAGCTCATATACGATTCCATCCATTTTCGTGCATCTCATTGTGT
CTCGGGCTTAATTGCTGAAATGAAGGACCAGAGGCAATGCCTTGAGAAAGATGCCATTGCCCTGGAGAAGTCAGTAGACA
AGGGGAGCTTACATTGAAGGCAGAAATCACAAGGAATCTCAGTTTATCGCAAGTGAGTCATCACATTTGAGCTAACAAAT

TGGCTGAGAAGCATTCCTTTGTCCAACTAGATGACAAATCGGAGTGCAAATAAAGTTGAGTTTGGCATTCAAGATTCTGT
TCTAAAATGTCCTTTGCGTTACTCATTGGAAATGTTATCAATAAACATATGCATTTTCCTTGCGCTGCATGCTGTGCAGA
TGATAACCTCACGATGCTCTTGGTCATAAGAAGCTTATGAACATGTCAGAACTCTCTGAATTGTTGTTAAAAATGGCTTG
TGTTACTTTTGATCATGGCCTGACATTTGAAAATCACATGAAAAGCATATCCATGACAGCATACTTCCATCCCAAGGAAC
ATTTCTAGAAATACACTATATACATCTCACAGCCTTTCTGGACAAACATGATCATACAACATCATAGTTATGCTTAGAAC
ATTGTATGAGCTTCTGAAAGCATTATCTGGAAAGCCACTCTGCAATTGAAAGAATCCTGGCCTCAGCCCATTTCCTTGAA
CAATAGGGCAGTTGTATGCTCAAAACAGGGCACTTTCCTCCAAGGGCATACACTTCTTCCAGTGGTAGTGGGCCTCAGTC
AGAAGGCAACCATTTCTTCGGGCCACAAATAATGATGCCTCTCTGTTGAAAGCTTGAGATTCTCTCTTCTACTACACAAC
AGGATCCCTATTAGAGTTCAAACTTACTCCCTGAAAGCAAGAATAGGAACTGCCAACACACATCTAGTTCTGGGCTACAC
GCATCCAATTTAATATGGCATATTTAGCATTTGAGTACATTTTACAGGCAAAGGTGACATTGGATGAAAAACTGTTCCAG
GAGTACTACTCTGCCCTTGGAGAAGATTTCAAGCATCATGTTATACGCAACACTGATGGTAAAGAATGGGTAGCTGTTTT
CCCGCTTGTTATAAGTTACAACTGAAATCAAAAGCTGCCAGTCTGCCAGAATAGCTCCTTTGCTGAAGCCTGAAGAACAG
TAATGATCATCCACAGTGTAATATCCACCATGTTTAGTCGCGGGTTATCAGTTTATAATCTGTCATAAAGAATCATGTTG
AGAACAAAGGACATTTACATGATGTCATCAGACTGAATGCTTGGTTGGTGTTATTAGGTTTATACTTGACTGTAACCCTA
AGATGACCCTAAAGGAATGCACCACTGGTTTTACTGACATTTTCCCACTGATACAAGCTCAGAAACCATTCAAATGGGAT
AGCAGCACCTAGCTTTAGCCGTAGCTTTGTGCACGCTGGTGTATCATACAAGAGTCATACTCAAAATAAGTCTAAAGAAA
TTGCTTAGATGGAACTTTGTGTCATTGTAGCATGACTGTAAACACTGCCAAATCTGGAGAAATTGAGTTGAGATTAATAA
ACATGACCAAATACATTTCCAGATTCACATGTTCAATTTTCAAATTATTGAGCTAACTTATGTGCTAAAATGTAACAGTT
TATTTGTTAGATTTTAGACAGAGAAACATAATGATGAGAACTGCAAAGAAACATTTGGAACATATTGGAAATTCAACCCG
GGTTTATTGACTGACAGTACTTTAACAAAAACAAAGGAATTCGTAAGACTTTATCTTGCACAATACAGGTTTAGTGGACC
CACTTATCGTACGGGATGCCTTTAGAGGCCATTCCATTAAATATTCATCCCCCCCCCCCCAAAAAAATGGTACTGGTCAAT
GGAGATAAGACTTACAACAGAATAGAACACCTAACATTACACATCAATGGTATTGATAAATGTACTAAAGAAGCACTGAA
TAATCTAGACACAAAACAAAATAAGTTTGAGGAACTTAATCAGGAAAGATCAGGCTTCACTTATCTAAAAAAACTGAACA
AACTGGATGGAGAACGACAAAAAATTCCTAAACCTTCAATATATAAACGCAACAAAAGAATTACCTTACAGAAATACTTC
TCACCAAAGGAGATTTTGAATGAGGAAGTAAAATATTGTAAATAAATGTTCTATTGTCAAGTTCCTCCAACCTCACTTCA
CGATGAGTTGTGATATCCTTCTACCAATTAAAAAAAACTATATGACAAAAAACAAGATTCTTGTGAAGGTCTAATTACAC
AAAATGAATTATCGATGGCAATTGATTCCTTTCAGATGGGGAAAAGCCCTAGCCTTGACATTCCATAAGCGATATACACA
TTTTATGAACTACTGAAAGATCCATTACTATCATGTTTTAAATTACTTGTATATAAATGGCAAACTGTCAGACACACAAA
AAGAGGGACGGATCTCTCTCCTACTGAAGCAGGAAGCAGGGGGGCAGTGCAAAGTACAAAATTGGAGGCCCCTCACCCTT
CAATGTTGCAACACAAAAATATTGGCAAAATGTATTGCTCAGAATTTTTAAATAATCATCAGTCGGGATTCTTAAAAAAT
TATTGAAAAAATTATTGAAAATTAAGAAAATGCAGGGAAACCAGGTGTAATCTTTATAGAAGATTTTGAGAAAGCTTTTG
ATACAGTAAGTCCAGAATCTGTTTATAAGTGCCTGCATTTTTTTTACTTGAGAAACTCTTATATGCAGGGTAAAAGTAAT
GTACAACAATCCTTTGTGTAAAACTAAGTTATTTCTCTGAGAACTTTGAATTGTCAAGAGGAGTAAAACAAGGAAGTCCT
CTGTCACCATTCTTATTTATTATGATTATTGAACTGTTAGCTCTAAAAATCGGAGGTAATGATAACATTAAGGGGCTCAA
AATTTATGGATTAGAGAATACACAATGAAATATTAAGCCATTAAACCTCTCAAAACCCCCATTATGCCGAAATTATATCT
AAATCCAAACTGGTTTTCCTAGTAGGCTACTCAGGCTACGTCTACACAGACAGCCCAATTCTGATATATATTTCTTGCAA
GAATTCGTCCTTTGACCAATGACATCGAACTTTCCCACCAGATCTTTTTCAGAGCTGATCTGATTGGTCAAAATACCAAT
TAGTGAAAAAAAGATCCAAATTGGGCTGCCTGTGTAAAAGTAGCCTAAGAGAGGCACAACCAACGTTCAAAGGGGGGCCT
TTTGCCATAATACAGATTAAAACCAACCATTTCTGTCACATCTGCTCCCGCTCTTCCCTCCCCTGGCGCTTGAGGGCGCC
AGGCTGCCCTGCATCACTCACTCCTATCATCTATTACGCACACCTGCCTTCCCTCGTCACGCGCATCAGCAATATTGGAC
TCACCTGGACTCACTCGTCATCTGTTTATTTCCTCCCCTATATTTGTCAGTTCCCTTGCTCTGTTCCCTGCTGCTGCATT
AATTGTTTTTTGTCTGTGTTACCTGTTTGCTGACGCTGTTACTGTCTCCTTCCATGTCCGGTCTATATTAAATGTTTTAC
TCCCCGTACCTGCTTCGTCTCTCCAGCGTCATCCGATGTGACAATTTCAGGTGGCTTGACAATGGAACCCTATTTAAAGT
GTCCTCTTTTTTAAATGAAGCCATACAGAGTTGGTTACAATTCCAATTTCATCCTCCAGAAGAGGCAGAACAAATATTAC
AGCTTAACTCCAATATACTGACAGAGGAAAGACCCATTTATTGGAAAAAAATGTATAAGGAAGGTATCATTATGAATGAC
ATTGTAAATAAGGACTGTAAAATTGTCACAATCACAATTAACAAAGGTGTATGGAAATGTACAGTATGTTCAATACAAAA
GTATAATAACTCATCACATCTCTGCCACAAAAATGGAAGAGGCAATTACTCAAAAGAGAAAGGAATTAATTGGTTTGTCT
GCCTTCAATGAAAAACCATGCATTGCTTAAAATGAATAGTATAAAGTATAAAATGTATCAGTTTCACTTAGTTTGACAAT
TGTAGAGTACTGGGAAAGATGGCTAAGCACGTGGACATCTGAGGGTTGGAATTAGAGTGTACAGTATATGTGAGTCAAAA
TAGTTTGAAGTAGCAGTAGAAGTTTACTCCAATCAGGGAAGGGAGGGTATGATAAAAAGTACACTCCCCTCCATATGTAT
TTCGATAGCGAATACTTTTTTGGTATTTTATTTGTTTTACATTTGGCTCTACACTCCAGCATATTGGATTTGAAGTAAAC
GTTTACGAAAATATCCTCAAATAAAAGGTGGCATTCTGTACTGTCGCCTCATATGAAAACATCTGTTTTACCGTTTAGGA
ATGAAAGCACTTTATGCATCTAGCCCCCAATTTGAAGCCAAGTACTGTATTTGGACAAATTCCCTTAGTGTATTAAAATA
GTCCAAAATTATTTAGTCCCATATTCCTTGCAATCAAGATTGTGACTTTACATTTGCAGTTTGTTTTGGTTGCATTTCGA
GTAAAACTTAGCCCAAAAGGAACTGAATGGTGAATAATGTATTGTCATTTTGGAGTCACTTTTATTGTAAGTACGAATAT
AAGATATTTCTGAACACTTCTACATTACTGTGGATGCTACCATGATTATGGATAATCATCAATGAATAGTTAATAATTAT
GTGAGGATACAGAGGCACAAAGACCCCCCCCCCCCCCCCCCCCCCAGAGGCAATATGAGAAGTTAGCATGTTTTGTTATAG
CCTCTAACCTTTTCACTCACCATTATTCATTCAAAATAAATAATGACATTATCAGGATTCATTCAGAAGTTTTCAGGGAC
TAGATAAAGTGCTTTCATTTCTGAACTGTAAAACAGATATGCATGAAAATACCCTCAAAAAGGTGACACCCTGTTCTGTT
GCCTCATGAAAACATTTGATCTAAAATCCAAAATGCAGAGCCACATTATACATTTCTGCTTCACTGTCCAAATACATATG
GAGAAGAGTGTAGCTGCCTCATCCTTTTAAGAAGTGAGAGTGCCCTCTAGTGTTTATTACTGAAGGTACAGCTTCACATG
GCTCAAATATTCAAGAGACACTGGACAACTGGAACTGTACAGTCTGAGGATAGTTCAATATCTATAGGTCATACAACTGT
GTGTGGGGGTGGGGGTGGTGGGGGGGTCAGGGATTAAATATTATTTAAAAGGGGCAATCAGGGATTGGTACATCCATTTT
AGGTAATTTAAATTAATATACTGTAGAGCAATTTAGTCTTGAAGAACATAACTTGTATTGTGATTTTATTTCAACTGTCT
TACCACATCAGGAACAAAAATATAACAATTTCCTGAGTCTGAAAATAACCCAGTTCTTCAATGGCCTGCATACTCTATGA
CCAACAGATGCATATCTGTATTCCCAGTCATGTGAAATCCATAGATTAGGGTCTAATGAATTTATTTCAATTGACTGATT
TCCTGATATGAACTGTAACTCAGTAAAATCTCTGAAGTTGTTGCATTTATACTTTTATTCAGTATATTTAGGTCAGACAG
CATACATTGTATTTGATGCATCCCAAAAATGTATAGTATGTAGAGAGGAACACCCAGTTAAATATACAAACATAACAAGT
GTTTATGACAGAGATGAACGATGGTAAAGGTGATATGACATTGAAAGACATGACGTGGGTGCTTGGTTTCAGCATATTCT

CTCATTGCATTTTAAGTGTGTTGCAGATAAAACATGTAAGAGGTTAGCATGTTTTTTTTATAGCCTCTAACCTTCTCACT
GACCACTATTCACTAAAAAGAAACCATGGCATTATCAGGATTCATTTAGAAGTGTTCAGTGACTAGATAACGTGCTTTCA
TTTCTAAACTGTAAAACAGATATGCATGAAAATACCCTCAAAAAGGTGACACCCTGTTCTGTTGCCTCATGAAACATTTG
ATCTAAAATCCAAAATGCAGAGCCACATTATACATTTCTGCTTCACTGTCCAAATACATATTGAGGGGAGTGTAGCTGCC
TCATCCTTTCAATAAGTGAGAATGCCCTCTAGTGTTTATTACTGAAGGTACAGCTTCACATGGATCAAATATTCAAGAGA
AACTGGACAAATAGAACTGTACAGTCTGAGGACAGTTCAGTATCTATAGGTCATATAACTGAGTGTGTGTGTGTGTGGGG
GGGGGGGGATTAAGTTTGAGGAACTTAATCAGAAAAATGCAGGCTTCACTTATCAAAAAAAACCTGAACAAACTGGATGG
AGAACAACAAAAAATGCCTCAACCTTCAAGATATAAACGCAACAAAAAAATTACCTACAGAAATACTTCTCACCAAAGGA
GATTTTGAATGAGGAAGTAAAATATTGTAAATAAATGTTATATTGTCAAGTTCCTCCAACCTCACTTCACGATGAGTTGT
GATATCCTTCTACCAATTTTTATATAACTATGTGACAAAAAACAAGATTCTTGTGAAGGTCTAATTGCACAAAATGAATT
ATCGATGACAATTGATTCCTTTCAGATTGGGAAAAGCCCTAGCAGTGACATTCCAATAGAGATATAAACATTTTATGAAC
TACTGAAAGATCCATTACTATCATGCTTTAAATTACTTGTATATAAATGGCAAACTGTCAGACAAAAAAAGACGGACCGA
TCTCTCTCCTACTGAAGCAGGAACCAGGGGGGAAGAACAAAGTACAATATTTGTGATTGCATGAGGCAATGTAGCATTCC
ACCATTCACATGCAATCACAAACTGGTTTGCGGCCCATCCAAGAATGGTTTCCCTTTTCCTCCCCACCTACTCGCCTTTT
TTTCTGCCTGGAGGTGGAAAGTGTATGATCATCGGCCACATGACCAAATGTCCCTCCTGGATGTAATGGATGCTGGCTGC
AGAGACTTCTCAGCTGAAGACTGCCAGGGGTGGATAAGGCATGCCAAGCATTTCTATCCAAGGTGCATAGCGAGAGACGA
CATAAGATGTGATGTGGACGAGAACGGCCTTCCCTGTAGCTCAGTTGGTAGAGCATGGTGTTTGCAACACCAGGGTTGTG
GGTTCGATTCCCACGGGGGGCCAGCACAGAAAAAAAAAAAAAAAATTAAAAAAAAAAAACATGTATGAAATGTATGCATTC
ACTACTGTAAGTCGCTCTGGATAAGAGCGTCTGCTAAATGACTCAAATGTAAATGCAAATGTAAACATGTGGCCAAATGC
TGAAGATCGTATAGATTAGAACAGGACTGTTCATTTTGTGTTTTTTTTTCCCTCTGTGCCTTTTGTACTGTAATTGTGTT
TTATTTTTACACATTTGGAACCAAAGGCCATGTATGTTGGGTTTTTTGTTGATCACCGGCAGCAATTTCATGTTGCTATT
AAAGCTTTTACTGCAGCAATTCTGTCACTTACAATTTTTTCTACTCTACGTTCTATAAAGTAAAGATGACTCATTCACTT
TTAGATAGTATGTTGCCAAAAATGCACACATTGTCACGACTTCCGCCGAAGTCGGCTCCTCTCCTTGTTCGGGCGGCGTT
CGGCGGTCGACATCACCGGCCTTCTAGCCATCGCTGCTCCATTTTTCATTGTTCCATTTGTTTTGTCTTGTTCCCCGCAC
ACCTGGTTTACATTCCCTAATCAACTGCATATTTAAATTCCTCTGTTCCCCCCATGTCTTTGTGTGGGATTGTTTTGTTA
CGTGTTTTGTATTGACGCGGCTAGCTGGTTTTTCCCCGGGTACTATGTGTCAACCCGGAGTATGTTTAATTATTAAACAT
TTTTGCTTATTTGTGACTTTGTCACGCTTTGCACTTTTGCCTGTGGCTTGAGGTTTGTTGGACACTGTTGTGTCCGTCTG
TTGTTTGCTTCTGCCGAATAAAGTGTGCGCCTGATCACAACTCTCTGCTCTCCCGCACCTGACTTCAACACCAGTAGCGC
ATACGCTGACCCACATTCAACACTCAACCAAAAAATGTAGTGGTTTACAGTAAAAGGAAATTGAAAATAAAAAAATATGG
ATTGTCTATTGTATGCAGGTAGAACTGAAACATGAAAAGTGCTGCAGTTTTTGTAATGCCATCAACTGTTAGTGTTTTGA
AAGTCGCTGTGCTGTGATTGCCTATATGTTCCTCTTGGATAGAAAACATGTGTAAGTGTTTTGACAATGATTAGATATTG
AGTCGGGATTGCCTTGTTTTGATAGAGTTAGTGTATGTAGAGAAAGTTTTTTGTATTTTGAAATGTAGAGTTGGTGTTTA
AGGAACAAAATGTGGTTTTGAGCATAACATTTACTATTTGGCTAATTGTGTGATGTTGCTGTGTTAAGACTTTAGAAAAA
GTATCTTAAGTATAGGTAAACGCTTGTTAGCAATTGTAAAAAACTGTACATATTATTTAAAAGAGGCAATCAGGGATTGG
TACATCCATTTTAGGACATTTCAATTAATATACTGTATAGCCATTGAGTCTTGTAGAATATAACTTACAAAATGGATTGT
GATTTTATTTAAACTGTTTTACCACATCAGGACCAAAAATATTACAATTTCCAGAAGCTGAAAATGACCCAGTTCTTCCA
TGGCCTGCATACTCATGGCCTGCATGTCACCCATTGAGCATGTTTGGGATGCTCTGAATCGACGCGTACGACAGCATGTT
CCAGTTCCCGCCAATATCCAGCAACTTCGCACAACATTTGAAGAGGAGTGGGACAACAATCGACAGGCCACAATCAACAG
CCTGGTCAACTCAAGGTGAAGGAGATGTCGTGCTGCACGAGGCAAATGGTTGTCACAACAGATACTGACTGGTTCTGATC
CACGCCCTTACATTCTTTTAAAGGAATCTATTACCAACAGATGCATATCTGTATTCCCAGTCATGTGAAATCCATAGATT
AGGGTGTAATGAATTAATTTCAATTGACTGATTTCCTTATATGAACTGTAACTCAGTAAAATCTTTGAAGTTCTTGCATT
TATACTTTTATTCATTATATTCAGTTCAGACAGCATACATTGTATTTGCTGCATCCCAAAAATTTATAGTATGTAGAGAG
GAACACCCAGTTAAATATACAAACATAACAAGTGTTCTTGACAGATTTGAATGATGGTAAAGGTGATATGACATTGAAAG
ACATGACGTGGGTGCTTGGTTTCAGCATATTCTCTCATTGCATTTTAAGTGTGTTGCAGATAAAACATGTAAGAGGTTAG
CATGTTTTTTTATAGCCTCTAACCTTCTCACTGACCATTATTCACTAAAAAGAAACCATGGTATTATCAGGATTCATTTA
GAAGTGTTCAGGGACTAGATAAGGTGCTTTCATTTCTAAACTGTAAAACAGATATGCATGAAAATAGCCACAAAAAGGTG
ACAACCTGTTCTGTTGCCTCATGAAACATTTGATCTAAAATCCAAATTGCAGAGCCACATTTTACATTTCTGCTTCACTG
TCCAAATACATATGGAGGGGAGTGTAGCTGCCTCATCCTTTTAATAAGTGAGAATGCCCTCTCGTGTTTATTACTGAAGG
TACAGCTTCACATGGCTCAAATATTCAAGAGACACTGGACAACTGGAACTGTACAGTCTGAGGATAGTTCAATATCTATA
GGTCATACAACTGAGTGTGGGGGTGGGGGGGGGTTCAGGATTTAAAACTGATTTAAGGGGGCAATCAGGGATTGGTACAA
CAATTTTAGGTAATTTCAATTAATATACTGTATAGCCATTGAGTCTTGAAGAACATAACTTGGATTGTGATTTTATTTTA
ACTGTATTACCACATCAGGACCAAAAATATAACAATTTCCTGAATCGGAAAATAACCCAGTTCTTCCATGGCCTCCATAC
TCACCAGACATGTCACCCATTGAGCATGTTTGGGATGCTCTGGATCGATGCGTACGGCAGCGTTTTCCAGTTCACGCCAA
TATCCAGCAACTTCACACAGCCATTGAAGAGGAGAGGGACAACAATTGACAGACCACAATCAACATCCTGACCAACTCAA
TGCTAAAAGATGTCGTGCTGCATGAGGCAAGTGGTTGNNNNNNNNNNNNNNNNNNNNNNNNNNNNNNNNNNNNNNNNNNN
NNNNNNNNNNNNNNNNNNNNNNNNNNNNNNNNNNNNNNNNNNNNNNNNNNNNNNNNNNNNNNNNNNNNNNNNNNNNNNNN
NNNNNNNNNNNNNNNNNNNNNNNNNNNNNNNNNNNNNNNNNNNNNNNNNNNNNNNNNNNNNNNNNNNNNNNNNNNNNNNN
NNNNNNNNNNNNNNNNNNNNNNNNNNNNNNNNNNNNNNNNNNNNNNNNNNNNNNNNNNNNNNNNNNNNNNNNNNNNNNNN
NNNNNNNNNNNNNNNNNNNNNNNNNNNNNNNNNNNNNNNNNNNNNNNNNNNNNNNNNNNNNNNNNNNNNNNNNNNNNNNN
NNNNNNNNNNNNNNNNNNNNNNNNNNNNNNNNNNNNNNNNNNNNNNNNNNNNNNNNNNNNNNNNNNNNNNNNNNNNNNNN
NNNNNNNNNNNNNNNNNNNNNNNNNNNNNNNNNNNNNNNNNNNNNNNNNNNNNNNNNNNNNNNNNNNNNNNNNNNNNNNN
NNNNNNNNNNNNNNNNNNNNNNNNNNNNNNNNNNNNNNNNNNNNNNNNNNNNNNNNNNNNNNNNNNNNNNNNNNNNNNNN
NNNNNNNNNNNNNNNNNNNNNNNNNNNNNNNNNNNNNNNNNNNNNNNNNNNNNNNNNNNNNNNNNNNNNNNNNNNNNNNN
NNNNNNNNNNNNNNNNNNNNNNNNNNNNNNNNNNNNNNNNNNNNNNNNNNNNNNNNNNNNNNNNNNNNNNNNNNNNNNNN
NNNNNNNNNNNNNNNNNNNNNNNNNNNNNNNNNNNNNNNNNNNNNNNNNNNNNNNNNNNNNNNNNNNNNNNNNNNNNNNN
NNNNNNNNNNNNNNNNNNNNNNNNNNNNNNNNNNNNNNNNNNNNNNNNNNNNNNNNNNNNNNNNNNNNNNNNNNNNNNNN
NNNNNNNNNNNNNNNNNNNNNNNNNNNNNNNNNNNNNNNNNNNNNNNNNNNNNNNNNNNNNNNNNNNNNNNNNNNNNNNN
NNNNNNNNNNNNNNNNNNNNNNNNNNNNNNNNNNNNNNNNNNNNNNNNNNNNNNNNNNNNNNNNNNNNNNNNNNNNNNNN
NNNNNNNNNNNNNNNNNNNNNNNNNNNNNNNNNNNNNNNNNNNNNNNNNNNNNNNNNNNNNNNNNNNNNNNNNNNNNNNN

NNNNNNNNNNNNNNNNNNNNNNNNNNNNNNNNNNNNNNNNNNNNNNNNNNNNNNNNNNNNNNNNNNNNNNNNNNNNNNNN
NNNNNNNNNNNNNNNNNNNNNNNNNNNNNNNNNNNNNNNNNNNNNNNNNNNNNNNNNNNNNNNNNNNNNNNNNNNNNNNN
NNNNNNNNNNNNNNNNNNNNNNNNNNNNNNNNNNNNNNNNNNNNNNNNNNNNNNNNNNNNNNNNNNNNNNNNNNNNNNNN
NNNNNNNNNNNNNNNNNNNNNNNNNNNNNNNNNNNNNNNNNNNNNNNNNNNNNNNNNNNNNNNNNNNNNNNNNNNNNNNN
NNNNNNNNNNNNNNNNNNNNNNNNNNNNNNNNNNNNNNNNNNNNNNNNNNNNNNNNNNNNNNNNNNNNNNNNNNNNNNNN
NNNNNNNNNNNNNNNNNNNNNNNNNNNNNNNNNNNNNNNNNNNNNNNNNNNNNNNNNNNNNNNNNNNNNNNNNNNNNNNN
NNNNNNNNNNNNNNNNNNNNNNNNNNNNNNNNNNNNNNNNNNNNNNNNNNNNNNNNNNNNNNNNNNNNNNNNNNNNNNNN
NNNNNNNNNNNNNNNNNNNNNNNNNNNNNNNNNNNNNNNNNNNNNNNNNNNNNNNNNNNNNNNNNNNNNNNNNNNNNNNN
NNNNNNNNNNNNNNNNNNNNNNNNNNNNNNNNNNNNNNGGTTTTTTGTTGATCACCGGCAGCAATTTCATGTTGCTATTA
AAGCTTTTACTGCAGCAATTCTGTCACTTACTATTTTTTCTACTCTACGTTCTATAAAGTAAAGATGACTCATTCACTTT
TAGATAGTATGTTGCCAAAAATGCACACATTGTCACGACTTCCGCCGAAGTTGGCTCCTCTCCTTGTTCGGGCGGCGTTC
GGCGGTCGACATCACCGGCCTTCTAGCCATCGCTGCTCCATTTGTCATTGTTCCATTTGTTTTGTCTTGTTCCCCGCACA
CCTGGTTTACATTCCCTAATCAACTGCATATTTAAATTCCTCTGTTCCCCCCATGTCTTTGTGTGGGATTGTTTTGTTAC
GTGTTTTGTATTGACGCGGCTAGCTGGTTTTTCCCCGGGTACTATGTGTCAACCCGGAGTATGTTTAATTATTAAACATT
TTTGCTTATTTGCGACTTTGTCACGCTTTGCACTTTTGCCTGTGGCTTGAGGTTTGTTGGACACTGTTGCGTCCATCTGT
TGTTTGCTTCTGCAGATTAAAGTGTGCGCCTGATCACAACTCTCTGCTCTCCTGCACCTGACTTCAACACCAGTAGCGCA
TACGCTGACACACATTCAACACTCAACCAAAAAATGTAGTGGTTTACAGTAAAAAGAAATTGAAATATAAAAAAATATGG
ATTGTCTATTGTATGCAGGTAGAACTAAAACATGAAAAGTACTGCAGTTTTTGTAATGCCATCAACTGTTAGTATTTTGA
AAGTCGCTGTGCTGTGATTGCCTATATGTTCCTCTTGGATAGAAAACATGTGTAAGTGTTTTGACAATGATTAGATATTG
AGTCGGGATTGCCGTGTTTTGATAGAGTTAGTGTATGTAGAGAAAGTTTTTTGTATTTTGAAATGTAGAGTTGGTGTTTA
AGGAACAAAATGTGGTTTTGAGCATAACATTTACTATTTGGCTAATTGTGTGATGTTGCTGTGTTAAGACTTTAGAAAAA
GTATCTTAAGTATAGGTAAACGCTTCTTAGCAATTGTAAAAAACTGTAAATATTATTTAAAAGTGGCAATCAGGGATTGG
TACATCCATTTTAGGACATTTCAATTAATATACTGTATAGCCATTGAGTCTTGAAGAATATAACTTACAAAATGGATTGT
GATTTTATTTCAACTGTCTTACCACATCAGGACCAAAAATATTACAATTTCCAGAAGCTGAAAATGACCCAGTTCTTCCA
TGGCCTGCATACTCATGGCCTGCATGTCACCCATTGAGCATGTTTGGGATGCTCTGAATCGACGCGTACGACAGCATGTT
CCAGTTCCCGCCAATATCCAGCAACTTCGCACAACATTTGAAGAGGAGTGGGACAACAATCGACAGGCCACAATCAACAG
CCTGGTCAACTCAAGGTGAAGGAGATGTCGTGCTGCACGAGGCAAATGGTTGTCACAACAGATACTGACTGGTTCTGATC
CACGCCCTTACATTCTTTTAAAGGAATCTATTACCAACAGATGCATATCTGTATTCCCAGTCATGTGAAATCCATAGATT
AGGGTCTAATGAATTTATTTCAATTGACTGATTTCCTGATATGAACTGTAACTCAGTAAAATCTCTGAAGTTCTTGCATT
TATACTTTTATTCAGTATTTTCAGTTCAGACAGCATACATTGTATTTGCTGCATCCCAAAAATGTATAGTATGTAGAGAG
GAACACCCAGTTAAATATACAAACATAACAAGTGACAGAGATGAATGAATGTTAAGGTGATATGACATTGAAAGACATGA
CGTGGGTGCTTGGTTTCAGCATATTCTCTCATTGCATTTTAGGTGTGTTGCAGATCAAAAAAATATTATTCTCATAAATC
TACCTACCCCCTTTCTATGTGCCCTCTAGACATACTGTATTTCAGCCTTTTTAGGGATAGAGGGAATCCGTTTGAAAAAT
ACTAATTACACCTTGATTATGACAGACATTTGTTTATTAGCAATGATACACAGTCCATTCAGTGATTTATTGGTTTATTA
ATAAAAGTTAATGAGATCTTGATTTAAAGTCAAGTCATAAGCATAATCAAATATTCTTATTGAGTGTGGCAGGTAGCCTA
GTGGTTAGAGCATTGGGCCAGTAACTGAAAGGTTGCNNNNNNNNNNNNNNNNNNNNNNNNNNNNNNNNNNNNNNNNNNNN
NNNNNNNNNNNNNNNNNNNNNNNNNNNNNNNNNNNNNNNNNNNNNNNNNNNNNNNNNNNNNNNNNNNNNNNNNNNNNNNN
NNNNNNNNNNNNNNNNNNNNNNNNNNNNNNNNNNNNNNNNNNNNNNNNNNNNNNNNNNNNNNNNNNNNNNNNNNNNNNNN
NNNNNNNNNNNNNNNNNNNNNNNNNNNNNNNNNNNNNNNNNNNNNNNNNNNNNNNNNNNNNNNNNNNNNNNNNNNNNNNN
NNNNNNNNNNNNNNNNNNNNNNNNNNNNNNNNNNNNNNNNNNNNNNNNNNNNNNNNNNNNNNNNNNNNNNNNNNNNNNNN
NNNNNNNNNNNNNNNNNNNNNNNNNNNNNNNNNNNNNNNNNNNNNNNNNNNNNNNNNNNNNNNNNNNNNNNNNNNNNNNN
NNNNNNNNNNNNNNNNNNNNNNNNNNNNNNNNNNNNNNNNNNNNNNNNNNNNNNNNNNNNNNNNNNNNNNNNNNNNNNNN
NNNNNNNNNNNNNNNNNNNNNNNNNNNNNNNNNNNNNNNNNNNNNNNNNNNNNNNNNNNNNNNNNNNNNNNNNNNNNNNN
NNNNNNNNNNNNNNNNNNNNNNNNNNNNNNNNNNNNNNNNNNNNNNNNNNNNNNNNNNNNNNNNNNNNNNNNNNNNNNNN
NNNNNNNNNNNNNNNNNNNNNNNNNNNNNNNNNNNNNNNNNNNNNNNNNNNNNNNNNNNNNNNNNNNNNNNNNNNNNNNN
NNNNNNNNNNNNNNNNNNNNNNNNNNNNNNNNNNNNNNNNNNNNNNNNNNNNNNNNNNNNNNNNNNNNNNNNNNNNNNNN
NNNNNNNNNNNNNNNNNNNNNNNNNNNNNNNNNNNNNNNNNNNNNNNNNNNNNNNNNNNNNNNNNNNNNNNNNNNNNNNN
NNNNNNNNNNNNNNNNNNNNNNNNNNNNNNNNNNNNNNNNNNNNNNNNNNNNNNNNNNNNNNNNNNNNNNNNNNNNNNNN
NNNNNNNNNNNNNNNNNNNNNNNNNNNNNNNNNNNNNNNNNNNNNNNNNNNNNNNNNNNNNNNNNNNNNNNNNNNNNNNN
NNNNNNNNNNNNNNNNNNNNNNNNNNNNNNNNNNNNNNNNNNNNNNNNNNNNNNNNNNNNNNNNNNNNNNNNNNNNNNNN
NNNNNNNNNNNNNNNNNNNNNNNNNNNNNNNNNNNNNNNNNNNNNNNNNNNNNNNNNNNNNNNNNNNNNNNNNNNNNNNN
NNNNNNNNNNNNNNNNNNNNNNNNNNNNNNNNNNNNNNNNNNNNNNNNNNNNNNNNNNNNNNNNNNNNNNNNNNNNNNNN
NNNNNNNNNNNNNNNNNNNNNNNNNNNNNNNNNNNNNNNNNNNNNNNNNNNNNNNNNNNNNNNNNNNNNNNNNNNNNNNN
NNNNNNNNNNNNNNNNNNNNNNNNNNNNNNNNNNNNNNNNNNNNNNNNNNNNNNNNNNNNNNNNNNNNNNNNNNNNNNNN
NNNNNNNNNNNNNNNNNNNNNNNNNNNNNNNNNNNNNNNNNNNNNNNNNNNNNNNNNNNNNNNNNNNNNNNNNNNNNNNN
NNNNNNNNNNNNNNNNNNNNNNNNNNNNNNNNNNNNNNNNNNNNNNNNNNNNNNNNNNNNNNNNNNNNNNNNNNNNNNNN
NNNNNNNNNNNNNNNNNNNNNNNNNNNNNNNNNNNNNNNNNNNNNNNNNNNNNNNNNNNNNNNNNNNNNNNNNNNNNNNN
NNNNNNNNNNNNNNNNNNNNNNNNNNNNNNNNNNNNNNNNNNNNNNNNNNNNNNNNNNNNNNNNNNNNNNNNNNNNNNNN
NNNNNNNNNNNNNNNNNNNNNNNNNNNNNNNNNNNNNNNNNNNNNNNNNNNNNNNNNNNNNNNNNNNNNNNNNNNNNNNN
NNNNNNNNNNNNNNNNNNNNNNNNNNNNNNNNNNNNNNNNNNNNNNNNNNNNNNNNNNNNNNNNNNNNNNNNNNNNNNNN
NNNNNNNNNNNNNNNNNNNNNNNNNNNNNNNNNNNNNNNNNNNNNNNNNNNNNNNNNNNNNNNNNNNNNNNNNNNNNNNN
NNNNNNGAAGGATAATGCTTCTATTTGACTGAATTTTACAACAGGATTTGGTCATACACACTAAACAGTCATTATTGTGG
CCCAGACACATTGGCTAGACAGACCCCAAAGGCAAAGTCTTCAAAGGCTAGTTAATCTGACTGACAAAAGTGCAAGCTAG
AACAAACTCAAGGAAGACTATTTGACATTGTGAGCTCCCTAATTGGTGTACCATTCCAAACAAAGAAATGCATCCACTCA
TATGCAGTCAACGCCATCCGTAATTGTTATTTTATTTACACTAGTATTTACAGAGCGCTTCTCCTTTCTTTGTGGAGGGC
TGCAGAGCGGAGAAACTACACCCCCATTCACTTACAAATTCATATTTACTTCTGTCAGCTTCTCAATGCTGGTTTCTGGA
TGCATTGTTGTTCTGCATCTATTGTGTAATATTGACAGCCTCCGCATCATTAGTAGTGAAAAAAAAGGTGAGCCTACCAC
AGTCACGAATATAGCCCGGCGTTGCCTATAAAAGGAAAAAGAACAGCATCAAGCGACACTCAACCTCTTGAAGACCCATG
ATCAGGTAAGCCTCTTCGCTCTGCAAGAATTACAGATCTTTAGAGCATTAACTATTCGAGAACACGCATGCTAAAATATG
AATTCATCAGTCTCCGTGATGTGCAAAGGCTAGTCTCTTGATTCTCAAAGGATTTCCTATTTTTATTGTTTAGTAACATT

TTCGCTTATGGCTATCAATGTGAATAGGTTAGTGAGAAAAGACTAAAGTTGATGTTAAAATAAGGTAACAAAAATGCCAT
AGATGCTATCATACAGTTGTGCAAAGGTTCTGACAACAGGTCAACTAAGGCAAGGTACTCCATACTGATCATGTGGATTC
TTATTCTAGATAGGTGCAGTCTCTCTCTCCCGGATTTCAGACACAATGTTTGCGATATTGGTTTGCATCCTGACACTGCA
TGACGTGTACTCTGCGTGCAATGTGGTAAGTACTCCTACTAATTGCTGAAAAGAAAACCACATATGTAATCTGAAACAGT
ACATTAGATGTGAGGGCAATTACATGTTAAAGTATTTATGAAAGATCTTATTCTGAAAGCTTTTCATTCTTTCCCTTGAG
CCATCTCCTACTGCTGTGTCCACAGTCAAGTAAGTACAATGCCTTACGAACATTCTAACACAGTGCCCAGAGAAAGTCTA
CACACCCCTTGCACAGCCTCCGCATTTTGCTGCCTTAACATTTAAAAAGCGATTCATTTTCATATGTTCCCCAACCTACT
CCAGATTGTAAAAGTGAAAGACTGTTATAGAACATTTTCCAAATCAATTCTAAATTAAATGAAAATGTCTTGATTGCATA
TGTCTTGACACCCCAGAGTTCATACTTGGTTGAAGCACCTTTGGCAGCCCTTAAAGCATTGAATCATTTTGAATAAGCAA
TATTCAAACCTTTTTTAAGGAGATTTAGTTCAGGATTGCGACTGGACCATTGAGGAACAAGTAAACACCTCTTGGAAAGC
CATTCAGGTATGTCTTTGGCATTAAAATAGTTATAATTTTCCTGAGAAAAAAAAAAAAACCTCCTGGTTGGTATTATGGTG
GCAGCATCATGTTATATGTATTGCTTGTCATCGGCAGGGACTGGGGAGTTTGTCAATTTCAAAATAAATATGAAAGCCGC
AAAGCCCAGGTAAATATTGACAAAAAAAAAACCCTCCTCAGTCTTCTGAAAACCCTGGGATAGAGTTGTATTTTTCAGTGA
GACAATTATACCTAATCTTATGTCAGACACACCCGAATGGATTTCCAAAAGGTGTTGTGTCGTCGAGTGGTCTGGACTCA
GTCCTGACATAAATCTGCTTGAAAAGAGGTTTAAATATGCTTTCCATCAATGATACCCAACCAAATTTACTGCGCTTGAG
CAATTTTGACAAAAAATAATTGGCATATGCTGCCCTAAGAGTTGTGCAAAGTCGGTAGAATGTTATTCTAAATGATTTAC
ATCTGTAATGGCTGCTCCACCAAGTATTAACTCTGGGGTGTGAAGACATACGCAAAAACAAAACATCTTTGTTGTTGTTG
TTGTGTGTTTACATTTTCTATAACTTTATTTTACTTCAGAAAGGTGGAGTAGGTTGAGTAGATCTGTACTGTAGGAGAGA
AAAAAAACACCGCATTTAATCCCTTTCAATTTCATGCAGCAAAACGTGAAGACTGCAAACGATGTGTACTTTCACTATCC
TCTACCAACATGATTCTAAACTAATTCAACTGGATTTAGTCATTACACACAATGTTTAATTCATCATGTGCTGTTTTTCA
AAAGAAAGTGGATTGATTTGGGACTCATAATCAAGATGTTGGAAAAGATATTAGGAGTAAATAGTTGCTTTGTATTATGG
TTTAGTGAAGAATTTACCTGTGGGGTAAGTAATGTGCATATAATCCCACAATATAATGTGTGTGTGTGTGTGTGTGTGTG
TGTATAGAAATTTGCCCAAACAACACAGTAGTTCAAGCTGAGATCCTTAACCAGCACAACGCCTTCCGGAGAGCGGTTAC
GCCAACTGCTAGAAACATGCTCAAGATGGTACGCTTTCATGATTCATATACTTTCCATCAGTAACAACCTCAGAATCAAT
GTTTCCATCGTAAAGGGTATAATCCCTGATATGTAATTCATAGAGCAGCCCTATGGTATACTCCTTGGACACTAGTATGT
GGATGTCAGTCTTGTGGATGGCATTATTCTAGAGCTTTTTGTATCTGATCTTCAGAACTGGAGTGAGGAGGCAGCGGCCA
GTGCTCAGGCTTGGGTTGACACCTGCTCCATGGATCATGGCCCACCCAGCAGTCGCATGCTTGGCGGTACGGTTTCCCCT
TTGTCTCTCCTTGACATCTCTTGTCTTTCCTCTGATGTAGAGCAAACTAAGACAAATATATTTTATTGATTCATGCAACT
CAAGACTTTCGCTGCAATGGTATCAATGTGAGCGCAATCTTAAGCTCAAATATATTTGGAATGTCATGATTTCTTATCAC
CAGTGACAATGGTAGCTTACCTGTTATCTTATATTTGTTCACATCTTAAATCCTGCATTCAAATACATTTTAGTCAACTG
TTCCAGCTTGATTTCACTGTTACACACCTACAGTATGTAGTAAACTTTTCCATGCATGCTTTACTTTTGAACAGATTACG
AAATGGGTGAGAACCTGTTCATGTCCTCCGCTTTTAGGAACTGGACTGCAGTAGTTACAGCCTGGAACAGTGAGGTCAAA
GATTACTCCTATCCCAATGGATCTATCAATGGTAAACCCATCGGCCACTACACACAGGTAACATTCATCTTATGTGCAGA
TACATTATGACATGAAGAAAGTATTCAGACCCCTTGACTTTTTCCACATTTTGTTACGTTAGCCTTATTCTAAAATGGAT
TAAATAAAACAACTTCCTCCACATACAATACCCCATCATGACGAAGCAAAAACAGTTTTTTTTTTTAATTTTGCACATTT
ATTAAAAACAAAAAACAGAAATACCTTATTTAAATAGGTATTCAGACCCTTTGCTATGAGACTTGAAAACAGGATGCACC
TGAGCTCAATTTCCATTGATCATCCTTGAGATGTTTCTACAACTTGATTGGAGTCCACCTGTGGTAAATTCATTGATTGG
ACATGATTTGGAAAGGCACACACCTGTCTATGTAAGGTCCCACAGTTGACAGTGCATGTCAGAGCAAAAACCAAGCCATG
AGGTCGAAGGAATTGTCTGAGAGCTCCGAGACAGGATTGTGTGAAGGCACAGATCTTGGGAAGGGTACCAAAAAAATATC
TGCAGGTCCCCAAGAACACAGTGGCCTCCATCATTCTTAAATGGAAGAAGTTTGGAACCAACAAGACTCTTCCTAGATCT
GTCTGCCCGGCCAAACTGAGCAATCAGGGGAGAAGGGCCATGGTCAGAGAGGTGACCAAGAACCCGATGGTCACTCTGAC
AGAGCTCCAGAGTTCCTCTGTGGAGATGGGAGAACCTTCCAGAAGGACAACTATCTCTGCAGCACTCCACCAATTAGGCC
TTTATGGTAGAGTGGCCAGACAGATGCCACTCCTCAATAAAAAGACACATGAAAGCCCACTATGAGTTTGCCAAAAGGCA
CTTAAAGGACTCTCAGACCATGAGAAACAAGATTCTCTGGTCTGATGAAACCAAGATTGAACTATTTGGCCTGAATGCCT
AGCGTCACATCTGGAGGAAACCTGGCACAATCCATACAGTGAAGCATGGTGGTGGTGTCAGCATCATGCTGTGAGGATGT
TATTCAGCGGCAGGGACTGGGAGACTAGTCAGGGTAGAGGCAAAGATGAACAGAGCAAAGTACAGAGAGAGATCCTTGAT
GAAAACCTGCTCCAGAGTGATCAGGATCTCAGACTGTGGCAAAGGTTCACCTTCCAACAGGACAACGACGCTAAGCACAC
AGCCAAGACAACGCAAGAGTGGCTTCGGGACAAGTCTCTGAATGTCCTTGAGTGGCCCAGCCAGAGCCCGGACTTGAACC
CGATCAAACATCTCTGGAGAGACCTGAAAATAGCTGTGCAGCAACATTCCCCATCCAACCTGACAGAGCTTGAGAGGATC
TTCAGAGAAGAATGGGAGAAACTCCCCAAATACAGGTGTGCCAATCTTGTAGCATCATACCCAAGAAGACTCGAGGCTGT
AATCGCTGCCAAAGGTGCTTCAACAAAGTACTGAGTAAAGGGTCTGAATACTTATGTAAATGTGATATCAGTTTTTCTTA
TTTGATAACAGATATCAGTTTATCTTTTCGGATAACAAAAAACAGTTTTTGCTTTGTCATTATGGGATATTGTGTGTAGA
TTGATGACTCAAAAAAAACTATTTATCAATTTTAGAATAAGGCTGTAACCTAACAACAAAAAAGTAAAAGGGTCTGAATA
CTTTCCAAAGGCACTGTGCAACATCTGATCAAATCACAGGTTCTATAGGCCCATTTATAGAGAAACAGTCTGAGGCCGAT
ATCAAAAAGAACAATAAGTTTAATGAATCAGGTATAAGTTTGCTTGTTTAAAAAATGTACAGTAAAACAATCCGAAAACA
CACCTCCGTTCCTTTCACTGGATGGCTCTATCAATTTCTAAATATCTACAGTGCTTCCACAGAGACAACTGACCACGTTC
ACAACGGACCAAAAACAGTGACAAACAACTTCTGCATGAAAGAAAACAGCAGTTAGCCACTGCATCTACACAAACACTAA
CTATTTTAGAAATACAAAATGGATTTACTTAAACAGTCAATATACTGCTTGGACCCCCATGAACATCTCTCCAAACACAT
CAAAGACAACTGCACCATAACTTTCAAAGTAATACATGTGCTGTCAAATTTGAATAAACAAACACAGATAGAGAAATAAA
CATACCTTGTGAACCGATTTGACCACACCCAAAACAGAGTGCACAGCAACAACACTATACAGTCATAACACTGGACCTTT
AGGTACAAAAGGACCCGCAATGGACTATGGGGTATTTTTTATACATTTGACATGTCCACCACTAATTAGGGTCAGATAGG
AAACACACACCAAAACTACAGTTTATATTCTGTCACGTATATTTTCAGGGATATTTAATCGCATATATCTGTTGACACCA
AAGGAGTATACTTACAAAATAGATTACTAACTTATTGACATTTCTTTTGGTAGCAATTTCAGTCATTGTATTGTACATAC
ACTGAGTATACCAAACATTAGGAACACCTTCCTAATATTGAGTTGCACCACCACAAACCGGTGCGCCTGGCACCTACTAC
CATACCCCGTTCAAAGACACTTAAATCGTTTGTCTTGCCCATTCACCCTCTGAATGGCACACATACACAATCCATGTCTA
AATTGTCTCGAGGCTTAAAAATCTTTCTTTAATCCGTCTCCTCCCCTTCATCTACACTGATTGAAGTGGATTTAACAAGT
GACATCAATAAGGGATCATAGCTTTCAGCTGGATTCACCTGGTCAGTCTGTCATGGAAAGAGCAGGTGTTCTTCGTTCTT
AACATTTTGTATACTGTATATTGTTATGGACCATTGTGCTACACCTGCAATAACACCTGCTAAATATGTTTATGCGACCA
AGAAAATGTGATTTGATTTGCTATGCTGTATGGTAGTCAGAGAGGTGTGTAGTTACCGACAAGTGTCATCATGGTTATTT

GCCAATGAAACTCTGATAACACTAAACTGCATTTCCTTTCATATAGGTGGTTTGGAACAGTTCATACAAGGTTGGCTGTG
GTGTGGCCCTGTGTCCTGGTTCAGTCTACTTCTATGGATGCCAATACTACAGAGCGTATGTTGCGCCAATCACACCCAAA
ATGCACCTAACACCTGTGTACAATTTCACCCTATTTGCTATAACTGGTAGTGGAAGGTAGGCATCATCCAGCAATCATTT
CAATACTTTGGTTAACAGGGGAAACTACAAAGGAGTGGCTCCATACAAGGAAGGAGCTACATGTGCTGACTGCCCCAACT
CCTGCGAAAATAAGCTTTGCAGTAAGTATCAGACGGGATGAAAACACATGCATGCCGTCTAAAATCTTACCAATATGACT
CAGCAATGTTAGTGATGCCTTTTAAAAACATGTTCACACGGTTCAATGTACAATAATCACCAAATATAAACTGTCAACAT
ACAATGAATTGACTGCCAATGTTTATTCTCATGTGTCATTTCAGCCAATCCCTGTCCTTACATAAACAAGTATTCCAACT
GTGACGCCATGAAAAAACAGGCCGGGTGCACCAATCCTTTGGTGTACGCCTGGTGTCCTGCCCTCTGCCTGTGCACCTCC
CAAATCTCATGAAACCACCAGACCTCTTCAAAATGGTCCACTGAAAGATAACTTTGAATGATTCCTCTGTTGTTTCCTTG
TCTATGAGAAGATACACCTGGCTGATCTTTCTACTGTACTTCTGGTGAAGTCAGTTAATTTGAAAAATACTAACTATCAC
TAATAATATCTTAATTATCACTGTAGGTCTAAGGTACAGTTAAAACATTAAAACATTATTCTTATTCTGGGGGTAACATA
AGGTGTCTAACCTATAGTAACTAATGTTAGTTGGAAGCGTAGCAATTTATTTTGTTCCCTTTTATTGATTTCAATGTCTT
CATACCAATTTGACCCCCCAAAAAATCTGCATTTGATTTGCATGCAAGTTGAATAAGAATATCTTCTACATTTTGAGAAG
GCAATATTTCCATCAGTTACTGTAACAGTGAACATTTTGATAATTCCATTTCTGCTAATAAACTACACAGTAATATGGTT
ATACAGAACTGTAATACCTCAACACATTGAACTTTAAGTTGATTCAGGGCATCATTTTATGTTTGATGAAATGCAAGGAA
TGCTGCTCATAATTTTCTCTATCAACATGAAATGTTTTCACGTGACAGAATTATATAACCCTTATTGTTTTCCTCAATCA
CATTAAATGATCATGAAGATTAATTCATGGAGTCTGTGTTAATGGTTTTACAACCCCCCAAAGCGCAACCTCTTCTTATG
TTTTTAAATGGGCAACTGAGATGATGGAAATAGAACCAAAGGCCTCAGTAGTCACTCTCAGATAGTCAGCGTCACCACCA
GATGCCTCTGTTGTACAACAGTACATTCTGTATCAGAATAACATGACTATCTGCAGGCTTGATATACACCGTCCCTTCTC
GTGTCCCTTACAACATCCTGGAACTGATCAATAGGTCTTTTTAACAATTGGTTTATGTGAACTAATCCTTAATAAGAAAA
AATTGCACATGACTTTAATAAGAACCCTGAGAAGGCAACACAAGGTGGAACTGGCACGCTGATATAGAGGTAAGGTAGTC
TGCAGTATATTTAATGTCTGGAAATTGATGCAAAAAGGTTCAATTGAACTGTTTATGGTCATGTCTAATGTGTAGGCTGC
ATAAACTTCAGCAATGCATACCCTCCTGCACATAAACTCAGACATGGTTAACTTTGACTAAAATATTAGAATGTAAACTG
TAAGGATCCTTTTTGAATAGCTAAAGTGAATAAGAGCTGCTACAAGCAAAACAATGAATTATACTGTTATCTGTTTCAAT
AACTCTATAGGCTATGCTATTGAAGGAACATATAGTCTTCTGTTGATAATTGACATAAATACAGCATATTGATAACGTCT
CAGATTGACATTTTTCAGACCGTAAAATGCTATATTTTTACCCAGGAAATCATTTGGATTGACACAATCTGAATTAAATC
CAAGATCTTAACATTTGCTGATTAACATACCAAGCGAAAAATGTGTAAGATTTTAATATATTACAAGTATACAAAAAATT
AGAATATACATTGAATACGAGATGTATTTTTCTTGTATATCTTAAGTATACTATAACTATAATCTCATTACACTTATCAT
TACACACTTATTAAAAGTGTACTTTAAATTAATTGTTTTTAAGCTTTTAGTATACTATAAATATACTATCGTCAACCTTC
AATACACTTATTAAAAATATAGTTTAAATTAATTGCTTTTCAATCTTTTAGTATACTATATGTTCACTATCTTTAAACTT
AAACACACTCATTAAAACTGTACTTCAATTTGAAACGCTTTAATTGTCATTTTAGTATACTAATTCAAAATACACTTGGA
GTTGTTTTTAAATTGAAACATTGATTTAAAAATATATATATATTATTTTTTACCCCTTTTTCTCCCCAATTTCGTGATAT
CCAATTGGTAGTTACTGTCAGAGTCTAGGTGATGTGGGTAGGCGGAGTCAGGCGCAGGACACAGAGATGAGTAATAATAG
TAACTTCACTCAAAAATAATCCTCCAACAAGGAGAACGAAAATCCAGAATCACATAAACGAGACAACTGACAACAATAAA
CACGCACAAAGCCATGATGGCTCCAGAGGGTTAAATAGGGAAATAATTCAAATGAAATGGGAACCAGGTGTGTACAATAC
AGACAAAACAAATGGAAAAAGAAATGTAGATCGGTGGATAGCCTGTGACGTCGATCGCCGAACGCCACCCGAACAAGGAG
AGGCACCAACTTCGGCGGAAGTCGTGACAGTTACAGTCTTGTCCCATCGCTGCAACTCCGGTACGGAGTACCTTGAGACA
AGGGTTATGGTCCAAACACTTAAGACACACCCTCGTCCACTCACCCTCGCCATATGTCCTTGGGGGAATCCATCTTGGAG
GGTGGTCCAAATGATTAGCCAAGCAAGGGAAGTTTGCAACGTAAGGCCCTCAGCCCTCGTTTTTAGTTGGCTTTGCGAGT
GTATAATTATGTTCACTCCAGGGGCCTGAAACTCCCCATAATTCAATTTGCGACGATTGTACATCCGCTAAGAAAAGTCA
GCGAAAACTTAAAAACAACATCAATGGAGAAGTCAACATACAAGTGTAAGTAAAAATGAATGCAAATACTGTAAGTTAGA
AATTGTGCTACTAATGCACATGACGGCACGTCTTGTAAAAAGTAAATATGTTTTTGTTTGGTTATGTTTTTGAAATTGTA
GAAATTAAACATTTTCCTTCTTCAGAATTTCCAGCTAGGCAGGCTAACGTTAGTTAGCTAATTAATTTGCTAATTGGTAG
GTGTATATTAATAATTATATATTTAATATAAGTAGACATGCAATCATAATTGAATGTAGAGCATATAAAGCACCCCACAAG
TTACCGGTGGCTGAAAACACAATCATCGCTGGATGAATGAGTGACGAATTTCTGGCCAAGGGTGGTCCATTTAAAAAAAC
ATTCCTTCCCTCGCCCCTTGCGTATACTCGTCAGACGTCATCAGAAGTGTCCACTTAATTTGAGGGCTGAGGGGATAGGG
TGTGTCTTTTAAGTGTTTGGAATGCAGCCAAGGTACAGACTTGGGAGAGGCGAAGGTCGAGAGCCAAGCATCCTCTGAAA
CACAACCCTGCCAAGCGACACTGCTTCTTGACACACTGCTCGCTTAACCCGGAAGCCAGCCGCACCAATGTGTCGGAGGA
AACACTGTACAGCTGGCGACCGAAGTCAGCGTGCATGCGCCCGGCCCAGGACAGGAGTCACTAGAGTGCGACGGGACAAG
GACATCCCAGCCGGCCAAACCCTCCCCTAAGCCGGACGACGCTGGGCCAATTGTGTGCCGCCTCATGGGTCTCGAACCTG
GATCTGTAGGGATGCCTCTAGCACTACGATGCAGTGCCTTAGACCACTGTGCCACTCGGGAGGCCCCGCATTGATAATAA
AAAAACCATTAAACTCTGCTTGTGAGTGATCAAGTACATTATAAGTGCCTTGCAAAAGTATTCATACCCCTTGTATTTCG
TCACATTTTATTGTGTTAAAAAGTGGGATTAAAATGTATTTAATTGTCGTGAGTTGTTAAAATACTCTAATGTCAAAATG
ATATGATGATATATGATTTTTTTTATGACTAACCCTTCCTCTGTCCCCCATACATACGACATCTGTAAGGTCCCTCAGTC
ATGTATTGAATATCAAGCACAGATTCAACTACAAAGACCAAGGATCTTTTTGAAAGAAGGGCAGTGATTGGTAGATGGGT
AACAATCTCAAACCAGACATTGAATATCTCTTTAAGCATGGTCAAGTTATTAATTATGCTGTGGAATATGTATTAAACCA
CCCAGATACATTAAATATACAGTCGTCCTTCTGAACTGAGCTGCAGGACAGGAATGAAACTCTTCAGGGATGTTACCATG
AGGCCATTGGTGATTGTAAAATACTTACTGAGTTTAATGGCTGTGACGGGAGAAAACTGAGGATGGATCAACAACATTGT
CGTGACACAGACGGGCTTAAATACCCATCTAATAACTAAATAGAACACAGGTGCAACAAATAAAGGCAAAACCAACAGAA
AAGGAAAAAGGGATCGGTGGCAGCTAGTAGGCCGGTGACGACGACCGCCGAGTGCCACCCGAACAGGGAGACACAGTGTC
AATACATACTATAAACTCAGGGAGCTGAATACTTATGCAACGACTATATTTTTGTTGTTTAATTTCTATCAATCTTTCCA
AAACAATAGATAGAAATCTTCCACTTTAACATTAGAGTACTTTGTGTAGATTGTTGACAATTAAATCCATTTGAATCCCA
CTTTGTAACAACAAAATGTGAATAAATCCAAGGGGTATGAATACTTTTGCAAGTGCCAATAATTTGTTTTGAAGTACTTT
CTGAATTCCTGTTCAACTGCTTTATAAGGGCTCCCGAGTGGCGCAGTGGTCTTAGGCACTGCATCTCAGTGCAAGAGGCG
TCACTACAGACCCTGGTTTGATTCCAGGCTGTATCACATCCGGACGTAATTGGGAGTCCCATAGGGCGGCGCACAATTGG
CCCAGCGTCGTCTGGGTTTGGCCGTGGTAAGCCGTCATTGTAATANNNNNNNNNNNNNNNNNNNNNNNNNNNNNNNNNNN
NNNNNNNNNNNNNNNNNNNNNNNNNNNNNNNNNNNNNNNNNNNNNNNNNNNNNNNNNNNNNNNNNNNNGTGTTTTGATCAATG
ACGCACAATGCATTCGGAAAGTATTCAGACTCCTTGACTTTTCCACATGTTGTTACATTACAGCCTTGTTTAAAAATTGA
TTACACCGTTTTTTCCTTATCAATCTACCCACAATACCCCATAATGACAAACCAAAAATAGGTTGACATTTTTGAAAATG

TATTAAAAATAAAAACTCAAGTATTCAGACCCTTTACTCAGTACTTAATAAAGCACCTTTGGCAGCGATTACAGCCTCAA
GTCTTCTTGGGTATGACGCTACAAGCTTGGCACACCTGTATTTGGGGAGTTTCTCCTATTCTTCTCTGAAGATCCTCTCA
AGCTCTGTCAGGTTGGATGGGGAGCATTACTGAACAGCTATTTTCAGGTCTCTCCAGAGATGTTCGATCGGGTTCAAGTT
CAGGCTCTGGCTAGGCCACTCAAGGACATTCAGAGACTTGTCCTGAAGCCATTCCTGCGTTGTCTTTGCTGTGTGCTTAG
GGTCATTGTCCTGTTGGAAGGTGAACCTTGAGCGCTCTAGAGCAGGTTTTCATCAAGGATCTCTCTGTACTTTGCAACGT
TCATATTTCCCTAGATCCTGACTAGTCTCCCAGTCCCTCCTGCTGAAAAACATCCCCACAGCATGATGCTGCCACCACCA
TGCTTCACCGTAGGGATGGTGCCAGGTTTCCTCCAGACATGATGCTTGGCATTCCGGCCAAAGAGTTCCATCTTGGTTTC
CTCAGACCAGAGAATGTTGTTTCTCATGGTCTGAGAGTCCTTTAGGGGCCTTTTGGCAAACTCCAAGCGGGCTGTCATGT
GCCTTTTACTGAGCAGGGGCTTCTGTCTGGCCACTCTATCATAAAGGCCTGATTGGCGGAGTGCTGCAGAGATGGTTGTC
CTGGAAGGTTCTCTCATCTCCACAGGAACTCTTGAGCTCTGTCAGAGTGACCATCAGGTTCTTGATCACCTCCCGTACCT
AGGCCCTTCTCCCCTGATTGTTCAGTTTGGCCAGGCGGCTCTAGGAAGAGTCTTGCTGGTTCCAAACTTCTTCCATTTAA
GAATGATGGAGGCCACTGTGTTCTTGGGGACCTTCAAAGCTGCAGACATGTTTTGGTACCCTTCCCCAGATATATGCCTC
GACACAACCCTGTCTCTGAGCTCTACGGCCATTTCCTTTGTCCTCATGGTTTGGTTTTTGCTCTGAAATGCACTGTCAAT
TGTGGACCTTACATAGACAGTTGTGTGCCTTTCCAAATCATGTCCAATTAATTGAATTTACCACAGGTGGACTCCAATCA
AGTTGTAGAAACATCTCAAGGATGATCAATGGAAATAGGATGCATCTGAGCTCAATTTCGAGTCTCATAGCAAAGGGTCT
GAATACTTATGTAAATAAGGTATTCTTTGTTTTTTATTTTTAATAAATGTACACAAATTTCTACAAACATGTTTTAATTT
GGTCATTATGGGGTGCTGTGTGTAGATTGATGAGGGAAAAAAATAATTTAATCAATATTAGAATAAGGCTGTAACGTAAC
AAAATATGGAAAAAGGGAAGTGGTCTGAATACTTTCTGAATGCACTGTATATAAACCCTGGATGGAAAATGCTATATATT
GGGCAATGAGACTTTTGAAGCCACTGGTCGGCCATATTGGCACTCCACAGAAGGAATCCTCCAAAGGAATGAATGGATTT
CTACAGTATTTCAGTTAAATGTATCAAGGACAGAATTACATGCATACGTATTTTGTTGTTGTAGTGGGGAAAGGAACATG
AGTACTTTCAAAAATGATACTTTAAGGATATATCTTTTTTAATATATATAACGTATGTTTAACTCACATAATATAATTGT
AAAGTATGCATTAAGATGTTTGTAATAGAATACACGTGGGAAAAAAGTGTTTAGACATTAATAAATGCATTTCTATAGCT
TCCAAAATGTATTTTTTCTACTGTGGTGGGTGCGTGCCAAGATGTAGGCGCGGTGGCTTCAAAACTGTGCCCGGTGGGAG
TCTTCTAGTGTAATTAAAAAGAACTGGATTTGATCGATGAAAGCAGTTCTCATGTATTTCAATAAGACATACCTTTTTCT
ATTAGCAAGGTTTGATTGACAGAAGGTTGTCGTTGCTGCCTGTTACAAGTAGTTCGTTCACCTTTCGACTTTAGCTTAAC
GGGTAGCTCGCTAGATAAATATTTAGATAGCAAATGTATTGACAGTTGGGTTTAGTCGGTGACTACTTGCTAACTAACGT
TAGCAGCATTGCAAGCTAGCTAATGCAGCAACTGGTGGTGATGCATGGACTGAACAGTAGTCTCGTTGCAGCTGGATAGC
ACAACAGAATGGAACTTTGTTCTCTTGACAATAGTTTATCAAGCAAGGCAAGTGTGATAACATTAAATTATTTTGGCATG
TGTGGCTGTAGAACAGATACACGAGAGGGTTAGTTAAAACAAATATTTAGCTGTAACGTTAGTTTGCCAGGTTAGTTAAT
GTTAGCTAACTATTGGCAAGTTGATTAAATGTGGGCTATTTTGTCAGTTAGTTAACGTAACATGACTGAGTAAGGACTAA
GTTTGCTAACTATAACTAAATTAGCTCTAGTCTAGATATACAACACAGCAGCTAACAAACAAGCCACTATCCCAGACGAT
CGCAAATATGGCTAACTTGTTGACTAACGTTAACTAATTTACTAACGTTGCATAGTTAGGCAGCTAACTATCGAATGACT
TGCCAGACAGAATTTCAAATGAGACGATGTAGCTAGCTAATGCATCCATTATGAAATCTAATTTTGCAACGTTATACCGA
CAGCTTTGCAAATTGAAAATGCCATTGAATGCAATTGCACTGTTTTAACGTAATTAACTAACGTTAGTCAGCTACTATTC
AAAGTCACATTATTAGACATCTTGTGTGCATGCAATTTCAGATAATGTCTAGCATTGAACAGTTTAATCTATTGGTATTC
AATGTATCTTGTTGATGTTTTGTTTTACAGAGCACAATCCATGGAGCACGCGACATTAAATACCAGTCAAATATGGACCG
AATGATGATACGGAACGATTTTGTGCAGTTGTTATAACGAGGTAGCTAGCTAGCAAGCCAGAACATTGGATAAATACAAC
AGCGTTGCAAGTGAAACACCTACATAGCAGCTTGACAGCTACGCTTGGTCTGTCTGTGTGCGTGGAGTGCCTATGGCGGA
GTTTGGAGAGAACGACGGCTCATCTCCATCGACTACTGGGGATTCGGTGGCTTTGCCCAGTGATAAAACAGACGGGAAGA
CGCAGTGCGAAGTATCGGTCTTCAATGGGGACTGTGGGATACCGGCAGACATGGTTGGATCAGAAACGTTACCTGACAGC
CCAGGGCTTGAAACGGCTAACAACTCCTCTGTCGGGTTGGATTCCAAATCCGGAATACCACGCAGGAGCAGCATTATAAA
GGTAAAGCATATTTGTATGCAGATTTACACAGATTTGAGAACACAAAAAGAGGTCCATGGTTGGCTAGATTGTTAGCTTA
GTGGATGCATCCTTAGATTTGATCTTCCACTAAGTGTACATGTTATGCACATTGGTTCAGCAACCTCGACCAAATGAGCA
CTTGCAATCCAAAAACCACTGTTCACACATACATTAACAATGCAAACAGTCAGGTGAAAGTAGAGGTCTTGTCTGAATTT
CATTGCAAGTGTCAATTCACCATTCAGAGAAAATTAAGTAACTTCTGTGTTGTCAGCTCATGGTAAATATCTACACTATT
TGACTGAATTCTGGTTAGGCTACTACTAGGAGGGGATAAACCTACGTTATCAGATTGAGTGTTTTTCTTATAAAATAGCC
TAGGCCAAGTCCTCACTGACTAATGAATGAAATTGAAGATGGGGGACTAATAAACACCTTAATATGAGGACATAGGGAAT
TAGTATGGGCCAACAATGTCTAGGCTACAGGCATTCTTACAAAGACGTTCTGTTCTGTTCTGTTCTTTTGGAAAGCATTG
CTTGAGTGCTTTGCATAAACTGAATTTGGAGACCAAAGTGATCCGTTTGCGGCATCTTTCAAGCAACAATTTTGAGGGCT
TGACGTTTACCATATGTTGCCATTCCAAATTTGGGAAAGTCGGGACCAATTCCTGTGGTTAAGAACAGTTTCCAAATTAA
ATTGGTGCCATTGTTGCTTCTGAACACATTGGTTTGGTAAGCAGTTCATCACATGAGACAAGGCTTTTCCTCATTAAGAC
CTCTCAGCTTTGAATTCCGTTGTCTTAATTTAGATAGTGTTGTCAGTTGACCTCAATATTAGCCAACAACAGATGGCCTC
ATGAAGGAAATTATGCATTCACCTATTTCTCATATTGACTGTGTTGCAAGGTTGTTTGACTTGTTTTCCCCGAAAGTGAA
GGCTACAAATCCCTAAATAGGCCTACAAATTGCATTCAAGTGTAGCCTAGGTCTTTCAGACTGAGGCTATCACATTGCTA
AACTCACAGCTTTTTTGTATTTCTCTTATTGCTGCTCACTAGGCAGGTTTCCATTGCTTTTGTTGAATAAACCTGGGTCA
ATGTCGCAAAAAAAACATGGCGACTTGTAATGGAAACGGCAGCTATCGAAGAAATGTTCTAAAGATCAACAACATTTTCT
ATCCATTCAACGGGTGGATTTGTCTTGTCAAACTTTCTTCATTGTAAGAAATGATGATGGAAACAGTTTTTCAGAATAAA
TGAATAATTGAAGGTACTCTGGGCACGTGAAGTCACACGTAACTATAAAGCTTCACTACACATTTTAATTCTAAATGTCT
ACTGCTTGCAAAATCATTTTTTCCCCCAACTATAAAATGTTTATTTGCAGAACAAGGATTGTCTTGACTTTCAAGAATGC
CTGAATTGTTTTGCCTTGCTTTTCTGGTTGGCTGGATGCAAGTTTTACAATCCAATTATTTCAGCTCTACAGGAGTGCAA
GTTTCACAATAGCACAGACCATGGCGATACATTGGCACGTGAGAATTATTAGAATATGGCAAAGATGAGTCAATTCTTGC
ATTTTATCCATGCTGGCTTTTGCGGGCTACCTGAGACATGAAACATACTGTAGGTGGGAAGGCATACAGACTGTCACATT
TATTAATGCGCAAATTCCCTAAATGGATCATGGAAACAGTTCAACAACTTTTTTTTTATTTGGCACTCAAGGTTTTTGCG
AACATTGTAATTTTTTGGTGGAGGGGGTTGACGTCATTACGTTTTTTTGTCACAAGACAGTTCAGTAGAAACAAAGGCAG
TTGTTGCAATTTCTATGCAAACTTTATAAATGTCAACCAAAAATTGCTGGACAAGTTAATGGAAATGGCTTATGTAGGCC
TAGGTCTATCCCATATTGCTATGGATCTTTTGATTTTTAAGCTGGTTATGGCTTACACTCTAGCCCAGGGATGAGCAACT
CCAGTCCTGTGTGGCCTGATTGGTGTCACATTTTTGCCCCAGCTAACACACCTGGCTCCAATAATCAACTAATGATCTTT
AGTTTAGAATGCAATTAGTTTAATCAGATGTGTTTGCTAGGGTTTGGGGGAAAAGTGTGACAACCTCTTTGGCCCCTGAG
GACTGGAGTTGCCCATCCCTGCTCTAGCCTTCTTAGTGATAAATGTATCAGATCTGCAAGTCTACTGTGTAGCCTTATGA

ATGGATAGTCCACACTGTGACCAAAGTTGCATCAATATTTTTACACCAGCTAATTTCTGGACCAACTGTAGATGAGGCTC
TCAATCAACTATGTTATTCATTGTGAGCCTTTTCATTGTGTTATTGTTGATTTGCCAGCAATGAACACGGTTCTTTCCAG
TTAAGCTCTTTTCCAAGCCAGGTTTTGTGGGCAGCACTTTCTTGGCACATTGAAAGCGTTGAACTTTGATCCTCTCTCTG
CCTGGAGATTGATGGAGGAAATATTGACATTTCTGCCATGTAAAGTTCCAAGTAATCTCTCGACCACACAAGGACCTGTC
TCACTTCCCAGCTGTGAAAAACGATTTATTTTATCTGATGTCCACTCCTGTGACTCAATGTTCAATATCCTCTGTTTTAA
TAGGTCAATTATGTCTATTGAAATGTATTGGAGAGCAGAGAAATCTGTATTTACCCCCTCAAGATTTACTGCACATACTT
TCACTCACCTAGCTCTTGGTTGGTCCAGGAAGCCCACATTTAGGAAACCTTTTGTGTCAACAAACCTCTCATTACTATTG
AACAAAGCTTAATGCACTTGTCGTGCAAGAACTGACTGGTTCGAAGGTAGTCTACTGCCAAAGGATCCACTCAGCAAATG
ATCACATTTTAAAATGTATTTTATTTCACCTTTATTTAACCAGGTAGGTCAGTTGAGAACAAGTTCTCATTTACAACTGC
GACCTGGCCAAGATAAAGCAAAGCAGTGTGACAAAAACAACAACACAGAGTTACACATGGTATAAACAAATGTACAGTCA
ACTATAGAAAAATATGTATACAGTGTGTGCAAATGAAGTAAGGAGGTAAGGCAATAAATAGGCCAATAGTGGTGAAGTAA
TTACAATTTAGCAATTTACGCTGGAGTGATATATGTGCAGATGAGGATGTGCAAGTAGAAATACTGGTGTGCAAAAGAGC
AGAAACACAAATATGGGGATGAGGTAGGTAGGTAGTTGGTTGGATGGGCTATTTACAGATGGGCTGTGTACAGCTGCAGC
GATCGGTAAGCTGCTCTGACAGCTGACGCTTAAAGTTAGTGAGGGCGATATATAAGTCTCCAACTTCAGTTTTTTATTTT
TTATTTTTTTTGCAATTCATTCCAGTCATTGGCAGCAGAGAACTGGAAGGAAAGGCGGCCAAAGGAGGTGTTGGCTTTGG
GGATGACCTGTGAAATATACCTGCTGGAGCGTGTGCTACGGTTGGGTGTTGCTATGGTGACCAGTGAGCTGAGATAAGGC
GGAGCTTTACCTAGCAAAGACTTATAGATGACCTGGAGCCAGTGGGTTTGGCGACGAATATGTATCGAGGACCAGCCAAC
GAGAGCATACAGGTCGCAGTGGTGGGTAGTGTATGGGGCTTTGGTGACAAAACGGATGGCACTGTGATAGACTGCATCCA
ATTTGCTGAGTAGTGTTGGAGGGTAATTTGTAAATGACATCGCTGAAGTCAAGGATCCGTAGGATAGTCAGTTTTTCGAG
GGTATGTTTGGTAGCATGAGTGAAGGAGGCTTTGTTGCGAAATAGGAAGCCGATTTCAAACTGTAACTTTGGATTGGAGA
TGCTTAATGTCAGTCTGGAAGGAGAGTTTACAGTCTAGCCAGACACCTAGAATTTGTGGACAACTATAAATACCTAAGTC
AGAACCATCCAGAGTAGTGATGCTAGTCGGGCGGGCCGGTGCGGGCAGCGATCGGTTGAAGAGCATACATTTCGATTTAC
TAGCAGTTGGAGGCCACGGAAGTAGTGTTGTATGGCGTTGAAGCTCGTTTGGAGGTTTGTTAACACCGTGTCCAAAGAAG
GGCCAGATGTATACAGAATGGTGTCGTCAGTGTAGAGGTGGATCAAAGTATCACCCGCAGCAAGCGCGACCTCATTGATA
TATAGATGGATGGATGGATGGATAGATGGATAAAAGAGTCGGCCCAAGAATTTAACCCTGTGGCACCCCCATAGAGACTG
CCAGAGGTCCGGACAACAGGCCTTCCGATTTGACACACTGAACTCTATCTGAGAAGTAGTTAGTGAACCAGGCGAGGCAG
TCATTAGAGAAACCAAGGCTTTTGAGTCTGCCGATAAGAATACGGTGATTGACAGAGTCGAAAGCCTTGGCCAGGTCGAT
GAAGACGGCTGCACATGTGGCTTACCACCAATGTCCCAAATGCAATAAGGGACTGTAAGACTACACAATGACAGCCTTCT
CTGCCAGCCTTGACAAGTTGGTATAATACAACACTGGTCCATATGCAAATCAATATTGGGCAAATTTCCAGGCTAATCAT
GCTGGTCCTGTCAGACCAGAGTTAAAATGGACAACTCCCATTAGTAGCAGTGGGTTCTGCTACAGGTCTTGGACCTGTTC
ATAACTGTGTAGCCATATTACACGCTCACATCTCTCCATTCCACTTCCACCTTACACTGTAGCCTTGCAAATATGTCAGA
GTGAATAGAAAGTGATACCACCATGCTTTTTAGAAATGACACTGAATAAAAATATAAACGCAACATGCAATAATTTCAAA
GATTTTACTGAGTTACAGTTCATATAAGGAAATCAGTCAATTTAAATACATTCATTATGCCCTAATCTATGGATTTCACA
TGACTGGGAATACAGATATGCATCTGTTGGTCACCGATACAGTACCTTTAAAAAATAAAATAAAAAGATAGTGGTGTGGA
TCAGAAAAACAGTCAGTATCTGGTGTGATCACCATTTGCCTCATGCAGCGTGACACATCTTTCTCATATAGTTGATCAGG
CTGTTGATTGTGGCCTGTGGATTGTGGCCACTTTCACTTTCTGTCCTCACACTCATAAAATGACTGCTGATTTTCAACTG
TATTATGGAGCGCAAATCCCCATTTGCACAAGGTGAAAGTTTTCCTTCATTTTTTAACCTTTTGAAGTCAACATTGTTTT
TACATTTAGATTATTTACTCAACAAATTTTCACTGAGAGAGCAGCTTGCTGTGGAACGGGTAAGATATTTGTTTACATGT
TTGATTGGTCAGATAAGTGCAGGCTTGTGGTAATGGCTGGAGTAGAATAAGTGGAATGGTATCAAATACGTCAAACGCAT
CGTTTCCATGTGTTTGATGCTATTCCATTTGCTTCGTTCCGGCCATTATTATGCCTCCTGTGATGTGGGGAGAGAGCGGG
GTGAGGATGGAGGCGGCTTGACATGAAGCACTGAACATCATGATGTGAATTGGAATGTGTTTAGGATATTACATGCATTA
TTACAGAATGATGCACCAGAAATGTGTAATATTTTTAGAACATGGAACAAGGAAAAACATTATTAACCGGTTCTTAAGAT
TTGAAAATAATGGTTCTGTCCCGAACACTAGATATCACTTTTGTTTCTGTTCTCAAAATGGTTTTGTTCTTTTTTTTCTTT
TCTTTTTTTTTTAAAAAAGGTTCCAATCCCTGACTAATATAATAACCTACTTCTCTACTAAAGCAGATTTAGTTTTTCTG
CAGTGATGAGAGGATGTGGTCAGCAGTGGTGGAAGAAGTACCCAATTGTCATACTTATGTAAAAGTAAAGATGTCTTAAA
AGAAAACTACTCAAGTGAATGTCACTTTAGCCTAATAGTTTAAAATTATCTGTTCAGTTTTGACCAACCTTTGGAAATGA
CTGATTTTGAATGAGTTGAATGAACCTATGGCTAAATCATTTTTTTTCACAGTTAGTTCACTTAAGAAGGTCCCTCCATTG
TTTGATGGCTATTAATTCTCTTCTATATCTTGTTGCCAAACCACAGCTCAGAGCATGTCCCTGATTAGTGATCATCCAAG
CAAAATGTAGGCCTAGGATTTTTCCAACTGTCCACTGCAAATAAATCAGTCTTCTGATCTGGTCCAGGCAGAACAAAAGT
AGCAATACATTCTCTTCCAATTCAGATGCTCCTTATTTCAAGGGGAGCCTTATACTGCTACTTAGTTAGCTTAGGAATAC
CTTGCAGCAATTAATTTTTCTCCCTTTTCTCAAGATCAGGGTTATTCGTCGATATTGAGTAGTTTACATGACACAATCCT
ACACCAGTTTATGATGCCTGATATTTCTTGCACCCTAGTGCTAACCAAACCCAGTTGAATGTGAAATGTCACTGGTCTCA
TAGACTAGACGTAACATGGTAAATGTAAATCCTGGACACTGAAATTGGAATGGTATGTTACATTTGCTTTGGTTACATAA
GACGGAAGATTACTTGAGGCAAAAATGAAAGGAGGGTTTGTATGGTCGGGGTGGGTGTATAACGTGAATGTCTAGCAACC
CAAAGGTTGTGTGTTCGAATCTCATAATTTATCATTTTAGCTAAATTTGAGATTTTGCTACTACTTACTACTTTTTAGCT
ACTTTGCAACTACTTAGCATGTTAGCTAACCTTTCTTCTAACCTTAACCCTTTTAGCTAGCCCTAACCTAACTCCTAACC
TTGACCCCTAACATCTAGCAACCCAAAGGTTGTGTGCGAATCTCATCAAGGACAATTTTAGTTAATTAACAACATTGCAT
GTTAGCTAACGCTTTTCCTAACCTTAACCCTTTTAGCTAACCCTAACCTAACTCCTAGCCCTAACCTTGACCCCTAACTC
TAGCCCCTAGCCACCTAACTAACGTTAGCCATAACAATTTGGAATTCGTAACATATCCTACATTTGGAAATATGTAAAAT
ATTGTACGAATTGCAATTTGTAACATATACGAAATGGATGATGGACATCCACAAAATAATAAATGCCATACGAAACATTA
CATATCATACTAAATGCAGTGTCTCAGAATTGTATGCACAGAATAATACAAAATGCTCTGAGACTAGGTTGGAAATGTCT
GGTGTGTGGCTGCTATTACGGATGGGCATTAGAAATAATTTCACTACTCAAATATCATGATTCTTTCCCCCCTCAAATAT
CATGATTCTTTCCCCTACATGTTTTTAATCTGAGCCTTGCGCGAGGGGGAGACGAAGAGGGTTCCACTATGATGATGCTG
CTGCTACAGCAGTAGGCAGAGGAGGGTTCGGTTGTGTGAGTGTGAGATGGAGAGAGACAGACTAGAAAGTAGCCACGCTG
GCTCATGCTAGTTAGTTAACCAGCAGCTAATTTTATGTTTATCTATCAGCCCATAACAGTGCCTGTCTTGACTCTAGGGC
TGGGCGGTATACCGGTATTGATGCACGGACCAGTTTGGGTTTTTACGTTACCTTCTATAACGGTGTTTGAATGTTTGGTT
TGTTAAATGTGATATAGTTTACAAAGCTACTTTAGTCATCTTTCTCAGCTCTCTCTCCATGTCGCTATCCACACAGACCT
AGCCCCGCCCCCTGTCTCTCAAGGTGCAAATTTGTTGTTCCTCGACCACGAGACACTTGCGTTCAGTCTGCATGGTCAAT
GCAGAGCATGCAACAATGTTGATGACAATGATGCTGTTTTTGCTTTGCTTTTTAATATAAATCTATAATTACACTATTAG

TTTGTGATTCTTACATCTACAAACAGCAAGTTTGGCCTAAATATTGTTAGCCACTAATGCAAATTGCTAGCTAGCTAATA
AATATACTGAGTCAGAGAAAACGTAATTAGATATACAGCCTGATAATACCAGCGAACATTAAATGTAGCCAAAGACTATT
GAGTCCCCTAGGAAACACTTCTCAACACTTTGGTTCCTACCCTGTCACAATAACTCCTCTCTGGCATTTTAATTTGTTGT
CATGTCAAACACTGTATTTAAAGTGCCCACTATATTATATTCGAACTATAGAATTAGAATAATCATATTTCCATGATTCC
AACAGTTCACCCAAGTGTTTTGCTCTAAATCGCTAGTGAAAATCGCAATTGCAACATTTGGTTAAAAATAAGGCCTAGAT
TGTTTGCCCATGTCGTGCAGCCCTACATGGCAGTGTGGACATGATCTCAAATGAGTGCAGGAAATGGATAAATGCGTAAA
ATCATTTTTGTTTGAAGTTTAATTGAACGGTATAAAACAGAATGGAGAAATACCCATTGCAATCACATAGAATGTATGTG
TTGCCGCTATAGGGTCACACACTACTCATAAAGCAAATGTATAACTTGTATTATTCAAAAACATAAAATATTGTCATAAC
GTCCACTTTAAAAAAATAAATAATTAAATACCGTGTTATAATATTTTGGCCATATCACTCAGTCCTACTTGACTGCATCA
AATCGATAGCTAGCTGCGCAAAGTTAGCCAGCTAGCTATCCAGGTAAATAAGGCTACAATATGCTGCGCTCCCTGTCCTT
GTAAACAAATAACATCATAATTCAGATTAAACAGCCTACCTTGTTTGTTGTAGCCCACTCATTCTCATTTAGCTGACGTT
TAATTACGTAATTTCTTTCCATTTTGCATTGATTAGAGATCTCTTGCTACACATGGAACCTTGGACTGTAGAGCAGTTTT
GATTGGCTGACAAAATCAACAAGCTACATGTGTGTAGGTTACCGGCATAGATGGCTACCAGTATGCATGTCTTTTTAAAA
AAACATTATGGGGCACACGTCATACACTACATTTTACTTCAAAAAAATAAATAAATTAGGAGTTTCAAATGTCATCCTTG
TATGTAACAGTGTCATGTGGATAATTTACTTAAGACAAGGCATTTTCATTCCTAATTAAATTAGGCCTAAAAAAAATACA
CAAGTATTCAAATACTAACATCCGTTTCAGATATTCAAATATTTGAATAACTGTGCCCATCCCTAGCTACACTCTAAATT
AAATCAATTAGCAACAGACTGGCTCATGATTAAAATGTGCAAAGTTATTAGGGCTGTCCTCGACTAAAAAAGATCTTGGT
CGATCGAGAGTCGTCTGTTCTTTCGACCAATCAATTGGTAGAAATGTAAAACAGGGTGTGTCCATATATGGAAAAATACA
TATGTTTGAATACAGTCAACTATATGTAGGCTACTGAGCTTGTCTGATGCTTTAAGCACTGCGATTTAAAAATGAATACA
CAAATTACTGAAGAGAGAGCCAGAGATCAATATAGCCTAACCTGAAGAAAAAAAACCTGTTCCCGACACTCTTCCTCCCG
CTGCTGCTGGCCTTTGCAGATTATGCCAGTTTATGCTCCTGAAGTTGTCGGTAAAGGGCTACACCAGCGGTCAGCAACTT
TTTCCATATGGAGTGCAAAGTTATCATACTATTTCTACTGATCTGCATGCCAGTTATGATTTTCAAATTCACATTTTCAT
GGAACAGTTTTATATATCTCAATCATTTACATTTACATTTAAGTCATTTAGCAGACGCTCTTATCCAGAGCGACTTACAA
ATTGGTGCATTCACCTTATGATATCCAGTGGAAAAACCACTTTACAATAGTGCATCTAAATCTTTTAAGGGGGGTTAGAA
GGATTACTTTAACCTATCCTAGGTATTCCTTAAAGAGGTGGGGTTTCAGGTGTCTCCGGAAGGTGGTGATTGACTCCGCT
GTCCTGGCGTCGTGAGGGAGCTTGTTCCACCATTGGGCTGCCAGAGCAGCGAACAGTTTTGACTGGGCTGAGCGGGAACT
GTGCTTCCTCAGAGGTAGGGAGGCGAGCAGGCCAGAGGTGGATGAACGCAGTGCCCTTGTTTGGGTGTAGGGCCTGATCA
GAGCCTGAAGGTACGGAGGTGCCGTTCCCCTCACAGCTCCGTAGGCAAGCACCATGGTCTTGTAGCGGATGCGAGCTTCA
ACTGGAAGCCAGTGGAGAGAGCGGAGGAGCGGGGTGACGTGAGAGAACTTGGGAAGGTTGAACACCAGACGGGCTGCGGC
GTTCTGGATGAGTTGTAGGGGTTTAATGGCACAGGCAGGGAGCCCAGCCAACAGCGAGTTGCAGTAATCCAGACGGGAGA
TGACAAGTGCCTGGATTAGGACCTGCGCCGCTTCCTGTGTGAGGCAGGGTCGTACTCTGCGAATGTTGTAGAGCNNNNNN
NNNNNNNNNNNNNNNNNNNNNNNNNNNNNNNNNNNNNNNNNNNNNNNNNNNNNNNNNNNNNNNNNNNNNNNNNNNNNNNN
NNNNNNNNNNNNNNNNNNNNNNNNNNNNNNNNNNNNNNNNNNNNNNNNNNNNNNNNNNNNNNNNNNNNNNNNNNNNNNNN
NNNNNNNNNNNNNNNNNNNNNNNNNNNNNNNNNNNNNNNNNNNNNNNNNNNNNNNNNNNNNNNNNNNNNNNNNNNNNNNN
NNNNNNNNNNNNNNNNNNNNNNNNNNNNNNNNNNNNNNNNNNNNNNNNNNNNNNNNNNNNNNNNNNNNNNNNNNNNNNNN
NNNNNNNNNNNNNNNNNNNNNNNNNNNNNNNNNNNNNNNNNNNNNNNNNNNNNNNNNNNNNNNNNNNNNNNNNNNNNNNN
NNNNNNNNNNNNNNNNNNNNNNNNNNNNNNNNNNNNNNNNNNNNNNNNNNNNNNNNNNNNNNNNNNNNNNNNNNNNNNNN
NNNNNNNNNNNNNNNNNNNNNNNNNNNNNNNNNNNNNNNNNNNNNNNNNNNNNNNNNNNNNNNCCTGTATGAGCGGAATGC
ATTAACAGAAATTACCTTAACCAAACAAACATTGTAGATTTTAGAAATAATGGGAATTAATTGGTAAATGTACTACTGGT
GTGCCATCCCTGCGGCCTCTGCGATGGATTAGTCCGCACATACAGGCGTGAATCAGACGGGTGCCTCGTGTGCCATACAT
TTTTTTTATATTTGCCACTGCTCGACTAAAAAAAATCTTGGTCGACCAACAGCCTATCGACCAAACAATCGACCAGTCGA
CTTAATGGAGTCAGCCCTAAGAGCTATGAAGGGAACATGGAGCAAATGGATTGCGGGAGCAATACCTCATTGATCCTGTA
TTTACTTATCATTGGGTAGGCTATATTATGGTTGCTATTTCAGTAATCGGCCATCGGATGCATGTAGGCATACATAATTG
TCACACATGCACTGTGCATCACTGTTCATCATATTTTTTGGAATTCATATTTCACCATGTTATATAAATGGCTGCAAGAA
TATTAGAAACTAAGCACTGCGTCAATATTCTGCCTCGGTTAAAAGGACAGAGCCTAGGCCTCTTAGCCTAGTATGCTGCG
CAACATTTGCTGTCAGTGAAAAGCCGGAAAATATGTGTAGGACATAGAAAGTTCAAAAGCCAACCCATTTTCATGCTAAG
CTACTTGGTCACAGGTCAGAGGAGCAGTAGTACGTTTTTAGGGAAGACCGCAGTAGTGGTGCGTGGGTAAAATCACAGGG
GAAGCCAGGAAAAAAAGCAATATTACAACCTATGTGTTGTGATAATTGCATTGTTTGCTCTTTAACCTGTTACTTCATAC
GCCTTGCAACTGTGATATATATAGGCCTAAATGCAGAGACAATAAGAAGACCGTAGCAGAATAAATTCAACCACACCTTT
GTTTCATCAAAACAACAGACAGCAACCTCTGTCCAGTGAAGTCCCCAAAGCATATTGCATATAACTGACAGTTACATGAC
CTACAGCATGGTCAAGCAAAGTAATGTTTCTGACATTTTCGGACCACTAAACAACTATTGATTTAGAACCATGGAGAGTT
ACCACGAGTCTCAAAGAAAACAGGAGCTGCCTCCACTATTCCAGCACCATTTCAACTTCATCATCATCAAATCACCTACA
GTATGCTTAGTCTAATACAGTGACTACCAAAAAAGATTTGGTCCATTCAACGTAAGCTAATATGATGTGGCTGTCCATGG
TTCTGATTTCTGTGTGCGTATGCGTTCGTGCAAGTAGAGAGAACATGTTGACTCGCCTTACTTGAAGAGAAAGGCCAATG
CCATCCTTCTCATTCATGTTGATGAAACAGTCTATTACTCTGTCATACAGTACACTATTATTTTTTGTGGTCCTAGACTA
CCTGGATAAAATGCTTGTTCGCTAGCCTAACTTCCTTTCATGGGCAATGTTAGCCAATTAACATTAGCATTCTACATCTA
GGTATATATTGAACTACCATCCTCTCAGGCCAGGGGCACAATGTATGAATTGATGGTTGGATCAGAATTGCCGTTATAAG
CATTGACCAGTACAGAGAATTAAGTAAAAACACAAGTCCTAATCCCTATCTCCATGGCTAATTTAGGAAGGGGACAATTT
TAGCTAGCTTGCCACCAGAGGACAACACAACGAGATGCAACAATTTAAGTTGTTTCTGTCAATGACATTTGCTCTCAATG
CGATTTGATAGGAGTGACGCCAAACCCAAACTGGCTTCCATTGACACGTATTTTTTGTAATTTTTTTGGGGTGCGCCAGG
ACCAATTCACAGTTGAGCTGACGCTGATTAGCTATTATTTTATACTATTTTTATCAAGAGAAGCCAAATACTTGCTGGCT
TCCCTTGCATTCAATGGTACGGGCTGCATCATACTCTTTCGGACAAGACCAATGGCCTACACATAGAGACAGAGGGGCGC
TGTTTCGCTTGCTCAGATACTTTGTCAGATGAGATGCATTCAGCCTCTTGTGATTTGAAGGAAAATTATGAAAAACAAAG
ATATACATTTTTTTGGTATGTTTTCTTTCTTGGTAATTTTTGGGGGAAGTCTGGCTACCCTTCGCATCCATGAATACACA
CCACTGGAAGACTGTCCGTCCCCCATGTACCAGTATCATTTATTCATTCATCCACTCAGGCGTCACTCATTTACAGGGCT
GGGGACTTGACGGAGAGCTACCGGCTATAACCTGTTATTTCATCGGGACCACAGATGATTTGGCAGTACTCAAGTGGCTT
TACCAGGGTAATGTTTGTAGTAGACCACCATTAATTGAAGGGAAGTGCATAAGAGCGCCATAACGCAGGTATACACATTT
CTGACACCTTCAATTATTAACAGATCTTAGCATCTGTTCACTTTAAACCCAAGTCCGTTTTTGAATTGTATAGTGGTAGG
GAATAGGCAACTAGATTCAGCTGTGGTCTGATTTTTGTCAGAGCGGATGTTCGGGGGTCCAGAACATAATTATTTGTACA

CTGCAAAATTGAGTAGTAAGCCCAAAAATATATTGTATTTGAAAACAGTTTTGTACCTGATTTTTATATTGACCCGATCA
CATCTCTTTTTATTTGTGGGAATACTTGATATACATTTTAACTGAATTCCTGGTGATTTAAGTATGTTTTGCAGAAAAAC
TGAAATCCTTGGTGATATTAGTCTTGTTTGTTTTTTTTTTTACAAAAATCTTTGGGGGGGGGGGAAATCACTCACAGGCT
GGTTTTGGCCCATGGGATGCCTGTCACCGACCCCTGCCATACATCTTCAAGAGTTTGCCCAGGATATGACAACTGTTCGA
TAGCGTACATTTTGCTCAGTCTGCAGTCACCGGCTGCAATGTAGGCTAGTCAATCTCAAACACGGCCGGCATCTTTTGAT
CTGTGATTGAATGCTAGAAAGGCAGTCACTGTCACGACGCATTGGCATTGTACTGTAGAACTTTAGTACAATTATTGATC
TCACAAGATTTCTCTTCTCACATCCTAATTTGTTCATGATTGGCATATAGATTTCTTTGACCTGACCACATTTCCTCTGT
CCCAGCACCATTCTCTTCTCCTGTTTGACCTTTGGTCTCTTCCAGCTATGCAGCCGATAAATCAGCAAAACCGTAATCAC
AAAGTTTATGCAGGCTTTGGGCATCTTGATACCCTTGGCAAAAACTAAACAGTAGGGCCTCATTTTATTCACAGTGCAGT
ACTGTGGTGTTAAGAAGCCTTCCAGCAGGATGAGTTAGGCTAGTCTCATTAAAGATGCTGAACTATATTGAAGCTGCTCA
CTCTCCTTCATGGATATATATTGTGTGACACGACACCTATGATTTATTGCTGCTTGCTTCTTTAAAGTGATATTTTGGTA
AGAGTCCGATGAATTGTGGATACCATGTTTATGTCTGTCCAGTATGAAGGAAGTGGAGGCAGTTTTGTGAGCCAATGCTA
ACTAGTGTTAGCACAATGACGAAGTCTATGGGGGATCTGCTAGCATGGGTTTGGACCTATGCAATACGATGACTGCACAT
TTTGCCTTACTTCCTGGATCCAGTCCTATACTGTTTTTGAATGAGCTTTAAGATTATATAATCAAATTAATGAAAACACG
GTCATTTGAGATGTTTATATGAATTTATATTTGTGGATTAATTTACTTCTACCTAATACATTTTATAAAGATTTTTGATT
TCATATCTTATTTTTTTTTCTTTGCTTGAAGACCATTGAATAAACGGAAGTGGTCACACTTTCCCATCGTAAAGCGATGT
TATCACTGATATTGCACATAGGTCTACCTTTCATTTACCATATTGGCTATTGTTTCTCAAATAACATTATAGTCTAATAC
AAATCTGGTTCAGGTCAGATTAAAGAAGGGTTCCTAATTAGGGCAGGGCTCAACATTAACGCTTGTCCTCTTGTCCAGGA
CAACTTGAATCTGAGCGGAGGCGGTCAGCATTTTAAAACTATTTAATCATAATTGTTTAAAATCTGGATGTTTTATGTCA
TTTTATGAAGCATGTATTACCTTTTTTCAAAGTAGCGTAGCTACAATTCAGCCTTTTTATAAATGTTGAGGGAATTATTT
TATAAACACTTAACATGCTATTGAAACCAGCATTTTTCTCATGTTCTGTTTTTTTTTTAAATCTATTTTGTTTTATTGTC
CAGCAGCCAAAGGCATAATCCTAGTCATAGTTTTTGAGAACGGCGTTTTCCCTCTAATTGCATTTTGGAACATTTGCGCG
TCGCCTACAGCCATGTGTGCTTTGTTGAGCTTACAATATGAAAATATAAAACTAATCACATTTTTTTTGTAAGCTAAACG
ATCTGATCTGTCCTCCCAGAACTGTCCCCAGAGCCGGTTTTGAACCAAATACACATTTGTTTTATCACCTCCAATTTCGA
GCCCTGGAGGAACTTATTTTATAAGGCATAAAAAGTATTTGATTGTAATGTTGCAATATTTTATAGGCTACCAAGAGTGG
CCAACCATCCTTCAGGAGAGCGTTAAAGAGCCATCCTTCAGGAGAGCGTGTTCTATTTTGAGACAGGCTGGAATATGCAA
AGAAGCCAATATGCAGACAGTAGCCTACATTTTTGTCTGACTAATGCATTTCATTTTGTAAAGTGGTTTCTTGCATCATA
CAGGGATGTAAAAATGGTGTTACAGTACTAAATCAGTAATCCAAAGGACAAGTATCTAAAATAGTTCATGTCAAGCCCTT
ATGTGGGTCTCACCCCCCCACAGGTGGTCGCCAAAGTTCCCTGGTGGGTCACAGGACCTTCTTTGATTTGGGGCGGGGGA
CATTTTTTCTAATGTGGAAAAATAAACCTGGATTTAAGGACTCAAGCCTCTTGCTAGACTAGCTGTGAAAGTGTCTGTGA
AGTTGCTTAGCTTCCTCACACACTAGTGATGTATCCATTGATTGCATCACTCAAATCACACTCTGTGTGAGGAGTGCATC
AGGGATTGACTGGAGCTAAATCAATCAATGTGGATTTGGGTAGCTTCAGGGGCTCTATCGACGTCCTGCAGCTGCAGCAG
TTCTCTTCTGACTGTCTGTCTCTGTTTTGTCATGCCTGTCTGATTGGTAACCCAGCTTGGCCCTTTGTCATTCTGCTTTC
CTAAAGCTCAGTATATGTTCACTCAAGGGGTGTATGTTCGTTATGAAATAAGAACTGATTGGTGTAGTGCAGTGTTTCCC
AAACTCTCCACGGGACCCAAAGAGGTGCACATTTTGGTTTTTGCCCTAGCACTACACGGCTGATTCAAATAATCATCAAG
CTTTGATTATTTGAATCAACTGTGTAGTGCTAGGGCAAAAAACTAAACGTACACCCCTTGGGGTCCCGAGGACAGAGTTT
GGGAAACACTGGTGTAGTGGAAATGGCCCAATTCACTGGGGTTGGTAATTGCTTAGTCTTAGAACACTCTCCAGTCCACA
TGTGAATTTATGTATCGCTATAGTCAATGTTTTTGAAGTAGCTGTGAACTAATTGAAATGGGCAATCTGAATACTGCAGT
AGAAAAGAACCAGGCCCAGATAATTACCACAACAGGTATGCTTTGTCGTGTGTGAGGCACCCAGTGAGGAAGCCACCAGG
TGTTTTGGAGATGCTTGTGTGTTTACTGTTTTTGTTAGTTCACTTTTTGGATTGCAATTTCTTTCACCCAGGATGAGCTA
CTGAATATTAGGGACAAGTTACCAGGTGGTTTTTGTTCTGAGATTCTGTCCTCATCCGACGATATACTGCTGAAAACCCC
AGACCACGAGTCAGATTTCTGAGGCTATGCGACTGTGCTTTGGAGAAAGAGGGGGAGATGCGCCAGGGTTGAAGGTGAGG
TCTCCGTTTCCTGCTCCTAGGTCATTTCCTCTCCAATGTGCATTCACTCACCTCAAAATCGACATGCTACAACTTCTGAT
TAGAACAAAAAAGGACTTCTGTCACTTCTGCTTCACTGAATCTTGTCTAAATGACGACACACCTGAAGCTGCTGTGGATA
TACCAGGCTTCCAGCAATACTGGGCTGATGGTGTCCCAGATCTGTGCAAAAAATCCTAAGGTGGCGCTGTATGCTTTTAC
GTCAAACAACGTTGGTGCAATGATGTCACTTTACTGGCGAAATCTTTATATCCTGACTTGGAATGTATTTTCATCAACTG
CCATCTGTTTTAGTCACCACGTCCCTCGCCCCCTGGTTTTGGGGTTCCGGGGTCCCCTCCAGATAATTATGTATAAGGAC
TTTTTATCAGGACATTCTATTTCAAAATGAATGAAAAATAAAATGATGCTGACACTTCTAAAATATAATGTCCTCCTCTA
GAAATTATTCCAATTACATATTGGGTTTTTGTTACAATTATTTTAACATATTGGACCATACAAATAGCCTATGCATGGTC
CATATTATCAGGTATGCTAATAGCAATAAGCATTAATACCTGTAGTTGGGGCTGTTGCGGTGACTGTATTACCGCCACAC
CGGTGGTCACGAGTCATGAAGGCAGTCAGAGTCTACGTGACCATTTAGTCACGGTAATTAGGCTTCTCCATGCTCTGATG
CTGCTGATGGTCATTAGTAGCCTATCAAACTTGCTTAACTGCCTGGTACTCAGCACTCTTGTCCCTCTAATAACTCTGAC
ATCAATGCAAATGTAATCGAAAATCTAATCAAACACTTCATGAGAGCCCATGAGCTCATGTTGTGCAATGTTTCTATAGG
CTATGCAATTGCGTGAGAACAGAGTGATGGCCTCTACTAAAAAGAGGAGGATCCTATCAGCTTTCTATAGGCTAGGCCTA
CTATTTTTCTCAACTATTTTCTCAACTTTCCTAATATTAAGCACATTGCTTATCATTACAACATGAGTATAGCCTACCTG
GCTGGCATGAAAATGACCCATGGGAAAAGCATTCTCTATTTGCTATTTAATAGATTGCATGTATTTTTTCCACTGCCCCT
GTTTCGAGACAGGTGCATGATTTAATGGTTCATTCTAAATCCAAACAAATTTCACGCATATATTATTTATTATATGTAAA
GACAAGATTAAATTAAGAATAGGCTAATATTGTCACCCATCAGACTATCATTTGTGAATTATATATTATCACTGAATGAT
GCCCAGCATAAGGCAAGAAACAATGCCTTTTATTATTTTGCGCCTTTTCGAATCATCTCGTAGTATAGGCCTATATGTTT
TGAAGGTTTGTATCACAACTAAAGTGGGCAAATAACTTCTTAAAATGAAGCACATTAATCTGCTTTACAAAGGGTGTAGA
GCCTAACTGGCATACATAACAGCATGTGAGTTTCAAGGTTGGGGAAGATAATTTTCACCATAAAAATGCACCTTTATAAT
AAAAGCATTACATGTATCATCGCATTTGCGGTGACTTTTGATAATGGGGTTTTCCCGCTAATGGAACATTTTGCACTTAT
ATCATACTGCCATGTACACATCGCTGCGCTTATAATATGAAGAAATAAACTATAAAAATGTTAAGCTAAACGTTCTGATC
TGTTGCATCAGCCACATTGTGCTAAACGTTTTTGGGATGCTAGTGGTTGTATTAATTTGGGATCCGAATTGGATAAGGAG
GCGCGCAGTTGCATCCCCGATGTGTCTGTCTTCAGTTGTAACCTGTGAGAAAGACCTGATCACGTGATGGAGAGCCATAT
GAGTGAACGGTGCTTCAGAGCGCGCAACACTCAGGGAGAAGGGCACAACACAGCACTCCGAGCCAAGGGCACAACGGGTG
CATTACGGCCACACAAAGGGGATGTCGCCGTGAAATTCGAGGCATTATCAAGTGCTTGTCAAATTGTGAATGAGAGACTG
ATGAAGTGTGTACAGCCTGTGCAAAAAAAAAGCAGAGCTCATACCTTTCAAGCGACTTTTTTCAAATCATCATACAATGT
ATTAAAAATCAAAACATATAACCCAATGTTTGTAGAACAACTAAAGTTACATTAACAACACTAAATGAATCACAGAGAAG

TACCTATTTCTTTGTTCACCACCCAACACAGAATAGCCGCATGTGCGCACTCCCTCAAATTGTTTGGAGAAAATATCCTT
TCAATTTTATTCAGCTTTGTTCAATTGTATTTTTCATACTATGAAATCATATAAAATAATGCCACGGAATTCTAAGCAAA
TCTTGTCTGCTATATGAACTAGTGTAGCCCACATCCATTTGGCGTAGCCATGTCAGGACCTAACTTGGTCTCACGGCACA
TGGGGTAACATGCCCCCTGCCTGTACTTCTCACAGAAACCACTTCATGTCACCATTTCCCCAACACTTTCCCTGGTTGCC
ACTACTCTTACTCAACCAAAAATTGTCTCACAATTTAAGTCACTACCCAAAAATGATTTTGAGGTAGGGTGGCGTTTTAG
CCCAAGTTACCCTACAATTGCACCATGTTACATTTAGCCCCACTCCTCCCCTATGCACTCATAGTTAATTGCACAAGGCC
ATGACGCTGCTAAAATCTCCGGGTTTAAAATGGTGCAGTTTGCGCATATTTAGTACTTAAGAAACAAAGGCAATTAAAAC
GGCTGTTTCCCCTGCTATTGCAAGAATTGTTGTGCATTGACTGCTTGTCAGAATGCAGGTTTCCCTCCCTATGGCACTCA
TAACTTGAATGCGCCTTGAGAGGAATATTTATCTTGCATAGAATACACAACAACAAGCCGACTAGGTAAATCGATAGATA
TAAACTATTCTACTATGGGATTGTCTGGTTTTTCTATTCATTACTTATTTTGTTTGCAGAGGAAAAGTAAATGTGGACTG
TTCTAGCATCTTCATATTTTTTTTATGTTTCATATTATTTTAGCGTGGCGGTAATGATTTTGCCACCAAAATGACTGCAG
AGGAAACACTGTTGCTCCACCTCATCCCCTCATACAGACAGAAACTGAAACCTGCAAAACCTGTCAAACATACAGTGAGG
CAATTGGGGAGTTAAAGGACTGCCTGGGCTCCACAGATTGGGACATTTTTCAAGGAAGACCATATCAACATGACAACACG
GACAGGGTAGCATCTTACATCAGTGTCTGTGGAAAACTGCTTTCCAACCAGTACTTTTGTCACATTCAACAATGCCAAAC
GCTGGGTCAATCCAGAGCTAAGAGGCCTAAGCTCAGCCAAGGAAGAGGCACATAGGAGTGGCAACATGGACCATTACAGA
GTGACCCTACCTCCCTACCCCTTGTCAACAGCATCACAAACGTCAGGAAAGCTGCAGGCCCAGACTGGGTTTTCCCTGCC
ACAGTAAAGCACTGCGCTGACCAGCTCTCCCCTGTATTCACGGACACCTTCAATCCATCACTTCAACTATGCATTGTTCC
TGCCTGTCTTGAATCCTACATTGTTCCCGTTCCAAAGAAATAGAAATATCCTGCCTGAATTACTACAGACCTGTTGCACT
CACATCAGTCATCATGAAGTCCTTCGAGTGCCTTGTCCTATCCCACCTCAAAACCATCACTGACGCCCTCCTTGACCCCC
TGTAGTTCGCCTACAGAGCCAACAGGTCTGTGGATTACGCTGTCAACATTGGCCTCCACTGCATCCTTCAGCACCTCTAC
AACCCAAAGGACATACGTAAGGATAGTGTTTGTGGACACCATCATCCCAGATCTGCTGCAGGACAAAAGAAACTCACCCA
ACGGAACATATCCAATGGGGCAGTTTTAGAGGCCATCTTGGCTGCAGAGACAATGTTGCTGTTTTTAAAGCTAATTTCCT
ACGATTCTAATCAGTTTGCCATGGCAAAGTCAATGGGGGCCCCATGCCATACATTTTTTTTGAATTTTAGATTCTCTATC
TAGTTTTTATTTTGGTGATTTAGTTTTCAAAAATGATCTTTATTTAAAAATGTATTGCTCCATTATATTTTCAAAATACT
TTAAAACTGATTTTAGTTGTTAAATTTTACAATGAAAACATTTTACACTAAAAACATACCGCAGTAGCGGCCAGGATTTA
GCAGCGGTCATGTGCCGTTGCTGAATGCATATAGGGGAAACACTGCTTATTCATCGGCCACCTGCTCCACATTGATCTTC
CCACTCAGTCTATGCACCCTGCACTTTCATACTAGACTGCACATTGCTTTCTGCACTGGCTTAGGGCTCTGCTTCAGACT
GACTCCCACATACTGTGCCTTCTGAAAGTGTTCGGACCCCTTGACTTTTTCCACAATTTGTTAGGTAACAGCCTTATTCT
AAATTGATTATATACTTTTTCCTCATGAATCTACACACACTACCCTGTAATGACAGAGCAAAAACAGGTTTTTAGAAAAT
GATGCAAATTGAAATATCACATTTTACAGAAGTATTCAGACTCTTTACTCACTACTTCGTTGAAACACCTTTGGCAGCGA
TTACAGCCTCAAGTCTTCTTGGGTATGATGCTACAAGCTTGGCACATGTATTTGGGGAGTTTCTCCCATTCTTCTATGCA
GATCCTCTCAAGCTTTGTCAGGTTGGATGGGGAGCGTCGCTGCACCGCTATTTTCAGGTCTCTCCAGAGATGTTCGATCG
GGTTCACGTCCGGGCTCTGGCTGGGCCACTCAAGGACATTCAGAGACTTGTCCCGAAGCCACTTCTGCGTTGTGTTGGCT
GTGTGCTTAGGGTCGTTGTCCTGTTGGAAGGTGAACCTTTGCCTCAGTCTGAGGTCCTGAGCGCTCTGAAGCAGGTTTTC
GTTAAGGATCTCTCTGTATTTTGCTCCGTTCATTTTTGCCTCGATTCTGACTAGTCTCCCAGTCCCTACCACTGAAAACA
TCCTCACAGCATGATGCTGCCACCATGCTTCACCATAGGGATGGTGCTAAGTTTCCTCCACCCATTCCTCCAGAGTTCAA
TCTTGGTTTCATCAGACCAGAGAATATTGTTTCTCACGGTCTGAGAGTCTTTAGTTGCCTTTTGGCAAACTCATAGCAGG
CTGTCATGTGCCTTTTTATGGAGGAGTGGCTTCCGTCTGGCCACTCTACCATTAAGGCCTGATTGGTGGAGTGCTGCAGA
GATGGGTGTCCTTCTGGAAGGTTCTCTTATCTCCACAGAGGAACTCTGGAGTTCTGTCAGAGTGACCATCGGGTTCTTGG
TCACCTCCCTGACCAAGGCCCTTCTCCCCTGATTGATCCGTTTGGCTGGGCGGCCAGCTCTAGGAAGATTCTTGGTGGTT
CCAAACGTCTTCCTTTTTAAGAATGGTGGAGGCCACTGTGTTCTTGGGGACCTTCAATGCTGCAGAGATTTTGCTCTGAC
ATCCACTGTCAACTGTGGGACCTAATATAGACAGGTGTGTGCCTTTCCAAATCATGTACAAATCAATTGAATTTATCACA
GGTGGACTCCAATGAAGTTGTAGAAACATCAAGGATGATCAATGGAAACAGGATGCACCTGAGCTCAATGTTGAGTCTCA
TAGCAAAGGGTCTGAATACTTGTGTAAATAAATTATTTGTTTTTTATTTGTAATAAATTCGCAAAGATTTATTAACCTGT
TTTCGCTTTGTCATTATGGGGTATTGTGTGTAGATTGCTGAGGATAAAAAAAAAAAAAAACACATTTTGAATAATTCTGT
AACGTAGCAAAATGTGGAAAAAGTCAAGGGGTCTGAATACTTTTCGAATGCACAGTATAGAAACTCAAAAGAAACGTCCC
TTTTTCAGGACCCTGTCTTTCAAAGATAATTCGTAAAAATCCAAATAACTTCACAGATATTCATTGTAAAGTGTTTAAAC
ACTTTCCCATGCTTGTTCAATGAACCATAAACAATGAACATGCACCTGTGGAACGGTTGTTAAGACACTAACAGCTTACA
GACGGTAGGCAATTAAGGTCACAGTTATGAAAACTTAGGACACTAAAGAGGCCTTCCTACTGACTCTGAAAAATACCAAA
AGAAAGATGCCCAGGGTCATCTGCGTGAACGTGCCTTAGGCATGTTGCAAGGAGGCATGAGGACTGCACATGTGGTCAGG
GCAATAAATTGCAATGTCCGTACTGTGAGACGCCTAAGACAGCGCTACAGCGAGACAGGACGGACAGCTGATTGTCCTCG
CAGTGGCAGGCCACGTGTAACAACACCTGCACAGGATTGGTACATCCGAACATCACACCTGCGGGACAGGTACAGGATGG
CAACAACAACTGCTCGAGTTACACCAGGAACGCACAATCCCTCCATCAGTGCTCTGACTGGCCGCAATAGGCTGAGAGAG
GGGGTGCTGAGACTGTCCGCAATAGGCTGAGAGAGGCAGGTCCTCACCAGACATCACCGGCAACAACGTTGCCTATGGGC
ACAAATCCACCGTCGCTGGACCAGACAGGACTGGCAAAAAGTGCTCTTCACTGACGAGTCGTGGTTTTGTCTCACCAGGA
GTGATGGTCGGATTCGCCTTTATCGTCAAAGAAATGAGCGTTACACCGAGGCCTGTACTCTGGAGCGGGATTGAGGTGGA
GGGTCCGTCATGGTCTGGGGCGGTGTGTCACAGCATCATGGGACTGAGCTTGTTGTCATTTGCAGGCAATCTCAACGCTG
TGCATTACAGGGAAGACATCCTCCTCCCTCATGTGGTACCCTCCCTGCAAGCTCATCCTGACATGACCCTCCAGCATGAC
AATGCCACCAGCCACAGCCATACTGCTCGTTCTGTGCGTGATTTCCTGCAAGACAGGAATGTCAGTGTTCCGCCATGGCC
AGCGACGAGCCCGGATCTCAATCCCATTGAGCACATCTGGAACCTGTTGGCTCGGAGGGTGAGGGCTAGGGCCATTCCCC
CCCAGAAATGTCCGGGAACTTGCAGGTACCTTGGTGGAAGAGTGGGGTAACATCTCACAGCAAGAACTGGCAAATCTGGT
GCAATCCATGAGGAGGAGATGCACTGCAGTACTTAATGCAGCTGGTGGCCACACCAGATACTGACTGTTACTTTTGAACC
CCCCTTTGTTCAGGGACACATTATTCCATTTCTGTTAGTCACGTCTGTGGAACTTGTTTTAGTTTATGTCTGTTGTTGAA
TGTTGTTATGTTCATACAAATATGTACACATGTTAAATTTGCTGAAAATAAACGCAGTTGACAGTGAGAGGATGTTTCTT
TTTTTGCTGATTTTATTAACTAACTATTTTGCAGTCTTATGGTTTCGGGGTAGAAGCTATTCAGGGTCATGTTGGTTCCA
GACTTGGTGCATTGGTACTGATTGCCATGCGGTAGCAGAGAGAACAGTTTATTACTTGGGTGGATGGAGTCTTTGACAAT
ATTTAGGGCCTACTACACCAGCTTCAGAGCTGGTGTAGTAGGATTCGATTTTGGTTCTGTATTGACGCTTTGCCTGTTTG
ATTGTTCGTTGGAGGGCTTAGCACGATTTCTAATTAGCGTCCGGATTAGTGTCCCACTCCTTGAAAGCGTCCGGATTAGT
GTCCCACTCCTTGAAAGCGGCAGCTCGAGCTTTTACCTCAGATGTTACCTGTAATCATGGCTTCTGGTTGGGATGTGTAC

ATACAGTCAATGTGCTCCTACAAAAATTAAGGATTCTAGCTTTATTACCTCAAATGTTTTTGTCTGTCTGTAACTGTGCA
CAGCACCATTTCAGAGTCAAATTCCCGTACAATCAAATGTACATGGTGAATGAAGCTGATTCTGATTCATTTCACCCACA
ATTAACTTTGCTTAACACTAAAAGCATGTATTTTGCATGAACGCTTGCAAATGGGGTCTAGTTTCTTACAGGGAGAGCAG
CAGAATGAGTTTTAACTAAGCCCTCAATTTTGAGAGAGACACCTGATCCTGGAGCTTAAGCAGACGTTGCAATATTCATT
TCTGAAAGACCATGGCTTTAGCTTCAAAACAGGGTTTTTAAGCTAATTAATATCAGTTCAGGCACACACAATTCAGGAAA
TGTACTCTATAGCTCCCATGCTAGTTATTTAAATAAATAAATAAAGGTCAAATAAAAAATAAAATAAATACATGTATTTT
ATGGTTGATTTGACTCTTGAGGTCTGATACTGAATTACAGTGTGAACAGCTGGGCATTTGTCAGGTTCCTCAGTGGTATA
ACAGCTGTGGATTGAAAGTCTTGTATTTTAAGCATTGGCCCATATGTTCCACCTAAAACAGGCTTTTCCTCCATGTTCCT
ATATCCAACACCCACCATGTTCCTAAATATGAAATATTCTTGAAAGCACTGTTTGCCAATGTGATCAACCCTGGAAATAG
CCTACCATGAACATATCTGATATTTGCTGTGACTCCTAGCCTATTAATGCATCTAAATCCCCCTTTTTATATAGTAAAAC
CTAAGCCAGCATGGCTGACTCCTAATGGCTGTTTTCAGATGTGCTCTTTAACATGTGACGATGGTCTCAATCTGCTCTGT
GGATGTGAATGAACCCTGGTGTTTGGAGGTCAGAGTGGTAGATGAGGTTGTAGTCACAGAGTGGGGGCACGAGCCTTCTC
CACATAGGGAGGCTTCTTCACGTTATAGTCGGGGGGGGGGGGAGTTCAGTCTGGTCCGCAGTCTGGGCTCAGATGTCAAAG
CCAATGCTCTTTTCCATTTTGAGCATTTGATGTTATTGAGACGAGAGCCTGATAAGGGACAAAATGATGAGCAATATCCC
CTTTATGCTATAATAAGATTTCAGGTTACACATTTAGCTGCCAACCTATCTGCGCTTCTGGAGTTTAGTGCTGATGAATG
TCCCTTGGTAACATTATCATTTGAATGATTAAAGCGTTTTATAAAAGTCATATCGGTGTTGATGATTCAATAGTATTTAA
CCTAGCATAGATGTAGACTAATATCGATGATCACACTATGCAGCAGTTGTGGTTGGTCTCTTGTGTGATGCTCAAGACTG
CTAGCTCTATTCAATGCTCTTGGTCTGGACTGTTAATGGGTCCCCGGTGATTACTTCAGATGTACAGACAGTCCTGAACT
GCTCATTGAACCAGTAAAGGGGGTTTCAGTCTATTAAGGAATTCAGAATTCTATGGGGGTTTTTGGCAGCCGAACACACT
CAAATGTAATGCGGGGAGACAAAAATACTCTCCAAATCTCCCCACGCCACTAAGCCCTGCACAGGTTTAGCTAGGCCTAT
GTTATTAAATGTATCGAACATCATGTCTCTGTGGTTTTACTTTTCCCAGAAATACCTACGTACAAAATGACTGGAACTCC
AGGTGTAGACCTTTGCAGAATCTGACCTGCCTACGTATTAGGCCTATACATTTGCAAAGGAAATCAGGCTTTGCCAACGC
AGGCCTATGCGTGATGATAATTGGGCCTGTGTCATTCTTAATTACGACAGCACCTCAGATCAGCAGACTCCTGGGATTGT
CTGGATACAGCATCATCTTTCGAATAACAAAACACTGAGATTTTGTGTTACTTAAGAAATTGGGACATTTTATTATCCAG
TTAAAATCTATTAATGAAATGGATTGTGTACCAGTATGCTGGTAATAAAGCAAAAAAAGTAAATATCTCTGCTGAGCAAG
TAGGCTAAATTAAAACATTGGAGATTGTATGGTTGTGGTTATCGTCCCTAACAATTATGTTCTTGAGGATTCTGTTTCCT
TTTAGTATCAAGAAATGTTTCTCTTTCAGGATAATTTTGAGCCTTAATGAAATCATGTGTAAGTAACTAAAAACACCCTC
CTGACTTTGGCCATTACATAAGACACAGAATTGTATTTTCAGGAAATAAATAACCACAGTTCGGGGAGGGGGGGGGGGGC
GTTCTGTGGTATCGTTTTCTTGCTTGGGGAGCCGAAGCCAGTCCTCACTGAGTGCAAGAGGAAATCCTGATTAGTAGTCC
TCGCCTGGAAGCCGTTGGCCATTCAACAGGAACACTACTACACTGTTGCAGGCAGCCACACCTAAAGGCCAACACGAATG
AAAACTCCCAAAGACAAAGTCTAAATTCTTGTCCAGATCACATGGATTATCTTCAGTCATGCATTTTTGGATTTTTCTAA
GCCTCAGGAAAGCTCCTCCAGTAGCGGTTCTGTCTGGAGTGATGTACTTATTTTGAACACTTTACTTTTAGATTAGATCC
TTCCTCACAGCACAGAGATGTATGTGAAACTCTGGTTAGGTTCTGCAGGGGGTAGAAATGAAGTGGTGGGTCCCTGAGGT
TGATTAGTACCCATTTAAAGTACATCCTGCCCTCCCTTCAATTACACCACAGAGCAAGGGCGACAAAGTGTTCGATCAAC
ATGGCCAGAGTCCTGTCACCAGATGTGACGTGTTGATGCAAGAGATGAATGTGTCTCTGGAATGCCATGGCTTCAAGGAA
TACAGTTTTCATGGCTTCATGCCGCCAAACCCTGAAGAATGTCATGGTTGAGACTTTCACCTGCATTTGTAGACTAGGCT
AAATCTAACTCATAAATTCCACACACTTTGCCAGCTGATAAGCTTGACTTTTAAGATTTTGCAGATGGCCCCAAGGTATC
AAAGCTATAATGTTACTAACCGTTTATGGCAAAACCAGGCCAGCCGTGATGTACGAGTATGATCCAAGTTGCCAAGAGCA
AAAACGACCACTTTTTTTTTTTTTTTTTTACCTTTCCTTAAGATGTCTTGGGGCTACCAAGTGGCGCAGCGGTCTAAGGCA
CTGCATCTCAGGGCTAGAGGCTTCAGTACAGAACCTGGTTTGATTCCAGGCTGTATCACAACCGGCTATGATTGGGAGTC
CCATAGGGCAGCGCACAATTGGCCAAGCGTCATTAGGGTTTGGCCGGGGTAGGCCATCATTGTAAGTAAGAATTTGTTCT
TAACTGACTTGCTAGTTAAAGGCATTTCCTGAAAATGCCAAAAGAATTGAAGGCACGAGTAACATTTTAAAGAGTTTGTT
CATGTAGAGACAATGTTTCTCCTAGCAAGGGCTGGGAATTTCCAGGGACCACATGATTATATATTATCACGATACTTAGG
TGCCGATACGATATGTGTTGCGATTCTATGTATCACAATTCTGTGTTGCAATTCGATACTGTGTTTTTATTGTGATTTGT
TGTTCCAAACACATTTTTCACTACATGTTTGCTGCAGAGGAACAAGAGAAAGCCATGAGAGGTAAAAGTACTCAAAACAT
GTTGGCTCACCATTTAAAACAATGAAAAAGATTGGTATTTATCGTCCTATAGCTAGTTCCCAGAGTTTTGGGGCAGGTAC
ACTGACTAGTGATCGCTGACGTTACAAATCGATACTTGGAGTCACAGTATCAATATAAAATCGGCAAAGAAAATATCACG
ATAATCTACTGTATTAATGTTTTTTCCTTAACCACAACATACACTTGTGGATGAGTTTGTGTCTATCTAATGCAGTGTTG
ACCAATATTTTTATAGCGCGCTCCTCAATCTAGAATTTGGCGTTTTGAAACAAATAGTGATGCAACAGTCCTCCTTTTTT
TTACGCACAAAGCCAAGCCTAATTTAGAATGACTGGTCAATTTAAAAAAAACTTTCAGCTAATTTCTCACTGACTGGCTT
TGTTAAAACAGCTATGATGAGCTCTTATGCAATGTATAACGTGTAAAGATGTGCTAATGTATAGCTTGTAAAGATGTGCT
AATTAGAGGTCGACCGATTTAATCGGAATGGCCGATTTTCAAGTTTTCATAACAATCGGAATTCAGTATTTTTGGTCACC
GATTTAGCCGATTTAAAAAAAAAAAAGAAATATATATATATATATATATTTTTGTAATTAATTTAACACCTTTATTTAAC
TAGGCAAGTCAGTTAAGGACACATTCTTATTTTCAATGGCGGCCTAGGAACGGTGGGTTAACTGCCTTGTTCAGGGGCAG
AACGACTGATTTTTACCTTGTCAGCTCGGGGATTCAATCTTGCAACCTTACGGTTAACTAGTCCAACGCTCTAACCACCT
GTCTTACATTGCACTTCAAGAGGAGCCTGCCTGTTACGCGAATGCCGTAAGAAGCCAAGGTAAGTTGCTAGCTAGCATTA
CATTTATCTTATAAAAAAACAATCAATCAATCATAATCACTGGTTAACTACACATGGTTGATGATATTACTAGTTTATCT
AGCCTGTCCTGCATTGCATATAATCGATGCGGTGCGCATTCGCGAAAAAGGACTGTCGTTCCTCCAACGTGTACCTAACC
ATAAACGTCAATGCCTTTCTTAAAATCAATACACAAGTATATATTTTTAAACCTGCATATTTAGTTAATATTGCCTGCTA
ACATGAATTTCTTTTAACTAGGGAAAATGTGTCACTTCTCTTGCAACAGAGTCGGGGTATATGCAGCAGCAGTTTGGGCC
GCCTGGCTCGTTGCGAACTGTGTGAAGACTATTTCCTCCTAACAAAGACAGCTGACTTCGCCGAACGGGGGATGATTTAA
CAAAAGCGCATTTGCGAAAAAAGCACAATCGTTGCACGACTGTACCTAACCATAAACACCAATGCCTTCCTTAAAATCAA
TACACCGAAGTATATATTTTTAAACCTGCATATTTAGCTAAAAGAAATCCAGGTTAGCAAGCAATATTAACCAGGTGAAA
TTGTCACTTCTCTTGCGTTCATTGCATGCAGAGTCAGGTTATATGCAACAGTTTGGGCCGCCTGGCTCATTGCGAACTAA
TTTGCCAGAATTTTACGTAATTATGACATAACATTGAAGGTTGTGCAATGTAACAGGAATATTTACACTTAGGGATGCCA
CCCGTTAGATAAAATACAGAACGGTTCCGTATTTCACTGAAAGAATAAACGTTTTGTTTTTGAGATGGTAGTTTCCGGAT
TTGACCATATTAATGACCTAAGGCTCGTATTTCTGTGTGTTTATTATATTATAATTAAGTCTATGATTTGATATTTGATA
GAGTAGTCTGACTGAACAGTGGAGGCACCAGCAGGCTCGTAAGCATTCATTCAAACAGCACTTTCGAGCGTTTGCCAGCA
GCTCTTCGCAATGCTTCAAGCATTGAGCTGTTTATGACTTCAAGCCTATCAACTCACAAGATTAGGCTGGTGTAACCGAT

GTGAAATGGCTAGCTAGTTAGCGGGGTGCGCGCTAATAGCGTTTCAAACGTCACTCGCTCTGAGACTTGGAGTAGTTGTT
CCCCTTGCTCTGCATGGGTAACACTGCTTCGAGGGTGGTTGTTGTCGGTGTTCCTGGTTCGAGCCCAGGTAGGAGCGAGG
AGAGGGACGGAAGCTATACTGTTACACTGGCAATACTAAAGTGCCTATAAGAACATCCAATAGTCAAAGGTATATGAAAT
ACTAATCGTATAGAGAGAAATATTCCTATAATTCCTATAACTACTACAACCTAAAACTTCTTACCTGGGAATATTGAAGA
GTCATGTTAAAAGGAACCACCAGCTTTCATATGTTCTCATGTTCTGAGCAAGGAACTTAAACGTTAGCTTTTTTTACATG
GCACATATTGCACTTTTACTTTCTTCTCCAACACTTTGTTTTTGCATTATTTAAACCAAATTGAACATGTTTCATTATTT
ATTTGAGGCTAAATTGATTTTATTGATGTATTAAGTTAAAATAAGTGTTCATTCAGTATTGTTGTAATTGTCATCATTAC
AAATAAATAAAAAAAGAATCGTCCGATTAATCGGCACCGGCTTTTCTTGATCCTCCAATAATCGGTATCGGTGTTGAAAA
ATCATAATCGGTCGACCTCTAGTGCTAATGTGTAGCTTGTAAGGATGTGCTAACTGCTAATGTATAGGTTGTAAATGAAA
GATGTGCTGATTTCTTGTTGGACAGTGTTTTGTCTGCAGTCATTTTTCTACAAGACTAGACAAGTGGGGGTTACAGTAAA
GTTTAGACCTCTGCCAGTTATGGTGTTAAACAAAATAGCTCACCAGAAGTTCTTCATCACAATTGCCACTTATTCATATG
AATACTGAAGCTTTCTCAAAGGTAGAAGATACATATGCTACAATCTGTGGACCATACCACCCAAACATTTTTGCGATGGT
TTACGTTTTGGAATCCGGTCATACAATTGAGATGTAGACCTATAGAGTGTATTGGAGGATCTTTCATTTCTCTGTGATGT
AGTCCTATATCTCTGAACATGTCGCCCAGCACTGTTTTGAACATGAAATCCTTTGAGTTGTACCTTTTGTTTAACCCTCT
CAGTGTACACCTGCAGACTATAGAATGAAAGAAAAGCTCTAGCGCTGCCTGCAGAGGCTTGTCTGTCTTTTTCAAAGTCC
AATCATGGCTTACTGTGGCCTTCCCTTTGTCTCATTTGAACCTCAAGCCTGAGAGCGAGGGACAGGAAGTAGCTGACTAG
AATAGATAAAATGTCAGGATGCTTGTTGCTATTCTTTCACCAGGGCGGAGAACCACAGCATCAGACGGGTCAACACTTTC
CTATACCGTACTAAATAAAATATGTGATCTTGCCTACTATTGTTTGTAGGCTATGAAGTCTGTAGTGTACTCACAGCCTG
TGATTGTTATTTAGCTAAATCTTATAGCAAACTTCAAGTGTTACAACAAAATCAACTGAAACACTAGGCTATGCAACCGT
TTGACAAAAATTAAATTTGAATTTTCTTCATCAGCAAAGCATGTTAGAATTTCTACTCAAACTATCTTGCCATTCAACAG
GGTTGATAGGAATACACTGAAAAAAATATATATAAATGCAACATCCAACAATTTTTAAAGATTTTTATTGAGTTACATTT
CATATAAGGAAATCAGTCATTTGAAATAAATTCATTAGACCCTAAACTATGTATTTCACATGACTGGGAATACAAATATG
CATCTGTTGGTCGCAGACACCTTAAAGGTAGTCGTGGATCAGAAAACAAGTCGGTATCTGGTGTGGCCACCATTTGCCTC
ATACAACGTGACAAATCTCCTTCGCATAGAGTTGATCAGACTGTTGATTGTGGCCTGTGGAATCTTGTCCCACTCTTCTT
CAATGGCTGTGCGAAGTTGCTGGAAATTGGCGGAAACTGGAACACGCTGTCGTACACGTCTATCCAGAGCATCCCTAACA
TGCTCAATTGTGACGTGTGGTGAGTATGCAGGCCATGGAAGAACTGGGACATTTCCAGCTTCCAGGAATTGTGTACAGAT
CCTTCCGACATAGGGCCGTGCATTATCATGCTGAAACATGAGGTGATGGTGGCGGATGAATGGCCAAACAATGGGCCTCA
GGATCTCGTCATGGTATCTCTGTGCATTCAAATTGCCATTGATAAAATCCGTAGCTTATGCCTGCCCATACCATAACTCC
ACCGCCATCATGGGGCACTCTGTTCATAAAGTTGACATCAGCAAATCGCTCGCCACATGACGTCATACACGTGGTCTGCG
GTTGTAAGACCGTTGGACGTACTGCCAAATTCTCTAAAACAACTTGAGGCAGTTTATGGTAGAGAAATGAACATTAAATT
ATCTGGCAACAGCTCTGGTGGACATTCCTGCAGTCAGCATTCCAATTCCACACTCCCTCAACTTGAGACGTCTGTGGCAT
TGTGTGACAAAACTGCACCTGTGTAATGATCATGCTGTTTAATCAGCTTCTTGATATGCCACACCTGGCAGGTGGATGGA
TTATCTTGGCAACGAGAAATGCTCACTAACAGGGATGTAAACAAATTTGTGCACAAAAGTTTTAGAGAAATAAGCCTGTG
TATGGGACATTTCTGGGATCTTATTTTAGCTCATGAAACATATTTTTTGTTCTGTATATATTTGGCGTTGTTCATAGTCA
ATCATAGACATTAACTGCCGAATATTAAAGTTTGTCATTCAACTCTTCTATCTAAGAACAGGTTTGACAAAACTAGATAC
TCTACAAAAAGGGAAGTCGGATTCTTCTTGACCCGAAGATAATACATTTTCAATGGACTTTGCCCTGGGGGTGAAGCACA
GACTTTCATGAGAGCCATTACTCTTAGTAAGTGCACACAAGCGTGAGCAAGCTCACATGGCAGGCCTTGGGACATTGAAT
GTCGCAGGCCATTAGATCAGGGATGGCAGTGCTTTGGTTGCAGGACGATGGACGCTAAAGGCTTCCCCACATCTAAAGTG
CCATATGCATGCTGTTGATTTCAGTATAAACAACTTACATGAGAACTAGCCGCCCGCATGTCTTTTTCTGTTGAATGCCC
ATTTTCAACTTCCAGCTGGCCTTAAAAAAAGGCATCAATGTGTATTGGGTGAAAACAAATTGGAACCTCTTAGTAATATC
ATGAATCTTTTCATCACCAAATGGAATCTGTTCATTCATGTCAGGCACTTTCAGCGTGCATTGAAGACTTCATCTGATGC
TAGTCAATTGATGCAGAATATATGTTTTATTTAGTTCAGCTTGAATCTGGATGAAGGTTGAAAATAAAGTGCTGGAACAC
AACAATGACACGGACTACTTGGGAAATGTCACGCACGTGACTACAGAGACTACTAGATCTGTGTGAGCTATATCCTTTTT
AGGGGGGACTCATTGTGGTTCACCTATAATGCACTGTGACAGGCACCTTAAGTCGAGCAACACAATTGTTCAAGGAGTTC
CTTCAAGGTAACGCCTCAGTTCTCAAGAAAAATAGAAGGGGTTGGGGGGCGTTAGGAAAGACGTATTCACAAAAGATATG
TCAACAACCATTGTTCTTCACATCATGTGACGCACCATATATATGGTATGCTGCTCTCAACTTTACTTTGACCTCTGTGT
TAGCATCTCATTAAATCAGTTTATTGGATGCGTACACAGTTTAGCAGATGTTATATTAAGTGCAGTGAAATGCTTATGTT
ACTAGCGCCTAACAATGCAGTAAAATGTCAAATAATCCCCCCCAAAATGTCGGAACGAACCCGAATAGCACTGTAACAGT
AATCCAAATGCAATGTATACATATATACACTTGATGAATTTAAACAAGATATACTAAGAATATGTACAGCAGTAGATACT
GTATATTAGAGTGAGACATGATGCATTAATTGACCCACAATAACAATTGTTTATGTTCCCTCTCAGCCGAGTTATTTTAA
GCTCTCTACAGCCTATAGTTTCAGTATGAAGCATTTATCCAAGGTCAAATATTTATAGACATTAGAATAGTTCCTTAGAG
GATGTAATTCATCTTAAAATGTTGTGCTGCCTGCTCCCCATAAAAAGACAGGTAGCCGACCGGTAGCCTTCAGGTGTCGT
GTGTAGCTTGTTGTTTTATGTTGGCCAATCAAAGTGGCAACGAAGGCTCAATATGTAGCAAGTGGAGTGAGTGTCCACTA
ATTCAATGCAAGATGGAATACAATTATGGAATTAAAAGTTAGCAAAATGAGAAGTAGGCTACAACGAGCAACCAACAGGT
AGGCTATTGTTTTATCTGAATGGGGTTGGGGAGAAAGCGCAGCATTTAGCCTAGCTAGCTATAGCTTCTCTAGGCTGCTC
CAGTCAAGACAGGTACTGTTGTATGGGATGATGCATAACATTGTGGCTGCTACAAGTTAGCTAGTCTTGGCTATAGCCGA
GATCTCTTTCTCTCTACTCTCCTGTCTCTAATATTCCAGTCTCTGACATTGCTCGTTCTGATATTTCTTTATTTTTCTGG
ATTGTCTTTTATTGCTAGGTATTACTGCACTGCTGGAGCTAGAAACACAAGCGTTTCCCTGCACCTACGATAACATCTGC
AAATCTGTGTACACGACCAATAAAATGTTGTTTGAGCCTCTCTCCCTATTCGTATAGTGAAATGTATTTAAAATCCCCAT
CCCTACTGCCTTAGCCCAACTGATTGTATCCCATATACTGGCCTCTTCCTGTTTGATCTGAGTGTGACTTGCTTCCACAG
AGTCAAGAATTTAACTGCACGCATTTCACAGAAATGTTGTAATTATTTTCTTTATTGAACCAAGTTTAGTTCGATAGTTC
TCTCCCTCTGGGCTCTGAGAGGTAGGGCTGAAAAGGAAACATTGATGACCTCTAGACCATAAAGGCCTGCCATCACCAGC
AGCAACATGGACAAAGAATTAGCAGGCTGCCATACTGCTGCATTATGCCCAATATTTGAACATTATGTTTTATACTTCAC
ATGGCTATTTTAAGAAATTATATCAGTGTTCTTTTGGTCATCTGAAACTGTGCTTAATTAATGAATACATAGTTCCTCAG
GGGTTAAGCCTGAGCGGTAAAATACTGTTTTATAGAGATTCATCTAGTAAATAGTTTTCTCTATGAAAAGCAATGCTCAC
GATAATGCAGTTAGAAGTCAAACACTGACATTAGGCAGCTCCAATCATCAAGGAATGCTAAGGAGTTAAATAGATTTCAC
CGCAAACGGCAGTGAAATAATCAACCCATTTCAATATCCAGACACATGAGCAAATGCTTAGTCATACAGAGTCGGGAATA
CCCCCACTCCCACGTTTTCAGTTGGCCTATAGTATATCTCAAATCAAATATTTTGCTTCCTGCTCAGAGAAGTGATTGAA
AGAGTATTCAGCTCCTCTTTTTACCTCTAAATCTAAAGTGATATACTTATTTTCTACTGGTTTACAGATCAGGTATTCAG

GTTTTTGCTTTTCTCTTCCTTAGGATGGCTCGAGGCAGCGCAAAGAGAGGAAGAAGACGGTTTCGTTCAGCAGCATGCCC
ACTGAGAAGAAGATCAGCAGTGCGAGCGACTGCATCAACGCCATGGTGGACGGCTCTGAACTGAAGAAGGTCCGCTCCAA
CTCCCGTGTTTACCACCGCTACTTCCTCCTGGATGCCGACATGCAGTCTCTAAGGTGGGAACCCTCCAAAAAAGAGTCAG
AGAAAGCCAAAATTGATGTCAAGTCCATCAAAGAGGTGCGGACAGGGAAAAACACAGAAACTTTTAGGACCAACGGAATA
TATGATCAGATATCAGAGGACTGTGCCTTCTCAATCATCTATGGGGATAACTATGAGTCTTTGGACTTGATTGCAAACTC
TGCCGATATGGCCAACATATGGGTGACCGGGCTTAGATACCTGATATCCTATGGGAAACACACCTTGAATATGATTGAAA
GCAACCAGAACAACATGCGCTCCTCCTGGCTCGGTGACCTCTTTGAGGAGGCTGATGCCAACAACAGCAAGCACATCAGT
CTATGTGCTGCTGTTCAGCTAATTAAAAACCTAAACCCTGGTCTCAAAAACATCAAGATTGAACTCAAGTTCAAGGAGTT
TCACAAATCTAAAGATAAAGTGGGATGTGGTGTGACTAAGGAGGAGTTCATTGAAGTCTTTCATGACCTTTGCACAAGAC
CAGAAATGTATTTCCTTCTTGTCCAGTTCTCTAGCAACAAGGAATTTCTAGATACCAAGGACTTAACTATGTTTCTGGAG
GCTGAGCAGGGTATGGCACAAGTAAGTGAAGACACCAGTCTGGAGGTCATTCAGAGCTATGAACCTTCCAAAGAGGGGCA
GCTCAAGGGCTGGCTCTCCCTTGATGGGTTTACCAATTATCTCATGTCGCCAGAGTGCCACATCTTTGACCCTGAACAAA
AAACAGTGTGTCAGGACATGAACCAGCCCTTGTCCCACTATTACATCAACGCTTCCCACAACACATACCTGATCGAGGAT
CAGTTTAGAGGCCCCTCTGACGTTACAGGCTACATCCGTGCCCTCAAGATGGGCTGTCGTAGTGTAGAACTAGATGTCTG
GGATGGACCAGATAATGAACCTGTCATTTACACTGGCCACACAATGACCTCGCAGATAGTCTTCCGCAGTGTCTGTGACG
TCATCAACAAATATGCCTTTGTTGCATCTGACTTTCCCGTGATATTGTGTCTGGAGAACCACTGCTCCTTAAAGCAGCAG
AGGGTCATGTTTCAGCACTTGAAAAAGATTCTTGGTGATAAGATTCACATGGATCCACCCTCATCTGAGGACAGCTACCT
GCCCTCACCCTTTGACCTCAAGTATAAAATCCTGCTGAAGGGGAAGAAGCTGGGCACGATCTGCATTAGCTCGGAGGGCG
AAGTGACTGATGAGGATGAGGGGGCTGAGATGTCCCAAAGAATGAACATTGAAGCCGACGAACAACAGAGCATCCCACTG
AAAAAGTTCCAGCTGTCCAAGGACCTCTCTGACCTGGTAACGTTGTGTAAATCGATTGCGTTCAAAGACTTCCCAACAGC
TTTCCAAAGCCAGAGGCACTGGGAACTTTGCTCGTTCAATGAAGTTTTTGCCAGTCGCTGTGCCAGTGAATTCCCAGGTG
ACTTTGTTAACTACAACAAGAAATTATTGGCGAGAGTCTACCCCAGCCCAATGCGGATTGACTCCAGTAACATGAACCCT
CAGGACTTCTGGAAGTGTGGTTGCCAGATCGTGGCAATGAACTACCAGACCCCCGGCCTGATGATGGACCTAAACATTGG
CTGGTTCCGTCAGAATGGAAACTGTGGTTATGTGCTGCGACCAGCCATAATGAGGGAGCAGGTGTCATATTTCAGTGCCA
ACACTAAAGACTCAGTGCCTGGTGTGTCTCCTCAGCTCTTGCACATAAAGATCATAAGTGGACAAAACTTCCCCAAACCC
AAAGGCTCAGGTGCCAAAGGAGATGTTGTTGACCCTTACGTCTATGTGGAGATCCATGGCATCCCTGCTGATTGTGCAGA
GCAAAGGACTAAAACTGTCAATCAGAATGGGGAGAACCCACTGTTTGACGAAAGCTTTGAGTTTCAGATAAACCTACCTG
AGCTGGCCATGGTGCGCTTCGTGGTGCTTGATGATGACTACATTGGCGACGAGTTCATCGGCCAGTACACAATCCCCTTC
GAGTGTCTCCAGCCGGGATTCCGGCACACACCATTACAATCGCTAACGGGGGAAGTCCTTCCCCCACGCCTGGCTGTTTGT
CCATGTGGCCATCACCAACCGAAGGGGCGGGGGCAAGCCTCACAAGAGGGGACTGTCTGTGCGTAAGGGCAAGAGAAGTC
GGGAGTACGCCACCATGAGGGTGCTGGTCATCAAGGCTGTGGACGACATCTTCAAGACAGCCATACTGCCACTGAGGGAA
GCCACCGACCTCAGAGAAAACATGCAGGTAAGGGAGTGAACATTATTTGTAATAAGGGTTACATGGAGAAAATCGGACCT
CTCTGAAATGAAACTGGATGGCCCTCCCTTAAGCAAAATATATTTTACCTAATCCCTCACTGAACATAAAAAAAATAGGA
AAAAAAGTGACCATCCCCTATACCCAAAATAATAATTAAAACAACGTGGATAGCAGAGAACATGTCTGCCGTTTACCCTC
ACGTCTTGGACAGACCAGAGCATTACATGGCAACGCAGGAATTCTTTATAGTGCACAGGGCTGCGGGCCAGAAGGTTATG
GGTTGGCAGATCACCATGGACAACAGTAGGGATAGAATGATCTCCTTCATAGTAAAATCATTGCGTGAATATATTATTGT
ATTTGCAGGTCTGAGGATTTATTTTTTATTTTTATAAAAATGTCATGCAATTCTACATAATCTTTTTTAATACCACACTA
ATTACCAAAATTACAGGCTAAGGATGGACTGAGCAATAGCTTATGTGTTCATCTTATCATATTTCTCCAATGCCAAATCA
GTGTGTCTGGTTTGCAACGAAGCTGTCCGTGTTTGCAAATGATTAGGAATCTGAGCATGGGTTCTTTTAAAAGTTAGGCC
TACCTTTCCACCCCAGACAGTAGGCCTACCGACTCATACAAATGTAAGCTTTATCTGCTGGCCACTTTTCTTCTGTGGTT
TAACCCAACGAGAGATGGTGAGAAGTGTTTCCCTAAAAGCTGTCTGGGTTGAAACATCTTCTATTGTACACAGTGGACAG
CTTAATCAACTGATAGTGACAGAATTAGATTACTTCCCAACCAAAGTCTGTTTTGTCTCATCGGCTATAGAAGGTTGTAG
CTCAGCCTCTTAATGTCAAAGAAAGGATGCATCGGAGTCACGTCTTTCCTGGGTATTTTACAGCTTGTTTCTAGCATAAA
ACATTGCGGACCAAAGCGGCGTTATAGATCACTTTACATAATCGGATCCCATTTTATTTTCTTCAAAATCTTATGCCAAC
AAGGGGTAGCCCTGTGCTTTTTGCCATATTTAAATAGAAGATAGGCCTATGGCATACCCTCTCAACTTGAGTGTTGATTT
TAACTTAACAGCCCTTCACTGACACGGGGTAGGATATTTAAAGGATGCATGATGGGTGGAGGAATTGACTGACAAATCTA
TGGCTATAGCAGCCTATAGCCTAATGTAGTCTGTCAAACAGGTGGGCCTGGGTAACTATTCTGTAAATTTCGATCCATCC
CTAAGGATTGACTTGTAAAACATCACCAATTTATGCCCAGTTTCTATTGGCCAGGCTAGCCTACATGATGTCATATTTTG
GGGGGAATGTGCATGCTTTAATAAAGTCGTGGATGAGTCGTTTGAATCAAGTTTATTTACTCACCCGCCTACATGGGAAT
GTTTTTGTTAGAATATGAACACTGACCCCTAGAGTCAAGCGGGGATCACAGCGAGATTTACTACAAAGGCTGTGTAGCAT
CTAAAAAAGAAAGCCCCTTTTGGCAAGAGAAAACAAAACCACCAAGCTTGATTTAATCAGATTTAATCTAATCCATGTGT
ATTGCACAACAGTCGAGGCAGACTCAAGGATACTTCGAAGCTCAGAGGCAGTCTGGTGACATCCCCACTGGAGCACTGAG
GACTGGGGCTGATCTTCATATATCCAGGTAGTGGCTTATGAGCATACCTTCTAATATATAAAATGGAAAACGTGTTATGT
AATACTGCGCTGTGGCGCTTATGGGCTTGTCAGTAATAATTCTAGAAGGAGATTACTGTATGCAGTCTAAAGGACAGATT
ACCACAAATTATGTTGAATTGGAGTGGAAACGAGACTAGTGCGTCACCTATTTTAAAAGGGCAAAAAACAGATATGGTGT
TTTACTTCAAATTTGCACTCAGTAAGCTGTTAGGAGCAATGATCAATCTTTTATTGGTTGAAACAGGACATAACAGAAAG
ATATCGACCAAAAGATGAACAGTAGGCTATTGTTACCCAGGACCCACCTGACAATTTGCCTGTTCAGACTATCCATCTGC
TTTTGTGCATTCCTGTGTGCTCACTAGCTTCCCACTAGGCCCCCCCATGAGCTAGCTACTTCAAACAGCTGGTCTTTTCT
GATCTTCGCCATCTTTTTCCCACTGAAGCAATACCCAGCAATGTGTCAGAAGGAAGTCACATACTCGATTGTTCCCCCCT
CTGTGGTTGTTTTTTTGTGGTGCATTGTGTATTAGGGTTTATTTTGGTCGCTCTGTTTACATTGCAGTCATTATGTGATA
ACAGGCCACTGGCAATGAGACTAACACATCTTGGGCGTTCTCTCTGTGGCTGTTTGTCCTCCCGGTTGTTTACACGCTGT
TTAAATTGGGCCTTTCACAGGAAGGTTAGGATTTGTGTGTGTGTGTGTGTGTGTGTGTGTGTGTGTGTGTGTGTGTGTGT
GTGTGTGTGTGATCGAATGACTAACAAAAACCAACAAAGGTTTTTGAAGGTAACATGTACTCTTAACTACTTCGTATCAC
AGGGTGTTTCACTGTACTTGTACTTCTAGCCTGTTAATTAGTAACTACATAGGCCACATTAACTTAGGTAACTTAACAGA
GCTCAGTTGTTATTTTCTACGTTACATAATTGAAAACATTAGGAATCATCTAATCACTGATCTTCATTCATAATGAATTA
AATGAACCAGCTGATTGAGATAATTACTCAAGTGAACTGTCACGGCAGTGAGCTTTATCTTAAATGACTAAAACCAAACT
GAAATGAAAACAAACAGGAAGTCTGAACCGTAATAATTCACTTGTCAGTCATGCCCTAGTATCATTGAGGCTAATGCTGT
TTTGCAACTTTGTCTGCAATAGGGGTGTGAACGTTTTAACGTTAAAGAAATTGGGATCCCTAACCGAATCCCTAACCAAC
ATTTTTATTTGTATTTTTTTTAATGTAGATCTAGAAAGTAAACGGCGTGACGAACCCATGCATATGTGCAGATACTATGT

GATAGTGACGTGTTCCATCTCTCTCATCTCTACAGCCTAGCTTATTCATTGATGATAGGCCCTGCTTTACTGAAGTTGCG
AGATAGAAAAAGAACTTGCGAGAGAGCTTTTGATTGCCGATAGATAGCCTAGGCCTTGTGCACGGTTCTGACTGTTGTTC
GACCTGCCTATACTGTGCCCCTCCTCTCTCTCTCCATCCCTCGCTAGCTCGCTATGAAAGATGCAAGTGTTTGATTAG
TTGCCGTACTTCCAAAAACACAGTCTCCGCCGTTCTAAAAATACTAAATCTTAGGAGTCAATTCTTAGCGGAATCTAATC
TTGACACTCAGAAATGGGAGTCGAGGAATCAATTATTTTGAAAAATGGCAGAGAAAGGCAAGCGACAGCAGCGAAGTCCT
GTATGGCAATACTTTGAGAAGGAAATGCAAACATTGCAAGGTGGAATTGAGGTACATTAATGGCAGTACAGGTGCAATGC
TTAACCATCACAGGGACGCACCGTGAGACCCAAACCGTTGCTGGCAGCATCGCATCTGCATTGTGAATTCACATGGAATT
TGATGAATTTTTCTTATTGTTTTAACAAAATTGTTTAATTTGTCACATCCCTAGTCTGCAGTACAATATGCTGCATCTTG
TCCATTATAGAAACCAACTCAAGCTTTCATCTTCCACTTTGTCCAGTGTTCTTTGTCTTCAATACAACAACATGCTTAGT
CTTTAGTTCATCATCAGGACACCAACCCAAGCGTTCATGTTCCTTTGCAGAATGCCATAGTGCCCTTCAAGGAACTATGT
GGCCTCTCGGCAGTGGCCAACCTGAAGCAGTGCATCCTGGCCCTGTCCTCCCGGCTGACAGGGGCTGACAACAACCCCCT
CCTGGTGTTCAACCTCAAGGACCAGTACCCCACCATGGAGCCCCAGGGCCAGCTACCAGATGTCCTGAAGAAGGTGGTCA
CCACCTATGACATGGTGAGTACAGGAGGACTTTTGTTTGAACAGTATCATGTTTTTTTAAATGTATTTCCTTTGTTTTGT
AAGGTTAGTTGATCGAGGACATTCTTTTTTAGGACCAGGACTTGAAATCGGGGATCTTACTGCATATTTTAATCTTAATA
AAAGCCTTTCTCATTAAGACCACCACGAATGTGCTGATTGTAAAAACAACCTCAAAACACCTAGAACATTAGTTTGATTC
TAATCAAGTCAAGAGGGGGGTTTAGTGGGATTTGAATGGTAGCATGGGGTTAAGAGGGTTAGGTAATAGGGCAGGGGAGA
GTTGTTTTAATCAAGTAATTTAATCAATGTTGACAAAGGACTGAATTTATACCACACCCCCTCGGGCCTTAATTATATAT
GGCCAATATACCACGGCTAAGGGCTGTTTTTAAGCACGACACATCNNNNNNNNNNNNNNNNNNNNNNNNNNNNNNNNNNN
NNNNNNNNNNNNNNNNNNNNNNNNNNNNNNNNNNNNNNNNNNNNNNNNNNNNNNNNNNNNNNNNNATTACGGTGAGGTGT
AAGGCGTTATATGTCGGTATTGAAATAGCCTCAATGACGCATTATAAACTGGGTGTTCAGCCCTGAGTGCTGATAGACCC
ACAGCCGTGGTATACGAGACCATATACCACAAACCCCCGAGGTGCCTTATTGCTATTATAAACTGGTTACCAATGTAATT
AGAGCAGTAAAAATACATGTTTTGTCATACCCATGGTATATGGTCTCGTATACCACGGCTGTCAGTCAATCAGCACTCAG
GGCTGAACACCCAGTTTATAATGCGTCATTGAGGCTATTTCAATACAGACCTATTACGCCTTACACCTCACCGTAATCAA
TGTGCCTTAATAATACAAGAGTTTTATAGAATGCCCTTCGTCCCCCATTTTGAATATTTCATTACTACTGTATTTGCTAC
GATACAGAGCACAAGTTAACAACAACTTGAGAGCTCTACTATATTATCCAACGTGGCCATGGAAAAACACTTAAGGAACA
TCTGATATTTCATTCTCATCTGTAGCCTATTTGCTCTGAATATGTATTGAATACGCTTTAAAATATTCATTCTTTTGATA
TTTCTATTTGACACCAGCTGTTTTCGGTGTCAGACATGTTTGCCGTGTACAAAGCATAAATCCTGGAGAAGTATTAGTGA
AGCTTATTATCCACTGTAGAAACACAGGTCAAAGGCTACAGACTGCATTAGTACACCATTAGCATAGCATTGCATGTCAT
TTACCTAATGTCTTTCTCTTGACTTTAGAACCCCCACACAGAGCATGAAGGTAATGTGCTAACTAACCTGACAACAAATG
AGTCATGCGTAATTTTACTCAAAAGTGTCTCATTCATTGTGAGTTATGGACAGGCGTCAGTGAAGTTTGGATCATGAGTT
AAGCGTTAGACTCGTGTGTCCTCAGCAAATTTGATGAAAATGTGAAGTGAACGAAGATCCAGTCGTGTCGTCGTCCCCTG
GATACATTTTATGTAGAAGGACTGAGAAGCTCAGTTTGGGTGACATTTTAAAAGGACATTCTGCTCCAAAAGGAATGAAT
ACAATTATGTTGACACGCCCCTTGCCTGTACTTCTCACAGAAACCACTTCATGTACCCCCCCCCCCACCCCCCCCAACACT
TTCCCTGGTTGCCACTACTCTTGCTCAACTAAATATGTAATCGGGCTCATCACTCCCAAAAATGATGAGGTAGGGAAGTG
TTTTACCCCAAGTTACCCTACAATTGACCCGTGTTAAGTTTAGCTCCACTTTAGGTGAATTGCGGAGCGCTAACGTGCTC
AACCATGCCGCTGCTAAAAACTCTGGGTTTAAAATAGTGCAGTTCAGTGTATTTAGGAGTTGAGAAATAAGTATGAATGG
AAAGCCAGGATCCACCTCTTTATTTTTTTTTAACAAATGCTATTTAAAACTGTTATTTTCCCCCACGATTGAATTAAGAA
TTGTTGTGCATTGACTGCTTGTCAGAATGCAGGTTTCCCTCCCTATGGCACTCATAACTTGAATGCGCCTTGTGAGGAAT
ATTTATCTTGCATAGAATACACAACAACAAGCCGACTAGGTAAATACCGTTGAAGTCGGAGGTTTACATACACTTAGGTT
GGAGTCATTAAAACTTGTTTTTCAACCACTCCACAAATTTCTTGTTAACAAACTATAGTTTTGGCAAGTCGGTTAGGACA
TCTACTTTGTGCATGACACAAGTCAATTTTCCAACAATTGTTTACAGACAGATTATTTCACTTATAATTCAGTGTATCCC
AATTAGAGGTCGACCGATTATGATTTTTCAACGCCGCTACCGATTATTGGAGGACCAAAAAAATCTGATACCGATTAATC
ACCCAATTTTATTATTTAAACAATAATCGGGTGATTAATCGGTATCAGATTTTTTTATTTTTATTATTATTCTTTTTTTG
GTTGTTGTAATAATGACAATTACAACAATACTGAATGAACACTTATTTTCACTTAATATAATACATAAATAAAATCAATT
TAGCCTCAAATAAATAATGGAACATGTTCAATTTGGTTTAAATAATGCAAAAACAAAGTGTTGGAGAAGAAAGTAAAAGT
GCAATATGTGCCATGTAAAAAAGCTAACGTTTAAGTTCCTTGCTCAGAACATGAGAACATATGAAAGCTGGTGGTTCCTT
TTAACATGAGTCTTCAATATTCCCAGGTAAGAAGTTTTAGGTTGTAGTTATTATAGGAATTATAGGACTATTTCTCTCTA
TACGATTTGTATTCCATATACCTTTGACTATTGGATGTTCTTATAGGCACTTTAGTATTGCCAGTGTAACAGTATAGCTT
CCGTCCCTCTCCTCGCTCCTACCTGGGCTCGAACCAGGAGCACATCGACAACAACCACCCTCGTTACCCATGCAGAGCAA
GAGAAACAACTACTCCAAGTCTCAGAGCGAGTGACGTTTGAAACAATATTAGCGCTCACCCCCGCTAACTAGCTAGCTATT
TCACATCGGTTACACCAGCCTAATCTTGGGAGTTGATAGGCTTGAAGTCATAAACAGCGCAATGCTTGAAGCACAGCGAA
GAGCTGCTGGCAAAACGCACAAAAGTGCTGTTTGAATGAATGCTTACGAGCCTGCTGCTGCCTACCACCGCTCAGTCAGA
CTGCTCTATCAAATCATATACTTAATTATAATATAATAAACACACAGAAATACGAGCCTTTGGTCATTAATATGGTCGAA
TCCGGAAACTATCATCTCGAAAACAAAACGTTTTTCCTTTCAGTGAAATACGGACGCAAGAGAAGTGACACAATTTCACC
TGGTTAATATTGCTTGCTAACCTGGATTTCTTTTAGCTAAATATGCAGATTTTACAAATATATACTTCTGTGTATTGATT
TTAATAAAGGCATTGATGTTTATGGTTAGGTACAGTCGTGCAATGATTGTGCTTTTTTCGCAAATGCACTTTTGTTAAAT
CATCCCCCGTTTGGCAAAGTCGGCTGTCTTTGTTAGGAAGAAATAGTCATCACAGTTCGCAACGAGCCAGGCGGCCCAAA
CTGCTGCATATACCCTGACTCTGTTGCAAGAGAAGTGACACATTTTCTCTAGTTAAAAGAAATTCATGTTAGCAGGCAAT
ATTAACTAAATATGCAGGTTTAAAAATATATACTTCTGTATTGATTTTAAGAAAGGCGTTGATGTTTATGGTTAGGTACA
CGTTGGAGCAACGTGTACCTAAGCGATTATATGCAACGCAGGACAGGCTAGATAAAGTAGTAATATCATCAACCATGTGT
AGTTAACTAGTGATTATGATTGATTGATTGATTGTTTTTTACAAGATAAGTTTAATGCTAGCTAGCAACTTACCTTGGCT
TCTTACTGCATTCGCGTAACAGGTAGGCTCCTCGTGGAGTGCAATGTAAAGCAGGTGGTTAGAGCGTTGGACTAGTTAAC
CGTAAGGTTGCAAGATTGAATCCCCGAGCTGACAAGGTAAAAATCTGTCGTTCTGCCCCTGAACAAGGCAGTTAACCCAC
CATTCCTAGGCCGTCATTGAAAATAAGAATGTGTTCTTAACTGACTTGCCTAGTTAAATAAAGGTGTAAAAAAAAAAAAA
TCTGCGTCCAAAAATACCGATTTCCGATTGTTATAAAAACTTGAAATCAGCCCTAATTAGTCGGCCATTCCGATTAATCG
GTCTACCTGTAATCCCAATTCCAGTGGGTCAGAAGTTTACATACACTAAGTTGACTGTGCCTTTAAACAGCTTGGAAAAT
TCCAGAAAATGATGCCCCGGCTTTAGAAGCTTCTGATAGGCTAATTGACATCATTTGAGCCGTACCTGTGGATGCATTTC
AAAGCCTACCTTGAAACTCAGTGCCTCTTTGCTTGACATCATGGAAAATCTAAATAAATCAGCCAAGACCTCAGAAAAGA
AATTGTAGACCTCCACAAGTCTGGTTCATCCTTGGGAGCAATTTTCAAACACCTGAAGGTACCACGTTCATCTGTACAAA

CAGTAGTACGCAAGTATACACACCATGGGACCACGCAGCCATCATACCACTCAGGAAGGAGACACATTCTGTCTCCTAGA
GATGAACGTACTTTGGTAAGGAAAACTGCAAATCACACCTACGAACAACAGCAAAGGACTTTGTGAATATGCTGTATGAA
ACAGTTCAAACGTATCAATATCCACAGTAAAACCAGTCCTGTATCGACATAACCTGAAAGTCCGCTCAGCAAGGAAGAAG
CCACTGCTCCAAAACCGCCATAAAAAAGCCAGACTACGGTTTGCAACTGCACATGGGGACAAAGATAGTACTCTTTGGAG
AAATGTCCTCTGGTCTGATAAAATATAACTGTTTGGCCATAATGACCATCGTTAGGGGGAGCCTTGCAAGCCGAAGAACA
CCATCCCAACTGTGAAGCACAGGGGTGGCAGCATCATGGTGTGGGGGTGCTTTTCTGCAGGAGGGACTGGTGCACTTCAC
AAAATAGATGGTATCATGAGTGAAATGATGTGTATATATTGAAGCAACATCTCAAGACTTCAGTCAGGAAGTTAAATCTT
GGTCGCAAATGGGTCTTCCTAATGGACAAGGACACCAAGCATACTTGCAAGCAAAGTTGTGGCAAAATGGCTCAAGGACA
ACAAAGTCAACATATTGGAGTGGCCATCACAAAGCCCTGACCTGAATCCTATAGAAAAATTATGGGCAGAACTGAAAAAG
CATGTGCGAGACAGGAGGCCTACAAACCTGACTCAGTTACACCAGCTCTGTTAGGAGGAATGGGCCAAAATTCACCCAAC
TTATTGTGGGAAGCTTGTGGAAGGCTACCCGAAACGTTTGACCCAAGTTACACAATTTAAAGGCAACGCTACCAAATACT
AATTGAGTGTAAACTTCTGACCCACTGGGAATGTGATGAAAGAAATAAAAGCTGAAATAAATCATTCTCTCTCCTATTAT
TCTGACATTTCACATTCTTAAAATAAGGTGGTGATCCTAACTGACCTAAGACAGGGAATTTTTACTAGGATTAAATGTCA
GGAATTGTGAAAACTGAGTTTAAGTGTATTTGGCTAAGGTGTATGTAAACTTCGGACTTCAACTGTAGAGATAAACTACT
CTATTATGGGTTTGTCTGGTTTTGTTTGCAGATGAAAAGTATAAGTGGATTCTTCAAGTATCTTCAAAGTGCTCCTCTGC
AGAAATTGTCTTAATTGAATGTGCTGTTCTTCGTGTTGTAAATGTATTGTGTAGATTTTTCTAGAATAAAAGGAAGGGAA
GTACCAATGTGTTACAAAGTAGTAGATTGTTTATGATAACCATTACAATTTTGTAGCCTTGTAAAACACTCCCAGTAGCA
CCACAAAATACACAAATCAACATCGNNNNNNNNNNNNNNNNNNNNNNNNNNNNNNNNNNNNNNNNNNNNNNNNNNNNNN
NNNNNNNNNNNNNNNNNNNNNNNNNNNNNNNNNNNNNNNNNNNNNNATGTTCATTCACGATCGACATAAAATGGCAGCTAC
TCTCAGTACGAAGCTAGCTTTAGCTGCAACCGAGCAGGGCTCATATTACTCTGCAAACTTAACTCTAACTTCCTCAAGAT
GTTACTAAGACACACCTTAAACACTCTTGACATGTTAGCGAATTATTACATTTAAATGACTCTGTTTTCATCAATAACGT
ACCTGAAAAAGGGGAGAAATAATCACGAGAGTGATACGTATTATTTCCTCAGTGAGAACTTTTACTGTAATGAGGAAAAG
ACCGCGATTTCCCCGCGAACTTGGGTGGACACCAGAGCAATCGATCTCAGGCTCATAGATTAAAGAGCATTTAGTAGATC
ACAGATTAACACTTTTCCCCTTCAGTTAGGCACCACAAAATAAAGTAATCAATATAACGTTTACACATTCCTTTTACAAT
TCTTACCTTTTGTACGCTTGATGGATTTGAACTTTAACACAATTATGAATGTTATCAATCTCTATCAACTAATACAGAAT
GCATGATCTTTTTAACCTTAGATGTTTTAAGATCCTCACATACTATGAACTTACTAATACTTTTTAATGTATAACAGGCT
GAGTATTTCCTTTAACCTGTCACATTACACTCATCAGACTGTAGAAATGCTCTGGGTTGCCCTCGTAAAACTAAGTTTGA
TGCCCACCTCAATAAATATATAGGCCATATTATTAATTGTCAGTGCTTTCAAATAATTTTGTAAGTCAGTCCTAATTTTC
AAATGATGTATCACCAAAGAACATTTTGTAATTGCTTTATTACCTAATATCCCCACTTGGGTGTAGAGGGCTGGAGTCTC
TGTTTGAAACCTTCATCTCCATTTTCACTGCCTGAAGTGGAACGCCCAGCCCAGTAGTCCACCATGGACAGGCTGTCACA
GACACACAGTCACAGTGCTGCTGATCCCCTTAGCTCCTGTCACATCGCTCCTAAGTAGGTACTCTCCTCTTTCTCACTGG
CTGGAAAACTGGCACAGCAAAAGGATCGATCTCAGGGGCGGAGGGGGGCATGTTGGGTCACTGGCCGACCGTCAGAGATT
GACTTGGCAACTGGAGCTCTGCTGAAACAGCTGCCCACCCCAAGCCCCCCGCCCGGTCTGCCTTGGCCATGGAACAAGAC
TGCCTGCTTTTATTATGTGACCTTGCAGTGCTCTTAAGTCACTGAGGGAATGTACAAGTCAATTACGCCAGAGCAGCGAA
CATAAAGCCCAGCACCCGTGGCACTAATTGAAACGGAAGGCTGCCGTAACTGTGTCATCTCGCCCCAGAATCACATTAAG
AAGCCTATTCTAATTTTAGATGATACATCATTTCCTTTAGATGATCGTATTAATTGCACTCCGAGAGACAGTAAGAAGGG
AGAAAGATCCAGATGGTCGTACCTGTGTGTACGTCTCCTGGGTCGTGCAGTGGGTATTTACCAGGATCCATGACAGTAAA
CACATGCATCATTAACCTCCCGAAATACCCCCGATTTACCAGTTAGGTATTTTGGGGTGTTTATTTTTTGGTCAGGCATT
AGAAGTGCAAATGGGCCTCTCTGTGATCAGAGCCACAATGACAATTTCAACACAGTCATGGAGGTCAGAAGTGTGTGCAC
TCAATTAAGGCAGGGAGCTAAATGTAGCAGTCCGCTGCCCAGCTGTAATGCCACAGATAAGGCTGTGTTACGCCTCCGAA
CAAGGCCTCTCCTATTTACAACAGATTGCTGTAATAAACAATAATAACCTACTCTTGAAATCACATGTTTTCAGACCAAC
TCTGAAACAGGACTTTTATAACGGTAACACGCAATTACTGTAATACCCTAGTTGTCTTGCAACTTTCAATAACATGCTCT
GATACACTCCCCTCCCTCCGTTTTTACCGTGCCTCAGCACAACTCCCGCTGCTTGGCCCTCTATCTCTGTAATAAGGGCT
TAGGCTTTTTTCTCCCCCGTACTGGATACATACTCCACAAACAAAAAAATTAAGATTGCTCACCACCGCAGGATGCCAGG
CTGCTGTAAAGGGAATAAAAAGGTTCAAATGGACTGTGTTTTCTCCTCTCCTGACAGATGATCCAGACCAGCAAGACTCT
GCTGGAGAACTCTGAGGGAGTCTACGACCGAATACTGCTGACCCAGAAAGCAGGTAAGAAAGGGGATACCCAGTCAGTTG
TCCAAATTAATGTATTCAACTGAAATGTGTCTTCCGCATTTAACTGAATCAGAGAGGTGCAGGGGGGCTGTCTTAATCGA
CATCCACGTCTTCGGCACCCTGGGAACAGTGGGTTAACTGCCTTGCTCAGGGGCAGAACGACACATTTTTTCCTTGTCAG
CTCGGGGGATTCGATCCAGCAACCTTCCGGTTACTGGTCCAACACTCGCGAGGGATATAGCTGAATAAAGAGCAACGTTT
AGCATGAAAAGCCCATTTCTCTGCAAAGATCCACTGTCCCTCAGATCTTCCCTTCTTCCTTTCATATACAATCATCCCCC
CTCTCTGAAGTATGACATGGAGAGCGGCAGCTAACAAAAAAGATGGACTGATTATGCCGGCCCTGTTACATAAGAGTTAT
TAACATTTGATTCATGCTCATTTCTGTGACCCGGATGCTTTGAAACGCTGCTTTGGATGTGCCTGGTTTTTGATGATTCT
CACGGCGTCGGCCTCGCTGCTCCTCTAATTAGGAGGGTTGCAGCTGAGGTGGTAAACACCCAGGTTCTGAACAGAACAAC
ATCAAAGACTGGCAGGCGGCTGCGGTCTCTTTATGCCTGGTTTCCACCAGGAACCGCACCACTACAAGACGTGCAGAATG
AGATCAATCTCATTTATAACAAATGAACGGGAATCATTTACCAGAATGGATGAAAATATTTTATAAAAAAAAAAAATGCA
TTTTGGCAATTGATTAGAAAATAATTGATCTCATTGTTGGGAGAATTCTGTGTTTGCACTTTGATATGAATATTGCAGCT
TGTGTGCTCTTGCAATTGTATTATATCTGTGCTCTTAGTACTGTTTATCCCGCTCCTCTGGTGAGTCTCTGTGAGAATAG
CCTTCTGGCCCTGCATAATGTGTTGACTTGACTTGGGCAGGGGCAGTGCTAATCCAGTCTGCTCTGGGGCGTGGGGGATC
GGGAGGATCTTGGAGCCTTGATAGTGTGATTGGCAGCTCATCAATGGAAGACGCTGGCAGAGATCCCACCCCTCGCTCCA
AGGTTGGGAGAGTCTTGGCTTGAGAAGCTGTCAGAATGTTCACTCTCTCTTCCTTCCCCCATTATGAGGGGAACACAACA
AGGTGGATTTTTCCGGAAAGAGCTCTAGTGGTGCAAACTCTGCAAAACCAAATTGTGTGCTTTAAAAGCAGCATAGAATT
GACCTGTAGATTTACTACAGTACAAATAAAATCATATTCAATGAGAGGATAATTTAGACGTCTGTGTCTTTTAATCTAAT
GCAGCGTATTTCAGCAACGTTTCCAATCTAAGCATTATAGATGTTTAATTTATCATGGAGTAACTTGCTTGGAGCGCCGA
GTGAGGAAAAACAGGCCATCGTTCCGGGGGCTGGGATTTATTGGTTTCCAGATGGATGACATATAAAATATTCATAGTCT
ATTGTCTTGGCTCATCAAGGGGGCGGAGGAGTGTCTTTTTAACTGAGGTGTGCGTCGCAAATCAATGGCTTCTGGCCATC
ATCTCCTCTCTTAGTATTCATCACAACACTTACAGGACCTGCCTTCAAGCTGCTGCCCGAGACATTAGCCATTGGTAGTC
TGAAAGTTAAGATGTGAACTGCCTGAACGAATGGTGCTGAAAGAGACAACAAAAACAACTCCTACTCTCGGCAGCAGGGT
GATACGGAATTGTGCTCAGATGGTTCACTTTTTGGTGTTAGCTAGATCTATAGTATTACAGTATATTGTGTCCATAGTCT
GAAAGCATTCCTCTTCAAAGTTGCTATGAAACCATCACATTTTTTCAATGTATGGATCTTTGAAATGTCTATAAAAAATG

AACCTAAAAGTAACTAGGGATGGGCACACATACACAGTGCTCGATTTAAAGTTTGCATTTGTTTACTAATTACTTGCATA
CAAGTACTCGAAATTCACAAATGCAACTATTTAGTAGGTTACATTTATAAGCACTGACAATTTCAAACTCAGGTAAGGTG
TGTGTGTGTACTCCACAAAGAATGTCAAGTGCAGTGAAAAATGTGTTAAGTTTGGTTTTCAGTTGCGTATCAGAACAACC
AGCATAGCGCAAACTTTTTTGCCTTCAGCCCTGTTTCACACTAACATGTGAGTACTTAATTATTTCCATGAGTACTCAAA
CACAGAAATTCTTTCAAATTCCCATCCCTAAGATGTACCTAACTAGGCCACTCAGTTGAGCCAAAACGATAGGCTACTGA
AGCTAGGAAATGCTTCATGTATTTGTGTTATTTCATTAGTTAATATGGGCACACAAGACAACTAAACAAGTTAAACATGG
GCTTCCACATTAGCCGCCCAAGGCTTCTTTCTGGTCACTACCAGGCACCATATCAAATCTACTGGGCTGGATGTTGTAAT
TTCATCGATACGTATTGTCAGCTGTGAGTGGAAAATGGGTCAATACAAACTGTATTCCCTCCAGACAGTGAGCAGAACAT
TCCAGCAAAAAGCTACTGGAGTTCGGAGTAATCCATCATAACCGCTCAGAGAGAGCAAACCGCCTCAAAAGACCCTCTGG
GTAACAATAAACAAAGATCAGTACTTATTTTTTGTATTTAACTAGGCAAGTCAGTTAAGAACAAAAGGACGGCCTAGGAA
CAGTGGGTTAACTGCCTTGTTCAGAGGCAGAACGACAGGTTTGTACCTTGTCAGCTCGGGGATTCGAACTTGCAACCTTT
CGGTTACTAGCCCAACGCTCTAACCACTAGGCTACCCTGCCGCCCCTTTAGACTTACTGTCTAAAGCGCACAGATCAAAT
GCAATACTTGACACTCACACAGGTACCAAATAACATCTGGGACAATTCAATAGCTGAATCCCAAAGTACACCTAGTCTGG
ACAGAGTAACGCTAAGGTTAACTTTTACAAACTGCCTAAAAAAAAACAAGCTAAAGAGAAAATGTGGAAGATAAGATGAA
TGGAAAAACCCCATTACAGTAATGGTGATTATACTACAGGGTCAAATGCATAATCTATTCAGGAGGATTGGGGACACTCA
ATCAATGTGTTGCTTTTGCGATTCAATCCATTATTAATAAAGCTAGTAAACATTTTAGCCTAGGCTACAATACAGGTTGG
GGAAACGGTTTCATTAAATATTTCCTATGACTGGGCACTCTGGTCCAAGTTCATTATGCTGAGTTATTGAGCTCCAGGCA
GACGAGGGAGAAGATTACACCATATAAAGAAGCTAATCCCTGTTGTATTGTCATTGGCCACGGCAAGCCCCTTTCCTACC
TGCCGCTCGGTTATGGACTGTCAAAGGTGACTGAGAACCTTTTGGTGGCTCTATACAGTCTCTGTCAGCGAGCATAAGCT
TGCAGAGAATTGAGTTTGAAAGGGTTTGCTCAGAATGACAGAATTAGTCCCCCCCAACTCTACACTGCGTTGCTAAGCAA
CCCCTGCTGAGAGTTGGTGTGTGTGTGTGTCTTTCCAGCACAGTCATGCTTCATGAGTACAACAGTAGTGACTGTAGTTACA
GCAGCACAGCACCTGAGGGTGGGGGATAATCATTCCGGAATCATTCAGATGTTTCACAGTGCACAACATAAAAAAGGCTG
GGAGGAACAGATTATTAGCACTATGTATGGTAAAAAAAAAAAAAAAAAAGTATATGCCCTACACATTCTCTGTAAGAGAA
TACAACACTAGCCTCAACCAGGGCCTAATTTGTGAACATTTCTTTGGCCATTTTCTGTCTCAACTCTTCATCTAGGTCAG
GTATGTGCTAACTGCTGGAGTGCCAATATCACAGACCTCCTCAGAATCTCCTCTCCTCAACATCCATCCATCCAACCCAC
ACCCACCCCACTGTATATCTCAAATGTACGGTATTTGGTGTTGCCATGGAAACAACACCAACAGGGCTGGGAACCGACCC
AGAGCTGATAGAGCACCGCGATAGATCTGAGCATGCTCAGTCCGACCGTTTCAGCTTGTGTTTGTGTGTGTGTGTGTGTG
TGTGTGTGTGTGTGTGTGTGTGTGTGTGTGTGTGTGTGTGTGTGTGTGTGTGTCTGGTGAAGCTATTAGTAGTATACCAAGC
TCCCGTTACAGAGCACCTTAACGGCCAGACCCTCAGCTGTTGCTCTGATGTTGTGGACTCCACTGCACCGTGACTCAAAT
CAAACACACGGCAATGTTCGTCTGTGTATCCTAACGGAGAGCAGCACGCAGTCCGAGAGAGAGGAGGAAAGAGGGGAAGG
AGAGGAGGAAGGGAGCTCCTTGGGAGACAAGTCCCTCCTCTGCTCAGCGCCTTGATGCTAATCCACAGTGAGGCGATGTC
GTGTCTGTCTGCCCTGCCCATGCTAGTCTTGCTCTCTCTTTCTCTCTCTCACGCTCTCAAGAGAAGATGTATCGCACTCC
ATCTTGACCTCACCAGCTTTGCTTCCTTCCTCATGTTGTCTGCTGGGCAGCTGTTTTCAGGCTGGTGTAGTTTCTATAGA
CAATGAAAACAAAAACTGAACCAGGTAGTTCTAGTACTGTATGTACACTGAGAGATGGTTGAATCCGAAATGAATACTTA
ACGTATGATCATCTAAAGTGTGTTGGTGTTTTATCATTGTCTCACACGTCAAGAATGAAACCAAATGTGTGAAGGCCTTA
ACTTAAGGCTTTTCCCATAGTGCTGACACTTGCCTAACCCCAATTTGGCATTTCATGAAAACCTAGCATATAAATGCATT
GAGCATTGTACGTCAATGCTAGCCTCTGTGACAAATGTGTGTGAATACATAGCGTAAAACTCATGTGTCCCCTGACCCCT
CTCTCCCCAGCGATGGAGTTCCATGAGAACCTCCATGACATGGCGGTAAAGGAGGGCCTGAAGGGCAGGAAGCTGGCCAA
GGCTGTGGAGAGCTTCACATGGAACATAACTATCCTCAAGGTAAGGATAATGACCACCTAGTTTGAGTAATCTTTTTTTT
TTGACAGTTTTTAGCTTTTGTTTAATCTATTTTAGCTACCAATAAACTGTTTTAAATCGATCTGTGGACACCATAGATCA
AAATGGCTTGCATTGAGTGGTAGCCCTGATTCTCAGACACAGGAGTATGTCTCTTCTGCCTGTATGAAGCAGGATGTGAA
ATCTGACACCACTTGCAGCAGGGATGTCCCTCCTACCTTTAATTCGCTCTTCCGACTGTCTATCAACTCCTGTCTGAAGC
CTAGAGTGCCCTCGGAAAGTATTCAGACCCCTTGACTTTTTTCACATTCAATCTACACACAATACCCCAATAATGACAAA
GCGAAAACAGGTTTTTAGAAATGTTTTGAAAATGTGTTAATAACAAAAAACAGATACCTTATTTTTGTAAGTATTCAGAC
TCTTTGCTATGAGACTCAAAATTGAGCTCAGGTGCATCCTGTTTCCATTCATCATCCTTGAGATGTTTCTACACCTTGAT
TGGAGTCCACTTGTGGTAAATTCAATTGATTGGACATGATTTGGAAAGACACACCTGTCTATATAAGGTTCCACAGTTGA
CAGTGCATGTCAGAGCAAAAACCAAGCCATGAGGTCGAAGGAATTGTCCATGGAGCTCTGAGACAGGATTGTGTCGAGGC
ACAGATCTGGGGAAGGGTATTGAACGATTTCTGCAGCATTGAAGGTCCCCATGAACACAGTGGCCTCTATCATTCTTAAA
TGGAAGAAGTTTGGAACCACCAACCACCCGGCCAAACTGAGCAGGAACCGCCCGGCTAAACTGAGCAATCAGGGAAGGGC
CTTGGTCAGGGAGATGACCAAGAACCTGATGGTCACTCTGACAGAGCTCTAGAGTTCGTCTGTGGAGACGGGAGAACCTT
CCAGAAGGACAACCATCTCTGCAGCACTCCACCAATCAGGCCTTTATGGTAGAGTGGCCAGACGGAAGACACTCCTCAAT
AAAAGGCACATGACAGCCCGTTTGCCAAAAGGCCCTTTGAAGACTCTCAGACCATGAGAAACAAGATTCAATGGTCTGAT
GAAACCAAGATTGAACTCTTTGGCTTGAATGCCAAGGGTCACGTCCGAAGGAAACCTGGCACCATCCCTGCGGTGAAGTG
TGGTGGCAGCATAATGCTGTGGGGATGTTTTTCAGCAACAAGGACTGGGAGACTAGTCAGGATCGAGGCAAAGATGAACG
GAGCAAAGTACAGAGAGATCCTTAACGAAAACCTGCTCCAGAGCTCTGACTGGGGCGAAGGTTCACCTTCTAATAGGACA
ACAACCCTAAGCACACAGCCAAGACAACGCAGGAGTGGCTTCGGGACAGGTCTCTGAATGTCCTTGAGTGGCCCAGCCAG
AGCCCTGACTTGAAGCCAATCTAACATCTCTAGAGAAACCTGAAAATTGCTGTGCAGCAACGCTGCCCATCCAACCTGAC
AGAGCTTGAGAGGATCTGCGGAGAAGAATGGGAGAAACTCCCAAATACAGGTGTGCCAAGCTTGTAGCGTCATACCCAAG
AAGACGCCAAAGGTGCTTCAAGAAAGTACTGAGTAAAGGGTCTGAATACTTATGTAATATTTCTAAAATAATTAATAGAT
TAACAAAAAACACTTTGTTTGTCGTAGATGGAGAATATTAAAATGAGATGAAAGGCGAGTTCCTAACGAGTTCAGGAAGC
CAAGCTAGCGCTCTGAGAGGCGTTCAAAGCGATGGGGCCGATGTTTTGATCGAGAGACCTTTAGAAACTTTGCACATTTT
CTCTAACACTAAATATACAAATATGTTTTTTACAGTTATAAGGAAGGTGAAATCTTAATGGATTAGAAAAAAATGAAATT
TCAAGCCTTATACATAGTTTTGTTGAATAGGAAATAATCAAAATATGATCTGTGTAATTAAGCTTGTGTCCTCAATAGTG
ACTACACCTCTGCAATTTATAACTATTTTTACTAGAAAAGTGAGAGTTTAACCTTGGAGTATGCCGCATTACTAGTTATT
GTTTATCTCATGATAAATAATTACTTTTGGGGATTACGTAGATTACATTTTCGATTACATTTAGTTACATGTAAGAGGTT
TAACTTAATGTTTGTTTTGAGGACAGACATAAGCAGTGAATTTATATTCAAGAAACAACTTCAGAGCGAACACTTTAGAG
ATTATGATTCCACAGTGCAGTTGAGCTTCACTAAATAAGGAAAAAACAATGTTTTTTGAGAGTTTGTTTACAGAAGGGAC
ATAAGGTAGCTAGGTTTAAATTGACATTTGTTTTTCAGCTGCAGCGCGGTGGAATAAGAGCCTATGTCTGAGCTAGGTAG
ATTGCACACACTGTCTGATGTATTTGTCTTTATGCCGTTCATTTAATCCCTGTGTCAATGCAAGGGGTAATTATCGAGGA

ATGACCTTCCGCATATTTATTTGTGATATTTGCATGCGTTAATTGCATGCTCACCTCATGAGCGTTTGGGCTCATCATTT
ATACATGGTCCCCGGGAAATTGAGTTGGTGCCTGGTGAGTGTTTGTCTACGCTGCATAATTCATTATACACAGAAGCAGC
GGTTACGCTCCGTGGGGATTGGGGAGATGTAGGACTCCAATGTCTGACCGCATACAACTAAGATTAAACAGTTAGTACCA
CACTACTCTCTCGCTTCGTGGCCTTTTTGAAGGTACAATTGGTTTCACCCTTTGTCTTTCTGTTTAAACTGGAGATTCAA
GTTGTTGATACATGCTCCATTGTCTCTATATTGCCATTCCTGTCATGTCGCTGCCTCCCCTCCAGGGCCAGGCGGATCTG
CTGAAGCACGCAAAGAGTGAGGTCCAGGAGAACCTGAAGCAGATCCACTATGCCGCCCTCACCTGTAACCTCAGTAAGGA
CGGGCCATCAGGGAGCACGGCAGGCTCCGAGTCCAGGACCCGACCACGCAGCCTGGACGCCATCCCCGAGAAAGCCACGG
GGGAGGACGAGCTCTCTGAGGAGGACAACTGAAGGCTTACGCCCTAAACCCTTCATACCTCCAAGCCCCTTGCCCTACCC
TAGTGGCACTCAACCCCCCCCCCCCAAGCCCCTTGCCCTACCCTAGTGGCATACATAAACCTCCTTACCGCAGTCCCACA
CAAACCTAACTACAGTATAACTGTTCTAGTGCCGTTGCCACACAACTTTTCTTATTCCAGTGCCGTATACAACCATTCCA
TTCCCACCCCAATCTCACTCAAACCTCCCTTTGCTTAACCAGGTACCACACAAAAAGCCCTTAACCCCTCCTGCCCACCT
CAACGTCCAACAGCAAGATGTTGCGGGTCAACTAGGAGGGTCAAGTGAACTTGCAGCCACGGGACAGTCTTAAAGCAGTG
ACCTACTGTGGAGACAGTGCACTTATTTACAGGCAGACCCCAGGGTCTGACCCTAGGAGAGCTGCTCTAAACGGTCCAAG
GATTTCTTAAGCACAATTTATGGGTTTCTTACCAACCAGAAATTAGAGCTGTGAATGTAATAGGAAACAGGAACATTGGA
GAGAAGGAAAGAAGAAAAGGGAGGCGAGAGCTGTTTACAATAATGAACTTGTTCTTGTCTGCCAAAATAGTATGTGTATA
TATGGAGTTCTGTTACTTTTTTTGCAACGTGCTTCATTTTATTATTGTATTCTTGAGAAATAAGATNNNNNNNNNNNNNN
NNNNNNNNNNNNNNNNNNNNNNNNNNNNNNNNNNNNNNNNNNNNNNNNNNNNNNNNNNNNNNNNNNNNNNNNNNNNNNNN
NNNNNNNNNNNNNNNNNNNNNNNNNNNNNNNNNNNNNNNNNNNNNNNNNNNNNNNNNNNNNNNNNNNNNNNNNNNNNNNN
