# Supplementary material for: Comprehensive analysis of MHC class II genes in teleost fish genomes reveals dispensability of the peptide-loading DM system in a large part of vertebrates
Source: BMC Evol Biol. 2013 Nov 26;13:260. doi: 10.1186/1471-2148-13-260 (PMC4219347; doi:10.1186/1471-2148-13-260)
Supplement: Additional file 9: Text S3 — Discussion of potential polymorphism of MHC class II genes in selected teleosts and comparison with previous studies. [file 1471-2148-13-260-S9.pdf]

Supplementary Text 3 (Text S3): Discussion of potential polymorphism of MHC class II genes in selected teleosts and comparison with previous studies

In the below paragraphs the MHC class II sequence findings in the present study are discussed in relation to possible polymorphism and compared with previous reports on the investigated teleost species. For figures and gene nomenclature we refer to the main text (e.g. Fig. 2) and to other supplementary files. For literature references see at the end of this file.

Atlantic salmon (*Salmo salar*)

Arguments for true, classical-type polymorphism of the highly variable DA group *DAA* and *DAB* sequences in salmonid fishes (http://www.ebi.ac.uk/ipd/mhc/fish/index.html) are (1) that only one sequence each could be amplified per haploid genome, (2) which were found to segregate in single *DAA*+*DAB* haplotype fashion upon pedigree analysis, and (3) which encode polymorphic putative peptide binding residues shown to be under balancing selection [e.g. Shum et al. 2001; Stet et al. 2002]. Tight genomic linkage between salmon *DAA* and *DAB* loci was already known, but the present study provides the first evidence that they are neighboring genes (Additional file 2, Fig. S2, and Additional file 8, Text S2).

For the DB group we previously reported that salmon *DBA*, *DBB*, *DCA* and *DDA* loci are non-polymorphic and thus can be classified as nonclassical [Harstad et al. 2008]. In the present study it was found that also the DB group locus *DCB* is non-polymorphic (Additional file 8, Text S2).

The DE group *DEA* and *DEB* loci also are non-polymorphic (Additional file 8, Text S2).

Zebrafish (*Danio rerio*)

Although zebrafish was initially thought to have four classical DA group class II *B* loci, designated *DAB1* to *-4* [Ono et al. 1992], they were later concluded to be alleles of a single *DAB* locus, corresponding to *D8.37B3* in main text Fig. 2 [Sültmann et al. 1994; Bingulac-Popovic et al. 1997; Graser et al., 1998; Kuroda et al. 2002]. Zebrafish DA group class II *A* polymorphism has not been resolved at the locus level yet. Sültmann and coworkers [Sultmann et al. 1993] reported variable II *A* sequences, but these sequences, two examples of which are shown as "zebrafish (4)" and "zebrafish (5)" in main text Fig. 4, Additional file 3, Fig. S3, and Additional file 10, Text S4, can not easily be assigned to loci discernable in the Ensembl database. A reported "*DAA*" gene on the other side of *SLC7A4* than *DAB* (*D8.37B3*) [Kuroda et al. 2002] is not present in the Ensembl database (Additional file 2, Fig. S2), suggesting haplotype variation ("haplotype variation" in this article is used to distinguish from "allelic variation" and refers to differences in gene copy number or order of genes between individuals of the same species). In addition to DA group *DAA* and *DAB*, previous reports also described the zebrafish DA group loci *DDB* (on Chr.8), *DEA* (Chr.8), *DEB* (Chr.8) and *DFB* (Chr.4), as well as DB group loci *DBB* (Chr.18), *DCA* (Chr.8), *DCB* (Chr.8); hitherto for none of these non-*DAA*/*DAB* loci transcription had been reported in article form, some of these loci are obvious pseudogenes, and at least several display

haplotype variation [Sültmann et al. 1994; Bingulac-Popovic et al. 1997; Graser et al. 1998; Sültmann et al. 2000; Kuroda et al. 2002]. Main text Fig. 4, Additional file 3, Fig. S3, and Additional file 10, Text S4, show that some of these previously reported sequences are (nearly) identical with the Ensembl database, whereas others are not. Importantly, we found indications that eleven more zebrafish class II loci are expressed than previously reported in article form (assuming that those previously discussed transcripts all mapped to the region around *SLC7A4*, and haplotype-dependent concerned the *D8.37A3*/*D8.37B3*/*D8.37A4* loci), including DA group *D8.35A1* (cDNA report in GenBank accession DT880928), *D8.35A2* (e.g. GenBank EH498789), *D8.35B2* (GenBank CK030151), *D8.35B3* (e.g. GenBank DN896667), *D8.37A2* (e.g. Genbank EG576857), *D8.37B1* (e.g. CN023898), *D4A* (e.g. GenBank CF996478) and *D4B* (GenBank EB769231), and DB group *D8.45B1* (GenBank CO798204), *D8.46A* (e.g. GenBank EH450259), *D18B* (GenBank EH441901) (Additional file 5, Table S2). Future research should clarify whether these additionally expressed loci are mono-, oligo- or polymorphic.

Stickleback (*Gasterosteus aculeatus*)

Stickleback was estimated to have, on average, six DA group class II *B* loci per haploid genome [Sato et al. 1998; Reusch et al. 2001], which agrees well with the Ensembl database (main text Fig. 2 and Additional file 2, Fig. S2). Despite many sequence reports [e.g. Sato et al. 1998; Reusch et al. 2001; Reusch and Langefors 2005] there is no conclusive evidence on stickleback class II *B* allelic polymorphism, in part due to haplotype variation, recent gene duplications, and interlocus recombination events [Reusch et al. 2001; 2004; 2005]. Difference in gene copy number also hampers the distinction between allelic versus haplotype variation when comparing the scaffold G131 sequence (main text Fig. 2) with a region that, according to non-MHC genes, is allelic and harbors "*DAA*", *DAB*", *DBA*" and "*DBB*" loci [reported by Reusch and co-workers, 2004] (those class II sequences are included for comparison in main text Fig. 4, Additional file 3, Fig. S3, and Additional file 10, Text S4).

Stickleback DB group genes such as those found on GXVII and GXX (main text Fig. 2) have, to our knowledge, not been discussed before. We found the *GXVII B* gene expressed (Additional file 5, Table S2), but, as typical for DB group genes, the matching ESTs (GenBank DW594610, DW039681, DN724325, DT950896, DN681207, DN670562) are not suggestive of polymorphism.

Medaka (*Oryzias latipes*)

There are not many publications on medaka MHC class II, and the available information doesn't allow firm conclusions on polymorphism. However, sequence comparison (Additional file 3, Fig. S3, and Additional file 10, Text S4) suggests that the DA group class II *B* gene on scaffold 873 (M873) is an allelic variant of *DBB* [Nonaka et al. 2001]. *DBB* was mapped together with the quite similar *DAB* locus to chromosome 18 (sequence details of that region are not known), apart from DA group *DCB* which was mapped to chromosome 3 and which is nearly identical to Ensembl *M3B* [Naruse et al. 2000a; 2000b; Nonaka et al. 2001; Additional file 3, Fig. S3, and Additional file 10, Text S4]. Medaka DB group genes map to Chr.16 [identified by Ohashi et al. 2010] and Chr.5 (main text Fig. 2), the latter of which to our knowledge have not been discussed

before. A cDNA clone represented by two ESTs (GenBank BJ877464, BJ891116) fully matches *M5A*, and this DB group gene may be non-polymorphic.

Fugu (*Takifugu rubripes*)

Fugu class II genes have hardly been studied, and there are few ESTs in the database. The neoteleost specific intron in the β2 domain coding sequence, however, has been noted (Lim and Brenner 1995). All the detected class II genes belong to the same subfamily within the DA group (main text Fig. 4 and Additional file 3, Fig. S3), half of them representing pseudogenes (main text Fig. 2, Additional file 2, Fig. S2, and Additional file 5, Table S2). Reported cDNAs are similar though not identical to the intact *A* and *B* genes on scaffold F7533 and *A* gene on F402 (e.g. AB453019 and CA846190 to F7533A; Additional file 7, Text S1), which is suggestive but not conclusive for allelic polymorphism.

Tetraodon (*Tetraodon nigroviridis*)

Also Tetraodon class II genes have hardly been studied, but there are quite a number of class II single pass read cDNA sequences in the NCBI non-redundant (nr) database (Jaillon et al. 2004). As in Fugu, all the detected class II genes belong to the same subfamily within the DA group (main text Fig. 4 and Additional file 3, Fig. S3). Comparison between the reported cDNA sequences and the Ensembl genomic sequences suggests that the genes *T19A* (e.g. match with cDNA in GenBank accession CR731060), *T19B* (e.g. GenBank CR727059), *T55A* (similar GenBank matches as for *T19A*, although not as perfect, so maybe only *T19A* and not *T55A* is expressed) and *T61A* (e.g. GenBank CR682088) are expressed (Additional file 5, Table S2) and polymorphic (e.g. GenBank CR727059 vs. CR697461; Additional file 7, Text S1), but more research is needed.

Tilapia (*Oreochromis niloticus*)

For the cichlid tilapia, we found 60 *A* and *B* sequences in the Ensembl genome of which 30 presumably are pseudogenes. For layout reasons the short scaffolds O42, O49, O50, O77, O745, O779, OA35 and OA78 (Additional file 2, Fig. S2), which we deem to provide no extra information, are not shown in main text Fig. 2. Matching cDNAs were found for the DA group loci *O79A3* (e.g. GenBank GR671091), *O79B2* (GenBank FF281534), *O80B* (GenBank GR702301) and *O845A* (GenBank GR696948), and the DB group loci *O29A3* (e.g. GenBank GR667686) and *O57B* (GenBank GR627548) (Additional file 5, Table S2), and they suggest that there is some limited polymorphism in the DA group loci *O79A3*, *O79B2*, and *O845A* (Additional file 7, Text S1). The DB group *O29A3* locus was designated "*DAA*" and found non-polymorphic [Murray et al. 2000]; the "*DAA*" name may have to be reconsidered since this locus belongs to the nonclassical DB group (main text Fig. 4). Previously reported tilapia DA group sequences were distinguished as "DBA-type" and "DCA-type" by Murray and co-workers [2000], and are exemplified in main text Fig. 4 by the full-length tilapia and related cichlid *A. hansbeanschi* sequences DBA and DCA, respectively. The tilapia DA group "*DBA*-type" and "*DCA*-type" genes were reported to map to multiple loci linked within a single chromosome with extensive haplotype variation [Murray et al. 2000], also including DA group *B* genes [Malaga-Trillo et al. 1998; Murray et al. 2000].

Southern blot analysis showed that tilapia haploid genomes contain more than ten *B* loci of the DA group [Malago-Trillo et al. 1998], consistent with the Ensembl database (main text Fig. 2 and Additional file 2, Fig. S2). Recently, while preparing our manuscript, an extensive MHC class II analysis of tilapia, investigating the tilapia Ensembl database as well as BAC sequences, was published by Sato and coworkers [2012], confirming haplotype variation between individuals and showing clustering of related genes within single genomic regions. They also provided the first descriptions of DB group sequences in this species other than "*DAA*". In their nomenclature the DB molecules encoded by the region represented by Ensembl scaffold O29 (our "S1 synteny region"; main text Fig. 2) are designated as "A-type", by O33 and O57 as "Y-type", and by O97 (our "S2 synteny region") as "Z-type". They collectively designate the DA group sequences as "IIb family" and the DB group sequences as "IIa family", which refers to the original naming of the nonclassical tilapia II *A* locus as "*DAA*". They did not extensively compare features between the DA and DB families, and they did not perform a comprehensive MHC class II gene search across teleost species.

References

Bingulac-Popovic J, Figueroa F, Sato A, Talbot WS, Johnson SL, Gates M, Postlethwait JH, Klein J (1997) Mapping of mhc class I and class II regions to different linkage groups in the zebrafish, *Danio rerio*. *Immunogenetics* 46(2):129-134.

Graser R, Vincek V, Takami K, Klein J (1998) Analysis of zebrafish Mhc using BAC clones. *Immunogenetics* 47(4):318-325.

Harstad H, Lukacs MF, Bakke HG, Grimholt U (2008) Multiple expressed MHC class II loci in salmonids; details of one non-classical region in Atlantic salmon (*Salmo salar*). *BMC Genomics* 9:193.

Jaillon O, et al. (2004) Genome duplication in the teleost fish *Tetraodon nigroviridis* reveals the early vertebrate proto-karyotype. *Nature* 431(7011): 946-957.

Kuroda N, Figueroa F, O'hUigin C, Klein J (2002) Evidence that the separation of Mhc class II from class I loci in the zebrafish, *Danio rerio*, occurred by translocation. *Immunogenetics* 54(6):418-430.

Lim EH, Brenner S (1995) Sequence analysis of Mhc class II beta-like fragments in the pufferfish Fugu rubripes. *Immunogenetics* 42(5):432-433.

Málaga-Trillo E, Zaleska-Rutczynska Z, McAndrew B, Vincek V, Figueroa F, Sültmann H, Klein J (1998) Linkage relationships and haplotype polymorphism among cichlid Mhc class II B loci. *Genetics* 149(3): 1527-1537.

Murray BW, Shintani S, Sültmann H, Klein J (2000) Major histocompatibility complex class II A genes in cichlid fishes: identification, expression, linkage relationships, and haplotype variation. *Immunogenetics* 51(7):576-586.

Naruse K, et al. (2000a) A detailed linkage map of medaka, *Oryzias latipes*: comparative genomics and genome evolution. *Genetics* 154(4):1773-1784.

Naruse K, Shima A, Nonaka M (2000b) in *Major Histocompatibility Complex Evolution, Structure, and Function*, ed Kasahara M (Springer-Verlag, Tokyo), pp 91-109.

Nonaka M, Matsuo M, Naruse K, Shima A (2001) Comparative genomics of medaka: the major histocompatibility complex (MHC). *Mar Biotechnol (NY)* 3(Supplement 1):S141-4.

Ohashi K, Takizawa F, Tokumaru N, Nakayasu C, Toda H, Fischer U, Moritomo T, Hashimoto K, Nakanishi T, Dijkstra JM (2010) A molecule in teleost fish, related with human MHC-encoded G6F, has a cytoplasmic tail with ITAM and marks the surface of thrombocytes and in some fishes also of erythrocytes. *Immunogenetics* 62(8):543-559.

Ono H, Klein D, Vincek V, Figueroa F, O'hUigin C, Tichy H, Klein J (1992) Major histocompatibility complex class II genes of zebrafish. *Proc Natl Acad Sci U S A* 89(24):11886-11890.

Reusch TB, Häberli MA, Aeschlimann PB, Milinski M (2001) Female sticklebacks count alleles in a strategy of sexual selection explaining MHC polymorphism. *Nature* 414(6861): 300-302.

Reusch TB, Schaschl H, Wegner KM (2004) Recent duplication and inter-locus gene conversion in major histocompatibility class II genes in a teleost, the three-spined stickleback. *Immunogenetics* 56(6):427-437.

Reusch TB, Langefors A (2005) Inter- and intralocus recombination drive MHC class IIB gene diversification in a teleost, the three-spined stickleback *Gasterosteus aculeatus*. *J Mol Evol* 61(4):531-541.

Sato A, Figueroa F, O'hUigin C, Steck N, Klein J (1998). Cloning of major histocompatibility complex (Mhc) genes from threespine stickleback, *Gasterosteus aculeatus*. *Mol Mar Biol Biotechnol* **7(3)**:221-231.

Sato A, Dongak R, Hao L, Shintani S, Sato T (2012) Organization of Mhc class II A and B genes in the tilapiine fish Oreochromis. *Immunogenetics* 64(9):679-690.

Shum BP, Guethlein L, Flodin LR, Adkison MA, Hedrick RP, Nehring RB, Stet RJ, Secombes C, Parham P (2001) Modes of salmonid MHC class I and II evolution differ from the primate paradigm. *J Immunol* 166(5):3297-3308.

Stet RJ, de Vries B, Mudde K, Hermsen T, van Heerwaarden J, Shum BP, Grimholt U (2002) Unique haplotypes of co-segregating major histocompatibility class II A and class II B alleles in Atlantic salmon (*Salmo salar*) give rise to diverse class II

genotypes. *Immunogenetics* 54(5):320-331.

Sültmann H, Mayer WE, Figueroa F, O'HUigin C, Klein J (1994) Organization of Mhc class II B genes in the zebrafish (*Brachydanio rerio*). *Genomics* 23(1):1-14.

Sültmann, Sato A, Murray BW, Takezaki N, Geisler R, Rauch GJ, Klein J (2000) Conservation of Mhc class III region synteny between zebrafish and human as determined by radiation hybrid mapping. *J Immunol* 165(12):6984-6993.
