# Supplementary material for: Comprehensive analysis of MHC class II genes in teleost fish genomes reveals dispensability of the peptide-loading DM system in a large part of vertebrates
Source: BMC Evol Biol. 2013 Nov 26;13:260. doi: 10.1186/1471-2148-13-260 (PMC4219347; doi:10.1186/1471-2148-13-260)
Supplement: Additional file 10: Text S4 — MHC class II domain sequence alignments. [file 1471-2148-13-260-S10.pdf]

Supplementary Text 4 (Text S4): MHC class II domain sequence alignments

Table of Contents		Page
Figure legend		1
	MHC class II alpha sequence references	2
	MHC class II beta sequence references	3
Text S4*A*	Alpha 1 domain sequence alignment	4
Text S4*B*	Beta 1 domain sequence alignment	7
Text S4*C*	Alpha 2 domain sequence alignment	10
Text S4*D*	Beta 2 domain sequence alignment	13
Text S4*E*	Transmembrane and cytoplasmic domains alignment	17
	(a) MHCIIα transmembrane/cytoplasmic domains	17
	(b) MHCIIβ transmembrane/cytoplasmic domains	17

Figure legend:

Alignments of deduced amino acid sequences of MHC class II α1 domains (Text S4*A*), β1 domains (Text S4*B*), α2 domains (Text S4*C*), β2 domains (Text S4*D*) and transmembrane/ cytoplasmic domains (Text S4*E*) of teleost fish and other vertebrates. Also some sequences encoded by pseudogenes and/or genes that are only known partially are shown (both types with "X"), as they also may contain information on family history. Further, it is indicated which genes were found highly expressed in the present study (high). Names shaded pink, blue and green belong to teleost MHC class II groups DA, DB and DE, respectively. In the sequences, basic residues are depicted in red font, acidic residues in blue font, and green font residues are more hydrophilic than the orange ones [Hopp TP, Woods KR (1981) Prediction of protein antigenic determinants from amino acid sequences. *Proc Natl Acad Sci USA* 78(6)**:**3824-3828]; cysteines are in purple font. Numbers between brackets in the alignment figures refer to the sequence reference lists (pages 2-3), which provide the species common name, species Latin name, gene name (if given), and database accession.

Text S4*A*-to-*D*: Above the alignment interesting motifs that are found in some of the aligned sequences are indicated. Yellow shaded asparagine and histidine residues in the α1 and β1 domain alignments are highly conserved in classical MHC class II for interaction with the backbone of the peptide ligand [Painter CA, Stern LJ (2012) Conformational variation in structures of classical and non-classical MHCII proteins and functional implications. *Immunol Rev* 250(1):144-157] (see also Additional file 6, Table S3). Pink shaded serine and glutamic acid residues in the β2 domain can interact with CD4 [Wang XX, et al. (2011) Affinity maturation of human CD4 by yeast surface display and crystal structure of a CD4-HLA-DR1 complex. *Proc Natl Acad Sci USA* 108(38):15960-15965] and are highly conserved throughout classical tetrapod MHC class II. The aqua-color shaded tryptophan residue in the α1 domain alignment (W43) is critical in classical MHC class II for allowing DM assistance in peptide loading, a function for which in DM the tan-color shaded asparagine residue in the α2 domain alignment (N125) is involved [Pos W, et al. (2012) Crystal structure of the HLA-DM-HLA-DR1 complex defines mechanisms for rapid peptide selection. Cell 151(7):1557-1568]. Conserved N-glycosylation motifs are shaded gray. The downward arrowhead in Text S4*D* corresponds with the position where the DA-lineage β2 domain coding sequences in neoteleost fishes are separated by an unusual intron [first reported by: Ono H, et al. (1993) Exon-intron organization of fish major histocompatibility complex class II B genes. *Immunogenetics 3*8(3):223-234] providing evidence that they are monophyletic and derived from a single gene ancestor in an early neoteleost. Deduction based on available information and parsimony suggests that the S3 genomic region (main text Fig. 2) was the genomic site of this neotelost gene ancestor.

Text S4*E*: Aligment of transmembrane and cytoplasmic domain regions of MHC class II α (a) and II β (b). These small domains are difficult to predict reliably from genomic sequences, and therefore we did not attempt to make a comprehensive analysis of teleost molecules. Only a few sequences are compared in order to show principles. For all presented sequences cDNA information is available, except for those of gar (*). Cart., cartilaginous fish classical type. T.cl., tetrapod classical type. Putative transmembrane regions are underlined according to prediction by TMPRED software (http://www.ch.embnet.org/software/TMPRED_form.html).

Classical MHC class II molecules are co-transported to MHC class II loading compartments by binding to invariant chain and do not have proper endosomal sorting motifs themselves. In contrast, DM molecules are transported to these compartments due to their YXXΦ motif (X can be any residue, Φ denotes a hydrophobic residue) in the DMB cytoplasmic tail [Lindstedt R, et al. (1995) The MHC class II molecule H2-M is targeted to an endosomal compartment by a tyrosine-based targeting motif. *Immunity* 3(5): 561-572.]; in the figure these tyrosine-based motifs are shaded yellow. Other endosomal sorting motifs contain "dileucine"(-similar) motifs LL, IL, or LI, for which an acidic residue positioned several residues N-terminal thereof is important for efficiency [Kozik P, et al. (2010) A screen for endocytic motifs. *Traffic* 11(6):843-855]; such motifs are shaded green in the alignment figures. However, although such motif in the DOB cytoplasmic tail has been demonstrated to be functional, transport of DO molecules importantly depends on their interaction with DM [Brunet A, et al. (2000) Functional characterization of a lysosomal sorting motif in the cytoplasmic tail of HLA-DObeta. *J Biol Chem* 275(47):37062-37071].

Gray and blue shading are used to show two conserved sets of small residues glycine, alanine, serine, or cysteine, positioned seven residues apart (presumably positioned above each other at approximately two complete helical turns, forming one side of the helix), which maybe be involved in helix packing and binding between the MHC class II α and β chains [Cosson P, Bonifacino JS (1992) Role of transmembrane domain interactions in the assembly of class II MHC molecules. Science 258(5082):659-662; Moore DT, et al. (2008) Protein-protein interactions in the membrane: Sequence, structural and biological motifs. *Structure Review* 16(7):991-1001]

Pink shading is used to show an ancestral MHC class II β cytoplasmic tail motif which was not previously reported, consisting of a glycine encoded by an intron-interrupted codon and a proline five residues C-terminal thereof.

<u>MHC class II alpha sequence references</u>

(1) Atlantic salmon, *Salmo salar*, DAA*0101, GenBank L77086
(2) rainbow trout, *Oncorhynchus mykiss*, DAA*0101, GenBank CAB96450
(3) Arctic char, *Salvelinus alpinus*, DAA, GenBank ACI05079
(4) Zebrafish, *Danio rerio*, 1.3.4, GenBank AAA16368
(5) Zebrafish, *Danio rerio*, 11.2, GenBank AAA72019
(6) Common carp, *Cyprinus carpio*, DXA1*01 , GenBank CAA64707
(7) Atlantic herring, *Clupea harengus*, DAA*0101, GenBank CAM34665
(8) Three-spined stickleback, *Gasterosteus aculeatus*, DAA*01, GenBank AAU01917
(9) Three-spined stickleback, *Gasterosteus aculeatus*, DBA*01, GenBank AAU01919
(10) Nile tilapia, *Oreochromis niloticus*, DBA*s01, GenBank AAF66843
(11) Fort Maguire Aulonocara, *Aulonocara hansbaenschi*, DCA*s01, GenBank AAF65681
(12) Nile tilapia, *Oreochromis niloticus*, DAA*0101, GenBank AF214705
(13) Zebrafish, *Danio rerio*, DCA, GenBank AL591180
(14) Red seabream, *Sparus aurata*, EST sequence, GenBank AM965522
(15) Common carp, *Cyprinus carpio*, GSS sequence, GenBank HN151524
(16) Japanese loach, *Misgurnus anguillicaudatus*, EST sequence, GenBank BJ838834
(17) White sturgeon, *Acipenser transmontanus*, EST sequence, GenBank DR975335
(18) Little skate, *Leucoraja erinacea*, EST sequence, GenBank FL670178

(19) Nurse shark, *Ginglymostoma cirratum*, pSα5-1, GenBank AAA49311
(20) Coelacanth, *Latimeria chalumnae*, Ensembl LatCha1 scaffold JH122224
(21) African clawed frog, *Xenopus laevis*, DAAf1, GenBank AF454374
(22) Chicken, *Gallus gallus*, B-LA, GenBank AAR14673
(23) Human, *Homo sapiens*, HLA-DQA1 , GenBank AAA59758
(24) Human, *Homo sapiens*, HLA-DRA1, GenBank NP_061984
(25) Mouse, *Mus musculus*, H2-DOA , GenBank AAB46387
(26) Human, *Homo sapiens*, HLA-DOA , GenBank AAA60075
(27) African clawed frog, *Xenopus laevis*, DMA, GenBank AAH61681
(28) Anole lizard, *Anolis carolinensis*, Ensembl AnoCar2.0 Chr.4
(29) Chicken, *Gallus gallus*, BMA1, GenBank CAA18966
(30) Human, *Homo sapiens*, HLA-DMA , GenBank CAA54170

MHC class II beta sequence references

(31) Atlantic salmon, *Salmo salar*, DAB*0101, GenBank CAA49725
(32) Rainbow trout, *Oncorhynchus mykiss*, DAB*1602, GenBank AF115531
(33) Arctic char, *Salvelinus alpinus*, DAB, GenBank ACI05078
(34) Zebrafish, *Danio rerio*, DAB1*01, GenBank AAA50043
(35) Zebrafish, *Danio rerio*, DAB2*01, GenBank AAA50044
(36) Zebrafish, *Danio rerio*, DAB3*01, GenBank AAA50045
(37) Zebrafish, *Danio rerio*, DAB4, GenBank AAA87891
(38) Zebrafish, *Danio rerio*, DDB, GenBank AAA87893
(39) Zebrafish, *Danio rerio*, DEB, GenBank AAA87895
(40) Zebrafish, *Danio rerio*, DFB, GenBank AAA87892
(41) Common carp, *Cyprinus carpio*, DAB4*01, GenBank CAA64709
(42) Three-spined stickleback, *Gasterosteus aculeatus*, DAB*01, GenBank AAU01918
(43) Three-spined stickleback, *Gasterosteus aculeatus*, DBB*01 , GenBank AAU01920
(44) Medaka, *Oryzias latipes*, DAB, GenBank BAA94279
(45) Medaka, *Oryzias latipes*, DBB, GenBank BAA94280
(46) Medaka, *Oryzias latipes*, DCB, GenBank BAA94282
(47) Pigmy swordtail, *Xiphophorus pygmaeus*, DXB*01, GenBank AAS55044
(48) Zebrafish, *Danio rerio*, DBB, GenBank BRU08869
(49) Zebrafish, *Danio rerio*, DCB, GenBank AAA87894
(50) Fathead minnow, *Pimephales promelas*, EST sequence, GenBank DT151632
(51) Mississippi paddlefish, *Polyodon spathula*, DAB*01, GenBank ACZ26346
(52) Mississippi paddlefish, *Polyodon spathula*, DAB*02, GenBank ACZ26347
(53) Mississippi paddlefish, *Polyodon spathula*, DAB*03, GenBank ACZ26348
(54) Pacific electric ray, *Torpedo californica*, EST sequence, GenBank EW694773
(55) Nurse shark, *Ginglymostoma cirratum*, clone 11, GenBank L20274
(56) Coelacanth, *Latimeria chalumnae*, Ensembl LatCha1 scaffold JH135383
(57) African clawed frog, *Xenopus laevis*, DAB, GenBank BAA08759
(58) Chicken, *Gallus gallus*, B-LB, GenBank AAA48948
(59) Human, *Homo sapiens*, HLA-DQB*05011, GenBank AAQ88039
(60) Human, *Homo sapiens*, HLA-DRB1*010101, GenBank CAM34749
(61) Mouse, *Mus musculus*, H2-DOB, GenBank AAI27028
(62) Human, *Homo sapiens*, HLA-DOB, GenBank CAG46631
(63) African clawed frog, *Xenopus laevis*, DMB, GenBank ABB85336
(64) Anole lizard, *Anolis carolinensis*, Ensembl AnoCar2.0 Chr.4
(65) Chicken, *Gallus gallus*, BMB2, GenBank CAA18967
(66) Human, *Homo sapiens*, HLA-DMB, GenBank AAB60387

Text S4A: Alpha 1 domain sequence alignment

```
                                                                                    W43
salmon DAA (1)  high       1:VLHID-LVITGCSDSD--------GLDMYGLDGEEMWYADFNKQ-------EGVVALPP 43
trout DAA (2)              1:VLHTD-IYINGCSDSD--------GVDMYGLDGEELWYADFNKK-------EGVVALPP 43
Arctic char DAA (3)        1:LLHID-LYIAGCSDSD--------GVDMYGLDGEELWYADFKKG-------EVVMPLPP 43
D8.35 A1                   1:IVHDY-FSIHGCSYTDEEGMQAVDGEDMHGIDNEEMWHADYNLK-------TGVLSLPD 51
D8.35 A2  high            1:VVHED-FAIRGCSDTE--------KEDTYGMDEEELWHADFNQK-------KGVETLPD 43
D8.37 A1                   1:FEHEV-NQIQVCSNTE--------QEFFIGYDGEEIWHADIAHR-------RGVKTTPS 43
D8.37 A2                   1:FVREE-LEIYVCSDSE--------SEAMVGADGEELWHADFKQK-------TGIITLPD 43
D8.37 A3  high            1:AEHKD-FGFVGCSDME--------KEFLFGFDGEELFHLDFIRK-------EGVATAPD 43
D8.37 A4  high            1:AEHRD-FQFTGCSDTE--------KDDLNGFDGEVLYYTDFIRK-------EGVVIAPD 43
D4 A                       1:VKHNL-FMFNGCSDTE--------KEALYGSEGEEMWHSDFKLK-------QGVATLPE 43
zebrafish (4)              1:AEHRD-VDFFGCSDTE--------KEYLQGFDGEELYHSDFIRK-------VGVVTAPD 43
zebrafish (5)              1:VVHED-IVMDGCSDTE--------KEYISVLDGEEMYHTDFSGK-------RGEMTLPD 43
carp DXA (6)               1:VVNRD-VQFVGCSDTE--------REFLIGFDGEELWHADFIRK-------EGVVTVPD 43
Atlantic herring DAA (7)   1:IVHVD-IALVGCTDSD--------GEKMFGLDGEEKGHADFTKG-------KFIMTLPE 43
G131 A1  high             1:GPHKD-IAIVGCSDSD--------GEEMYGLDGEEVWYADFKHG-------KGVRPQPS 43
G131 A2                    1:GPHKD-IRVLGCSDSD--------GENMFGLDGEEVWYADFKHG-------KGVRPLPS 43
G131 A3                    1:GPHKD-IAIDGCSDSD--------GEKMYGLDGEEVWYADFKHG-------KGVKPQPS 43
GVII A                     1:VPHEA-IRISGCSDSD--------GEEMFGLDGEELWYADFKLG-------KGVSLLPS 43
stickleback DAA (8)        1:GPHRD-IAITGCSDSD--------GEDMYGLDGEELWYADFKHG-------KGVKPQPS 43
stickleback DBA (9)        1:GPHED-FAITGCSDSD--------GEFMYGLDGEEIWYADFKHG-------KGVKPQPS 43
M3 A                       1:VLHED-FGILGCSDYD--------QEYMYALDGEEKWYADYKKE-------TGVGPQLP 43
M615 A                     1:VFHED-FQITGCSDYD--------EEFMVNLDGEEIVYTDFKKG-------TEVDAQPS 43
M873 A  high              1:VFHED-LAITGCSDSD--------GEDMYALDGEEVWYADFKKQ-------TGVEPQPP 43
F402 A                     1:-QHED-IRILGCSDLD--------GEFMYGLDSEEVWYADFSKG-------EGVDINPP 30
F713 A  X                 1:-QHKD-INIVGCSDLD--------GEFMYGLDSEELWYADFSKG-------EGVYGTPP 42
F7533 A                    1:SQHKD-IGIVGCSDLD--------GEFMYGLDSEELWYADFSKG-------EGVYGTPP 43
F7721 A                    1:SQHKD-IRITGCSDLD--------GEDMFGLDSEELWYADFSKG-------EGVDGTPP 43
T19 A                      1:GQHED-IRIVGCSDFD--------GEYMHGLDGEELWYADFRRG-------EGVYAQPD 43
T55 A                      1:GQHED-IRIVGCSDFD--------GEYMYGLDGEELWYADFRRG-------EGVYAQPD 43
T61 A                      1:GRHED-VHIRGCSDFD--------GEDMYGLDGEERWYADFKRG-------EGVYALPD 43
O1 A                       1:FLHED-IHIVGCSESN--------GEVMFGLDGEELWYADFENK-------IGVSPQPS 43
O18 A1                     1:VLHED-IGIVGCSDSD--------GEDMYGLDGEEVRYADFKKQ-------EDINSLPP 43
O18 A2                     1:ALHSD-IAINGCSDSD--------GEFMYGLDGEEKWYADFNKH-------TGVDAPPD 43
O18 A3                     1:PLHSD-IAIGGCSESD--------GENMYGLDGEEIAYADFNKQ-------EFIYPQPP 43
O22 A1                     1:GLHET-SSVSGCSDSD--------GEDVLILDDEVVWYADFYNQ-------KGVDSLPA 43
O22 A2  X                 1:-LYEM-NSVSGCSDTD--------GENVLILDDEVVWYADFNKQ-------KGVDSLPA 42
O22 A3  X                 1:-LYEM-NSVSGCSDTD--------GEDVLILDDEVVWYADFNKQ-------KGVDSLPA 42
O22 A4  X                 1:-LHET-NSISGCSDTD--------GEDALILDDEVVWYADFNKH-------TGVDSLPA 42
O22 A5  X                 1:-LYEM-NSVSSCSDTD--------GEDVLILDDEVVWYADFNKR-------KGVDSLPA 42
O22 A6  X                 1:-LYEM-NSVSSCSDTD--------GEDVLILDDEVVWYADFNKR-------KGVDSLPA 42
O31 A3                     1:SLHTD-IHIVGCSDFN--------GEVMFGLDREELWYADFEKQ-------MDVYPQPP 43
O31 A4                     1:ALHTD-IHVIGCSDFN--------GEVMFGLDREELWYADFEKQ-------MGVSPQPS 43
O39 A  X                  1:GLHGD-VHINGCSDFD--------AEDMYGLDGEEVAYADFNNH-------TEVYILPP 43
O42 A                      1:GLHGD-VHINGCSDFD--------AEDMYGLDGEEVAYADFNNH-------TEVYILPP 43
O79 A1                     1:DAHGS-NFIIGCSDAD--------GENVLILDDEAVWYADFNKQ-------TGVNALPA 43
O79 A3                     1:ALHGD-NWISGCSDTD--------GEAVLILDDEAVWYADFNKQ-------EGIYPLPP 43
O745 A                     1:PLHSD-IAVGGCSESD--------EENMYGLDGEELAYTDFNKK-------EEIYPLPP 43
O845 A                     1:GLYEM-NSVSGCSDTD--------GEDVLILDDEVVWYADFNKQ-------KGVDSLPA 43
tilapia DBA (10)           1:GQHTD-INIVGCSDFD--------GEVMYGLDGEEKWYADFNKH-------IGVYPQPP 43
A.hansbaenschi DCA (11)    1:ALHTD-IHVVGCSYIH--------GGVMFGLNREELWYADFEKQ-------MDVYPQPP 43
---------------------------------------------------------------------------------------------
salmon DBA                 1:IHHEI-HFIFGCFESS-------DPAVGLEIDGDEVFYGDFNKNSNTCLIADVVFTLPK 51
GXVII A                    1:SSHDF-HYTYGCYESG-------EVRVDVLLDGDVVAYADFGRE-------EVVFLIPR 44
M5 A                       1:PRHMF-HFIYGCYETD-------EVRVDLVVDDDTIGYADFTKQ-------EMVWCLPY 44
O97 A                      1:QSHNF-HYIYGCYESD-------DVRVDAVVDDDVVAYADFNKE-------EVVWVLPH 44
salmon DCA                 1:IPHET-VYVLGCLEKT-----KVKAEAELQLDGEEVVYADFQSG-------QEVWTLPE 46
M16 A                      1:VEHEI-SYFIGCFAEG-------STEVLLEFDSEEILYVDFEKE-------AVVFTGPS 44
O29 A3                     1:IPHEM-TYVVGCFVQG-------LTEVQFEFDGEEFLYADFPKE-------EIVYTVPK 44
O9 A                       1:AVHEE-TLFYSCSESG-------DGQVQLHYDGDQITYADFKKQ-------HAVWTAPL 44
O33 A                      1:GRHTL-CHIYGCFDSS-------DTQICAAFDGDEVYYADFKKD-------ELIWESKI 44
O57 A                      1:GSIKL-CYTFGCFKSS-------DTQVCLTMDGDEVFYANFKNG-------SLIWDSKI 44
```

```
D8.45 A1                 1:SYPEF-GFIETCRGSS-------YDKIVFILDTEEAAYKDIEKE-------KLVFTLPD 44
D8.45 A2                 1:MDLEY-GLMESCTGSP-------DDEIVFTFNTELAAYLVVVDG-------TLVLTVPN 44
zebrafish DCA (13)       1:MDLEY-GLMESCTGSP-------DDEIVFTFNTELAAYLVVVDG-------TLVLTVPN 44
D8.46 A                  1:DYHKY-GLIGACGDAD-------QEDFIVQFDDEQLAHVDFKEQ-------KDVITLPE 44
D18 A   X                1:DYHKY-GLIGASGDAD-------QEDFIVQFDDEQLAHVDFKEQ-------KDVITLPE 44
salmon DDA               1:EQHVF-NVINTRSETE-------EFNMTIVVDDNEYLHVYLNNK-------EGVVTLPE 44
spotted gar 501 A2       1:TSHID-INVIGCKSDDP------VAQDEAQLDGDEMFYADFDKK-------EMILTLPA 45
salmon DEA               1:SKHLL-RFLTFCQKNVP-----SDEEYDVEFDGDELFYVDPITY-------QVERRLSE 46
seabream DEA (14)        1:----------------------EKPDDIEFDGDELLYLDPVTY-------QAVQRLPE 29
spotted gar 615 A        1:IRHLD-RLLTFCVSNST------EAELEKEHDEDEIFYVDLETK-------QSVQRLPE 45
sturgeon (17)            1:VTHLF-RALVACQTNGT------LPEDDYELDDDELFHIDFDKK-------EAVQRIPD 45
spotted gar 501 A1       1:VEHTY-EVAYFCQSENR------KGSRNEVFDDDEMFHIDPDRK-------VDEPRLPE 45
little skate (18)        1:YRHEE-HLVYLVQDGSP------DKQFDMLVDDDEVFYMDFKLK-------KEVARIPA 45
nurse shark (19)         1:YLYDF-TQVYFVQQRSP------EKHFDVMEDGDEIFYMDFNLK-------KEVARIPE 45
coelacanth (20)          1:TSLLY-DKTLVCQTAEP------KVQFNWEIDEDELVRVDIDKQ-------KMEWRLPE 45
Xenopus DAA (21)         1:VDYFD-YGAMFYQSYGP------SGEYLFDYEGNEMFHVDLESK-------SVVWTLPG 45
chicken BLA (22)         1:KPHVL-LQAEFYQRSEG--PDKAWAQFGFHFDADELFHVELDAA-------QTVWRLPE 49
human DQA (23)           1:ADHVASCGVNLYQFYGP------SGQYTHEFDGDEQFYVDLERK-------ETAWRWPE 46
human DRA (24)           1:EEHVI-IQAEFYLNPDQ------SGEFMFDFDGDEIFHVDMAKK-------ETVWRLEE 45
mouse DOA (25)           1:ADHMGSYGPAFYQSYDA------SGQFTHEFDGEQIFSVDLKNE-------EVVWRLPE 46
human DOA (26)           1:ADHMGSYGPAFYQSYGA------SGQFTHEFDEEQLFSVDLKKS-------EAVWRLPE 46
Xenopus DMA (27)         1:QDHSL-KQVLFCQPQSP------SPVLLKMFDEEQMFQYNFADK-------SVVPRIPN 45
anole lizard DMA (28)    1:PAHLF-SEVFFCQPGDP------SLGLAQALDQEPLFRFNFSTQ-------QWRSILPE 45
chicken DMA (29)         1:SLHTL-SEVLFCQPDTP------SLGLSVAFDSEQLFSFDVPNS-------QWLPQLPD 45
human DMA (30)           1:QNHTF-LHTVYCQDGSP------SVGLSEAYDEDQLFFFDFSQN-------TRVPRLPE 45

                                                      N62    N69   NxS/T NxS/T
salmon DAA (1)  high            44:FA-----DPFTF---PGF-YEQAVGNQGVCKGNLAKCIKAY---KNP-EEKI     82
trout DAA (2)                   44:FA-----DQISF---PGY-YEQAVGNQRTCKGDLGVDIKAY---KNP-PETI     82
Arctic char DAA (3)             44:FA-----DPFTY---PGA-YEQAVGNQGACKANLATSIKAY---KNP-PETI     82
D8.35 A1                        52:FG-----DPITF---PRF-YETSVIEMTGCKANIVSLTKIF---NSP-PPEM     90
D8.35 A2   high                 44:FG-----DPMTF---PGF-YEGSEGEMAVCKSNLALRIKGF---KSP-PPEM     82
D8.37 A1                        44:FA-----QYAVI---PDI-YERALAFLDFCKYDLHAYIKYF---NNP-PPEM     82
D8.37 A2                        44:FA-----DRIGF---PGF-YEISVDSIAGCRRNLANNIRGF---KSP-PPAM     82
D8.37 A3   high                 44:FA-----DPLSF---PGF-YEAGVAQMEVCKQNLATDIRAY---NSP-QEQL     82
D8.37 A4   high                 44:FA-----DPLSF---PGG-YETGVAQMEVCKQNLATDIKAY---NSP-QEQL     82
D4 A                            44:FA-----DPLSF---PGA-YEESVADQEVCKQNLATSIKAY---NSP-PEEM     82
zebrafish (4)                   44:FA-----DPMSY---PGF-YENSVAQMEVCKQDLATDIKAY---NSP-EEQL     82
zebrafish (5)                   44:FA-----DPFTY---PGT-YEQSLADYETCKHNLDVAAKAY---KSP-LEKL     82
carp DXA (6)                    44:FA-----DPIGF---PGF-YETGVALMEVCKQNLALNIKVY---KPT-DEQL     82
Atlantic herring DAA (7)        44:FA-----DPFKY--EEGA-YEGAVRDKEVCKQNLQVAIQAY---KSP-AEAE     83
G131 A1   high                  44:FV-----DPIDY---REGTYETAVGNQQICRNNLKIDLKAF---KDF-PLEK     83
G131 A2                         44:FV-----DPIEF---PGGAYEQAVTDLQICRSNLKTHVKGL---KDV-PLEK     83
G131 A3                         44:FV-----DPIDY---PAGAYELAVANQQTCRNDLKTDLEAY---KDV-PLEK     83
GVII A                          44:FL-----DPITY---PGG-YEVAVAEQQFCRNNLRIDLEAY---K-F-PLER     81
stickleback DAA (8)             44:FV-----DPIEF---QEGTYELAVGNQQICRINLKNRLKGL---KDV-PLEK     83
stickleback DBA (9)             44:FV-----DPIDY---QEGAYESAVANQQICRNNLKTHVKVF---KDV-PLEK     83
M3 A                            44:LG-----GHFIN---PDG-YQFAKVYEQVCRRNLKALKKTT---KGL-QLEQ     82
M615 A                          44:FV-----GHITY---AGG-YGATVLELQGCRNDLEVIRRFT---KDL-PLKQ     82
M873 A   high                   44:FV-----DHVSY---PGG-YEQAVANQQICRSNLKISRIAM---KDL-PLER     82
F402 A                          31:FI-----DPITY--EEGA-YSSAVADLQTCKTSLDITRKSL---KDM-PPER     70
F713 A   X                      43:FI-----DPFRY--EEGV-YSQAVTELQTCKTNLDSERKAY---KDM-PPET     82
F7721 A                         44:FI-----DPVRF---EGF-YSTAVTNLQICKSNMDVVRKGN---KDM-PPET     82
F7533 A                         44:FI-----DPFR----PTA---AAVADLQTCKYNLDVDRKGN---KDM-PTET     79
T19 A                           44:FI-----DPIKY---EGF-YSTAVAVLQICKFNLGIFRKGY---KDM-PREL     82
T55 A                           44:FI-----DPLKY--AEDP-YSLAVATLQMCKFNLGIFRKGY---KDM-PREL     83
T61 A                           44:FV-----DPLKF--PEGT-YSQAVADQQICKINLGISRKGN---KDM-PREL     83
O1 A                            44:FV-----DPISF--GDGA-YETAVINLYICKTNMQVSQEGM---KDY-PLNH     83
O18 A1                          44:FA-----DLITC---PGC-YEQAVANQQVCKQNLQTLRKMM---KDY-PLEH     82
O18 A2                          44:FI-----GSFNY--QEGA-YENAVANQQICRANLKVDSKGM---KDF-PLEH     83
O18 A3                          44:FI-----DPITV---PGG-YESSVAFQHSCRENMKLFGKGM---KDY-PPEQ     82
O22 A1                          44:FA-----DHPHW--KDGM-YEQAVVNQQICRYNLKMARTAM---KDF-PKQR     83
O22 A2   X                      43:FA-----DHPGF---EGK-YEEAVANQQICRQNLKMARLAM---KDF-PKE-     80
```

```
O22 A3   X              43:FA-----DHPGF---EGK-YEEAVANQQICRQNLKMARMAM---KDF-P---       78
O22 A4   X              43:FA-----DHPHW--QEGM-YEEAVANQQICRQNLKMARSAM---KDF-PKER       82
O22 A5   X              43:FA-----DHPSF---EGK-YEEAVANQQVCRQNLKVARTAM---KDF-PKE-       80
O22 A6   X              43:FA-----DHPSF---EGK-YEEAVANQQVCRQNLKVARTAM---KDF-PKE-       80
O31 A3                  44:FI-----DPISF--GDGA-YETAVSNLYTCRQLLQLSRQGM---KDY-PPLR       83
O31 A4                  44:FI-----DPISF--GAGA-YKTAVSNLNTCRQNLQITREGI---KDF-PPLR       83
O39 A   X               44:FA-----EPMIY---PGA-YKQAVTELKVCRHNLKALRAVM---KDY-PSNN       82
O42 A                   44:FA-----EPMIY---PGA-YKQAVTELKVCRHNLKALSAVM---KDY-PSNN       82
O79 A1                  44:FV-----DPISF---PGF-YEEAVADQQICKQNLMVTRKAM---KDF-PKEL       82
O79 A3                  44:FV-----DPITF---PGQ-YEQAVANQQICRANLKVTRQAM---KDV-PKEL       82
O745 A                  44:FI-----DPITY--GEDA-YESSVAFQKTCRKNMKQFGEGM---KDF-PPEQ       83
O845 A                  44:FA-----DHPSF---EGK-YEEAVANQQICRQNLKMARTAM---KDF-PKQH       82
tilapia DBA (10)        44:FVV----NPFHY--QEGT-YEGAVTNVQVCQNALKIVREAM---KDF-PPKH       84
A.hansbaenschi DCA (11) 44:FV-----QPISF--GDGA-YNIAVANLYGCRQFLQLSETAM---KDF-PPRR       83
salmon DBA              52:FI-----SITPED-KERA-CEYATISRVWCKDCIAWGKQSE---PKI-PKIK       92
GXVII A                 45:L------PPFLRDLKKLG-YEFAKSSFTHCRSVLAKAKRAS---PNV-TIPQ       85
M5 A                    45:V------PPSGKDLEKEA-FKFAKNSIANCHSVLAKAKKAD---HGT-PLRQ       85
O97 A                   45:L------PQPAKDFKKMA-YGVAKASTAHCHSVLGKAKNAD---PGA-PLRQ       85
salmon DCA              47:FL-----GPFSSSTVRNF-YKNAVKGRRLCRDALALWIFEE---KSP-PEVK       88
M16 A                   45:FFKANLSERIQGL---TT-YKNGKKNRIWCQLA-DQYFTA----ETE-ERDK       86
O29 A3                  45:FIFPDPSHVLVGL---SI-LKDALDNRDWCLIITKIAAMEE---KYP-PEEK       88
O9 A                    45:L------KDLETAILSWF-FPLALDSRTRCEFYLDRAVRLD---NSS-MTL-       84
O33 A                   45:P------ISFHLP---DA-YKYAVFYRSMCKNDIYR-WKPD---KSV-IKKK       81
O57 A                   45:P------TAFHVP---YA-YSQALYSQRQCRDFVHDDWKQH---KTI-MNTK       82
D8.45 A1                45:MAN----PQYSLN---DL-RLNDVGIPAFCTEIYHKILSLN---VSEDTEPL       85
D8.45 A2                45:FIKDYP-GFYELPL-LDA-SPATVGLTALCKVVYEKTLNLN---VS---EPL       87
zebrafish DCA (13)      45:FIKDYP-GFYELPL-LDA-SPATVGLTALCKVVYEKTLNLN---VS---EPL       87
D8.46 A                 45:FA-----GQAVLP---PI-YVDAKRAEFNCKAYLAVLREVY---ASS-PEVL       83
D18 A    X              45:FA-----DLAVFQ---PL-YVDAKRA-FNGKAFLAVLREVY---ASS-PEVL       82
salmon DDA              45:W------GNYVDC---PI-CVRVAESRRA--DLNNDIKLFN---LET-PEAK       80
spotted gar 501 A2      46:FA-----DSFGVD--PGW-VQGAIANRQICINNLEVAIKAE---NNP-PENT       85
salmon DEA              47:FA-----QQWTPD--PGL-PHEVYVSLGTCQYNIPRCIVGE---KSP-PEAI       86
seabream DEA (14)       30:FA-----KQWTPD--PRL-AEDAYVDLGTCHYNIPIAMKRD---NNT-PEAI       69
spotted gar 615 A       46:FA-----EKWHVG--PEW-PPFAQQEVETCRLYL--AAKGE---GFP-EEKL       83
sturgeon (17)           46:FA-----KYWSPP--EGA-PARAETDRQTCINNVAVAAKCH---KYP-PERQ       85
spotted gar 501 A1      46:F------EKWAWN-DSSL-IQGALANLGICENHLKGVMKAI---PDE-PAVK       85
little skate (18)       46:FQ-----HITIE----GG-EAGITADIAFAKQNFNVWKNLS---HGS-PEPK       83
nurse shark (19)        46:FA-----HLYMQ----GG-EAGISANIAIVKNNLKVVMNLS---GGT-PEPK       83
coelacanth (20)         46:F------KGHKID---SL-VEWTRQNIPICEHNLDLLIKRT---NGL-LGKT       83
Xenopus DAA (21)        46:L------EKYTS----YD-PQGGLQNINVAKYNLDGYKKSR---NST-AATS       82
chicken BLA (22)        50:F------GRFAS----FE-AQGALQNMAVGKQNLEVMIGNS---NRS-QQDF       86
human DQA (23)          47:F------SKFGG----FD-PQGALRNMAVAKHNLNIMIKRY---NST-AATN       83
human DRA (24)          46:F------GRFAS----FE-AQGALANIAVDKANLEIMTKRS---NYT-PITN       82
mouse DOA (25)          47:F------GDFAH----SD-FQSGLMSISMIKAHLDILVERS---NRT-RAVS       83
human DOA (26)          47:F------GDFAR----FD-PQGGLAGIAAIKAHLDILVERS---NRS-RAIN       83
Xenopus DMA (27)        46:L------KKWANQDLFSN-SSDLAFDIQLCTEAMQNFTQAVV--NIT-PETK       87
anole lizard DMA (28)   46:L------QPEEGN--RTS-LDQVQLLGVICQDILQFLTNISE--AYM-PEAK       85
chicken DMA (29)        46:G------PSWPAD--IEQ-PHELLHDAALCRELLDLLTRIATGPNPM-PEAK       87
human DMA (30)          46:F------ADWAQE--QGD-APAILFDKEFCEWMIQQIGPKLD--GKI-PVSR       85
```

Text S4*B*: Beta 1 domain sequence alignment

```
                                                   NxS/T                           NxS/T
salmon DAB (31)    high       1:DGYFEQVVRQCRY--SSKDLQGIEFIDSYVFNKAEYIRFNSTVGKFVGY---------TEL 50
trout DAB (32)                1:DGYASDVVTRCLY--SSIDMHGAEFIQSYTFNKVEHLRFNSTVGEFVGY---------TEL 50
Arctic char DAB (33)          1:DGYFLQMVRQCQY--SSKDLHGIESIDSYVFNQVENIRFNSTVGKYVGY---------TEV 50
D8.35 B2                      1:NVYYMYRVSRCIF--SSSNISAMVYFDRTYFNKNLFIQFNSNLGRFEGF---------NEY 50
D8.35 B3  high                1:NVYYTYRWCRCIY--SSPDLSDMVYIDSKYFNKYLFAQFNSTLGRYVGF---------SEY 50
D8.37 B1                      1:NVYYRYTWIQCIS--SSKNLSDMVFIESLYFNKYLFTQFNSNIGKYVGF---------SEY 50
D8.37 B3  high                1:DGYYDYIKQQCFY--STSDYSDMVYLASYSFNKVVDTQFNSSVGKFVGY---------TEQ 50
D4 B                          1:DGYYFSTWSQCIY--SYPDLSDMVFILSFNFNKWMFLQFNSTVGIFVGY---------TEQ 50
zebrafish DAB1 (34)           1:DGYYQYTMLECIY--STSDYSDMVLLESGSFNKVVDVQYNSTVGKYVGY---------TEQ 50
zebrafish DAB2 (35)           1:DGYYDYTKYECIY--STSDYSDMVFLQSYSFNKIVDVKCNSQEVKCVGY---------TEQ 50
zebrafish DAB3 (36)          1:------------Y--SSSDYSDMVLLISYSFNKVVDTQFNSSVGKFVGY---------TEQ 38
zebrafish DAB4 (37)           1:DGYYDYIKQQCFY--STSDYSDMVYLASYSFNKVVDTQFNSSVGKFVGY---------TEQ 50
zebrafish DEB (39)            1:DGYYHSRLTKCIF--QLQDLSDIGVHDNYIFNKDVYIRFNSTLGYFVGY---------TEH 50
zebrafish DFB (40)            1:DGYYSSTWSQCIY--SYPDFRDMEFIVGYYFNKWMDIQFNSTVGKFVGY---------TEQ 50
carp DAB (41)                 1:DGYYEYTMYECVY--STSDYSDMVYLVSLSFNQVVDVQCNSSAVKCVGY---------TEE 50
G131 B1                       1:DGFMEFVATECVF--NSTELKDIEYIQSYYYNKLEYTRFSSSVGKFVGF---------TER 50
G131 B2  X                    1:DGFLEFMTDECVF--NSTELKDIEYITSYYYNKLELTRF---------------------- 37
G131 B3                       1:DGFMEFRTDECVF--NSTELKDIEFIRSSYYNKKEDTRFSSSVGKFVGF---------TEQ 50
GIII B                        1:DGFLEFMTDECVF--NSTELKDIEYIRSYYYNKLELTRFSSSVGKYVGF---------TEY 50
GVII B                        1:GGFMEFVKDQCVF--NSTDLKGIEFIRSTYFNKLQYTKFSSSVGKFVGF---------TEQ 50
stickleback DAB (42)          1:DGFMMFVTDECVF--NSTELKDIEFIRSSYFNKKEDTRFSSSVGKFVGF---------TEQ 50
stickleback DBB (43)          1:DGFMEFRTFRCVF--NSTELKDIEYIDSYFFNKLELTRFSSSVGKFVGF---------TEY 50
M3 B                          1:DGFIHYILNRCLF--NSSDLKDIENIYSYYYNKEEFLRFSSSSGKFVGY---------TEI 50
M615 B                        1:DGFLEYDMHRCLF--NSSDMKDIEYIYSKHYNKEEVIRFSSSLGKYVGY---------TKF 50
M873 B  high                  1:DAFLRYDVDRCVF--NSTDLKDIEYIYSMYYNKKEFTRFSSSLGKYVGY---------TEY 50
medaka DAB (44)               1:DAFMEYAVNRCEF--NSSDLTDIEYIYSMYYNRKEYARFSSSLGKYVGY---------TEF 50
F7533 B                       1:GGFQSYVVDSCDF--NSTDLKDIEYTRSLYYNRVMYARFSSRVGKFEGY---------TKY 50
F7721 B  X                    1:GGFQSYRVDRCDF--NSTDLKDIEYSRSHYYNKLMYVRFRSSVGKFEGY---------TKD 50
F8524 B  X                    1:GGFQSYRVDRCDF--NSTDLKDIEYSRSHYYNKLMYVRFRSSVGKFEGY---------TKD 50
T19 B                         1:GAFEHYGLRRCDF--TSAEPKDMEYSLSVYYNKHLMARFSSSVGKFVGY---------DKY 50
T61 B   X                     1:GGFEHYEVDLCDF--TSAELKDIEYSRSLYYNKHLYARFSSSVGTFVGF---------TKY 50
O1 B                          1:DGFLEYIVDRCDF--NSTELENMEYIYSHYYNKMEYIRFSSNVGQYVGF---------TDF 50
O18 B1                       1:GGFMHYSVDRCNF--NSSEPQDIEYIRSYYFRKVEFTRFDSSVGKYVGY---------TEY 50
O18 B2                       1:DGFMEYHVDRCNF--NSTDLKDIEYIYSCYYNKIEYARFSSSVGKFVGF---------TKH 50
O18 B3   X                    1:DGFKMYVVSRCDF--NSTELKDIKYIRSYYYNKIEVTRFDSDVRKFVGF---------TGF 50
O22 B1                       1:DGYEYYTSSRCLF--NSTELRDIEYIRSYYYNKTEYVRFSSSVGKFVGY---------TEY 50
O22 B2   X                    1:-GYEYYTSSRCLF--NSTELRDIEYIRSYYYNRIEYVRFSSSVGMFVGY---------TEY 49
O31 B1                       1:DGFLEYMVERCKF--NSTELKDMEYIYSHYYNKIEYIRFSSSVGKYVGV---------TEF 50
O31 B2                       1:--FLEYIVDRCEF--NSTELKDMEYIYSHYYNKIEYIRFSSSVEQYVGF---------TEY 48
O31 B3                       1:DGFLEYIVDRCEF--NSTELKEMEYIYSHYYNKIEYIRFSSSVGKYVGV---------TDF 50
O79 B1   X                    1:DGYELYNSDRCLF--NSTELQDVEFIRSCYYNKIEFVRFSSDVGKFVGY---------TDF 41
O79 B2                       1:DGYECYSSTRCLF--NSTELKDIEFITSYYYNKIEYVRFSSDVGKFVGY---------TEY 41
O80 B                        1:DGFMTYRLSRCDF--NSTELKDIQFTDSYYYNMIEIIRFDSNVGKFVGF---------TDF 50
O745 B                       1:DGFKMYVLSRCDF--NSTELKDMKYIRSYYYNKIEVTRFDSDVRKFVGY---------TEY 50
O845 B                       1:DGYEYYTSSRCLF--NSTELRDIEYIRSYYYNKTEYVRFSSSVGKFVGY---------TEY 50
OA35 B   X                    1:-GYEYYTSSRCLF--NSTELRDIEYIRSYYYNRIEYVRFSSSLGMFVGY---------TEY 49
OA78 B   X                    1:DGYEYYTSSRCLF--NSSELRDIEYVRSYYYNRIEYVRFSSSVGMFVGY---------TEY 50
----------------------------------------------------------------------------------------------
salmon DBB                    1:DEDFAHDDAWCRF--SSRDLHNMEYILEHHFNKILVAQYNSTTERWTGY---------TAW 50
GXVII B                       1:HGYFMYADFWCNM--QTARPQQVEYLVDWYFNKEFTMQYNSTVGKWTGF---------TAA 50
M5 B1                         1:HGYFMFSDFFCYI--PSRNPKEVQYLIDWYFNMELTMQYNSSVGGWTGF---------TPA 50
M5 B2                         1:HGYFMFSDFLCYL--PSRNPKEVQYLIDWYFNMELTMQYNSSVGGWTGF---------TPA 23
O97 B1   X                    1:DGYFMFADFCCVM--HSRDPKTVEYLIDWYFNKEFTMQYNSTVGKWTGL---------TPA 50
O97 B2   X                    1:DGYFMFADFWCAV--HSRDREEVEYLIDWYFNKEFTMQYNSTVGKWTGL---------TPA 50
salmon DCB                    1:DGYFGHFEMRCWF--SSEDPRDIEYLLQVYGNKKLLGQYNSTTEKCTVY---------TQW 50
M16 B                         1:NAFYGHGTLKCQF--TSS--HDLVYLEQVYFNKRLMVQYNSTLGKYEGY---------TKK 48
O29 B1                       1:DAYFGYGVVRCLF--TSKD--DVVYLAQVYLNKMLLGQYNSSLGKYVGY---------TEK 48
O29 B2   X                    1:-------LIRCLF--TSKD--DVVYLAQIYFNKVLLVQYNSTLGKYVGY---------TEK 41
O29 B3   X                    1:--------------------------EQFYFNKVLECQYNSSVGKVVGY---------TKN 26
swordtail DXB (47)            1:GAFYLSVLERCQS--SLTDGHDAVLLDQVYFNKILEGQYNSTAGKVIGY---------TEK 50
O9 B                          1:DEKYFHILKTCQFI-EKSSQSDVHYQVQYWYNGLLQAFYNSSTEKVVGF---------THY 51
```

```
O57 B                       1:DEDYFQSRACCTF--KGPHFEDMEYTRIISLNKIAVLEYNHTRGSWIGF---------TPY 50
D18 B                       1:HGHYGFVQFTCHM--LGS-LQNVEVTYSIYFDTTELLRFNSTENKAVAY---------TEY 49
zebrafish DBB (48)          1:HGHYGFVQFTCHM--LGS-LQNVEVTYSIYFDTTELLRFNSTENKAVAY---------TEY 49
D8.45 B1                    1:HAYYTYAQIQCHV--SDS-LQKIEFIFSVTYNMIELVRYNSTEDTFFGY---------TAI 49
D8.45 B2                    1:CGNYGYLQSQCRV--LSS-TKKVELIFSFIFNKIEYIRYNSTDQKIVGY---------TEF 49
zebrafish DCB (49)          1:CGNYGYLQSQCRV--LSS-TKKVELIFSFIFNKIEYIRYNSTDQKIVGY---------TEF 49
salmon DEB                  1:GGYQFQGIVDCEY--DDT-IDNMIYFVKNIFNQKLTTIYDSRVQKYVGF---------GEF 49
fathead minnow DEB (50)     1:DVYVFQNIVECEY--SKVDLSDMVYIIKLVFNQKLLCSYDSRLGKYVGY---------DEF 50
spotted gar 501 B1          1:DDFVYFFRSECHF--SASDLRDLVYVRSMTFNGKERVRFNSTVGKYVGF---------DTW 50
spotted gar 615 B           1:DGNMYQFVHDCEY--NDH-LEDFLYTRRDIFNKIEILRYDSNIQTFVGY---------TPL 49
paddlefish DAB01 (51)       1:EGYLMQTLVDCEY--SDSSMTDMVYSWNYVFNKQEVVHYDSKIKKYVGN---------TAC 50
paddlefish DAB02 (52)       1:EGYLMQTLVDCEY--SDSSMTDMVLSWNYVFNKQEVVHYDSKIKKYVGN---------TAC 50
paddlefish DAB03 (53)       1:EGYLMEMMADCEY--SDSSMTDMVYSRSYVFNKQEFIHYDSKIKKYVGN---------TAC 50
ray (54)                    1:GAHSLTKIGGCAF--NSSAPGEWTYFSKGIYDGEVIWYFDFDQRKFVAV---------KGW 50
nurse shark(55)             1:GAHSEISLHRCVF--NST--GDWVFLKQQVYDQELIAYYDYNQRKFIAV---------KAW 48
coelacanth (56)             1:ISNVEQYQWECHYT-NGT--QDIDFIHRVIYNGQEESYFDSRIGKFTGV---------TEW 49
Xenopus (57)                1:EDYVYQYKAQCYFR-NGT--DNVRLLWRHYYNLEETDYFDSDVGLFIAK---------TEL 49
chicken BLB (58)            1:AFFFCGAISECHYL-NGT--ERVRYLQRYIYNRQQFTHFDSDVGKFVAD---------SPL 49
human DQB (59)              1:EDFVYQFKGLCYFT-NGT--ERVRGVTRHIYNREEYVRFDSDVGVYRAV---------TPQ 49
human DRB (60)              1:PRFLEQVKHECHFF-NGT--ERVRFLDRYFYHQEEYVRFDSDVGEYRAV---------TEL 49
mouse DOB (61)              1:ENFVIQAKADCYFT-NGT--EKVHLLVRFIFNLEEYLHFDSDLGMFVAL---------TEL 49
human DOB (62)              1:EDFVIQAKADCYFT-NGT--EKVQFVVRFIFNLEEYVRFDSDVGMFVAL---------TKL 49
Xenopus DMB (63)            1:SGFVVQEMIDCSFENNET----ATFHYSLTFNRMTVVAYDATRQKFTYCDPYPCIKQIYMV 57
anole lizard DMB (64)      1:GAFVLHLETDCFLSPSGR---ALWANWTMAFNQLPLVCYDNKGGHFLPCGLGEVNPW-FPV 57
chicken DMB (65)            1:CAFVVHMASSCPLLANGS---LGSFDLTMAFNKNPLLCYDPDVHRFYPCDWGLL----HTV 54
human DMB (66)              1:GGFVAHVESTCLLDDAGT---PKDFTYCISFNKDLLTCWDPEENKMAPCEFGVL----NSL 54
```

```
                                                       81HN82
salmon DAB (31)   high     51:GVKNAEAWNSD--------AAVLAVERGELERYCKHNADLHYST--ILDKT        91
trout DAB (32)             51:GLKNAKRLNRG---------QEVVQMRGELERLCEPNADVHYRA--ILDKT        90
Arctic char DAB (33)       51:GLKNAETWNKG---------SELARELGQLERFCKHNADIHYSA--VLDKT        90
D8.35 B2                   51:GLKLAEFWNNG---------TFVDQEKDVVEFFCKYNSQIYENS--ILDKA        90
D8.35 B3   high            51:GIRNAEYWNNG---------TFVEQERNEVERFCKHNAQIRDSA--VISKA        90
D8.37 B1                   51:GIRNAEFRNND--------TNLLLKKKAAVNKVCKNNAINHDKF--IRNKA        91
D8.37 B3   high            51:GLIFAENFNKD--------QAYLHQLKAQVDTFCRHNAQIWDSA--VRDKA        91
D4 B                       51:GVKFAENFNKN--------EAYLQQLRAEVDIFCRHNAEIYESA--VFDKA        91
zebrafish DAB1 (34)        51:GVIFARNFNKN--------QAYLQQRKAEVESFCRHNAQISDSA--VRDKA        91
zebrafish DAB2 (35)        51:GVKFAENFNKD--------QAILQQLKAAVDTFCRHNAEIFDSA--VRDKA        91
zebrafish DAB3 (36)        39:GVKSAENFNKN--------QAYLQQLKAGVDTFCRHNAQISDSA--VRDKA        79
zebrafish DAB4 (37)        51:GLIFAENFNKD--------QAYLHQLKAQVDTFCRHNAQIWDSA--VRDKA        91
zebrafish DEB (39)         51:GVYNAQLWKQR--------YQLLEQERAHEDRFCKYNAEIDYNN--ILGKT        91
zebrafish DFB (40)         51:GVKFAENFNKD--------QALLQQMKSEVDSICRHNAEIYESA--VFD--        89
carp DAB (41)              51:GVKYAENFNKD--------PSVLQDLKTSVDTYCRSNAQLADSS--VRDKA        91
G131 B1                    51:GVKNAAAWNNN--------PSYLSRAKAQKEVYCLNHVPVYYNN--MLTKS        91
G131 B2  X                 37:---------------------------------------------------        37
G131 B3                    51:GVKYAANWNKD--------ASYLSAMRAQKEVYCLNNVQIKYDN--ALTKS        91
GIII B                     51:GVRNAANWNKD--------ASELIAMRAQKEVYCLNSIQIKYNN--VLTKS        91
GVII B                     51:GMKNAAASNNN--------NMYMASIRAAKETICQPNIQRMYDN--ILTKS        91
stickleback DAB (42)       51:GVKIAANWNKD--------ASFLSAMKAQKEVYCLNHVPVYYTA--ALTKS        91
stickleback DBB (43)       51:GVRNAEYWNNN--------PSYLSAMKAQKEVYCLNHVPVYYSN--ALTKS        91
M3 B                       51:GVKTAELANND--------PEKMSRRRAEKETFCKPNIDNDYST--ILTKS        91
M615 B                     51:GVMAAGYWNKD--------LEGLRERRADKERLCKTNIDLDYLN--ILTKS        91
M873 B  high               51:GVKTAERANKD--------TSELSARKAQKETYCKHNIDNWYKN--MLSKS        91
medaka DAB (44)            51:GVKNAERFNKD--------TSELSVRRAQKETYCKHNIDIDYQT--ALSKS        91
F7533 B                    51:GLFQADYWNNQ--------SSILEGLRETKESICQPNIKIDYSN--ILSKS        91
F7721 B  X                 51:GLIQADYWNNI--------SSYLEQMRDEKERYCEPNIKVWYSN--ILSKS        91
F8524 B  X                 51:GLIQADYWNNI--------SSYLEQMRDEKERYCEPNIKVWYSN--ILSKS        91
T19 B                      51:GQYQADYWNNQ--------SSFLEAMRSSKQRLCQHNIPLWYSH--ILSKS        91
T61 B   X                  51:GLHQADYWNNQ--------SSYLDRLKEEKQRYCHRNIQNWYSH--ILSKS        91
O1 B                       51:GVRLAEVWNND--------TADLNLMRLKIATYCHHNIDINDRA--ILTKS        91
O18 B1                     51:GVKNAEEWNKD--------KGNLAAMNAQKEVYCQKKIDNRYNA--ILPKS        91
O18 B2                     51:GVYNADKWNND--------PAELNNRRAQKERYCHNNIGVDYQV--ALTKS        91
O18 B3  X                  51:GVKQAKHFNSQ--------PGRLATLKAEKERYCQHNIGIWYSN--VLPKS        91
```

```
O22 B1                  51:GVREADQWNKDQGQGSWTRKGQLAAMRAQKETYCKPKIDFWYQS--VLTKA     99
O22 B2     X            50:GVKEAEYFNND--------PGQLAAMRAQKETYCTPNIDLWYRS--VLTKS     90
O31 B1                  51:GENLAKHWNND--------TEELNSTRSKIVTYCLNNIDIHDRA--ILAKS     91
O31 B2                  49:GVMLAEILNNN--------TALLNSSRQMITEYCYHNIDVHDQA--IIAKS     89
O31 B3                  51:GVNLAKRWNNN--------TALLNSTRSKIATYCLSNIGIHDRA--IIAKS     91
O79 B1     X            42:GMKNAEMWNKD--------QGYLAAMRAQKETYCKHNIDLWYTN--VLSKS     82
O79 B2                  42:GVKNAEYWNKN--------QALLAQMRAQKETYCQPNIGIWYAD--VLSKS     82
O80 B                   51:GVKTAETWNNI--------PARLASMRAQKGTYCKSNIDVRYHK--LTSKS     91
O745 B                  51:GVKKAETWNND--------QAHMAAFRVEKERYCYTNIGIWYRN--ILSKS     91
O845 B                  51:GVREADQWNKDQGQGSWTRKGQLAAMRAQKETYCKPKIDFWYQS--VLTKA     99
OA35 B     X            50:GVKEADQWNND--------PGQLAAMRAQKEIYCTPNIDLWYRS--VLTK-     89
OA78 B     X            51:GVREADQWNKD--------QGQLAVMRAQKETYCTPNIDLWYRS--VLTKY     91
salmon DBB              51:GVISAEKWNED--------PDEIPRRRTDMGVLCKPYANRIYNA--TEMFM     91
GXVII B                 51:GLVSAAVFNGN--------HFDVLQRKEERRLICVDNVGHALNA--TEDNM     91
M5 B1                   51:GLITAAKFNAD--------KYDVVQRILERELVCQRSVEMVYNG--TEEAK     91
M5 B2                   24:GLITAAKFNAD--------KHDVVQRILERELVCQRSVEIIYET--TETAK     64
O97 B1     X            51:GLITASQFNED--------KFDVDQRKAEKQLTCVNNVDAVFNG--VQENI     91
O97 B2     X            51:GLITASRFNED--------SADILQRKVEKQLICVNNVDAVFNI--TVENM     91
salmon DCB              51:MKNFTETACKG--------PAFLADRREEMKKYCSSNVPVVYGY--LLDKA     91
M16 B                   49:AKDLADGFSKS--------KPFLEQAVKNREK-CRTHMDLVFE---LQSHP     87
O29 B1                  49:TKEIADNLNKN---------KGFLEHEKKNKEKCRTNIPLVLD---ILSRP     87
O29 B2     X            42:LKEVADELNKN---------ERFLEQEKKNEQECRTNIPLVFN---VLSRP     80
O29 B3     X            27:GIEFADKLNGD---------KDFMKHEVWKSNLCKRNVQMFYD---GL---     62
swordtail DXB (47)      51:AEAVAIILNNN---------PEFITHEIWKTNLCKRNTPLAQK----LLTP     88
O9 B                    52:GKVFADEVNKS--------PDYLKVRRNDLNYFCKVLGAVLYKD--IQVKA     92
O57 B                   51:TIEIAKFWNLN--------PFGTSEAKA----LCSTNLGYIQI---LSNVT     86
D18 B                   50:AMKWANDLNKQ--------PKWLHEQVEKNIADCKLFGETYFP---LVAKT     89
zebrafish DBB (48)      50:AMKWANDLNKQ--------PKWLHEQVEKNIADCKLFGETYFP---LVAKT     89
D8.45 B1                50:GQKFAEEYNKD---------KVLLAQHDFVLNQCRELGDVILPN--AVWLA     89
D8.45 B2                50:GEKFVENYKNN---------TFVLVLAEFGIDNCKKIAKALISDGMLNHVT     91
zebrafish DCB (49)      50:GEKFVENYKNN---------TFVLVLAEFGIYNCKKIAKALISDGMLNHVT     91
salmon DEB              50:GIRNADRYNSQ--------AWKMAIRKAEVETICRYSAIFFKLS--TLERI     90
fathead minnow DEB (50) 51:GIRNADHYNSQ--------GWKMKQRKEELETLCRANARLYVNS--T-RRK     90
spotted gar 501 B1      51:GEKQANYWNGQ--------KDYIARLSADKDRFCKYNVQLMASG--MQDRK     91
spotted gar 615 B       50:GIKYAERFNQD--------KEYLAGLKDDLDNYCKHNAGVYKST--MTDRK     90
paddlefish DAB01 (51)   51:GVKNAEVWNKD--------TAQLAGLLGDVDRYCKHNAELYMLF--TTDRK     91
paddlefish DAB02 (52)   51:GVKNAEVWNKD--------TAQLAGLLGDVDRYCKHNAELYMLF--TTDRK     91
paddlefish DAB03 (53)   51:GVKNAEEWNKD--------TARLAGLLGDVDRYCKRNAELYMLF--TTDRK     91
ray (54)                51:MKGNMDRWNK-----------EAAESTYQRGLSMCENNIPLYGRE--VLPRR     89
nurse shark (55)        49:MKSNVDRWNR-----------EGAEEQYESGKAYCEHNIPIVYES--ALARQ     87
coelacanth (56)         50:GKKDADYWNKD--------KENLAQWRIQEDRWCRNNYNWMQGW--AVGKQ     90
Xenopus DAB (57)        50:GKPSADYWNSQ--------KETLEQKRAAVDTVCRHNYPFDKPF--TIDRK     90
Chicken BLB (58)        50:GEPQAEYWNSN--------AELLENRMNEVDRFCRHNYGGVESF--TVQRS     90
human DQB (59)          50:GRPVAEYWNSQ--------KEVLEGARASVDRVCRHNYEVAYRG--ILQRR     90
human DRB (60)          50:GRPDAEYWNSQ--------KDLLEQRRAAVDTYCRHNYGVVESF--TVQRR     90
mouse DOB (61)          50:GEPDADQWNKR--------LDLLETSRAAVNMVCRQKYRLGAPF--TVERN     90
human DOB (62)          50:GQPDAEQWNSR--------LDLLERSRQAVDGVCRHNYRLGAPF--TVGRK     90
Xenopus DMB (63)        58:AAGIAKKLNNK---------PGIVTRMQQEKSKCQAQVKEFWEN--TMERR     97
anole DMA (64)          58:ASSISQWLDTN-----------APPQGPHAR-QACQNQTQPIWKR--TAERR     95
chicken DMB (65)        55:ATLLAAILNDD---------TTWVQRAEARRQACTELAAQFWTH--TALRR     94
human DMB (66)          55:ANVLSQHLNQK---------DTLMQRLRNGLQNCATHTQPFWGS--LTNRT     94
```

Text S4*C*: Alpha 2 domain sequence alignment

```
                                                              N125   NxS/T    NxS/T
salmon DAA (1)   high        1:DPPH-SSIYPRDDVDLGVENTLICHVSGFFPAPVRVRWTRNNQNLT-EGVRLSTPYPNAD 58
trout DAA (2)                1:DPPH-SSIYPRDDVELGVENTLICHVSGFFPSPVRVRWTRNDQNVT-EGGRISTPYPNTD 58
Arctic char DAA (3)          1:APPH-SSIYPRDDVELGVENTLICHVSGFHPAPVRVRWTRNNQNVT-EGVRTSTPYPNTD 58
D8.35 A1                     1:DPPQ-TSIYPKDDVELGVQNTLVCHATGFYPPSIRILWTMNNVNVT-EGISLSQYRPRVD 58
D8.35 A2    high             1:DAPQ-TSIYPKDDVELGVQNTLVCHVTGFYPPSVSVSWTKNNINVS-EDITLSQYRPRID 58
D8.37 A1                     1:DAPQ-TSIYSKGNVQLGIQNTLVCHVTGFYPPFVNISWTKNNVIVT-EGMSLSQYHPRVD 58
D8.37 A2                     1:DAPH-SSVYPKDDVELGVQNSLICHATGFYPPSITISWKKNNNNVT-ESINLSQYRPRAD 58
D8.37 A3    high             1:DPPV-TSIYSKDEVVLDKKNTLICHVTGFFPPPVNVSWTKNNDIVL-EEISFSQYRENSD 58
D8.37 A4    high             1:DPPV-TSIYSEDEVVLDEKNTLICYVTGFFPPPVNVSWTKNNDIVT-EDISFSQYRDNSD 58
D4 A                         1:EPPE-TSIYPRNEVLLGVDNVLICHVTGFFPPPVDVSWSRNNVKVT-EGVDVSQYRPRSD 58
zebrafish (4)                1:DPPV-TSIYSEDEVVLDERNTLICHVTGFFPPPVNVSWTKNNDIVT-EEISFSQYRRNSD 58
zebrafish (5)                1:DPPQ-TSIYSRDDVQPDIENKLICHVTGFFPPPVRVSWTKNNEIVT-EGMSVSQYRPNND 58
carp DXA (6)                 1:APPD-ASVYSEGDVVLGVQNTLICHVTGLFPPPVNVSWTKNNQIVT-EDVSLSQYRRKND 58
Atlantic herring DAA (7)     1:APPM-SSIYPRHEVKVGTVNTLICYIAGFYPPRLTVRWTRNNKNVTQGVSSSQLRLNVND 59
G129 A   X                   1:----SHMIYRKHGVELGEKN---CHVTGFFPAPVTFSWTKSQENVT-EGTCKNVPFLNKD 52
G131 A1   high               1:DPPSSHMIYPKDGVELGEKNSLICHVTGFYPAPVTFSWTKNQDNVT-EGSSRNVPYLNND 59
G131 A2                      1:DPPSSHMIYPKDGVELGEKNSLICHVTGFYPAPVTFSWTKNQENVT-EESSRNVPYLNND 59
G131 A3                      1:DPPSSHMIYPKDGVELGEKNSLICHVTGFYPAPVTFSWTKNQENVT-EGSSRNVPYPNND 59
GVII A                       1:DPPSSHMIFPKDAVELGEKNSLICHVTGFYPAPVAFSWTKNQENAT-EGTSRNIPFPNND 59
stickleback DAA (8)          1:DPPSSHMIYPKDGVELGEKNSLICHVTGFYPAPVTFSWTKNQENVN-EGSSRNVPFPNND 59
stickleback DBA (9)          1:DPPSSHMIYPKDGVELGEKNSLICHVTGFYPAPVTFSWTKNQENVT-EGSSRNVPYLNND 59
M3 A                         1:DPPSNVMVYSRDEVELGQQNTLICHVSGFYPAPVNVSWTRNGEKVP---GSINVPLPSSD 57
M615 A                       1:DPPSNVVVYSRNKVELGQQNTLICHVSGFYPAPVNVSWTRNGEKVS---GSINVPRPSSD 57
M873 A   high                1:DPPSNVVVYSRDEVELGEQNTLICHVSGFYPAPVNVSWTKNGERVS---GSINIPFPSSD 54
F402 A                       1:VAPTSPVIYTKEEVQLSQQNTLICFVTGFYPAPVNVSWTRNGEHVT-QGTSINVPYPNKE 59
F7533 A                      1:VAPTSPVIYTKEEVQLSQQNTLICFVTGFYPAPVNVSWTRNGEHVT-QGTSINVPYPNKE 59
F7721 A                      1:VAPTSPVIYTKEEVQLSQQNTLVCFVTGFYPAPVNVSWTRNGEHVT-QGTSINVPYPNKE 59
T19 A                        1:DPPTSPMVYTRNEVQLTEPNTLVCLVTGFYPAPVNVSWTRNGEEVT-QGTSINVPYPNNE 59
T55 A                        1:VPPTSPMVYTRSEVQLTEPNTLVCLVTGFYPAPVNVSWTRNGEEVT-QGTSINVPYPNNE 59
T61 A                        1:VPPTSPMVYPSSEVQLTEPNTLVCLVTGFYPAPVNVSWTRNGE-VT-QGTSINVPYPNNE 58
O1 A                         1:DAPSGVMIYTRNEVELRVTNTLICHVTGFYPGPVKVSWTKNGQKLT-EGFSINVPYPNKD 59
O18 A1                       1:DAPSAVMIYTRDEVEFGEKNTLICHVTGFYPAPVNVSWTKNEQKVT-ERSSINVPYPNKD 59
O18 A2                       1:DAPSGVMIYTRDEVEFGEENTLICHVTGFYPAPVNVSWTKNEQKVT-QGSSINVPYPNKD 59
O18 A3                       1:DAPSAVMIYTRDEVEFGEKNTLICHVTGFYPAPVNVSWTKNEQKVT-QGSTINVPYPNKD 59
O22 A1                       1:DAPSSVNIYTTLNVEIGVQNTLICHVTGFYPAPVKVTWKKNIKKVV-EGTSITVPLSNKD 59
O31 A1   X                   1:-----VMIYTRNEVELRVKNTLICHVTGFYPAPVKVSWTKNGQITT-GGSSINTPYPNKD 54
O31 A2   X                   1:-APSCVMIYTRNEVEFTVKNTLICHVTGFYPAPVKVSWTKNGRITT-GGSSINNPYPNKN 58
O31 A3                       1:DAPSGVMIYTRDEVELTVKNTLICHVTGFYPAPVKVSWTKNEQITT-EGSSINTPYPNKD 59
O31 A4                       1:DAPSGVMIYTRDEMELRVNNTLICHVTGFYPAPVKVSWTKNGRITT-EGSSINTPYPNKN 59
O39 A   X                    1:HAPSAVMIYTRDEVEFGEENTLICHVTGFYPAPVNVSWTKNGQKVT-EGSSTNVPYPNKD 59
O42 A                        1:HAPSAVMIYTRDEVEFGEENTLICHVTGFYPAPVNVSWTKNGQKVT-EGSSTNVPYPNKN 59
O79 A1                       1:DPPSSLIIYTVLNVELGVQNTLICHVTGFYPAPVNVTWTKNEQKVT-EGTNTSVPFPNKD 59
O79 A2   X                   1:DPPSSLIIYTVLNVELGVQNFLICHVTGFYPAPVNVTWTKNEQKVT-EGTNTSVPFPNKD 59
O79 A3                       1:DPPSSLIIYTVLNVELGVPNILICHVTGFYPAPVNVTWTKNEQ-VT-EGTSTSVPFPNKD 58
O79 A4   X                   1:DPPSCLVIYTLLHVELGVQNTLICHVTGFYPAPVNITWNKNEQKVT-EGTSTSILFPNKD 59
O745 A                       1:DVPSAVMIYTRDEVEFGEKNTLICHVTGFYPAPVNVSWTKNQQKVT-QGSSINVPYLNKD 59
O845 A                       1:EAPSSVAIYTTLNVEIGVQNTLICHVTGFYPAPVNVTWNKNTKKVV-EGTSITVPLSNKD 59
OA35 A   X                   1:-------------VEIGVQNTLICHVTGFYPAPVNVTWNKNTKKVV-EGTSITVPLSNKD 46
OA78 A                       1:APDS-VEIYTIVNVEIGVQNTLICHVTGFYPAPVNVTWNKNTKKVV-EGTSITVPLSNKD 58
tilapia DBA (10)             1:IAPSAVMIYTRDEVEFGVQNILICHVTGFYPAPVNVSWTKNGQKVT-EGSSINVPYINKD 59
A.hansbaenschi DCA (11)      1:DAPSGVTIYTRNEVELRAKNTLVCHVTGFYPAPVKVSWTKNGQITT-EGSSINNPYPNKN 59
----------------------------------------------------------------------------------------------
salmon DBA                   1:DAPE-STIYPRDEVELGVENTLICFVNDFFPPPVKVYWTKNEMEVT-EGLSLSRYYPNKD 58
GXVII A                      1:DAPV-LSVYSRHEGRGGAANTLFCLADGFYPPSVNFTWTKNGARVT-GGVWDLPYGHNRD 58
M5 A                         1:EPPD-LSIYTRYKAEEGVLDTLFCSANHFYPPTINFTWTKNGAEVT-EGLLNLRFSHNKD 58
O97 A                        1:DPPD-IYIYTRYEGEASVMNTLFCVANHFYPPTINFTWTKNGVELT-EGVANLRYRHNRD 58
salmon DCA                   1:DAPE-STIYPRAEEELGVENTLICFANHFYPPPVKVNWTKNGLEVT-EGTSLSRYYPNED 58
M16 A                        1:DPPGEVLIYTSAEVQPGVENTIICFVNGFYPPSIKVSWTKNGNPVS-EGVSHSRYYPNKD 59
O29 A1   X                   1:DPPE-SILCLAEEVELGVENSLICFVNHFYPPSINFS*TKNGHPVS-ERVSFSRYVPNKD 57
O29 A2   X                   1:DPPE-SMLYPADEVELGVENSLICFVNYFYPPSINVSWTKNGHPVS-EGVSLSRYFPNKD 58
O29 A3                       1:DPPE-IVLFSSDKVELGVENSLICLVNHFYPPSINVTWTKNGHPVS-TGVSLSRYFPNKD 58
```

```
tilapia DAA (12)            1:---------------------------------------KNGHPVS-TGVSLSRYFPNKD 20
O9 A                        1:KAPA-VVIYPMDEAETGKNNTIYCHIRHFYPPSNNVTWTRNGIRVT-EGVNLSNPYPQTD 58
O33 A                       1:EPPE-FIIYPRDEVITGEDNTLICFINHFFPPSINIKWTKNDVEVT-VEDPFIKTLSNPD 58
O57 A                       1:APPE-IIIYPRDEVMKEEDNSLICLIKKFFPPSIKIKWTKNDIEVK-VEDPFIKCLPNPD 58
D8.45 A1                    1:EPPW-TTVYSRNDVKLNVKNTLICHVTGFFPPPVRVLWTKNNVNVT-DGSTISRYYPNKD 58
D8.45 A2                    1:EPPW-VLLYPRNDVKLNLKNTLICHVTGFFPPPVRVLWTKNNVNVT-DGSTISRYYPDND 58
zebrafish DCA (13)          1:EPPW-VLLYPRNDVKLNLKNTLICHVTGFFPPPVRVLWTKNNVNVT-DGSTISRYYPDND 58
D8.46 A                     1:EPPW-SSIYPKSDPQLNLKNTLICHVTGFFPPPVRVLWTKNNVNVT-DGSTISRYYPNKD 58
D18 A   X                   1:EPPL-SSIYPKSDPQLNVKNTLICQVTGFFPPPVRVLWTKNNVNVT-DGSTISRYYPNKD 58
salmon DDA                  1:VPPE-IKLYAKDEVKLGINNSLVCFVNNFFPPPVQVKWTKNDENVP-KGVKVGQYATNSD 58
spotted gar 501 A2          1:DAPV-NTIYPRDEVELGKPNTLICLANNFFPPPVKVRWTKNDVDVS-EQATLSRYYPNSD 58
salmon DEA                  1:EVPT-SHIYSQREVELGVPNTLICRVSDFHPTPVDVTWTRNEQPVAERTIIQTQYYSNED 59
seabream DEA (14)           1:ASPT-SLIYSKQEMELGVPNTLICFVSDFHPPEVQITWTRNGQPVDQSDVSQTQYYSNSD 59
carp DEA (15)               1:----------------------------FHPPPVNISWMRNGEPVSEQDVSETQYYSNPD 32
loach DEA (16)              1:----------------------------FHPPPVNISWTRNGEAVSELDVYETQYYSNPD 32
spotted gar 501 A3   X      1:DAPT-AMVYPRDSVELGKPNTLICSVTDFHPMGIGVTWTHNDRPVT-EGVTQTTPLSGRD 58
spotted gar 615 A           1:DPPI-SRLYSENEVELGVPNALICFITDFHPAPVKVSWTRNTEPVT-QGFNVTQYYSNKD 58
sturgeon (17)               1:VSPR-VTLYPEKDLELGKPNTLICFITDFHPAAIKVTWTKNTLPVT-EGVSLTQYYSNKD 58
spotted gar 501 A1          1:VPPK-PAVFPEEPVELGWPNTLICALNDFTPPTAQLRWLKNGQPVT-SDVSSTDYIPLSS 58
little skate (18)           1:VPPQ-TAMYPEDPVEWGESNTLICLADGFYPPRINMKWKRNNEPVT-EGIDTTEYYVKRD 58
nurse shark (19)            1:VPPE-VSVYSEDLVEWGQLNTLICFADGFYPPHITMKWRRNNEPMT-DGDNITEFYIKDD 58
coelacanth (20)             1:VPPE-VTMYPENQVEFGKANILICFMDNFYPPVLNFTWYKNGNQVS-EGFYNTDFYSKVN 58
Xenopus DAA (21)            1:IPPL-ITLYSAKPVVQGEPNILICCVNNIFPPVMNTTWFKNGQKIS-DGFSETSFLPAKD 58
chicken BLA (22)            1:VTPE-LALFPAEAVSLEEPNVLICYADKFWPPVATMEWRRNGAVVS-EGVYDSVYYGRPD 58
human DQA (23)              1:EVPE-VTVFSKSPVTLGQPNTLICLVDNIFPPVVNITWLSNGQSVT-EGVSETSFLSKSD 58
human DRA (24)              1:VPPE-VTVLTNSPVELREPNVLICFIDKFTPPVVNVTWLRNGKPVT-TGVSETVFLPRED 58
mouse DOA (25)              1:VPPR-VTVLPKTRVELGKPNVLICIVDDIFPPVINVTWLRNSQPIT-KGVAQTSFYSQPN
human DOA (26)              1:VPPR-VTVLPKSRVELGQPNILICIVDNIFPPVINITWLRNGQTVT-EGVAQTSFYSQPD 58
Xenopus DMA (27)            1:GILD-IKVFTLHPLTIGKPNTLVCFISNIIPPDLNITWRKNGILLT-EGISYTGYFALSN 58
anole lizard DMA (28)       1:GAPQ-IDVFTLRPLEMGKPNTLVCAASNVFPPTISLHWELDGQPVSSRVSTPSQVSPVQG 59
chicken DMA (29)            1:GIPV-ADVFLQQPLQLGYPNTLICMVGNIFPPAITISWQRDGIPVT-DGVTHLTYTPTED 58
human DMA (30)              1:GFPI-AEVFTLKPLEFGKPNTLVCFVSNLFPPMLTVNWQHHSVPV--EGFGPTFVSAVDG 57


salmon DAA (1)   high      59:FTLNQFSSLPFTPEEGDIYGCTVEHKG-L-AEPLTRIW                      94
trout DAA (2)              59:VTFNQFSSLSFTPEEGDIYGCTVEHKA-L-TEPLTRIW                      94
Arctic char DAA (3)        59:FTLNQFSSLTFTPEEEDIYGCTVEHKGHI-HEPLTRIW                      95
D8.35 A1                   59:GTFNIFSTFRFTPAEGDIYSCRVIHEA-LLGQPQAKIY                      95
D8.35 A2   high            59:GTFNIFSTLKFTPAEGDIYSCMVKHRA-IKDQPQTKTW                      95
D8.37 A1                   59:GTFNIFSTLKYTPAEGDIYSCTVNHRT-LQGQPQTRIW                      95
D8.37 A2                   59:GSFNIFSTLKFTPAEGDIYSCTVNHEA-LQGQAQTKTW                      95
D8.37 A3   high            59:GTFNMFSALKFTPAEGDIYSCTVKHRS-IQGQTNTKTW                      95
D8.37 A4   high            59:GTFNMFSALKFTPAEGDIYSCTVKHRS-IQGQPNTKTW                      95
D4 A                       59:QTFHMFSSLKIRPAAGDIYGCTVNHAA-LQEQPQTKLW                      95
zebrafish (4)              59:GTFNMFSALKFTPAEGDIYSCTVNHRS-IQGQPNTKTW                      95
zebrafish (5)              59:GTYNIFSTLRFTPVEGDIYSCSVNHKT-L-EQPQTKAW                      94
carp DXA (6)               59:GTFNIFSSLKFTPAEGDIYSCTVYHKA-LESRFITKTW                      95
Atlantic herring DAA (7)   60:LSFNQFFTLNFTPQEGDMYTCTVEHQA-L-EGPMTREF                      95
G129 A   X                 53:RTFNQFSTLEFTPKLGDIYSSRVEHLA-L-DHPLVKFY                      88
G131 A1   high             60:GTFNQFSTLEFTPKLGDIYSCMVEHLA-L-DHPLVKFY                      95
G131 A2                    60:GTFNQFSTLKFTPKLGDIYSCMVEHLA-L-DHPLVKFY                      95
G131 A3                    60:GTFNQFSTLEFTPKLGDIYSCMVEHLA-L-DHPLVKCV                      95
GVII A                     60:GTFNQFSTLEFTPELGDIYSCMVEHLA-L-DHPLVRFY                      95
stickleback DAA (8)        60:GTFNQFSTLEFTPKLGDIYSCMVEHLA-L-DHPLVKFY                      95
stickleback DBA (9)        60:GTFNQFSTLEFTPKLGDIYSCMVEHLA-L-DHPLVKFY                      95
M3 A                       58:GTLTQISRLQFVPQLGDIYSCSVEHPA-L-PEVQTKIW                      93
M615 A                     58:GTFSQISRLQFVPQLGDIYSCSVEHPA-L-PEVQTMIW                      93
M873 A   high              55:GTFTQISRLPFVPQLGDIYSCSVEHPA-L-TEVQTKIW                      80
F402 A                     60:GTFTQISRLAFVPQQGDIYSCRVQHPA-L-SGQDTRMW                      95
F7533 A                    60:GTFTQISRLAFVPQQGDIYSCRVQHPA-L-SGLDTRMW                      95
F7721 A                    60:GTFTQISRLAFVPQQGDIYSCRVQHPA-L-SGQDTRMW                      95
T19 A                      60:GTFTQISRLDFVPQQGDIYSCRVQHPA-L-SEADTRMW                      95
T55 A                      60:GTFTQISRLDFVPQQGDIYSCRVQHPA-L-SEADTRMW                      95
```

```
T61 A                        59:GTFTQISRLDFVPQQGDIYSCRVQHPA-L-SEADARMW     94
O1 A                         60:GTFKQIARLQFIPQQGDIYSCTVEHLA-L-KEPLTKIY     95
O18 A1                       60:GTFTQISRLQFTPQQGDIYSCTVQHLA-L-TQPATKIY     95
O18 A2                       60:GTFTQISRLEFTPQLGDIYSCKVQHLA-L-TQPLTKIY     95
O18 A3                       60:GTFTQISRLQFTPQQGDIYSCAVQHLA-L-TQPLTKIY     95
O22 A1                       60:GSFSQTSKLDFVPKGGDVYACIVEHMA-L-TQPITKVY     95
O31 A1     X                 55:GTFTQIARLQFIPQQGDVYSCTVEHLA-L-TKPLTKIY     90
O31 A2     X                 59:GTFTQIARLKFILH------CTVEHLA-L-TDPLTKT-     87
O31 A3                       60:GTFTQIARLKFIPQQGDVYSCTVEHLA-L-TKPLTKIY     95
O31 A4                       60:GTFTQIARLKFIPQQGDVYSCTVEHLA-L-TKPLIKIY     95
O39 A    X                   59:--------------------------------------     59
O42 A                        60:GPFTQISRLQFTAQLGDIYSCTVQHLA-L-TEPLTKIY     95
O79 A1                       60:GSFKQTSRLQFIPKQGDVYSCTVEHVA-L-IQPMTKFY     95
O79 A2     X                 60:GSFKQTSRLDFIPKQGDVYSCTVEHVA-L-IQPMTKFY     95
O79 A3                       59:GSFKQTSRLNFIPKQGDVYSCTVEHVA-L-TQPMTKFY     94
O79 A4     X                 60:GSFKQTSRLDLIPKKRDVYSCTVEHVG-L-TQPTTKFD     95
O745 A                       60:GTFTQISRLQFTPQLGDIYSCAVQHLA-L-TQPLTKIY     95
O845 A                       60:GTFTQTSKLDFVPKGGDVYACIVEHVA-L-TQPLTKVY     95
OA35 8     X                 47:GTFSQTSKLDFVPKGGDVYVCIVEHVS-L-TAPLTKIY     82
OA78 A     X                 59:GTFSQTSKLDFVPKGGDVYVCIVEHVS-L-TAPLTKIY     94
tilapia DAB (10)             60:GSFKQTSRLDFTPQLGDMYSCTVEHVS-L-TEPLTKIY     95
A.hansbaenschi DCA (11)      60:GTFTQIARLQFIPQQGDVYSCTVEHLG-L-TKPLIKIY     95
------------------------------------------------------------------------------
salmon DBA                   59:GTFHQFSSLSFTPQKEDVYICAVAHTA-L-KEPKTREY     94
GXVII A                      59:GTFHRISTLSTTPREGDVYSCSVEHRA-A-RRPLASSW     94
M5 A                         59:GTFRGISTLSFTPQRGDVYSCWVSHEA-L-ERPRIITW     94
O97 A                        59:GTFHMISTLLFTPQPGDHYICTVEHQA-A-KVPVSKSW     94
salmon DCA                   59:GTFHQFSSLSFTPQEGDVYGCTVKHTA-L-EDPKTRFW     94
M16 A                        60:QTFHQFSTLSFTPSWTDVYSCTVEHPA-L-ESPKTVLW     95
O29 A1     X                 58:QIFHQFSTLTFSLSEGDIYSCTAEH-------------     82
O29 A2     X                 59:QTFHQFSTLVFTPSEGDIYSCTV---------------     81
O29 A3                       59:QTFHQLSTLTFTPSEGDFYSCTVEHSA-L-ETPQTRIW     94
tilapia DAA (12)             21:QTFHQLSTLTFTPSEGDFYSCTVEHSA-L-ETPQTRIW     56
O9 A                         59:GTFNQLASFSFQPQSDDVVECSVQHQA-L-SRPLITTW     94
O33 A                        59:GTAYVLSYLNFVPEKGDIYSCTVEHEA-L-KEPQTRFW     94
O57 A                        59:GTFYVFSHLDFVPEDGDIYSCSVEHEA-L-TEPLTKLW     94
D8.45 A1                     59:GTMNVFSRLSFIPEEGDVYGCSVEHKA-L-QQPQTRTW     94
D8.45 A2                     59:RNFNVFSQLSFIPEEGDVYSCSVEHKA-L-QQPQTRTW     94
zebrafish DCA (13)           59:RNFNVFSQLSFIPEEGDVYSCSVEHKA-L-QQPQTRTW     94
D8.46 A                      59:GTMNVFSRLSFIPEEGDIYSCSVEHKA-L-QQPQTRTW     94
D18 A    X                   59:GTMNVFS*LSFIPEEGDIHSCLVEHKA-L-QQPQTRTW     93
salmon DDA                   59:YTFYRFSTLTFEPQEGDIYTCIVDHTA-L-DEPLTRTW     94
------------------------------------------------------------------------------
spotted gar 501 A2           59:ATFYQFSTLSFTPQLGDVYSCSVEHKA-L-PEPKTRIW     94
------------------------------------------------------------------------------
salmon DEA                   60:FSFRIFSYLSITPQEGDIYSCSVGHVS-L-QEPLTRIW     95
seabream DEA (14)            60:FSFRLSSYLDFTPQQGDIYSCSVGHIS-L-RAPLNRFW     95
carp DEA (15)                33:FSLRLFSYLNFRPERGDIYTCSVRHRG-L-EQDITRFW     68
loach DEA (16)               33:FSFRIFSYLDFSPEQGDIYTCTVRHRS-L-EQGITRFW     68
------------------------------------------------------------------------------
spotted gar 501 A3   X       59:FSFKVFSFLPFTPRLGDVYSCQVQHSA-L-PEPLARLW     94
spotted gar 615 A            59:YSLRLFSYLSFTPQAGDVYSCSVEHRA-L-QEPLTRLW     94
sturgeon (17)                59:FTLQMFSYLSFTPELGDVYSCQVQHSA-L-PETLTTFW     94
------------------------------------------------------------------------------
spotted gar 501 A1           59:NKFAMFSYLSFTPQEGDIYTCHVEHTA-L-SEPVSVFW     94
------------------------------------------------------------------------------
little skate (18)            59:YTFQRFTYLNFIPSPGDMYSCHVEHEG-L-ERPTTVFW     94
nurse shark (19)             59:FTYRRFSYLSIVPSPGDMYSCHVEHSS-L-QDPVTVFW     94
------------------------------------------------------------------------------
coelacanth (20)              59:YRFRKFSYLNFTPEVGDIYSCQVEHWG-L-EEPINKFW     94
Xenopus DAA (21)             59:YSFGRLHYLAFLPNEHDIYTCEVEHWG-L-DRPTRRVW     94
chicken BLA (22)             59:LLFRKFSYLPFVPQRGDVYSCAVRHWG-A-EGPVQRMW     94
human DQA (23)               59:HSFFKISYLTFLPSADEIYDCKVEHWG-L-DQPLLKHW     94
human DRA (24)               59:HLFRKFHYLPFLPSTEDVYDCRVEHWG-L-DEPLLKHW     94
------------------------------------------------------------------------------
mouse DOA (25)               59:HRFRKFHYLTFVPSAEDVYDCKVEHWG-L-DTPLLQHW     94
human DOA (26)               59:HLFRKFHYLPFVPSAEDVYDCQVEHWG-L-DAPLLRHW     94
------------------------------------------------------------------------------
Xenopus DMA (27)             59:IEYQTFSYLNITPTYTDSYTCNAQ-EG-S-T-TAVAYW     92
anole lizard DMA (28)        60:WVFQAFSYLEITPQEGQVYSCTARSSS-D-PFSSVAFW     95
chicken DMA (29)             59:LGFMRFSYLAVTPHSGDIYACIVTRER-D-NISVVAYW     94
human DMA (30)               58:LSFQAFSYLNFTPEPSDIFSCIVTHEI-D-RYTAIAYW     93
```

Text S4*D*: Beta 2 domain sequence alignment

```
                                                                                S144
salmon DAB (31)   high     1:VEPHVRLSSVAPPSGRHPAMLMCSAYDFYPKPIRVTWLRDGREVK-SDVTS-TEELANGD 58
trout DAB (32)             1:VEPHVRLSSVTPPSGRHPAMLMCSAYDFYPKPIRVTWLRDGREVK-SDVTS-TEELANGD 58
Arctic char (33)           1:VEPHVRLSSVTPPSGRHPAMLMCSAYDFYPKPIRVTWLRDGHEVK-SDVTS-TEELANGD 58
D8.35 B1   X               1:---------------------MCSAYDFYPPHINVFWLRNGEVMT-SEVTS-TMEMADGD 37
D8.35 B2                   1:VKPKVKLSSVTRAGGRQSTVLMCSAYDFYPPHINVFWLRNGEVMT-SEVTS-TMEMADGD 58
D8.35 B3   high            1:VKPKVKLSSVTRAGGRHPAVLMCSAYDFYPPHIKISWLRDGQVVT-SDVTS-TEEMADGD 58
D8.37 B1                   1:VKPKVKLTLVKPAGGRHPAVLMCSAYEFYPPHIRVYWLRNGEVIT-SDVTS-TMEMADGD 58
D8.37 B2   X               1:-KPTVVLSSVTQANGRHPAMLICSAYEFYPRHIKVSWLKGGKSVT-SEVTS-TMEMADGD 39
D8.37 B3   high            1:VLPEVTIKSVRQAEGRHPAVLLCSAYEFYPKKIKMSWLRDGKEVT-SDVTS-TMEMADGD 58
D4 B                       1:VMPKVNLSLVQKGDSRHPYLLRCSAYDFYPQQIKMSWLRDGRVV--SDVTS-SEQMPNGD 57
zebrafish DAB1 (34)        1:VKPKVTIQSVMQAEGKHPAMLLCDAYEFYPKKIKMSWLRDDKVVT-SDVTS-TIEMANGN 58
zebrafish DAB2 (35)        1:VKPKVTMKSVKQAEGRHPAMLLCSAYEFYPKKIKMYWLRDSKVIT-SDVTS-TMEMANGN 58
zebrafish DAB3 (36)        1:VLPEVTIKSVRQAEGKHPAVLLCSAYEFYPKKIKMSWLRDGKEVT-SDVTS-TMEMADGD 58
zebrafish DAB4 (37)        1:VLPEVTIKSVRQAEGRHPAVLLCSAYEFYPKKIKMSWLRDGKEVT-SDVTS-TMEMADGD 58
zebrafish DDB (38)         1:-KPTVVLSSVTQANGRHPAMLICSAYEFYPRHIKVSWLKGGKAVT-SEVTS-TMEMADGD 57
zebrafish DEB (39)         1:VKPQVKLNSVKQAGGRQPAVLVCSAYDFYPKRIKVTWLRNGKPVT-TDVTS-TEELADGD 58
carp DAB (41)              1:VQPKITLRSARQAGGSRPAVLMCSAYEFYPKKIKVSWLRDGKEMT-SDVTS-TMEMANGN 58
G129 B   X                 1:-EPYVRLHSETPPGGGPLSMLVCSVYDFYPKKIIVRWTRDGRPET-TGVTS-TDELAGG- 56
G131 B1                    1:AEPYVRLHSETPPGGGPLSMLVCSVYDFYPKKIIVRWTRDGRPET-TGVTS-TDELADGD 58
G131 B2   X                1:AEPYVRLHSETPPGGGPLSMLVCSVYDFYPKKIIVRWTRDGRPET-TGVTS-TDELADGD 58
G131 B3                    1:AEPYVRLHSETPPGGGPLSMLVCSVYDFYPKKIIVRWTRDGRPET-TGVTS-TDELADGD 58
GIII B                     1:AEPYVRLHSETPPGGGPLSMLVCSVYDFYPKKIIVRWTRDGRPET-TGVTS-TDELADGD 58
GVII B                     1:AQPYVRLHSETPLGGRTSSMLVCSVYDFYPQNIIVRWTRDGRPEA-TGVSS-TDELADGD 58
stickleback DAB (42)       1:AEPYVRLHSETPPGGGPLSMLVCSVYDFYPKKIIVRWTRDGRPET-TGVTS-TDELADGD 58
stickleback DBB (43)       1:AEPYVRLHSETPPGGGPLSMLVCSVYDFYPKKIIVRWTRDGRPET-TGVTS-TDELADGD 58
M3 B                       1:VQPRVRVQSLEPSGGNHPAMLICSVYDFYPKKIKVSWLQDQEEVS-SDVTS-TAEMEDGD 58
M615 B                     1:VKPSVMIESVTSSGGHHPAMLVCSVYDFYPKYIKVSWLRDQEEVS-SDVTS-TTELEDGD 58
M873 B   high              1:VQPRVRVQSLAPSGGHHPAMLVCSVYDFYPKTIRVSWLRGKEEVS-SDVTS-TAEMEDGD 58
medaka DAB (44)            1:VQPRVRVQSLAPSGGHHPAMLVCSVYDFYPKTIRVSWLRGKEEVS-SDVTS-TAEMEDGD 58
medaka DBB (45)            1:-QPSVMIESVTPSGGHHPAMLVCSVYDFYPKTIRVSWLRGKEEVS-SDVTS-TAEMEDGD 57
medaka DCB (46)            1:-QPRVRVHSLEPSGGNHPAMLICSVYDFYPKKIKVSWLQDQEEVS-SDVTS-TA------ 51
F7533 B                    1:VEPTVRVHSVVPPAGGHPAMLVCSVYDFYPRYIKVSWQRDGEEVS-QDVTS-TDELADGD 58
F8524 B   X                1:VEPTVRVHSVVPPAGGHPAMLVCSVYDFYPRYIKVSWQRDGEEVS-QDVTS-TDELADGD 58
T19 B                      1:VLKDVFVYSVAPPAGGHPAMLVCSVYDFYPKKIKVSWRRDGQEVS-HDVTS-TDELADGD 58
T48 B   X                  1:-LKDVLVYSVAPPAGGHAAMLVCSVYDFYPKKIKVSGRRDGQEVS-HDVTS-TDELADGD 57
T61 B   X                  1:-LREVVLHSVAPPAGGHAA-------------------------S-HDVTS-TDELADGD 14
T70 B   X                  1:IAPTVRVHLVAPPAGGHAAMLVCSVYDFYPKKIKVSWRRDGQEVS-HDVTS-TDELADGD 58
O1 B1                      1:VQPSVRIKSMTPPSGQHPAMLVCSVYDFFPSKIKVSWLRDGQAVS-SDITS-TEEMPNGD 58
O18 B1                     1:AKPYVKLHSTMPLSSHHPAMLVCSVYDFFPSEIKVSWQRDGQEVT-SDVTS-TEEMADGD 58
O18 B2                     1:AKPYVKLHSMTPASSHHPAMLVCSVYDFFPSKIKVSWLRDGQEVS-SDVTS-TEEMADGD 58
O18 B3   X                 1:---------------------------------------------------------EEADGD 7
O22 B1                     1:VKPSVRLHSIPPVY-LNPAILVCSVYNFYPKEIKVSWLRNGQQVS-PDITS-TVEFANAD 57
O31 B1                     1:VQPRVRIKSKTPLSGQHPAMLVCSVYDFFPTKIKVSWLRDGQEVY-SDVTS-TEVMPNGD 58
O31 B2                     1:VRPRVRIKSKTPLSGQHPAMLVCSVYDFFPNKIKVRWLRDEQEVT-SDVTS-TEVMPNGD 58
O31 B3                     1:VRPSVRIKSKTPLSGQHPAMLVCSVYNFFPRKIKVSWLRDGQKVT-SDVTS-TQEMPNGD 58
O49 B   X                  1:-KPYVRLHSTTPAYSQHPAMLVCSVYDFFPKYIKVSWLRDGQEVT-SDVTS-TEEMADGD 39
O50 B   X                  1:-------------------------YQNIPSKIKVSWHRDGQEVT-SDVTS-TEEMEDGD 36
O77 B   X                  1:----VKLHSTTPLSSHHPAMLVCSVFDFFPSKIKVSWHRDGQEVT-SDVTS-TEEMEDGD 54
O79 B1   X                 1:STPSVRLHSTMAPGCHHPAMLVCSVYDFYPKLIKVSWLRNGQEIT-SDITS-TEEFANGD 58
O79 B2                     1:VTPSVRLHSTMAPGCHHPAMLVCSVYDFYPKLIKVSWLRNGQEIT-SDITS-TEEFANGD 58
O80 B                      1:AQPTVKLHSTTPLSSHHPAMLVCSVYDFFPSKIKVSWHRDGQEVT-SDVTS-TEEMEDGD 58
O745 B                     1:AKPYVKLHSTTPDYSHHPAMLVCSVYDFFPSKIKVSWLRDGQEVT-SDVTS-TEEMADGD 58
O779 B   X                 1:-------------------------YDFYPSEIKVSWHTDAQEVT-SDITS-TEEMTDGD 33
O845 B                     1:VKPSVRLHSIPPVY-LNPAILVCSVYNFYPKEIKVSWLRNGQQVS-PDITS-TVEFANAD 57
OA78 B   X                 1:VKPFVRLHWTKPPVDHHPAILACSIYDFYPKYIKVTWLRNGQRVT-SNTTT-TEELPDAD 58
--------------------------------------------------------------------------------------------
salmon DBB                 1:VEPNVTLRLEGPSS---DSSLVCSVHFFYPKHIRVTWLRNGEEVT-SDVTS-TDVLANGL 55
GXVII B                    1:AAPSVRLAEA--SGSGHNTTLVCSAYDFYPRRIRLAWLRDGQEVT-SGATF-SEVTTNGN 56
M5 B1                      1:VEPNVSLQTVE----DNDSTLECSALDFYPKHIRLTWFSNGQEVT-EGVTF-SDVLPNGD 54
M5 B2                      1:VEPNVSLQTVE----DNDSTLECSALDFYPKHIRLTWFSNGQEVT-EGVTF-SDVLPNGD 54
O97 B1   X                 1:-APSMTLQEGD-HTSTHDTILVCSVYDFYPKHILVTWYLNGQEV---------------- 42
```

```
O97 B2   X                 1:AAPSMTLQEGD-HTSSHDTILVCSVYDFYPKHILVTWYLNGQEVT-E------------- 45
salmon DCB                 1:VEPYISLRSVEPFSTRHPAMLVCSAYDFYSKPIRVMWLKNGQEVT-SNVTS-TEELVNGD 58
M16 B                      1:VEPSVRVTPVVRQGSSHQAMLACSAYNFYPKQIRLTWLRNGEKVI-NYVTS-TEELPDGN 58
GXX B                      1:-EPSVRLRSVHAADSRHPRMLLCTGYGFYPKQIRVMWLRNGNNVT-SDVTS-TEELSNGN 57
O29 B1                     1:VKPSVRLRLVESADSKHPGLFMCSAYNFYPKQIKLTWLRDGKEAT-SNVTS-TDELPNGN 58
swordtail DXB (47)         1:VEPYVQLRLEKAEYSQHQQMLICSAYDFYPKQIKVTWLRDGKEVT-SDVTS-TDELPNGN 58
O9 B                       1:VPPVSRLKSVTSNSSEDSEVLVCSAYNFYPQQIRLSWLRDGVVIP-DPPGMMITEMPGGE 59
O33 B1   X                 1:------------------STLVCSAYNFYPKQIRMMWMRNGQEVT-TDVSY-SDVMPDGD 40
O33 B2   X                 1:-----------------------------------------------------------D 1
O57 B                      1:DIPTIRVKSVKQHSGGHPAMLVCSAFNFYPKQIRMIWLRNQKEVT-TGVSY-SEVMPDGD 58
D18 B                      1:VKPEVFVRSLREASGKRPALLSCSAYNFYPKHIKLTWMRDDKVVT-ADVMS-TKVMADGD 58
zebrafish DBB (48)         1:VKPEVFVRSLREASGKRPALLSCSAYNFYPKHIKLTWMRDDKVVT-ADVMS-TKVMADGD 58
D8.45 B1                   1:VKPEVIIRSVTEAKGNRKAVLVCSAYDFYPKGIKLTWMRDDKKVT-AELTS-SEVMADGH 58
D8.45 B2                   1:VKPEVIIRSVTEAKGNQKAVLVCSAYDFYPKAIKLTWMRNDKKVT-ADVMS-IEEMADGD 58
zebrafish DCB (49)         1:VKPEVIIRSVTEAKGNQKAVLVCSEYDFYPKAIKLTWMRNDKRVT-ADVTS-IEEMADGD 58
spotted gar 501 B2   X     1:-EPDVRLSSTKPSSQKHPAMLMCSALGFYPKQIKVSWLRDGQTVT-SDVTS-TEELADGD 57
salmon DEB                 1:VPPIVKVRLTKPSRYGELSMLECSVLGFYPQEVRVSWLRDGRETT-TAVTS-TDTLANGD 58
fathead minnow DEB (50)    1:VPPAVTIRPTKKAHYGQLSTLVCHAYNFYPQAINITWLLDGSEVT-GDVIS-TEFMDNGD 58
spotted gar 501 B1         1:IKPEVKIYPAKTASQGHTHMLVCHAHGFYPRQIAVSWLRNGQPVS-SDLTS-MSFASDGD 58
spotted gar 615 B          1:VPPSVKVSATKLLSSKHPTMLVCHVTGFYPQRITVTWLRDGLEIK-TDVTS-TDLLANGD 58
paddlefish DAB01 (51)      1:IPPSVRVRSMKPFSSQHQTMLVCNAFGFYPREIQMTWLRNGVKVT-ADVSS-SELLSDGD 58
paddlefish DAB02 (52)      1:IPPSVRVRSMKPFSSQHQTMLVCNVFGFYPREIQMTWLRNGVKVT-ADVSS-SELLSDGD 58
paddlefish DAB03 (53)      1:IPPSVRVRSMKPFSSQHQTMLVCNAFGFYPREIQMTWLRNGVKVT-ADVSS-SELLSDGD 58
ray (54)                   1:VEPEITVRPKVSSHSGPSALLTCHVTGFYPPEIEVKWLKNGAPVP-TGAIN-TVLLSDGD 58
nurse shark (55)           1:VEPKVTIRTKESTYPGPSAILICYAVGFYPAKISVTWLKNGQKVS-DADVT-VELLSNGD 58
coelacanth (56)            1:ISPAAVITPTKGMSSSHPNMLVCYVTGFFPSKITVTWFRNGKEVD-SHVTS-SELLQNGD 58
Xenopus DAB (57)           1:SQPNVKIVNTKTLDLEHENLITCFVGGFFPPLIKVTWLKNGIEEG-EQVTS-SELLQNGD 58
chicken BLB (58)           1:VEPKVRVSALQSGSLPETDRLACYVTGFYPPEIEVKWFLNGREET-ERVVS-TDVMQNGD 58
human DQB (59)             1:VEPTVTISPSRTEALNHHNLLICSVTDFYPSQIKVRWFRNDQEET-AGVVS-TPLIRNGD 58
human DRB (60)             1:VEPKVTVYPSKTQPLQHHNLLVCSVSGFYPGSIEVRWFRNQQEEK-AGVVS-TGLIRNGD 58
mouse DOB (61)             1:VPPEVTVYPERTPLLQQHNLLLCSVTGFYPGDISVKWFRNGQEER-SGVMS-TGLVRNGD 58
human DOB (62)             1:VQPEVTVYPERTPLLHQHNLLHCSVTGFYPGDIKIKWFLNGQEER-AGVMS-TGPIRNGD 58
Xenopus DMB (63)           1:VQPSMKVFLPDIVHEGSIPHLVCHVWGFYPADIVVLWLLNDTILV-KNYTN---AVPVGD 56
anole lizard DMB (64)      1:APPQVLIHPVTRQGPPSARVLSCVAWGFFPPEVDIAWFWNGAPVE-AQQGP-LSLRSNGD 58
chicken DMB (65)           1:TPPQVRIVPIPISNDPDTVHLICHVWGFYPPAVTIQWLHNGLVV---ASGD-TKLLPNGD 56
human DMB (66)             1:RPPSVQVAKTTPFNTREPVMLACYVWGFYPAEVTITWRKNGKLVMPHSSAH-KTAQPNGD 59
```

```
                                      E162▼
salmon DAB (31)   high    59:WYYQIHSHLEYTPRSGE-KISCMVEHISLTEPMVYHW                       94
trout DAB (32)            59:WYYQIHSHLEYTPKSGE-KISCMVEHISLTEPMMYHW                       94
Arctic char (33)          59:WYYQIHSHLEYTPKSGE-KISCMVEHNSLTEPMVYHW                       94
D8.35 B1   X              38:WYYQIHSELEYSPKPGE-RISCVIEHASSNKPMIYDW                       73
D8.35 B2                  59:WYYQIHSELEYSPKPGE-RISCVIEHASSNKPMIYDW                       94
D8.35 B3   high           59:WYYQIHSELEYSPKSGE-KISCAVEHASSTKPIIYDW                       94
D8.37 B1                  59:WYYQIHSELEYSPKSGE-RISCGIEHASSNKPIITEW                       94
D8.37 B2   X              40:WYYQIHSELEYTPGPGE-KISCLVEHASSSEPMIYDW                       75
D8.37 B3   high           59:WYYQIHSHLEYTPKSGE-KIQCLVEHASLTQPLTKDW                       94
D4 B                      58:WYYQSHSELVFSPKSGE-TISCMVEHSSLTGPVVIDW                       93
zebrafish DAB1 (34)       59:WYYQIHSHLEYTPKSGE-KIQCVVEHASSTQPITKEW                       94
zebrafish DAB2 (35)       59:WYYQIHSHLEYTPKNGE-KIQCVVEHASSTQPITKEW                       94
zebrafish DAB3 (36)       59:WYYQIHSHLEYTPKSGE-KIQCLVEHASLTQPLTKDW                       94
zebrafish DAB4 (37)       59:WYYQIHSHLEYTPKSGE-KIQCLVEHASLTQPLTKDW                       94
zebrafish DDB (38)        58:WYYQIHSELEYTPRPGE-KISCMVEHASSSEPMIYDW                       93
zebrafish DEB (39)        59:WYYQIHSHLEYTPKSGE-KISCMVDHASSTEPIIIAW                       94
carp DAB (41)             59:WFYQIHSELEYTPKSGE-KISCMVEHASFSKPMITDW                       94
G129 B   X                57:-------------RSGE-KMSCVVEHISLSKPLVTDW                       79
G131 B1                   59:WYYQTHSHLEYTPRSGE-KISCVVEHISLSKPLVTDW                       94
G131 B2   X               59:WYYQTHSHLEYTPRSGE-KISCVVEHISLSKPLVTDW                       94
G131 B3                   59:WYYQTHSHLEYTPRSGE-KISCVVEHISLSKPLVTDW                       94
GIII B                    59:WYYQTHSHLEYTPRSGE-KISCVVEHISLSKPLVTDW                       94
GVII B                    59:WYYQTHSYLEYTPRSGE-KIACVVEHISLSKPLVTDW                       94
stickleback DAB (42)      59:WYYQTHSHLEYTPRSGE-KISCVVEHISLSKPLVTDW                       94
stickleback DBB (43)      59:WYYQTHSHLEYTPRSGE-KISCVVEHISLSKPLVTDW                       94
```

```
M3 B                          59:WFYQIHSYLEYTPRSGG-KISCRVEHISLKDPLITDW   94
M615 B                        59:WYYQIHSHLEYTPRSGE-KISCRVEHASLKDPLITDW   94
M873 B   high                 59:WYYQIHSHLEYTPRSGE-KISCKVEHASLKDPLVTEW   94
medaka DAB (44)               59:WYYQIHSHLEYTPRSGE-KISCKVEHASLKDPLVTDW   94
medaka DBB (45)               58:WYYQIHSHLEYTPRSGE-KISCKVEHASLKDPLVTEW   93
medaka DCB (46)               51:-------------------------------------   51
F7533 B                       59:WFYQLHSHLEYTPRSGE-KISCVVEHASLKTPLVKDW   94
F8524 B   X                   59:WFYQLHSHLEYLP------------------------   71
T19 B                         59:WFYQLHSHLEYTPRSGE-KISCVVEHASLKTPLVKDW   94
T48 B   X                     58:WFYQLHSHLEYTPRSGE-KISCVVEHASLKTPLVRDW   93
T61 B   X                     15:WFYQLHSHLEYTPRSGE-KISCVVEHASLKTPLVKDW   50
T70 B   X                     59:WFYQLHSHLEYTPRSGE-KISCVVEHASLKTPLVKDW   94
O1 B1                         59:WYYQTHSQLEYTPRSGE-KISCVVEHASLKEPLITDW   94
O18 B1                        59:WYYQTHSHLEYTPRSGE-KISCVVEHASLEKPLITDW   94
O18 B2                        59:WYYQTHSHLEYTPRSGE-KISCKVEHASLEKPLITDW   94
O18 B3   X                     8:WYYQIHSHLEYTPRSGE-KISCKVEHASLEKPLITDW   43
O22 B1                        58:WLYQTHSYMEYTPRSGE-KISCMVEHASLKEPLVTDW   93
O31 B1                        59:WYYQTHSHLEYKPRSGE-KISCVVEHVSLSEPLITDW   94
O31 B2                        59:WYYQTHSYLEYTPRSGE-KISCVVEHVSLSEPLITDW   94
O31 B3                        59:WYYQTHSHLEYTPRSGE-NISCVVEHASLKEPLITDW   94
O49 B   X                     40:WYYQTHSHLEHTPRSGE-KISCKVEHASLEKPLITDW   75
O50 B   X                     37:WYYQTHCYLEYTPRSGE-KISCVVEHASLEKPLITNW   72
O77 B   X                     55:WYYQTHCYLEYTPR-----------------------   68
O79 B1   X                    59:WLYQVHSHLEY--------------------------   69
O79 B2                        59:WLYQVHSHLEYMPRSGE-KISCVVEHASLEKPLIINW   94
O80 B                         59:WYYQTHCYLEYTPRSGE-KISCVVEHASLEKPLITNW   94
O745 B                        59:WYYQTHSHLEYTPRSGE-KISCKVEHASLEKPLITDW   94
O779 B   X                    34:*YYQIHSHLEYTPRSGE-NISCVVEHASLKEPLITDW   68
O845 B                        58:WLYQTHSYMEYTPRSGE-KISCMVEHASLKEPLVTDW   93
OA78 B   X                    59:WFYQTHSYLEYTP------------------------   71
---------------------------------------------------------------------------
salmon DBB                    56:WSYQIQSYLKYTPTTGE-RITCMVEHISQTEPKLYYW   91
GXVII B                       57:WTYAVHSSLSFTPGGRD-RVSCKVEHAGLQEPALRTW   92
M5 B1                         55:WTYQAHTYLTLTPGKQD-HISCMVQHTSLKEPKIYNW   90
M5 B2                         55:WTYQAHTYLTLTPGKQD-HISCMVQHTSLKEPKIYNW   90
O97 B1   X                    42:-------------------------------------   42
O97 B2   X                    46:------------------RVTCTVEHVSLKEPKSSYW   68
salmon DCB                    59:WTYQIHSHLEYTPTPGE-RIPCMVEHFSLTEPKLYDW   94
GXX B                         58:WLYQIHSYLEFTPRSGG-EISCVVEHASLTQPKVYDW   92
M16 B                         59:WLYQIHSYLEYTPSPRE-EITCMVEHASPKEPKLYNW   94
O29 B1                        59:WLYQIHSYIEISSKPGE-KISCVVEHASLTEPKVIDW   94
swordtail DXB (47)            59:WLYQIHTYLEFTPKPGE-KITCMVEHASLKEPSLYDW   94
O9 B                          60:WRYQIHSYLQHKLNSGQ-NITCMIEHRGLQEPQLLKL   95
O33 B1   X                    41:WYYQTHSYLEYIPTSGE-KIACMVEHLGLSEPMFVVW   76
O33 B2   X                     2:WYYQTHSYLEYIPTSGE-KIACMVEHLGLSEPMFVVW   37
O57 B                         59:WYYQIHSYLEYTPTHGE-KITCVVEHLSVSEPILAIW   94
D18 B                         59:WYYQIHSHLEYFPQPGE-KISCVVEHASSHKPMIYYW   94
zebrafish DBB (48)            59:WYYQIHSHLEYFPQPGE-KISCVVEHASSHKPMIYYW   94
D8.45B1                       59:WHYQIHSYLEYFPQTGE-KISCVVDHASSLKPMIYYW   94
D8.45B2                       59:WYYQIHSHLEYFPQPGE-KISCVVDHASFHKPMIYYW   94
zebrafish DCB (49)            59:WYYQIHSHLEYFPQPGE-KISCVVDHASFHKPMIYYW   94
---------------------------------------------------------------------------
spotted gar 501 B2   X        58:WYYQIHSHLEYTPRAGE-SIVCRVEHSSFATPKELTW   93
---------------------------------------------------------------------------
salmon DEB                    59:WSYQLHSYLEFRPQRGE-SVSCMVEHPSLDEPLEVVW   94
fathead minnow DEB (50)       59:WRYQMHSHLDLVLRRGV-SVSCRVEHSGLEKPLVVQW   94
---------------------------------------------------------------------------
spotted gar 501 B1            59:WTYQTLLYLEFTPQGGE-TFECAVDHVALDGTLKLRW   94
spotted gar 615 B             59:WTYQVHSHLELTPRAGE-TVACRVEHSSLERPLEVTW   94
paddlefish DAB01 (51)         59:WTYQIHSYLELTPQSGD-SYSCRVEHSSLAEAIEVKW   94
paddlefish DAB02 (52)         59:WTYQIHSYLELTPQSGD-SYSCRVEHSSLAEAIEVKW   94
paddlefish DAB03 (53)         59:WTYQIHSYLELTPQSGD-SYSCRVEHSSLAEAIEVKW   94
---------------------------------------------------------------------------
ray (54)                      59:WTYQVEELLQYHPVSGD-VYTCHVEHVSLTEPMTVDW   94
nurse shark (55)              59:WTYQVRQYLQYEPVYGD-KYTCHVEHSSLTSPMSVDW   94
---------------------------------------------------------------------------
coelacanth (56)               59:WTYQIHVFLEMTPKSGD-VYTCRVEHSSLEDPMELTW   94
Xenopus DAB (57)              59:WTFEIHVMLETTIKHGD-TFTCRVEHSSLQQPVYLNW   94
chicken BLB (58)              59:WTYQVLVVLETVPRRGD-SYVCRVEHASLRQPISQAW   94
```

human DQB (59)	59:WTFQILVMLEMTPQRGD-VYTCHVEHPSLQSPITVEW	94
human DRB (60)	59:WTFQTLVMLETVPRSGE-VYTCQVEHPSVTSPLTVEW	94
mouse DOB (61)	59:WTFQTTVMLEMIPELGD-IYSCLVEHPGLLRPVSVAW	94
human DOB (62)	59:WTFQTVVMLEMTPELGH-VYTCLVDHSSLLSPISVEW	94
Xenopus DMB (63)	57:WTYQIVALLDMRGSLPENKYTCVVQHSSLQDPMTEDW	93
anole lizard DMB (64)	59:WTFQAERSLALEPRPRG-IYSCRVNHPSLQEPIVVEW	94
chicken DMB (65)	57:WTYRTQVALRASTAAGS-TYTCSVWHSSLEQPLQEDW	92
human DMB (66)	60:WTYQTLSHLALTPSYGD-TYTCVVEHIGAPEPILRDW	95

Text S4*E*: Transmembrane and cytoplasmic domains alignment

(a) MHC class IIα transmembrane/cytoplasmic domains

```
DA     salmon DAA (1)              VFCGVGLTLGLLGVAAGTFFLIKGNQCN
       stickleback DAA (1)     VGPAVFCGVGLTVGLLGVAAGTFFLIKGNECS
       zebrafish (4)           VGPAVFCGVGLVLGLLGVAAGTFFLIKGNNCN
DB     salmon DBA                PAVFCGVGLTLGLLGVATGIFLIYKGKRATESQE
       salmon DCA              AGPAVFCGVGLTLGLLGVATGTFLYVKGQQFN
       zebrafish D8.46 A           VFCGVGLALGLLGFATGVFFTAKGNNCN
DE     salmon DEA                 TAVCVGGVTLGVVGVATGVWFIKKAKRSGWALRT
       loach DEA (16)              AVLVVGIIVGFIGLVAGIMVIVMSKLELPAV
       spotted gar 615 A*          AVCGVGLTLGLLGVAAGTFFLIKGNKCN
cart.  nurse shark (19)            IICALGLTLGIISAVVGIILLIKERQRLQAQQHGI
t.cl.  Xenopus DAA (21)            VICALGLAVGIIGIIAGVMLIIKGMKQSAAQGRSQR
       chicken BLA (22)          ATLWCAVGLAVGIAGIAAGTALILRAVRRNAANRQPGLL
       human DQA (23)              VVCALGLSVGLMGIVVGTVFIIQGLRSVGASRHQGPL
DO     mouse DOA (25)              LICGLGLVLGLMGCLLGTVLMITGTRRPSIRR
       human DOA (26)              LVCALGLAIGLVGFLVGTVLIIMGTYVSSVPR
DM     Xenopus DMA (27)            VLCYLAIAIGIVFLFLGLLFLFLTWKQHRND
       chicken DMA (29)        VLATAVCGAVTALGILLALLGLGLLLSARRRSMWGQWRQQGHPPRTH
       human DMA (30)              VLCGVAFGLGVLGIIVGIVLIIYFRKPCSGD
```

(b) MHC class IIβ transmembrane/cytoplasmic domains

```
DA     salmon DAB (31)            IAIGASGLVLGAILALAGLI--YYKKKSS--GVL
       stickleback DAB (42)       VAIGASGLILGLTLSLAGFI--YYKRKAR--GRILVPSH
       zebrafish DAB (34)         FAIGASGLVLGIIIAIAGLI--YYKKKST--GRILVPN
DB     salmon DBB                 IVIGVCGLLLGVVFVVAGLI--YWAKST---GRLLGLIGER-D-YGTCD
       stickleback GXVII B     TGFLVGGVCALLLGAACLSSGLI--VHRRKYSNIS
       salmon DCB                 MVIGACGLLLGVVFIAAGLI--YYRKKSTE-GRVLEPTMALPESYGTI
       swordtail DXB (47)         IVVGPAGLLLGLVFSIAGFI--YYKTTSN--GQVVVPTTEDVCPEETL
       zebrafish D8.45 B1         IILGAVGLLMGIFTAAAGVI--YYKRKQT--GFYRLPVCLLPMETMNDTELQ
DE     salmon DEB                 MAIGVCSLFIGVAMAIGGGVYYWWKN-----RSGFRRVNR
       fathead minnow DEB (50)    LAVGCFSFLLGLIVAFSAA-YIYYKRRHR--GFSSLPL
       spotted gar 615B*          IVIGVSGLILGLIITAAGVI--YYKKKSS--GRILVPSD
       paddlefish DAB01 (51)      IIIGTSGIVLGLVILAAGLI--YYKKKAK--GRILVPSD
cart.  ray (54)                   IIVGALGFVFGLLILLAGV---IMRLKNA--KAILDSASHGPRLMGPAIS
       nurse shark (55)           IIVGVLGFGFGLIILLAGV---IMRLKNA--KAILDS-NHGPRLMGPAVS
t.cl.  Xenopus DAB (57)           MLTGIIGFVLGSIFIIVGLV-VYLRSKK---TMAHFSSVQNENLM
       chicken BLB (58)           LLTGVGGFVLGLVFLALGLF-VFLRGQK---GRPVAAAPGMLN
       human DQB (59)             MLSGVGGFVLGLIFLGLGL---IIRQRSRK-G-LLH
DO     mouse DOB (61)             ILSGAAAFLLGLIVFLVGVV-IHLKAQK---ASVETQ-PGNEASRE-LLHSQP
       human DOB (62)             MLSGIAAFLLGLIFLLVGIV-IQLRAQK---GYVRTQMSGNEVSRAVLLPQSC
DM     Xenopus DMB (63)           ISIATVVFVLGLVTLIAGFV--LWRNAKKS-GYIPIPG--YNEGN
       chicken BMB (65)           VAVATVVMVLGLSLLFIGVY--CWRAQPPAPGYAPLPGHNYPSGSI
       human DMB (66)             VSVSAVTLGLGLIIFSLGVI--SWRRAGHS-SYTPLPGSNYSEGWHIS
```
